# Molecular Characterization and Phylogenetic Analysis of Casein Gene Family in *Camelus ferus*

**DOI:** 10.3390/genes14020256

**Published:** 2023-01-18

**Authors:** Shakeela Parveen, Peng Zhu, Laiba Shafique, Hong Lan, Dingyun Xu, Sana Ashraf, Saba Ashraf, Maryam Sherazi, Qingyou Liu

**Affiliations:** 1 Guangdong Provincial Key Laboratory of Animal Molecular Design and Precise Breeding, School of Life Science and Engineering, Foshan University, Foshan 528225, China; 2 Guangxi Key Laboratory of Beibu Gulf Marine Biodiversity Conservation, Beibu Gulf University, Guangxi 535011, China; 3 Department of Zoology, Government Sadiq College Women University, Bahawalpur 63100, Pakistan; 4 State Key Laboratory for Conservation and Utilization of Subtropical Agro-Bioresources, Guangxi University, Nanning 530005, China; 5 Department of Dairy Technology, University of Veterinary and Animal Sciences, Lahore 54000, Pakistan

**Keywords:** casein, genome, phylogenetic, promoter region, nuclear hormone

## Abstract

Camel milk is known for its exceptional medical uses. It has been used since ancient times to treat infant diarrhea, hepatitis, insulin-dependent diabetes (IDDM), lactose intolerance, alcohol-induced liver damage, allergies, and autism. It has the power to treat several diseases, with cancer being the most significant. This study investigated the evolutionary relationship, physiochemical characteristics, and comparative genomic analysis of the casein gene family (CSN1S1, CSN2, CSN1S2, and CSN3) in *Camelus ferus*. Molecular phylogenetics showing the camelid species clustered casein nucleotide sequences into four groups: CSN1S1, CSN2, CSN1S2, and CSN3. The casein proteins from camels were evaluated and found to be unstable, thermostable, and hydrophilic. CSN1S2, CSN2, and CSN3 were acidic, but CSN1S1 was basic. CSN1S1 showed positive selection for one amino acid (Q), CSN1S2 and CSN2 for three (T, K, Q), and CSN3 showed no positive selection. We also compared high-milk-output species such as cattle (*Bos Tarus*) and low-milk-yield species such as sheep (*Ovies Aries*) with camels (*Camel ferus*) and discovered that YY1 sites are more frequent in sheep than in camels and very low in cattle. We concluded that the ratio of YY1 sites in these species may affect milk production.

## 1. Introduction

Genome analysis and a significant number of genomic sequences are new approaches to understanding gene control and molecular evolution [1]. Comparative genomics can lead to the discovery of novel genes and the identification of functional elements. In this approach, two or more genes are investigated and compared holistically to find similarities and differences between each genome [2].

In both the ancient and present eras, camels are utilized for transportation (for beast of lifting burden), for food (for meat and milk), for fiber (hair and wool), and for riding animals. These animals play a major economic and cultural role in nomadic Asia, Africa, and rural South America [3]. Although camelids can survive in harsh environments on a limited number of resources, they have not historically been considered an important source of food and milk. Camel milk, for instance, accounts for only 10% of all milk production [4].

Camel milk is renowned for its extraordinary therapeutic benefits. It has the ability to treat a variety of diseases, with cancer being the most significant [5]. Since ancient times, camel milk has been used to treat a variety of ailments, including autism infant diarrhea [6], hepatitis [7], lactose intolerance [8], alcohol-induced liver damage [9], hepatitis [10], and insulin dependent diabetic mellitus (IDDM). There are numerous chemicals that are crucial for immunology, such as lysozymes, lactoperoxidase, and lactoferrin, which can be attributed to camel milk’s health advantages. The amount of an insulin-like molecule in camel milk is quite high [5]. Moreover, camel milk has a therapeutic effect on a number of illnesses, including piles, jaundice, dropsy, food allergies, asthma, and tuberculosis [11]. Additionally, it has been noted that camelid milk can be used as a supplement to mother’s milk and has positive effects on patients with extreme exhaustion and liver illness [6,12]. Camel milk is generally opaque white [7,10], and normally it has a sweet and sharp taste, but sometimes it is salty [8]. It is frothy when shaken slightly [9]. The changes in taste are caused by the type of fodder and the availability of drinking water. Camel milk is somehow different from cow milk in its chemical composition, but it contains all the essential nutrients in cow milk [13]. Unlike cow milk, it was found that camel milk can be preserved for a longer time at 30 °C, and most importantly the camel milk can be kept at 4 °C for more than three months without any visible change [10].

Physiochemically, milk proteins are divided into whey (serum) and casein families. The primary milk protein is casein (CN), which accounts for 80% of the total milk proteins, including CSN1S1, CSN1S2, CSN2, and CSN3. Each casein protein has distinct genetic, functional, and amino acid features [11]. In addition to being linked to milk parameter estimation and lactation behavior in dairy animals, milk caseins are physiologically significant because they provide nutrition for newborns. The casein protein is composed of calcium-sensitive CSN1S1, CSN1S2, and CN2 caseins, which support bone development in young animals by supplying calcium- and phosphorus-rich stable micelles, and Ca-insensitive CN3 casein [12]. In camel milk, αs1-CN (22%) is the second main fraction after β-CN (65%) and before αs2-CN (9.5%) and κ-CN (3.5%) [14]. 

Erhardt et al. discovered D, a novel variation. The CSN1S1 gene polymorphism is intensively examined in camels, but its effect on milk production has not yet been established, and the relevance of these SNPs in comparison with animal phenotypic data remains to be studied.

This protein’s CSN1S2 gene has rarely been examined in animals, including camels. No literature has been found about this gene’s single-nucleotide polymorphisms (SNP) affecting camel milk production [15]. β-Casein is the most abundant protein in camel milk, and its coding gene (CSN2) is called the “primary” gene in other species [3].

The presence of different levels of β-casein phosphorylation has been shown to affect the availability and distribution of calcium, in addition to the stability of micelles [16]. These findings suggest that β-CN plays an important role in the development of the technological properties of milk and dairy products, as well as in nutrition. A number of studies on ruminants have discovered a correlation between the β-casein gene polymorphism and economically important properties of milk. Kappa-caseins are essential to the stabilization of casein micelles, which determine the specific qualities of milk [17]

The transcription factors (TFs) and the locations within the DNA to which they bind are considered to be one of the most essential functional components of any genome. Defects in these interactions between protein and DNA can contribute to the advancement of a variety of disorders. These interactions control many crucial activities, including critical phases in development and responses to environmental stimuli.

There has been a lot of recent advancement in the accumulation and analysis of mRNA transcript profiles of a variety of tissue and cell types, such as those associated with different human disorders [18]. Despite this, there is still a lot that needs to be known about transcriptional pathways which regulate various expression patterns [3,15,16,17,19].

It will be possible to map the regulatory pathways within cells in a more thorough and quantitative manner by having a better understanding of transcription factors, their DNA binding sites, and interactions. This will also help us understand the potential activities of specific genes that may be regulated by newly discovered DNA-binding sites. The selection of markers in dairy mammals (GATA, TATA, STAT, and OCT1) and one repressor binding site (YY1) for comparative genomic analysis can assist the identification of crucial regulatory areas necessary for the expression of the CSN genes [18,20,21].

YY1 uses multiple suppression mechanisms. YY1 competes with activator factors and overlaps the gene’s binding site, inhibiting transcription. YY1 competes with a -CN activating promoter in mammary epithelial cells, repressing transcription [21]. In addition to YY1 overlap sites, the c-fos promoter has two extra YY1 sites between the TATA box and CRE [22].

In the field of evolutionary studies, casein is regarded as an important molecular model [23]. It is also important for understanding the genomic architecture of less-studied species, mammalian phylogeny, and domestic animal genomics. Different species or breeds have varying milk yield and composition features, including protein, fat, and solid contents, suggesting the importance of gene regulatory regions. Understanding the camel casein gene family’s regulatory mechanisms requires studying its genomic architecture and evolution. Current research emphasizes the need to observe and understand the physiochemistry, comparative genomics, and gene regulatory areas of the casein gene family in camels.

## 2. Materials and Methods

The NCBI genome database was used to collect the data. The protein sequences, genome sequences, and CDS sequences may all be accessed via NCBI. Data sets for gene research were constructed in advance for any upcoming analysis. The Maximum Likelihood technique was used to infer an origin and evolution for several species utilized in the JTT table-based model [24]. Using 1000 replicates of the bootstrap method, a tree made of 28 amino acids sequences was constructed to show the nodes on tree.

For better results, gaps and mistakes in the sequence were removed, and MEGA7 [25] was used to construct a phylogenetic tree. Coding sequences of the camel casein gene were further analyzed with GSDS (Gene Structure Display Server) [26]. For gene structure display, we prepared two files, CDS and genomic. Both files were added into the server for results. Furthermore, the MEME Suite was used to evaluate 10 conserved MEME motifs of casein. For motif analysis we prepared a casein protein file and exported it into MEME. After obtaining results, the Xml file was used for further analysis of motif results [27]. We developed a web server (Selection) that computes synonymous and non-synonymous substitutions from selected sequences and files and saves them as codon alignment to perform selection analysis. For selection analysis, we prepared CDS files and deleted the stop codon for better results [28]. ShinyGo 0.76.1 was used for gene enrichment analysis. For gene enrichment analysis, we prepared a number of gene lists and exported them into the server that provides gene information for different functions of genes [29]. ProtParam tool was implied to display the physicochemical parameters of camel casein proteins, including the instability index amino acid number, aliphatic index, isoelectric point, grand molecular weight, and average hydropathy [30]. The genetic sequencing of the camel milk protein genes was presented to Promoter 2.0 Prediction Server to spot possible transcriptional interacting factor indications. The binding site score >1.0 as an anticipated strong probability area and the sequencing of the putative interaction factor site were investigated within 1000 bp upstream regions from the high-likelihood predicted site [31]. In particular, genomic transcripts were evaluated using the TRANSFAC transcription factor databases’ weight matrix and TFBIND tool to locate transcription factor sites to bind properly [32]. As stated before, there are four possible transcriptional binding locations (GATA, STAT, OCT1, and TATA) [3,33,34,35,36] and one suppression position (YY1) [37] in the casein gene family in camels. NHR Scan was used for the analysis of nuclear hormone receptor sites in *Camelus ferus*.

## 3. Results

**Figure 1:**
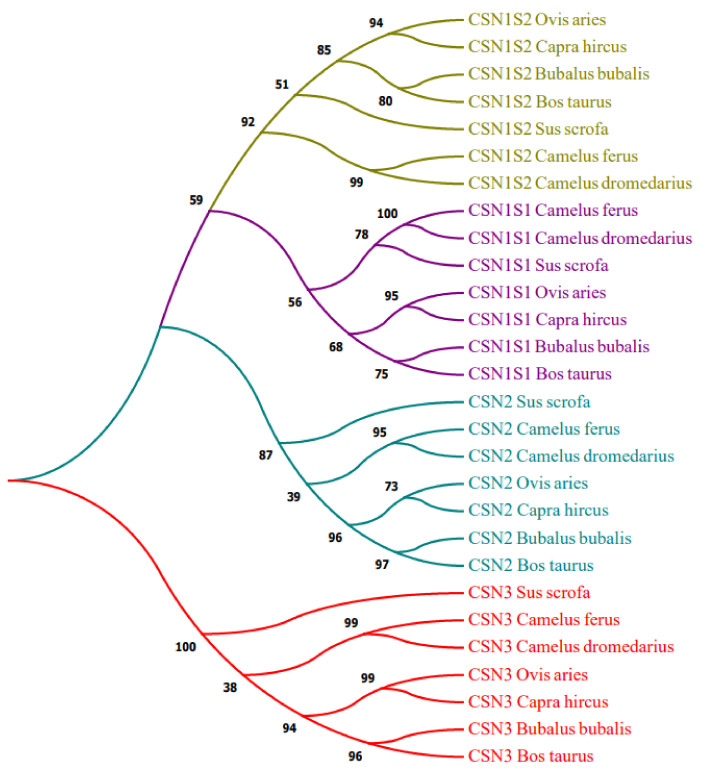
Phylogenetic relationship study of the casein gene family (purple: CSN1S1, olive green: CSN1S2, Teal: CSN2, and red: CSN3).

A morphological and molecular evolutionary investigation of typical camelid species indicated that all casein nucleotide sequences fall into four distinct groups: CSN1S1, CSN2, CSN1S2, and CSN3 (Figure 1). Acession numbers of all selected species also represented in (Supplementary Table S1).

**Figure 2:**
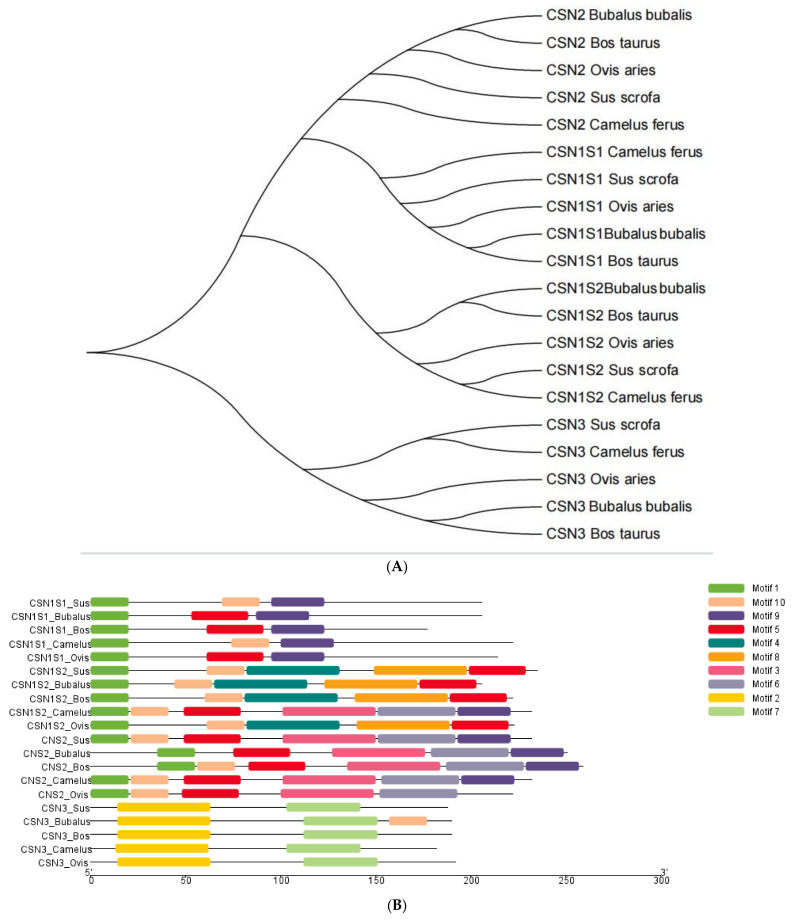

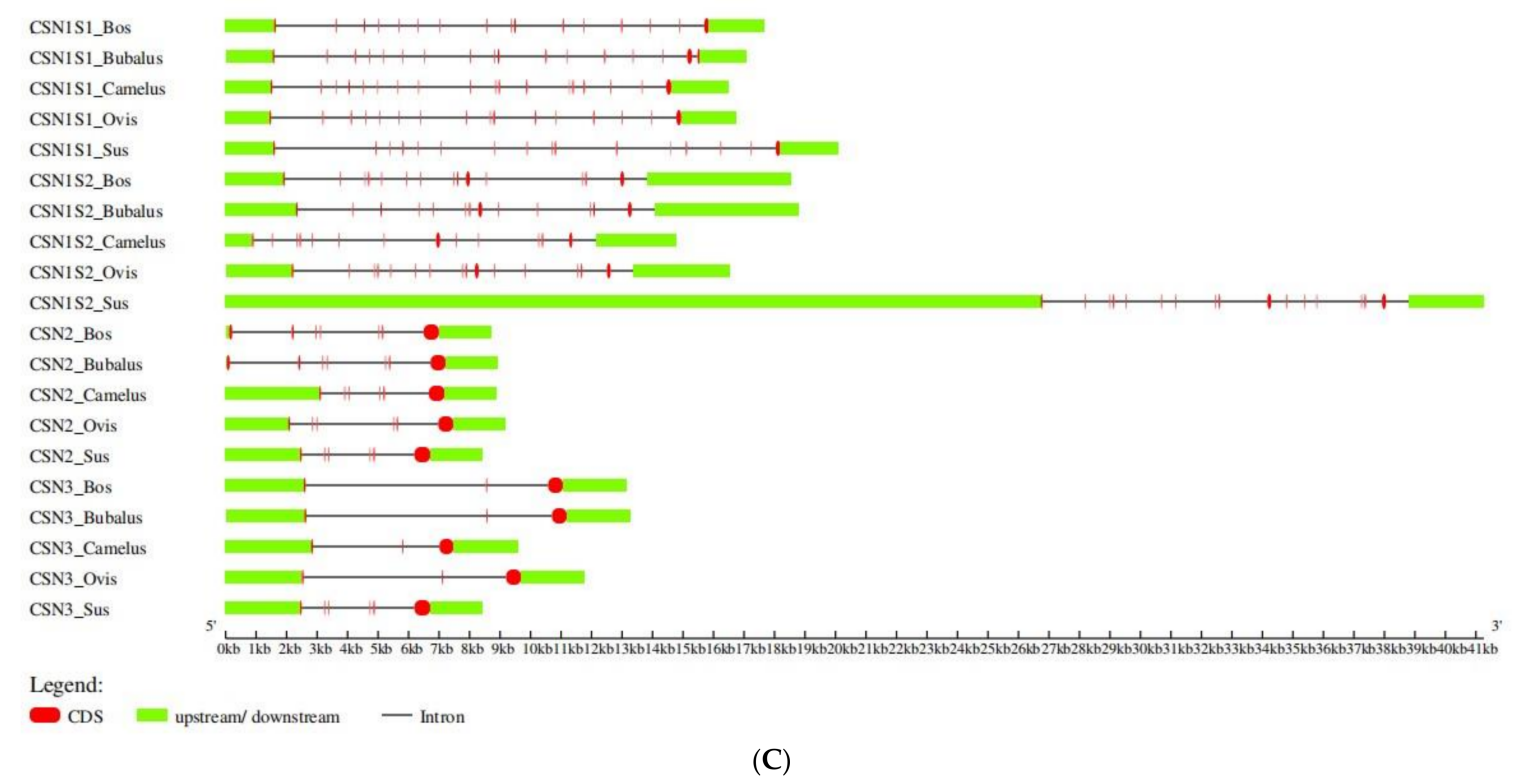
Genome structure, motif patterns, and conserved regions of the casein protein gene family phylogenetic connections. (**A**) The phylogenetic connection of the 20 amino acid sequences of casein proteins. (**B**) A recurring motif. In the casein gene family, there are many structural variants (**C**). Color-coded boxes denote ten distinct themes. Table 1 provides further information about the patterns.

**Figure 3:**
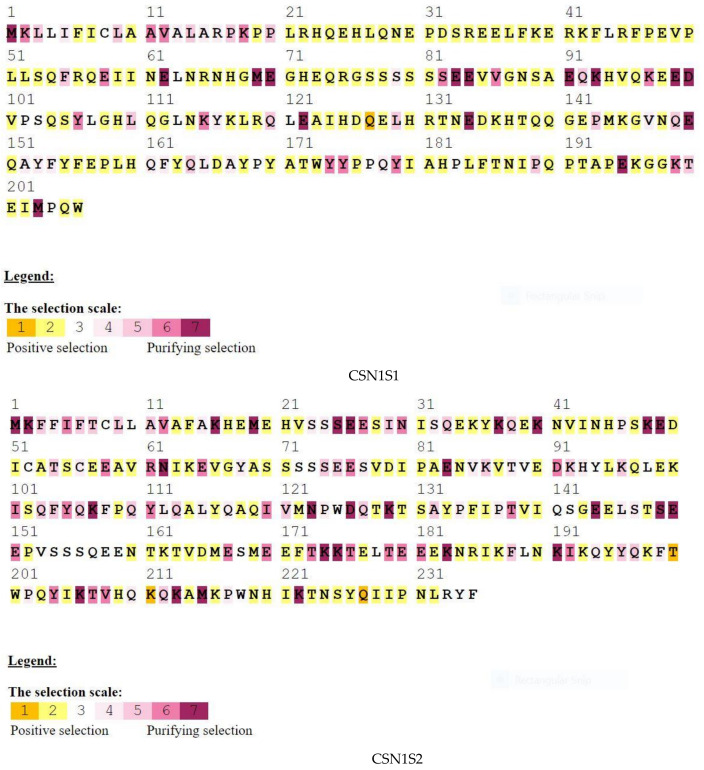

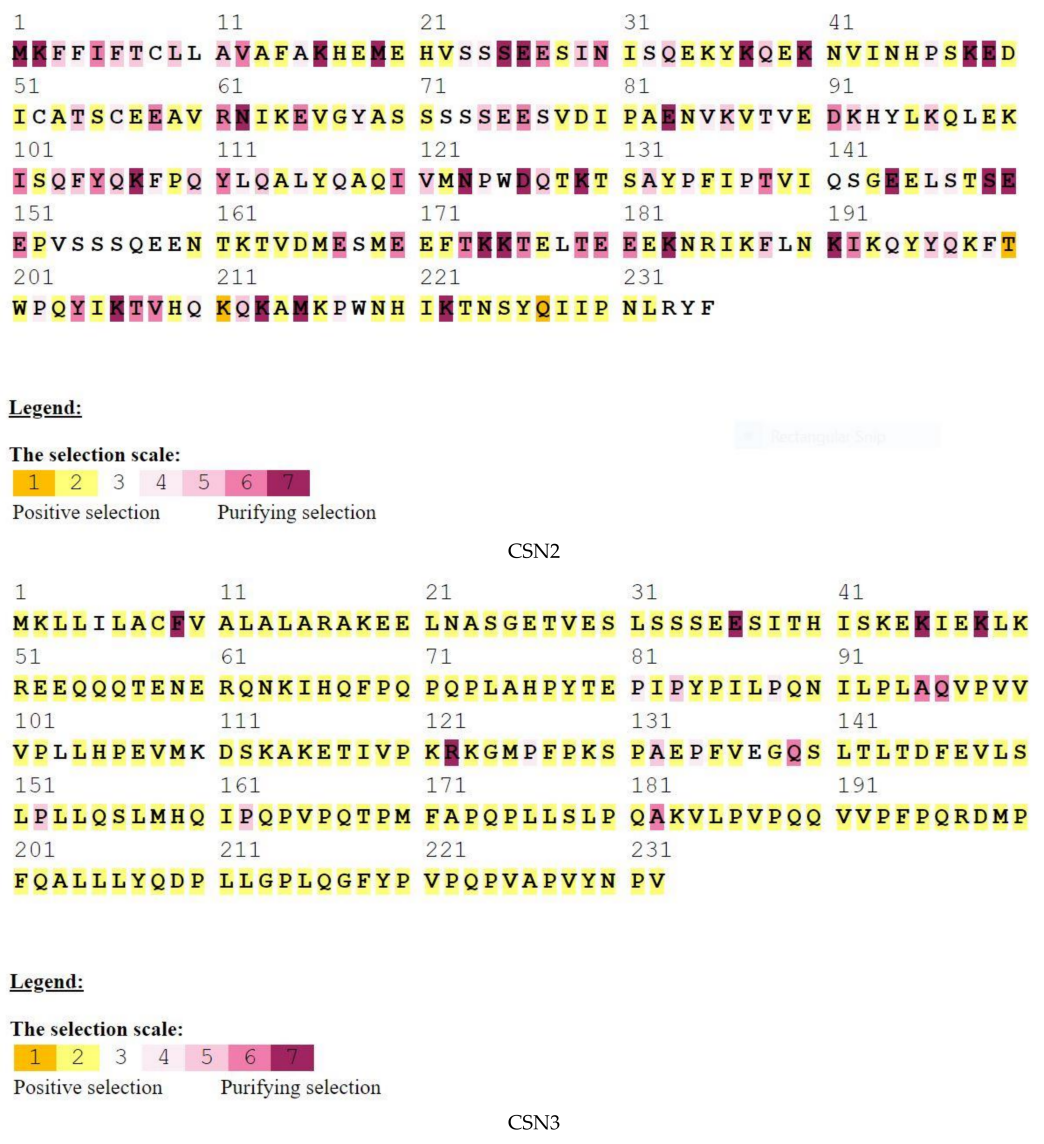
Selection analyses of CSN1S1, CSN1S2, CSN2, CSN3 gene.

**Figure 4:**
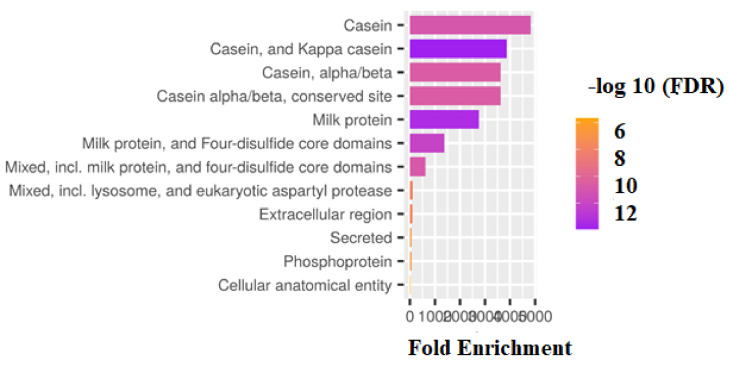
Casein gene enrichment analysis.

In addition, to undertake structural characterization of the CSN genomic family in several species, phylogenetic analyses of gene organization, motif patterns, and conserved areas were conducted (Figure 2). Ten conserved MEME motifs were discovered in casein genes (Figure 2B). After a Pfams analysis, motif 8 comprising 50 amino acid residues was identified as the casein kappa (kappa-CN) area, whilst motifs 2, 5, and 7 were marked as the casein region (Table 1). In addition, the downstream and upstream untranslated regions (UTRs) and intron structure of cattle CSN coding genes in the similar assembly varied considerably. However, structural examination of the gene revealed that these genes had the same integer of introns and exons (Figure 2C). Figure 3 depicts the preference investigation of various casein genomic groups, with CSN1S1 showing a stronger correlation in the particular subset (M8 and M8a) but CSN1S2, CSN2, and CSN3 showing no significant relation. CSN1S1 demonstrated +ve selection for one amino acid (Q), CSN1S2 and CSN2 demonstrated +ve selection for three amino acids (T, K, and Q), and CSN3 demonstrated no +ve selection. Positive selection is also favored by mutation, and these changes due to environmental modifications and expressions could cause harmful or neutral effects on alleles to become positive. Figure 4 depicts a genomic enrichment examination of various casein genomic groups that illustrate gene functional properties. The enrichment analysis also provided the knowledge about the list of casein genes that we analyzed in our study.

The physical and chemical characteristics of the CSN genomic family in *Camelus ferus* were evaluated based on their chromosomal allocation, molecular mass (Da), the number of amino acid residues in protein, the aliphatic index (AI), the iso-electric point (pI), the instability index (II), and the grand average of hydropathicity index (GRAVY), as shown in Table 2. All CSN gene products were discovered on chromosome no. 2, which contains a vast variety of exons and a different length of the gene with residues of amino acids, as shown in Table 2. CN peptides had molecular weights ranging from 20 to 26 kDa. As the aliphatic score for all casein proteins was greater than 65, the CN proteins of camel were demonstrated to be destabilizing but thermally stable peptides. Furthermore, scores indicated that all CN peptides, s2-CN, -CN, and CN, were low-pH peptides, with the exception of ‘s1-CN,’ which was gently alkaline in its origin, as shown in Table 2. Reduced GRAVY values suggest that camel CN peptides are water soluble (Table 2). A promoter region analysis is also shown in Supplementary Table S2. 

**Figure 5:**
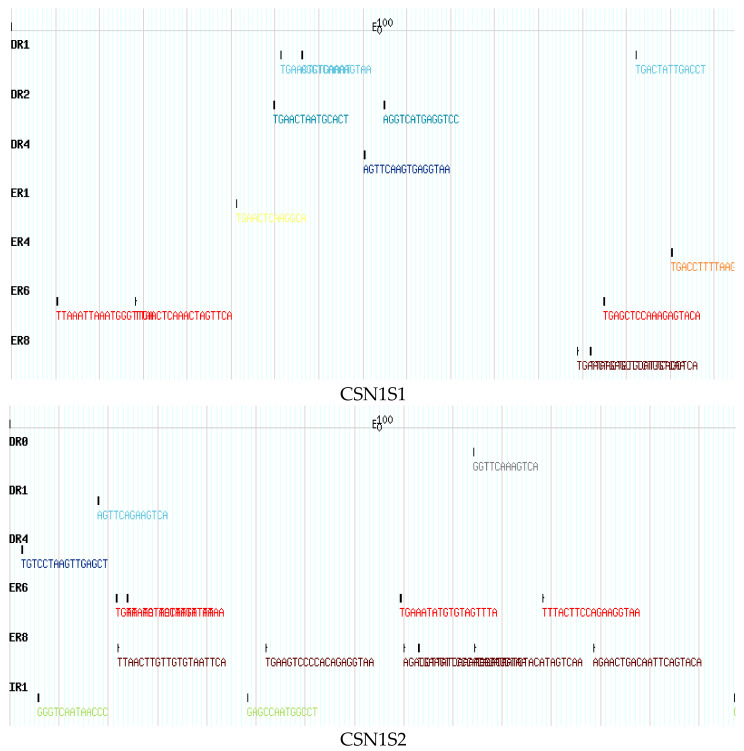

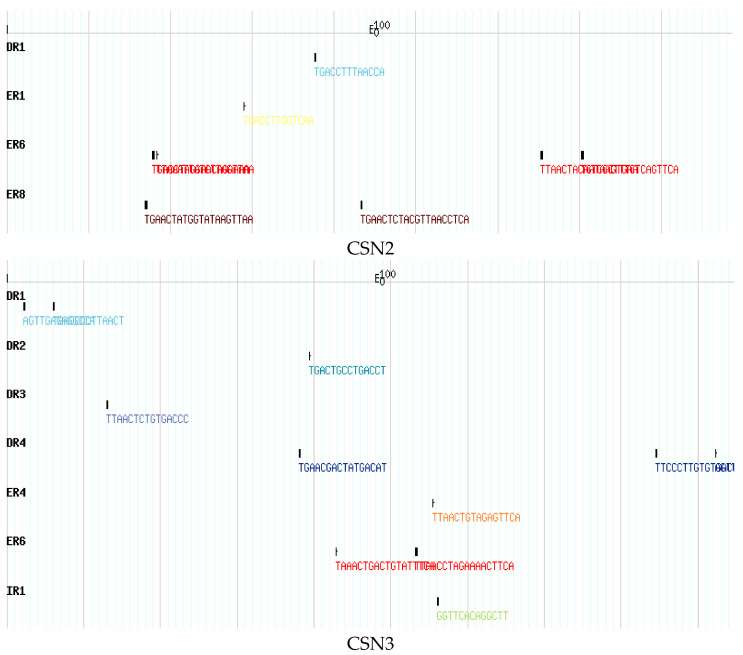
Nuclear hormone receptor site patterns in the casein gene family of *Camelus ferus*.

**Figure 6:**
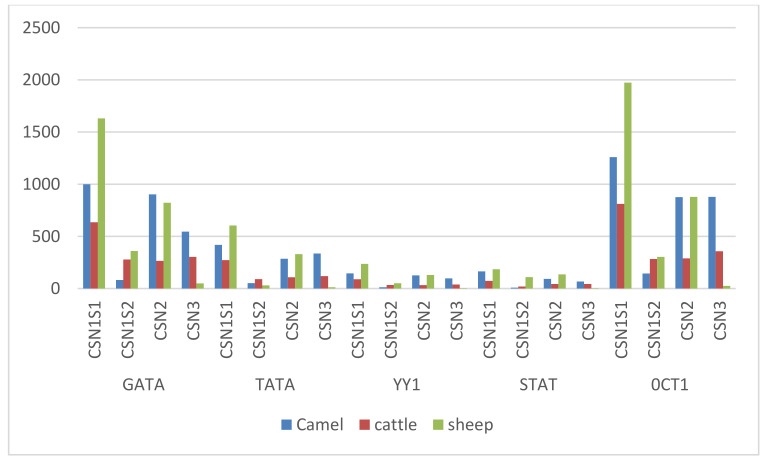
In the genomes of the casein protein, a comparison of the distribution of potential transcription binding sites in *Camelus ferus*, *Bos Tarus*, and *Ovis Aries*.

Using genome sequencing data sets from *Camelus ferus*, the arrangement of nuclear hormone receptor (NHR) regions in the camel CSN gene family was studied. The camel CSN gene family has a total of 48 NHRs sites (Supplementary Table S3). In particular, there were 13, 16, 8, and 11 NHRs found in CSN1S1, CSN1S2, CSN2, and CSN3, correspondingly (Supplementary Table S3). Four inverted repeats (IR) were found in numerous CSN genes that act primarily as hormone response elements (HREs) for steroid receptors. CSN3 included one IR and CSN1S2 contained four IRs, but CSN2 and CSN1S1 lacked IRs (Supplementary Table S3). In camel CSN genes, a total of 17 direct repeats (DRs) and 27 aberrant repeats (ERs) were identified. The distribution of DRs in CSN1S1, CSN1S2, CSN2, and CSN3 was 6, 3, 1, and 7, whereas the distribution of ERs was 7, 10, 7, and 3, accordingly (Figure 5 and Supplementary Table S3). The genetic code patterns of *Camelus ferus*, *Bos tararus*, and *Ovis aries*, as well as the CSN genomic family, were screened for possible transcription factor binding sites using four previously disclosed transcription sites (STAT, GATA, TATA, and OCT1) and the YY1 repressor site. Camelus ferus showed a GATA distribution of 999, 82, 903, and 546 corresponding to CSN1S1, CSN1S2, CSN2, and CSN3, while Ovis aries had 1631, 364, 828, and 49 and Bos tarus had 635, 278, 277, and 303, respectively (Figure 6 and Table S4). In addition, the distribution of TATA sites in Camelus ferus was 419, 52, 288, and 336 in CSN1S1, CSN1S2, CSN2, and CSN3, whereas in Ovis aries it was 601, 110, 330, and 12 and in Bos tarus it was 273, 90, 108, and 119, correspondingly. The distribution of OCT1 transcription sites in Camelus ferus was 1260, 144, 979, and 878 for CSN1S1, CSN1S2, CSN2, and CSN3, whereas Ovis aries had 1974, 304, 882, and 26, and Bos tarus had 812, 284, 287, and 357, accordingly. Camelus ferus contained 165, 9, 96, and 67 STAT transcription sites corresponding to CSN1S1, CSN1S2, CSN2, and CSN3, whereas Ovis aries had 185, 31, 139, and 4, and Bos tarus had 71, 20, 43, and 43, accordingly. The pattern of YY1 regulator direct binding in Camelus ferus was 146, 13, 129, and 97 for CSN1S1, CSN1S2, CSN2, and CSN3, while Ovis aries had 237, 51, 133, and 4, and Bos tarus had 89, 34, 36, and 39, correspondingly.

## 4. Discussion

Camel is commonly regarded as the “ship of the desert.” Camel milk is chemically different from cow milk yet provides all vital elements. Camel owners use milk for tea preparation, either raw or boiled. A variety of camel dairy products, including flavored milk, fermented milk, cheese, tea, and coffee, are manufactured and commercially marketed. The level of vitamin C is 1.5 times higher in camel milk than in human milk and three times higher than in cow milk. Dropsy, jaundice, spleen issues, tuberculosis, asthma, anemia, and piles are all diseases that are treated using camel milk [38]. Next-generation sequencing has led to the sequencing of animal genomes, which offers up new techniques to explore genomic architecture at the molecular level. Comparative genomics reveals new genes and their functions. Understanding the regulation mechanisms of physiologically significant genes such as the CSN gene family in mammals requires examining the genetics and evolution [39]. Milk proteins and associated encoding loci have been explored extensively as a food source for newborns due to their widespread distribution in mammalian species. All mammalian CSN genes continuously evolve and are categorized as CSN1S1, CSN2, CSN1S2, and CSN3 [1,2]. Approximately 250 kb of these genes are found on chromosome 6 in cattle and goats [40], and at the genetic level, these genes are distinct [41], transcriptomic [12], and have different protein levels [42]. Additionally, genetic variations and polymorphisms in casein genes are also reported in many species [43], such as goats [44], cattle [45,46,47], and sheep [48], in which cattle and goats have the highest genetic variability. The distinct variations have been associated with varied gene expression and protein biosynthesis rates [44,48]. In addition, recent research suggests that casein gene variations may be associated with milk composition and ratio [49]. In camels, genetic variants were formerly observed for CSN1S1 [50,51], CSN2 [52], and CSN3 [12]. Figure 1 represents a cladistics investigation of typical camelid species having four distinct groups: CSN1S1, CSN2, CSN1S2, and CSN3.

All CSN genes have considerable variance in their aligned sequences, even if closely related species share conserved and non-conserved genomic regions [46,53]. The MEME analysis of CN protein sequences in camels showed 10 common motifs (Figure 2C). After a Pfams analysis, motif 8, including 50 amino acid residues, was identified as the casein kappa (kappa-CN) area, while motifs 2, 5, and 7 were marked as the casein zone (Table 1). Camel casein genes in the same group have a consistent number of exons and introns but different patterns of exons and introns (Figure 2B). Present developments in DNA sequencing technology and engagements in a number of sizeable genome sequencing projects have activated the examination of predictable gene recognition tools. Thus far, broad and greater numbers of sequenced protein coding genes have been described via recognition either of their related cDNAs or homologous genes [54]. 

The current study revealed +ve selection in each CNS group, as well as genomic enrichment assessment for better undersigning of functional properties of the casein family gene sequence. In their findings of a genetic study, [55] analyzed selection and genomic enrichment (Nei-like gene in vertebrate). The idea of systematically connecting a group of genes with a functional biological word was initially proposed by the Gene Ontology (GO) database, which was first published in 2000 [56] (1). The development of GO made it possible to analyze gene lists in light of existing knowledge [57].

Under this advancement, it is pertinent to re-evaluate some of the physical and chemical data by considering expecting new knowledge about results [58]. The application of polymorphism data to pinpoint geographic areas that support recent adaptations has drawn a lot of attention. A straightforward positive selection model, in which a mutation is favored as soon as it occurs, serves as the basis for these searches. This assumption could not be accurate because environmental modifications and range expansions could cause previously harmful or neutral alleles to become positive [59]. In the present investigation, the physicochemical properties of the CSN genes coding in Camelus ferus were determined based on their division according to molecular mass (Da), chromosome position, number of amino acids (AA) in each polypeptide, grand average hydropathy index (GRAVY), instability index (II), isoelectric point (pl), and aliphatic index (AI) (Table 2). Caseins cannot be classified as hydrophobic proteins due to their elastic extended conformation and the presence of the poly-L-proline II secondary protein structure [60]. Similarly, the lower values of the grand mean of the hydrophilicity index expressed the hydrophilic nature of the camel casein proteins. Furthermore, the short phosphorylated pattern and pliable conformation remarkably improve casein’s capacity to keep calcium phosphate nanostructures and to shape a thick protein casing around calcium phosphate to create a thermochemical stable core–shell cluster, given the increased concentration range of calcium and phosphate [61]. In the current investigation, the aliphatic index indicated that all CN proteins had values more than 65, indicating that these are thermally stable. The structure of casein micelles plays an important role in phosphate and calcium transfer from mother to infant with milk [61]. Furthermore, our study showed that isoelectric point (pl) values indicated that all αs2-CN, β-CN, and ķCN casein proteins had peptides with acidic behavior, with the exception of αs1-CN which exhibited basic physical behavior.

Nuclear hormone receptor (NHR) positions in the camel CSN protein were investigated using *Camelus ferus* genomic data sets. A total of 48 NHR positions in the camel CSN gene family were observed. It has become more important to understand the role of nuclear hormone receptors (NHRs) in gene regulation. Ligand-activated transcription factors (LATs) play an important role in the control of cell maintenance, fertility, growth, and diversification. NHRs are part of this superfamily [33,35] and potential repressor sites such as C1S3, YY1, SOCS-1, and SOCS3 [37]. In the present study, *Camelus ferus*, *Bos Tarus*, and *Ovis aries* genome sequences were used to detect the transcriptional binding sites (STAT, GATA, TATA, and OCT1) and the YY1 repressor site on the basis of previous reports. Due to its reduced inhibitory effect in DNA binding and increased expression of the CSN gene, the characteristic of OCT1 is acute myeloid leukemia (AML). STAT is activated by phosphorylation and dimerization and moves to the nucleus, where it binds to DNA and promotes transcription [62]. YY1 uses different mechanisms for transcriptional repression. Most often YY1 is assimilated to activating signals and imbricates the binding position, resulting in repression of gene transcription. The mammary gland factor (MGF) or b-CN transcriptional activator (YY1) in breast endothelial cells is used to characterize transcriptional suppression. In combination with the contiguous YYQ locations, the c-fos promoter has an extra two YY1 locations between both the “TATA box” and the Ca or C- AMP response element (CRE) [63]. In the nucleus, YY1 and CREB work together to stifle transcription [23]. Therefore, cofactor interactions are mostly required with YY1 repression sites to accelerate repression, such as mRPD3 or similar members of its family [64]. By studying how the casein gene family is regulated in depth, we were able to determine that STAT and YY1 localization may be linked to lower-than-expected milk production capacity. This is why we conducted a comparative study of camel, sheep, and cattle to find out which species has a higher number of YY1 repressor sites, because this may be one of the reasons for low milk production. In addition, we describe critical results related to genetic variation in transcriptional activators and the repressor element from an evolutionary perspective. The present study provides fundamental information about the camel casein gene that will be useful for new researchers that study camel milk, which is food for rural populations and newborns and a remedy for many diseases. 

## 5. Conclusions

The casein gene has been studied in many animals, but little information is available on camels. Camel milk is known for its exceptional medical uses. It has been used for centuries to treat newborn diarrhea, hepatitis, IDDM, lactose intolerance, alcohol-induced liver damage, allergies, and autism. It treats numerous disorders, most notably cancer. The current study presents for the first time an extensive understanding of the molecular structure and mechanisms of the casein gene family in *Camelus ferus*, which have been thoroughly studied in the current study, and the results are compelling. A casein gene’s evolutionarily conserved type is explained by the phylogenetics, gene mechanism, and pattern. Versatile, hydrodynamic, and thermostable camel casein proteins were identified. Except for CSN1S1, which exhibited some basic behavior, the CSN1S2, CSN2, and CSN3 peptides behaved as acidic proteins. CSN1S1 showed positive selection for one amino acid (Q), CSN1S2 and CSN2 for three (T, K, and Q), and CSN3 showed no positive selection. When we analyzed high-dairy species such as *Bos tarus* and low-dairy species such as *Ovis aries* compared to *Camelus ferus*, we found that YY1 sites are higher in *Ovis aries* than in *Camelus ferus* and very low in *Bos tarus* and concluded that the ratio of YY1 sites in these species may influence milk production. 

## Figures and Tables

**Table 1:** In the casein protein gene family, ten distinct conserved motifs were identified (CSN1S1, CSN1S2, CSN2, and CSN3).

Motif	Sequence	Length	Pfam Domain
1	MKLLILTCLVAVALARPKEEL	21	----
2	TEVFTKKTKLTEEEKNRLNFLKKISQYYQKFAWPQYLKTVYQYQKAMKPW	50	Casein
3	KFPQYLLPLYQGPIVVPPWDQ	21	----
4	AGEEEESLSSSSEEIVHISKEQKKIQKED	29	----
5	EQLHSMKEGNHAQQKEPMIGVNQELAYFYPELFRQFYQLDAYPSGAWYY	49	Casein
6	EVMGVSKVKETIVPKHKEMPFPKYPVEPFTESQSLTLTDVE	41	-----
7	LPLLQSWMHQPPQPLPPTPMFPPQSLLSLSQAKVLPVPQKAVP	43	Casein
8	MKSFFLVVTILALTLPFLGAQEQNQEQPIRCEKDERFFNDKIAKYIPIQY	50	K-casein
9	PRNALPFQAIPLKEQPDKEEINGLNTIIG	29	----
10	QQQTEDELQDKIHPFPQPQSLVYPYTGPI	29	----

**Table 2:** Physicochemical parameters of the casein gene family in camelids (*Camelus ferus*).

Species	Gene	Chromosome	MW(Da)	AA	pI	AI	II	Gravy
*Camelus ferus*	CSN1S1	2	26,861.4	230	4.96	84.3	64.07	−0.661
*Camelus ferus*	CSN1S2	2	22,964.1	193	6	67.62	58.11	−0.661
*Camelus ferus*	CSN2	2	26,174.81	232	5.43	101.59	97.41	−0.147
*Camelus ferus*	CSN3	2	20,373.53	182	8.44	90.49	44.72	−0.152

## Data Availability

Data are contained within the article or Supplementary Material.

## Supplementary Materials

The following are available online at https://www.mdpi.com/article/10.3390/genes14020256/s1, Table S1. Accession number of selected species for phylogenetic analysis. Table S2. Promoter region in CNS family. Table S3. Nuclear hormone receptor site patterns in the casein gene family of *Camelus ferus.* Table S4: Transcription binding sites (GATA, TATA, STAT, and OCT1) and repressor site (YY1) in the genomic sequences of *Camelus ferus*, *Bos tarus*, and *Ovis aries* casein gene family.
