# Supplementary material for: Molecular Characterization and Phylogenetic Analysis of Casein Gene Family in Camelus ferus
Source: Genes (Basel). 2023 Jan 18;14(2):256. doi: 10.3390/genes14020256 (PMC9957437; doi:10.3390/genes14020256)
Supplement: Supplementary file 1 [file genes-14-00256-s001.zip › genes-2019620-supplementary.pdf]

Table S1. Acession Number of Selected Species for Phylogenetic analysis.

Species Name	CNS1N1	CNS2N1	CSN2	CSN3
Sus scrofa	NP_001004029.1	XP_020956156.1	NP_999599.1	NP_001004026.1
Bubalus bubalis	XP_006071188.1	XP_044801432.1	XP_044801438.1	NP_001277901.1
Bos taurus	XP_005208086.1	NP_776953.1	XP_010804480.2	NP_776719.1
Camelus ferus	XP_006188730.1	XP_006188732.1	XP_032314521.1	XP_006188734.1
Camelus dromedarius	NP_001290495.1	NP_001290490.1	NP_001290492.1	NP_001290489.1
Capra hircus	XP_017904619.1	NP_001272514.1	XP_013820153.1	NP_001272516.1
Ovis aries	XP_012034747.1	NP_001009363.1	NP_001009373.1	NP_001009378.1

Table S2. Promoter region in CNS family.

CSN1S1_Camelus ferus	Position	Score	Likelihood
	6900	1.163	Highly likely prediction
	8300	1.209	Highly likely prediction
	10600	0.717	Marginal prediction
	12000	0.687	Marginal prediction
	13800	1.114	Highly likely prediction
	16000	1.081	Highly likely prediction
**CSN1S2_Camelus ferus**			
	2700	1.191	Highly likely prediction
	5400	0.737	Marginal prediction
	6700	0.701	Marginal prediction
	9800	1.132	Highly likely prediction
	12400	0.705	Marginal prediction
	13100	1.101	Highly likely prediction
**CSN2_Camelus ferus**			
	500	0.703	Marginal prediction
	2300	1.223	Highly likely prediction
	4600	1.144	Highly likely prediction
	6800	0.675	Marginal prediction
	7900	0.589	Marginal prediction
**CSN3_Camelus ferus**			
	3600	1.143	Highly likely prediction

Table S3. Nuclear hormone receptor sites patterns in the casein gene family of *Camelus ferus.*

Site type	Sequence	Start	End	log (Viterbi probabily)	log (Forward probaility)	log (Back-ground state probability)	Forward-Background
	**CSN1S1_Camelus ferus**						
DR2	TGAACTAATGCACT	5969	5982	-20.439	-20.1527	-22.3315	2.1788
DR1	TGAACTCTGAAAT	6129	6141	-20.0489	-19.2623	-20.9351	1.6728
DR1	AGGTCAAAAGTAA	6608	6620	-20.7279	-19.7657	-20.9351	1.1694
DR4	AGTTCAAGTGAGGTAA	8025	8040	-22.8772	-22.7222	-25.1242	2.402
DR2	AGGTCATGAGGTCC	8486	8499	-20.2558	-20.0864	-22.3315	2.2451
DR1	TGACTATTGACCT	14219	14231	-20.5707	-19.2199	-20.9351	1.7152
ER6	TTAAATTAAATGGGTTCA	1028	1045	-27.2404	-26.2097	-27.9168	1.7071
ER6	TTAACTCAAACTAGTTCA	2821	2838	-23.7463	-23.242	-27.9168	4.6748

ER1	TGAACTCAAGGCA	5121	5133	-20.8658	-19.6403	-20.9351	1.2948
ER8	TGAACTGAGCTTCTGGTCAA	12890	12909	-30.2121	-29.4007	-30.7095	1.3088
ER8	TTAACTGTGCATTCAGCTCA	13180	13199	-28.4771	-28.1717	-30.7095	2.5378
ER6	TGAGCTCCAAAGAGTACA	13476	13493	-24.7639	-24.6383	-27.9168	3.2785
ER4	TGACCTTTTAAGTTTA	15034	15049	-23.6185	-23.109	-25.1242	2.0152
**CSN1S2_Camelus ferus**							
DR4	TGTCCTAAGTTGAGCT	247	262	-24.222	-23.47	-25.124	1.6544
DR1	AGTTCAGAAGTCA	1788	1800	-18.978	-18.784	-20.935	2.151
DR0	GGTTCAAAGTCA	9418	9429	-19.169	-18.353	-19.539	1.1858
IR1	GGGTCAATAACCC	578	590	-20.684	-20.024	-20.935	0.9109
IR1	GAGCCAATGGCCT	4825	4837	-20.884	-20.109	-20.935	0.826
IR1	GGGCCACAGTACT	14708	14720	-19.644	-19.393	-20.935	1.5422
ER6	TGAAATATAATAAGTTAA	2154	2171	-25.284	-24.929	-27.917	2.9879
ER8	TTAACTTGTTGTGTAATTCA	2197	2216	-29.699	-28.931	-30.71	1.7785
ER6	TTAACTTCCTTTATTTAA	2373	2390	-27.255	-26.384	-27.917	1.533
ER8	TGAAGTCCCCACAGAGGTAA	5197	5216	-30.461	-29.643	-30.71	1.0667
ER6	TGAAATATGTGTAGTTTA	7925	7942	-26.637	-25.994	-27.917	1.9232
ER8	AGACCTTATCAAAAAGTTAA	8005	8024	-29.291	-28.86	-30.71	1.8494
ER8	TGAAGTTCCCACAGAGGTAA	8293	8312	-30.371	-29.537	-30.71	1.1727
ER8	TGAACTATCTACATAGTCAA	9446	9465	-28.734	-28.325	-30.71	2.385
ER6	TTTACTTCCAGAAGGTAA	10825	10842	-26.677	-26.279	-27.917	1.6375
ER8	AGAACTGACAATTCAGTACA	11861	11880	-29.604	-29.068	-30.71	1.6418
**CSN2_Camelus ferus**							
DR1	TGACCTTTAACCA	3762	3774	-18.9937	-18.5865	-20.9351	2.3486
ER8	TGAACTATGGTATAAGTTAA	1688	1707	-27.0496	-26.5408	-30.7095	4.1687
ER6	TGACCTAGGAACAGAAAA	1780	1797	-27.2834	-26.3376	-27.9168	1.5792
ER6	TTAAATTATCTTAGTTAA	1830	1847	-27.2404	-26.1623	-27.9168	1.7545
ER1	TGACCTTGGTCAA	2897	2909	-20.6327	-19.5318	-20.9351	1.4033
ER8	TGAACTCTACGTTAACCTCA	4329	4348	-29.4232	-28.8108	-30.7095	1.8987
ER6	TTAACTACAATAAGTGAA	6531	6548	-27.2404	-26.3565	-27.9168	1.5603
ER6	TGTCCCTTTCTCAGTTCA	7028	7045	-26.2495	-25.7218	-27.9168	2.195
**CSN3_Camelus ferus**							
DR1	AGTTGAGAGGCCA	215	227	-20.4947	-19.8153	-20.9351	1.1198
DR1	TGACCCCTTAACT	600	612	-20.7052	-19.9071	-20.9351	1.028
DR3	TTAACTCTGTGACCC	1298	1312	-22.4135	-21.9795	-23.7278	1.7483
DR4	TGAACGACTATGACAT	3802	3817	-24.735	-23.9925	-25.1242	1.1317
DR2	TGACTGCCTGACCT	3938	3951	-22.0781	-20.8256	-22.3315	1.5059
DR4	TTCCCTTGTGTGACCT	8455	8470	-23.7854	-23.0836	-25.1242	2.0406
DR4	AGTTCACTATATGTCA	9231	9246	-24.4762	-23.9021	-25.1242	1.2221
IR1	GGTTCACAGGCTT	5604	5616	-20.6559	-20.0319	-20.9351	0.9032
ER6	TAAACTGACTGTATTTCA	4282	4299	-26.726	-26.0827	-27.9168	1.8341
ER6	TTAACCTAGAAAACTTCA	5324	5341	-27.366	-26.6181	-27.9168	1.2987
ER4	TTAACTGTAGAGTTCA	5547	5562	-22.8528	-22.2538	-25.1242	2.8704

Table S4. Transcription binding site (GATA, TATA, STAT, and OCT1) and repressor site (YY1) in the genomic sequences of *Camelus ferus*, *Bos tarus*, *Ovis aries* casein gene family.

**CSN1S1_Camelus ferus**							
**AC**	**ID**	**SCORE**	**LOCATION**	**STRANDS**	**CONSENSUS**	**SEQUENCES**
M0007						

5	V$GATA1_01	0.827246	3	(-)	SNNGATNNNN	GATCATCAAC
M00076	V$GATA2_01	0.797925	59	(-)	NNNGATRNNN	ACATATGGCA
M00075	V$GATA1_01	0.821816	73	(+)	SNNGATNNNN	TATGATGATT
M00076	V$GATA2_01	0.839874	73	(+)	NNNGATRNNN	TATGATGATT
M00075	V$GATA1_01	0.837117	76	(+)	SNNGATNNNN	GATGATTTCA
M00076	V$GATA2_01	0.822282	76	(+)	NNNGATRNNN	GATGATTTCA
M00127	V$GATA1_03	0.812837	112	(-)	RNSNNGATAANNGN	TCATTTATATGCAT
M00126	V$GATA1_02	0.784062	140	(+)	NNNNNGATANKGNN	ATATTGATAACTTG
M00127	V$GATA1_03	0.869917	140	(+)	RNSNNGATAANNGN	ATATTGATAACTTG
M00128	V$GATA1_04	0.817096	141	(+)	NNCWGATARNNNN	TATTGATAACTTG
M00075	V$GATA1_01	0.777394	142	(+)	SNNGATNNNN	ATTGATAACT
M00126	V$GATA1_02	0.8225	183	(+)	NNNNNGATANKGNN	ATGTTGATACTATC
M00127	V$GATA1_03	0.856688	183	(+)	RNSNNGATAANNGN	ATGTTGATACTATC
M00075	V$GATA1_01	0.846002	185	(+)	SNNGATNNNN	GTTGATACTA
M00076	V$GATA2_01	0.797474	185	(+)	NNNGATRNNN	GTTGATACTA
M00126	V$GATA1_02	0.844375	188	(-)	NNNNNGATANKGNN	GATACTATCTATCT
M00127	V$GATA1_03	0.868447	188	(-)	RNSNNGATAANNGN	GATACTATCTATCT
M00075	V$GATA1_01	0.786278	190	(-)	SNNGATNNNN	TACTATCTAT
M00076	V$GATA2_01	0.7871	190	(-)	NNNGATRNNN	TACTATCTAT
M00077	V$GATA3_01	0.902525	190	(-)	NNGATARNG	TACTATCTA
M00203	V$GATA_C	0.853992	191	(-)	NGATAAGNMNN	ACTATCTATCT
M00126	V$GATA1_02	0.8125	192	(-)	NNNNNGATANKGNN	CTATCTATCTTTAG
M00127	V$GATA1_03	0.83268	192	(-)	RNSNNGATAANNGN	CTATCTATCTTTAG
M00128	V$GATA1_04	0.893382	192	(-)	NNCWGATARNNNN	CTATCTATCTTTA
M00075	V$GATA1_01	0.818361	194	(-)	SNNGATNNNN	ATCTATCTTT
M00076	V$GATA2_01	0.858367	194	(-)	NNNGATRNNN	ATCTATCTTT
M00077	V$GATA3_01	0.875055	194	(-)	NNGATARNG	ATCTATCTT
M00075	V$GATA1_01	0.816387	215	(-)	SNNGATNNNN	AAAAATCATA
M00076	V$GATA2_01	0.818674	215	(-)	NNNGATRNNN	AAAAATCATA
M00075	V$GATA1_01	0.804047	234	(-)	SNNGATNNNN	AAACATCAAA
M00075	V$GATA1_01	0.785785	250	(+)	SNNGATNNNN	TTTGATGACT
M00127	V$GATA1_03	0.824841	286	(+)	RNSNNGATAANNGN	AAGTAGATACATAA
M00075	V$GATA1_01	0.821323	288	(+)	SNNGATNNNN	GTAGATACAT

M00076	V$GATA2_01	0.798376	288	(+)	NNNGATRNNN	GTAGATACAT
M00203	V$GATA_C	0.888785	290	(+)	NGATAAGNMNN	AGATACATAAT
M00203	V$GATA_C	0.901833	328	(-)	NGATAAGNMNN	TTGAATTATCT
M00126	V$GATA1_02	0.844375	329	(-)	NNNNNGATANKGNN	TGAATTATCTTTGT
M00127	V$GATA1_03	0.856688	329	(-)	RNSNNGATAANNGN	TGAATTATCTTTGT
M00075	V$GATA1_01	0.792695	331	(-)	SNNGATNNNN	AATTATCTTT
M00076	V$GATA2_01	0.847091	331	(-)	NNNGATRNNN	AATTATCTTT
M00077	V$GATA3_01	0.866637	331	(-)	NNGATARNG	AATTATCTT
M00077	V$GATA3_01	0.864865	377	(-)	NNGATARNG	TATAATCTT
M00126	V$GATA1_02	0.855625	394	(+)	NNNNNGATANKGNN	TCATGGATAAGAGC
M00127	V$GATA1_03	0.808672	394	(+)	RNSNNGATAANNGN	TCATGGATAAGAGC
M00077	V$GATA3_01	0.849801	397	(+)	NNGATARNG	TGGATAAGA
M00203	V$GATA_C	0.941286	398	(+)	NGATAAGNMNN	GGATAAGAGCA
M00126	V$GATA1_02	0.775	471	(+)	NNNNNGATANKGNN	TAAAGGATATGTCA
M00075	V$GATA1_01	0.818855	473	(+)	SNNGATNNNN	AAGGATATGT
M00076	V$GATA2_01	0.882724	473	(+)	NNNGATRNNN	AAGGATATGT
M00076	V$GATA2_01	0.810104	475	(-)	NNNGATRNNN	GGATATGTCA
M00203	V$GATA_C	0.888475	475	(+)	NGATAAGNMNN	GGATATGTCAA
M00126	V$GATA1_02	0.83625	482	(+)	NNNNNGATANKGNN	TCAAAGATAGACAA
M00075	V$GATA1_01	0.803554	484	(+)	SNNGATNNNN	AAAGATAGAC
M00076	V$GATA2_01	0.854759	484	(+)	NNNGATRNNN	AAAGATAGAC
M00077	V$GATA3_01	0.875055	485	(+)	NNGATARNG	AAGATAGAC
M00203	V$GATA_C	0.890028	486	(+)	NGATAAGNMNN	AGATAGACAAA
M00126	V$GATA1_02	0.7875	494	(+)	NNNNNGATANKGNN	AAATAGATATAACT
M00128	V$GATA1_04	0.825368	495	(+)	NNCWGATARNNNN	AATAGATATAACT
M00127	V$GATA1_03	0.791524	496	(+)	RNSNNGATAANNGN	ATAGATATAACTAA
M00077	V$GATA3_01	0.822331	497	(+)	NNGATARNG	TAGATATAA
M00203	V$GATA_C	0.871699	498	(+)	NGATAAGNMNN	AGATATAACTA
M00075	V$GATA1_01	0.857848	520	(+)	SNNGATNNNN	AAAGATGGTT
M00076	V$GATA2_01	0.870095	520	(+)	NNNGATRNNN	AAAGATGGTT
M00077	V$GATA3_01	0.841382	521	(+)	NNGATARNG	AAGATGGTT
M00127	V$GATA1_03	0.820676	524	(+)	RNSNNGATAANNGN	ATGGTTATAGTTGG
M00076	V$GATA2_01	0.823636	534	(+)	NNNGATRNNN	TTGGATATTC

M00076	V$GATA2_01	0.789355	546	(+)	NNNGATRNNN	AAGGATGCAA
M00126	V$GATA1_02	0.781875	554	(+)	NNNNNGATANKGNN	AAGTGGATAAAAGT
M00127	V$GATA1_03	0.89319	554	(+)	RNSNNGATAANNGN	AAGTGGATAAAAGT
M00128	V$GATA1_04	0.820772	555	(+)	NNCWGATARNNNN	AGTGGATAAAAGT
M00203	V$GATA_C	0.898105	558	(+)	NGATAAGNMNN	GGATAAAAGTA
M00075	V$GATA1_01	0.848963	615	(+)	SNNGATNNNN	TCAGATTCTG
M00076	V$GATA2_01	0.862427	615	(+)	NNNGATRNNN	TCAGATTCTG
M00077	V$GATA3_01	0.843155	616	(+)	NNGATARNG	CAGATTCTG
M00126	V$GATA1_02	0.780312	640	(-)	NNNNNGATANKGNN	AACAGTATATATAA
M00076	V$GATA2_01	0.800631	653	(-)	NNNGATRNNN	AAATATGGCG
M00075	V$GATA1_01	0.801086	688	(+)	SNNGATNNNN	TATGATCTGT
M00076	V$GATA2_01	0.824989	688	(+)	NNNGATRNNN	TATGATCTGT
M00075	V$GATA1_01	0.775913	768	(+)	SNNGATNNNN	TTTGATGGAA
M00126	V$GATA1_02	0.80625	790	(+)	NNNNNGATANKGNN	AACATGATATGACA
M00128	V$GATA1_04	0.867953	791	(+)	NNCWGATARNNNN	ACATGATATGACA
M00075	V$GATA1_01	0.877591	792	(+)	SNNGATNNNN	CATGATATGA
M00076	V$GATA2_01	0.868742	792	(+)	NNNGATRNNN	CATGATATGA
M00077	V$GATA3_01	0.863979	793	(+)	NNGATARNG	ATGATATGA
M00203	V$GATA_C	0.89562	794	(+)	NGATAAGNMNN	TGATATGACAT
M00075	V$GATA1_01	0.804541	799	(-)	SNNGATNNNN	TGACATCTGA
M00076	V$GATA2_01	0.863329	799	(-)	NNNGATRNNN	TGACATCTGA
M00075	V$GATA1_01	0.788746	805	(-)	SNNGATNNNN	CTGAATCTTA
M00076	V$GATA2_01	0.814163	805	(-)	NNNGATRNNN	CTGAATCTTA
M00077	V$GATA3_01	0.828977	805	(-)	NNGATARNG	CTGAATCTT
M00075	V$GATA1_01	0.848963	813	(+)	SNNGATNNNN	TATGATTCTG
M00076	V$GATA2_01	0.843031	813	(+)	NNNGATRNNN	TATGATTCTG
M00077	V$GATA3_01	0.825432	814	(+)	NNGATARNG	ATGATTCTG
M00127	V$GATA1_03	0.785154	854	(+)	RNSNNGATAANNGN	ATGGATATACCAAT
M00203	V$GATA_C	0.851196	856	(+)	NGATAAGNMNN	GGATATACCAA
M00126	V$GATA1_02	0.842187	915	(+)	NNNNNGATANKGNN	TGGAAGATAAGTTT
M00127	V$GATA1_03	0.822636	915	(+)	RNSNNGATAANNGN	TGGAAGATAAGTTT
M00128	V$GATA1_04	0.897672	916	(+)	NNCWGATARNNNN	GGAAGATAAGTTT
M00075	V$GATA1_01	0.869694	917	(+)	SNNGATNNNN	GAAGATAAGT

M00076	V$GATA2_01	0.900316	917	(+)	NNNGATRNNN	GAAGATAAGT
M00077	V$GATA3_01	0.887461	918	(+)	NNGATARNG	AAGATAAGT
M00203	V$GATA_C	0.923268	919	(+)	NGATAAGNMNN	AGATAAGTTTC
M00126	V$GATA1_02	0.778125	927	(+)	NNNNNGATANKGNN	TTCTATATAATGCA
M00075	V$GATA1_01	0.882034	974	(+)	SNNGATNNNN	CAGGATGCTC
M00076	V$GATA2_01	0.897158	974	(+)	NNNGATRNNN	CAGGATGCTC
M00077	V$GATA3_01	0.839167	1024	(+)	NNGATARNG	CAGATTAAA
M00075	V$GATA1_01	0.771964	1073	(-)	SNNGATNNNN	ACAAATCTAA
M00203	V$GATA_C	0.858652	1167	(+)	NGATAAGNMNN	TGACAAAACCC
M00126	V$GATA1_02	0.789062	1173	(-)	NNNNNGATANKGNN	AACCCTATTACCAC
M00127	V$GATA1_03	0.787849	1176	(-)	RNSNNGATAANNGN	CCTATTACCACTTC
M00075	V$GATA1_01	0.770977	1213	(+)	SNNGATNNNN	AGTGTTAGTG
M00127	V$GATA1_03	0.795198	1230	(+)	RNSNNGATAANNGN	ATATTAATAAGAGA
M00203	V$GATA_C	0.974216	1249	(-)	NGATAAGNMNN	ATTTCTTATCT
M00126	V$GATA1_02	0.821875	1250	(-)	NNNNNGATANKGNN	TTTCTTATCTCACA
M00128	V$GATA1_04	0.930147	1250	(-)	NNCWGATARNNNN	TTTCTTATCTCAC
M00075	V$GATA1_01	0.806515	1252	(-)	SNNGATNNNN	TCTTATCTCA
M00076	V$GATA2_01	0.864231	1252	(-)	NNNGATRNNN	TCTTATCTCA
M00077	V$GATA3_01	0.936642	1252	(-)	NNGATARNG	TCTTATCTC
M00126	V$GATA1_02	0.7975	1273	(+)	NNNNNGATANKGNN	AGCAAGATAATTTG
M00127	V$GATA1_03	0.914993	1273	(+)	RNSNNGATAANNGN	AGCAAGATAATTTG
M00128	V$GATA1_04	0.85386	1274	(+)	NNCWGATARNNNN	GCAAGATAATTTG
M00075	V$GATA1_01	0.861797	1275	(+)	SNNGATNNNN	CAAGATAATT
M00076	V$GATA2_01	0.888588	1275	(+)	NNNGATRNNN	CAAGATAATT
M00077	V$GATA3_01	0.866637	1276	(+)	NNGATARNG	AAGATAATT
M00075	V$GATA1_01	0.826259	1386	(-)	SNNGATNNNN	GGAAATCAAG
M00075	V$GATA1_01	0.782823	1393	(+)	SNNGATNNNN	AAGGATTTTT
M00076	V$GATA2_01	0.811908	1393	(+)	NNNGATRNNN	AAGGATTTTT
M00203	V$GATA_C	0.884126	1422	(-)	NGATAAGNMNN	TTTTATTATCA
M00126	V$GATA1_02	0.78375	1423	(-)	NNNNNGATANKGNN	TTTATTATCAGACC
M00127	V$GATA1_03	0.823616	1423	(-)	RNSNNGATAANNGN	TTTATTATCAGACC
M00128	V$GATA1_04	0.90625	1423	(-)	NNCWGATARNNNN	TTTATTATCAGAC
M00075	V$GATA1_01	0.807009	1425	(-)	SNNGATNNNN	TATTATCAGA

M00076	V$GATA2_01	0.84664	1425	(-)	NNNGATRNNN	TATTATCAGA
M00077	V$GATA3_01	0.869296	1425	(-)	NNGATARNG	TATTATCAG
M00128	V$GATA1_04	0.821385	1440	(-)	NNCWGATARNNNN	CTCTTTTTCTGTT
M00127	V$GATA1_03	0.784664	1455	(-)	RNSNNGATAANNGN	TTTCTTACCTAGGT
M00127	V$GATA1_03	0.815532	1482	(-)	RNSNNGATAANNGN	AAGCTTCTCATCCT
M00127	V$GATA1_03	0.790544	1485	(-)	RNSNNGATAANNGN	CTTCTCATCCTTAC
M00076	V$GATA2_01	0.809653	1487	(-)	NNNGATRNNN	TCTCATCCTT
M00077	V$GATA3_01	0.820558	1487	(-)	NNGATARNG	TCTCATCCT
M00127	V$GATA1_03	0.843459	1491	(-)	RNSNNGATAANNGN	ATCCTTACCTGCCT
M00128	V$GATA1_04	0.836091	1491	(-)	NNCWGATARNNNN	ATCCTTACCTGCC
M00075	V$GATA1_01	0.788253	1509	(+)	SNNGATNNNN	GCTGTTGCGC
M00075	V$GATA1_01	0.865745	1571	(-)	SNNGATNNNN	TGAAATCACC
M00076	V$GATA2_01	0.830401	1571	(-)	NNNGATRNNN	TGAAATCACC
M00128	V$GATA1_04	0.859988	1578	(+)	NNCWGATARNNNN	ACCTGATATCACA
M00075	V$GATA1_01	0.885982	1579	(+)	SNNGATNNNN	CCTGATATCA
M00076	V$GATA2_01	0.88949	1579	(+)	NNNGATRNNN	CCTGATATCA
M00128	V$GATA1_04	0.861213	1579	(-)	NNCWGATARNNNN	CCTGATATCACAT
M00077	V$GATA3_01	0.821444	1580	(+)	NNGATARNG	CTGATATCA
M00075	V$GATA1_01	0.833169	1581	(-)	SNNGATNNNN	TGATATCACA
M00076	V$GATA2_01	0.880469	1581	(-)	NNNGATRNNN	TGATATCACA
M00077	V$GATA3_01	0.844484	1581	(-)	NNGATARNG	TGATATCAC
M00075	V$GATA1_01	0.780849	1586	(-)	SNNGATNNNN	TCACATCAAA
M00126	V$GATA1_02	0.842812	1596	(+)	NNNNNGATANKGNN	CACAAGATACTGAG
M00127	V$GATA1_03	0.841989	1596	(+)	RNSNNGATAANNGN	CACAAGATACTGAG
M00128	V$GATA1_04	0.829044	1597	(+)	NNCWGATARNNNN	ACAAGATACTGAG
M00075	V$GATA1_01	0.912636	1598	(+)	SNNGATNNNN	CAAGATACTG
M00076	V$GATA2_01	0.92783	1598	(+)	NNNGATRNNN	CAAGATACTG
M00077	V$GATA3_01	0.882588	1599	(+)	NNGATARNG	AAGATACTG
M00127	V$GATA1_03	0.784174	1602	(+)	RNSNNGATAANNGN	ATACTGAGAATAAT
M00127	V$GATA1_03	0.784174	1605	(+)	RNSNNGATAANNGN	CTGAGAATAATAAA
M00076	V$GATA2_01	0.780334	1622	(+)	NNNGATRNNN	TTGGATGATC
M00075	V$GATA1_01	0.845015	1625	(+)	SNNGATNNNN	GATGATCTCT
M00076	V$GATA2_01	0.852052	1625	(+)	NNNGATRNNN	GATGATCTCT

M00076	V$GATA2_01	0.7871	1625	(-)	NNNGATRNNN	GATGATCTCT
M00126	V$GATA1_02	0.807187	1649	(+)	NNNNNGATANKGNN	GTCTTGATATTAAA
M00075	V$GATA1_01	0.82922	1651	(+)	SNNGATNNNN	CTTGATATTA
M00076	V$GATA2_01	0.780785	1651	(+)	NNNGATRNNN	CTTGATATTA
M00076	V$GATA2_01	0.811908	1662	(-)	NNNGATRNNN	AACTATGATA
M00126	V$GATA1_02	0.797187	1663	(+)	NNNNNGATANKGNN	ACTATGATAATCCT
M00127	V$GATA1_03	0.796178	1663	(+)	RNSNNGATAANNGN	ACTATGATAATCCT
M00128	V$GATA1_04	0.8125	1664	(+)	NNCWGATARNNNN	CTATGATAATCCT
M00075	V$GATA1_01	0.807009	1665	(+)	SNNGATNNNN	TATGATAATC
M00076	V$GATA2_01	0.864682	1665	(+)	NNNGATRNNN	TATGATAATC
M00077	V$GATA3_01	0.843155	1666	(+)	NNGATARNG	ATGATAATC
M00203	V$GATA_C	0.882883	1667	(+)	NGATAAGNMNN	TGATAATCCTT
M00076	V$GATA2_01	0.793415	1668	(-)	NNNGATRNNN	GATAATCCTT
M00075	V$GATA1_01	0.780355	1724	(-)	SNNGATNNNN	TAAAATCTCT
M00077	V$GATA3_01	0.850244	1724	(-)	NNGATARNG	TAAAATCTC
M00075	V$GATA1_01	0.776407	1743	(-)	SNNGATNNNN	ATAAATCTCT
M00126	V$GATA1_02	0.800625	1751	(+)	NNNNNGATANKGNN	CTCATGATAAAAAA
M00127	V$GATA1_03	0.855708	1751	(+)	RNSNNGATAANNGN	CTCATGATAAAAAA
M00128	V$GATA1_04	0.892463	1752	(+)	NNCWGATARNNNN	TCATGATAAAAAA
M00075	V$GATA1_01	0.807502	1753	(+)	SNNGATNNNN	CATGATAAAA
M00076	V$GATA2_01	0.807397	1753	(+)	NNNGATRNNN	CATGATAAAA
M00077	V$GATA3_01	0.855117	1754	(+)	NNGATARNG	ATGATAAAA
M00203	V$GATA_C	0.936626	1755	(+)	NGATAAGNMNN	TGATAAAAAAT
M00127	V$GATA1_03	0.784174	1764	(+)	RNSNNGATAANNGN	ATAATAATAATAAT
M00127	V$GATA1_03	0.784174	1767	(+)	RNSNNGATAANNGN	ATAATAATAATAAA
M00126	V$GATA1_02	0.80125	1794	(+)	NNNNNGATANKGNN	GAAGAAATAAGGTA
M00075	V$GATA1_01	0.825271	1803	(-)	SNNGATNNNN	AGGTATCAAA
M00076	V$GATA2_01	0.831755	1803	(-)	NNNGATRNNN	AGGTATCAAA
M00075	V$GATA1_01	0.810464	1825	(-)	SNNGATNNNN	GAGCATCAAA
M00076	V$GATA2_01	0.780785	1825	(-)	NNNGATRNNN	GAGCATCAAA
M00075	V$GATA1_01	0.820336	1856	(-)	SNNGATNNNN	CTGCATCCGT
M00076	V$GATA2_01	0.840325	1856	(-)	NNNGATRNNN	CTGCATCCGT
M00126	V$GATA1_02	0.772813	1939	(-)	NNNNNGATANKGNN	TCCAGTATTTTCTA

M00075	V$GATA1_01	0.847976	1980	(+)	SNNGATNNNN	TGAGATGTCT
M00076	V$GATA2_01	0.893099	1980	(+)	NNNGATRNNN	TGAGATGTCT
M00127	V$GATA1_03	0.784174	2013	(+)	RNSNNGATAANNGN	GTATAGAAAATCAG
M00075	V$GATA1_01	0.883021	2018	(-)	SNNGATNNNN	GAAAATCAGG
M00076	V$GATA2_01	0.832206	2018	(-)	NNNGATRNNN	GAAAATCAGG
M00075	V$GATA1_01	0.917078	2036	(+)	SNNGATNNNN	GCTGATTTGG
M00076	V$GATA2_01	0.860622	2036	(+)	NNNGATRNNN	GCTGATTTGG
M00077	V$GATA3_01	0.859548	2037	(+)	NNGATARNG	CTGATTTGG
M00075	V$GATA1_01	0.852419	2044	(+)	SNNGATNNNN	GGAGATTGAA
M00076	V$GATA2_01	0.819125	2044	(+)	NNNGATRNNN	GGAGATTGAA
M00077	V$GATA3_01	0.890563	2045	(+)	NNGATARNG	GAGATTGAA
M00126	V$GATA1_02	0.860625	2080	(+)	NNNNNGATANKGNN	AATTAGATAATGTA
M00127	V$GATA1_03	0.78295	2080	(+)	RNSNNGATAANNGN	AATTAGATAATGTA
M00128	V$GATA1_04	0.83701	2081	(+)	NNCWGATARNNNN	ATTAGATAATGTA
M00075	V$GATA1_01	0.776407	2082	(+)	SNNGATNNNN	TTAGATAATG
M00076	V$GATA2_01	0.811006	2082	(+)	NNNGATRNNN	TTAGATAATG
M00077	V$GATA3_01	0.909171	2083	(+)	NNGATARNG	TAGATAATG
M00203	V$GATA_C	0.863312	2084	(+)	NGATAAGNMNN	AGATAATGTAA
M00126	V$GATA1_02	0.779687	2104	(-)	NNNNNGATANKGNN	ACCCTTCTCAAAAA
M00075	V$GATA1_01	0.835637	2175	(+)	SNNGATNNNN	TGTGATGCAC
M00076	V$GATA2_01	0.830401	2175	(+)	NNNGATRNNN	TGTGATGCAC
M00126	V$GATA1_02	0.781875	2181	(+)	NNNNNGATANKGNN	GCACAGCTAGTGCA
M00126	V$GATA1_02	0.78625	2192	(+)	NNNNNGATANKGNN	GCAGTGAAAGGAGT
M00075	V$GATA1_01	0.922507	2210	(-)	SNNGATNNNN	AGGAATCACC
M00076	V$GATA2_01	0.871448	2210	(-)	NNNGATRNNN	AGGAATCACC
M00128	V$GATA1_04	0.810662	2217	(+)	NNCWGATARNNNN	ACCTGACAAGAAA
M00203	V$GATA_C	0.871078	2220	(+)	NGATAAGNMNN	TGACAAGAAAA
M00127	V$GATA1_03	0.784174	2221	(+)	RNSNNGATAANNGN	GACAAGAAAACAAT
M00126	V$GATA1_02	0.805313	2233	(+)	NNNNNGATANKGNN	ATTCAGATAAAATA
M00127	V$GATA1_03	0.790299	2233	(+)	RNSNNGATAANNGN	ATTCAGATAAAATA
M00128	V$GATA1_04	0.896446	2234	(+)	NNCWGATARNNNN	TTCAGATAAAATA
M00076	V$GATA2_01	0.817772	2235	(+)	NNNGATRNNN	TCAGATAAAA
M00077	V$GATA3_01	0.87284	2236	(+)	NNGATARNG	CAGATAAAA

M00203	V$GATA_C	0.903075	2237	(+)	NGATAAGNMNN	AGATAAAATAA
M00075	V$GATA1_01	0.800099	2253	(+)	SNNGATNNNN	TTGGATGCTT
M00076	V$GATA2_01	0.810104	2253	(+)	NNNGATRNNN	TTGGATGCTT
M00075	V$GATA1_01	0.790227	2261	(+)	SNNGATNNNN	TTGGATATTG
M00076	V$GATA2_01	0.840325	2261	(+)	NNNGATRNNN	TTGGATATTG
M00077	V$GATA3_01	0.832078	2262	(+)	NNGATARNG	TGGATATTG
M00126	V$GATA1_02	0.8125	2270	(-)	NNNNNGATANKGNN	GAAACTATCAAATA
M00075	V$GATA1_01	0.850938	2272	(-)	SNNGATNNNN	AACTATCAAA
M00076	V$GATA2_01	0.838069	2272	(-)	NNNGATRNNN	AACTATCAAA
M00077	V$GATA3_01	0.847142	2272	(-)	NNGATARNG	AACTATCAA
M00075	V$GATA1_01	0.777887	2283	(+)	SNNGATNNNN	ACAGATGCAA
M00076	V$GATA2_01	0.78304	2283	(+)	NNNGATRNNN	ACAGATGCAA
M00126	V$GATA1_02	0.828125	2304	(+)	NNNNNGATANKGNN	GCTGAGATACTCTA
M00075	V$GATA1_01	0.854393	2306	(+)	SNNGATNNNN	TGAGATACTC
M00076	V$GATA2_01	0.910239	2306	(+)	NNNGATRNNN	TGAGATACTC
M00077	V$GATA3_01	0.832964	2307	(+)	NNGATARNG	GAGATACTC
M00127	V$GATA1_03	0.784664	2325	(-)	RNSNNGATAANNGN	CTAATTTTCTATAT
M00203	V$GATA_C	0.86238	2330	(-)	NGATAAGNMNN	TTTCTATATCC
M00075	V$GATA1_01	0.791214	2355	(+)	SNNGATNNNN	CAGGATCACC
M00075	V$GATA1_01	0.906219	2355	(-)	SNNGATNNNN	CAGGATCACC
M00076	V$GATA2_01	0.853857	2355	(+)	NNNGATRNNN	CAGGATCACC
M00076	V$GATA2_01	0.865584	2355	(-)	NNNGATRNNN	CAGGATCACC
M00127	V$GATA1_03	0.83366	2375	(-)	RNSNNGATAANNGN	CTTCTTAACTTCTT
M00128	V$GATA1_04	0.813113	2375	(-)	NNCWGATARNNNN	CTTCTTAACTTCT
M00075	V$GATA1_01	0.815893	2430	(-)	SNNGATNNNN	AGAAATCTCT
M00076	V$GATA2_01	0.824538	2430	(-)	NNNGATRNNN	AGAAATCTCT
M00126	V$GATA1_02	0.781875	2476	(-)	NNNNNGATANKGNN	AAATGTATCATTGG
M00075	V$GATA1_01	0.813425	2478	(-)	SNNGATNNNN	ATGTATCATT
M00076	V$GATA2_01	0.821831	2478	(-)	NNNGATRNNN	ATGTATCATT
M00203	V$GATA_C	0.99503	2493	(-)	NGATAAGNMNN	AGTCCTTATCT
M00126	V$GATA1_02	0.873437	2494	(-)	NNNNNGATANKGNN	GTCCTTATCTTGTT
M00127	V$GATA1_03	0.930181	2494	(-)	RNSNNGATAANNGN	GTCCTTATCTTGTT
M00128	V$GATA1_04	0.962316	2494	(-)	NNCWGATARNNNN	GTCCTTATCTTGT

M00075	V$GATA1_01	0.885489	2496	(-)	SNNGATNNNN	CCTTATCTTG
M00076	V$GATA2_01	0.907984	2496	(-)	NNNGATRNNN	CCTTATCTTG
M00077	V$GATA3_01	0.954364	2496	(-)	NNGATARNG	CCTTATCTT
M00075	V$GATA1_01	0.85538	2579	(-)	SNNGATNNNN	CCACATCATT
M00076	V$GATA2_01	0.841678	2579	(-)	NNNGATRNNN	CCACATCATT
M00077	V$GATA3_01	0.84537	2579	(-)	NNGATARNG	CCACATCAT
M00075	V$GATA1_01	0.870681	2638	(+)	SNNGATNNNN	GAAGATTGAG
M00076	V$GATA2_01	0.853857	2638	(+)	NNNGATRNNN	GAAGATTGAG
M00077	V$GATA3_01	0.908285	2639	(+)	NNGATARNG	AAGATTGAG
M00126	V$GATA1_02	0.791562	2642	(+)	NNNNNGATANKGNN	ATTGAGATAAATTT
M00127	V$GATA1_03	0.78099	2642	(+)	RNSNNGATAANNGN	ATTGAGATAAATTT
M00128	V$GATA1_04	0.872549	2643	(+)	NNCWGATARNNNN	TTGAGATAAATTT
M00075	V$GATA1_01	0.790227	2644	(+)	SNNGATNNNN	TGAGATAAAT
M00076	V$GATA2_01	0.847542	2644	(+)	NNNGATRNNN	TGAGATAAAT
M00077	V$GATA3_01	0.863979	2645	(+)	NNGATARNG	GAGATAAAT
M00203	V$GATA_C	0.907735	2646	(+)	NGATAAGNMNN	AGATAAATTTT
M00126	V$GATA1_02	0.826875	2669	(+)	NNNNNGATANKGNN	TTGGTGATAGAAAT
M00127	V$GATA1_03	0.78197	2669	(+)	RNSNNGATAANNGN	TTGGTGATAGAAAT
M00128	V$GATA1_04	0.88511	2670	(+)	NNCWGATARNNNN	TGGTGATAGAAAT
M00075	V$GATA1_01	0.896841	2671	(+)	SNNGATNNNN	GGTGATAGAA
M00076	V$GATA2_01	0.87235	2671	(+)	NNNGATRNNN	GGTGATAGAA
M00077	V$GATA3_01	0.900753	2672	(+)	NNGATARNG	GTGATAGAA
M00203	V$GATA_C	0.885368	2673	(+)	NGATAAGNMNN	TGATAGAAATA
M00075	V$GATA1_01	0.780355	2682	(-)	SNNGATNNNN	TAAAATCCAG
M00127	V$GATA1_03	0.823861	2686	(+)	RNSNNGATAANNGN	ATCCAGACAAGCAA
M00203	V$GATA_C	0.867971	2690	(+)	NGATAAGNMNN	AGACAAGCAAC
M00075	V$GATA1_01	0.779862	2795	(-)	SNNGATNNNN	CTAAATCATT
M00077	V$GATA3_01	0.824546	2795	(-)	NNGATARNG	CTAAATCAT
M00203	V$GATA_C	0.937558	2803	(-)	NGATAAGNMNN	TTTCTTTATCA
M00126	V$GATA1_02	0.804375	2804	(-)	NNNNNGATANKGNN	TTCTTTATCATTCT
M00127	V$GATA1_03	0.882166	2804	(-)	RNSNNGATAANNGN	TTCTTTATCATTCT
M00128	V$GATA1_04	0.904718	2804	(-)	NNCWGATARNNNN	TTCTTTATCATTC
M00075	V$GATA1_01	0.785291	2806	(-)	SNNGATNNNN	CTTTATCATT

M00076	V$GATA2_01	0.808751	2806	(-)	NNNGATRNNN	CTTTATCATT
M00077	V$GATA3_01	0.89012	2806	(-)	NNGATARNG	CTTTATCAT
M00076	V$GATA2_01	0.80018	2891	(+)	NNNGATRNNN	CACCATACTC
M00077	V$GATA3_01	0.82366	2903	(+)	NNGATARNG	AAGAGAGGA
M00203	V$GATA_C	0.847779	2928	(-)	NGATAAGNMNN	AATTTTTGTCA
M00075	V$GATA1_01	0.779862	2979	(+)	SNNGATNNNN	ACAGATTTAG
M00077	V$GATA3_01	0.842268	2980	(+)	NNGATARNG	CAGATTTAG
M00075	V$GATA1_01	0.781836	3049	(+)	SNNGATNNNN	TGTGATCTTA
M00076	V$GATA2_01	0.797023	3049	(+)	NNNGATRNNN	TGTGATCTTA
M00076	V$GATA2_01	0.80424	3049	(-)	NNNGATRNNN	TGTGATCTTA
M00127	V$GATA1_03	0.782215	3052	(-)	RNSNNGATAANNGN	GATCTTATTATTAT
M00127	V$GATA1_03	0.784174	3055	(-)	RNSNNGATAANNGN	CTTATTATTATTGT
M00075	V$GATA1_01	0.827246	3071	(+)	SNNGATNNNN	TTTGATTGCT
M00076	V$GATA2_01	0.792512	3071	(+)	NNNGATRNNN	TTTGATTGCT
M00075	V$GATA1_01	0.871175	3128	(-)	SNNGATNNNN	AAATATCCTC
M00076	V$GATA2_01	0.92332	3128	(-)	NNNGATRNNN	AAATATCCTC
M00075	V$GATA1_01	0.772458	3133	(-)	SNNGATNNNN	TCCTCTCAGG
M00076	V$GATA2_01	0.793866	3173	(-)	NNNGATRNNN	GTCTATGTTC
M00126	V$GATA1_02	0.822188	3216	(-)	NNNNNGATANKGNN	TGGTGTATCGGTAA
M00075	V$GATA1_01	0.813425	3218	(-)	SNNGATNNNN	GTGTATCGGT
M00076	V$GATA2_01	0.834461	3218	(-)	NNNGATRNNN	GTGTATCGGT
M00126	V$GATA1_02	0.850938	3253	(-)	NNNNNGATANKGNN	TCCCCTCTCTATAA
M00077	V$GATA3_01	0.834293	3255	(-)	NNGATARNG	CCCTCTCTA
M00126	V$GATA1_02	0.813438	3290	(-)	NNNNNGATANKGNN	TATAATATCTTCAG
M00075	V$GATA1_01	0.840079	3292	(-)	SNNGATNNNN	TAATATCTTC
M00076	V$GATA2_01	0.879116	3292	(-)	NNNGATRNNN	TAATATCTTC
M00077	V$GATA3_01	0.866637	3292	(-)	NNGATARNG	TAATATCTT
M00075	V$GATA1_01	0.774926	3319	(-)	SNNGATNNNN	GAATATCGAT
M00075	V$GATA1_01	0.852419	3323	(-)	SNNGATNNNN	ATCGATCTGC
M00076	V$GATA2_01	0.848895	3323	(-)	NNNGATRNNN	ATCGATCTGC
M00075	V$GATA1_01	0.866239	3340	(-)	SNNGATNNNN	AACCATCTTA
M00076	V$GATA2_01	0.902571	3340	(-)	NNNGATRNNN	AACCATCTTA
M00077	V$GATA3_01	0.841382	3340	(-)	NNGATARNG	AACCATCTT

M00076	V$GATA2_01	0.801985	3358	(+)	NNNGATRNNN	GCACATAGAC
M00126	V$GATA1_02	0.789375	3418	(+)	NNNNNGATANKGNN	TTAAGGATATGTTA
M00075	V$GATA1_01	0.818855	3420	(+)	SNNGATNNNN	AAGGATATGT
M00076	V$GATA2_01	0.882724	3420	(+)	NNNGATRNNN	AAGGATATGT
M00076	V$GATA2_01	0.801985	3422	(-)	NNNGATRNNN	GGATATGTTA
M00127	V$GATA1_03	0.782215	3425	(-)	RNSNNGATAANNGN	TATGTTAGCATGAT
M00075	V$GATA1_01	0.837611	3433	(+)	SNNGATNNNN	CATGATTTAT
M00076	V$GATA2_01	0.787551	3433	(+)	NNNGATRNNN	CATGATTTAT
M00127	V$GATA1_03	0.791769	3457	(-)	RNSNNGATAANNGN	GCTTCTTTCTAGCT
M00075	V$GATA1_01	0.845015	3522	(-)	SNNGATNNNN	CTGAATCTTC
M00076	V$GATA2_01	0.828597	3522	(-)	NNNGATRNNN	CTGAATCTTC
M00077	V$GATA3_01	0.828977	3522	(-)	NNGATARNG	CTGAATCTT
M00076	V$GATA2_01	0.819576	3625	(+)	NNNGATRNNN	CAGCATAGAG
M00127	V$GATA1_03	0.794708	3654	(-)	RNSNNGATAANNGN	CCAGCTATTCTCTC
M00127	V$GATA1_03	0.814307	3657	(-)	RNSNNGATAANNGN	GCTATTCTCTCTTT
M00076	V$GATA2_01	0.798376	3670	(+)	NNNGATRNNN	TCAGTTAGCT
M00076	V$GATA2_01	0.839874	3676	(-)	NNNGATRNNN	AGCTATGATG
M00075	V$GATA1_01	0.821816	3679	(+)	SNNGATNNNN	TATGATGACT
M00076	V$GATA2_01	0.858818	3679	(+)	NNNGATRNNN	TATGATGACT
M00127	V$GATA1_03	0.822636	3708	(-)	RNSNNGATAANNGN	TCATTTATTTTCCT
M00127	V$GATA1_03	0.782705	3724	(-)	RNSNNGATAANNGN	AATTGTATCCAGGT
M00075	V$GATA1_01	0.785785	3726	(-)	SNNGATNNNN	TTGTATCCAG
M00075	V$GATA1_01	0.783317	3790	(-)	SNNGATNNNN	TTCAATCCCT
M00076	V$GATA2_01	0.786649	3790	(-)	NNNGATRNNN	TTCAATCCCT
M00077	V$GATA3_01	0.84537	3790	(-)	NNGATARNG	TTCAATCCC
M00126	V$GATA1_02	0.79	3807	(+)	NNNNNGATANKGNN	CATTTGATAAGTAT
M00128	V$GATA1_04	0.906863	3808	(+)	NNCWGATARNNNN	ATTTGATAAGTAT
M00075	V$GATA1_01	0.798124	3809	(+)	SNNGATNNNN	TTTGATAAGT
M00076	V$GATA2_01	0.792963	3809	(+)	NNNGATRNNN	TTTGATAAGT
M00077	V$GATA3_01	0.83961	3810	(+)	NNGATARNG	TTGATAAGT
M00203	V$GATA_C	0.9242	3811	(+)	NGATAAGNMNN	TGATAAGTATG
M00076	V$GATA2_01	0.797925	3815	(-)	NNNGATRNNN	AAGTATGTTA
M00076	V$GATA2_01	0.783491	3856	(-)	NNNGATRNNN	AAATATGGTA

M00126	V$GATA1_02	0.793125	4020	(-)	NNNNNGATANKGNN	TTTAATATCTACAG
M00127	V$GATA1_03	0.787604	4020	(-)	RNSNNGATAANNGN	TTTAATATCTACAG
M00075	V$GATA1_01	0.804047	4022	(-)	SNNGATNNNN	TAATATCTAC
M00076	V$GATA2_01	0.797474	4022	(-)	NNNGATRNNN	TAATATCTAC
M00077	V$GATA3_01	0.842268	4022	(-)	NNGATARNG	TAATATCTA
M00075	V$GATA1_01	0.80849	4051	(+)	SNNGATNNNN	AAAGATTCTT
M00076	V$GATA2_01	0.808751	4051	(+)	NNNGATRNNN	AAAGATTCTT
M00203	V$GATA_C	0.889407	4075	(-)	NGATAAGNMNN	AAGTGTTATCC
M00127	V$GATA1_03	0.879716	4076	(-)	RNSNNGATAANNGN	AGTGTTATCCACTT
M00075	V$GATA1_01	0.781343	4078	(-)	SNNGATNNNN	TGTTATCCAC
M00076	V$GATA2_01	0.815968	4078	(-)	NNNGATRNNN	TGTTATCCAC
M00127	V$GATA1_03	0.828025	4084	(-)	RNSNNGATAANNGN	CCACTTATTCTTCT
M00075	V$GATA1_01	0.836624	4119	(-)	SNNGATNNNN	AAAAATCAAC
M00076	V$GATA2_01	0.780785	4125	(+)	NNNGATRNNN	CAACATAATT
M00075	V$GATA1_01	0.801579	4213	(-)	SNNGATNNNN	TGAAATCAGA
M00076	V$GATA2_01	0.815968	4213	(-)	NNNGATRNNN	TGAAATCAGA
M00127	V$GATA1_03	0.805977	4216	(+)	RNSNNGATAANNGN	AATCAGATAAACAA
M00128	V$GATA1_04	0.934743	4217	(+)	NNCWGATARNNNN	ATCAGATAAACAA
M00076	V$GATA2_01	0.843933	4218	(+)	NNNGATRNNN	TCAGATAAAC
M00077	V$GATA3_01	0.840939	4219	(+)	NNGATARNG	CAGATAAAC
M00203	V$GATA_C	0.932588	4220	(+)	NGATAAGNMNN	AGATAAACAAA
M00126	V$GATA1_02	0.788125	4324	(+)	NNNNNGATANKGNN	TTCATTATAATGGA
M00203	V$GATA_C	0.943771	4340	(-)	NGATAAGNMNN	GTTCTTTATCT
M00126	V$GATA1_02	0.863438	4341	(-)	NNNNNGATANKGNN	TTCTTTATCTCTAG
M00127	V$GATA1_03	0.849339	4341	(-)	RNSNNGATAANNGN	TTCTTTATCTCTAG
M00128	V$GATA1_04	0.904412	4341	(-)	NNCWGATARNNNN	TTCTTTATCTCTA
M00075	V$GATA1_01	0.793189	4343	(-)	SNNGATNNNN	CTTTATCTCT
M00076	V$GATA2_01	0.828146	4343	(-)	NNNGATRNNN	CTTTATCTCT
M00077	V$GATA3_01	0.930882	4343	(-)	NNGATARNG	CTTTATCTC
M00126	V$GATA1_02	0.859063	4349	(+)	NNNNNGATANKGNN	CTCTAGATAATACC
M00127	V$GATA1_03	0.865507	4349	(+)	RNSNNGATAANNGN	CTCTAGATAATACC
M00128	V$GATA1_04	0.851409	4350	(+)	NNCWGATARNNNN	TCTAGATAATACC
M00075	V$GATA1_01	0.790227	4351	(+)	SNNGATNNNN	CTAGATAATA

M00077	V$GATA3_01	0.874169	4352	(+)	NNGATARNG	TAGATAATA
M00203	V$GATA_C	0.909289	4353	(+)	NGATAAGNMNN	AGATAATACCC
M00126	V$GATA1_02	0.77875	4361	(-)	NNNNNGATANKGNN	CCCACTATGTGTTG
M00076	V$GATA2_01	0.811908	4363	(-)	NNNGATRNNN	CACTATGTGT
M00203	V$GATA_C	0.845915	4395	(-)	NGATAAGNMNN	CTGACTTGTCA
M00126	V$GATA1_02	0.834688	4451	(+)	NNNNNGATANKGNN	TATTTGATAGGCAA
M00128	V$GATA1_04	0.873162	4452	(+)	NNCWGATARNNNN	ATTTGATAGGCAA
M00075	V$GATA1_01	0.84847	4453	(+)	SNNGATNNNN	TTTGATAGGC
M00076	V$GATA2_01	0.840776	4453	(+)	NNNGATRNNN	TTTGATAGGC
M00077	V$GATA3_01	0.867966	4454	(+)	NNGATARNG	TTGATAGGC
M00203	V$GATA_C	0.90028	4455	(+)	NGATAAGNMNN	TGATAGGCAAC
M00075	V$GATA1_01	0.807996	4479	(+)	SNNGATNNNN	AATGATTTTT
M00076	V$GATA2_01	0.786198	4479	(+)	NNNGATRNNN	AATGATTTTT
M00076	V$GATA2_01	0.7871	4488	(+)	NNNGATRNNN	TAACATAACC
M00075	V$GATA1_01	0.773445	4513	(-)	SNNGATNNNN	AGGTTTCACC
M00127	V$GATA1_03	0.810632	4533	(+)	RNSNNGATAANNGN	AGACAGGTAAGAAA
M00203	V$GATA_C	0.830693	4537	(+)	NGATAAGNMNN	AGGTAAGAAAT
M00128	V$GATA1_04	0.823836	4553	(+)	NNCWGATARNNNN	ACAAGATATACTG
M00075	V$GATA1_01	0.825765	4554	(+)	SNNGATNNNN	CAAGATATAC
M00076	V$GATA2_01	0.85972	4554	(+)	NNNGATRNNN	CAAGATATAC
M00127	V$GATA1_03	0.784174	4565	(+)	RNSNNGATAANNGN	GTAGAGTTAACAAA
M00127	V$GATA1_03	0.800833	4590	(-)	RNSNNGATAANNGN	TTTGTTCTCTCCAG
M00075	V$GATA1_01	0.774926	4614	(+)	SNNGATNNNN	GGTGTTGTGC
M00127	V$GATA1_03	0.824841	4644	(+)	RNSNNGATAANNGN	ATGGAAATAAACAC
M00126	V$GATA1_02	0.8375	4669	(+)	NNNNNGATANKGNN	AACCAGATAGATGA
M00127	V$GATA1_03	0.869672	4669	(+)	RNSNNGATAANNGN	AACCAGATAGATGA
M00128	V$GATA1_04	0.918199	4670	(+)	NNCWGATARNNNN	ACCAGATAGATGA
M00075	V$GATA1_01	0.908193	4671	(+)	SNNGATNNNN	CCAGATAGAT
M00076	V$GATA2_01	0.907984	4671	(+)	NNNGATRNNN	CCAGATAGAT
M00077	V$GATA3_01	0.869296	4672	(+)	NNGATARNG	CAGATAGAT
M00127	V$GATA1_03	0.785644	4673	(+)	RNSNNGATAANNGN	AGATAGATGAGATC
M00203	V$GATA_C	0.842187	4673	(+)	NGATAAGNMNN	AGATAGATGAG
M00077	V$GATA3_01	0.841382	4676	(+)	NNGATARNG	TAGATGAGA

M0007 5	V$GATA1_01	0.777887	4680	(-)	SNNGATNNNN	TGAGATCAAG
M0007 6	V$GATA2_01	0.796121	4680	(+)	NNNGATRNNN	TGAGATCAAG
M0012 6	V$GATA1_02	0.850937	4718	(+)	NNNNNGATANKGNN	TGAAAGATAAGAAC
M0012 7	V$GATA1_03	0.840274	4718	(+)	RNSNNGATAANNGN	TGAAAGATAAGAAC
M0012 8	V$GATA1_04	0.938113	4719	(+)	NNCWGATARNNNN	GAAAGATAAGAAC
M0007 6	V$GATA2_01	0.823636	4720	(+)	NNNGATRNNN	AAAGATAAGA
M0007 7	V$GATA3_01	0.919362	4721	(+)	NNGATARNG	AAGATAAGA
M0020 3	V$GATA_C	0.982293	4722	(+)	NGATAAGNMNN	AGATAAGAACA
M0012 6	V$GATA1_02	0.825625	4830	(+)	NNNNNGATANKGNN	GAAATGATATAGCA
M0012 8	V$GATA1_04	0.854167	4831	(+)	NNCWGATARNNNN	AAATGATATAGCA
M0007 5	V$GATA1_01	0.803554	4832	(+)	SNNGATNNNN	AATGATATAG
M0007 6	V$GATA2_01	0.823636	4832	(+)	NNNGATRNNN	AATGATATAG
M0007 7	V$GATA3_01	0.858219	4833	(+)	NNGATARNG	ATGATATAG
M0020 3	V$GATA_C	0.870146	4834	(+)	NGATAAGNMNN	TGATATAGCAA
M0007 6	V$GATA2_01	0.784393	4904	(-)	NNNGATRNNN	AGGTATGTTT
M0007 6	V$GATA2_01	0.79567	4918	(+)	NNNGATRNNN	ATAGATATCC
M0007 5	V$GATA1_01	0.770484	4920	(-)	SNNGATNNNN	AGATATCCAT
M0007 6	V$GATA2_01	0.813712	4920	(-)	NNNGATRNNN	AGATATCCAT
M0007 5	V$GATA1_01	0.774926	4983	(-)	SNNGATNNNN	GAACATCGAT
M0012 7	V$GATA1_03	0.817001	4996	(+)	RNSNNGATAANNGN	CTGAAGGTAAGGAC
M0012 8	V$GATA1_04	0.811275	4997	(+)	NNCWGATARNNNN	TGAAGGTAAGGAC
M0020 3	V$GATA_C	0.840634	5000	(+)	NGATAAGNMNN	AGGTAAGGACC
M0007 5	V$GATA1_01	0.827739	5007	(-)	SNNGATNNNN	GACCATCAAT
M0012 6	V$GATA1_02	0.775625	5012	(+)	NNNNNGATANKGNN	TCAATGATATTTAA
M0007 5	V$GATA1_01	0.831688	5014	(+)	SNNGATNNNN	AATGATATTT
M0007 6	V$GATA2_01	0.850699	5014	(+)	NNNGATRNNN	AATGATATTT
M0012 6	V$GATA1_02	0.862812	5144	(+)	NNNNNGATANKGNN	CTTAAGATAATGTG
M0012 7	V$GATA1_03	0.798628	5144	(+)	RNSNNGATAANNGN	CTTAAGATAATGTG
M0012 8	V$GATA1_04	0.877757	5145	(+)	NNCWGATARNNNN	TTAAGATAATGTG
M0007 5	V$GATA1_01	0.812438	5146	(+)	SNNGATNNNN	TAAGATAATG
M0007 6	V$GATA2_01	0.892648	5146	(+)	NNNGATRNNN	TAAGATAATG
M0007 7	V$GATA3_01	0.93354	5147	(+)	NNGATARNG	AAGATAATG
M0012 6	V$GATA1_02	0.809375	5179	(+)	NNNNNGATANKGNN	GTAGAGAAAGTGTT

M00203	V$GATA_C	0.860516	5208	(-)	NGATAAGNMNN	AATATTTGTCT
M00075	V$GATA1_01	0.859329	5254	(+)	SNNGATNNNN	GCTGATGAAT
M00076	V$GATA2_01	0.822282	5254	(+)	NNNGATRNNN	GCTGATGAAT
M00126	V$GATA1_02	0.80125	5314	(+)	NNNNNGATANKGNN	GAAGAGACAGGGCC
M00203	V$GATA_C	0.871389	5318	(+)	NGATAAGNMNN	AGACAGGGCCT
M00126	V$GATA1_02	0.781563	5371	(+)	NNNNNGATANKGNN	TTATAGACAGTTGT
M00127	V$GATA1_03	0.828025	5377	(-)	RNSNNGATAANNGN	ACAGTTGTCTGCAC
M00126	V$GATA1_02	0.807187	5390	(-)	NNNNNGATANKGNN	CTTAGTATCAAAAC
M00075	V$GATA1_01	0.789733	5392	(-)	SNNGATNNNN	TAGTATCAAA
M00076	V$GATA2_01	0.78304	5392	(-)	NNNGATRNNN	TAGTATCAAA
M00126	V$GATA1_02	0.842812	5438	(-)	NNNNNGATANKGNN	CAACGTATCTGCTC
M00127	V$GATA1_03	0.847379	5438	(-)	RNSNNGATAANNGN	CAACGTATCTGCTC
M00128	V$GATA1_04	0.846507	5438	(-)	NNCWGATARNNNN	CAACGTATCTGCT
M00075	V$GATA1_01	0.929911	5440	(-)	SNNGATNNNN	ACGTATCTGC
M00076	V$GATA2_01	0.934596	5440	(-)	NNNGATRNNN	ACGTATCTGC
M00077	V$GATA3_01	0.830749	5440	(-)	NNGATARNG	ACGTATCTG
M00127	V$GATA1_03	0.800833	5473	(-)	RNSNNGATAANNGN	GTTATTATTCACTT
M00075	V$GATA1_01	0.807996	5495	(+)	SNNGATNNNN	AATGATTTTT
M00076	V$GATA2_01	0.786198	5495	(+)	NNNGATRNNN	AATGATTTTT
M00075	V$GATA1_01	0.795656	5504	(-)	SNNGATNNNN	TTGGATCTGG
M00076	V$GATA2_01	0.782138	5504	(+)	NNNGATRNNN	TTGGATCTGG
M00076	V$GATA2_01	0.788453	5504	(-)	NNNGATRNNN	TTGGATCTGG
M00075	V$GATA1_01	0.82231	5510	(+)	SNNGATNNNN	CTGGATTCTC
M00075	V$GATA1_01	0.877098	5522	(+)	SNNGATNNNN	CCAGATTTTT
M00076	V$GATA2_01	0.847091	5522	(+)	NNNGATRNNN	CCAGATTTTT
M00075	V$GATA1_01	0.817374	5546	(-)	SNNGATNNNN	AGAGATCACA
M00076	V$GATA2_01	0.845737	5546	(-)	NNNGATRNNN	AGAGATCACA
M00075	V$GATA1_01	0.838105	5555	(+)	SNNGATNNNN	ACAGATGCTC
M00076	V$GATA2_01	0.849346	5555	(+)	NNNGATRNNN	ACAGATGCTC
M00126	V$GATA1_02	0.79125	5571	(+)	NNNNNGATANKGNN	TGTGAGATAAAATA
M00128	V$GATA1_04	0.868873	5572	(+)	NNCWGATARNNNN	GTGAGATAAAATA
M00076	V$GATA2_01	0.817772	5573	(+)	NNNGATRNNN	TGAGATAAAA
M00077	V$GATA3_01	0.895879	5574	(+)	NNGATARNG	GAGATAAAA

M00203	V$GATA_C	0.903075	5575	(+)	NGATAAGNMNN	AGATAAAATAA
M00126	V$GATA1_02	0.772813	5582	(-)	NNNNNGATANKGNN	ATAAATATCTTTGT
M00075	V$GATA1_01	0.810958	5584	(-)	SNNGATNNNN	AAATATCTTT
M00076	V$GATA2_01	0.861976	5584	(-)	NNNGATRNNN	AAATATCTTT
M00077	V$GATA3_01	0.834736	5584	(-)	NNGATARNG	AAATATCTT
M00075	V$GATA1_01	0.774926	5614	(+)	SNNGATNNNN	AATGATTATC
M00203	V$GATA_C	0.889096	5614	(-)	NGATAAGNMNN	AATGATTATCT
M00126	V$GATA1_02	0.856563	5615	(-)	NNNNNGATANKGNN	ATGATTATCTTTGA
M00127	V$GATA1_03	0.798138	5615	(-)	RNSNNGATAANNGN	ATGATTATCTTTGA
M00128	V$GATA1_04	0.813113	5615	(-)	NNCWGATARNNNN	ATGATTATCTTTG
M00075	V$GATA1_01	0.777887	5617	(-)	SNNGATNNNN	GATTATCTTT
M00076	V$GATA2_01	0.843482	5617	(-)	NNNGATRNNN	GATTATCTTT
M00077	V$GATA3_01	0.866637	5617	(-)	NNGATARNG	GATTATCTT
M00203	V$GATA_C	0.885989	5624	(-)	NGATAAGNMNN	TTTGATTATCT
M00126	V$GATA1_02	0.867812	5625	(-)	NNNNNGATANKGNN	TTGATTATCTTTTC
M00127	V$GATA1_03	0.881186	5625	(-)	RNSNNGATAANNGN	TTGATTATCTTTTC
M00128	V$GATA1_04	0.875613	5625	(-)	NNCWGATARNNNN	TTGATTATCTTTT
M00075	V$GATA1_01	0.777887	5627	(-)	SNNGATNNNN	GATTATCTTT
M00076	V$GATA2_01	0.843482	5627	(-)	NNNGATRNNN	GATTATCTTT
M00077	V$GATA3_01	0.866637	5627	(-)	NNGATARNG	GATTATCTT
M00126	V$GATA1_02	0.770625	5630	(-)	NNNNNGATANKGNN	TATCTTTTCTTGAC
M00126	V$GATA1_02	0.821875	5641	(+)	NNNNNGATANKGNN	GACAGGATACTAGG
M00127	V$GATA1_03	0.868692	5641	(+)	RNSNNGATAANNGN	GACAGGATACTAGG
M00075	V$GATA1_01	0.861303	5643	(+)	SNNGATNNNN	CAGGATACTA
M00076	V$GATA2_01	0.899414	5643	(+)	NNNGATRNNN	CAGGATACTA
M00075	V$GATA1_01	0.777394	5671	(+)	SNNGATNNNN	TAAGATTATT
M00076	V$GATA2_01	0.815065	5671	(+)	NNNGATRNNN	TAAGATTATT
M00077	V$GATA3_01	0.832964	5672	(+)	NNGATARNG	AAGATTATT
M00126	V$GATA1_02	0.790625	5690	(+)	NNNNNGATANKGNN	TTAAATATAAGGAA
M00203	V$GATA_C	0.831625	5694	(+)	NGATAAGNMNN	ATATAAGGAAA
M00127	V$GATA1_03	0.791034	5696	(+)	RNSNNGATAANNGN	ATAAGGAAAAGAAA
M00127	V$GATA1_03	0.786134	5777	(+)	RNSNNGATAANNGN	AGACAGATATCTCT
M00128	V$GATA1_04	0.862745	5778	(+)	NNCWGATARNNNN	GACAGATATCTCT

M00075	V$GATA1_01	0.831688	5779	(+)	SNNGATNNNN	ACAGATATCT
M00076	V$GATA2_01	0.889039	5779	(+)	NNNGATRNNN	ACAGATATCT
M00126	V$GATA1_02	0.81125	5779	(-)	NNNNNGATANKGNN	ACAGATATCTCTAA
M00128	V$GATA1_04	0.835784	5779	(-)	NNCWGATARNNNN	ACAGATATCTCTA
M00075	V$GATA1_01	0.839585	5781	(-)	SNNGATNNNN	AGATATCTCT
M00076	V$GATA2_01	0.889039	5781	(-)	NNNGATRNNN	AGATATCTCT
M00077	V$GATA3_01	0.836066	5781	(-)	NNGATARNG	AGATATCTC
M00076	V$GATA2_01	0.835814	5811	(+)	NNNGATRNNN	CAGCATAGCA
M00075	V$GATA1_01	0.831194	5868	(-)	SNNGATNNNN	ATGGATCATG
M00076	V$GATA2_01	0.798827	5868	(-)	NNNGATRNNN	ATGGATCATG
M00127	V$GATA1_03	0.801568	5901	(+)	RNSNNGATAANNGN	GTCATGAAAACTGA
M00075	V$GATA1_01	0.948667	5991	(-)	SNNGATNNNN	CCACATCACC
M00076	V$GATA2_01	0.896707	5991	(-)	NNNGATRNNN	CCACATCACC
M00077	V$GATA3_01	0.86265	5991	(-)	NNGATARNG	CCACATCAC
M00127	V$GATA1_03	0.805977	6066	(+)	RNSNNGATAANNGN	AAGAAAATAAAGGT
M00127	V$GATA1_03	0.799363	6075	(-)	RNSNNGATAANNGN	AAGGTTATTTACAT
M00203	V$GATA_C	0.937248	6088	(-)	NGATAAGNMNN	TTGATTTATCA
M00126	V$GATA1_02	0.799063	6089	(-)	NNNNNGATANKGNN	TGATTTATCACAAC
M00128	V$GATA1_04	0.886336	6089	(-)	NNCWGATARNNNN	TGATTTATCACAA
M00075	V$GATA1_01	0.810958	6091	(-)	SNNGATNNNN	ATTTATCACA
M00076	V$GATA2_01	0.836265	6091	(-)	NNNGATRNNN	ATTTATCACA
M00077	V$GATA3_01	0.840496	6091	(-)	NNGATARNG	ATTTATCAC
M00128	V$GATA1_04	0.827819	6100	(+)	NNCWGATARNNNN	AACATATAAGAAT
M00075	V$GATA1_01	0.837117	6107	(-)	SNNGATNNNN	AAGAATCTAC
M00127	V$GATA1_03	0.844684	6140	(+)	RNSNNGATAANNGN	ATGCAGATTAGTGA
M00075	V$GATA1_01	0.866732	6142	(+)	SNNGATNNNN	GCAGATTAGT
M00076	V$GATA2_01	0.843933	6142	(+)	NNNGATRNNN	GCAGATTAGT
M00077	V$GATA3_01	0.848028	6143	(+)	NNGATARNG	CAGATTAGT
M00127	V$GATA1_03	0.797893	6164	(-)	RNSNNGATAANNGN	TTGGTTAGCCTGGT
M00127	V$GATA1_03	0.816512	6170	(+)	RNSNNGATAANNGN	AGCCTGGTAGGCAG
M00127	V$GATA1_03	0.791034	6189	(-)	RNSNNGATAANNGN	TTTGTTTTCCTTAT
M00203	V$GATA_C	0.963964	6194	(-)	NGATAAGNMNN	TTTCCTTATCA
M00126	V$GATA1_02	0.882188	6195	(-)	NNNNNGATANKGNN	TTCCTTATCACTTG

M00127	V$GATA1_03	0.855218	6195	(-)	RNSNNGATAANNGN	TTCCTTATCACTTG
M00128	V$GATA1_04	0.931373	6195	(-)	NNCWGATARNNNN	TTCCTTATCACTT
M00075	V$GATA1_01	0.865745	6197	(-)	SNNGATNNNN	CCTTATCACT
M00076	V$GATA2_01	0.863329	6197	(-)	NNNGATRNNN	CCTTATCACT
M00077	V$GATA3_01	0.948161	6197	(-)	NNGATARNG	CCTTATCAC
M00076	V$GATA2_01	0.80424	6265	(-)	NNNGATRNNN	TTACATCTGA
M00203	V$GATA_C	0.935384	6274	(-)	NGATAAGNMNN	AATATTTATCA
M00126	V$GATA1_02	0.774062	6275	(-)	NNNNNGATANKGNN	ATATTTATCATAAA
M00128	V$GATA1_04	0.908088	6275	(-)	NNCWGATARNNNN	ATATTTATCATAA
M00075	V$GATA1_01	0.78233	6277	(-)	SNNGATNNNN	ATTTATCATA
M00076	V$GATA2_01	0.828146	6277	(-)	NNNGATRNNN	ATTTATCATA
M00077	V$GATA3_01	0.823217	6277	(-)	NNGATARNG	ATTTATCAT
M00126	V$GATA1_02	0.798437	6302	(-)	NNNNNGATANKGNN	TCTCTTTTCTTTTC
M00127	V$GATA1_03	0.788339	6302	(-)	RNSNNGATAANNGN	TCTCTTTTCTTTTC
M00127	V$GATA1_03	0.795688	6309	(-)	RNSNNGATAANNGN	TCTTTTCTCAGGAT
M00075	V$GATA1_01	0.770484	6317	(+)	SNNGATNNNN	CAGGATCACA
M00075	V$GATA1_01	0.849951	6317	(-)	SNNGATNNNN	CAGGATCACA
M00076	V$GATA2_01	0.827695	6317	(+)	NNNGATRNNN	CAGGATCACA
M00076	V$GATA2_01	0.85115	6317	(-)	NNNGATRNNN	CAGGATCACA
M00075	V$GATA1_01	0.877591	6322	(-)	SNNGATNNNN	TCACATCATG
M00076	V$GATA2_01	0.840325	6322	(-)	NNNGATRNNN	TCACATCATG
M00203	V$GATA_C	0.849643	6342	(+)	NGATAAGNMNN	AGGTAAGACCT
M00076	V$GATA2_01	0.790708	6388	(-)	NNNGATRNNN	TAGTATGTGC
M00076	V$GATA2_01	0.818223	6397	(-)	NNNGATRNNN	CACTATGCTT
M00203	V$GATA_C	0.898726	6406	(-)	NGATAAGNMNN	TTACTTTATCA
M00126	V$GATA1_02	0.829063	6407	(-)	NNNNNGATANKGNN	TACTTTATCAAAGC
M00077	V$GATA3_01	0.865751	6409	(-)	NNGATARNG	CTTTATCAA
M00076	V$GATA2_01	0.804691	6418	(-)	NNNGATRNNN	AGCTGTCTTC
M00075	V$GATA1_01	0.828233	6438	(+)	SNNGATNNNN	CAAGATTGAA
M00076	V$GATA2_01	0.805593	6438	(+)	NNNGATRNNN	CAAGATTGAA
M00077	V$GATA3_01	0.873283	6439	(+)	NNGATARNG	AAGATTGAA
M00126	V$GATA1_02	0.842188	6490	(+)	NNNNNGATANKGNN	TGCCAGATAAAGTT
M00127	V$GATA1_03	0.783439	6490	(+)	RNSNNGATAANNGN	TGCCAGATAAAGTT

M00128	V$GATA1_04	0.913909	6491	(+)	NNCWGATARNNNN	GCCAGATAAAGTT
M00075	V$GATA1_01	0.854393	6492	(+)	SNNGATNNNN	CCAGATAAAG
M00076	V$GATA2_01	0.869644	6492	(+)	NNNGATRNNN	CCAGATAAAG
M00077	V$GATA3_01	0.907842	6493	(+)	NNGATARNG	CAGATAAAG
M00203	V$GATA_C	0.907114	6494	(+)	NGATAAGNMNN	AGATAAAGTTA
M00126	V$GATA1_02	0.833125	6530	(+)	NNNNNGATANKGNN	TAAATGATAATCAG
M00127	V$GATA1_03	0.804508	6530	(+)	RNSNNGATAANNGN	TAAATGATAATCAG
M00128	V$GATA1_04	0.883578	6531	(+)	NNCWGATARNNNN	AAATGATAATCAG
M00075	V$GATA1_01	0.798618	6532	(+)	SNNGATNNNN	AATGATAATC
M00076	V$GATA2_01	0.832206	6532	(+)	NNNGATRNNN	AATGATAATC
M00077	V$GATA3_01	0.843155	6533	(+)	NNGATARNG	ATGATAATC
M00075	V$GATA1_01	0.795656	6535	(-)	SNNGATNNNN	GATAATCAGT
M00126	V$GATA1_02	0.79	6555	(+)	NNNNNGATANKGNN	TCAGAGATTAGGTT
M00075	V$GATA1_01	0.821323	6557	(+)	SNNGATNNNN	AGAGATTAGG
M00076	V$GATA2_01	0.810104	6557	(+)	NNNGATRNNN	AGAGATTAGG
M00077	V$GATA3_01	0.937971	6558	(+)	NNGATARNG	GAGATTAGG
M00203	V$GATA_C	0.891271	6565	(-)	NGATAAGNMNN	GGTTCTTGTCT
M00127	V$GATA1_03	0.817001	6566	(-)	RNSNNGATAANNGN	GTTCTTGTCTCCTC
M00075	V$GATA1_01	0.798124	6593	(+)	SNNGATNNNN	AGAGATGAGA
M00076	V$GATA2_01	0.803338	6593	(+)	NNNGATRNNN	AGAGATGAGA
M00077	V$GATA3_01	0.883031	6594	(+)	NNGATARNG	GAGATGAGA
M00075	V$GATA1_01	0.807996	6624	(+)	SNNGATNNNN	GAGGATTTAT
M00076	V$GATA2_01	0.818674	6624	(+)	NNNGATRNNN	GAGGATTTAT
M00126	V$GATA1_02	0.785	6638	(+)	NNNNNGATANKGNN	GTATAGAAAGGACG
M00127	V$GATA1_03	0.820676	6657	(+)	RNSNNGATAANNGN	AAGGGGAGAGCGGG
M00075	V$GATA1_01	0.785291	6659	(+)	SNNGATNNNN	GGGGAGAGCG
M00076	V$GATA2_01	0.792512	6659	(+)	NNNGATRNNN	GGGGAGAGCG
M00075	V$GATA1_01	0.781343	6671	(-)	SNNGATNNNN	CAGCCTCAGG
M00075	V$GATA1_01	0.822804	6765	(+)	SNNGATNNNN	GGGGTTGGGG
M00076	V$GATA2_01	0.799278	6765	(+)	NNNGATRNNN	GGGGTTGGGG
M00075	V$GATA1_01	0.883021	6783	(+)	SNNGATNNNN	CCTGATTTTC
M00076	V$GATA2_01	0.832206	6783	(+)	NNNGATRNNN	CCTGATTTTC
M00127	V$GATA1_03	0.83317	6784	(-)	RNSNNGATAANNGN	CTGATTTTCATCCC

M00076	V$GATA2_01	0.803789	6789	(-)	NNNGATRNNN	TTTCATCCCA
M00075	V$GATA1_01	0.789733	6803	(-)	SNNGATNNNN	CACCTTCCCG
M00075	V$GATA1_01	0.815893	6821	(+)	SNNGATNNNN	AGAGATTTTT
M00076	V$GATA2_01	0.805593	6821	(+)	NNNGATRNNN	AGAGATTTTT
M00203	V$GATA_C	0.83877	6830	(-)	NGATAAGNMNN	TGTTCTTATTT
M00077	V$GATA3_01	0.844927	6838	(-)	NNGATARNG	TTTAATCTT
M00075	V$GATA1_01	0.828233	6874	(+)	SNNGATNNNN	TCTGATCCGC
M00075	V$GATA1_01	0.799112	6874	(-)	SNNGATNNNN	TCTGATCCGC
M00076	V$GATA2_01	0.840776	6874	(+)	NNNGATRNNN	TCTGATCCGC
M00076	V$GATA2_01	0.828597	6874	(-)	NNNGATRNNN	TCTGATCCGC
M00075	V$GATA1_01	0.783317	6881	(+)	SNNGATNNNN	CGCGAGGCTG
M00075	V$GATA1_01	0.857848	6887	(+)	SNNGATNNNN	GCTGATTTTA
M00076	V$GATA2_01	0.811457	6887	(+)	NNNGATRNNN	GCTGATTTTA
M00076	V$GATA2_01	0.865133	6906	(+)	NNNGATRNNN	GAGCATAGTG
M00075	V$GATA1_01	0.815893	6931	(+)	SNNGATNNNN	TTAGATGCTG
M00076	V$GATA2_01	0.808751	6931	(+)	NNNGATRNNN	TTAGATGCTG
M00075	V$GATA1_01	0.901777	6940	(+)	SNNGATNNNN	GGAGATTCCT
M00076	V$GATA2_01	0.882724	6940	(+)	NNNGATRNNN	GGAGATTCCT
M00126	V$GATA1_02	0.778125	6957	(-)	NNNNNGATANKGNN	CCACCTTTCTTTTC
M00127	V$GATA1_03	0.817001	6962	(-)	RNSNNGATAANNGN	TTTCTTTTCCTGCT
M00076	V$GATA2_01	0.788453	6978	(+)	NNNGATRNNN	CAGGACACCT
M00127	V$GATA1_03	0.804508	6982	(-)	RNSNNGATAANNGN	ACACCTGTCACCCC
M00075	V$GATA1_01	0.811451	6984	(-)	SNNGATNNNN	ACCTGTCACC
M00076	V$GATA2_01	0.788904	6984	(-)	NNNGATRNNN	ACCTGTCACC
M00203	V$GATA_C	0.960236	7006	(-)	NGATAAGNMNN	AATTCTTATCA
M00126	V$GATA1_02	0.843438	7007	(-)	NNNNNGATANKGNN	ATTCTTATCAGTCC
M00127	V$GATA1_03	0.897354	7007	(-)	RNSNNGATAANNGN	ATTCTTATCAGTCC
M00128	V$GATA1_04	0.9519	7007	(-)	NNCWGATARNNNN	ATTCTTATCAGTC
M00075	V$GATA1_01	0.810958	7009	(-)	SNNGATNNNN	TCTTATCAGT
M00076	V$GATA2_01	0.820478	7009	(-)	NNNGATRNNN	TCTTATCAGT
M00077	V$GATA3_01	0.89012	7009	(-)	NNGATARNG	TCTTATCAG
M00127	V$GATA1_03	0.807692	7029	(-)	RNSNNGATAANNGN	CTGCTTTTCTCTGT
M00127	V$GATA1_03	0.815287	7031	(-)	RNSNNGATAANNGN	GCTTTTCTCTGTCT

M00075	V$GATA1_01	0.840573	7067	(+)	SNNGATNNNN	CAAGATGTAT
M00076	V$GATA2_01	0.834912	7067	(+)	NNNGATRNNN	CAAGATGTAT
M00127	V$GATA1_03	0.796178	7086	(-)	RNSNNGATAANNGN	CCATTTAGCTGGTT
M00126	V$GATA1_02	0.8225	7100	(-)	NNNNNGATANKGNN	CCCCCTTTCTCTGC
M00127	V$GATA1_03	0.78099	7100	(-)	RNSNNGATAANNGN	CCCCCTTTCTCTGC
M00077	V$GATA3_01	0.840496	7102	(-)	NNGATARNG	CCCTTTCTC
M00127	V$GATA1_03	0.805488	7102	(-)	RNSNNGATAANNGN	CCCTTTCTCTGCTC
M00128	V$GATA1_04	0.828431	7102	(-)	NNCWGATARNNNN	CCCTTTCTCTGCT
M00075	V$GATA1_01	0.850938	7251	(-)	SNNGATNNNN	TGCCATCCAG
M00076	V$GATA2_01	0.833559	7251	(-)	NNNGATRNNN	TGCCATCCAG
M00076	V$GATA2_01	0.824538	7260	(-)	NNNGATRNNN	GGGTATGAGC
M00076	V$GATA2_01	0.815516	7334	(+)	NNNGATRNNN	TAGGATCCAT
M00077	V$GATA3_01	0.896323	7355	(-)	NNGATARNG	CCTAATCTA
M00075	V$GATA1_01	0.88154	7365	(+)	SNNGATNNNN	AAAGATGGGG
M00076	V$GATA2_01	0.88949	7365	(+)	NNNGATRNNN	AAAGATGGGG
M00077	V$GATA3_01	0.929109	7366	(+)	NNGATARNG	AAGATGGGG
M00075	V$GATA1_01	0.833169	7401	(-)	SNNGATNNNN	GTTAATCACG
M00075	V$GATA1_01	0.781836	7418	(+)	SNNGATNNNN	AGAGATGAAT
M00076	V$GATA2_01	0.786649	7418	(+)	NNNGATRNNN	AGAGATGAAT
M00075	V$GATA1_01	0.794669	7446	(-)	SNNGATNNNN	ATCAATCTTT
M00076	V$GATA2_01	0.793866	7446	(-)	NNNGATRNNN	ATCAATCTTT
M00077	V$GATA3_01	0.841382	7446	(-)	NNGATARNG	ATCAATCTT
M00076	V$GATA2_01	0.809202	7457	(-)	NNNGATRNNN	AAATATGTCC
M00075	V$GATA1_01	0.827246	7500	(-)	SNNGATNNNN	AAACATCCGT
M00076	V$GATA2_01	0.856112	7500	(-)	NNNGATRNNN	AAACATCCGT
M00076	V$GATA2_01	0.824087	7540	(+)	NNNGATRNNN	AAGCATAGCT
M00203	V$GATA_C	0.896241	7716	(-)	NGATAAGNMNN	CATTTTTATCA
M00126	V$GATA1_02	0.794375	7717	(-)	NNNNNGATANKGNN	ATTTTTATCACAAA
M00128	V$GATA1_04	0.907475	7717	(-)	NNCWGATARNNNN	ATTTTTATCACAA
M00075	V$GATA1_01	0.77542	7719	(-)	SNNGATNNNN	TTTTATCACA
M00076	V$GATA2_01	0.806495	7719	(-)	NNNGATRNNN	TTTTATCACA
M00077	V$GATA3_01	0.872397	7719	(-)	NNGATARNG	TTTTATCAC
M00075	V$GATA1_01	0.869694	7755	(-)	SNNGATNNNN	GTCAATCATG

M00076	V$GATA2_01	0.820478	7755	(-)	NNNGATRNNN	GTCAATCATG
M00203	V$GATA_C	0.861758	7759	(-)	NGATAAGNMNN	ATCATGTATCT
M00126	V$GATA1_02	0.820938	7760	(-)	NNNNNGATANKGNN	TCATGTATCTGTGT
M00127	V$GATA1_03	0.828515	7760	(-)	RNSNNGATAANNGN	TCATGTATCTGTGT
M00075	V$GATA1_01	0.813425	7762	(-)	SNNGATNNNN	ATGTATCTGT
M00076	V$GATA2_01	0.841227	7762	(-)	NNNGATRNNN	ATGTATCTGT
M00203	V$GATA_C	0.860826	7796	(-)	NGATAAGNMNN	TGAAGTTATCA
M00127	V$GATA1_03	0.786379	7797	(-)	RNSNNGATAANNGN	GAAGTTATCAGGGA
M00128	V$GATA1_04	0.816483	7797	(-)	NNCWGATARNNNN	GAAGTTATCAGGG
M00075	V$GATA1_01	0.903258	7799	(-)	SNNGATNNNN	AGTTATCAGG
M00076	V$GATA2_01	0.904375	7799	(-)	NNNGATRNNN	AGTTATCAGG
M00077	V$GATA3_01	0.821444	7799	(-)	NNGATARNG	AGTTATCAG
M00076	V$GATA2_01	0.79567	7831	(+)	NNNGATRNNN	CAACATATTT
M00127	V$GATA1_03	0.785154	7912	(+)	RNSNNGATAANNGN	AAGGAGTTAGCACC
M00076	V$GATA2_01	0.78304	7914	(+)	NNNGATRNNN	GGAGTTAGCA
M00127	V$GATA1_03	0.798873	7914	(-)	RNSNNGATAANNGN	GGAGTTAGCACCCC
M00075	V$GATA1_01	0.773939	7941	(+)	SNNGATNNNN	GGGGCTAGTT
M00075	V$GATA1_01	0.809477	7963	(-)	SNNGATNNNN	TAAAATCACA
M00076	V$GATA2_01	0.797023	7963	(-)	NNNGATRNNN	TAAAATCACA
M00077	V$GATA3_01	0.826761	7963	(-)	NNGATARNG	TAAAATCAC
M00075	V$GATA1_01	0.818361	7971	(+)	SNNGATNNNN	CAGGATCAGT
M00075	V$GATA1_01	0.833662	7971	(-)	SNNGATNNNN	CAGGATCAGT
M00076	V$GATA2_01	0.844835	7971	(+)	NNNGATRNNN	CAGGATCAGT
M00076	V$GATA2_01	0.818674	7971	(-)	NNNGATRNNN	CAGGATCAGT
M00075	V$GATA1_01	0.813919	7984	(+)	SNNGATNNNN	ACTGATTTTC
M00076	V$GATA2_01	0.790708	7984	(+)	NNNGATRNNN	ACTGATTTTC
M00127	V$GATA1_03	0.800833	7985	(-)	RNSNNGATAANNGN	CTGATTTTCTTTTT
M00075	V$GATA1_01	0.898322	8011	(-)	SNNGATNNNN	AGGAATCTGG
M00076	V$GATA2_01	0.877312	8011	(-)	NNNGATRNNN	AGGAATCTGG
M00203	V$GATA_C	0.894066	8056	(-)	NGATAAGNMNN	ATTCATTATCC
M00126	V$GATA1_02	0.790938	8057	(-)	NNNNNGATANKGNN	TTCATTATCCCAAT
M00127	V$GATA1_03	0.830475	8057	(-)	RNSNNGATAANNGN	TTCATTATCCCAAT
M00128	V$GATA1_04	0.822304	8057	(-)	NNCWGATARNNNN	TTCATTATCCCAA

M00075	V$GATA1_01	0.836624	8059	(-)	SNNGATNNNN	CATTATCCCA
M00076	V$GATA2_01	0.915201	8059	(-)	NNNGATRNNN	CATTATCCCA
M00077	V$GATA3_01	0.905627	8059	(-)	NNGATARNG	CATTATCCC
M00128	V$GATA1_04	0.816176	8063	(-)	NNCWGATARNNNN	ATCCCAATCAGAA
M00075	V$GATA1_01	0.9077	8065	(-)	SNNGATNNNN	CCCAATCAGA
M00076	V$GATA2_01	0.882724	8065	(-)	NNNGATRNNN	CCCAATCAGA
M00077	V$GATA3_01	0.919805	8065	(-)	NNGATARNG	CCCAATCAG
M00203	V$GATA_C	0.883504	8107	(-)	NGATAAGNMNN	TTTACATATCC
M00075	V$GATA1_01	0.827246	8110	(-)	SNNGATNNNN	ACATATCCTA
M00076	V$GATA2_01	0.915201	8110	(-)	NNNGATRNNN	ACATATCCTA
M00075	V$GATA1_01	0.84847	8125	(+)	SNNGATNNNN	TCTGATTGCA
M00076	V$GATA2_01	0.852503	8125	(+)	NNNGATRNNN	TCTGATTGCA
M00077	V$GATA3_01	0.848028	8126	(+)	NNGATARNG	CTGATTGCA
M00076	V$GATA2_01	0.813261	8154	(-)	NNNGATRNNN	CACTGTCCTC
M00127	V$GATA1_03	0.784664	8174	(+)	RNSNNGATAANNGN	ACATGGATATCAAC
M00076	V$GATA2_01	0.783942	8176	(+)	NNNGATRNNN	ATGGATATCA
M00127	V$GATA1_03	0.817491	8176	(-)	RNSNNGATAANNGN	ATGGATATCAACCC
M00075	V$GATA1_01	0.845508	8178	(-)	SNNGATNNNN	GGATATCAAC
M00076	V$GATA2_01	0.831304	8178	(-)	NNNGATRNNN	GGATATCAAC
M00075	V$GATA1_01	0.772458	8186	(-)	SNNGATNNNN	ACCCAACAGA
M00075	V$GATA1_01	0.792201	8191	(+)	SNNGATNNNN	ACAGATGTAT
M00076	V$GATA2_01	0.801534	8191	(+)	NNNGATRNNN	ACAGATGTAT
M00203	V$GATA_C	0.849332	8208	(-)	NGATAAGNMNN	AATATTTGTCA
M00203	V$GATA_C	0.864865	8244	(+)	NGATAAGNMNN	GGACAAGCAAT
M00075	V$GATA1_01	0.829714	8249	(-)	SNNGATNNNN	AGCAATCCTT
M00076	V$GATA2_01	0.867388	8249	(-)	NNNGATRNNN	AGCAATCCTT
M00126	V$GATA1_02	0.854688	8260	(-)	NNNNNGATANKGNN	CTGACTATCTGTGC
M00127	V$GATA1_03	0.873836	8260	(-)	RNSNNGATAANNGN	CTGACTATCTGTGC
M00128	V$GATA1_04	0.826593	8260	(-)	NNCWGATARNNNN	CTGACTATCTGTG
M00075	V$GATA1_01	0.863771	8262	(-)	SNNGATNNNN	GACTATCTGT
M00076	V$GATA2_01	0.903022	8262	(-)	NNNGATRNNN	GACTATCTGT
M00077	V$GATA3_01	0.889233	8262	(-)	NNGATARNG	GACTATCTG
M00075	V$GATA1_01	0.88845	8312	(+)	SNNGATNNNN	CCAGATTTTG

M00076	V$GATA2_01	0.860171	8312	(+)	NNNGATRNNN	CCAGATTTTG
M00077	V$GATA3_01	0.862206	8313	(+)	NNGATARNG	CAGATTTTG
M00126	V$GATA1_02	0.80625	8316	(+)	NNNNNGATANKGNN	ATTTTGATAGAACT
M00128	V$GATA1_04	0.856924	8317	(+)	NNCWGATARNNNN	TTTTGATAGAACT
M00075	V$GATA1_01	0.775913	8318	(+)	SNNGATNNNN	TTTGATAGAA
M00077	V$GATA3_01	0.859105	8319	(+)	NNGATARNG	TTGATAGAA
M00203	V$GATA_C	0.890339	8320	(+)	NGATAAGNMNN	TGATAGAACTA
M00075	V$GATA1_01	0.828233	8328	(-)	SNNGATNNNN	CTAAATCTTG
M00076	V$GATA2_01	0.811908	8328	(-)	NNNGATRNNN	CTAAATCTTG
M00077	V$GATA3_01	0.848028	8328	(-)	NNGATARNG	CTAAATCTT
M00203	V$GATA_C	0.835353	8330	(-)	NGATAAGNMNN	AAATCTTGTCA
M00127	V$GATA1_03	0.793974	8356	(-)	RNSNNGATAANNGN	ATGGCTATGTTTCT
M00076	V$GATA2_01	0.806044	8358	(-)	NNNGATRNNN	GGCTATGTTT
M00075	V$GATA1_01	0.79615	8375	(+)	SNNGATNNNN	AATGATATCA
M00076	V$GATA2_01	0.839874	8375	(+)	NNNGATRNNN	AATGATATCA
M00127	V$GATA1_03	0.792504	8375	(-)	RNSNNGATAANNGN	AATGATATCAACAT
M00077	V$GATA3_01	0.827204	8376	(+)	NNGATARNG	ATGATATCA
M00075	V$GATA1_01	0.824778	8377	(-)	SNNGATNNNN	TGATATCAAC
M00076	V$GATA2_01	0.805142	8377	(-)	NNNGATRNNN	TGATATCAAC
M00203	V$GATA_C	0.938801	8387	(-)	NGATAAGNMNN	ATGTTTTATCC
M00127	V$GATA1_03	0.814552	8388	(-)	RNSNNGATAANNGN	TGTTTTATCCTGTG
M00128	V$GATA1_04	0.83701	8388	(-)	NNCWGATARNNNN	TGTTTTATCCTGT
M00075	V$GATA1_01	0.78233	8390	(-)	SNNGATNNNN	TTTTATCCTG
M00076	V$GATA2_01	0.833108	8390	(-)	NNNGATRNNN	TTTTATCCTG
M00077	V$GATA3_01	0.833407	8390	(-)	NNGATARNG	TTTTATCCT
M00075	V$GATA1_01	0.836624	8403	(-)	SNNGATNNNN	AGAAATCAAC
M00075	V$GATA1_01	0.816387	8422	(-)	SNNGATNNNN	GCTAATCAGA
M00076	V$GATA2_01	0.814614	8422	(-)	NNNGATRNNN	GCTAATCAGA
M00077	V$GATA3_01	0.824546	8422	(-)	NNGATARNG	GCTAATCAG
M00076	V$GATA2_01	0.812359	8435	(-)	NNNGATRNNN	TGGAATCCTT
M00127	V$GATA1_03	0.801813	8462	(+)	RNSNNGATAANNGN	AAGGAGAAAAAGAG
M00076	V$GATA2_01	0.789806	8477	(+)	NNNGATRNNN	AGGCATAGGA
M00127	V$GATA1_03	0.795198	8552	(-)	RNSNNGATAANNGN	TCAGTTATGTGGAG

M00076	V$GATA2_01	0.807848	8554	(-)	NNNGATRNNN	AGTTATGTGG
M00075	V$GATA1_01	0.811945	8595	(-)	SNNGATNNNN	TCACATCCAC
M00076	V$GATA2_01	0.789806	8595	(-)	NNNGATRNNN	TCACATCCAC
M00126	V$GATA1_02	0.804688	8637	(+)	NNNNNGATANKGNN	GCAGTGAGAAGGGA
M00077	V$GATA3_01	0.824103	8640	(+)	NNGATARNG	GTGAGAAGG
M00075	V$GATA1_01	0.82231	8686	(-)	SNNGATNNNN	ATTCATCTTG
M00076	V$GATA2_01	0.820027	8686	(-)	NNNGATRNNN	ATTCATCTTG
M00127	V$GATA1_03	0.794463	8746	(+)	RNSNNGATAANNGN	AGCAATATAGTCTC
M00128	V$GATA1_04	0.827819	8772	(-)	NNCWGATARNNNN	ATTCTTACCTGAA
M00075	V$GATA1_01	0.800099	8806	(-)	SNNGATNNNN	AACTAACATG
M00076	V$GATA2_01	0.783942	8809	(+)	NNNGATRNNN	TAACATGGCA
M00076	V$GATA2_01	0.780785	8814	(+)	NNNGATRNNN	TGGCATGTTT
M00128	V$GATA1_04	0.819853	8861	(+)	NNCWGATARNNNN	GTGAGATATATTT
M00075	V$GATA1_01	0.80849	8862	(+)	SNNGATNNNN	TGAGATATAT
M00076	V$GATA2_01	0.862427	8862	(+)	NNNGATRNNN	TGAGATATAT
M00077	V$GATA3_01	0.832078	8863	(+)	NNGATARNG	GAGATATAT
M00203	V$GATA_C	0.890339	8892	(-)	NGATAAGNMNN	TAGCATTATCC
M00126	V$GATA1_02	0.837812	8893	(-)	NNNNNGATANKGNN	AGCATTATCCAGGA
M00075	V$GATA1_01	0.832675	8895	(-)	SNNGATNNNN	CATTATCCAG
M00076	V$GATA2_01	0.834461	8895	(-)	NNNGATRNNN	CATTATCCAG
M00077	V$GATA3_01	0.863979	8895	(-)	NNGATARNG	CATTATCCA
M00075	V$GATA1_01	0.887957	8902	(+)	SNNGATNNNN	CAGGATATGT
M00076	V$GATA2_01	0.924222	8902	(+)	NNNGATRNNN	CAGGATATGT
M00076	V$GATA2_01	0.801985	8904	(-)	NNNGATRNNN	GGATATGTTA
M00203	V$GATA_C	0.844672	8904	(+)	NGATAAGNMNN	GGATATGTTAA
M00127	V$GATA1_03	0.851298	8968	(+)	RNSNNGATAANNGN	AGGAAGATATGCCC
M00128	V$GATA1_04	0.871936	8969	(+)	NNCWGATARNNNN	GGAAGATATGCCC
M00075	V$GATA1_01	0.873149	8970	(+)	SNNGATNNNN	GAAGATATGC
M00076	V$GATA2_01	0.911592	8970	(+)	NNNGATRNNN	GAAGATATGC
M00077	V$GATA3_01	0.85556	8971	(+)	NNGATARNG	AAGATATGC
M00076	V$GATA2_01	0.84258	8972	(-)	NNNGATRNNN	AGATATGCCC
M00203	V$GATA_C	0.911463	8972	(+)	NGATAAGNMNN	AGATATGCCCT
M00126	V$GATA1_02	0.8625	8986	(-)	NNNNNGATANKGNN	AACGCTATCTGGTA

M00128	V$GATA1_04	0.898284	8986	(-)	NNCWGATARNNNN	AACGCTATCTGGT
M00075	V$GATA1_01	0.959033	8988	(-)	SNNGATNNNN	CGCTATCTGG
M00076	V$GATA2_01	0.980153	8988	(-)	NNNGATRNNN	CGCTATCTGG
M00077	V$GATA3_01	0.940186	8988	(-)	NNGATARNG	CGCTATCTG
M00203	V$GATA_C	0.885679	9011	(-)	NGATAAGNMNN	AAAGTTTATCA
M00127	V$GATA1_03	0.784174	9025	(+)	RNSNNGATAANNGN	GCAAAGGTAACAAG
M00127	V$GATA1_03	0.800343	9038	(+)	RNSNNGATAANNGN	GGAATGAGAAGAAC
M00126	V$GATA1_02	0.812813	9055	(+)	NNNNNGATANKGNN	GCACTGATAAATTA
M00127	V$GATA1_03	0.805243	9055	(+)	RNSNNGATAANNGN	GCACTGATAAATTA
M00075	V$GATA1_01	0.794669	9057	(+)	SNNGATNNNN	ACTGATAAAT
M00076	V$GATA2_01	0.803789	9057	(+)	NNNGATRNNN	ACTGATAAAT
M00203	V$GATA_C	0.888475	9059	(+)	NGATAAGNMNN	TGATAAATTAC
M00075	V$GATA1_01	0.844028	9122	(+)	SNNGATNNNN	CTAGATGTTT
M00076	V$GATA2_01	0.793415	9122	(+)	NNNGATRNNN	CTAGATGTTT
M00127	V$GATA1_03	0.781725	9130	(-)	RNSNNGATAANNGN	TTACTGATCCCCTC
M00075	V$GATA1_01	0.807502	9132	(+)	SNNGATNNNN	ACTGATCCCC
M00075	V$GATA1_01	0.842547	9132	(-)	SNNGATNNNN	ACTGATCCCC
M00076	V$GATA2_01	0.820929	9132	(+)	NNNGATRNNN	ACTGATCCCC
M00076	V$GATA2_01	0.858367	9132	(-)	NNNGATRNNN	ACTGATCCCC
M00077	V$GATA3_01	0.830306	9168	(-)	NNGATARNG	TTAAATCTC
M00076	V$GATA2_01	0.782589	9227	(-)	NNNGATRNNN	TTAGATCCGA
M00127	V$GATA1_03	0.811857	9301	(-)	RNSNNGATAANNGN	CCTTTTAGCAGCAC
M00075	V$GATA1_01	0.784798	9317	(+)	SNNGATNNNN	GGTGTTAGAT
M00126	V$GATA1_02	0.815625	9319	(+)	NNNNNGATANKGNN	TGTTAGATAGTTTA
M00075	V$GATA1_01	0.830207	9321	(+)	SNNGATNNNN	TTAGATAGTT
M00076	V$GATA2_01	0.849346	9321	(+)	NNNGATRNNN	TTAGATAGTT
M00077	V$GATA3_01	0.870625	9322	(+)	NNGATARNG	TAGATAGTT
M00126	V$GATA1_02	0.844063	9449	(-)	NNNNNGATANKGNN	GCTAGTATCAGAAA
M00128	V$GATA1_04	0.829963	9449	(-)	NNCWGATARNNNN	GCTAGTATCAGAA
M00075	V$GATA1_01	0.846496	9451	(-)	SNNGATNNNN	TAGTATCAGA
M00076	V$GATA2_01	0.872801	9451	(-)	NNNGATRNNN	TAGTATCAGA
M00075	V$GATA1_01	0.831194	9463	(+)	SNNGATNNNN	CATGATCACT
M00075	V$GATA1_01	0.802073	9463	(-)	SNNGATNNNN	CATGATCACT

M00076	V$GATA2_01	0.831755	9463	(+)	NNNGATRNNN	CATGATCACT
M00076	V$GATA2_01	0.792512	9463	(-)	NNNGATRNNN	CATGATCACT
M00077	V$GATA3_01	0.835623	9482	(-)	NNGATARNG	CTTCATCTA
M00075	V$GATA1_01	0.801579	9495	(+)	SNNGATNNNN	TCTGATTTCA
M00076	V$GATA2_01	0.815968	9495	(+)	NNNGATRNNN	TCTGATTTCA
M00075	V$GATA1_01	0.772458	9516	(-)	SNNGATNNNN	TTAAATCCTC
M00076	V$GATA2_01	0.788904	9516	(-)	NNNGATRNNN	TTAAATCCTC
M00075	V$GATA1_01	0.857354	9540	(-)	SNNGATNNNN	AGTCATCTTC
M00076	V$GATA2_01	0.884529	9540	(-)	NNNGATRNNN	AGTCATCTTC
M00076	V$GATA2_01	0.783942	9578	(+)	NNNGATRNNN	CACCATAAGT
M00126	V$GATA1_02	0.79625	9596	(-)	NNNNNGATANKGNN	CTGAATATCATTAA
M00128	V$GATA1_04	0.816176	9596	(-)	NNCWGATARNNNN	CTGAATATCATTA
M00075	V$GATA1_01	0.816881	9598	(-)	SNNGATNNNN	GAATATCATT
M00076	V$GATA2_01	0.847091	9598	(-)	NNNGATRNNN	GAATATCATT
M00128	V$GATA1_04	0.870711	9605	(-)	NNCWGATARNNNN	ATTAATATCTGAC
M00075	V$GATA1_01	0.804541	9607	(-)	SNNGATNNNN	TAATATCTGA
M00076	V$GATA2_01	0.872801	9607	(-)	NNNGATRNNN	TAATATCTGA
M00077	V$GATA3_01	0.860877	9607	(-)	NNGATARNG	TAATATCTG
M00077	V$GATA3_01	0.82942	9686	(+)	NNGATARNG	TAGATTAGT
M00203	V$GATA_C	0.845294	9687	(+)	NGATAAGNMNN	AGATTAGTCCA
M00076	V$GATA2_01	0.78304	9727	(+)	NNNGATRNNN	TGAGATTAAT
M00077	V$GATA3_01	0.830306	9728	(+)	NNGATARNG	GAGATTAAT
M00127	V$GATA1_03	0.793974	9742	(-)	RNSNNGATAANNGN	CTCATTCTCTACTC
M00203	V$GATA_C	0.833178	9759	(+)	NGATAAGNMNN	AGAAAAGGCAC
M00075	V$GATA1_01	0.770977	9767	(+)	SNNGATNNNN	CACGTTAGGA
M00075	V$GATA1_01	0.7769	9772	(+)	SNNGATNNNN	TAGGATTCTA
M00076	V$GATA2_01	0.825891	9772	(+)	NNNGATRNNN	TAGGATTCTA
M00075	V$GATA1_01	0.843534	9802	(+)	SNNGATNNNN	TGGGATTGGA
M00076	V$GATA2_01	0.865584	9802	(+)	NNNGATRNNN	TGGGATTGGA
M00077	V$GATA3_01	0.886132	9803	(+)	NNGATARNG	GGGATTGGA
M00126	V$GATA1_02	0.773125	9815	(-)	NNNNNGATANKGNN	CATGGTATCTAAGC
M00076	V$GATA2_01	0.813261	9817	(-)	NNNGATRNNN	TGGTATCTAA
M00075	V$GATA1_01	0.77542	9964	(+)	SNNGATNNNN	GGGGTTTGCT

M00075	V$GATA1_01	0.806515	10011	(+)	SNNGATNNNN	CTTGATCATG
M00075	V$GATA1_01	0.80306	10011	(-)	SNNGATNNNN	CTTGATCATG
M00076	V$GATA2_01	0.785747	10011	(-)	NNNGATRNNN	CTTGATCATG
M00076	V$GATA2_01	0.788002	10041	(-)	NNNGATRNNN	CCATATGCTT
M00203	V$GATA_C	0.87698	10103	(-)	NGATAAGNMNN	GTGCTGTATCA
M00126	V$GATA1_02	0.823437	10104	(-)	NNNNNGATANKGNN	TGCTGTATCACATC
M00128	V$GATA1_04	0.826287	10104	(-)	NNCWGATARNNNN	TGCTGTATCACAT
M00075	V$GATA1_01	0.861797	10106	(-)	SNNGATNNNN	CTGTATCACA
M00076	V$GATA2_01	0.875507	10106	(-)	NNNGATRNNN	CTGTATCACA
M00077	V$GATA3_01	0.856447	10106	(-)	NNGATARNG	CTGTATCAC
M00203	V$GATA_C	0.841876	10121	(-)	NGATAAGNMNN	AATTACTATCC
M00126	V$GATA1_02	0.799375	10122	(-)	NNNNNGATANKGNN	ATTACTATCCAGAG
M00127	V$GATA1_03	0.841009	10122	(-)	RNSNNGATAANNGN	ATTACTATCCAGAG
M00075	V$GATA1_01	0.850938	10124	(-)	SNNGATNNNN	TACTATCCAG
M00076	V$GATA2_01	0.843031	10124	(-)	NNNGATRNNN	TACTATCCAG
M00077	V$GATA3_01	0.857333	10124	(-)	NNGATARNG	TACTATCCA
M00075	V$GATA1_01	0.775913	10158	(-)	SNNGATNNNN	CTACATCAAA
M00075	V$GATA1_01	0.824284	10163	(-)	SNNGATNNNN	TCAAATCCTC
M00076	V$GATA2_01	0.835363	10163	(-)	NNNGATRNNN	TCAAATCCTC
M00075	V$GATA1_01	0.846989	10183	(+)	SNNGATNNNN	CCAGATGATA
M00076	V$GATA2_01	0.838521	10183	(+)	NNNGATRNNN	CCAGATGATA
M00077	V$GATA3_01	0.839167	10184	(+)	NNGATARNG	CAGATGATA
M00075	V$GATA1_01	0.856861	10186	(+)	SNNGATNNNN	GATGATATAT
M00076	V$GATA2_01	0.857465	10186	(+)	NNNGATRNNN	GATGATATAT
M00203	V$GATA_C	0.899658	10204	(-)	NGATAAGNMNN	GGAATTTATCC
M00127	V$GATA1_03	0.83758	10205	(-)	RNSNNGATAANNGN	GAATTTATCCTGTC
M00128	V$GATA1_04	0.812194	10205	(-)	NNCWGATARNNNN	GAATTTATCCTGT
M00075	V$GATA1_01	0.817868	10207	(-)	SNNGATNNNN	ATTTATCCTG
M00076	V$GATA2_01	0.862878	10207	(-)	NNNGATRNNN	ATTTATCCTG
M00126	V$GATA1_02	0.771875	10215	(-)	NNNNNGATANKGNN	TGTCCTGTCTTTAT
M00127	V$GATA1_03	0.811122	10221	(-)	RNSNNGATAANNGN	GTCTTTATTTTCCC
M00075	V$GATA1_01	0.897828	10245	(+)	SNNGATNNNN	CTGGATGGCG
M00076	V$GATA2_01	0.87641	10245	(+)	NNNGATRNNN	CTGGATGGCG

M00077	V$GATA3_01	0.822774	10246	(+)	NNGATARNG	TGGATGGCG
M00126	V$GATA1_02	0.842187	10282	(+)	NNNNNGATANKGNN	GCACAGATATGCCA
M00128	V$GATA1_04	0.812194	10283	(+)	NNCWGATARNNNN	CACAGATATGCCA
M00075	V$GATA1_01	0.82922	10284	(+)	SNNGATNNNN	ACAGATATGC
M00076	V$GATA2_01	0.872801	10284	(+)	NNNGATRNNN	ACAGATATGC
M00077	V$GATA3_01	0.849801	10285	(+)	NNGATARNG	CAGATATGC
M00076	V$GATA2_01	0.828146	10286	(-)	NNNGATRNNN	AGATATGCCA
M00203	V$GATA_C	0.891581	10286	(+)	NGATAAGNMNN	AGATATGCCAA
M00075	V$GATA1_01	0.919052	10335	(-)	SNNGATNNNN	AAGAATCAGG
M00076	V$GATA2_01	0.847091	10335	(-)	NNNGATRNNN	AAGAATCAGG
M00203	V$GATA_C	0.831625	10363	(-)	NGATAAGNMNN	TGCTTTTGTCA
M00075	V$GATA1_01	0.858342	10387	(-)	SNNGATNNNN	AAGAATCAGA
M00076	V$GATA2_01	0.838069	10387	(-)	NNNGATRNNN	AAGAATCAGA
M00075	V$GATA1_01	0.778875	10399	(+)	SNNGATNNNN	GTTGATTAAT
M00203	V$GATA_C	0.912706	10405	(-)	NGATAAGNMNN	TAATCTTATCC
M00126	V$GATA1_02	0.825625	10406	(-)	NNNNNGATANKGNN	AATCTTATCCCGGA
M00075	V$GATA1_01	0.862784	10408	(-)	SNNGATNNNN	TCTTATCCCG
M00076	V$GATA2_01	0.887686	10408	(-)	NNNGATRNNN	TCTTATCCCG
M00077	V$GATA3_01	0.891449	10408	(-)	NNGATARNG	TCTTATCCC
M00203	V$GATA_C	0.845604	10425	(-)	NGATAAGNMNN	TGTCCTTATGT
M00127	V$GATA1_03	0.797893	10449	(-)	RNSNNGATAANNGN	ATAGTTATTCTCAC
M00075	V$GATA1_01	0.852419	10459	(-)	SNNGATNNNN	TCACATCCTG
M00076	V$GATA2_01	0.866035	10459	(-)	NNNGATRNNN	TCACATCCTG
M00075	V$GATA1_01	0.777394	10468	(+)	SNNGATNNNN	GGTGTTATTT
M00075	V$GATA1_01	0.898815	10491	(-)	SNNGATNNNN	AGCAATCTTC
M00076	V$GATA2_01	0.899865	10491	(-)	NNNGATRNNN	AGCAATCTTC
M00077	V$GATA3_01	0.84537	10491	(-)	NNGATARNG	AGCAATCTT
M00126	V$GATA1_02	0.789062	10504	(-)	NNNNNGATANKGNN	TCAGTTATCCCTTC
M00127	V$GATA1_03	0.882411	10504	(-)	RNSNNGATAANNGN	TCAGTTATCCCTTC
M00128	V$GATA1_04	0.820159	10504	(-)	NNCWGATARNNNN	TCAGTTATCCCTT
M00075	V$GATA1_01	0.816881	10506	(-)	SNNGATNNNN	AGTTATCCCT
M00076	V$GATA2_01	0.888588	10506	(-)	NNNGATRNNN	AGTTATCCCT
M00077	V$GATA3_01	0.822774	10506	(-)	NNGATARNG	AGTTATCCC

M00128	V$GATA1_04	0.82598	10512	(-)	NNCWGATARNNNN	CCCTTCATCTGGT
M00075	V$GATA1_01	0.854393	10514	(-)	SNNGATNNNN	CTTCATCTGG
M00076	V$GATA2_01	0.841227	10514	(-)	NNNGATRNNN	CTTCATCTGG
M00077	V$GATA3_01	0.854231	10514	(-)	NNGATARNG	CTTCATCTG
M00127	V$GATA1_03	0.804018	10559	(-)	RNSNNGATAANNGN	TCTCCTAACCCCAC
M00075	V$GATA1_01	0.775913	10561	(-)	SNNGATNNNN	TCCTAACCCC
M00076	V$GATA2_01	0.784844	10561	(-)	NNNGATRNNN	TCCTAACCCC
M00075	V$GATA1_01	0.773939	10568	(-)	SNNGATNNNN	CCCACTCCCC
M00126	V$GATA1_02	0.789375	10575	(-)	NNNNNGATANKGNN	CCCAGTCTCTGTAA
M00076	V$GATA2_01	0.794317	10599	(-)	NNNGATRNNN	GCCCATGAGA
M00075	V$GATA1_01	0.771471	10632	(-)	SNNGATNNNN	TTTCATCAAG
M00076	V$GATA2_01	0.820929	10653	(-)	NNNGATRNNN	AACTATGATG
M00075	V$GATA1_01	0.804541	10656	(+)	SNNGATNNNN	TATGATGTCA
M00076	V$GATA2_01	0.843933	10656	(+)	NNNGATRNNN	TATGATGTCA
M00075	V$GATA1_01	0.863771	10680	(+)	SNNGATNNNN	ACTGATGGTA
M00076	V$GATA2_01	0.837167	10680	(+)	NNNGATRNNN	ACTGATGGTA
M00077	V$GATA3_01	0.844041	10681	(+)	NNGATARNG	CTGATGGTA
M00203	V$GATA_C	0.835974	10701	(-)	NGATAAGNMNN	AAAATGTATCA
M00126	V$GATA1_02	0.809063	10702	(-)	NNNNNGATANKGNN	AAATGTATCAATTA
M00075	V$GATA1_01	0.777394	10704	(-)	SNNGATNNNN	ATGTATCAAT
M00075	V$GATA1_01	0.773939	10741	(+)	SNNGATNNNN	TTTGATCCTT
M00127	V$GATA1_03	0.808427	10895	(+)	RNSNNGATAANNGN	AGGAAGAGAGAAGT
M00203	V$GATA_C	0.832867	10901	(+)	NGATAAGNMNN	AGAGAAGTCAA
M00075	V$GATA1_01	0.773939	10910	(+)	SNNGATNNNN	AATGATGAAT
M00126	V$GATA1_02	0.800312	10991	(-)	NNNNNGATANKGNN	AAATATATCTGTAC
M00128	V$GATA1_04	0.835478	10991	(-)	NNCWGATARNNNN	AAATATATCTGTA
M00075	V$GATA1_01	0.792201	10993	(-)	SNNGATNNNN	ATATATCTGT
M00076	V$GATA2_01	0.82995	10993	(-)	NNNGATRNNN	ATATATCTGT
M00075	V$GATA1_01	0.773939	11014	(-)	SNNGATNNNN	ATTCATCATT
M00203	V$GATA_C	0.830693	11021	(-)	NGATAAGNMNN	ATTTCTGATCT
M00126	V$GATA1_02	0.783438	11060	(-)	NNNNNGATANKGNN	TACCTTAGCTGTGA
M00075	V$GATA1_01	0.866732	11069	(+)	SNNGATNNNN	TGTGATGCGA
M00076	V$GATA2_01	0.850699	11069	(+)	NNNGATRNNN	TGTGATGCGA

M00075	V$GATA1_01	0.786278	11129	(-)	SNNGATNNNN	TGTCATCATA
M00076	V$GATA2_01	0.829048	11129	(-)	NNNGATRNNN	TGTCATCATA
M00076	V$GATA2_01	0.784393	11132	(+)	NNNGATRNNN	CATCATATTT
M00075	V$GATA1_01	0.8154	11146	(+)	SNNGATNNNN	GTTGATCCCC
M00075	V$GATA1_01	0.775913	11146	(-)	SNNGATNNNN	GTTGATCCCC
M00076	V$GATA2_01	0.8083	11146	(-)	NNNGATRNNN	GTTGATCCCC
M00075	V$GATA1_01	0.823791	11155	(+)	SNNGATNNNN	CAGGATTTAG
M00076	V$GATA2_01	0.826342	11155	(+)	NNNGATRNNN	CAGGATTTAG
M00127	V$GATA1_03	0.794708	11201	(-)	RNSNNGATAANNGN	TCTGTTATTCAGAC
M00162	V$OCT1_06	0.800781	32	(+)	CWNAWTKWSATRYN	CAGTCTTGGGTTCA
M00135	V$OCT1_01	0.805227	50	(-)	NNNNWTATGCAAATNTNNN	ATTATGTTTACATATGGCA
M00137	V$OCT1_03	0.86132	51	(-)	NNNRTAATNANNN	TTATGTTTACATA
M00162	V$OCT1_06	0.806641	52	(+)	CWNAWTKWSATRYN	TATGTTTACATATG
M00138	V$OCT1_04	0.826014	55	(+)	NNNNNNNWATGCAAATNNNWNNW	GTTTACATATGG-CAAAATTATGA
M00135	V$OCT1_01	0.752957	57	(+)	NNNNWTATGCAAATNTNNN	TTACATATGGCAAAATTAT
M00162	V$OCT1_06	0.842187	60	(-)	CWNAWTKWSATRYN	CATATGGCAAAATT
M00137	V$OCT1_03	0.849862	66	(-)	NNNRTAATNANNN	GCAAAATTATGAT
M00138	V$OCT1_04	0.819113	66	(+)	NNNNNNNWATGCAAATNNNWNNW	GCAAAATTATGATGAT-TTCACTT
M00135	V$OCT1_01	0.768218	68	(+)	NNNNWTATGCAAATNTNNN	AAAATTATGATGATTTCAC
M00248	V$OCT1_07	0.799449	71	(+)	TNTATGNTAATT	ATTATGATGATT
M00138	V$OCT1_04	0.785864	107	(-)	NNNNNNNWATGCAAATNNNWNNW	ACTCCTCATTTA-TATGCATTTGT
M00138	V$OCT1_04	0.83166	111	(-)	NNNNNNNWATGCAAATNNNWNNW	CTCATTTATATGCATTT-GTAACT
M00162	V$OCT1_06	0.894531	111	(+)	CWNAWTKWSATRYN	CTCATTTATATGCA
M00195	V$OCT1_Q6	0.854531	111	(-)	NNNNATGCAAATNAN	CTCATTTATATGCAT
M00138	V$OCT1_04	0.80092	112	(+)	NNNNNNNWATGCAAATNNNWNNW	TCATTTATATGCATTT-GTAACTT
M00135	V$OCT1_01	0.760969	113	(-)	NNNNWTATGCAAATNTNNN	CATTTATATGCATTTGTAA
M00135	V$OCT1_01	0.744945	114	(+)	NNNNWTATGCAAATNTNNN	ATTTATATGCATTTGTAAC
M00136	V$OCT1_02	0.865728	114	(+)	NNGAATATKCANNNN	ATTTATATGCATTTG
M00136	V$OCT1_02	0.785057	117	(-)	NNGAATATKCANNNN	TATATGCATTTGTAA
M00135	V$OCT1_01	0.730065	120	(+)	NNNNWTATGCAAATNTNNN	ATGCATTTGTAACTTTTCA
M00138	V$OCT1_04	0.804475	126	(+)	NNNNNNNWATGCAAATNNNWNNW	TTGTAACTTTTCAAA-TATTGATA
M00162	V$OCT1_06	0.821094	130	(-)	CWNAWTKWSATRYN	AACTTTTCAAATAT
M00136	V$OCT1_02	0.801299	133	(-)	NNGAATATKCANNNN	TTTTCAAATATTGAT

M00138	V$OCT1_04	0.797783	162	(-)	NNNNNNNNWATGCAAATNNNWNNW	AGCTTAAAAATGCATTT-GCAAAT
M00136	V$OCT1_02	0.810504	165	(+)	NNGAATATKCANNNN	TTAAAAATGCATTTG
M00136	V$OCT1_02	0.835679	168	(-)	NNGAATATKCANNNN	AAAATGCATTTGCAA
M00138	V$OCT1_04	0.828733	169	(+)	NNNNNNNNWATGCAAATNNNWNNW	AAATGCATTTGCAAATGTT-GATA
M00135	V$OCT1_01	0.814765	170	(-)	NNNNWTATGCAAATNTNNN	AATGCATTTGCAAATGTTG
M00135	V$OCT1_01	0.862457	171	(+)	NNNNWTATGCAAATNTNNN	ATGCATTTGCAAATGTTGA
M00136	V$OCT1_02	0.799675	171	(+)	NNGAATATKCANNNN	ATGCATTTGCAAATG
M00195	V$OCT1_Q6	0.80595	172	(-)	NNNNATGCAAATNAN	TGCATTTGCAAATGT
M00162	V$OCT1_06	0.821484	174	(-)	CWNAWTKWSATRYN	CATTTGCAAATGTT
M00138	V$OCT1_04	0.801757	175	(+)	NNNNNNNNWATGCAAATNNNWNNW	ATTTGCAAATGTTGATACTA-TCT
M00162	V$OCT1_06	0.878516	180	(+)	CWNAWTKWSATRYN	CAAATGTTGATACT
M00136	V$OCT1_02	0.795885	209	(-)	NNGAATATKCANNNN	TAGGTGAAAAATCAT
M00136	V$OCT1_02	0.780184	212	(+)	NNGAATATKCANNNN	GTGAAAAATCATATA
M00162	V$OCT1_06	0.821484	216	(-)	CWNAWTKWSATRYN	AAAATCATATAGTT
M00138	V$OCT1_04	0.817859	225	(-)	NNNNNNNNWATGCAAATNNNWNNW	TAGTTTTATAAACATCAAA-TAAT
M00162	V$OCT1_06	0.932031	235	(-)	CWNAWTKWSATRYN	AACATCAAATAATT
M00162	V$OCT1_06	0.834375	245	(+)	CWNAWTKWSATRYN	AATTTTTTGATGAC
M00137	V$OCT1_03	0.849862	259	(+)	NNNRTAATNANNN	TTGGTAACTAAAT
M00162	V$OCT1_06	0.821484	261	(+)	CWNAWTKWSATRYN	GGTAACTAAATTCT
M00162	V$OCT1_06	0.826562	261	(-)	CWNAWTKWSATRYN	GGTAACTAAATTCT
M00162	V$OCT1_06	0.808203	268	(-)	CWNAWTKWSATRYN	AAATTCTCATAGAT
M00248	V$OCT1_07	0.829341	269	(-)	TNTATGNTAATT	AATTCTCATAGA
M00138	V$OCT1_04	0.800711	272	(+)	NNNNNNNNWATGCAAATNNNWNNW	TCTCATA-GATGAAAAAGTAGATA
M00162	V$OCT1_06	0.867969	283	(+)	CWNAWTKWSATRYN	AAAAAGTAGATACA
M00138	V$OCT1_04	0.792974	284	(+)	NNNNNNNNWATGCAAATNNNWNNW	AAAAGTAGATA-CATAATTTCAGT
M00162	V$OCT1_06	0.804297	295	(+)	CWNAWTKWSATRYN	CATAATTTCAGTAA
M00248	V$OCT1_07	0.810473	298	(-)	TNTATGNTAATT	AATTTCAGTAAA
M00137	V$OCT1_03	0.850257	302	(+)	NNNRTAATNANNN	TCAGTAAAGACTT
M00138	V$OCT1_04	0.804266	319	(-)	NNNNNNNNWATGCAAATNNNWNNW	TGAAGTAAGTT-GAATTATCTTTG
M00136	V$OCT1_02	0.83595	325	(-)	NNGAATATKCANNNN	AAGTTGAATTATCTT
M00138	V$OCT1_04	0.790882	343	(+)	NNNNNNNNWATGCAAATNNNWNNW	TCAATTCTATGGAAAGTT-GTTAT
M00135	V$OCT1_01	0.741511	345	(+)	NNNNWTATGCAAATNTNNN	AATTCTATGGAAAGTTGTT
M00162	V$OCT1_06	0.804297	348	(-)	CWNAWTKWSATRYN	TCTATGGAAAGTTG

M00248	V$OCT1_07	0.874073	348	(+)	TNTATGNTAATT	TCTATGGAAAGT
M00138	V$OCT1_04	0.804684	355	(-)	NNNNNNNWATGCAAATNNNWNNW	AAAGTTGTTATGCAGAA-TAATTT
M00137	V$OCT1_03	0.845516	356	(-)	NNNRTAATNANNN	AAGTTGTTATGCA
M00138	V$OCT1_04	0.86261	356	(+)	NNNNNNNWATGCAAATNNNWNNW	AAGTTGTTATGCAGAA-TAATTTA
M00135	V$OCT1_01	0.731591	358	(+)	NNNNWTATGCAAATNTNNN	GTTGTTATGCAGAATAATT
M00136	V$OCT1_02	0.801029	358	(+)	NNGAATATKCANNNN	GTTGTTATGCAGAAT
M00138	V$OCT1_04	0.819113	366	(+)	NNNNNNNWATGCAAATNNNWNNW	GCAGAA-TAATTTATAATCTTTTT
M00138	V$OCT1_04	0.798202	368	(-)	NNNNNNNWATGCAAATNNNWNNW	AGAA-TAATTTATAATCTTTTTAA
M00195	V$OCT1_Q6	0.801583	371	(-)	NNNNATGCAAATNAN	ATAATTTATAATCTT
M00137	V$OCT1_03	0.868827	375	(+)	NNNRTAATNANNN	TTTATAATCTTTT
M00162	V$OCT1_06	0.816016	388	(-)	CWNAWTKWSATRYN	TAAGTGTCATGGAT
M00162	V$OCT1_06	0.834375	405	(-)	CWNAWTKWSATRYN	AGCATTTCTGGTAG
M00136	V$OCT1_02	0.814293	413	(-)	NNGAATATKCANNNN	TGGTAGAATATTTCA
M00162	V$OCT1_06	0.877734	419	(-)	CWNAWTKWSATRYN	AATATTTCAAGGCC
M00138	V$OCT1_04	0.788373	434	(-)	NNNNNNNWATGCAAATNNNWNNW	CTTTTTTCCATGCATAAGTTTAA
M00162	V$OCT1_06	0.88125	434	(+)	CWNAWTKWSATRYN	CTTTTTTCCATGCA
M00195	V$OCT1_Q6	0.805677	434	(-)	NNNNATGCAAATNAN	CTTTTTTCCATGCAT
M00138	V$OCT1_04	0.814722	435	(+)	NNNNNNNWATGCAAATNNNWNNW	TTTTTTCCATGCATAAGTTTAAT
M00136	V$OCT1_02	0.810233	440	(-)	NNGAATATKCANNNN	TCCATGCATAAGTTT
M00138	V$OCT1_04	0.80343	447	(+)	NNNNNNNWATGCAAATNNNWNNW	ATAAGTTTAATTAAATTAATTCT
M00248	V$OCT1_07	0.807717	452	(+)	TNTATGNTAATT	TTTAATTAAATT
M00162	V$OCT1_06	0.839844	453	(-)	CWNAWTKWSATRYN	TTAATTAAATTAAT
M00248	V$OCT1_07	0.812381	455	(-)	TNTATGNTAATT	AATTAAATTAAT
M00162	V$OCT1_06	0.826562	456	(+)	CWNAWTKWSATRYN	ATTAAATTAATTCT
M00162	V$OCT1_06	0.898437	476	(-)	CWNAWTKWSATRYN	GATATGTCAAAGAT
M00162	V$OCT1_06	0.831641	478	(-)	CWNAWTKWSATRYN	TATGTCAAAGATAG
M00138	V$OCT1_04	0.809285	482	(+)	NNNNNNNWATGCAAATNNNWNNW	TCAAAGATAGACAAATAGA-TATA
M00162	V$OCT1_06	0.855078	491	(+)	CWNAWTKWSATRYN	GACAAATAGATATA
M00135	V$OCT1_01	0.741129	510	(+)	NNNNWTATGCAAATNTNNN	CTACTTTTGTAAAGATGGT
M00137	V$OCT1_03	0.878309	515	(+)	NNNRTAATNANNN	TTTGTAAAGATGG
M00138	V$OCT1_04	0.797156	516	(+)	NNNNNNNWATGCAAATNNNWNNW	TTGTAAAGATGGTTATAGTT-GGA
M00138	V$OCT1_04	0.786073	525	(-)	NNNNNNNWATGCAAATNNNWNNW	TGGTTATAGTTGGA-TATTCAGAA
M00135	V$OCT1_01	0.784052	527	(-)	NNNNWTATGCAAATNTNNN	GTTATAGTTGGATATTCAG

M00162	V$OCT1_06	0.804297	529	(+)	CWNAWTKWSATRYN	TATAGTTGGATATT
M00136	V$OCT1_02	0.888468	531	(-)	NNGAATATKCANNNN	TAGTTGGATATTCAG
M00138	V$OCT1_04	0.798202	531	(-)	NNNNNNNWATGCAAATNNNWNNW	TAGTTGGA-TATTCAGAAGGATGC
M00136	V$OCT1_02	0.858148	534	(+)	NNGAATATKCANNNN	TTGGATATTCAGAAG
M00138	V$OCT1_04	0.803011	542	(+)	NNNNNNNWATGCAAATNNNWNNW	TCAGAAGGATGCAAGTGGA-TAAA
M00135	V$OCT1_01	0.747997	544	(+)	NNNNWTATGCAAATNTNNN	AGAAGGATGCAAGTGGATA
M00162	V$OCT1_06	0.803125	564	(+)	CWNAWTKWSATRYN	AAGTAGTAAATTTA
M00162	V$OCT1_06	0.839453	565	(-)	CWNAWTKWSATRYN	AGTAGTAAATTTAT
M00162	V$OCT1_06	0.813672	569	(+)	CWNAWTKWSATRYN	GTAAATTTATTTG
M00138	V$OCT1_04	0.7844	571	(-)	NNNNNNNWATGCAAATNNNWNNW	AAATTTATTTT-GGGTTTATAAAT
M00138	V$OCT1_04	0.783982	579	(-)	NNNNNNNWATGCAAATNNNWNNW	TTTGGGTTTATAAA-TATGTCTTC
M00138	V$OCT1_04	0.7867	580	(+)	NNNNNNNWATGCAAATNNNWNNW	TTGGGTTTATAAA-TATGTCTTCT
M00162	V$OCT1_06	0.804297	583	(-)	CWNAWTKWSATRYN	GGTTTATAAATATG
M00136	V$OCT1_02	0.778289	585	(-)	NNGAATATKCANNNN	TTTATAAATATGTCT
M00137	V$OCT1_03	0.877914	585	(+)	NNNRTAATNANNN	TTTATAAATATGT
M00138	V$OCT1_04	0.7821	585	(-)	NNNNNNNWATGCAAATNNNWNNW	TTTATAAA-TATGTCTTCTTTACT
M00136	V$OCT1_02	0.778831	588	(+)	NNGAATATKCANNNN	ATAAATATGTCTTCT
M00137	V$OCT1_03	0.877519	597	(-)	NNNRTAATNANNN	TCTTCTTTACTAA
M00162	V$OCT1_06	0.813672	610	(-)	CWNAWTKWSATRYN	TGCATTCAGATTCT
M00195	V$OCT1_Q6	0.799672	644	(+)	NNNNATGCAAATNAN	GTATATATAAAATAT
M00162	V$OCT1_06	0.847266	645	(-)	CWNAWTKWSATRYN	TATATATAAAATAT
M00162	V$OCT1_06	0.821484	646	(+)	CWNAWTKWSATRYN	ATATATAAAATATG
M00162	V$OCT1_06	0.804297	647	(-)	CWNAWTKWSATRYN	TATATAAAATATGG
M00162	V$OCT1_06	0.808203	682	(-)	CWNAWTKWSATRYN	AGTATGTATGATCT
M00138	V$OCT1_04	0.794437	685	(-)	NNNNNNNWATGCAAATNNNWNNW	ATGTATGATCTGTATTTTTTC-TA
M00195	V$OCT1_Q6	0.838155	689	(-)	NNNNATGCAAATNAN	ATGATCTGTATTTTT
M00135	V$OCT1_01	0.732163	696	(+)	NNNNWTATGCAAATNTNNN	GTATTTTTTCTAATTTTTC
M00248	V$OCT1_07	0.838457	699	(+)	TNTATGNTAATT	TTTTTTCTAATT
M00138	V$OCT1_04	0.843162	728	(+)	NNNNNNNWATGCAAATNNNWNNW	TCTAG-CACATGTAAATGAAAGTA
M00135	V$OCT1_01	0.770889	730	(+)	NNNNWTATGCAAATNTNNN	TAGCACATGTAAATGAAAG
M00195	V$OCT1_Q6	0.84607	732	(+)	NNNNATGCAAATNAN	GCACATGTAAATGAA
M00162	V$OCT1_06	0.876172	733	(-)	CWNAWTKWSATRYN	CACATGTAAATGAA
M00138	V$OCT1_04	0.833333	734	(+)	NNNNNNNWATGCAAATNNNWNNW	ACATGTAAATGAAAGTATTTCTA

M00162	V$OCT1_06	0.924609	739	(-)	CWNAWTKWSATRYN	TAAATGAAAGTATT
M00162	V$OCT1_06	0.808203	747	(-)	CWNAWTKWSATRYN	AGTATTTCTAGGTT
M00137	V$OCT1_03	0.884631	761	(+)	NNNRTAATNANNN	CTTATAATTTGAT
M00162	V$OCT1_06	0.825	763	(+)	CWNAWTKWSATRYN	TATAATTTGATGGA
M00162	V$OCT1_06	0.855469	800	(-)	CWNAWTKWSATRYN	GACATCTGAATCTT
M00162	V$OCT1_06	0.842187	806	(-)	CWNAWTKWSATRYN	TGAATCTTATGATT
M00162	V$OCT1_06	0.826562	808	(+)	CWNAWTKWSATRYN	AATCTTATGATTCT
M00162	V$OCT1_06	0.88125	840	(+)	CWNAWTKWSATRYN	CATATAGTCATGTC
M00162	V$OCT1_06	0.842187	845	(+)	CWNAWTKWSATRYN	AGTCATGTCATGGA
M00162	V$OCT1_06	0.837109	846	(-)	CWNAWTKWSATRYN	GTCATGTCATGGAT
M00136	V$OCT1_02	0.781267	851	(-)	NNGAATATKCANNNN	GTCATGGATATACCA
M00138	V$OCT1_04	0.802384	852	(+)	NNNNNNNWATGCAAATNNNWNNW	TCATGGATATAC-CAATGCAGCAA
M00135	V$OCT1_01	0.742274	854	(+)	NNNNWTATGCAAATNTNNN	ATGGATATACCAATGCAGC
M00138	V$OCT1_04	0.784818	858	(+)	NNNNNNNWATGCAAATNNNWNNW	ATATACCAATGCAGCAAAA-TATA
M00162	V$OCT1_06	0.803906	867	(-)	CWNAWTKWSATRYN	TGCAGCAAAATATA
M00162	V$OCT1_06	0.88125	875	(-)	CWNAWTKWSATRYN	AATATAAAATAATG
M00136	V$OCT1_02	0.779101	904	(+)	NNGAATATKCANNNN	AAAATAATTCATGGA
M00162	V$OCT1_06	0.800391	910	(+)	CWNAWTKWSATRYN	ATTCATGGAAGATA
M00162	V$OCT1_06	0.873437	983	(+)	CWNAWTKWSATRYN	CTAAAATAGATTTA
M00162	V$OCT1_06	0.847656	989	(-)	CWNAWTKWSATRYN	TAGATTTAAAAGAG
M00162	V$OCT1_06	0.85	1000	(+)	CWNAWTKWSATRYN	GAGAAAGACATTTT
M00162	V$OCT1_06	0.855078	1001	(-)	CWNAWTKWSATRYN	AGAAAGACATTTTT
M00138	V$OCT1_04	0.809912	1007	(-)	NNNNNNNWATGCAAATNNNWNNW	ACATTTTTTTAAATGTCAGATT
M00162	V$OCT1_06	0.842187	1011	(+)	CWNAWTKWSATRYN	TTTTTTTAAATGTC
M00162	V$OCT1_06	0.803906	1016	(+)	CWNAWTKWSATRYN	TTAAATGTCAGATT
M00162	V$OCT1_06	0.85	1017	(-)	CWNAWTKWSATRYN	TAAATGTCAGATTA
M00162	V$OCT1_06	0.808594	1029	(-)	CWNAWTKWSATRYN	TAAATTAAATGGGT
M00138	V$OCT1_04	0.850899	1054	(-)	NNNNNNNWATGCAAATNNNWNNW	TAGTTTACTATA-CATTTTAACAA
M00138	V$OCT1_04	0.820786	1060	(-)	NNNNNNNWATGCAAATNNNWNNW	ACTATA-CATTTTAACAAATCTAA
M00135	V$OCT1_01	0.731973	1064	(-)	NNNNWTATGCAAATNTNNN	TACATTTTAACAAATCTAA
M00162	V$OCT1_06	0.90625	1074	(-)	CWNAWTKWSATRYN	CAAATCTAATGATC
M00137	V$OCT1_03	0.842355	1076	(+)	NNNRTAATNANNN	AATCTAATGATCT
M00136	V$OCT1_02	0.776665	1089	(-)	NNGAATATKCANNNN	AAGAGGAATTTTTTT

M00162	V$OCT1_06	0.826562	1097	(+)	CWNAWTKWSATRYN	TTTTTTTTAATTTT
M00248	V$OCT1_07	0.831673	1097	(+)	TNTATGNTAATT	TTTTTTTTAATT
M00138	V$OCT1_04	0.813258	1099	(-)	NNNNNNNWATGCAAATNNNWNNW	TTTTTTAATTTTAACTGAAA-TAT
M00138	V$OCT1_04	0.789419	1100	(-)	NNNNNNNWATGCAAATNNNWNNW	TTTTTAATTTTAACTGAAA-TATT
M00138	V$OCT1_04	0.810749	1104	(+)	NNNNNNNWATGCAAATNNNWNNW	TAATTTTAACTGAAA-TATTTAAA
M00138	V$OCT1_04	0.788791	1105	(-)	NNNNNNNWATGCAAATNNNWNNW	AATTTTAACTGAAA-TATTTAAAA
M00162	V$OCT1_06	0.860547	1109	(+)	CWNAWTKWSATRYN	TTAACTGAAATATT
M00162	V$OCT1_06	0.828906	1110	(-)	CWNAWTKWSATRYN	TAACTGAAATATTT
M00138	V$OCT1_04	0.833752	1112	(+)	NNNNNNNWATGCAAATNNNWNNW	ACTGAAATATTTAAAAATT-GAGT
M00135	V$OCT1_01	0.769935	1114	(+)	NNNNWTATGCAAATNTNNN	TGAAATATTTAAAAATTGA
M00136	V$OCT1_02	0.792907	1114	(+)	NNGAATATKCANNNN	TGAAATATTTAAAAA
M00162	V$OCT1_06	0.847266	1117	(+)	CWNAWTKWSATRYN	AATATTTAAAAATT
M00162	V$OCT1_06	0.911328	1117	(-)	CWNAWTKWSATRYN	AATATTTAAAAATT
M00136	V$OCT1_02	0.776123	1126	(-)	NNGAATATKCANNNN	AAATTGAGTAGTATT
M00138	V$OCT1_04	0.804893	1130	(+)	NNNNNNNWATGCAAATNNNWNNW	TGAGTAGTATTTAAA-TATGAATG
M00138	V$OCT1_04	0.819741	1131	(-)	NNNNNNNWATGCAAATNNNWNNW	GAGTAGTATTTAAA-TATGAATGC
M00135	V$OCT1_01	0.793399	1132	(+)	NNNNWTATGCAAATNTNNN	AGTAGTATTTAAATATGAA
M00136	V$OCT1_02	0.776394	1132	(+)	NNGAATATKCANNNN	AGTAGTATTTAAATA
M00135	V$OCT1_01	0.769935	1133	(-)	NNNNWTATGCAAATNTNNN	GTAGTATTTAAATATGAAT
M00162	V$OCT1_06	0.903906	1135	(+)	CWNAWTKWSATRYN	AGTATTTAAATATG
M00162	V$OCT1_06	0.963672	1135	(-)	CWNAWTKWSATRYN	AGTATTTAAATATG
M00248	V$OCT1_07	0.793301	1135	(+)	TNTATGNTAATT	AGTATTTAAATA
M00136	V$OCT1_02	0.81294	1137	(-)	NNGAATATKCANNNN	TATTTAAATATGAAT
M00138	V$OCT1_04	0.840234	1137	(-)	NNNNNNNWATGCAAATNNNWNNW	TATTTAAA-TATGAATGCCTCTCT
M00137	V$OCT1_03	0.903595	1174	(-)	NNNRTAATNANNN	ACCCTATTACCAC
M00138	V$OCT1_04	0.787118	1212	(+)	NNNNNNNWATGCAAATNNNWNNW	GAGTGTTAGTGAATAAAAA-TATT
M00138	V$OCT1_04	0.816604	1216	(+)	NNNNNNNWATGCAAATNNNWNNW	GTTAGTGAATAAAAA-TATTAATA
M00136	V$OCT1_02	0.815106	1217	(-)	NNGAATATKCANNNN	TTAGTGAATAAAAAT
M00138	V$OCT1_04	0.823505	1217	(-)	NNNNNNNWATGCAAATNNNWNNW	TTAGTGAATAAAAATATTAA-TAA
M00137	V$OCT1_03	0.857369	1221	(+)	NNNRTAATNANNN	TGAATAAAAATAT
M00162	V$OCT1_06	0.860547	1221	(-)	CWNAWTKWSATRYN	TGAATAAAAATATT
M00136	V$OCT1_02	0.796697	1223	(-)	NNGAATATKCANNNN	AATAAAAATATTAAT
M00138	V$OCT1_04	0.79611	1223	(-)	NNNNNNNWATGCAAATNNNWNNW	AATAAAAATATTAATAAGA-GACT

M00248	V$OCT1_07	0.812381	1223	(-)	TNTATGNTAATT	AATAAAAATATT
M00138	V$OCT1_04	0.786909	1224	(+)	NNNNNNNWATGCAAATNNNWNNW	ATAAAAATATTAATAAGA-GACTG
M00136	V$OCT1_02	0.777206	1226	(+)	NNGAATATKCANNNN	AAAAATATTAATAAG
M00195	V$OCT1_Q6	0.818504	1226	(+)	NNNNATGCAAATNAN	AAAAATATTAATAAG
M00162	V$OCT1_06	0.809766	1227	(-)	CWNAWTKWSATRYN	AAAATATTAATAAG
M00162	V$OCT1_06	0.81875	1229	(-)	CWNAWTKWSATRYN	AATATTAATAAGAG
M00162	V$OCT1_06	0.808984	1240	(+)	CWNAWTKWSATRYN	GAGACTGGTATTTC
M00162	V$OCT1_06	0.817187	1273	(-)	CWNAWTKWSATRYN	AGCAAGATAATTTG
M00138	V$OCT1_04	0.81514	1275	(-)	NNNNNNNWATGCAAATNNNWNNW	CAAGATAATTTGAATGAT-TAACA
M00137	V$OCT1_03	0.847491	1276	(+)	NNNRTAATNANNN	AAGATAATTTGAA
M00135	V$OCT1_01	0.772987	1277	(-)	NNNNWTATGCAAATNTNNN	AGATAATTTGAATGATTAA
M00161	V$OCT1_05	0.875145	1279	(+)	MKNATTTGCATAYY	ATAATTTGAATGAT
M00162	V$OCT1_06	0.839844	1279	(+)	CWNAWTKWSATRYN	ATAATTTGAATGAT
M00195	V$OCT1_Q6	0.844705	1279	(-)	NNNNATGCAAATNAN	ATAATTTGAATGATT
M00248	V$OCT1_07	0.839093	1281	(-)	TNTATGNTAATT	AATTTGAATGAT
M00138	V$OCT1_04	0.844207	1284	(-)	NNNNNNNWATGCAAATNNNWNNW	TTGAATGATTAACATTTA-TATTG
M00135	V$OCT1_01	0.744945	1286	(-)	NNNNWTATGCAAATNTNNN	GAATGATTAACATTTATAT
M00162	V$OCT1_06	0.847656	1288	(+)	CWNAWTKWSATRYN	ATGATTAACATTTA
M00195	V$OCT1_Q6	0.873908	1288	(-)	NNNNATGCAAATNAN	ATGATTAACATTTAT
M00138	V$OCT1_04	0.790673	1289	(+)	NNNNNNNWATGCAAATNNNWNNW	TGATTAACATTTATATTGAA-TAG
M00248	V$OCT1_07	0.836337	1290	(-)	TNTATGNTAATT	GATTAACATTTA
M00136	V$OCT1_02	0.781267	1301	(-)	NNGAATATKCANNNN	ATATTGAATAGCAAA
M00138	V$OCT1_04	0.80092	1302	(+)	NNNNNNNWATGCAAATNNNWNNW	TATTGAA-TAGCAAAATTAAAAAT
M00138	V$OCT1_04	0.805939	1308	(+)	NNNNNNNWATGCAAATNNNWNNW	ATAGCAAAATTAAAAATTTAAAT
M00138	V$OCT1_04	0.789837	1310	(-)	NNNNNNNWATGCAAATNNNWNNW	AG-CAAAATTAAAAATTTAAAT-TA
M00162	V$OCT1_06	0.88125	1312	(+)	CWNAWTKWSATRYN	CAAAATTAAAAATT
M00162	V$OCT1_06	0.890625	1313	(-)	CWNAWTKWSATRYN	AAAATTAAAAATTT
M00138	V$OCT1_04	0.869511	1315	(+)	NNNNNNNWATGCAAATNNNWNNW	AATTAAAAATTTAAATTAG-TAAA
M00138	V$OCT1_04	0.860937	1316	(-)	NNNNNNNWATGCAAATNNNWNNW	ATTAAAAATTTAAATTAG-TAAAA
M00135	V$OCT1_01	0.732354	1317	(+)	NNNNWTATGCAAATNTNNN	TTAAAAATTTAAATTAGTA
M00135	V$OCT1_01	0.741892	1318	(-)	NNNNWTATGCAAATNTNNN	TAAAAATTTAAATTAGTAA
M00162	V$OCT1_06	0.834375	1319	(+)	CWNAWTKWSATRYN	AAAAATTTAAATTA

M00195	V$OCT1_Q6	0.864083	1319	(+)	NNNNATGCAAATNAN	AAAAATTTAAATTAG
M00162	V$OCT1_06	0.834766	1320	(+)	CWNAWTKWSATRYN	AAAATTTAAATTAG
M00162	V$OCT1_06	0.942969	1320	(-)	CWNAWTKWSATRYN	AAAATTTAAATTAG
M00248	V$OCT1_07	0.832309	1320	(+)	TNTATGNTAATT	AAAATTTAAATT
M00138	V$OCT1_04	0.806148	1322	(-)	NNNNNNNWATGCAAATNNNWNNW	AATTTAAATTAGTAAAAA-TATTA
M00248	V$OCT1_07	0.806869	1322	(-)	TNTATGNTAATT	AATTTAAATTAG
M00135	V$OCT1_01	0.754865	1324	(-)	NNNNWTATGCAAATNTNNN	TTTAAATTAGTAAAAATAT
M00138	V$OCT1_04	0.812631	1324	(+)	NNNNNNNWATGCAAATNNNWNNW	TTTAAATTAGTAAAAA-TATTAAA
M00248	V$OCT1_07	0.800297	1328	(-)	TNTATGNTAATT	AATTAGTAAAAA
M00137	V$OCT1_03	0.870802	1330	(+)	NNNRTAATNANNN	TTAGTAAAAATAT
M00138	V$OCT1_04	0.815767	1331	(+)	NNNNNNNWATGCAAATNNNWNNW	TAGTAAAAA-TATTAAACAAATGA
M00136	V$OCT1_02	0.787223	1335	(+)	NNGAATATKCANNNN	AAAAATATTAAACAA
M00138	V$OCT1_04	0.784818	1336	(+)	NNNNNNNWATGCAAATNNNWNNW	AAAA-TATTAAACAAATGAAACTA
M00162	V$OCT1_06	0.826562	1338	(-)	CWNAWTKWSATRYN	AATATTAAACAAAT
M00138	V$OCT1_04	0.795483	1342	(+)	NNNNNNNWATGCAAATNNNWNNW	TTAAACAAATGAAAC-TATTTTTA
M00162	V$OCT1_06	0.855469	1347	(-)	CWNAWTKWSATRYN	CAAATGAAACTATT
M00138	V$OCT1_04	0.837097	1350	(+)	NNNNNNNWATGCAAATNNNWNNW	ATGAAAC-TATTTTTATTATAACA
M00138	V$OCT1_04	0.819532	1351	(-)	NNNNNNNWATGCAAATNNNWNNW	TGAAAC-TATTTTTATTATAACAC
M00248	V$OCT1_07	0.817257	1355	(+)	TNTATGNTAATT	ACTATTTTTATT
M00162	V$OCT1_06	0.831641	1356	(+)	CWNAWTKWSATRYN	CTATTTTTATTATA
M00137	V$OCT1_03	0.898064	1359	(-)	NNNRTAATNANNN	TTTTTATTATAAC
M00138	V$OCT1_04	0.79862	1396	(-)	NNNNNNNWATGCAAATNNNWNNW	GATTTTTTTTTCAT-ATGAAACC
M00138	V$OCT1_04	0.820995	1397	(+)	NNNNNNNWATGCAAATNNNWNNW	ATTTTTTTTTCATATGAAAC-CA
M00135	V$OCT1_01	0.763068	1398	(-)	NNNNWTATGCAAATNTNNN	TTTTTTTTTCATATGAAA
M00162	V$OCT1_06	0.847266	1400	(+)	CWNAWTKWSATRYN	TTTTTTTTCATATG
M00162	V$OCT1_06	0.826562	1412	(-)	CWNAWTKWSATRYN	TGAAACCAAATTTT
M00162	V$OCT1_06	0.852734	1418	(+)	CWNAWTKWSATRYN	CAAATTTTATTATC
M00162	V$OCT1_06	0.842578	1418	(-)	CWNAWTKWSATRYN	CAAATTTTATTATC
M00137	V$OCT1_03	0.915844	1421	(-)	NNNRTAATNANNN	ATTTTATTATCAG
M00137	V$OCT1_03	0.856183	1489	(-)	NNNRTAATNANNN	TCATCCTTACCTG
M00162	V$OCT1_06	0.85	1490	(+)	CWNAWTKWSATRYN	CATCCTTACCTGCC
M00136	V$OCT1_02	0.772875	1604	(-)	NNGAATATKCANNNN	ACTGAGAATAATAAA
M00137	V$OCT1_03	0.887396	1608	(+)	NNNRTAATNANNN	AGAATAATAAAGT

M00162	V$OCT1_06	0.813672	1610	(-)	CWNAWTKWSATRYN	AATAATAAAGTGTT
M00137	V$OCT1_03	0.858949	1631	(-)	NNNRTAATNANNN	CTCTAATTAGCTA
M00138	V$OCT1_04	0.799247	1643	(+)	NNNNNNNWATGCAAATNNNWNNW	ATTGAAGTCTTGA-TATTAAAACT
M00138	V$OCT1_04	0.780217	1650	(-)	NNNNNNNWATGCAAATNNNWNNW	TCTTGATATTAAAACTATGA-TAA
M00162	V$OCT1_06	0.826562	1654	(-)	CWNAWTKWSATRYN	GATATTAAAACTAT
M00138	V$OCT1_04	0.794019	1658	(+)	NNNNNNNWATGCAAATNNNWNNW	TTAAAACTATGA-TAATCCTTCAC
M00135	V$OCT1_01	0.748951	1660	(+)	NNNNWTATGCAAATNTNNN	AAAACTATGATAATCCTTC
M00195	V$OCT1_Q6	0.814138	1662	(+)	NNNNATGCAAATNAN	AACTATGATAATCCT
M00161	V$OCT1_05	0.880292	1663	(-)	MKNATTTGCATAYY	ACTATGATAATCCT
M00248	V$OCT1_07	0.804325	1663	(+)	TNTATGNTAATT	ACTATGATAATC
M00162	V$OCT1_06	0.821484	1672	(+)	CWNAWTKWSATRYN	ATCCTTCACATTTT
M00195	V$OCT1_Q6	0.812773	1672	(-)	NNNNATGCAAATNAN	ATCCTTCACATTTTG
M00162	V$OCT1_06	0.817187	1673	(-)	CWNAWTKWSATRYN	TCCTTCACATTTTG
M00137	V$OCT1_03	0.879494	1685	(-)	NNNRTAATNANNN	TGGTCATTATTAT
M00137	V$OCT1_03	0.924931	1688	(-)	NNNRTAATNANNN	TCATTATTATGTA
M00136	V$OCT1_02	0.771251	1690	(+)	NNGAATATKCANNNN	ATTATTATGTAGTTC
M00162	V$OCT1_06	0.808203	1696	(+)	CWNAWTKWSATRYN	ATGTAGTTCATTCA
M00162	V$OCT1_06	0.860547	1697	(-)	CWNAWTKWSATRYN	TGTAGTTCATTCAG
M00162	V$OCT1_06	0.916797	1732	(+)	CWNAWTKWSATRYN	CTGCTTGAAATATA
M00162	V$OCT1_06	0.842578	1738	(-)	CWNAWTKWSATRYN	GAAATATAAATCTC
M00138	V$OCT1_04	0.7867	1746	(+)	NNNNNNNWATGCAAATNNNWNNW	AATCTCTCATGATAAAAAA-TAAT
M00138	V$OCT1_04	0.799247	1756	(+)	NNNNNNNWATGCAAATNNNWNNW	GATAAAAAATAATAATAA-TAATA
M00138	V$OCT1_04	0.827269	1757	(-)	NNNNNNNWATGCAAATNNNWNNW	ATAAAAAATAATAATAA-TAATAA
M00138	V$OCT1_04	0.819322	1759	(+)	NNNNNNNWATGCAAATNNNWNNW	AAAAAATAATAATAATAA-TAAAA
M00138	V$OCT1_04	0.814722	1760	(-)	NNNNNNNWATGCAAATNNNWNNW	AAAAATAATAATAATAA-TAAAAA
M00137	V$OCT1_03	0.878704	1761	(+)	NNNRTAATNANNN	AAAATAATAATAA
M00138	V$OCT1_04	0.816604	1762	(+)	NNNNNNNWATGCAAATNNNWNNW	AAATAATAATAATAA-TAAAAAAG
M00138	V$OCT1_04	0.813258	1763	(-)	NNNNNNNWATGCAAATNNNWNNW	AATAATAATAATAA-TAAAAAAGT
M00137	V$OCT1_03	0.899644	1764	(+)	NNNRTAATNANNN	ATAATAATAATAA
M00138	V$OCT1_04	0.791092	1765	(+)	NNNNNNNWATGCAAATNNNWNNW	TAATAATAATAA-TAAAAAAGTAA
M00137	V$OCT1_03	0.899644	1767	(+)	NNNRTAATNANNN	ATAATAATAATAA
M00138	V$OCT1_04	0.859682	1768	(+)	NNNNNNNWATGCAAATNNNWNNW	TAATAATAA-TAAAAAAGTAAAGA
M00137	V$OCT1_03	0.880284	1770	(+)	NNNRTAATNANNN	ATAATAATAAAAA

M00195	V$OCT1_Q6	0.809225	1778	(+)	NNNNATGCAAATNAN	AAAAAAGTAAAGAAT
M00162	V$OCT1_06	0.821094	1779	(-)	CWNAWTKWSATRYN	AAAAAGTAAAGAAT
M00162	V$OCT1_06	0.860547	1804	(-)	CWNAWTKWSATRYN	GGTATCAAACAATG
M00162	V$OCT1_06	0.9375	1826	(-)	CWNAWTKWSATRYN	AGCATCAAAGAATG
M00162	V$OCT1_06	0.883984	1833	(+)	CWNAWTKWSATRYN	AAGAATGACATGAA
M00162	V$OCT1_06	0.9375	1834	(-)	CWNAWTKWSATRYN	AGAATGACATGAAG
M00138	V$OCT1_04	0.833542	1865	(-)	NNNNNNNWATGCAAATNNNWNNW	TTACAGAATTAGCGTATTT-GTAA
M00135	V$OCT1_01	0.838039	1867	(-)	NNNNWTATGCAAATNTNNN	ACAGAATTAGCGTATTTGT
M00161	V$OCT1_05	0.903702	1869	(+)	MKNATTTGCATAYY	AGAATTAGCGTATT
M00195	V$OCT1_Q6	0.800491	1869	(-)	NNNNATGCAAATNAN	AGAATTAGCGTATTT
M00248	V$OCT1_07	0.877888	1871	(-)	TNTATGNTAATT	AATTAGCGTATT
M00135	V$OCT1_01	0.74132	1875	(-)	NNNNWTATGCAAATNTNNN	AGCGTATTTGTAAACATAT
M00195	V$OCT1_Q6	0.824782	1877	(-)	NNNNATGCAAATNAN	CGTATTTGTAAACAT
M00137	V$OCT1_03	0.888977	1881	(+)	NNNRTAATNANNN	TTTGTAAACATAT
M00162	V$OCT1_06	0.813281	1893	(-)	CWNAWTKWSATRYN	TGTGTCCAATGTAT
M00162	V$OCT1_06	0.855078	1902	(-)	CWNAWTKWSATRYN	TGTATGTCTTTACC
M00137	V$OCT1_03	0.900435	1905	(-)	NNNRTAATNANNN	ATGTCTTTACCAA
M00162	V$OCT1_06	0.873437	1917	(-)	CWNAWTKWSATRYN	AATATAACATAAAG
M00248	V$OCT1_07	0.792453	1918	(-)	TNTATGNTAATT	ATATAACATAAA
M00162	V$OCT1_06	0.813672	1934	(-)	CWNAWTKWSATRYN	TATAGTCCAGTATT
M00135	V$OCT1_01	0.783098	2028	(+)	NNNNWTATGCAAATNTNNN	CTCATTCTGCTGATTTGGA
M00135	V$OCT1_01	0.750858	2035	(-)	NNNNWTATGCAAATNTNNN	TGCTGATTTGGAGATTGAA
M00162	V$OCT1_06	0.844922	2037	(+)	CWNAWTKWSATRYN	CTGATTTGGAGATT
M00195	V$OCT1_Q6	0.823144	2037	(-)	NNNNATGCAAATNAN	CTGATTTGGAGATTG
M00162	V$OCT1_06	0.842187	2045	(+)	CWNAWTKWSATRYN	GAGATTGAATTGTT
M00162	V$OCT1_06	0.804297	2077	(+)	CWNAWTKWSATRYN	TGAAATTAGATAAT
M00162	V$OCT1_06	0.834766	2078	(-)	CWNAWTKWSATRYN	GAAATTAGATAATG
M00138	V$OCT1_04	0.916562	2081	(+)	NNNNNNNWATGCAAATNNNWNNW	ATTAGA-TAATGTAAATTAAAATA
M00135	V$OCT1_01	0.790538	2083	(+)	NNNNWTATGCAAATNTNNN	TAGATAATGTAAATTAAAA
M00137	V$OCT1_03	0.854208	2083	(+)	NNNRTAATNANNN	TAGATAATGTAAA
M00162	V$OCT1_06	0.834375	2085	(+)	CWNAWTKWSATRYN	GATAATGTAAATTA
M00195	V$OCT1_Q6	0.865721	2085	(+)	NNNNATGCAAATNAN	GATAATGTAAATTAA
M00162	V$OCT1_06	0.804297	2086	(-)	CWNAWTKWSATRYN	ATAATGTAAATTAA

M00248	V$OCT1_07	0.891032	2086	(+)	TNTATGNTAATT	ATAATGTAAATT
M00138	V$OCT1_04	0.81514	2087	(+)	NNNNNNNWATGCAAATNNNWNNW	TAATGTAAATTAAAA-TAACCCTT
M00138	V$OCT1_04	0.823296	2088	(-)	NNNNNNNWATGCAAATNNNWNNW	AATGTAAATTAAAA-TAACCCTTC
M00135	V$OCT1_01	0.771461	2090	(-)	NNNNWTATGCAAATNTNNN	TGTAAATTAAAATAACCCT
M00195	V$OCT1_Q6	0.830786	2091	(+)	NNNNATGCAAATNAN	GTAAATTAAAATAAC
M00162	V$OCT1_06	0.908984	2092	(-)	CWNAWTKWSATRYN	TAAATTAAAATAAC
M00138	V$OCT1_04	0.781681	2094	(-)	NNNNNNNWATGCAAATNNNWNNW	AATTAAAA-TAACCCTTCTCAAAA
M00248	V$OCT1_07	0.896544	2094	(-)	TNTATGNTAATT	AATTAAAATAAC
M00136	V$OCT1_02	0.839469	2126	(+)	NNGAATATKCANNNN	AAAACTATTCAGTTA
M00135	V$OCT1_01	0.790538	2135	(-)	NNNNWTATGCAAATNTNNN	CAGTTACTTACATAAAGCA
M00162	V$OCT1_06	0.860547	2137	(+)	CWNAWTKWSATRYN	GTTACTTACATAAA
M00195	V$OCT1_Q6	0.804858	2137	(-)	NNNNATGCAAATNAN	GTTACTTACATAAAG
M00162	V$OCT1_06	0.800391	2147	(-)	CWNAWTKWSATRYN	TAAAGCAAAAAGTT
M00162	V$OCT1_06	0.804297	2152	(+)	CWNAWTKWSATRYN	CAAAAAGTTATTCT
M00137	V$OCT1_03	0.888186	2201	(+)	NNNRTAATNANNN	GGAGTAAACAGGA
M00138	V$OCT1_04	0.821205	2225	(+)	NNNNNNNWATGCAAATNNNWNNW	AGAAAACAATTCAGA-TAAAATAA
M00248	V$OCT1_07	0.799237	2232	(-)	TNTATGNTAATT	AATTCAGATAAA
M00162	V$OCT1_06	0.808594	2248	(+)	CWNAWTKWSATRYN	TGGTCTTGGATGCT
M00135	V$OCT1_01	0.739985	2254	(-)	NNNNWTATGCAAATNTNNN	TGGATGCTTGGATATTGAA
M00162	V$OCT1_06	0.834375	2256	(+)	CWNAWTKWSATRYN	GATGCTTGGATATT
M00136	V$OCT1_02	0.841094	2258	(-)	NNGAATATKCANNNN	TGCTTGGATATTGAA
M00136	V$OCT1_02	0.771792	2261	(+)	NNGAATATKCANNNN	TTGGATATTGAAACT
M00162	V$OCT1_06	0.829297	2272	(+)	CWNAWTKWSATRYN	AACTATCAAATACA
M00162	V$OCT1_06	0.852734	2273	(-)	CWNAWTKWSATRYN	ACTATCAAATACAG
M00162	V$OCT1_06	0.817187	2278	(+)	CWNAWTKWSATRYN	CAAATACAGATGCA
M00138	V$OCT1_04	0.815767	2279	(+)	NNNNNNNWATGCAAATNNNWNNW	AAATACAGATGCAAGTTTCTGAA
M00135	V$OCT1_01	0.824685	2281	(+)	NNNNWTATGCAAATNTNNN	ATACAGATGCAAGTTTCTG
M00162	V$OCT1_06	0.842578	2284	(-)	CWNAWTKWSATRYN	CAGATGCAAGTTTC
M00162	V$OCT1_06	0.898437	2293	(+)	CWNAWTKWSATRYN	GTTTCTGAAATGCT
M00162	V$OCT1_06	0.855078	2301	(+)	CWNAWTKWSATRYN	AATGCTGAGATACT
M00162	V$OCT1_06	0.813672	2313	(+)	CWNAWTKWSATRYN	CTCTATTGGACTCT
M00162	V$OCT1_06	0.842187	2320	(-)	CWNAWTKWSATRYN	GGACTCTAATTTTC
M00138	V$OCT1_04	0.83187	2322	(-)	NNNNNNNWATGCAAATNNNWNNW	ACTCTAATTTTCTA-TATCCATAT

M00135	V$OCT1_01	0.749523	2324	(-)	NNNNWTATGCAAATNTNNN	TCTAATTTTCTATATCCAT
M00138	V$OCT1_04	0.788582	2324	(-)	NNNNNNNWATGCAAATNNNWNNW	TCTAATTTTCTA-TATCCATATTT
M00138	V$OCT1_04	0.823505	2328	(-)	NNNNNNNWATGCAAATNNNWNNW	ATTTTCTATATCCATATTTA-GAA
M00138	V$OCT1_04	0.810121	2329	(+)	NNNNNNNWATGCAAATNNNWNNW	TTTTCTATATCCATATTTA-GAAA
M00135	V$OCT1_01	0.780427	2330	(-)	NNNNWTATGCAAATNTNNN	TTTCTATATCCATATTTAG
M00135	V$OCT1_01	0.787867	2331	(+)	NNNNWTATGCAAATNTNNN	TTCTATATCCATATTTAGA
M00136	V$OCT1_02	0.77098	2331	(+)	NNGAATATKCANNNN	TTCTATATCCATATT
M00136	V$OCT1_02	0.796697	2334	(-)	NNGAATATKCANNNN	TATATCCATATTTAG
M00136	V$OCT1_02	0.783433	2337	(+)	NNGAATATKCANNNN	ATCCATATTTAGAAA
M00135	V$OCT1_01	0.735025	2338	(-)	NNNNWTATGCAAATNTNNN	TCCATATTTAGAAAATGCA
M00162	V$OCT1_06	0.809375	2340	(+)	CWNAWTKWSATRYN	CATATTTAGAAAAT
M00162	V$OCT1_06	0.800391	2340	(-)	CWNAWTKWSATRYN	CATATTTAGAAAAT
M00136	V$OCT1_02	0.850839	2346	(+)	NNGAATATKCANNNN	TAGAAAATGCAGGAT
M00137	V$OCT1_03	0.858159	2396	(-)	NNNRTAATNANNN	TTTTGTTTATGCT
M00138	V$OCT1_04	0.782518	2396	(+)	NNNNNNNWATGCAAATNNNWNNW	TTTTGTTTATGC-TACACCCTAAA
M00135	V$OCT1_01	0.759252	2398	(+)	NNNNWTATGCAAATNTNNN	TTGTTTATGCTACACCCTA
M00162	V$OCT1_06	0.800781	2431	(-)	CWNAWTKWSATRYN	GAAATCTCTGACAG
M00138	V$OCT1_04	0.781472	2443	(+)	NNNNNNNWATGCAAATNNNWNNW	AGTAAGTTACAAAAA-TAAATCCA
M00162	V$OCT1_06	0.812109	2452	(+)	CWNAWTKWSATRYN	CAAAAATAAATCCA
M00162	V$OCT1_06	0.870312	2458	(-)	CWNAWTKWSATRYN	TAAATCCAAGTCCT
M00138	V$OCT1_04	0.783563	2503	(+)	NNNNNNNWATGCAAATNNNWNNW	TTGTTCTTTTACAAATGAAATGG
M00162	V$OCT1_06	0.816406	2514	(-)	CWNAWTKWSATRYN	CAAATGAAATGGGT
M00248	V$OCT1_07	0.792453	2605	(-)	TNTATGNTAATT	AACAAACATAAA
M00162	V$OCT1_06	0.847656	2607	(+)	CWNAWTKWSATRYN	CAAACATAAATTTT
M00138	V$OCT1_04	0.793392	2628	(+)	NNNNNNNWATGCAAATNNNWNNW	CAGTTAAAATGAAGATTGA-GATA
M00136	V$OCT1_02	0.798863	2633	(-)	NNGAATATKCANNNN	AAAATGAAGATTGAG
M00138	V$OCT1_04	0.795483	2634	(+)	NNNNNNNWATGCAAATNNNWNNW	AAATGAAGATTGAGA-TAAATTTT
M00162	V$OCT1_06	0.860547	2639	(+)	CWNAWTKWSATRYN	AAGATTGAGATAAA
M00138	V$OCT1_04	0.783354	2647	(-)	NNNNNNNWATGCAAATNNNWNNW	GATAAATTTTTGTATGAT-TTAAT
M00135	V$OCT1_01	0.735406	2649	(-)	NNNNWTATGCAAATNTNNN	TAAATTTTTGTATGATTTA
M00136	V$OCT1_02	0.776665	2680	(+)	NNGAATATKCANNNN	AATAAAATCCAGACA
M00162	V$OCT1_06	0.834766	2697	(+)	CWNAWTKWSATRYN	CAACATTTAATTAG
M00162	V$OCT1_06	0.902344	2698	(-)	CWNAWTKWSATRYN	AACATTTAATTAGG

M00138	V$OCT1_04	0.79632	2699	(-)	NNNNNNNNWATGCAAATNNNWNNW	ACATTTAATTAG-GAAAAGTATAC
M00137	V$OCT1_03	0.864085	2700	(+)	NNNRTAATNANNN	CATTTAATTAGGA
M00137	V$OCT1_03	0.894903	2701	(-)	NNNRTAATNANNN	ATTTAATTAGGAA
M00138	V$OCT1_04	0.818904	2701	(+)	NNNNNNNNWATGCAAATNNNWNNW	ATTTAATTAGGAAAAGTATA-CAT
M00137	V$OCT1_03	0.88068	2737	(+)	NNNRTAATNANNN	TGTATAATGGTTT
M00248	V$OCT1_07	0.839093	2764	(-)	TNTATGNTAATT	AACTACCATGTA
M00162	V$OCT1_06	0.842187	2767	(+)	CWNAWTKWSATRYN	TACCATGTAATATA
M00162	V$OCT1_06	0.806641	2768	(-)	CWNAWTKWSATRYN	ACCATGTAATATAA
M00138	V$OCT1_04	0.810749	2769	(-)	NNNNNNNNWATGCAAATNNNWNNW	CCATGTAATATAAATT-GTTCAGA
M00162	V$OCT1_06	0.813672	2790	(-)	CWNAWTKWSATRYN	GAAAACTAAATCAT
M00138	V$OCT1_04	0.792137	2800	(-)	NNNNNNNNWATGCAAATNNNWNNW	TCATTTCTTTATCATTCTG-GATT
M00137	V$OCT1_03	0.868827	2802	(-)	NNNRTAATNANNN	ATTTCTTTATCAT
M00162	V$OCT1_06	0.813281	2852	(+)	CWNAWTKWSATRYN	GTTTTTTTGAAGTC
M00135	V$OCT1_01	0.752194	2862	(-)	NNNNWTATGCAAATNTNNN	AGTCTTTTTACATAGTTTT
M00162	V$OCT1_06	0.834375	2912	(+)	CWNAWTKWSATRYN	AATAATTTACTTTT
M00162	V$OCT1_06	0.847266	2913	(+)	CWNAWTKWSATRYN	ATAATTTACTTTTT
M00195	V$OCT1_Q6	0.802675	2913	(-)	NNNNATGCAAATNAN	ATAATTTACTTTTTA
M00248	V$OCT1_07	0.793089	2915	(-)	TNTATGNTAATT	AATTTACTTTTT
M00138	V$OCT1_04	0.794437	2916	(-)	NNNNNNNNWATGCAAATNNNWNNW	ATTTACTTTTTAAATTTTT-GTCA
M00162	V$OCT1_06	0.821484	2920	(+)	CWNAWTKWSATRYN	ACTTTTTAAATTTT
M00248	V$OCT1_07	0.793301	2920	(+)	TNTATGNTAATT	ACTTTTTAAATT
M00138	V$OCT1_04	0.798829	2922	(-)	NNNNNNNNWATGCAAATNNNWNNW	TTTTTAAATTTTTGTCAA-TAAAA
M00138	V$OCT1_04	0.786491	2925	(+)	NNNNNNNNWATGCAAATNNNWNNW	TTAAATTTTTGTCAATAAAA-TAT
M00162	V$OCT1_06	0.847656	2935	(+)	CWNAWTKWSATRYN	GTCAATAAAATATA
M00162	V$OCT1_06	0.873437	2980	(+)	CWNAWTKWSATRYN	CAGATTTAGCTATT
M00138	V$OCT1_04	0.852781	2983	(+)	NNNNNNNNWATGCAAATNNNWNNW	ATTTAGC-TATTAAAATACAAACT
M00138	V$OCT1_04	0.793392	2984	(-)	NNNNNNNNWATGCAAATNNNWNNW	TTTAGC-TATTAAAATACAAACTT
M00138	V$OCT1_04	0.785655	2989	(+)	NNNNNNNNWATGCAAATNNNWNNW	CTATTAAAATACAAACTTTTCTT
M00248	V$OCT1_07	0.836549	2990	(-)	TNTATGNTAATT	TATTAAAATACA
M00162	V$OCT1_06	0.932422	3018	(+)	CWNAWTKWSATRYN	CAACATTAAATTCA
M00162	V$OCT1_06	0.924609	3019	(-)	CWNAWTKWSATRYN	AACATTAAATTCAT
M00138	V$OCT1_04	0.788373	3032	(-)	NNNNNNNNWATGCAAATNNNWNNW	TAGTATTA-TATGTATATTGTGAT
M00162	V$OCT1_06	0.801562	3032	(+)	CWNAWTKWSATRYN	TAGTATTATATGTA

M0016 2	V$OCT1_06	0.826562	3033	(-)	CWNAWTKWSATRYN	AGTATTATATGTAT
M0013 5	V$OCT1_01	0.735597	3034	(-)	NNNNWTATGCAAATNTNNN	GTATTATATGTATATTGTG
M0013 5	V$OCT1_01	0.755246	3035	(+)	NNNNWTATGCAAATNTNNN	TATTATATGTATATTGTGA
M0013 6	V$OCT1_02	0.818896	3038	(-)	NNGAATATKCANNNN	TATATGTATATTGTG
M0024 8	V$OCT1_07	0.866441	3038	(+)	TNTATGNTAATT	TATATGTATATT
M0013 8	V$OCT1_04	0.793392	3039	(+)	NNNNNNNWATGCAAATNNNW NNW	ATATGTATATTGTGATCTTAT-TA
M0016 2	V$OCT1_06	0.817187	3044	(+)	CWNAWTKWSATRYN	TATATTGTGATCTT
M0013 7	V$OCT1_03	0.916239	3053	(-)	NNNRTAATNANNN	ATCTTATTATTAT
M0013 7	V$OCT1_03	0.873568	3056	(-)	NNNRTAATNANNN	TTATTATTATTGT
M0016 2	V$OCT1_06	0.805859	3061	(+)	CWNAWTKWSATRYN	ATTATTGTACTTTG
M0024 8	V$OCT1_07	0.815137	3061	(+)	TNTATGNTAATT	ATTATTGTACTT
M0016 2	V$OCT1_06	0.800391	3067	(+)	CWNAWTKWSATRYN	GTACTTTGATTGCT
M0016 2	V$OCT1_06	0.809375	3092	(+)	CWNAWTKWSATRYN	TTTATTGTTATATA
M0013 8	V$OCT1_04	0.807821	3111	(-)	NNNNNNNWATGCAAATNNNW NNW	TGCTTTGTTTTTCACAGAAA-TAT
M0013 6	V$OCT1_02	0.782079	3126	(+)	NNGAATATKCANNNN	AGAAATATCCTCTCA
M0016 2	V$OCT1_06	0.803125	3150	(+)	CWNAWTKWSATRYN	AAGTCTTTCAAGTG
M0016 2	V$OCT1_06	0.855469	3270	(+)	CWNAWTKWSATRYN	CAGTTTCACATTCA
M0016 2	V$OCT1_06	0.839453	3271	(-)	CWNAWTKWSATRYN	AGTTTCACATTCAC
M0013 6	V$OCT1_02	0.770168	3273	(+)	NNGAATATKCANNNN	TTTCACATTCACTAA
M0013 7	V$OCT1_03	0.840379	3288	(+)	NNNRTAATNANNN	GGTATAATATCTT
M0013 6	V$OCT1_02	0.857336	3314	(-)	NNGAATATKCANNNN	GAACTGAATATCGAT
M0013 8	V$OCT1_04	0.821623	3359	(-)	NNNNNNNWATGCAAATNNNW NNW	CACATAGACTTACATAAA-TATTT
M0013 5	V$OCT1_01	0.802556	3361	(-)	NNNNWTATGCAAATNTNNN	CATAGACTTACATAAATAT
M0013 8	V$OCT1_04	0.782518	3362	(+)	NNNNNNNWATGCAAATNNNW NNW	ATAGACTTACATAAA-TATTTATG
M0013 8	V$OCT1_04	0.807194	3363	(-)	NNNNNNNWATGCAAATNNNW NNW	TAGACTTACATAAA-TATTTATGA
M0016 2	V$OCT1_06	0.800391	3364	(-)	CWNAWTKWSATRYN	AGACTTACATAAAT
M0016 2	V$OCT1_06	0.852734	3367	(+)	CWNAWTKWSATRYN	CTTACATAAATATT
M0013 6	V$OCT1_02	0.84732	3369	(-)	NNGAATATKCANNNN	TACATAAATATTTAT
M0013 7	V$OCT1_03	0.860529	3369	(+)	NNNRTAATNANNN	TACATAAATATTT
M0013 8	V$OCT1_04	0.821832	3369	(-)	NNNNNNNWATGCAAATNNNW NNW	TACATAAATATTTATGAT-TAATT
M0013 8	V$OCT1_04	0.814513	3370	(+)	NNNNNNNWATGCAAATNNNW NNW	ACATAAATATTTATGAT-TAATTA
M0013 6	V$OCT1_02	0.834326	3372	(+)	NNGAATATKCANNNN	ATAAATATTTATGAT
M0013 8	V$OCT1_04	0.780427	3373	(-)	NNNNNNNWATGCAAATNNNW NNW	TAAATATTTATGAT-TAATTAATA

M00137	V$OCT1_03	0.868036	3374	(-)	NNNRTAATNANNN	AAATATTTATGAT
M00138	V$OCT1_04	0.812631	3374	(+)	NNNNNNNNWATGCAAATNNNWNNW	AAATATTTATGATTAATTAA-TAC
M00137	V$OCT1_03	0.849862	3380	(-)	NNNRTAATNANNN	TTATGATTAATTA
M00162	V$OCT1_06	0.808203	3384	(+)	CWNAWTKWSATRYN	GATTAATTAATACC
M00137	V$OCT1_03	0.930067	3395	(-)	NNNRTAATNANNN	ACCTTATTATTCA
M00135	V$OCT1_01	0.754101	3397	(+)	NNNNWTATGCAAATNTNNN	CTTATTATTCAAGTGCAAT
M00136	V$OCT1_02	0.851651	3397	(+)	NNGAATATKCANNNN	CTTATTATTCAAGTG
M00136	V$OCT1_02	0.777477	3420	(+)	NNGAATATKCANNNN	AAGGATATGTTAGCA
M00138	V$OCT1_04	0.808867	3421	(-)	NNNNNNNNWATGCAAATNNNWNNW	AGGATATGTTAGCATGAT-TTATT
M00135	V$OCT1_01	0.758489	3423	(-)	NNNNWTATGCAAATNTNNN	GATATGTTAGCATGATTTA
M00138	V$OCT1_04	0.792137	3433	(+)	NNNNNNNNWATGCAAATNNNWNNW	CATGAT-TTATTTAAATTATTAT
M00138	V$OCT1_04	0.875575	3435	(-)	NNNNNNNNWATGCAAATNNNWNNW	TGAT-TTATTTAAATTATTATTG
M00248	V$OCT1_07	0.831673	3438	(+)	TNTATGNTAATT	TTTATTTTAAAT
M00162	V$OCT1_06	0.847266	3440	(-)	CWNAWTKWSATRYN	TATTTTAAATTATT
M00137	V$OCT1_03	0.88147	3446	(-)	NNNRTAATNANNN	AAATTATTATTGC
M00138	V$OCT1_04	0.806357	3469	(-)	NNNNNNNNWATGCAAATNNNWNNW	CTCTTTTACTT-GCATTCAAACAT
M00162	V$OCT1_06	0.855078	3469	(+)	CWNAWTKWSATRYN	CTCTTTTACTTGCA
M00135	V$OCT1_01	0.731209	3471	(-)	NNNNWTATGCAAATNTNNN	CTTTTACTTGCATTCAAAC
M00162	V$OCT1_06	0.847656	3473	(+)	CWNAWTKWSATRYN	TTTACTTGCATTCA
M00195	V$OCT1_Q6	0.796124	3473	(-)	NNNNATGCAAATNAN	TTTACTTGCATTCAA
M00138	V$OCT1_04	0.789628	3474	(+)	NNNNNNNNWATGCAAATNNNWNNW	TTACTTGCATTCAAACATA-TAAT
M00162	V$OCT1_06	0.808594	3486	(+)	CWNAWTKWSATRYN	AAACATATAATTTG
M00162	V$OCT1_06	0.819922	3487	(-)	CWNAWTKWSATRYN	AACATATAATTTGC
M00138	V$OCT1_04	0.804266	3488	(-)	NNNNNNNNWATGCAAATNNNWNNW	ACATATAATTTGCAATTT-GCTCT
M00135	V$OCT1_01	0.781	3490	(-)	NNNNWTATGCAAATNTNNN	ATATAATTTGCAATTTGCT
M00135	V$OCT1_01	0.745326	3491	(+)	NNNNWTATGCAAATNTNNN	TATAATTTGCAATTTGCTC
M00136	V$OCT1_02	0.787764	3491	(+)	NNGAATATKCANNNN	TATAATTTGCAATTT
M00161	V$OCT1_05	0.897393	3492	(+)	MKNATTTGCATAYY	ATAATTTGCAATTT
M00162	V$OCT1_06	0.826562	3492	(+)	CWNAWTKWSATRYN	ATAATTTGCAATTT
M00195	V$OCT1_Q6	0.8881	3492	(-)	NNNNATGCAAATNAN	ATAATTTGCAATTTG
M00248	V$OCT1_07	0.799873	3494	(-)	TNTATGNTAATT	AATTTGCAATTT
M00135	V$OCT1_01	0.735597	3508	(-)	NNNNWTATGCAAATNTNNN	TCTCATTTTACACACTGAA
M00136	V$OCT1_02	0.781808	3519	(-)	NNGAATATKCANNNN	ACACTGAATCTTCCA

M00162	V$OCT1_06	0.801562	3592	(+)	CWNAWTKWSATRYN	CTTATTTTTTTATT
M00138	V$OCT1_04	0.789419	3599	(-)	NNNNNNNWATGCAAATNNNWNNW	TTTTATTCTTT-GCAGAATGAACC
M00135	V$OCT1_01	0.771652	3601	(-)	NNNNWTATGCAAATNTNNN	TTATTCTTTGCAGAATGAA
M00195	V$OCT1_Q6	0.804858	3603	(-)	NNNNATGCAAATNAN	ATTCTTTGCAGAATG
M00162	V$OCT1_06	0.813672	3663	(+)	CWNAWTKWSATRYN	CTCTCTTTCAGTTA
M00162	V$OCT1_06	0.824219	3685	(+)	CWNAWTKWSATRYN	GACTGTTGCATGTC
M00162	V$OCT1_06	0.868359	3701	(+)	CWNAWTKWSATRYN	CCTCATTTCATTTA
M00162	V$OCT1_06	0.855469	3702	(-)	CWNAWTKWSATRYN	CTCATTTCATTTAT
M00138	V$OCT1_04	0.811167	3707	(-)	NNNNNNNWATGCAAATNNNWNNW	TTCATTTATTTCCTTCAATTGT
M00162	V$OCT1_06	0.808594	3757	(+)	CWNAWTKWSATRYN	CTCTCTTAATTGTC
M00162	V$OCT1_06	0.804297	3761	(+)	CWNAWTKWSATRYN	CTTAATTGTCTGTT
M00248	V$OCT1_07	0.836549	3769	(+)	TNTATGNTAATT	TCTGTTTTAATT
M00137	V$OCT1_03	0.852627	3772	(+)	NNNRTAATNANNN	GTTTTAATTAAAT
M00162	V$OCT1_06	0.855469	3774	(+)	CWNAWTKWSATRYN	TTTAATTAAATTCC
M00248	V$OCT1_07	0.807717	3774	(+)	TNTATGNTAATT	TTTAATTAAATT
M00138	V$OCT1_04	0.781472	3775	(+)	NNNNNNNWATGCAAATNNNWNNW	TTAATTAAATTCC-TATTCAATCC
M00162	V$OCT1_06	0.814062	3775	(-)	CWNAWTKWSATRYN	TTAATTAAATTCCT
M00136	V$OCT1_02	0.807526	3783	(+)	NNGAATATKCANNNN	ATTCCTATTCAATCC
M00138	V$OCT1_04	0.806775	3806	(-)	NNNNNNNWATGCAAATNNNWNNW	ACATTTGA-TAAGTATGTTACTAA
M00137	V$OCT1_03	0.850257	3810	(+)	NNNRTAATNANNN	TTGATAAGTATGT
M00135	V$OCT1_01	0.73884	3813	(+)	NNNNWTATGCAAATNTNNN	ATAAGTATGTTACTAAACT
M00137	V$OCT1_03	0.870012	3839	(+)	NNNRTAATNANNN	CTGGTAAAGAAGG
M00162	V$OCT1_06	0.916797	3867	(+)	CWNAWTKWSATRYN	CTCCTTTAGATTTT
M00162	V$OCT1_06	0.844922	3875	(-)	CWNAWTKWSATRYN	GATTTTAAAATTAG
M00162	V$OCT1_06	0.832812	3880	(+)	CWNAWTKWSATRYN	TAAAATTAGATCCT
M00162	V$OCT1_06	0.862891	3899	(-)	CWNAWTKWSATRYN	TACAACTAATTCTC
M00162	V$OCT1_06	0.801562	3904	(+)	CWNAWTKWSATRYN	CTAATTCTCTTATT
M00195	V$OCT1_Q6	0.790666	3904	(-)	NNNNATGCAAATNAN	CTAATTCTCTTATTT
M00136	V$OCT1_02	0.782079	3922	(-)	NNGAATATKCANNNN	CTAAGAAATATTCTG
M00136	V$OCT1_02	0.839199	3925	(+)	NNGAATATKCANNNN	AGAAATATTCTGGTT
M00138	V$OCT1_04	0.781263	3961	(+)	NNNNNNNWATGCAAATNNNWNNW	TTGTTCAAATGGAAAACAT-ACTC
M00162	V$OCT1_06	0.808594	3966	(-)	CWNAWTKWSATRYN	CAAATGGAAAACAT
M00136	V$OCT1_02	0.796156	3989	(-)	NNGAATATKCANNNN	GGGGTGCATTTTTCC

M0013 7	V$OCT1_03	0.86369	4001	(-)	NNNRTAATNANNN	TCCTTTTTATAAT
M0016 2	V$OCT1_06	0.832031	4003	(+)	CWNAWTKWSATRYN	CTTTTTATAATTCA
M0013 8	V$OCT1_04	0.79381	4004	(+)	NNNNNNNNWATGCAAATNNNW NNW	TTTTTATAATTCACAATTTAA-TA
M0013 6	V$OCT1_02	0.77098	4006	(+)	NNGAATATKCANNNN	TTTATAATTCACAAT
M0016 2	V$OCT1_06	0.937891	4015	(+)	CWNAWTKWSATRYN	CACAATTTAATATC
M0016 2	V$OCT1_06	0.808594	4016	(-)	CWNAWTKWSATRYN	ACAATTTAATATCT
M0016 2	V$OCT1_06	0.813672	4103	(-)	CWNAWTKWSATRYN	GACAGCCAAATTCT
M0016 2	V$OCT1_06	0.800391	4112	(+)	CWNAWTKWSATRYN	ATTCTTGAAAAATC
M0013 6	V$OCT1_02	0.772604	4113	(-)	NNGAATATKCANNNN	TTCTTGAAAAATCAA
M0013 8	V$OCT1_04	0.83166	4116	(-)	NNNNNNNNWATGCAAATNNNW NNW	TTGAAAAATCAACATAATTTT TG
M0013 5	V$OCT1_01	0.77871	4118	(-)	NNNNWTATGCAAATNTNNN	GAAAAATCAACATAATTTT
M0016 2	V$OCT1_06	0.831641	4120	(-)	CWNAWTKWSATRYN	AAAATCAACATAAT
M0024 8	V$OCT1_07	0.871105	4122	(-)	TNTATGNTAATT	AATCAACATAAT
M0013 8	V$OCT1_04	0.782936	4131	(-)	NNNNNNNNWATGCAAATNNNW NNW	AATTTTTGTTT-GCAAATGTTTTT
M0013 8	V$OCT1_04	0.805521	4132	(+)	NNNNNNNNWATGCAAATNNNW NNW	ATTTTTGTTT-GCAAATGTTTTTT
M0013 5	V$OCT1_01	0.814384	4134	(+)	NNNNWTATGCAAATNTNNN	TTTTGTTTGCAAATGTTTT
M0016 2	V$OCT1_06	0.834375	4137	(-)	CWNAWTKWSATRYN	TGTTTGCAAATGTT
M0013 7	V$OCT1_03	0.8629	4161	(-)	NNNRTAATNANNN	ACTTGATTAAACC
M0016 2	V$OCT1_06	0.847266	4173	(+)	CWNAWTKWSATRYN	CTTTACTTCATTCA
M0013 6	V$OCT1_02	0.851922	4202	(-)	NNGAATATKCANNNN	AAAAAGAATATTGAA
M0013 8	V$OCT1_04	0.840025	4203	(+)	NNNNNNNNWATGCAAATNNNW NNW	AAAAGAA-TATTGAAATCAGATAA
M0013 5	V$OCT1_01	0.752766	4205	(+)	NNNNWTATGCAAATNTNNN	AAGAATATTGAAATCAGAT
M0013 6	V$OCT1_02	0.842447	4205	(+)	NNGAATATKCANNNN	AAGAATATTGAAATC
M0019 5	V$OCT1_Q6	0.84143	4207	(+)	NNNNATGCAAATNAN	GAATATTGAAATCAG
M0016 1	V$OCT1_05	0.890752	4208	(-)	MKNATTTGCATAYY	AATATTGAAATCAG
M0016 2	V$OCT1_06	0.866016	4208	(-)	CWNAWTKWSATRYN	AATATTGAAATCAG
M0024 8	V$OCT1_07	0.791181	4208	(+)	TNTATGNTAATT	AATATTGAAATC
M0016 2	V$OCT1_06	0.813672	4214	(-)	CWNAWTKWSATRYN	GAAATCAGATAAAC
M0013 5	V$OCT1_01	0.762304	4218	(+)	NNNNWTATGCAAATNTNNN	TCAGATAAACAAATATTAA
M0013 7	V$OCT1_03	0.852232	4219	(+)	NNNRTAATNANNN	CAGATAAACAAAT
M0013 6	V$OCT1_02	0.78262	4223	(-)	NNGAATATKCANNNN	TAAACAAATATTAAA
M0013 8	V$OCT1_04	0.797365	4224	(+)	NNNNNNNNWATGCAAATNNNW NNW	AAACAAATATTAAAAA-GCTGCTT
M0016 2	V$OCT1_06	0.869922	4229	(-)	CWNAWTKWSATRYN	AATATTAAAAAGCT

M00138	V$OCT1_04	0.7867	4236	(+)	NNNNNNNNWATGCAAATNNNWNNW	AAAA-GCTGCTTTAAATTTTAATT
M00162	V$OCT1_06	0.903906	4241	(+)	CWNAWTKWSATRYN	CTGCTTTAAATTTT
M00195	V$OCT1_Q6	0.802129	4241	(-)	NNNNATGCAAATNAN	CTGCTTTAAATTTTA
M00138	V$OCT1_04	0.819532	4243	(-)	NNNNNNNNWATGCAAATNNNWNNW	GCTTTAAATTTTAATTT-GTACCT
M00162	V$OCT1_06	0.847656	4247	(+)	CWNAWTKWSATRYN	TAAATTTTAATTTG
M00162	V$OCT1_06	0.873828	4247	(-)	CWNAWTKWSATRYN	TAAATTTTAATTTG
M00248	V$OCT1_07	0.875556	4247	(+)	TNTATGNTAATT	TAAATTTTAATT
M00138	V$OCT1_04	0.788373	4249	(-)	NNNNNNNNWATGCAAATNNNWNNW	AATTTTAATTTGTACCTTT-GCAA
M00195	V$OCT1_Q6	0.802129	4253	(-)	NNNNATGCAAATNAN	TTAATTTGTACCTTT
M00135	V$OCT1_01	0.751049	4259	(-)	NNNNWTATGCAAATNTNNN	TGTACCTTTGCAAAACACT
M00195	V$OCT1_Q6	0.809771	4262	(+)	NNNNATGCAAATNAN	ACCTTTGCAAAACAC
M00162	V$OCT1_06	0.868359	4278	(+)	CWNAWTKWSATRYN	CATGTTTTCATTTG
M00138	V$OCT1_04	0.898578	4280	(-)	NNNNNNNNWATGCAAATNNNWNNW	TGTTTTCATTTGCATAAC-CTATG
M00138	V$OCT1_04	0.7867	4281	(+)	NNNNNNNNWATGCAAATNNNWNNW	GTTTTCATTTGCATAAC-CTATGT
M00135	V$OCT1_01	0.876574	4282	(-)	NNNNWTATGCAAATNTNNN	TTTTCATTTGCATAACCTA
M00161	V$OCT1_05	0.896895	4284	(+)	MKNATTTGCATAYY	TTCATTTGCATAAC
M00195	V$OCT1_Q6	0.88619	4284	(-)	NNNNATGCAAATNAN	TTCATTTGCATAACC
M00136	V$OCT1_02	0.822956	4286	(-)	NNGAATATKCANNNN	CATTTGCATAACCTA
M00248	V$OCT1_07	0.840153	4286	(-)	TNTATGNTAATT	CATTTGCATAAC
M00138	V$OCT1_04	0.804475	4292	(+)	NNNNNNNNWATGCAAATNNNWNNW	CATAAC-CTATGTAGAATTTTACA
M00162	V$OCT1_06	0.828906	4305	(+)	CWNAWTKWSATRYN	GAATTTTACAATTT
M00162	V$OCT1_06	0.839453	4306	(-)	CWNAWTKWSATRYN	AATTTTACAATTTC
M00138	V$OCT1_04	0.875784	4308	(-)	NNNNNNNNWATGCAAATNNNWNNW	TTTTACAATTTCCATGTTCAT-TA
M00135	V$OCT1_01	0.813621	4310	(-)	NNNNWTATGCAAATNTNNN	TTACAATTTCCATGTTCAT
M00161	V$OCT1_05	0.880624	4312	(+)	MKNATTTGCATAYY	ACAATTTCCATGTT
M00162	V$OCT1_06	0.812109	4312	(+)	CWNAWTKWSATRYN	ACAATTTCCATGTT
M00195	V$OCT1_Q6	0.835972	4312	(-)	NNNNATGCAAATNAN	ACAATTTCCATGTTC
M00136	V$OCT1_02	0.776394	4314	(-)	NNGAATATKCANNNN	AATTTCCATGTTCAT
M00248	V$OCT1_07	0.893788	4314	(-)	TNTATGNTAATT	AATTTCCATGTT
M00248	V$OCT1_07	0.841213	4318	(+)	TNTATGNTAATT	TCCATGTTCATT
M00138	V$OCT1_04	0.78921	4319	(+)	NNNNNNNNWATGCAAATNNNWNNW	CCATGTTCATTATAATGGAATGT
M00137	V$OCT1_03	0.873173	4322	(-)	NNNRTAATNANNN	TGTTCATTATAAT
M00137	V$OCT1_03	0.866061	4327	(+)	NNNRTAATNANNN	ATTATAATGGAAT

M00162	V$OCT1_06	0.876172	4329	(+)	CWNAWTKWSATRYN	TATAATGGAATGTT
M00136	V$OCT1_02	0.834326	4331	(-)	NNGAATATKCANNNN	TAATGGAATGTTCTT
M00162	V$OCT1_06	0.855078	4363	(+)	CWNAWTKWSATRYN	CACTATGTGTTGTA
M00138	V$OCT1_04	0.862401	4370	(-)	NNNNNNNWATGCAAATNNNWNNW	TGTTGTATTTTCAA-TAATTTTGT
M00162	V$OCT1_06	0.808203	4375	(-)	CWNAWTKWSATRYN	TATTTTCAATAATT
M00248	V$OCT1_07	0.791181	4376	(-)	TNTATGNTAATT	ATTTTCAATAAT
M00137	V$OCT1_03	0.847096	4380	(+)	NNNRTAATNANNN	TCAATAATTTTGT
M00162	V$OCT1_06	0.829297	4391	(+)	CWNAWTKWSATRYN	GTGACTGACTTGTC
M00162	V$OCT1_06	0.855078	4397	(-)	CWNAWTKWSATRYN	GACTTGTCAAGTAG
M00162	V$OCT1_06	0.847656	4415	(+)	CWNAWTKWSATRYN	CAAACAGAAATTTT
M00162	V$OCT1_06	0.826562	4421	(+)	CWNAWTKWSATRYN	GAAATTTTACTTCA
M00162	V$OCT1_06	0.847656	4441	(+)	CWNAWTKWSATRYN	CTGTATAAAATATT
M00162	V$OCT1_06	0.855078	4442	(-)	CWNAWTKWSATRYN	TGTATAAAATATTT
M00162	V$OCT1_06	0.834375	4448	(+)	CWNAWTKWSATRYN	AAATATTTGATAGG
M00138	V$OCT1_04	0.789	4479	(-)	NNNNNNNWATGCAAATNNNWNNW	AATGATTTTTAACATAAC-CTTCC
M00135	V$OCT1_01	0.751622	4481	(-)	NNNNWTATGCAAATNTNNN	TGATTTTTAACATAACCTT
M00162	V$OCT1_06	0.816016	4534	(-)	CWNAWTKWSATRYN	GACAGGTAAGAAAT
M00162	V$OCT1_06	0.842187	4538	(-)	CWNAWTKWSATRYN	GGTAAGAAATTGTC
M00135	V$OCT1_01	0.752766	4576	(+)	NNNNWTATGCAAATNTNNN	AAATGGAAGCAAATTTTGT
M00162	V$OCT1_06	0.860547	4580	(-)	CWNAWTKWSATRYN	GGAAGCAAATTTTG
M00162	V$OCT1_06	0.839453	4606	(+)	CWNAWTKWSATRYN	AGTATTGTGGTGTT
M00137	V$OCT1_03	0.84354	4636	(-)	NNNRTAATNANNN	GCCTTTTTATGGA
M00138	V$OCT1_04	0.831451	4636	(+)	NNNNNNNWATGCAAATNNNWNNW	GCCTTTTTATGGAAA-TAAACACT
M00135	V$OCT1_01	0.816101	4638	(+)	NNNNWTATGCAAATNTNNN	CTTTTTATGGAAATAAACA
M00248	V$OCT1_07	0.869197	4641	(+)	TNTATGNTAATT	TTTATGGAAATA
M00136	V$OCT1_02	0.806443	4687	(+)	NNGAATATKCANNNN	AAGAGAATTCAGAAT
M00136	V$OCT1_02	0.785598	4693	(-)	NNGAATATKCANNNN	ATTCAGAATAATGAC
M00138	V$OCT1_04	0.79611	4695	(+)	NNNNNNNWATGCAAATNNNWNNW	TCAGAA-TAATGACTAAAACAAAA
M00137	V$OCT1_03	0.899249	4697	(+)	NNNRTAATNANNN	AGAATAATGACTA
M00162	V$OCT1_06	0.88125	4713	(+)	CWNAWTKWSATRYN	CAAAATGAAAGATA
M00162	V$OCT1_06	0.834375	4714	(-)	CWNAWTKWSATRYN	AAAATGAAAGATAA
M00162	V$OCT1_06	0.803906	4729	(-)	CWNAWTKWSATRYN	AACAACAAAATTTA
M00138	V$OCT1_04	0.832497	4748	(-)	NNNNNNNWATGCAAATNNNWNNW	ACAAAATTTTAAAA-TATTTTTAA

M00162	V$OCT1_06	0.839453	4750	(+)	CWNAWTKWSATRYN	AAAATTTTAAAATA
M00162	V$OCT1_06	0.800391	4751	(-)	CWNAWTKWSATRYN	AAATTTTAAAATAT
M00162	V$OCT1_06	0.834375	4752	(+)	CWNAWTKWSATRYN	AATTTTAAAATATT
M00162	V$OCT1_06	0.839453	4752	(-)	CWNAWTKWSATRYN	AATTTTAAAATATT
M00138	V$OCT1_04	0.780427	4758	(-)	NNNNNNNWATGCAAATNNNWNNW	AAAATATTTTTAAGTG-CACATTT
M00136	V$OCT1_02	0.796697	4768	(-)	NNGAATATKCANNNN	TAAGTGCACATTTAC
M00138	V$OCT1_04	0.81744	4770	(-)	NNNNNNNWATGCAAATNNNWNNW	AGTGCACATTTACAT-ACTGACAA
M00135	V$OCT1_01	0.878863	4772	(-)	NNNNWTATGCAAATNTNNN	TGCACATTTACATACTGAC
M00162	V$OCT1_06	0.971484	4774	(+)	CWNAWTKWSATRYN	CACATTTACATACT
M00162	V$OCT1_06	0.821484	4774	(-)	CWNAWTKWSATRYN	CACATTTACATACT
M00195	V$OCT1_Q6	0.835153	4774	(-)	NNNNATGCAAATNAN	CACATTTACATACTG
M00248	V$OCT1_07	0.852025	4776	(-)	TNTATGNTAATT	CATTTACATACT
M00162	V$OCT1_06	0.812109	4782	(+)	CWNAWTKWSATRYN	CATACTGACAACTT
M00162	V$OCT1_06	0.826562	4791	(-)	CWNAWTKWSATRYN	AACTTTAAAGAAAG
M00162	V$OCT1_06	0.808203	4815	(+)	CWNAWTKWSATRYN	AGACACTTAATGCA
M00162	V$OCT1_06	0.800781	4821	(-)	CWNAWTKWSATRYN	TTAATGCAAGAAAT
M00162	V$OCT1_06	0.826562	4866	(-)	CWNAWTKWSATRYN	AAAAACACATTCAT
M00136	V$OCT1_02	0.77477	4868	(+)	NNGAATATKCANNNN	AAACACATTCATCTT
M00162	V$OCT1_06	0.903906	4877	(+)	CWNAWTKWSATRYN	CATCTTTTAATTTG
M00138	V$OCT1_04	0.880176	4879	(-)	NNNNNNNWATGCAAATNNNWNNW	TCTTTTAATTTGCAGTAG-CAATG
M00135	V$OCT1_01	0.763831	4881	(-)	NNNNWTATGCAAATNTNNN	TTTTAATTTGCAGTAGCAA
M00195	V$OCT1_Q6	0.793668	4883	(-)	NNNNATGCAAATNAN	TTAATTTGCAGTAGC
M00162	V$OCT1_06	0.821484	4895	(+)	CWNAWTKWSATRYN	AGCAATGTAAGGTA
M00195	V$OCT1_Q6	0.792849	4895	(+)	NNNNATGCAAATNAN	AGCAATGTAAGGTAT
M00162	V$OCT1_06	0.808594	4896	(-)	CWNAWTKWSATRYN	GCAATGTAAGGTAT
M00138	V$OCT1_04	0.785027	4910	(+)	NNNNNNNWATGCAAATNNNWNNW	GTTTTCTCATAGA-TATCCATTCA
M00138	V$OCT1_04	0.804266	4915	(-)	NNNNNNNWATGCAAATNNNWNNW	CTCATAGA-TATCCATTCATTTGT
M00136	V$OCT1_02	0.808609	4918	(+)	NNGAATATKCANNNN	ATAGATATCCATTCA
M00138	V$OCT1_04	0.78189	4925	(-)	NNNNNNNWATGCAAATNNNWNNW	TCCATTCATTT-GTGTCCCTCTTT
M00137	V$OCT1_03	0.88384	4940	(-)	NNNRTAATNANNN	CCCTCTTTACTAA
M00162	V$OCT1_06	0.855469	5011	(+)	CWNAWTKWSATRYN	ATCAATGATATTTA
M00138	V$OCT1_04	0.860728	5012	(+)	NNNNNNNWATGCAAATNNNWNNW	TCAATGATATTTAAATTATTT-TA
M00138	V$OCT1_04	0.832915	5013	(-)	NNNNNNNWATGCAAATNNNWNNW	CAATGA-TATTTAAATTATTTTAA

M0013 5	V$OCT1_01	0.734071	5014	(+)	NNNNWTATGCAAATNTNNN	AATGATATTTAAATTATTT
M0013 6	V$OCT1_02	0.794532	5014	(+)	NNGAATATKCANNNN	AATGATATTTAAATT
M0013 5	V$OCT1_01	0.7438	5015	(-)	NNNNWTATGCAAATNTNNN	ATGATATTTAAATTATTTT
M0019 5	V$OCT1_Q6	0.804312	5016	(+)	NNNNATGCAAATNAN	TGATATTTAAATTAT
M0016 2	V$OCT1_06	0.847656	5017	(+)	CWNAWTKWSATRYN	GATATTTAAATTAT
M0016 2	V$OCT1_06	0.916797	5017	(-)	CWNAWTKWSATRYN	GATATTTAAATTAT
M0024 8	V$OCT1_07	0.872589	5017	(+)	TNTATGNTAATT	GATATTTAAATT
M0013 8	V$OCT1_04	0.81054	5021	(+)	NNNNNNNWATGCAAATNNNW NNW	TTTAAATTATTTTAAAGTTA GT
M0016 2	V$OCT1_06	0.841797	5057	(+)	CWNAWTKWSATRYN	ATATTTTACAAGTT
M0016 2	V$OCT1_06	0.826562	5074	(+)	CWNAWTKWSATRYN	CTTTATTGGTTTCA
M0016 2	V$OCT1_06	0.839453	5106	(-)	CWNAWTKWSATRYN	AGAAACACAAAATG
M0013 5	V$OCT1_01	0.731973	5141	(+)	NNNNWTATGCAAATNTNNN	TTTCTTAAGATAATGTGGC
M0013 7	V$OCT1_03	0.846701	5147	(+)	NNNRTAATNANNN	AAGATAATGTGGC
M0016 2	V$OCT1_06	0.806641	5163	(-)	CWNAWTKWSATRYN	AAAATGTATTCTTG
M0016 2	V$OCT1_06	0.813672	5187	(+)	CWNAWTKWSATRYN	AGTGTTTTAATTTA
M0024 8	V$OCT1_07	0.817257	5187	(+)	TNTATGNTAATT	AGTGTTTTAATT
M0013 6	V$OCT1_02	0.84732	5202	(-)	NNGAATATKCANNNN	AAAAAGAATATTTGT
M0013 6	V$OCT1_02	0.829995	5205	(+)	NNGAATATKCANNNN	AAGAATATTTGTCTT
M0016 2	V$OCT1_06	0.826172	5209	(+)	CWNAWTKWSATRYN	ATATTTGTCTTACT
M0016 2	V$OCT1_06	0.821094	5230	(-)	CWNAWTKWSATRYN	TACTTCCCATAAAC
M0024 8	V$OCT1_07	0.800933	5231	(-)	TNTATGNTAATT	ACTTCCCATAAA
M0016 2	V$OCT1_06	0.834375	5240	(-)	CWNAWTKWSATRYN	AAACTTTCAAAATG
M0016 2	V$OCT1_06	0.821094	5272	(+)	CWNAWTKWSATRYN	GAATTCTAAATATT
M0016 2	V$OCT1_06	0.834375	5272	(-)	CWNAWTKWSATRYN	GAATTCTAAATATT
M0013 6	V$OCT1_02	0.826746	5274	(-)	NNGAATATKCANNNN	ATTCTAAATATTGAT
M0013 6	V$OCT1_02	0.786952	5277	(+)	NNGAATATKCANNNN	CTAAATATTGATTTT
M0016 2	V$OCT1_06	0.805469	5287	(+)	CWNAWTKWSATRYN	ATTTTATTGATTTA
M0016 2	V$OCT1_06	0.821484	5299	(-)	CWNAWTKWSATRYN	TAACTTTAAAGCTG
M0016 2	V$OCT1_06	0.847266	5334	(-)	CWNAWTKWSATRYN	TGAATGAATAAATG
M0016 2	V$OCT1_06	0.847266	5354	(-)	CWNAWTKWSATRYN	TGAATGAATAAATG
M0013 8	V$OCT1_04	0.805102	5357	(+)	NNNNNNNWATGCAAATNNNW NNW	ATGAA-TAAATGCAATTATAGACA
M0013 5	V$OCT1_01	0.767074	5359	(+)	NNNNWTATGCAAATNTNNN	GAATAAATGCAATTATAGA
M0013 6	V$OCT1_02	0.771792	5359	(+)	NNGAATATKCANNNN	GAATAAATGCAATTA

M00162	V$OCT1_06	0.876172	5362	(-)	CWNAWTKWSATRYN	TAAATGCAATTATA
M00137	V$OCT1_03	0.841565	5365	(-)	NNNRTAATNANNN	ATGCAATTATAGA
M00162	V$OCT1_06	0.834375	5400	(-)	CWNAWTKWSATRYN	AAACTGAAAGAATG
M00162	V$OCT1_06	0.839844	5424	(-)	CWNAWTKWSATRYN	GGTATTATATAAAG
M00138	V$OCT1_04	0.838561	5453	(-)	NNNNNNNWATGCAAATNNNWNNW	AAGAATGTTTTACATAATT-AGTT
M00135	V$OCT1_01	0.790919	5455	(-)	NNNNWTATGCAAATNTNNN	GAATGTTTTACATAATTAG
M00162	V$OCT1_06	0.834375	5457	(+)	CWNAWTKWSATRYN	ATGTTTTACATAAT
M00195	V$OCT1_Q6	0.832697	5457	(-)	NNNNATGCAAATNAN	ATGTTTTACATAATT
M00162	V$OCT1_06	0.821094	5458	(-)	CWNAWTKWSATRYN	TGTTTTACATAATT
M00137	V$OCT1_03	0.926906	5463	(+)	NNNRTAATNANNN	TACATAATTAGTT
M00137	V$OCT1_03	0.873173	5464	(-)	NNNRTAATNANNN	ACATAATTAGTTA
M00162	V$OCT1_06	0.894141	5465	(+)	CWNAWTKWSATRYN	CATAATTAGTTATT
M00137	V$OCT1_03	0.886211	5471	(-)	NNNRTAATNANNN	TAGTTATTATTCA
M00136	V$OCT1_02	0.872225	5473	(+)	NNGAATATKCANNNN	GTTATTATTCACTTT
M00162	V$OCT1_06	0.804297	5493	(-)	CWNAWTKWSATRYN	AAAATGATTTTTTG
M00162	V$OCT1_06	0.813672	5499	(+)	CWNAWTKWSATRYN	ATTTTTTGGATCTG
M00162	V$OCT1_06	0.805859	5524	(+)	CWNAWTKWSATRYN	AGATTTTTAATAGA
M00162	V$OCT1_06	0.821484	5525	(-)	CWNAWTKWSATRYN	GATTTTTAATAGAG
M00162	V$OCT1_06	0.826172	5566	(+)	CWNAWTKWSATRYN	AAATTTGTGAGATA
M00138	V$OCT1_04	0.785445	5575	(-)	NNNNNNNWATGCAAATNNNWNNW	AGATAAAATAAATATCTTT-GTAA
M00162	V$OCT1_06	0.834375	5577	(+)	CWNAWTKWSATRYN	ATAAAATAAATATC
M00136	V$OCT1_02	0.779643	5579	(-)	NNGAATATKCANNNN	AAAATAAATATCTTT
M00162	V$OCT1_06	0.839453	5598	(+)	CWNAWTKWSATRYN	CTATTCTTCATACC
M00138	V$OCT1_04	0.788791	5602	(-)	NNNNNNNWATGCAAATNNNWNNW	TCTTCATACCTGAATGAT-TATCT
M00136	V$OCT1_02	0.771792	5608	(-)	NNGAATATKCANNNN	TACCTGAATGATTAT
M00137	V$OCT1_03	0.862505	5613	(-)	NNNRTAATNANNN	GAATGATTATCTT
M00137	V$OCT1_03	0.86211	5623	(-)	NNNRTAATNANNN	CTTTGATTATCTT
M00138	V$OCT1_04	0.812422	5670	(+)	NNNNNNNWATGCAAATNNNWNNW	GTAAGATTATTTTTAAAC-TATTA
M00136	V$OCT1_02	0.771251	5672	(+)	NNGAATATKCANNNN	AAGATTATTTTTAAA
M00138	V$OCT1_04	0.801547	5673	(-)	NNNNNNNWATGCAAATNNNWNNW	AGATTATTTTTAAAC-TATTAAAT
M00162	V$OCT1_06	0.90625	5685	(+)	CWNAWTKWSATRYN	AACTATTAAATATA
M00137	V$OCT1_03	0.867641	5710	(+)	NNNRTAATNANNN	GACGTAAAAACAT
M00162	V$OCT1_06	0.828906	5717	(+)	CWNAWTKWSATRYN	AAACATTTCCTTTA

M00162	V$OCT1_06	0.821484	5723	(+)	CWNAWTKWSATRYN	TTCCTTTAAATACC
M00136	V$OCT1_02	0.773416	5743	(-)	NNGAATATKCANNNN	AAAATGAATAAATAA
M00137	V$OCT1_03	0.848281	5747	(+)	NNNRTAATNANNN	TGAATAAATAAGT
M00162	V$OCT1_06	0.800781	5769	(-)	CWNAWTKWSATRYN	CAAAACTAAGACAG
M00162	V$OCT1_06	0.821094	5782	(-)	CWNAWTKWSATRYN	GATATCTCTAATTC
M00138	V$OCT1_04	0.809076	5784	(+)	NNNNNNNWATGCAAATNNNWNNW	TATCTCTAATTCAAA-GAAAAGAA
M00135	V$OCT1_01	0.754865	5809	(-)	NNNNWTATGCAAATNTNNN	TACAGCATAGCATATGAAA
M00162	V$OCT1_06	0.842578	5811	(+)	CWNAWTKWSATRYN	CAGCATAGCATATG
M00136	V$OCT1_02	0.77477	5813	(-)	NNGAATATKCANNNN	GCATAGCATATGAAA
M00162	V$OCT1_06	0.847656	5819	(-)	CWNAWTKWSATRYN	CATATGAAAGCCAG
M00162	V$OCT1_06	0.800391	5831	(-)	CWNAWTKWSATRYN	AGAATTAACTGAAT
M00136	V$OCT1_02	0.775582	5836	(-)	NNGAATATKCANNNN	TAACTGAATAACTAA
M00138	V$OCT1_04	0.790882	5849	(+)	NNNNNNNWATGCAAATNNNWNNW	AAACCAACACTCAAATTC-TATGG
M00162	V$OCT1_06	0.821875	5854	(+)	CWNAWTKWSATRYN	AACACTCAAATTCT
M00162	V$OCT1_06	0.813672	5854	(-)	CWNAWTKWSATRYN	AACACTCAAATTCT
M00136	V$OCT1_02	0.778289	5933	(+)	NNGAATATKCANNNN	AAAAAAATCCATTTT
M00162	V$OCT1_06	0.847656	5935	(-)	CWNAWTKWSATRYN	AAAAATCCATTTTG
M00138	V$OCT1_04	0.7867	5937	(-)	NNNNNNNWATGCAAATNNNWNNW	AAATCCATTTTGCTTAAA-TATTT
M00135	V$OCT1_01	0.789966	5939	(-)	NNNNWTATGCAAATNTNNN	ATCCATTTTGCTTAAATAT
M00136	V$OCT1_02	0.840552	5947	(-)	NNGAATATKCANNNN	TGCTTAAATATTTAT
M00138	V$OCT1_04	0.823505	5947	(-)	NNNNNNNWATGCAAATNNNWNNW	TGCTTAAATATTTA-TATGCCATT
M00138	V$OCT1_04	0.80343	5948	(+)	NNNNNNNWATGCAAATNNNWNNW	GCTTAAATATTTA-TATGCCATTG
M00136	V$OCT1_02	0.791012	5950	(+)	NNGAATATKCANNNN	TTAAATATTTATATG
M00137	V$OCT1_03	0.84196	5952	(-)	NNNRTAATNANNN	AAATATTTATATG
M00162	V$OCT1_06	0.876172	5953	(+)	CWNAWTKWSATRYN	AATATTTATATGCC
M00162	V$OCT1_06	0.813672	5953	(-)	CWNAWTKWSATRYN	AATATTTATATGCC
M00136	V$OCT1_02	0.801841	5956	(+)	NNGAATATKCANNNN	ATTTATATGCCATTG
M00162	V$OCT1_06	0.868359	5959	(-)	CWNAWTKWSATRYN	TATATGCCATTGAA
M00137	V$OCT1_03	0.860529	5982	(-)	NNNRTAATNANNN	TTTTATTTACCAC
M00162	V$OCT1_06	0.855078	5992	(-)	CWNAWTKWSATRYN	CACATCACCTTTTT
M00162	V$OCT1_06	0.813672	5998	(+)	CWNAWTKWSATRYN	ACCTTTTTAATATA
M00162	V$OCT1_06	0.804297	6003	(+)	CWNAWTKWSATRYN	TTTAATATAATGCT
M00162	V$OCT1_06	0.808594	6024	(-)	CWNAWTKWSATRYN	AACATCCAAGACCA

M00138	V$OCT1_04	0.814931	6047	(+)	NNNNNNNNWATGCAAATNNNWNNW	AAGAAGTAAA-GCTAATTAAAAGA
M00248	V$OCT1_07	0.810049	6052	(+)	TNTATGNTAATT	GTAAAGCTAATT
M00138	V$OCT1_04	0.812003	6053	(+)	NNNNNNNNWATGCAAATNNNWNNW	TAAAGCTAATTAAAA-GAAAATAA
M00137	V$OCT1_03	0.847491	6056	(-)	NNNRTAATNANNN	AGCTAATTAAAAG
M00248	V$OCT1_07	0.831673	6060	(-)	TNTATGNTAATT	AATTAAAAGAAA
M00138	V$OCT1_04	0.820159	6074	(-)	NNNNNNNNWATGCAAATNNNWNNW	AAAGGTTATTTACATTGAT-TTAT
M00136	V$OCT1_02	0.772604	6075	(+)	NNGAATATKCANNNN	AAGGTTATTTACATT
M00162	V$OCT1_06	0.860547	6078	(+)	CWNAWTKWSATRYN	GTTATTTACATTGA
M00195	V$OCT1_Q6	0.916485	6078	(-)	NNNNATGCAAATNAN	GTTATTTACATTGAT
M00248	V$OCT1_07	0.817257	6080	(-)	TNTATGNTAATT	TATTTACATTGA
M00162	V$OCT1_06	0.85	6100	(-)	CWNAWTKWSATRYN	AACATATAAGAATC
M00162	V$OCT1_06	0.826562	6108	(-)	CWNAWTKWSATRYN	AGAATCTACGGTAG
M00248	V$OCT1_07	0.800085	6112	(+)	TNTATGNTAATT	TCTACGGTAGTT
M00162	V$OCT1_06	0.893359	6131	(+)	CWNAWTKWSATRYN	AACTCTGAAATGCA
M00138	V$OCT1_04	0.790673	6132	(+)	NNNNNNNNWATGCAAATNNNWNNW	ACTCTGAAATGCAGATTAG-TGAG
M00135	V$OCT1_01	0.7438	6134	(+)	NNNNWTATGCAAATNTNNN	TCTGAAATGCAGATTAGTG
M00195	V$OCT1_Q6	0.849891	6136	(+)	NNNNATGCAAATNAN	TGAAATGCAGATTAG
M00136	V$OCT1_02	0.790742	6137	(-)	NNGAATATKCANNNN	GAAATGCAGATTAGT
M00161	V$OCT1_05	0.886103	6137	(-)	MKNATTTGCATAYY	GAAATGCAGATTAG
M00162	V$OCT1_06	0.839844	6137	(-)	CWNAWTKWSATRYN	GAAATGCAGATTAG
M00138	V$OCT1_04	0.81033	6145	(+)	NNNNNNNNWATGCAAATNNNWNNW	GATTAG-TGAGGTAAATTCTTTGG
M00137	V$OCT1_03	0.840379	6193	(-)	NNNRTAATNANNN	TTTTCCTTATCAC
M00162	V$OCT1_06	0.805859	6201	(+)	CWNAWTKWSATRYN	ATCACTTGGCTATT
M00162	V$OCT1_06	0.852344	6214	(-)	CWNAWTKWSATRYN	TGTCTCAAATTTAT
M00135	V$OCT1_01	0.760588	6217	(-)	NNNNWTATGCAAATNTNNN	CTCAAATTTATATTTTCTT
M00162	V$OCT1_06	0.894531	6219	(+)	CWNAWTKWSATRYN	CAAATTTATATTTT
M00162	V$OCT1_06	0.821484	6219	(-)	CWNAWTKWSATRYN	CAAATTTATATTTT
M00195	V$OCT1_Q6	0.815775	6219	(-)	NNNNATGCAAATNAN	CAAATTTATATTTTC
M00248	V$OCT1_07	0.794149	6221	(-)	TNTATGNTAATT	AATTTATATTTT
M00138	V$OCT1_04	0.839398	6227	(-)	NNNNNNNNWATGCAAATNNNWNNW	TATTTTCTTTGGCATCTATA-TAT
M00162	V$OCT1_06	0.855078	6237	(-)	CWNAWTKWSATRYN	GGCATCTATATATT
M00135	V$OCT1_01	0.750095	6244	(+)	NNNNWTATGCAAATNTNNN	ATATATTTGGTAATTGTTA
M00248	V$OCT1_07	0.893152	6247	(+)	TNTATGNTAATT	TATTTGGTAATT

M00138	V$OCT1_04	0.796529	6248	(+)	NNNNNNNWATGCAAATNNNWNNW	ATTTGGTAATT-GTTATTTTACAT
M00137	V$OCT1_03	0.872382	6250	(+)	NNNRTAATNANNN	TTGGTAATTGTTA
M00162	V$OCT1_06	0.852734	6253	(+)	CWNAWTKWSATRYN	GTAATTGTTATTTT
M00138	V$OCT1_04	0.827896	6256	(-)	NNNNNNNWATGCAAATNNNWNNW	ATTGTTATTTTACATCTGAA-TAT
M00138	V$OCT1_04	0.844207	6262	(-)	NNNNNNNWATGCAAATNNNWNNW	ATTTTACATCTGAA-TATTTATCA
M00138	V$OCT1_04	0.823714	6263	(+)	NNNNNNNWATGCAAATNNNWNNW	TTTTACATCTGAA-TATTTATCAT
M00135	V$OCT1_01	0.784433	6264	(-)	NNNNWTATGCAAATNTNNN	TTTACATCTGAATATTTAT
M00162	V$OCT1_06	0.88125	6266	(-)	CWNAWTKWSATRYN	TACATCTGAATATT
M00136	V$OCT1_02	0.895777	6268	(-)	NNGAATATKCANNNN	CATCTGAATATTTAT
M00136	V$OCT1_02	0.857066	6271	(+)	NNGAATATKCANNNN	CTGAATATTTATCAT
M00138	V$OCT1_04	0.819532	6271	(-)	NNNNNNNWATGCAAATNNNWNNW	CTGAA-TATTTATCATAAAAAATA
M00138	V$OCT1_04	0.808448	6273	(+)	NNNNNNNWATGCAAATNNNWNNW	GAATATTTATCATAAAAAA-TAAA
M00138	V$OCT1_04	0.797365	6276	(+)	NNNNNNNWATGCAAATNNNWNNW	TATTTATCATAAAAAA-TAAAGTT
M00248	V$OCT1_07	0.818741	6277	(-)	TNTATGNTAATT	ATTTATCATAAA
M00162	V$OCT1_06	0.826562	6291	(+)	CWNAWTKWSATRYN	ATAAAGTTAATTCT
M00248	V$OCT1_07	0.817045	6291	(+)	TNTATGNTAATT	ATAAAGTTAATT
M00162	V$OCT1_06	0.829297	6292	(-)	CWNAWTKWSATRYN	TAAAGTTAATTCTC
M00162	V$OCT1_06	0.825	6318	(-)	CWNAWTKWSATRYN	AGGATCACATCATG
M00162	V$OCT1_06	0.834375	6325	(+)	CWNAWTKWSATRYN	CATCATGGAAGACA
M00138	V$OCT1_04	0.819532	6367	(-)	NNNNNNNWATGCAAATNNNWNNW	TGTATACTTATGTATTATA-CATA
M00195	V$OCT1_Q6	0.791485	6401	(-)	NNNNATGCAAATNAN	ATGCTTTACTTTATC
M00162	V$OCT1_06	0.821484	6439	(+)	CWNAWTKWSATRYN	AAGATTGAACTTTT
M00248	V$OCT1_07	0.791605	6500	(-)	TNTATGNTAATT	AGTTAACATTAG
M00162	V$OCT1_06	0.911328	6518	(+)	CWNAWTKWSATRYN	GAATTTGTCATTTA
M00138	V$OCT1_04	0.785027	6519	(+)	NNNNNNNWATGCAAATNNNWNNW	AATTTGTCATTTAAATGA-TAATC
M00162	V$OCT1_06	0.811719	6519	(-)	CWNAWTKWSATRYN	AATTTGTCATTTAA
M00138	V$OCT1_04	0.813676	6520	(-)	NNNNNNNWATGCAAATNNNWNNW	ATTTGTCATTTAAATGA-TAATCA
M00162	V$OCT1_06	0.847656	6524	(+)	CWNAWTKWSATRYN	GTCATTTAAATGAT
M00162	V$OCT1_06	0.834766	6524	(-)	CWNAWTKWSATRYN	GTCATTTAAATGAT
M00138	V$OCT1_04	0.81765	6525	(+)	NNNNNNNWATGCAAATNNNWNNW	TCATTTAAATGATAATCAG-TGAA
M00195	V$OCT1_Q6	0.823417	6529	(+)	NNNNATGCAAATNAN	TTAAATGATAATCAG
M00161	V$OCT1_05	0.85904	6530	(-)	MKNATTTGCATAYY	TAAATGATAATCAG
M00162	V$OCT1_06	0.866016	6530	(-)	CWNAWTKWSATRYN	TAAATGATAATCAG

M00137	V$OCT1_03	0.90004	6533	(+)	NNNRTAATNANNN	ATGATAATCAGTG
M00162	V$OCT1_06	0.808203	6549	(-)	CWNAWTKWSATRYN	AGTTTGTCAGAGAT
M00162	V$OCT1_06	0.813672	6586	(-)	CWNAWTKWSATRYN	AGAATTCAGAGATG
M00195	V$OCT1_Q6	0.795579	6636	(+)	NNNNATGCAAATNAN	AGGTATAGAAAGGAC
M00137	V$OCT1_03	0.84275	6709	(-)	NNNRTAATNANNN	AGCTGGTTACTAG
M00137	V$OCT1_03	0.8629	6725	(+)	NNNRTAATNANNN	AGTGTAAACATAA
M00136	V$OCT1_02	0.787223	6740	(-)	NNGAATATKCANNNN	GGGCGGAATGTTCAT
M00136	V$OCT1_02	0.792366	6743	(+)	NNGAATATKCANNNN	CGGAATGTTCATTGG
M00248	V$OCT1_07	0.808141	6744	(+)	TNTATGNTAATT	GGAATGTTCATT
M00161	V$OCT1_05	0.873651	6784	(+)	MKNATTTGCATAYY	CTGATTTTCATCCC
M00162	V$OCT1_06	0.873828	6784	(+)	CWNAWTKWSATRYN	CTGATTTTCATCCC
M00195	V$OCT1_Q6	0.847707	6784	(-)	NNNNATGCAAATNAN	CTGATTTTCATCCCA
M00138	V$OCT1_04	0.791301	6823	(+)	NNNNNNNWATGCAAATNNNWNNW	AGATTTTT-GTTCTTATTTAATCT
M00162	V$OCT1_06	0.834375	6846	(-)	CWNAWTKWSATRYN	TGTTTGAAAGTGTT
M00162	V$OCT1_06	0.852734	6888	(+)	CWNAWTKWSATRYN	CTGATTTTATTGTA
M00138	V$OCT1_04	0.786073	6889	(-)	NNNNNNNWATGCAAATNNNWNNW	TGATTTTATTGTAATGAGAG-CAT
M00248	V$OCT1_07	0.834429	6893	(+)	TNTATGNTAATT	TTTATTGTAATG
M00137	V$OCT1_03	0.924931	6896	(+)	NNNRTAATNANNN	ATTGTAATGAGAG
M00136	V$OCT1_02	0.777206	6903	(-)	NNGAATATKCANNNN	TGAGAGCATAGTGAA
M00162	V$OCT1_06	0.889062	6926	(+)	CWNAWTKWSATRYN	TACATTTAGATGCT
M00162	V$OCT1_06	0.821484	6926	(-)	CWNAWTKWSATRYN	TACATTTAGATGCT
M00195	V$OCT1_Q6	0.799945	6997	(+)	NNNNATGCAAATNAN	AAATGTGTGAATTCT
M00138	V$OCT1_04	0.806148	6999	(+)	NNNNNNNWATGCAAATNNNWNNW	ATGTGTGAATTCTTATCAG-TCCT
M00136	V$OCT1_02	0.77856	7000	(-)	NNGAATATKCANNNN	TGTGTGAATTCTTAT
M00162	V$OCT1_06	0.804297	7080	(-)	CWNAWTKWSATRYN	TTCCTGCCATTTAG
M00248	V$OCT1_07	0.804113	7107	(+)	TNTATGNTAATT	TCTCTGCTCATT
M00138	V$OCT1_04	0.797574	7134	(+)	NNNNNNNWATGCAAATNNNWNNW	AAAAAACATT-GCTTATTTATTAA
M00162	V$OCT1_06	0.828906	7137	(+)	CWNAWTKWSATRYN	AAACATTGCTTATT
M00136	V$OCT1_02	0.790742	7139	(-)	NNGAATATKCANNNN	ACATTGCTTATTTAT
M00138	V$OCT1_04	0.800293	7139	(-)	NNNNNNNWATGCAAATNNNWNNW	ACATTGCTTATTTATTAAAA-TAA
M00138	V$OCT1_04	0.871602	7144	(+)	NNNNNNNWATGCAAATNNNWNNW	GCTTATTTATTAAAATAAA-GAGT
M00135	V$OCT1_01	0.731973	7146	(+)	NNNNWTATGCAAATNTNNN	TTATTTATTAAAATAAAGA
M00135	V$OCT1_01	0.731973	7147	(-)	NNNNWTATGCAAATNTNNN	TATTTATTAAAATAAAGAG

M00248	V$OCT1_07	0.831673	7151	(-)	TNTATGNTAATT	TATTAAAATAAA
M00137	V$OCT1_03	0.846306	7155	(+)	NNNRTAATNANNN	AAAATAAAGAGTA
M00162	V$OCT1_06	0.800391	7167	(-)	CWNAWTKWSATRYN	AAAAAGCAAAATAT
M00162	V$OCT1_06	0.865625	7168	(-)	CWNAWTKWSATRYN	AAAAGCAAAATATG
M00138	V$OCT1_04	0.814304	7170	(-)	NNNNNNNWATGCAAATNNNWNNW	AAGCAAAA-TATGTATGAATGTTT
M00136	V$OCT1_02	0.814835	7173	(+)	NNGAATATKCANNNN	CAAAATATGTATGAA
M00138	V$OCT1_04	0.791092	7174	(-)	NNNNNNNWATGCAAATNNNWNNW	AAAA-TATGTATGAATGTTTAAAA
M00138	V$OCT1_04	0.832706	7175	(+)	NNNNNNNWATGCAAATNNNWNNW	AAA-TATGTATGAATGTTTAAAAT
M00162	V$OCT1_06	0.855078	7176	(-)	CWNAWTKWSATRYN	AATATGTATGAATG
M00136	V$OCT1_02	0.811586	7180	(-)	NNGAATATKCANNNN	TGTATGAATGTTTAA
M00138	V$OCT1_04	0.820368	7181	(+)	NNNNNNNWATGCAAATNNNWNNW	GTATGAATGTTTAAAATGAACTA
M00162	V$OCT1_06	0.833984	7386	(-)	CWNAWTKWSATRYN	AGCCTCAAAAATTT
M00135	V$OCT1_01	0.77852	7391	(-)	NNNNWTATGCAAATNTNNN	CAAAAATTTGGTTAATCAC
M00137	V$OCT1_03	0.881075	7399	(+)	NNNRTAATNANNN	TGGTTAATCACGT
M00136	V$OCT1_02	0.862209	7419	(-)	NNGAATATKCANNNN	GAGATGAATAGTCTG
M00138	V$OCT1_04	0.793183	7425	(-)	NNNNNNNWATGCAAATNNNWNNW	AATAGTCTGTT-GCATATTAAAAT
M00135	V$OCT1_01	0.777757	7427	(-)	NNNNWTATGCAAATNTNNN	TAGTCTGTTGCATATTAAA
M00162	V$OCT1_06	0.821484	7429	(+)	CWNAWTKWSATRYN	GTCTGTTGCATATT
M00136	V$OCT1_02	0.883324	7431	(-)	NNGAATATKCANNNN	CTGTTGCATATTAAA
M00138	V$OCT1_04	0.823714	7432	(+)	NNNNNNNWATGCAAATNNNWNNW	TGTT-GCATATTAAAATCAATCTT
M00162	V$OCT1_06	0.839844	7437	(-)	CWNAWTKWSATRYN	CATATTAAAATCAA
M00138	V$OCT1_04	0.79151	7446	(-)	NNNNNNNWATGCAAATNNNWNNW	ATCAATCTTTTAAA-TATGTCCTT
M00162	V$OCT1_06	0.877734	7450	(+)	CWNAWTKWSATRYN	ATCTTTTAAATATG
M00162	V$OCT1_06	0.804297	7450	(-)	CWNAWTKWSATRYN	ATCTTTTAAATATG
M00162	V$OCT1_06	0.812109	7458	(-)	CWNAWTKWSATRYN	AATATGTCCTTGGG
M00138	V$OCT1_04	0.785027	7475	(-)	NNNNNNNWATGCAAATNNNWNNW	AAAAATTA-TATCCACATGCAAAT
M00162	V$OCT1_06	0.819922	7475	(+)	CWNAWTKWSATRYN	AAAAATTATATCCA
M00136	V$OCT1_02	0.776394	7478	(+)	NNGAATATKCANNNN	AATTATATCCACATG
M00138	V$OCT1_04	0.898787	7482	(+)	NNNNNNNWATGCAAATNNNWNNW	ATATCCACATGCAAA-TATAAACA
M00135	V$OCT1_01	0.890309	7484	(+)	NNNNWTATGCAAATNTNNN	ATCCACATGCAAATATAAA
M00136	V$OCT1_02	0.813481	7484	(+)	NNGAATATKCANNNN	ATCCACATGCAAATA
M00195	V$OCT1_Q6	0.80595	7486	(+)	NNNNATGCAAATNAN	CCACATGCAAATATA
M00162	V$OCT1_06	0.876172	7487	(-)	CWNAWTKWSATRYN	CACATGCAAATATA

M00136	V$OCT1_02	0.805089	7521	(+)	NNGAATATKCANNNN	AAGAAAACTCAATAT
M00162	V$OCT1_06	0.834375	7523	(-)	CWNAWTKWSATRYN	GAAAACTCAATATT
M00162	V$OCT1_06	0.821484	7531	(-)	CWNAWTKWSATRYN	AATATTTCAAAGCA
M00135	V$OCT1_01	0.761923	7552	(+)	NNNNWTATGCAAATNTNNN	TTTAAAAAGCTAATGTAAT
M00162	V$OCT1_06	0.847656	7560	(+)	CWNAWTKWSATRYN	GCTAATGTAATTTT
M00248	V$OCT1_07	0.806233	7560	(+)	TNTATGNTAATT	GCTAATGTAATT
M00162	V$OCT1_06	0.807031	7561	(-)	CWNAWTKWSATRYN	CTAATGTAATTTTA
M00162	V$OCT1_06	0.805859	7566	(+)	CWNAWTKWSATRYN	GTAATTTTAGTTCC
M00138	V$OCT1_04	0.821205	7592	(+)	NNNNNNNWATGCAAATNNNWNNW	GTTGTTA-TATTTTAATGTTTCCA
M00138	V$OCT1_04	0.849017	7593	(-)	NNNNNNNWATGCAAATNNNWNNW	TTGTTA-TATTTTAATGTTTCCAA
M00135	V$OCT1_01	0.745135	7594	(+)	NNNNWTATGCAAATNTNNN	TGTTATATTTTAATGTTTC
M00162	V$OCT1_06	0.88125	7597	(+)	CWNAWTKWSATRYN	TATATTTTAATGTT
M00162	V$OCT1_06	0.847656	7597	(-)	CWNAWTKWSATRYN	TATATTTTAATGTT
M00248	V$OCT1_07	0.831673	7597	(+)	TNTATGNTAATT	TATATTTTAATG
M00162	V$OCT1_06	0.81875	7607	(-)	CWNAWTKWSATRYN	TGTTTCCAAGTTAT
M00137	V$OCT1_03	0.856974	7625	(+)	NNNRTAATNANNN	AGCATAAAAAGGG
M00162	V$OCT1_06	0.868359	7645	(-)	CWNAWTKWSATRYN	TGTATGTGAGAATG
M00136	V$OCT1_02	0.799404	7653	(-)	NNGAATATKCANNNN	AGAATGAATGCTCAG
M00162	V$OCT1_06	0.800781	7665	(+)	CWNAWTKWSATRYN	CAGCCTGGGGTTCT
M00137	V$OCT1_03	0.874753	7715	(-)	NNNRTAATNANNN	TCATTTTTATCAC
M00138	V$OCT1_04	0.806985	7718	(+)	NNNNNNNWATGCAAATNNNWNNW	TTTTTATCACAAAAATTCAT-ACA
M00162	V$OCT1_06	0.831641	7720	(-)	CWNAWTKWSATRYN	TTTATCACAAAAAT
M00138	V$OCT1_04	0.79151	7724	(+)	NNNNNNNWATGCAAATNNNWNNW	TCACAAAAATTCATA-CACATTTT
M00136	V$OCT1_02	0.833785	7726	(+)	NNGAATATKCANNNN	ACAAAAATTCATACA
M00162	V$OCT1_06	0.894141	7727	(+)	CWNAWTKWSATRYN	CAAAAATTCATACA
M00138	V$OCT1_04	0.780845	7736	(-)	NNNNNNNWATGCAAATNNNWNNW	ATACACATTTTACATTCTAG-TCA
M00162	V$OCT1_06	0.842187	7740	(+)	CWNAWTKWSATRYN	ACATTTTACATTCT
M00248	V$OCT1_07	0.797965	7742	(-)	TNTATGNTAATT	ATTTTACATTCT
M00138	V$OCT1_04	0.796738	7769	(-)	NNNNNNNWATGCAAATNNNWNNW	TGTGTTTGTTTTAA-TAAAAAATT
M00138	V$OCT1_04	0.7844	7774	(+)	NNNNNNNWATGCAAATNNNWNNW	TTGTTTTAATAAAAAATT-GTTTT
M00137	V$OCT1_03	0.84196	7776	(+)	NNNRTAATNANNN	GTTTTAATAAAAA
M00138	V$OCT1_04	0.78189	7831	(-)	NNNNNNNWATGCAAATNNNWNNW	CAACATATTTTAAATGAAATTGA
M00162	V$OCT1_06	0.834375	7835	(+)	CWNAWTKWSATRYN	ATATTTTAAATGAA

M00195	V$OCT1_Q6	0.790393	7840	(+)	NNNNATGCAAATNAN	TTAAATGAAATTGAC
M00162	V$OCT1_06	0.924609	7841	(-)	CWNAWTKWSATRYN	TAAATGAAATTGAC
M00162	V$OCT1_06	0.855078	7846	(+)	CWNAWTKWSATRYN	GAAATTGACAGACC
M00136	V$OCT1_02	0.775311	7863	(-)	NNGAATATKCANNNN	AAAGTACATAGTATT
M00248	V$OCT1_07	0.800297	7885	(+)	TNTATGNTAATT	TATATACTACTT
M00162	V$OCT1_06	0.852734	7894	(+)	CWNAWTKWSATRYN	CTTGTTTTAATTCT
M00135	V$OCT1_01	0.769172	7925	(+)	NNNNWTATGCAAATNTNNN	CCCGGTATGAAAGTGTGGG
M00162	V$OCT1_06	0.937891	7928	(-)	CWNAWTKWSATRYN	GGTATGAAAGTGTG
M00162	V$OCT1_06	0.821484	7957	(+)	CWNAWTKWSATRYN	CACTTTTAAAATCA
M00162	V$OCT1_06	0.890625	7964	(-)	CWNAWTKWSATRYN	AAAATCACAGGATC
M00161	V$OCT1_05	0.864851	7985	(+)	MKNATTTGCATAYY	CTGATTTTCTTTTT
M00162	V$OCT1_06	0.852734	7985	(+)	CWNAWTKWSATRYN	CTGATTTTCTTTTT
M00195	V$OCT1_Q6	0.849618	7985	(-)	NNNNATGCAAATNAN	CTGATTTTCTTTTTT
M00162	V$OCT1_06	0.812109	8036	(-)	CWNAWTKWSATRYN	GGTAAATCATTTTG
M00161	V$OCT1_05	0.853561	8041	(+)	MKNATTTGCATAYY	ATCATTTTGATGTC
M00162	V$OCT1_06	0.916797	8041	(+)	CWNAWTKWSATRYN	ATCATTTTGATGTC
M00138	V$OCT1_04	0.822041	8042	(+)	NNNNNNNWATGCAAATNNNWNNW	TCATTTT-GATGTCAATTCATTAT
M00248	V$OCT1_07	0.800933	8047	(+)	TNTATGNTAATT	TTGATGTCAATT
M00137	V$OCT1_03	0.920585	8055	(-)	NNNRTAATNANNN	AATTCATTATCCC
M00135	V$OCT1_01	0.751049	8057	(+)	NNNNWTATGCAAATNTNNN	TTCATTATCCCAATCAGAA
M00195	V$OCT1_Q6	0.803766	8059	(+)	NNNNATGCAAATNAN	CATTATCCCAATCAG
M00162	V$OCT1_06	0.858203	8060	(-)	CWNAWTKWSATRYN	ATTATCCCAATCAG
M00137	V$OCT1_03	0.850257	8063	(+)	NNNRTAATNANNN	ATCCCAATCAGAA
M00138	V$OCT1_04	0.788164	8069	(+)	NNNNNNNWATGCAAATNNNWNNW	ATCAGAAAATGTTTATGAAAGCT
M00137	V$OCT1_03	0.891347	8075	(-)	NNNRTAATNANNN	AAATGTTTATGAA
M00138	V$OCT1_04	0.809494	8075	(+)	NNNNNNNWATGCAAATNNNWNNW	AAATGTTTATGAAA-GCTTATTGT
M00138	V$OCT1_04	0.780217	8099	(-)	NNNNNNNWATGCAAATNNNWNNW	CCATGAAGTTTACATATCC-TACA
M00135	V$OCT1_01	0.807325	8101	(-)	NNNNWTATGCAAATNTNNN	ATGAAGTTTACATATCCTA
M00162	V$OCT1_06	0.862891	8103	(+)	CWNAWTKWSATRYN	GAAGTTTACATATC
M00195	V$OCT1_Q6	0.794487	8103	(-)	NNNNATGCAAATNAN	GAAGTTTACATATCC
M00136	V$OCT1_02	0.800758	8105	(-)	NNGAATATKCANNNN	AGTTTACATATCCTA
M00248	V$OCT1_07	0.833369	8105	(-)	TNTATGNTAATT	AGTTTACATATC
M00137	V$OCT1_03	0.849467	8124	(-)	NNNRTAATNANNN	GTCTGATTGCACA

M00135	V$OCT1_01	0.764403	8137	(-)	NNNNWTATGCAAATNTNNN	AGGCACTTTGTATACAGCA
M00195	V$OCT1_Q6	0.829967	8139	(-)	NNNNATGCAAATNAN	GCACTTTGTATACAG
M00137	V$OCT1_03	0.850257	8159	(-)	NNNRTAATNANNN	TCCTCATTTTGAC
M00162	V$OCT1_06	0.857812	8161	(+)	CWNAWTKWSATRYN	CTCATTTTGACACA
M00162	V$OCT1_06	0.867969	8163	(+)	CWNAWTKWSATRYN	CATTTTGACACACA
M00138	V$OCT1_04	0.799875	8168	(+)	NNNNNNNWATGCAAATNNNWNNW	TGACACACATGGA-TATCAACCCA
M00162	V$OCT1_06	0.826953	8171	(+)	CWNAWTKWSATRYN	CACACATGGATATC
M00136	V$OCT1_02	0.791554	8173	(-)	NNGAATATKCANNNN	CACATGGATATCAAC
M00138	V$OCT1_04	0.81995	8191	(+)	NNNNNNNWATGCAAATNNNWNNW	ACAGATGTATGAAAACAAA-TATT
M00162	V$OCT1_06	0.803906	8196	(-)	CWNAWTKWSATRYN	TGTATGAAAACAAA
M00162	V$OCT1_06	0.808203	8201	(-)	CWNAWTKWSATRYN	GAAAACAAATATTT
M00162	V$OCT1_06	0.808203	8210	(-)	CWNAWTKWSATRYN	TATTTGTCAGAGAC
M00137	V$OCT1_03	0.858949	8222	(-)	NNNRTAATNANNN	ACAAAATTACAGA
M00162	V$OCT1_06	0.825	8223	(+)	CWNAWTKWSATRYN	CAAAATTACAGAAT
M00162	V$OCT1_06	0.877734	8224	(-)	CWNAWTKWSATRYN	AAAATTACAGAATT
M00162	V$OCT1_06	0.826562	8264	(+)	CWNAWTKWSATRYN	CTATCTGTGCTGCC
M00138	V$OCT1_04	0.851736	8281	(-)	NNNNNNNWATGCAAATNNNWNNW	ACATTTTTTTTCCTAATTTTTT
M00135	V$OCT1_01	0.731591	8285	(+)	NNNNWTATGCAAATNTNNN	TTTTTTTTCCTAATTTTTT
M00248	V$OCT1_07	0.800297	8288	(+)	TNTATGNTAATT	TTTTTCCTAATT
M00137	V$OCT1_03	0.845121	8291	(+)	NNNRTAATNANNN	TTCCTAATTTTTT
M00162	V$OCT1_06	0.878906	8313	(+)	CWNAWTKWSATRYN	CAGATTTTGATAGA
M00138	V$OCT1_04	0.788791	8327	(-)	NNNNNNNWATGCAAATNNNWNNW	ACTAAATCTT-GTCATTAAAAATT
M00162	V$OCT1_06	0.834375	8331	(+)	CWNAWTKWSATRYN	AATCTTGTCATTAA
M00137	V$OCT1_03	0.879099	8335	(-)	NNNRTAATNANNN	TTGTCATTAAAAA
M00162	V$OCT1_06	0.896094	8344	(-)	CWNAWTKWSATRYN	AAAATTACAGTAAT
M00248	V$OCT1_07	0.815137	8346	(-)	TNTATGNTAATT	AATTACAGTAAT
M00138	V$OCT1_04	0.791928	8348	(+)	NNNNNNNWATGCAAATNNNWNNW	TTACAGTAATGGC-TATGTTTCTA
M00162	V$OCT1_06	0.826562	8378	(-)	CWNAWTKWSATRYN	GATATCAACATGTT
M00137	V$OCT1_03	0.86369	8420	(+)	NNNRTAATNANNN	AGGCTAATCAGAC
M00162	V$OCT1_06	0.821875	8430	(+)	CWNAWTKWSATRYN	GACACTGGAATCCT
M00162	V$OCT1_06	0.805859	8499	(+)	CWNAWTKWSATRYN	CTTCCTTTAACTTT
M00162	V$OCT1_06	0.911719	8518	(+)	CWNAWTKWSATRYN	AAGACTTTCATTCA
M00138	V$OCT1_04	0.787537	8519	(+)	NNNNNNNWATGCAAATNNNWNNW	AGACTTTCATTCAAGTTACAAAA

M00162	V$OCT1_06	0.826953	8524	(-)	CWNAWTKWSATRYN	TTCATTCAAGTTAC
M00138	V$OCT1_04	0.782518	8525	(-)	NNNNNNNNWATGCAAATNNNWNNW	TCATTCAAGTTACAAAA-TAAAAC
M00248	V$OCT1_07	0.800933	8566	(+)	TNTATGNTAATT	TTTCTGGGAATT
M00136	V$OCT1_02	0.8817	8583	(-)	NNGAATATKCANNNN	AAAGTGAATATTTCA
M00136	V$OCT1_02	0.800487	8586	(+)	NNGAATATKCANNNN	GTGAATATTTCACAT
M00162	V$OCT1_06	0.828906	8588	(+)	CWNAWTKWSATRYN	GAATATTTCACATC
M00162	V$OCT1_06	0.821484	8589	(-)	CWNAWTKWSATRYN	AATATTTCACATCC
M00136	V$OCT1_02	0.773146	8612	(-)	NNGAATATKCANNNN	ATTCTGAATAACTTC
M00162	V$OCT1_06	0.857812	8663	(+)	CWNAWTKWSATRYN	GAGTATAACATGCA
M00162	V$OCT1_06	0.834375	8664	(-)	CWNAWTKWSATRYN	AGTATAACATGCAC
M00136	V$OCT1_02	0.791554	8666	(+)	NNGAATATKCANNNN	TATAACATGCACCTC
M00162	V$OCT1_06	0.855078	8675	(-)	CWNAWTKWSATRYN	CACCTCAAATTATT
M00137	V$OCT1_03	0.841169	8678	(-)	NNNRTAATNANNN	CTCAAATTATTCA
M00136	V$OCT1_02	0.83595	8680	(+)	NNGAATATKCANNNN	CAAATTATTCATCTT
M00162	V$OCT1_06	0.828906	8729	(+)	CWNAWTKWSATRYN	GTTTATTACAGTTA
M00162	V$OCT1_06	0.858203	8730	(-)	CWNAWTKWSATRYN	TTTATTACAGTTAG
M00137	V$OCT1_03	0.86448	8760	(+)	NNNRTAATNANNN	TGGTTAATTAGTA
M00195	V$OCT1_Q6	0.814138	8763	(-)	NNNNATGCAAATNAN	TTAATTAGTATTCTT
M00248	V$OCT1_07	0.829765	8765	(-)	TNTATGNTAATT	AATTAGTATTCT
M00136	V$OCT1_02	0.797239	8766	(+)	NNGAATATKCANNNN	ATTAGTATTCTTACC
M00162	V$OCT1_06	0.842187	8776	(+)	CWNAWTKWSATRYN	TTACCTGAAATGTA
M00138	V$OCT1_04	0.844207	8777	(+)	NNNNNNNNWATGCAAATNNNWNNW	TACCTGAAATGTAAATTAA-TAAA
M00135	V$OCT1_01	0.758871	8779	(+)	NNNNWTATGCAAATNTNNN	CCTGAAATGTAAATTAATA
M00195	V$OCT1_Q6	0.85262	8781	(+)	NNNNATGCAAATNAN	TGAAATGTAAATTAA
M00162	V$OCT1_06	0.868359	8782	(-)	CWNAWTKWSATRYN	GAAATGTAAATTAA
M00248	V$OCT1_07	0.877252	8782	(+)	TNTATGNTAATT	GAAATGTAAATT
M00138	V$OCT1_04	0.81995	8783	(+)	NNNNNNNNWATGCAAATNNNWNNW	AAATGTAAATTAA-TAAATCAAAA
M00162	V$OCT1_06	0.800391	8788	(-)	CWNAWTKWSATRYN	TAAATTAATAAATC
M00138	V$OCT1_04	0.790464	8791	(+)	NNNNNNNNWATGCAAATNNNWNNW	ATTAA-TAAATCAAAAAACTAACA
M00162	V$OCT1_06	0.882812	8796	(-)	CWNAWTKWSATRYN	TAAATCAAAAAACT
M00248	V$OCT1_07	0.808141	8806	(-)	TNTATGNTAATT	AACTAACATGGC
M00162	V$OCT1_06	0.870703	8809	(+)	CWNAWTKWSATRYN	TAACATGGCATGTT
M00162	V$OCT1_06	0.870703	8810	(-)	CWNAWTKWSATRYN	AACATGGCATGTTT

M00162	V$OCT1_06	0.88125	8817	(+)	CWNAWTKWSATRYN	CATGTTTTAATGTT
M00162	V$OCT1_06	0.826562	8827	(-)	CWNAWTKWSATRYN	TGTTTTTAAGGAAG
M00137	V$OCT1_03	0.886606	8866	(-)	NNNRTAATNANNN	ATATATTTACCAA
M00138	V$OCT1_04	0.79381	8871	(+)	NNNNNNNWATGCAAATNNNWNNW	TTTAC-CAAATTTAAAATATATTA
M00138	V$OCT1_04	0.7844	8873	(-)	NNNNNNNWATGCAAATNNNWNNW	TAC-CAAATTTAAAATATATTAGC
M00135	V$OCT1_01	0.731782	8875	(-)	NNNNWTATGCAAATNTNNN	CCAAATTTAAAATATATTA
M00162	V$OCT1_06	0.877734	8876	(-)	CWNAWTKWSATRYN	CAAATTTAAAATAT
M00162	V$OCT1_06	0.834375	8877	(+)	CWNAWTKWSATRYN	AAATTTAAAATATA
M00248	V$OCT1_07	0.831673	8879	(-)	TNTATGNTAATT	ATTTAAAATATA
M00138	V$OCT1_04	0.843998	8883	(-)	NNNNNNNWATGCAAATNNNWNNW	AAAATATATTAG-CATTATCCAGG
M00135	V$OCT1_01	0.856925	8885	(-)	NNNNWTATGCAAATNTNNN	AATATATTAGCATTATCCA
M00248	V$OCT1_07	0.823829	8889	(-)	TNTATGNTAATT	TATTAGCATTAT
M00137	V$OCT1_03	0.845121	8891	(-)	NNNRTAATNANNN	TTAGCATTATCCA
M00136	V$OCT1_02	0.777748	8893	(+)	NNGAATATKCANNNN	AGCATTATCCAGGAT
M00138	V$OCT1_04	0.780845	8900	(+)	NNNNNNNWATGCAAATNNNWNNW	TCCAGGA-TATGTTAAAATTTAAT
M00135	V$OCT1_01	0.793209	8902	(+)	NNNNWTATGCAAATNTNNN	CAGGATATGTTAAAATTTA
M00138	V$OCT1_04	0.786909	8902	(+)	NNNNNNNWATGCAAATNNNWNNW	CAGGA-TATGTTAAAATTTAATTA
M00162	V$OCT1_06	0.842187	8905	(-)	CWNAWTKWSATRYN	GATATGTTAAAATT
M00138	V$OCT1_04	0.815767	8909	(-)	NNNNNNNWATGCAAATNNNWNNW	TGTTAAAATTTAATTAAACAATT
M00136	V$OCT1_02	0.770709	8910	(+)	NNGAATATKCANNNN	GTTAAAATTTAATTA
M00162	V$OCT1_06	0.868359	8913	(-)	CWNAWTKWSATRYN	AAAATTTAATTAAA
M00248	V$OCT1_07	0.807717	8915	(-)	TNTATGNTAATT	AATTTAATTAAA
M00137	V$OCT1_03	0.846701	8916	(-)	NNNRTAATNANNN	ATTTAATTAAACA
M00138	V$OCT1_04	0.80092	8940	(-)	NNNNNNNWATGCAAATNNNWNNW	TTTTTTTTTTAG-CAGAAGGACAT
M00135	V$OCT1_01	0.735979	8942	(-)	NNNNWTATGCAAATNTNNN	TTTTTTTTAGCAGAAGGAC
M00162	V$OCT1_06	0.876172	8952	(+)	CWNAWTKWSATRYN	CAGAAGGACATTCT
M00162	V$OCT1_06	0.842187	8953	(-)	CWNAWTKWSATRYN	AGAAGGACATTCTC
M00136	V$OCT1_02	0.773687	8970	(+)	NNGAATATKCANNNN	GAAGATATGCCCTCC
M00138	V$OCT1_04	0.791301	8995	(-)	NNNNNNNWATGCAAATNNNWNNW	TGG-TAAAATTTTATTAAAAGTTT
M00138	V$OCT1_04	0.833333	8999	(+)	NNNNNNNWATGCAAATNNNWNNW	AAAATTTTATTAAAAGTTTATCA
M00137	V$OCT1_03	0.852232	9002	(-)	NNNRTAATNANNN	ATTTTATTAAAAG
M00162	V$OCT1_06	0.826562	9004	(-)	CWNAWTKWSATRYN	TTTATTAAAAGTTT
M00138	V$OCT1_04	0.787746	9015	(+)	NNNNNNNWATGCAAATNNNWNNW	TTTATCAAAGGCAAAGGTAACAA

M00137	V$OCT1_03	0.840379	9028	(+)	NNNRTAATNANNN	AAGGTAACAAGGA
M00195	V$OCT1_Q6	0.818504	9037	(+)	NNNNATGCAAATNAN	AGGAATGAGAAGAAC
M00162	V$OCT1_06	0.813672	9038	(-)	CWNAWTKWSATRYN	GGAATGAGAAGAAC
M00138	V$OCT1_04	0.820577	9088	(+)	NNNNNNNWATGCAAATNNNWNNW	AAACATTAATTCATAGTAAGTAA
M00162	V$OCT1_06	0.821094	9117	(+)	CWNAWTKWSATRYN	AGACTCTAGATGTT
M00162	V$OCT1_06	0.81875	9162	(+)	CWNAWTKWSATRYN	CTTTTTTTAAATCT
M00162	V$OCT1_06	0.890625	9169	(-)	CWNAWTKWSATRYN	TAAATCTCAGGTAT
M00162	V$OCT1_06	0.864844	9177	(+)	CWNAWTKWSATRYN	AGGTATTTAATTCA
M00162	V$OCT1_06	0.911719	9178	(-)	CWNAWTKWSATRYN	GGTATTTAATTCAT
M00162	V$OCT1_06	0.841797	9181	(+)	CWNAWTKWSATRYN	ATTTAATTCATGCT
M00162	V$OCT1_06	0.868359	9189	(+)	CWNAWTKWSATRYN	CATGCTTTCATTTA
M00138	V$OCT1_04	0.814931	9190	(+)	NNNNNNNWATGCAAATNNNWNNW	ATGCTTTCATTTAAA-TATTGTGT
M00138	V$OCT1_04	0.798202	9191	(-)	NNNNNNNWATGCAAATNNNWNNW	TGCTTTCATTTAAA-TATTGTGTT
M00135	V$OCT1_01	0.763449	9193	(-)	NNNNWTATGCAAATNTNNN	CTTTCATTTAAATATTGTG
M00162	V$OCT1_06	0.860547	9195	(+)	CWNAWTKWSATRYN	TTCATTTAAATATT
M00162	V$OCT1_06	0.868359	9195	(-)	CWNAWTKWSATRYN	TTCATTTAAATATT
M00195	V$OCT1_Q6	0.792031	9195	(-)	NNNNATGCAAATNAN	TTCATTTAAATATTG
M00136	V$OCT1_02	0.804548	9197	(-)	NNGAATATKCANNNN	CATTTAAATATTGTG
M00162	V$OCT1_06	0.839453	9203	(+)	CWNAWTKWSATRYN	AATATTGTGTTTTA
M00162	V$OCT1_06	0.808594	9244	(-)	CWNAWTKWSATRYN	GGTATTTAAAGGGT
M00138	V$OCT1_04	0.780217	9347	(-)	NNNNNNNWATGCAAATNNNWNNW	GCC-TATAACTTAAATTATAAAAT
M00162	V$OCT1_06	0.826953	9351	(+)	CWNAWTKWSATRYN	ATAACTTAAATTAT
M00162	V$OCT1_06	0.839844	9357	(-)	CWNAWTKWSATRYN	TAAATTATAAAATG
M00138	V$OCT1_04	0.820577	9360	(+)	NNNNNNNWATGCAAATNNNWNNW	ATTATAAAATGACAA-TATAATAA
M00162	V$OCT1_06	0.88125	9365	(-)	CWNAWTKWSATRYN	AAAATGACAATATA
M00248	V$OCT1_07	0.834429	9367	(-)	TNTATGNTAATT	AATGACAATATA
M00138	V$OCT1_04	0.823505	9369	(-)	NNNNNNNWATGCAAATNNNWNNW	TGACAATATAATAATAAA-TATTA
M00138	V$OCT1_04	0.793392	9371	(+)	NNNNNNNWATGCAAATNNNWNNW	ACAATATAATAATAAA-TATTAAA
M00137	V$OCT1_03	0.88305	9373	(+)	NNNRTAATNANNN	AATATAATAATAA
M00138	V$OCT1_04	0.883103	9374	(+)	NNNNNNNWATGCAAATNNNWNNW	ATATAATAATAAA-TATTAAATGA
M00137	V$OCT1_03	0.868036	9376	(+)	NNNRTAATNANNN	ATAATAATAAATA
M00136	V$OCT1_02	0.818896	9379	(-)	NNGAATATKCANNNN	ATAATAAATATTAAA
M00136	V$OCT1_02	0.792907	9382	(+)	NNGAATATKCANNNN	ATAAATATTAAATGA

M00162	V$OCT1_06	0.85	9384	(+)	CWNAWTKWSATRYN	AAATATTAAATGAT
M00162	V$OCT1_06	0.9375	9385	(-)	CWNAWTKWSATRYN	AATATTAAATGATG
M00138	V$OCT1_04	0.819741	9388	(+)	NNNNNNNWATGCAAATNNNWNNW	ATTAAATGATGTAAA-GCAATTAA
M00195	V$OCT1_Q6	0.818777	9392	(+)	NNNNATGCAAATNAN	AATGATGTAAAGCAA
M00162	V$OCT1_06	0.816016	9401	(+)	CWNAWTKWSATRYN	AAGCAATTAATGCA
M00195	V$OCT1_Q6	0.796124	9406	(+)	NNNNATGCAAATNAN	ATTAATGCAATCCAT
M00162	V$OCT1_06	0.81875	9452	(-)	CWNAWTKWSATRYN	AGTATCAGAAACAT
M00162	V$OCT1_06	0.852734	9482	(+)	CWNAWTKWSATRYN	CTTCATCTAATATT
M00162	V$OCT1_06	0.862891	9483	(-)	CWNAWTKWSATRYN	TTCATCTAATATTC
M00136	V$OCT1_02	0.77098	9484	(-)	NNGAATATKCANNNN	TCATCTAATATTCTG
M00135	V$OCT1_01	0.768409	9487	(+)	NNNNWTATGCAAATNTNNN	TCTAATATTCTGATTTCAA
M00136	V$OCT1_02	0.811857	9487	(+)	NNGAATATKCANNNN	TCTAATATTCTGATT
M00162	V$OCT1_06	0.839844	9490	(+)	CWNAWTKWSATRYN	AATATTCTGATTTC
M00248	V$OCT1_07	0.819165	9490	(+)	TNTATGNTAATT	AATATTCTGATT
M00138	V$OCT1_04	0.794437	9492	(-)	NNNNNNNWATGCAAATNNNWNNW	TATTCTGATTTCAATT-GGCCAAA
M00162	V$OCT1_06	0.825391	9511	(+)	CWNAWTKWSATRYN	CAAAGTTAAATCCT
M00162	V$OCT1_06	0.821484	9543	(-)	CWNAWTKWSATRYN	CATCTTCAATTTTC
M00136	V$OCT1_02	0.772875	9544	(-)	NNGAATATKCANNNN	ATCTTCAATTTTCTT
M00162	V$OCT1_06	0.808594	9568	(+)	CWNAWTKWSATRYN	GACACTGAAACACC
M00135	V$OCT1_01	0.754292	9578	(+)	NNNNWTATGCAAATNTNNN	CACCATAAGTAAAATTGCC
M00162	V$OCT1_06	0.855078	9581	(-)	CWNAWTKWSATRYN	CATAAGTAAAATTG
M00136	V$OCT1_02	0.87033	9593	(-)	NNGAATATKCANNNN	TGCCTGAATATCATT
M00162	V$OCT1_06	0.868359	9597	(-)	CWNAWTKWSATRYN	TGAATATCATTAAT
M00137	V$OCT1_03	0.876729	9600	(-)	NNNRTAATNANNN	ATATCATTAATAT
M00162	V$OCT1_06	0.826953	9618	(+)	CWNAWTKWSATRYN	TTCACTGTAATTTA
M00138	V$OCT1_04	0.786282	9625	(-)	NNNNNNNWATGCAAATNNNWNNW	TAATTTATTTTAAGTTAACAGTA
M00248	V$OCT1_07	0.831673	9628	(+)	TNTATGNTAATT	TTTATTTTAAGT
M00138	V$OCT1_04	0.794019	9656	(+)	NNNNNNNWATGCAAATNNNWNNW	AAGTGTTTTTTTTAATTTTTAAA
M00138	V$OCT1_04	0.825387	9657	(-)	NNNNNNNWATGCAAATNNNWNNW	AG-TGTTTTTTTTAATTTTTAAAA
M00162	V$OCT1_06	0.826562	9661	(+)	CWNAWTKWSATRYN	TTTTTTTTAATTTT
M00248	V$OCT1_07	0.831673	9661	(+)	TNTATGNTAATT	TTTTTTTTAATT
M00138	V$OCT1_04	0.800084	9662	(+)	NNNNNNNWATGCAAATNNNWNNW	TTTTTTTAATTTTTAAAAAA-TAA
M00138	V$OCT1_04	0.784818	9663	(-)	NNNNNNNWATGCAAATNNNWNNW	TTTTTTAATTTTTAAAAAA-TAAT

M00138	V$OCT1_04	0.80573	9664	(-)	NNNNNNNNWATGCAAATNNNWNNW	TTTTTAATTTTTAAAAAA-TAATT
M00138	V$OCT1_04	0.797156	9665	(+)	NNNNNNNNWATGCAAATNNNWNNW	TTTTAATTTTTAAAAAA-TAATTA
M00138	V$OCT1_04	0.805939	9665	(-)	NNNNNNNNWATGCAAATNNNWNNW	TTTTAATTTTTAAAAAA-TAATTA
M00162	V$OCT1_06	0.813281	9669	(-)	CWNAWTKWSATRYN	AATTTTTAAAAAAT
M00137	V$OCT1_03	0.899249	9678	(+)	NNNRTAATNANNN	AAAATAATTAGAT
M00137	V$OCT1_03	0.845516	9679	(-)	NNNRTAATNANNN	AAATAATTAGATT
M00162	V$OCT1_06	0.855469	9680	(+)	CWNAWTKWSATRYN	AATAATTAGATTAG
M00248	V$OCT1_07	0.824041	9683	(-)	TNTATGNTAATT	AATTAGATTAGT
M00248	V$OCT1_07	0.795209	9713	(-)	TNTATGNTAATT	AAATAACATCAA
M00162	V$OCT1_06	0.942969	9717	(-)	CWNAWTKWSATRYN	AACATCAAAGTGAG
M00162	V$OCT1_06	0.817578	9722	(+)	CWNAWTKWSATRYN	CAAAGTGAGATTAA
M00137	V$OCT1_03	0.860529	9782	(-)	NNNRTAATNANNN	GCTTTATTACTTG
M00136	V$OCT1_02	0.790471	9789	(-)	NNGAATATKCANNNN	TACTTGAAAATGCTG
M00162	V$OCT1_06	0.834375	9789	(-)	CWNAWTKWSATRYN	TACTTGAAAATGCT
M00136	V$OCT1_02	0.789117	9792	(+)	NNGAATATKCANNNN	TTGAAAATGCTGGGA
M00162	V$OCT1_06	0.800781	9803	(+)	CWNAWTKWSATRYN	GGGATTGGACTGCA
M00162	V$OCT1_06	0.834375	9818	(-)	CWNAWTKWSATRYN	GGTATCTAAGCAAC
M00162	V$OCT1_06	0.800391	9837	(+)	CWNAWTKWSATRYN	ATATTTTTCTTTTC
M00162	V$OCT1_06	0.834375	9861	(-)	CWNAWTKWSATRYN	AGCTTCACAGACTG
M00195	V$OCT1_Q6	0.820961	9876	(+)	NNNNATGCAAATNAN	ACAAATACAAACTAC
M00162	V$OCT1_06	0.826562	9893	(+)	CWNAWTKWSATRYN	CAGCTGGTAATATT
M00137	V$OCT1_03	0.873568	9896	(+)	NNNRTAATNANNN	CTGGTAATATTTT
M00138	V$OCT1_04	0.912798	9897	(-)	NNNNNNNNWATGCAAATNNNWNNW	TGGTAATATTTTCATTATAA-TAA
M00135	V$OCT1_01	0.785578	9899	(-)	NNNNWTATGCAAATNTNNN	GTAATATTTTCATTATAAT
M00162	V$OCT1_06	0.860547	9901	(+)	CWNAWTKWSATRYN	AATATTTTCATTAT
M00248	V$OCT1_07	0.812381	9901	(+)	TNTATGNTAATT	AATATTTTCATT
M00138	V$OCT1_04	0.828733	9902	(+)	NNNNNNNNWATGCAAATNNNWNNW	ATATTTTCATTATAA-TAATACAA
M00138	V$OCT1_04	0.82936	9903	(-)	NNNNNNNNWATGCAAATNNNWNNW	TATTTTCATTATAA-TAATACAAA
M00137	V$OCT1_03	0.911102	9905	(-)	NNNRTAATNANNN	TTTTCATTATAAT
M00138	V$OCT1_04	0.790255	9905	(+)	NNNNNNNNWATGCAAATNNNWNNW	TTTTCATTATAA-TAATACAAAAT
M00138	V$OCT1_04	0.783145	9906	(-)	NNNNNNNNWATGCAAATNNNWNNW	TTTCATTATAA-TAATACAAAATT
M00135	V$OCT1_01	0.744945	9907	(+)	NNNNWTATGCAAATNTNNN	TTCATTATAATAATACAAA
M00137	V$OCT1_03	0.874358	9910	(+)	NNNRTAATNANNN	ATTATAATAATAC

M00138	V$OCT1_04	0.854245	9911	(+)	NNNNNNNNWATGCAAATNNNWNNW	TTATAATAATACAAAATT-GTATT
M00135	V$OCT1_01	0.768791	9913	(+)	NNNNWTATGCAAATNTNNN	ATAATAATACAAAATTGTA
M00162	V$OCT1_06	0.813281	9915	(-)	CWNAWTKWSATRYN	AATAATACAAAATT
M00162	V$OCT1_06	0.881641	9922	(+)	CWNAWTKWSATRYN	CAAAATTGTATTCT
M00138	V$OCT1_04	0.792765	9925	(-)	NNNNNNNNWATGCAAATNNNWNNW	AATTGTATTCTG-CAGAATTAAAG
M00138	V$OCT1_04	0.829987	9926	(+)	NNNNNNNNWATGCAAATNNNWNNW	ATTGTATTCTG-CAGAATTAAAGT
M00162	V$OCT1_06	0.826953	9938	(-)	CWNAWTKWSATRYN	AGAATTAAAGTAGT
M00248	V$OCT1_07	0.817257	9940	(-)	TNTATGNTAATT	AATTAAAGTAGT
M00138	V$OCT1_04	0.818068	9941	(+)	NNNNNNNNWATGCAAATNNNWNNW	ATTAAAGTAG-TTAAATTTTAAAT
M00162	V$OCT1_06	0.834766	9946	(+)	CWNAWTKWSATRYN	AGTAGTTAAATTTT
M00162	V$OCT1_06	0.847266	9946	(-)	CWNAWTKWSATRYN	AGTAGTTAAATTTT
M00248	V$OCT1_07	0.793301	9946	(+)	TNTATGNTAATT	AGTAGTTAAATT
M00162	V$OCT1_06	0.829297	9953	(+)	CWNAWTKWSATRYN	AAATTTTAAATGGG
M00138	V$OCT1_04	0.804266	9966	(-)	NNNNNNNNWATGCAAATNNNWNNW	GGTTTGCTTTTAAA-TATGTTTTT
M00162	V$OCT1_06	0.808594	9970	(+)	CWNAWTKWSATRYN	TGCTTTTAAATATG
M00162	V$OCT1_06	0.88125	9970	(-)	CWNAWTKWSATRYN	TGCTTTTAAATATG
M00138	V$OCT1_04	0.828524	10010	(+)	NNNNNNNNWATGCAAATNNNWNNW	CCTTGATCATGAATAA-TAATTGG
M00136	V$OCT1_02	0.886844	10015	(-)	NNGAATATKCANNNN	ATCATGAATAATAAT
M00137	V$OCT1_03	0.871987	10019	(+)	NNNRTAATNANNN	TGAATAATAATTG
M00136	V$OCT1_02	0.774499	10036	(-)	NNGAATATKCANNNN	ATGCTCCATATGCTT
M00135	V$OCT1_01	0.743037	10039	(+)	NNNNWTATGCAAATNTNNN	CTCCATATGCTTCTCTATG
M00136	V$OCT1_02	0.791012	10039	(+)	NNGAATATKCANNNN	CTCCATATGCTTCTC
M00136	V$OCT1_02	0.772604	10049	(+)	NNGAATATKCANNNN	TTCTCTATGCACTTA
M00137	V$OCT1_03	0.877519	10081	(-)	NNNRTAATNANNN	TTCTATTTATGCT
M00135	V$OCT1_01	0.746089	10083	(+)	NNNNWTATGCAAATNTNNN	CTATTTATGCTGAGACTAA
M00138	V$OCT1_04	0.785027	10106	(-)	NNNNNNNNWATGCAAATNNNWNNW	CTGTATCACATCCA-TAATTACTA
M00162	V$OCT1_06	0.847266	10107	(-)	CWNAWTKWSATRYN	TGTATCACATCCAT
M00137	V$OCT1_03	0.896484	10116	(+)	NNNRTAATNANNN	TCCATAATTACTA
M00137	V$OCT1_03	0.894508	10117	(-)	NNNRTAATNANNN	CCATAATTACTAT
M00248	V$OCT1_07	0.803901	10121	(-)	TNTATGNTAATT	AATTACTATCCA
M00138	V$OCT1_04	0.786491	10138	(-)	NNNNNNNNWATGCAAATNNNWNNW	AAGTTTACTTTAACTAAATTCTA
M00162	V$OCT1_06	0.862891	10159	(-)	CWNAWTKWSATRYN	TACATCAAATCCTC
M00138	V$OCT1_04	0.792765	10190	(-)	NNNNNNNNWATGCAAATNNNWNNW	ATATATTATATTCAG-GAATTTAT

M00162	V$OCT1_06	0.842187	10190	(+)	CWNAWTKWSATRYN	ATATATTATATTCA
M00162	V$OCT1_06	0.866016	10191	(-)	CWNAWTKWSATRYN	TATATTATATTCAG
M00136	V$OCT1_02	0.819166	10193	(+)	NNGAATATKCANNNN	TATTATATTCAGGAA
M00138	V$OCT1_04	0.786491	10220	(-)	NNNNNNNWATGCAAATNNNWNNW	TGTCTTTATTTTCCCAAATTCAC
M00136	V$OCT1_02	0.794532	10260	(+)	NNGAATATKCANNNN	ATAAAAATGCTTGCT
M00138	V$OCT1_04	0.852363	10282	(+)	NNNNNNNWATGCAAATNNNWNNW	GCACAGATATGCCAAA-TAAACTT
M00135	V$OCT1_01	0.768791	10284	(+)	NNNNWTATGCAAATNTNNN	ACAGATATGCCAAATAAAC
M00136	V$OCT1_02	0.787223	10284	(+)	NNGAATATKCANNNN	ACAGATATGCCAAAT
M00195	V$OCT1_Q6	0.800491	10286	(+)	NNNNATGCAAATNAN	AGATATGCCAAATAA
M00162	V$OCT1_06	0.834375	10287	(-)	CWNAWTKWSATRYN	GATATGCCAAATAA
M00162	V$OCT1_06	0.842187	10299	(+)	CWNAWTKWSATRYN	AAACTTTCCATTTA
M00248	V$OCT1_07	0.815137	10301	(-)	TNTATGNTAATT	ACTTTCCATTTA
M00162	V$OCT1_06	0.808203	10346	(+)	CWNAWTKWSATRYN	GTCCTAGACATTCA
M00138	V$OCT1_04	0.784191	10347	(+)	NNNNNNNWATGCAAATNNNWNNW	TCCTAGACATTCAAATT-GCTTTT
M00135	V$OCT1_01	0.775849	10349	(+)	NNNNWTATGCAAATNTNNN	CTAGACATTCAAATTGCTT
M00195	V$OCT1_Q6	0.842522	10351	(+)	NNNNATGCAAATNAN	AGACATTCAAATTGC
M00162	V$OCT1_06	0.834766	10352	(-)	CWNAWTKWSATRYN	GACATTCAAATTGC
M00248	V$OCT1_07	0.825313	10352	(+)	TNTATGNTAATT	GACATTCAAATT
M00136	V$OCT1_02	0.785057	10370	(+)	NNGAATATKCANNNN	GTCAAAATTCTCTAA
M00162	V$OCT1_06	0.800781	10388	(-)	CWNAWTKWSATRYN	AGAATCAGAGAGTT
M00136	V$OCT1_02	0.773958	10397	(-)	NNGAATATKCANNNN	GAGTTGATTAATCTT
M00162	V$OCT1_06	0.829297	10432	(+)	CWNAWTKWSATRYN	ATGTATGAAATGGA
M00138	V$OCT1_04	0.79862	10433	(+)	NNNNNNNWATGCAAATNNNWNNW	TGTATGAAATGGAAA-TATAGTTA
M00162	V$OCT1_06	0.855078	10433	(-)	CWNAWTKWSATRYN	TGTATGAAATGGAA
M00135	V$OCT1_01	0.776803	10435	(+)	NNNNWTATGCAAATNTNNN	TATGAAATGGAAATATAGT
M00162	V$OCT1_06	0.847266	10438	(+)	CWNAWTKWSATRYN	GAAATGGAAATATA
M00195	V$OCT1_Q6	0.80595	10452	(-)	NNNNATGCAAATNAN	GTTATTCTCACATCC
M00135	V$OCT1_01	0.782335	10470	(-)	NNNNWTATGCAAATNTNNN	TGTTATTTTGAATACTGAA
M00162	V$OCT1_06	0.826562	10472	(+)	CWNAWTKWSATRYN	TTATTTTGAATACT
M00136	V$OCT1_02	0.831348	10474	(-)	NNGAATATKCANNNN	ATTTTGAATACTGAA
M00248	V$OCT1_07	0.800085	10474	(-)	TNTATGNTAATT	ATTTTGAATACT
M00162	V$OCT1_06	0.834375	10541	(+)	CWNAWTKWSATRYN	AAAATGTTCATTCC
M00248	V$OCT1_07	0.817045	10541	(+)	TNTATGNTAATT	AAAATGTTCATT

M00135	V$OCT1_01	0.739794	10577	(+)	NNNNWTATGCAAATNTNNN	CAGTCTCTGTAAATTTTGC
M00248	V$OCT1_07	0.871317	10580	(+)	TNTATGNTAATT	TCTCTGTAAATT
M00137	V$OCT1_03	0.922165	10615	(-)	NNNRTAATNANNN	ACAACATTATGAA
M00162	V$OCT1_06	0.816406	10639	(+)	CWNAWTKWSATRYN	AAGAAGTGCATTTG
M00136	V$OCT1_02	0.792907	10641	(-)	NNGAATATKCANNNN	GAAGTGCATTTGAAC
M00162	V$OCT1_06	0.809375	10703	(-)	CWNAWTKWSATRYN	AATGTATCAATTAG
M00138	V$OCT1_04	0.840443	10705	(-)	NNNNNNNWATGCAAATNNNWNNW	TGTATCAATTAG-CACAACCCTTC
M00135	V$OCT1_01	0.797406	10707	(-)	NNNNWTATGCAAATNTNNN	TATCAATTAGCACAACCCT
M00248	V$OCT1_07	0.864109	10711	(-)	TNTATGNTAATT	AATTAGCACAAC
M00248	V$OCT1_07	0.804113	10726	(+)	TNTATGNTAATT	TCTTTGCTACTT
M00162	V$OCT1_06	0.823828	10752	(-)	CWNAWTKWSATRYN	TGACTGTAATAGTC
M00162	V$OCT1_06	0.813281	10787	(+)	CWNAWTKWSATRYN	ATTTTTTTCTTTTA
M00138	V$OCT1_04	0.798411	10796	(+)	NNNNNNNWATGCAAATNNNWNNW	TTTTACTTATAGCAATTAAACTT
M00137	V$OCT1_03	0.853418	10819	(+)	NNNRTAATNANNN	GACTTAATTAAGA
M00137	V$OCT1_03	0.881075	10820	(-)	NNNRTAATNANNN	ACTTAATTAAGAC
M00138	V$OCT1_04	0.793183	10850	(-)	NNNNNNNWATGCAAATNNNWNNW	GCCTTAGTTTAGCAAAATTA-TAT
M00135	V$OCT1_01	0.789012	10852	(-)	NNNNWTATGCAAATNTNNN	CTTAGTTTAGCAAAATTAT
M00162	V$OCT1_06	0.902344	10862	(+)	CWNAWTKWSATRYN	CAAAATTATATTTT
M00162	V$OCT1_06	0.847656	10863	(-)	CWNAWTKWSATRYN	AAAATTATATTTTC
M00138	V$OCT1_04	0.7844	10864	(-)	NNNNNNNWATGCAAATNNNWNNW	AAATTATATTTTCACATTTT-GTT
M00135	V$OCT1_01	0.772415	10866	(-)	NNNNWTATGCAAATNTNNN	ATTATATTTTCACATTTTG
M00162	V$OCT1_06	0.803906	10868	(+)	CWNAWTKWSATRYN	TATATTTTCACATT
M00162	V$OCT1_06	0.809375	10871	(-)	CWNAWTKWSATRYN	ATTTTCACATTTTG
M00162	V$OCT1_06	0.821484	10903	(-)	CWNAWTKWSATRYN	AGAAGTCAATGATG
M00136	V$OCT1_02	0.777477	10911	(-)	NNGAATATKCANNNN	ATGATGAATTTAATT
M00162	V$OCT1_06	0.891016	10914	(+)	CWNAWTKWSATRYN	ATGAATTTAATTTG
M00162	V$OCT1_06	0.881641	10915	(-)	CWNAWTKWSATRYN	TGAATTTAATTTGG
M00248	V$OCT1_07	0.820013	10978	(+)	TNTATGNTAATT	TCCAAGGAAATT
M00138	V$OCT1_04	0.812422	10979	(+)	NNNNNNNWATGCAAATNNNWNNW	CCAAGGAAATTTAAATATATCTG
M00138	V$OCT1_04	0.811794	10980	(-)	NNNNNNNWATGCAAATNNNWNNW	CAAGGAAATTTAAATATATCTGT
M00135	V$OCT1_01	0.819916	10982	(-)	NNNNWTATGCAAATNTNNN	AGGAAATTTAAATATATCT
M00162	V$OCT1_06	0.9375	10984	(+)	CWNAWTKWSATRYN	GAAATTTAAATATA
M00162	V$OCT1_06	0.855469	10984	(-)	CWNAWTKWSATRYN	GAAATTTAAATATA

M00195	V$OCT1_Q6	0.792849	10984	(-)	NNNNATGCAAATNAN	GAAATTTAAATATAT
M00162	V$OCT1_06	0.800391	10985	(-)	CWNAWTKWSATRYN	AAATTTAAATATAT
M00248	V$OCT1_07	0.90566	10986	(-)	TNTATGNTAATT	AATTTAAATATA
M00138	V$OCT1_04	0.801966	11033	(-)	NNNNNNNWATGCAAATNNNWNNW	AGAAACTACTTGAA-TAATCTATT
M00135	V$OCT1_01	0.75124	11035	(-)	NNNNWTATGCAAATNTNNN	AAACTACTTGAATAATCTA
M00136	V$OCT1_02	0.897672	11039	(-)	NNGAATATKCANNNN	TACTTGAATAATCTA
M00162	V$OCT1_06	0.826953	11050	(-)	CWNAWTKWSATRYN	TCTATTTCAGTACC
M00162	V$OCT1_06	0.808203	11059	(+)	CWNAWTKWSATRYN	GTACCTTAGCTGTG
M00162	V$OCT1_06	0.825391	11124	(+)	CWNAWTKWSATRYN	CAGAATGTCATCAT
M00162	V$OCT1_06	0.814453	11125	(-)	CWNAWTKWSATRYN	AGAATGTCATCATA
M00162	V$OCT1_06	0.803125	11135	(-)	CWNAWTKWSATRYN	CATATTTCGTAGTT
M00135	V$OCT1_01	0.750477	11155	(-)	NNNNWTATGCAAATNTNNN	CAGGATTTAGCTTAGTCCT
M00162	V$OCT1_06	0.821484	11178	(-)	CWNAWTKWSATRYN	TGAATGAAAGCACC
M00162	V$OCT1_06	0.834375	11195	(-)	CWNAWTKWSATRYN	TGAATGTCTGTTAT
M00162	V$OCT1_06	0.800781	11198	(+)	CWNAWTKWSATRYN	ATGTCTGTTATTCA
M00162	V$OCT1_06	0.804297	11199	(-)	CWNAWTKWSATRYN	TGTCTGTTATTCAG
M00059	V$YY1_01	0.805046	55	(-)	NNNNNCCATNTWNNNWN	GTTTACATATGGCAAAA
M00059	V$YY1_01	0.821756	163	(-)	NNNNNCCATNTWNNNWN	GCTTAAAAATGCATTTG
M00059	V$YY1_01	0.809633	175	(-)	NNNNNCCATNTWNNNWN	ATTTGCAAATGTTGATA
M00059	V$YY1_01	0.796855	215	(+)	NNNNNCCATNTWNNNWN	AAAAATCATATAGTTTT
M00059	V$YY1_01	0.816514	401	(+)	NNNNNCCATNTWNNNWN	TAAGAGCATTTCTGGTA
M00059	V$YY1_01	0.89384	516	(-)	NNNNNCCATNTWNNNWN	TTGTAAAGATGGTTATA
M00069	V$YY1_02	0.764727	647	(-)	NNNCGGCCATCTTGNCTSNW	TATATAAAATATGGCGGTAT
M00059	V$YY1_01	0.895478	649	(-)	NNNNNCCATNTWNNNWN	TATAAAATATGGCGGTA
M00069	V$YY1_02	0.75116	715	(+)	NNNCGGCCATCTTGNCTSNW	TGGAAACCATTTTTCTAGCA
M00059	V$YY1_01	0.909895	716	(+)	NNNNNCCATNTWNNNWN	GGAAACCATTTTTCTAG
M00059	V$YY1_01	0.823722	785	(-)	NNNNNCCATNTWNNNWN	AGTCAAACATGATATGA
M00059	V$YY1_01	0.779489	834	(+)	NNNNNCCATNTWNNNWN	CTTCCTCATATAGTCAT
M00059	V$YY1_01	0.780472	846	(-)	NNNNNCCATNTWNNNWN	GTCATGTCATGGATATA
M00059	V$YY1_01	0.775229	961	(-)	NNNNNCCATNTWNNNWN	GACTCTGAATGAGCAGG
M00059	V$YY1_01	0.794233	1002	(+)	NNNNNCCATNTWNNNWN	GAAAGACATTTTTTTTA
M00059	V$YY1_01	0.800459	1029	(-)	NNNNNCCATNTWNNNWN	TAAATTAAATGGGTTCA
M00059	V$YY1_01	0.79325	1060	(+)	NNNNNCCATNTWNNNWN	ACTATACATTTTAACAA

M00059	V$YY1_01	0.858126	1138	(-)	NNNNNCCATNTWNNNWN	ATTTAAATATGAATGCC
M00059	V$YY1_01	0.815203	1170	(+)	NNNNNCCATNTWNNNWN	CAAAACCCTATTACCAC
M00059	V$YY1_01	0.796855	1183	(+)	NNNNNCCATNTWNNNWN	CCACTTCATGGATCAAA
M00059	V$YY1_01	0.809633	1290	(+)	NNNNNCCATNTWNNNWN	GATTAACATTTATATTG
M00059	V$YY1_01	0.788008	1472	(+)	NNNNNCCATNTWNNNWN	GACCACCATGAAGCTTC
M00059	V$YY1_01	0.788663	1658	(-)	NNNNNCCATNTWNNNWN	TTAAAACTATGATAATC
M00059	V$YY1_01	0.81291	1829	(-)	NNNNNCCATNTWNNNWN	ATCAAAGAATGACATGA
M00059	V$YY1_01	0.837811	2239	(-)	NNNNNCCATNTWNNNWN	ATAAAATAATGGTCTTG
M00059	V$YY1_01	0.813893	2294	(-)	NNNNNCCATNTWNNNWN	TTTCTGAAATGCTGAGA
M00059	V$YY1_01	0.922346	2334	(+)	NNNNNCCATNTWNNNWN	TATATCCATATTTAGAA
M00059	V$YY1_01	0.771625	2344	(-)	NNNNNCCATNTWNNNWN	TTTAGAAAATGCAGGAT
M00059	V$YY1_01	0.801769	2509	(-)	NNNNNCCATNTWNNNWN	TTTTACAAATGAAATGG
M00059	V$YY1_01	0.792923	2514	(-)	NNNNNCCATNTWNNNWN	CAAATGAAATGGGTATA
M00059	V$YY1_01	0.892202	2579	(+)	NNNNNCCATNTWNNNWN	CCACATCATTTTTAATG
M00059	V$YY1_01	0.859764	2628	(-)	NNNNNCCATNTWNNNWN	CAGTTAAAATGAAGATT
M00059	V$YY1_01	0.790301	2694	(+)	NNNNNCCATNTWNNNWN	AAGCAACATTTAATTAG
M00059	V$YY1_01	0.807995	2728	(-)	NNNNNCCATNTWNNNWN	CTCCAAAAATGTATAAT
M00059	V$YY1_01	0.815531	2764	(+)	NNNNNCCATNTWNNNWN	AACTACCATGTAATATA
M00059	V$YY1_01	0.789646	2888	(+)	NNNNNCCATNTWNNNWN	CTCCACCATACTCTAAA
M00059	V$YY1_01	0.791612	3264	(+)	NNNNNCCATNTWNNNWN	TAAAGTCAGTTTCACAT
M00059	V$YY1_01	0.871232	3337	(+)	NNNNNCCATNTWNNNWN	ACAAACCATCTTAGAAA
M00059	V$YY1_01	0.795544	3531	(+)	NNNNNCCATNTWNNNWN	CCACACCCTTCTACAAC
M00059	V$YY1_01	0.799803	3545	(+)	NNNNNCCATNTWNNNWN	AACAGCCATTAATCCAC
M00059	V$YY1_01	0.799803	3565	(-)	NNNNNCCATNTWNNNWN	GTCCTCTAATGGAATTA
M00059	V$YY1_01	0.831258	3703	(+)	NNNNNCCATNTWNNNWN	TCATTTCATTTATTTTC
M00059	V$YY1_01	0.791612	3727	(+)	NNNNNCCATNTWNNNWN	TGTATCCAGGTACACAA
M00059	V$YY1_01	0.778178	3780	(+)	NNNNNCCATNTWNNNWN	TAAATTCCTATTCAATC
M00059	V$YY1_01	0.77097	3791	(+)	NNNNNCCATNTWNNNWN	TCAATCCCTCCTTTGAC
M00059	V$YY1_01	0.876802	3852	(-)	NNNNNCCATNTWNNNWN	AACCAAATATGGTACCT
M00059	V$YY1_01	0.778506	3961	(-)	NNNNNCCATNTWNNNWN	TTGTTCAAATGGAAAAC
M00059	V$YY1_01	0.820446	3989	(+)	NNNNNCCATNTWNNNWN	GGGGTGCATTTTTCCTT
M00059	V$YY1_01	0.854194	4093	(-)	NNNNNCCATNTWNNNWN	CTTCTAAAATGACAGCC
M00059	V$YY1_01	0.780144	4138	(-)	NNNNNCCATNTWNNNWN	GTTTGCAAATGTTTTTT

M00059	V$YY1_01	0.784731	4272	(+)	NNNNNCCATNTWNNNWN	AACACTCATGTTTTCAT
M00059	V$YY1_01	0.825033	4314	(+)	NNNNNCCATNTWNNNWN	AATTTCCATGTTCATTA
M00059	V$YY1_01	0.780144	4472	(-)	NNNNNCCATNTWNNNWN	TTGTCTGAATGATTTTT
M00059	V$YY1_01	0.855177	4570	(-)	NNNNNCCATNTWNNNWN	GTTAACAAATGGAAGCA
M00059	V$YY1_01	0.775229	4595	(+)	NNNNNCCATNTWNNNWN	TCTCTCCAGTAAGTATT
M00059	V$YY1_01	0.783093	4636	(-)	NNNNNCCATNTWNNNWN	GCCTTTTTATGGAAATA
M00059	V$YY1_01	0.77097	4770	(+)	NNNNNCCATNTWNNNWN	AGTGCACATTTACATAC
M00059	V$YY1_01	0.840433	4871	(+)	NNNNNCCATNTWNNNWN	CACATTCATCTTTTAAT
M00059	V$YY1_01	0.794561	4921	(+)	NNNNNCCATNTWNNNWN	GATATCCATTCATTTGT
M00059	V$YY1_01	0.792267	4935	(+)	NNNNNCCATNTWNNNWN	TGTGTCCCTCTTTACTA
M00059	V$YY1_01	0.800786	4980	(+)	NNNNNCCATNTWNNNWN	GGAGAACATCGATGAAC
M00059	V$YY1_01	0.774246	4983	(-)	NNNNNCCATNTWNNNWN	GAACATCGATGAACTGA
M00069	V$YY1_02	0.778472	5003	(+)	NNNCGGCCATCTTGNCTSNW	TAAGGACCATCAATGATATT
M00059	V$YY1_01	0.786697	5004	(+)	NNNNNCCATNTWNNNWN	AAGGACCATCAATGATA
M00059	V$YY1_01	0.81422	5007	(-)	NNNNNCCATNTWNNNWN	GACCATCAATGATATTT
M00059	V$YY1_01	0.844037	5109	(-)	NNNNNCCATNTWNNNWN	AACACAAAATGATGAAC
M00059	V$YY1_01	0.818152	5218	(+)	NNNNNCCATNTWNNNWN	TTACTTCATTTATACTT
M00069	V$YY1_02	0.835059	5241	(-)	NNNCGGCCATCTTGNCTSNW	AACTTTCAAAATGGCTGATG
M00059	V$YY1_01	0.833879	5243	(-)	NNNNNCCATNTWNNNWN	CTTTCAAAATGGCTGAT
M00059	V$YY1_01	0.835518	5337	(-)	NNNNNCCATNTWNNNWN	ATGAATAAATGGATGAA
M00059	V$YY1_01	0.837156	5357	(-)	NNNNNCCATNTWNNNWN	ATGAATAAATGCAATTA
M00059	V$YY1_01	0.886632	5488	(-)	NNNNNCCATNTWNNNWN	CTTGAAAAATGATTTTT
M00059	V$YY1_01	0.770315	5502	(-)	NNNNNCCATNTWNNNWN	TTTTGGATCTGGATTCT
M00059	V$YY1_01	0.802097	5607	(-)	NNNNNCCATNTWNNNWN	ATACCTGAATGATTATC
M00059	V$YY1_01	0.772608	5671	(+)	NNNNNCCATNTWNNNWN	TAAGATTATTTTTAAAC
M00059	V$YY1_01	0.787353	5738	(-)	NNNNNCCATNTWNNNWN	TGCCCAAAATGAATAAA
M00059	V$YY1_01	0.801114	5903	(-)	NNNNNCCATNTWNNNWN	CATGAAAACTGAACGCA
M00059	V$YY1_01	0.794561	5920	(+)	NNNNNCCATNTWNNNWN	ACACACCAGTTGGAAAA
M00059	V$YY1_01	0.858453	5936	(+)	NNNNNCCATNTWNNNWN	AAAATCCATTTTGCTTA
M00059	V$YY1_01	0.846003	6096	(+)	NNNNNCCATNTWNNNWN	TCACAACATATAAGAAT
M00059	V$YY1_01	0.770642	6132	(-)	NNNNNCCATNTWNNNWN	ACTCTGAAATGCAGATT
M00059	V$YY1_01	0.785059	6476	(-)	NNNNNCCATNTWNNNWN	AATGTACAATGCTGTGC
M00059	V$YY1_01	0.77654	6525	(-)	NNNNNCCATNTWNNNWN	TCATTTAAATGATAATC

M00059	V$YY1_01	0.778506	6624	(-)	NNNNNCCATNTWNNNWN	GAGGATTTATGAAGGTA
M00059	V$YY1_01	0.795872	6703	(-)	NNNNNCCATNTWNNNWN	TTGGCAAGCTGGTTACT
M00059	V$YY1_01	0.837811	6730	(-)	NNNNNCCATNTWNNNWN	AAACATAAATGGGCGGA
M00059	V$YY1_01	0.77654	6913	(-)	NNNNNCCATNTWNNNWN	GTGAACAAAGGGTTACA
M00059	V$YY1_01	0.773591	7063	(-)	NNNNNCCATNTWNNNWN	TTCACAAGATGTATGGT
M00059	V$YY1_01	0.807995	7067	(-)	NNNNNCCATNTWNNNWN	CAAGATGTATGGTTTCC
M00069	V$YY1_02	0.812924	7080	(+)	NNNCGGCCATCTTGNCTSNW	TTCCTGCCATTTAGCTGGTT
M00059	V$YY1_01	0.803408	7081	(+)	NNNNNCCATNTWNNNWN	TCCTGCCATTTAGCTGG
M00069	V$YY1_02	0.833809	7247	(+)	NNNCGGCCATCTTGNCTSNW	CCTCTGCCATCCAGGGTATG
M00059	V$YY1_01	0.821756	7361	(-)	NNNNNCCATNTWNNNWN	CTAAAAAGATGGGGAAA
M00059	V$YY1_01	0.83945	7453	(-)	NNNNNCCATNTWNNNWN	TTTTAAATATGTCCTTG
M00059	V$YY1_01	0.781782	7525	(+)	NNNNNCCATNTWNNNWN	AAACTCAATATTTCAAA
M00059	V$YY1_01	0.805374	7581	(-)	NNNNNCCATNTWNNNWN	CACTTAAACTGGTTGTT
M00059	V$YY1_01	0.782765	7652	(-)	NNNNNCCATNTWNNNWN	GAGAATGAATGCTCAGC
M00059	V$YY1_01	0.792595	7710	(+)	NNNNNCCATNTWNNNWN	CAGGTTCATTTTTATCA
M00059	V$YY1_01	0.781455	7735	(+)	NNNNNCCATNTWNNNWN	CATACACATTTTACATT
M00059	V$YY1_01	0.782438	7828	(+)	NNNNNCCATNTWNNNWN	CTCCAACATATTTTAAA
M00059	V$YY1_01	0.826016	7836	(-)	NNNNNCCATNTWNNNWN	TATTTTAAATGAAATTG
M00059	V$YY1_01	0.77097	7904	(-)	NNNNNCCATNTWNNNWN	TTCTGAACAAGGAGTTA
M00059	V$YY1_01	0.771298	8053	(+)	NNNNNCCATNTWNNNWN	TCAATTCATTATCCCAA
M00059	V$YY1_01	0.843381	8069	(-)	NNNNNCCATNTWNNNWN	ATCAGAAAATGTTTATG
M00059	V$YY1_01	0.799148	8093	(-)	NNNNNCCATNTWNNNWN	ATTGTACCATGAAGTTT
M00069	V$YY1_02	0.771689	8093	(+)	NNNCGGCCATCTTGNCTSNW	ATTGTACCATGAAGTTTACA
M00059	V$YY1_01	0.871232	8157	(+)	NNNNNCCATNTWNNNWN	TGTCCTCATTTTGACAC
M00059	V$YY1_01	0.776868	8168	(-)	NNNNNCCATNTWNNNWN	TGACACACATGGATATC
M00059	V$YY1_01	0.770642	8276	(+)	NNNNNCCATNTWNNNWN	CCCAAACATTTTTTTTT
M00059	V$YY1_01	0.813893	8307	(+)	NNNNNCCATNTWNNNWN	CCTTCCCAGATTTTGAT
M00059	V$YY1_01	0.856815	8380	(+)	NNNNNCCATNTWNNNWN	TATCAACATGTTTTATC
M00059	V$YY1_01	0.799803	8426	(-)	NNNNNCCATNTWNNNWN	ATCAGACACTGGAATCC
M00059	V$YY1_01	0.774246	8721	(+)	NNNNNCCATNTWNNNWN	TAAAGTCAGTTTATTAC
M00069	V$YY1_02	0.751874	8803	(-)	NNNCGGCCATCTTGNCTSNW	AAAAACTAACATGGCATGTT
M00059	V$YY1_01	0.81422	8805	(-)	NNNNNCCATNTWNNNWN	AAACTAACATGGCATGT
M00059	V$YY1_01	0.797182	8811	(+)	NNNNNCCATNTWNNNWN	ACATGGCATGTTTTAAT

M00059	V$YY1_01	0.784731	9118	(-)	NNNNNCCATNTWNNNWN	GACTCTAGATGTTTACT
M00059	V$YY1_01	0.794233	9191	(+)	NNNNNCCATNTWNNNWN	TGCTTTCATTTAAATAT
M00059	V$YY1_01	0.790629	9198	(-)	NNNNNCCATNTWNNNWN	ATTTAAATATTGTGTTT
M00059	V$YY1_01	0.782765	9245	(-)	NNNNNCCATNTWNNNWN	GTATTTAAAGGGTACTC
M00059	V$YY1_01	0.782438	9295	(+)	NNNNNCCATNTWNNNWN	GCTCTACCTTTTAGCAG
M00059	V$YY1_01	0.861075	9360	(-)	NNNNNCCATNTWNNNWN	ATTATAAAATGACAATA
M00059	V$YY1_01	0.809305	9385	(-)	NNNNNCCATNTWNNNWN	AATATTAAATGATGTAA
M00059	V$YY1_01	0.789646	9412	(+)	NNNNNCCATNTWNNNWN	GCAATCCATAGCACAAA
M00059	V$YY1_01	0.814875	9436	(-)	NNNNNCCATNTWNNNWN	CTCAGTAAATGTGGCTA
M00059	V$YY1_01	0.82405	9479	(+)	NNNNNCCATNTWNNNWN	CCACTTCATCTAATATT
M00059	V$YY1_01	0.797837	9537	(+)	NNNNNCCATNTWNNNWN	ACAAGTCATCTTCAATT
M00059	V$YY1_01	0.798493	9556	(+)	NNNNNCCATNTWNNNWN	CTTAGCCATTCTGACAC
M00059	V$YY1_01	0.787025	9575	(+)	NNNNNCCATNTWNNNWN	AAACACCATAAGTAAAA
M00059	V$YY1_01	0.782438	9605	(-)	NNNNNCCATNTWNNNWN	ATTAATATCTGACTTCA
M00059	V$YY1_01	0.797182	9790	(-)	NNNNNCCATNTWNNNWN	ACTTGAAAATGCTGGGA
M00059	V$YY1_01	0.809961	9847	(-)	NNNNNCCATNTWNNNWN	TTTCGAATAGGAAGAGC
M00059	V$YY1_01	0.771625	9918	(-)	NNNNNCCATNTWNNNWN	AATACAAAATTGTATTC
M00059	V$YY1_01	0.865007	9954	(-)	NNNNNCCATNTWNNNWN	AATTTTAAATGGGGTTT
M00059	V$YY1_01	0.827654	9973	(-)	NNNNNCCATNTWNNNWN	TTTTAAATATGTTTTTT
M00059	V$YY1_01	0.783093	10112	(+)	NNNNNCCATNTWNNNWN	CACATCCATAATTACTA
M00059	V$YY1_01	0.777523	10241	(-)	NNNNNCCATNTWNNNWN	ACTACTGGATGGCGAGT
M00059	V$YY1_01	0.884666	10301	(+)	NNNNNCCATNTWNNNWN	ACTTTCCATTTAAGGAA
M00059	V$YY1_01	0.817169	10433	(-)	NNNNNCCATNTWNNNWN	TGTATGAAATGGAAATA
M00059	V$YY1_01	0.798493	10458	(-)	NNNNNCCATNTWNNNWN	CTCACATCCTGGTGTTA
M00059	V$YY1_01	0.777851	10511	(+)	NNNNNCCATNTWNNNWN	TCCCTTCATCTGGTATG
M00059	V$YY1_01	0.832569	10649	(-)	NNNNNCCATNTWNNNWN	TTTGAACTATGATGTCA
M00059	V$YY1_01	0.802425	10681	(-)	NNNNNCCATNTWNNNWN	CTGATGGTATGGACAGA
M00059	V$YY1_01	0.788991	10696	(-)	NNNNNCCATNTWNNNWN	GATCAAAAATGTATCAA
M00059	V$YY1_01	0.806684	11014	(+)	NNNNNCCATNTWNNNWN	ATTCATCATTTCTGATC
M00059	V$YY1_01	0.872543	11129	(+)	NNNNNCCATNTWNNNWN	TGTCATCATATTTCGTA
M00252	V$TATA_01	0.799543	57	(+)	STATAAAWRNNNNNN	TTACATATGGCAAAA
M00252	V$TATA_01	0.815275	114	(+)	STATAAAWRNNNNNN	ATTTATATGCATTTG
M00216	V$TATA_C	0.750198	132	(+)	NCTATAAAAR	CTTTTCAAAT

M00216	V$TATA_C	0.893847	162	(+)	NCTATAAAAR	AGCTTAAAAA
M00216	V$TATA_C	0.811724	163	(+)	NCTATAAAAR	GCTTAAAAAT
M00252	V$TATA_01	0.832276	163	(+)	STATAAAWRNNNNNN	GCTTAAAAATGCATT
M00216	V$TATA_C	0.813837	227	(+)	NCTATAAAAR	GTTTTATAAA
M00252	V$TATA_01	0.800812	228	(+)	STATAAAWRNNNNNN	TTTTATAAACATCAA
M00216	V$TATA_C	0.793768	229	(+)	NCTATAAAAR	TTTATAAACA
M00252	V$TATA_01	0.79117	230	(+)	STATAAAWRNNNNNN	TTATAAACATCAAAT
M00216	V$TATA_C	0.746501	348	(+)	NCTATAAAAR	TCTATGGAAA
M00216	V$TATA_C	0.759176	373	(+)	NCTATAAAAR	AATTTATAAT
M00216	V$TATA_C	0.787959	382	(+)	NCTATAAAAR	TCTTTTTAAG
M00252	V$TATA_01	0.774676	398	(+)	STATAAAWRNNNNNN	GGATAAGAGCATTTC
M00216	V$TATA_C	0.878004	467	(+)	NCTATAAAAR	TCTATAAAGG
M00252	V$TATA_01	0.838873	468	(+)	STATAAAWRNNNNNN	CTATAAAGGATATGT
M00252	V$TATA_01	0.792692	500	(+)	STATAAAWRNNNNNN	ATATAACTAACTACT
M00216	V$TATA_C	0.851862	557	(+)	NCTATAAAAR	TGGATAAAAG
M00252	V$TATA_01	0.828724	558	(+)	STATAAAWRNNNNNN	GGATAAAAGTAGTAA
M00216	V$TATA_C	0.852126	583	(+)	NCTATAAAAR	GGTTTATAAA
M00252	V$TATA_01	0.833545	584	(+)	STATAAAWRNNNNNN	GTTTATAAATATGTC
M00216	V$TATA_C	0.793768	585	(+)	NCTATAAAAR	TTTATAAATA
M00252	V$TATA_01	0.855874	586	(+)	STATAAAWRNNNNNN	TTATAAATATGTCTT
M00216	V$TATA_C	0.760496	643	(+)	NCTATAAAAR	AGTATATATA
M00252	V$TATA_01	0.864248	644	(+)	STATAAAWRNNNNNN	GTATATATAAAATAT
M00216	V$TATA_C	0.809348	645	(+)	NCTATAAAAR	TATATATAAA
M00252	V$TATA_01	0.860188	646	(+)	STATAAAWRNNNNNN	ATATATAAAATATGG
M00216	V$TATA_C	0.856351	647	(+)	NCTATAAAAR	TATATAAAAT
M00252	V$TATA_01	0.859934	648	(+)	STATAAAWRNNNNNN	ATATAAAATATGGCG
M00252	V$TATA_01	0.776706	683	(+)	STATAAAWRNNNNNN	GTATGTATGATCTGT
M00216	V$TATA_C	0.780829	759	(+)	NCTATAAAAR	TTCTTATAAT
M00216	V$TATA_C	0.758912	777	(+)	NCTATAAAAR	ACTCTAGAAG
M00252	V$TATA_01	0.798021	874	(+)	STATAAAWRNNNNNN	AAATATAAAATAATG
M00216	V$TATA_C	0.849221	875	(+)	NCTATAAAAR	AATATAAAAT
M00252	V$TATA_01	0.857904	876	(+)	STATAAAWRNNNNNN	ATATAAAATAATGGC
M00216	V$TATA_C	0.841035	896	(+)	NCTATAAAAR	TCTTTAAGAA

M00252	V$TATA_01	0.77493	897	(+)	STATAAAWRNNNNNN	CTTTAAGAAAATAAT
M00216	V$TATA_C	0.906786	928	(+)	NCTATAAAAR	TCTATATAAT
M00252	V$TATA_01	0.894189	929	(+)	STATAAAWRNNNNNN	CTATATAATGCACCA
M00216	V$TATA_C	0.740692	953	(+)	NCTATAAAAR	TTCTTAAAGA
M00216	V$TATA_C	0.845524	980	(+)	NCTATAAAAR	GCTCTAAAAT
M00216	V$TATA_C	0.756007	982	(+)	NCTATAAAAR	TCTAAAATAG
M00216	V$TATA_C	0.847637	991	(+)	NCTATAAAAR	GATTTAAAAG
M00252	V$TATA_01	0.859173	992	(+)	STATAAAWRNNNNNN	ATTTAAAAGAGAAAG
M00216	V$TATA_C	0.750726	1012	(+)	NCTATAAAAR	TTTTTTAAAT
M00216	V$TATA_C	0.779509	1013	(+)	NCTATAAAAR	TTTTTAAATG
M00252	V$TATA_01	0.806902	1014	(+)	STATAAAWRNNNNNN	TTTTAAATGTCAGAT
M00216	V$TATA_C	0.761025	1067	(+)	NCTATAAAAR	ATTTTAACAA
M00252	V$TATA_01	0.798275	1118	(+)	STATAAAWRNNNNNN	ATATTTAAAAATTGA
M00216	V$TATA_C	0.848165	1119	(+)	NCTATAAAAR	TATTTAAAAA
M00216	V$TATA_C	0.743597	1120	(+)	NCTATAAAAR	ATTTAAAAAT
M00252	V$TATA_01	0.840396	1120	(+)	STATAAAWRNNNNNN	ATTTAAAAATTGAGT
M00216	V$TATA_C	0.760496	1135	(+)	NCTATAAAAR	AGTATTTAAA
M00252	V$TATA_01	0.821365	1136	(+)	STATAAAWRNNNNNN	GTATTTAAATATGAA
M00252	V$TATA_01	0.853844	1138	(+)	STATAAAWRNNNNNN	ATTTAAATATGAATG
M00252	V$TATA_01	0.808171	1144	(+)	STATAAAWRNNNNNN	ATATGAATGCCTCTC
M00216	V$TATA_C	0.873515	1221	(+)	NCTATAAAAR	TGAATAAAAA
M00252	V$TATA_01	0.799797	1222	(+)	STATAAAWRNNNNNN	GAATAAAAATATTAA
M00252	V$TATA_01	0.825679	1230	(+)	STATAAAWRNNNNNN	ATATTAATAAGAGAC
M00252	V$TATA_01	0.784572	1297	(+)	STATAAAWRNNNNNN	ATTTATATTGAATAG
M00216	V$TATA_C	0.760496	1314	(+)	NCTATAAAAR	AAATTAAAAA
M00252	V$TATA_01	0.789901	1323	(+)	STATAAAWRNNNNNN	ATTTAAATTAGTAAA
M00252	V$TATA_01	0.772139	1333	(+)	STATAAAWRNNNNNN	GTAAAAATATTAAAC
M00252	V$TATA_01	0.791677	1339	(+)	STATAAAWRNNNNNN	ATATTAAACAAATGA
M00216	V$TATA_C	0.760232	1364	(+)	NCTATAAAAR	ATTATAACAC
M00252	V$TATA_01	0.793453	1409	(+)	STATAAAWRNNNNNN	ATATGAAACCAAATT
M00216	V$TATA_C	0.744389	1608	(+)	NCTATAAAAR	AGAATAATAA
M00216	V$TATA_C	0.745973	1640	(+)	NCTATAAAAR	GCTATTGAAG
M00216	V$TATA_C	0.776076	1655	(+)	NCTATAAAAR	ATATTAAAAC

M00252	V$TATA_01	0.820858	1655	(+)	STATAAAWRNNNNNN	ATATTAAAACTATGA
M00216	V$TATA_C	0.774756	1718	(+)	NCTATAAAAR	TCCAAATAAA
M00216	V$TATA_C	0.775284	1720	(+)	NCTATAAAAR	CAAATAAAAT
M00252	V$TATA_01	0.784065	1721	(+)	STATAAAWRNNNNNN	AAATAAAATCTCTGC
M00216	V$TATA_C	0.761553	1733	(+)	NCTATAAAAR	TGCTTGAAAT
M00252	V$TATA_01	0.799036	1739	(+)	STATAAAWRNNNNNN	AAATATAAATCTCTC
M00252	V$TATA_01	0.841411	1741	(+)	STATAAAWRNNNNNN	ATATAAATCTCTCAT
M00216	V$TATA_C	0.795088	1754	(+)	NCTATAAAAR	ATGATAAAAA
M00252	V$TATA_01	0.786602	1755	(+)	STATAAAWRNNNNNN	TGATAAAAAATAATA
M00216	V$TATA_C	0.828096	1773	(+)	NCTATAAAAR	ATAATAAAAA
M00252	V$TATA_01	0.798275	1774	(+)	STATAAAWRNNNNNN	TAATAAAAAAGTAAA
M00216	V$TATA_C	0.756007	1775	(+)	NCTATAAAAR	AATAAAAAAG
M00252	V$TATA_01	0.790916	1776	(+)	STATAAAWRNNNNNN	ATAAAAAAGTAAAGA
M00216	V$TATA_C	0.757592	1788	(+)	NCTATAAAAR	AGAATAGAAG
M00216	V$TATA_C	0.793768	1804	(+)	NCTATAAAAR	GGTATCAAAC
M00216	V$TATA_C	0.80882	1826	(+)	NCTATAAAAR	AGCATCAAAG
M00252	V$TATA_01	0.782289	1878	(+)	STATAAAWRNNNNNN	GTATTTGTAAACATA
M00252	V$TATA_01	0.771885	1888	(+)	STATAAAWRNNNNNN	ACATATGTGTCCAAT
M00252	V$TATA_01	0.801319	1923	(+)	STATAAAWRNNNNNN	ACATAAAGCCGTATA
M00216	V$TATA_C	0.822023	2012	(+)	NCTATAAAAR	GGTATAGAAA
M00252	V$TATA_01	0.819081	2013	(+)	STATAAAWRNNNNNN	GTATAGAAAATCAGG
M00252	V$TATA_01	0.771885	2015	(+)	STATAAAWRNNNNNN	ATAGAAAATCAGGCT
M00216	V$TATA_C	0.745973	2066	(+)	NCTATAAAAR	CCTATAGACA
M00216	V$TATA_C	0.743068	2093	(+)	NCTATAAAAR	AAATTAAAAT
M00216	V$TATA_C	0.740692	2108	(+)	NCTATAAAAR	TTCTCAAAAA
M00216	V$TATA_C	0.759704	2114	(+)	NCTATAAAAR	AAAATAAAAC
M00252	V$TATA_01	0.783811	2115	(+)	STATAAAWRNNNNNN	AAATAAAACAAAAAA
M00252	V$TATA_01	0.808678	2142	(+)	STATAAAWRNNNNNN	TTACATAAAGCAAAA
M00252	V$TATA_01	0.786602	2144	(+)	STATAAAWRNNNNNN	ACATAAAGCAAAAAG
M00216	V$TATA_C	0.752575	2222	(+)	NCTATAAAAR	ACAAGAAAAC
M00216	V$TATA_C	0.742276	2236	(+)	NCTATAAAAR	CAGATAAAAT
M00252	V$TATA_01	0.78305	2237	(+)	STATAAAWRNNNNNN	AGATAAAATAATGGT
M00216	V$TATA_C	0.842091	2273	(+)	NCTATAAAAR	ACTATCAAAT

M00252	V$TATA_01	0.774423	2341	(+)	STATAAAWRNNNNNN	ATATTTAGAAAATGC
M00216	V$TATA_C	0.77634	2344	(+)	NCTATAAAAR	TTTAGAAAAT
M00216	V$TATA_C	0.752046	2384	(+)	NCTATAAAAR	TTCTTAACAG
M00216	V$TATA_C	0.781885	2448	(+)	NCTATAAAAR	GTTACAAAAA
M00252	V$TATA_01	0.794215	2449	(+)	STATAAAWRNNNNNN	TTACAAAAATAAATC
M00252	V$TATA_01	0.795483	2455	(+)	STATAAAWRNNNNNN	AAATAAATCCAAGTC
M00216	V$TATA_C	0.769475	2508	(+)	NCTATAAAAR	CTTTTACAAA
M00252	V$TATA_01	0.780766	2511	(+)	STATAAAWRNNNNNN	TTACAAATGAAATGG
M00216	V$TATA_C	0.793768	2537	(+)	NCTATAAAAR	CCAGTAAAAA
M00216	V$TATA_C	0.824927	2539	(+)	NCTATAAAAR	AGTAAAAAAA
M00252	V$TATA_01	0.786349	2540	(+)	STATAAAWRNNNNNN	GTAAAAAAATAGTTT
M00216	V$TATA_C	0.763929	2678	(+)	NCTATAAAAR	GAAATAAAAT
M00252	V$TATA_01	0.806648	2679	(+)	STATAAAWRNNNNNN	AAATAAAATCCAGAC
M00216	V$TATA_C	0.740956	2727	(+)	NCTATAAAAR	GCTCCAAAAA
M00216	V$TATA_C	0.777132	2745	(+)	NCTATAAAAR	GGTTTAAAGT
M00252	V$TATA_01	0.808171	2746	(+)	STATAAAWRNNNNNN	GTTTAAAGTCACCTT
M00216	V$TATA_C	0.815949	2757	(+)	NCTATAAAAR	CCTTTAGAAC
M00216	V$TATA_C	0.750198	2768	(+)	NCTATAAAAR	ACCATGTAAT
M00216	V$TATA_C	0.758912	2773	(+)	NCTATAAAAR	GTAATATAAA
M00252	V$TATA_01	0.839127	2776	(+)	STATAAAWRNNNNNN	ATATAAATTGTTCAG
M00216	V$TATA_C	0.759176	2805	(+)	NCTATAAAAR	TCTTTATCAT
M00216	V$TATA_C	0.745709	2897	(+)	NCTATAAAAR	ACTCTAAAGA
M00216	V$TATA_C	0.769475	2920	(+)	NCTATAAAAR	ACTTTTTAAA
M00216	V$TATA_C	0.750198	2921	(+)	NCTATAAAAR	CTTTTTAAAT
M00216	V$TATA_C	0.750726	2922	(+)	NCTATAAAAR	TTTTTAAATT
M00216	V$TATA_C	0.890679	2936	(+)	NCTATAAAAR	TCAATAAAAT
M00216	V$TATA_C	0.838659	2958	(+)	NCTATAAAAR	CCCAGAAAAA
M00216	V$TATA_C	0.854766	2988	(+)	NCTATAAAAR	GCTATTAAAA
M00216	V$TATA_C	0.791656	2989	(+)	NCTATAAAAR	CTATTAAAAT
M00252	V$TATA_01	0.859173	2989	(+)	STATAAAWRNNNNNN	CTATTAAAATACAAA
M00216	V$TATA_C	0.740692	3007	(+)	NCTATAAAAR	TTCTTAAATA
M00252	V$TATA_01	0.802588	3008	(+)	STATAAAWRNNNNNN	TCTTAAATAGCAACA
M00216	V$TATA_C	0.822023	3098	(+)	NCTATAAAAR	GTTATATAAT

M00252	V$TATA_01	0.824156	3099	(+)	STATAAAWRNNNNNN	TTATATAATTCTTGC
M00216	V$TATA_C	0.810404	3258	(+)	NCTATAAAAR	TCTCTATAAA
M00252	V$TATA_01	0.794722	3259	(+)	STATAAAWRNNNNNN	CTCTATAAAGTCAGT
M00216	V$TATA_C	0.849221	3260	(+)	NCTATAAAAR	TCTATAAAGT
M00252	V$TATA_01	0.846232	3261	(+)	STATAAAWRNNNNNN	CTATAAAGTCAGTTT
M00216	V$TATA_C	0.802746	3288	(+)	NCTATAAAAR	GGTATAATAT
M00252	V$TATA_01	0.780513	3289	(+)	STATAAAWRNNNNNN	GTATAATATCTTCAG
M00252	V$TATA_01	0.817559	3370	(+)	STATAAAWRNNNNNN	ACATAAATATTTATG
M00252	V$TATA_01	0.777721	3376	(+)	STATAAAWRNNNNNN	ATATTTATGATTAAT
M00216	V$TATA_C	0.743597	3441	(+)	NCTATAAAAR	ATTTTAAATT
M00216	V$TATA_C	0.757328	3487	(+)	NCTATAAAAR	AACATATAAT
M00252	V$TATA_01	0.79523	3490	(+)	STATAAAWRNNNNNN	ATATAATTTGCAATT
M00216	V$TATA_C	0.756007	3540	(+)	NCTATAAAAR	TCTACAACAG
M00252	V$TATA_01	0.775438	3550	(+)	STATAAAWRNNNNNN	CCATTAATCCACTCT
M00252	V$TATA_01	0.773408	3576	(+)	STATAAAWRNNNNNN	GAATTAATACCTTTT
M00216	V$TATA_C	0.865593	3876	(+)	NCTATAAAAR	ATTTTAAAAT
M00252	V$TATA_01	0.801827	3877	(+)	STATAAAWRNNNNNN	TTTTAAAATTAGATC
M00252	V$TATA_01	0.789394	3963	(+)	STATAAAWRNNNNNN	GTTCAAATGGAAAAC
M00216	V$TATA_C	0.808292	4004	(+)	NCTATAAAAR	TTTTTATAAT
M00216	V$TATA_C	0.797465	4041	(+)	NCTATAAAAR	TCAACAAAAG
M00216	V$TATA_C	0.783998	4093	(+)	NCTATAAAAR	CTTCTAAAAT
M00216	V$TATA_C	0.740692	4113	(+)	NCTATAAAAR	TTCTTGAAAA
M00216	V$TATA_C	0.841035	4114	(+)	NCTATAAAAR	TCTTGAAAAA
M00216	V$TATA_C	0.757064	4198	(+)	NCTATAAAAR	ACTGAAAAAG
M00216	V$TATA_C	0.744653	4229	(+)	NCTATAAAAR	AATATTAAAA
M00216	V$TATA_C	0.802482	4230	(+)	NCTATAAAAR	ATATTAAAAA
M00252	V$TATA_01	0.848008	4230	(+)	STATAAAWRNNNNNN	ATATTAAAAAGCTGC
M00216	V$TATA_C	0.761553	4242	(+)	NCTATAAAAR	TGCTTTAAAT
M00216	V$TATA_C	0.811724	4243	(+)	NCTATAAAAR	GCTTTAAATT
M00252	V$TATA_01	0.788632	4244	(+)	STATAAAWRNNNNNN	CTTTAAATTTTAATT
M00216	V$TATA_C	0.745445	4307	(+)	NCTATAAAAR	ATTTTACAAT
M00216	V$TATA_C	0.800634	4325	(+)	NCTATAAAAR	TCATTATAAT
M00252	V$TATA_01	0.780766	4326	(+)	STATAAAWRNNNNNN	CATTATAATGGAATG

M00216	V$TATA_C	0.78479	4350	(+)	NCTATAAAAR	TCTAGATAAT
M00216	V$TATA_C	0.803274	4440	(+)	NCTATAAAAR	ACTGTATAAA
M00216	V$TATA_C	0.936625	4442	(+)	NCTATAAAAR	TGTATAAAAT
M00252	V$TATA_01	0.839635	4443	(+)	STATAAAWRNNNNNN	GTATAAAATATTTGA
M00216	V$TATA_C	0.750726	4484	(+)	NCTATAAAAR	TTTTTAACAT
M00252	V$TATA_01	0.797767	4638	(+)	STATAAAWRNNNNNN	CTTTTTATGGAAATA
M00216	V$TATA_C	0.761553	4730	(+)	NCTATAAAAR	ACAACAAAAT
M00216	V$TATA_C	0.865593	4753	(+)	NCTATAAAAR	ATTTTAAAAT
M00252	V$TATA_01	0.792692	4763	(+)	STATAAAWRNNNNNN	ATTTTTAAGTGCACA
M00252	V$TATA_01	0.777468	4765	(+)	STATAAAWRNNNNNN	TTTTAAGTGCACATT
M00216	V$TATA_C	0.833905	4792	(+)	NCTATAAAAR	ACTTTAAAGA
M00252	V$TATA_01	0.815022	4793	(+)	STATAAAWRNNNNNN	CTTTAAAGAAAGCTT
M00252	V$TATA_01	0.771378	4848	(+)	STATAAAWRNNNNNN	ATATTAGTGCAGTCA
M00216	V$TATA_C	0.754423	4859	(+)	NCTATAAAAR	GTCACAAAAA
M00216	V$TATA_C	0.752575	4862	(+)	NCTATAAAAR	ACAAAAAAAC
M00252	V$TATA_01	0.78711	4880	(+)	STATAAAWRNNNNNN	CTTTTAATTTGCAGT
M00252	V$TATA_01	0.77087	5018	(+)	STATAAAWRNNNNNN	ATATTTAAATTATTT
M00216	V$TATA_C	0.894375	5029	(+)	NCTATAAAAR	ATTTTAAAAG
M00252	V$TATA_01	0.784826	5029	(+)	STATAAAWRNNNNNN	ATTTTAAAAGTTAGT
M00252	V$TATA_01	0.79117	5030	(+)	STATAAAWRNNNNNN	TTTTAAAAGTTAGTC
M00216	V$TATA_C	0.840507	5044	(+)	NCTATAAAAR	CCTTTCAAAA
M00216	V$TATA_C	0.767626	5045	(+)	NCTATAAAAR	CTTTCAAAAA
M00216	V$TATA_C	0.748878	5047	(+)	NCTATAAAAR	TTCAAAAAAT
M00252	V$TATA_01	0.771885	5055	(+)	STATAAAWRNNNNNN	ATATATTTTACAAGT
M00216	V$TATA_C	0.774228	5059	(+)	NCTATAAAAR	ATTTTACAAG
M00216	V$TATA_C	0.792712	5157	(+)	NCTATAAAAR	GGCAAAAAAA
M00216	V$TATA_C	0.7568	5158	(+)	NCTATAAAAR	GCAAAAAAAT
M00216	V$TATA_C	0.793768	5197	(+)	NCTATAAAAR	TTTAGAAAAA
M00216	V$TATA_C	0.821231	5235	(+)	NCTATAAAAR	CCCATAAACT
M00216	V$TATA_C	0.833905	5242	(+)	NCTATAAAAR	ACTTTCAAAA
M00216	V$TATA_C	0.750198	5243	(+)	NCTATAAAAR	CTTTCAAAAT
M00216	V$TATA_C	0.807499	5301	(+)	NCTATAAAAR	ACTTTAAAGC
M00252	V$TATA_01	0.773915	5302	(+)	STATAAAWRNNNNNN	CTTTAAAGCTGTGAA

M00252	V$TATA_01	0.802081	5331	(+)	STATAAAWRNNNNNN	GTATGAATGAATAAA
M00216	V$TATA_C	0.762873	5338	(+)	NCTATAAAAR	TGAATAAATG
M00252	V$TATA_01	0.832022	5339	(+)	STATAAAWRNNNNNN	GAATAAATGGATGAA
M00216	V$TATA_C	0.762873	5358	(+)	NCTATAAAAR	TGAATAAATG
M00252	V$TATA_01	0.827709	5359	(+)	STATAAAWRNNNNNN	GAATAAATGCAATTA
M00216	V$TATA_C	0.824927	5393	(+)	NCTATAAAAR	AGTATCAAAA
M00252	V$TATA_01	0.79726	5394	(+)	STATAAAWRNNNNNN	GTATCAAAACTGAAA
M00216	V$TATA_C	0.844204	5427	(+)	NCTATAAAAR	ATTATATAAA
M00252	V$TATA_01	0.924638	5428	(+)	STATAAAWRNNNNNN	TTATATAAAGCAACG
M00252	V$TATA_01	0.826947	5430	(+)	STATAAAWRNNNNNN	ATATAAAGCAACGTA
M00216	V$TATA_C	0.740956	5448	(+)	NCTATAAAAR	GCTCTAAGAA
M00216	V$TATA_C	0.740692	5486	(+)	NCTATAAAAR	TTCTTGAAAA
M00216	V$TATA_C	0.841035	5487	(+)	NCTATAAAAR	TCTTGAAAAA
M00216	V$TATA_C	0.779509	5527	(+)	NCTATAAAAR	TTTTTAATAG
M00252	V$TATA_01	0.787364	5527	(+)	STATAAAWRNNNNNN	TTTTTAATAGAGGCC
M00216	V$TATA_C	0.761553	5560	(+)	NCTATAAAAR	TGCTCAAAAT
M00252	V$TATA_01	0.799036	5580	(+)	STATAAAWRNNNNNN	AAATAAATATCTTTG
M00216	V$TATA_C	0.750198	5589	(+)	NCTATAAAAR	TCTTTGTAAC
M00216	V$TATA_C	0.750726	5679	(+)	NCTATAAAAR	TTTTTAAACT
M00216	V$TATA_C	0.842091	5686	(+)	NCTATAAAAR	ACTATTAAAT
M00252	V$TATA_01	0.82644	5687	(+)	STATAAAWRNNNNNN	CTATTAAATATAAGG
M00216	V$TATA_C	0.740164	5691	(+)	NCTATAAAAR	TAAATATAAG
M00252	V$TATA_01	0.838873	5692	(+)	STATAAAWRNNNNNN	AAATATAAGGAAAAG
M00252	V$TATA_01	0.772139	5694	(+)	STATAAAWRNNNNNN	ATATAAGGAAAAGAA
M00216	V$TATA_C	0.796145	5724	(+)	NCTATAAAAR	TCCTTTAAAT
M00216	V$TATA_C	0.840507	5725	(+)	NCTATAAAAR	CCTTTAAATA
M00252	V$TATA_01	0.883786	5726	(+)	STATAAAWRNNNNNN	CTTTAAATACCCTGC
M00216	V$TATA_C	0.751518	5747	(+)	NCTATAAAAR	TGAATAAATA
M00252	V$TATA_01	0.827201	5748	(+)	STATAAAWRNNNNNN	GAATAAATAAGTCTC
M00252	V$TATA_01	0.805887	5818	(+)	STATAAAWRNNNNNN	GCATATGAAAGCCAG
M00216	V$TATA_C	0.762609	5819	(+)	NCTATAAAAR	CATATGAAAG
M00252	V$TATA_01	0.81832	5820	(+)	STATAAAWRNNNNNN	ATATGAAAGCCAGAA
M00216	V$TATA_C	0.754423	5901	(+)	NCTATAAAAR	GTCATGAAAA

M00216	V$TATA_C	0.778981	5947	(+)	NCTATAAAAR	TGCTTAAATA
M00252	V$TATA_01	0.814514	5954	(+)	STATAAAWRNNNNNN	ATATTTATATGCCAT
M00252	V$TATA_01	0.837605	5956	(+)	STATAAAWRNNNNNN	ATTTATATGCCATTG
M00216	V$TATA_C	0.758648	5999	(+)	NCTATAAAAR	CCTTTTTAAT
M00216	V$TATA_C	0.750726	6001	(+)	NCTATAAAAR	TTTTTAATAT
M00216	V$TATA_C	0.753367	6004	(+)	NCTATAAAAR	TTAATATAAT
M00252	V$TATA_01	0.7729	6005	(+)	STATAAAWRNNNNNN	TAATATAATGCTCCT
M00216	V$TATA_C	0.761553	6019	(+)	NCTATAAAAR	TGCTTAACAT
M00216	V$TATA_C	0.778981	6059	(+)	NCTATAAAAR	TAATTAAAAG
M00216	V$TATA_C	0.78611	6100	(+)	NCTATAAAAR	AACATATAAG
M00252	V$TATA_01	0.805887	6101	(+)	STATAAAWRNNNNNN	ACATATAAGAATCTA
M00216	V$TATA_C	0.751254	6102	(+)	NCTATAAAAR	CATATAAGAA
M00252	V$TATA_01	0.854098	6103	(+)	STATAAAWRNNNNNN	ATATAAGAATCTACG
M00216	V$TATA_C	0.78479	6241	(+)	NCTATAAAAR	TCTATATATT
M00252	V$TATA_01	0.844456	6242	(+)	STATAAAWRNNNNNN	CTATATATTTGGTAA
M00252	V$TATA_01	0.782035	6244	(+)	STATAAAWRNNNNNN	ATATATTTGGTAATT
M00216	V$TATA_C	0.881172	6281	(+)	NCTATAAAAR	ATCATAAAAA
M00216	V$TATA_C	0.760496	6282	(+)	NCTATAAAAR	TCATAAAAAA
M00252	V$TATA_01	0.814514	6282	(+)	STATAAAWRNNNNNN	TCATAAAAAATAAAG
M00216	V$TATA_C	0.756271	6355	(+)	NCTATAAAAR	GTTTTAATAA
M00252	V$TATA_01	0.789901	6355	(+)	STATAAAWRNNNNNN	GTTTTAATAAACTGT
M00216	V$TATA_C	0.769475	6408	(+)	NCTATAAAAR	ACTTTATCAA
M00216	V$TATA_C	0.805123	6410	(+)	NCTATAAAAR	TTTATCAAAG
M00216	V$TATA_C	0.748878	6461	(+)	NCTATAAAAR	ACTATTATAG
M00252	V$TATA_01	0.799543	6462	(+)	STATAAAWRNNNNNN	CTATTATAGCCAGGA
M00216	V$TATA_C	0.762873	6491	(+)	NCTATAAAAR	GCCAGATAAA
M00252	V$TATA_01	0.777214	6505	(+)	STATAAAWRNNNNNN	ACATTAGAGGGGAGA
M00216	V$TATA_C	0.743068	6525	(+)	NCTATAAAAR	TCATTTAAAT
M00252	V$TATA_01	0.811469	6527	(+)	STATAAAWRNNNNNN	ATTTAAATGATAATC
M00252	V$TATA_01	0.78305	6628	(+)	STATAAAWRNNNNNN	ATTTATGAAGGTATA
M00216	V$TATA_C	0.822023	6637	(+)	NCTATAAAAR	GGTATAGAAA
M00252	V$TATA_01	0.840142	6638	(+)	STATAAAWRNNNNNN	GTATAGAAAGGACGC
M00252	V$TATA_01	0.880995	6732	(+)	STATAAAWRNNNNNN	ACATAAATGGCGGA

M00216	V$TATA_C	0.817798	6846	(+)	NCTATAAAAR	TGTTTGAAAG
M00216	V$TATA_C	0.740956	7126	(+)	NCTATAAAAR	GCTCTAACAA
M00216	V$TATA_C	0.744653	7128	(+)	NCTATAAAAR	TCTAACAAAA
M00216	V$TATA_C	0.793768	7149	(+)	NCTATAAAAR	TTTATTAAAA
M00216	V$TATA_C	0.792184	7150	(+)	NCTATAAAAR	TTATTAAAAT
M00252	V$TATA_01	0.831515	7150	(+)	STATAAAWRNNNNNN	TTATTAAAATAAAGA
M00252	V$TATA_01	0.776199	7156	(+)	STATAAAWRNNNNNN	AAATAAAGAGTAAAA
M00216	V$TATA_C	0.752575	7162	(+)	NCTATAAAAR	AGAGTAAAAA
M00252	V$TATA_01	0.779498	7181	(+)	STATAAAWRNNNNNN	GTATGAATGTTTAAA
M00216	V$TATA_C	0.911011	7188	(+)	NCTATAAAAR	TGTTTAAAAT
M00252	V$TATA_01	0.836336	7189	(+)	STATAAAWRNNNNNN	GTTTAAAATGAACTA
M00216	V$TATA_C	0.833113	7200	(+)	NCTATAAAAR	ACTATAAGAC
M00252	V$TATA_01	0.841411	7201	(+)	STATAAAWRNNNNNN	CTATAAGACTCAAAG
M00216	V$TATA_C	0.752839	7358	(+)	NCTATAAAAR	AATCTAAAAA
M00216	V$TATA_C	0.744917	7359	(+)	NCTATAAAAR	ATCTAAAAAG
M00252	V$TATA_01	0.794722	7359	(+)	STATAAAWRNNNNNN	ATCTAAAAAGATGGG
M00216	V$TATA_C	0.744653	7360	(+)	NCTATAAAAR	TCTAAAAAGA
M00252	V$TATA_01	0.782796	7361	(+)	STATAAAWRNNNNNN	CTAAAAAGATGGGGA
M00216	V$TATA_C	0.80169	7387	(+)	NCTATAAAAR	GCCTCAAAAA
M00216	V$TATA_C	0.740164	7435	(+)	NCTATAAAAR	TGCATATTAA
M00216	V$TATA_C	0.751254	7437	(+)	NCTATAAAAR	CATATTAAAA
M00216	V$TATA_C	0.785054	7438	(+)	NCTATAAAAR	ATATTAAAAT
M00252	V$TATA_01	0.853844	7438	(+)	STATAAAWRNNNNNN	ATATTAAAATCAATC
M00216	V$TATA_C	0.823607	7451	(+)	NCTATAAAAR	TCTTTTAAAT
M00216	V$TATA_C	0.767626	7452	(+)	NCTATAAAAR	CTTTTAAATA
M00252	V$TATA_01	0.804618	7453	(+)	STATAAAWRNNNNNN	TTTTAAATATGTCCT
M00252	V$TATA_01	0.825425	7494	(+)	STATAAAWRNNNNNN	AAATATAAACATCCG
M00216	V$TATA_C	0.744653	7495	(+)	NCTATAAAAR	AATATAAACA
M00252	V$TATA_01	0.827201	7496	(+)	STATAAAWRNNNNNN	ATATAAACATCCGTG
M00216	V$TATA_C	0.806443	7549	(+)	NCTATAAAAR	TGTTTTAAAA
M00216	V$TATA_C	0.878268	7550	(+)	NCTATAAAAR	GTTTTAAAAA
M00252	V$TATA_01	0.791677	7550	(+)	STATAAAWRNNNNNN	GTTTTAAAAAGCTAA
M00216	V$TATA_C	0.779509	7551	(+)	NCTATAAAAR	TTTTAAAAAG

M00252	V$TATA_01	0.844202	7551	(+)	STATAAAWRNNNNNN	TTTTAAAAAGCTAAT
M00216	V$TATA_C	0.919461	7625	(+)	NCTATAAAAR	AGCATAAAAA
M00216	V$TATA_C	0.759968	7626	(+)	NCTATAAAAR	GCATAAAAAG
M00252	V$TATA_01	0.912966	7626	(+)	STATAAAWRNNNNNN	GCATAAAAAGGGCCC
M00252	V$TATA_01	0.780005	7628	(+)	STATAAAWRNNNNNN	ATAAAAAGGGCCCCA
M00216	V$TATA_C	0.759176	7723	(+)	NCTATAAAAR	ATCACAAAAA
M00216	V$TATA_C	0.756271	7776	(+)	NCTATAAAAR	GTTTTAATAA
M00252	V$TATA_01	0.78914	7776	(+)	STATAAAWRNNNNNN	GTTTTAATAAAAAAT
M00216	V$TATA_C	0.835226	7779	(+)	NCTATAAAAR	TTAATAAAAA
M00216	V$TATA_C	0.772379	7837	(+)	NCTATAAAAR	ATTTTAAATG
M00252	V$TATA_01	0.797767	7838	(+)	STATAAAWRNNNNNN	TTTTAAATGAAATTG
M00216	V$TATA_C	0.776076	7881	(+)	NCTATAAAAR	CCTTTATATA
M00252	V$TATA_01	0.819081	7882	(+)	STATAAAWRNNNNNN	CTTTATATACTACTT
M00252	V$TATA_01	0.772139	7897	(+)	STATAAAWRNNNNNN	GTTTTAATTCTGAAC
M00216	V$TATA_C	0.831529	7928	(+)	NCTATAAAAR	GGTATGAAAG
M00252	V$TATA_01	0.844456	7929	(+)	STATAAAWRNNNNNN	GTATGAAAGTGTGGG
M00216	V$TATA_C	0.833905	7958	(+)	NCTATAAAAR	ACTTTTAAAA
M00216	V$TATA_C	0.872194	7959	(+)	NCTATAAAAR	CTTTTAAAAT
M00252	V$TATA_01	0.804872	7959	(+)	STATAAAWRNNNNNN	CTTTTAAAATCACAG
M00252	V$TATA_01	0.847754	7960	(+)	STATAAAWRNNNNNN	TTTTAAAATCACAGG
M00252	V$TATA_01	0.792438	7998	(+)	STATAAAWRNNNNNN	TTTTTTAAGCGAAAG
M00216	V$TATA_C	0.741748	8069	(+)	NCTATAAAAR	ATCAGAAAAT
M00216	V$TATA_C	0.742012	8078	(+)	NCTATAAAAR	TGTTTATGAA
M00252	V$TATA_01	0.775691	8079	(+)	STATAAAWRNNNNNN	GTTTATGAAAGCTTA
M00216	V$TATA_C	0.805123	8080	(+)	NCTATAAAAR	TTTATGAAAG
M00252	V$TATA_01	0.77493	8081	(+)	STATAAAWRNNNNNN	TTATGAAAGCTTATT
M00252	V$TATA_01	0.78102	8178	(+)	STATAAAWRNNNNNN	GGATATCAACCCAAC
M00216	V$TATA_C	0.832057	8196	(+)	NCTATAAAAR	TGTATGAAAA
M00252	V$TATA_01	0.847501	8197	(+)	STATAAAWRNNNNNN	GTATGAAAACAAATA
M00216	V$TATA_C	0.754423	8337	(+)	NCTATAAAAR	GTCATTAAAA
M00216	V$TATA_C	0.882493	8338	(+)	NCTATAAAAR	TCATTAAAAA
M00216	V$TATA_C	0.742012	8363	(+)	NCTATAAAAR	TGTTTCTAAA
M00252	V$TATA_01	0.770363	8364	(+)	STATAAAWRNNNNNN	GTTTCTAAACCAATG

M00216	V$TATA_C	0.764457	8533	(+)	NCTATAAAAR	GTTACAAAAT
M00216	V$TATA_C	0.759704	8538	(+)	NCTATAAAAR	AAAATAAAAC
M00252	V$TATA_01	0.802081	8539	(+)	STATAAAWRNNNNNN	AAATAAAACCAACTC
M00216	V$TATA_C	0.805651	8577	(+)	NCTATAAAAR	TCAGTAAAAG
M00216	V$TATA_C	0.807499	8664	(+)	NCTATAAAAR	AGTATAACAT
M00252	V$TATA_01	0.8102	8665	(+)	STATAAAWRNNNNNN	GTATAACATGCACCT
M00216	V$TATA_C	0.753367	8827	(+)	NCTATAAAAR	TGTTTTTAAG
M00252	V$TATA_01	0.802334	8828	(+)	STATAAAWRNNNNNN	GTTTTTAAGGAAGTT
M00252	V$TATA_01	0.810962	8866	(+)	STATAAAWRNNNNNN	ATATATTTACCAAAT
M00216	V$TATA_C	0.823607	8878	(+)	NCTATAAAAR	AATTTAAAAT
M00252	V$TATA_01	0.782035	8884	(+)	STATAAAWRNNNNNN	AAATATATTAGCATT
M00216	V$TATA_C	0.752046	8908	(+)	NCTATAAAAR	ATGTTAAAAT
M00216	V$TATA_C	0.793768	9004	(+)	NCTATAAAAR	TTTATTAAAA
M00216	V$TATA_C	0.820966	9005	(+)	NCTATAAAAR	TTATTAAAAG
M00252	V$TATA_01	0.778229	9005	(+)	STATAAAWRNNNNNN	TTATTAAAAGTTTAT
M00216	V$TATA_C	0.805123	9015	(+)	NCTATAAAAR	TTTATCAAAG
M00252	V$TATA_01	0.79523	9016	(+)	STATAAAWRNNNNNN	TTATCAAAGGCAAAG
M00216	V$TATA_C	0.787167	9073	(+)	NCTATAAAAR	TCCTTAACAC
M00216	V$TATA_C	0.752311	9140	(+)	NCTATAAAAR	CCTCTGAAAA
M00216	V$TATA_C	0.750726	9164	(+)	NCTATAAAAR	TTTTTTAAAT
M00216	V$TATA_C	0.741748	9165	(+)	NCTATAAAAR	TTTTTAAATC
M00252	V$TATA_01	0.797006	9166	(+)	STATAAAWRNNNNNN	TTTTAAATCTCAGGT
M00252	V$TATA_01	0.816037	9179	(+)	STATAAAWRNNNNNN	GTATTTAATTCATGC
M00216	V$TATA_C	0.743068	9196	(+)	NCTATAAAAR	TCATTTAAAT
M00252	V$TATA_01	0.802081	9198	(+)	STATAAAWRNNNNNN	ATTTAAATATTGTGT
M00216	V$TATA_C	0.755743	9244	(+)	NCTATAAAAR	GGTATTTAAA
M00252	V$TATA_01	0.867039	9245	(+)	STATAAAWRNNNNNN	GTATTTAAAGGGTAC
M00252	V$TATA_01	0.791677	9247	(+)	STATAAAWRNNNNNN	ATTTAAAGGGTACTC
M00216	V$TATA_C	0.891207	9360	(+)	NCTATAAAAR	ATTATAAAAT
M00252	V$TATA_01	0.872621	9361	(+)	STATAAAWRNNNNNN	TTATAAAATGACAAT
M00216	V$TATA_C	0.819118	9371	(+)	NCTATAAAAR	ACAATATAAT
M00252	V$TATA_01	0.773915	9372	(+)	STATAAAWRNNNNNN	CAATATAATAATAAA
M00216	V$TATA_C	0.744653	9373	(+)	NCTATAAAAR	AATATAATAA

M00252	V$TATA_01	0.801319	9374	(+)	STATAAAWRNNNNNN	ATATAATAATAAATA
M00252	V$TATA_01	0.803096	9386	(+)	STATAAAWRNNNNNN	ATATTAAATGATGTA
M00252	V$TATA_01	0.773408	9404	(+)	STATAAAWRNNNNNN	CAATTAATGCAATCC
M00252	V$TATA_01	0.771378	9580	(+)	STATAAAWRNNNNNN	CCATAAGTAAAATTG
M00216	V$TATA_C	0.743068	9603	(+)	NCTATAAAAR	TCATTAATAT
M00216	V$TATA_C	0.761025	9670	(+)	NCTATAAAAR	ATTTTTAAAA
M00216	V$TATA_C	0.890151	9671	(+)	NCTATAAAAR	TTTTTAAAAA
M00216	V$TATA_C	0.768154	9672	(+)	NCTATAAAAR	TTTTAAAAAA
M00252	V$TATA_01	0.798528	9672	(+)	STATAAAWRNNNNNN	TTTTAAAAAATAATT
M00216	V$TATA_C	0.77634	9673	(+)	NCTATAAAAR	TTTAAAAAAT
M00252	V$TATA_01	0.785587	9709	(+)	STATAAAWRNNNNNN	GTAGAAATAACATCA
M00252	V$TATA_01	0.796752	9729	(+)	STATAAAWRNNNNNN	AGATTAATAGCCCCT
M00216	V$TATA_C	0.816477	9790	(+)	NCTATAAAAR	ACTTGAAAAT
M00216	V$TATA_C	0.767098	9818	(+)	NCTATAAAAR	GGTATCTAAG
M00252	V$TATA_01	0.806394	9819	(+)	STATAAAWRNNNNNN	GTATCTAAGCAACCG
M00252	V$TATA_01	0.774676	9874	(+)	STATAAAWRNNNNNN	GAACAAATACAAACT
M00216	V$TATA_C	0.800634	9908	(+)	NCTATAAAAR	TCATTATAAT
M00216	V$TATA_C	0.786639	9910	(+)	NCTATAAAAR	ATTATAATAA
M00252	V$TATA_01	0.790409	9911	(+)	STATAAAWRNNNNNN	TTATAATAATACAAA
M00252	V$TATA_01	0.773154	9919	(+)	STATAAAWRNNNNNN	ATACAAAATTGTATT
M00216	V$TATA_C	0.755743	9938	(+)	NCTATAAAAR	AGAATTAAAG
M00216	V$TATA_C	0.772379	9955	(+)	NCTATAAAAR	ATTTTAAATG
M00252	V$TATA_01	0.777721	9955	(+)	STATAAAWRNNNNNN	ATTTTAAATGGGGTT
M00252	V$TATA_01	0.829739	9956	(+)	STATAAAWRNNNNNN	TTTTAAATGGGGTTT
M00216	V$TATA_C	0.811724	9971	(+)	NCTATAAAAR	GCTTTTAAAT
M00216	V$TATA_C	0.767626	9972	(+)	NCTATAAAAR	CTTTTAAATA
M00252	V$TATA_01	0.784065	9973	(+)	STATAAAWRNNNNNN	TTTTAAATATGTTTT
M00216	V$TATA_C	0.751518	10019	(+)	NCTATAAAAR	TGAATAATAA
M00252	V$TATA_01	0.858412	10083	(+)	STATAAAWRNNNNNN	CTATTTATGCTGAGA
M00216	V$TATA_C	0.961183	10257	(+)	NCTATAAAAR	TCCATAAAAA
M00216	V$TATA_C	0.74254	10258	(+)	NCTATAAAAR	CCATAAAAAT
M00252	V$TATA_01	0.869576	10258	(+)	STATAAAWRNNNNNN	CCATAAAAATGCTTG
M00216	V$TATA_C	0.777132	10273	(+)	NCTATAAAAR	CTCATGAAAG

M00252	V$TATA_01	0.777468	10274	(+)	STATAAAWRNNNNNN	TCATGAAAGCACAGA
M00216	V$TATA_C	0.762873	10292	(+)	NCTATAAAAR	GCCAAATAAA
M00216	V$TATA_C	0.78611	10305	(+)	NCTATAAAAR	TCCATTTAAG
M00252	V$TATA_01	0.819589	10306	(+)	STATAAAWRNNNNNN	CCATTTAAGGAAACT
M00216	V$TATA_C	0.752839	10327	(+)	NCTATAAAAR	TCTGGAAAAA
M00216	V$TATA_C	0.752839	10378	(+)	NCTATAAAAR	TCTCTAAGAA
M00216	V$TATA_C	0.756007	10380	(+)	NCTATAAAAR	TCTAAGAAAG
M00216	V$TATA_C	0.814629	10433	(+)	NCTATAAAAR	TGTATGAAAT
M00252	V$TATA_01	0.820096	10434	(+)	STATAAAWRNNNNNN	GTATGAAATGGAAAT
M00216	V$TATA_C	0.890151	10536	(+)	NCTATAAAAR	CCAATAAAAT
M00252	V$TATA_01	0.774423	10537	(+)	STATAAAWRNNNNNN	CAATAAAATGTTCAT
M00252	V$TATA_01	0.773662	10706	(+)	STATAAAWRNNNNNN	GTATCAATTAGCACA
M00216	V$TATA_C	0.757064	10754	(+)	NCTATAAAAR	ACTGTAATAG
M00216	V$TATA_C	0.747821	10807	(+)	NCTATAAAAR	GCAATTAAAC
M00252	V$TATA_01	0.77696	10887	(+)	STATAAAWRNNNNNN	CTATGTGTAGGAAGA
M00216	V$TATA_C	0.789015	10969	(+)	NCTATAAAAR	TGTTTAAAGT
M00252	V$TATA_01	0.855113	10970	(+)	STATAAAWRNNNNNN	GTTTAAAGTCCAAGG
M00252	V$TATA_01	0.818828	10987	(+)	STATAAAWRNNNNNN	ATTTAAATATATCTG
M00252	V$TATA_01	0.775184	10991	(+)	STATAAAWRNNNNNN	AAATATATCTGTACT
M00216	V$TATA_C	0.762873	11178	(+)	NCTATAAAAR	TGAATGAAAG
M00223	V$STAT_01	0.846483	208	(-)	TTCCCRKAA	TTAGGTGAA
M00223	V$STAT_01	0.815478	271	(+)	TTCCCRKAA	TTCTCATAG
M00223	V$STAT_01	0.826821	300	(-)	TTCCCRKAA	TTTCAGTAA
M00223	V$STAT_01	0.796068	410	(+)	TTCCCRKAA	TTCTGGTAG
M00223	V$STAT_01	0.822536	410	(-)	TTCCCRKAA	TTCTGGTAG
M00223	V$STAT_01	0.8606	711	(+)	TTCCCRKAA	TTTCTGGAA
M00223	V$STAT_01	0.792034	711	(-)	TTCCCRKAA	TTTCTGGAA
M00223	V$STAT_01	0.81699	712	(+)	TTCCCRKAA	TTCTGGAAA
M00223	V$STAT_01	0.870179	712	(-)	TTCCCRKAA	TTCTGGAAA
M00223	V$STAT_01	0.905218	759	(+)	TTCCCRKAA	TTCTTATAA
M00225	V$STAT3_01	0.724258	845	(-)	NGNNATTTCCSGGAARTGNNN	AGTCATGTCATGGATATACCA
M00223	V$STAT_01	0.8606	911	(+)	TTCCCRKAA	TTCATGGAA
M00223	V$STAT_01	0.792034	911	(-)	TTCCCRKAA	TTCATGGAA

M0022 3	V$STAT_01	0.817746	1088	(-)	TTCCCRKAA	TAAGAGGAA
M0022 3	V$STAT_01	0.799849	1164	(+)	TTCCCRKAA	TTCTGACAA
M0022 3	V$STAT_01	0.826317	1164	(-)	TTCCCRKAA	TTCTGACAA
M0022 3	V$STAT_01	0.861356	1362	(+)	TTCCCRKAA	TTATTATAA
M0022 3	V$STAT_01	0.818503	1381	(-)	TTCCCRKAA	TGTTGGGAA
M0022 3	V$STAT_01	0.799597	1424	(+)	TTCCCRKAA	TTATTATCA
M0022 3	V$STAT_01	0.8243	1603	(-)	TTCCCRKAA	TACTGAGAA
M0022 3	V$STAT_01	0.791782	1838	(-)	TTCCCRKAA	TGACATGAA
M0022 4	V$STAT1_01	0.722542	1846	(-)	NNNSANTTCCGGGAANTGNSN	AGCTCTTTCCCTGCATCCGTT
M0022 3	V$STAT_01	0.835644	1852	(+)	TTCCCRKAA	TTCCCTGCA
M0022 3	V$STAT_01	0.8364	2108	(+)	TTCCCRKAA	TTCTCAAAA
M0022 3	V$STAT_01	0.8606	2220	(-)	TTCCCRKAA	TGACAAGAA
M0022 3	V$STAT_01	0.826569	2234	(+)	TTCCCRKAA	TTCAGATAA
M0022 3	V$STAT_01	0.80968	2234	(-)	TTCCCRKAA	TTCAGATAA
M0022 3	V$STAT_01	0.8606	2439	(-)	TTCCCRKAA	TGACAGTAA
M0022 3	V$STAT_01	0.80968	2449	(-)	TTCCCRKAA	TTACAAAAA
M0022 3	V$STAT_01	0.802622	2707	(-)	TTCCCRKAA	TTAGGAAAA
M0022 3	V$STAT_01	0.866398	2956	(+)	TTCCCRKAA	CTCCCAGAA
M0022 3	V$STAT_01	0.803126	2956	(-)	TTCCCRKAA	CTCCCAGAA
M0022 5	V$STAT3_01	0.723968	3115	(+)	NGNNATTTCCSGGAARTGNNN	TTGTTTTTCACAGAAATATCC
M0022 5	V$STAT3_01	0.735409	3115	(-)	NGNNATTTCCSGGAARTGNNN	TTGTTTTTCACAGAAATATCC
M0022 3	V$STAT_01	0.870179	3121	(+)	TTCCCRKAA	TTCACAGAA
M0022 3	V$STAT_01	0.81699	3121	(-)	TTCCCRKAA	TTCACAGAA
M0022 3	V$STAT_01	0.866398	3143	(+)	TTCCCRKAA	TACCCAGAA
M0022 3	V$STAT_01	0.80489	3143	(-)	TTCCCRKAA	TACCCAGAA
M0022 3	V$STAT_01	0.902445	3180	(+)	TTCCCRKAA	TTCTGAGAA
M0022 3	V$STAT_01	0.938997	3180	(-)	TTCCCRKAA	TTCTGAGAA
M0022 3	V$STAT_01	0.795815	3344	(+)	TTCCCRKAA	ATCTTAGAA
M0022 3	V$STAT_01	0.815982	3565	(+)	TTCCCRKAA	GTCCTCTAA
M0022 3	V$STAT_01	0.796068	3837	(+)	TTCCCRKAA	GTCTGGTAA
M0022 3	V$STAT_01	0.822536	3837	(-)	TTCCCRKAA	GTCTGGTAA
M0022 3	V$STAT_01	0.839677	3889	(+)	TTCCCRKAA	ATCCTAGAA
M0022 3	V$STAT_01	0.846483	3917	(+)	TTCCCRKAA	TTCACCTAA

M00223	V$STAT_01	0.792538	4004	(+)	TTCCCRKAA	TTTTTATAA
M00223	V$STAT_01	0.849761	4028	(-)	TTCCCRKAA	CTACAGGAA
M00223	V$STAT_01	0.888581	4091	(+)	TTCCCRKAA	TTCTTCTAA
M00223	V$STAT_01	0.826821	4113	(+)	TTCCCRKAA	TTCTTGAAA
M00223	V$STAT_01	0.81573	4190	(+)	TTCCCRKAA	TTCCAAGCA
M00223	V$STAT_01	0.812957	4190	(-)	TTCCCRKAA	TTCCAAGCA
M00223	V$STAT_01	0.819259	4487	(+)	TTCCCRKAA	TTAACATAA
M00223	V$STAT_01	0.812957	4598	(-)	TTCCCRKAA	CTCCAGTAA
M00224	V$STAT1_01	0.737362	4635	(-)	NNNSANTTCCGGGAANTGNSN	TGCCTTTTTATGGAAATAAAC
M00225	V$STAT3_01	0.710934	4635	(-)	NGNNATTTCCSGGAARTGNNN	TGCCTTTTTATGGAAATAAAC
M00223	V$STAT_01	0.863121	4642	(-)	TTCCCRKAA	TTATGGAAA
M00223	V$STAT_01	0.815478	4913	(+)	TTCCCRKAA	TTCTCATAG
M00223	V$STAT_01	0.800605	4977	(-)	TTCCCRKAA	TAAGGAGAA
M00223	V$STAT_01	0.822536	5099	(-)	TTCCCRKAA	TTCTGAGAG
M00223	V$STAT_01	0.802622	5145	(-)	TTCCCRKAA	TTAAGATAA
M00224	V$STAT1_01	0.753334	5227	(-)	NNNSANTTCCGGGAANTGNSN	TTATACTTCCCATAAACTTTC
M00225	V$STAT3_01	0.720782	5227	(+)	NGNNATTTCCSGGAARTGNNN	TTATACTTCCCATAAACTTTC
M00223	V$STAT_01	0.9758	5233	(+)	TTCCCRKAA	TTCCCATAA
M00223	V$STAT_01	0.875725	5233	(-)	TTCCCRKAA	TTCCCATAA
M00223	V$STAT_01	0.8243	5266	(-)	TTCCCRKAA	TACTGAGAA
M00223	V$STAT_01	0.795815	5449	(-)	TTCCCRKAA	CTCTAAGAA
M00223	V$STAT_01	0.826821	5486	(+)	TTCCCRKAA	TTCTTGAAA
M00223	V$STAT_01	0.841442	5648	(-)	TTCCCRKAA	TACTAGGAA
M00225	V$STAT3_01	0.789283	5717	(-)	NGNNATTTCCSGGAARTGNNN	AAACATTTCCTTTAAATACCC
M00223	V$STAT_01	0.863625	5723	(+)	TTCCCRKAA	TTCCTTTAA
M00223	V$STAT_01	0.826821	5793	(-)	TTCCCRKAA	TTCAAAGAA
M00223	V$STAT_01	0.792538	6004	(+)	TTCCCRKAA	TTAATATAA
M00223	V$STAT_01	0.819763	6062	(-)	TTCCCRKAA	TTAAAAGAA
M00223	V$STAT_01	0.792538	6093	(+)	TTCCCRKAA	TTATCACAA
M00223	V$STAT_01	0.888077	6279	(+)	TTCCCRKAA	TTATCATAA
M00223	V$STAT_01	0.851525	6279	(-)	TTCCCRKAA	TTATCATAA
M00223	V$STAT_01	0.8243	6313	(+)	TTCCCRKAA	TTCTCAGGA
M00223	V$STAT_01	0.795059	6469	(-)	TTCCCRKAA	AGCCAGGAA

M00223	V$STAT_01	0.79758	6715	(+)	TTCCCRKAA	TTACTAGTA
M00224	V$STAT1_01	0.735057	6774	(-)	NNNSANTTCCGGGAANTGNSN	GGTCCTTTCCCTGATTTTCAT
M00225	V$STAT3_01	0.718465	6774	(+)	NGNNATTTCCSGGAARTGNNN	GGTCCTTTCCCTGATTTTCAT
M00225	V$STAT3_01	0.721651	6774	(-)	NGNNATTTCCSGGAARTGNNN	GGTCCTTTCCCTGATTTTCAT
M00223	V$STAT_01	0.878498	6894	(+)	TTCCCRKAA	TTATTGTAA
M00223	V$STAT_01	0.826569	6894	(-)	TTCCCRKAA	TTATTGTAA
M00225	V$STAT3_01	0.728892	6969	(+)	NGNNATTTCCSGGAARTGNNN	TCCTGCTTCCAGGACACCTGT
M00225	V$STAT3_01	0.722375	6969	(-)	NGNNATTTCCSGGAARTGNNN	TCCTGCTTCCAGGACACCTGT
M00223	V$STAT_01	0.856819	6975	(-)	TTCCCRKAA	TTCCAGGAC
M00223	V$STAT_01	0.843459	7008	(+)	TTCCCRKAA	TTCTTATCA
M00223	V$STAT_01	0.808924	7080	(+)	TTCCCRKAA	TTCCTGCCA
M00224	V$STAT1_01	0.774741	7507	(+)	NNNSANTTCCGGGAANTGNSN	CGTGACTTAGGGGAAA-GAAAA
M00224	V$STAT1_01	0.743455	7507	(-)	NNNSANTTCCGGGAANTGNSN	CGTGACTTAGGGGAAA-GAAAA
M00225	V$STAT3_01	0.724403	7507	(+)	NGNNATTTCCSGGAARTGNNN	CGTGACTTAGGGGAAA-GAAAA
M00225	V$STAT3_01	0.751195	7507	(-)	NGNNATTTCCSGGAARTGNNN	CGTGACTTAGGGGAAA-GAAAA
M00223	V$STAT_01	0.806907	7513	(+)	TTCCCRKAA	TTAGGGGAA
M00223	V$STAT_01	0.959163	7513	(-)	TTCCCRKAA	TTAGGGGAA
M00223	V$STAT_01	0.81573	7620	(+)	TTCCCRKAA	TTCCAAGCA
M00223	V$STAT_01	0.812957	7620	(-)	TTCCCRKAA	TTCCAAGCA
M00224	V$STAT1_01	0.75284	7669	(+)	NNNSANTTCCGGGAANTGNSN	CTGGGGTTCTGGGAA-GCCCAG
M00224	V$STAT1_01	0.73835	7669	(-)	NNNSANTTCCGGGAANTGNSN	CTGGGGTTCTGGGAA-GCCCAG
M00225	V$STAT3_01	0.746271	7669	(+)	NGNNATTTCCSGGAARTGNNN	CTGGGGTTCTGGGAA-GCCCAG
M00225	V$STAT3_01	0.725127	7669	(-)	NGNNATTTCCSGGAARTGNNN	CTGGGGTTCTGGGAA-GCCCAG
M00223	V$STAT_01	0.919587	7675	(+)	TTCCCRKAA	TTCTGGGAA
M00223	V$STAT_01	0.982859	7675	(-)	TTCCCRKAA	TTCTGGGAA
M00223	V$STAT_01	0.792538	7721	(+)	TTCCCRKAA	TTATCACAA
M00223	V$STAT_01	0.795815	7750	(-)	TTCCCRKAA	TTCTAGTCA
M00223	V$STAT_01	0.802622	8289	(+)	TTCCCRKAA	TTTTCCTAA
M00224	V$STAT1_01	0.726	8303	(-)	NNNSANTTCCGGGAANTGNSN	TTGCCCTTCCCAGATTTTGAT
M00223	V$STAT_01	0.866398	8309	(+)	TTCCCRKAA	TTCCCAGAT
M00223	V$STAT_01	0.803126	8309	(-)	TTCCCRKAA	TTCCCAGAT
M00223	V$STAT_01	0.843711	8348	(+)	TTCCCRKAA	TTACAGTAA
M00223	V$STAT_01	0.922359	8348	(-)	TTCCCRKAA	TTACAGTAA

M00223	V$STAT_01	0.802622	8454	(+)	TTCCCRKAA	TTAACCTAA
M00223	V$STAT_01	0.863625	8500	(+)	TTCCCRKAA	TTCCTTTAA
M00223	V$STAT_01	0.819763	8511	(-)	TTCCCRKAA	TTGGGAGAA
M00224	V$STAT1_01	0.816565	8561	(+)	NNNSANTTCCGGGAANTGNSN	TGGAGTTTCTGGGAATTCAGT
M00224	V$STAT1_01	0.737362	8561	(-)	NNNSANTTCCGGGAANTGNSN	TGGAGTTTCTGGGAATTCAGT
M00225	V$STAT3_01	0.825199	8561	(+)	NGNNATTTCCSGGAARTGNNN	TGGAGTTTCTGGGAATTCAGT
M00225	V$STAT3_01	0.777987	8561	(-)	NGNNATTTCCSGGAARTGNNN	TGGAGTTTCTGGGAATTCAGT
M00223	V$STAT_01	0.919587	8567	(+)	TTCCCRKAA	TTCTGGGAA
M00223	V$STAT_01	0.982859	8567	(-)	TTCCCRKAA	TTCTGGGAA
M00223	V$STAT_01	0.805899	8734	(-)	TTCCCRKAA	TTACAGTTA
M00224	V$STAT1_01	0.746583	8770	(+)	NNNSANTTCCGGGAANTGNSN	GTATTCTTACCTGAAATGTAA
M00224	V$STAT1_01	0.730611	8770	(-)	NNNSANTTCCGGGAANTGNSN	GTATTCTTACCTGAAATGTAA
M00225	V$STAT3_01	0.740478	8770	(-)	NGNNATTTCCSGGAARTGNNN	GTATTCTTACCTGAAATGTAA
M00223	V$STAT_01	0.853542	8776	(+)	TTCCCRKAA	TTACCTGAA
M00223	V$STAT_01	0.843711	8776	(-)	TTCCCRKAA	TTACCTGAA
M00223	V$STAT_01	0.812957	8845	(-)	TTCCCRKAA	TTCCAGTAC
M00223	V$STAT_01	0.826317	8948	(+)	TTCCCRKAA	TTAGCAGAA
M00223	V$STAT_01	0.87875	8948	(-)	TTCCCRKAA	TTAGCAGAA
M00223	V$STAT_01	0.796068	8992	(+)	TTCCCRKAA	ATCTGGTAA
M00223	V$STAT_01	0.822536	8992	(-)	TTCCCRKAA	ATCTGGTAA
M00223	V$STAT_01	0.826821	9042	(-)	TTCCCRKAA	TGAGAAGAA
M00223	V$STAT_01	0.790522	9098	(-)	TTCCCRKAA	TCATAGTAA
M00223	V$STAT_01	0.853542	9338	(+)	TTCCCRKAA	TTGCTGTAA
M00223	V$STAT_01	0.819763	9483	(+)	TTCCCRKAA	TTCATCTAA
M00223	V$STAT_01	0.805899	9619	(+)	TTCCCRKAA	TCACTGTAA
M00223	V$STAT_01	0.807663	9640	(-)	TTCCCRKAA	TAACAGTAA
M00223	V$STAT_01	0.8243	9754	(-)	TTCCCRKAA	TCCTGAGAA
M00223	V$STAT_01	0.826821	9788	(+)	TTCCCRKAA	TTACTTGAA
M00223	V$STAT_01	0.804638	9799	(-)	TTCCCRKAA	TGCTGGGAT
M00223	V$STAT_01	0.861356	9911	(-)	TTCCCRKAA	TTATAATAA
M00223	V$STAT_01	0.826821	9987	(-)	TTCCCRKAA	TTTCAAGAA
M00223	V$STAT_01	0.8606	10061	(+)	TTCCCRKAA	TTACTGTCA
M00223	V$STAT_01	0.819763	10142	(+)	TTCCCRKAA	TTACTTTAA

M00224	V$STAT1_01	0.745266	10223	(-)	NNNSANTTCCGGGAANTGNSN	CTTTATTTTCCCAAATTCACT
M00224	V$STAT1_01	0.755804	10304	(+)	NNNSANTTCCGGGAANTGNSN	TTCCATTTAAGGAAACTGTAG
M00224	V$STAT1_01	0.722707	10304	(-)	NNNSANTTCCGGGAANTGNSN	TTCCATTTAAGGAAACTGTAG
M00223	V$STAT_01	0.795815	10379	(-)	TTCCCRKAA	CTCTAAGAA
M00224	V$STAT1_01	0.767166	10406	(-)	NNNSANTTCCGGGAANTGNSN	AATCTTATCCCGGAACTTGTG
M00225	V$STAT3_01	0.730775	10406	(-)	NGNNATTTCCSGGAARTGNNN	AATCTTATCCCGGAACTTGTG
M00223	V$STAT_01	0.883539	10412	(+)	TTCCCRKAA	ATCCCGGAA
M00223	V$STAT_01	0.846988	10412	(-)	TTCCCRKAA	ATCCCGGAA
M00223	V$STAT_01	0.915301	10558	(+)	TTCCCRKAA	TTCTCCTAA
M00223	V$STAT_01	0.799849	10610	(+)	TTCCCRKAA	TTCTGACAA
M00223	V$STAT_01	0.826317	10610	(-)	TTCCCRKAA	TTCTGACAA
M00223	V$STAT_01	0.805899	10752	(+)	TTCCCRKAA	TGACTGTAA
M00223	V$STAT_01	0.8364	10802	(-)	TTCCCRKAA	TTATAGCAA
M00224	V$STAT1_01	0.786267	10827	(+)	NNNSANTTCCGGGAANTGNSN	TAAGACTTTAGGGAAAA-GAGC
M00225	V$STAT3_01	0.737002	10827	(-)	NGNNATTTCCSGGAARTGNNN	TAAGACTTTAGGGAAAA-GAGC
M00223	V$STAT_01	0.794807	10833	(-)	TTCCCRKAA	TTTAGGGAA
M00223	V$STAT_01	0.846483	10834	(-)	TTCCCRKAA	TTAGGGAAA
M00223	V$STAT_01	0.795815	11028	(-)	TTCCCRKAA	ATCTAAGAA
M00223	V$STAT_01	0.822536	11120	(+)	TTCCCRKAA	TGCTCAGAA
M00223	V$STAT_01	0.840686	11120	(-)	TTCCCRKAA	TGCTCAGAA
**CSN1S2_*Camelus ferus***						
M00135	V$OCT1_01	0.765548	2	(+)	NNNNWTATGCAAATNTNNN	GGAAATAAGGTAATATTTT
M00162	V$OCT1_06	0.847266	5	(+)	CWNAWTKWSATRYN	AATAAGGTAATATT
M00138	V$OCT1_04	0.878712	9	(-)	NNNNNNNWATGCAAATNNNWNNW	AGGTAATATTTTCATTTATCTTC
M00135	V$OCT1_01	0.788821	11	(-)	NNNNWTATGCAAATNTNNN	GTAATATTTTCATTTATCT
M00162	V$OCT1_06	0.9375	13	(+)	CWNAWTKWSATRYN	AATATTTTCATTTA
M00195	V$OCT1_Q6	0.822325	13	(-)	NNNNATGCAAATNAN	AATATTTTCATTTAT
M00248	V$OCT1_07	0.812381	13	(+)	TNTATGNTAATT	AATATTTTCATT
M00138	V$OCT1_04	0.818277	49	(+)	NNNNNNNWATGCAAATNNNWNNW	TTATGACTTTGTAAAACTTAATA
M00162	V$OCT1_06	0.800391	53	(+)	CWNAWTKWSATRYN	GACTTTGTAAAACT
M00137	V$OCT1_03	0.840379	63	(+)	NNNRTAATNANNN	AACTTAATAACAA
M00136	V$OCT1_02	0.788305	115	(+)	NNGAATATKCANNNN	CTTAAGATGCACTGA
M00162	V$OCT1_06	0.842187	140	(-)	CWNAWTKWSATRYN	GATAACTAATTGTT

M00137	V$OCT1_03	0.840379	143	(-)	NNNRTAATNANNN	AACTAATTGTTTG
M00162	V$OCT1_06	0.804297	163	(+)	CWNAWTKWSATRYN	CTGATTTACCTCCT
M00195	V$OCT1_Q6	0.836517	163	(-)	NNNNATGCAAATNAN	CTGATTTACCTCCTT
M00138	V$OCT1_04	0.797365	179	(-)	NNNNNNNWATGCAAATNNNWNNW	TCAGTGCTTCTGAATACAATTAA
M00195	V$OCT1_Q6	0.826965	188	(+)	NNNNATGCAAATNAN	CTGAATACAATTAAC
M00162	V$OCT1_06	0.847656	189	(-)	CWNAWTKWSATRYN	TGAATACAATTAAC
M00135	V$OCT1_01	0.762304	192	(-)	NNNNWTATGCAAATNTNNN	ATACAATTAACGTATAGAT
M00248	V$OCT1_07	0.890396	196	(-)	TNTATGNTAATT	AATTAACGTATA
M00135	V$OCT1_01	0.756772	301	(+)	NNNNWTATGCAAATNTNNN	TTCTACCTGCTAATTCTTC
M00248	V$OCT1_07	0.843121	304	(+)	TNTATGNTAATT	TACCTGCTAATT
M00137	V$OCT1_03	0.887791	317	(-)	NNNRTAATNANNN	TTCTCCTTATGAG
M00162	V$OCT1_06	0.852734	364	(+)	CWNAWTKWSATRYN	CTGATTTAAAAATT
M00135	V$OCT1_01	0.734643	379	(-)	NNNNWTATGCAAATNTNNN	GTTCCTCTTGCATTTTGCA
M00136	V$OCT1_02	0.801029	383	(-)	NNGAATATKCANNNN	CTCTTGCATTTTGCA
M00138	V$OCT1_04	0.864074	384	(-)	NNNNNNNWATGCAAATNNNWNNW	TCTTGCATTTTGCATTTCTGATA
M00135	V$OCT1_01	0.812476	386	(-)	NNNNWTATGCAAATNTNNN	TTGCATTTTGCATTTCTGA
M00162	V$OCT1_06	0.808594	388	(+)	CWNAWTKWSATRYN	GCATTTTGCATTTC
M00195	V$OCT1_Q6	0.853712	388	(-)	NNNNATGCAAATNAN	GCATTTTGCATTTCT
M00162	V$OCT1_06	0.834375	394	(-)	CWNAWTKWSATRYN	TGCATTTCTGATAG
M00162	V$OCT1_06	0.852734	401	(+)	CWNAWTKWSATRYN	CTGATAGTCATTCT
M00195	V$OCT1_Q6	0.823144	430	(+)	NNNNATGCAAATNAN	AGAGTTGTAAATAAT
M00137	V$OCT1_03	0.840774	433	(+)	NNNRTAATNANNN	GTTGTAAATAATA
M00138	V$OCT1_04	0.842325	436	(-)	NNNNNNNWATGCAAATNNNWNNW	GTAAATAATATGCATATGTCAAT
M00162	V$OCT1_06	0.812109	436	(+)	CWNAWTKWSATRYN	GTAAATAATATGCA
M00138	V$OCT1_04	0.880176	437	(+)	NNNNNNNWATGCAAATNNNWNNW	TAAATAATATGCATATGTCAATA
M00135	V$OCT1_01	0.830217	438	(-)	NNNNWTATGCAAATNTNNN	AAATAATATGCATATGTCA
M00135	V$OCT1_01	0.85578	439	(+)	NNNNWTATGCAAATNTNNN	AATAATATGCATATGTCAA
M00136	V$OCT1_02	0.929616	439	(+)	NNGAATATKCANNNN	AATAATATGCATATG
M00161	V$OCT1_05	0.901046	440	(+)	MKNATTTGCATAYY	ATAATATGCATATG
M00162	V$OCT1_06	0.826562	440	(+)	CWNAWTKWSATRYN	ATAATATGCATATG
M00195	V$OCT1_Q6	0.81714	440	(-)	NNNNATGCAAATNAN	ATAATATGCATATGT
M00136	V$OCT1_02	0.842989	442	(-)	NNGAATATKCANNNN	AATATGCATATGTCA
M00162	V$OCT1_06	0.834375	442	(-)	CWNAWTKWSATRYN	AATATGCATATGTC

M00248	V$OCT1_07	0.828493	442	(-)	TNTATGNTAATT	AATATGCATATG
M00138	V$OCT1_04	0.847135	443	(+)	NNNNNNNWATGCAAATNNNWNNW	ATATGCATATGTCAATAATATAT
M00135	V$OCT1_01	0.774895	445	(+)	NNNNWTATGCAAATNTNNN	ATGCATATGTCAATAATAT
M00136	V$OCT1_02	0.794532	445	(+)	NNGAATATKCANNNN	ATGCATATGTCAATA
M00195	V$OCT1_Q6	0.832424	447	(+)	NNNNATGCAAATNAN	GCATATGTCAATAAT
M00162	V$OCT1_06	0.9375	448	(-)	CWNAWTKWSATRYN	CATATGTCAATAAT
M00138	V$OCT1_04	0.834379	454	(+)	NNNNNNNWATGCAAATNNNWNNW	TCAATAATATATAAATAATATAT
M00138	V$OCT1_04	0.814513	455	(-)	NNNNNNNWATGCAAATNNNWNNW	CAATAATATATAAATAATATATA
M00135	V$OCT1_01	0.750858	456	(+)	NNNNWTATGCAAATNTNNN	AATAATATATAAATAATAT
M00138	V$OCT1_04	0.799665	456	(+)	NNNNNNNWATGCAAATNNNWNNW	AATAATATATAAATAATATATAA
M00195	V$OCT1_Q6	0.827784	458	(+)	NNNNATGCAAATNAN	TAATATATAAATAAT
M00162	V$OCT1_06	0.873437	459	(-)	CWNAWTKWSATRYN	AATATATAAATAAT
M00162	V$OCT1_06	0.804297	464	(+)	CWNAWTKWSATRYN	ATAAATAATATATA
M00138	V$OCT1_04	0.805312	465	(+)	NNNNNNNWATGCAAATNNNWNNW	TAAATAATATATAAATTTTTTTC
M00138	V$OCT1_04	0.812631	466	(-)	NNNNNNNWATGCAAATNNNWNNW	AAATAATATATAAATTTTTTTCA
M00248	V$OCT1_07	0.793513	466	(-)	TNTATGNTAATT	AAATAATATATA
M00135	V$OCT1_01	0.79359	467	(+)	NNNNWTATGCAAATNTNNN	AATAATATATAAATTTTTT
M00162	V$OCT1_06	0.834375	470	(+)	CWNAWTKWSATRYN	AATATATAAATTTT
M00162	V$OCT1_06	0.88125	470	(-)	CWNAWTKWSATRYN	AATATATAAATTTT
M00248	V$OCT1_07	0.848209	470	(+)	TNTATGNTAATT	AATATATAAATT
M00138	V$OCT1_04	0.838352	475	(-)	NNNNNNNWATGCAAATNNNWNNW	ATAAATTTTTTTCATTACTGTAT
M00162	V$OCT1_06	0.813672	479	(+)	CWNAWTKWSATRYN	ATTTTTTTCATTAC
M00137	V$OCT1_03	0.888186	483	(-)	NNNRTAATNANNN	TTTTCATTACTGT
M00162	V$OCT1_06	0.813672	514	(+)	CWNAWTKWSATRYN	GTACCTGGCATAGT
M00162	V$OCT1_06	0.833984	525	(-)	CWNAWTKWSATRYN	AGTAGCAAATATTC
M00136	V$OCT1_02	0.808067	526	(-)	NNGAATATKCANNNN	GTAGCAAATATTCCT
M00136	V$OCT1_02	0.831348	529	(+)	NNGAATATKCANNNN	GCAAATATTCCTTAA
M00162	V$OCT1_06	0.808203	532	(-)	CWNAWTKWSATRYN	AATATTCCTTAATT
M00162	V$OCT1_06	0.853125	539	(+)	CWNAWTKWSATRYN	CTTAATTTAATTAG
M00248	V$OCT1_07	0.793301	542	(-)	TNTATGNTAATT	AATTTAATTAGT
M00137	V$OCT1_03	0.862505	543	(-)	NNNRTAATNANNN	ATTTAATTAGTCA
M00137	V$OCT1_03	0.84196	555	(-)	NNNRTAATNANNN	AATTAATTAACCA
M00138	V$OCT1_04	0.840652	633	(-)	NNNNNNNWATGCAAATNNNWNNW	ACCAAGATTTTACATTATGTTGT

M00135	V$OCT1_01	0.759634	635	(-)	NNNNWTATGCAAATNTNNN	CAAGATTTTACATTATGTT
M00162	V$OCT1_06	0.821484	637	(+)	CWNAWTKWSATRYN	AGATTTTACATTAT
M00162	V$OCT1_06	0.860547	638	(-)	CWNAWTKWSATRYN	GATTTTACATTATG
M00248	V$OCT1_07	0.793089	639	(-)	TNTATGNTAATT	ATTTTACATTAT
M00137	V$OCT1_03	0.855393	641	(-)	NNNRTAATNANNN	TTTACATTATGTT
M00138	V$OCT1_04	0.780636	646	(+)	NNNNNNNWATGCAAATNNNWNNW	ATTATGTTGTGTTAATTTCTCCT
M00135	V$OCT1_01	0.753338	648	(+)	NNNNWTATGCAAATNTNNN	TATGTTGTGTTAATTTCTC
M00248	V$OCT1_07	0.857325	651	(+)	TNTATGNTAATT	GTTGTGTTAATT
M00137	V$OCT1_03	0.86211	662	(-)	NNNRTAATNANNN	TTCTCCTTATAAA
M00137	V$OCT1_03	0.840379	667	(+)	NNNRTAATNANNN	CTTATAAAGAACT
M00138	V$OCT1_04	0.798829	671	(-)	NNNNNNNWATGCAAATNNNWNNW	TAAAGAACTTGTCATATTCACAT
M00135	V$OCT1_01	0.732736	673	(-)	NNNNWTATGCAAATNTNNN	AAGAACTTGTCATATTCAC
M00162	V$OCT1_06	0.924219	675	(+)	CWNAWTKWSATRYN	GAACTTGTCATATT
M00162	V$OCT1_06	0.857422	676	(-)	CWNAWTKWSATRYN	AACTTGTCATATTC
M00136	V$OCT1_02	0.770709	677	(-)	NNGAATATKCANNNN	ACTTGTCATATTCAC
M00138	V$OCT1_04	0.809912	677	(-)	NNNNNNNWATGCAAATNNNWNNW	ACTTGTCATATTCACATCCATGT
M00136	V$OCT1_02	0.835409	680	(+)	NNGAATATKCANNNN	TGTCATATTCACATC
M00162	V$OCT1_06	0.838281	683	(+)	CWNAWTKWSATRYN	CATATTCACATCCA
M00162	V$OCT1_06	0.808594	689	(-)	CWNAWTKWSATRYN	CACATCCATGTGTT
M00162	V$OCT1_06	0.800391	692	(+)	CWNAWTKWSATRYN	ATCCATGTGTTTCT
M00138	V$OCT1_04	0.783354	725	(-)	NNNNNNNWATGCAAATNNNWNNW	AGTCTTCATTTCCCTTTCCCAAT
M00137	V$OCT1_03	0.864085	741	(-)	NNNRTAATNANNN	TCCCAATTATTTG
M00162	V$OCT1_06	0.833984	745	(+)	CWNAWTKWSATRYN	AATTATTTGGTACA
M00138	V$OCT1_04	0.834588	752	(+)	NNNNNNNWATGCAAATNNNWNNW	TGGTACAAATATAAATTAATATA
M00138	V$OCT1_04	0.830406	753	(-)	NNNNNNNWATGCAAATNNNWNNW	GGTACAAATATAAATTAATATAT
M00195	V$OCT1_Q6	0.814956	756	(+)	NNNNATGCAAATNAN	ACAAATATAAATTAA
M00138	V$OCT1_04	0.856754	758	(+)	NNNNNNNWATGCAAATNNNWNNW	AAATATAAATTAATATATAATTA
M00138	V$OCT1_04	0.823296	759	(-)	NNNNNNNWATGCAAATNNNWNNW	AATATAAATTAATATATAATTAA
M00135	V$OCT1_01	0.755246	761	(-)	NNNNWTATGCAAATNTNNN	TATAAATTAATATATAATT
M00138	V$OCT1_04	0.847762	762	(+)	NNNNNNNWATGCAAATNNNWNNW	ATAAATTAATATATAATTAATGA
M00248	V$OCT1_07	0.891456	765	(-)	TNTATGNTAATT	AATTAATATATA
M00162	V$OCT1_06	0.88125	769	(-)	CWNAWTKWSATRYN	AATATATAATTAAT
M00137	V$OCT1_03	0.845911	771	(+)	NNNRTAATNANNN	TATATAATTAATG

M00137	V$OCT1_03	0.841565	772	(-)	NNNRTAATNANNN	ATATAATTAATGA
M00137	V$OCT1_03	0.878704	775	(+)	NNNRTAATNANNN	TAATTAATGAGGA
M00162	V$OCT1_06	0.821094	787	(+)	CWNAWTKWSATRYN	AAATATGGGAAATA
M00162	V$OCT1_06	0.898437	796	(+)	CWNAWTKWSATRYN	AAATATGTGATTTC
M00162	V$OCT1_06	0.812109	797	(-)	CWNAWTKWSATRYN	AATATGTGATTTCA
M00162	V$OCT1_06	0.81875	804	(-)	CWNAWTKWSATRYN	GATTTCAAAAAGAG
M00137	V$OCT1_03	0.863295	859	(+)	NNNRTAATNANNN	TAAGTAAACATGA
M00162	V$OCT1_06	0.90625	864	(+)	CWNAWTKWSATRYN	AAACATGAAATTTT
M00162	V$OCT1_06	0.966016	865	(-)	CWNAWTKWSATRYN	AACATGAAATTTTT
M00138	V$OCT1_04	0.801547	867	(-)	NNNNNNNWATGCAAATNNNWNNW	CATGAAATTTTTCATTTTTACCT
M00135	V$OCT1_01	0.756581	869	(-)	NNNNWTATGCAAATNTNNN	TGAAATTTTTCATTTTTAC
M00162	V$OCT1_06	0.911328	871	(+)	CWNAWTKWSATRYN	AAATTTTTCATTTT
M00162	V$OCT1_06	0.855078	872	(-)	CWNAWTKWSATRYN	AATTTTTCATTTTT
M00137	V$OCT1_03	0.864876	878	(-)	NNNRTAATNANNN	TCATTTTTACCTG
M00162	V$OCT1_06	0.862891	879	(+)	CWNAWTKWSATRYN	CATTTTTACCTGCC
M00195	V$OCT1_Q6	0.840066	904	(+)	NNNNATGCAAATNAN	GTTCTTGCAAAGCAT
M00135	V$OCT1_01	0.736169	911	(+)	NNNNWTATGCAAATNTNNN	CAAAGCATGTAAGTATAAT
M00162	V$OCT1_06	0.896875	914	(-)	CWNAWTKWSATRYN	AGCATGTAAGTATA
M00137	V$OCT1_03	0.914658	922	(+)	NNNRTAATNANNN	AGTATAATAAGAT
M00162	V$OCT1_06	0.825	924	(+)	CWNAWTKWSATRYN	TATAATAAGATATT
M00138	V$OCT1_04	0.79151	926	(-)	NNNNNNNWATGCAAATNNNWNNW	TAATAAGATATTTATAAGTACCT
M00138	V$OCT1_04	0.783772	947	(+)	NNNNNNNWATGCAAATNNNWNNW	CTCCTGTTATTTAAATATTAGAA
M00138	V$OCT1_04	0.835425	948	(-)	NNNNNNNWATGCAAATNNNWNNW	TCCTGTTATTTAAATATTAGAAG
M00135	V$OCT1_01	0.767837	949	(+)	NNNNWTATGCAAATNTNNN	CCTGTTATTTAAATATTAG
M00135	V$OCT1_01	0.775849	950	(-)	NNNNWTATGCAAATNTNNN	CTGTTATTTAAATATTAGA
M00162	V$OCT1_06	0.929688	952	(+)	CWNAWTKWSATRYN	GTTATTTAAATATT
M00162	V$OCT1_06	0.847656	952	(-)	CWNAWTKWSATRYN	GTTATTTAAATATT
M00195	V$OCT1_Q6	0.818231	952	(-)	NNNNATGCAAATNAN	GTTATTTAAATATTA
M00136	V$OCT1_02	0.805089	954	(-)	NNGAATATKCANNNN	TATTTAAATATTAGA
M00162	V$OCT1_06	0.829297	974	(+)	CWNAWTKWSATRYN	GACTCTTTTATGCA
M00138	V$OCT1_04	0.792765	1028	(+)	NNNNNNNWATGCAAATNNNWNNW	GTGTGAACTTTTATAATCAAAGA
M00137	V$OCT1_03	0.888977	1037	(+)	NNNRTAATNANNN	TTTATAATCAAAG
M00162	V$OCT1_06	0.826562	1039	(-)	CWNAWTKWSATRYN	TATAATCAAAGATG

M00059	V$YY1_01	0.842071	15	(+)	NNNNNCCATNTWNNNWN	TATTTTCATTTATCTTC
M00059	V$YY1_01	0.772608	113	(-)	NNNNNCCATNTWNNNWN	ACCTTAAGATGCACTGA
M00059	V$YY1_01	0.881062	336	(+)	NNNNNCCATNTWNNNWN	AGACTCCATGTTTTGGC
M00059	V$YY1_01	0.81979	677	(+)	NNNNNCCATNTWNNNWN	ACTTGTCATATTCACAT
M00059	V$YY1_01	0.782438	725	(+)	NNNNNCCATNTWNNNWN	AGTCTTCATTTCCCTTT
M00059	V$YY1_01	0.78768	731	(+)	NNNNNCCATNTWNNNWN	CATTTCCCTTTCCCAAT
M00059	V$YY1_01	0.803408	783	(-)	NNNNNCCATNTWNNNWN	GAGGAAATATGGGAAAT
M00059	V$YY1_01	0.820446	834	(+)	NNNNNCCATNTWNNNWN	CAGCCCCATATCTGTTC
M00059	V$YY1_01	0.821429	860	(-)	NNNNNCCATNTWNNNWN	AAGTAAACATGAAATTT
M00059	V$YY1_01	0.775557	910	(+)	NNNNNCCATNTWNNNWN	GCAAAGCATGTAAGTAT
M00059	V$YY1_01	0.783748	984	(-)	NNNNNCCATNTWNNNWN	TGCAGACAATGGCAAGG
M00069	V$YY1_02	0.863442	1040	(-)	NNNCGGCCATCTTGNCTSNW	ATAATCAAAGATGGCACTGG
M00059	V$YY1_01	0.867955	1042	(-)	NNNNNCCATNTWNNNWN	AATCAAAGATGGCACTG
M00203	V$GATA_C	0.916434	19	(-)	NGATAAGNMNN	TTCATTTATCT
M00126	V$GATA1_02	0.798438	20	(-)	NNNNNGATANKGNN	TCATTTATCTTCCT
M00127	V$GATA1_03	0.935816	20	(-)	RNSNNGATAANNGN	TCATTTATCTTCCT
M00128	V$GATA1_04	0.902574	20	(-)	NNCWGATARNNNN	TCATTTATCTTCC
M00075	V$GATA1_01	0.817868	22	(-)	SNNGATNNNN	ATTTATCTTC
M00076	V$GATA2_01	0.853857	22	(-)	NNNGATRNNN	ATTTATCTTC
M00077	V$GATA3_01	0.846699	22	(-)	NNGATARNG	ATTTATCTT
M00126	V$GATA1_02	0.7775	93	(+)	NNNNNGATANKGNN	TTAAAGAAACTAGT
M00075	V$GATA1_01	0.786278	117	(+)	SNNGATNNNN	TAAGATGCAC
M00076	V$GATA2_01	0.833559	117	(+)	NNNGATRNNN	TAAGATGCAC
M00075	V$GATA1_01	0.862784	129	(+)	SNNGATNNNN	AGAGATTGTT
M00076	V$GATA2_01	0.842129	129	(+)	NNNGATRNNN	AGAGATTGTT
M00077	V$GATA3_01	0.8786	130	(+)	NNGATARNG	GAGATTGTT
M00075	V$GATA1_01	0.779862	137	(+)	SNNGATNNNN	TTCGATAACT
M00076	V$GATA2_01	0.813712	137	(+)	NNNGATRNNN	TTCGATAACT
M00075	V$GATA1_01	0.774432	162	(+)	SNNGATNNNN	ACTGATTTAC
M00126	V$GATA1_02	0.77375	203	(+)	NNNNNGATANKGNN	GTATAGATGTTGGA
M00075	V$GATA1_01	0.786278	205	(+)	SNNGATNNNN	ATAGATGTTG
M00077	V$GATA3_01	0.82366	206	(+)	NNGATARNG	TAGATGTTG
M00075	V$GATA1_01	0.849457	232	(+)	SNNGATNNNN	CAAGATTCAG

M00076	V$GATA2_01	0.823184	232	(+)	NNNGATRNNN	CAAGATTCAG
M00077	V$GATA3_01	0.828977	233	(+)	NNGATARNG	AAGATTCAG
M00076	V$GATA2_01	0.788453	244	(-)	NNNGATRNNN	GCCTGTCCTA
M00075	V$GATA1_01	0.771471	262	(+)	SNNGATNNNN	TGGGATCAGC
M00075	V$GATA1_01	0.851431	262	(-)	SNNGATNNNN	TGGGATCAGC
M00076	V$GATA2_01	0.840325	262	(+)	NNNGATRNNN	TGGGATCAGC
M00076	V$GATA2_01	0.841678	262	(-)	NNNGATRNNN	TGGGATCAGC
M00127	V$GATA1_03	0.795198	270	(-)	RNSNNGATAANNGN	GCAATTCTCCTTGT
M00127	V$GATA1_03	0.791034	313	(-)	RNSNNGATAANNGN	ATTCTTCTCCTTAT
M00128	V$GATA1_04	0.827206	319	(-)	NNCWGATARNNNN	CTCCTTATGAGGA
M00076	V$GATA2_01	0.797023	321	(-)	NNNGATRNNN	CCTTATGAGG
M00075	V$GATA1_01	0.780849	358	(-)	SNNGATNNNN	TGAAATCTGA
M00076	V$GATA2_01	0.827244	358	(-)	NNNGATRNNN	TGAAATCTGA
M00126	V$GATA1_02	0.824063	398	(+)	NNNNNGATANKGNN	TTTCTGATAGTCAT
M00128	V$GATA1_04	0.887868	399	(+)	NNCWGATARNNNN	TTCTGATAGTCAT
M00075	V$GATA1_01	0.892892	400	(+)	SNNGATNNNN	TCTGATAGTC
M00076	V$GATA2_01	0.924222	400	(+)	NNNGATRNNN	TCTGATAGTC
M00077	V$GATA3_01	0.865751	401	(+)	NNGATARNG	CTGATAGTC
M00203	V$GATA_C	0.835353	402	(+)	NGATAAGNMNN	TGATAGTCATT
M00077	V$GATA3_01	0.844927	419	(+)	NNGATARNG	AAGATTAAA
M00076	V$GATA2_01	0.797474	447	(-)	NNNGATRNNN	GCATATGTCA
M00203	V$GATA_C	0.849332	489	(-)	NGATAAGNMNN	TTACTGTATCT
M00126	V$GATA1_02	0.875938	490	(-)	NNNNNGATANKGNN	TACTGTATCTTCAC
M00127	V$GATA1_03	0.824841	490	(-)	RNSNNGATAANNGN	TACTGTATCTTCAC
M00075	V$GATA1_01	0.868707	492	(-)	SNNGATNNNN	CTGTATCTTC
M00076	V$GATA2_01	0.893099	492	(-)	NNNGATRNNN	CTGTATCTTC
M00077	V$GATA3_01	0.86265	492	(-)	NNGATARNG	CTGTATCTT
M00076	V$GATA2_01	0.815968	519	(+)	NNNGATRNNN	TGGCATAGTA
M00126	V$GATA1_02	0.810937	569	(+)	NNNNNGATANKGNN	TTATACATAGGGTC
M00127	V$GATA1_03	0.832925	589	(-)	RNSNNGATAANNGN	CCTACTAACTTGCT
M00075	V$GATA1_01	0.820829	635	(+)	SNNGATNNNN	CAAGATTTTA
M00076	V$GATA2_01	0.809202	635	(+)	NNNGATRNNN	CAAGATTTTA
M00077	V$GATA3_01	0.832964	636	(+)	NNGATARNG	AAGATTTTA

M00076	V$GATA2_01	0.793866	645	(-)	NNNGATRNNN	CATTATGTTG
M00203	V$GATA_C	0.853681	674	(-)	NGATAAGNMNN	AGAACTTGTCA
M00203	V$GATA_C	0.917676	715	(-)	NGATAAGNMNN	TGCTTTTATCA
M00126	V$GATA1_02	0.803125	716	(-)	NNNNNGATANKGNN	GCTTTTATCAGTCT
M00127	V$GATA1_03	0.918667	716	(-)	RNSNNGATAANNGN	GCTTTTATCAGTCT
M00128	V$GATA1_04	0.932904	716	(-)	NNCWGATARNNNNN	GCTTTTATCAGTC
M00077	V$GATA3_01	0.849358	718	(-)	NNGATARNG	TTTTATCAG
M00076	V$GATA2_01	0.79161	787	(-)	NNNGATRNNN	AAATATGGGA
M00076	V$GATA2_01	0.794768	796	(-)	NNNGATRNNN	AAATATGTGA
M00075	V$GATA1_01	0.809477	801	(+)	SNNGATNNNN	TGTGATTTCA
M00076	V$GATA2_01	0.815968	801	(+)	NNNGATRNNN	TGTGATTTCA
M00075	V$GATA1_01	0.807996	828	(-)	SNNGATNNNN	ATTGATCAGC
M00076	V$GATA2_01	0.786198	828	(-)	NNNGATRNNN	ATTGATCAGC
M00076	V$GATA2_01	0.819576	837	(+)	NNNGATRNNN	CCCCATATCT
M00126	V$GATA1_02	0.903438	837	(-)	NNNNNGATANKGNN	CCCCATATCTGTTC
M00127	V$GATA1_03	0.814307	837	(-)	RNSNNGATAANNGN	CCCCATATCTGTTC
M00128	V$GATA1_04	0.932598	837	(-)	NNCWGATARNNNNN	CCCCATATCTGTT
M00075	V$GATA1_01	0.85538	839	(-)	SNNGATNNNN	CCATATCTGT
M00076	V$GATA2_01	0.88949	839	(-)	NNNGATRNNN	CCATATCTGT
M00077	V$GATA3_01	0.916704	839	(-)	NNGATARNG	CCATATCTG
M00127	V$GATA1_03	0.811612	880	(-)	RNSNNGATAANNGN	ATTTTTACCTGCCT
M00127	V$GATA1_03	0.783684	919	(+)	RNSNNGATAANNGN	GTAAGTATAATAAG
M00128	V$GATA1_04	0.850184	928	(+)	NNCWGATARNNNNN	ATAAGATATTTAT
M00075	V$GATA1_01	0.819348	929	(+)	SNNGATNNNN	TAAGATATTT
M00076	V$GATA2_01	0.894452	929	(+)	NNNGATRNNN	TAAGATATTT
M00077	V$GATA3_01	0.834736	930	(+)	NNGATARNG	AAGATATTT
M00127	V$GATA1_03	0.781725	933	(+)	RNSNNGATAANNGN	ATATTTATAAGTAC
M00075	V$GATA1_01	0.773939	949	(+)	SNNGATNNNN	CCTGTTATTT
M00127	V$GATA1_03	0.863302	983	(+)	RNSNNGATAANNGN	ATGCAGACAATGGC
M00216	V$TATA_C	0.897808	39	(+)	NCTATAAAAR	TCTATATAAC
M00252	V$TATA_01	0.834052	40	(+)	STATAAAWRNNNNNN	CTATATAACTTATGA
M00216	V$TATA_C	0.769475	54	(+)	NCTATAAAAR	ACTTTGTAAA
M00216	V$TATA_C	0.775548	56	(+)	NCTATAAAAR	TTTGTAAAAC

M00216	V$TATA_C	0.743068	64	(+)	NCTATAAAAR	ACTTAATAAC
M00216	V$TATA_C	0.752839	77	(+)	NCTATAAAAR	TCTCCAAAAA
M00216	V$TATA_C	0.772379	89	(+)	NCTATAAAAR	ATTTTTAAAG
M00252	V$TATA_01	0.794976	89	(+)	STATAAAWRNNNNNN	ATTTTTAAAGAAACT
M00216	V$TATA_C	0.768154	90	(+)	NCTATAAAAR	TTTTTAAAGA
M00252	V$TATA_01	0.774676	91	(+)	STATAAAWRNNNNNN	TTTTAAAGAAACTAG
M00216	V$TATA_C	0.789015	113	(+)	NCTATAAAAR	ACCTTAAGAT
M00216	V$TATA_C	0.760496	320	(+)	NCTATAAAAR	TCCTTATGAG
M00216	V$TATA_C	0.836282	366	(+)	NCTATAAAAR	GATTTAAAAA
M00216	V$TATA_C	0.743597	367	(+)	NCTATAAAAR	ATTTAAAAAT
M00252	V$TATA_01	0.802334	367	(+)	STATAAAWRNNNNNN	ATTTAAAAATTAGTT
M00252	V$TATA_01	0.792185	420	(+)	STATAAAWRNNNNNN	AGATTAAAGAAGAGT
M00216	V$TATA_C	0.750198	435	(+)	NCTATAAAAR	TGTAAATAAT
M00216	V$TATA_C	0.768682	454	(+)	NCTATAAAAR	TCAATAATAT
M00216	V$TATA_C	0.802218	459	(+)	NCTATAAAAR	AATATATAAA
M00252	V$TATA_01	0.846993	460	(+)	STATAAAWRNNNNNN	ATATATAAATAATAT
M00216	V$TATA_C	0.751782	461	(+)	NCTATAAAAR	TATATAAATA
M00252	V$TATA_01	0.829739	462	(+)	STATAAAWRNNNNNN	ATATAAATAATATAT
M00216	V$TATA_C	0.802218	470	(+)	NCTATAAAAR	AATATATAAA
M00252	V$TATA_01	0.821619	471	(+)	STATAAAWRNNNNNN	ATATATAAATTTTTT
M00252	V$TATA_01	0.795483	473	(+)	STATAAAWRNNNNNN	ATATAAATTTTTTTC
M00252	V$TATA_01	0.776199	569	(+)	STATAAAWRNNNNNN	TTATACATAGGGTCA
M00216	V$TATA_C	0.801162	624	(+)	NCTATAAAAR	AGTTTAGAAA
M00252	V$TATA_01	0.792692	625	(+)	STATAAAWRNNNNNN	GTTTAGAAACCAAGA
M00216	V$TATA_C	0.871138	665	(+)	NCTATAAAAR	TCCTTATAAA
M00252	V$TATA_01	0.79929	666	(+)	STATAAAWRNNNNNN	CCTTATAAAGAACTT
M00216	V$TATA_C	0.79324	667	(+)	NCTATAAAAR	CTTATAAAGA
M00252	V$TATA_01	0.829992	668	(+)	STATAAAWRNNNNNN	TTATAAAGAACTTGT
M00252	V$TATA_01	0.822126	754	(+)	STATAAAWRNNNNNN	GTACAAATATAAATT
M00252	V$TATA_01	0.781528	758	(+)	STATAAAWRNNNNNN	AAATATAAATTAATA
M00252	V$TATA_01	0.841411	760	(+)	STATAAAWRNNNNNN	ATATAAATTAATATA
M00216	V$TATA_C	0.78479	769	(+)	NCTATAAAAR	AATATATAAT
M00252	V$TATA_01	0.830246	770	(+)	STATAAAWRNNNNNN	ATATATAATTAATGA

M00252	V$TATA_01	0.812484	772	(+)	STATAAAWRNNNNNN	ATATAATTAATGAGG
M00216	V$TATA_C	0.761025	805	(+)	NCTATAAAAR	ATTTCAAAAA
M00216	V$TATA_C	0.744389	914	(+)	NCTATAAAAR	AGCATGTAAG
M00216	V$TATA_C	0.824927	922	(+)	NCTATAAAAR	AGTATAATAA
M00252	V$TATA_01	0.843441	923	(+)	STATAAAWRNNNNNN	GTATAATAAGATATT
M00252	V$TATA_01	0.827709	933	(+)	STATAAAWRNNNNNN	ATATTTATAAGTACC
M00216	V$TATA_C	0.795088	934	(+)	NCTATAAAAR	TATTTATAAG
M00252	V$TATA_01	0.824664	935	(+)	STATAAAWRNNNNNN	ATTTATAAGTACCTC
M00252	V$TATA_01	0.820858	937	(+)	STATAAAWRNNNNNN	TTATAAGTACCTCCT
M00252	V$TATA_01	0.777721	953	(+)	STATAAAWRNNNNNN	TTATTTAAATATTAG
M00252	V$TATA_01	0.787617	955	(+)	STATAAAWRNNNNNN	ATTTAAATATTAGAA
M00252	V$TATA_01	0.788886	961	(+)	STATAAAWRNNNNNN	ATATTAGAAGAGTGA
M00216	V$TATA_C	0.807763	1035	(+)	NCTATAAAAR	CTTTTATAAT
M00252	V$TATA_01	0.807917	1036	(+)	STATAAAWRNNNNNN	TTTTATAATCAAAGA
M00216	V$TATA_C	0.806443	1066	(+)	NCTATAAAAR	CTTATACAAG
M00223	V$STAT_01	0.819763	76	(+)	TTCCCRKAA	TTCTCCAAA
M00223	V$STAT_01	0.800605	317	(+)	TTCCCRKAA	TTCTCCTTA
M00223	V$STAT_01	0.815478	323	(-)	TTCCCRKAA	TTATGAGGA
M00223	V$STAT_01	0.805899	489	(+)	TTCCCRKAA	TTACTGTAT
M00223	V$STAT_01	0.795059	515	(+)	TTCCCRKAA	TACCTGGCA
M00223	V$STAT_01	0.800605	662	(+)	TTCCCRKAA	TTCTCCTTA
M00224	V$STAT1_01	0.730282	783	(+)	NNNSANTTCCGGGAANTGNSN	GAGGAAATATGGGAAATATGT
M00223	V$STAT_01	0.85934	789	(-)	TTCCCRKAA	ATATGGGAA
M00223	V$STAT_01	0.826821	905	(+)	TTCCCRKAA	TTCTTGCAA
CSN2_Camelus ferus						
M00059	V$YY1_01	0.791284	8281	(-)	NNNNNCCATNTWNNNWN	GTCCAGATATGACTTAA
M00069	V$YY1_02	0.797037	8434	(-)	NNNCGGCCATCTT-GNCTSNW	AAGGCACAACATGGTGCATT
M00059	V$YY1_01	0.805374	8436	(-)	NNNNNCCATNTWNNNWN	GGCACAACATGGTGCAT
M00059	V$YY1_01	0.774246	8437	(+)	NNNNNCCATNTWNNNWN	GCACAACATGGTGCATT
M00059	V$YY1_01	0.783748	8615	(+)	NNNNNCCATNTWNNNWN	CAAAACGATTTTTAAAT
M00059	V$YY1_01	0.81291	8633	(+)	NNNNNCCATNTWNNNWN	ACTCTCCATGGAAAAAA
M00059	V$YY1_01	0.798493	8642	(-)	NNNNNCCATNTWNNNWN	GGAAAAAAATGAAACTG
M00059	V$YY1_01	0.796199	8678	(-)	NNNNNCCATNTWNNNWN	TGCTTTACATGGCACTC
M00059	V$YY1_01	0.847969	8775	(-)	NNNNNCCATNTWNNNWN	TATCAAAAATGTTTATA
M00059	V$YY1_01	0.782438	8791	(+)	NNNNNCCATNTWNNNWN	AAAAATCATTTGTGTAA
M00223	V$STAT_01	0.822536	125	(-)	TTCCCRKAA	TTCTGAGGA

M00223	V$STAT_01	0.878498	146	(+)	TTCCCRKAA	TTATTGTAA
M00223	V$STAT_01	0.826569	146	(-)	TTCCCRKAA	TTATTGTAA
M00223	V$STAT_01	0.792034	216	(+)	TTCCCRKAA	TTCCATGAA
M00223	V$STAT_01	0.8606	216	(-)	TTCCCRKAA	TTCCATGAA
M00223	V$STAT_01	0.805899	246	(+)	TTCCCRKAA	TCACTGTAA
M00223	V$STAT_01	0.79758	407	(+)	TTCCCRKAA	TGCCCATCA
M00223	V$STAT_01	0.815478	519	(+)	TTCCCRKAA	TTCTCATAT
M00223	V$STAT_01	0.812957	830	(+)	TTCCCRKAA	TTACTGGAC
M00223	V$STAT_01	0.866398	900	(+)	TTCCCRKAA	TCCCCAGAA
M00223	V$STAT_01	0.80489	900	(-)	TTCCCRKAA	TCCCCAGAA
M00223	V$STAT_01	0.80489	910	(+)	TTCCCRKAA	TTCTGGGGA
M00223	V$STAT_01	0.866398	910	(-)	TTCCCRKAA	TTCTGGGGA
M00223	V$STAT_01	0.790522	923	(-)	TTCCCRKAA	TAATAAGAA
M00224	V$STAT1_01	0.790219	951	(+)	NNNSANTTCCGG-GAANTGNSN	GTGGAATTCTGGAAATTCAAA
M00224	V$STAT1_01	0.756792	951	(-)	NNNSANTTCCGG-GAANTGNSN	GTGGAATTCTGGAAATTCAAA
M00225	V$STAT3_01	0.750326	951	(+)	NGNNATTTCCSG-GAARTGNNN	GTGGAATTCTGGAAATTCAAA
M00225	V$STAT3_01	0.743085	951	(-)	NGNNATTTCCSG-GAARTGNNN	GTGGAATTCTGGAAATTCAAA
M00223	V$STAT_01	0.81699	957	(+)	TTCCCRKAA	TTCTGGAAA
M00223	V$STAT_01	0.870179	957	(-)	TTCCCRKAA	TTCTGGAAA
M00223	V$STAT_01	0.802622	1242	(-)	TTCCCRKAA	TTAGGTTAA
M00223	V$STAT_01	0.843459	1276	(-)	TTCCCRKAA	TGATAAGAA
M00224	V$STAT1_01	0.744772	1382	(+)	NNNSANTTCCGG-GAANTGNSN	GATCAATTTCTTGAATTAAGT
M00223	V$STAT_01	0.799597	1590	(+)	TTCCCRKAA	TTTTTAGAA
M00223	V$STAT_01	0.888581	1593	(-)	TTCCCRKAA	TTAGAAGAA
M00223	V$STAT_01	0.856819	1762	(-)	TTCCCRKAA	TTCCAGGAG
M00223	V$STAT_01	0.843459	1967	(-)	TTCCCRKAA	TGACGATAA
M00223	V$STAT_01	0.846483	2003	(-)	TTCCCRKAA	TTAGGGAAA
M00223	V$STAT_01	0.792538	2158	(+)	TTCCCRKAA	TTTTTATAA
M00223	V$STAT_01	0.853542	2171	(-)	TTCCCRKAA	TTACAGAAA
M00223	V$STAT_01	0.815478	2773	(+)	TTCCCRKAA	CTCTCATAA
M00223	V$STAT_01	0.809932	2823	(-)	TTCCCRKAA	TTAGCTGAA
M00223	V$STAT_01	0.802622	2933	(+)	TTCCCRKAA	TTTTCCTAA
M00223	V$STAT_01	0.853542	2935	(+)	TTCCCRKAA	TTCCTAAAA
M00223	V$STAT_01	0.863625	2981	(-)	TTCCCRKAA	TTAAAGGAA
M00223	V$STAT_01	0.795815	3253	(-)	TTCCCRKAA	TTCTAAGAT
M00223	V$STAT_01	0.849761	3297	(-)	TTCCCRKAA	TTACAGGAT
M00223	V$STAT_01	0.849761	3352	(-)	TTCCCRKAA	CTACAGGAA
M00223	V$STAT_01	0.79884	3505	(+)	TTCCCRKAA	TTACCCTAG
M00223	V$STAT_01	0.822536	3585	(+)	TTCCCRKAA	TACTCAGAA
M00223	V$STAT_01	0.79758	3659	(+)	TTCCCRKAA	TACCCATCA
M00224	V$STAT1_01	0.75037	3673	(-)	NNNSANTTCCGG-GAANTGNSN	TCTTCATTCAGGTAAAATAAA
M00223	V$STAT_01	0.843711	3679	(+)	TTCCCRKAA	TTCAGGTAA
M00223	V$STAT_01	0.853542	3679	(-)	TTCCCRKAA	TTCAGGTAA

M00223	V$STAT_01	0.826821	3782	(+)	TTCCCRKAA	TTTCTCGAA
M00223	V$STAT_01	0.833627	3936	(+)	TTCCCRKAA	TTCAGAGAA
M00223	V$STAT_01	0.853542	3936	(-)	TTCCCRKAA	TTCAGAGAA
M00223	V$STAT_01	0.79884	3983	(-)	TTCCCRKAA	CTAGGAGAA
M00223	V$STAT_01	0.819763	4084	(+)	TTCCCRKAA	TTACCCAAA
M00223	V$STAT_01	0.888581	4384	(-)	TTCCCRKAA	TTAGAGTAA
M00223	V$STAT_01	0.839677	4489	(-)	TTCCCRKAA	ATCTAGGAA
M00223	V$STAT_01	0.849761	4650	(-)	TTCCCRKAA	TTACAGGAG
M00223	V$STAT_01	0.861356	4722	(+)	TTCCCRKAA	TTATTATAA
M00223	V$STAT_01	0.819259	4785	(+)	TTCCCRKAA	TTGTCATAA
M00223	V$STAT_01	0.790522	4788	(-)	TTCCCRKAA	TCATAAGAA
M00223	V$STAT_01	0.826317	5108	(+)	TTCCCRKAA	TTTTCAGAA
M00223	V$STAT_01	0.799849	5108	(-)	TTCCCRKAA	TTTTCAGAA
M00223	V$STAT_01	0.826317	5171	(+)	TTCCCRKAA	TTAGCAGAA
M00223	V$STAT_01	0.87875	5171	(-)	TTCCCRKAA	TTAGCAGAA
M00223	V$STAT_01	0.895387	5229	(+)	TTCCCRKAA	TTACGATAA
M00223	V$STAT_01	0.905218	5229	(-)	TTCCCRKAA	TTACGATAA
M00223	V$STAT_01	0.832619	5330	(-)	TTCCCRKAA	GTATAGGAA
M00223	V$STAT_01	0.799597	5487	(+)	TTCCCRKAA	TTAGTAGAA
M00223	V$STAT_01	0.809932	5487	(-)	TTCCCRKAA	TTAGTAGAA
M00223	V$STAT_01	0.8243	5528	(+)	TTCCCRKAA	TTCTCAGGA
M00223	V$STAT_01	0.815478	5662	(+)	TTCCCRKAA	TACTCATAA
M00223	V$STAT_01	0.80968	5694	(-)	TTCCCRKAA	TTACATTAA
M00223	V$STAT_01	0.853542	5899	(+)	TTCCCRKAA	TTGCTGTAA
M00223	V$STAT_01	0.817242	6470	(-)	TTCCCRKAA	TAATGAGAA
M00223	V$STAT_01	0.856819	6661	(-)	TTCCCRKAA	TTCCAGGAT
M00223	V$STAT_01	0.79884	6741	(+)	TTCCCRKAA	TTACCCTAT
M00224	V$STAT1_01	0.727812	6890	(+)	NNNSANTTCCGG-GAANTGNSN	TGCCCTTTACTGAAAGCCAGA
M00224	V$STAT1_01	0.729788	6890	(-)	NNNSANTTCCGG-GAANTGNSN	TGCCCTTTACTGAAAGCCAGA
M00223	V$STAT_01	0.826821	6896	(+)	TTCCCRKAA	TTACTGAAA
M00223	V$STAT_01	0.804638	7059	(+)	TTCCCRKAA	TCCCCAGCA
M00223	V$STAT_01	0.856819	7117	(-)	TTCCCRKAA	TTCCAGGAG
M00223	V$STAT_01	0.844719	7238	(-)	TTCCCRKAA	TTAGAATAA
M00223	V$STAT_01	0.81699	7452	(-)	TTCCCRKAA	TGAGCAGAA
M00223	V$STAT_01	0.8364	7592	(+)	TTCCCRKAA	TTTCTATAA
M00223	V$STAT_01	0.846483	7722	(-)	TTCCCRKAA	TTAGGGAAA
M00223	V$STAT_01	0.843459	7734	(+)	TTCCCRKAA	TTCTTATCA
M00223	V$STAT_01	0.792538	7737	(+)	TTCCCRKAA	TTATCAAAA
M00224	V$STAT1_01	0.736539	7817	(-)	NNNSANTTCCGG-GAANTGNSN	CCTCACTTTTGGTAAGCTTTA
M00223	V$STAT_01	0.799849	7823	(+)	TTCCCRKAA	TTTTGGTAA
M00223	V$STAT_01	0.8364	7823	(-)	TTCCCRKAA	TTTTGGTAA
M00223	V$STAT_01	0.805899	7865	(-)	TTCCCRKAA	TTACAGTTA
M00223	V$STAT_01	0.853542	8108	(-)	TTCCCRKAA	TTTTAGGAA
M00223	V$STAT_01	0.802622	8110	(-)	TTCCCRKAA	TTAGGAAAA
M00223	V$STAT_01	0.815982	8405	(-)	TTCCCRKAA	CTAGAGGAA

M00225	V$STAT3_01	0.711224	8550	(+)	NGNNATTTCCSG-GAARTGNNN	CTCTATTCCACAGAATTGACT
M00225	V$STAT3_01	0.712527	8550	(-)	NGNNATTTCCSG-GAARTGNNN	CTCTATTCCACAGAATTGACT
M00223	V$STAT_01	0.8364	8611	(+)	TTCCCRKAA	TTACCAAAA
M00223	V$STAT_01	0.799849	8611	(-)	TTCCCRKAA	TTACCAAAA
M00223	V$STAT_01	0.815478	8763	(+)	TTCCCRKAA	ATACCATAA
M00223	V$STAT_01	0.792538	8774	(+)	TTCCCRKAA	TTATCAAAA
M00223	V$STAT_01	0.792538	8787	(-)	TTCCCRKAA	TTATAAAAA
CSN3_Camelus ferus						
M00127	V$GATA1_03	0.821166	6	(-)	RNSNNGATAANNGN	TCTCTTTTCAAGCT
M00127	V$GATA1_03	0.78001	16	(+)	RNSNNGATAANNGN	AGCTTTATAAATGA
M00127	V$GATA1_03	0.804508	41	(-)	RNSNNGATAANNGN	CCTCCTATGCACTT
M00075	V$GATA1_01	0.792695	74	(+)	SNNGATNNNN	AAAGATGACT
M00076	V$GATA2_01	0.837618	74	(+)	NNNGATRNNN	AAAGATGACT
M00126	V$GATA1_02	0.849688	83	(-)	NNNNNGATANKGNN	TCTGCTATCGTCAA
M00075	V$GATA1_01	0.901777	85	(-)	SNNGATNNNN	TGCTATCGTC
M00076	V$GATA2_01	0.931439	85	(-)	NNNGATRNNN	TGCTATCGTC
M00077	V$GATA3_01	0.881258	85	(-)	NNGATARNG	TGCTATCGT
M00077	V$GATA3_01	0.853788	93	(-)	NNGATARNG	TCAAATCTT
M00127	V$GATA1_03	0.792014	104	(-)	RNSNNGATAANNGN	CTTTTTGTCATCTT
M00127	V$GATA1_03	0.843459	107	(-)	RNSNNGATAANNGN	TTTGTCATCTTCCT
M00075	V$GATA1_01	0.821816	109	(-)	SNNGATNNNN	TGTCATCTTC
M00076	V$GATA2_01	0.854759	109	(-)	NNNGATRNNN	TGTCATCTTC
M00077	V$GATA3_01	0.828977	109	(-)	NNGATARNG	TGTCATCTT
M00126	V$GATA1_02	0.770625	138	(+)	NNNNNGATANKGNN	AGGAAGATAAATCA
M00127	V$GATA1_03	0.890495	138	(+)	RNSNNGATAANNGN	AGGAAGATAAATCA
M00128	V$GATA1_04	0.896446	139	(+)	NNCWGATARNNNN	GGAAGATAAATCA
M00075	V$GATA1_01	0.817868	140	(+)	SNNGATNNNN	GAAGATAAAT
M00076	V$GATA2_01	0.853857	140	(+)	NNNGATRNNN	GAAGATAAAT
M00077	V$GATA3_01	0.846699	141	(+)	NNGATARNG	AAGATAAAT
M00203	V$GATA_C	0.943461	142	(+)	NGATAAGNMNN	AGATAAATCAC
M00075	V$GATA1_01	0.805528	144	(-)	SNNGATNNNN	ATAAATCACA
M00076	V$GATA2_01	0.786649	144	(-)	NNNGATRNNN	ATAAATCACA
M00076	V$GATA2_01	0.790708	149	(+)	NNNGATRNNN	TCACATGCTG
M00075	V$GATA1_01	0.835637	164	(-)	SNNGATNNNN	CACTAACACC
M00076	V$GATA2_01	0.79567	164	(-)	NNNGATRNNN	CACTAACACC

M00127	V$GATA1_03	0.854483	187	(-)	RNSNNGATAANNGN	CCAGTTATTTACCT
M00075	V$GATA1_01	0.793189	251	(+)	SNNGATNNNN	CCTGACAGGC
M00203	V$GATA_C	0.833178	253	(+)	NGATAAGNMNN	TGACAGGCACA
M00127	V$GATA1_03	0.826801	265	(+)	RNSNNGATAANNGN	GGGAAGGTAATCAA
M00077	V$GATA3_01	0.826761	292	(+)	NNGATARNG	GAGAGAATG
M00127	V$GATA1_03	0.784664	312	(+)	RNSNNGATAANNGN	CAGGAGTTAATCAT
M00075	V$GATA1_01	0.817374	369	(-)	SNNGATNNNN	TGGCATCATT
M00076	V$GATA2_01	0.822733	369	(-)	NNNGATRNNN	TGGCATCATT
M00075	V$GATA1_01	0.792201	404	(+)	SNNGATNNNN	TTAGATTCTG
M00077	V$GATA3_01	0.824546	405	(+)	NNGATARNG	TAGATTCTG
M00077	V$GATA3_01	0.821444	423	(+)	NNGATARNG	ATGATTAAA
M00127	V$GATA1_03	0.785154	435	(-)	RNSNNGATAANNGN	ATTTTTAACTTCAC
M00076	V$GATA2_01	0.810104	545	(-)	NNNGATRNNN	ACATATGTGG
M00076	V$GATA2_01	0.801083	557	(-)	NNNGATRNNN	GGCTAACTTC
M00127	V$GATA1_03	0.789809	569	(+)	RNSNNGATAANNGN	AGAAAGATGAATGT
M00075	V$GATA1_01	0.797631	588	(-)	SNNGATNNNN	AATAATCCAG
M00203	V$GATA_C	0.896552	626	(-)	NGATAAGNMNN	TATTTCTATCT
M00126	V$GATA1_02	0.80375	627	(-)	NNNNNGATANKGNN	ATTTCTATCTAAAG
M00128	V$GATA1_04	0.869485	627	(-)	NNCWGATARNNNN	ATTTCTATCTAAA
M00077	V$GATA3_01	0.882588	629	(-)	NNGATARNG	TTCTATCTA
M00077	V$GATA3_01	0.864865	638	(+)	NNGATARNG	AAGATTATA
M00203	V$GATA_C	0.878534	686	(-)	NGATAAGNMNN	GTTACATATCC
M00127	V$GATA1_03	0.807692	687	(-)	RNSNNGATAANNGN	TTACATATCCTCAC
M00075	V$GATA1_01	0.883514	689	(-)	SNNGATNNNN	ACATATCCTC
M00076	V$GATA2_01	0.929635	689	(-)	NNNGATRNNN	ACATATCCTC
M00075	V$GATA1_01	0.836624	734	(-)	SNNGATNNNN	TTCCATCAAG
M00076	V$GATA2_01	0.78304	771	(-)	NNNGATRNNN	AATTATGGTC
M00203	V$GATA_C	0.987263	776	(-)	NGATAAGNMNN	TGGTCTTATCT
M00126	V$GATA1_02	0.850625	777	(-)	NNNNNGATANKGNN	GGTCTTATCTCTCT
M00127	V$GATA1_03	0.914993	777	(-)	RNSNNGATAANNGN	GGTCTTATCTCTCT
M00128	V$GATA1_04	0.928002	777	(-)	NNCWGATARNNNN	GGTCTTATCTCTC
M00075	V$GATA1_01	0.798124	779	(-)	SNNGATNNNN	TCTTATCTCT
M00076	V$GATA2_01	0.831755	779	(-)	NNNGATRNNN	TCTTATCTCT

M00077	V$GATA3_01	0.936642	779	(-)	NNGATARNG	TCTTATCTC
M00075	V$GATA1_01	0.837117	788	(+)	SNNGATNNNN	TCTGATTTCT
M00076	V$GATA2_01	0.845737	788	(+)	NNNGATRNNN	TCTGATTTCT
M00075	V$GATA1_01	0.794669	813	(-)	SNNGATNNNN	GAGGATCTAC
M00076	V$GATA2_01	0.815065	813	(+)	NNNGATRNNN	GAGGATCTAC
M00075	V$GATA1_01	0.854886	832	(+)	SNNGATNNNN	ACAGATTGCT
M00076	V$GATA2_01	0.861074	832	(+)	NNNGATRNNN	ACAGATTGCT
M00077	V$GATA3_01	0.83961	833	(+)	NNGATARNG	CAGATTGCT
M00075	V$GATA1_01	0.814906	895	(-)	SNNGATNNNN	AAACATCCTA
M00076	V$GATA2_01	0.880469	895	(-)	NNNGATRNNN	AAACATCCTA
M00076	V$GATA2_01	0.848895	907	(-)	NNNGATRNNN	AGAGATCTTA
M00126	V$GATA1_02	0.838125	937	(+)	NNNNNGATANKGNN	GAAATGATAATTAT
M00127	V$GATA1_03	0.855218	937	(+)	RNSNNGATAANNGN	GAAATGATAATTAT
M00128	V$GATA1_04	0.885417	938	(+)	NNCWGATARNNNN	AAATGATAATTAT
M00075	V$GATA1_01	0.813425	939	(+)	SNNGATNNNN	AATGATAATT
M00076	V$GATA2_01	0.835814	939	(+)	NNNGATRNNN	AATGATAATT
M00077	V$GATA3_01	0.843155	940	(+)	NNGATARNG	ATGATAATT
M00203	V$GATA_C	0.851196	941	(+)	NGATAAGNMNN	TGATAATTATG
M00126	V$GATA1_02	0.871875	953	(+)	NNNNNGATANKGNN	GACATGATAGAAGT
M00127	V$GATA1_03	0.852523	953	(+)	RNSNNGATAANNGN	GACATGATAGAAGT
M00128	V$GATA1_04	0.902267	954	(+)	NNCWGATARNNNN	ACATGATAGAAGT
M00075	V$GATA1_01	0.872655	955	(+)	SNNGATNNNN	CATGATAGAA
M00076	V$GATA2_01	0.858818	955	(+)	NNNGATRNNN	CATGATAGAA
M00077	V$GATA3_01	0.883474	956	(+)	NNGATARNG	ATGATAGAA
M00203	V$GATA_C	0.894688	967	(-)	NGATAAGNMNN	GGTAATTATCA
M00126	V$GATA1_02	0.840313	968	(-)	NNNNNGATANKGNN	GTAATTATCACTAC
M00127	V$GATA1_03	0.887555	968	(-)	RNSNNGATAANNGN	GTAATTATCACTAC
M00128	V$GATA1_04	0.870098	968	(-)	NNCWGATARNNNN	GTAATTATCACTA
M00075	V$GATA1_01	0.842053	970	(-)	SNNGATNNNN	AATTATCACT
M00076	V$GATA2_01	0.843933	970	(-)	NNNGATRNNN	AATTATCACT
M00077	V$GATA3_01	0.860434	970	(-)	NNGATARNG	AATTATCAC
M00075	V$GATA1_01	0.877098	985	(-)	SNNGATNNNN	GGCAATCACA
M00076	V$GATA2_01	0.878665	985	(-)	NNNGATRNNN	GGCAATCACA

M0007		0.83916				
7	V$GATA3_01	7	985	(-)	NNGATARNG	GGCAATCAC
M0007		0.81342				
5	V$GATA1_01	5	1013	(-)	SNNGATNNNN	TCAAATCAAC
M0012		0.78564				
7	V$GATA1_03	4	1047	(-)	RNSNNGATAANNGN	AATGTTATATGTCT
M0012		0.81185				
7	V$GATA1_03	7	1084	(+)	RNSNNGATAANNGN	AAAAAGGTAACTGG
M0007		0.77443				
5	V$GATA1_01	2	1088	(-)	SNNGATNNNN	AGGTAACTGG
M0007		0.78213				
6	V$GATA2_01	8	1088	(-)	NNNGATRNNN	AGGTAACTGG
M0012		0.77531				
6	V$GATA1_02	3	1106	(+)	NNNNNGATANKGNN	GAATTAATAGGGAA
M0007		0.79431				
6	V$GATA2_01	7	1153	(-)	NNNGATRNNN	GTAGATCTCA
M0007		0.81688				
5	V$GATA1_01	1	1182	(+)	SNNGATNNNN	AATGATATCC
M0007		0.86603				
6	V$GATA2_01	5	1182	(+)	NNNGATRNNN	AATGATATCC
M0007		0.77936				
5	V$GATA1_01	8	1184	(-)	SNNGATNNNN	TGATATCCTA
M0007						
6	V$GATA2_01	0.89806	1184	(-)	NNNGATRNNN	TGATATCCTA
M0020						
3	V$GATA_C	0.88164	1186	(-)	NGATAAGNMNN	ATATCCTATCA
M0012		0.82968				
6	V$GATA1_02	8	1187	(-)	NNNNNGATANKGNN	TATCCTATCAGAGG
M0012						
8	V$GATA1_04	0.83364	1187	(-)	NNCWGATARNNNN	TATCCTATCAGAG
M0007		0.88450				
5	V$GATA1_01	1	1189	(-)	SNNGATNNNN	TCCTATCAGA
M0007		0.90437				
6	V$GATA2_01	5	1189	(-)	NNNGATRNNN	TCCTATCAGA
M0007		0.91847				
7	V$GATA3_01	6	1189	(-)	NNGATARNG	TCCTATCAG
M0007		0.79318				
5	V$GATA1_01	9	1204	(-)	SNNGATNNNN	TGAAATCAGT
M0007		0.78349				
6	V$GATA2_01	1	1204	(-)	NNNGATRNNN	TGAAATCAGT
M0012		0.79103				
7	V$GATA1_03	4	1227	(-)	RNSNNGATAANNGN	TTTCTAATCATTAT
M0007		0.86220				
7	V$GATA3_01	6	1229	(-)	NNGATARNG	TCTAATCAT
M0020		0.86082				
3	V$GATA_C	6	1235	(-)	NGATAAGNMNN	CATTATTATCT
M0012		0.85843				
6	V$GATA1_02	8	1236	(-)	NNNNNGATANKGNN	ATTATTATCTGTGA
M0012		0.79127				
7	V$GATA1_03	9	1236	(-)	RNSNNGATAANNGN	ATTATTATCTGTGA
M0012		0.84926				
8	V$GATA1_04	5	1236	(-)	NNCWGATARNNNN	ATTATTATCTGTG
M0007		0.77788				
5	V$GATA1_01	7	1238	(-)	SNNGATNNNN	TATTATCTGT
M0007						
6	V$GATA2_01	0.82544	1238	(-)	NNNGATRNNN	TATTATCTGT
M0007		0.89277				
7	V$GATA3_01	8	1238	(-)	NNGATARNG	TATTATCTG
M0012		0.78882				
7	V$GATA1_03	9	1278	(-)	RNSNNGATAANNGN	CCCTTTACCAAGCC
M0012		0.78218				
6	V$GATA1_02	7	1308	(-)	NNNNNGATANKGNN	GACCCTCTCTTGTA
M0007		0.77788				
5	V$GATA1_01	7	1310	(-)	SNNGATNNNN	CCCTCTCTTG
M0007		0.85866				
7	V$GATA3_01	2	1310	(-)	NNGATARNG	CCCTCTCTT

M00077	V$GATA3_01	0.82942	1340	(-)	NNGATARNG	TCAAATCTA
M00075	V$GATA1_01	0.903751	1378	(+)	SNNGATNNNN	CAAGATGTGG
M00076	V$GATA2_01	0.894452	1378	(+)	NNNGATRNNN	CAAGATGTGG
M00077	V$GATA3_01	0.868852	1379	(+)	NNGATARNG	AAGATGTGG
M00127	V$GATA1_03	0.821166	1438	(-)	RNSNNGATAANNGN	ACTGTTATATGTAT
M00203	V$GATA_C	0.880087	1443	(-)	NGATAAGNMNN	TATATGTATCT
M00127	V$GATA1_03	0.83415	1444	(-)	RNSNNGATAANNGN	ATATGTATCTTTCT
M00128	V$GATA1_04	0.817708	1444	(-)	NNCWGATARNNNN	ATATGTATCTTTC
M00075	V$GATA1_01	0.792695	1446	(-)	SNNGATNNNN	ATGTATCTTT
M00076	V$GATA2_01	0.833108	1446	(-)	NNNGATRNNN	ATGTATCTTT
M00075	V$GATA1_01	0.780355	1474	(-)	SNNGATNNNN	AAAAATCAAA
M00128	V$GATA1_04	0.832721	1478	(+)	NNCWGATARNNNN	ATCAAATAAGCAG
M00127	V$GATA1_03	0.830475	1496	(-)	RNSNNGATAANNGN	ACTCCTATACTCCT
M00126	V$GATA1_02	0.893437	1512	(+)	NNNNNGATANKGNN	AGAAAGATAAGGCT
M00127	V$GATA1_03	0.898824	1512	(+)	RNSNNGATAANNGN	AGAAAGATAAGGCT
M00128	V$GATA1_04	0.943627	1513	(+)	NNCWGATARNNNN	GAAAGATAAGGCT
M00075	V$GATA1_01	0.816387	1514	(+)	SNNGATNNNN	AAAGATAAGG
M00076	V$GATA2_01	0.866486	1514	(+)	NNNGATRNNN	AAAGATAAGG
M00077	V$GATA3_01	0.954364	1515	(+)	NNGATARNG	AAGATAAGG
M00203	V$GATA_C	0.977322	1516	(+)	NGATAAGNMNN	AGATAAGGCTA
M00203	V$GATA_C	0.84343	1553	(-)	NGATAAGNMNN	AAGCCCTGTCA
M00076	V$GATA2_01	0.813712	1565	(+)	NNNGATRNNN	TGTCATACCT
M00076	V$GATA2_01	0.797023	1571	(-)	NNNGATRNNN	ACCTATGTTT
M00075	V$GATA1_01	0.887957	1588	(-)	SNNGATNNNN	AACAATCAAG
M00076	V$GATA2_01	0.782589	1588	(-)	NNNGATRNNN	AACAATCAAG
M00127	V$GATA1_03	0.808182	1592	(+)	RNSNNGATAANNGN	ATCAAGTTAATCTC
M00077	V$GATA3_01	0.830306	1597	(-)	NNGATARNG	GTTAATCTC
M00075	V$GATA1_01	0.790227	1602	(-)	SNNGATNNNN	TCTCATCATT
M00076	V$GATA2_01	0.783942	1602	(-)	NNNGATRNNN	TCTCATCATT
M00077	V$GATA3_01	0.842268	1602	(-)	NNGATARNG	TCTCATCAT
M00075	V$GATA1_01	0.882527	1612	(+)	SNNGATNNNN	CATGATGACT
M00076	V$GATA2_01	0.867839	1612	(+)	NNNGATRNNN	CATGATGACT
M00127	V$GATA1_03	0.78197	1657	(-)	RNSNNGATAANNGN	CCCTCCATCTCCTC

M00075	V$GATA1_01	0.923001	1659	(-)	SNNGATNNNN	CTCCATCTCC
M00076	V$GATA2_01	0.89806	1659	(-)	NNNGATRNNN	CTCCATCTCC
M00077	V$GATA3_01	0.905627	1659	(-)	NNGATARNG	CTCCATCTC
M00075	V$GATA1_01	0.838105	1668	(-)	SNNGATNNNN	CTCCATCTTA
M00076	V$GATA2_01	0.875507	1668	(-)	NNNGATRNNN	CTCCATCTTA
M00077	V$GATA3_01	0.888347	1668	(-)	NNGATARNG	CTCCATCTT
M00075	V$GATA1_01	0.936328	1692	(-)	SNNGATNNNN	ACCAATCATG
M00076	V$GATA2_01	0.870546	1692	(-)	NNNGATRNNN	ACCAATCATG
M00077	V$GATA3_01	0.858662	1692	(-)	NNGATARNG	ACCAATCAT
M00126	V$GATA1_02	0.895	1761	(+)	NNNNNGATANKGNN	GCCCAGATAAAGGA
M00127	V$GATA1_03	0.88584	1761	(+)	RNSNNGATAANNGN	GCCCAGATAAAGGA
M00128	V$GATA1_04	0.877145	1762	(+)	NNCWGATARNNNN	CCCAGATAAAGGA
M00075	V$GATA1_01	0.854393	1763	(+)	SNNGATNNNN	CCAGATAAAG
M00076	V$GATA2_01	0.869644	1763	(+)	NNNGATRNNN	CCAGATAAAG
M00077	V$GATA3_01	0.907842	1764	(+)	NNGATARNG	CAGATAAAG
M00203	V$GATA_C	0.921715	1765	(+)	NGATAAGNMNN	AGATAAAGGAA
M00126	V$GATA1_02	0.909062	1799	(+)	NNNNNGATANKGNN	GTAGAGATAGAGCC
M00127	V$GATA1_03	0.83317	1799	(+)	RNSNNGATAANNGN	GTAGAGATAGAGCC
M00128	V$GATA1_04	0.898591	1800	(+)	NNCWGATARNNNN	TAGAGATAGAGCC
M00075	V$GATA1_01	0.858342	1801	(+)	SNNGATNNNN	AGAGATAGAG
M00076	V$GATA2_01	0.879567	1801	(+)	NNNGATRNNN	AGAGATAGAG
M00077	V$GATA3_01	0.959238	1802	(+)	NNGATARNG	GAGATAGAG
M00203	V$GATA_C	0.931034	1803	(+)	NGATAAGNMNN	AGATAGAGCCT
M00075	V$GATA1_01	0.772458	1811	(+)	SNNGATNNNN	CCTGAGATGG
M00075	V$GATA1_01	0.819842	1813	(+)	SNNGATNNNN	TGAGATGGAA
M00076	V$GATA2_01	0.840776	1813	(+)	NNNGATRNNN	TGAGATGGAA
M00077	V$GATA3_01	0.870625	1814	(+)	NNGATARNG	GAGATGGAA
M00075	V$GATA1_01	0.779862	1869	(-)	SNNGATNNNN	ACCACTCAGC
M00076	V$GATA2_01	0.793866	1919	(+)	NNNGATRNNN	TGGCATGGGA
M00203	V$GATA_C	0.875427	1962	(-)	NGATAAGNMNN	CAACTTTATCT
M00126	V$GATA1_02	0.867812	1963	(-)	NNNNNGATANKGNN	AACTTTATCTTTCC
M00127	V$GATA1_03	0.850318	1963	(-)	RNSNNGATAANNGN	AACTTTATCTTTCC
M00128	V$GATA1_04	0.886642	1963	(-)	NNCWGATARNNNN	AACTTTATCTTTC

M00076	V$GATA2_01	0.820027	1965	(-)	NNNGATRNNN	CTTTATCTTT
M00077	V$GATA3_01	0.913602	1965	(-)	NNGATARNG	CTTTATCTT
M00126	V$GATA1_02	0.797188	1974	(-)	NNNNNGATANKGNN	TCCCTTTTCTCCAT
M00127	V$GATA1_03	0.853993	1974	(-)	RNSNNGATAANNGN	TCCCTTTTCTCCAT
M00076	V$GATA2_01	0.791159	1998	(+)	NNNGATRNNN	ATGGATATTC
M00203	V$GATA_C	0.831625	2011	(-)	NGATAAGNMNN	AGGCTTTCTCT
M00077	V$GATA3_01	0.830306	2040	(-)	NNGATARNG	ATTAATCTC
M00126	V$GATA1_02	0.857812	2065	(-)	NNNNNGATANKGNN	ACCTGTATCTCCCA
M00127	V$GATA1_03	0.796913	2065	(-)	RNSNNGATAANNGN	ACCTGTATCTCCCA
M00128	V$GATA1_04	0.836397	2065	(-)	NNCWGATARNNNN	ACCTGTATCTCCC
M00075	V$GATA1_01	0.897335	2067	(-)	SNNGATNNNN	CTGTATCTCC
M00076	V$GATA2_01	0.901218	2067	(-)	NNNGATRNNN	CTGTATCTCC
M00077	V$GATA3_01	0.879929	2067	(-)	NNGATARNG	CTGTATCTC
M00075	V$GATA1_01	0.836624	2091	(+)	SNNGATNNNN	GTTGATTTCT
M00203	V$GATA_C	0.830693	2095	(-)	NGATAAGNMNN	ATTTCTTAACT
M00128	V$GATA1_04	0.816176	2096	(-)	NNCWGATARNNNN	TTTCTTAACTGAA
M00127	V$GATA1_03	0.817001	2118	(+)	RNSNNGATAANNGN	ATGTAGAAAGCCAC
M00127	V$GATA1_03	0.790789	2145	(-)	RNSNNGATAANNGN	TCGACTTTCTTTCC
M00127	V$GATA1_03	0.840274	2179	(-)	RNSNNGATAANNGN	GCAACTCTCTTCCT
M00075	V$GATA1_01	0.781343	2196	(+)	SNNGATNNNN	ATTGATGCTA
M00126	V$GATA1_02	0.811875	2232	(+)	NNNNNGATANKGNN	ACCTTGATAATCTC
M00127	V$GATA1_03	0.879226	2232	(+)	RNSNNGATAANNGN	ACCTTGATAATCTC
M00075	V$GATA1_01	0.831688	2234	(+)	SNNGATNNNN	CTTGATAATC
M00076	V$GATA2_01	0.792061	2234	(+)	NNNGATRNNN	CTTGATAATC
M00203	V$GATA_C	0.849953	2236	(+)	NGATAAGNMNN	TGATAATCTCA
M00075	V$GATA1_01	0.791214	2237	(-)	SNNGATNNNN	GATAATCTCA
M00076	V$GATA2_01	0.819576	2237	(-)	NNNGATRNNN	GATAATCTCA
M00077	V$GATA3_01	0.850244	2237	(-)	NNGATARNG	GATAATCTC
M00127	V$GATA1_03	0.856933	2342	(-)	RNSNNGATAANNGN	ACCATTATGTGCCT
M00076	V$GATA2_01	0.807397	2344	(-)	NNNGATRNNN	CATTATGTGC
M00126	V$GATA1_02	0.795313	2383	(+)	NNNNNGATANKGNN	GTATACATAGGGAC
M00127	V$GATA1_03	0.784174	2432	(-)	RNSNNGATAANNGN	CTACCTGTCCTTCT
M00203	V$GATA_C	0.847158	2437	(-)	NGATAAGNMNN	TGTCCTTCTCT

M00126	V$GATA1_02	0.860625	2569	(+)	NNNNNGATANKGNN	GTAATGATACTTTT
M00127	V$GATA1_03	0.797893	2569	(+)	RNSNNGATAANNGN	GTAATGATACTTTT
M00075	V$GATA1_01	0.852912	2571	(+)	SNNGATNNNN	AATGATACTT
M00076	V$GATA2_01	0.861976	2571	(+)	NNNGATRNNN	AATGATACTT
M00075	V$GATA1_01	0.780355	2595	(-)	SNNGATNNNN	AAAAATCAAA
M00203	V$GATA_C	0.895309	2665	(-)	NGATAAGNMNN	TGGCTATATCT
M00126	V$GATA1_02	0.783438	2666	(-)	NNNNNGATANKGNN	GGCTATATCTACCC
M00128	V$GATA1_04	0.836091	2666	(-)	NNCWGATARNNNNN	GGCTATATCTACC
M00075	V$GATA1_01	0.811451	2668	(-)	SNNGATNNNN	CTATATCTAC
M00076	V$GATA2_01	0.80018	2668	(-)	NNNGATRNNN	CTATATCTAC
M00077	V$GATA3_01	0.857333	2668	(-)	NNGATARNG	CTATATCTA
M00075	V$GATA1_01	0.780849	2680	(-)	SNNGATNNNN	ATAAATCTAG
M00075	V$GATA1_01	0.860316	2724	(-)	SNNGATNNNN	CCAAATCACT
M00076	V$GATA2_01	0.813712	2724	(-)	NNNGATRNNN	CCAAATCACT
M00077	V$GATA3_01	0.882588	2724	(-)	NNGATARNG	CCAAATCAC
M00075	V$GATA1_01	0.88845	2801	(-)	SNNGATNNNN	TGCAATCATG
M00076	V$GATA2_01	0.853406	2801	(-)	NNNGATRNNN	TGCAATCATG
M00077	V$GATA3_01	0.853788	2801	(-)	NNGATARNG	TGCAATCAT
M00203	V$GATA_C	0.84343	2828	(-)	NGATAAGNMNN	TGTGACTATCC
M00126	V$GATA1_02	0.783125	2829	(-)	NNNNNGATANKGNN	GTGACTATCCTGGC
M00127	V$GATA1_03	0.880206	2829	(-)	RNSNNGATAANNGN	GTGACTATCCTGGC
M00075	V$GATA1_01	0.9077	2831	(-)	SNNGATNNNN	GACTATCCTG
M00076	V$GATA2_01	0.950834	2831	(-)	NNNGATRNNN	GACTATCCTG
M00077	V$GATA3_01	0.849801	2831	(-)	NNGATARNG	GACTATCCT
M00126	V$GATA1_02	0.785	2840	(-)	NNNNNGATANKGNN	GGCATTAACTCTAC
M00127	V$GATA1_03	0.791524	2943	(-)	RNSNNGATAANNGN	ATAGTTATATTTAT
M00127	V$GATA1_03	0.830475	2959	(+)	RNSNNGATAANNGN	AGCAAAATAGCTGC
M00127	V$GATA1_03	0.78001	3009	(-)	RNSNNGATAANNGN	ACATTTATTTCTAT
M00075	V$GATA1_01	0.79615	3034	(+)	SNNGATNNNN	AAAGATGTTC
M00076	V$GATA2_01	0.82995	3034	(+)	NNNGATRNNN	AAAGATGTTC
M00075	V$GATA1_01	0.837611	3045	(-)	SNNGATNNNN	GGACATCAGT
M00076	V$GATA2_01	0.845737	3045	(-)	NNNGATRNNN	GGACATCAGT
M00075	V$GATA1_01	0.845015	3067	(-)	SNNGATNNNN	AGAAATCACA

M00076	V$GATA2_01	0.845737	3067	(-)	NNNGATRNNN	AGAAATCACA
M00203	V$GATA_C	0.851817	3074	(-)	NGATAAGNMNN	ACACTTTATCA
M00126	V$GATA1_02	0.845625	3075	(-)	NNNNNGATANKGNN	CACTTTATCATGTA
M00128	V$GATA1_04	0.894914	3075	(-)	NNCWGATARNNNNN	CACTTTATCATGT
M00075	V$GATA1_01	0.854393	3077	(-)	SNNGATNNNN	CTTTATCATG
M00076	V$GATA2_01	0.850248	3077	(-)	NNNGATRNNN	CTTTATCATG
M00077	V$GATA3_01	0.89012	3077	(-)	NNGATARNG	CTTTATCAT
M00075	V$GATA1_01	0.772458	3092	(-)	SNNGATNNNN	GAAAATCTTT
M00076	V$GATA2_01	0.793866	3092	(-)	NNNGATRNNN	GAAAATCTTT
M00126	V$GATA1_02	0.802188	3110	(+)	NNNNNGATANKGNN	CATATGATAGAGAA
M00128	V$GATA1_04	0.910233	3111	(+)	NNCWGATARNNNNN	ATATGATAGAGAA
M00075	V$GATA1_01	0.858835	3112	(+)	SNNGATNNNN	TATGATAGAG
M00076	V$GATA2_01	0.892648	3112	(+)	NNNGATRNNN	TATGATAGAG
M00077	V$GATA3_01	0.918476	3113	(+)	NNGATARNG	ATGATAGAG
M00203	V$GATA_C	0.894998	3114	(+)	NGATAAGNMNN	TGATAGAGAAA
M00126	V$GATA1_02	0.793125	3149	(+)	NNNNNGATANKGNN	CTGAAGATATTTTT
M00075	V$GATA1_01	0.875617	3151	(+)	SNNGATNNNN	GAAGATATTT
M00076	V$GATA2_01	0.908886	3151	(+)	NNNGATRNNN	GAAGATATTT
M00077	V$GATA3_01	0.834736	3152	(+)	NNGATARNG	AAGATATTT
M00076	V$GATA2_01	0.785747	3169	(-)	NNNGATRNNN	TAATATGCTC
M00075	V$GATA1_01	0.816387	3207	(-)	SNNGATNNNN	AAAAATCATA
M00076	V$GATA2_01	0.818674	3207	(-)	NNNGATRNNN	AAAAATCATA
M00075	V$GATA1_01	0.782823	3220	(+)	SNNGATNNNN	CAGGATCAGA
M00075	V$GATA1_01	0.842053	3220	(-)	SNNGATNNNN	CAGGATCAGA
M00076	V$GATA2_01	0.815065	3220	(+)	NNNGATRNNN	CAGGATCAGA
M00076	V$GATA2_01	0.85115	3220	(-)	NNNGATRNNN	CAGGATCAGA
M00203	V$GATA_C	0.92886	3244	(-)	NGATAAGNMNN	AATTCCTATCT
M00126	V$GATA1_02	0.834063	3245	(-)	NNNNNGATANKGNN	ATTCCTATCTCACA
M00128	V$GATA1_04	0.926164	3245	(-)	NNCWGATARNNNNN	ATTCCTATCTCAC
M00075	V$GATA1_01	0.871668	3247	(-)	SNNGATNNNN	TCCTATCTCA
M00076	V$GATA2_01	0.915652	3247	(-)	NNNGATRNNN	TCCTATCTCA
M00077	V$GATA3_01	0.964998	3247	(-)	NNGATARNG	TCCTATCTC
M00126	V$GATA1_02	0.798125	3262	(-)	NNNNNGATANKGNN	ACCCCAATCACTTT

M00075	V$GATA1_01	0.907206	3264	(-)	SNNGATNNNN	CCCAATCACT
M00076	V$GATA2_01	0.850248	3264	(-)	NNNGATRNNN	CCCAATCACT
M00077	V$GATA3_01	0.942844	3264	(-)	NNGATARNG	CCCAATCAC
M00126	V$GATA1_02	0.788438	3290	(+)	NNNNNGATANKGNN	TAAAAGATTGGGAA
M00075	V$GATA1_01	0.857848	3292	(+)	SNNGATNNNN	AAAGATTGGG
M00076	V$GATA2_01	0.853406	3292	(+)	NNNGATRNNN	AAAGATTGGG
M00077	V$GATA3_01	0.949047	3293	(+)	NNGATARNG	AAGATTGGG
M00075	V$GATA1_01	0.837117	3340	(-)	SNNGATNNNN	GCACATCTAC
M00076	V$GATA2_01	0.801534	3340	(-)	NNNGATRNNN	GCACATCTAC
M00126	V$GATA1_02	0.807187	3418	(+)	NNNNNGATANKGNN	CCAGGGATACTGTC
M00127	V$GATA1_03	0.791034	3418	(+)	RNSNNGATAANNGN	CCAGGGATACTGTC
M00075	V$GATA1_01	0.86772	3420	(+)	SNNGATNNNN	AGGGATACTG
M00076	V$GATA2_01	0.908886	3420	(+)	NNNGATRNNN	AGGGATACTG
M00077	V$GATA3_01	0.854674	3421	(+)	NNGATARNG	GGGATACTG
M00075	V$GATA1_01	0.773445	3432	(-)	SNNGATNNNN	CATAATCAAA
M00077	V$GATA3_01	0.852016	3432	(-)	NNGATARNG	CATAATCAA
M00077	V$GATA3_01	0.82942	3437	(-)	NNGATARNG	TCAAATCTA
M00126	V$GATA1_02	0.799063	3485	(-)	NNNNNGATANKGNN	AAAGCTATCTGAAA
M00128	V$GATA1_04	0.878064	3485	(-)	NNCWGATARNNNNN	AAAGCTATCTGAA
M00075	V$GATA1_01	0.886969	3487	(-)	SNNGATNNNN	AGCTATCTGA
M00076	V$GATA2_01	0.958051	3487	(-)	NNNGATRNNN	AGCTATCTGA
M00077	V$GATA3_01	0.873283	3487	(-)	NNGATARNG	AGCTATCTG
M00075	V$GATA1_01	0.876111	3514	(+)	SNNGATNNNN	GGGGATTTTT
M00076	V$GATA2_01	0.866937	3514	(+)	NNNGATRNNN	GGGGATTTTT
M00127	V$GATA1_03	0.791034	3541	(-)	RNSNNGATAANNGN	CTTGTTTTCATTGT
M00203	V$GATA_C	0.832246	3558	(-)	NGATAAGNMNN	TTTGGCTATCA
M00127	V$GATA1_03	0.930181	3559	(-)	RNSNNGATAANNGN	TTGGCTATCACCCT
M00128	V$GATA1_04	0.855392	3559	(-)	NNCWGATARNNNNN	TTGGCTATCACCC
M00075	V$GATA1_01	0.957058	3561	(-)	SNNGATNNNN	GGCTATCACC
M00076	V$GATA2_01	0.9576	3561	(-)	NNNGATRNNN	GGCTATCACC
M00077	V$GATA3_01	0.87284	3561	(-)	NNGATARNG	GGCTATCAC
M00075	V$GATA1_01	0.859329	3587	(-)	SNNGATNNNN	TTCAATCGGC
M00076	V$GATA2_01	0.815968	3587	(-)	NNNGATRNNN	TTCAATCGGC

M00077	V$GATA3_01	0.837838	3587	(-)	NNGATARNG	TTCAATCGG
M00075	V$GATA1_01	0.853406	3594	(+)	SNNGATNNNN	GGCGATCCCA
M00075	V$GATA1_01	0.824284	3594	(-)	SNNGATNNNN	GGCGATCCCA
M00076	V$GATA2_01	0.849797	3594	(+)	NNNGATRNNN	GGCGATCCCA
M00076	V$GATA2_01	0.904375	3594	(-)	NNNGATRNNN	GGCGATCCCA
M00128	V$GATA1_04	0.820159	3605	(-)	NNCWGATARNNNN	GCACTTATTTGCA
M00075	V$GATA1_01	0.834156	3643	(+)	SNNGATNNNN	TACGATGTTT
M00076	V$GATA2_01	0.862878	3643	(+)	NNNGATRNNN	TACGATGTTT
M00203	V$GATA_C	0.854613	3740	(-)	NGATAAGNMNN	AGTTAGTATCT
M00126	V$GATA1_02	0.82625	3741	(-)	NNNNNGATANKGNN	GTTAGTATCTCCTA
M00127	V$GATA1_03	0.791769	3741	(-)	RNSNNGATAANNGN	GTTAGTATCTCCTA
M00128	V$GATA1_04	0.811275	3741	(-)	NNCWGATARNNNN	GTTAGTATCTCCT
M00075	V$GATA1_01	0.889931	3743	(-)	SNNGATNNNN	TAGTATCTCC
M00076	V$GATA2_01	0.898512	3743	(-)	NNNGATRNNN	TAGTATCTCC
M00077	V$GATA3_01	0.864865	3743	(-)	NNGATARNG	TAGTATCTC
M00076	V$GATA2_01	0.816419	3807	(-)	NNNGATRNNN	GACTATGACA
M00127	V$GATA1_03	0.796668	3820	(-)	RNSNNGATAANNGN	ACATTTATTTTCTT
M00203	V$GATA_C	0.838459	3903	(+)	NGATAAGNMNN	TGACAGGGATT
M00075	V$GATA1_01	0.783317	3907	(+)	SNNGATNNNN	AGGGATTGAA
M00076	V$GATA2_01	0.786649	3907	(+)	NNNGATRNNN	AGGGATTGAA
M00077	V$GATA3_01	0.84537	3908	(+)	NNGATARNG	GGGATTGAA
M00128	V$GATA1_04	0.852941	3917	(-)	NNCWGATARNNNN	CTTTGTATCAGAA
M00075	V$GATA1_01	0.807009	3919	(-)	SNNGATNNNN	TTGTATCAGA
M00076	V$GATA2_01	0.832657	3919	(-)	NNNGATRNNN	TTGTATCAGA
M00075	V$GATA1_01	0.801579	4058	(-)	SNNGATNNNN	AAGAATCAAA
M00076	V$GATA2_01	0.796572	4087	(-)	NNNGATRNNN	TTCAATCTTA
M00077	V$GATA3_01	0.873283	4087	(-)	NNGATARNG	TTCAATCTT
M00126	V$GATA1_02	0.788438	4106	(+)	NNNNNGATANKGNN	AAAATGATACAAAT
M00127	V$GATA1_03	0.807692	4106	(+)	RNSNNGATAANNGN	AAAATGATACAAAT
M00128	V$GATA1_04	0.823836	4107	(+)	NNCWGATARNNNN	AAATGATACAAAT
M00075	V$GATA1_01	0.777887	4108	(+)	SNNGATNNNN	AATGATACAA
M00076	V$GATA2_01	0.792061	4108	(+)	NNNGATRNNN	AATGATACAA
M00203	V$GATA_C	0.873253	4110	(+)	NGATAAGNMNN	TGATACAAATT

M00203	V$GATA_C	0.835663	4172	(-)	NGATAAGNMNN	ATTCAATATCT
M00126	V$GATA1_02	0.836562	4173	(-)	NNNNNGATANKGNN	TTCAATATCTTGTA
M00128	V$GATA1_04	0.846814	4173	(-)	NNCWGATARNNNNN	TTCAATATCTTGT
M00075	V$GATA1_01	0.891412	4175	(-)	SNNGATNNNN	CAATATCTTG
M00076	V$GATA2_01	0.916554	4175	(-)	NNNGATRNNN	CAATATCTTG
M00077	V$GATA3_01	0.901639	4175	(-)	NNGATARNG	CAATATCTT
M00076	V$GATA2_01	0.8083	4247	(-)	NNNGATRNNN	CATTATGCTG
M00076	V$GATA2_01	0.785747	4352	(-)	NNNGATRNNN	AGCAATGTGA
M00127	V$GATA1_03	0.788829	4355	(+)	RNSNNGATAANNGN	AATGTGATAGCATC
M00128	V$GATA1_04	0.859069	4356	(+)	NNCWGATARNNNNN	ATGTGATAGCATC
M00075	V$GATA1_01	0.880059	4357	(+)	SNNGATNNNN	TGTGATAGCA
M00076	V$GATA2_01	0.917005	4357	(+)	NNNGATRNNN	TGTGATAGCA
M00077	V$GATA3_01	0.904741	4358	(+)	NNGATARNG	GTGATAGCA
M00075	V$GATA1_01	0.796644	4362	(-)	SNNGATNNNN	TAGCATCTTT
M00076	V$GATA2_01	0.815065	4362	(-)	NNNGATRNNN	TAGCATCTTT
M00203	V$GATA_C	0.83908	4386	(-)	NGATAAGNMNN	ATTCCTTCTCT
M00077	V$GATA3_01	0.847585	4389	(-)	NNGATARNG	CCTTCTCTC
M00126	V$GATA1_02	0.869687	4411	(+)	NNNNNGATANKGNN	GTTAAGATAATATT
M00127	V$GATA1_03	0.798628	4411	(+)	RNSNNGATAANNGN	GTTAAGATAATATT
M00128	V$GATA1_04	0.860907	4412	(+)	NNCWGATARNNNNN	TTAAGATAATATT
M00076	V$GATA2_01	0.849797	4413	(+)	NNNGATRNNN	TAAGATAATA
M00077	V$GATA3_01	0.898538	4414	(+)	NNGATARNG	AAGATAATA
M00203	V$GATA_C	0.859584	4415	(+)	NGATAAGNMNN	AGATAATATTT
M00075	V$GATA1_01	0.77542	4440	(+)	SNNGATNNNN	TGAGATGAAC
M00076	V$GATA2_01	0.815516	4440	(+)	NNNGATRNNN	TGAGATGAAC
M00077	V$GATA3_01	0.830306	4452	(-)	NNGATARNG	CATAATCCA
M00127	V$GATA1_03	0.801323	4527	(-)	RNSNNGATAANNGN	CTTTTTGTCCCCCC
M00126	V$GATA1_02	0.83625	4557	(-)	NNNNNGATANKGNN	AAAACTATCTTAAG
M00128	V$GATA1_04	0.840074	4557	(-)	NNCWGATARNNNNN	AAAACTATCTTAA
M00075	V$GATA1_01	0.866239	4559	(-)	SNNGATNNNN	AACTATCTTA
M00076	V$GATA2_01	0.930988	4559	(-)	NNNGATRNNN	AACTATCTTA
M00077	V$GATA3_01	0.894993	4559	(-)	NNGATARNG	AACTATCTT
M00075	V$GATA1_01	0.793189	4569	(-)	SNNGATNNNN	AGGAATCAAT

M0007		0.83761				
5	V$GATA1_01	1	4598	(+)	SNNGATNNNN	ACTGATATCC
M0007		0.87415				
6	V$GATA2_01	4	4598	(+)	NNNGATRNNN	ACTGATATCC
M0012		0.82116				
7	V$GATA1_03	6	4598	(-)	RNSNNGATAANNGN	ACTGATATCCAGAT
M0007		0.80404				
5	V$GATA1_01	7	4600	(-)	SNNGATNNNN	TGATATCCAG
M0007						
6	V$GATA2_01	0.82544	4600	(-)	NNNGATRNNN	TGATATCCAG
M0007		0.86525				
5	V$GATA1_01	2	4606	(+)	SNNGATNNNN	CCAGATGTTA
M0007		0.85340				
6	V$GATA2_01	6	4606	(+)	NNNGATRNNN	CCAGATGTTA
M0007		0.81688				
5	V$GATA1_01	1	4615	(-)	SNNGATNNNN	ATGCATCCCT
M0007		0.82724				
6	V$GATA2_01	4	4615	(-)	NNNGATRNNN	ATGCATCCCT
M0012		0.78466				
7	V$GATA1_03	4	4688	(+)	RNSNNGATAANNGN	CAGGACATAATCAT
M0007		0.79070				
6	V$GATA2_01	8	4690	(+)	NNNGATRNNN	GGACATAATC
M0007		0.80108				
5	V$GATA1_01	6	4693	(-)	SNNGATNNNN	CATAATCATT
M0007		0.78439				
6	V$GATA2_01	3	4693	(-)	NNNGATRNNN	CATAATCATT
M0007		0.87638				
7	V$GATA3_01	5	4693	(-)	NNGATARNG	CATAATCAT
M0007		0.82002				
6	V$GATA2_01	7	4741	(-)	NNNGATRNNN	AACTATGAGA
M0012		0.86093				
6	V$GATA1_02	8	4744	(+)	NNNNNGATANKGNN	TATGAGATAATGAA
M0012		0.90563				
8	V$GATA1_04	7	4745	(+)	NNCWGATARNNNN	ATGAGATAATGAA
M0007		0.84106				
5	V$GATA1_01	6	4746	(+)	SNNGATNNNN	TGAGATAATG
M0007		0.90076				
6	V$GATA2_01	7	4746	(+)	NNNGATRNNN	TGAGATAATG
M0007						
7	V$GATA3_01	0.95082	4747	(+)	NNGATARNG	GAGATAATG
M0020		0.90214				
3	V$GATA_C	4	4748	(+)	NGATAAGNMNN	AGATAATGAAA
M0007		0.78134				
5	V$GATA1_01	3	4769	(+)	SNNGATNNNN	AGAGATCATG
M0007		0.84945				
5	V$GATA1_01	7	4769	(-)	SNNGATNNNN	AGAGATCATG
M0007		0.80378				
6	V$GATA2_01	9	4769	(+)	NNNGATRNNN	AGAGATCATG
M0007						
6	V$GATA2_01	0.84664	4769	(-)	NNNGATRNNN	AGAGATCATG
M0007		0.83517				
7	V$GATA3_01	9	4770	(+)	NNGATARNG	GAGATCATG
M0007		0.81243				
5	V$GATA1_01	8	4794	(+)	SNNGATNNNN	TAAGATGCAG
M0007		0.85024				
6	V$GATA2_01	8	4794	(+)	NNNGATRNNN	TAAGATGCAG
M0012		0.77281				
6	V$GATA1_02	2	4799	(+)	NNNNNGATANKGNN	TGCAGGATACGAAA
M0012						
7	V$GATA1_03	0.78197	4799	(+)	RNSNNGATAANNGN	TGCAGGATACGAAA
M0007		0.87364				
5	V$GATA1_01	3	4801	(+)	SNNGATNNNN	CAGGATACGA
M0007		0.90572				
6	V$GATA2_01	8	4801	(+)	NNNGATRNNN	CAGGATACGA
M0007		0.82321				
7	V$GATA3_01	7	4802	(+)	NNGATARNG	AGGATACGA

M00203	V$GATA_C	0.893756	4803	(+)	NGATAAGNMNN	GGATACGAAAA
M00075	V$GATA1_01	0.819348	4860	(+)	SNNGATNNNN	TAAGATGTCT
M00076	V$GATA2_01	0.88498	4860	(+)	NNNGATRNNN	TAAGATGTCT
M00127	V$GATA1_03	0.800343	4866	(+)	RNSNNGATAANNGN	GTCTAGATTATCAG
M00075	V$GATA1_01	0.787266	4868	(+)	SNNGATNNNN	CTAGATTATC
M00126	V$GATA1_02	0.811562	4869	(-)	NNNNNGATANKGNN	TAGATTATCAGAAT
M00127	V$GATA1_03	0.812347	4869	(-)	RNSNNGATAANNGN	TAGATTATCAGAAT
M00128	V$GATA1_04	0.85386	4869	(-)	NNCWGATARNNNNN	TAGATTATCAGAA
M00075	V$GATA1_01	0.827739	4871	(-)	SNNGATNNNN	GATTATCAGA
M00076	V$GATA2_01	0.872801	4871	(-)	NNNGATRNNN	GATTATCAGA
M00077	V$GATA3_01	0.837395	4871	(-)	NNGATARNG	GATTATCAG
M00203	V$GATA_C	0.840012	4967	(-)	NGATAAGNMNN	AGTCTCTGTCT
M00127	V$GATA1_03	0.798138	4993	(-)	RNSNNGATAANNGN	TCTATTATTTTTTT
M00076	V$GATA2_01	0.78304	5111	(-)	NNNGATRNNN	AATTATGACC
M00075	V$GATA1_01	0.868213	5119	(-)	SNNGATNNNN	CCTCATCGCA
M00076	V$GATA2_01	0.875507	5119	(-)	NNNGATRNNN	CCTCATCGCA
M00077	V$GATA3_01	0.888347	5119	(-)	NNGATARNG	CCTCATCGC
M00075	V$GATA1_01	0.827246	5217	(-)	SNNGATNNNN	TGCAATCCAG
M00076	V$GATA2_01	0.797474	5217	(-)	NNNGATRNNN	TGCAATCCAG
M00075	V$GATA1_01	0.836624	5259	(+)	SNNGATNNNN	GTTGATTTTT
M00075	V$GATA1_01	0.872655	5341	(-)	SNNGATNNNN	AAAAATCATC
M00076	V$GATA2_01	0.833108	5341	(-)	NNNGATRNNN	AAAAATCATC
M00075	V$GATA1_01	0.801086	5344	(-)	SNNGATNNNN	AATCATCTTA
M00076	V$GATA2_01	0.85115	5344	(-)	NNNGATRNNN	AATCATCTTA
M00127	V$GATA1_03	0.804018	5348	(+)	RNSNNGATAANNGN	ATCTTAATAGGAGG
M00126	V$GATA1_02	0.783125	5450	(-)	NNNNNGATANKGNN	GCAAATATCACGTG
M00127	V$GATA1_03	0.788339	5450	(-)	RNSNNGATAANNGN	GCAAATATCACGTG
M00128	V$GATA1_04	0.831189	5450	(-)	NNCWGATARNNNNN	GCAAATATCACGT
M00075	V$GATA1_01	0.929418	5452	(-)	SNNGATNNNN	AAATATCACG
M00076	V$GATA2_01	0.900316	5452	(-)	NNNGATRNNN	AAATATCACG
M00077	V$GATA3_01	0.828533	5452	(-)	NNGATARNG	AAATATCAC
M00075	V$GATA1_01	0.807009	5482	(+)	SNNGATNNNN	TCAGATGATC
M00076	V$GATA2_01	0.855661	5482	(+)	NNNGATRNNN	TCAGATGATC

M00075	V$GATA1_01	0.791214	5485	(+)	SNNGATNNNN	GATGATCACA
M00075	V$GATA1_01	0.784304	5485	(-)	SNNGATNNNN	GATGATCACA
M00076	V$GATA2_01	0.807397	5485	(+)	NNNGATRNNN	GATGATCACA
M00076	V$GATA2_01	0.8083	5485	(-)	NNNGATRNNN	GATGATCACA
M00127	V$GATA1_03	0.793484	5509	(-)	RNSNNGATAANNGN	GAATTTTTCTCCCT
M00075	V$GATA1_01	0.807996	5527	(+)	SNNGATNNNN	AATGATTTTT
M00076	V$GATA2_01	0.786198	5527	(+)	NNNGATRNNN	AATGATTTTT
M00127	V$GATA1_03	0.784174	5559	(-)	RNSNNGATAANNGN	TTCATTATTACTAT
M00075	V$GATA1_01	0.806022	5580	(-)	SNNGATNNNN	GTATATCACT
M00076	V$GATA2_01	0.815065	5580	(-)	NNNGATRNNN	GTATATCACT
M00077	V$GATA3_01	0.82942	5619	(+)	NNGATARNG	TAGATTAGT
M00203	V$GATA_C	0.866729	5685	(-)	NGATAAGNMNN	ATGTTTTGTCT
M00126	V$GATA1_02	0.8225	5704	(-)	NNNNNGATANKGNN	AAAGGTATCTTTAA
M00075	V$GATA1_01	0.832182	5706	(-)	SNNGATNNNN	AGGTATCTTT
M00076	V$GATA2_01	0.892197	5706	(-)	NNNGATRNNN	AGGTATCTTT
M00075	V$GATA1_01	0.820336	5727	(-)	SNNGATNNNN	AAAAATCTAG
M00203	V$GATA_C	0.833799	5769	(-)	NGATAAGNMNN	ATTACTTCTCT
M00128	V$GATA1_04	0.810968	5770	(-)	NNCWGATARNNNNN	TTACTTCTCTGAA
M00075	V$GATA1_01	0.817374	5778	(-)	SNNGATNNNN	CTGAATCTCA
M00076	V$GATA2_01	0.822282	5778	(-)	NNNGATRNNN	CTGAATCTCA
M00077	V$GATA3_01	0.846256	5778	(-)	NNGATARNG	CTGAATCTC
M00203	V$GATA_C	0.836595	5844	(+)	NGATAAGNMNN	AGACATGGACT
M00126	V$GATA1_02	0.816875	5890	(+)	NNNNNGATANKGNN	TTAATGATATTAAA
M00128	V$GATA1_04	0.811581	5891	(+)	NNCWGATARNNNNN	TAATGATATTAAA
M00075	V$GATA1_01	0.79615	5892	(+)	SNNGATNNNN	AATGATATTA
M00076	V$GATA2_01	0.820929	5892	(+)	NNNGATRNNN	AATGATATTA
M00077	V$GATA3_01	0.843155	5893	(+)	NNGATARNG	ATGATATTA
M00127	V$GATA1_03	0.784664	5949	(-)	RNSNNGATAANNGN	CTAATAATCTGTGT
M00075	V$GATA1_01	0.789733	5951	(-)	SNNGATNNNN	AATAATCTGT
M00076	V$GATA2_01	0.790708	5951	(-)	NNNGATRNNN	AATAATCTGT
M00077	V$GATA3_01	0.827204	5951	(-)	NNGATARNG	AATAATCTG
M00075	V$GATA1_01	0.834156	5966	(-)	SNNGATNNNN	AGTCATCAGT
M00076	V$GATA2_01	0.834461	5966	(-)	NNNGATRNNN	AGTCATCAGT

M00075	V$GATA1_01	0.818855	5977	(-)	SNNGATNNNN	AGCAATCAAT
M00075	V$GATA1_01	0.884501	5981	(-)	SNNGATNNNN	ATCAATCATG
M00076	V$GATA2_01	0.824087	5981	(-)	NNNGATRNNN	ATCAATCATG
M00076	V$GATA2_01	0.78304	5994	(-)	NNNGATRNNN	ATTAATCTCA
M00077	V$GATA3_01	0.830306	5994	(-)	NNGATARNG	ATTAATCTC
M00075	V$GATA1_01	0.856367	6014	(-)	SNNGATNNNN	AAAAATCTTG
M00076	V$GATA2_01	0.838972	6014	(-)	NNNGATRNNN	AAAAATCTTG
M00127	V$GATA1_03	0.888535	6020	(-)	RNSNNGATAANNGN	CTTGGTATCATCTT
M00128	V$GATA1_04	0.824449	6020	(-)	NNCWGATARNNNN	CTTGGTATCATCT
M00075	V$GATA1_01	0.882034	6022	(-)	SNNGATNNNN	TGGTATCATC
M00076	V$GATA2_01	0.89806	6022	(-)	NNNGATRNNN	TGGTATCATC
M00076	V$GATA2_01	0.82138	6025	(-)	NNNGATRNNN	TATCATCTTA
M00077	V$GATA3_01	0.844927	6025	(-)	NNGATARNG	TATCATCTT
M00126	V$GATA1_02	0.78375	6070	(+)	NNNNNGATANKGNN	TTAGCAATAAGAGA
M00075	V$GATA1_01	0.846989	6174	(-)	SNNGATNNNN	ATCCATCTCT
M00076	V$GATA2_01	0.838069	6174	(-)	NNNGATRNNN	ATCCATCTCT
M00077	V$GATA3_01	0.838724	6174	(-)	NNGATARNG	ATCCATCTC
M00126	V$GATA1_02	0.7925	6197	(-)	NNNNNGATANKGNN	ACACTTATATTTAC
M00127	V$GATA1_03	0.795198	6197	(-)	RNSNNGATAANNGN	ACACTTATATTTAC
M00127	V$GATA1_03	0.787114	6206	(-)	RNSNNGATAANNGN	TTTACTTTCTCCTT
M00075	V$GATA1_01	0.848963	6311	(+)	SNNGATNNNN	GTTGATTTGT
M00076	V$GATA2_01	0.794768	6345	(+)	NNNGATRNNN	TGACATATTT
M00076	V$GATA2_01	0.790708	6363	(+)	NNNGATRNNN	GCACATAATC
M00077	V$GATA3_01	0.852016	6366	(-)	NNGATARNG	CATAATCAA
M00126	V$GATA1_02	0.79	6408	(+)	NNNNNGATANKGNN	TTACTGATATTTAA
M00128	V$GATA1_04	0.830882	6409	(+)	NNCWGATARNNNN	TACTGATATTTAA
M00075	V$GATA1_01	0.852419	6410	(+)	SNNGATNNNN	ACTGATATTT
M00076	V$GATA2_01	0.858818	6410	(+)	NNNGATRNNN	ACTGATATTT
M00126	V$GATA1_02	0.852812	6419	(+)	NNNNNGATANKGNN	TAACAGATAAAATT
M00128	V$GATA1_04	0.908088	6420	(+)	NNCWGATARNNNN	AACAGATAAAATT
M00076	V$GATA2_01	0.785295	6421	(+)	NNNGATRNNN	ACAGATAAAA
M00077	V$GATA3_01	0.87284	6422	(+)	NNGATARNG	CAGATAAAA
M00203	V$GATA_C	0.90028	6423	(+)	NGATAAGNMNN	AGATAAAATTA

M00075	V$GATA1_01	0.800099	6454	(-)	SNNGATNNNN	ATACATCCAG
M00075	V$GATA1_01	0.832182	6472	(-)	SNNGATNNNN	CTACATCACT
M00076	V$GATA2_01	0.803338	6472	(-)	NNNGATRNNN	CTACATCACT
M00077	V$GATA3_01	0.821887	6472	(-)	NNGATARNG	CTACATCAC
M00128	V$GATA1_04	0.844363	6493	(+)	NNCWGATARNNNN	TTCTGATATAATT
M00075	V$GATA1_01	0.785785	6494	(+)	SNNGATNNNN	TCTGATATAA
M00076	V$GATA2_01	0.82138	6494	(+)	NNNGATRNNN	TCTGATATAA
M00075	V$GATA1_01	0.897335	6516	(+)	SNNGATNNNN	GGGGATGTGC
M00076	V$GATA2_01	0.905728	6516	(+)	NNNGATRNNN	GGGGATGTGC
M00126	V$GATA1_02	0.790312	6530	(-)	NNNNNGATANKGNN	AGCCTTCTCTCTAG
M00077	V$GATA3_01	0.847585	6532	(-)	NNGATARNG	CCTTCTCTC
M00076	V$GATA2_01	0.81687	6657	(-)	NNNGATRNNN	GAGTATGCCA
M00076	V$GATA2_01	0.792963	6672	(-)	NNNGATRNNN	CTCTATGAGA
M00075	V$GATA1_01	0.836624	6677	(+)	SNNGATNNNN	TGAGATTTGT
M00076	V$GATA2_01	0.844384	6677	(+)	NNNGATRNNN	TGAGATTTGT
M00077	V$GATA3_01	0.839167	6678	(+)	NNGATARNG	GAGATTTGT
M00203	V$GATA_C	0.854613	6678	(-)	NGATAAGNMNN	GAGATTTGTCT
M00203	V$GATA_C	0.907735	6767	(-)	NGATAAGNMNN	ATGAATTATCT
M00126	V$GATA1_02	0.808125	6768	(-)	NNNNNGATANKGNN	TGAATTATCTTACC
M00127	V$GATA1_03	0.799118	6768	(-)	RNSNNGATAANNGN	TGAATTATCTTACC
M00128	V$GATA1_04	0.880515	6768	(-)	NNCWGATARNNNN	TGAATTATCTTAC
M00075	V$GATA1_01	0.801086	6770	(-)	SNNGATNNNN	AATTATCTTA
M00076	V$GATA2_01	0.879567	6770	(-)	NNNGATRNNN	AATTATCTTA
M00077	V$GATA3_01	0.866637	6770	(-)	NNGATARNG	AATTATCTT
M00216	V$TATA_C	0.886718	17	(+)	NCTATAAAAR	GCTTTATAAA
M00252	V$TATA_01	0.833037	18	(+)	STATAAAWRNNNNNN	CTTTATAAATGACAA
M00216	V$TATA_C	0.805123	19	(+)	NCTATAAAAR	TTTATAAATG
M00252	V$TATA_01	0.889368	20	(+)	STATAAAWRNNNNNN	TTATAAATGACAACT
M00252	V$TATA_01	0.791423	142	(+)	STATAAAWRNNNNNN	AGATAAATCACATGC
M00252	V$TATA_01	0.78508	341	(+)	STATAAAWRNNNNNN	GTTTTTATTGCTAAG
M00216	V$TATA_C	0.792976	410	(+)	NCTATAAAAR	TCTGTATAAT
M00252	V$TATA_01	0.795991	545	(+)	STATAAAWRNNNNNN	ACATATGTGGAGGGC
M00216	V$TATA_C	0.754423	583	(+)	NCTATAAAAR	GTCATAATAA

M00252	V$TATA_01	0.770617	584	(+)	STATAAAWRNNNNNN	TCATAATAATCCAGA
M00216	V$TATA_C	0.823607	613	(+)	NCTATAAAAR	AATTTAAAAT
M00216	V$TATA_C	0.802218	630	(+)	NCTATAAAAR	TCTATCTAAA
M00216	V$TATA_C	0.758912	639	(+)	NCTATAAAAR	AGATTATAAT
M00216	V$TATA_C	0.768154	669	(+)	NCTATAAAAR	TTTTTTAAAA
M00216	V$TATA_C	0.890151	670	(+)	NCTATAAAAR	TTTTTAAAAA
M00216	V$TATA_C	0.750726	671	(+)	NCTATAAAAR	TTTTAAAAAT
M00252	V$TATA_01	0.782289	671	(+)	STATAAAWRNNNNNN	TTTTAAAAATTATGA
M00252	V$TATA_01	0.772393	687	(+)	STATAAAWRNNNNNN	TTACATATCCTCACG
M00216	V$TATA_C	0.778981	803	(+)	NCTATAAAAR	TGCTAAAAAA
M00216	V$TATA_C	0.866121	804	(+)	NCTATAAAAR	GCTAAAAAAG
M00252	V$TATA_01	0.825933	805	(+)	STATAAAWRNNNNNN	CTAAAAAAGAGGATC
M00252	V$TATA_01	0.773662	855	(+)	STATAAAWRNNNNNN	GTGTAAATCTGTATA
M00252	V$TATA_01	0.774423	865	(+)	STATAAAWRNNNNNN	GTATATGTGTTATAA
M00216	V$TATA_C	0.859519	926	(+)	NCTATAAAAR	ACTACAAAAA
M00252	V$TATA_01	0.831515	927	(+)	STATAAAWRNNNNNN	CTACAAAAAAGAAAT
M00252	V$TATA_01	0.804872	998	(+)	STATAAAWRNNNNNN	CAATATATAAATATA
M00216	V$TATA_C	0.802218	999	(+)	NCTATAAAAR	AATATATAAA
M00252	V$TATA_01	0.877696	1000	(+)	STATAAAWRNNNNNN	ATATATAAATATATC
M00216	V$TATA_C	0.751782	1001	(+)	NCTATAAAAR	TATATAAATA
M00252	V$TATA_01	0.855874	1002	(+)	STATAAAWRNNNNNN	ATATAAATATATCAA
M00216	V$TATA_C	0.79456	1025	(+)	NCTATAAAAR	GTCATATAAT
M00252	V$TATA_01	0.808932	1033	(+)	STATAAAWRNNNNNN	ATTTAAATTTACACA
M00216	V$TATA_C	0.764721	1070	(+)	NCTATAAAAR	GTGTTAAAAA
M00216	V$TATA_C	0.806443	1071	(+)	NCTATAAAAR	TGTTAAAAAA
M00216	V$TATA_C	0.764457	1072	(+)	NCTATAAAAR	GTTAAAAAAT
M00252	V$TATA_01	0.82238	1106	(+)	STATAAAWRNNNNNN	GAATTAATAGGGAAC
M00216	V$TATA_C	0.743068	1131	(+)	NCTATAAAAR	TCATTGAAAT
M00216	V$TATA_C	0.755479	1190	(+)	NCTATAAAAR	CCTATCAGAG
M00216	V$TATA_C	0.759176	1225	(+)	NCTATAAAAR	TCTTTCTAAT
M00216	V$TATA_C	0.751518	1247	(+)	NCTATAAAAR	TGAATCAAAA
M00216	V$TATA_C	0.77502	1316	(+)	NCTATAAAAR	CTTGTAAAAC
M00216	V$TATA_C	0.879852	1345	(+)	NCTATAAAAR	TCTATACAAG

M0025		0.80106				
2	V$TATA_01	6	1346	(+)	STATAAAWRNNNNNN	CTATACAAGTAGGTC
M0021		0.79746				
6	V$TATA_C	5	1359	(+)	NCTATAAAAR	TCAATAACAG
M0021		0.74465				
6	V$TATA_C	3	1422	(+)	NCTATAAAAR	AATATTAAAA
M0021		0.81383				
6	V$TATA_C	7	1423	(+)	NCTATAAAAR	ATATTAAAAG
M0025		0.83024				
2	V$TATA_01	6	1423	(+)	STATAAAWRNNNNNN	ATATTAAAAGCTTAA
M0021		0.75442				
6	V$TATA_C	3	1431	(+)	NCTATAAAAR	AGCTTAAACT
M0021		0.77660				
6	V$TATA_C	4	1451	(+)	NCTATAAAAR	TCTTTCTAAA
M0021		0.80195				
6	V$TATA_C	4	1453	(+)	NCTATAAAAR	TTTCTAAAAA
M0021		0.75204				
6	V$TATA_C	6	1454	(+)	NCTATAAAAR	TTCTAAAAAG
M0021		0.74465				
6	V$TATA_C	3	1455	(+)	NCTATAAAAR	TCTAAAAAGA
M0021		0.76102				
6	V$TATA_C	5	1467	(+)	NCTATAAAAR	ATTTTTAAAA
M0021		0.89015				
6	V$TATA_C	1	1468	(+)	NCTATAAAAR	TTTTTAAAAA
M0021		0.76815				
6	V$TATA_C	4	1469	(+)	NCTATAAAAR	TTTTAAAAAA
M0025		0.80411				
2	V$TATA_01	1	1469	(+)	STATAAAWRNNNNNN	TTTTAAAAAATCAAA
M0021						
6	V$TATA_C	0.77634	1470	(+)	NCTATAAAAR	TTTAAAAAAT
M0021		0.84235				
6	V$TATA_C	5	1507	(+)	NCTATAAAAR	CCTTTAGAAA
M0021		0.84103				
6	V$TATA_C	5	1534	(+)	NCTATAAAAR	TCTTTAAATA
M0025		0.81070				
2	V$TATA_01	8	1535	(+)	STATAAAWRNNNNNN	CTTTAAATATTTCAG
M0021		0.79376				
6	V$TATA_C	8	1580	(+)	NCTATAAAAR	TTTATTAAAA
M0021		0.78320				
6	V$TATA_C	6	1581	(+)	NCTATAAAAR	TTATTAAAAC
M0025		0.81882				
2	V$TATA_01	8	1581	(+)	STATAAAWRNNNNNN	TTATTAAAACAATCA
M0021		0.76287				
6	V$TATA_C	3	1712	(+)	NCTATAAAAR	ATTTTAGAAA
M0025						
2	V$TATA_01	0.78102	1737	(+)	STATAAAWRNNNNNN	TCATTTATAGGTAAG
M0021		0.77422				
6	V$TATA_C	8	1762	(+)	NCTATAAAAR	CCCAGATAAA
M0021		0.80829				
6	V$TATA_C	2	1889	(+)	NCTATAAAAR	TGTTTACAAA
M0025		0.78051				
2	V$TATA_01	3	1890	(+)	STATAAAWRNNNNNN	GTTTACAAAGCCTTG
M0021		0.77475				
6	V$TATA_C	6	1983	(+)	NCTATAAAAR	TCCATTTAAA
M0021						
6	V$TATA_C	0.74254	1984	(+)	NCTATAAAAR	CCATTTAAAT
M0025		0.80182				
2	V$TATA_01	7	1984	(+)	STATAAAWRNNNNNN	CCATTTAAATGCCCA
M0025		0.89063				
2	V$TATA_01	7	1986	(+)	STATAAAWRNNNNNN	ATTTAAATGCCCATG
M0025		0.81502				
2	V$TATA_01	2	2038	(+)	STATAAAWRNNNNNN	CTATTAATCTCTGCA
M0025		0.86678				
2	V$TATA_01	5	2114	(+)	STATAAAWRNNNNNN	CTATATGTAGAAAGC
M0021		0.76366				
6	V$TATA_C	5	2157	(+)	NCTATAAAAR	CCTCTAACAG

M00216	V$TATA_C	0.758648	2233	(+)	NCTATAAAAR	CCTTGATAAT
M00216	V$TATA_C	0.776604	2266	(+)	NCTATAAAAR	TCTTTATATA
M00252	V$TATA_01	0.834306	2267	(+)	STATAAAWRNNNNNN	CTTTATATATATAAG
M00252	V$TATA_01	0.849277	2269	(+)	STATAAAWRNNNNNN	TTATATATATAAGCT
M00252	V$TATA_01	0.860442	2271	(+)	STATAAAWRNNNNNN	ATATATATAAGCTAA
M00216	V$TATA_C	0.820702	2272	(+)	NCTATAAAAR	TATATATAAG
M00252	V$TATA_01	0.877442	2273	(+)	STATAAAWRNNNNNN	ATATATAAGCTAAAA
M00252	V$TATA_01	0.833291	2291	(+)	STATAAAWRNNNNNN	TTATTTATAGGTAAG
M00216	V$TATA_C	0.877476	2313	(+)	NCTATAAAAR	CCTATAAATG
M00252	V$TATA_01	0.914489	2314	(+)	STATAAAWRNNNNNN	CTATAAATGTACATA
M00252	V$TATA_01	0.771124	2322	(+)	STATAAAWRNNNNNN	GTACATATATATTTA
M00252	V$TATA_01	0.788632	2324	(+)	STATAAAWRNNNNNN	ACATATATATTTATA
M00252	V$TATA_01	0.856889	2326	(+)	STATAAAWRNNNNNN	ATATATATTTATAGG
M00252	V$TATA_01	0.785334	2328	(+)	STATAAAWRNNNNNN	ATATATTTATAGGCA
M00252	V$TATA_01	0.871099	2330	(+)	STATAAAWRNNNNNN	ATATTTATAGGCACC
M00252	V$TATA_01	0.799543	2332	(+)	STATAAAWRNNNNNN	ATTTATAGGCACCAT
M00252	V$TATA_01	0.873382	2361	(+)	STATAAAWRNNNNNN	TTATATATGTGTATG
M00252	V$TATA_01	0.783557	2363	(+)	STATAAAWRNNNNNN	ATATATGTGTATGTA
M00252	V$TATA_01	0.792185	2371	(+)	STATAAAWRNNNNNN	GTATGTATATGTGTA
M00252	V$TATA_01	0.828216	2375	(+)	STATAAAWRNNNNNN	GTATATGTGTATACA
M00252	V$TATA_01	0.847754	2383	(+)	STATAAAWRNNNNNN	GTATACATAGGGACA
M00252	V$TATA_01	0.796498	2385	(+)	STATAAAWRNNNNNN	ATACATAGGGACACA
M00216	V$TATA_C	0.781885	2414	(+)	NCTATAAAAR	AGTTTAAAGT
M00216	V$TATA_C	0.756007	2445	(+)	NCTATAAAAR	TCTACAACAG
M00216	V$TATA_C	0.848957	2453	(+)	NCTATAAAAR	AGAATAAAAT
M00252	V$TATA_01	0.792438	2454	(+)	STATAAAWRNNNNNN	GAATAAAATTCTCAT
M00252	V$TATA_01	0.79117	2478	(+)	STATAAAWRNNNNNN	TTATTTATAAAAAAT
M00216	V$TATA_C	0.783734	2479	(+)	NCTATAAAAR	TATTTATAAA
M00252	V$TATA_01	0.810454	2480	(+)	STATAAAWRNNNNNN	ATTTATAAAAAATTG
M00216	V$TATA_C	0.915764	2481	(+)	NCTATAAAAR	TTTATAAAAA
M00252	V$TATA_01	0.851053	2482	(+)	STATAAAWRNNNNNN	TTATAAAAAATTGTG
M00216	V$TATA_C	0.749142	2507	(+)	NCTATAAAAR	TCCTTATTAA
M00252	V$TATA_01	0.829485	2510	(+)	STATAAAWRNNNNNN	TTATTAATGGCCTAC

M0025		0.79091				
2	V$TATA_01	6	2551	(+)	STATAAAWRNNNNNN	GAATATATGATTAAC
M0025		0.77010				
2	V$TATA_01	9	2553	(+)	STATAAAWRNNNNNN	ATATATGATTAACCT
M0021		0.75019				
6	V$TATA_C	8	2578	(+)	NCTATAAAAR	CTTTTAATAT
M0025		0.77416				
2	V$TATA_01	9	2584	(+)	STATAAAWRNNNNNN	ATATTATAGCCAAAA
M0021		0.76815				
6	V$TATA_C	4	2639	(+)	NCTATAAAAR	TTTTTAATAA
M0021		0.81779				
6	V$TATA_C	8	2642	(+)	NCTATAAAAR	TTAATAAAAT
M0025		0.83354				
2	V$TATA_01	5	2668	(+)	STATAAAWRNNNNNN	CTATATCTACCCATA
M0021		0.81225				
6	V$TATA_C	2	2677	(+)	NCTATAAAAR	CCCATAAATC
M0025		0.83532				
2	V$TATA_01	1	2678	(+)	STATAAAWRNNNNNN	CCATAAATCTAGTGG
M0021		0.78716				
6	V$TATA_C	7	2699	(+)	NCTATAAAAR	TCCTTAAAGC
M0021		0.83205				
6	V$TATA_C	7	2887	(+)	NCTATAAAAR	TGTATTAAAA
M0021		0.77132				
6	V$TATA_C	3	2888	(+)	NCTATAAAAR	GTATTAAAAC
M0025		0.81933				
2	V$TATA_01	5	2888	(+)	STATAAAWRNNNNNN	GTATTAAAACTTTAA
M0021		0.83390				
6	V$TATA_C	5	2896	(+)	NCTATAAAAR	ACTTTAAGAA
M0025		0.82948				
2	V$TATA_01	5	2910	(+)	STATAAAWRNNNNNN	TTATTTAAAGGCTCT
M0025		0.77670				
2	V$TATA_01	6	2912	(+)	STATAAAWRNNNNNN	ATTTAAAGGCTCTAA
M0025		0.79345				
2	V$TATA_01	3	2947	(+)	STATAAAWRNNNNNN	TTATATTTATAGAGC
M0025		0.82872				
2	V$TATA_01	4	2949	(+)	STATAAAWRNNNNNN	ATATTTATAGAGCAA
M0025		0.81806				
2	V$TATA_01	6	2951	(+)	STATAAAWRNNNNNN	ATTTATAGAGCAAAA
M0025		0.77188				
2	V$TATA_01	5	2980	(+)	STATAAAWRNNNNNN	CTATATTTTCATAAA
M0021		0.89965				
6	V$TATA_C	7	2987	(+)	NCTATAAAAR	TTCATAAAAG
M0025		0.81578				
2	V$TATA_01	3	2988	(+)	STATAAAWRNNNNNN	TCATAAAAGTAACTC
M0021		0.74465				
6	V$TATA_C	3	3018	(+)	NCTATAAAAR	TCTATTACAA
M0021						
6	V$TATA_C	0.76921	3021	(+)	NCTATAAAAR	ATTACAAAAT
M0021						
6	V$TATA_C	0.85239	3028	(+)	NCTATAAAAR	AATTTAAAAG
M0025		0.80411				
2	V$TATA_01	1	3029	(+)	STATAAAWRNNNNNN	ATTTAAAAGATGTTC
M0021		0.75204				
6	V$TATA_C	6	3076	(+)	NCTATAAAAR	ACTTTATCAT
M0025		0.77619				
2	V$TATA_01	9	3084	(+)	STATAAAWRNNNNNN	ATGTATAAGAAAATC
M0021		0.83205				
6	V$TATA_C	7	3085	(+)	NCTATAAAAR	TGTATAAGAA
M0025		0.82999				
2	V$TATA_01	2	3086	(+)	STATAAAWRNNNNNN	GTATAAGAAAATCTT
M0021		0.94560				
6	V$TATA_C	3	3097	(+)	NCTATAAAAR	TCTTTAAAAT
M0025		0.80487				
2	V$TATA_01	2	3098	(+)	STATAAAWRNNNNNN	CTTTAAAATTCTCAT
M0021						
6	V$TATA_C	0.76921	3165	(+)	NCTATAAAAR	ATTATAATAT

M00216	V$TATA_C	0.862952	3175	(+)	NCTATAAAAR	GCTCTAAAAA
M00216	V$TATA_C	0.740164	3176	(+)	NCTATAAAAR	CTCTAAAAAA
M00216	V$TATA_C	0.849221	3177	(+)	NCTATAAAAR	TCTAAAAAAT
M00216	V$TATA_C	0.766306	3189	(+)	NCTATAAAAR	TTCACAAAAA
M00252	V$TATA_01	0.825679	3197	(+)	STATAAAWRNNNNNN	AAATAAATGCAAAAA
M00216	V$TATA_C	0.789015	3232	(+)	NCTATAAAAR	TGTTTTAAAT
M00216	V$TATA_C	0.874043	3286	(+)	NCTATAAAAR	TTCTTAAAAG
M00216	V$TATA_C	0.810668	3324	(+)	NCTATAAAAR	AGTTTCAAAG
M00216	V$TATA_C	0.768154	3458	(+)	NCTATAAAAR	TTTTTTAAAA
M00216	V$TATA_C	0.901505	3459	(+)	NCTATAAAAR	TTTTTAAAAG
M00252	V$TATA_01	0.777975	3460	(+)	STATAAAWRNNNNNN	TTTTAAAAGTTACAT
M00216	V$TATA_C	0.843412	3479	(+)	NCTATAAAAR	TGTATTAAAG
M00252	V$TATA_01	0.840142	3480	(+)	STATAAAWRNNNNNN	GTATTAAAGCTATCT
M00216	V$TATA_C	0.764193	3492	(+)	NCTATAAAAR	TCTGAAAAAG
M00216	V$TATA_C	0.850277	3669	(+)	NCTATAAAAR	ACTGTAAAAT
M00216	V$TATA_C	0.76921	3715	(+)	NCTATAAAAR	ATTATTAAAT
M00216	V$TATA_C	0.784262	3751	(+)	NCTATAAAAR	CCTAAATAAT
M00216	V$TATA_C	0.833905	3787	(+)	NCTATAAAAR	ACTTTTAAAA
M00216	V$TATA_C	0.889622	3788	(+)	NCTATAAAAR	CTTTTAAAAA
M00216	V$TATA_C	0.768154	3789	(+)	NCTATAAAAR	TTTTAAAAAA
M00252	V$TATA_01	0.776453	3789	(+)	STATAAAWRNNNNNN	TTTTAAAAAATTTTG
M00216	V$TATA_C	0.77634	3790	(+)	NCTATAAAAR	TTTAAAAAAT
M00216	V$TATA_C	0.823607	3842	(+)	NCTATAAAAR	TCTTTGAAAT
M00216	V$TATA_C	0.772379	3876	(+)	NCTATAAAAR	ATTTTGAAAG
M00252	V$TATA_01	0.78102	4044	(+)	STATAAAWRNNNNNN	AAATAAATCAAAGGA
M00216	V$TATA_C	0.755743	4059	(+)	NCTATAAAAR	AGAATCAAAG
M00252	V$TATA_01	0.780259	4112	(+)	STATAAAWRNNNNNN	ATACAAATTGAAAAA
M00216	V$TATA_C	0.744125	4191	(+)	NCTATAAAAR	CCTATAATGA
M00252	V$TATA_01	0.796498	4192	(+)	STATAAAWRNNNNNN	CTATAATGAAAAAGA
M00216	V$TATA_C	0.744653	4206	(+)	NCTATAAAAR	AATATGAAAA
M00252	V$TATA_01	0.833037	4207	(+)	STATAAAWRNNNNNN	ATATGAAAACGAATA
M00216	V$TATA_C	0.767626	4224	(+)	NCTATAAAAR	TGTATATATA
M00252	V$TATA_01	0.935296	4225	(+)	STATAAAWRNNNNNN	GTATATATATGCATG

M00252	V$TATA_01	0.874651	4227	(+)	STATAAAWRNNNNNN	ATATATATGCATGAC
M00252	V$TATA_01	0.777214	4298	(+)	STATAAAWRNNNNNN	CAATTTAAAAAAAGA
M00216	V$TATA_C	0.841035	4299	(+)	NCTATAAAAR	AATTTAAAAA
M00216	V$TATA_C	0.761025	4300	(+)	NCTATAAAAR	ATTTAAAAAA
M00252	V$TATA_01	0.843948	4300	(+)	STATAAAWRNNNNNN	ATTTAAAAAAAGAAA
M00216	V$TATA_C	0.793768	4301	(+)	NCTATAAAAR	TTTAAAAAAA
M00252	V$TATA_01	0.779751	4302	(+)	STATAAAWRNNNNNN	TTAAAAAAAGAAAAA
M00216	V$TATA_C	0.755743	4310	(+)	NCTATAAAAR	AGAAAAAAAG
M00216	V$TATA_C	0.76921	4326	(+)	NCTATAAAAR	ATTATTAAAT
M00216	V$TATA_C	0.740692	4335	(+)	NCTATAAAAR	TTCTTCAAAA
M00216	V$TATA_C	0.841035	4336	(+)	NCTATAAAAR	TCTTCAAAAA
M00216	V$TATA_C	0.766306	4338	(+)	NCTATAAAAR	TTCAAAAAAA
M00216	V$TATA_C	0.78611	4339	(+)	NCTATAAAAR	TCAAAAAAAA
M00216	V$TATA_C	0.78611	4399	(+)	NCTATAAAAR	AAAATAAAAA
M00252	V$TATA_01	0.799797	4400	(+)	STATAAAWRNNNNNN	AAATAAAAATAGTTA
M00252	V$TATA_01	0.772393	4402	(+)	STATAAAWRNNNNNN	ATAAAAATAGTTAAG
M00252	V$TATA_01	0.801827	4420	(+)	STATAAAWRNNNNNN	ATATTTATTACTAGC
M00252	V$TATA_01	0.854352	4475	(+)	STATAAAWRNNNNNN	CTATAAGTTGGCACA
M00216	V$TATA_C	0.799313	4505	(+)	NCTATAAAAR	AGTTTAATAA
M00216	V$TATA_C	0.744653	4517	(+)	NCTATAAAAR	TCTACAAAGA
M00216	V$TATA_C	0.767626	4550	(+)	NCTATAAAAR	CTTTTGAAAA
M00216	V$TATA_C	0.768154	4551	(+)	NCTATAAAAR	TTTTGAAAAA
M00216	V$TATA_C	0.78611	4574	(+)	NCTATAAAAR	TCAATGAAAA
M00216	V$TATA_C	0.795088	4583	(+)	NCTATAAAAR	ACTATTTAAA
M00252	V$TATA_01	0.856128	4584	(+)	STATAAAWRNNNNNN	CTATTTAAAAGGCCA
M00216	V$TATA_C	0.859519	4585	(+)	NCTATAAAAR	TATTTAAAAG
M00252	V$TATA_01	0.892413	4586	(+)	STATAAAWRNNNNNN	ATTTAAAAGGCCACT
M00216	V$TATA_C	0.802218	4624	(+)	NCTATAAAAR	TCTATATACA
M00252	V$TATA_01	0.778483	4625	(+)	STATAAAWRNNNNNN	CTATATACAATAAAA
M00216	V$TATA_C	0.883549	4631	(+)	NCTATAAAAR	ACAATAAAAT
M00216	V$TATA_C	0.768154	4644	(+)	NCTATAAAAR	TTTTTGAAAA
M00216	V$TATA_C	0.768154	4645	(+)	NCTATAAAAR	TTTTGAAAAA
M00216	V$TATA_C	0.750726	4712	(+)	NCTATAAAAR	TTTTTAAACT

M00216	V$TATA_C	0.870874	4719	(+)	NCTATAAAAR	ACTATAAATG
M00252	V$TATA_01	0.917026	4720	(+)	STATAAAWRNNNNNN	CTATAAATGTGTAAG
M00216	V$TATA_C	0.824927	4733	(+)	NCTATAAAAR	AGTATGAAAA
M00252	V$TATA_01	0.810454	4734	(+)	STATAAAWRNNNNNN	GTATGAAAACTATGA
M00216	V$TATA_C	0.740692	4780	(+)	NCTATAAAAR	TTCTTGAAAA
M00216	V$TATA_C	0.841035	4781	(+)	NCTATAAAAR	TCTTGAAAAA
M00216	V$TATA_C	0.751518	4784	(+)	NCTATAAAAR	TGAAAAAAAA
M00216	V$TATA_C	0.79324	4838	(+)	NCTATAAAAR	TACATATAAG
M00252	V$TATA_01	0.806902	4839	(+)	STATAAAWRNNNNNN	ACATATAAGTTAATA
M00252	V$TATA_01	0.774423	4841	(+)	STATAAAWRNNNNNN	ATATAAGTTAATATT
M00252	V$TATA_01	0.780766	4854	(+)	STATAAAWRNNNNNN	TTATATTAAGATGTC
M00252	V$TATA_01	0.784319	4856	(+)	STATAAAWRNNNNNN	ATATTAAGATGTCTA
M00216	V$TATA_C	0.769475	4920	(+)	NCTATAAAAR	ACTTTTTAAA
M00216	V$TATA_C	0.750198	4921	(+)	NCTATAAAAR	CTTTTTAAAT
M00216	V$TATA_C	0.750726	4922	(+)	NCTATAAAAR	TTTTTAAATT
M00216	V$TATA_C	0.761025	4929	(+)	NCTATAAAAR	ATTTTAAACA
M00252	V$TATA_01	0.78102	4946	(+)	STATAAAWRNNNNNN	TAATAAATTTAGAGA
M00216	V$TATA_C	0.750726	5002	(+)	NCTATAAAAR	TTTTTCAAAT
M00216	V$TATA_C	0.753367	5025	(+)	NCTATAAAAR	ACATTCAAAA
M00252	V$TATA_01	0.7932	5048	(+)	STATAAAWRNNNNNN	TTTTATATCCAAGGA
M00252	V$TATA_01	0.770363	5061	(+)	STATAAAWRNNNNNN	GAATATGTGTGCAAT
M00216	V$TATA_C	0.865593	5095	(+)	NCTATAAAAR	ATTTTAAAAT
M00252	V$TATA_01	0.807409	5103	(+)	STATAAAWRNNNNNN	ATATTTAAAATTATG
M00216	V$TATA_C	0.830737	5104	(+)	NCTATAAAAR	TATTTAAAAT
M00252	V$TATA_01	0.780513	5105	(+)	STATAAAWRNNNNNN	ATTTAAAATTATGAC
M00216	V$TATA_C	0.856879	5131	(+)	NCTATAAAAR	AGTTTATAAA
M00252	V$TATA_01	0.825933	5132	(+)	STATAAAWRNNNNNN	GTTTATAAAACTTTT
M00216	V$TATA_C	0.889358	5133	(+)	NCTATAAAAR	TTTATAAAAC
M00252	V$TATA_01	0.818828	5134	(+)	STATAAAWRNNNNNN	TTATAAAACTTTTGG
M00216	V$TATA_C	0.810668	5150	(+)	NCTATAAAAR	AGTTTTAAAG
M00252	V$TATA_01	0.823395	5160	(+)	STATAAAWRNNNNNN	TTTTATATATACATG
M00252	V$TATA_01	0.869323	5162	(+)	STATAAAWRNNNNNN	TTATATATACATGCC
M00252	V$TATA_01	0.7932	5164	(+)	STATAAAWRNNNNNN	ATATATACATGCCTA

M00252	V$TATA_01	0.811469	5166	(+)	STATAAAWRNNNNNN	ATATACATGCCTATG
M00216	V$TATA_C	0.745973	5175	(+)	NCTATAAAAR	CCTATGGAAA
M00216	V$TATA_C	0.981516	5240	(+)	NCTATAAAAR	ACTATAAAAA
M00252	V$TATA_01	0.951789	5241	(+)	STATAAAWRNNNNNN	CTATAAAAACAAATG
M00216	V$TATA_C	0.761025	5263	(+)	NCTATAAAAR	ATTTTTAAAA
M00216	V$TATA_C	0.890151	5264	(+)	NCTATAAAAR	TTTTTAAAAA
M00252	V$TATA_01	0.78305	5264	(+)	STATAAAWRNNNNNN	TTTTTAAAAAGCATA
M00216	V$TATA_C	0.779509	5265	(+)	NCTATAAAAR	TTTTAAAAAG
M00252	V$TATA_01	0.823649	5265	(+)	STATAAAWRNNNNNN	TTTTAAAAAGCATAA
M00216	V$TATA_C	0.882229	5279	(+)	NCTATAAAAR	ATTATAAAAC
M00252	V$TATA_01	0.785334	5280	(+)	STATAAAWRNNNNNN	TTATAAAACATTTAT
M00252	V$TATA_01	0.855113	5289	(+)	STATAAAWRNNNNNN	ATTTATATATCGATG
M00252	V$TATA_01	0.830246	5291	(+)	STATAAAWRNNNNNN	TTATATATCGATGTT
M00216	V$TATA_C	0.839715	5328	(+)	NCTATAAAAR	CCTAGAAAAC
M00216	V$TATA_C	0.833905	5336	(+)	NCTATAAAAR	ACTTCAAAAA
M00216	V$TATA_C	0.744917	5348	(+)	NCTATAAAAR	ATCTTAATAG
M00216	V$TATA_C	0.768154	5391	(+)	NCTATAAAAR	TTTTTAACAA
M00216	V$TATA_C	0.787959	5408	(+)	NCTATAAAAR	TCAATACAAA
M00216	V$TATA_C	0.820438	5419	(+)	NCTATAAAAR	TGAATATAAG
M00252	V$TATA_01	0.784319	5420	(+)	STATAAAWRNNNNNN	GAATATAAGTTATTC
M00252	V$TATA_01	0.775945	5442	(+)	STATAAAWRNNNNNN	TCATTTAAGCAAATA
M00252	V$TATA_01	0.788886	5463	(+)	STATAAAWRNNNNNN	GTATTTGTTGGCACA
M00216	V$TATA_C	0.761025	5531	(+)	NCTATAAAAR	ATTTTTAAAA
M00216	V$TATA_C	0.901505	5532	(+)	NCTATAAAAR	TTTTTAAAAG
M00252	V$TATA_01	0.795991	5532	(+)	STATAAAWRNNNNNN	TTTTTAAAAGACAAC
M00252	V$TATA_01	0.840903	5533	(+)	STATAAAWRNNNNNN	TTTTAAAAGACAACT
M00216	V$TATA_C	0.86929	5642	(+)	NCTATAAAAR	GCTTTATAAT
M00216	V$TATA_C	0.77634	5644	(+)	NCTATAAAAR	TTTATAATAT
M00216	V$TATA_C	0.745445	5652	(+)	NCTATAAAAR	ATTTTACAAT
M00216	V$TATA_C	0.78611	5665	(+)	NCTATAAAAR	AAAATAAAAA
M00252	V$TATA_01	0.85359	5666	(+)	STATAAAWRNNNNNN	AAATAAAAACAAAGA
M00216	V$TATA_C	0.823607	5693	(+)	NCTATAAAAR	TCTTTAATAT
M00216	V$TATA_C	0.770795	5696	(+)	NCTATAAAAR	TTAATATAAA

M00252	V$TATA_01	0.777214	5697	(+)	STATAAAWRNNNNNN	TAATATAAAAGGTAT
M00216	V$TATA_C	0.878004	5698	(+)	NCTATAAAAR	AATATAAAAG
M00252	V$TATA_01	0.874905	5699	(+)	STATAAAWRNNNNNN	ATATAAAAGGTATCT
M00216	V$TATA_C	0.936625	5711	(+)	NCTATAAAAR	TCTTTAAAAC
M00252	V$TATA_01	0.85562	5712	(+)	STATAAAWRNNNNNN	CTTTAAAACCACAAA
M00216	V$TATA_C	0.832057	5719	(+)	NCTATAAAAR	ACCACAAAAA
M00216	V$TATA_C	0.778981	5722	(+)	NCTATAAAAR	ACAAAAAAAA
M00216	V$TATA_C	0.785054	5896	(+)	NCTATAAAAR	ATATTAAAAT
M00252	V$TATA_01	0.842172	5896	(+)	STATAAAWRNNNNNN	ATATTAAAATGCCTT
M00216	V$TATA_C	0.840243	5912	(+)	NCTATAAAAR	TGTCTAAAAA
M00252	V$TATA_01	0.802842	5913	(+)	STATAAAWRNNNNNN	GTCTAAAAACTAAGT
M00216	V$TATA_C	0.760496	5925	(+)	NCTATAAAAR	AGTATTTAAA
M00252	V$TATA_01	0.875412	5926	(+)	STATAAAWRNNNNNN	GTATTTAAACCCCAT
M00216	V$TATA_C	0.943227	5936	(+)	NCTATAAAAR	CCCATAAAAT
M00252	V$TATA_01	0.802334	5937	(+)	STATAAAWRNNNNNN	CCATAAAATAATCTA
M00216	V$TATA_C	0.851598	5958	(+)	NCTATAAAAR	TGTGTAAAAG
M00252	V$TATA_01	0.800304	5959	(+)	STATAAAWRNNNNNN	GTGTAAAAGTCATCA
M00216	V$TATA_C	0.801426	6009	(+)	NCTATAAAAR	CTTCTAAAAA
M00252	V$TATA_01	0.783557	6066	(+)	STATAAAWRNNNNNN	GTATTTAGCAATAAG
M00216	V$TATA_C	0.785582	6073	(+)	NCTATAAAAR	GCAATAAGAG
M00252	V$TATA_01	0.775945	6074	(+)	STATAAAWRNNNNNN	CAATAAGAGAAAACT
M00216	V$TATA_C	0.745709	6104	(+)	NCTATAAAAR	ACTGGAAAAA
M00216	V$TATA_C	0.772907	6131	(+)	NCTATAAAAR	AGTTTAATAC
M00216	V$TATA_C	0.780829	6152	(+)	NCTATAAAAR	ACTTTTTAAG
M00252	V$TATA_01	0.788886	6153	(+)	STATAAAWRNNNNNN	CTTTTTAAGACTTGG
M00216	V$TATA_C	0.741748	6154	(+)	NCTATAAAAR	TTTTTAAGAC
M00252	V$TATA_01	0.77696	6248	(+)	STATAAAWRNNNNNN	ATACATAAATATGTC
M00252	V$TATA_01	0.82035	6250	(+)	STATAAAWRNNNNNN	ACATAAATATGTCTT
M00216	V$TATA_C	0.792712	6274	(+)	NCTATAAAAR	GAAATAAAAG
M00252	V$TATA_01	0.814768	6275	(+)	STATAAAWRNNNNNN	AAATAAAAGTGTAAT
M00252	V$TATA_01	0.849277	6323	(+)	STATAAAWRNNNNNN	CTATTTATATGGAAA
M00252	V$TATA_01	0.852068	6325	(+)	STATAAAWRNNNNNN	ATTTATATGGAAAGA
M00252	V$TATA_01	0.783811	6327	(+)	STATAAAWRNNNNNN	TTATATGGAAAGACC

M0021		0.80829				
6	V$TATA_C	2	6352	(+)	NCTATAAAAR	TTTTTATAAT
M0025		0.79979				
2	V$TATA_01	7	6353	(+)	STATAAAWRNNNNNN	TTTTATAATTGCACA
M0025		0.80969				
2	V$TATA_01	3	6355	(+)	STATAAAWRNNNNNN	TTATAATTGCACATA
M0021		0.77053				
6	V$TATA_C	1	6371	(+)	NCTATAAAAR	TCAATAGAAT
M0021		0.79271				
6	V$TATA_C	2	6386	(+)	NCTATAAAAR	CAAATAAAAA
M0025		0.82364				
2	V$TATA_01	9	6387	(+)	STATAAAWRNNNNNN	AAATAAAAATAGAAA
M0025		0.78736				
2	V$TATA_01	4	6389	(+)	STATAAAWRNNNNNN	ATAAAAATAGAAAAT
M0025		0.77112				
2	V$TATA_01	4	6414	(+)	STATAAAWRNNNNNN	ATATTTAACAGATAA
M0021		0.74227				
6	V$TATA_C	6	6422	(+)	NCTATAAAAR	CAGATAAAAT
M0025		0.78381				
2	V$TATA_01	1	6423	(+)	STATAAAWRNNNNNN	AGATAAAATTAAGCT
M0021		0.77185				
6	V$TATA_C	1	6434	(+)	NCTATAAAAR	AGCTGAAAAA
M0021		0.74095				
6	V$TATA_C	6	6435	(+)	NCTATAAAAR	GCTGAAAAAA
M0025		0.81984				
2	V$TATA_01	3	6498	(+)	STATAAAWRNNNNNN	ATATAATTAGGTTGG
M0021		0.75574				
6	V$TATA_C	3	6543	(+)	NCTATAAAAR	GGTAAATAAA
M0021		0.77581				
6	V$TATA_C	2	6545	(+)	NCTATAAAAR	TAAATAAAAT
M0025		0.77416				
2	V$TATA_01	9	6584	(+)	STATAAAWRNNNNNN	ATACATATATATATA
M0025		0.81705				
2	V$TATA_01	2	6586	(+)	STATAAAWRNNNNNN	ACATATATATATACA
M0025		0.88226				
2	V$TATA_01	3	6588	(+)	STATAAAWRNNNNNN	ATATATATATACATA
M0025						
2	V$TATA_01	0.88607	6590	(+)	STATAAAWRNNNNNN	ATATATATACATATA
M0025		0.77213				
2	V$TATA_01	9	6592	(+)	STATAAAWRNNNNNN	ATATATACATATAAA
M0025		0.79066				
2	V$TATA_01	2	6596	(+)	STATAAAWRNNNNNN	ATACATATAAATATG
M0021		0.78188				
6	V$TATA_C	5	6597	(+)	NCTATAAAAR	TACATATAAA
M0025		0.80258				
2	V$TATA_01	8	6598	(+)	STATAAAWRNNNNNN	ACATATAAATATGAA
M0021		0.75125				
6	V$TATA_C	4	6599	(+)	NCTATAAAAR	CATATAAATA
M0025		0.89089				
2	V$TATA_01	1	6600	(+)	STATAAAWRNNNNNN	ATATAAATATGAAAA
M0021		0.74465				
6	V$TATA_C	3	6605	(+)	NCTATAAAAR	AATATGAAAA
M0025		0.80614				
2	V$TATA_01	1	6606	(+)	STATAAAWRNNNNNN	ATATGAAAAAATATG
M0025		0.77569				
2	V$TATA_01	1	6726	(+)	STATAAAWRNNNNNN	CTTTTTATATGTGCC
M0025						
2	V$TATA_01	0.79929	6728	(+)	STATAAAWRNNNNNN	TTTTATATGTGCCAA
M0025		0.84394				
2	V$TATA_01	8	6730	(+)	STATAAAWRNNNNNN	TTATATGTGCCAAGC
M0005		0.84993				
9	V$YY1_01	4	18	(-)	NNNNNCCATNTWNNNWN	CTTTATAAATGACAACT
M0005		0.81159				
9	V$YY1_01	9	112	(+)	NNNNNCCATNTWNNNWN	CATCTTCCTATTGGGTG
M0005		0.81913				
9	V$YY1_01	5	167	(+)	NNNNNCCATNTWNNNWN	TAACACCCTTTAATTAG

M00059	V$YY1_01	0.842071	182	(+)	NNNNNCCATNTWNNNWN	AGTCTCCAGTTATTTAC
M00059	V$YY1_01	0.775557	284	(+)	NNNNNCCATNTWNNNWN	AATGTCCAGAGAGAATG
M00059	V$YY1_01	0.774574	355	(-)	NNNNNCCATNTWNNNWN	GCTTGAATATGCCTTGG
M00059	V$YY1_01	0.870904	369	(+)	NNNNNCCATNTWNNNWN	TGGCATCATTTTGACAT
M00059	V$YY1_01	0.770315	540	(+)	NNNNNCCATNTWNNNWN	TACAGACATATGTGGAG
M00059	V$YY1_01	0.77654	684	(+)	NNNNNCCATNTWNNNWN	GAGTTACATATCCTCAC
M00059	V$YY1_01	0.820773	767	(-)	NNNNNCCATNTWNNNWN	AGTAAATTATGGTCTTA
M00059	V$YY1_01	0.783421	820	(+)	NNNNNCCATNTWNNNWN	TACTGTCATTTTACAGA
M00059	V$YY1_01	0.779817	855	(-)	NNNNNCCATNTWNNNWN	GTGTAAATCTGTATATG
M00059	V$YY1_01	0.779489	932	(-)	NNNNNCCATNTWNNNWN	AAAAAGAAATGATAATT
M00059	V$YY1_01	0.777195	975	(-)	NNNNNCCATNTWNNNWN	TCACTACAATGGCAATC
M00059	V$YY1_01	0.849279	1072	(-)	NNNNNCCATNTWNNNWN	GTTAAAAAATGAAAAAA
M00059	V$YY1_01	0.82536	1117	(+)	NNNNNCCATNTWNNNWN	GAACTCCATTCTTTTCA
M00059	V$YY1_01	0.783093	1204	(+)	NNNNNCCATNTWNNNWN	TGAAATCAGTTTAGTAG
M00059	V$YY1_01	0.808978	1253	(-)	NNNNNCCATNTWNNNWN	AAAAGCAAATGGAGTCT
M00059	V$YY1_01	0.774902	1260	(-)	NNNNNCCATNTWNNNWN	AATGGAGTCTGGTGTGT
M00059	V$YY1_01	0.775885	1283	(-)	NNNNNCCATNTWNNNWN	TACCAAGCCTGGACTTT
M00059	V$YY1_01	0.781127	1321	(+)	NNNNNCCATNTWNNNWN	AAAACTCAGTTTGCAAG
M00059	V$YY1_01	0.781782	1460	(+)	NNNNNCCATNTWNNNWN	AAAGACTATTTTTAAAA
M00059	V$YY1_01	0.839777	1602	(+)	NNNNNCCATNTWNNNWN	TCTCATCATTCATGATG
M00059	V$YY1_01	0.777523	1605	(-)	NNNNNCCATNTWNNNWN	CATCATTCATGATGACT
M00069	V$YY1_02	0.78115	1606	(-)	NNNCGGCCATCTTGNCTSNW	ATCATTCATGATGACTGGAG
M00069	V$YY1_02	0.774902	1664	(+)	NNNCGGCCATCTTGNCTSNW	TCTCCTCCATCTTACATGTT
M00059	V$YY1_01	0.820773	1665	(+)	NNNNNCCATNTWNNNWN	CTCCTCCATCTTACATG
M00059	V$YY1_01	0.789646	1671	(-)	NNNNNCCATNTWNNNWN	CATCTTACATGTTCCTG
M00059	V$YY1_01	0.843054	1691	(-)	NNNNNCCATNTWNNNWN	GACCAATCATGGAATTA
M00059	V$YY1_01	0.797837	1732	(+)	NNNNNCCATNTWNNNWN	AACTTTCATTTATAGGT
M00059	V$YY1_01	0.796527	1809	(-)	NNNNNCCATNTWNNNWN	AGCCTGAGATGGAACTC
M00059	V$YY1_01	0.806356	1979	(+)	NNNNNCCATNTWNNNWN	TTTCTCCATTTAAATGC
M00059	V$YY1_01	0.772608	2096	(-)	NNNNNCCATNTWNNNWN	TTTCTTAACTGAAAACT
M00069	V$YY1_02	0.754552	2254	(+)	NNNCGGCCATCTTGNCTSNW	ACAGTGCCTTGTTCTTTATA
M00059	V$YY1_01	0.81848	2312	(-)	NNNNNCCATNTWNNNWN	ACCTATAAATGTACATA
M00059	V$YY1_01	0.786042	2319	(+)	NNNNNCCATNTWNNNWN	AATGTACATATATATTT

M00069	V$YY1_02	0.758301	2337	(+)	NNNCGGCCATCTTGNCTSNW	TAGGCACCATTATGTGCCTA
M00059	V$YY1_01	0.801114	2338	(+)	NNNNNCCATNTWNNNWN	AGGCACCATTATGTGCC
M00059	V$YY1_01	0.786042	2359	(-)	NNNNNCCATNTWNNNWN	CATTATATATGTGTATG
M00059	V$YY1_01	0.77097	2371	(-)	NNNNNCCATNTWNNNWN	GTATGTATATGTGTATA
M00059	V$YY1_01	0.771298	2492	(-)	NNNNNCCATNTWNNNWN	TTGTGTCTATGACTTTC
M00059	V$YY1_01	0.848624	2541	(-)	NNNNNCCATNTWNNNWN	CATGTTAAATGAATATA
M00059	V$YY1_01	0.805701	2549	(-)	NNNNNCCATNTWNNNWN	ATGAATATATGATTAAC
M00059	V$YY1_01	0.824377	2800	(-)	NNNNNCCATNTWNNNWN	GTGCAATCATGAAGAGC
M00059	V$YY1_01	0.792923	2868	(+)	NNNNNCCATNTWNNNWN	TAATTGCATCTAATGAA
M00059	V$YY1_01	0.771625	2935	(-)	NNNNNCCATNTWNNNWN	ATTTCTAAATAGTTATA
M00059	V$YY1_01	0.795544	2996	(+)	NNNNNCCATNTWNNNWN	GTAACTCATTTTTACAT
M00059	V$YY1_01	0.825688	3030	(-)	NNNNNCCATNTWNNNWN	TTTAAAAGATGTTCTGG
M00059	V$YY1_01	0.858453	3045	(+)	NNNNNCCATNTWNNNWN	GGACATCAGTTTTCAAG
M00059	V$YY1_01	0.787353	3165	(-)	NNNNNCCATNTWNNNWN	ATTATAATATGCTCTAA
M00059	V$YY1_01	0.811599	3317	(+)	NNNNNCCATNTWNNNWN	TTTTACCAGTTTCAAAG
M00059	V$YY1_01	0.828637	3426	(+)	NNNNNCCATNTWNNNWN	ACTGTCCATAATCAAAT
M00059	V$YY1_01	0.842071	3549	(+)	NNNNNCCATNTWNNNWN	CATTGTCATTTTGGCTA
M00059	V$YY1_01	0.778506	3803	(-)	NNNNNCCATNTWNNNWN	GAACGACTATGACATTC
M00059	V$YY1_01	0.827982	3869	(+)	NNNNNCCATNTWNNNWN	CCTGCACATTTTGAAAG
M00059	V$YY1_01	0.801114	4201	(-)	NNNNNCCATNTWNNNWN	AAAAGAATATGAAAACG
M00059	V$YY1_01	0.771625	4216	(+)	NNNNNCCATNTWNNNWN	CGAATACATGTATATAT
M00059	V$YY1_01	0.780799	4269	(+)	NNNNNCCATNTWNNNWN	TGACACAATATTGTAAA
M00059	V$YY1_01	0.773919	4278	(-)	NNNNNCCATNTWNNNWN	ATTGTAAACTGACTGTA
M00059	V$YY1_01	0.813565	4359	(+)	NNNNNCCATNTWNNNWN	TGATAGCATCTTTTATG
M00059	V$YY1_01	0.783093	4374	(-)	NNNNNCCATNTWNNNWN	TGTGCTAAATGTATTCC
M00059	V$YY1_01	0.810944	4436	(-)	NNNNNCCATNTWNNNWN	ATTATGAGATGAACTGC
M00059	V$YY1_01	0.792267	4601	(+)	NNNNNCCATNTWNNNWN	GATATCCAGATGTTATG
M00059	V$YY1_01	0.775229	4653	(-)	NNNNNCCATNTWNNNWN	AAAGCTTTATGGTTAGC
M00059	V$YY1_01	0.782438	4677	(+)	NNNNNCCATNTWNNNWN	AACAAACATTTCAGGAC
M00059	V$YY1_01	0.79751	4693	(+)	NNNNNCCATNTWNNNWN	CATAATCATTGATCATT
M00059	V$YY1_01	0.770315	4700	(+)	NNNNNCCATNTWNNNWN	ATTGATCATTTATTTTT
M00059	V$YY1_01	0.799148	4728	(-)	NNNNNCCATNTWNNNWN	GTGTAAGTATGAAAACT
M00059	V$YY1_01	0.811927	4769	(+)	NNNNNCCATNTWNNNWN	AGAGATCATGTTTCTTG

M00059	V$YY1_01	0.839777	4805	(-)	NNNNNCCATNTWNNNWN	ATACGAAAATGAATGTA
M00059	V$YY1_01	0.794233	4908	(+)	NNNNNCCATNTWNNNWN	AATATTCATATTACTTT
M00059	V$YY1_01	0.870249	5088	(+)	NNNNNCCATNTWNNNWN	AACTGCCATTTTAAAAT
M00059	V$YY1_01	0.838139	5311	(+)	NNNNNCCATNTWNNNWN	AGGAGCCATTTTTTTAA
M00059	V$YY1_01	0.779489	5337	(-)	NNNNNCCATNTWNNNWN	CTTCAAAAATCATCTTA
M00059	V$YY1_01	0.804391	5341	(+)	NNNNNCCATNTWNNNWN	AAAAATCATCTTAATAG
M00059	V$YY1_01	0.778834	5371	(-)	NNNNNCCATNTWNNNWN	TATTCTCAATGTTCTTC
M00059	V$YY1_01	0.805046	5410	(-)	NNNNNCCATNTWNNNWN	AATACAAACTGAATATA
M00059	V$YY1_01	0.777195	5437	(+)	NNNNNCCATNTWNNNWN	ATTTTTCATTTAAGCAA
M00059	V$YY1_01	0.778178	5677	(-)	NNNNNCCATNTWNNNWN	AAGAAAACATGTTTTGT
M00059	V$YY1_01	0.803735	5678	(+)	NNNNNCCATNTWNNNWN	AGAAAACATGTTTTGTC
M00059	V$YY1_01	0.857798	5840	(-)	NNNNNCCATNTWNNNWN	GTAGAGACATGGACTCC
M00059	V$YY1_01	0.778834	5896	(-)	NNNNNCCATNTWNNNWN	ATATTAAAATGCCTTTT
M00059	V$YY1_01	0.794233	5932	(+)	NNNNNCCATNTWNNNWN	AAACCCCATAAAATAAT
M00059	V$YY1_01	0.810944	5966	(+)	NNNNNCCATNTWNNNWN	AGTCATCAGTTAGCAAT
M00059	V$YY1_01	0.774574	6022	(+)	NNNNNCCATNTWNNNWN	TGGTATCATCTTATAGG
M00059	V$YY1_01	0.816186	6107	(-)	NNNNNCCATNTWNNNWN	GGAAAAAAATGAAAACA
M00059	V$YY1_01	0.843709	6171	(+)	NNNNNCCATNTWNNNWN	AAGATCCATCTCTGAAT
M00059	V$YY1_01	0.785059	6236	(-)	NNNNNCCATNTWNNNWN	CTGTTTGAATGAATACA
M00059	V$YY1_01	0.858453	6323	(-)	NNNNNCCATNTWNNNWN	CTATTTATATGGAAAGA
M00059	V$YY1_01	0.805374	6342	(+)	NNNNNCCATNTWNNNWN	TAATGACATATTTTTAT
M00059	V$YY1_01	0.787025	6371	(-)	NNNNNCCATNTWNNNWN	TCAATAGAATGGAGTCA
M00059	V$YY1_01	0.788008	6565	(-)	NNNNNCCATNTWNNNWN	TTACAAACATGTGGTGA
M00059	V$YY1_01	0.778506	6581	(+)	NNNNNCCATNTWNNNWN	AGAATACATATATATAT
M00059	V$YY1_01	0.783421	6593	(+)	NNNNNCCATNTWNNNWN	TATATACATATAAATAT
M00059	V$YY1_01	0.791612	6600	(-)	NNNNNCCATNTWNNNWN	ATATAAATATGAAAAAA
M00059	V$YY1_01	0.806029	6610	(-)	NNNNNCCATNTWNNNWN	GAAAAAATATGTACGTA
M00059	V$YY1_01	0.774246	6707	(+)	NNNNNCCATNTWNNNWN	ATGCAACATTTACTGAG
M00162	V$OCT1_06	0.834375	23	(-)	CWNAWTKWSATRYN	TAAATGACAACTCT
M00162	V$OCT1_06	0.808203	95	(+)	CWNAWTKWSATRYN	AAATCTTTCCTTTT
M00162	V$OCT1_06	0.873437	104	(+)	CWNAWTKWSATRYN	CTTTTTGTCATCTT
M00137	V$OCT1_03	0.852627	125	(+)	NNNRTAATNANNN	GGTGTAATGTAGG
M00195	V$OCT1_Q6	0.803493	138	(+)	NNNNATGCAAATNAN	AGGAAGATAAATCAC

M00162	V$OCT1_06	0.889062	144	(+)	CWNAWTKWSATRYN	ATAAATCACATGCT
M00162	V$OCT1_06	0.924609	145	(-)	CWNAWTKWSATRYN	TAAATCACATGCTG
M00195	V$OCT1_Q6	0.805404	152	(+)	NNNNATGCAAATNAN	CATGCTGCAAAGCAC
M00162	V$OCT1_06	0.801172	170	(+)	CWNAWTKWSATRYN	CACCCTTTAATTAG
M00137	V$OCT1_03	0.842355	205	(-)	NNNRTAATNANNN	TCTTCTTTACAGT
M00162	V$OCT1_06	0.834375	206	(+)	CWNAWTKWSATRYN	CTTCTTTACAGTTG
M00162	V$OCT1_06	0.816016	248	(+)	CWNAWTKWSATRYN	AGACCTGACAGGCA
M00137	V$OCT1_03	0.899249	268	(+)	NNNRTAATNANNN	AAGGTAATCAAAT
M00138	V$OCT1_04	0.797992	271	(+)	NNNNNNNWATGCAAATNNNWNNW	GTAATCAAATTGAAATGTCCAGA
M00162	V$OCT1_06	0.958594	276	(+)	CWNAWTKWSATRYN	CAAATTGAAATGTC
M00162	V$OCT1_06	0.821875	276	(-)	CWNAWTKWSATRYN	CAAATTGAAATGTC
M00162	V$OCT1_06	0.808203	277	(-)	CWNAWTKWSATRYN	AAATTGAAATGTCC
M00137	V$OCT1_03	0.861715	315	(+)	NNNRTAATNANNN	GAGTTAATCATAA
M00137	V$OCT1_03	0.858949	321	(+)	NNNRTAATNANNN	ATCATAATTTCTT
M00162	V$OCT1_06	0.825	323	(+)	CWNAWTKWSATRYN	CATAATTTCTTCCT
M00136	V$OCT1_02	0.921494	354	(-)	NNGAATATKCANNNN	AGCTTGAATATGCCT
M00138	V$OCT1_04	0.817231	354	(-)	NNNNNNNWATGCAAATNNNWNNW	AGCTTGAATATGCCTTGGCATCA
M00136	V$OCT1_02	0.861938	357	(+)	NNGAATATKCANNNN	TTGAATATGCCTTGG
M00138	V$OCT1_04	0.785027	360	(-)	NNNNNNNWATGCAAATNNNWNNW	AATATGCCTTGGCATCATTTTGA
M00162	V$OCT1_06	0.831641	373	(+)	CWNAWTKWSATRYN	ATCATTTTGACATT
M00162	V$OCT1_06	0.855469	375	(+)	CWNAWTKWSATRYN	CATTTTGACATTAG
M00162	V$OCT1_06	0.898437	399	(+)	CWNAWTKWSATRYN	AAACCTTAGATTCT
M00138	V$OCT1_04	0.794647	402	(-)	NNNNNNNWATGCAAATNNNWNNW	CCTTAGATTCTGTATAATGTTAT
M00137	V$OCT1_03	0.887791	412	(+)	NNNRTAATNANNN	TGTATAATGTTAT
M00138	V$OCT1_04	0.799665	415	(+)	NNNNNNNWATGCAAATNNNWNNW	ATAATGTTATGATTAAATTTATT
M00137	V$OCT1_03	0.840774	421	(-)	NNNRTAATNANNN	TTATGATTAAATT
M00162	V$OCT1_06	0.826562	446	(+)	CWNAWTKWSATRYN	CACTTTGGGTTATT
M00138	V$OCT1_04	0.825596	487	(+)	NNNNNNNWATGCAAATNNNWNNW	TGAAGACAATGTAAATTGTAAAG
M00135	V$OCT1_01	0.753148	489	(+)	NNNNWTATGCAAATNTNNN	AAGACAATGTAAATTGTAA
M00162	V$OCT1_06	0.808594	491	(+)	CWNAWTKWSATRYN	GACAATGTAAATTG
M00195	V$OCT1_Q6	0.862718	491	(+)	NNNNATGCAAATNAN	GACAATGTAAATTGT
M00248	V$OCT1_07	0.895908	492	(+)	TNTATGNTAATT	ACAATGTAAATT
M00138	V$OCT1_04	0.79611	493	(+)	NNNNNNNWATGCAAATNNNWNNW	CAATGTAAATTGTAAAGAAAAGT

M00137	V$OCT1_03	0.86211	501	(+)	NNNRTAATNANNN	ATTGTAAAGAAAA
M00162	V$OCT1_06	0.847266	561	(-)	CWNAWTKWSATRYN	AACTTCTCAGAAAG
M00137	V$OCT1_03	0.871987	583	(+)	NNNRTAATNANNN	GTCATAATAATCC
M00162	V$OCT1_06	0.800781	588	(-)	CWNAWTKWSATRYN	AATAATCCAGACTG
M00138	V$OCT1_04	0.79381	606	(+)	NNNNNNNWATGCAAATNNNWNNW	CTTAACTAATTTAAAATTTTTAT
M00138	V$OCT1_04	0.82476	608	(-)	NNNNNNNWATGCAAATNNNWNNW	TAACTAATTTAAAATTTTTATTT
M00162	V$OCT1_06	0.852734	611	(+)	CWNAWTKWSATRYN	CTAATTTAAAATTT
M00162	V$OCT1_06	0.808594	611	(-)	CWNAWTKWSATRYN	CTAATTTAAAATTT
M00195	V$OCT1_Q6	0.799672	611	(-)	NNNNATGCAAATNAN	CTAATTTAAAATTTT
M00162	V$OCT1_06	0.826562	612	(-)	CWNAWTKWSATRYN	TAATTTAAAATTTT
M00138	V$OCT1_04	0.816813	614	(-)	NNNNNNNWATGCAAATNNNWNNW	ATTTAAAATTTTTATTTCTATCT
M00162	V$OCT1_06	0.847656	618	(+)	CWNAWTKWSATRYN	AAAATTTTTATTTC
M00138	V$OCT1_04	0.845253	619	(+)	NNNNNNNWATGCAAATNNNWNNW	AAATTTTTATTTCTATCTAAAGA
M00162	V$OCT1_06	0.800391	619	(+)	CWNAWTKWSATRYN	AAATTTTTATTTCT
M00138	V$OCT1_04	0.782309	625	(+)	NNNNNNNWATGCAAATNNNWNNW	TTATTTCTATCTAAAGATTATAA
M00162	V$OCT1_06	0.847266	630	(-)	CWNAWTKWSATRYN	TCTATCTAAAGATT
M00137	V$OCT1_03	0.848676	636	(-)	NNNRTAATNANNN	TAAAGATTATAAT
M00162	V$OCT1_06	0.826953	638	(+)	CWNAWTKWSATRYN	AAGATTATAATTTT
M00137	V$OCT1_03	0.857764	641	(+)	NNNRTAATNANNN	ATTATAATTTTAT
M00162	V$OCT1_06	0.812109	643	(+)	CWNAWTKWSATRYN	TATAATTTTATACG
M00138	V$OCT1_04	0.783772	663	(+)	NNNNNNNWATGCAAATNNNWNNW	TATAACTTTTTTAAAAATTATGA
M00138	V$OCT1_04	0.781681	663	(-)	NNNNNNNWATGCAAATNNNWNNW	TATAACTTTTTTAAAAATTATGA
M00162	V$OCT1_06	0.839453	668	(+)	CWNAWTKWSATRYN	CTTTTTTAAAAATT
M00137	V$OCT1_03	0.877914	674	(-)	NNNRTAATNANNN	TAAAAATTATGAG
M00138	V$OCT1_04	0.794856	678	(-)	NNNNNNNWATGCAAATNNNWNNW	AATTATGAGTTACATATCCTCAC
M00162	V$OCT1_06	0.841797	678	(+)	CWNAWTKWSATRYN	AATTATGAGTTACA
M00162	V$OCT1_06	0.855469	682	(+)	CWNAWTKWSATRYN	ATGAGTTACATATC
M00195	V$OCT1_Q6	0.816048	682	(-)	NNNNATGCAAATNAN	ATGAGTTACATATCC
M00136	V$OCT1_02	0.797239	684	(-)	NNGAATATKCANNNN	GAGTTACATATCCTC
M00136	V$OCT1_02	0.816188	703	(-)	NNGAATATKCANNNN	AAGCTGAATAGTTCT
M00138	V$OCT1_04	0.837097	751	(-)	NNNNNNNWATGCAAATNNNWNNW	TGGTTTAAGTAACATAAGTAAAT
M00135	V$OCT1_01	0.747425	753	(-)	NNNNWTATGCAAATNTNNN	GTTTAAGTAACATAAGTAA
M00248	V$OCT1_07	0.845665	757	(-)	TNTATGNTAATT	AAGTAACATAAG

M00135	V$OCT1_01	0.773178	760	(+)	NNNNWTATGCAAATNTNNN	TAACATAAGTAAATTATGG
M00195	V$OCT1_Q6	0.820142	762	(+)	NNNNATGCAAATNAN	ACATAAGTAAATTAT
M00162	V$OCT1_06	0.847266	763	(-)	CWNAWTKWSATRYN	CATAAGTAAATTAT
M00248	V$OCT1_07	0.821709	763	(+)	TNTATGNTAATT	CATAAGTAAATT
M00138	V$OCT1_04	0.781472	788	(+)	NNNNNNNWATGCAAATNNNWNNW	TCTGATTTCTGTTTATGCTAAAA
M00137	V$OCT1_03	0.887396	794	(-)	NNNRTAATNANNN	TTCTGTTTATGCT
M00138	V$OCT1_04	0.832079	794	(+)	NNNNNNNWATGCAAATNNNWNNW	TTCTGTTTATGCTAAAAAAGAGG
M00135	V$OCT1_01	0.830027	796	(+)	NNNNWTATGCAAATNTNNN	CTGTTTATGCTAAAAAAGA
M00136	V$OCT1_02	0.779101	796	(+)	NNGAATATKCANNNN	CTGTTTATGCTAAAA
M00248	V$OCT1_07	0.799237	799	(+)	TNTATGNTAATT	TTTATGCTAAAA
M00135	V$OCT1_01	0.744372	823	(-)	NNNNWTATGCAAATNTNNN	TGTCATTTTACAGATTGCT
M00162	V$OCT1_06	0.813672	826	(-)	CWNAWTKWSATRYN	CATTTTACAGATTG
M00138	V$OCT1_04	0.78189	847	(+)	NNNNNNNWATGCAAATNNNWNNW	GACTGTGTGTGTAAATCTGTATA
M00135	V$OCT1_01	0.776803	849	(+)	NNNNWTATGCAAATNTNNN	CTGTGTGTGTAAATCTGTA
M00195	V$OCT1_Q6	0.822325	851	(+)	NNNNATGCAAATNAN	GTGTGTGTAAATCTG
M00162	V$OCT1_06	0.88125	852	(-)	CWNAWTKWSATRYN	TGTGTGTAAATCTG
M00138	V$OCT1_04	0.83187	854	(-)	NNNNNNNWATGCAAATNNNWNNW	TGTGTAAATCTGTATATGTGTTA
M00138	V$OCT1_04	0.797992	855	(+)	NNNNNNNWATGCAAATNNNWNNW	GTGTAAATCTGTATATGTGTTAT
M00135	V$OCT1_01	0.776803	856	(-)	NNNNWTATGCAAATNTNNN	TGTAAATCTGTATATGTGT
M00138	V$OCT1_04	0.786491	860	(-)	NNNNNNNWATGCAAATNNNWNNW	AATCTGTATATGTGTTATAATTT
M00162	V$OCT1_06	0.826172	865	(+)	CWNAWTKWSATRYN	GTATATGTGTTATA
M00137	V$OCT1_03	0.841169	873	(+)	NNNRTAATNANNN	GTTATAATTTTGC
M00162	V$OCT1_06	0.800781	887	(-)	CWNAWTKWSATRYN	TGAATTGAAAACAT
M00138	V$OCT1_04	0.780008	912	(+)	NNNNNNNWATGCAAATNNNWNNW	TCTTAACTATTCCAACTACAAAA
M00138	V$OCT1_04	0.844835	932	(+)	NNNNNNNWATGCAAATNNNWNNW	AAAAAGAAATGATAATTATGTGA
M00195	V$OCT1_Q6	0.837336	936	(+)	NNNNATGCAAATNAN	AGAAATGATAATTAT
M00161	V$OCT1_05	0.921966	937	(-)	MKNATTTGCATAYY	GAAATGATAATTAT
M00162	V$OCT1_06	0.839844	937	(-)	CWNAWTKWSATRYN	GAAATGATAATTAT
M00248	V$OCT1_07	0.829553	937	(+)	TNTATGNTAATT	GAAATGATAATT
M00137	V$OCT1_03	0.928487	940	(+)	NNNRTAATNANNN	ATGATAATTATGT
M00137	V$OCT1_03	0.888186	941	(-)	NNNRTAATNANNN	TGATAATTATGTG
M00162	V$OCT1_06	0.821094	945	(+)	CWNAWTKWSATRYN	AATTATGTGACATG
M00248	V$OCT1_07	0.806021	962	(+)	TNTATGNTAATT	GAAGTGGTAATT

M00137	V$OCT1_03	0.898459	965	(+)	NNNRTAATNANNN	GTGGTAATTATCA
M00137	V$OCT1_03	0.88226	966	(-)	NNNRTAATNANNN	TGGTAATTATCAC
M00195	V$OCT1_Q6	0.792303	979	(+)	NNNNATGCAAATNAN	TACAATGGCAATCAC
M00137	V$OCT1_03	0.867246	983	(+)	NNNRTAATNANNN	ATGGCAATCACAT
M00138	V$OCT1_04	0.783354	986	(+)	NNNNNNNWATGCAAATNNNWNNW	GCAATCACATTACAATATATAAA
M00135	V$OCT1_01	0.772034	989	(-)	NNNNWTATGCAAATNTNNN	ATCACATTACAATATATAA
M00137	V$OCT1_03	0.897669	989	(-)	NNNRTAATNANNN	ATCACATTACAAT
M00162	V$OCT1_06	0.868359	991	(-)	CWNAWTKWSATRYN	CACATTACAATATA
M00248	V$OCT1_07	0.834429	993	(-)	TNTATGNTAATT	CATTACAATATA
M00138	V$OCT1_04	0.823714	994	(+)	NNNNNNNWATGCAAATNNNWNNW	ATTACAATATATAAATATATCAA
M00138	V$OCT1_04	0.820577	995	(-)	NNNNNNNWATGCAAATNNNWNNW	TTACAATATATAAATATATCAAA
M00135	V$OCT1_01	0.798359	996	(+)	NNNNWTATGCAAATNTNNN	TACAATATATAAATATATC
M00138	V$OCT1_04	0.797365	997	(-)	NNNNNNNWATGCAAATNNNWNNW	ACAATATATAAATATATCAAATC
M00162	V$OCT1_06	0.847266	999	(+)	CWNAWTKWSATRYN	AATATATAAATATA
M00162	V$OCT1_06	0.825	999	(-)	CWNAWTKWSATRYN	AATATATAAATATA
M00137	V$OCT1_03	0.850652	1001	(+)	NNNRTAATNANNN	TATATAAATATAT
M00138	V$OCT1_04	0.810749	1002	(+)	NNNNNNNWATGCAAATNNNWNNW	ATATAAATATATCAAATCAACAT
M00162	V$OCT1_06	0.860156	1009	(-)	CWNAWTKWSATRYN	TATATCAAATCAAC
M00162	V$OCT1_06	0.812109	1014	(+)	CWNAWTKWSATRYN	CAAATCAACATGTC
M00162	V$OCT1_06	0.813672	1014	(-)	CWNAWTKWSATRYN	CAAATCAACATGTC
M00248	V$OCT1_07	0.803265	1016	(-)	TNTATGNTAATT	AATCAACATGTC
M00162	V$OCT1_06	0.958203	1019	(+)	CWNAWTKWSATRYN	CAACATGTCATATA
M00162	V$OCT1_06	0.883594	1020	(-)	CWNAWTKWSATRYN	AACATGTCATATAA
M00138	V$OCT1_04	0.825387	1025	(+)	NNNNNNNWATGCAAATNNNWNNW	GTCATATAATTTAAATTTACACA
M00138	V$OCT1_04	0.895232	1026	(-)	NNNNNNNWATGCAAATNNNWNNW	TCATATAATTTAAATTTACACAA
M00162	V$OCT1_06	0.916797	1030	(+)	CWNAWTKWSATRYN	ATAATTTAAATTTA
M00195	V$OCT1_Q6	0.845524	1030	(-)	NNNNATGCAAATNAN	ATAATTTAAATTTAC
M00248	V$OCT1_07	0.832309	1030	(+)	TNTATGNTAATT	ATAATTTAAATT
M00162	V$OCT1_06	0.826562	1031	(-)	CWNAWTKWSATRYN	TAATTTAAATTTAC
M00138	V$OCT1_04	0.834588	1032	(-)	NNNNNNNWATGCAAATNNNWNNW	AATTTAAATTTACACAATGTTAT
M00248	V$OCT1_07	0.851601	1032	(-)	TNTATGNTAATT	AATTTAAATTTA
M00135	V$OCT1_01	0.810568	1034	(-)	NNNNWTATGCAAATNTNNN	TTTAAATTTACACAATGTT
M00248	V$OCT1_07	0.847149	1038	(-)	TNTATGNTAATT	AATTTACACAAT

M00162	V$OCT1_06	0.881641	1044	(+)	CWNAWTKWSATRYN	CACAATGTTATATG
M00162	V$OCT1_06	0.807031	1046	(+)	CWNAWTKWSATRYN	CAATGTTATATGTC
M00138	V$OCT1_04	0.783563	1047	(+)	NNNNNNNWATGCAAATNNNWNNW	AATGTTATATGTCTAATATATTT
M00162	V$OCT1_06	0.833984	1052	(-)	CWNAWTKWSATRYN	TATATGTCTAATAT
M00162	V$OCT1_06	0.833984	1054	(-)	CWNAWTKWSATRYN	TATGTCTAATATAT
M00138	V$OCT1_04	0.790046	1059	(-)	NNNNNNNWATGCAAATNNNWNNW	CTAATATATTTGTGTTAAAAAAT
M00138	V$OCT1_04	0.812422	1062	(+)	NNNNNNNWATGCAAATNNNWNNW	ATATATTTGTGTTAAAAAATGAA
M00162	V$OCT1_06	0.839453	1069	(-)	CWNAWTKWSATRYN	TGTGTTAAAAAATG
M00162	V$OCT1_06	0.860547	1077	(-)	CWNAWTKWSATRYN	AAAATGAAAAAAGG
M00162	V$OCT1_06	0.821484	1090	(+)	CWNAWTKWSATRYN	GTAACTGGCAAGTG
M00162	V$OCT1_06	0.826562	1124	(+)	CWNAWTKWSATRYN	ATTCTTTTCATTGA
M00195	V$OCT1_Q6	0.805131	1124	(-)	NNNNATGCAAATNAN	ATTCTTTTCATTGAA
M00138	V$OCT1_04	0.817231	1126	(-)	NNNNNNNWATGCAAATNNNWNNW	TCTTTTCATTGAAATTTGAGTTT
M00162	V$OCT1_06	0.834766	1130	(+)	CWNAWTKWSATRYN	TTCATTGAAATTTG
M00162	V$OCT1_06	0.817578	1130	(-)	CWNAWTKWSATRYN	TTCATTGAAATTTG
M00248	V$OCT1_07	0.854357	1130	(+)	TNTATGNTAATT	TTCATTGAAATT
M00138	V$OCT1_04	0.801129	1132	(-)	NNNNNNNWATGCAAATNNNWNNW	CATTGAAATTTGAGTTTGCTTGT
M00162	V$OCT1_06	0.800781	1136	(+)	CWNAWTKWSATRYN	GAAATTTGAGTTTG
M00162	V$OCT1_06	0.848047	1136	(-)	CWNAWTKWSATRYN	GAAATTTGAGTTTG
M00138	V$OCT1_04	0.782518	1223	(-)	NNNNNNNWATGCAAATNNNWNNW	AGTCTTTCTAATCATTATTATCT
M00137	V$OCT1_03	0.88305	1227	(+)	NNNRTAATNANNN	TTTCTAATCATTA
M00137	V$OCT1_03	0.913868	1231	(-)	NNNRTAATNANNN	TAATCATTATTAT
M00137	V$OCT1_03	0.913868	1234	(-)	NNNRTAATNANNN	TCATTATTATCTG
M00162	V$OCT1_06	0.826562	1247	(-)	CWNAWTKWSATRYN	TGAATCAAAAGCAA
M00162	V$OCT1_06	0.826562	1253	(-)	CWNAWTKWSATRYN	AAAAGCAAATGGAG
M00137	V$OCT1_03	0.860529	1276	(-)	NNNRTAATNANNN	TTCCCTTTACCAA
M00162	V$OCT1_06	0.860547	1320	(-)	CWNAWTKWSATRYN	TAAAACTCAGTTTG
M00135	V$OCT1_01	0.797596	1325	(+)	NNNNWTATGCAAATNTNNN	CTCAGTTTGCAAGTCTCAA
M00162	V$OCT1_06	0.821484	1328	(-)	CWNAWTKWSATRYN	AGTTTGCAAGTCTC
M00138	V$OCT1_04	0.785864	1348	(+)	NNNNNNNWATGCAAATNNNWNNW	ATACAAGTAGGTCAATAACAGTA
M00162	V$OCT1_06	0.860156	1353	(-)	CWNAWTKWSATRYN	AGTAGGTCAATAAC
M00195	V$OCT1_Q6	0.852347	1384	(-)	NNNNATGCAAATNAN	GTGGTTAGCATTTAT
M00162	V$OCT1_06	0.842187	1390	(-)	CWNAWTKWSATRYN	AGCATTTATTTCAC

M00162	V$OCT1_06	0.818359	1396	(-)	CWNAWTKWSATRYN	TATTTCACAAATAC
M00248	V$OCT1_07	0.795209	1404	(-)	TNTATGNTAATT	AAATACCACATA
M00162	V$OCT1_06	0.826172	1405	(-)	CWNAWTKWSATRYN	AATACCACATATAC
M00138	V$OCT1_04	0.795274	1409	(+)	NNNNNNNNWATGCAAATNNNWNNW	CCACATATACTGAAATATTAAAA
M00162	V$OCT1_06	0.868359	1414	(+)	CWNAWTKWSATRYN	TATACTGAAATATT
M00162	V$OCT1_06	0.836719	1421	(+)	CWNAWTKWSATRYN	AAATATTAAAAGCT
M00162	V$OCT1_06	0.890625	1422	(-)	CWNAWTKWSATRYN	AATATTAAAAGCTT
M00138	V$OCT1_04	0.795065	1437	(-)	NNNNNNNNWATGCAAATNNNWNNW	AACTGTTATATGTATCTTTCTAA
M00138	V$OCT1_04	0.787118	1438	(+)	NNNNNNNNWATGCAAATNNNWNNW	ACTGTTATATGTATCTTTCTAAA
M00138	V$OCT1_04	0.817859	1459	(+)	NNNNNNNNWATGCAAATNNNWNNW	AAAAGACTATTTTTAAAAAATCA
M00138	V$OCT1_04	0.780845	1462	(-)	NNNNNNNNWATGCAAATNNNWNNW	AGACTATTTTTAAAAAATCAAAT
M00162	V$OCT1_06	0.813281	1466	(-)	CWNAWTKWSATRYN	TATTTTTAAAAAAT
M00195	V$OCT1_Q6	0.812227	1473	(+)	NNNNATGCAAATNAN	AAAAAATCAAATAAG
M00162	V$OCT1_06	0.832031	1474	(-)	CWNAWTKWSATRYN	AAAAATCAAATAAG
M00162	V$OCT1_06	0.834375	1475	(-)	CWNAWTKWSATRYN	AAAATCAAATAAGC
M00137	V$OCT1_03	0.840774	1480	(+)	NNNRTAATNANNN	CAAATAAGCAGGT
M00138	V$OCT1_04	0.805312	1527	(+)	NNNNNNNNWATGCAAATNNNWNNW	GTTTATTTCTTTAAATATTTCAG
M00138	V$OCT1_04	0.835634	1528	(-)	NNNNNNNNWATGCAAATNNNWNNW	TTTATTTCTTTAAATATTTCAGT
M00162	V$OCT1_06	0.847266	1532	(+)	CWNAWTKWSATRYN	TTTCTTTAAATATT
M00136	V$OCT1_02	0.779913	1534	(-)	NNGAATATKCANNNN	TCTTTAAATATTTCA
M00162	V$OCT1_06	0.828906	1539	(+)	CWNAWTKWSATRYN	AAATATTTCAGTCA
M00162	V$OCT1_06	0.847656	1540	(-)	CWNAWTKWSATRYN	AATATTTCAGTCAA
M00138	V$OCT1_04	0.79862	1556	(-)	NNNNNNNNWATGCAAATNNNWNNW	CCCTGTCATTGTCATACCTATGT
M00135	V$OCT1_01	0.731782	1558	(-)	NNNNWTATGCAAATNTNNN	CTGTCATTGTCATACCTAT
M00137	V$OCT1_03	0.84275	1558	(-)	NNNRTAATNANNN	CTGTCATTGTCAT
M00161	V$OCT1_05	0.856052	1560	(+)	MKNATTTGCATAYY	GTCATTGTCATACC
M00162	V$OCT1_06	0.916797	1560	(+)	CWNAWTKWSATRYN	GTCATTGTCATACC
M00195	V$OCT1_Q6	0.832697	1560	(-)	NNNNATGCAAATNAN	GTCATTGTCATACCT
M00137	V$OCT1_03	0.854603	1575	(-)	NNNRTAATNANNN	ATGTTTTTATTAA
M00138	V$OCT1_04	0.826851	1576	(-)	NNNNNNNNWATGCAAATNNNWNNW	TGTTTTTATTAAAACAATCAAGT
M00137	V$OCT1_03	0.84196	1578	(-)	NNNRTAATNANNN	TTTTTATTAAAAC
M00162	V$OCT1_06	0.807031	1623	(-)	CWNAWTKWSATRYN	GAGAAGTAATTGTG
M00137	V$OCT1_03	0.858159	1625	(+)	NNNRTAATNANNN	GAAGTAATTGTGA

M00137	V$OCT1_03	0.865666	1626	(-)	NNNRTAATNANNN	AAGTAATTGTGAA
M00138	V$OCT1_04	0.786491	1666	(-)	NNNNNNNWATGCAAATNNNWNNW	TCCTCCATCTTACATGTTCCTGT
M00162	V$OCT1_06	0.876172	1670	(+)	CWNAWTKWSATRYN	CCATCTTACATGTT
M00162	V$OCT1_06	0.808203	1671	(-)	CWNAWTKWSATRYN	CATCTTACATGTTC
M00136	V$OCT1_02	0.788847	1672	(-)	NNGAATATKCANNNN	ATCTTACATGTTCCT
M00162	V$OCT1_06	0.864844	1701	(-)	CWNAWTKWSATRYN	GGAATTACAGAATT
M00162	V$OCT1_06	0.800781	1709	(-)	CWNAWTKWSATRYN	AGAATTTTAGAAAT
M00162	V$OCT1_06	0.833984	1710	(+)	CWNAWTKWSATRYN	GAATTTTAGAAATT
M00162	V$OCT1_06	0.800391	1712	(+)	CWNAWTKWSATRYN	ATTTTAGAAATTTT
M00138	V$OCT1_04	0.782309	1713	(+)	NNNNNNNWATGCAAATNNNWNNW	TTTTAGAAATTTTAGTTCAAACT
M00138	V$OCT1_04	0.7867	1714	(-)	NNNNNNNWATGCAAATNNNWNNW	TTTAGAAATTTTAGTTCAAACTT
M00162	V$OCT1_06	0.826562	1718	(+)	CWNAWTKWSATRYN	GAAATTTTAGTTCA
M00138	V$OCT1_04	0.784609	1726	(-)	NNNNNNNWATGCAAATNNNWNNW	AGTTCAAACTTTCATTTATAGGT
M00135	V$OCT1_01	0.74628	1728	(-)	NNNNWTATGCAAATNTNNN	TTCAAACTTTCATTTATAG
M00162	V$OCT1_06	0.950781	1730	(+)	CWNAWTKWSATRYN	CAAACTTTCATTTA
M00162	V$OCT1_06	0.834375	1731	(-)	CWNAWTKWSATRYN	AAACTTTCATTTAT
M00136	V$OCT1_02	0.775041	1746	(-)	NNGAATATKCANNNN	GGTAAGAATAGTAAG
M00248	V$OCT1_07	0.795845	1767	(+)	TNTATGNTAATT	ATAAAGGAAATT
M00162	V$OCT1_06	0.834375	1768	(-)	CWNAWTKWSATRYN	TAAAGGAAATTACT
M00135	V$OCT1_01	0.780618	1784	(-)	NNNNWTATGCAAATNTNNN	GACAAAATTGCACAAGTAG
M00162	V$OCT1_06	0.829297	1808	(+)	CWNAWTKWSATRYN	GAGCCTGAGATGGA
M00137	V$OCT1_03	0.846306	1886	(-)	NNNRTAATNANNN	CCTTGTTTACAAA
M00162	V$OCT1_06	0.855859	1914	(+)	CWNAWTKWSATRYN	CAGACTGGCATGGG
M00162	V$OCT1_06	0.811719	1929	(-)	CWNAWTKWSATRYN	AGCCTCTCCTTTTG
M00162	V$OCT1_06	0.826562	1934	(+)	CWNAWTKWSATRYN	CTCCTTTTGCTGCC
M00138	V$OCT1_04	0.785236	1979	(-)	NNNNNNNWATGCAAATNNNWNNW	TTTCTCCATTTAAATGCCCATGG
M00162	V$OCT1_06	0.834766	1983	(-)	CWNAWTKWSATRYN	TCCATTTAAATGCC
M00138	V$OCT1_04	0.803639	1990	(+)	NNNNNNNWATGCAAATNNNWNNW	AAATGCCCATGGATATTCAGAAG
M00136	V$OCT1_02	0.858419	1995	(-)	NNGAATATKCANNNN	CCCATGGATATTCAG
M00136	V$OCT1_02	0.890633	1998	(+)	NNGAATATKCANNNN	ATGGATATTCAGAAG
M00138	V$OCT1_04	0.812631	2018	(-)	NNNNNNNWATGCAAATNNNWNNW	CTCTTCTATTTGAATGTTCTCTA
M00135	V$OCT1_01	0.753529	2020	(-)	NNNNWTATGCAAATNTNNN	CTTCTATTTGAATGTTCTC
M00195	V$OCT1_Q6	0.797216	2022	(-)	NNNNATGCAAATNAN	TCTATTTGAATGTTC

M00136	V$OCT1_02	0.881159	2024	(-)	NNGAATATKCANNNN	TATTTGAATGTTCTC
M00138	V$OCT1_04	0.816395	2039	(-)	NNNNNNNWATGCAAATNNNWNNW	TATTAATCTCTGCATGAGTAAGT
M00135	V$OCT1_01	0.739603	2105	(-)	NNNNWTATGCAAATNTNNN	TGAAAACTTCTATATGTAG
M00135	V$OCT1_01	0.737314	2112	(+)	NNNNWTATGCAAATNTNNN	TTCTATATGTAGAAAGCCA
M00162	V$OCT1_06	0.860156	2191	(+)	CWNAWTKWSATRYN	CTACAATTGATGCT
M00137	V$OCT1_03	0.873173	2235	(+)	NNNRTAATNANNN	TTGATAATCTCAA
M00162	V$OCT1_06	0.808594	2248	(-)	CWNAWTKWSATRYN	CACTTGACAGTGCC
M00138	V$OCT1_04	0.79611	2260	(-)	NNNNNNNWATGCAAATNNNWNNW	CCTTGTTCTTTATATATATAAGC
M00138	V$OCT1_04	0.780845	2262	(-)	NNNNNNNWATGCAAATNNNWNNW	TTGTTCTTTATATATATAAGCTA
M00162	V$OCT1_06	0.847266	2264	(+)	CWNAWTKWSATRYN	GTTCTTTATATATA
M00195	V$OCT1_Q6	0.855076	2264	(-)	NNNNATGCAAATNAN	GTTCTTTATATATAT
M00138	V$OCT1_04	0.789419	2271	(+)	NNNNNNNWATGCAAATNNNWNNW	ATATATATAAGCTAAAATTTTTA
M00135	V$OCT1_01	0.783289	2273	(+)	NNNNWTATGCAAATNTNNN	ATATATAAGCTAAAATTTT
M00138	V$OCT1_04	0.809494	2279	(+)	NNNNNNNWATGCAAATNNNWNNW	AAGCTAAAATTTTTATTTATAGG
M00138	V$OCT1_04	0.794437	2280	(-)	NNNNNNNWATGCAAATNNNWNNW	AGCTAAAATTTTTATTTATAGGT
M00162	V$OCT1_06	0.860547	2284	(+)	CWNAWTKWSATRYN	AAAATTTTTATTTA
M00138	V$OCT1_04	0.810121	2285	(+)	NNNNNNNWATGCAAATNNNWNNW	AAATTTTTATTTATAGGTAAGAA
M00138	V$OCT1_04	0.844207	2313	(-)	NNNNNNNWATGCAAATNNNWNNW	CCTATAAATGTACATATATATTT
M00135	V$OCT1_01	0.730446	2314	(+)	NNNNWTATGCAAATNTNNN	CTATAAATGTACATATATA
M00138	V$OCT1_04	0.804684	2314	(+)	NNNNNNNWATGCAAATNNNWNNW	CTATAAATGTACATATATATTTA
M00135	V$OCT1_01	0.810187	2315	(-)	NNNNWTATGCAAATNTNNN	TATAAATGTACATATATAT
M00138	V$OCT1_04	0.788164	2316	(+)	NNNNNNNWATGCAAATNNNWNNW	ATAAATGTACATATATATTTATA
M00162	V$OCT1_06	0.811719	2317	(+)	CWNAWTKWSATRYN	TAAATGTACATATA
M00138	V$OCT1_04	0.842325	2318	(+)	NNNNNNNWATGCAAATNNNWNNW	AAATGTACATATATATTTATAGG
M00248	V$OCT1_07	0.866441	2319	(-)	TNTATGNTAATT	AATGTACATATA
M00138	V$OCT1_04	0.780008	2324	(+)	NNNNNNNWATGCAAATNNNWNNW	ACATATATATTTATAGGCACCAT
M00137	V$OCT1_03	0.849072	2328	(-)	NNNRTAATNANNN	ATATATTTATAGG
M00138	V$OCT1_04	0.803011	2329	(-)	NNNNNNNWATGCAAATNNNWNNW	TATATTTATAGGCACCATTATGT
M00137	V$OCT1_03	0.873568	2340	(-)	NNNRTAATNANNN	GCACCATTATGTG
M00135	V$OCT1_01	0.742274	2343	(-)	NNNNWTATGCAAATNTNNN	CCATTATGTGCCTATACAT
M00136	V$OCT1_02	0.79778	2347	(-)	NNGAATATKCANNNN	TATGTGCCTATACAT
M00162	V$OCT1_06	0.842187	2356	(+)	CWNAWTKWSATRYN	ATACATTATATATG
M00138	V$OCT1_04	0.833961	2358	(-)	NNNNNNNWATGCAAATNNNWNNW	ACATTATATATGTGTATGTATAT

M00138	V$OCT1_04	0.793601	2364	(-)	NNNNNNNWATGCAAATNNNWNNW	TATATGTGTATGTATATGTGTAT
M00138	V$OCT1_04	0.871602	2365	(+)	NNNNNNNWATGCAAATNNNWNNW	ATATGTGTATGTATATGTGTATA
M00135	V$OCT1_01	0.78348	2367	(+)	NNNNWTATGCAAATNTNNN	ATGTGTATGTATATGTGTA
M00138	V$OCT1_04	0.824969	2370	(-)	NNNNNNNWATGCAAATNNNWNNW	TGTATGTATATGTGTATACATAG
M00162	V$OCT1_06	0.88125	2370	(-)	CWNAWTKWSATRYN	TGTATGTATATGTG
M00138	V$OCT1_04	0.81054	2371	(+)	NNNNNNNWATGCAAATNNNWNNW	GTATGTATATGTGTATACATAGG
M00136	V$OCT1_02	0.787493	2373	(+)	NNGAATATKCANNNN	ATGTATATGTGTATA
M00135	V$OCT1_01	0.752385	2374	(-)	NNNNWTATGCAAATNTNNN	TGTATATGTGTATACATAG
M00138	V$OCT1_04	0.785445	2377	(+)	NNNNNNNWATGCAAATNNNWNNW	ATATGTGTATACATAGGGACACA
M00162	V$OCT1_06	0.804297	2411	(+)	CWNAWTKWSATRYN	CAAAGTTTAAAGTT
M00138	V$OCT1_04	0.804475	2417	(+)	NNNNNNNWATGCAAATNNNWNNW	TTAAAGTTATTCAAACTACCTGT
M00135	V$OCT1_01	0.730256	2419	(+)	NNNNWTATGCAAATNTNNN	AAAGTTATTCAAACTACCT
M00136	V$OCT1_02	0.808338	2419	(+)	NNGAATATKCANNNN	AAAGTTATTCAAACT
M00195	V$OCT1_Q6	0.79012	2421	(+)	NNNNATGCAAATNAN	AGTTATTCAAACTAC
M00162	V$OCT1_06	0.826562	2432	(+)	CWNAWTKWSATRYN	CTACCTGTCCTTCT
M00162	V$OCT1_06	0.86875	2452	(+)	CWNAWTKWSATRYN	CAGAATAAAATTCT
M00162	V$OCT1_06	0.855469	2453	(-)	CWNAWTKWSATRYN	AGAATAAAATTCTC
M00136	V$OCT1_02	0.780726	2455	(+)	NNGAATATKCANNNN	AATAAAATTCTCATG
M00162	V$OCT1_06	0.88125	2458	(+)	CWNAWTKWSATRYN	AAAATTCTCATGTA
M00138	V$OCT1_04	0.785445	2459	(+)	NNNNNNNWATGCAAATNNNWNNW	AAATTCTCATGTAATTTAATTAT
M00162	V$OCT1_06	0.819922	2464	(-)	CWNAWTKWSATRYN	CTCATGTAATTTAA
M00138	V$OCT1_04	0.822877	2465	(-)	NNNNNNNWATGCAAATNNNWNNW	TCATGTAATTTAATTATTTATAA
M00137	V$OCT1_03	0.84196	2466	(+)	NNNRTAATNANNN	CATGTAATTTAAT
M00248	V$OCT1_07	0.836549	2468	(+)	TNTATGNTAATT	TGTAATTTAATT
M00162	V$OCT1_06	0.839453	2469	(+)	CWNAWTKWSATRYN	GTAATTTAATTATT
M00162	V$OCT1_06	0.842578	2469	(-)	CWNAWTKWSATRYN	GTAATTTAATTATT
M00138	V$OCT1_04	0.812212	2471	(-)	NNNNNNNWATGCAAATNNNWNNW	AATTTAATTATTTATAAAAAATT
M00137	V$OCT1_03	0.883445	2472	(-)	NNNRTAATNANNN	ATTTAATTATTTA
M00138	V$OCT1_04	0.833542	2472	(+)	NNNNNNNWATGCAAATNNNWNNW	ATTTAATTATTTATAAAAAATTG
M00138	V$OCT1_04	0.782936	2473	(-)	NNNNNNNWATGCAAATNNNWNNW	TTTAATTATTTATAAAAAATTGT
M00138	V$OCT1_04	0.785655	2475	(-)	NNNNNNNWATGCAAATNNNWNNW	TAATTATTTATAAAAAATTGTGT
M00135	V$OCT1_01	0.734643	2478	(+)	NNNNWTATGCAAATNTNNN	TTATTTATAAAAAATTGTG
M00162	V$OCT1_06	0.804297	2483	(-)	CWNAWTKWSATRYN	TATAAAAAATTGTG

M00162	V$OCT1_06	0.803125	2496	(+)	CWNAWTKWSATRYN	GTCTATGACTTTCC
M00137	V$OCT1_03	0.850652	2507	(-)	NNNRTAATNANNN	TCCTTATTAATGG
M00162	V$OCT1_06	0.847266	2512	(+)	CWNAWTKWSATRYN	ATTAATGGCCTACT
M00138	V$OCT1_04	0.785027	2536	(-)	NNNNNNNNWATGCAAATNNNWNNW	TGGTACATGTTAAATGAATATAT
M00248	V$OCT1_07	0.836337	2539	(+)	TNTATGNTAATT	TACATGTTAAAT
M00138	V$OCT1_04	0.798202	2541	(+)	NNNNNNNNWATGCAAATNNNWNNW	CATGTTAAATGAATATATGATTA
M00162	V$OCT1_06	0.800391	2541	(-)	CWNAWTKWSATRYN	CATGTTAAATGAAT
M00162	V$OCT1_06	0.826562	2544	(+)	CWNAWTKWSATRYN	GTTAAATGAATATA
M00136	V$OCT1_02	0.811586	2546	(-)	NNGAATATKCANNNN	TAAATGAATATATGA
M00162	V$OCT1_06	0.873437	2546	(-)	CWNAWTKWSATRYN	TAAATGAATATATG
M00138	V$OCT1_04	0.782518	2549	(+)	NNNNNNNNWATGCAAATNNNWNNW	ATGAATATATGATTAACCTTGTA
M00162	V$OCT1_06	0.804297	2552	(-)	CWNAWTKWSATRYN	AATATATGATTAAC
M00138	V$OCT1_04	0.785655	2553	(-)	NNNNNNNNWATGCAAATNNNWNNW	ATATATGATTAACCTTGTAATGA
M00137	V$OCT1_03	0.870012	2555	(-)	NNNRTAATNANNN	ATATGATTAACCT
M00162	V$OCT1_06	0.821484	2563	(+)	CWNAWTKWSATRYN	AACCTTGTAATGAT
M00137	V$OCT1_03	0.925326	2566	(+)	NNNRTAATNANNN	CTTGTAATGATAC
M00161	V$OCT1_05	0.868504	2575	(+)	MKNATTTGCATAYY	ATACTTTTAATATT
M00162	V$OCT1_06	0.895703	2575	(+)	CWNAWTKWSATRYN	ATACTTTTAATATT
M00162	V$OCT1_06	0.813281	2588	(-)	CWNAWTKWSATRYN	TATAGCCAAAAATC
M00162	V$OCT1_06	0.855469	2595	(+)	CWNAWTKWSATRYN	AAAAATCAAATTCA
M00162	V$OCT1_06	0.916797	2596	(-)	CWNAWTKWSATRYN	AAAATCAAATTCAT
M00136	V$OCT1_02	0.819166	2598	(+)	NNGAATATKCANNNN	AATCAAATTCATACA
M00162	V$OCT1_06	0.847266	2599	(+)	CWNAWTKWSATRYN	ATCAAATTCATACA
M00137	V$OCT1_03	0.865271	2609	(-)	NNNRTAATNANNN	TACACATTATACT
M00138	V$OCT1_04	0.840443	2632	(-)	NNNNNNNNWATGCAAATNNNWNNW	TCTAAAGTTTTTAATAAAATAAA
M00162	V$OCT1_06	0.813672	2636	(+)	CWNAWTKWSATRYN	AAGTTTTTAATAAA
M00137	V$OCT1_03	0.848676	2639	(+)	NNNRTAATNANNN	TTTTTAATAAAAT
M00162	V$OCT1_06	0.834375	2645	(+)	CWNAWTKWSATRYN	ATAAAATAAATTTA
M00162	V$OCT1_06	0.826562	2646	(-)	CWNAWTKWSATRYN	TAAAATAAATTTAC
M00138	V$OCT1_04	0.787327	2733	(-)	NNNNNNNNWATGCAAATNNNWNNW	TCCTAAATTATTAGTAATAGATC
M00137	V$OCT1_03	0.871592	2737	(-)	NNNRTAATNANNN	AAATTATTAGTAA
M00162	V$OCT1_06	0.869922	2753	(+)	CWNAWTKWSATRYN	ATCCTTTTAATTTA
M00138	V$OCT1_04	0.783772	2754	(-)	NNNNNNNNWATGCAAATNNNWNNW	TCCTTTTAATTTAATTTTTTTT

M00138	V$OCT1_04	0.794437	2755	(-)	NNNNNNNWATGCAAATNNNWNNW	CCTTTTAATTTAATTTTTTTTTA
M00162	V$OCT1_06	0.860547	2758	(+)	CWNAWTKWSATRYN	TTTAATTTAATTTT
M00248	V$OCT1_07	0.831673	2758	(+)	TNTATGNTAATT	TTTAATTTAATT
M00162	V$OCT1_06	0.855469	2759	(-)	CWNAWTKWSATRYN	TTAATTTAATTTTT
M00138	V$OCT1_04	0.833752	2765	(-)	NNNNNNNWATGCAAATNNNWNNW	TAATTTTTTTTTAATTTTTTAAT
M00162	V$OCT1_06	0.826562	2769	(+)	CWNAWTKWSATRYN	TTTTTTTTAATTTT
M00248	V$OCT1_07	0.831673	2769	(+)	TNTATGNTAATT	TTTTTTTTAATT
M00138	V$OCT1_04	0.857173	2773	(-)	NNNNNNNWATGCAAATNNNWNNW	TTTTAATTTTTTAATTTTTATTT
M00162	V$OCT1_06	0.890625	2777	(+)	CWNAWTKWSATRYN	AATTTTTTAATTTT
M00248	V$OCT1_07	0.812381	2777	(+)	TNTATGNTAATT	AATTTTTTAATT
M00138	V$OCT1_04	0.817859	2778	(+)	NNNNNNNWATGCAAATNNNWNNW	ATTTTTTAATTTTTATTTTTAGG
M00162	V$OCT1_06	0.800391	2778	(+)	CWNAWTKWSATRYN	ATTTTTTAATTTTT
M00138	V$OCT1_04	0.831242	2779	(-)	NNNNNNNWATGCAAATNNNWNNW	TTTTTTAATTTTTATTTTTAGGT
M00195	V$OCT1_Q6	0.823144	2783	(-)	NNNNATGCAAATNAN	TTAATTTTTATTTTT
M00137	V$OCT1_03	0.875543	2799	(+)	NNNRTAATNANNN	GGTGCAATCATGA
M00162	V$OCT1_06	0.868359	2866	(+)	CWNAWTKWSATRYN	GTTAATTGCATCTA
M00195	V$OCT1_Q6	0.834334	2866	(-)	NNNNATGCAAATNAN	GTTAATTGCATCTAA
M00162	V$OCT1_06	0.932031	2872	(-)	CWNAWTKWSATRYN	TGCATCTAATGAAT
M00137	V$OCT1_03	0.865666	2874	(+)	NNNRTAATNANNN	CATCTAATGAATT
M00136	V$OCT1_02	0.782891	2877	(-)	NNGAATATKCANNNN	CTAATGAATTTGTAT
M00138	V$OCT1_04	0.836679	2877	(-)	NNNNNNNWATGCAAATNNNWNNW	CTAATGAATTTGTATTAAAACTT
M00135	V$OCT1_01	0.737696	2879	(-)	NNNNWTATGCAAATNTNNN	AATGAATTTGTATTAAAAC
M00136	V$OCT1_02	0.775582	2880	(+)	NNGAATATKCANNNN	ATGAATTTGTATTAA
M00138	V$OCT1_04	0.833961	2882	(+)	NNNNNNNWATGCAAATNNNWNNW	GAATTTGTATTAAAACTTTAAGA
M00248	V$OCT1_07	0.820225	2883	(-)	TNTATGNTAATT	AATTTGTATTAA
M00162	V$OCT1_06	0.847266	2887	(-)	CWNAWTKWSATRYN	TGTATTAAAACTTT
M00135	V$OCT1_01	0.762877	2895	(+)	NNNNWTATGCAAATNTNNN	AACTTTAAGAAAATTTTAT
M00248	V$OCT1_07	0.794785	2898	(+)	TNTATGNTAATT	TTTAAGAAAATT
M00162	V$OCT1_06	0.868359	2903	(+)	CWNAWTKWSATRYN	GAAAATTTTATTTA
M00138	V$OCT1_04	0.780845	2905	(-)	NNNNNNNWATGCAAATNNNWNNW	AAATTTTATTTAAAGGCTCTAAT
M00162	V$OCT1_06	0.800781	2909	(-)	CWNAWTKWSATRYN	TTTATTTAAAGGCT
M00162	V$OCT1_06	0.825	2918	(-)	CWNAWTKWSATRYN	AGGCTCTAATTATG
M00137	V$OCT1_03	0.872382	2920	(+)	NNNRTAATNANNN	GCTCTAATTATGT

M0013		0.90952				
7	V$OCT1_03	2	2921	(-)	NNNRTAATNANNN	CTCTAATTATGTA
M0013		0.86177			NNNNNNNWATGCAAATNNN	
8	V$OCT1_04	3	2921	(+)	WNNW	CTCTAATTATGTAAATTTCTAAA
M0013		0.79360			NNNNNNNWATGCAAATNNN	
8	V$OCT1_04	1	2922	(-)	WNNW	TCTAATTATGTAAATTTCTAAAT
M0013		0.92159				
5	V$OCT1_01	5	2923	(+)	NNNNWTATGCAAATNTNNN	CTAATTATGTAAATTTCTA
M0016		0.80820				
2	V$OCT1_06	3	2925	(+)	CWNAWTKWSATRYN	AATTATGTAAATTT
M0019		0.84061				
5	V$OCT1_Q6	1	2925	(+)	NNNNATGCAAATNAN	AATTATGTAAATTTC
M0016		0.81367				
2	V$OCT1_06	2	2926	(+)	CWNAWTKWSATRYN	ATTATGTAAATTTC
M0016						
2	V$OCT1_06	0.88125	2926	(-)	CWNAWTKWSATRYN	ATTATGTAAATTTC
M0024		0.94509				
8	V$OCT1_07	2	2926	(+)	TNTATGNTAATT	ATTATGTAAATT
M0013		0.83751			NNNNNNNWATGCAAATNNN	
8	V$OCT1_04	6	2930	(-)	WNNW	TGTAAATTTCTAAATAGTTATAT
M0013		0.78230			NNNNNNNWATGCAAATNNN	
8	V$OCT1_04	9	2936	(-)	WNNW	TTTCTAAATAGTTATATTTATAG
M0013		0.83145			NNNNNNNWATGCAAATNNN	
8	V$OCT1_04	1	2937	(+)	WNNW	TTCTAAATAGTTATATTTATAGA
M0013		0.78607			NNNNNNNWATGCAAATNNN	
8	V$OCT1_04	3	2941	(+)	WNNW	AAATAGTTATATTTATAGAGCAA
M0013		0.78544			NNNNNNNWATGCAAATNNN	
8	V$OCT1_04	5	2944	(-)	WNNW	TAGTTATATTTATAGAGCAAAAT
M0013		0.73731				
5	V$OCT1_01	4	2962	(-)	NNNNWTATGCAAATNTNNN	AAAATAGCTGCATACTAAC
M0013		0.84082				
6	V$OCT1_02	3	2966	(-)	NNGAATATKCANNNN	TAGCTGCATACTAAC
M0013		0.87348			NNNNNNNWATGCAAATNNN	
8	V$OCT1_04	4	2977	(-)	WNNW	TAACTATATTTCATAAAAGTAA
M0013		0.82678				
5	V$OCT1_01	4	2979	(-)	NNNNWTATGCAAATNTNNN	ACTATATTTCATAAAAGT
M0016		0.81718				
2	V$OCT1_06	7	2981	(+)	CWNAWTKWSATRYN	TATATTTTCATAAA
M0024		0.79478				
8	V$OCT1_07	5	2983	(-)	TNTATGNTAATT	TATTTTCATAAA
M0016		0.86796				
2	V$OCT1_06	9	2995	(-)	CWNAWTKWSATRYN	AGTAACTCATTTTT
M0013		0.80698			NNNNNNNWATGCAAATNNN	
8	V$OCT1_04	5	2998	(-)	WNNW	AACTCATTTTTACATTTATTTCT
M0013		0.77127				
5	V$OCT1_01	1	3000	(-)	NNNNWTATGCAAATNTNNN	CTCATTTTTACATTTATTT
M0019		0.79912				
5	V$OCT1_Q6	7	3000	(-)	NNNNATGCAAATNAN	CTCATTTTTACATTT
M0013		0.84037				
7	V$OCT1_03	9	3001	(-)	NNNRTAATNANNN	TCATTTTTACATT
M0016		0.94531				
2	V$OCT1_06	2	3002	(+)	CWNAWTKWSATRYN	CATTTTTACATTTA
M0013		0.82099			NNNNNNNWATGCAAATNNN	
8	V$OCT1_04	5	3007	(+)	WNNW	TTACATTTATTTCTATTACAAAA
M0013					NNNNNNNWATGCAAATNNN	
8	V$OCT1_04	0.81033	3008	(-)	WNNW	TACATTTATTTCTATTACAAAAT
M0013		0.78983			NNNNNNNWATGCAAATNNN	
8	V$OCT1_04	7	3013	(+)	WNNW	TTATTTCTATTACAAAATTTAAA
M0013		0.85381				
7	V$OCT1_03	3	3016	(-)	NNNRTAATNANNN	TTTCTATTACAAA
M0016		0.82656				
2	V$OCT1_06	2	3018	(-)	CWNAWTKWSATRYN	TCTATTACAAAATT
M0016		0.87773				
2	V$OCT1_06	4	3026	(-)	CWNAWTKWSATRYN	AAAATTTAAAAGAT
M0016		0.82656				
2	V$OCT1_06	2	3027	(-)	CWNAWTKWSATRYN	AAATTTAAAAGATG

M00162	V$OCT1_06	0.800391	3060	(-)	CWNAWTKWSATRYN	AGAATTAAGAAATC
M00162	V$OCT1_06	0.868359	3075	(+)	CWNAWTKWSATRYN	CACTTTATCATGTA
M00138	V$OCT1_04	0.798202	3076	(+)	NNNNNNNWATGCAAATNNNWNNW	ACTTTATCATGTATAAGAAAATC
M00138	V$OCT1_04	0.807612	3082	(+)	NNNNNNNWATGCAAATNNNWNNW	TCATGTATAAGAAAATCTTTAAA
M00135	V$OCT1_01	0.759443	3084	(+)	NNNNWTATGCAAATNTNNN	ATGTATAAGAAAATCTTTA
M00162	V$OCT1_06	0.847266	3087	(-)	CWNAWTKWSATRYN	TATAAGAAAATCTT
M00162	V$OCT1_06	0.800391	3094	(+)	CWNAWTKWSATRYN	AAATCTTTAAAATT
M00162	V$OCT1_06	0.821094	3095	(-)	CWNAWTKWSATRYN	AATCTTTAAAATTC
M00162	V$OCT1_06	0.800781	3096	(+)	CWNAWTKWSATRYN	ATCTTTAAAATTCT
M00136	V$OCT1_02	0.779101	3099	(+)	NNGAATATKCANNNN	TTTAAAATTCTCATA
M00135	V$OCT1_01	0.765548	3100	(-)	NNNNWTATGCAAATNTNNN	TTAAAATTCTCATATGATA
M00162	V$OCT1_06	0.860547	3102	(+)	CWNAWTKWSATRYN	AAAATTCTCATATG
M00162	V$OCT1_06	0.812109	3122	(-)	CWNAWTKWSATRYN	AAAATATATTTTTG
M00138	V$OCT1_04	0.791928	3151	(+)	NNNNNNNWATGCAAATNNNWNNW	GAAGATATTTTTTAATTATAATA
M00138	V$OCT1_04	0.845253	3152	(-)	NNNNNNNWATGCAAATNNNWNNW	AAGATATTTTTTAATTATAATAT
M00248	V$OCT1_07	0.831673	3156	(+)	TNTATGNTAATT	TATTTTTTAATT
M00162	V$OCT1_06	0.813281	3157	(+)	CWNAWTKWSATRYN	ATTTTTTAATTATA
M00138	V$OCT1_04	0.837934	3158	(-)	NNNNNNNWATGCAAATNNNWNNW	TTTTTTAATTATAATATGCTCTA
M00137	V$OCT1_03	0.865271	3159	(+)	NNNRTAATNANNN	TTTTTAATTATAA
M00137	V$OCT1_03	0.888582	3160	(-)	NNNRTAATNANNN	TTTTAATTATAAT
M00248	V$OCT1_07	0.813229	3164	(-)	TNTATGNTAATT	AATTATAATATG
M00136	V$OCT1_02	0.845425	3167	(+)	NNGAATATKCANNNN	TATAATATGCTCTAA
M00138	V$OCT1_04	0.793392	3180	(+)	NNNNNNNWATGCAAATNNNWNNW	AAAAAATAATTCACAAAAAATAA
M00136	V$OCT1_02	0.785328	3182	(+)	NNGAATATKCANNNN	AAAATAATTCACAAA
M00248	V$OCT1_07	0.798177	3187	(-)	TNTATGNTAATT	AATTCACAAAAA
M00162	V$OCT1_06	0.862891	3194	(+)	CWNAWTKWSATRYN	AAAAAATAAATGCA
M00138	V$OCT1_04	0.842744	3195	(+)	NNNNNNNWATGCAAATNNNWNNW	AAAAATAAATGCAAAAATCATAG
M00135	V$OCT1_01	0.829073	3197	(+)	NNNNWTATGCAAATNTNNN	AAATAAATGCAAAAATCAT
M00136	V$OCT1_02	0.779643	3197	(+)	NNGAATATKCANNNN	AAATAAATGCAAAAA
M00195	V$OCT1_Q6	0.82369	3199	(+)	NNNNATGCAAATNAN	ATAAATGCAAAAATC
M00162	V$OCT1_06	0.898437	3200	(-)	CWNAWTKWSATRYN	TAAATGCAAAAATC
M00138	V$OCT1_04	0.781263	3225	(+)	NNNNNNNWATGCAAATNNNWNNW	TCAGAAATGTTTTAAATTAAATT
M00138	V$OCT1_04	0.796947	3227	(-)	NNNNNNNWATGCAAATNNNWNNW	AGAAATGTTTTAAATTAAATTCC

M00162	V$OCT1_06	0.800781	3231	(+)	CWNAWTKWSATRYN	ATGTTTTAAATTAA
M00138	V$OCT1_04	0.781054	3232	(-)	NNNNNNNWATGCAAATNNNWNNW	TGTTTTAAATTAAATTCCTATCT
M00162	V$OCT1_06	0.855469	3236	(+)	CWNAWTKWSATRYN	TTAAATTAAATTCC
M00162	V$OCT1_06	0.891016	3237	(-)	CWNAWTKWSATRYN	TAAATTAAATTCCT
M00162	V$OCT1_06	0.800781	3242	(-)	CWNAWTKWSATRYN	TAAATTCCTATCTC
M00162	V$OCT1_06	0.807031	3371	(-)	CWNAWTKWSATRYN	TACATTTTATTGTA
M00135	V$OCT1_01	0.751049	3429	(+)	NNNNWTATGCAAATNTNNN	GTCCATAATCAAATCTAAT
M00137	V$OCT1_03	0.914658	3430	(+)	NNNRTAATNANNN	TCCATAATCAAAT
M00162	V$OCT1_06	0.825391	3432	(+)	CWNAWTKWSATRYN	CATAATCAAATCTA
M00162	V$OCT1_06	0.932422	3438	(-)	CWNAWTKWSATRYN	CAAATCTAATTTTC
M00138	V$OCT1_04	0.78921	3439	(-)	NNNNNNNWATGCAAATNNNWNNW	AAATCTAATTTTCAGGCATTTTT
M00162	V$OCT1_06	0.886328	3443	(+)	CWNAWTKWSATRYN	CTAATTTTCAGGCA
M00195	V$OCT1_Q6	0.825328	3443	(-)	NNNNATGCAAATNAN	CTAATTTTCAGGCAT
M00136	V$OCT1_02	0.791012	3449	(-)	NNGAATATKCANNNN	TTCAGGCATTTTTTT
M00135	V$OCT1_01	0.733499	3462	(-)	NNNNWTATGCAAATNTNNN	TTAAAAGTTACATTCTGTG
M00162	V$OCT1_06	0.876172	3464	(+)	CWNAWTKWSATRYN	AAAAGTTACATTCT
M00162	V$OCT1_06	0.847656	3465	(-)	CWNAWTKWSATRYN	AAAGTTACATTCTG
M00248	V$OCT1_07	0.797965	3466	(-)	TNTATGNTAATT	AAGTTACATTCT
M00162	V$OCT1_06	0.826562	3479	(-)	CWNAWTKWSATRYN	TGTATTAAAGCTAT
M00138	V$OCT1_04	0.824341	3518	(+)	NNNNNNNWATGCAAATNNNWNNW	ATTTTTTTCTTTTAATTTTTAGA
M00162	V$OCT1_06	0.813281	3518	(+)	CWNAWTKWSATRYN	ATTTTTTTCTTTTA
M00138	V$OCT1_04	0.800502	3519	(-)	NNNNNNNWATGCAAATNNNWNNW	TTTTTTTCTTTTAATTTTTAGAC
M00162	V$OCT1_06	0.826562	3523	(+)	CWNAWTKWSATRYN	TTTCTTTTAATTTT
M00248	V$OCT1_07	0.831673	3523	(+)	TNTATGNTAATT	TTTCTTTTAATT
M00138	V$OCT1_04	0.837307	3525	(-)	NNNNNNNWATGCAAATNNNWNNW	TCTTTTAATTTTTAGACTTGTTT
M00162	V$OCT1_06	0.808203	3531	(+)	CWNAWTKWSATRYN	AATTTTTAGACTTG
M00138	V$OCT1_04	0.811376	3537	(-)	NNNNNNNWATGCAAATNNNWNNW	TAGACTTGTTTTCATTGTCATTT
M00195	V$OCT1_Q6	0.819869	3541	(-)	NNNNATGCAAATNAN	CTTGTTTTCATTGTC
M00138	V$OCT1_04	0.802175	3543	(-)	NNNNNNNWATGCAAATNNNWNNW	TGTTTTCATTGTCATTTTGGCTA
M00137	V$OCT1_03	0.854998	3545	(-)	NNNRTAATNANNN	TTTTCATTGTCAT
M00162	V$OCT1_06	0.860547	3547	(+)	CWNAWTKWSATRYN	TTCATTGTCATTTT
M00162	V$OCT1_06	0.812109	3548	(-)	CWNAWTKWSATRYN	TCATTGTCATTTTG
M00162	V$OCT1_06	0.834375	3571	(+)	CWNAWTKWSATRYN	CTGCTTTACTTTCA

M00195	V$OCT1_Q6	0.832969	3571	(-)	NNNNATGCAAATNAN	CTGCTTTACTTTCAC
M00135	V$OCT1_01	0.813239	3606	(-)	NNNNWTATGCAAATNTNNN	CACTTATTTGCAAACAGCA
M00135	V$OCT1_01	0.734262	3607	(+)	NNNNWTATGCAAATNTNNN	ACTTATTTGCAAACAGCAA
M00162	V$OCT1_06	0.865625	3608	(+)	CWNAWTKWSATRYN	CTTATTTGCAAACA
M00195	V$OCT1_Q6	0.891103	3608	(-)	NNNNATGCAAATNAN	CTTATTTGCAAACAG
M00162	V$OCT1_06	0.828906	3667	(-)	CWNAWTKWSATRYN	AGACTGTAAAATTT
M00136	V$OCT1_02	0.779372	3701	(+)	NNGAATATKCANNNN	TTCATAATTCACTTA
M00138	V$OCT1_04	0.803639	3707	(+)	NNNNNNNWATGCAAATNNNWNNW	ATTCACTTATTATTAAATTTAGA
M00137	V$OCT1_03	0.887791	3710	(-)	NNNRTAATNANNN	CACTTATTATTAA
M00162	V$OCT1_06	0.85	3714	(+)	CWNAWTKWSATRYN	TATTATTAAATTTA
M00162	V$OCT1_06	0.878906	3715	(-)	CWNAWTKWSATRYN	ATTATTAAATTTAG
M00162	V$OCT1_06	0.860547	3732	(-)	CWNAWTKWSATRYN	TGCTTTTAAGTTAG
M00162	V$OCT1_06	0.833984	3744	(-)	CWNAWTKWSATRYN	AGTATCTCCTAAAT
M00138	V$OCT1_04	0.816395	3759	(+)	NNNNNNNWATGCAAATNNNWNNW	ATTCTATGAGGTAAATTAGTGGA
M00162	V$OCT1_06	0.821094	3786	(-)	CWNAWTKWSATRYN	AACTTTTAAAAAAT
M00138	V$OCT1_04	0.806566	3792	(-)	NNNNNNNWATGCAAATNNNWNNW	TAAAAAATTTTGAACGACTATGA
M00162	V$OCT1_06	0.914062	3807	(+)	CWNAWTKWSATRYN	GACTATGACATTCA
M00162	V$OCT1_06	0.860547	3808	(-)	CWNAWTKWSATRYN	ACTATGACATTCAC
M00135	V$OCT1_01	0.731209	3811	(-)	NNNNWTATGCAAATNTNNN	ATGACATTCACATTTATTT
M00162	V$OCT1_06	0.855469	3813	(+)	CWNAWTKWSATRYN	GACATTCACATTTA
M00195	V$OCT1_Q6	0.797216	3813	(-)	NNNNATGCAAATNAN	GACATTCACATTTAT
M00162	V$OCT1_06	0.829297	3819	(-)	CWNAWTKWSATRYN	CACATTTATTTTCT
M00138	V$OCT1_04	0.795483	3823	(-)	NNNNNNNWATGCAAATNNNWNNW	TTTATTTTCTTTCATTTATTCTT
M00162	V$OCT1_06	0.834375	3827	(+)	CWNAWTKWSATRYN	TTTTCTTTCATTTA
M00162	V$OCT1_06	0.808594	3841	(+)	CWNAWTKWSATRYN	TTCTTTGAAATTTG
M00162	V$OCT1_06	0.860547	3846	(+)	CWNAWTKWSATRYN	TGAAATTTGATTTT
M00162	V$OCT1_06	0.813672	3847	(+)	CWNAWTKWSATRYN	GAAATTTGATTTTA
M00162	V$OCT1_06	0.842187	3941	(+)	CWNAWTKWSATRYN	CTGCCTGACCTGCA
M00162	V$OCT1_06	0.865625	3962	(+)	CWNAWTKWSATRYN	CTTACTGAGTTACT
M00162	V$OCT1_06	0.908984	4047	(-)	CWNAWTKWSATRYN	TAAATCAAAGGAAG
M00162	V$OCT1_06	0.860547	4059	(-)	CWNAWTKWSATRYN	AGAATCAAAGTTTA
M00162	V$OCT1_06	0.800391	4067	(+)	CWNAWTKWSATRYN	AGTTTATAGATTTT
M00138	V$OCT1_04	0.79632	4073	(-)	NNNNNNNWATGCAAATNNNWNNW	TAGATTTTTTACCATTCAATCTT

M00138	V$OCT1_04	0.824341	4087	(-)	NNNNNNNWATGCAAATNNNWNNW	TTCAATCTTATGAATGATCAAAA
M00138	V$OCT1_04	0.783772	4088	(+)	NNNNNNNWATGCAAATNNNWNNW	TCAATCTTATGAATGATCAAAAT
M00136	V$OCT1_02	0.785057	4093	(-)	NNGAATATKCANNNN	CTTATGAATGATCAA
M00138	V$OCT1_04	0.7821	4095	(+)	NNNNNNNWATGCAAATNNNWNNW	TATGAATGATCAAAATGATACAA
M00138	V$OCT1_04	0.854872	4104	(+)	NNNNNNNWATGCAAATNNNWNNW	TCAAAATGATACAAATTGAAAAA
M00135	V$OCT1_01	0.749332	4106	(+)	NNNNWTATGCAAATNTNNN	AAAATGATACAAATTGAAA
M00162	V$OCT1_06	0.842187	4121	(+)	CWNAWTKWSATRYN	GAAAAAGTAATGTG
M00137	V$OCT1_03	0.865666	4124	(+)	NNNRTAATNANNN	AAAGTAATGTGAA
M00162	V$OCT1_06	0.839453	4126	(+)	CWNAWTKWSATRYN	AGTAATGTGAAACA
M00136	V$OCT1_02	0.85896	4166	(+)	NNGAATATKCANNNN	AACTATATTCAATAT
M00162	V$OCT1_06	0.847656	4168	(+)	CWNAWTKWSATRYN	CTATATTCAATATC
M00162	V$OCT1_06	0.877734	4169	(-)	CWNAWTKWSATRYN	TATATTCAATATCT
M00138	V$OCT1_04	0.783772	4189	(+)	NNNNNNNWATGCAAATNNNWNNW	AACCTATAATGAAAAAGAATATG
M00137	V$OCT1_03	0.902015	4191	(+)	NNNRTAATNANNN	CCTATAATGAAAA
M00136	V$OCT1_02	0.807255	4200	(-)	NNGAATATKCANNNN	AAAAAGAATATGAAA
M00138	V$OCT1_04	0.782936	4200	(-)	NNNNNNNWATGCAAATNNNWNNW	AAAAAGAATATGAAAACGAATAC
M00138	V$OCT1_04	0.847553	4201	(+)	NNNNNNNWATGCAAATNNNWNNW	AAAAGAATATGAAAACGAATACA
M00136	V$OCT1_02	0.837845	4203	(+)	NNGAATATKCANNNN	AAGAATATGAAAACG
M00138	V$OCT1_04	0.853199	4215	(+)	NNNNNNNWATGCAAATNNNWNNW	ACGAATACATGTATATATATGCA
M00162	V$OCT1_06	0.819531	4220	(-)	CWNAWTKWSATRYN	TACATGTATATATA
M00138	V$OCT1_04	0.889377	4224	(-)	NNNNNNNWATGCAAATNNNWNNW	TGTATATATATGCATGACTGAAA
M00135	V$OCT1_01	0.768027	4226	(-)	NNNNWTATGCAAATNTNNN	TATATATATGCATGACTGA
M00135	V$OCT1_01	0.741129	4227	(+)	NNNNWTATGCAAATNTNNN	ATATATATGCATGACTGAA
M00136	V$OCT1_02	0.845425	4227	(+)	NNGAATATKCANNNN	ATATATATGCATGAC
M00162	V$OCT1_06	0.821484	4230	(-)	CWNAWTKWSATRYN	TATATGCATGACTG
M00162	V$OCT1_06	0.821094	4234	(-)	CWNAWTKWSATRYN	TGCATGACTGAAAC
M00162	V$OCT1_06	0.813672	4237	(+)	CWNAWTKWSATRYN	ATGACTGAAACATT
M00137	V$OCT1_03	0.847886	4243	(-)	NNNRTAATNANNN	GAAACATTATGCT
M00136	V$OCT1_02	0.783162	4245	(+)	NNGAATATKCANNNN	AACATTATGCTGTAC
M00162	V$OCT1_06	0.819141	4286	(+)	CWNAWTKWSATRYN	CTGACTGTATTTCA
M00138	V$OCT1_04	0.826014	4287	(-)	NNNNNNNWATGCAAATNNNWNNW	TGACTGTATTTCAATTTAAAAAA
M00162	V$OCT1_06	0.834375	4290	(+)	CWNAWTKWSATRYN	CTGTATTTCAATTT
M00162	V$OCT1_06	0.88125	4291	(-)	CWNAWTKWSATRYN	TGTATTTCAATTTA

M00248	V$OCT1_07	0.842273	4291	(+)	TNTATGNTAATT	TGTATTTCAATT
M00138	V$OCT1_04	0.82706	4292	(+)	NNNNNNNWATGCAAATNNNWNNW	GTATTTCAATTTAAAAAAAGAAA
M00138	V$OCT1_04	0.783982	4293	(-)	NNNNNNNWATGCAAATNNNWNNW	TATTTCAATTTAAAAAAAGAAAA
M00162	V$OCT1_06	0.805859	4298	(-)	CWNAWTKWSATRYN	CAATTTAAAAAAAG
M00248	V$OCT1_07	0.807717	4299	(-)	TNTATGNTAATT	AATTTAAAAAAA
M00162	V$OCT1_06	0.839453	4306	(-)	CWNAWTKWSATRYN	AAAAAGAAAAAAAG
M00162	V$OCT1_06	0.821094	4314	(-)	CWNAWTKWSATRYN	AAAAAGAAATAAAT
M00162	V$OCT1_06	0.834375	4318	(-)	CWNAWTKWSATRYN	AGAAATAAATTATT
M00162	V$OCT1_06	0.90625	4325	(+)	CWNAWTKWSATRYN	AATTATTAAATTCT
M00162	V$OCT1_06	0.847656	4326	(-)	CWNAWTKWSATRYN	ATTATTAAATTCTT
M00138	V$OCT1_04	0.839398	4329	(+)	NNNNNNNWATGCAAATNNNWNNW	ATTAAATTCTTCAAAAAAAAAAA
M00162	V$OCT1_06	0.826953	4352	(+)	CWNAWTKWSATRYN	AGCAATGTGATAGC
M00135	V$OCT1_01	0.750286	4369	(+)	NNNNWTATGCAAATNTNNN	TTTTATGTGCTAAATGTAT
M00248	V$OCT1_07	0.799237	4372	(+)	TNTATGNTAATT	TATGTGCTAAAT
M00162	V$OCT1_06	0.805859	4398	(-)	CWNAWTKWSATRYN	CAAAATAAAAATAG
M00248	V$OCT1_07	0.817257	4401	(-)	TNTATGNTAATT	AATAAAAATAGT
M00135	V$OCT1_01	0.738649	4408	(+)	NNNNWTATGCAAATNTNNN	ATAGTTAAGATAATATTTA
M00138	V$OCT1_04	0.780636	4412	(+)	NNNNNNNWATGCAAATNNNWNNW	TTAAGATAATATTTATTACTAGC
M00136	V$OCT1_02	0.815376	4416	(+)	NNGAATATKCANNNN	GATAATATTTATTAC
M00137	V$OCT1_03	0.848281	4418	(-)	NNNRTAATNANNN	TAATATTTATTAC
M00137	V$OCT1_03	0.892533	4421	(-)	NNNRTAATNANNN	TATTTATTACTAG
M00137	V$OCT1_03	0.870802	4430	(+)	NNNRTAATNANNN	CTAGCAATTATGA
M00137	V$OCT1_03	0.874358	4431	(-)	NNNRTAATNANNN	TAGCAATTATGAG
M00162	V$OCT1_06	0.862891	4435	(+)	CWNAWTKWSATRYN	AATTATGAGATGAA
M00195	V$OCT1_Q6	0.795306	4444	(-)	NNNNATGCAAATNAN	ATGAACTGCATAATC
M00136	V$OCT1_02	0.857878	4446	(-)	NNGAATATKCANNNN	GAACTGCATAATCCA
M00162	V$OCT1_06	0.847656	4488	(+)	CWNAWTKWSATRYN	CAACTTTTCATTAC
M00137	V$OCT1_03	0.905966	4492	(-)	NNNRTAATNANNN	TTTTCATTACAGC
M00195	V$OCT1_Q6	0.808952	4573	(+)	NNNNATGCAAATNAN	ATCAATGAAAACTAT
M00135	V$OCT1_01	0.733689	4598	(+)	NNNNWTATGCAAATNTNNN	ACTGATATCCAGATGTTAT
M00162	V$OCT1_06	0.813672	4601	(-)	CWNAWTKWSATRYN	GATATCCAGATGTT
M00138	V$OCT1_04	0.787746	4606	(-)	NNNNNNNWATGCAAATNNNWNNW	CCAGATGTTATGCATCCCTCTAT
M00136	V$OCT1_02	0.790742	4609	(+)	NNGAATATKCANNNN	GATGTTATGCATCCC

M00138	V$OCT1_04	0.789419	4620	(-)	NNNNNNNWATGCAAATNNNWNNW	TCCCTCTATATACAATAAAATAT
M00162	V$OCT1_06	0.847266	4630	(-)	CWNAWTKWSATRYN	TACAATAAAATATT
M00137	V$OCT1_03	0.854603	4665	(+)	NNNRTAATNANNN	TTAGCAATAACGA
M00162	V$OCT1_06	0.829297	4681	(-)	CWNAWTKWSATRYN	AACATTTCAGGACA
M00137	V$OCT1_03	0.897274	4691	(+)	NNNRTAATNANNN	GACATAATCATTG
M00138	V$OCT1_04	0.788582	4703	(+)	NNNNNNNWATGCAAATNNNWNNW	GATCATTTATTTTTAAACTATAA
M00162	V$OCT1_06	0.808203	4703	(+)	CWNAWTKWSATRYN	GATCATTTATTTTT
M00138	V$OCT1_04	0.7821	4705	(-)	NNNNNNNWATGCAAATNNNWNNW	TCATTTATTTTTAAACTATAAAT
M00138	V$OCT1_04	0.796529	4713	(-)	NNNNNNNWATGCAAATNNNWNNW	TTTTAAACTATAAATGTGTAAGT
M00162	V$OCT1_06	0.816016	4717	(+)	CWNAWTKWSATRYN	AAACTATAAATGTG
M00135	V$OCT1_01	0.764403	4722	(+)	NNNNWTATGCAAATNTNNN	ATAAATGTGTAAGTATGAA
M00162	V$OCT1_06	0.88125	4725	(-)	CWNAWTKWSATRYN	AATGTGTAAGTATG
M00138	V$OCT1_04	0.845462	4728	(+)	NNNNNNNWATGCAAATNNNWNNW	GTGTAAGTATGAAAACTATGAGA
M00135	V$OCT1_01	0.738268	4730	(+)	NNNNWTATGCAAATNTNNN	GTAAGTATGAAAACTATGA
M00195	V$OCT1_Q6	0.827511	4732	(+)	NNNNATGCAAATNAN	AAGTATGAAAACTAT
M00161	V$OCT1_05	0.888594	4733	(-)	MKNATTTGCATAYY	AGTATGAAAACTAT
M00162	V$OCT1_06	0.860156	4733	(-)	CWNAWTKWSATRYN	AGTATGAAAACTAT
M00162	V$OCT1_06	0.842187	4741	(+)	CWNAWTKWSATRYN	AACTATGAGATAAT
M00162	V$OCT1_06	0.804297	4742	(-)	CWNAWTKWSATRYN	ACTATGAGATAATG
M00138	V$OCT1_04	0.850899	4745	(+)	NNNNNNNWATGCAAATNNNWNNW	ATGAGATAATGAAAAAATAATAT
M00137	V$OCT1_03	0.911102	4747	(+)	NNNRTAATNANNN	GAGATAATGAAAA
M00138	V$OCT1_04	0.813467	4774	(+)	NNNNNNNWATGCAAATNNNWNNW	TCATGTTTCTTGAAAAAAAATAA
M00162	V$OCT1_06	0.889062	4789	(+)	CWNAWTKWSATRYN	AAAAATAAGATGCA
M00136	V$OCT1_02	0.797239	4792	(+)	NNGAATATKCANNNN	AATAAGATGCAGGAT
M00136	V$OCT1_02	0.774228	4807	(+)	NNGAATATKCANNNN	ACGAAAATGAATGTA
M00162	V$OCT1_06	0.847656	4808	(+)	CWNAWTKWSATRYN	CGAAAATGAATGTA
M00138	V$OCT1_04	0.797574	4809	(+)	NNNNNNNWATGCAAATNNNWNNW	GAAAATGAATGTATAGCAGTTGT
M00195	V$OCT1_Q6	0.790939	4813	(+)	NNNNATGCAAATNAN	ATGAATGTATAGCAG
M00162	V$OCT1_06	0.834375	4814	(-)	CWNAWTKWSATRYN	TGAATGTATAGCAG
M00162	V$OCT1_06	0.828906	4829	(-)	CWNAWTKWSATRYN	TGTACGTAATACAT
M00162	V$OCT1_06	0.812109	4838	(-)	CWNAWTKWSATRYN	TACATATAAGTTAA
M00135	V$OCT1_01	0.77108	4839	(+)	NNNNWTATGCAAATNTNNN	ACATATAAGTTAATATTAT
M00138	V$OCT1_04	0.799875	4839	(+)	NNNNNNNWATGCAAATNNNWNNW	ACATATAAGTTAATATTATATTA

M00248	V$OCT1_07	0.792453	4842	(+)	TNTATGNTAATT	TATAAGTTAATA
M00136	V$OCT1_02	0.785869	4844	(-)	NNGAATATKCANNNN	TAAGTTAATATTATA
M00138	V$OCT1_04	0.79632	4850	(+)	NNNNNNNWATGCAAATNNNWNNW	AATATTATATTAAGATGTCTAGA
M00162	V$OCT1_06	0.878906	4850	(-)	CWNAWTKWSATRYN	AATATTATATTAAG
M00162	V$OCT1_06	0.812109	4855	(+)	CWNAWTKWSATRYN	TATATTAAGATGTC
M00162	V$OCT1_06	0.826562	4855	(-)	CWNAWTKWSATRYN	TATATTAAGATGTC
M00138	V$OCT1_04	0.79381	4865	(-)	NNNNNNNWATGCAAATNNNWNNW	TGTCTAGATTATCAGAATTTTTG
M00135	V$OCT1_01	0.736551	4867	(-)	NNNNWTATGCAAATNTNNN	TCTAGATTATCAGAATTTT
M00137	V$OCT1_03	0.872382	4867	(-)	NNNRTAATNANNN	TCTAGATTATCAG
M00162	V$OCT1_06	0.808203	4880	(+)	CWNAWTKWSATRYN	AATTTTTGAGTGTA
M00135	V$OCT1_01	0.732926	4887	(+)	NNNNWTATGCAAATNTNNN	GAGTGTAGGCAAACTTTCT
M00136	V$OCT1_02	0.800758	4902	(-)	NNGAATATKCANNNN	TTCTACAATATTCAT
M00138	V$OCT1_04	0.847972	4902	(-)	NNNNNNNWATGCAAATNNNWNNW	TTCTACAATATTCATATTACTTT
M00138	V$OCT1_04	0.813258	4903	(+)	NNNNNNNWATGCAAATNNNWNNW	TCTACAATATTCATATTACTTTT
M00135	V$OCT1_01	0.756009	4904	(-)	NNNNWTATGCAAATNTNNN	CTACAATATTCATATTACT
M00135	V$OCT1_01	0.758489	4905	(+)	NNNNWTATGCAAATNTNNN	TACAATATTCATATTACTT
M00136	V$OCT1_02	0.906605	4905	(+)	NNGAATATKCANNNN	TACAATATTCATATT
M00136	V$OCT1_02	0.817271	4908	(-)	NNGAATATKCANNNN	AATATTCATATTACT
M00138	V$OCT1_04	0.786909	4908	(-)	NNNNNNNWATGCAAATNNNWNNW	AATATTCATATTACTTTTTAAAT
M00162	V$OCT1_06	0.81875	4908	(-)	CWNAWTKWSATRYN	AATATTCATATTAC
M00248	V$OCT1_07	0.795209	4908	(+)	TNTATGNTAATT	AATATTCATATT
M00138	V$OCT1_04	0.798829	4916	(-)	NNNNNNNWATGCAAATNNNWNNW	TATTACTTTTTAAATTTTAAACA
M00162	V$OCT1_06	0.821484	4920	(+)	CWNAWTKWSATRYN	ACTTTTTAAATTTT
M00248	V$OCT1_07	0.793301	4920	(+)	TNTATGNTAATT	ACTTTTTAAATT
M00138	V$OCT1_04	0.80092	4921	(+)	NNNNNNNWATGCAAATNNNWNNW	CTTTTTAAATTTTAAACAAATGA
M00138	V$OCT1_04	0.801338	4923	(-)	NNNNNNNWATGCAAATNNNWNNW	TTTTAAATTTTAAACAAATGATT
M00162	V$OCT1_06	0.808203	4943	(+)	CWNAWTKWSATRYN	ATTTAATAAATTTA
M00138	V$OCT1_04	0.793183	4944	(+)	NNNNNNNWATGCAAATNNNWNNW	TTTAATAAATTTAGAGAAAAACA
M00138	V$OCT1_04	0.797156	4945	(-)	NNNNNNNWATGCAAATNNNWNNW	TTAATAAATTTAGAGAAAAACAA
M00162	V$OCT1_06	0.834766	4988	(-)	CWNAWTKWSATRYN	TGAATTCTATTATT
M00138	V$OCT1_04	0.817231	4995	(-)	NNNNNNNWATGCAAATNNNWNNW	TATTATTTTTTTCAAATTTCTAT
M00138	V$OCT1_04	0.807821	4996	(+)	NNNNNNNWATGCAAATNNNWNNW	ATTATTTTTTTCAAATTTCTATA
M00135	V$OCT1_01	0.755628	4998	(+)	NNNNWTATGCAAATNTNNN	TATTTTTTTCAAATTTCTA

M00162	V$OCT1_06	0.826172	4999	(+)	CWNAWTKWSATRYN	ATTTTTTTCAAATT
M00248	V$OCT1_07	0.814501	5001	(+)	TNTATGNTAATT	TTTTTTCAAATT
M00138	V$OCT1_04	0.825178	5003	(-)	NNNNNNNNWATGCAAATNNNWNNW	TTTTCAAATTTCTATACCTACAA
M00135	V$OCT1_01	0.778901	5005	(-)	NNNNWTATGCAAATNTNNN	TTCAAATTTCTATACCTAC
M00162	V$OCT1_06	0.817578	5007	(+)	CWNAWTKWSATRYN	CAAATTTCTATACC
M00162	V$OCT1_06	0.800781	5007	(-)	CWNAWTKWSATRYN	CAAATTTCTATACC
M00248	V$OCT1_07	0.842061	5009	(-)	TNTATGNTAATT	AATTTCTATACC
M00136	V$OCT1_02	0.788576	5021	(+)	NNGAATATKCANNNN	TACAACATTCAAAAG
M00162	V$OCT1_06	0.808594	5024	(-)	CWNAWTKWSATRYN	AACATTCAAAAGGT
M00162	V$OCT1_06	0.808594	5043	(+)	CWNAWTKWSATRYN	GAGTCTTTTATATC
M00162	V$OCT1_06	0.882812	5051	(-)	CWNAWTKWSATRYN	TATATCCAAGGAAT
M00136	V$OCT1_02	0.841094	5059	(+)	NNGAATATKCANNNN	AGGAATATGTGTGCA
M00195	V$OCT1_Q6	0.801037	5065	(+)	NNNNATGCAAATNAN	ATGTGTGCAATGTCC
M00162	V$OCT1_06	0.808203	5066	(-)	CWNAWTKWSATRYN	TGTGTGCAATGTCC
M00162	V$OCT1_06	0.860937	5086	(+)	CWNAWTKWSATRYN	CGAACTGCCATTTT
M00135	V$OCT1_01	0.754674	5092	(-)	NNNNWTATGCAAATNTNNN	GCCATTTTAAAATATTTAA
M00162	V$OCT1_06	0.860547	5094	(+)	CWNAWTKWSATRYN	CATTTTAAAATATT
M00162	V$OCT1_06	0.826562	5094	(-)	CWNAWTKWSATRYN	CATTTTAAAATATT
M00138	V$OCT1_04	0.800293	5096	(-)	NNNNNNNNWATGCAAATNNNWNNW	TTTTAAAATATTTAAAATTATGA
M00138	V$OCT1_04	0.806148	5097	(+)	NNNNNNNNWATGCAAATNNNWNNW	TTTAAAATATTTAAAATTATGAC
M00135	V$OCT1_01	0.763258	5099	(+)	NNNNWTATGCAAATNTNNN	TAAAATATTTAAAATTATG
M00136	V$OCT1_02	0.792637	5099	(+)	NNGAATATKCANNNN	TAAAATATTTAAAAT
M00162	V$OCT1_06	0.821094	5101	(+)	CWNAWTKWSATRYN	AAATATTTAAAATT
M00162	V$OCT1_06	0.834375	5102	(+)	CWNAWTKWSATRYN	AATATTTAAAATTA
M00162	V$OCT1_06	0.855078	5102	(-)	CWNAWTKWSATRYN	AATATTTAAAATTA
M00162	V$OCT1_06	0.817187	5104	(-)	CWNAWTKWSATRYN	TATTTAAAATTATG
M00137	V$OCT1_03	0.886606	5107	(-)	NNNRTAATNANNN	TTAAAATTATGAC
M00162	V$OCT1_06	0.826562	5150	(-)	CWNAWTKWSATRYN	AGTTTTAAAGTTTT
M00138	V$OCT1_04	0.840652	5153	(-)	NNNNNNNNWATGCAAATNNNWNNW	TTTAAAGTTTTATATATACATGC
M00135	V$OCT1_01	0.736932	5155	(-)	NNNNWTATGCAAATNTNNN	TAAAGTTTTATATATACAT
M00162	V$OCT1_06	0.842187	5157	(+)	CWNAWTKWSATRYN	AAGTTTTATATATA
M00138	V$OCT1_04	0.804266	5159	(-)	NNNNNNNNWATGCAAATNNNWNNW	GTTTTATATATACATGCCTATGG
M00162	V$OCT1_06	0.806641	5163	(+)	CWNAWTKWSATRYN	TATATATACATGCC

M00138	V$OCT1_04	0.817231	5164	(+)	NNNNNNNWATGCAAATNNNWNNW	ATATATACATGCCTATGGAAATT
M00138	V$OCT1_04	0.820786	5170	(+)	NNNNNNNWATGCAAATNNNWNNW	ACATGCCTATGGAAATTACTTTC
M00135	V$OCT1_01	0.813239	5172	(+)	NNNNWTATGCAAATNTNNN	ATGCCTATGGAAATTACTT
M00195	V$OCT1_Q6	0.862445	5174	(+)	NNNNATGCAAATNAN	GCCTATGGAAATTAC
M00161	V$OCT1_05	0.866678	5175	(-)	MKNATTTGCATAYY	CCTATGGAAATTAC
M00248	V$OCT1_07	0.927284	5175	(+)	TNTATGNTAATT	CCTATGGAAATT
M00162	V$OCT1_06	0.842187	5180	(+)	CWNAWTKWSATRYN	GGAAATTACTTTCT
M00162	V$OCT1_06	0.808594	5181	(-)	CWNAWTKWSATRYN	GAAATTACTTTCTC
M00137	V$OCT1_03	0.887396	5203	(+)	NNNRTAATNANNN	CTCATAATGTGTG
M00248	V$OCT1_07	0.793513	5227	(-)	TNTATGNTAATT	AATTAAGAAAAA
M00248	V$OCT1_07	0.796269	5236	(-)	TNTATGNTAATT	AAATACTATAAA
M00138	V$OCT1_04	0.786909	5245	(+)	NNNNNNNWATGCAAATNNNWNNW	AAAAACAAATGAAAGTTGATTTT
M00138	V$OCT1_04	0.791928	5257	(-)	NNNNNNNWATGCAAATNNNWNNW	AAGTTGATTTTTAAAAAGCATAA
M00138	V$OCT1_04	0.822877	5263	(-)	NNNNNNNWATGCAAATNNNWNNW	ATTTTTAAAAAGCATAATTATAA
M00138	V$OCT1_04	0.79862	5264	(+)	NNNNNNNWATGCAAATNNNWNNW	TTTTTAAAAAGCATAATTATAAA
M00135	V$OCT1_01	0.735025	5265	(-)	NNNNWTATGCAAATNTNNN	TTTTAAAAAGCATAATTAT
M00136	V$OCT1_02	0.818896	5269	(-)	NNGAATATKCANNNN	AAAAAGCATAATTAT
M00137	V$OCT1_03	0.924141	5273	(+)	NNNRTAATNANNN	AGCATAATTATAA
M00137	V$OCT1_03	0.898064	5274	(-)	NNNRTAATNANNN	GCATAATTATAAA
M00138	V$OCT1_04	0.793183	5282	(-)	NNNNNNNWATGCAAATNNNWNNW	ATAAAACATTTATATATCGATGT
M00135	V$OCT1_01	0.787867	5284	(-)	NNNNWTATGCAAATNTNNN	AAAACATTTATATATCGAT
M00162	V$OCT1_06	0.855469	5286	(+)	CWNAWTKWSATRYN	AACATTTATATATC
M00162	V$OCT1_06	0.855078	5286	(-)	CWNAWTKWSATRYN	AACATTTATATATC
M00162	V$OCT1_06	0.800391	5333	(-)	CWNAWTKWSATRYN	AAAACTTCAAAAAT
M00136	V$OCT1_02	0.780184	5366	(+)	NNGAATATKCANNNN	AAATATATTCTCAAT
M00138	V$OCT1_04	0.781263	5386	(-)	NNNNNNNWATGCAAATNNNWNNW	TCTTATTTTTAACAATATAGATT
M00136	V$OCT1_02	0.835138	5415	(-)	NNGAATATKCANNNN	AAACTGAATATAAGT
M00162	V$OCT1_06	0.847656	5419	(-)	CWNAWTKWSATRYN	TGAATATAAGTTAT
M00138	V$OCT1_04	0.812212	5422	(-)	NNNNNNNWATGCAAATNNNWNNW	ATATAAGTTATTCATATTTTTCA
M00138	V$OCT1_04	0.856336	5423	(+)	NNNNNNNWATGCAAATNNNWNNW	TATAAGTTATTCATATTTTTCAT
M00135	V$OCT1_01	0.749142	5425	(+)	NNNNWTATGCAAATNTNNN	TAAGTTATTCATATTTTTC
M00136	V$OCT1_02	0.78262	5425	(+)	NNGAATATKCANNNN	TAAGTTATTCATATT
M00162	V$OCT1_06	0.821094	5426	(+)	CWNAWTKWSATRYN	AAGTTATTCATATT

M00136	V$OCT1_02	0.784786	5428	(-)	NNGAATATKCANNNN	GTTATTCATATTTTT
M00195	V$OCT1_Q6	0.82833	5428	(-)	NNNNATGCAAATNAN	GTTATTCATATTTTT
M00138	V$OCT1_04	0.814513	5431	(-)	NNNNNNNWATGCAAATNNNWNNW	ATTCATATTTTTCATTTAAGCAA
M00162	V$OCT1_06	0.903516	5435	(+)	CWNAWTKWSATRYN	ATATTTTTCATTTA
M00138	V$OCT1_04	0.819741	5440	(+)	NNNNNNNWATGCAAATNNNWNNW	TTTCATTTAAGCAAATATCACGT
M00135	V$OCT1_01	0.855589	5442	(+)	NNNNWTATGCAAATNTNNN	TCATTTAAGCAAATATCAC
M00162	V$OCT1_06	0.826172	5453	(-)	CWNAWTKWSATRYN	AATATCACGTGTAT
M00138	V$OCT1_04	0.783354	5498	(-)	NNNNNNNWATGCAAATNNNWNNW	TCTTCAACCTTGAATTTTTCTCC
M00162	V$OCT1_06	0.891016	5502	(+)	CWNAWTKWSATRYN	CAACCTTGAATTTT
M00136	V$OCT1_02	0.822144	5504	(-)	NNGAATATKCANNNN	ACCTTGAATTTTTCT
M00137	V$OCT1_03	0.86132	5522	(+)	NNNRTAATNANNN	TAGACAATGATTT
M00137	V$OCT1_03	0.888977	5557	(-)	NNNRTAATNANNN	AGTTCATTATTAC
M00137	V$OCT1_03	0.913473	5560	(-)	NNNRTAATNANNN	TCATTATTACTAT
M00136	V$OCT1_02	0.771251	5565	(+)	NNGAATATKCANNNN	ATTACTATTTAGTTA
M00248	V$OCT1_07	0.800721	5630	(+)	TNTATGNTAATT	ACCATGGAAAAT
M00136	V$OCT1_02	0.803465	5634	(+)	NNGAATATKCANNNN	TGGAAAATGCTTTAT
M00137	V$OCT1_03	0.854998	5644	(+)	NNNRTAATNANNN	TTTATAATATTTT
M00138	V$OCT1_04	0.812212	5646	(-)	NNNNNNNWATGCAAATNNNWNNW	TATAATATTTTACAATACTAAAA
M00138	V$OCT1_04	0.786282	5647	(-)	NNNNNNNWATGCAAATNNNWNNW	ATAATATTTTACAATACTAAAAT
M00162	V$OCT1_06	0.81875	5651	(-)	CWNAWTKWSATRYN	TATTTTACAATACT
M00138	V$OCT1_04	0.809494	5652	(+)	NNNNNNNWATGCAAATNNNWNNW	ATTTTACAATACTAAAATAAAAA
M00138	V$OCT1_04	0.807612	5654	(+)	NNNNNNNWATGCAAATNNNWNNW	TTTACAATACTAAAATAAAAACA
M00162	V$OCT1_06	0.813672	5690	(+)	CWNAWTKWSATRYN	TTGTCTTTAATATA
M00162	V$OCT1_06	0.826562	5698	(-)	CWNAWTKWSATRYN	AATATAAAAGGTAT
M00162	V$OCT1_06	0.821094	5728	(-)	CWNAWTKWSATRYN	AAAATCTAGAATTC
M00138	V$OCT1_04	0.796947	5743	(-)	NNNNNNNWATGCAAATNNNWNNW	TGCTTTTTTTTAACTTTGTGTTT
M00162	V$OCT1_06	0.800391	5754	(+)	CWNAWTKWSATRYN	AACTTTGTGTTTCT
M00137	V$OCT1_03	0.909522	5764	(-)	NNNRTAATNANNN	TTCTTATTACTTC
M00138	V$OCT1_04	0.782518	5872	(-)	NNNNNNNWATGCAAATNNNWNNW	AAGTTTATTCTGCAAAGTTTAAT
M00138	V$OCT1_04	0.794019	5873	(+)	NNNNNNNWATGCAAATNNNWNNW	AGTTTATTCTGCAAAGTTTAATG
M00135	V$OCT1_01	0.800458	5875	(+)	NNNNWTATGCAAATNTNNN	TTTATTCTGCAAAGTTTAA
M00162	V$OCT1_06	0.817578	5884	(+)	CWNAWTKWSATRYN	CAAAGTTTAATGAT
M00137	V$OCT1_03	0.875148	5887	(+)	NNNRTAATNANNN	AGTTTAATGATAT

M00138	V$OCT1_04	0.796529	5890	(+)	NNNNNNNWATGCAAATNNNWNNW	TTAATGATATTAAAATGCCTTTT
M00161	V$OCT1_05	0.857214	5895	(-)	MKNATTTGCATAYY	GATATTAAAATGCC
M00162	V$OCT1_06	0.855469	5895	(+)	CWNAWTKWSATRYN	GATATTAAAATGCC
M00162	V$OCT1_06	0.883203	5895	(-)	CWNAWTKWSATRYN	GATATTAAAATGCC
M00136	V$OCT1_02	0.816188	5898	(+)	NNGAATATKCANNNN	ATTAAAATGCCTTTT
M00162	V$OCT1_06	0.834375	5917	(-)	CWNAWTKWSATRYN	AAAAACTAAGTATT
M00162	V$OCT1_06	0.821484	5925	(-)	CWNAWTKWSATRYN	AGTATTTAAACCCC
M00162	V$OCT1_06	0.842187	5944	(-)	CWNAWTKWSATRYN	ATAATCTAATAATC
M00138	V$OCT1_04	0.795692	5948	(-)	NNNNNNNWATGCAAATNNNWNNW	TCTAATAATCTGTGTAAAAGTCA
M00137	V$OCT1_03	0.848281	5949	(+)	NNNRTAATNANNN	CTAATAATCTGTG
M00162	V$OCT1_06	0.821094	5984	(+)	CWNAWTKWSATRYN	AATCATGTGAATTA
M00195	V$OCT1_Q6	0.805677	5984	(+)	NNNNATGCAAATNAN	AATCATGTGAATTAA
M00248	V$OCT1_07	0.822769	5985	(+)	TNTATGNTAATT	ATCATGTGAATT
M00162	V$OCT1_06	0.821484	5995	(-)	CWNAWTKWSATRYN	TTAATCTCAAAGTT
M00162	V$OCT1_06	0.81875	5997	(-)	CWNAWTKWSATRYN	AATCTCAAAGTTCT
M00137	V$OCT1_03	0.843935	6070	(+)	NNNRTAATNANNN	TTAGCAATAAGAG
M00162	V$OCT1_06	0.860547	6093	(+)	CWNAWTKWSATRYN	CAGAATTTAGTACT
M00162	V$OCT1_06	0.829297	6094	(-)	CWNAWTKWSATRYN	AGAATTTAGTACTG
M00138	V$OCT1_04	0.795692	6097	(+)	NNNNNNNWATGCAAATNNNWNNW	ATTTAGTACTGGAAAAAAATGAA
M00138	V$OCT1_04	0.80092	6107	(+)	NNNNNNNWATGCAAATNNNWNNW	GGAAAAAAATGAAAACAAAAGTA
M00195	V$OCT1_Q6	0.79476	6111	(+)	NNNNATGCAAATNAN	AAAAATGAAAACAAA
M00162	V$OCT1_06	0.816016	6122	(-)	CWNAWTKWSATRYN	CAAAAGTAAAGTTT
M00162	V$OCT1_06	0.804297	6128	(+)	CWNAWTKWSATRYN	TAAAGTTTAATACT
M00162	V$OCT1_06	0.803125	6129	(-)	CWNAWTKWSATRYN	AAAGTTTAATACTT
M00162	V$OCT1_06	0.826562	6177	(+)	CWNAWTKWSATRYN	CATCTCTGAATACT
M00136	V$OCT1_02	0.813752	6179	(-)	NNGAATATKCANNNN	TCTCTGAATACTTCT
M00162	V$OCT1_06	0.839453	6186	(+)	CWNAWTKWSATRYN	ATACTTCTCATACA
M00195	V$OCT1_Q6	0.818504	6186	(-)	NNNNATGCAAATNAN	ATACTTCTCATACAC
M00162	V$OCT1_06	0.828906	6187	(-)	CWNAWTKWSATRYN	TACTTCTCATACAC
M00135	V$OCT1_01	0.734071	6200	(-)	NNNNWTATGCAAATNTNNN	CTTATATTTACTTTCTCCT
M00162	V$OCT1_06	0.834375	6202	(-)	CWNAWTKWSATRYN	TATATTTACTTTCT
M00162	V$OCT1_06	0.805859	6214	(+)	CWNAWTKWSATRYN	CTCCTTTTGTTTCC
M00138	V$OCT1_04	0.809494	6231	(-)	NNNNNNNWATGCAAATNNNWNNW	ACATTCTGTTTGAATGAATACAT

M00138	V$OCT1_04	0.802384	6242	(+)	NNNNNNNWATGCAAATNNNWNNW	GAATGAATACATAAATATGTCTT
M00135	V$OCT1_01	0.732926	6244	(+)	NNNNWTATGCAAATNTNNN	ATGAATACATAAATATGTC
M00162	V$OCT1_06	0.821484	6247	(+)	CWNAWTKWSATRYN	AATACATAAATATG
M00162	V$OCT1_06	0.817187	6247	(-)	CWNAWTKWSATRYN	AATACATAAATATG
M00136	V$OCT1_02	0.785328	6249	(-)	NNGAATATKCANNNN	TACATAAATATGTCT
M00137	V$OCT1_03	0.879099	6249	(+)	NNNRTAATNANNN	TACATAAATATGT
M00138	V$OCT1_04	0.804893	6249	(-)	NNNNNNNWATGCAAATNNNWNNW	TACATAAATATGTCTTCTTTTAT
M00136	V$OCT1_02	0.778831	6252	(+)	NNGAATATKCANNNN	ATAAATATGTCTTCT
M00162	V$OCT1_06	0.847656	6279	(+)	CWNAWTKWSATRYN	AAAAGTGTAATTTT
M00162	V$OCT1_06	0.862891	6280	(-)	CWNAWTKWSATRYN	AAAGTGTAATTTTT
M00137	V$OCT1_03	0.840379	6282	(+)	NNNRTAATNANNN	AGTGTAATTTTTA
M00162	V$OCT1_06	0.839844	6285	(+)	CWNAWTKWSATRYN	GTAATTTTTATTTG
M00195	V$OCT1_Q6	0.835426	6285	(-)	NNNNATGCAAATNAN	GTAATTTTTATTTGC
M00138	V$OCT1_04	0.856754	6287	(-)	NNNNNNNWATGCAAATNNNWNNW	AATTTTTATTTGCAGTGTAATTT
M00195	V$OCT1_Q6	0.791485	6291	(-)	NNNNATGCAAATNAN	TTTATTTGCAGTGTA
M00137	V$OCT1_03	0.865666	6300	(+)	NNNRTAATNANNN	AGTGTAATTTTGT
M00138	V$OCT1_04	0.83898	6317	(+)	NNNNNNNWATGCAAATNNNWNNW	TTGTTTCTATTTATATGGAAAGA
M00138	V$OCT1_04	0.780217	6318	(-)	NNNNNNNWATGCAAATNNNWNNW	TGTTTCTATTTATATGGAAAGAC
M00137	V$OCT1_03	0.857369	6321	(-)	NNNRTAATNANNN	TTCTATTTATATG
M00138	V$OCT1_04	0.792346	6322	(-)	NNNNNNNWATGCAAATNNNWNNW	TCTATTTATATGGAAAGACCTAA
M00138	V$OCT1_04	0.79381	6323	(+)	NNNNNNNWATGCAAATNNNWNNW	CTATTTATATGGAAAGACCTAAT
M00135	V$OCT1_01	0.790729	6325	(+)	NNNNWTATGCAAATNTNNN	ATTTATATGGAAAGACCTA
M00195	V$OCT1_Q6	0.802402	6327	(+)	NNNNATGCAAATNAN	TTATATGGAAAGACC
M00162	V$OCT1_06	0.834375	6328	(-)	CWNAWTKWSATRYN	TATATGGAAAGACC
M00137	V$OCT1_03	0.907151	6338	(+)	NNNRTAATNANNN	GACCTAATGACAT
M00162	V$OCT1_06	0.915234	6340	(+)	CWNAWTKWSATRYN	CCTAATGACATATT
M00162	V$OCT1_06	0.829297	6341	(-)	CWNAWTKWSATRYN	CTAATGACATATTT
M00136	V$OCT1_02	0.780184	6342	(-)	NNGAATATKCANNNN	TAATGACATATTTTT
M00138	V$OCT1_04	0.802802	6343	(+)	NNNNNNNWATGCAAATNNNWNNW	AATGACATATTTTTATAATTGCA
M00138	V$OCT1_04	0.817231	6344	(-)	NNNNNNNWATGCAAATNNNWNNW	ATGACATATTTTTATAATTGCAC
M00135	V$OCT1_01	0.760588	6346	(-)	NNNNWTATGCAAATNTNNN	GACATATTTTTATAATTGC
M00162	V$OCT1_06	0.822656	6348	(+)	CWNAWTKWSATRYN	CATATTTTTATAAT
M00137	V$OCT1_03	0.854208	6349	(-)	NNNRTAATNANNN	ATATTTTTATAAT

M0013<br>7	V$OCT1_03	0.85934<br>4	6355	(-)	NNNRTAATNANNN	TTATAATTGCACA
M0013<br>7	V$OCT1_03	0.90438<br>6	6364	(+)	NNNRTAATNANNN	CACATAATCAATA
M0013<br>8	V$OCT1_04	0.80907<br>6	6375	(+)	NNNNNNNWATGCAAATNNN<br>WNNW	TAGAATGGAGTCAAATAAAAATA
M0013<br>8	V$OCT1_04	0.79297<br>4	6387	(+)	NNNNNNNWATGCAAATNNN<br>WNNW	AAATAAAAATAGAAAATTTCCTT
M0024<br>8	V$OCT1_07	0.83654<br>9	6388	(-)	TNTATGNTAATT	AATAAAAATAGA
M0013<br>5	V$OCT1_01	0.80255<br>6	6397	(-)	NNNNWTATGCAAATNTNNN	AGAAAATTTCCTTACTGAT
M0016<br>2	V$OCT1_06	0.82148<br>4	6399	(-)	CWNAWTKWSATRYN	AAAATTTCCTTACT
M0024<br>8	V$OCT1_07	0.85478<br>1	6401	(-)	TNTATGNTAATT	AATTTCCTTACT
M0016<br>2	V$OCT1_06	0.87382<br>8	6407	(+)	CWNAWTKWSATRYN	CTTACTGATATTTA
M0013<br>8	V$OCT1_04	0.78251<br>8	6410	(-)	NNNNNNNWATGCAAATNNN<br>WNNW	ACTGATATTTAACAGATAAAATT
M0016<br>2	V$OCT1_06	0.80859<br>4	6413	(-)	CWNAWTKWSATRYN	GATATTTAACAGAT
M0024<br>8	V$OCT1_07	0.79245<br>3	6416	(-)	TNTATGNTAATT	ATTTAACAGATA
M0016<br>2	V$OCT1_06	0.81757<br>8	6422	(-)	CWNAWTKWSATRYN	CAGATAAAATTAAG
M0013<br>8	V$OCT1_04	0.80677<br>5	6437	(+)	NNNNNNNWATGCAAATNNN<br>WNNW	TGAAAAAAATCCAAAATATACAT
M0019<br>5	V$OCT1_Q6	0.81086<br>2	6441	(+)	NNNNATGCAAATNAN	AAAAATCCAAAATAT
M0016<br>2	V$OCT1_06	0.88281<br>2	6442	(-)	CWNAWTKWSATRYN	AAAATCCAAAATAT
M0013<br>6	V$OCT1_02	0.7902	6448	(+)	NNGAATATKCANNNN	CAAAATATACATCCA
M0016<br>2	V$OCT1_06	0.86796<br>9	6473	(-)	CWNAWTKWSATRYN	TACATCACTTTATT
M0013<br>8	V$OCT1_04	0.80426<br>6	6479	(-)	NNNNNNNWATGCAAATNNN<br>WNNW	ACTTTATTTTTCTATTCTGATAT
M0013<br>8	V$OCT1_04	0.81095<br>8	6484	(+)	NNNNNNNWATGCAAATNNN<br>WNNW	ATTTTTCTATTCTGATATAATTA
M0013<br>7	V$OCT1_03	0.92335	6497	(+)	NNNRTAATNANNN	GATATAATTAGGT
M0013<br>7	V$OCT1_03	0.87988<br>9	6498	(-)	NNNRTAATNANNN	ATATAATTAGGTT
M0016<br>2	V$OCT1_06	0.83476<br>6	6544	(+)	CWNAWTKWSATRYN	GTAAATAAAATTTG
M0013<br>8	V$OCT1_04	0.78168<br>1	6546	(-)	NNNNNNNWATGCAAATNNN<br>WNNW	AAATAAAATTTGTAAACAATTAC
M0013<br>5	V$OCT1_01	0.73349<br>9	6548	(-)	NNNNWTATGCAAATNTNNN	ATAAAATTTGTAAACAATT
M0013<br>7	V$OCT1_03	0.85736<br>9	6554	(+)	NNNRTAATNANNN	TTTGTAAACAATT
M0013<br>7	V$OCT1_03	0.87159<br>2	6559	(-)	NNNRTAATNANNN	AAACAATTACAAA
M0016<br>2	V$OCT1_06	0.86289<br>1	6560	(+)	CWNAWTKWSATRYN	AACAATTACAAACA
M0013<br>8	V$OCT1_04	0.83312<br>4	6576	(+)	NNNNNNNWATGCAAATNNN<br>WNNW	TGGTGAGAATACATATATATATA
M0013<br>8	V$OCT1_04	0.80928<br>5	6578	(+)	NNNNNNNWATGCAAATNNN<br>WNNW	GTGAGAATACATATATATATACA
M0013<br>8	V$OCT1_04	0.7844	6580	(+)	NNNNNNNWATGCAAATNNN<br>WNNW	GAGAATACATATATATATACATA
M0013<br>8	V$OCT1_04	0.84378<br>9	6581	(-)	NNNNNNNWATGCAAATNNN<br>WNNW	AGAATACATATATATATACATAT
M0013<br>8	V$OCT1_04	0.84901<br>7	6582	(+)	NNNNNNNWATGCAAATNNN<br>WNNW	GAATACATATATATATACATATA

M00138	V$OCT1_04	0.821623	6583	(-)	NNNNNNNWATGCAAATNNNWNNW	AATACATATATATATACATATAA
M00138	V$OCT1_04	0.788373	6584	(+)	NNNNNNNWATGCAAATNNNWNNW	ATACATATATATATACATATAAA
M00138	V$OCT1_04	0.796947	6586	(+)	NNNNNNNWATGCAAATNNNWNNW	ACATATATATATACATATAAATA
M00138	V$OCT1_04	0.876412	6587	(-)	NNNNNNNWATGCAAATNNNWNNW	CATATATATATACATATAAATAT
M00162	V$OCT1_06	0.817187	6587	(+)	CWNAWTKWSATRYN	CATATATATATACA
M00138	V$OCT1_04	0.860937	6588	(+)	NNNNNNNWATGCAAATNNNWNNW	ATATATATATACATATAAATATG
M00135	V$OCT1_01	0.783098	6589	(-)	NNNNWTATGCAAATNTNNN	TATATATATACATATAAAT
M00135	V$OCT1_01	0.732736	6590	(+)	NNNNWTATGCAAATNTNNN	ATATATATACATATAAATA
M00138	V$OCT1_04	0.783563	6590	(+)	NNNNNNNWATGCAAATNNNWNNW	ATATATATACATATAAATATGAA
M00162	V$OCT1_06	0.811719	6591	(+)	CWNAWTKWSATRYN	TATATATACATATA
M00138	V$OCT1_04	0.840234	6592	(+)	NNNNNNNWATGCAAATNNNWNNW	ATATATACATATAAATATGAAAA
M00136	V$OCT1_02	0.797239	6593	(-)	NNGAATATKCANNNN	TATATACATATAAAT
M00138	V$OCT1_04	0.841907	6593	(-)	NNNNNNNWATGCAAATNNNWNNW	TATATACATATAAATATGAAAAA
M00135	V$OCT1_01	0.747425	6594	(+)	NNNNWTATGCAAATNTNNN	ATATACATATAAATATGAA
M00138	V$OCT1_04	0.840862	6594	(+)	NNNNNNNWATGCAAATNNNWNNW	ATATACATATAAATATGAAAAAA
M00195	V$OCT1_Q6	0.798854	6596	(+)	NNNNATGCAAATNAN	ATACATATAAATATG
M00162	V$OCT1_06	0.915234	6597	(-)	CWNAWTKWSATRYN	TACATATAAATATG
M00137	V$OCT1_03	0.853813	6599	(+)	NNNRTAATNANNN	CATATAAATATGA
M00138	V$OCT1_04	0.823505	6599	(-)	NNNNNNNWATGCAAATNNNWNNW	CATATAAATATGAAAAAATATGT
M00138	V$OCT1_04	0.890841	6600	(+)	NNNNNNNWATGCAAATNNNWNNW	ATATAAATATGAAAAAATATGTA
M00135	V$OCT1_01	0.827928	6602	(+)	NNNNWTATGCAAATNTNNN	ATAAATATGAAAAAATATG
M00136	V$OCT1_02	0.809691	6602	(+)	NNGAATATKCANNNN	ATAAATATGAAAAAA
M00162	V$OCT1_06	0.867969	6605	(-)	CWNAWTKWSATRYN	AATATGAAAAAATA
M00138	V$OCT1_04	0.788791	6609	(-)	NNNNNNNWATGCAAATNNNWNNW	TGAAAAAATATGTACGTATTTTT
M00135	V$OCT1_01	0.732736	6612	(+)	NNNNWTATGCAAATNTNNN	AAAAATATGTACGTATTTT
M00136	V$OCT1_02	0.837304	6612	(+)	NNGAATATKCANNNN	AAAAATATGTACGTA
M00162	V$OCT1_06	0.855078	6615	(-)	CWNAWTKWSATRYN	AATATGTACGTATT
M00162	V$OCT1_06	0.821094	6624	(+)	CWNAWTKWSATRYN	GTATTTTTCCTGTG
M00138	V$OCT1_04	0.804893	6644	(+)	NNNNNNNWATGCAAATNNNWNNW	GTATGTTTGTGCAGAGTATGCCA
M00135	V$OCT1_01	0.765357	6655	(+)	NNNNWTATGCAAATNTNNN	CAGAGTATGCCAAAGCTCT
M00136	V$OCT1_02	0.802924	6655	(+)	NNGAATATKCANNNN	CAGAGTATGCCAAAG
M00162	V$OCT1_06	0.877734	6658	(-)	CWNAWTKWSATRYN	AGTATGCCAAAGCT
M00162	V$OCT1_06	0.911719	6672	(+)	CWNAWTKWSATRYN	CTCTATGAGATTTG

M00162	V$OCT1_06	0.813281	6679	(+)	CWNAWTKWSATRYN	AGATTTGTCTTATT
M00162	V$OCT1_06	0.801562	6687	(+)	CWNAWTKWSATRYN	CTTATTGTTGTACT
M00162	V$OCT1_06	0.805859	6696	(+)	CWNAWTKWSATRYN	GTACTTCTAATATG
M00138	V$OCT1_04	0.786909	6698	(-)	NNNNNNNWATGCAAATNNNWNNW	ACTTCTAATATGCAACATTTACT
M00135	V$OCT1_01	0.808661	6701	(+)	NNNNWTATGCAAATNTNNN	TCTAATATGCAACATTTAC
M00136	V$OCT1_02	0.876557	6701	(+)	NNGAATATKCANNNN	TCTAATATGCAACAT
M00162	V$OCT1_06	0.842187	6704	(-)	CWNAWTKWSATRYN	AATATGCAACATTT
M00162	V$OCT1_06	0.808203	6733	(-)	CWNAWTKWSATRYN	TATGTGCCAAGCTC
M00195	V$OCT1_Q6	0.827511	6755	(-)	NNNNATGCAAATNAN	GTGGTTTACATTATG
M00162	V$OCT1_06	0.804297	6756	(-)	CWNAWTKWSATRYN	TGGTTTACATTATG
M00138	V$OCT1_04	0.803848	6758	(-)	NNNNNNNWATGCAAATNNNWNNW	GTTTACATTATGAATTATCTTAC
M00137	V$OCT1_03	0.889767	6759	(-)	NNNRTAATNANNN	TTTACATTATGAA
M00162	V$OCT1_06	0.842187	6763	(+)	CWNAWTKWSATRYN	CATTATGAATTATC
M00136	V$OCT1_02	0.842718	6764	(-)	NNGAATATKCANNNN	ATTATGAATTATCTT
M00138	V$OCT1_04	0.81284	6768	(-)	NNNNNNNWATGCAAATNNNWNNW	TGAATTATCTTACCTAATTTTTT
M00223	V$STAT_01	0.817746	332	(+)	TTCCCRKAA	TTCCTCTTA
M00224	V$STAT1_01	0.729952	558	(+)	NNNSANTTCCGG-GAANTGNSN	GCTAACTTCTCAGAAAGATGA
M00223	V$STAT_01	0.938997	564	(+)	TTCCCRKAA	TTCTCAGAA
M00223	V$STAT_01	0.902445	564	(-)	TTCCCRKAA	TTCTCAGAA
M00223	V$STAT_01	0.796068	657	(+)	TTCCCRKAA	TTACGGTAT
M00223	V$STAT_01	0.832619	657	(-)	TTCCCRKAA	TTACGGTAT
M00223	V$STAT_01	0.801361	733	(+)	TTCCCRKAA	TTTCCATCA
M00223	V$STAT_01	0.834888	800	(+)	TTCCCRKAA	TTATGCTAA
M00223	V$STAT_01	0.819259	800	(-)	TTCCCRKAA	TTATGCTAA
M00223	V$STAT_01	0.805899	1314	(+)	TTCCCRKAA	CTCTTGTAA
M00223	V$STAT_01	0.799597	1454	(-)	TTCCCRKAA	TTCTAAAAA
M00223	V$STAT_01	0.851525	1682	(+)	TTCCCRKAA	TTCCTGTTA
M00223	V$STAT_01	0.832619	1741	(-)	TTCCCRKAA	TTATAGGTA
M00223	V$STAT_01	0.819763	1828	(+)	TTCCCRKAA	TTCTCCAAA
M00223	V$STAT_01	0.932443	2155	(+)	TTCCCRKAA	TTCCTCTAA
M00223	V$STAT_01	0.853542	2188	(+)	TTCCCRKAA	TTCCTACAA
M00223	V$STAT_01	0.832619	2295	(-)	TTCCCRKAA	TTATAGGTA
M00223	V$STAT_01	0.832619	2311	(+)	TTCCCRKAA	TACCTATAA

M0022		0.83261				
3	V$STAT_01	9	2334	(-)	TTCCCRKAA	TTATAGGCA
M0022		0.81673				
3	V$STAT_01	8	2405	(-)	TTCCCRKAA	TTCCAACAA
M0022		0.79253				
3	V$STAT_01	8	2482	(-)	TTCCCRKAA	TTATAAAAA
M0022		0.85858				
3	V$STAT_01	3	2532	(+)	TTCCCRKAA	TTCCTGGTA
M0022						
3	V$STAT_01	0.85934	2675	(+)	TTCCCRKAA	TACCCATAA
M0022		0.79505				
3	V$STAT_01	9	2835	(+)	TTCCCRKAA	ATCCTGGCA
M0022		0.81925				
3	V$STAT_01	9	2985	(+)	TTCCCRKAA	TTTTCATAA
M0022		0.82682				
3	V$STAT_01	1	3055	(-)	TTCCCRKAA	TTTCAAGAA
M0022		0.81547				
3	V$STAT_01	8	3106	(+)	TTCCCRKAA	TTCTCATAT
M0022		0.72912			NNNSANTTCCGG-	
4	V$STAT1_01	9	3126	(+)	GAANTGNSN	TATATTTTTGGGGAAAAACAA
M0022		0.86362				
3	V$STAT_01	5	3132	(-)	TTCCCRKAA	TTTGGGGAA
M0022						
3	V$STAT_01	0.80968	3286	(+)	TTCCCRKAA	TTCTTAAAA
M0022						
3	V$STAT_01	0.88732	3352	(+)	TTCCCRKAA	TTACCGTCA
M0022		0.79606				
3	V$STAT_01	8	3352	(-)	TTCCCRKAA	TTACCGTCA
M0022		0.88026				
3	V$STAT_01	2	3388	(+)	TTCCCRKAA	TTCCCAAAA
M0022		0.80690				
3	V$STAT_01	7	3388	(-)	TTCCCRKAA	TTCCCAAAA
M0022		0.81976				
3	V$STAT_01	3	3524	(+)	TTCCCRKAA	TTCTTTTAA
M0022						
3	V$STAT_01	0.79884	3747	(+)	TTCCCRKAA	ATCTCCTAA
M0022		0.82682				
3	V$STAT_01	1	3841	(+)	TTCCCRKAA	TTCTTTGAA
M0022		0.81295				
3	V$STAT_01	7	3971	(+)	TTCCCRKAA	TTACTGGAG
M0022						
3	V$STAT_01	0.80968	4182	(-)	TTCCCRKAA	TTGTAGTAA
M0022		0.79304				
3	V$STAT_01	3	4335	(+)	TTCCCRKAA	TTCTTCAAA
M0022		0.80262				
3	V$STAT_01	2	4412	(-)	TTCCCRKAA	TTAAGATAA
M0022		0.85051				
3	V$STAT_01	7	4427	(+)	TTCCCRKAA	TTACTAGCA
M0022		0.81547				
3	V$STAT_01	8	4437	(-)	TTCCCRKAA	TTATGAGAT
M0022		0.81547				
3	V$STAT_01	8	4659	(-)	TTCCCRKAA	TTATGGTTA
M0022		0.82682				
3	V$STAT_01	1	4780	(+)	TTCCCRKAA	TTCTTGAAA
M0022		0.79253				
3	V$STAT_01	8	4854	(-)	TTCCCRKAA	TTATATTAA
M0022		0.89513				
3	V$STAT_01	5	4873	(+)	TTCCCRKAA	TTATCAGAA
M0022		0.89538				
3	V$STAT_01	7	4873	(-)	TTCCCRKAA	TTATCAGAA
M0022		0.81976				
3	V$STAT_01	3	4954	(-)	TTCCCRKAA	TTAGAGAAA
M0022		0.79253				
3	V$STAT_01	8	5696	(+)	TTCCCRKAA	TTAATATAA
M0022		0.79052				
3	V$STAT_01	2	5764	(+)	TTCCCRKAA	TTCTTATTA

M00225	V$STAT3_01	0.726575	5768	(-)	NGNNATTTCCSG-GAARTGNNN	TATTACTTCTCTGAATCTCAC
M00223	V$STAT_01	0.853542	5774	(+)	TTCCCRKAA	TTCTCTGAA
M00223	V$STAT_01	0.833627	5774	(-)	TTCCCRKAA	TTCTCTGAA
M00223	V$STAT_01	0.888581	6007	(+)	TTCCCRKAA	TTCTTCTAA
M00223	V$STAT_01	0.799597	6010	(-)	TTCCCRKAA	TTCTAAAAA
M00223	V$STAT_01	0.822536	6089	(+)	TTCCCRKAA	CTCTCAGAA
M00223	V$STAT_01	0.812957	6102	(+)	TTCCCRKAA	GTACTGGAA
M00223	V$STAT_01	0.815478	6190	(+)	TTCCCRKAA	TTCTCATAC
M00225	V$STAT3_01	0.722954	6262	(-)	NGNNATTTCCSG-GAARTGNNN	CTTCTTTTATGAGAAATAAAA
M00223	V$STAT_01	0.858583	6268	(+)	TTCCCRKAA	TTATGAGAA
M00223	V$STAT_01	0.931938	6268	(-)	TTCCCRKAA	TTATGAGAA
M00223	V$STAT_01	0.792538	6352	(+)	TTCCCRKAA	TTTTTATAA
M00224	V$STAT1_01	0.759427	6452	(-)	NNNSANTTCCGG-GAANTGNSN	ATATACATCCAGGAAGTCTCC
M00225	V$STAT3_01	0.736857	6452	(+)	NGNNATTTCCSG-GAARTGNNN	ATATACATCCAGGAAGTCTCC
M00225	V$STAT3_01	0.764374	6452	(-)	NGNNATTTCCSG-GAARTGNNN	ATATACATCCAGGAAGTCTCC
M00223	V$STAT_01	0.856819	6458	(-)	TTCCCRKAA	ATCCAGGAA
CSN1S1						
M00223	V$STAT_01	0.814721	7	(+)	TTCCCRKAA	TTCTTGGGA
M00223	V$STAT_01	0.912276	55	(+)	TTCCCRKAA	TTCTTAGAA
M00223	V$STAT_01	0.833627	55	(-)	TTCCCRKAA	TTCTTAGAA
M00223	V$STAT_01	0.834384	315	(+)	TTCCCRKAA	TTCCTATGA
M00223	V$STAT_01	0.868666	479	(+)	TTCCCRKAA	TTATGGTAA
M00223	V$STAT_01	0.931938	479	(-)	TTCCCRKAA	TTATGGTAA
M00225	V$STAT3_01	0.717161	552	(-)	NGNNATTTCCSGGAARTGNNN	TTTGTTTTCATAGAAATAACA
M00223	V$STAT_01	0.843459	558	(+)	TTCCCRKAA	TTCATAGAA
M00223	V$STAT_01	0.796068	574	(+)	TTCCCRKAA	TTCTGGTAG
M00223	V$STAT_01	0.822536	574	(-)	TTCCCRKAA	TTCTGGTAG
M00223	V$STAT_01	0.802622	614	(-)	TTCCCRKAA	TTAGGTTAA
M00223	V$STAT_01	0.808419	904	(+)	TTCCCRKAA	TTTCCAGCA
M00223	V$STAT_01	0.816738	946	(+)	TTCCCRKAA	TTAATGGAA
M00223	V$STAT_01	0.817746	1229	(-)	TTCCCRKAA	TAAGAGGAA
M00223	V$STAT_01	0.817746	1303	(+)	TTCCCRKAA	TTCCTCTGA
M00223	V$STAT_01	0.819763	1346	(+)	TTCCCRKAA	TTACTTTAA

M00223	V$STAT_01	0.888077	1488	(+)	TTCCCRKAA	TTATCATAA
M00223	V$STAT_01	0.851525	1488	(-)	TTCCCRKAA	TTATCATAA
M00223	V$STAT_01	0.816738	1506	(+)	TTCCCRKAA	TTGTTGGAA
M00223	V$STAT_01	0.861356	1547	(+)	TTCCCRKAA	TTATTATAA
M00223	V$STAT_01	0.815478	1796	(-)	TTCCCRKAA	CTATGGTAA
M00223	V$STAT_01	0.832871	2264	(+)	TTCCCRKAA	TTCCAGGCA
M00223	V$STAT_01	0.856819	2264	(-)	TTCCCRKAA	TTCCAGGCA
M00223	V$STAT_01	0.826821	2499	(+)	TTCCCRKAA	TTCTTCTCA
M00223	V$STAT_01	0.8364	2502	(+)	TTCCCRKAA	TTCTCAAAA
M00224	V$STAT1_01	0.728635	2631	(-)	NNNSANTTCCGGGAANTGNSN	CCCCCATCCCTGGGATTCTCC
M00223	V$STAT_01	0.832871	2791	(+)	TTCCCRKAA	TTCCAGGCA
M00223	V$STAT_01	0.856819	2791	(-)	TTCCCRKAA	TTCCAGGCA
M00223	V$STAT_01	0.8606	2877	(-)	TTCCCRKAA	TGACAAGAA
M00223	V$STAT_01	0.8606	3096	(-)	TTCCCRKAA	TGACAGTAA
M00223	V$STAT_01	0.80968	3106	(-)	TTCCCRKAA	TTACAAAAA
M00223	V$STAT_01	0.851525	3321	(+)	TTCCCRKAA	TTATGATAA
M00223	V$STAT_01	0.888077	3321	(-)	TTCCCRKAA	TTATGATAA
M00223	V$STAT_01	0.799597	3398	(-)	TTCCCRKAA	TTCTAAAAA
M00223	V$STAT_01	0.826317	3603	(+)	TTCCCRKAA	TTAACAGAA
M00223	V$STAT_01	0.809932	3603	(-)	TTCCCRKAA	TTAACAGAA
M00223	V$STAT_01	0.877489	3662	(+)	TTCCCRKAA	TTCCAAGAA
M00223	V$STAT_01	0.929418	3662	(-)	TTCCCRKAA	TTCCAAGAA
M00223	V$STAT_01	0.805899	3672	(+)	TTCCCRKAA	TCACTGTAA
M00223	V$STAT_01	0.826317	3696	(-)	TTCCCRKAA	TGATGATAA
M00223	V$STAT_01	0.795815	3742	(-)	TTCCCRKAA	CTCTAGTAA
M00223	V$STAT_01	0.805899	3925	(+)	TTCCCRKAA	TTACTGTAT
M00223	V$STAT_01	0.805899	4037	(-)	TTCCCRKAA	TTACAAGAG
M00223	V$STAT_01	0.819763	4133	(+)	TTCCCRKAA	TTACCCCAA
M00223	V$STAT_01	0.849761	4530	(-)	TTCCCRKAA	CTACAGGAA
M00224	V$STAT1_01	0.763544	4608	(+)	NNNSANTTCCGGGAANTGNSN	TGTATTTTTCTGGAAAAATCA
M00223	V$STAT_01	0.8606	4614	(+)	TTCCCRKAA	TTTCTGGAA
M00223	V$STAT_01	0.792034	4614	(-)	TTCCCRKAA	TTTCTGGAA
M00223	V$STAT_01	0.81699	4615	(+)	TTCCCRKAA	TTCTGGAAA

M00223	V$STAT_01	0.870179	4615	(-)	TTCCCRKAA	TTCTGGAAA
M00223	V$STAT_01	0.817242	4829	(+)	TTCCCRKAA	TTCTCATTA
M00223	V$STAT_01	0.792538	4990	(+)	TTCCCRKAA	TTAATATAA
M00224	V$STAT1_01	0.732752	5012	(+)	NNNSANTTCCGGGAANTGNSN	TAGCCTTTTCCAGAAGTGTTT
M00224	V$STAT1_01	0.720896	5012	(-)	NNNSANTTCCGGGAANTGNSN	TAGCCTTTTCCAGAAGTGTTT
M00225	V$STAT3_01	0.751774	5012	(+)	NGNNATTTCCSGGAARTGNNN	TAGCCTTTTCCAGAAGTGTTT
M00223	V$STAT_01	0.870179	5018	(+)	TTCCCRKAA	TTTCCAGAA
M00223	V$STAT_01	0.81699	5018	(-)	TTCCCRKAA	TTTCCAGAA
M00223	V$STAT_01	0.929418	5237	(+)	TTCCCRKAA	TTACTGGAA
M00223	V$STAT_01	0.887572	5237	(-)	TTCCCRKAA	TTACTGGAA
M00223	V$STAT_01	0.805899	5321	(-)	TTCCCRKAA	ATACAAGAA
M00223	V$STAT_01	0.800605	5678	(-)	TTCCCRKAA	TAAGGAGAA
M00223	V$STAT_01	0.794807	5799	(-)	TTCCCRKAA	TTGAGGGAA
M00223	V$STAT_01	0.853542	5892	(+)	TTCCCRKAA	TTCATGTAA
M00223	V$STAT_01	0.8243	5943	(-)	TTCCCRKAA	TACTGAGAA
M00223	V$STAT_01	0.792538	6005	(+)	TTCCCRKAA	TTAGTATAA
M00223	V$STAT_01	0.812957	6129	(-)	TTCCCRKAA	CTCCAAGAA
M00223	V$STAT_01	0.867658	6298	(+)	TTCCCRKAA	TTCTTGGCA
M00223	V$STAT_01	0.844719	6342	(+)	TTCCCRKAA	TTATTCTAA
M00224	V$STAT1_01	0.72353	6361	(+)	NNNSANTTCCGGGAANTGNSN	TATAATATAAGGGAAATAAAG
M00225	V$STAT3_01	0.728602	6361	(-)	NGNNATTTCCSGGAARTGNNN	TATAATATAAGGGAAATAAAG
M00223	V$STAT_01	0.826821	6476	(-)	TTCCCRKAA	TTCAAAGAA
M00076	V$GATA2_01	0.807848	40	(-)	NNNGATRNNN	TCCTATGTGA
M00075	V$GATA1_01	0.820829	74	(+)	SNNGATNNNN	CCTGTTGGTT
M00075	V$GATA1_01	0.822804	111	(-)	SNNGATNNNN	ACTAATCAGT
M00076	V$GATA2_01	0.785747	111	(-)	NNNGATRNNN	ACTAATCAGT
M00077	V$GATA3_01	0.824546	111	(-)	NNGATARNG	ACTAATCAG
M00127	V$GATA1_03	0.804508	147	(-)	RNSNNGATAANNGN	TCTGCCATCACCTT
M00075	V$GATA1_01	0.936328	149	(-)	SNNGATNNNN	TGCCATCACC
M00076	V$GATA2_01	0.903022	149	(-)	NNNGATRNNN	TGCCATCACC
M00077	V$GATA3_01	0.85113	149	(-)	NNGATARNG	TGCCATCAC
M00075	V$GATA1_01	0.780355	158	(+)	SNNGATNNNN	CTTGATCATC
M00075	V$GATA1_01	0.798618	158	(-)	SNNGATNNNN	CTTGATCATC

M00076	V$GATA2_01	0.791159	158	(-)	NNNGATRNNN	CTTGATCATC
M00127	V$GATA1_03	0.800833	159	(-)	RNSNNGATAANNGN	TTGATCATCAACCC
M00075	V$GATA1_01	0.827246	161	(-)	SNNGATNNNN	GATCATCAAC
M00127	V$GATA1_03	0.800833	215	(+)	RNSNNGATAANNGN	ATACATATAACAAA
M00075	V$GATA1_01	0.801579	234	(+)	SNNGATNNNN	TATGATTTTC
M00076	V$GATA2_01	0.815065	234	(+)	NNNGATRNNN	TATGATTTTC
M00076	V$GATA2_01	0.795219	249	(-)	NNNGATRNNN	TCTCATCTTT
M00077	V$GATA3_01	0.865751	249	(-)	NNGATARNG	TCTCATCTT
M00075	V$GATA1_01	0.856367	293	(+)	SNNGATNNNN	TGTGATTGCA
M00076	V$GATA2_01	0.852503	293	(+)	NNNGATRNNN	TGTGATTGCA
M00077	V$GATA3_01	0.871068	294	(+)	NNGATARNG	GTGATTGCA
M00076	V$GATA2_01	0.788453	316	(-)	NNNGATRNNN	TCCTATGATA
M00075	V$GATA1_01	0.785785	319	(+)	SNNGATNNNN	TATGATATAC
M00076	V$GATA2_01	0.839423	319	(+)	NNNGATRNNN	TATGATATAC
M00126	V$GATA1_02	0.8225	354	(+)	NNNNNGATANKGNN	ATGTTGATACTATC
M00127	V$GATA1_03	0.856688	354	(+)	RNSNNGATAANNGN	ATGTTGATACTATC
M00075	V$GATA1_01	0.846002	356	(+)	SNNGATNNNN	GTTGATACTA
M00076	V$GATA2_01	0.797474	356	(+)	NNNGATRNNN	GTTGATACTA
M00126	V$GATA1_02	0.844375	359	(-)	NNNNNGATANKGNN	GATACTATCTATCT
M00127	V$GATA1_03	0.868447	359	(-)	RNSNNGATAANNGN	GATACTATCTATCT
M00075	V$GATA1_01	0.786278	361	(-)	SNNGATNNNN	TACTATCTAT
M00076	V$GATA2_01	0.7871	361	(-)	NNNGATRNNN	TACTATCTAT
M00077	V$GATA3_01	0.902525	361	(-)	NNGATARNG	TACTATCTA
M00203	V$GATA_C	0.853992	362	(-)	NGATAAGNMNN	ACTATCTATCT
M00126	V$GATA1_02	0.770937	363	(-)	NNNNNGATANKGNN	CTATCTATCTCAGA
M00128	V$GATA1_04	0.837316	363	(-)	NNCWGATARNNNN	CTATCTATCTCAG
M00075	V$GATA1_01	0.85538	365	(-)	SNNGATNNNN	ATCTATCTCA
M00076	V$GATA2_01	0.898963	365	(-)	NNNGATRNNN	ATCTATCTCA
M00077	V$GATA3_01	0.892335	365	(-)	NNGATARNG	ATCTATCTC
M00126	V$GATA1_02	0.779375	452	(+)	NNNNNGATANKGNN	ATGTAGATACATAA
M00127	V$GATA1_03	0.841009	452	(+)	RNSNNGATAANNGN	ATGTAGATACATAA
M00075	V$GATA1_01	0.821323	454	(+)	SNNGATNNNN	GTAGATACAT
M00076	V$GATA2_01	0.798376	454	(+)	NNNGATRNNN	GTAGATACAT

M00203	V$GATA_C	0.877912	456	(+)	NGATAAGNMNN	AGATACATAAC
M00126	V$GATA1_02	0.818438	648	(+)	NNNNNGATANKGNN	TCAATGATAGACAA
M00075	V$GATA1_01	0.824284	650	(+)	SNNGATNNNN	AATGATAGAC
M00076	V$GATA2_01	0.843482	650	(+)	NNNGATRNNN	AATGATAGAC
M00077	V$GATA3_01	0.851573	651	(+)	NNGATARNG	ATGATAGAC
M00203	V$GATA_C	0.884747	652	(+)	NGATAAGNMNN	TGATAGACAAT
M00128	V$GATA1_04	0.841605	661	(+)	NNCWGATARNNNN	ATTAGATATAAAT
M00077	V$GATA3_01	0.822331	663	(+)	NNGATARNG	TAGATATAA
M00203	V$GATA_C	0.835042	664	(+)	NGATAAGNMNN	AGATATAAATG
M00127	V$GATA1_03	0.784664	684	(+)	RNSNNGATAANNGN	ATAAAGATGATTAA
M00075	V$GATA1_01	0.792695	686	(+)	SNNGATNNNN	AAAGATGATT
M00076	V$GATA2_01	0.818674	686	(+)	NNNGATRNNN	AAAGATGATT
M00075	V$GATA1_01	0.779368	689	(+)	SNNGATNNNN	GATGATTAAA
M00077	V$GATA3_01	0.821444	690	(+)	NNGATARNG	ATGATTAAA
M00075	V$GATA1_01	0.778875	700	(+)	SNNGATNNNN	TTGGATATTT
M00076	V$GATA2_01	0.827244	700	(+)	NNNGATRNNN	TTGGATATTT
M00126	V$GATA1_02	0.782188	710	(+)	NNNNNGATANKGNN	GTAAGGATACAAAT
M00127	V$GATA1_03	0.807202	710	(+)	RNSNNGATAANNGN	GTAAGGATACAAAT
M00076	V$GATA2_01	0.817772	712	(+)	NNNGATRNNN	AAGGATACAA
M00203	V$GATA_C	0.864554	714	(+)	NGATAAGNMNN	GGATACAAATA
M00075	V$GATA1_01	0.801579	782	(+)	SNNGATNNNN	GTAGATTCTA
M00075	V$GATA1_01	0.789733	821	(-)	SNNGATNNNN	AATAATCTGT
M00076	V$GATA2_01	0.790708	821	(-)	NNNGATRNNN	AATAATCTGT
M00077	V$GATA3_01	0.827204	821	(-)	NNGATARNG	AATAATCTG
M00077	V$GATA3_01	0.840496	868	(-)	NNGATARNG	TATAATCTA
M00127	V$GATA1_03	0.794463	880	(-)	RNSNNGATAANNGN	TTTCTTATGTATCT
M00203	V$GATA_C	0.851196	883	(-)	NGATAAGNMNN	CTTATGTATCT
M00126	V$GATA1_02	0.773125	884	(-)	NNNNNGATANKGNN	TTATGTATCTGAAA
M00128	V$GATA1_04	0.839767	884	(-)	NNCWGATARNNNN	TTATGTATCTGAA
M00075	V$GATA1_01	0.821816	886	(-)	SNNGATNNNN	ATGTATCTGA
M00076	V$GATA2_01	0.873703	886	(-)	NNNGATRNNN	ATGTATCTGA
M00126	V$GATA1_02	0.785938	967	(+)	NNNNNGATANKGNN	AACGTGATATGTTT
M00128	V$GATA1_04	0.834865	968	(+)	NNCWGATARNNNN	ACGTGATATGTTT

M00075	V$GATA1_01	0.941757	969	(+)	SNNGATNNNN	CGTGATATGT
M00076	V$GATA2_01	0.906631	969	(+)	NNNGATRNNN	CGTGATATGT
M00077	V$GATA3_01	0.849358	970	(+)	NNGATARNG	GTGATATGT
M00075	V$GATA1_01	0.848963	985	(+)	SNNGATNNNN	TATGATTCTG
M00076	V$GATA2_01	0.843031	985	(+)	NNNGATRNNN	TATGATTCTG
M00077	V$GATA3_01	0.825432	986	(+)	NNGATARNG	ATGATTCTG
M00075	V$GATA1_01	0.779368	995	(-)	SNNGATNNNN	TTTAATCATC
M00077	V$GATA3_01	0.821444	995	(-)	NNGATARNG	TTTAATCAT
M00127	V$GATA1_03	0.817491	996	(-)	RNSNNGATAANNGN	TTAATCATCTTCAT
M00075	V$GATA1_01	0.857354	998	(-)	SNNGATNNNN	AATCATCTTC
M00076	V$GATA2_01	0.865584	998	(-)	NNNGATRNNN	AATCATCTTC
M00203	V$GATA_C	0.878844	1022	(-)	NGATAAGNMNN	TGGATATATCA
M00128	V$GATA1_04	0.810355	1023	(-)	NNCWGATARNNNN	GGATATATCAACC
M00203	V$GATA_C	0.862069	1023	(+)	NGATAAGNMNN	GGATATATCAA
M00075	V$GATA1_01	0.820829	1025	(-)	SNNGATNNNN	ATATATCAAC
M00075	V$GATA1_01	0.813919	1055	(+)	SNNGATNNNN	CTAGATCCTT
M00076	V$GATA2_01	0.784844	1055	(-)	NNNGATRNNN	CTAGATCCTT
M00126	V$GATA1_02	0.778125	1091	(+)	NNNNNGATANKGNN	TTCTACATAATGCA
M00127	V$GATA1_03	0.805488	1132	(+)	RNSNNGATAANNGN	AGCAGGCTAAAGGA
M00126	V$GATA1_02	0.79375	1139	(+)	NNNNNGATANKGNN	TAAAGGATAAAGAC
M00128	V$GATA1_04	0.86489	1140	(+)	NNCWGATARNNNN	AAAGGATAAAGAC
M00076	V$GATA2_01	0.834461	1141	(+)	NNNGATRNNN	AAGGATAAAG
M00077	V$GATA3_01	0.868409	1142	(+)	NNGATARNG	AGGATAAAG
M00203	V$GATA_C	0.948742	1143	(+)	NGATAAGNMNN	GGATAAAGACA
M00126	V$GATA1_02	0.828125	1164	(+)	NNNNNGATANKGNN	TTACAGATATTAAA
M00128	V$GATA1_04	0.844363	1165	(+)	NNCWGATARNNNN	TACAGATATTAAA
M00075	V$GATA1_01	0.79615	1166	(+)	SNNGATNNNN	ACAGATATTA
M00076	V$GATA2_01	0.840325	1166	(+)	NNNGATRNNN	ACAGATATTA
M00077	V$GATA3_01	0.860877	1167	(+)	NNGATARNG	CAGATATTA
M00203	V$GATA_C	0.882572	1197	(-)	NGATAAGNMNN	TTAGTTTATCA
M00126	V$GATA1_02	0.8125	1198	(-)	NNNNNGATANKGNN	TAGTTTATCAATTT
M00127	V$GATA1_03	0.821901	1198	(-)	RNSNNGATAANNGN	TAGTTTATCAATTT
M00128	V$GATA1_04	0.815564	1198	(-)	NNCWGATARNNNN	TAGTTTATCAATT

M00075	V$GATA1_01	0.816881	1288	(+)	SNNGATNNNN	ATTGATGCTT
M00203	V$GATA_C	0.858652	1309	(+)	NGATAAGNMNN	TGACAAAACCC
M00127	V$GATA1_03	0.78099	1318	(-)	RNSNNGATAANNGN	CCTACTATTACTTT
M00075	V$GATA1_01	0.785291	1332	(-)	SNNGATNNNN	CAGGATCAAA
M00126	V$GATA1_02	0.810312	1360	(+)	NNNNNGATANKGNN	AGTAAGATATTGGT
M00128	V$GATA1_04	0.860294	1361	(+)	NNCWGATARNNNN	GTAAGATATTGGT
M00075	V$GATA1_01	0.830701	1362	(+)	SNNGATNNNN	TAAGATATTG
M00076	V$GATA2_01	0.907533	1362	(+)	NNNGATRNNN	TAAGATATTG
M00077	V$GATA3_01	0.901639	1363	(+)	NNGATARNG	AAGATATTG
M00203	V$GATA_C	0.974216	1374	(-)	NGATAAGNMNN	ATTTCTTATCT
M00126	V$GATA1_02	0.84875	1375	(-)	NNNNNGATANKGNN	TTTCTTATCTTATA
M00128	V$GATA1_04	0.945159	1375	(-)	NNCWGATARNNNN	TTTCTTATCTTAT
M00075	V$GATA1_01	0.777887	1377	(-)	SNNGATNNNN	TCTTATCTTA
M00076	V$GATA2_01	0.856112	1377	(-)	NNNGATRNNN	TCTTATCTTA
M00077	V$GATA3_01	0.919362	1377	(-)	NNGATARNG	TCTTATCTT
M00203	V$GATA_C	0.835353	1386	(+)	NGATAAGNMNN	ATATAAGCACT
M00127	V$GATA1_03	0.801813	1398	(+)	RNSNNGATAANNGN	AGCAAAATAATTTG
M00203	V$GATA_C	0.906803	1483	(-)	NGATAAGNMNN	AACTTTTATCA
M00126	V$GATA1_02	0.821875	1484	(-)	NNNNNGATANKGNN	ACTTTTATCATAAT
M00127	V$GATA1_03	0.825821	1484	(-)	RNSNNGATAANNGN	ACTTTTATCATAAT
M00128	V$GATA1_04	0.915135	1484	(-)	NNCWGATARNNNN	ACTTTTATCATAA
M00076	V$GATA2_01	0.798376	1486	(-)	NNNGATRNNN	TTTTATCATA
M00077	V$GATA3_01	0.855117	1486	(-)	NNGATARNG	TTTTATCAT
M00203	V$GATA_C	0.838459	1550	(+)	NGATAAGNMNN	TTATAAGACCT
M00075	V$GATA1_01	0.78924	1580	(-)	SNNGATNNNN	ATAGATCTTG
M00076	V$GATA2_01	0.798827	1580	(-)	NNNGATRNNN	ATAGATCTTG
M00127	V$GATA1_03	0.797893	1584	(+)	RNSNNGATAANNGN	ATCTTGACAACCAT
M00127	V$GATA1_03	0.799363	1599	(-)	RNSNNGATAANNGN	AAACTTCTCATCCT
M00127	V$GATA1_03	0.790544	1602	(-)	RNSNNGATAANNGN	CTTCTCATCCTTAC
M00076	V$GATA2_01	0.809653	1604	(-)	NNNGATRNNN	TCTCATCCTT
M00077	V$GATA3_01	0.820558	1604	(-)	NNGATARNG	TCTCATCCT
M00127	V$GATA1_03	0.810632	1608	(-)	RNSNNGATAANNGN	ATCCTTACCTGTCT
M00128	V$GATA1_04	0.830882	1608	(-)	NNCWGATARNNNN	ATCCTTACCTGTC

M00076	V$GATA2_01	0.786649	1614	(-)	NNNGATRNNN	ACCTGTCTTG
M00075	V$GATA1_01	0.775913	1626	(+)	SNNGATNNNN	GCTGTTGCTC
M00075	V$GATA1_01	0.837611	1668	(+)	SNNGATNNNN	GAAGATTCTA
M00076	V$GATA2_01	0.825891	1668	(+)	NNNGATRNNN	GAAGATTCTA
M00075	V$GATA1_01	0.84156	1675	(+)	SNNGATNNNN	CTAGATTCTT
M00075	V$GATA1_01	0.829714	1691	(-)	SNNGATNNNN	AGTCATCTCA
M00076	V$GATA2_01	0.878214	1691	(-)	NNNGATRNNN	AGTCATCTCA
M00075	V$GATA1_01	0.823297	1719	(-)	SNNGATNNNN	TCTCATCAAG
M00126	V$GATA1_02	0.83	1729	(+)	NNNNNGATANKGNN	TGCAAGATATTGTA
M00075	V$GATA1_01	0.891412	1731	(+)	SNNGATNNNN	CAAGATATTG
M00076	V$GATA2_01	0.916554	1731	(+)	NNNGATRNNN	CAAGATATTG
M00077	V$GATA3_01	0.901639	1732	(+)	NNGATARNG	AAGATATTG
M00075	V$GATA1_01	0.83613	1754	(+)	SNNGATNNNN	TTTGATGGTC
M00076	V$GATA2_01	0.806044	1754	(+)	NNNGATRNNN	TTTGATGGTC
M00126	V$GATA1_02	0.800625	1781	(+)	NNNNNGATANKGNN	GTGTTGATATTAAA
M00127	V$GATA1_03	0.793974	1781	(+)	RNSNNGATAANNGN	GTGTTGATATTAAA
M00075	V$GATA1_01	0.824778	1783	(+)	SNNGATNNNN	GTTGATATTA
M00076	V$GATA2_01	0.786198	1783	(+)	NNNGATRNNN	GTTGATATTA
M00076	V$GATA2_01	0.838972	1794	(-)	NNNGATRNNN	AGCTATGGTA
M00076	V$GATA2_01	0.812359	1800	(-)	NNNGATRNNN	GGTAATCCTT
M00203	V$GATA_C	0.834421	1842	(-)	NGATAAGNMNN	AAGACTTAACT
M00126	V$GATA1_02	0.775938	1910	(+)	NNNNNGATANKGNN	AAAGAAATAAGGTA
M00075	V$GATA1_01	0.810464	1941	(-)	SNNGATNNNN	GAGCATCAAA
M00076	V$GATA2_01	0.780785	1941	(-)	NNNGATRNNN	GAGCATCAAA
M00127	V$GATA1_03	0.824351	1949	(+)	RNSNNGATAANNGN	AAGAAGGTAACTAG
M00076	V$GATA2_01	0.786649	1990	(+)	NNNGATRNNN	TAACATATTT
M00203	V$GATA_C	0.854303	2007	(-)	NGATAAGNMNN	TATATATATCC
M00076	V$GATA2_01	0.7871	2010	(-)	NNNGATRNNN	ATATATCCAA
M00075	V$GATA1_01	0.7769	2041	(+)	SNNGATNNNN	GCTGCTGCTG
M00075	V$GATA1_01	0.807009	2089	(+)	SNNGATNNNN	ATTGATGGCA
M00075	V$GATA1_01	0.846496	2112	(-)	SNNGATNNNN	TGCCATCCCT
M00076	V$GATA2_01	0.881822	2112	(-)	NNNGATRNNN	TGCCATCCCT
M00077	V$GATA3_01	0.82942	2112	(-)	NNGATARNG	TGCCATCCC

M00075	V$GATA1_01	0.826259	2121	(+)	SNNGATNNNN	TGGGATTCTC
M00076	V$GATA2_01	0.860171	2121	(+)	NNNGATRNNN	TGGGATTCTC
M00075	V$GATA1_01	0.786278	2174	(+)	SNNGATNNNN	CATGAAAGTG
M00127	V$GATA1_03	0.785889	2178	(+)	RNSNNGATAANNGN	AAAGTGAAAAGTGA
M00127	V$GATA1_03	0.797648	2217	(-)	RNSNNGATAANNGN	ACTCCTAGCAACCC
M00075	V$GATA1_01	0.78924	2248	(-)	SNNGATNNNN	CTCCATCCAT
M00075	V$GATA1_01	0.805035	2257	(+)	SNNGATNNNN	TGGGATTTTC
M00076	V$GATA2_01	0.848895	2257	(+)	NNNGATRNNN	TGGGATTTTC
M00127	V$GATA1_03	0.822391	2335	(-)	RNSNNGATAANNGN	CAGTGTATCCACTT
M00075	V$GATA1_01	0.802073	2337	(-)	SNNGATNNNN	GTGTATCCAC
M00076	V$GATA2_01	0.809202	2337	(-)	NNNGATRNNN	GTGTATCCAC
M00075	V$GATA1_01	0.894867	2374	(+)	SNNGATNNNN	TGAGATGGCT
M00076	V$GATA2_01	0.929635	2374	(+)	NNNGATRNNN	TGAGATGGCT
M00077	V$GATA3_01	0.842712	2375	(+)	NNGATARNG	GAGATGGCT
M00127	V$GATA1_03	0.78099	2407	(+)	RNSNNGATAANNGN	GTACAGAAAGTTGG
M00075	V$GATA1_01	0.839585	2430	(+)	SNNGATNNNN	GCTGATTCAA
M00076	V$GATA2_01	0.782589	2430	(+)	NNNGATRNNN	GCTGATTCAA
M00126	V$GATA1_02	0.8875	2472	(+)	NNNNNGATANKGNN	AACTAGATAATGTA
M00127	V$GATA1_03	0.865997	2472	(+)	RNSNNGATAANNGN	AACTAGATAATGTA
M00128	V$GATA1_04	0.827819	2473	(+)	NNCWGATARNNNN	ACTAGATAATGTA
M00075	V$GATA1_01	0.837117	2474	(+)	SNNGATNNNN	CTAGATAATG
M00076	V$GATA2_01	0.820027	2474	(+)	NNNGATRNNN	CTAGATAATG
M00077	V$GATA3_01	0.909171	2475	(+)	NNGATARNG	TAGATAATG
M00203	V$GATA_C	0.863312	2476	(+)	NGATAAGNMNN	AGATAATGTAA
M00076	V$GATA2_01	0.793415	2605	(+)	NNNGATRNNN	CCCCATAGAC
M00075	V$GATA1_01	0.905726	2632	(-)	SNNGATNNNN	CCCCATCCCT
M00076	V$GATA2_01	0.912043	2632	(-)	NNNGATRNNN	CCCCATCCCT
M00077	V$GATA3_01	0.901196	2632	(-)	NNGATARNG	CCCCATCCC
M00075	V$GATA1_01	0.826259	2641	(+)	SNNGATNNNN	TGGGATTCTC
M00076	V$GATA2_01	0.860171	2641	(+)	NNNGATRNNN	TGGGATTCTC
M00075	V$GATA1_01	0.786278	2694	(+)	SNNGATNNNN	CATGAAAGTG
M00126	V$GATA1_02	0.785938	2698	(+)	NNNNNGATANKGNN	AAAGTGAGAAGGGA
M00127	V$GATA1_03	0.795198	2698	(+)	RNSNNGATAANNGN	AAAGTGAGAAGGGA

M00077	V$GATA3_01	0.824103	2701	(+)	NNGATARNG	GTGAGAAGG
M00075	V$GATA1_01	0.805035	2784	(+)	SNNGATNNNN	TGGGATTTTC
M00076	V$GATA2_01	0.848895	2784	(+)	NNNGATRNNN	TGGGATTTTC
M00126	V$GATA1_02	0.881563	2841	(+)	NNNNNGATANKGNN	CTCTAGATAATGAT
M00127	V$GATA1_03	0.874326	2841	(+)	RNSNNGATAANNGN	CTCTAGATAATGAT
M00128	V$GATA1_04	0.865502	2842	(+)	NNCWGATARNNNN	TCTAGATAATGAT
M00075	V$GATA1_01	0.837117	2843	(+)	SNNGATNNNN	CTAGATAATG
M00076	V$GATA2_01	0.820027	2843	(+)	NNNGATRNNN	CTAGATAATG
M00077	V$GATA3_01	0.909171	2844	(+)	NNGATARNG	TAGATAATG
M00203	V$GATA_C	0.899348	2845	(+)	NGATAAGNMNN	AGATAATGATA
M00126	V$GATA1_02	0.794687	2847	(+)	NNNNNGATANKGNN	ATAATGATAAATAA
M00127	V$GATA1_03	0.863057	2847	(+)	RNSNNGATAANNGN	ATAATGATAAATAA
M00128	V$GATA1_04	0.884498	2848	(+)	NNCWGATARNNNN	TAATGATAAATAA
M00075	V$GATA1_01	0.773939	2849	(+)	SNNGATNNNN	AATGATAAAT
M00076	V$GATA2_01	0.79567	2849	(+)	NNNGATRNNN	AATGATAAAT
M00077	V$GATA3_01	0.823217	2850	(+)	NNGATARNG	ATGATAAAT
M00203	V$GATA_C	0.932277	2851	(+)	NGATAAGNMNN	TGATAAATAAA
M00075	V$GATA1_01	0.857848	2867	(-)	SNNGATNNNN	AGGAATCAAC
M00076	V$GATA2_01	0.781687	2867	(-)	NNNGATRNNN	AGGAATCAAC
M00203	V$GATA_C	0.839391	2877	(+)	NGATAAGNMNN	TGACAAGAAAG
M00075	V$GATA1_01	0.782823	2886	(+)	SNNGATNNNN	AGTGATTCAA
M00126	V$GATA1_02	0.857813	2895	(+)	NNNNNGATANKGNN	AATAAGATAATAGT
M00127	V$GATA1_03	0.844439	2895	(+)	RNSNNGATAANNGN	AATAAGATAATAGT
M00128	V$GATA1_04	0.912684	2896	(+)	NNCWGATARNNNN	ATAAGATAATAGT
M00076	V$GATA2_01	0.849797	2897	(+)	NNNGATRNNN	TAAGATAATA
M00077	V$GATA3_01	0.898538	2898	(+)	NNGATARNG	AAGATAATA
M00203	V$GATA_C	0.871389	2899	(+)	NGATAAGNMNN	AGATAATAGTT
M00075	V$GATA1_01	0.778875	2910	(+)	SNNGATNNNN	TTGGATATTT
M00076	V$GATA2_01	0.827244	2910	(+)	NNNGATRNNN	TTGGATATTT
M00126	V$GATA1_02	0.8125	2927	(-)	NNNNNGATANKGNN	CAAACTATCAAATA
M00128	V$GATA1_04	0.811887	2927	(-)	NNCWGATARNNNN	CAAACTATCAAAT
M00075	V$GATA1_01	0.850938	2929	(-)	SNNGATNNNN	AACTATCAAA
M00076	V$GATA2_01	0.838069	2929	(-)	NNNGATRNNN	AACTATCAAA

M00077	V$GATA3_01	0.847142	2929	(-)	NNGATARNG	AACTATCAA
M00126	V$GATA1_02	0.789062	2963	(+)	NNNNNGATANKGNN	GCTGAGATATTCTA
M00075	V$GATA1_01	0.833169	2965	(+)	SNNGATNNNNN	TGAGATATTC
M00076	V$GATA2_01	0.898963	2965	(+)	NNNGATRNNN	TGAGATATTC
M00077	V$GATA3_01	0.852016	2966	(+)	NNGATARNG	GAGATATTC
M00126	V$GATA1_02	0.772813	2984	(-)	NNNNNGATANKGNN	ACTCTTATTTTCTA
M00128	V$GATA1_04	0.810355	2984	(-)	NNCWGATARNNNN	ACTCTTATTTTCT
M00075	V$GATA1_01	0.779862	3008	(+)	SNNGATNNNNN	AATGATTGAA
M00077	V$GATA3_01	0.849801	3009	(+)	NNGATARNG	ATGATTGAA
M00075	V$GATA1_01	0.830207	3016	(-)	SNNGATNNNNN	AAGGATCACT
M00076	V$GATA2_01	0.815968	3016	(+)	NNNGATRNNN	AAGGATCACT
M00076	V$GATA2_01	0.805593	3016	(-)	NNNGATRNNN	AAGGATCACT
M00127	V$GATA1_03	0.800833	3024	(-)	RNSNNGATAANNGN	CTAATAATCCAGCT
M00075	V$GATA1_01	0.797631	3026	(-)	SNNGATNNNNN	AATAATCCAG
M00075	V$GATA1_01	0.825271	3073	(-)	SNNGATNNNNN	AAGCATCAAA
M00076	V$GATA2_01	0.784393	3073	(-)	NNNGATRNNN	AAGCATCAAA
M00075	V$GATA1_01	0.782823	3081	(+)	SNNGATNNNNN	AAAGATGGAA
M00076	V$GATA2_01	0.80018	3081	(+)	NNNGATRNNN	AAAGATGGAA
M00077	V$GATA3_01	0.853345	3082	(+)	NNGATARNG	AAGATGGAA
M00203	V$GATA_C	0.84343	3086	(-)	NGATAAGNMNN	TGGAAATATCT
M00128	V$GATA1_04	0.863664	3087	(-)	NNCWGATARNNNN	GGAAATATCTGAC
M00075	V$GATA1_01	0.840079	3089	(-)	SNNGATNNNNN	AAATATCTGA
M00076	V$GATA2_01	0.902571	3089	(-)	NNNGATRNNN	AAATATCTGA
M00077	V$GATA3_01	0.828977	3089	(-)	NNGATARNG	AAATATCTG
M00203	V$GATA_C	0.922647	3151	(-)	NGATAAGNMNN	AGTCCATATCT
M00126	V$GATA1_02	0.861875	3152	(-)	NNNNNGATANKGNN	GTCCATATCTTTTC
M00127	V$GATA1_03	0.793974	3152	(-)	RNSNNGATAANNGN	GTCCATATCTTTTC
M00128	V$GATA1_04	0.904105	3152	(-)	NNCWGATARNNNN	GTCCATATCTTTT
M00075	V$GATA1_01	0.83465	3154	(-)	SNNGATNNNNN	CCATATCTTT
M00076	V$GATA2_01	0.881371	3154	(-)	NNNGATRNNN	CCATATCTTT
M00077	V$GATA3_01	0.922463	3154	(-)	NNGATARNG	CCATATCTT
M00203	V$GATA_C	0.850885	3164	(-)	NGATAAGNMNN	TCCTTTTATCA
M00126	V$GATA1_02	0.844063	3165	(-)	NNNNNGATANKGNN	CCTTTTATCAGTGA

M00127	V$GATA1_03	0.792994	3165	(-)	RNSNNGATAANNGN	CCTTTTATCAGTGA
M00128	V$GATA1_04	0.870098	3165	(-)	NNCWGATARNNNN	CCTTTTATCAGTG
M00077	V$GATA3_01	0.849358	3167	(-)	NNGATARNG	TTTTATCAG
M00126	V$GATA1_02	0.79125	3178	(+)	NNNNNGATANKGNN	AAACAGATATAGAT
M00128	V$GATA1_04	0.90288	3179	(+)	NNCWGATARNNNN	AACAGATATAGAT
M00075	V$GATA1_01	0.803554	3180	(+)	SNNGATNNNN	ACAGATATAG
M00076	V$GATA2_01	0.843031	3180	(+)	NNNGATRNNN	ACAGATATAG
M00077	V$GATA3_01	0.875942	3181	(+)	NNGATARNG	CAGATATAG
M00203	V$GATA_C	0.868593	3182	(+)	NGATAAGNMNN	AGATATAGATC
M00075	V$GATA1_01	0.808983	3186	(-)	SNNGATNNNN	ATAGATCCCC
M00076	V$GATA2_01	0.826793	3186	(-)	NNNGATRNNN	ATAGATCCCC
M00075	V$GATA1_01	0.82922	3202	(+)	SNNGATNNNN	ACAGATTCTT
M00076	V$GATA2_01	0.81687	3202	(+)	NNNGATRNNN	ACAGATTCTT
M00075	V$GATA1_01	0.831688	3229	(-)	SNNGATNNNN	AAACATCATT
M00076	V$GATA2_01	0.822282	3229	(-)	NNNGATRNNN	AAACATCATT
M00075	V$GATA1_01	0.816881	3263	(-)	SNNGATNNNN	ATAAATCTTG
M00076	V$GATA2_01	0.798827	3263	(-)	NNNGATRNNN	ATAAATCTTG
M00075	V$GATA1_01	0.891412	3289	(+)	SNNGATNNNN	GCAGATTGAG
M00076	V$GATA2_01	0.861976	3289	(+)	NNNGATRNNN	GCAGATTGAG
M00077	V$GATA3_01	0.902525	3290	(+)	NNGATARNG	CAGATTGAG
M00126	V$GATA1_02	0.794375	3320	(+)	NNNNNGATANKGNN	TTTATGATAAAAAA
M00128	V$GATA1_04	0.901654	3321	(+)	NNCWGATARNNNN	TTATGATAAAAAA
M00076	V$GATA2_01	0.798376	3322	(+)	NNNGATRNNN	TATGATAAAA
M00077	V$GATA3_01	0.855117	3323	(+)	NNGATARNG	ATGATAAAA
M00203	V$GATA_C	0.936626	3324	(+)	NGATAAGNMNN	TGATAAAAAAT
M00075	V$GATA1_01	0.780355	3334	(-)	SNNGATNNNN	TAAAATCCAG
M00127	V$GATA1_03	0.808182	3338	(+)	RNSNNGATAANNGN	ATCCAGACAAACAG
M00075	V$GATA1_01	0.798124	3356	(+)	SNNGATNNNN	TCAGATTATT
M00076	V$GATA2_01	0.823184	3356	(+)	NNNGATRNNN	TCAGATTATT
M00077	V$GATA3_01	0.827204	3357	(+)	NNGATARNG	CAGATTATT
M00203	V$GATA_C	0.861758	3363	(-)	NGATAAGNMNN	ATTTTTTGTCT
M00127	V$GATA1_03	0.785644	3372	(-)	RNSNNGATAANNGN	CTTTTTATATACTT
M00127	V$GATA1_03	0.807692	3380	(-)	RNSNNGATAANNGN	ATACTTTTCTCCAC

M00076	V$GATA2_01	0.788904	3391	(+)	NNNGATRNNN	CACCATATTC
M00126	V$GATA1_02	0.83	3407	(+)	NNNNNGATANKGNN	CAGAAGATAATTTA
M00127	V$GATA1_03	0.856688	3407	(+)	RNSNNGATAANNGN	CAGAAGATAATTTA
M00128	V$GATA1_04	0.849265	3408	(+)	NNCWGATARNNNNN	AGAAGATAATTTA
M00075	V$GATA1_01	0.857354	3409	(+)	SNNGATNNNN	GAAGATAATT
M00076	V$GATA2_01	0.894001	3409	(+)	NNNGATRNNN	GAAGATAATT
M00077	V$GATA3_01	0.866637	3410	(+)	NNGATARNG	AAGATAATT
M00203	V$GATA_C	0.85306	3411	(+)	NGATAAGNMNN	AGATAATTTAC
M00127	V$GATA1_03	0.807692	3418	(-)	RNSNNGATAANNGN	TTACTTTTCTTGAT
M00075	V$GATA1_01	0.841066	3426	(+)	SNNGATNNNN	CTTGATTTTT
M00203	V$GATA_C	0.836906	3429	(-)	NGATAAGNMNN	GATTTTTGTCA
M00126	V$GATA1_02	0.775313	3540	(+)	NNNNNGATANKGNN	CTTGTGATATTATT
M00075	V$GATA1_01	0.833169	3542	(+)	SNNGATNNNN	TGTGATATTA
M00076	V$GATA2_01	0.861525	3542	(+)	NNNGATRNNN	TGTGATATTA
M00077	V$GATA3_01	0.860434	3543	(+)	NNGATARNG	GTGATATTA
M00075	V$GATA1_01	0.814906	3610	(-)	SNNGATNNNN	AAACATCCTA
M00076	V$GATA2_01	0.880469	3610	(-)	NNNGATRNNN	AAACATCCTA
M00126	V$GATA1_02	0.85125	3613	(-)	NNNNNGATANKGNN	CATCCTATCAAGCA
M00127	V$GATA1_03	0.78197	3613	(-)	RNSNNGATAANNGN	CATCCTATCAAGCA
M00128	V$GATA1_04	0.844669	3613	(-)	NNCWGATARNNNNN	CATCCTATCAAGC
M00075	V$GATA1_01	0.88845	3615	(-)	SNNGATNNNN	TCCTATCAAG
M00076	V$GATA2_01	0.823636	3615	(-)	NNNGATRNNN	TCCTATCAAG
M00077	V$GATA3_01	0.899867	3615	(-)	NNGATARNG	TCCTATCAA
M00075	V$GATA1_01	0.806515	3694	(+)	SNNGATNNNN	AGTGATGATA
M00076	V$GATA2_01	0.785747	3694	(+)	NNNGATRNNN	AGTGATGATA
M00077	V$GATA3_01	0.838724	3695	(+)	NNGATARNG	GTGATGATA
M00126	V$GATA1_02	0.807187	3695	(+)	NNNNNGATANKGNN	GTGATGATAAATTG
M00127	V$GATA1_03	0.870407	3695	(+)	RNSNNGATAANNGN	GTGATGATAAATTG
M00128	V$GATA1_04	0.867953	3696	(+)	NNCWGATARNNNNN	TGATGATAAATTG
M00075	V$GATA1_01	0.838598	3697	(+)	SNNGATNNNN	GATGATAAAT
M00076	V$GATA2_01	0.84258	3697	(+)	NNNGATRNNN	GATGATAAAT
M00077	V$GATA3_01	0.823217	3698	(+)	NNGATARNG	ATGATAAAT
M00203	V$GATA_C	0.835663	3699	(+)	NGATAAGNMNN	TGATAAATTGC

M00126	V$GATA1_02	0.834063	3776	(-)	NNNNNGATANKGNN	TGTAATATCTTCAA
M00128	V$GATA1_04	0.820772	3776	(-)	NNCWGATARNNNN	TGTAATATCTTCA
M00075	V$GATA1_01	0.840079	3778	(-)	SNNGATNNNN	TAATATCTTC
M00076	V$GATA2_01	0.879116	3778	(-)	NNNGATRNNN	TAATATCTTC
M00077	V$GATA3_01	0.866637	3778	(-)	NNGATARNG	TAATATCTT
M00075	V$GATA1_01	0.787266	3804	(-)	SNNGATNNNN	ATTGATCTGC
M00076	V$GATA2_01	0.797474	3804	(-)	NNNGATRNNN	ATTGATCTGC
M00075	V$GATA1_01	0.793189	3935	(+)	SNNGATNNNN	ATTGATTCTT
M00203	V$GATA_C	0.846536	3941	(-)	NGATAAGNMNN	TCTTTCTATCT
M00126	V$GATA1_02	0.84375	3942	(-)	NNNNNGATANKGNN	CTTTCTATCTCTTC
M00127	V$GATA1_03	0.841989	3942	(-)	RNSNNGATAANNGN	CTTTCTATCTCTTC
M00128	V$GATA1_04	0.904718	3942	(-)	NNCWGATARNNNN	CTTTCTATCTCTT
M00075	V$GATA1_01	0.811451	3944	(-)	SNNGATNNNN	TTCTATCTCT
M00076	V$GATA2_01	0.836716	3944	(-)	NNNGATRNNN	TTCTATCTCT
M00077	V$GATA3_01	0.924236	3944	(-)	NNGATARNG	TTCTATCTC
M00075	V$GATA1_01	0.777887	3984	(+)	SNNGATNNNN	CTTGATCTCA
M00076	V$GATA2_01	0.796121	3984	(-)	NNNGATRNNN	CTTGATCTCA
M00126	V$GATA1_02	0.809375	4013	(+)	NNNNNGATANKGNN	AATTAGATACTCTA
M00075	V$GATA1_01	0.789733	4015	(+)	SNNGATNNNN	TTAGATACTC
M00076	V$GATA2_01	0.820478	4015	(+)	NNNGATRNNN	TTAGATACTC
M00203	V$GATA_C	0.862069	4019	(-)	NGATAAGNMNN	ATACTCTATCA
M00126	V$GATA1_02	0.81625	4020	(-)	NNNNNGATANKGNN	TACTCTATCACACA
M00128	V$GATA1_04	0.85723	4020	(-)	NNCWGATARNNNN	TACTCTATCACAC
M00075	V$GATA1_01	0.887463	4022	(-)	SNNGATNNNN	CTCTATCACA
M00076	V$GATA2_01	0.900767	4022	(-)	NNNGATRNNN	CTCTATCACA
M00077	V$GATA3_01	0.935755	4022	(-)	NNGATARNG	CTCTATCAC
M00075	V$GATA1_01	0.794176	4048	(-)	SNNGATNNNN	ATTAATCTTC
M00076	V$GATA2_01	0.789355	4048	(-)	NNNGATRNNN	ATTAATCTTC
M00126	V$GATA1_02	0.85625	4058	(-)	NNNNNGATANKGNN	CACCCTATCCACTA
M00075	V$GATA1_01	0.905726	4060	(-)	SNNGATNNNN	CCCTATCCAC
M00076	V$GATA2_01	0.897609	4060	(-)	NNNGATRNNN	CCCTATCCAC
M00077	V$GATA3_01	0.913159	4060	(-)	NNGATARNG	CCCTATCCA
M00127	V$GATA1_03	0.823616	4102	(+)	RNSNNGATAANNGN	AGCCAGACAGCAGA

M00126	V$GATA1_02	0.893437	4113	(+)	NNNNNGATANKGNN	AGAGAGATAAGGTT
M00127	V$GATA1_03	0.898824	4113	(+)	RNSNNGATAANNGN	AGAGAGATAAGGTT
M00128	V$GATA1_04	0.904105	4114	(+)	NNCWGATARNNNNN	GAGAGATAAGGTT
M00075	V$GATA1_01	0.845015	4115	(+)	SNNGATNNNN	AGAGATAAGG
M00076	V$GATA2_01	0.874605	4115	(+)	NNNGATRNNN	AGAGATAAGG
M00077	V$GATA3_01	0.971644	4116	(+)	NNGATARNG	GAGATAAGG
M00203	V$GATA_C	0.928549	4117	(+)	NGATAAGNMNN	AGATAAGGTTC
M00076	V$GATA2_01	0.798376	4154	(+)	NNNGATRNNN	TCAGTTAGCT
M00076	V$GATA2_01	0.830853	4160	(-)	NNNGATRNNN	AGCTATGATA
M00128	V$GATA1_04	0.848346	4162	(+)	NNCWGATARNNNNN	CTATGATAAATAC
M00075	V$GATA1_01	0.78233	4163	(+)	SNNGATNNNN	TATGATAAAT
M00076	V$GATA2_01	0.828146	4163	(+)	NNNGATRNNN	TATGATAAAT
M00077	V$GATA3_01	0.823217	4164	(+)	NNGATARNG	ATGATAAAT
M00203	V$GATA_C	0.952159	4165	(+)	NGATAAGNMNN	TGATAAATACT
M00127	V$GATA1_03	0.843459	4174	(-)	RNSNNGATAANNGN	CTGAATATCTTCTT
M00128	V$GATA1_04	0.841605	4174	(-)	NNCWGATARNNNNN	CTGAATATCTTCT
M00075	V$GATA1_01	0.860809	4176	(-)	SNNGATNNNN	GAATATCTTC
M00076	V$GATA2_01	0.905277	4176	(-)	NNNGATRNNN	GAATATCTTC
M00077	V$GATA3_01	0.834736	4176	(-)	NNGATARNG	GAATATCTT
M00127	V$GATA1_03	0.798873	4220	(-)	RNSNNGATAANNGN	AATTGTATCCAGAT
M00075	V$GATA1_01	0.785785	4222	(-)	SNNGATNNNN	TTGTATCCAG
M00126	V$GATA1_02	0.811562	4226	(+)	NNNNNGATANKGNN	ATCCAGATACTCAG
M00127	V$GATA1_03	0.897844	4226	(+)	RNSNNGATAANNGN	ATCCAGATACTCAG
M00128	V$GATA1_04	0.817708	4227	(+)	NNCWGATARNNNNN	TCCAGATACTCAG
M00075	V$GATA1_01	0.907206	4228	(+)	SNNGATNNNN	CCAGATACTC
M00076	V$GATA2_01	0.91926	4228	(+)	NNNGATRNNN	CCAGATACTC
M00126	V$GATA1_02	0.836563	4234	(+)	NNNNNGATANKGNN	ACTCAGATAGTTGA
M00127	V$GATA1_03	0.811612	4234	(+)	RNSNNGATAANNGN	ACTCAGATAGTTGA
M00128	V$GATA1_04	0.830576	4235	(+)	NNCWGATARNNNNN	CTCAGATAGTTGA
M00075	V$GATA1_01	0.886969	4236	(+)	SNNGATNNNN	TCAGATAGTT
M00076	V$GATA2_01	0.939107	4236	(+)	NNNGATRNNN	TCAGATAGTT
M00077	V$GATA3_01	0.889233	4237	(+)	NNGATARNG	CAGATAGTT
M00203	V$GATA_C	0.833178	4238	(+)	NGATAAGNMNN	AGATAGTTGAT

M00075	V$GATA1_01	0.803554	4243	(+)	SNNGATNNNN	GTTGATTACC
M00127	V$GATA1_03	0.791034	4244	(-)	RNSNNGATAANNGN	TTGATTACCCTCTC
M00126	V$GATA1_02	0.795625	4249	(-)	NNNNNGATANKGNN	TACCCTCTCACTTC
M00077	V$GATA3_01	0.852459	4251	(-)	NNGATARNG	CCCTCTCAC
M00127	V$GATA1_03	0.795688	4256	(-)	RNSNNGATAANNGN	TCACTTCTCTGTTT
M00128	V$GATA1_04	0.812806	4256	(-)	NNCWGATARNNNN	TCACTTCTCTGTT
M00127	V$GATA1_03	0.794708	4263	(-)	RNSNNGATAANNGN	TCTGTTTTCATTAC
M00126	V$GATA1_02	0.79	4301	(+)	NNNNNGATANKGNN	CATTTGATAAGTAT
M00128	V$GATA1_04	0.906863	4302	(+)	NNCWGATARNNNN	ATTTGATAAGTAT
M00075	V$GATA1_01	0.798124	4303	(+)	SNNGATNNNN	TTTGATAAGT
M00076	V$GATA2_01	0.792963	4303	(+)	NNNGATRNNN	TTTGATAAGT
M00077	V$GATA3_01	0.83961	4304	(+)	NNGATARNG	TTGATAAGT
M00203	V$GATA_C	0.9242	4305	(+)	NGATAAGNMNN	TGATAAGTATG
M00076	V$GATA2_01	0.792512	4353	(-)	NNNGATRNNN	AAATATGGTG
M00075	V$GATA1_01	0.780849	4369	(+)	SNNGATNNNN	TCAGATTTTA
M00076	V$GATA2_01	0.8083	4369	(+)	NNNGATRNNN	TCAGATTTTA
M00077	V$GATA3_01	0.827204	4370	(+)	NNGATARNG	CAGATTTTA
M00075	V$GATA1_01	0.780849	4382	(-)	SNNGATNNNN	TAAAATCATA
M00076	V$GATA2_01	0.788904	4382	(-)	NNNGATRNNN	TAAAATCATA
M00203	V$GATA_C	0.849953	4400	(-)	NGATAAGNMNN	ACTAATTATCT
M00126	V$GATA1_02	0.839062	4401	(-)	NNNNNGATANKGNN	CTAATTATCTCTTA
M00127	V$GATA1_03	0.798138	4401	(-)	RNSNNGATAANNGN	CTAATTATCTCTTA
M00128	V$GATA1_04	0.892463	4401	(-)	NNCWGATARNNNN	CTAATTATCTCTT
M00075	V$GATA1_01	0.821323	4403	(-)	SNNGATNNNN	AATTATCTCT
M00076	V$GATA2_01	0.85521	4403	(-)	NNNGATRNNN	AATTATCTCT
M00077	V$GATA3_01	0.883917	4403	(-)	NNGATARNG	AATTATCTC
M00127	V$GATA1_03	0.78001	4440	(+)	RNSNNGATAANNGN	ACCTAAATAAATGG
M00128	V$GATA1_04	0.847426	4482	(-)	NNCWGATARNNNN	CTCCTTTTCTGAC
M00203	V$GATA_C	0.903386	4577	(-)	NGATAAGNMNN	AAGTATTATCT
M00126	V$GATA1_02	0.850313	4578	(-)	NNNNNGATANKGNN	AGTATTATCTACTT
M00127	V$GATA1_03	0.882656	4578	(-)	RNSNNGATAANNGN	AGTATTATCTACTT
M00128	V$GATA1_04	0.863358	4578	(-)	NNCWGATARNNNN	AGTATTATCTACT
M00075	V$GATA1_01	0.785785	4580	(-)	SNNGATNNNN	TATTATCTAC

M00076	V$GATA2_01	0.782589	4580	(-)	NNNGATRNNN	TATTATCTAC
M00077	V$GATA3_01	0.874169	4580	(-)	NNGATARNG	TATTATCTA
M00075	V$GATA1_01	0.836624	4621	(-)	SNNGATNNNN	AAAAATCAAC
M00077	V$GATA3_01	0.875498	4800	(-)	NNGATARNG	CATAATCTA
M00126	V$GATA1_02	0.781875	4825	(-)	NNNNNGATANKGNN	TCCATTCTCATTAT
M00127	V$GATA1_03	0.804508	4825	(-)	RNSNNGATAANNGN	TCCATTCTCATTAT
M00076	V$GATA2_01	0.782589	4833	(-)	NNNGATRNNN	CATTATGATG
M00075	V$GATA1_01	0.811945	4836	(+)	SNNGATNNNN	TATGATGGAA
M00076	V$GATA2_01	0.82138	4836	(+)	NNNGATRNNN	TATGATGGAA
M00077	V$GATA3_01	0.829863	4837	(+)	NNGATARNG	ATGATGGAA
M00203	V$GATA_C	0.95278	4845	(-)	NGATAAGNMNN	ATGTTTATCT
M00126	V$GATA1_02	0.878437	4846	(-)	NNNNNGATANKGNN	TGTTTTATCTCTAC
M00127	V$GATA1_03	0.840519	4846	(-)	RNSNNGATAANNGN	TGTTTTATCTCTAC
M00128	V$GATA1_04	0.887868	4846	(-)	NNCWGATARNNNNN	TGTTTTATCTCTA
M00076	V$GATA2_01	0.785295	4848	(-)	NNNGATRNNN	TTTTATCTCT
M00077	V$GATA3_01	0.895879	4848	(-)	NNGATARNG	TTTTATCTC
M00075	V$GATA1_01	0.851925	4866	(+)	SNNGATNNNN	CTGGATGTGT
M00076	V$GATA2_01	0.814163	4866	(+)	NNNGATRNNN	CTGGATGTGT
M00203	V$GATA_C	0.886921	4938	(+)	NGATAAGNMNN	AGACAAGTATT
M00077	V$GATA3_01	0.853788	4952	(+)	NNGATARNG	AAGATTTGA
M00126	V$GATA1_02	0.839375	4954	(+)	NNNNNGATANKGNN	GATTTGATAGGCAA
M00128	V$GATA1_04	0.873162	4955	(+)	NNCWGATARNNNNN	ATTTGATAGGCAA
M00075	V$GATA1_01	0.84847	4956	(+)	SNNGATNNNN	TTTGATAGGC
M00076	V$GATA2_01	0.840776	4956	(+)	NNNGATRNNN	TTTGATAGGC
M00077	V$GATA3_01	0.867966	4957	(+)	NNGATARNG	TTGATAGGC
M00203	V$GATA_C	0.90028	4958	(+)	NGATAAGNMNN	TGATAGGCAAC
M00075	V$GATA1_01	0.807996	4982	(+)	SNNGATNNNN	AATGATTTTT
M00076	V$GATA2_01	0.786198	4982	(+)	NNNGATRNNN	AATGATTTTT
M00077	V$GATA3_01	0.864865	4994	(-)	NNGATARNG	TATAATCTT
M00127	V$GATA1_03	0.793974	5033	(+)	RNSNNGATAANNGN	GGAAAGGTAAGAAA
M00203	V$GATA_C	0.830693	5037	(+)	NGATAAGNMNN	AGGTAAGAAAT
M00075	V$GATA1_01	0.774926	5102	(+)	SNNGATNNNN	GGTGTTATGC
M00076	V$GATA2_01	0.783491	5104	(-)	NNNGATRNNN	TGTTATGCCA

M00075	V$GATA1_01	0.836624	5136	(-)	SNNGATNNNN	AAAAATCACT
M00076	V$GATA2_01	0.794317	5136	(-)	NNNGATRNNN	AAAAATCACT
M00075	V$GATA1_01	0.793189	5177	(+)	SNNGATNNNN	AGAGATGAAG
M00076	V$GATA2_01	0.799729	5177	(+)	NNNGATRNNN	AGAGATGAAG
M00077	V$GATA3_01	0.877271	5178	(+)	NNGATARNG	GAGATGAAG
M00126	V$GATA1_02	0.793125	5181	(+)	NNNNNGATANKGNN	ATGAAGATAACTCA
M00127	V$GATA1_03	0.912543	5181	(+)	RNSNNGATAANNGN	ATGAAGATAACTCA
M00128	V$GATA1_04	0.884498	5182	(+)	NNCWGATARNNNN	TGAAGATAACTCA
M00075	V$GATA1_01	0.857354	5183	(+)	SNNGATNNNN	GAAGATAACT
M00076	V$GATA2_01	0.912945	5183	(+)	NNNGATRNNN	GAAGATAACT
M00077	V$GATA3_01	0.850687	5184	(+)	NNGATARNG	AAGATAACT
M00203	V$GATA_C	0.870146	5185	(+)	NGATAAGNMNN	AGATAACTCAG
M00076	V$GATA2_01	0.787551	5196	(-)	NNNGATRNNN	AACTATGACT
M00127	V$GATA1_03	0.789319	5208	(+)	RNSNNGATAANNGN	AACAAGATGAAAGA
M00075	V$GATA1_01	0.786772	5210	(+)	SNNGATNNNN	CAAGATGAAA
M00076	V$GATA2_01	0.790257	5210	(+)	NNNGATRNNN	CAAGATGAAA
M00077	V$GATA3_01	0.824989	5211	(+)	NNGATARNG	AAGATGAAA
M00126	V$GATA1_02	0.844688	5215	(+)	NNNNNGATANKGNN	TGAAAGATAATAAT
M00127	V$GATA1_03	0.817246	5215	(+)	RNSNNGATAANNGN	TGAAAGATAATAAT
M00128	V$GATA1_04	0.889706	5216	(+)	NNCWGATARNNNN	GAAAGATAATAAT
M00076	V$GATA2_01	0.817321	5217	(+)	NNNGATRNNN	AAAGATAATA
M00077	V$GATA3_01	0.898538	5218	(+)	NNGATARNG	AAGATAATA
M00127	V$GATA1_03	0.821166	5218	(+)	RNSNNGATAANNGN	AAGATAATAATAGT
M00203	V$GATA_C	0.892513	5219	(+)	NGATAAGNMNN	AGATAATAATA
M00126	V$GATA1_02	0.850313	5312	(+)	NNNNNGATANKGNN	AAGGAGATAATACA
M00127	V$GATA1_03	0.898824	5312	(+)	RNSNNGATAANNGN	AAGGAGATAATACA
M00128	V$GATA1_04	0.882353	5313	(+)	NNCWGATARNNNN	AGGAGATAATACA
M00075	V$GATA1_01	0.850444	5314	(+)	SNNGATNNNN	GGAGATAATA
M00076	V$GATA2_01	0.87235	5314	(+)	NNNGATRNNN	GGAGATAATA
M00077	V$GATA3_01	0.915817	5315	(+)	NNGATARNG	GAGATAATA
M00203	V$GATA_C	0.90028	5316	(+)	NGATAAGNMNN	AGATAATACAA
M00075	V$GATA1_01	0.801086	5400	(+)	SNNGATNNNN	TAAGATACAT
M00076	V$GATA2_01	0.865584	5400	(+)	NNNGATRNNN	TAAGATACAT

M00203	V$GATA_C	0.836285	5402	(+)	NGATAAGNMNN	AGATACATTTC
M00075	V$GATA1_01	0.775913	5426	(+)	SNNGATNNNN	TTTGATGGAA
M00127	V$GATA1_03	0.826801	5447	(-)	RNSNNGATAANNGN	TTTATTATATTCAT
M00127	V$GATA1_03	0.826801	5456	(-)	RNSNNGATAANNGN	TTCATTTTCTCCAT
M00203	V$GATA_C	0.856167	5487	(-)	NGATAAGNMNN	ACATTTTATCT
M00126	V$GATA1_02	0.86625	5488	(-)	NNNNNGATANKGNN	CATTTTATCTTTAC
M00127	V$GATA1_03	0.856688	5488	(-)	RNSNNGATAANNGN	CATTTTATCTTTAC
M00128	V$GATA1_04	0.876225	5488	(-)	NNCWGATARNNNN	CATTTTATCTTTA
M00077	V$GATA3_01	0.8786	5490	(-)	NNGATARNG	TTTTATCTT
M00126	V$GATA1_02	0.8425	5497	(-)	NNNNNGATANKGNN	TTTACTATCTTGCA
M00127	V$GATA1_03	0.809897	5497	(-)	RNSNNGATAANNGN	TTTACTATCTTGCA
M00128	V$GATA1_04	0.865196	5497	(-)	NNCWGATARNNNN	TTTACTATCTTGC
M00075	V$GATA1_01	0.891412	5499	(-)	SNNGATNNNN	TACTATCTTG
M00076	V$GATA2_01	0.910239	5499	(-)	NNNGATRNNN	TACTATCTTG
M00077	V$GATA3_01	0.926894	5499	(-)	NNGATARNG	TACTATCTT
M00203	V$GATA_C	0.89593	5506	(-)	NGATAAGNMNN	TTGCATTATCA
M00126	V$GATA1_02	0.842812	5507	(-)	NNNNNGATANKGNN	TGCATTATCAAATG
M00128	V$GATA1_04	0.862439	5507	(-)	NNCWGATARNNNN	TGCATTATCAAAT
M00075	V$GATA1_01	0.797137	5509	(-)	SNNGATNNNN	CATTATCAAA
M00076	V$GATA2_01	0.799729	5509	(-)	NNNGATRNNN	CATTATCAAA
M00077	V$GATA3_01	0.885689	5509	(-)	NNGATARNG	CATTATCAA
M00127	V$GATA1_03	0.804508	5560	(+)	RNSNNGATAANNGN	GTGGAGAAAGCTGT
M00075	V$GATA1_01	0.778381	5562	(+)	SNNGATNNNN	GGAGAAAGCT
M00127	V$GATA1_03	0.793974	5602	(+)	RNSNNGATAANNGN	AGCTTGATGATGAA
M00075	V$GATA1_01	0.857848	5604	(+)	SNNGATNNNN	CTTGATGATG
M00076	V$GATA2_01	0.780334	5604	(+)	NNNGATRNNN	CTTGATGATG
M00077	V$GATA3_01	0.832078	5605	(+)	NNGATARNG	TTGATGATG
M00075	V$GATA1_01	0.80306	5607	(+)	SNNGATNNNN	GATGATGAAA
M00076	V$GATA2_01	0.784393	5607	(+)	NNNGATRNNN	GATGATGAAA
M00127	V$GATA1_03	0.815042	5608	(+)	RNSNNGATAANNGN	ATGATGAAAACGCC
M00126	V$GATA1_02	0.775	5633	(+)	NNNNNGATANKGNN	TTATAGATATCCAT
M00076	V$GATA2_01	0.79567	5635	(+)	NNNGATRNNN	ATAGATATCC
M00075	V$GATA1_01	0.770484	5637	(-)	SNNGATNNNN	AGATATCCAT

M00076	V$GATA2_01	0.813712	5637	(-)	NNNGATRNNN	AGATATCCAT
M00127	V$GATA1_03	0.810142	5663	(-)	RNSNNGATAANNGN	CTTCTTTTCTGTCC
M00128	V$GATA1_04	0.823223	5663	(-)	NNCWGATARNNNN	CTTCTTTTCTGTC
M00127	V$GATA1_03	0.826801	5700	(+)	RNSNNGATAANNGN	AGCAAGGTAAGGAA
M00127	V$GATA1_03	0.822636	5709	(+)	RNSNNGATAANNGN	AGGAACATAAATGA
M00126	V$GATA1_02	0.782188	5716	(+)	NNNNNGATANKGNN	TAAATGATATTTAA
M00128	V$GATA1_04	0.818321	5717	(+)	NNCWGATARNNNN	AAATGATATTTAA
M00075	V$GATA1_01	0.831688	5718	(+)	SNNGATNNNN	AATGATATTT
M00076	V$GATA2_01	0.850699	5718	(+)	NNNGATRNNN	AATGATATTT
M00203	V$GATA_C	0.855235	5737	(-)	NGATAAGNMNN	TAAAGTTATCT
M00126	V$GATA1_02	0.793438	5738	(-)	NNNNNGATANKGNN	AAAGTTATCTCAAA
M00128	V$GATA1_04	0.863358	5738	(-)	NNCWGATARNNNN	AAAGTTATCTCAA
M00075	V$GATA1_01	0.829714	5740	(-)	SNNGATNNNN	AGTTATCTCA
M00076	V$GATA2_01	0.906631	5740	(-)	NNNGATRNNN	AGTTATCTCA
M00077	V$GATA3_01	0.867966	5740	(-)	NNGATARNG	AGTTATCTC
M00075	V$GATA1_01	0.827739	5748	(-)	SNNGATNNNN	CAAAATCATA
M00076	V$GATA2_01	0.831755	5748	(-)	NNNGATRNNN	CAAAATCATA
M00077	V$GATA3_01	0.844484	5748	(-)	NNGATARNG	CAAAATCAT
M00075	V$GATA1_01	0.831194	5772	(+)	SNNGATNNNN	ATTGATTGGT
M00077	V$GATA3_01	0.834293	5773	(+)	NNGATARNG	TTGATTGGT
M00127	V$GATA1_03	0.785154	5818	(+)	RNSNNGATAANNGN	ATGAAAATACGAAA
M00076	V$GATA2_01	0.795219	5839	(-)	NNNGATRNNN	TGGTATGTCA
M00075	V$GATA1_01	0.810958	5931	(+)	SNNGATNNNN	ACTGATGAGA
M00076	V$GATA2_01	0.792061	5931	(+)	NNNGATRNNN	ACTGATGAGA
M00077	V$GATA3_01	0.836509	5932	(+)	NNGATARNG	CTGATGAGA
M00076	V$GATA2_01	0.794317	5936	(+)	NNNGATRNNN	TGAGATCTAC
M00076	V$GATA2_01	0.800631	5985	(-)	NNNGATRNNN	AACCATGAGG
M00127	V$GATA1_03	0.78001	5989	(+)	RNSNNGATAANNGN	ATGAGGAGACAAGG
M00126	V$GATA1_02	0.780312	5991	(+)	NNNNNGATANKGNN	GAGGAGACAAGGCA
M00203	V$GATA_C	0.899969	5995	(+)	NGATAAGNMNN	AGACAAGGCAT
M00126	V$GATA1_02	0.828125	6020	(+)	NNNNNGATANKGNN	GAATGGATAAAAGA
M00127	V$GATA1_03	0.843704	6020	(+)	RNSNNGATAANNGN	GAATGGATAAAAGA
M00203	V$GATA_C	0.900901	6024	(+)	NGATAAGNMNN	GGATAAAAGAA

M00127	V$GATA1_03	0.784664	6059	(-)	RNSNNGATAANNGN	CAGACTATTTTCAC
M00075	V$GATA1_01	0.833169	6071	(+)	SNNGATNNNN	ACTGATCGCC
M00075	V$GATA1_01	0.861797	6071	(-)	SNNGATNNNN	ACTGATCGCC
M00076	V$GATA2_01	0.846189	6071	(+)	NNNGATRNNN	ACTGATCGCC
M00076	V$GATA2_01	0.840776	6071	(-)	NNNGATRNNN	ACTGATCGCC
M00128	V$GATA1_04	0.85723	6118	(-)	NNCWGATARNNNN	CAATATATCTGCT
M00075	V$GATA1_01	0.856861	6120	(-)	SNNGATNNNN	ATATATCTGC
M00076	V$GATA2_01	0.876861	6120	(-)	NNNGATRNNN	ATATATCTGC
M00075	V$GATA1_01	0.807996	6155	(+)	SNNGATNNNN	AATGATTTTT
M00076	V$GATA2_01	0.786198	6155	(+)	NNNGATRNNN	AATGATTTTT
M00126	V$GATA1_02	0.786563	6303	(+)	NNNNNGATANKGNN	GGCAGGATATTGGG
M00127	V$GATA1_03	0.839784	6303	(+)	RNSNNGATAANNGN	GGCAGGATATTGGG
M00075	V$GATA1_01	0.886969	6305	(+)	SNNGATNNNN	CAGGATATTG
M00076	V$GATA2_01	0.930988	6305	(+)	NNNGATRNNN	CAGGATATTG
M00077	V$GATA3_01	0.856447	6306	(+)	NNGATARNG	AGGATATTG
M00075	V$GATA1_01	0.803554	6318	(-)	SNNGATNNNN	GTGAATCAAC
M00075	V$GATA1_01	0.816881	6333	(+)	SNNGATNNNN	TAAGATTCTT
M00076	V$GATA2_01	0.841227	6333	(+)	NNNGATRNNN	TAAGATTCTT
M00126	V$GATA1_02	0.783125	6361	(+)	NNNNNGATANKGNN	TATAATATAAGGGA
M00128	V$GATA1_04	0.817708	6362	(+)	NNCWGATARNNNN	ATAATATAAGGGA
M00127	V$GATA1_03	0.811612	6370	(+)	RNSNNGATAANNGN	AGGGAAATAAAGAA
M00075	V$GATA1_01	0.814906	6389	(-)	SNNGATNNNN	AAATATCCTA
M00076	V$GATA2_01	0.908886	6389	(-)	NNNGATRNNN	AAATATCCTA
M00075	V$GATA1_01	0.785785	6395	(-)	SNNGATNNNN	CCTAATCAAA
M00077	V$GATA3_01	0.87284	6395	(-)	NNGATARNG	CCTAATCAA
M00126	V$GATA1_02	0.784375	6400	(-)	NNNNNGATANKGNN	TCAAATATCCTTTA
M00075	V$GATA1_01	0.806515	6402	(-)	SNNGATNNNN	AAATATCCTT
M00076	V$GATA2_01	0.87641	6402	(-)	NNNGATRNNN	AAATATCCTT
M00075	V$GATA1_01	0.83613	6413	(-)	SNNGATNNNN	AAGTATCCTA
M00076	V$GATA2_01	0.920162	6413	(-)	NNNGATRNNN	AAGTATCCTA
M00075	V$GATA1_01	0.785785	6419	(-)	SNNGATNNNN	CCTAATCAAA
M00077	V$GATA3_01	0.87284	6419	(-)	NNGATARNG	CCTAATCAA
M00203	V$GATA_C	0.844051	6449	(+)	NGATAAGNMNN	GGACAAAACTA

M00128	V$GATA1_04	0.871936	6461	(+)	NNCWGATARNNNN	AACAGATATCTCT
M00075	V$GATA1_01	0.831688	6462	(+)	SNNGATNNNN	ACAGATATCT
M00076	V$GATA2_01	0.889039	6462	(+)	NNNGATRNNN	ACAGATATCT
M00126	V$GATA1_02	0.81125	6462	(-)	NNNNNGATANKGNN	ACAGATATCTCTAA
M00128	V$GATA1_04	0.835784	6462	(-)	NNCWGATARNNNN	ACAGATATCTCTA
M00075	V$GATA1_01	0.839585	6464	(-)	SNNGATNNNN	AGATATCTCT
M00076	V$GATA2_01	0.889039	6464	(-)	NNNGATRNNN	AGATATCTCT
M00077	V$GATA3_01	0.836066	6464	(-)	NNGATARNG	AGATATCTC
M00127	V$GATA1_03	0.888045	6517	(+)	RNSNNGATAANNGN	GGCTGGATAACTAA
M00075	V$GATA1_01	0.821323	6519	(+)	SNNGATNNNN	CTGGATAACT
M00076	V$GATA2_01	0.840325	6519	(+)	NNNGATRNNN	CTGGATAACT
M00203	V$GATA_C	0.882883	6521	(+)	NGATAAGNMNN	GGATAACTAAA
M00076	V$GATA2_01	0.802436	6533	(+)	NNNGATRNNN	CAACATAGTA
M00075	V$GATA1_01	0.854393	6563	(-)	SNNGATNNNN	GAGCATCTCA
M00076	V$GATA2_01	0.881822	6563	(-)	NNNGATRNNN	GAGCATCTCA
M00076	V$GATA2_01	0.823636	6586	(-)	NNNGATRNNN	TGCTATGGTC
M00127	V$GATA1_03	0.792504	6621	(+)	RNSNNGATAANNGN	ATGGAGAAAATTCA
M00075	V$GATA1_01	0.792201	6651	(-)	SNNGATNNNN	ATACATCATT
M00076	V$GATA2_01	0.782138	6651	(-)	NNNGATRNNN	ATACATCATT
M00203	V$GATA_C	0.932277	6681	(-)	NGATAAGNMNN	TTTATTTATCA
M00128	V$GATA1_04	0.902574	6682	(-)	NNCWGATARNNNN	TTATTTATCACAT
M00075	V$GATA1_01	0.810958	6684	(-)	SNNGATNNNN	ATTTATCACA
M00076	V$GATA2_01	0.836265	6684	(-)	NNNGATRNNN	ATTTATCACA
M00077	V$GATA3_01	0.840496	6684	(-)	NNGATARNG	ATTTATCAC
M00126	V$GATA1_02	0.780312	6690	(-)	NNNNNGATANKGNN	CACATAATCTTTAA
M00075	V$GATA1_01	0.780355	6692	(-)	SNNGATNNNN	CATAATCTTT
M00076	V$GATA2_01	0.79567	6692	(-)	NNNGATRNNN	CATAATCTTT
M00077	V$GATA3_01	0.899867	6692	(-)	NNGATARNG	CATAATCTT
M00075	V$GATA1_01	0.772458	6749	(+)	SNNGATNNNN	GAAGTTAGGC
M00076	V$GATA2_01	0.788453	6749	(+)	NNNGATRNNN	GAAGTTAGGC
M00162	V$OCT1_06	0.808203	53	(+)	CWNAWTKWSATRYN	AATTCTTAGAATTT
M00138	V$OCT1_04	0.910707	56	(-)	NNNNNNNWATGCAAATNNNWNNW	TCTTAGAATTTAAATAAACCTGT
M00135	V$OCT1_01	0.78367	58	(-)	NNNNWTATGCAAATNTNNN	TTAGAATTTAAATAAACCT

M00162	V$OCT1_06	0.839844	60	(+)	CWNAWTKWSATRYN	AGAATTTAAATAAA
M00162	V$OCT1_06	0.860547	60	(-)	CWNAWTKWSATRYN	AGAATTTAAATAAA
M00248	V$OCT1_07	0.90566	62	(-)	TNTATGNTAATT	AATTTAAATAAA
M00162	V$OCT1_06	0.800391	89	(-)	CWNAWTKWSATRYN	GAAACCACAAAATT
M00138	V$OCT1_04	0.853199	93	(-)	NNNNNNNWATGCAAATNNNWNNW	CCACAAAATTAGCATTTTACT-AA
M00135	V$OCT1_01	0.867035	95	(-)	NNNNWTATGCAAATNTNNN	ACAAAATTAGCATTTTACT
M00161	V$OCT1_05	0.891582	97	(+)	MKNATTTGCATAYY	AAAATTAGCATTTT
M00162	V$OCT1_06	0.847656	97	(+)	CWNAWTKWSATRYN	AAAATTAGCATTTT
M00136	V$OCT1_02	0.801299	99	(-)	NNGAATATKCANNNN	AATTAGCATTTTACT
M00248	V$OCT1_07	0.921772	99	(-)	TNTATGNTAATT	AATTAGCATTTT
M00137	V$OCT1_03	0.888582	109	(+)	NNNRTAATNANNN	TTACTAATCAGTA
M00162	V$OCT1_06	0.800781	190	(+)	CWNAWTKWSATRYN	CAGTCTTGGGTTCA
M00138	V$OCT1_04	0.783982	202	(-)	NNNNNNNWATGCAAATNNNWNNW	CAAGGTATTATGTATACATA-TAA
M00136	V$OCT1_02	0.779101	205	(+)	NNGAATATKCANNNN	GGTATTATGTATACA
M00138	V$OCT1_04	0.843371	207	(+)	NNNNNNNWATGCAAATNNNWNNW	TATTATGTATACATA-TAACAAAA
M00135	V$OCT1_01	0.735788	209	(+)	NNNNWTATGCAAATNTNNN	TTATGTATACATATAACAA
M00138	V$OCT1_04	0.781681	209	(+)	NNNNNNNWATGCAAATNNNWNNW	TTATGTATACATA-TAACAAAATT
M00195	V$OCT1_Q6	0.790393	211	(+)	NNNNATGCAAATNAN	ATGTATACATATAAC
M00138	V$OCT1_04	0.7821	213	(+)	NNNNNNNWATGCAAATNNNWNNW	GTATACATATAACAAAATTTC-TA
M00162	V$OCT1_06	0.834375	218	(-)	CWNAWTKWSATRYN	CATATAACAAAATT
M00162	V$OCT1_06	0.852344	220	(-)	CWNAWTKWSATRYN	TATAACAAAATTTC
M00138	V$OCT1_04	0.7867	222	(-)	NNNNNNNWATGCAAATNNNWNNW	TAACAAAATTTCTATGATTTTCC
M00135	V$OCT1_01	0.731973	224	(-)	NNNNWTATGCAAATNTNNN	ACAAAATTTCTATGATTTT
M00162	V$OCT1_06	0.813672	226	(-)	CWNAWTKWSATRYN	AAAATTTCTATGAT
M00248	V$OCT1_07	0.796905	228	(-)	TNTATGNTAATT	AATTTCTATGAT
M00162	V$OCT1_06	0.805859	229	(+)	CWNAWTKWSATRYN	ATTTCTATGATTTT
M00162	V$OCT1_06	0.829297	252	(-)	CWNAWTKWSATRYN	CATCTTTCATTCTT
M00138	V$OCT1_04	0.788373	254	(-)	NNNNNNNWATGCAAATNNNWNNW	TCTTTCATTCTTCACTAATACGC
M00136	V$OCT1_02	0.799675	267	(+)	NNGAATATKCANNNN	ACTAATACGCAGTTG
M00137	V$OCT1_03	0.876729	292	(-)	NNNRTAATNANNN	ATGTGATTGCAAG
M00162	V$OCT1_06	0.821484	295	(-)	CWNAWTKWSATRYN	TGATTGCAAGTATT
M00162	V$OCT1_06	0.813672	309	(+)	CWNAWTKWSATRYN	GGTACTTTCCTATG
M00138	V$OCT1_04	0.804057	329	(-)	NNNNNNNWATGCAAATNNNWNNW	TGTTAGCTTAAAAATATATTTGC

M00138	V$OCT1_04	0.788164	339	(-)	NNNNNNNWATGCAAATNNNWNNW	AAAATATATTTGCAAATGTT-GAT
M00138	V$OCT1_04	0.837725	340	(+)	NNNNNNNWATGCAAATNNNWNNW	AAATATATTTGCAAATGTTGA-TA
M00135	V$OCT1_01	0.842617	341	(-)	NNNNWTATGCAAATNTNNN	AATATATTTGCAAATGTTG
M00135	V$OCT1_01	0.858642	342	(+)	NNNNWTATGCAAATNTNNN	ATATATTTGCAAATGTTGA
M00195	V$OCT1_Q6	0.814683	344	(+)	NNNNATGCAAATNAN	ATATTTGCAAATGTT
M00162	V$OCT1_06	0.834375	345	(-)	CWNAWTKWSATRYN	TATTTGCAAATGTT
M00138	V$OCT1_04	0.801757	346	(+)	NNNNNNNWATGCAAATNNNWNNW	ATTTGCAAATGTTGATACTATCT
M00162	V$OCT1_06	0.878516	351	(+)	CWNAWTKWSATRYN	CAAATGTTGATACT
M00162	V$OCT1_06	0.800781	366	(-)	CWNAWTKWSATRYN	TCTATCTCAGAGCT
M00162	V$OCT1_06	0.945312	387	(+)	CWNAWTKWSATRYN	AAAAATTAAATACT
M00162	V$OCT1_06	0.813672	387	(-)	CWNAWTKWSATRYN	AAAAATTAAATACT
M00162	V$OCT1_06	0.885547	388	(-)	CWNAWTKWSATRYN	AAAATTAAATACTT
M00248	V$OCT1_07	0.793301	389	(-)	TNTATGNTAATT	AAATTAAATACT
M00137	V$OCT1_03	0.840774	401	(+)	NNNRTAATNANNN	TTTATAAAGACCA
M00138	V$OCT1_04	0.788164	427	(+)	NNNNNNNWATGCAAATNNNWNNW	TAAACGAAATTCTTATA-TACTGA
M00162	V$OCT1_06	0.842187	427	(-)	CWNAWTKWSATRYN	TAAACGAAATTCTT
M00162	V$OCT1_06	0.842187	434	(+)	CWNAWTKWSATRYN	AATTCTTATATACT
M00138	V$OCT1_04	0.780217	444	(+)	NNNNNNNWATGCAAATNNNWNNW	TACTGAAAATGTAGATA-CATAAC
M00136	V$OCT1_02	0.788847	446	(+)	NNGAATATKCANNNN	CTGAAAATGTAGATA
M00162	V$OCT1_06	0.860156	449	(+)	CWNAWTKWSATRYN	AAAATGTAGATACA
M00138	V$OCT1_04	0.789419	450	(+)	NNNNNNNWATGCAAATNNNWNNW	AAATGTAGATA-CATAACTTCAGT
M00138	V$OCT1_04	0.833752	473	(+)	NNNNNNNWATGCAAATNNNWNNW	ATAGATTTATGGTAAAA-TAATTT
M00135	V$OCT1_01	0.794353	475	(+)	NNNNWTATGCAAATNTNNN	AGATTTATGGTAAAATAAT
M00248	V$OCT1_07	0.795209	478	(+)	TNTATGNTAATT	TTTATGGTAAAA
M00138	V$OCT1_04	0.856545	485	(-)	NNNNNNNWATGCAAATNNNWNNW	TAAAATAATTTGAATCATTTTTG
M00135	V$OCT1_01	0.736169	487	(-)	NNNNWTATGCAAATNTNNN	AAATAATTTGAATCATTTT
M00162	V$OCT1_06	0.847266	488	(+)	CWNAWTKWSATRYN	AATAATTTGAATCA
M00195	V$OCT1_Q6	0.822871	489	(-)	NNNNATGCAAATNAN	ATAATTTGAATCATT
M00248	V$OCT1_07	0.797965	491	(-)	TNTATGNTAATT	AATTTGAATCAT
M00162	V$OCT1_06	0.826172	501	(+)	CWNAWTKWSATRYN	ATTTTTGTCAAATT
M00137	V$OCT1_03	0.846701	514	(+)	NNNRTAATNANNN	TCTGTAAAAAGTT
M00162	V$OCT1_06	0.867969	521	(+)	CWNAWTKWSATRYN	AAAGTTGTCATACA
M00138	V$OCT1_04	0.790464	522	(+)	NNNNNNNWATGCAAATNNNWNNW	AAGTTGTCATACAGAATAATT-TA

M00138	V$OCT1_04	0.826642	532	(+)	NNNNNNNWATGCAAATNNNWNNW	ACAGAATAATTTATAATATTTTT
M00138	V$OCT1_04	0.829778	534	(-)	NNNNNNNWATGCAAATNNNWNNW	AGAATAATTTATAATATTTTTGT
M00137	V$OCT1_03	0.854998	541	(+)	NNNRTAATNANNN	TTTATAATATTTT
M00138	V$OCT1_04	0.785445	542	(-)	NNNNNNNWATGCAAATNNNWNNW	TTATAATATTTTTGTTTTCATAG
M00138	V$OCT1_04	0.839398	548	(-)	NNNNNNNWATGCAAATNNNWNNW	TATTTTTGTTTTCATAGAAATAA
M00138	V$OCT1_04	0.782727	553	(+)	NNNNNNNWATGCAAATNNNWNNW	TTGTTTTCATAGAAATAACATTT
M00138	V$OCT1_04	0.810121	554	(-)	NNNNNNNWATGCAAATNNNWNNW	TGTTTTCATAGAAATAACATTTC
M00162	V$OCT1_06	0.842578	563	(+)	CWNAWTKWSATRYN	AGAAATAACATTTC
M00162	V$OCT1_06	0.834766	564	(-)	CWNAWTKWSATRYN	GAAATAACATTTCT
M00248	V$OCT1_07	0.803265	565	(-)	TNTATGNTAATT	AAATAACATTTC
M00162	V$OCT1_06	0.834375	569	(-)	CWNAWTKWSATRYN	AACATTTCTGGTAG
M00136	V$OCT1_02	0.814293	577	(-)	NNGAATATKCANNNN	TGGTAGAATATTTCA
M00162	V$OCT1_06	0.877734	583	(-)	CWNAWTKWSATRYN	AATATTTCAAGGCC
M00138	V$OCT1_04	0.799247	597	(-)	NNNNNNNWATGCAAATNNNWNNW	ATTTTTATTTTGTGTAATTAGGT
M00137	V$OCT1_03	0.945871	607	(+)	NNNRTAATNANNN	TGTGTAATTAGGT
M00138	V$OCT1_04	0.851108	608	(+)	NNNNNNNWATGCAAATNNNWNNW	GTGTAATTAGGTTAATAAAAT-TA
M00135	V$OCT1_01	0.736551	610	(+)	NNNNWTATGCAAATNTNNN	GTAATTAGGTTAATAAAAT
M00162	V$OCT1_06	0.826562	622	(+)	CWNAWTKWSATRYN	ATAAAATTAATTTT
M00162	V$OCT1_06	0.826562	624	(-)	CWNAWTKWSATRYN	AAAATTAATTTTAT
M00162	V$OCT1_06	0.842187	636	(+)	CWNAWTKWSATRYN	ATAAAGGAAATGTC
M00195	V$OCT1_Q6	0.842249	641	(+)	NNNNATGCAAATNAN	GGAAATGTCAATGAT
M00162	V$OCT1_06	0.911719	642	(-)	CWNAWTKWSATRYN	GAAATGTCAATGAT
M00137	V$OCT1_03	0.860925	655	(+)	NNNRTAATNANNN	TAGACAATTAGAT
M00162	V$OCT1_06	0.945312	657	(+)	CWNAWTKWSATRYN	GACAATTAGATATA
M00138	V$OCT1_04	0.783354	658	(+)	NNNNNNNWATGCAAATNNNWNNW	ACAATTAGATATAAATGAC-TACT
M00138	V$OCT1_04	0.801966	659	(-)	NNNNNNNWATGCAAATNNNWNNW	CAATTAGATATAAATGAC-TACTT
M00195	V$OCT1_Q6	0.796943	662	(+)	NNNNATGCAAATNAN	TTAGATATAAATGAC
M00138	V$OCT1_04	0.781681	677	(-)	NNNNNNNWATGCAAATNNNWNNW	TACTTTTATAAAGATGATTAAAT
M00137	V$OCT1_03	0.893718	681	(+)	NNNRTAATNANNN	TTTATAAAGATGA
M00138	V$OCT1_04	0.819113	682	(+)	NNNNNNNWATGCAAATNNNWNNW	TTATAAAGATGATTAAATTT-GGA
M00162	V$OCT1_06	0.837109	689	(+)	CWNAWTKWSATRYN	GATGATTAAATTTG
M00138	V$OCT1_04	0.893768	691	(-)	NNNNNNNWATGCAAATNNNWNNW	TGATTAAATTTGGATATTTGTAA
M00135	V$OCT1_01	0.876765	693	(-)	NNNNWTATGCAAATNTNNN	ATTAAATTTGGATATTTGT

M00135	V$OCT1_01	0.740176	694	(+)	NNNNWTATGCAAATNTNNN	TTAAATTTGGATATTTGTA
M00162	V$OCT1_06	0.873437	695	(+)	CWNAWTKWSATRYN	TAAATTTGGATATT
M00195	V$OCT1_Q6	0.791212	695	(-)	NNNNATGCAAATNAN	TAAATTTGGATATTT
M00136	V$OCT1_02	0.835138	697	(-)	NNGAATATKCANNNN	AATTTGGATATTTGT
M00248	V$OCT1_07	0.854993	697	(-)	TNTATGNTAATT	AATTTGGATATT
M00138	V$OCT1_04	0.837516	708	(+)	NNNNNNNWATGCAAATNNNWNNW	TTGTAAGGATA-CAAATATATGAA
M00135	V$OCT1_01	0.758871	710	(+)	NNNNWTATGCAAATNTNNN	GTAAGGATACAAATATATG
M00135	V$OCT1_01	0.736169	720	(+)	NNNNWTATGCAAATNTNNN	AAATATATGAAAACAGTAG
M00162	V$OCT1_06	0.812109	749	(-)	CWNAWTKWSATRYN	GGCCTATAAATATG
M00137	V$OCT1_03	0.855393	751	(+)	NNNRTAATNANNN	CCTATAAATATGT
M00138	V$OCT1_04	0.807403	751	(-)	NNNNNNNWATGCAAATNNNWNNW	CCTATAAATATGTCTTTTTTAAC
M00136	V$OCT1_02	0.783433	754	(+)	NNGAATATKCANNNN	ATAAATATGTCTTTT
M00162	V$OCT1_06	0.828906	756	(+)	CWNAWTKWSATRYN	AAATATGTCTTTTT
M00162	V$OCT1_06	0.875781	757	(-)	CWNAWTKWSATRYN	AATATGTCTTTTTT
M00162	V$OCT1_06	0.855078	814	(-)	CWNAWTKWSATRYN	TATATAAAATAATC
M00138	V$OCT1_04	0.78189	840	(+)	NNNNNNNWATGCAAATNNNWNNW	GCTGACTAAGGTATAAACAAATT
M00138	V$OCT1_04	0.781054	846	(+)	NNNNNNNWATGCAAATNNNWNNW	TAAGGTATAAACAAATTTCATGT
M00135	V$OCT1_01	0.791683	848	(+)	NNNNWTATGCAAATNTNNN	AGGTATAAACAAATTTCAT
M00137	V$OCT1_03	0.856183	849	(+)	NNNRTAATNANNN	GGTATAAACAAAT
M00162	V$OCT1_06	0.889062	856	(+)	CWNAWTKWSATRYN	ACAAATTTCATGTA
M00138	V$OCT1_04	0.807821	857	(+)	NNNNNNNWATGCAAATNNNWNNW	CAAATTTCATGTATAATCTAATT
M00162	V$OCT1_06	0.885547	857	(-)	CWNAWTKWSATRYN	CAAATTTCATGTAT
M00136	V$OCT1_02	0.788847	862	(-)	NNGAATATKCANNNN	TTCATGTATAATCTA
M00138	V$OCT1_04	0.782936	863	(+)	NNNNNNNWATGCAAATNNNWNNW	TCATGTATAATCTAATTTTTCTT
M00135	V$OCT1_01	0.753338	865	(+)	NNNNWTATGCAAATNTNNN	ATGTATAATCTAATTTTTC
M00137	V$OCT1_03	0.855788	866	(+)	NNNRTAATNANNN	TGTATAATCTAAT
M00248	V$OCT1_07	0.838457	868	(+)	TNTATGNTAATT	TATAATCTAATT
M00162	V$OCT1_06	0.868359	869	(-)	CWNAWTKWSATRYN	ATAATCTAATTTTT
M00135	V$OCT1_01	0.740939	873	(-)	NNNNWTATGCAAATNTNNN	TCTAATTTTTCTTATGTAT
M00162	V$OCT1_06	0.808203	888	(+)	CWNAWTKWSATRYN	GTATCTGAAACGCA
M00162	V$OCT1_06	0.868359	911	(+)	CWNAWTKWSATRYN	CACATATAAATGTA
M00162	V$OCT1_06	0.807031	911	(-)	CWNAWTKWSATRYN	CACATATAAATGTA
M00138	V$OCT1_04	0.792555	912	(+)	NNNNNNNWATGCAAATNNNWNNW	ACATATAAATGTATGTATTTTTG

M0016<br>2	V$OCT1_06	0.84218<br>7	917	(-)	CWNAWTKWSATRYN	TAAATGTATGTATT
M0016<br>2	V$OCT1_06	0.90195<br>3	921	(-)	CWNAWTKWSATRYN	TGTATGTATTTTTG
M0016<br>2	V$OCT1_06	0.80078<br>1	935	(+)	CWNAWTKWSATRYN	GGTCTTGCAATTTA
M0016<br>2	V$OCT1_06	0.80820<br>3	975	(+)	CWNAWTKWSATRYN	ATGTTTGACTTATG
M0013<br>8	V$OCT1_04	0.81116<br>7	984	(+)	NNNNNNNWATGCAAATNNNWNN<br>W	TTATGATTCTGTTTAATCATCTT
M0013<br>8	V$OCT1_04	0.78147<br>2	989	(-)	NNNNNNNWATGCAAATNNNWNN<br>W	ATTCTGTTTAATCATCTTCATTC
M0013<br>6	V$OCT1_02	0.77070<br>9	998	(+)	NNGAATATKCANNNN	AATCATCTTCATTCA
M0016<br>2	V$OCT1_06	0.82656<br>2	999	(+)	CWNAWTKWSATRYN	ATCATCTTCATTCA
M0016<br>2	V$OCT1_06	0.84218<br>7	1007	(+)	CWNAWTKWSATRYN	CATTCAGTCATGTC
M0016<br>2	V$OCT1_06	0.84218<br>7	1012	(+)	CWNAWTKWSATRYN	AGTCATGTCATGGA
M0016<br>2	V$OCT1_06	0.83710<br>9	1013	(-)	CWNAWTKWSATRYN	GTCATGTCATGGAT
M0016<br>2	V$OCT1_06	0.80429<br>7	1039	(+)	CWNAWTKWSATRYN	CAAAATTAAGTAAT
M0016<br>2	V$OCT1_06	0.80078<br>1	1039	(-)	CWNAWTKWSATRYN	CAAAATTAAGTAAT
M0016<br>2	V$OCT1_06	0.86835<br>9	1067	(+)	CWNAWTKWSATRYN	AAAAATTTAATGAA
M0013<br>8	V$OCT1_04	0.80259<br>3	1068	(+)	NNNNNNNWATGCAAATNNNWNN<br>W	AAAATTTAATGAAGGTTAA-<br>TAGT
M0016<br>2	V$OCT1_06	0.92460<br>9	1068	(-)	CWNAWTKWSATRYN	AAAATTTAATGAAG
M0013<br>5	V$OCT1_01	0.73903<br>1	1086	(-)	NNNNWTATGCAAATNTNNN	ATAGTTTCTACATAATGCA
M0016<br>2	V$OCT1_06	0.83437<br>5	1107	(+)	CWNAWTKWSATRYN	ATGTTTTTCATGAA
M0016<br>2	V$OCT1_06	0.84726<br>6	1108	(-)	CWNAWTKWSATRYN	TGTTTTTCATGAAG
M0013<br>7	V$OCT1_03	0.87356<br>8	1142	(+)	NNNRTAATNANNN	AGGATAAAGACAT
M0016<br>2	V$OCT1_06	0.86289<br>1	1144	(+)	CWNAWTKWSATRYN	GATAAAGACATTTT
M0016<br>2	V$OCT1_06	0.80039<br>1	1152	(-)	CWNAWTKWSATRYN	CATTTTAAAAAATT
M0013<br>7	V$OCT1_03	0.84037<br>9	1158	(-)	NNNRTAATNANNN	AAAAAATTACAGA
M0013<br>8	V$OCT1_04	0.81054	1158	(+)	NNNNNNNWATGCAAATNNNWNN<br>W	AAAAAATTACAGA-<br>TATTAAATGT
M0016<br>2	V$OCT1_06	0.87578<br>1	1159	(+)	CWNAWTKWSATRYN	AAAAATTACAGATA
M0016<br>2	V$OCT1_06	0.86992<br>2	1160	(-)	CWNAWTKWSATRYN	AAAATTACAGATAT
M0016<br>2	V$OCT1_06	0.90625	1168	(+)	CWNAWTKWSATRYN	AGATATTAAATGTA
M0016<br>2	V$OCT1_06	0.83437<br>5	1169	(-)	CWNAWTKWSATRYN	GATATTAAATGTAA
M0016<br>2	V$OCT1_06	0.86054<br>7	1173	(+)	CWNAWTKWSATRYN	TTAAATGTAATTTA
M0019<br>5	V$OCT1_Q6	0.83133<br>2	1173	(+)	NNNNATGCAAATNAN	TTAAATGTAATTTAC
M0016<br>2	V$OCT1_06	0.94531<br>2	1174	(-)	CWNAWTKWSATRYN	TAAATGTAATTTAC
M0024<br>8	V$OCT1_07	0.81238<br>1	1174	(+)	TNTATGNTAATT	TAAATGTAATTT
M0013<br>8	V$OCT1_04	0.80447<br>5	1195	(-)	NNNNNNNWATGCAAATNNNWNN<br>W	TTTTAGTTTATCAATTTTAACAA

M00138	V$OCT1_04	0.780636	1200	(+)	NNNNNNNWATGCAAATNNNWNNW	GTTTATCAATTTTAACAAATCCA
M00138	V$OCT1_04	0.800711	1201	(-)	NNNNNNNWATGCAAATNNNWNNW	TTTATCAATTTTAACAAATCCAA
M00162	V$OCT1_06	0.885547	1215	(-)	CWNAWTKWSATRYN	CAAATCCAATGATC
M00162	V$OCT1_06	0.834375	1234	(+)	CWNAWTKWSATRYN	GGAAATTTCTTTTT
M00138	V$OCT1_04	0.796947	1236	(-)	NNNNNNNWATGCAAATNNNWNNW	AAATTTCTTTTTAATTTTTTTTG
M00162	V$OCT1_06	0.813672	1240	(+)	CWNAWTKWSATRYN	TTCTTTTTAATTTT
M00138	V$OCT1_04	0.810958	1247	(-)	NNNNNNNWATGCAAATNNNWNNW	TAATTTTTTTTGTAGTATTTTAA
M00138	V$OCT1_04	0.785027	1255	(+)	NNNNNNNWATGCAAATNNNWNNW	TTTGTAGTATTTTAAAATTTGTA
M00138	V$OCT1_04	0.802175	1256	(-)	NNNNNNNWATGCAAATNNNWNNW	TTGTAGTATTTTAAAATTTGTAA
M00162	V$OCT1_06	0.81875	1260	(+)	CWNAWTKWSATRYN	AGTATTTTAAAATT
M00162	V$OCT1_06	0.834375	1260	(-)	CWNAWTKWSATRYN	AGTATTTTAAAATT
M00162	V$OCT1_06	0.800391	1261	(+)	CWNAWTKWSATRYN	GTATTTTAAAATTT
M00162	V$OCT1_06	0.865625	1262	(-)	CWNAWTKWSATRYN	TATTTTAAAATTTG
M00138	V$OCT1_04	0.807612	1264	(-)	NNNNNNNWATGCAAATNNNWNNW	TTTTAAAATTTGTAATATTTAAA
M00138	V$OCT1_04	0.816813	1265	(-)	NNNNNNNWATGCAAATNNNWNNW	TTTAAAATTTGTAATATTTAAAT
M00162	V$OCT1_06	0.903516	1269	(+)	CWNAWTKWSATRYN	AAATTTGTAATATT
M00138	V$OCT1_04	0.7821	1270	(+)	NNNNNNNWATGCAAATNNNWNNW	AATTTGTAATATTTAAATATTGA
M00162	V$OCT1_06	0.849609	1270	(-)	CWNAWTKWSATRYN	AATTTGTAATATTT
M00137	V$OCT1_03	0.855788	1272	(+)	NNNRTAATNANNN	TTTGTAATATTTA
M00138	V$OCT1_04	0.813676	1272	(+)	NNNNNNNWATGCAAATNNNWNNW	TTTGTAATATTTAAATATTGATG
M00138	V$OCT1_04	0.865119	1273	(-)	NNNNNNNWATGCAAATNNNWNNW	TTGTAATATTTAAATATTGATGC
M00135	V$OCT1_01	0.804273	1274	(+)	NNNNWTATGCAAATNTNNN	TGTAATATTTAAATATTGA
M00136	V$OCT1_02	0.817001	1274	(+)	NNGAATATKCANNNN	TGTAATATTTAAATA
M00135	V$OCT1_01	0.820679	1275	(-)	NNNNWTATGCAAATNTNNN	GTAATATTTAAATATTGAT
M00162	V$OCT1_06	0.9375	1277	(+)	CWNAWTKWSATRYN	AATATTTAAATATT
M00162	V$OCT1_06	0.9375	1277	(-)	CWNAWTKWSATRYN	AATATTTAAATATT
M00136	V$OCT1_02	0.857607	1279	(-)	NNGAATATKCANNNN	TATTTAAATATTGAT
M00138	V$OCT1_04	0.7867	1279	(-)	NNNNNNNWATGCAAATNNNWNNW	TATTTAAATATTGATGCTTCTCT
M00136	V$OCT1_02	0.772063	1282	(+)	NNGAATATKCANNNN	TTAAATATTGATGCT
M00162	V$OCT1_06	0.803906	1283	(+)	CWNAWTKWSATRYN	TAAATATTGATGCT
M00162	V$OCT1_06	0.807031	1332	(+)	CWNAWTKWSATRYN	CAGGATCAAATGCT
M00162	V$OCT1_06	0.842187	1333	(-)	CWNAWTKWSATRYN	AGGATCAAATGCTT
M00162	V$OCT1_06	0.834766	1365	(+)	CWNAWTKWSATRYN	GATATTGGTATTTC

M00135	V$OCT1_01	0.735979	1391	(+)	NNNNWTATGCAAATNTNNN	AGCACTAAGCAAAATAATT
M00138	V$OCT1_04	0.83187	1400	(-)	NNNNNNNWATGCAAATNNNWNNW	CAAAATAATTTGAATGGTAAA-TA
M00162	V$OCT1_06	0.839844	1404	(+)	CWNAWTKWSATRYN	ATAATTTGAATGGT
M00195	V$OCT1_Q6	0.81905	1404	(-)	NNNNATGCAAATNAN	ATAATTTGAATGGTA
M00138	V$OCT1_04	0.806566	1405	(+)	NNNNNNNWATGCAAATNNNWNNW	TAATTTGAATGGTAAATATTTAT
M00248	V$OCT1_07	0.843969	1406	(-)	TNTATGNTAATT	AATTTGAATGGT
M00248	V$OCT1_07	0.843969	1410	(+)	TNTATGNTAATT	TGAATGGTAAAT
M00136	V$OCT1_02	0.815918	1413	(-)	NNGAATATKCANNNN	ATGGTAAATATTTAT
M00137	V$OCT1_03	0.860925	1413	(+)	NNNRTAATNANNN	ATGGTAAATATTT
M00138	V$OCT1_04	0.80343	1414	(+)	NNNNNNNWATGCAAATNNNWNNW	TGGTAAATATTTATATTGAA-GAG
M00136	V$OCT1_02	0.826475	1416	(+)	NNGAATATKCANNNN	GTAAATATTTATATT
M00162	V$OCT1_06	0.88125	1437	(+)	CWNAWTKWSATRYN	CAAAATTAAAAACT
M00162	V$OCT1_06	0.821484	1438	(-)	CWNAWTKWSATRYN	AAAATTAAAAACTA
M00138	V$OCT1_04	0.804057	1445	(+)	NNNNNNNWATGCAAATNNNWNNW	AAAACTAAATGAATAAAAA-TATT
M00162	V$OCT1_06	0.800391	1445	(-)	CWNAWTKWSATRYN	AAAACTAAATGAAT
M00138	V$OCT1_04	0.792765	1449	(+)	NNNNNNNWATGCAAATNNNWNNW	CTAAATGAATAAAAA-TATTACTT
M00136	V$OCT1_02	0.828912	1450	(-)	NNGAATATKCANNNN	TAAATGAATAAAAAT
M00138	V$OCT1_04	0.830615	1450	(-)	NNNNNNNWATGCAAATNNNWNNW	TAAATGAATAAAAA-TATTACTTT
M00137	V$OCT1_03	0.857369	1454	(+)	NNNRTAATNANNN	TGAATAAAAATAT
M00162	V$OCT1_06	0.860547	1454	(-)	CWNAWTKWSATRYN	TGAATAAAAATATT
M00136	V$OCT1_02	0.783974	1456	(-)	NNGAATATKCANNNN	AATAAAAATATTACT
M00248	V$OCT1_07	0.812381	1456	(-)	TNTATGNTAATT	AATAAAAATATT
M00162	V$OCT1_06	0.836719	1461	(+)	CWNAWTKWSATRYN	AAATATTACTTTCA
M00162	V$OCT1_06	0.834375	1465	(+)	CWNAWTKWSATRYN	ATTACTTTCAAGTG
M00135	V$OCT1_01	0.736742	1482	(-)	NNNNWTATGCAAATNTNNN	CAACTTTTATCATAATATA
M00138	V$OCT1_04	0.785655	1511	(-)	NNNNNNNWATGCAAATNNNWNNW	GGAAAAATTAAGAATTTTTTTTT
M00162	V$OCT1_06	0.800391	1514	(-)	CWNAWTKWSATRYN	AAAATTAAGAATTT
M00136	V$OCT1_02	0.781267	1517	(-)	NNGAATATKCANNNN	ATTAAGAATTTTTTT
M00138	V$OCT1_04	0.801338	1522	(-)	NNNNNNNWATGCAAATNNNWNNW	GAATTTTTTTTTCATGAATCAAA
M00162	V$OCT1_06	0.834766	1535	(+)	CWNAWTKWSATRYN	ATGAATCAAATTTT
M00162	V$OCT1_06	0.88125	1536	(-)	CWNAWTKWSATRYN	TGAATCAAATTTTA
M00162	V$OCT1_06	0.865625	1541	(+)	CWNAWTKWSATRYN	CAAATTTTATTATA
M00137	V$OCT1_03	0.908337	1544	(-)	NNNRTAATNANNN	ATTTTATTATAAG

M00162	V$OCT1_06	0.829297	1561	(+)	CWNAWTKWSATRYN	AACTATTTTATTTT
M00248	V$OCT1_07	0.817257	1562	(+)	TNTATGNTAATT	ACTATTTTATTT
M00138	V$OCT1_04	0.797574	1567	(-)	NNNNNNNWATGCAAATNNNWNNW	TTTATTTTCTTACATAGATCTTG
M00137	V$OCT1_03	0.856183	1606	(-)	NNNRTAATNANNN	TCATCCTTACCTG
M00162	V$OCT1_06	0.85	1607	(+)	CWNAWTKWSATRYN	CATCCTTACCTGTC
M00162	V$OCT1_06	0.800391	1670	(+)	CWNAWTKWSATRYN	AGATTCTAGATTCT
M00135	V$OCT1_01	0.750858	1696	(+)	NNNNWTATGCAAATNTNNN	TCTCAAATGCAATTTGATG
M00162	V$OCT1_06	0.868359	1703	(+)	CWNAWTKWSATRYN	TGCAATTTGATGCA
M00138	V$OCT1_04	0.807612	1704	(+)	NNNNNNNWATGCAAATNNNWNNW	GCAATTT-GATGCAAGTCTCATCA
M00135	V$OCT1_01	0.755246	1706	(+)	NNNNWTATGCAAATNTNNN	AATTTGATGCAAGTCTCAT
M00137	V$OCT1_03	0.884631	1743	(+)	NNNRTAATNANNN	GTCATAAAGAATT
M00162	V$OCT1_06	0.868359	1749	(+)	CWNAWTKWSATRYN	AAGAATTTGATGGT
M00162	V$OCT1_06	0.816406	1750	(-)	CWNAWTKWSATRYN	AGAATTTGATGGTC
M00137	V$OCT1_03	0.858949	1763	(-)	NNNRTAATNANNN	CTCTAATTAGCTA
M00162	V$OCT1_06	0.814062	1772	(-)	CWNAWTKWSATRYN	GCTATTAAAGTGTT
M00162	V$OCT1_06	0.813672	1786	(-)	CWNAWTKWSATRYN	GATATTAAAGCTAT
M00135	V$OCT1_01	0.746662	1792	(+)	NNNNWTATGCAAATNTNNN	AAAGCTATGGTAATCCTTC
M00195	V$OCT1_Q6	0.792849	1794	(+)	NNNNATGCAAATNAN	AGCTATGGTAATCCT
M00161	V$OCT1_05	0.869002	1795	(-)	MKNATTTGCATAYY	GCTATGGTAATCCT
M00248	V$OCT1_07	0.864957	1795	(+)	TNTATGNTAATT	GCTATGGTAATC
M00162	V$OCT1_06	0.821484	1804	(+)	CWNAWTKWSATRYN	ATCCTTCACATTTT
M00195	V$OCT1_Q6	0.812773	1804	(-)	NNNNATGCAAATNAN	ATCCTTCACATTTTG
M00162	V$OCT1_06	0.817187	1805	(-)	CWNAWTKWSATRYN	TCCTTCACATTTTG
M00138	V$OCT1_04	0.791301	1806	(-)	NNNNNNNWATGCAAATNNNWNNW	CCTTCACATTTTGATCATTATTA
M00162	V$OCT1_06	0.809766	1810	(+)	CWNAWTKWSATRYN	CACATTTTGATCAT
M00138	V$OCT1_04	0.783563	1814	(+)	NNNNNNNWATGCAAATNNNWNNW	TTTTGATCATTATTATTCAGTTT
M00137	V$OCT1_03	0.888186	1817	(-)	NNNRTAATNANNN	TGATCATTATTAT
M00137	V$OCT1_03	0.913473	1820	(-)	NNNRTAATNANNN	TCATTATTATTCA
M00138	V$OCT1_04	0.788164	1820	(+)	NNNNNNNWATGCAAATNNNWNNW	TCATTATTATTCAGTTTATTCAA
M00136	V$OCT1_02	0.873849	1822	(+)	NNGAATATKCANNNN	ATTATTATTCAGTTT
M00135	V$OCT1_01	0.746852	1831	(+)	NNNNWTATGCAAATNTNNN	CAGTTTATTCAAAGACTTA
M00136	V$OCT1_02	0.817001	1831	(+)	NNGAATATKCANNNN	CAGTTTATTCAAAGA
M00195	V$OCT1_Q6	0.790393	1833	(+)	NNNNATGCAAATNAN	GTTTATTCAAAGACT

M00162	V$OCT1_06	0.860937	1853	(+)	CWNAWTKWSATRYN	CTAAATAAAATTTC
M00162	V$OCT1_06	0.847656	1854	(-)	CWNAWTKWSATRYN	TAAATAAAATTTCT
M00138	V$OCT1_04	0.787537	1862	(+)	NNNNNNNWATGCAAATNNNWNNW	ATTTCTAACTTGAAA-TATAAACA
M00162	V$OCT1_06	0.855078	1867	(+)	CWNAWTKWSATRYN	TAACTTGAAATATA
M00137	V$OCT1_03	0.864085	1886	(+)	NNNRTAATNANNN	CTCACAATTAAAA
M00162	V$OCT1_06	0.868359	1888	(+)	CWNAWTKWSATRYN	CACAATTAAAAATT
M00162	V$OCT1_06	0.816016	1888	(-)	CWNAWTKWSATRYN	CACAATTAAAAATT
M00162	V$OCT1_06	0.813672	1889	(-)	CWNAWTKWSATRYN	ACAATTAAAAATTT
M00138	V$OCT1_04	0.805939	1891	(+)	NNNNNNNWATGCAAATNNNWNNW	AATTAAAAATTTAAAAAAAAAG
M00138	V$OCT1_04	0.802593	1892	(+)	NNNNNNNWATGCAAATNNNWNNW	ATTAAAAATTTAAAAAAAAA-GA
M00138	V$OCT1_04	0.789628	1892	(-)	NNNNNNNWATGCAAATNNNWNNW	ATTAAAAATTTAAAAAAAAA-GA
M00138	V$OCT1_04	0.783772	1893	(+)	NNNNNNNWATGCAAATNNNWNNW	TTAAAAATTTAAAAAAAAA-GAA
M00135	V$OCT1_01	0.755628	1912	(+)	NNNNWTATGCAAATNTNNN	AGAAATAAGGTAATTAACA
M00248	V$OCT1_07	0.873861	1915	(+)	TNTATGNTAATT	AATAAGGTAATT
M00137	V$OCT1_03	0.844725	1918	(+)	NNNRTAATNANNN	AAGGTAATTAACA
M00248	V$OCT1_07	0.803265	1923	(-)	TNTATGNTAATT	AATTAACAATAC
M00162	V$OCT1_06	0.860547	1942	(-)	CWNAWTKWSATRYN	AGCATCAAAGAAGG
M00138	V$OCT1_04	0.818486	1981	(-)	NNNNNNNWATGCAAATNNNWNNW	TTATAGGCTTAACATATTTGTAA
M00135	V$OCT1_01	0.776994	1983	(-)	NNNNWTATGCAAATNTNNN	ATAGGCTTAACATATTTGT
M00161	V$OCT1_05	0.873651	1985	(+)	MKNATTTGCATAYY	AGGCTTAACATATT
M00162	V$OCT1_06	0.821484	1985	(+)	CWNAWTKWSATRYN	AGGCTTAACATATT
M00195	V$OCT1_Q6	0.826146	1985	(-)	NNNNATGCAAATNAN	AGGCTTAACATATTT
M00138	V$OCT1_04	0.795065	1989	(-)	NNNNNNNWATGCAAATNNNWNNW	TTAACATATTTGTAAACATATAT
M00138	V$OCT1_04	0.780845	1990	(+)	NNNNNNNWATGCAAATNNNWNNW	TAACATATTTGTAAACATA-TATA
M00135	V$OCT1_01	0.759443	1991	(-)	NNNNWTATGCAAATNTNNN	AACATATTTGTAAACATAT
M00138	V$OCT1_04	0.795274	1993	(-)	NNNNNNNWATGCAAATNNNWNNW	CATATTTGTAAACATATATATAT
M00138	V$OCT1_04	0.802593	1996	(+)	NNNNNNNWATGCAAATNNNWNNW	ATTTGTAAACAT-ATATATATCCA
M00137	V$OCT1_03	0.888977	1997	(+)	NNNRTAATNANNN	TTTGTAAACATAT
M00138	V$OCT1_04	0.829987	1999	(-)	NNNNNNNWATGCAAATNNNWNNW	TGTAAACATATATATATCCAA-TA
M00138	V$OCT1_04	0.808867	2000	(+)	NNNNNNNWATGCAAATNNNWNNW	GTAAACATATATATATCCAA-TAT
M00138	V$OCT1_04	0.783354	2005	(-)	NNNNNNNWATGCAAATNNNWNNW	CATATATATATCCAATATTGTAT
M00135	V$OCT1_01	0.739222	2008	(+)	NNNNWTATGCAAATNTNNN	ATATATATCCAATATTGTA
M00136	V$OCT1_02	0.781808	2008	(+)	NNGAATATKCANNNN	ATATATATCCAATAT

M00162	V$OCT1_06	0.9375	2011	(-)	CWNAWTKWSATRYN	TATATCCAATATTG
M00162	V$OCT1_06	0.826562	2018	(+)	CWNAWTKWSATRYN	AATATTGTATTACC
M00135	V$OCT1_01	0.735406	2021	(-)	NNNNWTATGCAAATNTNNN	ATTGTATTACCAAATATAC
M00248	V$OCT1_07	0.795209	2025	(-)	TNTATGNTAATT	TATTACCAAATA
M00162	V$OCT1_06	0.857031	2116	(+)	CWNAWTKWSATRYN	ATCCCTGGGATTCT
M00162	V$OCT1_06	0.868359	2172	(-)	CWNAWTKWSATRYN	TGCATGAAAGTGAA
M00138	V$OCT1_04	0.806148	2173	(+)	NNNNNNNWATGCAAATNNNWNNW	GCATGAAAGTGAAAAGTGAAAGT
M00162	V$OCT1_06	0.877734	2252	(+)	CWNAWTKWSATRYN	ATCCATGGGATTTT
M00162	V$OCT1_06	0.834766	2328	(-)	CWNAWTKWSATRYN	TATATTCCAGTGTA
M00162	V$OCT1_06	0.873437	2338	(-)	CWNAWTKWSATRYN	TGTATCCACTTTTG
M00162	V$OCT1_06	0.862891	2439	(-)	CWNAWTKWSATRYN	AAGATCTAATAATT
M00137	V$OCT1_03	0.854998	2441	(+)	NNNRTAATNANNN	GATCTAATAATTT
M00137	V$OCT1_03	0.886606	2444	(+)	NNNRTAATNANNN	CTAATAATTTGGT
M00138	V$OCT1_04	0.831242	2473	(+)	NNNNNNNWATGCAAATNNNWNNW	ACTAGATAATGTAAAGTAACT-TA
M00137	V$OCT1_03	0.854208	2475	(+)	NNNRTAATNANNN	TAGATAATGTAAA
M00162	V$OCT1_06	0.855078	2477	(+)	CWNAWTKWSATRYN	GATAATGTAAAGTA
M00195	V$OCT1_Q6	0.836517	2477	(+)	NNNNATGCAAATNAN	GATAATGTAAAGTAA
M00248	V$OCT1_07	0.793089	2478	(+)	TNTATGNTAATT	ATAATGTAAAGT
M00162	V$OCT1_06	0.800781	2488	(+)	CWNAWTKWSATRYN	GTAACTTAAGTTTC
M00162	V$OCT1_06	0.852734	2508	(-)	CWNAWTKWSATRYN	AAAAACAAATTCAG
M00136	V$OCT1_02	0.786681	2510	(+)	NNGAATATKCANNNN	AAACAAATTCAGTTA
M00162	V$OCT1_06	0.809375	2513	(+)	CWNAWTKWSATRYN	CAAATTCAGTTATT
M00162	V$OCT1_06	0.808594	2513	(-)	CWNAWTKWSATRYN	CAAATTCAGTTATT
M00162	V$OCT1_06	0.821484	2519	(+)	CWNAWTKWSATRYN	CAGTTATTAATGTG
M00162	V$OCT1_06	0.852344	2524	(+)	CWNAWTKWSATRYN	ATTAATGTGAAACA
M00162	V$OCT1_06	0.800391	2531	(-)	CWNAWTKWSATRYN	TGAAACAAAAAGTT
M00162	V$OCT1_06	0.804297	2536	(+)	CWNAWTKWSATRYN	CAAAAAGTTATTCT
M00162	V$OCT1_06	0.857031	2636	(+)	CWNAWTKWSATRYN	ATCCCTGGGATTCT
M00195	V$OCT1_Q6	0.804039	2691	(+)	NNNNATGCAAATNAN	ATGCATGAAAGTGAG
M00162	V$OCT1_06	0.950781	2692	(-)	CWNAWTKWSATRYN	TGCATGAAAGTGAG
M00162	V$OCT1_06	0.808594	2721	(+)	CWNAWTKWSATRYN	CGCTCAGTCATGTC
M00162	V$OCT1_06	0.877734	2779	(+)	CWNAWTKWSATRYN	GTCCATGGGATTTT
M00138	V$OCT1_04	0.842953	2842	(+)	NNNNNNNWATGCAAATNNNWNNW	TCTAGATAATGATAAATAAA-TAA

M00137	V$OCT1_03	0.930462	2844	(+)	NNNRTAATNANNN	TAGATAATGATAA
M00138	V$OCT1_04	0.794647	2844	(-)	NNNNNNNWATGCAAATNNNWNNW	TAGATAATGATAAATAAA-TAAAT
M00138	V$OCT1_04	0.79151	2845	(+)	NNNNNNNWATGCAAATNNNWNNW	AGATAATGATAAATAAATAAA-TA
M00137	V$OCT1_03	0.846306	2850	(+)	NNNRTAATNANNN	ATGATAAATAAAT
M00137	V$OCT1_03	0.855788	2858	(+)	NNNRTAATNANNN	TAAATAAATAGGA
M00162	V$OCT1_06	0.821484	2878	(-)	CWNAWTKWSATRYN	GACAAGAAAGTGAT
M00162	V$OCT1_06	0.834375	2881	(+)	CWNAWTKWSATRYN	AAGAAAGTGATTCA
M00138	V$OCT1_04	0.820995	2882	(+)	NNNNNNNWATGCAAATNNNWNNW	AGAAAGTGATTCAAATAAGA-TAA
M00195	V$OCT1_Q6	0.825328	2886	(+)	NNNNATGCAAATNAN	AGTGATTCAAATAAG
M00138	V$OCT1_04	0.799247	2902	(+)	NNNNNNNWATGCAAATNNNWNNW	TAATAGTTTTGGATATTTGGACA
M00135	V$OCT1_01	0.795879	2903	(-)	NNNNWTATGCAAATNTNNN	AATAGTTTTGGATATTTGG
M00162	V$OCT1_06	0.834375	2905	(+)	CWNAWTKWSATRYN	TAGTTTTGGATATT
M00136	V$OCT1_02	0.791825	2907	(-)	NNGAATATKCANNNN	GTTTTGGATATTTGG
M00135	V$OCT1_01	0.786341	2911	(-)	NNNNWTATGCAAATNTNNN	TGGATATTTGGACACTCAA
M00162	V$OCT1_06	0.839453	2913	(+)	CWNAWTKWSATRYN	GATATTTGGACACT
M00162	V$OCT1_06	0.829297	2929	(+)	CWNAWTKWSATRYN	AACTATCAAATATA
M00162	V$OCT1_06	0.865625	2930	(-)	CWNAWTKWSATRYN	ACTATCAAATATAG
M00162	V$OCT1_06	0.817187	2935	(+)	CWNAWTKWSATRYN	CAAATATAGATGAA
M00138	V$OCT1_04	0.810749	2936	(+)	NNNNNNNWATGCAAATNNNWNNW	AAA-TATAGATGAAAAAGTTTCTG
M00162	V$OCT1_06	0.842187	2941	(-)	CWNAWTKWSATRYN	TAGATGAAAAAGTT
M00162	V$OCT1_06	0.898437	2952	(+)	CWNAWTKWSATRYN	GTTTCTGAAATGCT
M00162	V$OCT1_06	0.855078	2960	(+)	CWNAWTKWSATRYN	AATGCTGAGATATT
M00135	V$OCT1_01	0.738268	2965	(+)	NNNNWTATGCAAATNTNNN	TGAGATATTCTATTGTTAA
M00162	V$OCT1_06	0.821875	2968	(-)	CWNAWTKWSATRYN	GATATTCTATTGTT
M00138	V$OCT1_04	0.810121	2986	(-)	NNNNNNNWATGCAAATNNNWNNW	TCTTATTTTCTAAATTGTAAATA
M00195	V$OCT1_Q6	0.813865	2996	(+)	NNNNATGCAAATNAN	TAAATTGTAAATAAT
M00162	V$OCT1_06	0.847266	2997	(-)	CWNAWTKWSATRYN	AAATTGTAAATAAT
M00136	V$OCT1_02	0.783162	2999	(-)	NNGAATATKCANNNN	ATTGTAAATAATGAT
M00137	V$OCT1_03	0.883445	3003	(+)	NNNRTAATNANNN	TAAATAATGATTG
M00162	V$OCT1_06	0.950391	3074	(-)	CWNAWTKWSATRYN	AGCATCAAAAGATG
M00135	V$OCT1_01	0.809996	3079	(+)	NNNNWTATGCAAATNTNNN	CAAAAGATGGAAATATCTG
M00195	V$OCT1_Q6	0.796943	3081	(+)	NNNNATGCAAATNAN	AAAGATGGAAATATC
M00162	V$OCT1_06	0.821484	3082	(+)	CWNAWTKWSATRYN	AAGATGGAAATATC

M00162	V$OCT1_06	0.812109	3082	(-)	CWNAWTKWSATRYN	AAGATGGAAATATC
M00162	V$OCT1_06	0.865625	3092	(-)	CWNAWTKWSATRYN	TATCTGACAGTAAG
M00162	V$OCT1_06	0.826953	3116	(-)	CWNAWTKWSATRYN	AGGATCCAAGTTCT
M00162	V$OCT1_06	0.821484	3205	(+)	CWNAWTKWSATRYN	GATTCTTTAATCCT
M00138	V$OCT1_04	0.80092	3231	(-)	NNNNNNNWATGCAAATNNNWNNW	ACATCATTTTTTAATGCTAACAT
M00162	V$OCT1_06	0.9375	3235	(+)	CWNAWTKWSATRYN	CATTTTTTAATGCT
M00135	V$OCT1_01	0.746852	3238	(+)	NNNNWTATGCAAATNTNNN	TTTTTAATGCTAACATTTA
M00162	V$OCT1_06	0.842187	3249	(-)	CWNAWTKWSATRYN	AACATTTAACAAAC
M00138	V$OCT1_04	0.802802	3250	(-)	NNNNNNNWATGCAAATNNNWNNW	ACATTTAACAAACATAAATCTTG
M00248	V$OCT1_07	0.797329	3252	(-)	TNTATGNTAATT	ATTTAACAAACA
M00248	V$OCT1_07	0.792453	3256	(-)	TNTATGNTAATT	AACAAACATAAA
M00138	V$OCT1_04	0.815349	3279	(+)	NNNNNNNWATGCAAATNNNWNNW	CAGTTAAAATGCAGATTGAG-TTA
M00135	V$OCT1_01	0.758298	3281	(+)	NNNNWTATGCAAATNTNNN	GTTAAAATGCAGATTGAGT
M00136	V$OCT1_02	0.842989	3281	(+)	NNGAATATKCANNNN	GTTAAAATGCAGATT
M00136	V$OCT1_02	0.802653	3284	(-)	NNGAATATKCANNNN	AAAATGCAGATTGAG
M00248	V$OCT1_07	0.799873	3284	(+)	TNTATGNTAATT	AAAATGCAGATT
M00138	V$OCT1_04	0.790882	3289	(+)	NNNNNNNWATGCAAATNNNWNNW	GCAGATTGAG-TTAAAATTTTATA
M00162	V$OCT1_06	0.826562	3295	(-)	CWNAWTKWSATRYN	TGAGTTAAAATTTT
M00138	V$OCT1_04	0.797365	3298	(-)	NNNNNNNWATGCAAATNNNWNNW	GTTAAAATTTTATATAATTTAAT
M00135	V$OCT1_01	0.754865	3300	(-)	NNNNWTATGCAAATNTNNN	TAAAATTTTATATAATTTA
M00162	V$OCT1_06	0.825	3300	(+)	CWNAWTKWSATRYN	TAAAATTTTATATA
M00162	V$OCT1_06	0.868359	3310	(+)	CWNAWTKWSATRYN	TATAATTTAATTTA
M00248	V$OCT1_07	0.831673	3310	(+)	TNTATGNTAATT	TATAATTTAATT
M00138	V$OCT1_04	0.787118	3311	(+)	NNNNNNNWATGCAAATNNNWNNW	ATAATTTAATTTATGA-TAAAAAA
M00162	V$OCT1_06	0.847656	3311	(-)	CWNAWTKWSATRYN	ATAATTTAATTTAT
M00138	V$OCT1_04	0.781054	3313	(-)	NNNNNNNWATGCAAATNNNWNNW	AATTTAATTTATGATAAAAAA-TA
M00138	V$OCT1_04	0.847762	3315	(+)	NNNNNNNWATGCAAATNNNWNNW	TTTAATTTATGATAAAAAA-TAAA
M00136	V$OCT1_02	0.776665	3332	(+)	NNGAATATKCANNNN	AATAAAATCCAGACA
M00162	V$OCT1_06	0.855078	3349	(+)	CWNAWTKWSATRYN	CAGTATTTCAGATT
M00162	V$OCT1_06	0.842187	3350	(-)	CWNAWTKWSATRYN	AGTATTTCAGATTA
M00137	V$OCT1_03	0.845516	3355	(-)	NNNRTAATNANNN	TTCAGATTATTTT
M00138	V$OCT1_04	0.80803	3365	(-)	NNNNNNNWATGCAAATNNNWNNW	TTTTTGTCTTTTTATATACTTTT
M00138	V$OCT1_04	0.795483	3371	(-)	NNNNNNNWATGCAAATNNNWNNW	TCTTTTTATATACTTTTCTCCAC

M00135	V$OCT1_01	0.759443	3391	(+)	NNNNWTATGCAAATNTNNN	CACCATATTCTAAAAACAG
M00136	V$OCT1_02	0.802653	3391	(+)	NNGAATATKCANNNN	CACCATATTCTAAAA
M00162	V$OCT1_06	0.834375	3412	(+)	CWNAWTKWSATRYN	GATAATTTACTTTT
M00162	V$OCT1_06	0.834375	3413	(+)	CWNAWTKWSATRYN	ATAATTTACTTTTC
M00195	V$OCT1_Q6	0.840338	3413	(-)	NNNNATGCAAATNAN	ATAATTTACTTTTCT
M00162	V$OCT1_06	0.831641	3421	(+)	CWNAWTKWSATRYN	CTTTTCTTGATTTT
M00162	V$OCT1_06	0.865625	3470	(-)	CWNAWTKWSATRYN	GGTGTCAAATTTAG
M00162	V$OCT1_06	0.894141	3475	(+)	CWNAWTKWSATRYN	CAAATTTAGCTGTT
M00138	V$OCT1_04	0.79381	3478	(+)	NNNNNNNWATGCAAATNNNWNNW	ATTTAGCTGTTAAAATACAAACT
M00162	V$OCT1_06	0.940234	3511	(+)	CWNAWTKWSATRYN	CACTATTAAATGTA
M00138	V$OCT1_04	0.826223	3512	(+)	NNNNNNNWATGCAAATNNNWNNW	AC-TATTAAATGTATAGTATTACA
M00162	V$OCT1_06	0.826562	3512	(-)	CWNAWTKWSATRYN	ACTATTAAATGTAT
M00136	V$OCT1_02	0.772604	3517	(-)	NNGAATATKCANNNN	TAAATGTATAGTATT
M00162	V$OCT1_06	0.821094	3517	(-)	CWNAWTKWSATRYN	TAAATGTATAGTAT
M00162	V$OCT1_06	0.865625	3525	(+)	CWNAWTKWSATRYN	TAGTATTACATGTG
M00162	V$OCT1_06	0.834375	3526	(-)	CWNAWTKWSATRYN	AGTATTACATGTGC
M00162	V$OCT1_06	0.826562	3537	(+)	CWNAWTKWSATRYN	TGCCTTGTGATATT
M00138	V$OCT1_04	0.804893	3541	(-)	NNNNNNNWATGCAAATNNNWNNW	TTGTGATATTATTATTATTGTAT
M00138	V$OCT1_04	0.791719	3544	(-)	NNNNNNNWATGCAAATNNNWNNW	TGATATTATTATTATTGTATTTT
M00137	V$OCT1_03	0.906361	3546	(-)	NNNRTAATNANNN	ATATTATTATTAT
M00138	V$OCT1_04	0.791092	3546	(+)	NNNNNNNWATGCAAATNNNWNNW	ATATTATTATTATTGTATTTTGA
M00137	V$OCT1_03	0.873568	3549	(-)	NNNRTAATNANNN	TTATTATTATTGT
M00162	V$OCT1_06	0.805859	3554	(+)	CWNAWTKWSATRYN	ATTATTGTATTTTG
M00248	V$OCT1_07	0.815137	3554	(+)	TNTATGNTAATT	ATTATTGTATTT
M00138	V$OCT1_04	0.783772	3556	(-)	NNNNNNNWATGCAAATNNNWNNW	TATTGTATTTTGAGTGCTTTTGG
M00162	V$OCT1_06	0.800391	3560	(+)	CWNAWTKWSATRYN	GTATTTTGAGTGCT
M00162	V$OCT1_06	0.805859	3577	(-)	CWNAWTKWSATRYN	GGTTTTACAATTCT
M00138	V$OCT1_04	0.806775	3581	(-)	NNNNNNNWATGCAAATNNNWNNW	TTACAATTCTTGCATTTTTTTTT
M00135	V$OCT1_01	0.765929	3583	(-)	NNNNWTATGCAAATNTNNN	ACAATTCTTGCATTTTTTT
M00162	V$OCT1_06	0.885547	3585	(+)	CWNAWTKWSATRYN	AATTCTTGCATTTT
M00136	V$OCT1_02	0.850568	3587	(-)	NNGAATATKCANNNN	TTCTTGCATTTTTTT
M00138	V$OCT1_04	0.814304	3595	(-)	NNNNNNNWATGCAAATNNNWNNW	TTTTTTTTTTAACAGAAACATCC
M00162	V$OCT1_06	0.804297	3611	(-)	CWNAWTKWSATRYN	AACATCCTATCAAG

M00162	V$OCT1_06	0.80625	3671	(+)	CWNAWTKWSATRYN	CTCACTGTAAATTG
M00248	V$OCT1_07	0.817257	3672	(+)	TNTATGNTAATT	TCACTGTAAATT
M00138	V$OCT1_04	0.804684	3698	(+)	NNNNNNNWATGCAAATNNNWNNW	ATGATAAATT-GCTAATATATATA
M00135	V$OCT1_01	0.738459	3700	(+)	NNNNWTATGCAAATNTNNN	GATAAATTGCTAATATATA
M00138	V$OCT1_04	0.783982	3707	(-)	NNNNNNNWATGCAAATNNNWNNW	TGCTAATATATATATTGTAGTCT
M00162	V$OCT1_06	0.809375	3709	(+)	CWNAWTKWSATRYN	CTAATATATATATT
M00162	V$OCT1_06	0.842578	3756	(+)	CWNAWTKWSATRYN	CAGTTTCACATTCG
M00137	V$OCT1_03	0.847886	3774	(+)	NNNRTAATNANNN	GGTGTAATATCTT
M00136	V$OCT1_02	0.900379	3795	(-)	NNGAATATKCANNNN	GAGCTGAATATTGAT
M00136	V$OCT1_02	0.814023	3798	(+)	NNGAATATKCANNNN	CTGAATATTGATCTG
M00195	V$OCT1_Q6	0.80131	3839	(-)	NNNNATGCAAATNAN	GGCATTTGGACTTAC
M00138	V$OCT1_04	0.835843	3841	(-)	NNNNNNNWATGCAAATNNNWNNW	CATTTGGACTTACATATTTATGA
M00135	V$OCT1_01	0.809996	3843	(-)	NNNNWTATGCAAATNTNNN	TTTGGACTTACATATTTAT
M00162	V$OCT1_06	0.855469	3845	(+)	CWNAWTKWSATRYN	TGGACTTACATATT
M00195	V$OCT1_Q6	0.80786	3845	(-)	NNNNATGCAAATNAN	TGGACTTACATATTT
M00136	V$OCT1_02	0.828912	3847	(-)	NNGAATATKCANNNN	GACTTACATATTTAT
M00138	V$OCT1_04	0.784818	3848	(+)	NNNNNNNWATGCAAATNNNWNNW	ACTTACATATTTATGATTAATAA
M00137	V$OCT1_03	0.886606	3852	(-)	NNNRTAATNANNN	ACATATTTATGAT
M00138	V$OCT1_04	0.856545	3852	(+)	NNNNNNNWATGCAAATNNNWNNW	ACATATTTATGATTAA-TAAAAAT
M00138	V$OCT1_04	0.785236	3855	(+)	NNNNNNNWATGCAAATNNNWNNW	TATTTATGATTAATAAAAATTTT
M00137	V$OCT1_03	0.870802	3858	(-)	NNNRTAATNANNN	TTATGATTAATAA
M00138	V$OCT1_04	0.82936	3859	(+)	NNNNNNNWATGCAAATNNNWNNW	TATGATTAATAAAAATTTTATTA
M00138	V$OCT1_04	0.819741	3860	(-)	NNNNNNNWATGCAAATNNNWNNW	ATGATTAATAAAAATTTTATTAT
M00137	V$OCT1_03	0.851047	3864	(+)	NNNRTAATNANNN	TTAATAAAAATTT
M00162	V$OCT1_06	0.826562	3870	(+)	CWNAWTKWSATRYN	AAAATTTTATTATG
M00162	V$OCT1_06	0.881641	3870	(-)	CWNAWTKWSATRYN	AAAATTTTATTATG
M00137	V$OCT1_03	0.92098	3873	(-)	NNNRTAATNANNN	ATTTTATTATGCA
M00138	V$OCT1_04	0.783354	3873	(+)	NNNNNNNWATGCAAATNNNWNNW	ATTTTATTATGCAAGAGCAG-TAG
M00135	V$OCT1_01	0.794163	3875	(+)	NNNNWTATGCAAATNTNNN	TTTATTATGCAAGAGCAGT
M00136	V$OCT1_02	0.838116	3875	(+)	NNGAATATKCANNNN	TTTATTATGCAAGAG
M00136	V$OCT1_02	0.802653	3898	(-)	NNGAATATKCANNNN	AAACAGAATATGTAT
M00138	V$OCT1_04	0.788582	3899	(+)	NNNNNNNWATGCAAATNNNWNNW	AACAGAA-TATGTATATGTGGTCT
M00135	V$OCT1_01	0.757917	3900	(-)	NNNNWTATGCAAATNTNNN	ACAGAATATGTATATGTGG

M00135	V$OCT1_01	0.80351	3901	(+)	NNNNWTATGCAAATNTNNN	CAGAATATGTATATGTGGT
M00136	V$OCT1_02	0.854629	3901	(+)	NNGAATATKCANNNN	CAGAATATGTATATG
M00162	V$OCT1_06	0.88125	3904	(-)	CWNAWTKWSATRYN	AATATGTATATGTG
M00162	V$OCT1_06	0.805469	3930	(+)	CWNAWTKWSATRYN	GTATTATTGATTCT
M00162	V$OCT1_06	0.834375	3967	(+)	CWNAWTKWSATRYN	AAACATATGATTTA
M00162	V$OCT1_06	0.812109	3968	(-)	CWNAWTKWSATRYN	AACATATGATTTAC
M00195	V$OCT1_Q6	0.831332	3973	(-)	NNNNATGCAAATNAN	ATGATTTACAACTTG
M00135	V$OCT1_01	0.785006	3989	(-)	NNNNWTATGCAAATNTNNN	TCTCAATTTACACACTGAG
M00248	V$OCT1_07	0.852025	3993	(-)	TNTATGNTAATT	AATTTACACACT
M00136	V$OCT1_02	0.785869	4000	(-)	NNGAATATKCANNNN	ACACTGAGTATTAAA
M00136	V$OCT1_02	0.774228	4003	(+)	NNGAATATKCANNNN	CTGAGTATTAAATTA
M00162	V$OCT1_06	0.803516	4005	(+)	CWNAWTKWSATRYN	GAGTATTAAATTAG
M00162	V$OCT1_06	0.804297	4006	(-)	CWNAWTKWSATRYN	AGTATTAAATTAGA
M00162	V$OCT1_06	0.894141	4010	(+)	CWNAWTKWSATRYN	TTAAATTAGATACT
M00162	V$OCT1_06	0.808594	4011	(-)	CWNAWTKWSATRYN	TAAATTAGATACTC
M00138	V$OCT1_04	0.797156	4075	(-)	NNNNNNNWATGCAAATNNNWNNW	AGTTCATTTTTAATGTTTACAG
M00162	V$OCT1_06	0.9375	4079	(+)	CWNAWTKWSATRYN	CATTTTTAATGTT
M00138	V$OCT1_04	0.782518	4080	(+)	NNNNNNNWATGCAAATNNNWNNW	ATTTTTAATGTTTACAGAATGA
M00137	V$OCT1_03	0.843935	4086	(-)	NNNRTAATNANNN	TAATGTTTACAGA
M00137	V$OCT1_03	0.854208	4127	(-)	NNNRTAATNANNN	CTTTCTTTACCCC
M00138	V$OCT1_04	0.780636	4156	(+)	NNNNNNNWATGCAAATNNNWNNW	AGTTAGCTATGATAAA-TACTGAA
M00248	V$OCT1_07	0.790545	4161	(+)	TNTATGNTAATT	GCTATGATAAAT
M00136	V$OCT1_02	0.829995	4171	(-)	NNGAATATKCANNNN	ATACTGAATATCTTC
M00138	V$OCT1_04	0.819113	4182	(-)	NNNNNNNWATGCAAATNNNWNNW	CTTCTTCATTTCAATTTTCATTT
M00162	V$OCT1_06	0.847266	4185	(+)	CWNAWTKWSATRYN	CTTCATTTCAATTT
M00162	V$OCT1_06	0.868359	4186	(-)	CWNAWTKWSATRYN	TTCATTTCAATTTT
M00138	V$OCT1_04	0.863655	4188	(-)	NNNNNNNWATGCAAATNNNWNNW	CATTTCAATTTTCATTTGTATAC
M00135	V$OCT1_01	0.781	4190	(-)	NNNNWTATGCAAATNTNNN	TTTCAATTTTCATTTGTAT
M00136	V$OCT1_02	0.770709	4191	(+)	NNGAATATKCANNNN	TTCAATTTTCATTTG
M00195	V$OCT1_Q6	0.7994	4192	(-)	NNNNATGCAAATNAN	TCAATTTTCATTTGT
M00138	V$OCT1_04	0.836261	4194	(-)	NNNNNNNWATGCAAATNNNWNNW	AATTTTCATTTGTATACCTTCAT
M00248	V$OCT1_07	0.793937	4194	(-)	TNTATGNTAATT	AATTTTCATTTG
M00135	V$OCT1_01	0.765357	4196	(-)	NNNNWTATGCAAATNTNNN	TTTTCATTTGTATACCTTC

M00195	V$OCT1_Q6	0.858897	4198	(-)	NNNNATGCAAATNAN	TTCATTTGTATACCT
M00162	V$OCT1_06	0.834375	4211	(+)	CWNAWTKWSATRYN	CTTCATTTCAATTG
M00138	V$OCT1_04	0.787746	4218	(+)	NNNNNNNWATGCAAATNNNWNNW	TCAATTGTATCCAGA-TACTCAGA
M00162	V$OCT1_06	0.81875	4223	(-)	CWNAWTKWSATRYN	TGTATCCAGATACT
M00137	V$OCT1_03	0.876333	4242	(-)	NNNRTAATNANNN	AGTTGATTACCCT
M00137	V$OCT1_03	0.897669	4267	(-)	NNNRTAATNANNN	TTTTCATTACATT
M00162	V$OCT1_06	0.85	4268	(+)	CWNAWTKWSATRYN	TTTCATTACATTCC
M00162	V$OCT1_06	0.834766	4269	(-)	CWNAWTKWSATRYN	TTCATTACATTCCT
M00137	V$OCT1_03	0.84354	4304	(+)	NNNRTAATNANNN	TTGATAAGTATGA
M00162	V$OCT1_06	0.821094	4310	(-)	CWNAWTKWSATRYN	AGTATGAACTGACT
M00137	V$OCT1_03	0.870012	4336	(+)	NNNRTAATNANNN	CTGGTAAAGAAGG
M00162	V$OCT1_06	0.800391	4362	(+)	CWNAWTKWSATRYN	GTCTCTTTCAGATT
M00162	V$OCT1_06	0.839844	4364	(+)	CWNAWTKWSATRYN	CTCTTTCAGATTTT
M00138	V$OCT1_04	0.794856	4367	(+)	NNNNNNNWATGCAAATNNNWNNW	TTTCAGATTTTAAAA-TAAAATCA
M00162	V$OCT1_06	0.852734	4370	(+)	CWNAWTKWSATRYN	CAGATTTTAAAATA
M00162	V$OCT1_06	0.805859	4377	(-)	CWNAWTKWSATRYN	TAAAATAAAATCAT
M00162	V$OCT1_06	0.805859	4383	(-)	CWNAWTKWSATRYN	AAAATCATAAAACT
M00162	V$OCT1_06	0.855078	4396	(-)	CWNAWTKWSATRYN	TGAAACTAATTATC
M00137	V$OCT1_03	0.912288	4399	(-)	NNNRTAATNANNN	AACTAATTATCTC
M00138	V$OCT1_04	0.845462	4408	(-)	NNNNNNNWATGCAAATNNNWNNW	TCTCTTATTTTACCTAAGAAATA
M00162	V$OCT1_06	0.839453	4427	(+)	CWNAWTKWSATRYN	AATATTTTGGTTTA
M00138	V$OCT1_04	0.811167	4429	(-)	NNNNNNNWATGCAAATNNNWNNW	TATTTTGGTTTACCTAAATAAAT
M00138	V$OCT1_04	0.802802	4432	(+)	NNNNNNNWATGCAAATNNNWNNW	TTTGGTTTACCTAAA-TAAATGGA
M00138	V$OCT1_04	0.780427	4442	(+)	NNNNNNNWATGCAAATNNNWNNW	CTAAATAAATGGAGAATTT-GTGT
M00138	V$OCT1_04	0.813676	4455	(+)	NNNNNNNWATGCAAATNNNWNNW	GAATTT-GTGTTCAAATGGAAAAA
M00138	V$OCT1_04	0.828315	4461	(+)	NNNNNNNWATGCAAATNNNWNNW	GTGTTCAAATGGAAAAACATTCT
M00162	V$OCT1_06	0.832031	4490	(+)	CWNAWTKWSATRYN	CTGACTGTGTTTTT
M00162	V$OCT1_06	0.925	4517	(+)	CWNAWTKWSATRYN	CACAATTTAATTCC
M00162	V$OCT1_06	0.821875	4518	(-)	CWNAWTKWSATRYN	ACAATTTAATTCCT
M00138	V$OCT1_04	0.792974	4538	(+)	NNNNNNNWATGCAAATNNNWNNW	AG-TCCTCAATGAAAATTTACTCA
M00135	V$OCT1_01	0.736742	4540	(+)	NNNNWTATGCAAATNTNNN	TCCTCAATGAAAATTTACT
M00162	V$OCT1_06	0.847656	4542	(+)	CWNAWTKWSATRYN	CTCAATGAAAATTT
M00136	V$OCT1_02	0.779643	4543	(-)	NNGAATATKCANNNN	TCAATGAAAATTTAC

M00162	V$OCT1_06	0.804297	4543	(-)	CWNAWTKWSATRYN	TCAATGAAAATTTA
M00248	V$OCT1_07	0.843545	4543	(+)	TNTATGNTAATT	TCAATGAAAATT
M00136	V$OCT1_02	0.815918	4546	(+)	NNGAATATKCANNNN	ATGAAAATTTACTCA
M00162	V$OCT1_06	0.85	4604	(-)	CWNAWTKWSATRYN	CAAATGTATTTTTC
M00138	V$OCT1_04	0.813049	4609	(+)	NNNNNNNWATGCAAATNNNWNNW	GTATTTTTCTGGAAAAATCAACT
M00135	V$OCT1_01	0.735025	4611	(+)	NNNNWTATGCAAATNTNNN	ATTTTTCTGGAAAAATCAA
M00138	V$OCT1_04	0.80343	4633	(-)	NNNNNNNWATGCAAATNNNWNNW	AATTTCTATTTACAAACATTTTT
M00248	V$OCT1_07	0.816197	4633	(-)	TNTATGNTAATT	AATTTCTATTTA
M00135	V$OCT1_01	0.741511	4635	(-)	NNNNWTATGCAAATNTNNN	TTTCTATTTACAAACATTT
M00137	V$OCT1_03	0.88226	4636	(-)	NNNRTAATNANNN	TTCTATTTACAAA
M00138	V$OCT1_04	0.809494	4637	(-)	NNNNNNNWATGCAAATNNNWNNW	TCTATTTACAAACATTTTTTCAC
M00162	V$OCT1_06	0.834375	4649	(+)	CWNAWTKWSATRYN	CATTTTTTCACTTG
M00138	V$OCT1_04	0.781263	4654	(+)	NNNNNNNWATGCAAATNNNWNNW	TTTCACTTGGTCAAATATTTTCA
M00138	V$OCT1_04	0.805312	4663	(-)	NNNNNNNWATGCAAATNNNWNNW	GTCAAATATTTTCATTTACTCAC
M00135	V$OCT1_01	0.764975	4665	(-)	NNNNWTATGCAAATNTNNN	CAAATATTTTCATTTACTC
M00162	V$OCT1_06	0.9375	4667	(+)	CWNAWTKWSATRYN	AATATTTTCATTTA
M00195	V$OCT1_Q6	0.822325	4667	(-)	NNNNATGCAAATNAN	AATATTTTCATTTAC
M00248	V$OCT1_07	0.812381	4667	(+)	TNTATGNTAATT	AATATTTTCATT
M00136	V$OCT1_02	0.83189	4696	(-)	NNGAATATKCANNNN	ACAAAGAATATTGAA
M00138	V$OCT1_04	0.836888	4697	(+)	NNNNNNNWATGCAAATNNNWNNW	CAAAGAATATTGAAATTATA-TAT
M00135	V$OCT1_01	0.767264	4699	(+)	NNNNWTATGCAAATNTNNN	AAGAATATTGAAATTATAT
M00136	V$OCT1_02	0.856524	4699	(+)	NNGAATATKCANNNN	AAGAATATTGAAATT
M00195	V$OCT1_Q6	0.814956	4701	(+)	NNNNATGCAAATNAN	GAATATTGAAATTAT
M00161	V$OCT1_05	0.897393	4702	(-)	MKNATTTGCATAYY	AATATTGAAATTAT
M00162	V$OCT1_06	0.847656	4702	(+)	CWNAWTKWSATRYN	AATATTGAAATTAT
M00162	V$OCT1_06	0.852734	4702	(-)	CWNAWTKWSATRYN	AATATTGAAATTAT
M00248	V$OCT1_07	0.889124	4702	(+)	TNTATGNTAATT	AATATTGAAATT
M00138	V$OCT1_04	0.817022	4709	(-)	NNNNNNNWATGCAAATNNNWNNW	AAATTATATATGCAAA-TATTAAA
M00138	V$OCT1_04	0.928691	4710	(+)	NNNNNNNWATGCAAATNNNWNNW	AATTATATATGCAAA-TATTAAAA
M00135	V$OCT1_01	0.7562	4711	(-)	NNNNWTATGCAAATNTNNN	ATTATATATGCAAATATTA
M00135	V$OCT1_01	0.953453	4712	(+)	NNNNWTATGCAAATNTNNN	TTATATATGCAAATATTAA
M00136	V$OCT1_02	0.837845	4712	(+)	NNGAATATKCANNNN	TTATATATGCAAATA
M00195	V$OCT1_Q6	0.889465	4714	(+)	NNNNATGCAAATNAN	ATATATGCAAATATT

M00162	V$OCT1_06	0.9375	4715	(-)	CWNAWTKWSATRYN	TATATGCAAATATT
M00248	V$OCT1_07	0.873225	4715	(+)	TNTATGNTAATT	TATATGCAAATA
M00136	V$OCT1_02	0.775853	4717	(-)	NNGAATATKCANNNN	TATGCAAATATTAAA
M00138	V$OCT1_04	0.786282	4718	(+)	NNNNNNNWATGCAAATNNNWNNW	ATGCAAATATTAAAAA-GCTGCTT
M00162	V$OCT1_06	0.869922	4723	(-)	CWNAWTKWSATRYN	AATATTAAAAAGCT
M00162	V$OCT1_06	0.916797	4735	(+)	CWNAWTKWSATRYN	CTGCTTTAAATATT
M00195	V$OCT1_Q6	0.807314	4735	(-)	NNNNATGCAAATNAN	CTGCTTTAAATATTA
M00136	V$OCT1_02	0.822144	4737	(-)	NNGAATATKCANNNN	GCTTTAAATATTAAT
M00138	V$OCT1_04	0.781054	4737	(-)	NNNNNNNWATGCAAATNNNWNNW	GCTTTAAATATTAATCTGTACCT
M00162	V$OCT1_06	0.804687	4741	(-)	CWNAWTKWSATRYN	TAAATATTAATCTG
M00138	V$OCT1_04	0.80092	4757	(-)	NNNNNNNWATGCAAATNNNWNNW	CCTATACATTTGACTCTCTGTAA
M00162	V$OCT1_06	0.813281	4760	(+)	CWNAWTKWSATRYN	ATACATTTGACTCT
M00162	V$OCT1_06	0.805859	4770	(+)	CWNAWTKWSATRYN	CTCTCTGTAAAACA
M00138	V$OCT1_04	0.812631	4789	(+)	NNNNNNNWATGCAAATNNNWNNW	GCTGTTTTGTGCATAATCTATGT
M00135	V$OCT1_01	0.785387	4790	(-)	NNNNWTATGCAAATNTNNN	CTGTTTTGTGCATAATCTA
M00136	V$OCT1_02	0.889821	4794	(-)	NNGAATATKCANNNN	TTTGTGCATAATCTA
M00138	V$OCT1_04	0.802175	4800	(+)	NNNNNNNWATGCAAATNNNWNNW	CATAATCTATGTAAACTTTACAA
M00135	V$OCT1_01	0.743228	4802	(+)	NNNNWTATGCAAATNTNNN	TAATCTATGTAAACTTTAC
M00248	V$OCT1_07	0.871317	4805	(+)	TNTATGNTAATT	TCTATGTAAACT
M00138	V$OCT1_04	0.799038	4809	(-)	NNNNNNNWATGCAAATNNNWNNW	TGTAAACTTTACAATTTCCATTC
M00162	V$OCT1_06	0.828906	4812	(+)	CWNAWTKWSATRYN	AAACTTTACAATTT
M00162	V$OCT1_06	0.847266	4813	(-)	CWNAWTKWSATRYN	AACTTTACAATTTC
M00138	V$OCT1_04	0.850481	4815	(-)	NNNNNNNWATGCAAATNNNWNNW	CTTTACAATTTCCATTCTCATTA
M00135	V$OCT1_01	0.79607	4817	(-)	NNNNWTATGCAAATNTNNN	TTACAATTTCCATTCTCAT
M00161	V$OCT1_05	0.869334	4819	(+)	MKNATTTGCATAYY	ACAATTTCCATTCT
M00195	V$OCT1_Q6	0.83488	4819	(-)	NNNNATGCAAATNAN	ACAATTTCCATTCTC
M00248	V$OCT1_07	0.898664	4821	(-)	TNTATGNTAATT	AATTTCCATTCT
M00137	V$OCT1_03	0.962465	4829	(-)	NNNRTAATNANNN	TTCTCATTATGAT
M00162	V$OCT1_06	0.801953	4831	(+)	CWNAWTKWSATRYN	CTCATTATGATGGA
M00137	V$OCT1_03	0.843935	4883	(+)	NNNRTAATNANNN	TCTGTAATTTCAT
M00162	V$OCT1_06	0.808594	4894	(+)	CWNAWTKWSATRYN	ATGACTGAATTGTC
M00136	V$OCT1_02	0.789388	4895	(-)	NNGAATATKCANNNN	TGACTGAATTGTCAA
M00162	V$OCT1_06	0.834375	4900	(-)	CWNAWTKWSATRYN	GAATTGTCAAATAG

M00138	V$OCT1_04	0.806985	4913	(+)	NNNNNNNWATGCAAATNNNWNNW	GAACACAAA-TAGAAATTTTATTT
M00135	V$OCT1_01	0.7314	4915	(+)	NNNNWTATGCAAATNTNNN	ACACAAATAGAAATTTTAT
M00162	V$OCT1_06	0.860547	4918	(+)	CWNAWTKWSATRYN	CAAATAGAAATTTT
M00162	V$OCT1_06	0.834766	4923	(+)	CWNAWTKWSATRYN	AGAAATTTTATTTC
M00162	V$OCT1_06	0.826562	4924	(+)	CWNAWTKWSATRYN	GAAATTTTATTTCA
M00138	V$OCT1_04	0.783982	4925	(-)	NNNNNNNWATGCAAATNNNWNNW	AAATTTTATTTCAAGA-CAAGTAT
M00138	V$OCT1_04	0.833752	4938	(+)	NNNNNNNWATGCAAATNNNWNNW	AGACAAGTATTTAAAAGATTT-GA
M00162	V$OCT1_06	0.890625	4943	(-)	CWNAWTKWSATRYN	AGTATTTAAAAGAT
M00138	V$OCT1_04	0.836888	4980	(-)	NNNNNNNWATGCAAATNNNWNNW	TGAATGATTTTTAATATAATCTT
M00162	V$OCT1_06	0.826562	4984	(+)	CWNAWTKWSATRYN	TGATTTTTAATATA
M00162	V$OCT1_06	0.868359	5038	(-)	CWNAWTKWSATRYN	GGTAAGAAATTCTG
M00136	V$OCT1_02	0.794261	5051	(-)	NNGAATATKCANNNN	GAACAGAATATACTG
M00138	V$OCT1_04	0.797574	5055	(+)	NNNNNNNWATGCAAATNNNWNNW	AGAATATACTG-CAGAATTAACAA
M00248	V$OCT1_07	0.862201	5069	(-)	TNTATGNTAATT	AATTAACAAAGC
M00136	V$OCT1_02	0.789117	5074	(-)	NNGAATATKCANNNN	ACAAAGCATTTTATT
M00162	V$OCT1_06	0.829297	5077	(+)	CWNAWTKWSATRYN	AAGCATTTTATTCT
M00162	V$OCT1_06	0.807031	5078	(-)	CWNAWTKWSATRYN	AGCATTTTATTCTA
M00162	V$OCT1_06	0.804297	5088	(-)	CWNAWTKWSATRYN	TCTATGTTATTTAT
M00248	V$OCT1_07	0.895272	5088	(+)	TNTATGNTAATT	TCTATGTTATTT
M00138	V$OCT1_04	0.83166	5100	(+)	NNNNNNNWATGCAAATNNNWNNW	ATGGTGTTATGCCAATTCTTTAT
M00135	V$OCT1_01	0.80351	5102	(+)	NNNNWTATGCAAATNTNNN	GGTGTTATGCCAATTCTTT
M00195	V$OCT1_Q6	0.798581	5104	(+)	NNNNATGCAAATNAN	TGTTATGCCAATTCT
M00161	V$OCT1_05	0.890586	5105	(-)	MKNATTTGCATAYY	GTTATGCCAATTCT
M00162	V$OCT1_06	0.826953	5105	(-)	CWNAWTKWSATRYN	GTTATGCCAATTCT
M00248	V$OCT1_07	0.869833	5105	(+)	TNTATGNTAATT	GTTATGCCAATT
M00162	V$OCT1_06	0.834375	5117	(+)	CWNAWTKWSATRYN	CTTTATTGCCTTTT
M00162	V$OCT1_06	0.800391	5137	(-)	CWNAWTKWSATRYN	AAAATCACTTAGAT
M00162	V$OCT1_06	0.826953	5140	(+)	CWNAWTKWSATRYN	ATCACTTAGATTAT
M00137	V$OCT1_03	0.885421	5147	(-)	NNNRTAATNANNN	AGATTATTATTAG
M00135	V$OCT1_01	0.736169	5150	(-)	NNNNWTATGCAAATNTNNN	TTATTATTAGATTATTGAA
M00137	V$OCT1_03	0.847096	5150	(-)	NNNRTAATNANNN	TTATTATTAGATT
M00195	V$OCT1_Q6	0.795306	5177	(+)	NNNNATGCAAATNAN	AGAGATGAAGATAAC
M00138	V$OCT1_04	0.791092	5214	(-)	NNNNNNNWATGCAAATNNNWNNW	ATGAAAGATAATAA-TAGTCAAAT

M00137	V$OCT1_03	0.867641	5218	(+)	NNNRTAATNANNN	AAGATAATAATAG
M00138	V$OCT1_04	0.828524	5221	(+)	NNNNNNNWATGCAAATNNNWNNW	ATAATAA-TAGTCAAATTTACTGG
M00135	V$OCT1_01	0.763258	5223	(+)	NNNNWTATGCAAATNTNNN	AATAATAGTCAAATTTACT
M00136	V$OCT1_02	0.817001	5223	(+)	NNGAATATKCANNNN	AATAATAGTCAAATT
M00248	V$OCT1_07	0.795209	5226	(+)	TNTATGNTAATT	AATAGTCAAATT
M00138	V$OCT1_04	0.811585	5245	(+)	NNNNNNNWATGCAAATNNNWNNW	ACAAAATTTTTTAAATATTTTTA
M00138	V$OCT1_04	0.830615	5246	(-)	NNNNNNNWATGCAAATNNNWNNW	CAAAATTTTTTAAATATTTTTAA
M00162	V$OCT1_06	0.903516	5250	(+)	CWNAWTKWSATRYN	ATTTTTTAAATATT
M00136	V$OCT1_02	0.831077	5252	(-)	NNGAATATKCANNNN	TTTTTAAATATTTTT
M00138	V$OCT1_04	0.795483	5253	(+)	NNNNNNNWATGCAAATNNNWNNW	TTTTAAATATTTTTAAGCACACA
M00137	V$OCT1_03	0.856974	5273	(+)	NNNRTAATNANNN	ACAGTAATGAACT
M00137	V$OCT1_03	0.84275	5293	(+)	NNNRTAATNANNN	TAAATAAAGAAAA
M00138	V$OCT1_04	0.781054	5327	(+)	NNNNNNNWATGCAAATNNNWNNW	GAAATAATATTGCAAAAAA-TATT
M00138	V$OCT1_04	0.843998	5328	(+)	NNNNNNNWATGCAAATNNNWNNW	AAATAATATTGCAAAAAA-TATTA
M00135	V$OCT1_01	0.753338	5357	(-)	NNNNWTATGCAAATNTNNN	TCACAAAAAGCATATTTAT
M00136	V$OCT1_02	0.875744	5361	(-)	NNGAATATKCANNNN	AAAAAGCATATTTAT
M00136	V$OCT1_02	0.841635	5364	(+)	NNGAATATKCANNNN	AAGCATATTTATTTT
M00162	V$OCT1_06	0.804297	5367	(+)	CWNAWTKWSATRYN	CATATTTATTTTTT
M00162	V$OCT1_06	0.842187	5367	(-)	CWNAWTKWSATRYN	CATATTTATTTTTT
M00138	V$OCT1_04	0.78189	5369	(-)	NNNNNNNWATGCAAATNNNWNNW	TATTTATTTTTTAATTTGCAGCA
M00162	V$OCT1_06	0.821484	5373	(+)	CWNAWTKWSATRYN	TATTTTTTAATTTG
M00248	V$OCT1_07	0.831673	5373	(+)	TNTATGNTAATT	TATTTTTTAATT
M00138	V$OCT1_04	0.863446	5375	(-)	NNNNNNNWATGCAAATNNNWNNW	TTTTTTAATTTGCAGCAGCAAAA
M00135	V$OCT1_01	0.744182	5377	(-)	NNNNWTATGCAAATNTNNN	TTTTAATTTGCAGCAGCAA
M00162	V$OCT1_06	0.873437	5393	(+)	CWNAWTKWSATRYN	CAAAATGTAAGATA
M00162	V$OCT1_06	0.898437	5394	(-)	CWNAWTKWSATRYN	AAAATGTAAGATAC
M00162	V$OCT1_06	0.821094	5404	(+)	CWNAWTKWSATRYN	ATACATTTCTTTTT
M00162	V$OCT1_06	0.862891	5405	(-)	CWNAWTKWSATRYN	TACATTTCTTTTTT
M00138	V$OCT1_04	0.808867	5427	(+)	NNNNNNNWATGCAAATNNNWNNW	TTGATGGAATGAAAAAAAATTTT
M00162	V$OCT1_06	0.834375	5432	(-)	CWNAWTKWSATRYN	GGAATGAAAAAAAA
M00138	V$OCT1_04	0.793392	5440	(-)	NNNNNNNWATGCAAATNNNWNNW	AAAAAATTTTATTATATTCATTT
M00162	V$OCT1_06	0.839453	5442	(+)	CWNAWTKWSATRYN	AAAATTTTATTATA
M00137	V$OCT1_03	0.870407	5445	(-)	NNNRTAATNANNN	ATTTTATTATATT

M00138	V$OCT1_04	0.839189	5446	(-)	NNNNNNNWATGCAAATNNNWNNW	TTTTATTATATTCATTTTCTCCA
M00136	V$OCT1_02	0.840282	5449	(+)	NNGAATATKCANNNN	TATTATATTCATTTT
M00162	V$OCT1_06	0.839453	5450	(+)	CWNAWTKWSATRYN	ATTATATTCATTTT
M00136	V$OCT1_02	0.776936	5452	(-)	NNGAATATKCANNNN	TATATTCATTTTCTC
M00162	V$OCT1_06	0.813672	5452	(-)	CWNAWTKWSATRYN	TATATTCATTTTCT
M00162	V$OCT1_06	0.868359	5464	(+)	CWNAWTKWSATRYN	CTCCATTTTATGTT
M00137	V$OCT1_03	0.877124	5492	(-)	NNNRTAATNANNN	TTATCTTTACTAT
M00138	V$OCT1_04	0.843371	5497	(-)	NNNNNNNWATGCAAATNNNWNNW	TTTACTATCTTGCATTATCAAAT
M00135	V$OCT1_01	0.749905	5499	(-)	NNNNWTATGCAAATNTNNN	TACTATCTTGCATTATCAA
M00162	V$OCT1_06	0.826953	5501	(+)	CWNAWTKWSATRYN	CTATCTTGCATTAT
M00136	V$OCT1_02	0.818896	5503	(-)	NNGAATATKCANNNN	ATCTTGCATTATCAA
M00162	V$OCT1_06	0.808203	5520	(-)	CWNAWTKWSATRYN	GACAACTCAGAAAT
M00162	V$OCT1_06	0.803125	5523	(+)	CWNAWTKWSATRYN	AACTCAGAAATGCA
M00137	V$OCT1_03	0.861715	5552	(+)	NNNRTAATNANNN	TTGGTAAAGTGGA
M00162	V$OCT1_06	0.839844	5580	(+)	CWNAWTKWSATRYN	CTGTCTTTGATCTA
M00162	V$OCT1_06	0.877734	5589	(+)	CWNAWTKWSATRYN	ATCTATTTAATTCA
M00195	V$OCT1_Q6	0.791212	5589	(+)	NNNNATGCAAATNAN	ATCTATTTAATTCAG
M00162	V$OCT1_06	0.873828	5590	(-)	CWNAWTKWSATRYN	TCTATTTAATTCAG
M00162	V$OCT1_06	0.808203	5614	(-)	CWNAWTKWSATRYN	AAAACGCCATATAC
M00162	V$OCT1_06	0.833984	5624	(+)	CWNAWTKWSATRYN	ATACATTTCTTATA
M00162	V$OCT1_06	0.855078	5625	(-)	CWNAWTKWSATRYN	TACATTTCTTATAG
M00138	V$OCT1_04	0.806357	5626	(-)	NNNNNNNWATGCAAATNNNWNNW	ACATTTCTTATAGATATCCATTC
M00138	V$OCT1_04	0.784191	5627	(+)	NNNNNNNWATGCAAATNNNWNNW	CATTTCTTATAGATATCCATTCA
M00138	V$OCT1_04	0.809285	5632	(-)	NNNNNNNWATGCAAATNNNWNNW	CTTATAGATATCCATTCATTCAT
M00136	V$OCT1_02	0.808609	5635	(+)	NNGAATATKCANNNN	ATAGATATCCATTCA
M00162	V$OCT1_06	0.855078	5644	(+)	CWNAWTKWSATRYN	CATTCATTCATGTT
M00248	V$OCT1_07	0.792453	5708	(-)	TNTATGNTAATT	AAGGAACATAAA
M00162	V$OCT1_06	0.868359	5715	(+)	CWNAWTKWSATRYN	ATAAATGATATTTA
M00138	V$OCT1_04	0.860728	5716	(+)	NNNNNNNWATGCAAATNNNWNNW	TAAATGATATTTAAATTATTTTA
M00162	V$OCT1_06	0.804297	5716	(-)	CWNAWTKWSATRYN	TAAATGATATTTAA
M00138	V$OCT1_04	0.846717	5717	(-)	NNNNNNNWATGCAAATNNNWNNW	AAATGATATTTAAATTATTTTAA
M00135	V$OCT1_01	0.734071	5718	(+)	NNNNWTATGCAAATNTNNN	AATGATATTTAAATTATTT
M00136	V$OCT1_02	0.794532	5718	(+)	NNGAATATKCANNNN	AATGATATTTAAATT

M00135	V$OCT1_01	0.7438	5719	(-)	NNNNWTATGCAAATNTNNN	ATGATATTTAAATTATTTT
M00195	V$OCT1_Q6	0.804312	5720	(+)	NNNNATGCAAATNAN	TGATATTTAAATTAT
M00162	V$OCT1_06	0.847656	5721	(+)	CWNAWTKWSATRYN	GATATTTAAATTAT
M00162	V$OCT1_06	0.916797	5721	(-)	CWNAWTKWSATRYN	GATATTTAAATTAT
M00248	V$OCT1_07	0.872589	5721	(+)	TNTATGNTAATT	GATATTTAAATT
M00138	V$OCT1_04	0.806148	5725	(+)	NNNNNNNWATGCAAATNNNWNNW	TTTAAATTATTTTAAAGTTATCT
M00138	V$OCT1_04	0.781681	5727	(-)	NNNNNNNWATGCAAATNNNWNNW	TAAATTATTTTAAAGTTATCTCA
M00162	V$OCT1_06	0.839453	5730	(+)	CWNAWTKWSATRYN	ATTATTTTAAAGTT
M00162	V$OCT1_06	0.81875	5732	(-)	CWNAWTKWSATRYN	TATTTTAAAGTTAT
M00162	V$OCT1_06	0.834375	5741	(-)	CWNAWTKWSATRYN	GTTATCTCAAAATC
M00162	V$OCT1_06	0.831641	5743	(-)	CWNAWTKWSATRYN	TATCTCAAAATCAT
M00162	V$OCT1_06	0.825391	5748	(+)	CWNAWTKWSATRYN	CAAAATCATATTTT
M00162	V$OCT1_06	0.886719	5749	(-)	CWNAWTKWSATRYN	AAAATCATATTTTG
M00137	V$OCT1_03	0.841169	5783	(+)	NNNRTAATNANNN	TTGACAAAGAGAA
M00138	V$OCT1_04	0.803639	5803	(-)	NNNNNNNWATGCAAATNNNWNNW	GGGAAAAATTTTCG-TATGAAAAT
M00135	V$OCT1_01	0.765929	5805	(-)	NNNNWTATGCAAATNTNNN	GAAAAATTTTCGTATGAAA
M00162	V$OCT1_06	0.847266	5807	(+)	CWNAWTKWSATRYN	AAAATTTTCGTATG
M00138	V$OCT1_04	0.854245	5810	(+)	NNNNNNNWATGCAAATNNNWNNW	ATTTTCGTATGAAAATAC-GAAAA
M00135	V$OCT1_01	0.824876	5812	(+)	NNNNWTATGCAAATNTNNN	TTTCGTATGAAAATACGAA
M00195	V$OCT1_Q6	0.811135	5814	(+)	NNNNATGCAAATNAN	TCGTATGAAAATACG
M00161	V$OCT1_05	0.872987	5815	(-)	MKNATTTGCATAYY	CGTATGAAAATACG
M00162	V$OCT1_06	0.942969	5815	(-)	CWNAWTKWSATRYN	CGTATGAAAATACG
M00138	V$OCT1_04	0.801129	5818	(+)	NNNNNNNWATGCAAATNNNWNNW	ATGAAAATAC-GAAAATTAAGGTG
M00135	V$OCT1_01	0.739603	5820	(+)	NNNNWTATGCAAATNTNNN	GAAAATACGAAAATTAAGG
M00195	V$OCT1_Q6	0.800764	5822	(+)	NNNNATGCAAATNAN	AAATACGAAAATTAA
M00138	V$OCT1_04	0.782936	5835	(+)	NNNNNNNWATGCAAATNNNWNNW	AAGGTGG-TATGTCAAAAAATGCT
M00162	V$OCT1_06	0.855078	5840	(-)	CWNAWTKWSATRYN	GGTATGTCAAAAAA
M00162	V$OCT1_06	0.852344	5842	(-)	CWNAWTKWSATRYN	TATGTCAAAAAATG
M00138	V$OCT1_04	0.7844	5858	(+)	NNNNNNNWATGCAAATNNNWNNW	TGTGCAGTATGAAAAACATTT-TA
M00135	V$OCT1_01	0.753338	5860	(+)	NNNNWTATGCAAATNTNNN	TGCAGTATGAAAAACATTT
M00162	V$OCT1_06	0.842187	5862	(+)	CWNAWTKWSATRYN	CAGTATGAAAAACA
M00195	V$OCT1_Q6	0.822325	5862	(+)	NNNNATGCAAATNAN	CAGTATGAAAAACAT
M00161	V$OCT1_05	0.881953	5863	(-)	MKNATTTGCATAYY	AGTATGAAAAACAT

M00162	V$OCT1_06	0.903516	5863	(-)	CWNAWTKWSATRYN	AGTATGAAAAACAT
M00162	V$OCT1_06	0.966016	5886	(+)	CWNAWTKWSATRYN	AAAAATTTCATGTA
M00162	V$OCT1_06	0.842187	5887	(-)	CWNAWTKWSATRYN	AAAATTTCATGTAA
M00162	V$OCT1_06	0.821484	5917	(-)	CWNAWTKWSATRYN	GAACTTTCAAAATG
M00138	V$OCT1_04	0.788582	5945	(-)	NNNNNNNWATGCAAATNNNWNNW	CTGAGAATTCTGAATATTGATTT
M00135	V$OCT1_01	0.758108	5947	(-)	NNNNWTATGCAAATNTNNN	GAGAATTCTGAATATTGAT
M00162	V$OCT1_06	0.800391	5949	(+)	CWNAWTKWSATRYN	GAATTCTGAATATT
M00136	V$OCT1_02	0.919058	5951	(-)	NNGAATATKCANNNN	ATTCTGAATATTGAT
M00136	V$OCT1_02	0.829453	5954	(+)	NNGAATATKCANNNN	CTGAATATTGATTTC
M00138	V$OCT1_04	0.806775	5997	(-)	NNNNNNNWATGCAAATNNNWNNW	ACAAGGCATTAG-TATAAATGAAT
M00135	V$OCT1_01	0.802556	5999	(-)	NNNNWTATGCAAATNTNNN	AAGGCATTAGTATAAATGA
M00248	V$OCT1_07	0.800297	6003	(-)	TNTATGNTAATT	CATTAGTATAAA
M00162	V$OCT1_06	0.834375	6011	(-)	CWNAWTKWSATRYN	TAAATGAATGAATG
M00162	V$OCT1_06	0.842187	6031	(-)	CWNAWTKWSATRYN	AGAATGAATTAGTG
M00162	V$OCT1_06	0.839453	6104	(-)	CWNAWTKWSATRYN	AGTACTACATGAAG
M00138	V$OCT1_04	0.855709	6133	(-)	NNNNNNNWATGCAAATNNNWNNW	AA-GAATGTTTTACATAGTCACAA
M00135	V$OCT1_01	0.730446	6135	(-)	NNNNWTATGCAAATNTNNN	GAATGTTTTACATAGTCAC
M00162	V$OCT1_06	0.834375	6137	(+)	CWNAWTKWSATRYN	ATGTTTTACATAGT
M00195	V$OCT1_Q6	0.850437	6137	(-)	NNNNATGCAAATNAN	ATGTTTTACATAGTC
M00162	V$OCT1_06	0.808203	6138	(-)	CWNAWTKWSATRYN	TGTTTTACATAGTC
M00136	V$OCT1_02	0.790471	6139	(-)	NNGAATATKCANNNN	GTTTTACATAGTCAC
M00162	V$OCT1_06	0.808203	6199	(-)	CWNAWTKWSATRYN	TGTTTTTCAAAGTT
M00137	V$OCT1_03	0.852627	6222	(-)	NNNRTAATNANNN	TTGTTATTAAAAC
M00248	V$OCT1_07	0.797329	6231	(-)	TNTATGNTAATT	AAACAACATACA
M00138	V$OCT1_04	0.852363	6238	(+)	NNNNNNNWATGCAAATNNNWNNW	ATA-CAGGTATTTAAAAATTTACA
M00138	V$OCT1_04	0.782727	6239	(-)	NNNNNNNWATGCAAATNNNWNNW	TAC-AGGTATTTAAAAATTTACAC
M00162	V$OCT1_06	0.826562	6243	(+)	CWNAWTKWSATRYN	GGTATTTAAAAATT
M00162	V$OCT1_06	0.898437	6243	(-)	CWNAWTKWSATRYN	GGTATTTAAAAATT
M00138	V$OCT1_04	0.829987	6247	(-)	NNNNNNNWATGCAAATNNNWNNW	TTTAAAAATTTACACCTTCTTAA
M00137	V$OCT1_03	0.845516	6263	(+)	NNNRTAATNANNN	TTCTTAATTATTC
M00137	V$OCT1_03	0.884631	6264	(-)	NNNRTAATNANNN	TCTTAATTATTCT
M00138	V$OCT1_04	0.792346	6266	(-)	NNNNNNNWATGCAAATNNNWNNW	TTAATTATTCTTCATACCTGACT
M00135	V$OCT1_01	0.770126	6283	(+)	NNNNWTATGCAAATNTNNN	CTGACTAAGTAAATTTTCT

M00248	V$OCT1_07	0.852025	6286	(+)	TNTATGNTAATT	ACTAAGTAAATT
M00136	V$OCT1_02	0.813481	6288	(-)	NNGAATATKCANNNNN	TAAGTAAATTTTCTT
M00138	V$OCT1_04	0.806357	6336	(+)	NNNNNNNWATGCAAATNNNWNNW	GATTCTTTATTCTAAAACTATTA
M00135	V$OCT1_01	0.761923	6338	(+)	NNNNWTATGCAAATNTNNN	TTCTTTATTCTAAAACTAT
M00162	V$OCT1_06	0.90625	6351	(+)	CWNAWTKWSATRYN	AACTATTAAATATA
M00138	V$OCT1_04	0.795274	6362	(+)	NNNNNNNWATGCAAATNNNWNNW	ATAATATAAGGGAAATAAA-GAAG
M00137	V$OCT1_03	0.855788	6373	(+)	NNNRTAATNANNN	GAAATAAAGAAGT
M00138	V$OCT1_04	0.81765	6385	(+)	NNNNNNNWATGCAAATNNNWNNW	TAAAAAATATCCTAATCAAA-TAT
M00135	V$OCT1_01	0.789203	6387	(+)	NNNNWTATGCAAATNTNNN	AAAAATATCCTAATCAAAT
M00136	V$OCT1_02	0.776936	6387	(+)	NNGAATATKCANNNNN	AAAAATATCCTAATC
M00195	V$OCT1_Q6	0.812227	6389	(+)	NNNNATGCAAATNAN	AAATATCCTAATCAA
M00137	V$OCT1_03	0.891347	6393	(+)	NNNRTAATNANNN	ATCCTAATCAAAT
M00162	V$OCT1_06	0.804687	6395	(+)	CWNAWTKWSATRYN	CCTAATCAAATATC
M00162	V$OCT1_06	0.800781	6396	(-)	CWNAWTKWSATRYN	CTAATCAAATATCC
M00136	V$OCT1_02	0.790742	6397	(-)	NNGAATATKCANNNNN	TAATCAAATATCCTT
M00138	V$OCT1_04	0.792346	6407	(-)	NNNNNNNWATGCAAATNNNWNNW	TCCTTTAAGTATCCTAATCAAAA
M00138	V$OCT1_04	0.786491	6409	(+)	NNNNNNNWATGCAAATNNNWNNW	CTTTAAGTATCCTAATCAAAATG
M00135	V$OCT1_01	0.790538	6411	(+)	NNNNWTATGCAAATNTNNN	TTAAGTATCCTAATCAAAA
M00195	V$OCT1_Q6	0.804585	6413	(+)	NNNNATGCAAATNAN	AAGTATCCTAATCAA
M00137	V$OCT1_03	0.884631	6417	(+)	NNNRTAATNANNN	ATCCTAATCAAAA
M00162	V$OCT1_06	0.821094	6430	(-)	CWNAWTKWSATRYN	TGAATGAACAATTC
M00162	V$OCT1_06	0.813672	6452	(-)	CWNAWTKWSATRYN	CAAAACTAAAACAG
M00162	V$OCT1_06	0.821094	6465	(-)	CWNAWTKWSATRYN	GATATCTCTAATTC
M00138	V$OCT1_04	0.825178	6467	(+)	NNNNNNNWATGCAAATNNNWNNW	TATCTCTAATTCAAA-GAAAAAAA
M00138	V$OCT1_04	0.792765	6483	(+)	NNNNNNNWATGCAAATNNNWNNW	AAAAAAA-GATGCAACATGTATGT
M00136	V$OCT1_02	0.78641	6485	(+)	NNGAATATKCANNNNN	AAAAAGATGCAACAT
M00162	V$OCT1_06	0.804297	6510	(+)	CWNAWTKWSATRYN	CAAAATTGGCTGGA
M00162	V$OCT1_06	0.814062	6552	(-)	CWNAWTKWSATRYN	TAAATTCTATGGAG
M00162	V$OCT1_06	0.855469	6564	(-)	CWNAWTKWSATRYN	AGCATCTCACTGCT
M00162	V$OCT1_06	0.813672	6613	(-)	CWNAWTKWSATRYN	CATACTACATGGAG
M00136	V$OCT1_02	0.778018	6621	(-)	NNGAATATKCANNNNN	ATGGAGAAAATTCAT
M00136	V$OCT1_02	0.891716	6624	(+)	NNGAATATKCANNNNN	GAGAAAATTCATTTT
M00162	V$OCT1_06	0.834375	6625	(+)	CWNAWTKWSATRYN	AGAAAATTCATTTT

M00162	V$OCT1_06	0.855469	6626	(-)	CWNAWTKWSATRYN	GAAAATTCATTTTG
M00138	V$OCT1_04	0.809912	6628	(-)	NNNNNNNNWATGCAAATNNNWNNW	AAATTCATTTTGCTTAAATATTT
M00135	V$OCT1_01	0.779855	6630	(-)	NNNNWTATGCAAATNTNNN	ATTCATTTTGCTTAAATAT
M00138	V$OCT1_04	0.805312	6632	(-)	NNNNNNNNWATGCAAATNNNWNNW	TCATTTTGCTTAAATATTTATAC
M00136	V$OCT1_02	0.840552	6638	(-)	NNGAATATKCANNNN	TGCTTAAATATTTAT
M00138	V$OCT1_04	0.824341	6638	(-)	NNNNNNNNWATGCAAATNNNWNNW	TGCTTAAATATTTATACATCATT
M00136	V$OCT1_02	0.810233	6641	(+)	NNGAATATKCANNNN	TTAAATATTTATACA
M00138	V$OCT1_04	0.833124	6642	(-)	NNNNNNNNWATGCAAATNNNWNNW	TAAATATTTATACATCATTGAAT
M00137	V$OCT1_03	0.853418	6643	(-)	NNNRTAATNANNN	AAATATTTATACA
M00195	V$OCT1_Q6	0.808406	6667	(-)	NNNNATGCAAATNAN	ATGCTTTGTGTACTT
M00138	V$OCT1_04	0.788582	6678	(-)	NNNNNNNNWATGCAAATNNNWNNW	ACTTTTATTTATCACATAATCTT
M00137	V$OCT1_03	0.853023	6680	(-)	NNNRTAATNANNN	TTTTATTTATCAC
M00162	V$OCT1_06	0.847266	6685	(-)	CWNAWTKWSATRYN	TTTATCACATAATC
M00136	V$OCT1_02	0.785328	6686	(-)	NNGAATATKCANNNN	TTATCACATAATCTT
M00137	V$OCT1_03	0.866851	6690	(+)	NNNRTAATNANNN	CACATAATCTTTA
M00162	V$OCT1_06	0.808594	6700	(-)	CWNAWTKWSATRYN	TTAATCCAATGCTC
M00138	V$OCT1_04	0.799038	6747	(+)	NNNNNNNNWATGCAAATNNNWNNW	AAGAAGTTAGGCTAG-TTAAAGGA
M00252	V$TATA_01	0.782035	61	(+)	STATAAAWRNNNNNN	GAATTTAAATAAACC
M00252	V$TATA_01	0.839127	63	(+)	STATAAAWRNNNNNN	ATTTAAATAAACCTG
M00216	V$TATA_C	0.814629	92	(+)	NCTATAAAAR	ACCACAAAAT
M00216	V$TATA_C	0.79456	122	(+)	NCTATAAAAR	GGTTTAAATA
M00252	V$TATA_01	0.882771	123	(+)	STATAAAWRNNNNNN	GTTTAAATAGCTTGG
M00252	V$TATA_01	0.773662	209	(+)	STATAAAWRNNNNNN	TTATGTATACATATA
M00252	V$TATA_01	0.7729	213	(+)	STATAAAWRNNNNNN	GTATACATATAACAA
M00252	V$TATA_01	0.792185	215	(+)	STATAAAWRNNNNNN	ATACATATAACAAAA
M00216	V$TATA_C	0.755479	216	(+)	NCTATAAAAR	TACATATAAC
M00252	V$TATA_01	0.801066	217	(+)	STATAAAWRNNNNNN	ACATATAACAAAATT
M00216	V$TATA_C	0.751254	218	(+)	NCTATAAAAR	CATATAACAA
M00216	V$TATA_C	0.893847	333	(+)	NCTATAAAAR	AGCTTAAAAA
M00216	V$TATA_C	0.811724	334	(+)	NCTATAAAAR	GCTTAAAAAT
M00252	V$TATA_01	0.801319	334	(+)	STATAAAWRNNNNNN	GCTTAAAAATATATT
M00252	V$TATA_01	0.775945	340	(+)	STATAAAWRNNNNNN	AAATATATTTGCAAA
M00252	V$TATA_01	0.818066	342	(+)	STATAAAWRNNNNNN	ATATATTTGCAAATG

M00216	V$TATA_C	0.825191	399	(+)	NCTATAAAAR	CTTTTATAAA
M00252	V$TATA_01	0.821619	400	(+)	STATAAAWRNNNNNN	TTTTATAAAGACCAA
M00216	V$TATA_C	0.793768	401	(+)	NCTATAAAAR	TTTATAAAGA
M00252	V$TATA_01	0.871352	402	(+)	STATAAAWRNNNNNN	TTATAAAGACCAAAT
M00252	V$TATA_01	0.793453	422	(+)	STATAAAWRNNNNNN	ATTTTTAAACGAAAT
M00216	V$TATA_C	0.779509	423	(+)	NCTATAAAAR	TTTTTAAACG
M00216	V$TATA_C	0.874835	514	(+)	NCTATAAAAR	TCTGTAAAAA
M00252	V$TATA_01	0.78914	515	(+)	STATAAAWRNNNNNN	CTGTAAAAAGTTGTC
M00216	V$TATA_C	0.759176	539	(+)	NCTATAAAAR	AATTTATAAT
M00216	V$TATA_C	0.77634	541	(+)	NCTATAAAAR	TTTATAATAT
M00216	V$TATA_C	0.768154	558	(+)	NCTATAAAAR	TTCATAGAAA
M00216	V$TATA_C	0.817798	619	(+)	NCTATAAAAR	TTAATAAAAT
M00216	V$TATA_C	0.81859	631	(+)	NCTATAAAAR	ATTTTATAAA
M00252	V$TATA_01	0.837351	632	(+)	STATAAAWRNNNNNN	TTTTATAAAGGAAAT
M00216	V$TATA_C	0.805123	633	(+)	NCTATAAAAR	TTTATAAAGG
M00252	V$TATA_01	0.825171	634	(+)	STATAAAWRNNNNNN	TTATAAAGGAAATGT
M00252	V$TATA_01	0.807663	664	(+)	STATAAAWRNNNNNN	AGATATAAATGACTA
M00216	V$TATA_C	0.751254	665	(+)	NCTATAAAAR	GATATAAATG
M00252	V$TATA_01	0.897488	666	(+)	STATAAAWRNNNNNN	ATATAAATGACTACT
M00216	V$TATA_C	0.825191	679	(+)	NCTATAAAAR	CTTTTATAAA
M00252	V$TATA_01	0.805126	680	(+)	STATAAAWRNNNNNN	TTTTATAAAGATGAT
M00216	V$TATA_C	0.793768	681	(+)	NCTATAAAAR	TTTATAAAGA
M00252	V$TATA_01	0.821619	682	(+)	STATAAAWRNNNNNN	TTATAAAGATGATTA
M00252	V$TATA_01	0.770109	716	(+)	STATAAAWRNNNNNN	ATACAAATATATGAA
M00252	V$TATA_01	0.801573	720	(+)	STATAAAWRNNNNNN	AAATATATGAAAACA
M00252	V$TATA_01	0.839127	722	(+)	STATAAAWRNNNNNN	ATATATGAAAACAGT
M00216	V$TATA_C	0.751782	723	(+)	NCTATAAAAR	TATATGAAAA
M00252	V$TATA_01	0.815529	724	(+)	STATAAAWRNNNNNN	ATATGAAAACAGTAG
M00216	V$TATA_C	0.866121	751	(+)	NCTATAAAAR	CCTATAAATA
M00252	V$TATA_01	0.891398	752	(+)	STATAAAWRNNNNNN	CTATAAATATGTCTT
M00216	V$TATA_C	0.768154	766	(+)	NCTATAAAAR	TTTTTAACAA
M00252	V$TATA_01	0.777468	794	(+)	STATAAAWRNNNNNN	GTTTGTAAACTGAAG
M00216	V$TATA_C	0.923686	812	(+)	NCTATAAAAR	CCTATATAAA

M00252	V$TATA_01	0.88404	813	(+)	STATAAAWRNNNNNN	CTATATAAAATAATC
M00216	V$TATA_C	0.856351	814	(+)	NCTATAAAAR	TATATAAAAT
M00252	V$TATA_01	0.851561	815	(+)	STATAAAWRNNNNNN	ATATAAAATAATCTG
M00216	V$TATA_C	0.820174	849	(+)	NCTATAAAAR	GGTATAAACA
M00252	V$TATA_01	0.793707	850	(+)	STATAAAWRNNNNNN	GTATAAACAAATTTC
M00216	V$TATA_C	0.781357	911	(+)	NCTATAAAAR	CACATATAAA
M00252	V$TATA_01	0.846993	912	(+)	STATAAAWRNNNNNN	ACATATAAATGTATG
M00216	V$TATA_C	0.762609	913	(+)	NCTATAAAAR	CATATAAATG
M00252	V$TATA_01	0.863994	914	(+)	STATAAAWRNNNNNN	ATATAAATGTATGTA
M00252	V$TATA_01	0.770363	942	(+)	STATAAAWRNNNNNN	CAATTTAATGGAACT
M00252	V$TATA_01	0.802588	1023	(+)	STATAAAWRNNNNNN	GGATATATCAACCCA
M00252	V$TATA_01	0.843441	1025	(+)	STATAAAWRNNNNNN	ATATATCAACCCAGC
M00216	V$TATA_C	0.749142	1060	(+)	NCTATAAAAR	TCCTTTTAAA
M00216	V$TATA_C	0.840507	1061	(+)	NCTATAAAAR	CCTTTTAAAA
M00216	V$TATA_C	0.889622	1062	(+)	NCTATAAAAR	CTTTTAAAAA
M00216	V$TATA_C	0.750726	1063	(+)	NCTATAAAAR	TTTTAAAAAT
M00252	V$TATA_01	0.784065	1063	(+)	STATAAAWRNNNNNN	TTTTAAAAATTTAAT
M00216	V$TATA_C	0.78479	1092	(+)	NCTATAAAAR	TCTACATAAT
M00252	V$TATA_01	0.793961	1093	(+)	STATAAAWRNNNNNN	CTACATAATGCACAA
M00216	V$TATA_C	0.757064	1122	(+)	NCTATAAAAR	ACTCTGAAAG
M00252	V$TATA_01	0.798275	1143	(+)	STATAAAWRNNNNNN	GGATAAAGACATTTT
M00216	V$TATA_C	0.883021	1153	(+)	NCTATAAAAR	ATTTTAAAAA
M00216	V$TATA_C	0.768154	1154	(+)	NCTATAAAAR	TTTTAAAAAA
M00252	V$TATA_01	0.796245	1154	(+)	STATAAAWRNNNNNN	TTTTAAAAAATTACA
M00216	V$TATA_C	0.77634	1155	(+)	NCTATAAAAR	TTTAAAAAAT
M00252	V$TATA_01	0.802334	1170	(+)	STATAAAWRNNNNNN	ATATTAAATGTAATT
M00216	V$TATA_C	0.761025	1208	(+)	NCTATAAAAR	ATTTTAACAA
M00216	V$TATA_C	0.759176	1241	(+)	NCTATAAAAR	TCTTTTTAAT
M00216	V$TATA_C	0.865593	1263	(+)	NCTATAAAAR	ATTTTAAAAT
M00252	V$TATA_01	0.797513	1278	(+)	STATAAAWRNNNNNN	ATATTTAAATATTGA
M00252	V$TATA_01	0.832784	1280	(+)	STATAAAWRNNNNNN	ATTTAAATATTGATG
M00216	V$TATA_C	0.816477	1348	(+)	NCTATAAAAR	ACTTTAAAGT
M00252	V$TATA_01	0.772139	1349	(+)	STATAAAWRNNNNNN	CTTTAAAGTGTAGTA

M00252	V$TATA_01	0.774423	1382	(+)	STATAAAWRNNNNNN	TCTTATATAAGCACT
M00216	V$TATA_C	0.862166	1383	(+)	NCTATAAAAR	CTTATATAAG
M00252	V$TATA_01	0.864501	1384	(+)	STATAAAWRNNNNNN	TTATATAAGCACTAA
M00252	V$TATA_01	0.812992	1420	(+)	STATAAAWRNNNNNN	ATATTTATATTGAAG
M00252	V$TATA_01	0.801319	1422	(+)	STATAAAWRNNNNNN	ATTTATATTGAAGAG
M00216	V$TATA_C	0.760496	1439	(+)	NCTATAAAAR	AAATTAAAAA
M00216	V$TATA_C	0.873515	1454	(+)	NCTATAAAAR	TGAATAAAAA
M00252	V$TATA_01	0.805379	1455	(+)	STATAAAWRNNNNNN	GAATAAAAATATTAC
M00216	V$TATA_C	0.741748	1490	(+)	NCTATAAAAR	ATCATAATAT
M00216	V$TATA_C	0.756536	1547	(+)	NCTATAAAAR	TTATTATAAG
M00216	V$TATA_C	0.760232	1549	(+)	NCTATAAAAR	ATTATAAGAC
M00252	V$TATA_01	0.789901	1550	(+)	STATAAAWRNNNNNN	TTATAAGACCTAACT
M00216	V$TATA_C	0.805651	1593	(+)	NCTATAAAAR	ACCATGAAAC
M00216	V$TATA_C	0.789015	1684	(+)	NCTATAAAAR	TGTTTAAAGT
M00252	V$TATA_01	0.793961	1685	(+)	STATAAAWRNNNNNN	GTTTAAAGTCATCTC
M00216	V$TATA_C	0.754423	1743	(+)	NCTATAAAAR	GTCATAAAGA
M00216	V$TATA_C	0.866121	1772	(+)	NCTATAAAAR	GCTATTAAAG
M00252	V$TATA_01	0.841157	1773	(+)	STATAAAWRNNNNNN	CTATTAAAGTGTTGA
M00216	V$TATA_C	0.751254	1786	(+)	NCTATAAAAR	GATATTAAAG
M00252	V$TATA_01	0.836843	1787	(+)	STATAAAWRNNNNNN	ATATTAAAGCTATGG
M00216	V$TATA_C	0.745709	1850	(+)	NCTATAAAAR	ACTCTAAATA
M00216	V$TATA_C	0.802218	1852	(+)	NCTATAAAAR	TCTAAATAAA
M00216	V$TATA_C	0.775812	1854	(+)	NCTATAAAAR	TAAATAAAAT
M00252	V$TATA_01	0.828216	1874	(+)	STATAAAWRNNNNNN	AAATATAAACACCTC
M00216	V$TATA_C	0.744653	1875	(+)	NCTATAAAAR	AATATAAACA
M00252	V$TATA_01	0.812992	1876	(+)	STATAAAWRNNNNNN	ATATAAACACCTCAC
M00216	V$TATA_C	0.778981	1889	(+)	NCTATAAAAR	ACAATTAAAA
M00216	V$TATA_C	0.767098	1890	(+)	NCTATAAAAR	CAATTAAAAA
M00216	V$TATA_C	0.883021	1899	(+)	NCTATAAAAR	ATTTTAAAAA
M00252	V$TATA_01	0.788125	1899	(+)	STATAAAWRNNNNNN	ATTTTAAAAAAAAAG
M00216	V$TATA_C	0.768154	1900	(+)	NCTATAAAAR	TTTTAAAAAA
M00252	V$TATA_01	0.833799	1900	(+)	STATAAAWRNNNNNN	TTTTAAAAAAAAAGA
M00216	V$TATA_C	0.793768	1901	(+)	NCTATAAAAR	TTTAAAAAAA

M00216	V$TATA_C	0.763401	1928	(+)	NCTATAAAAR	ACAATACAAT
M00216	V$TATA_C	0.778981	1933	(+)	NCTATAAAAR	ACAATAAAGA
M00252	V$TATA_01	0.813245	1934	(+)	STATAAAWRNNNNNN	CAATAAAGAGCATCA
M00216	V$TATA_C	0.80882	1942	(+)	NCTATAAAAR	AGCATCAAAG
M00216	V$TATA_C	0.74967	1986	(+)	NCTATAAAAR	GGCTTAACAT
M00252	V$TATA_01	0.802081	2004	(+)	STATAAAWRNNNNNN	ACATATATATATCCA
M00252	V$TATA_01	0.891144	2006	(+)	STATAAAWRNNNNNN	ATATATATATCCAAT
M00252	V$TATA_01	0.83253	2008	(+)	STATAAAWRNNNNNN	ATATATATCCAATAT
M00216	V$TATA_C	0.815949	2172	(+)	NCTATAAAAR	TGCATGAAAG
M00252	V$TATA_01	0.778483	2173	(+)	STATAAAWRNNNNNN	GCATGAAAGTGAAAA
M00216	V$TATA_C	0.833113	2316	(+)	NCTATAAAAR	ACTATTAAAC
M00252	V$TATA_01	0.853337	2317	(+)	STATAAAWRNNNNNN	CTATTAAACCCTATA
M00252	V$TATA_01	0.772393	2461	(+)	STATAAAWRNNNNNN	GTAGAAAGAAAAACT
M00216	V$TATA_C	0.77766	2473	(+)	NCTATAAAAR	ACTAGATAAT
M00216	V$TATA_C	0.740692	2502	(+)	NCTATAAAAR	TTCTCAAAAA
M00216	V$TATA_C	0.752839	2503	(+)	NCTATAAAAR	TCTCAAAAAA
M00252	V$TATA_01	0.820604	2522	(+)	STATAAAWRNNNNNN	TTATTAATGTGAAAC
M00216	V$TATA_C	0.815949	2692	(+)	NCTATAAAAR	TGCATGAAAG
M00216	V$TATA_C	0.78479	2842	(+)	NCTATAAAAR	TCTAGATAAT
M00252	V$TATA_01	0.785334	2851	(+)	STATAAAWRNNNNNN	TGATAAATAAATAAA
M00252	V$TATA_01	0.828724	2855	(+)	STATAAAWRNNNNNN	AAATAAATAAATAGG
M00252	V$TATA_01	0.848769	2859	(+)	STATAAAWRNNNNNN	AAATAAATAGGAATC
M00252	V$TATA_01	0.777721	2912	(+)	STATAAAWRNNNNNN	GGATATTTGGACACT
M00216	V$TATA_C	0.842091	2930	(+)	NCTATAAAAR	ACTATCAAAT
M00252	V$TATA_01	0.771632	2936	(+)	STATAAAWRNNNNNN	AAATATAGATGAAAA
M00252	V$TATA_01	0.776199	2938	(+)	STATAAAWRNNNNNN	ATATAGATGAAAAAG
M00216	V$TATA_C	0.750198	3001	(+)	NCTATAAAAR	TGTAAATAAT
M00216	V$TATA_C	0.797465	3074	(+)	NCTATAAAAR	AGCATCAAAA
M00216	V$TATA_C	0.759968	3075	(+)	NCTATAAAAR	GCATCAAAAG
M00216	V$TATA_C	0.781885	3105	(+)	NCTATAAAAR	GTTACAAAAA
M00252	V$TATA_01	0.784572	3106	(+)	STATAAAWRNNNNNN	TTACAAAAAAAGGAT
M00216	V$TATA_C	0.790335	3108	(+)	NCTATAAAAR	ACAAAAAAAG
M00252	V$TATA_01	0.794976	3182	(+)	STATAAAWRNNNNNN	AGATATAGATCCCCC

M00252	V$TATA_01	0.779751	3184	(+)	STATAAAWRNNNNNN	ATATAGATCCCCCAG
M00252	V$TATA_01	0.77087	3261	(+)	STATAAAWRNNNNNN	ACATAAATCTTGTTC
M00252	V$TATA_01	0.771632	3281	(+)	STATAAAWRNNNNNN	GTTAAAATGCAGATT
M00216	V$TATA_C	0.833905	3306	(+)	NCTATAAAAR	TTTATATAAT
M00252	V$TATA_01	0.807156	3307	(+)	STATAAAWRNNNNNN	TTATATAATTTAATT
M00216	V$TATA_C	0.795088	3323	(+)	NCTATAAAAR	ATGATAAAAA
M00252	V$TATA_01	0.781528	3324	(+)	STATAAAWRNNNNNN	TGATAAAAAATAAAA
M00216	V$TATA_C	0.768682	3330	(+)	NCTATAAAAR	AAAATAAAAT
M00252	V$TATA_01	0.806648	3331	(+)	STATAAAWRNNNNNN	AAATAAAATCCAGAC
M00216	V$TATA_C	0.794824	3397	(+)	NCTATAAAAR	ATTCTAAAAA
M00252	V$TATA_01	0.790916	3398	(+)	STATAAAWRNNNNNN	TTCTAAAAACAGAAG
M00216	V$TATA_C	0.744653	3399	(+)	NCTATAAAAR	TCTAAAAACA
M00216	V$TATA_C	0.740956	3483	(+)	NCTATAAAAR	GCTGTTAAAA
M00216	V$TATA_C	0.758648	3484	(+)	NCTATAAAAR	CTGTTAAAAT
M00216	V$TATA_C	0.740692	3500	(+)	NCTATAAAAR	TTCTTAAACA
M00216	V$TATA_C	0.842091	3512	(+)	NCTATAAAAR	ACTATTAAAT
M00252	V$TATA_01	0.802588	3513	(+)	STATAAAWRNNNNNN	CTATTAAATGTATAG
M00216	V$TATA_C	0.740692	3578	(+)	NCTATAAAAR	GTTTTACAAT
M00216	V$TATA_C	0.779509	3600	(+)	NCTATAAAAR	TTTTTAACAG
M00252	V$TATA_01	0.787871	3699	(+)	STATAAAWRNNNNNN	TGATAAATTGCTAAT
M00252	V$TATA_01	0.843441	3712	(+)	STATAAAWRNNNNNN	ATATATATATTGTAG
M00252	V$TATA_01	0.832276	3714	(+)	STATAAAWRNNNNNN	ATATATATTGTAGTC
M00252	V$TATA_01	0.777721	3854	(+)	STATAAAWRNNNNNN	ATATTTATGATTAAT
M00216	V$TATA_C	0.835226	3864	(+)	NCTATAAAAR	TTAATAAAAA
M00252	V$TATA_01	0.780259	3905	(+)	STATAAAWRNNNNNN	ATATGTATATGTGGT
M00252	V$TATA_01	0.8102	3909	(+)	STATAAAWRNNNNNN	GTATATGTGGTCTAT
M00216	V$TATA_C	0.807499	4006	(+)	NCTATAAAAR	AGTATTAAAT
M00252	V$TATA_01	0.837351	4007	(+)	STATAAAWRNNNNNN	GTATTAAATTAGATA
M00216	V$TATA_C	0.780829	4034	(+)	NCTATAAAAR	CTTTTACAAG
M00252	V$TATA_01	0.807156	4165	(+)	STATAAAWRNNNNNN	TGATAAATACTGAAT
M00216	V$TATA_C	0.865593	4373	(+)	NCTATAAAAR	ATTTTAAAAT
M00252	V$TATA_01	0.771885	4373	(+)	STATAAAWRNNNNNN	ATTTTAAAATAAAAT
M00252	V$TATA_01	0.790409	4374	(+)	STATAAAWRNNNNNN	TTTTAAAATAAAATC

M00216	V$TATA_C	0.768682	4378	(+)	NCTATAAAAR	AAAATAAAAT
M00252	V$TATA_01	0.790916	4379	(+)	STATAAAWRNNNNNN	AAATAAAATCATAAA
M00216	V$TATA_C	0.854766	4386	(+)	NCTATAAAAR	ATCATAAAAC
M00252	V$TATA_01	0.808424	4387	(+)	STATAAAWRNNNNNN	TCATAAAACTGAAAC
M00216	V$TATA_C	0.80169	4441	(+)	NCTATAAAAR	CCTAAATAAA
M00252	V$TATA_01	0.819843	4442	(+)	STATAAAWRNNNNNN	CTAAATAAATGGAGA
M00252	V$TATA_01	0.839381	4444	(+)	STATAAAWRNNNNNN	AAATAAATGGAGAAT
M00252	V$TATA_01	0.783811	4463	(+)	STATAAAWRNNNNNN	GTTCAAATGGAAAAA
M00216	V$TATA_C	0.78611	4543	(+)	NCTATAAAAR	TCAATGAAAA
M00216	V$TATA_C	0.752839	4616	(+)	NCTATAAAAR	TCTGGAAAAA
M00252	V$TATA_01	0.878204	4712	(+)	STATAAAWRNNNNNN	TTATATATGCAAATA
M00216	V$TATA_C	0.744653	4723	(+)	NCTATAAAAR	AATATTAAAA
M00216	V$TATA_C	0.802482	4724	(+)	NCTATAAAAR	ATATTAAAAA
M00252	V$TATA_01	0.848008	4724	(+)	STATAAAWRNNNNNN	ATATTAAAAAGCTGC
M00216	V$TATA_C	0.761553	4736	(+)	NCTATAAAAR	TGCTTTAAAT
M00216	V$TATA_C	0.829152	4737	(+)	NCTATAAAAR	GCTTTAAATA
M00252	V$TATA_01	0.829231	4738	(+)	STATAAAWRNNNNNN	CTTTAAATATTAATC
M00252	V$TATA_01	0.811723	4744	(+)	STATAAAWRNNNNNN	ATATTAATCTGTACC
M00216	V$TATA_C	0.848429	4773	(+)	NCTATAAAAR	TCTGTAAAAC
M00216	V$TATA_C	0.802218	4805	(+)	NCTATAAAAR	TCTATGTAAA
M00216	V$TATA_C	0.818326	4814	(+)	NCTATAAAAR	ACTTTACAAT
M00216	V$TATA_C	0.760496	4943	(+)	NCTATAAAAR	AGTATTTAAA
M00252	V$TATA_01	0.830754	4944	(+)	STATAAAWRNNNNNN	GTATTTAAAAGATTT
M00216	V$TATA_C	0.859519	4945	(+)	NCTATAAAAR	TATTTAAAAG
M00252	V$TATA_01	0.791423	4946	(+)	STATAAAWRNNNNNN	ATTTAAAAGATTTGA
M00216	V$TATA_C	0.750726	4987	(+)	NCTATAAAAR	TTTTTAATAT
M00216	V$TATA_C	0.753367	4990	(+)	NCTATAAAAR	TTAATATAAT
M00252	V$TATA_01	0.781528	5094	(+)	STATAAAWRNNNNNN	TTATTTATGGTGTTA
M00216	V$TATA_C	0.750726	5251	(+)	NCTATAAAAR	TTTTTTAAAT
M00216	V$TATA_C	0.768154	5252	(+)	NCTATAAAAR	TTTTTAAATA
M00252	V$TATA_01	0.804618	5261	(+)	STATAAAWRNNNNNN	ATTTTTAAGCACACA
M00216	V$TATA_C	0.771851	5289	(+)	NCTATAAAAR	AGCTTAAATA
M00252	V$TATA_01	0.808171	5290	(+)	STATAAAWRNNNNNN	GCTTAAATAAAGAAA

M00252	V$TATA_01	0.772139	5292	(+)	STATAAAWRNNNNNN	TTAAATAAAGAAAAC
M00216	V$TATA_C	0.787167	5337	(+)	NCTATAAAAR	TGCAAAAAAT
M00216	V$TATA_C	0.766306	5356	(+)	NCTATAAAAR	TTCACAAAAA
M00216	V$TATA_C	0.751518	5436	(+)	NCTATAAAAR	TGAAAAAAAA
M00216	V$TATA_C	0.76921	5510	(+)	NCTATAAAAR	ATTATCAAAT
M00216	V$TATA_C	0.905202	5537	(+)	NCTATAAAAR	AGCTTAAAAG
M00252	V$TATA_01	0.829485	5538	(+)	STATAAAWRNNNNNN	GCTTAAAAGAGGAAT
M00216	V$TATA_C	0.78479	5590	(+)	NCTATAAAAR	TCTATTTAAT
M00252	V$TATA_01	0.813245	5591	(+)	STATAAAWRNNNNNN	CTATTTAATTCAGCT
M00216	V$TATA_C	0.762873	5619	(+)	NCTATAAAAR	GCCATATACA
M00252	V$TATA_01	0.772647	5633	(+)	STATAAAWRNNNNNN	TTATAGATATCCATT
M00252	V$TATA_01	0.782796	5713	(+)	STATAAAWRNNNNNN	ACATAAATGATATTT
M00252	V$TATA_01	0.77087	5722	(+)	STATAAAWRNNNNNN	ATATTTAAATTATTT
M00216	V$TATA_C	0.743597	5733	(+)	NCTATAAAAR	ATTTTAAAGT
M00216	V$TATA_C	0.831529	5815	(+)	NCTATAAAAR	CGTATGAAAA
M00252	V$TATA_01	0.817813	5816	(+)	STATAAAWRNNNNNN	GTATGAAAATACGAA
M00216	V$TATA_C	0.824927	5863	(+)	NCTATAAAAR	AGTATGAAAA
M00252	V$TATA_01	0.809947	5864	(+)	STATAAAWRNNNNNN	GTATGAAAAACATTT
M00216	V$TATA_C	0.762873	5875	(+)	NCTATAAAAR	ATTTTAGAAA
M00216	V$TATA_C	0.793768	5877	(+)	NCTATAAAAR	TTTAGAAAAA
M00216	V$TATA_C	0.744389	5880	(+)	NCTATAAAAR	AGAAAAAAAA
M00216	V$TATA_C	0.833905	5919	(+)	NCTATAAAAR	ACTTTCAAAA
M00216	V$TATA_C	0.750198	5920	(+)	NCTATAAAAR	CTTTCAAAAT
M00216	V$TATA_C	0.836282	6007	(+)	NCTATAAAAR	AGTATAAATG
M00252	V$TATA_01	0.885562	6008	(+)	STATAAAWRNNNNNN	GTATAAATGAATGAA
M00216	V$TATA_C	0.851862	6023	(+)	NCTATAAAAR	TGGATAAAAG
M00252	V$TATA_01	0.825425	6024	(+)	STATAAAWRNNNNNN	GGATAAAAGAATGAA
M00252	V$TATA_01	0.782035	6108	(+)	STATAAAWRNNNNNN	CTACATGAAGCAATA
M00252	V$TATA_01	0.789901	6118	(+)	STATAAAWRNNNNNN	CAATATATCTGCTCC
M00216	V$TATA_C	0.841035	6171	(+)	NCTATAAAAR	TCTTTAAACA
M00252	V$TATA_01	0.771378	6172	(+)	STATAAAWRNNNNNN	CTTTAAACACAAGTT
M00216	V$TATA_C	0.779509	6201	(+)	NCTATAAAAR	TTTTTCAAAG
M00216	V$TATA_C	0.767626	6210	(+)	NCTATAAAAR	GTTTTAATAG

M00252	V$TATA_01	0.809693	6210	(+)	STATAAAWRNNNNNN	GTTTTAATAGCTTTG
M00216	V$TATA_C	0.781885	6224	(+)	NCTATAAAAR	GTTATTAAAA
M00216	V$TATA_C	0.783206	6225	(+)	NCTATAAAAR	TTATTAAAAC
M00252	V$TATA_01	0.821365	6225	(+)	STATAAAWRNNNNNN	TTATTAAAACAACAT
M00216	V$TATA_C	0.755743	6243	(+)	NCTATAAAAR	GGTATTTAAA
M00252	V$TATA_01	0.810454	6244	(+)	STATAAAWRNNNNNN	GTATTTAAAAATTTA
M00216	V$TATA_C	0.848165	6245	(+)	NCTATAAAAR	TATTTAAAAA
M00216	V$TATA_C	0.743597	6246	(+)	NCTATAAAAR	ATTTAAAAAT
M00252	V$TATA_01	0.81223	6246	(+)	STATAAAWRNNNNNN	ATTTAAAAATTTACA
M00216	V$TATA_C	0.768418	6344	(+)	NCTATAAAAR	ATTCTAAAAC
M00216	V$TATA_C	0.842091	6352	(+)	NCTATAAAAR	ACTATTAAAT
M00252	V$TATA_01	0.797006	6353	(+)	STATAAAWRNNNNNN	CTATTAAATATAATA
M00252	V$TATA_01	0.776453	6358	(+)	STATAAAWRNNNNNN	AAATATAATATAAGG
M00252	V$TATA_01	0.780513	6360	(+)	STATAAAWRNNNNNN	ATATAATATAAGGGA
M00216	V$TATA_C	0.77502	6362	(+)	NCTATAAAAR	ATAATATAAG
M00252	V$TATA_01	0.818066	6363	(+)	STATAAAWRNNNNNN	TAATATAAGGGAAAT
M00252	V$TATA_01	0.77696	6374	(+)	STATAAAWRNNNNNN	AAATAAAGAAGTAAA
M00216	V$TATA_C	0.807499	6383	(+)	NCTATAAAAR	AGTAAAAAAT
M00216	V$TATA_C	0.748878	6456	(+)	NCTATAAAAR	ACTAAAACAG
M00216	V$TATA_C	0.755743	6481	(+)	NCTATAAAAR	AGAAAAAAAG
M00216	V$TATA_C	0.754423	6548	(+)	NCTATAAAAR	AGCTTAAATT
M00216	V$TATA_C	0.778981	6638	(+)	NCTATAAAAR	TGCTTAAATA
M00252	V$TATA_01	0.806902	6645	(+)	STATAAAWRNNNNNN	ATATTTATACATCAT
M00216	V$TATA_C	0.799313	6735	(+)	NCTATAAAAR	AGTTAAAAAA
M00252	V$TATA_01	0.777975	6735	(+)	STATAAAWRNNNNNN	AGTTAAAAAAAAAAG
M00216	V$TATA_C	0.781885	6736	(+)	NCTATAAAAR	GTTAAAAAAA
M00059	V$YY1_01	0.782765	99	(+)	NNNNNCCATNTWNNNWN	AATTAGCATTTTACTAA
M00059	V$YY1_01	0.797837	212	(+)	NNNNNCCATNTWNNNWN	TGTATACATATAACAAA
M00059	V$YY1_01	0.809633	346	(-)	NNNNNCCATNTWNNNWN	ATTTGCAAATGTTGATA
M00059	V$YY1_01	0.849279	415	(+)	NNNNNCCATNTWNNNWN	ATTGATCATTTTTAAAC
M00059	V$YY1_01	0.848952	444	(-)	NNNNNCCATNTWNNNWN	TACTGAAAATGTAGATA
M00059	V$YY1_01	0.785059	565	(+)	NNNNNCCATNTWNNNWN	AAATAACATTTCTGGTA
M00069	V$YY1_02	0.795787	589	(+)	NNNCGGCCATCTTGNCTSNW	TCAAGGCCATTTTTATTTTG

M00059	V$YY1_01	0.884993	590	(+)	NNNNNCCATNTWNNNWN	CAAGGCCATTTTTATTT
M00059	V$YY1_01	0.779489	718	(-)	NNNNNCCATNTWNNNWN	ACAAATATATGAAAACA
M00059	V$YY1_01	0.796527	752	(-)	NNNNNCCATNTWNNNWN	CTATAAATATGTCTTTT
M00059	V$YY1_01	0.79194	768	(-)	NNNNNCCATNTWNNNWN	TTTAACAAATGCAGGTA
M00059	V$YY1_01	0.833879	858	(+)	NNNNNCCATNTWNNNWN	AAATTTCATGTATAATC
M00059	V$YY1_01	0.828965	894	(+)	NNNNNCCATNTWNNNWN	GAAACGCATTTTTCCAG
M00059	V$YY1_01	0.824377	1001	(+)	NNNNNCCATNTWNNNWN	CATCTTCATTCAGTCAT
M00059	V$YY1_01	0.780472	1013	(-)	NNNNNCCATNTWNNNWN	GTCATGTCATGGATATA
M00059	V$YY1_01	0.785059	1099	(-)	NNNNNCCATNTWNNNWN	AATGCACAATGTTTTTC
M00059	V$YY1_01	0.81979	1146	(+)	NNNNNCCATNTWNNNWN	TAAAGACATTTTAAAAA
M00059	V$YY1_01	0.806356	1169	(-)	NNNNNCCATNTWNNNWN	GATATTAAATGTAATTT
M00059	V$YY1_01	0.775229	1688	(+)	NNNNNCCATNTWNNNWN	TAAAGTCATCTCAAATG
M00059	V$YY1_01	0.788008	1790	(-)	NNNNNCCATNTWNNNWN	TTAAAGCTATGGTAATC
M00059	V$YY1_01	0.790301	1973	(+)	NNNNNCCATNTWNNNWN	TGGGTCCATTATAGGCT
M00059	V$YY1_01	0.817169	1987	(+)	NNNNNCCATNTWNNNWN	GCTTAACATATTTGTAA
M00059	V$YY1_01	0.794233	1999	(+)	NNNNNCCATNTWNNNWN	TGTAAACATATATATAT
M00059	V$YY1_01	0.835518	2082	(+)	NNNNNCCATNTWNNNWN	CGACACCATTGATGGCA
M00069	V$YY1_02	0.75241	2083	(-)	NNNCGGCCATCTTGNCTSNW	GACACCATTGATGGCAGCCC
M00059	V$YY1_01	0.784731	2085	(-)	NNNNNCCATNTWNNNWN	CACCATTGATGGCAGCC
M00059	V$YY1_01	0.780144	2135	(-)	NNNNNCCATNTWNNNWN	CAAGAACACTGGAGTGG
M00059	V$YY1_01	0.783093	2151	(+)	NNNNNCCATNTWNNNWN	GGTTGCCATTTCCTTCT
M00059	V$YY1_01	0.781782	2225	(+)	NNNNNCCATNTWNNNWN	CAACCCCATGGACTGCC
M00059	V$YY1_01	0.813237	2248	(-)	NNNNNCCATNTWNNNWN	CTCCATCCATGGGATTT
M00059	V$YY1_01	0.78768	2338	(+)	NNNNNCCATNTWNNNWN	TGTATCCACTTTTGGAA
M00059	V$YY1_01	0.809961	2513	(+)	NNNNNCCATNTWNNNWN	CAAATTCAGTTATTAAT
M00069	V$YY1_02	0.799179	2615	(-)	NNNCGGCCATCTTGNCTSNW	AGCAGCCCACAAGGCTCCCC
M00059	V$YY1_01	0.780144	2655	(-)	NNNNNCCATNTWNNNWN	CAAGAACACTGGAGTGG
M00059	V$YY1_01	0.783093	2671	(+)	NNNNNCCATNTWNNNWN	GGTTGCCATTTCCTTCT
M00059	V$YY1_01	0.78768	2775	(-)	NNNNNCCATNTWNNNWN	CTCTGTCCATGGGATTT
M00059	V$YY1_01	0.813893	2953	(-)	NNNNNCCATNTWNNNWN	TTTCTGAAATGCTGAGA
M00059	V$YY1_01	0.915465	3077	(-)	NNNNNCCATNTWNNNWN	ATCAAAAGATGGAAATA
M00059	V$YY1_01	0.806356	3149	(+)	NNNNNCCATNTWNNNWN	GTAGTCCATATCTTTTC
M00059	V$YY1_01	0.906291	3229	(+)	NNNNNCCATNTWNNNWN	AAACATCATTTTTTAAT

M00059	V$YY1_01	0.787025	3245	(+)	NNNNNCCATNTWNNNWN	TGCTAACATTTAACAAA
M00059	V$YY1_01	0.827326	3279	(-)	NNNNNCCATNTWNNNWN	CAGTTAAAATGCAGATT
M00069	V$YY1_02	0.75116	3387	(+)	NNNCGGCCATCTTGNCTSNW	TCTCCACCATATTCTAAAAA
M00059	V$YY1_01	0.848624	3388	(+)	NNNNNCCATNTWNNNWN	CTCCACCATATTCTAAA
M00059	V$YY1_01	0.800786	3750	(+)	NNNNNCCATNTWNNNWN	AACAGCCAGTTTCACAT
M00069	V$YY1_02	0.775437	3827	(-)	NNNCGGCCATCTTGNCTSNW	TTTAGAGAAGAGGGCATTTG
M00059	V$YY1_01	0.813565	3899	(-)	NNNNNCCATNTWNNNWN	AACAGAATATGTATATG
M00059	V$YY1_01	0.797182	3987	(+)	NNNNNCCATNTWNNNWN	GATCTCAATTTACACAC
M00059	V$YY1_01	0.900393	4073	(+)	NNNNNCCATNTWNNNWN	TCAGTTCATTTTTTAAT
M00059	V$YY1_01	0.791284	4194	(+)	NNNNNCCATNTWNNNWN	AATTTTCATTTGTATAC
M00059	V$YY1_01	0.785387	4208	(+)	NNNNNCCATNTWNNNWN	TACCTTCATTTCAATTG
M00059	V$YY1_01	0.823067	4223	(+)	NNNNNCCATNTWNNNWN	TGTATCCAGATACTCAG
M00059	V$YY1_01	0.924967	4349	(-)	NNNNNCCATNTWNNNWN	AACCAAATATGGTGTCT
M00059	V$YY1_01	0.853539	4442	(-)	NNNNNCCATNTWNNNWN	CTAAATAAATGGAGAAT
M00059	V$YY1_01	0.794233	4461	(-)	NNNNNCCATNTWNNNWN	GTGTTCAAATGGAAAAA
M00059	V$YY1_01	0.796855	4526	(-)	NNNNNCCATNTWNNNWN	ATTCCTACAGGAAGTCC
M00059	V$YY1_01	0.841415	4643	(+)	NNNNNCCATNTWNNNWN	TACAAACATTTTTTCAC
M00059	V$YY1_01	0.869594	4669	(+)	NNNNNCCATNTWNNNWN	TATTTTCATTTACTCAC
M00059	V$YY1_01	0.820118	4710	(-)	NNNNNCCATNTWNNNWN	AATTATATATGCAAATA
M00059	V$YY1_01	0.792595	4821	(+)	NNNNNCCATNTWNNNWN	AATTTCCATTCTCATTA
M00059	V$YY1_01	0.773919	4975	(-)	NNNNNCCATNTWNNNWN	TAGCCTGAATGATTTTT
M00059	V$YY1_01	0.809633	5092	(-)	NNNNNCCATNTWNNNWN	TGTTATTTATGGTGTTA
M00059	V$YY1_01	0.783421	5361	(+)	NNNNNCCATNTWNNNWN	AAAAAGCATATTTATTT
M00059	V$YY1_01	0.78211	5422	(-)	NNNNNCCATNTWNNNWN	TTTTTTTGATGGAATGA
M00059	V$YY1_01	0.813893	5452	(+)	NNNNNCCATNTWNNNWN	TATATTCATTTTCTCCA
M00059	V$YY1_01	0.868938	5461	(+)	NNNNNCCATNTWNNNWN	TTTCTCCATTTTATGTT
M00059	V$YY1_01	0.804718	5482	(+)	NNNNNCCATNTWNNNWN	GGTTAACATTTTATCTT
M00059	V$YY1_01	0.770642	5539	(-)	NNNNNCCATNTWNNNWN	CTTAAAAGAGGAATTGG
M00059	V$YY1_01	0.850262	5615	(+)	NNNNNCCATNTWNNNWN	AAACGCCATATACATTT
M00059	V$YY1_01	0.781127	5621	(+)	NNNNNCCATNTWNNNWN	CATATACATTTCTTATA
M00059	V$YY1_01	0.863041	5638	(+)	NNNNNCCATNTWNNNWN	GATATCCATTCATTCAT
M00059	V$YY1_01	0.775229	5684	(-)	NNNNNCCATNTWNNNWN	GAAGGTCAATGAACTGA
M00059	V$YY1_01	0.839122	5711	(-)	NNNNNCCATNTWNNNWN	GAACATAAATGATATTT

M00059	V$YY1_01	0.821756	5748	(+)	NNNNNCCATNTWNNNWN	CAAAATCATATTTTGCA
M00059	V$YY1_01	0.847313	5845	(-)	NNNNNCCATNTWNNNWN	GTCAAAAAATGCTTGTG
M00059	V$YY1_01	0.851245	5868	(+)	NNNNNCCATNTWNNNWN	GAAAAACATTTTAGAAA
M00059	V$YY1_01	0.799476	5888	(+)	NNNNNCCATNTWNNNWN	AAATTTCATGTAACATT
M00069	V$YY1_02	0.752588	5918	(-)	NNNCGGCCATCTTGNCTSNW	AACTTTCAAAATGACTGATG
M00059	V$YY1_01	0.787353	5920	(-)	NNNNNCCATNTWNNNWN	CTTTCAAAATGACTGAT
M00059	V$YY1_01	0.782765	5981	(-)	NNNNNCCATNTWNNNWN	TTGCAACCATGAGGAGA
M00059	V$YY1_01	0.798165	6014	(-)	NNNNNCCATNTWNNNWN	ATGAATGAATGGATAAA
M00059	V$YY1_01	0.869921	6042	(-)	NNNNNCCATNTWNNNWN	GTGCAATTATGGACTGA
M00059	V$YY1_01	0.770642	6129	(-)	NNNNNCCATNTWNNNWN	CTCCAAGAATGTTTTAC
M00059	V$YY1_01	0.778178	6148	(-)	NNNNNCCATNTWNNNWN	AGTCACAAATGATTTTT
M00059	V$YY1_01	0.787025	6490	(-)	NNNNNCCATNTWNNNWN	GATGCAACATGTATGTT
M00059	V$YY1_01	0.790957	6582	(-)	NNNNNCCATNTWNNNWN	ATACTGCTATGGTCATG
M00059	V$YY1_01	0.856488	6595	(-)	NNNNNCCATNTWNNNWN	CATGGAAACTGGACACA
M00059	V$YY1_01	0.8519	6613	(-)	NNNNNCCATNTWNNNWN	CATACTACATGGAGAAA
M00059	V$YY1_01	0.811927	6627	(+)	NNNNNCCATNTWNNNWN	AAAATTCATTTTGCTTA
CSN1S2 Bos Tarus						
M00216	V$TATA_C	0.820174	13	(+)	NCTATAAAAR	GGTATAAATA
M00252	V$TATA_01	0.912205	14	(+)	STATAAAWRNNNNNN	GTATAAATAGTGTTG
M00216	V$TATA_C	0.768418	134	(+)	NCTATAAAAR	ATTGTAAAAC
M00216	V$TATA_C	0.779509	168	(+)	NCTATAAAAR	TTTTTTAAAG
M00252	V$TATA_01	0.778229	168	(+)	STATAAAWRNNNNNN	TTTTTTAAAGAAACT
M00216	V$TATA_C	0.768154	169	(+)	NCTATAAAAR	TTTTTAAAGA
M00216	V$TATA_C	0.789015	194	(+)	NCTATAAAAR	ACCTTAAGAT
M00216	V$TATA_C	0.862688	261	(+)	NCTATAAAAR	TTCTTAAAAA
M00216	V$TATA_C	0.841035	262	(+)	NCTATAAAAR	TCTTAAAAAA
M00216	V$TATA_C	0.79324	263	(+)	NCTATAAAAR	CTTAAAAAAA
M00252	V$TATA_01	0.771124	335	(+)	STATAAAWRNNNNNN	ATTTTAAGGAAGAGC
M00216	V$TATA_C	0.768154	364	(+)	NCTATAAAAR	TTTTTGAAAA
M00216	V$TATA_C	0.750726	365	(+)	NCTATAAAAR	TTTTGAAAAT
M00252	V$TATA_01	0.777721	365	(+)	STATAAAWRNNNNNN	TTTTGAAAATCCAGG
M00216	V$TATA_C	0.755479	444	(+)	NCTATAAAAR	CCTAACAAAG
M00216	V$TATA_C	0.742012	559	(+)	NCTATAAAAR	ATGATATAAG

M00252	V$TATA_01	0.786095	560	(+)	STATAAAWRNNNNNN	TGATATAAGCATTTC
M00216	V$TATA_C	0.768682	625	(+)	NCTATAAAAR	AAAATAAAAT
M00216	V$TATA_C	0.790335	660	(+)	NCTATAAAAR	GCTATATATA
M00252	V$TATA_01	0.881756	661	(+)	STATAAAWRNNNNNN	CTATATATAATGAAC
M00216	V$TATA_C	0.79192	662	(+)	NCTATAAAAR	TATATATAAT
M00252	V$TATA_01	0.855367	663	(+)	STATAAAWRNNNNNN	ATATATAATGAACTT
M00216	V$TATA_C	0.761025	678	(+)	NCTATAAAAR	ATTTCAAAAA
M00216	V$TATA_C	0.891207	693	(+)	NCTATAAAAR	ATTATAAAAT
M00252	V$TATA_01	0.820096	694	(+)	STATAAAWRNNNNNN	TTATAAAATTTAATA
M00252	V$TATA_01	0.770363	706	(+)	STATAAAWRNNNNNN	ATATATTTATCATCT
M00216	V$TATA_C	0.856615	722	(+)	NCTATAAAAR	ATTTTAAAAC
M00252	V$TATA_01	0.776453	722	(+)	STATAAAWRNNNNNN	ATTTTAAAACATTGT
M00216	V$TATA_C	0.76921	738	(+)	NCTATAAAAR	ATTATGAAAT
M00216	V$TATA_C	0.836546	747	(+)	NCTATAAAAR	TGCTTATAAA
M00252	V$TATA_01	0.806648	748	(+)	STATAAAWRNNNNNN	GCTTATAAAGTGAAT
M00216	V$TATA_C	0.775812	749	(+)	NCTATAAAAR	CTTATAAAGT
M00252	V$TATA_01	0.813499	750	(+)	STATAAAWRNNNNNN	TTATAAAGTGAATGT
M00216	V$TATA_C	0.763665	777	(+)	NCTATAAAAR	CCTGTAAGAG
M00216	V$TATA_C	0.775284	939	(+)	NCTATAAAAR	CAAATAAAAT
M00216	V$TATA_C	0.928439	954	(+)	NCTATAAAAR	TGTTTAAAAA
M00252	V$TATA_01	0.848769	955	(+)	STATAAAWRNNNNNN	GTTTAAAAATAATTG
M00252	V$TATA_01	0.770363	1005	(+)	STATAAAWRNNNNNN	TTATATGTCAAAAAG
M00216	V$TATA_C	0.744917	1020	(+)	NCTATAAAAR	TCATTAGAAT
M00216	V$TATA_C	0.757064	1064	(+)	NCTATAAAAR	ACTGGAAAAG
M00252	V$TATA_01	0.801319	1108	(+)	STATAAAWRNNNNNN	TTATATGTGGTCATA
M00216	V$TATA_C	0.781885	1169	(+)	NCTATAAAAR	AGTTAAAAAT
M00216	V$TATA_C	0.759968	1337	(+)	NCTATAAAAR	CCCTTATGAG
M00216	V$TATA_C	0.883021	1384	(+)	NCTATAAAAR	ATTTTAAAAA
M00216	V$TATA_C	0.779509	1385	(+)	NCTATAAAAR	TTTTAAAAAG
M00252	V$TATA_01	0.811977	1385	(+)	STATAAAWRNNNNNN	TTTTAAAAAGATTTC
M00216	V$TATA_C	0.821759	1429	(+)	NCTATAAAAR	TCCATAAGAT
M00252	V$TATA_01	0.780259	1437	(+)	STATAAAWRNNNNNN	ATTTAAGAAGAATTG
M00216	V$TATA_C	0.794824	1448	(+)	NCTATAAAAR	ATTGTAAAAA

M00216	V$TATA_C	0.832057	1450	(+)	NCTATAAAAR	TGTAAAAAAA
M00252	V$TATA_01	0.803857	1458	(+)	STATAAAWRNNNNNN	AAATATATGGCATTT
M00216	V$TATA_C	0.824927	1474	(+)	NCTATAAAAR	AGTATAAACA
M00252	V$TATA_01	0.809693	1475	(+)	STATAAAWRNNNNNN	GTATAAACATCATTA
M00252	V$TATA_01	0.77696	1547	(+)	STATAAAWRNNNNNN	GAATAAGTGGATAAA
M00252	V$TATA_01	0.792692	1555	(+)	STATAAAWRNNNNNN	GGATAAATTAATAAA
M00252	V$TATA_01	0.874905	1578	(+)	STATAAAWRNNNNNN	CTATTTATACATAGG
M00252	V$TATA_01	0.776199	1582	(+)	STATAAAWRNNNNNN	TTATACATAGGGTCA
M00252	V$TATA_01	0.783304	1717	(+)	STATAAAWRNNNNNN	GTATTCAAATCCATG
M00216	V$TATA_C	0.794824	1822	(+)	NCTATAAAAR	TGATTATAAG
M00252	V$TATA_01	0.82238	1823	(+)	STATAAAWRNNNNNN	GATTATAAGGAGAGT
M00252	V$TATA_01	0.774676	1825	(+)	STATAAAWRNNNNNN	TTATAAGGAGAGTAA
M00252	V$TATA_01	0.7729	1845	(+)	STATAAAWRNNNNNN	TTATTAATTAGCTTC
M00216	V$TATA_C	0.824927	1941	(+)	NCTATAAAAR	AGTATAATAA
M00252	V$TATA_01	0.849784	1942	(+)	STATAAAWRNNNNNN	GTATAATAAGATACA
M00252	V$TATA_01	0.770363	1969	(+)	STATAAAWRNNNNNN	TTATGTAAATATAAG
M00252	V$TATA_01	0.792438	1973	(+)	STATAAAWRNNNNNN	GTAAATATAAGAATA
M00216	V$TATA_C	0.740164	1974	(+)	NCTATAAAAR	TAAATATAAG
M00252	V$TATA_01	0.795991	1975	(+)	STATAAAWRNNNNNN	AAATATAAGAATATT
M00216	V$TATA_C	0.744653	1976	(+)	NCTATAAAAR	AATATAAGAA
M00252	V$TATA_01	0.784065	1977	(+)	STATAAAWRNNNNNN	ATATAAGAATATTAA
M00252	V$TATA_01	0.773154	1985	(+)	STATAAAWRNNNNNN	ATATTAAATTGTTTA
M00216	V$TATA_C	0.875363	1994	(+)	NCTATAAAAR	TGTTTATAAG
M00252	V$TATA_01	0.860188	1995	(+)	STATAAAWRNNNNNN	GTTTATAAGGATAAT
M00216	V$TATA_C	0.768682	2041	(+)	NCTATAAAAR	AGTGTATAAA
M00252	V$TATA_01	0.773154	2042	(+)	STATAAAWRNNNNNN	GTGTATAAACTTTGA
M00216	V$TATA_C	0.814629	2043	(+)	NCTATAAAAR	TGTATAAACT
M00252	V$TATA_01	0.77493	2044	(+)	STATAAAWRNNNNNN	GTATAAACTTTGAAA
M00216	V$TATA_C	0.807499	2050	(+)	NCTATAAAAR	ACTTTGAAAC
M00216	V$TATA_C	0.744125	2059	(+)	NCTATAAAAR	CCTATAATCA
M00216	V$TATA_C	0.740164	2088	(+)	NCTATAAAAR	CTTATGTAAG
M00252	V$TATA_01	0.773662	2098	(+)	STATAAAWRNNNNNN	TTATGAAGATGGACA
M00216	V$TATA_C	0.799313	2168	(+)	NCTATAAAAR	AGTTAAAAAA

M00216	V$TATA_C	0.755479	2169	(+)	NCTATAAAAR	GTTAAAAAAC
M00252	V$TATA_01	0.777721	2169	(+)	STATAAAWRNNNNNN	GTTAAAAAACGTACA
M00216	V$TATA_C	0.874043	2240	(+)	NCTATAAAAR	TTCTTAAAAG
M00216	V$TATA_C	0.832057	2263	(+)	NCTATAAAAR	ACCATAACAA
M00252	V$TATA_01	0.777214	2264	(+)	STATAAAWRNNNNNN	CCATAACAAGGACTT
M00216	V$TATA_C	0.85239	2278	(+)	NCTATAAAAR	TCTTTTAAAG
M00216	V$TATA_C	0.750198	2279	(+)	NCTATAAAAR	CTTTTAAAGT
M00252	V$TATA_01	0.81426	2279	(+)	STATAAAWRNNNNNN	CTTTTAAAGTTCAGG
M00126	V$GATA1_02	0.809375	14	(+)	NNNNNGATANKGNN	GTATAAATAGTGTT
M00075	V$GATA1_01	0.791214	30	(-)	SNNGATNNNN	GCCAATCCAT
M00075	V$GATA1_01	0.868213	34	(-)	SNNGATNNNN	ATCCATCAGA
M00076	V$GATA2_01	0.859269	34	(-)	NNNGATRNNN	ATCCATCAGA
M00126	V$GATA1_02	0.825625	39	(+)	NNNNNGATANKGNN	TCAGAGATATTCCA
M00075	V$GATA1_01	0.824778	41	(+)	SNNGATNNNN	AGAGATATTC
M00076	V$GATA2_01	0.866486	41	(+)	NNNGATRNNN	AGAGATATTC
M00077	V$GATA3_01	0.852016	42	(+)	NNGATARNG	GAGATATTC
M00203	V$GATA_C	0.836906	61	(-)	NGATAAGNMNN	ACTACTTGTCT
M00127	V$GATA1_03	0.850318	62	(-)	RNSNNGATAANNGN	CTACTTGTCTTCCT
M00203	V$GATA_C	0.916434	97	(-)	NGATAAGNMNN	TTCATTTATCT
M00126	V$GATA1_02	0.84625	98	(-)	NNNNNGATANKGNN	TCATTTATCTTTTT
M00127	V$GATA1_03	0.877021	98	(-)	RNSNNGATAANNGN	TCATTTATCTTTTT
M00128	V$GATA1_04	0.906556	98	(-)	NNCWGATARNNNN	TCATTTATCTTTT
M00076	V$GATA2_01	0.806946	100	(-)	NNNGATRNNN	ATTTATCTTT
M00077	V$GATA3_01	0.846699	100	(-)	NNGATARNG	ATTTATCTT
M00075	V$GATA1_01	0.816881	198	(+)	SNNGATNNNN	TAAGATTCTT
M00076	V$GATA2_01	0.841227	198	(+)	NNNGATRNNN	TAAGATTCTT
M00128	V$GATA1_04	0.837929	217	(+)	NNCWGATARNNNN	GTTTGATAGCTAA
M00075	V$GATA1_01	0.850938	218	(+)	SNNGATNNNN	TTTGATAGCT
M00076	V$GATA2_01	0.857014	218	(+)	NNNGATRNNN	TTTGATAGCT
M00077	V$GATA3_01	0.831192	219	(+)	NNGATARNG	TTGATAGCT
M00203	V$GATA_C	0.838148	220	(+)	NGATAAGNMNN	TGATAGCTAAC
M00076	V$GATA2_01	0.842129	227	(+)	NNNGATRNNN	TAACATAGCT
M00203	V$GATA_C	0.954334	238	(-)	NGATAAGNMNN	ATGATTTATCT

M00126	V$GATA1_02	0.7975	239	(-)	NNNNNGATANKGNN	TGATTTATCTCCTT
M00127	V$GATA1_03	0.864527	239	(-)	RNSNNGATAANNGN	TGATTTATCTCCTT
M00128	V$GATA1_04	0.899816	239	(-)	NNCWGATARNNNNN	TGATTTATCTCCT
M00075	V$GATA1_01	0.846496	241	(-)	SNNGATNNNN	ATTTATCTCC
M00076	V$GATA2_01	0.861976	241	(-)	NNNGATRNNN	ATTTATCTCC
M00077	V$GATA3_01	0.863979	241	(-)	NNGATARNG	ATTTATCTC
M00075	V$GATA1_01	0.817374	280	(+)	SNNGATNNNN	ACAGATGCTA
M00076	V$GATA2_01	0.823184	280	(+)	NNNGATRNNN	ACAGATGCTA
M00127	V$GATA1_03	0.817001	293	(-)	RNSNNGATAANNGN	ATTGTTATACTGCT
M00203	V$GATA_C	0.879466	302	(-)	NGATAAGNMNN	CTGCTCTATCT
M00126	V$GATA1_02	0.840313	303	(-)	NNNNNGATANKGNN	TGCTCTATCTTATG
M00128	V$GATA1_04	0.914216	303	(-)	NNCWGATARNNNNN	TGCTCTATCTTAT
M00075	V$GATA1_01	0.838105	305	(-)	SNNGATNNNN	CTCTATCTTA
M00076	V$GATA2_01	0.903924	305	(-)	NNNGATRNNN	CTCTATCTTA
M00077	V$GATA3_01	0.941958	305	(-)	NNGATARNG	CTCTATCTT
M00075	V$GATA1_01	0.853899	326	(-)	SNNGATNNNN	CACAATCTTA
M00076	V$GATA2_01	0.879567	326	(-)	NNNGATRNNN	CACAATCTTA
M00077	V$GATA3_01	0.928223	326	(-)	NNGATARNG	CACAATCTT
M00127	V$GATA1_03	0.821901	346	(-)	RNSNNGATAANNGN	GAGCTAATCATCAT
M00075	V$GATA1_01	0.851925	348	(-)	SNNGATNNNN	GCTAATCATC
M00076	V$GATA2_01	0.820929	348	(-)	NNNGATRNNN	GCTAATCATC
M00077	V$GATA3_01	0.830306	348	(-)	NNGATARNG	GCTAATCAT
M00075	V$GATA1_01	0.882527	351	(-)	SNNGATNNNN	AATCATCATG
M00076	V$GATA2_01	0.848895	351	(-)	NNNGATRNNN	AATCATCATG
M00075	V$GATA1_01	0.801086	369	(-)	SNNGATNNNN	GAAAATCCAG
M00075	V$GATA1_01	0.802073	406	(-)	SNNGATNNNN	CTGGATCACT
M00127	V$GATA1_03	0.793484	410	(-)	RNSNNGATAANNGN	ATCACTTTCCTCAT
M00203	V$GATA_C	0.831625	415	(-)	NGATAAGNMNN	TTTCCTCATCT
M00128	V$GATA1_04	0.828431	416	(-)	NNCWGATARNNNNN	TTCCTCATCTGTT
M00075	V$GATA1_01	0.837117	418	(-)	SNNGATNNNN	CCTCATCTGT
M00076	V$GATA2_01	0.846189	418	(-)	NNNGATRNNN	CCTCATCTGT
M00077	V$GATA3_01	0.894993	418	(-)	NNGATARNG	CCTCATCTG
M00076	V$GATA2_01	0.825891	482	(+)	NNNGATRNNN	CAACATACCT

M00075	V$GATA1_01	0.917572	511	(+)	SNNGATNNNN	GCAGATGCTT
M00076	V$GATA2_01	0.899865	511	(+)	NNNGATRNNN	GCAGATGCTT
M00126	V$GATA1_02	0.811875	528	(-)	NNNNNGATANKGNN	ACCACTATTTCTAT
M00127	V$GATA1_03	0.790789	528	(-)	RNSNNGATAANNGN	ACCACTATTTCTAT
M00203	V$GATA_C	0.873563	546	(-)	NGATAAGNMNN	CTCATTTATCA
M00126	V$GATA1_02	0.821875	547	(-)	NNNNNGATANKGNN	TCATTTATCAACAT
M00127	V$GATA1_03	0.900049	547	(-)	RNSNNGATAANNGN	TCATTTATCAACAT
M00128	V$GATA1_04	0.855086	547	(-)	NNCWGATARNNNN	TCATTTATCAACA
M00075	V$GATA1_01	0.802567	549	(-)	SNNGATNNNN	ATTTATCAAC
M00126	V$GATA1_02	0.770938	556	(+)	NNNNNGATANKGNN	AACATGATATAAGC
M00127	V$GATA1_03	0.805488	556	(+)	RNSNNGATAANNGN	AACATGATATAAGC
M00128	V$GATA1_04	0.86489	557	(+)	NNCWGATARNNNN	ACATGATATAAGC
M00075	V$GATA1_01	0.825765	558	(+)	SNNGATNNNN	CATGATATAA
M00076	V$GATA2_01	0.822282	558	(+)	NNNGATRNNN	CATGATATAA
M00077	V$GATA3_01	0.823217	559	(+)	NNGATARNG	ATGATATAA
M00203	V$GATA_C	0.845294	560	(+)	NGATAAGNMNN	TGATATAAGCA
M00203	V$GATA_C	0.846536	589	(-)	NGATAAGNMNN	AATATTTGTCC
M00126	V$GATA1_02	0.772813	629	(-)	NNNNNGATANKGNN	TAAAATATCTTAAG
M00075	V$GATA1_01	0.819348	631	(-)	SNNGATNNNN	AAATATCTTA
M00076	V$GATA2_01	0.894452	631	(-)	NNNGATRNNN	AAATATCTTA
M00077	V$GATA3_01	0.834736	631	(-)	NNGATARNG	AAATATCTT
M00203	V$GATA_C	0.929481	707	(-)	NGATAAGNMNN	TATATTTATCA
M00127	V$GATA1_03	0.879716	708	(-)	RNSNNGATAANNGN	ATATTTATCATCTT
M00128	V$GATA1_04	0.913297	708	(-)	NNCWGATARNNNN	ATATTTATCATCT
M00075	V$GATA1_01	0.838598	710	(-)	SNNGATNNNN	ATTTATCATC
M00076	V$GATA2_01	0.84258	710	(-)	NNNGATRNNN	ATTTATCATC
M00077	V$GATA3_01	0.823217	710	(-)	NNGATARNG	ATTTATCAT
M00076	V$GATA2_01	0.82138	713	(-)	NNNGATRNNN	TATCATCTTA
M00077	V$GATA3_01	0.844927	713	(-)	NNGATARNG	TATCATCTT
M00127	V$GATA1_03	0.783195	746	(+)	RNSNNGATAANNGN	ATGCTTATAAAGTG
M00203	V$GATA_C	0.888164	768	(-)	NGATAAGNMNN	GTGCATTATCC
M00126	V$GATA1_02	0.844375	769	(-)	NNNNNGATANKGNN	TGCATTATCCTGTA
M00128	V$GATA1_04	0.826593	769	(-)	NNCWGATARNNNN	TGCATTATCCTGT

M00075	V$GATA1_01	0.868707	771	(-)	SNNGATNNNN	CATTATCCTG
M00076	V$GATA2_01	0.916103	771	(-)	NNNGATRNNN	CATTATCCTG
M00077	V$GATA3_01	0.888347	771	(-)	NNGATARNG	CATTATCCT
M00126	V$GATA1_02	0.804688	792	(-)	NNNNNGATANKGNN	AAGAATATCTGGGA
M00075	V$GATA1_01	0.885982	794	(-)	SNNGATNNNN	GAATATCTGG
M00076	V$GATA2_01	0.907984	794	(-)	NNNGATRNNN	GAATATCTGG
M00077	V$GATA3_01	0.828977	794	(-)	NNGATARNG	GAATATCTG
M00075	V$GATA1_01	0.841066	801	(+)	SNNGATNNNN	TGGGATTCTT
M00076	V$GATA2_01	0.86378	801	(+)	NNNGATRNNN	TGGGATTCTT
M00126	V$GATA1_02	0.799063	816	(+)	NNNNNGATANKGNN	GGAATGATAAATTA
M00127	V$GATA1_03	0.83121	816	(+)	RNSNNGATAANNGN	GGAATGATAAATTA
M00128	V$GATA1_04	0.849265	817	(+)	NNCWGATARNNNN	GAATGATAAATTA
M00075	V$GATA1_01	0.773939	818	(+)	SNNGATNNNN	AATGATAAAT
M00076	V$GATA2_01	0.79567	818	(+)	NNNGATRNNN	AATGATAAAT
M00077	V$GATA3_01	0.823217	819	(+)	NNGATARNG	ATGATAAAT
M00203	V$GATA_C	0.893445	820	(+)	NGATAAGNMNN	TGATAAATTAA
M00076	V$GATA2_01	0.788453	842	(-)	NNNGATRNNN	GGCAATGCTA
M00075	V$GATA1_01	0.774926	848	(-)	SNNGATNNNN	GCTAATCTTA
M00076	V$GATA2_01	0.817772	848	(-)	NNNGATRNNN	GCTAATCTTA
M00077	V$GATA3_01	0.853788	848	(-)	NNGATARNG	GCTAATCTT
M00127	V$GATA1_03	0.803773	858	(+)	RNSNNGATAANNGN	AGACAGAGAATAAT
M00127	V$GATA1_03	0.791034	905	(+)	RNSNNGATAANNGN	GCAAAGATCACCAG
M00075	V$GATA1_01	0.873643	907	(-)	SNNGATNNNN	AAAGATCACC
M00076	V$GATA2_01	0.797925	907	(+)	NNNGATRNNN	AAAGATCACC
M00076	V$GATA2_01	0.841227	907	(-)	NNNGATRNNN	AAAGATCACC
M00075	V$GATA1_01	0.820829	943	(-)	SNNGATNNNN	TAAAATCTTG
M00076	V$GATA2_01	0.809202	943	(-)	NNNGATRNNN	TAAAATCTTG
M00077	V$GATA3_01	0.832964	943	(-)	NNGATARNG	TAAAATCTT
M00126	V$GATA1_02	0.825625	1076	(+)	NNNNNGATANKGNN	GCATTGATACGTAG
M00127	V$GATA1_03	0.813572	1076	(+)	RNSNNGATAANNGN	GCATTGATACGTAG
M00075	V$GATA1_01	0.82922	1078	(+)	SNNGATNNNN	ATTGATACGT
M00076	V$GATA2_01	0.786649	1078	(+)	NNNGATRNNN	ATTGATACGT
M00203	V$GATA_C	0.851196	1080	(+)	NGATAAGNMNN	TGATACGTAGT

M00203	V$GATA_C	0.840323	1103	(-)	NGATAAGNMNN	GGGACTTATAT
M00076	V$GATA2_01	0.804691	1116	(+)	NNNGATRNNN	GGTCATAGTA
M00126	V$GATA1_02	0.772188	1126	(+)	NNNNNGATANKGNN	TTAGAGATTGAGCT
M00075	V$GATA1_01	0.83465	1128	(+)	SNNGATNNNN	AGAGATTGAG
M00076	V$GATA2_01	0.815065	1128	(+)	NNNGATRNNN	AGAGATTGAG
M00077	V$GATA3_01	0.925565	1129	(+)	NNGATARNG	GAGATTGAG
M00075	V$GATA1_01	0.812932	1143	(+)	SNNGATNNNN	GGGGATCTTA
M00075	V$GATA1_01	0.774432	1143	(-)	SNNGATNNNN	GGGGATCTTA
M00076	V$GATA2_01	0.837167	1143	(+)	NNNGATRNNN	GGGGATCTTA
M00076	V$GATA2_01	0.856563	1143	(-)	NNNGATRNNN	GGGGATCTTA
M00075	V$GATA1_01	0.780355	1152	(-)	SNNGATNNNN	AGAAATCAAA
M00075	V$GATA1_01	0.828727	1157	(-)	SNNGATNNNN	TCAAATCTTC
M00076	V$GATA2_01	0.820929	1157	(-)	NNNGATRNNN	TCAAATCTTC
M00077	V$GATA3_01	0.853788	1157	(-)	NNGATARNG	TCAAATCTT
M00075	V$GATA1_01	0.824284	1173	(-)	SNNGATNNNN	AAAAATCTCA
M00076	V$GATA2_01	0.838069	1173	(-)	NNNGATRNNN	AAAAATCTCA
M00127	V$GATA1_03	0.797403	1187	(-)	RNSNNGATAANNGN	GTGGTTATTCTGTC
M00075	V$GATA1_01	0.845508	1207	(+)	SNNGATNNNN	AGTGATGTTC
M00076	V$GATA2_01	0.826793	1207	(+)	NNNGATRNNN	AGTGATGTTC
M00076	V$GATA2_01	0.796572	1240	(-)	NNNGATRNNN	GCCTGTCCCA
M00075	V$GATA1_01	0.771471	1258	(+)	SNNGATNNNN	TGGGATCAGC
M00075	V$GATA1_01	0.851431	1258	(-)	SNNGATNNNN	TGGGATCAGC
M00076	V$GATA2_01	0.840325	1258	(+)	NNNGATRNNN	TGGGATCAGC
M00076	V$GATA2_01	0.841678	1258	(-)	NNNGATRNNN	TGGGATCAGC
M00075	V$GATA1_01	0.771471	1280	(+)	SNNGATNNNN	TGGGATCAGC
M00075	V$GATA1_01	0.851431	1280	(-)	SNNGATNNNN	TGGGATCAGC
M00076	V$GATA2_01	0.840325	1280	(+)	NNNGATRNNN	TGGGATCAGC
M00076	V$GATA2_01	0.841678	1280	(-)	NNNGATRNNN	TGGGATCAGC
M00127	V$GATA1_03	0.823371	1293	(-)	RNSNNGATAANNGN	CTTCTTGTCCCCAC
M00127	V$GATA1_03	0.791034	1313	(-)	RNSNNGATAANNGN	GTTCTTTTCACCTG
M00076	V$GATA2_01	0.797023	1338	(-)	NNNGATRNNN	CCTTATGAGG
M00075	V$GATA1_01	0.780849	1375	(-)	SNNGATNNNN	TGAAATCTGA
M00076	V$GATA2_01	0.827244	1375	(-)	NNNGATRNNN	TGAAATCTGA

M00075	V$GATA1_01	0.801579	1380	(+)	SNNGATNNNN	TCTGATTTTA
M00076	V$GATA2_01	0.797023	1380	(+)	NNNGATRNNN	TCTGATTTTA
M00075	V$GATA1_01	0.787266	1391	(+)	SNNGATNNNN	AAAGATTTCT
M00076	V$GATA2_01	0.816419	1391	(+)	NNNGATRNNN	AAAGATTTCT
M00126	V$GATA1_02	0.807813	1414	(+)	NNNNNGATANKGNN	TTTCTGATATGATT
M00128	V$GATA1_04	0.867647	1415	(+)	NNCWGATARNNNN	TTCTGATATGATT
M00075	V$GATA1_01	0.837611	1416	(+)	SNNGATNNNN	TCTGATATGA
M00076	V$GATA2_01	0.867839	1416	(+)	NNNGATRNNN	TCTGATATGA
M00077	V$GATA3_01	0.858219	1417	(+)	NNGATARNG	CTGATATGA
M00203	V$GATA_C	0.838148	1418	(+)	NGATAAGNMNN	TGATATGATTC
M00075	V$GATA1_01	0.822804	1421	(+)	SNNGATNNNN	TATGATTCTC
M00076	V$GATA2_01	0.826342	1421	(+)	NNNGATRNNN	TATGATTCTC
M00075	V$GATA1_01	0.831688	1479	(-)	SNNGATNNNN	AAACATCATT
M00076	V$GATA2_01	0.822282	1479	(-)	NNNGATRNNN	AAACATCATT
M00126	V$GATA1_02	0.859375	1498	(-)	NNNNNGATANKGNN	CACTGTATCTTTAT
M00127	V$GATA1_03	0.824841	1498	(-)	RNSNNGATAANNGN	CACTGTATCTTTAT
M00075	V$GATA1_01	0.804047	1500	(-)	SNNGATNNNN	CTGTATCTTT
M00076	V$GATA2_01	0.846189	1500	(-)	NNNGATRNNN	CTGTATCTTT
M00077	V$GATA3_01	0.86265	1500	(-)	NNGATARNG	CTGTATCTT
M00076	V$GATA2_01	0.820478	1527	(+)	NNNGATRNNN	TGACATAGCA
M00127	V$GATA1_03	0.83856	1551	(+)	RNSNNGATAANNGN	AAGTGGATAAATTA
M00075	V$GATA1_01	0.777394	1553	(+)	SNNGATNNNN	GTGGATAAAT
M00076	V$GATA2_01	0.786649	1553	(+)	NNNGATRNNN	GTGGATAAAT
M00203	V$GATA_C	0.890649	1555	(+)	NGATAAGNMNN	GGATAAATTAA
M00126	V$GATA1_02	0.810937	1582	(+)	NNNNNGATANKGNN	TTATACATAGGGTC
M00076	V$GATA2_01	0.794317	1596	(-)	NNNGATRNNN	AATTATGCCA
M00076	V$GATA2_01	0.824087	1612	(-)	NNNGATRNNN	TGGTATGCCC
M00076	V$GATA2_01	0.80018	1647	(+)	NNNGATRNNN	TAAGATTTTA
M00077	V$GATA3_01	0.832964	1648	(+)	NNGATARNG	AAGATTTTA
M00075	V$GATA1_01	0.828233	1710	(-)	SNNGATNNNN	ACTCATCGTA
M00076	V$GATA2_01	0.854308	1710	(-)	NNNGATRNNN	ACTCATCGTA
M00075	V$GATA1_01	0.797631	1741	(-)	SNNGATNNNN	ATGAATCTAC
M00203	V$GATA_C	0.900901	1748	(-)	NGATAAGNMNN	TACTTTTATCA

M00126	V$GATA1_02	0.815313	1749	(-)	NNNNNGATANKGNN	ACTTTTATCAGTCT
M00127	V$GATA1_03	0.902499	1749	(-)	RNSNNGATAANNGN	ACTTTTATCAGTCT
M00128	V$GATA1_04	0.930453	1749	(-)	NNCWGATARNNNNN	ACTTTTATCAGTC
M00077	V$GATA3_01	0.849358	1751	(-)	NNGATARNG	TTTTATCAG
M00203	V$GATA_C	0.844051	1775	(-)	NGATAAGNMNN	TCTAATTATCT
M00126	V$GATA1_02	0.785	1776	(-)	NNNNNGATANKGNN	CTAATTATCTGAAG
M00127	V$GATA1_03	0.814307	1776	(-)	RNSNNGATAANNGN	CTAATTATCTGAAG
M00128	V$GATA1_04	0.922794	1776	(-)	NNCWGATARNNNNN	CTAATTATCTGAA
M00075	V$GATA1_01	0.821816	1778	(-)	SNNGATNNNNN	AATTATCTGA
M00076	V$GATA2_01	0.887686	1778	(-)	NNNGATRNNN	AATTATCTGA
M00077	V$GATA3_01	0.860877	1778	(-)	NNGATARNG	AATTATCTG
M00126	V$GATA1_02	0.844375	1784	(+)	NNNNNGATANKGNN	CTGAAGATAATTTA
M00127	V$GATA1_03	0.872856	1784	(+)	RNSNNGATAANNGN	CTGAAGATAATTTA
M00128	V$GATA1_04	0.837316	1785	(+)	NNCWGATARNNNNN	TGAAGATAATTTA
M00075	V$GATA1_01	0.857354	1786	(+)	SNNGATNNNNN	GAAGATAATT
M00076	V$GATA2_01	0.894001	1786	(+)	NNNGATRNNN	GAAGATAATT
M00077	V$GATA3_01	0.866637	1787	(+)	NNGATARNG	AAGATAATT
M00203	V$GATA_C	0.85803	1788	(+)	NGATAAGNMNN	AGATAATTTAA
M00075	V$GATA1_01	0.791214	1820	(+)	SNNGATNNNNN	TGTGATTATA
M00076	V$GATA2_01	0.782138	1820	(+)	NNNGATRNNN	TGTGATTATA
M00077	V$GATA3_01	0.858662	1821	(+)	NNGATARNG	GTGATTATA
M00127	V$GATA1_03	0.823371	1821	(+)	RNSNNGATAANNGN	GTGATTATAAGGAG
M00127	V$GATA1_03	0.790544	1821	(-)	RNSNNGATAANNGN	GTGATTATAAGGAG
M00127	V$GATA1_03	0.801323	1848	(-)	RNSNNGATAANNGN	TTAATTAGCTTCTT
M00127	V$GATA1_03	0.810632	1856	(-)	RNSNNGATAANNGN	CTTCTTGTCTATCT
M00203	V$GATA_C	0.858341	1859	(-)	NGATAAGNMNN	CTTGTCTATCT
M00126	V$GATA1_02	0.774687	1860	(-)	NNNNNGATANKGNN	TTGTCTATCTCACA
M00128	V$GATA1_04	0.879596	1860	(-)	NNCWGATARNNNNN	TTGTCTATCTCAC
M00075	V$GATA1_01	0.840573	1862	(-)	SNNGATNNNNN	GTCTATCTCA
M00076	V$GATA2_01	0.895354	1862	(-)	NNNGATRNNN	GTCTATCTCA
M00077	V$GATA3_01	0.892335	1862	(-)	NNGATARNG	GTCTATCTC
M00076	V$GATA2_01	0.79161	1895	(-)	NNNGATRNNN	CTTCATCTTT
M00077	V$GATA3_01	0.859991	1895	(-)	NNGATARNG	CTTCATCTT

M00127	V$GATA1_03	0.811612	1899	(-)	RNSNNGATAANNGN	ATCTTTACCTGCCT
M00128	V$GATA1_04	0.812806	1899	(-)	NNCWGATARNNNNN	ATCTTTACCTGCC
M00075	V$GATA1_01	0.775913	1917	(+)	SNNGATNNNN	GCTGTTGCCC
M00127	V$GATA1_03	0.783684	1938	(+)	RNSNNGATAANNGN	GTAAGTATAATAAG
M00126	V$GATA1_02	0.795625	1946	(+)	NNNNNGATANKGNN	AATAAGATACAGAT
M00128	V$GATA1_04	0.856311	1947	(+)	NNCWGATARNNNNN	ATAAGATACAGAT
M00075	V$GATA1_01	0.812438	1948	(+)	SNNGATNNNN	TAAGATACAG
M00076	V$GATA2_01	0.878665	1948	(+)	NNNGATRNNN	TAAGATACAG
M00077	V$GATA3_01	0.86265	1949	(+)	NNGATARNG	AAGATACAG
M00203	V$GATA_C	0.853681	1950	(+)	NGATAAGNMNN	AGATACAGATG
M00127	V$GATA1_03	0.800833	1952	(+)	RNSNNGATAANNGN	ATACAGATGAGAAT
M00128	V$GATA1_04	0.81587	1953	(+)	NNCWGATARNNNNN	TACAGATGAGAAT
M00075	V$GATA1_01	0.790227	1954	(+)	SNNGATNNNN	ACAGATGAGA
M00076	V$GATA2_01	0.803338	1954	(+)	NNNGATRNNN	ACAGATGAGA
M00077	V$GATA3_01	0.859991	1955	(+)	NNGATARNG	CAGATGAGA
M00127	V$GATA1_03	0.784174	1973	(+)	RNSNNGATAANNGN	GTAAATATAAGAAT
M00127	V$GATA1_03	0.782215	1993	(-)	RNSNNGATAANNGN	TTGTTTATAAGGAT
M00126	V$GATA1_02	0.840313	1999	(+)	NNNNNGATANKGNN	ATAAGGATAATGTC
M00127	V$GATA1_03	0.888535	1999	(+)	RNSNNGATAANNGN	ATAAGGATAATGTC
M00075	V$GATA1_01	0.799605	2001	(+)	SNNGATNNNN	AAGGATAATG
M00076	V$GATA2_01	0.874605	2001	(+)	NNNGATRNNN	AAGGATAATG
M00077	V$GATA3_01	0.888347	2002	(+)	NNGATARNG	AGGATAATG
M00203	V$GATA_C	0.863312	2003	(+)	NGATAAGNMNN	GGATAATGTCA
M00127	V$GATA1_03	0.823616	2015	(+)	RNSNNGATAANNGN	GGGTAGACAGCAGG
M00126	V$GATA1_02	0.780938	2029	(+)	NNNNNGATANKGNN	GAATAGAGAGGGAG
M00077	V$GATA3_01	0.834293	2032	(+)	NNGATARNG	TAGAGAGGG
M00127	V$GATA1_03	0.785154	2100	(+)	RNSNNGATAANNGN	ATGAAGATGGACAA
M00075	V$GATA1_01	0.868213	2102	(+)	SNNGATNNNN	GAAGATGGAC
M00076	V$GATA2_01	0.873252	2102	(+)	NNNGATRNNN	GAAGATGGAC
M00077	V$GATA3_01	0.821444	2103	(+)	NNGATARNG	AAGATGGAC
M00203	V$GATA_C	0.83908	2108	(+)	NGATAAGNMNN	GGACAAAAATA
M00127	V$GATA1_03	0.808672	2129	(-)	RNSNNGATAANNGN	AGTGTTCTCCTCCT
M00075	V$GATA1_01	0.814906	2142	(+)	SNNGATNNNN	TAGGATTGGA

M00076	V$GATA2_01	0.85746<br>5	2142	(+)	NNNGATRNNN	TAGGATTGGA
M00077	V$GATA3_01	0.86885<br>2	2143	(+)	NNGATARNG	AGGATTGGA
M00075	V$GATA1_01	0.85784<br>8	2231	(-)	SNNGATNNNN	AAGAATCACT
M00076	V$GATA2_01	0.80559<br>3	2231	(-)	NNNGATRNNN	AAGAATCACT
M00075	V$GATA1_01	0.82231	2311	(+)	SNNGATNNNN	GTAGATTCCC
M00076	V$GATA2_01	0.78935<br>5	2311	(+)	NNNGATRNNN	GTAGATTCCC
M00076	V$GATA2_01	0.79747<br>4	2333	(-)	NNNGATRNNN	GCATATGTCA
M00075	V$GATA1_01	0.77640<br>7	2379	(+)	SNNGATNNNN	AAGGATCCTT
M00075	V$GATA1_01	0.77640<br>7	2379	(-)	SNNGATNNNN	AAGGATCCTT
M00076	V$GATA2_01	0.82318<br>4	2379	(+)	NNNGATRNNN	AAGGATCCTT
M00076	V$GATA2_01	0.82318<br>4	2379	(-)	NNNGATRNNN	AAGGATCCTT
M00075	V$GATA1_01	0.84402<br>8	2393	(+)	SNNGATNNNN	AATGATGTGT
M00076	V$GATA2_01	0.82859<br>7	2393	(+)	NNNGATRNNN	AATGATGTGT
M00075	V$GATA1_01	0.80108<br>6	2421	(+)	SNNGATNNNN	GGAGTTGGGC
M00075	V$GATA1_01	0.85389<br>9	2483	(-)	SNNGATNNNN	CTCCATCCCT
M00076	V$GATA2_01	0.86558<br>4	2483	(-)	NNNGATRNNN	CTCCATCCCT
M00077	V$GATA3_01	0.86043<br>4	2483	(-)	NNGATARNG	CTCCATCCC
M00127	V$GATA1_03	0.78613<br>4	2498	(+)	RNSNNGATAANNGN	AGGAAGATCCCATG
M00075	V$GATA1_01	0.80997	2500	(+)	SNNGATNNNN	GAAGATCCCA
M00075	V$GATA1_01	0.77739<br>4	2500	(-)	SNNGATNNNN	GAAGATCCCA
M00076	V$GATA2_01	0.84483<br>5	2500	(+)	NNNGATRNNN	GAAGATCCCA
M00076	V$GATA2_01	0.84889<br>5	2500	(-)	NNNGATRNNN	GAAGATCCCA
M00136	V$OCT1_02	0.78451<br>5	41	(+)	NNGAATATKCANNNN	AGAGATATTCCATTG
M00162	V$OCT1_06	0.87812<br>5	44	(-)	CWNAWTKWSATRYN	GATATTCCATTGCC
M00137	V$OCT1_03	0.84907<br>2	86	(+)	NNNRTAATNANNN	GAGGTAATATTTT
M00138	V$OCT1_04	0.87871<br>2	87	(-)	NNNNNNNNWATGCAAATNNNW<br>NNW	AGGTAATATTTTCATTTATCTTT
M00135	V$OCT1_01	0.78882<br>1	89	(-)	NNNNWTATGCAAATNTNNN	GTAATATTTTCATTTATCT
M00162	V$OCT1_06	0.9375	91	(+)	CWNAWTKWSATRYN	AATATTTTCATTTA
M00195	V$OCT1_Q6	0.82232<br>5	91	(-)	NNNNATGCAAATNAN	AATATTTTCATTTAT
M00248	V$OCT1_07	0.81238<br>1	91	(+)	TNTATGNTAATT	AATATTTTCATT
M00138	V$OCT1_04	0.80593<br>9	93	(-)	NNNNNNNNWATGCAAATNNNW<br>NNW	TATTTTCATTTATCTTTTGTTA
M00162	V$OCT1_06	0.80546<br>9	104	(+)	CWNAWTKWSATRYN	ATCTTTTTGTTATT
M00162	V$OCT1_06	0.85273<br>4	106	(+)	CWNAWTKWSATRYN	CTTTTTGTTATTCT

M00162	V$OCT1_06	0.834375	123	(+)	CWNAWTKWSATRYN	TACCTTGTAATATT
M00162	V$OCT1_06	0.806641	124	(-)	CWNAWTKWSATRYN	ACCTTGTAATATTG
M00137	V$OCT1_03	0.884631	126	(+)	NNNRTAATNANNN	CTTGTAATATTGT
M00138	V$OCT1_04	0.804475	127	(+)	NNNNNNNWATGCAAATNNNWNNW	TTGTAATATTGTAAAACCTAATA
M00162	V$OCT1_06	0.826562	131	(+)	CWNAWTKWSATRYN	AATATTGTAAAACC
M00138	V$OCT1_04	0.786909	163	(-)	NNNNNNNWATGCAAATNNNWNNW	AAGTATTTTTAAAGAAACTAAT
M00162	V$OCT1_06	0.828906	230	(-)	CWNAWTKWSATRYN	CATAGCTAATGATT
M00137	V$OCT1_03	0.888977	232	(+)	NNNRTAATNANNN	TAGCTAATGATTT
M00138	V$OCT1_04	0.847762	265	(-)	NNNNNNNWATGCAAATNNNWNNW	TAAAAAAATTAACATACAGATGC
M00135	V$OCT1_01	0.858642	267	(-)	NNNNWTATGCAAATNTNNN	AAAAAATTAACATACAGAT
M00162	V$OCT1_06	0.88125	269	(+)	CWNAWTKWSATRYN	AAAATTAACATACA
M00138	V$OCT1_04	0.792137	270	(+)	NNNNNNNWATGCAAATNNNWNNW	AAATTAACATACAGATGCTAGAA
M00248	V$OCT1_07	0.993216	271	(-)	TNTATGNTAATT	AATTAACATACA
M00162	V$OCT1_06	0.873437	290	(+)	CWNAWTKWSATRYN	GAAATTGTTATACT
M00162	V$OCT1_06	0.804297	306	(-)	CWNAWTKWSATRYN	TCTATCTTATGATG
M00162	V$OCT1_06	0.801562	313	(+)	CWNAWTKWSATRYN	TATGATGAAATGCC
M00162	V$OCT1_06	0.816406	319	(-)	CWNAWTKWSATRYN	GAAATGCCACAATC
M00162	V$OCT1_06	0.804687	326	(+)	CWNAWTKWSATRYN	CACAATCTTATTTT
M00137	V$OCT1_03	0.843935	346	(+)	NNNRTAATNANNN	GAGCTAATCATCA
M00162	V$OCT1_06	0.855078	354	(+)	CWNAWTKWSATRYN	CATCATGAGGTTTT
M00135	V$OCT1_01	0.734071	361	(+)	NNNNWTATGCAAATNTNNN	AGGTTTTTGAAAATCCAGG
M00136	V$OCT1_02	0.785057	364	(-)	NNGAATATKCANNNN	TTTTTGAAAATCCAG
M00162	V$OCT1_06	0.862891	385	(+)	CWNAWTKWSATRYN	GCACATTAGATGTA
M00162	V$OCT1_06	0.821484	386	(-)	CWNAWTKWSATRYN	CACATTAGATGTAT
M00137	V$OCT1_03	0.866456	424	(+)	NNNRTAATNANNN	CTGTTAATCAGAC
M00138	V$OCT1_04	0.780008	470	(-)	NNNNNNNWATGCAAATNNNWNNW	AGAAAGAATTTACAACATACCTA
M00135	V$OCT1_01	0.752003	502	(-)	NNNNWTATGCAAATNTNNN	CTGACAATAGCAGATGCTT
M00138	V$OCT1_04	0.845044	557	(-)	NNNNNNNWATGCAAATNNNWNNW	ACATGATATAAGCATTTCTTTAA
M00135	V$OCT1_01	0.765548	559	(-)	NNNNWTATGCAAATNTNNN	ATGATATAAGCATTTCTTT
M00162	V$OCT1_06	0.855078	567	(-)	CWNAWTKWSATRYN	AGCATTTCTTTAAC
M00138	V$OCT1_04	0.787537	573	(-)	NNNNNNNWATGCAAATNNNWNNW	TCTTTAACTCTTAGTTAATATTT
M00136	V$OCT1_02	0.776665	583	(-)	NNGAATATKCANNNN	TTAGTTAATATTTGT
M00136	V$OCT1_02	0.783974	586	(+)	NNGAATATKCANNNN	GTTAATATTTGTCCA

M00162	V$OCT1_06	0.800781	599	(-)	CWNAWTKWSATRYN	CATGTTCCAGTTTC
M00162	V$OCT1_06	0.834375	608	(+)	CWNAWTKWSATRYN	GTTTCTTTCATCCA
M00138	V$OCT1_04	0.840234	613	(+)	NNNNNNNWATGCAAATNNNWNNW	TTTCATCCATGGAAAATAAAATA
M00248	V$OCT1_07	0.820013	618	(+)	TNTATGNTAATT	TCCATGGAAAAT
M00162	V$OCT1_06	0.855469	624	(+)	CWNAWTKWSATRYN	GAAAATAAAATATC
M00162	V$OCT1_06	0.813672	624	(-)	CWNAWTKWSATRYN	GAAAATAAAATATC
M00162	V$OCT1_06	0.821484	625	(-)	CWNAWTKWSATRYN	AAAATAAAATATCT
M00248	V$OCT1_07	0.798601	626	(-)	TNTATGNTAATT	AAATAAAATATC
M00135	V$OCT1_01	0.740939	634	(+)	NNNNWTATGCAAATNTNNN	TATCTTAAGTAAAATTGAG
M00138	V$OCT1_04	0.780427	655	(+)	NNNNNNNWATGCAAATNNNWNNW	AAGTGGCTATATATAATGAACTT
M00162	V$OCT1_06	0.855078	662	(-)	CWNAWTKWSATRYN	TATATATAATGAAC
M00137	V$OCT1_03	0.872382	664	(+)	NNNRTAATNANNN	TATATAATGAACT
M00138	V$OCT1_04	0.785236	670	(+)	NNNNNNNWATGCAAATNNNWNNW	ATGAACTTATTTCAAAAATTTAA
M00138	V$OCT1_04	0.78189	671	(-)	NNNNNNNWATGCAAATNNNWNNW	TGAACTTATTTCAAAAATTTAAA
M00162	V$OCT1_06	0.813672	675	(-)	CWNAWTKWSATRYN	CTTATTTCAAAAAT
M00162	V$OCT1_06	0.826172	677	(-)	CWNAWTKWSATRYN	TATTTCAAAAATTT
M00138	V$OCT1_04	0.874111	679	(+)	NNNNNNNWATGCAAATNNNWNNW	TTTCAAAAATTTAAATTATAAAA
M00138	V$OCT1_04	0.863446	680	(-)	NNNNNNNWATGCAAATNNNWNNW	TTCAAAAATTTAAATTATAAAAT
M00135	V$OCT1_01	0.739603	682	(-)	NNNNWTATGCAAATNTNNN	CAAAAATTTAAATTATAAA
M00162	V$OCT1_06	0.834375	683	(+)	CWNAWTKWSATRYN	AAAAATTTAAATTA
M00195	V$OCT1_Q6	0.848253	683	(+)	NNNNATGCAAATNAN	AAAAATTTAAATTAT
M00162	V$OCT1_06	0.847656	684	(+)	CWNAWTKWSATRYN	AAAATTTAAATTAT
M00162	V$OCT1_06	0.916797	684	(-)	CWNAWTKWSATRYN	AAAATTTAAATTAT
M00248	V$OCT1_07	0.832309	684	(+)	TNTATGNTAATT	AAAATTTAAATT
M00138	V$OCT1_04	0.791928	685	(+)	NNNNNNNWATGCAAATNNNWNNW	AAATTTAAATTATAAAATTTAAT
M00138	V$OCT1_04	0.783563	686	(-)	NNNNNNNWATGCAAATNNNWNNW	AATTTAAATTATAAAATTTAATA
M00248	V$OCT1_07	0.832309	686	(-)	TNTATGNTAATT	AATTTAAATTAT
M00137	V$OCT1_03	0.845121	688	(-)	NNNRTAATNANNN	TTTAAATTATAAA
M00162	V$OCT1_06	0.813672	690	(-)	CWNAWTKWSATRYN	TAAATTATAAAATT
M00162	V$OCT1_06	0.829297	692	(+)	CWNAWTKWSATRYN	AATTATAAAATTTA
M00138	V$OCT1_04	0.780636	693	(-)	NNNNNNNWATGCAAATNNNWNNW	ATTATAAAATTTAATATATTTAT
M00138	V$OCT1_04	0.814095	695	(-)	NNNNNNNWATGCAAATNNNWNNW	TATAAAATTTAATATATTTATCA
M00135	V$OCT1_01	0.755818	697	(-)	NNNNWTATGCAAATNTNNN	TAAAATTTAATATATTTAT

M00162	V$OCT1_06	0.88125	697	(+)	CWNAWTKWSATRYN	TAAAATTTAATATA
M00162	V$OCT1_06	0.898437	698	(-)	CWNAWTKWSATRYN	AAAATTTAATATAT
M00138	V$OCT1_04	0.798829	701	(-)	NNNNNNNWATGCAAATNNNWNNW	ATTTAATATATTTATCATCTTAT
M00138	V$OCT1_04	0.809494	704	(-)	NNNNNNNWATGCAAATNNNWNNW	TAATATATTTATCATCTTATTTT
M00137	V$OCT1_03	0.865666	706	(-)	NNNRTAATNANNN	ATATATTTATCAT
M00162	V$OCT1_06	0.857812	719	(+)	CWNAWTKWSATRYN	CTTATTTTAAAACA
M00138	V$OCT1_04	0.792765	725	(-)	NNNNNNNWATGCAAATNNNWNNW	TTAAAACATTGTCATTATGAAAT
M00162	V$OCT1_06	0.847656	729	(+)	CWNAWTKWSATRYN	AACATTGTCATTAT
M00162	V$OCT1_06	0.812109	730	(-)	CWNAWTKWSATRYN	ACATTGTCATTATG
M00137	V$OCT1_03	0.957329	733	(-)	NNNRTAATNANNN	TTGTCATTATGAA
M00162	V$OCT1_06	0.953125	737	(+)	CWNAWTKWSATRYN	CATTATGAAATGCT
M00138	V$OCT1_04	0.835425	738	(+)	NNNNNNNWATGCAAATNNNWNNW	ATTATGAAATGCTTATAAAGTGA
M00162	V$OCT1_06	0.842187	738	(-)	CWNAWTKWSATRYN	ATTATGAAATGCTT
M00137	V$OCT1_03	0.843935	749	(+)	NNNRTAATNANNN	CTTATAAAGTGAA
M00162	V$OCT1_06	0.821484	752	(+)	CWNAWTKWSATRYN	ATAAAGTGAATGTC
M00162	V$OCT1_06	0.932422	757	(+)	CWNAWTKWSATRYN	GTGAATGTCATGTG
M00162	V$OCT1_06	0.85	758	(-)	CWNAWTKWSATRYN	TGAATGTCATGTGC
M00135	V$OCT1_01	0.76612	761	(-)	NNNNWTATGCAAATNTNNN	ATGTCATGTGCATTATCCT
M00161	V$OCT1_05	0.850905	763	(+)	MKNATTTGCATAYY	GTCATGTGCATTAT
M00195	V$OCT1_Q6	0.804858	763	(-)	NNNNATGCAAATNAN	GTCATGTGCATTATC
M00136	V$OCT1_02	0.823227	765	(-)	NNGAATATKCANNNN	CATGTGCATTATCCT
M00136	V$OCT1_02	0.770439	789	(-)	NNGAATATKCANNNN	AACAAGAATATCTGG
M00162	V$OCT1_06	0.869922	796	(+)	CWNAWTKWSATRYN	ATATCTGGGATTCT
M00138	V$OCT1_04	0.827269	811	(+)	NNNNNNNWATGCAAATNNNWNNW	TAGTAGGAATGATAAATTAATAA
M00162	V$OCT1_06	0.831641	829	(-)	CWNAWTKWSATRYN	AATAACAAAAGCAG
M00138	V$OCT1_04	0.802593	838	(+)	NNNNNNNWATGCAAATNNNWNNW	AGCAGGCAATGCTAATCTTAAGA
M00135	V$OCT1_01	0.772987	840	(+)	NNNNWTATGCAAATNTNNN	CAGGCAATGCTAATCTTAA
M00195	V$OCT1_Q6	0.817959	842	(+)	NNNNATGCAAATNAN	GGCAATGCTAATCTT
M00248	V$OCT1_07	0.814925	843	(+)	TNTATGNTAATT	GCAATGCTAATC
M00138	V$OCT1_04	0.785864	867	(-)	NNNNNNNWATGCAAATNNNWNNW	ATAATTCCCATGCATACACATTC
M00162	V$OCT1_06	0.804297	867	(+)	CWNAWTKWSATRYN	ATAATTCCCATGCA
M00195	V$OCT1_Q6	0.820415	867	(-)	NNNNATGCAAATNAN	ATAATTCCCATGCAT
M00138	V$OCT1_04	0.794228	868	(+)	NNNNNNNWATGCAAATNNNWNNW	TAATTCCCATGCATACACATTCT

M00248	V$OCT1_07	0.849693	869	(-)	TNTATGNTAATT	AATTCCCATGCA
M00136	V$OCT1_02	0.812128	873	(-)	NNGAATATKCANNNN	CCCATGCATACACAT
M00162	V$OCT1_06	0.809375	886	(-)	CWNAWTKWSATRYN	ATTCTCTAAATTTG
M00138	V$OCT1_04	0.844835	888	(-)	NNNNNNNWATGCAAATNNNWNNW	TCTCTAAATTTGCACAGGCAAAG
M00135	V$OCT1_01	0.823922	890	(-)	NNNNWTATGCAAATNTNNN	TCTAAATTTGCACAGGCAA
M00248	V$OCT1_07	0.833369	894	(-)	TNTATGNTAATT	AATTTGCACAGG
M00138	V$OCT1_04	0.80343	895	(+)	NNNNNNNWATGCAAATNNNWNNW	ATTTGCACAGGCAAAGATCACCA
M00135	V$OCT1_01	0.742655	897	(+)	NNNNWTATGCAAATNTNNN	TTGCACAGGCAAAGATCAC
M00162	V$OCT1_06	0.816016	900	(-)	CWNAWTKWSATRYN	CACAGGCAAAGATC
M00162	V$OCT1_06	0.816406	919	(-)	CWNAWTKWSATRYN	CAAATTTAACAATT
M00138	V$OCT1_04	0.809076	928	(+)	NNNNNNNWATGCAAATNNNWNNW	CAATTTTGAGTCAAATAAAATCT
M00138	V$OCT1_04	0.787118	950	(-)	NNNNNNNWATGCAAATNNNWNNW	TTGCTGTTTAAAAATAATTGATT
M00162	V$OCT1_06	0.808203	961	(+)	CWNAWTKWSATRYN	AAATAATTGATTTC
M00162	V$OCT1_06	0.821484	962	(+)	CWNAWTKWSATRYN	AATAATTGATTTCA
M00138	V$OCT1_04	0.814931	963	(+)	NNNNNNNWATGCAAATNNNWNNW	ATAATTGATTTCAAATTTGTAGA
M00162	V$OCT1_06	0.839844	968	(-)	CWNAWTKWSATRYN	TGATTTCAAATTTG
M00138	V$OCT1_04	0.797783	970	(-)	NNNNNNNWATGCAAATNNNWNNW	ATTTCAAATTTGTAGATCTATAG
M00138	V$OCT1_04	0.81054	971	(+)	NNNNNNNWATGCAAATNNNWNNW	TTTCAAATTTGTAGATCTATAGA
M00135	V$OCT1_01	0.790538	972	(-)	NNNNWTATGCAAATNTNNN	TTCAAATTTGTAGATCTAT
M00138	V$OCT1_04	0.786909	986	(-)	NNNNNNNWATGCAAATNNNWNNW	TCTATAGAGTAAAATACTATTAT
M00137	V$OCT1_03	0.854998	999	(-)	NNNRTAATNANNN	ATACTATTATATG
M00162	V$OCT1_06	0.950391	1006	(-)	CWNAWTKWSATRYN	TATATGTCAAAAAG
M00162	V$OCT1_06	0.813281	1008	(-)	CWNAWTKWSATRYN	TATGTCAAAAAGTC
M00138	V$OCT1_04	0.820368	1015	(-)	NNNNNNNWATGCAAATNNNWNNW	AAAAGTCATTAGAATAACTTTAT
M00135	V$OCT1_01	0.775086	1017	(-)	NNNNWTATGCAAATNTNNN	AAGTCATTAGAATAACTTT
M00137	V$OCT1_03	0.880284	1017	(-)	NNNRTAATNANNN	AAGTCATTAGAAT
M00161	V$OCT1_05	0.850573	1019	(+)	MKNATTTGCATAYY	GTCATTAGAATAAC
M00248	V$OCT1_07	0.805385	1021	(-)	TNTATGNTAATT	CATTAGAATAAC
M00162	V$OCT1_06	0.805859	1028	(+)	CWNAWTKWSATRYN	ATAACTTTATTTCA
M00162	V$OCT1_06	0.847266	1032	(+)	CWNAWTKWSATRYN	CTTTATTTCACTTT
M00162	V$OCT1_06	0.808594	1040	(-)	CWNAWTKWSATRYN	CACTTTTCAGTTCT
M00138	V$OCT1_04	0.781263	1065	(-)	NNNNNNNWATGCAAATNNNWNNW	CTGGAAAAGTTGCATTGATACGT
M00135	V$OCT1_01	0.730446	1067	(-)	NNNNWTATGCAAATNTNNN	GGAAAAGTTGCATTGATAC

M00248	V$OCT1_07	0.824041	1071	(-)	TNTATGNTAATT	AAGTTGCATTGA
M00162	V$OCT1_06	0.829687	1103	(+)	CWNAWTKWSATRYN	GGGACTTATATGTG
M00162	V$OCT1_06	0.839844	1122	(-)	CWNAWTKWSATRYN	AGTATTAGAGATTG
M00162	V$OCT1_06	0.800781	1152	(-)	CWNAWTKWSATRYN	AGAAATCAAATCTT
M00162	V$OCT1_06	0.842187	1153	(-)	CWNAWTKWSATRYN	GAAATCAAATCTTC
M00162	V$OCT1_06	0.855469	1173	(+)	CWNAWTKWSATRYN	AAAAATCTCATTTG
M00162	V$OCT1_06	0.894531	1174	(-)	CWNAWTKWSATRYN	AAAATCTCATTTGG
M00136	V$OCT1_02	0.783433	1187	(+)	NNGAATATKCANNNN	GTGGTTATTCTGTCA
M00162	V$OCT1_06	0.800391	1202	(+)	CWNAWTKWSATRYN	GGTTCAGTGATGTT
M00162	V$OCT1_06	0.821484	1321	(-)	CWNAWTKWSATRYN	CACCTGCCAATTCT
M00138	V$OCT1_04	0.868256	1400	(-)	NNNNNNNWATGCAAATNNNWNNW	TGTTGTTTTTTGCATTTCTGATA
M00135	V$OCT1_01	0.795498	1402	(-)	NNNNWTATGCAAATNTNNN	TTGTTTTTTGCATTTCTGA
M00162	V$OCT1_06	0.877734	1404	(+)	CWNAWTKWSATRYN	GTTTTTTGCATTTC
M00195	V$OCT1_Q6	0.894378	1404	(-)	NNNNATGCAAATNAN	GTTTTTTGCATTTCT
M00162	V$OCT1_06	0.808203	1410	(-)	CWNAWTKWSATRYN	TGCATTTCTGATAT
M00162	V$OCT1_06	0.847656	1428	(+)	CWNAWTKWSATRYN	CTCCATAAGATTTA
M00138	V$OCT1_04	0.806566	1440	(+)	NNNNNNNWATGCAAATNNNWNNW	TAAGAAGAATTGTAAAAAAAATA
M00162	V$OCT1_06	0.898437	1460	(+)	CWNAWTKWSATRYN	ATATATGGCATTTT
M00162	V$OCT1_06	0.832812	1461	(-)	CWNAWTKWSATRYN	TATATGGCATTTTA
M00248	V$OCT1_07	0.800933	1461	(+)	TNTATGNTAATT	TATATGGCATTT
M00136	V$OCT1_02	0.78235	1462	(-)	NNGAATATKCANNNN	ATATGGCATTTTAGT
M00135	V$OCT1_01	0.781953	1466	(-)	NNNNWTATGCAAATNTNNN	GGCATTTTAGTATAAACAT
M00162	V$OCT1_06	0.805859	1466	(+)	CWNAWTKWSATRYN	GGCATTTTAGTATA
M00138	V$OCT1_04	0.846926	1470	(-)	NNNNNNNWATGCAAATNNNWNNW	TTTTAGTATAAACATCATTATTT
M00137	V$OCT1_03	0.918214	1481	(-)	NNNRTAATNANNN	ACATCATTATTTT
M00138	V$OCT1_04	0.828733	1483	(-)	NNNNNNNWATGCAAATNNNWNNW	ATCATTATTTTTCATCACTGTAT
M00137	V$OCT1_03	0.858949	1502	(-)	NNNRTAATNANNN	GTATCTTTATGTC
M00162	V$OCT1_06	0.821484	1522	(+)	CWNAWTKWSATRYN	GTACCTGACATAGC
M00138	V$OCT1_04	0.786491	1547	(+)	NNNNNNNWATGCAAATNNNWNNW	GAATAAGTGGATAAATTAATAAA
M00138	V$OCT1_04	0.794437	1553	(+)	NNNNNNNWATGCAAATNNNWNNW	GTGGATAAATTAATAAATAATTT
M00138	V$OCT1_04	0.796947	1557	(+)	NNNNNNNWATGCAAATNNNWNNW	ATAAATTAATAAATAATTTAGCT
M00248	V$OCT1_07	0.793513	1560	(-)	TNTATGNTAATT	AATTAATAAATA
M00162	V$OCT1_06	0.817187	1564	(-)	CWNAWTKWSATRYN	AATAAATAATTTAG

M00138	V$OCT1_04	0.807194	1565	(-)	NNNNNNNWATGCAAATNNNWNNW	ATAAATAATTTAGCTATTTATAC
M00162	V$OCT1_06	0.852344	1569	(+)	CWNAWTKWSATRYN	ATAATTTAGCTATT
M00138	V$OCT1_04	0.783563	1575	(-)	NNNNNNNWATGCAAATNNNWNNW	TAGCTATTTATACATAGGGTCAA
M00135	V$OCT1_01	0.735979	1594	(+)	NNNNWTATGCAAATNTNNN	TCAATTATGCCACTAATTT
M00162	V$OCT1_06	0.834375	1613	(-)	CWNAWTKWSATRYN	GGTATGCCCAAATG
M00138	V$OCT1_04	0.805939	1637	(+)	NNNNNNNWATGCAAATNNNWNNW	ATTTAGAAATTAAGATTTTACAT
M00135	V$OCT1_01	0.731782	1640	(-)	NNNNWTATGCAAATNTNNN	TAGAAATTAAGATTTTACA
M00162	V$OCT1_06	0.860547	1642	(+)	CWNAWTKWSATRYN	GAAATTAAGATTTT
M00162	V$OCT1_06	0.813672	1642	(-)	CWNAWTKWSATRYN	GAAATTAAGATTTT
M00248	V$OCT1_07	0.818105	1644	(-)	TNTATGNTAATT	AATTAAGATTTT
M00138	V$OCT1_04	0.828524	1645	(-)	NNNNNNNWATGCAAATNNNWNNW	ATTAAGATTTTACATTTCCTTCT
M00135	V$OCT1_01	0.739985	1647	(-)	NNNNWTATGCAAATNTNNN	TAAGATTTTACATTTCCTT
M00162	V$OCT1_06	0.885547	1649	(+)	CWNAWTKWSATRYN	AGATTTTACATTTC
M00195	V$OCT1_Q6	0.820688	1649	(-)	NNNNATGCAAATNAN	AGATTTTACATTTCC
M00162	V$OCT1_06	0.813672	1650	(-)	CWNAWTKWSATRYN	GATTTTACATTTCC
M00162	V$OCT1_06	0.85	1655	(-)	CWNAWTKWSATRYN	TACATTTCCTTCTC
M00135	V$OCT1_01	0.751049	1668	(-)	NNNNWTATGCAAATNTNNN	CCAGGTTTTACATTTTGTT
M00162	V$OCT1_06	0.885547	1670	(+)	CWNAWTKWSATRYN	AGGTTTTACATTTT
M00195	V$OCT1_Q6	0.821234	1670	(-)	NNNNATGCAAATNAN	AGGTTTTACATTTTG
M00162	V$OCT1_06	0.860547	1671	(-)	CWNAWTKWSATRYN	GGTTTTACATTTTG
M00138	V$OCT1_04	0.790255	1679	(+)	NNNNNNNWATGCAAATNNNWNNW	ATTTTGTTGTGTTAATTTCTTCT
M00135	V$OCT1_01	0.770698	1681	(+)	NNNNWTATGCAAATNTNNN	TTTGTTGTGTTAATTTCTT
M00248	V$OCT1_07	0.857325	1684	(+)	TNTATGNTAATT	GTTGTGTTAATT
M00137	V$OCT1_03	0.847886	1700	(+)	NNNRTAATNANNN	CTTGTAAAGAACT
M00135	V$OCT1_01	0.781572	1713	(+)	NNNNWTATGCAAATNTNNN	CATCGTATTCAAATCCATG
M00136	V$OCT1_02	0.782891	1713	(+)	NNGAATATKCANNNN	CATCGTATTCAAATC
M00162	V$OCT1_06	0.814062	1716	(-)	CWNAWTKWSATRYN	CGTATTCAAATCCA
M00162	V$OCT1_06	0.800391	1725	(+)	CWNAWTKWSATRYN	ATCCATGTGTTTCT
M00136	V$OCT1_02	0.775041	1738	(-)	NNGAATATKCANNNN	TCAATGAATCTACTT
M00137	V$OCT1_03	0.8629	1760	(-)	NNNRTAATNANNN	TCTTCATTGCCCT
M00248	V$OCT1_07	0.798601	1770	(+)	TNTATGNTAATT	CCTTTTCTAATT
M00137	V$OCT1_03	0.854208	1773	(+)	NNNRTAATNANNN	TTTCTAATTATCT
M00137	V$OCT1_03	0.921375	1774	(-)	NNNRTAATNANNN	TTCTAATTATCTG

M00138	V$OCT1_04	0.816186	1775	(-)	NNNNNNNWATGCAAATNNNWNNW	TCTAATTATCTGAAGATAATTTA
M00162	V$OCT1_06	0.9375	1789	(+)	CWNAWTKWSATRYN	GATAATTTAATACA
M00138	V$OCT1_04	0.856964	1790	(+)	NNNNNNNWATGCAAATNNNWNNW	ATAATTTAATACATAATTAATGA
M00162	V$OCT1_06	0.808594	1790	(-)	CWNAWTKWSATRYN	ATAATTTAATACAT
M00137	V$OCT1_03	0.868036	1799	(+)	NNNRTAATNANNN	TACATAATTAATG
M00137	V$OCT1_03	0.847886	1800	(-)	NNNRTAATNANNN	ACATAATTAATGA
M00137	V$OCT1_03	0.878704	1803	(+)	NNNRTAATNANNN	TAATTAATGAGGA
M00137	V$OCT1_03	0.896484	1819	(-)	NNNRTAATNANNN	GTGTGATTATAAG
M00162	V$OCT1_06	0.847266	1847	(+)	CWNAWTKWSATRYN	ATTAATTAGCTTCT
M00137	V$OCT1_03	0.849862	1878	(+)	NNNRTAATNANNN	AAAGTAAACATGA
M00162	V$OCT1_06	0.816016	1883	(+)	CWNAWTKWSATRYN	AAACATGAAGTTCT
M00162	V$OCT1_06	0.862891	1884	(-)	CWNAWTKWSATRYN	AACATGAAGTTCTT
M00137	V$OCT1_03	0.891347	1897	(-)	NNNRTAATNANNN	TCATCTTTACCTG
M00162	V$OCT1_06	0.862891	1898	(+)	CWNAWTKWSATRYN	CATCTTTACCTGCC
M00195	V$OCT1_Q6	0.811135	1923	(+)	NNNNATGCAAATNAN	GCCCTTGCAAAGAAT
M00135	V$OCT1_01	0.736169	1930	(+)	NNNNWTATGCAAATNTNNN	CAAAGAATGTAAGTATAAT
M00162	V$OCT1_06	0.876172	1933	(-)	CWNAWTKWSATRYN	AGAATGTAAGTATA
M00137	V$OCT1_03	0.914658	1941	(+)	NNNRTAATNANNN	AGTATAATAAGAT
M00162	V$OCT1_06	0.825	1943	(+)	CWNAWTKWSATRYN	TATAATAAGATACA
M00135	V$OCT1_01	0.730065	1953	(-)	NNNNWTATGCAAATNTNNN	TACAGATGAGAATATATTA
M00138	V$OCT1_04	0.888122	1963	(+)	NNNNNNNWATGCAAATNNNWNNW	AATATATTATGTAAATATAAGAA
M00135	V$OCT1_01	0.872758	1965	(+)	NNNNWTATGCAAATNTNNN	TATATTATGTAAATATAAG
M00162	V$OCT1_06	0.839453	1968	(+)	CWNAWTKWSATRYN	ATTATGTAAATATA
M00162	V$OCT1_06	0.825	1968	(-)	CWNAWTKWSATRYN	ATTATGTAAATATA
M00248	V$OCT1_07	0.847149	1968	(+)	TNTATGNTAATT	ATTATGTAAATA
M00138	V$OCT1_04	0.780427	1969	(+)	NNNNNNNWATGCAAATNNNWNNW	TTATGTAAATATAAGAATATTAA
M00137	V$OCT1_03	0.851442	1970	(+)	NNNRTAATNANNN	TATGTAAATATAA
M00138	V$OCT1_04	0.857382	1972	(-)	NNNNNNNWATGCAAATNNNWNNW	TGTAAATATAAGAATATTAAATT
M00135	V$OCT1_01	0.750286	1974	(-)	NNNNWTATGCAAATNTNNN	TAAATATAAGAATATTAAA
M00138	V$OCT1_04	0.795483	1977	(+)	NNNNNNNWATGCAAATNNNWNNW	ATATAAGAATATTAAATTGTTTA
M00136	V$OCT1_02	0.86248	1978	(-)	NNGAATATKCANNNN	TATAAGAATATTAAA
M00136	V$OCT1_02	0.838657	1981	(+)	NNGAATATKCANNNN	AAGAATATTAAATTG
M00162	V$OCT1_06	0.829297	1983	(+)	CWNAWTKWSATRYN	GAATATTAAATTGT

M00162	V$OCT1_06	0.924609	1984	(-)	CWNAWTKWSATRYN	AATATTAAATTGTT
M00137	V$OCT1_03	0.853418	1991	(-)	NNNRTAATNANNN	AATTGTTTATAAG
M00137	V$OCT1_03	0.852232	1996	(+)	NNNRTAATNANNN	TTTATAAGGATAA
M00137	V$OCT1_03	0.84354	2002	(+)	NNNRTAATNANNN	AGGATAATGTCAG
M00137	V$OCT1_03	0.866456	2059	(+)	NNNRTAATNANNN	CCTATAATCAAAG
M00162	V$OCT1_06	0.805859	2061	(-)	CWNAWTKWSATRYN	TATAATCAAAGACG
M00135	V$OCT1_01	0.795307	2085	(+)	NNNNWTATGCAAATNTNNN	TGCCTTATGTAAGTTATGA
M00162	V$OCT1_06	0.847656	2088	(-)	CWNAWTKWSATRYN	CTTATGTAAGTTAT
M00248	V$OCT1_07	0.821709	2088	(+)	TNTATGNTAATT	CTTATGTAAGTT
M00138	V$OCT1_04	0.799038	2154	(+)	NNNNNNNWATGCAAATNNNWNNW	GAGTTTTAGTTCAAAGTTAAAAA
M00162	V$OCT1_06	0.805859	2166	(-)	CWNAWTKWSATRYN	AAAGTTAAAAAACG
M00162	V$OCT1_06	0.842187	2174	(+)	CWNAWTKWSATRYN	AAAACGTACATTTT
M00136	V$OCT1_02	0.811316	2176	(-)	NNGAATATKCANNNN	AACGTACATTTTCTT
M00137	V$OCT1_03	0.872778	2190	(+)	NNNRTAATNANNN	TCCGTAAACATTT
M00138	V$OCT1_04	0.785236	2192	(-)	NNNNNNNWATGCAAATNNNWNNW	CGTAAACATTTGAATTTATTGGG
M00135	V$OCT1_01	0.767264	2194	(-)	NNNNWTATGCAAATNTNNN	TAAACATTTGAATTTATTG
M00162	V$OCT1_06	0.821094	2195	(+)	CWNAWTKWSATRYN	AAACATTTGAATTT
M00162	V$OCT1_06	0.891016	2196	(+)	CWNAWTKWSATRYN	AACATTTGAATTTA
M00162	V$OCT1_06	0.812109	2196	(-)	CWNAWTKWSATRYN	AACATTTGAATTTA
M00195	V$OCT1_Q6	0.792849	2196	(-)	NNNNATGCAAATNAN	AACATTTGAATTTAT
M00138	V$OCT1_04	0.787327	2204	(+)	NNNNNNNWATGCAAATNNNWNNW	AATTTATTGGGGAAATATTTTCA
M00162	V$OCT1_06	0.834375	2217	(+)	CWNAWTKWSATRYN	AATATTTTCACTCC
M00162	V$OCT1_06	0.808203	2244	(-)	CWNAWTKWSATRYN	TAAAAGTAAGAAAC
M00137	V$OCT1_03	0.842355	2263	(+)	NNNRTAATNANNN	ACCATAACAAGGA
M00162	V$OCT1_06	0.839453	2276	(+)	CWNAWTKWSATRYN	CTTCTTTTAAAGTT
M00138	V$OCT1_04	0.785864	2301	(+)	NNNNNNNWATGCAAATNNNWNNW	CAGAGAAAATGTAGATTCCCAAA
M00136	V$OCT1_02	0.783162	2303	(+)	NNGAATATKCANNNN	GAGAAAATGTAGATT
M00195	V$OCT1_Q6	0.833515	2305	(+)	NNNNATGCAAATNAN	GAAAATGTAGATTCC
M00162	V$OCT1_06	0.834375	2306	(+)	CWNAWTKWSATRYN	AAAATGTAGATTCC
M00162	V$OCT1_06	0.834375	2306	(-)	CWNAWTKWSATRYN	AAAATGTAGATTCC
M00248	V$OCT1_07	0.793089	2306	(+)	TNTATGNTAATT	AAAATGTAGATT
M00136	V$OCT1_02	0.780455	2331	(+)	NNGAATATKCANNNN	AAGCATATGTCAGAA
M00162	V$OCT1_06	0.90625	2334	(-)	CWNAWTKWSATRYN	CATATGTCAGAATT

M00162	V$OCT1_06	0.825391	2341	(+)	CWNAWTKWSATRYN	CAGAATTTCATCAT
M00162	V$OCT1_06	0.85	2342	(-)	CWNAWTKWSATRYN	AGAATTTCATCATT
M00162	V$OCT1_06	0.821484	2352	(+)	CWNAWTKWSATRYN	CATTCTTGACTGCA
M00161	V$OCT1_05	0.865516	2383	(+)	MKNATTTGCATAYY	ATCCTTTGCAAATG
M00195	V$OCT1_Q6	0.798308	2384	(+)	NNNNATGCAAATNAN	TCCTTTGCAAATGAT
M00136	V$OCT1_02	0.825393	2399	(-)	NNGAATATKCANNNN	GTGTTGAATAGTGCT
M00162	V$OCT1_06	0.829297	2459	(-)	CWNAWTKWSATRYN	CACCTGCAATGCAG
M00162	V$OCT1_06	0.847656	2501	(-)	CWNAWTKWSATRYN	AAGATCCCATGGAG
M00223	V$STAT_01	0.853542	75	(-)	TTCCCRKAA	TTTTAGGAA
M00223	V$STAT_01	0.816738	156	(-)	TTCCCRKAA	TTCCAAAAA
M00223	V$STAT_01	0.80968	261	(+)	TTCCCRKAA	TTCTTAAAA
M00224	V$STAT1_01	0.720896	331	(-)	NNNSANTTCCGGGAANTGNSN	TCTTATTTTAAGGAAGAGCTA
M00224	V$STAT1_01	0.751358	642	(+)	NNNSANTTCCGGGAANTGNSN	GTAAAATTGAGGTAAGTGGCT
M00223	V$STAT_01	0.849761	775	(+)	TTCCCRKAA	ATCCTGTAA
M00223	V$STAT_01	0.817746	781	(-)	TTCCCRKAA	TAAGAGGAA
M00223	V$STAT_01	0.844719	1023	(-)	TTCCCRKAA	TTAGAATAA
M00223	V$STAT_01	0.795815	1147	(+)	TTCCCRKAA	ATCTTAGAA
M00223	V$STAT_01	0.844467	1334	(+)	TTCCCRKAA	TTCCCCTTA
M00223	V$STAT_01	0.792538	1472	(+)	TTCCCRKAA	TTAGTATAA
M00223	V$STAT_01	0.922359	1698	(+)	TTCCCRKAA	TTCTTGTAA
M00223	V$STAT_01	0.833627	1698	(-)	TTCCCRKAA	TTCTTGTAA
M00223	V$STAT_01	0.80968	1780	(+)	TTCCCRKAA	TTATCTGAA
M00223	V$STAT_01	0.826569	1780	(-)	TTCCCRKAA	TTATCTGAA
M00223	V$STAT_01	0.812957	2227	(-)	TTCCCRKAA	CTCCAAGAA
M00223	V$STAT_01	0.80968	2240	(+)	TTCCCRKAA	TTCTTAAAA
M00223	V$STAT_01	0.819763	2277	(+)	TTCCCRKAA	TTCTTTTAA
M00224	V$STAT1_01	0.756792	2549	(+)	NNNSANTTCCGGGAANTGNSN	GGAGAATTCCTTGAACAGAAG
M00223	V$STAT_01	0.870683	2555	(+)	TTCCCRKAA	TTCCTTGAA
M00059	V$YY1_01	0.806029	67	(+)	NNNNNCCATNTWNNNWN	TGTCTTCCTTTTAGGAA
M00059	V$YY1_01	0.829292	93	(+)	NNNNNCCATNTWNNNWN	TATTTTCATTTATCTTT
M00059	V$YY1_01	0.782438	145	(+)	NNNNNCCATNTWNNNWN	TAATAGCATTGTTCCAA
M00059	V$YY1_01	0.794561	532	(-)	NNNNNCCATNTWNNNWN	CTATTTCTATGGTTCTC
M00059	V$YY1_01	0.85616	542	(+)	NNNNNCCATNTWNNNWN	GGTTCTCATTTATCAAC
M00059	V$YY1_01	0.77293	563	(+)	NNNNNCCATNTWNNNWN	TATAAGCATTTCTTTAA

		6				
M00059	V$YY1_01	0.812254	593	(+)	NNNNNCCATNTWNNNWN	TTTGTCCATGTTCCAGT
M00059	V$YY1_01	0.772608	613	(-)	NNNNNCCATNTWNNNWN	TTTCATCCATGGAAAAT
M00059	V$YY1_01	0.782765	662	(-)	NNNNNCCATNTWNNNWN	TATATATAATGAACTTA
M00059	V$YY1_01	0.790957	731	(+)	NNNNNCCATNTWNNNWN	CATTGTCATTATGAAAT
M00059	V$YY1_01	0.773263	733	(-)	NNNNNCCATNTWNNNWN	TTGTCATTATGAAATGC
M00059	V$YY1_01	0.794233	738	(-)	NNNNNCCATNTWNNNWN	ATTATGAAATGCTTATA
M00059	V$YY1_01	0.792595	792	(-)	NNNNNCCATNTWNNNWN	AAGAATATCTGGGATTC
M00059	V$YY1_01	0.793578	869	(+)	NNNNNCCATNTWNNNWN	AATTCCCATGCATACAC
M00059	V$YY1_01	0.793906	1353	(+)	NNNNNCCATNTWNNNWN	AGGCTCCAGGTTTTGGC
M00059	V$YY1_01	0.778834	1373	(-)	NNNNNCCATNTWNNNWN	CCTGAAATCTGATTTTA
M00059	V$YY1_01	0.804063	1414	(-)	NNNNNCCATNTWNNNWN	TTTCTGATATGATTCTC
M00059	V$YY1_01	0.836501	1456	(-)	NNNNNCCATNTWNNNWN	AAAAATATATGGCATTT
M00059	V$YY1_01	0.787025	1479	(+)	NNNNNCCATNTWNNNWN	AAACATCATTATTTTTC
M00059	V$YY1_01	0.788336	1616	(-)	NNNNNCCATNTWNNNWN	ATGCCCAAATGAGCCTC
M00059	V$YY1_01	0.80865	1733	(-)	NNNNNCCATNTWNNNWN	GTTTCTCAATGAATCTA
M00069	V$YY1_02	0.758122	1755	(-)	NNNCGGCCATCTTGNCTSNW	ATCAGTCTTCATTGCCCTTT
M00059	V$YY1_01	0.822084	1764	(+)	NNNNNCCATNTWNNNWN	CATTGCCCTTTTCTAAT
M00059	V$YY1_01	0.783748	1776	(-)	NNNNNCCATNTWNNNWN	CTAATTATCTGAAGATA
M00059	V$YY1_01	0.778506	1834	(-)	NNNNNCCATNTWNNNWN	GAGTAAAACTGTTATTA
M00059	V$YY1_01	0.869594	1879	(-)	NNNNNCCATNTWNNNWN	AAGTAAACATGAAGTTC
M00059	V$YY1_01	0.782438	1892	(+)	NNNNNCCATNTWNNNWN	GTTCTTCATCTTTACCT
M00069	V$YY1_02	0.781507	2062	(-)	NNNCGGCCATCTTGNCTSNW	ATAATCAAAGACGGCCTTGG
M00059	V$YY1_01	0.816514	2098	(-)	NNNNNCCATNTWNNNWN	TTATGAAGATGGACAAA
M00059	V$YY1_01	0.821101	2250	(-)	NNNNNCCATNTWNNNWN	TAAGAAACATGGAACCA
M00059	V$YY1_01	0.836173	2301	(-)	NNNNNCCATNTWNNNWN	CAGAGAAAATGTAGATT
M00059	V$YY1_01	0.822412	2386	(-)	NNNNNCCATNTWNNNWN	CTTTGCAAATGATGTGT
M00059	V$YY1_01	0.772936	2480	(+)	NNNNNCCATNTWNNNWN	CAGCTCCATCCCTGGGT
M00059	V$YY1_01	0.783093	2513	(-)	NNNNNCCATNTWNNNWN	AGAAGGAAATGGCAACC
CSN2 Bos Tarus						
M00216	V$TATA_C	0.813045	2	(+)	NCTATAAAAR	CCCTTTAAAA
M00216	V$TATA_C	0.945075	3	(+)	NCTATAAAAR	CCTTTAAAAT
M00252	V$TATA_01	0.846486	4	(+)	STATAAAWRNNNNNN	CTTTAAAATGCTCCC
M00216	V$TATA_C	0.81462	110	(+)	NCTATAAAAR	ACCACAAAAT

		9				
M00216	V$TATA_C	0.809876	126	(+)	NCTATAAAAR	GCCATTAAAT
M00216	V$TATA_C	0.759968	127	(+)	NCTATAAAAR	CCATTAAATA
M00252	V$TATA_01	0.771632	127	(+)	STATAAAWRNNNNNN	CCATTAAATACTATA
M00216	V$TATA_C	0.795088	136	(+)	NCTATAAAAR	ACTATATATA
M00252	V$TATA_01	0.902309	137	(+)	STATAAAWRNNNNNN	CTATATATAAACAAC
M00216	V$TATA_C	0.809348	138	(+)	NCTATAAAAR	TATATATAAA
M00252	V$TATA_01	0.895712	139	(+)	STATAAAWRNNNNNN	ATATATAAACAACCA
M00216	V$TATA_C	0.751782	140	(+)	NCTATAAAAR	TATATAAACA
M00252	V$TATA_01	0.831007	141	(+)	STATAAAWRNNNNNN	ATATAAACAACCACA
M00216	V$TATA_C	0.814629	150	(+)	NCTATAAAAR	ACCACAAAAT
M00252	V$TATA_01	0.777721	151	(+)	STATAAAWRNNNNNN	CCACAAAATCAGATC
M00216	V$TATA_C	0.790335	272	(+)	NCTATAAAAR	AGGTTAAAAT
M00216	V$TATA_C	0.883549	298	(+)	NCTATAAAAR	TGCTTAAAAT
M00216	V$TATA_C	0.855558	310	(+)	NCTATAAAAR	ATCTTAAAAA
M00216	V$TATA_C	0.823607	311	(+)	NCTATAAAAR	TCTTAAAAAT
M00252	V$TATA_01	0.790916	313	(+)	STATAAAWRNNNNNN	TTAAAAATAAGGATG
M00216	V$TATA_C	0.789015	326	(+)	NCTATAAAAR	TGTTTAAGAT
M00216	V$TATA_C	0.759176	361	(+)	NCTATAAAAR	TTGTTAAAAT
M00216	V$TATA_C	0.859519	378	(+)	NCTATAAAAR	ACTATAAAGA
M00252	V$TATA_01	0.832022	379	(+)	STATAAAWRNNNNNN	CTATAAAGATTTGTA
M00216	V$TATA_C	0.832057	437	(+)	NCTATAAAAR	TGTATTAAAA
M00216	V$TATA_C	0.809084	438	(+)	NCTATAAAAR	GTATTAAAAG
M00252	V$TATA_01	0.86171	438	(+)	STATAAAWRNNNNNN	GTATTAAAAGATCTT
M00216	V$TATA_C	0.838659	494	(+)	NCTATAAAAR	GCCATAAAGG
M00252	V$TATA_01	0.840396	495	(+)	STATAAAWRNNNNNN	CCATAAAGGCAAGCA
M00216	V$TATA_C	0.785582	561	(+)	NCTATAAAAR	CCAACAAAAA
M00252	V$TATA_01	0.85968	571	(+)	STATAAAWRNNNNNN	GTTTATAGAGCTAGC
M00252	V$TATA_01	0.776706	584	(+)	STATAAAWRNNNNNN	GCATATTTAGTCAAG
M00252	V$TATA_01	0.821365	599	(+)	STATAAAWRNNNNNN	AGATAAAGAGGGTTG
M00252	V$TATA_01	0.821365	639	(+)	STATAAAWRNNNNNN	GTATTTATAAAAGAA
M00216	V$TATA_C	0.783734	640	(+)	NCTATAAAAR	TATTTATAAA
M00252	V$TATA_01	0.848769	641	(+)	STATAAAWRNNNNNN	ATTTATAAAAGAAGA

M00216	V$TATA_C	0.927119	642	(+)	NCTATAAAAR	TTTATAAAAG
M00252	V$TATA_01	0.904339	643	(+)	STATAAAWRNNNNNN	TTATAAAAGAAGAGT
M00252	V$TATA_01	0.777721	656	(+)	STATAAAWRNNNNNN	GTATATTTATTAAAA
M00252	V$TATA_01	0.777721	658	(+)	STATAAAWRNNNNNN	ATATTTATTAAAATT
M00216	V$TATA_C	0.793768	661	(+)	NCTATAAAAR	TTTATTAAAA
M00216	V$TATA_C	0.792184	662	(+)	NCTATAAAAR	TTATTAAAAT
M00252	V$TATA_01	0.798782	662	(+)	STATAAAWRNNNNNN	TTATTAAAATTGCTC
M00216	V$TATA_C	0.761025	716	(+)	NCTATAAAAR	ATTTCAAAAA
M00216	V$TATA_C	0.744653	724	(+)	NCTATAAAAR	AATATTAAAA
M00216	V$TATA_C	0.785054	725	(+)	NCTATAAAAR	ATATTAAAAT
M00252	V$TATA_01	0.846739	725	(+)	STATAAAWRNNNNNN	ATATTAAAATAGATA
M00252	V$TATA_01	0.780259	756	(+)	STATAAAWRNNNNNN	AAATTAAAGAGAAAG
M00216	V$TATA_C	0.801954	779	(+)	NCTATAAAAR	TTTGTAAAAA
M00216	V$TATA_C	0.832057	781	(+)	NCTATAAAAR	TGTAAAAAAA
M00252	V$TATA_01	0.7729	782	(+)	STATAAAWRNNNNNN	GTAAAAAAAAATTCT
M00216	V$TATA_C	0.778981	828	(+)	NCTATAAAAR	ACAATGAAAA
M00216	V$TATA_C	0.889094	889	(+)	NCTATAAAAR	GGCTTAAAAA
M00216	V$TATA_C	0.811724	890	(+)	NCTATAAAAR	GCTTAAAAAT
M00252	V$TATA_01	0.794468	890	(+)	STATAAAWRNNNNNN	GCTTAAAAATTGGCA
M00216	V$TATA_C	0.850277	901	(+)	NCTATAAAAR	GGCATATAAA
M00252	V$TATA_01	0.8305	902	(+)	STATAAAWRNNNNNN	GCATATAAAATGCTA
M00216	V$TATA_C	0.855823	903	(+)	NCTATAAAAR	CATATAAAAT
M00252	V$TATA_01	0.883532	904	(+)	STATAAAWRNNNNNN	ATATAAAATGCTAAT
M00216	V$TATA_C	0.891207	917	(+)	NCTATAAAAR	ATTATAAAAT
M00252	V$TATA_01	0.822634	918	(+)	STATAAAWRNNNNNN	TTATAAAATAAACAA
M00216	V$TATA_C	0.778981	997	(+)	NCTATAAAAR	ACAATGAAAA
M00252	V$TATA_01	0.784319	1033	(+)	STATAAAWRNNNNNN	GGATAAAGTAAAACA
M00216	V$TATA_C	0.746501	1074	(+)	NCTATAAAAR	AATATAGAAA
M00216	V$TATA_C	0.751782	1076	(+)	NCTATAAAAR	TATAGAAAAA
M00216	V$TATA_C	0.749142	1167	(+)	NCTATAAAAR	AACTTATAAA
M00216	V$TATA_C	0.915236	1169	(+)	NCTATAAAAR	CTTATAAAAA
M00252	V$TATA_01	0.900025	1170	(+)	STATAAAWRNNNNNN	TTATAAAAATGCAAC
M00252	V$TATA_01	0.771124	1172	(+)	STATAAAWRNNNNNN	ATAAAAATGCAACAA

M00216	V$TATA_C	0.747821	1180	(+)	NCTATAAAAR	GCAACAAAAC
M00216	V$TATA_C	0.750198	1192	(+)	NCTATAAAAR	ACCATTTAAT
M00252	V$TATA_01	0.795991	1306	(+)	STATAAAWRNNNNNN	AGATAAGTACACAGT
M00216	V$TATA_C	0.857407	1352	(+)	NCTATAAAAR	TCTCTAAAAT
M00216	V$TATA_C	0.759176	1437	(+)	NCTATAAAAR	CCAAGAAAAC
M00216	V$TATA_C	0.792712	1503	(+)	NCTATAAAAR	GAAATAAAAG
M00252	V$TATA_01	0.780513	1504	(+)	STATAAAWRNNNNNN	AAATAAAAGATATGA
M00216	V$TATA_C	0.766306	1531	(+)	NCTATAAAAR	TTCATGAAAA
M00216	V$TATA_C	0.743068	1532	(+)	NCTATAAAAR	TCATGAAAAT
M00216	V$TATA_C	0.753103	1561	(+)	NCTATAAAAR	AAGATAAAAA
M00252	V$TATA_01	0.838366	1562	(+)	STATAAAWRNNNNNN	AGATAAAAATAAAGT
M00216	V$TATA_C	0.881701	1618	(+)	NCTATAAAAR	TCAATAAAAC
M00252	V$TATA_01	0.811723	1619	(+)	STATAAAWRNNNNNN	CAATAAAACTGAGTC
M00216	V$TATA_C	0.816477	1649	(+)	NCTATAAAAR	ACTTTAATAT
M00216	V$TATA_C	0.896224	1699	(+)	NCTATAAAAR	GCAATAAAAA
M00252	V$TATA_01	0.813499	1700	(+)	STATAAAWRNNNNNN	CAATAAAAATTAATA
M00252	V$TATA_01	0.782796	1710	(+)	STATAAAWRNNNNNN	TAATAAATAAATATT
M00216	V$TATA_C	0.768154	1723	(+)	NCTATAAAAR	TTTTTAATAA
M00216	V$TATA_C	0.812781	1786	(+)	NCTATAAAAR	TCCAAAAAAC
M00252	V$TATA_01	0.775184	1811	(+)	STATAAAWRNNNNNN	GAATAAGATGAAAAG
M00216	V$TATA_C	0.745445	1849	(+)	NCTATAAAAR	AGCTGAAAAC
M00216	V$TATA_C	0.77766	1935	(+)	NCTATAAAAR	ATGATAAAAT
M00216	V$TATA_C	0.744653	1946	(+)	NCTATAAAAR	TCTATTAATA
M00252	V$TATA_01	0.790155	1947	(+)	STATAAAWRNNNNNN	CTATTAATATTTTTC
M00216	V$TATA_C	0.784526	1958	(+)	NCTATAAAAR	TTTCTAAAAT
M00216	V$TATA_C	0.85239	2089	(+)	NCTATAAAAR	TCTTTTAAAG
M00216	V$TATA_C	0.778981	2090	(+)	NCTATAAAAR	CTTTTAAAGG
M00252	V$TATA_01	0.845724	2090	(+)	STATAAAWRNNNNNN	CTTTTAAAGGGAAGT
M00252	V$TATA_01	0.793961	2091	(+)	STATAAAWRNNNNNN	TTTTAAAGGGAAGTA
M00252	V$TATA_01	0.781528	2111	(+)	STATAAAWRNNNNNN	ACTTAAATAAGAAAA
M00216	V$TATA_C	0.740164	2112	(+)	NCTATAAAAR	CTTAAATAAG
M00252	V$TATA_01	0.804364	2255	(+)	STATAAAWRNNNNNN	AAATAAATAAGACTA
M00252	V$TATA_01	0.783811	2257	(+)	STATAAAWRNNNNNN	ATAAATAAGACTAGT

M00216	V$TATA_C	0.814629	2286	(+)	NCTATAAAAR	TGTAGAAAAT
M00252	V$TATA_01	0.813499	2287	(+)	STATAAAWRNNNNNN	GTAGAAAATCGCATT
M00216	V$TATA_C	0.832057	2316	(+)	NCTATAAAAR	TGTATAAATA
M00252	V$TATA_01	0.887084	2317	(+)	STATAAAWRNNNNNN	GTATAAATAATGCAC
M00216	V$TATA_C	0.744125	2353	(+)	NCTATAAAAR	GCTAAGAAAG
M00216	V$TATA_C	0.745973	2459	(+)	NCTATAAAAR	CCTAAAGAAA
M00223	V$STAT_01	0.866398	15	(+)	TTCCCRKAA	TCCCCAGAA
M00223	V$STAT_01	0.80489	15	(-)	TTCCCRKAA	TCCCCAGAA
M00224	V$STAT1_01	0.748065	67	(+)	NNNSANTTCCGGGAANTGNSN	GCAGATTTCTAG-GAATTCAAA
M00224	V$STAT1_01	0.780339	67	(-)	NNNSANTTCCGGGAANTGNSN	GCAGATTTCTAG-GAATTCAAA
M00225	V$STAT3_01	0.765098	67	(+)	NGNNATTTCCSGGAARTGNNN	GCAGATTTCTAG-GAATTCAAA
M00225	V$STAT3_01	0.787256	67	(-)	NGNNATTTCCSGGAARTGNNN	GCAGATTTCTAG-GAATTCAAA
M00223	V$STAT_01	0.850769	73	(+)	TTCCCRKAA	TTCTAGGAA
M00223	V$STAT_01	0.956138	73	(-)	TTCCCRKAA	TTCTAGGAA
M00223	V$STAT_01	0.802622	329	(-)	TTCCCRKAA	TTAAGATAA
M00223	V$STAT_01	0.805899	341	(-)	TTCCCRKAA	TTACAGTAT
M00223	V$STAT_01	0.85934	523	(-)	TTCCCRKAA	CTATGGGAA
M00223	V$STAT_01	0.822536	672	(+)	TTCCCRKAA	TGCTCAGAA
M00223	V$STAT_01	0.840686	672	(-)	TTCCCRKAA	TGCTCAGAA
M00225	V$STAT3_01	0.727734	806	(+)	NGNNATTTCCSGGAARTGNNN	AGGGAGTACCAG-GAAACAAAA
M00225	V$STAT3_01	0.735409	806	(-)	NGNNATTTCCSGGAARTGNNN	AGGGAGTACCAG-GAAACAAAA
M00223	V$STAT_01	0.858583	812	(-)	TTCCCRKAA	TACCAGGAA
M00223	V$STAT_01	0.791782	846	(-)	TTCCCRKAA	TGACAGAAA
M00223	V$STAT_01	0.801361	1139	(-)	TTCCCRKAA	TGATGGCAA
M00223	V$STAT_01	0.826821	1160	(-)	TTCCCRKAA	TTTCAAGAA
M00223	V$STAT_01	0.792538	1170	(-)	TTCCCRKAA	TTATAAAAA
M00223	V$STAT_01	0.812957	1435	(-)	TTCCCRKAA	GTCCAAGAA
M00224	V$STAT1_01	0.77507	1491	(+)	NNNSANTTCCGGGAANTGNSN	GAAGTCTTACTGGAAA-TAAAA
M00225	V$STAT3_01	0.77147	1491	(-)	NGNNATTTCCSGGAARTGNNN	GAAGTCTTACTGGAAA-TAAAA
M00223	V$STAT_01	0.929418	1497	(+)	TTCCCRKAA	TTACTGGAA
M00223	V$STAT_01	0.887572	1497	(-)	TTCCCRKAA	TTACTGGAA
M00224	V$STAT1_01	0.733575	1669	(-)	NNNSANTTCCGGGAANTGNSN	GCTCTAATCCCAGAATCTAAG
M00223	V$STAT_01	0.866398	1675	(+)	TTCCCRKAA	ATCCCAGAA

M00223	V$STAT_01	0.803126	1675	(-)	TTCCCRKAA	ATCCCAGAA
M00223	V$STAT_01	0.853542	1752	(+)	TTCCCRKAA	TTTCTGTAA
M00223	V$STAT_01	0.816738	1785	(-)	TTCCCRKAA	TTCCAAAAA
M00223	V$STAT_01	0.809932	1847	(-)	TTCCCRKAA	TTAGCTGAA
M00225	V$STAT3_01	0.714699	1878	(-)	NGNNATTTCCSGGAARTGNNN	AAACATTAACTT-GAAATGTAA
M00223	V$STAT_01	0.790522	1909	(-)	TTCCCRKAA	TAATAGTAA
M00223	V$STAT_01	0.819763	2088	(+)	TTCCCRKAA	TTCTTTTAA
M00224	V$STAT1_01	0.757616	2158	(-)	NNNSANTTCCGGGAANTGNSN	TTCCCATTCCCAG-GAATTGAG
M00224	V$STAT1_01	0.808003	2159	(+)	NNNSANTTCCGGGAANTGNSN	TCCCATTCCCAGGAATT-GAGA
M00224	V$STAT1_01	0.824469	2159	(-)	NNNSANTTCCGGGAANTGNSN	TCCCATTCCCAGGAATT-GAGA
M00225	V$STAT3_01	0.79609	2159	(+)	NGNNATTTCCSGGAARTGNNN	TCCCATTCCCAGGAATT-GAGA
M00225	V$STAT3_01	0.813034	2159	(-)	NGNNATTTCCSGGAARTGNNN	TCCCATTCCCAGGAATT-GAGA
M00223	V$STAT_01	0.868162	2164	(+)	TTCCCRKAA	TTCCCAGGA
M00223	V$STAT_01	0.803126	2164	(-)	TTCCCRKAA	TTCCCAGGA
M00223	V$STAT_01	0.858583	2165	(-)	TTCCCRKAA	TCCCAGGAA
M00223	V$STAT_01	0.850517	2352	(-)	TTCCCRKAA	TGCTAAGAA
M00127	V$GATA1_03	0.803283	35	(+)	RNSNNGATAANNGN	AGAAAAATAGGAAG
M00075	V$GATA1_01	0.835637	58	(-)	SNNGATNNNN	TCTAATCATG
M00076	V$GATA2_01	0.789355	58	(-)	NNNGATRNNN	TCTAATCATG
M00077	V$GATA3_01	0.862206	58	(-)	NNGATARNG	TCTAATCAT
M00075	V$GATA1_01	0.872655	67	(+)	SNNGATNNNN	GCAGATTTCT
M00076	V$GATA2_01	0.871448	67	(+)	NNNGATRNNN	GCAGATTTCT
M00075	V$GATA1_01	0.788253	83	(-)	SNNGATNNNN	TCAAATCCAC
M00126	V$GATA1_02	0.774062	133	(-)	NNNNNGATANKGNN	AATACTATATATAA
M00075	V$GATA1_01	0.84847	154	(-)	SNNGATNNNN	CAAAATCAGA
M00076	V$GATA2_01	0.839874	154	(-)	NNNGATRNNN	CAAAATCAGA
M00077	V$GATA3_01	0.838724	154	(-)	NNGATARNG	CAAAATCAG
M00075	V$GATA1_01	0.770484	159	(+)	SNNGATNNNN	TCAGATCATT
M00076	V$GATA2_01	0.823184	159	(+)	NNNGATRNNN	TCAGATCATT
M00203	V$GATA_C	0.880398	162	(-)	NGATAAGNMNN	GATCATTATCC
M00126	V$GATA1_02	0.843125	163	(-)	NNNNNGATANKGNN	ATCATTATCCATTC
M00127	V$GATA1_03	0.848359	163	(-)	RNSNNGATAANNGN	ATCATTATCCATTC
M00076	V$GATA2_01	0.792963	165	(-)	NNNGATRNNN	CATTATCCAT

M00077	V$GATA3_01	0.863979	165	(-)	NNGATARNG	CATTATCCA
M00127	V$GATA1_03	0.784664	211	(+)	RNSNNGATAANNGN	AAAAAGGTAAGAAT
M00075	V$GATA1_01	0.845508	219	(-)	SNNGATNNNN	AAGAATCTCA
M00076	V$GATA2_01	0.849346	219	(-)	NNNGATRNNN	AAGAATCTCA
M00076	V$GATA2_01	0.832657	226	(+)	NNNGATRNNN	TCAGATATAA
M00077	V$GATA3_01	0.840939	227	(+)	NNGATARNG	CAGATATAA
M00203	V$GATA_C	0.833799	228	(+)	NGATAAGNMNN	AGATATAATTT
M00126	V$GATA1_02	0.8075	239	(-)	NNNNNGATANKGNN	CATTGTATCTGCTA
M00128	V$GATA1_04	0.828431	239	(-)	NNCWGATARNNNN	CATTGTATCTGCT
M00075	V$GATA1_01	0.842547	241	(-)	SNNGATNNNN	TTGTATCTGC
M00076	V$GATA2_01	0.858367	241	(-)	NNNGATRNNN	TTGTATCTGC
M00077	V$GATA3_01	0.821887	241	(-)	NNGATARNG	TTGTATCTG
M00127	V$GATA1_03	0.807692	250	(-)	RNSNNGATAANNGN	CTACTCATCTTTAT
M00075	V$GATA1_01	0.805035	252	(-)	SNNGATNNNN	ACTCATCTTT
M00076	V$GATA2_01	0.824989	252	(-)	NNNGATRNNN	ACTCATCTTT
M00077	V$GATA3_01	0.83385	252	(-)	NNGATARNG	ACTCATCTT
M00127	V$GATA1_03	0.837825	287	(+)	RNSNNGATAANNGN	AAGAACATAATTGC
M00076	V$GATA2_01	0.786198	289	(+)	NNNGATRNNN	GAACATAATT
M00076	V$GATA2_01	0.789806	306	(-)	NNNGATRNNN	ATAGATCTTA
M00126	V$GATA1_02	0.790625	313	(+)	NNNNNGATANKGNN	TTAAAAATAAGGAT
M00075	V$GATA1_01	0.806515	321	(+)	SNNGATNNNN	AAGGATGTTT
M00076	V$GATA2_01	0.847993	321	(+)	NNNGATRNNN	AAGGATGTTT
M00126	V$GATA1_02	0.863125	328	(+)	NNNNNGATANKGNN	TTTAAGATAAAGTT
M00128	V$GATA1_04	0.895833	329	(+)	NNCWGATARNNNN	TTAAGATAAAGTT
M00075	V$GATA1_01	0.772952	330	(+)	SNNGATNNNN	TAAGATAAAG
M00076	V$GATA2_01	0.852503	330	(+)	NNNGATRNNN	TAAGATAAAG
M00077	V$GATA3_01	0.913602	331	(+)	NNGATARNG	AAGATAAAG
M00203	V$GATA_C	0.913016	332	(+)	NGATAAGNMNN	AGATAAAGTTT
M00127	V$GATA1_03	0.787849	375	(-)	RNSNNGATAANNGN	GCAACTATAAAGAT
M00075	V$GATA1_01	0.799605	383	(+)	SNNGATNNNN	AAAGATTTGT
M00076	V$GATA2_01	0.803789	383	(+)	NNNGATRNNN	AAAGATTTGT
M00077	V$GATA3_01	0.821887	384	(+)	NNGATARNG	AAGATTTGT
M00127	V$GATA1_03	0.810142	400	(-)	RNSNNGATAANNGN	GTTGCTATTTTCTT

M00126	V$GATA1_02	0.782188	403	(-)	NNNNNGATANKGNN	GCTATTTTCTTTAC
M00127	V$GATA1_03	0.813817	403	(-)	RNSNNGATAANNGN	GCTATTTTCTTTAC
M00127	V$GATA1_03	0.804508	422	(+)	RNSNNGATAANNGN	GACTAGTTAACAGG
M00127	V$GATA1_03	0.823861	424	(-)	RNSNNGATAANNGN	CTAGTTAACAGGCT
M00076	V$GATA2_01	0.797474	444	(+)	NNNGATRNNN	AAAGATCTTT
M00076	V$GATA2_01	0.797474	444	(-)	NNNGATRNNN	AAAGATCTTT
M00076	V$GATA2_01	0.784393	482	(+)	NNNGATRNNN	AAACATACCT
M00076	V$GATA2_01	0.798376	521	(-)	NNNGATRNNN	TACTATGGGA
M00076	V$GATA2_01	0.801083	582	(+)	NNNGATRNNN	TAGCATATTT
M00126	V$GATA1_02	0.836875	595	(+)	NNNNNGATANKGNN	CAAGAGATAAAGAG
M00127	V$GATA1_03	0.849339	595	(+)	RNSNNGATAANNGN	CAAGAGATAAAGAG
M00128	V$GATA1_04	0.932598	596	(+)	NNCWGATARNNNNN	AAGAGATAAAGAG
M00075	V$GATA1_01	0.793189	597	(+)	SNNGATNNNN	AGAGATAAAG
M00076	V$GATA2_01	0.828146	597	(+)	NNNGATRNNN	AGAGATAAAG
M00077	V$GATA3_01	0.930882	598	(+)	NNGATARNG	GAGATAAAG
M00203	V$GATA_C	0.864244	599	(+)	NGATAAGNMNN	AGATAAAGAGG
M00075	V$GATA1_01	0.796644	615	(+)	SNNGATNNNN	TAGGATACAT
M00076	V$GATA2_01	0.880018	615	(+)	NNNGATRNNN	TAGGATACAT
M00076	V$GATA2_01	0.795219	678	(-)	NNNGATRNNN	GAACATCCAA
M00203	V$GATA_C	0.848089	692	(-)	NGATAAGNMNN	CAAGTTTATCA
M00126	V$GATA1_02	0.833125	693	(-)	NNNNNGATANKGNN	AAGTTTATCATTTA
M00128	V$GATA1_04	0.863664	693	(-)	NNCWGATARNNNNN	AAGTTTATCATTT
M00076	V$GATA2_01	0.792061	695	(-)	NNNGATRNNN	GTTTATCATT
M00077	V$GATA3_01	0.823217	695	(-)	NNGATARNG	GTTTATCAT
M00203	V$GATA_C	0.922336	699	(-)	NGATAAGNMNN	ATCATTTATCT
M00126	V$GATA1_02	0.79875	700	(-)	NNNNNGATANKGNN	TCATTTATCTTACA
M00128	V$GATA1_04	0.909314	700	(-)	NNCWGATARNNNNN	TCATTTATCTTAC
M00076	V$GATA2_01	0.839423	702	(-)	NNNGATRNNN	ATTTATCTTA
M00077	V$GATA3_01	0.846699	702	(-)	NNGATARNG	ATTTATCTT
M00126	V$GATA1_02	0.834063	731	(+)	NNNNNGATANKGNN	AAATAGATACATGA
M00127	V$GATA1_03	0.812347	731	(+)	RNSNNGATAANNGN	AAATAGATACATGA
M00203	V$GATA_C	0.855856	735	(+)	NGATAAGNMNN	AGATACATGAA
M00075	V$GATA1_01	0.794176	839	(+)	SNNGATNNNN	TGTGATCTGA

M00076	V$GATA2_01	0.803338	839	(+)	NNNGATRNNN	TGTGATCTGA
M00076	V$GATA2_01	0.812359	839	(-)	NNNGATRNNN	TGTGATCTGA
M00075	V$GATA1_01	0.880059	1024	(-)	SNNGATNNNN	AGACATCTTG
M00076	V$GATA2_01	0.894001	1024	(-)	NNNGATRNNN	AGACATCTTG
M00126	V$GATA1_02	0.778438	1029	(+)	NNNNNGATANKGNN	TCTTGGATAAAGTA
M00076	V$GATA2_01	0.785295	1031	(+)	NNNGATRNNN	TTGGATAAAG
M00077	V$GATA3_01	0.844041	1032	(+)	NNGATARNG	TGGATAAAG
M00203	V$GATA_C	0.89593	1033	(+)	NGATAAGNMNN	GGATAAAGTAA
M00075	V$GATA1_01	0.87463	1103	(-)	SNNGATNNNN	GCAAATCTGG
M00076	V$GATA2_01	0.849797	1103	(-)	NNNGATRNNN	GCAAATCTGG
M00075	V$GATA1_01	0.791708	1110	(+)	SNNGATNNNN	TGGGATTGAA
M00076	V$GATA2_01	0.819125	1110	(+)	NNNGATRNNN	TGGGATTGAA
M00077	V$GATA3_01	0.84537	1111	(+)	NNGATARNG	GGGATTGAA
M00075	V$GATA1_01	0.887957	1117	(+)	SNNGATNNNN	GAAGATGTGT
M00076	V$GATA2_01	0.886784	1117	(+)	NNNGATRNNN	GAAGATGTGT
M00075	V$GATA1_01	0.850938	1131	(+)	SNNGATNNNN	AGAGATGTTG
M00076	V$GATA2_01	0.854759	1131	(+)	NNNGATRNNN	AGAGATGTTG
M00077	V$GATA3_01	0.865308	1132	(+)	NNGATARNG	GAGATGTTG
M00127	V$GATA1_03	0.800343	1135	(+)	RNSNNGATAANNGN	ATGTTGATGGCAAG
M00075	V$GATA1_01	0.871668	1137	(+)	SNNGATNNNN	GTTGATGGCA
M00076	V$GATA2_01	0.813261	1137	(+)	NNNGATRNNN	GTTGATGGCA
M00127	V$GATA1_03	0.781725	1241	(+)	RNSNNGATAANNGN	ACAAGGAGAAGTAG
M00127	V$GATA1_03	0.801323	1271	(+)	RNSNNGATAANNGN	GGCAAGAGAATCTG
M00075	V$GATA1_01	0.822804	1276	(-)	SNNGATNNNN	GAGAATCTGA
M00076	V$GATA2_01	0.845737	1276	(-)	NNNGATRNNN	GAGAATCTGA
M00075	V$GATA1_01	0.846989	1293	(-)	SNNGATNNNN	GTAAATCACC
M00076	V$GATA2_01	0.797474	1293	(-)	NNNGATRNNN	GTAAATCACC
M00126	V$GATA1_02	0.81	1302	(+)	NNNNNGATANKGNN	CGAGAGATAAGTAC
M00127	V$GATA1_03	0.897844	1302	(+)	RNSNNGATAANNGN	CGAGAGATAAGTAC
M00128	V$GATA1_04	0.927083	1303	(+)	NNCWGATARNNNN	GAGAGATAAGTAC
M00075	V$GATA1_01	0.833662	1304	(+)	SNNGATNNNN	AGAGATAAGT
M00076	V$GATA2_01	0.861525	1304	(+)	NNNGATRNNN	AGAGATAAGT
M00077	V$GATA3_01	0.904741	1305	(+)	NNGATARNG	GAGATAAGT

M00203	V$GATA_C	0.98384<br>6	1306	(+)	NGATAAGNMNN	AGATAAGTACA
M00127	V$GATA1_03	0.79348<br>4	1326	(+)	RNSNNGATAANNGN	GTAGAGAAAATAAG
M00127	V$GATA1_03	0.80769<br>2	1329	(+)	RNSNNGATAANNGN	GAGAAAATAAGCAT
M00076	V$GATA2_01	0.81822<br>3	1337	(+)	NNNGATRNNN	AAGCATAGTG
M00075	V$GATA1_01	0.78874<br>6	1347	(+)	SNNGATNNNN	TATGATCTCT
M00076	V$GATA2_01	0.83761<br>8	1347	(+)	NNNGATRNNN	TATGATCTCT
M00126	V$GATA1_02	0.7975	1375	(+)	NNNNNGATANKGNN	GGGGAGATAACATT
M00127	V$GATA1_03	0.91499<br>3	1375	(+)	RNSNNGATAANNGN	GGGGAGATAACATT
M00128	V$GATA1_04	0.86458<br>3	1376	(+)	NNCWGATARNNNN	GGGAGATAACATT
M00075	V$GATA1_01	0.85044<br>4	1377	(+)	SNNGATNNNN	GGAGATAACA
M00076	V$GATA2_01	0.89129<br>5	1377	(+)	NNNGATRNNN	GGAGATAACA
M00077	V$GATA3_01	0.89986<br>7	1378	(+)	NNGATARNG	GAGATAACA
M00203	V$GATA_C	0.85368<br>1	1379	(+)	NGATAAGNMNN	AGATAACATTA
M00076	V$GATA2_01	0.80559<br>3	1389	(-)	NNNGATRNNN	AGGCATGTGG
M00127	V$GATA1_03	0.79152<br>4	1395	(+)	RNSNNGATAANNGN	GTGGGGATGAAGAC
M00075	V$GATA1_01	0.85340<br>6	1397	(+)	SNNGATNNNN	GGGGATGAAG
M00076	V$GATA2_01	0.86107<br>4	1397	(+)	NNNGATRNNN	GGGGATGAAG
M00077	V$GATA3_01	0.83207<br>8	1398	(+)	NNGATARNG	GGGATGAAG
M00075	V$GATA1_01	0.86722<br>6	1422	(-)	SNNGATNNNN	AACAATCTAG
M00076	V$GATA2_01	0.79386<br>6	1422	(-)	NNNGATRNNN	AACAATCTAG
M00077	V$GATA3_01	0.83695<br>2	1422	(-)	NNGATARNG	AACAATCTA
M00127	V$GATA1_03	0.80303<br>8	1447	(+)	RNSNNGATAANNGN	ATGTGGATCAATGG
M00127	V$GATA1_03	0.78931<br>9	1500	(+)	RNSNNGATAANNGN	CTGGAAATAAAAGA
M00126	V$GATA1_02	0.87468<br>8	1507	(+)	NNNNNGATANKGNN	TAAAAGATATGAGG
M00128	V$GATA1_04	0.89583<br>3	1508	(+)	NNCWGATARNNNN	AAAAGATATGAGG
M00075	V$GATA1_01	0.78775<br>9	1509	(+)	SNNGATNNNN	AAAGATATGA
M00076	V$GATA2_01	0.83852<br>1	1509	(+)	NNNGATRNNN	AAAGATATGA
M00077	V$GATA3_01	0.88746<br>1	1510	(+)	NNGATARNG	AAGATATGA
M00076	V$GATA2_01	0.81145<br>7	1511	(-)	NNNGATRNNN	AGATATGAGG
M00127	V$GATA1_03	0.81847<br>1	1518	(+)	RNSNNGATAANNGN	AGGAAGACAAACAT
M00203	V$GATA_C	0.84964<br>3	1522	(+)	NGATAAGNMNN	AGACAAACATT
M00075	V$GATA1_01	0.82181<br>6	1536	(-)	SNNGATNNNN	GAAAATCACT
M00076	V$GATA2_01	0.79070<br>8	1536	(-)	NNNGATRNNN	GAAAATCACT

M00126	V$GATA1_02	0.785	1553	(+)	NNNNNGATANKGNN	GTAGAGAAAAGATA
M00126	V$GATA1_02	0.84375	1558	(+)	NNNNNGATANKGNN	GAAAAGATAAAAAT
M00127	V$GATA1_03	0.849339	1558	(+)	RNSNNGATAANNGN	GAAAAGATAAAAAT
M00128	V$GATA1_04	0.921569	1559	(+)	NNCWGATARNNNN	AAAAGATAAAAAT
M00077	V$GATA3_01	0.8786	1561	(+)	NNGATARNG	AAGATAAAA
M00203	V$GATA_C	0.939112	1562	(+)	NGATAAGNMNN	AGATAAAAATA
M00127	V$GATA1_03	0.785644	1574	(-)	RNSNNGATAANNGN	AGTATTACCTTCTT
M00075	V$GATA1_01	0.846496	1607	(+)	SNNGATNNNN	TCAGATGCCC
M00076	V$GATA2_01	0.900767	1607	(+)	NNNGATRNNN	TCAGATGCCC
M00075	V$GATA1_01	0.778381	1671	(-)	SNNGATNNNN	TCTAATCCCA
M00076	V$GATA2_01	0.814163	1671	(-)	NNNGATRNNN	TCTAATCCCA
M00077	V$GATA3_01	0.857776	1671	(-)	NNGATARNG	TCTAATCCC
M00075	V$GATA1_01	0.792201	1679	(-)	SNNGATNNNN	CAGAATCTAA
M00077	V$GATA3_01	0.824546	1679	(-)	NNGATARNG	CAGAATCTA
M00203	V$GATA_C	0.905561	1687	(-)	NGATAAGNMNN	AAGACATATCT
M00076	V$GATA2_01	0.781236	1688	(+)	NNNGATRNNN	AGACATATCT
M00126	V$GATA1_02	0.790937	1688	(-)	NNNNNGATANKGNN	AGACATATCTGGCA
M00128	V$GATA1_04	0.897978	1688	(-)	NNCWGATARNNNN	AGACATATCTGGC
M00075	V$GATA1_01	0.913129	1690	(-)	SNNGATNNNN	ACATATCTGG
M00076	V$GATA2_01	0.917907	1690	(-)	NNNGATRNNN	ACATATCTGG
M00077	V$GATA3_01	0.849801	1690	(-)	NNGATARNG	ACATATCTG
M00075	V$GATA1_01	0.844028	1737	(-)	SNNGATNNNN	ATCAATCACT
M00076	V$GATA2_01	0.790708	1737	(-)	NNNGATRNNN	ATCAATCACT
M00077	V$GATA3_01	0.835179	1737	(-)	NNGATARNG	ATCAATCAC
M00203	V$GATA_C	0.850264	1756	(-)	NGATAAGNMNN	TGTAAGTATCT
M00126	V$GATA1_02	0.834688	1757	(-)	NNNNNGATANKGNN	GTAAGTATCTGTAA
M00128	V$GATA1_04	0.81924	1757	(-)	NNCWGATARNNNN	GTAAGTATCTGTA
M00075	V$GATA1_01	0.852912	1759	(-)	SNNGATNNNN	AAGTATCTGT
M00076	V$GATA2_01	0.881371	1759	(-)	NNNGATRNNN	AAGTATCTGT
M00127	V$GATA1_03	0.781725	1794	(+)	RNSNNGATAANNGN	ACACTCATAAGTAC
M00203	V$GATA_C	0.835042	1798	(+)	NGATAAGNMNN	TCATAAGTACT
M00076	V$GATA2_01	0.781236	1814	(+)	NNNGATRNNN	TAAGATGAAA
M00077	V$GATA3_01	0.824989	1815	(+)	NNGATARNG	AAGATGAAA

M00127	V$GATA1_03	0.828025	1815	(+)	RNSNNGATAANNGN	AAGATGAAAAGAGT
M00126	V$GATA1_02	0.7925	1831	(+)	NNNNNGATANKGNN	AATAAGATATAGGC
M00128	V$GATA1_04	0.894608	1832	(+)	NNCWGATARNNNN	ATAAGATATAGGC
M00075	V$GATA1_01	0.791214	1833	(+)	SNNGATNNNN	TAAGATATAG
M00076	V$GATA2_01	0.867388	1833	(+)	NNNGATRNNN	TAAGATATAG
M00077	V$GATA3_01	0.881701	1834	(+)	NNGATARNG	AAGATATAG
M00203	V$GATA_C	0.869214	1835	(+)	NGATAAGNMNN	AGATATAGGCT
M00127	V$GATA1_03	0.821166	1841	(+)	RNSNNGATAANNGN	AGGCTGTTAGCTGA
M00075	V$GATA1_01	0.816387	1843	(+)	SNNGATNNNN	GCTGTTAGCT
M00076	V$GATA2_01	0.801534	1843	(+)	NNNGATRNNN	GCTGTTAGCT
M00075	V$GATA1_01	0.90079	1855	(-)	SNNGATNNNN	AAACATCTGG
M00076	V$GATA2_01	0.883175	1855	(-)	NNNGATRNNN	AAACATCTGG
M00127	V$GATA1_03	0.794708	1859	(+)	RNSNNGATAANNGN	ATCTGGATGGCTGG
M00075	V$GATA1_01	0.886476	1861	(+)	SNNGATNNNN	CTGGATGGCT
M00076	V$GATA2_01	0.863329	1861	(+)	NNNGATRNNN	CTGGATGGCT
M00126	V$GATA1_02	0.778125	1879	(-)	NNNNNGATANKGNN	AACATTAACTTGAA
M00076	V$GATA2_01	0.806044	1931	(-)	NNNGATRNNN	CCGTATGATA
M00128	V$GATA1_04	0.882659	1933	(+)	NNCWGATARNNNN	GTATGATAAAATG
M00076	V$GATA2_01	0.798376	1934	(+)	NNNGATRNNN	TATGATAAAA
M00077	V$GATA3_01	0.855117	1935	(+)	NNGATARNG	ATGATAAAA
M00203	V$GATA_C	0.844983	1936	(+)	NGATAAGNMNN	TGATAAAATGT
M00076	V$GATA2_01	0.789806	1985	(-)	NNNGATRNNN	TGCCATGAGG
M00075	V$GATA1_01	0.835637	1999	(+)	SNNGATNNNN	CAGGATCTTG
M00075	V$GATA1_01	0.861303	1999	(-)	SNNGATNNNN	CAGGATCTTG
M00076	V$GATA2_01	0.866486	1999	(+)	NNNGATRNNN	CAGGATCTTG
M00076	V$GATA2_01	0.863329	1999	(-)	NNNGATRNNN	CAGGATCTTG
M00126	V$GATA1_02	0.779062	2011	(+)	NNNNNGATANKGNN	TCCCTGATGAGGGA
M00075	V$GATA1_01	0.92695	2013	(+)	SNNGATNNNN	CCTGATGAGG
M00076	V$GATA2_01	0.87641	2013	(+)	NNNGATRNNN	CCTGATGAGG
M00077	V$GATA3_01	0.871511	2014	(+)	NNGATARNG	CTGATGAGG
M00075	V$GATA1_01	0.773939	2020	(-)	SNNGATNNNN	AGGGATCAAA
M00126	V$GATA1_02	0.800312	2055	(+)	NNNNNGATANKGNN	TCTTAGATATTTGT
M00075	V$GATA1_01	0.783317	2057	(+)	SNNGATNNNN	TTAGATATTT

M00076	V$GATA2_01	0.81281	2057	(+)	NNNGATRNNN	TTAGATATTT
M00126	V$GATA1_02	0.9075	2073	(-)	NNNNNGATANKGNN	TACACTATCTTTGG
M00127	V$GATA1_03	0.809652	2073	(-)	RNSNNGATAANNGN	TACACTATCTTTGG
M00075	V$GATA1_01	0.8692	2075	(-)	SNNGATNNNN	CACTATCTTT
M00076	V$GATA2_01	0.911592	2075	(-)	NNNGATRNNN	CACTATCTTT
M00077	V$GATA3_01	0.961896	2075	(-)	NNGATARNG	CACTATCTT
M00203	V$GATA_C	0.878534	2131	(+)	NGATAAGNMNN	TGACAAGTAAT
M00126	V$GATA1_02	0.774062	2141	(-)	NNNNNGATANKGNN	TACACTATTTCCTC
M00203	V$GATA_C	0.831625	2148	(-)	NGATAAGNMNN	TTTCCTCATCT
M00127	V$GATA1_03	0.826801	2149	(-)	RNSNNGATAANNGN	TTCCTCATCTTCCC
M00075	V$GATA1_01	0.881046	2151	(-)	SNNGATNNNN	CCTCATCTTC
M00076	V$GATA2_01	0.88498	2151	(-)	NNNGATRNNN	CCTCATCTTC
M00077	V$GATA3_01	0.900753	2151	(-)	NNGATARNG	CCTCATCTT
M00075	V$GATA1_01	0.811945	2191	(-)	SNNGATNNNN	CCTCATCCTT
M00076	V$GATA2_01	0.852503	2191	(-)	NNNGATRNNN	CCTCATCCTT
M00077	V$GATA3_01	0.85556	2191	(-)	NNGATARNG	CCTCATCCT
M00127	V$GATA1_03	0.785644	2228	(+)	RNSNNGATAANNGN	AGAGAGGTAAATAC
M00203	V$GATA_C	0.854924	2268	(-)	NGATAAGNMNN	TAGTACTATCT
M00126	V$GATA1_02	0.829063	2269	(-)	NNNNNGATANKGNN	AGTACTATCTGCCT
M00127	V$GATA1_03	0.901274	2269	(-)	RNSNNGATAANNGN	AGTACTATCTGCCT
M00128	V$GATA1_04	0.89951	2269	(-)	NNCWGATARNNNNN	AGTACTATCTGCC
M00075	V$GATA1_01	0.9077	2271	(-)	SNNGATNNNN	TACTATCTGC
M00076	V$GATA2_01	0.923771	2271	(-)	NNNGATRNNN	TACTATCTGC
M00077	V$GATA3_01	0.921134	2271	(-)	NNGATARNG	TACTATCTG
M00076	V$GATA2_01	0.801534	2279	(-)	NNNGATRNNN	GCCTATGTGT
M00127	V$GATA1_03	0.837335	2285	(+)	RNSNNGATAANNGN	GTGTAGAAAATCGC
M00075	V$GATA1_01	0.824284	2290	(-)	SNNGATNNNN	GAAAATCGCA
M00076	V$GATA2_01	0.831304	2290	(-)	NNNGATRNNN	GAAAATCGCA
M00127	V$GATA1_03	0.792014	2296	(-)	RNSNNGATAANNGN	CGCATTACCAACAT
M00126	V$GATA1_02	0.797188	2317	(+)	NNNNNGATANKGNN	GTATAAATAATGCA
M00075	V$GATA1_01	0.882527	2329	(-)	SNNGATNNNN	CACAATCTCA
M00076	V$GATA2_01	0.887686	2329	(-)	NNNGATRNNN	CACAATCTCA
M00077	V$GATA3_01	0.945503	2329	(-)	NNGATARNG	CACAATCTC
M00075	V$GATA1_01	0.816387	2336	(+)	SNNGATNNNN	TCAGATTTTT

M00076	V$GATA2_01	0.83806 9	2336	(+)	NNNGATRNNN	TCAGATTTTT
M00203	V$GATA_C	0.83845 9	2375	(-)	NGATAAGNMNN	ATCCACTATCT
M00126	V$GATA1_02	0.89406 3	2376	(-)	NNNNNGATANKGNN	TCCACTATCTCAGT
M00127	V$GATA1_03	0.82092 1	2376	(-)	RNSNNGATAANNGN	TCCACTATCTCAGT
M00128	V$GATA1_04	0.81770 8	2376	(-)	NNCWGATARNNNN	TCCACTATCTCAG
M00075	V$GATA1_01	0.90621 9	2378	(-)	SNNGATNNNN	CACTATCTCA
M00076	V$GATA2_01	0.95218 8	2378	(-)	NNNGATRNNN	CACTATCTCA
M00077	V$GATA3_01	0.97917 6	2378	(-)	NNGATARNG	CACTATCTC
M00075	V$GATA1_01	0.80059 2	2389	(-)	SNNGATNNNN	TAGTATCCTA
M00076	V$GATA2_01	0.89039 2	2389	(-)	NNNGATRNNN	TAGTATCCTA
M00076	V$GATA2_01	0.80469 1	2394	(-)	NNNGATRNNN	TCCTATGGGA
M00126	V$GATA1_02	0.77218 8	2437	(-)	NNNNNGATANKGNN	TGTACCATCTGTAC
M00075	V$GATA1_01	0.84304	2439	(-)	SNNGATNNNN	TACCATCTGT
M00076	V$GATA2_01	0.84844 4	2439	(-)	NNNGATRNNN	TACCATCTGT
M00077	V$GATA3_01	0.86752 3	2439	(-)	NNGATARNG	TACCATCTG
M00075	V$GATA1_01	0.80700 9	2448	(-)	SNNGATNNNN	TACCATCAAT
M00077	V$GATA3_01	0.82543 2	2448	(-)	NNGATARNG	TACCATCAA
M00075	V$GATA1_01	0.81885 5	2452	(-)	SNNGATNNNN	ATCAATCCCT
M00076	V$GATA2_01	0.81641 9	2452	(-)	NNNGATRNNN	ATCAATCCCT
M00162	V$OCT1_06	0.80078 1	47	(-)	CWNAWTKWSATRYN	AGAATTCATTTTCT
M00137	V$OCT1_03	0.87198 7	56	(+)	NNNRTAATNANNN	TTTCTAATCATGC
M00138	V$OCT1_04	0.83187	57	(+)	NNNNNNNWATGCAAATNNNWN NW	TTCTAATCATGCAGAT- TTCTAGG
M00135	V$OCT1_01	0.82392 2	59	(+)	NNNNWTATGCAAATNTNNN	CTAATCATGCAGATTTC- TA
M00136	V$OCT1_02	0.79263 7	62	(-)	NNGAATATKCANNNN	ATCATGCAGATTTCT
M00248	V$OCT1_07	0.79987 3	62	(+)	TNTATGNTAATT	ATCATGCAGATT
M00138	V$OCT1_04	0.78419 1	73	(+)	NNNNNNNWATGCAAATNNNWN NW	TTCTAG- GAATTCAAATCCACTAT
M00135	V$OCT1_01	0.73235 4	75	(+)	NNNNWTATGCAAATNTNNN	CTAG- GAATTCAAATCCACT
M00195	V$OCT1_Q6	0.83051 3	77	(+)	NNNNATGCAAATNAN	AGGAATTCAAATCCA
M00162	V$OCT1_06	0.80117 2	78	(-)	CWNAWTKWSATRYN	GGAATTCAAATCCA
M00162	V$OCT1_06	0.82148 4	84	(-)	CWNAWTKWSATRYN	CAAATCCACTATTG
M00162	V$OCT1_06	0.82148 4	90	(+)	CWNAWTKWSATRYN	CACTATTGGTTTTA
M00162	V$OCT1_06	0.80039 1	107	(-)	CWNAWTKWSATRYN	CAAACCACAAAATT
M00138	V$OCT1_04	0.81890 4	111	(-)	NNNNNNNWATGCAAATNNNWN NW	CCACAAAATTAG- CATGCCATTAA

M00135	V$OCT1_01	0.816482	113	(-)	NNNNWTATGCAAATNTNNN	ACAAAATTAG-CATGCCATT
M00161	V$OCT1_05	0.869002	115	(+)	MKNATTTGCATAYY	AAAATTAGCATGCC
M00162	V$OCT1_06	0.855469	115	(+)	CWNAWTKWSATRYN	AAAATTAGCATGCC
M00248	V$OCT1_07	0.912868	117	(-)	TNTATGNTAATT	AATTAGCATGCC
M00162	V$OCT1_06	0.889062	121	(-)	CWNAWTKWSATRYN	AGCATGCCATTAAA
M00162	V$OCT1_06	0.829297	125	(+)	CWNAWTKWSATRYN	TGCCATTAAATACT
M00138	V$OCT1_04	0.782936	130	(-)	NNNNNNNWATGCAAATNNNWNNW	TTAAATACTATA-TATAAACAACC
M00138	V$OCT1_04	0.814722	131	(+)	NNNNNNNWATGCAAATNNNWNNW	TAAATACTATA-TATAAACAACCA
M00248	V$OCT1_07	0.796269	132	(-)	TNTATGNTAATT	AAATACTATATA
M00195	V$OCT1_Q6	0.792031	137	(+)	NNNNATGCAAATNAN	CTATATATAAACAAC
M00137	V$OCT1_03	0.914263	161	(-)	NNNRTAATNANNN	AGATCATTATCCA
M00136	V$OCT1_02	0.818896	163	(+)	NNGAATATKCANNNN	ATCATTATCCATTCA
M00162	V$OCT1_06	0.816016	212	(-)	CWNAWTKWSATRYN	AAAAGGTAAGAATC
M00162	V$OCT1_06	0.890625	220	(-)	CWNAWTKWSATRYN	AGAATCTCAGATAT
M00162	V$OCT1_06	0.834375	221	(+)	CWNAWTKWSATRYN	GAATCTCAGATATA
M00137	V$OCT1_03	0.841565	229	(+)	NNNRTAATNANNN	GATATAATTTCAT
M00162	V$OCT1_06	0.812109	231	(+)	CWNAWTKWSATRYN	TATAATTTCATTGT
M00137	V$OCT1_03	0.846701	235	(-)	NNNRTAATNANNN	ATTTCATTGTATC
M00137	V$OCT1_03	0.868431	254	(-)	NNNRTAATNANNN	TCATCTTTATTTC
M00162	V$OCT1_06	0.860156	258	(+)	CWNAWTKWSATRYN	CTTTATTTCAGACT
M00135	V$OCT1_01	0.736932	290	(-)	NNNNWTATGCAAATNTNNN	AACATAATTGCTTAAAA-TA
M00137	V$OCT1_03	0.843145	290	(+)	NNNRTAATNANNN	AACATAATTGCTT
M00137	V$OCT1_03	0.853023	291	(-)	NNNRTAATNANNN	ACATAATTGCTTA
M00162	V$OCT1_06	0.804297	292	(+)	CWNAWTKWSATRYN	CATAATTGCTTAAA
M00135	V$OCT1_01	0.746471	347	(+)	NNNNWTATGCAAATNTNNN	TATTTTCAGCAAATTTGTT
M00138	V$OCT1_04	0.800084	355	(+)	NNNNNNNWATGCAAATNNNWNNW	GCAAATTTGTTAAAA-TAGAAGCA
M00137	V$OCT1_03	0.842355	378	(+)	NNNRTAATNANNN	ACTATAAAGATTT
M00137	V$OCT1_03	0.88305	407	(-)	NNNRTAATNANNN	TTTTCTTTACCAC
M00162	V$OCT1_06	0.903516	437	(-)	CWNAWTKWSATRYN	TGTATTAAAAGATC
M00138	V$OCT1_04	0.80092	454	(-)	NNNNNNNWATGCAAATNNNWNNW	TCTTGAATTAAA-TATTTTCAATT
M00162	V$OCT1_06	0.868359	456	(+)	CWNAWTKWSATRYN	TTGAATTAAATATT
M00162	V$OCT1_06	0.898437	457	(-)	CWNAWTKWSATRYN	TGAATTAAATATTT
M00136	V$OCT1_02	0.803194	458	(-)	NNGAATATKCANNNN	GAATTAAATATTTTC

M00138	V$OCT1_04	0.783982	460	(-)	NNNNNNNWATGCAAATNNNWNNW	ATTAAATATTTTCAATTT-GATTA
M00138	V$OCT1_04	0.810958	461	(-)	NNNNNNNWATGCAAATNNNWNNW	TTAAATATTTTCAATTT-GATTAA
M00162	V$OCT1_06	0.847266	464	(+)	CWNAWTKWSATRYN	AATATTTTCAATTT
M00162	V$OCT1_06	0.800781	465	(+)	CWNAWTKWSATRYN	ATATTTTCAATTTG
M00138	V$OCT1_04	0.815977	467	(-)	NNNNNNNWATGCAAATNNNWNNW	ATTTTCAATTTGAT-TAAACATAC
M00135	V$OCT1_01	0.745326	469	(-)	NNNNWTATGCAAATNTNNN	TTTCAATTTGATTAAACAT
M00138	V$OCT1_04	0.806357	473	(-)	NNNNNNNWATGCAAATNNNWNNW	AATTTGATTAAACAT-ACCTCAGC
M00248	V$OCT1_07	0.814501	473	(-)	TNTATGNTAATT	AATTTGATTAAA
M00137	V$OCT1_03	0.856578	474	(-)	NNNRTAATNANNN	ATTTGATTAAACA
M00162	V$OCT1_06	0.821484	507	(+)	CWNAWTKWSATRYN	GCACATTTAATTTA
M00162	V$OCT1_06	0.932422	508	(-)	CWNAWTKWSATRYN	CACATTTAATTTAT
M00135	V$OCT1_01	0.751049	519	(+)	NNNNWTATGCAAATNTNNN	TATACTATGGGAATTT-GAA
M00248	V$OCT1_07	0.88446	522	(+)	TNTATGNTAATT	ACTATGGGAATT
M00138	V$OCT1_04	0.87432	524	(-)	NNNNNNNWATGCAAATNNNWNNW	TATGGGAATTTGAA-TAATTGTTA
M00135	V$OCT1_01	0.865319	526	(-)	NNNNWTATGCAAATNTNNN	TGGGAATTTGAA-TAATTGT
M00161	V$OCT1_05	0.878798	528	(+)	MKNATTTGCATAYY	GGAATTTGAATAAT
M00162	V$OCT1_06	0.819141	528	(+)	CWNAWTKWSATRYN	GGAATTTGAATAAT
M00162	V$OCT1_06	0.826953	528	(-)	CWNAWTKWSATRYN	GGAATTTGAATAAT
M00195	V$OCT1_Q6	0.845797	528	(-)	NNNNATGCAAATNAN	GGAATTTGAATAATT
M00136	V$OCT1_02	0.850027	530	(-)	NNGAATATKCANNNN	AATTTGAATAATTGT
M00248	V$OCT1_07	0.893152	530	(-)	TNTATGNTAATT	AATTTGAATAAT
M00137	V$OCT1_03	0.847096	534	(+)	NNNRTAATNANNN	TGAATAATTGTTA
M00135	V$OCT1_01	0.778138	575	(-)	NNNNWTATGCAAATNTNNN	ATAGAGCTAG-CATATTTAG
M00136	V$OCT1_02	0.846779	579	(-)	NNGAATATKCANNNN	AGCTAGCATATTTAG
M00136	V$OCT1_02	0.790471	582	(+)	NNGAATATKCANNNN	TAGCATATTTAGTCA
M00137	V$OCT1_03	0.867246	598	(+)	NNNRTAATNANNN	GAGATAAAGAGGG
M00135	V$OCT1_01	0.737696	619	(+)	NNNNWTATGCAAATNTNNN	ATACATGTGCTATTT-GAAA
M00162	V$OCT1_06	0.826172	619	(+)	CWNAWTKWSATRYN	ATACATGTGCTATT
M00162	V$OCT1_06	0.847266	628	(+)	CWNAWTKWSATRYN	CTATTTGAAAGGTA
M00162	V$OCT1_06	0.813281	629	(-)	CWNAWTKWSATRYN	TATTTGAAAGGTAT
M00138	V$OCT1_04	0.811167	633	(+)	NNNNNNNWATGCAAATNNNWNNW	TGAAAGGTATTTATAAAAGAAGA
M00162	V$OCT1_06	0.826562	638	(-)	CWNAWTKWSATRYN	GGTATTTATAAAAG
M00138	V$OCT1_04	0.795692	650	(+)	NNNNNNNWATGCAAATNNNWNNW	AGAAGAG-TATATTTATTAAAATT

M00136	V$OCT1_02	0.804006	654	(+)	NNGAATATKCANNNN	GAGTATATTTATTAA
M00138	V$OCT1_04	0.836052	656	(+)	NNNNNNNWATGCAAATNNNWNNW	GTATATTTATTAAAATT-GCTCAG
M00138	V$OCT1_04	0.807403	657	(-)	NNNNNNNWATGCAAATNNNWNNW	TATATTTATTAAAATT-GCTCAGA
M00135	V$OCT1_01	0.753338	658	(+)	NNNNWTATGCAAATNTNNN	ATATTTATTAAAATTGCTC
M00248	V$OCT1_07	0.834005	661	(+)	TNTATGNTAATT	TTTATTAAAATT
M00138	V$OCT1_04	0.780217	674	(+)	NNNNNNNWATGCAAATNNNWNNW	CTCAGAACATCCAAATTTCAAGT
M00135	V$OCT1_01	0.819725	676	(+)	NNNNWTATGCAAATNTNNN	CAGAACATCCAAATTTCAA
M00136	V$OCT1_02	0.796156	676	(+)	NNGAATATKCANNNN	CAGAACATCCAAATT
M00195	V$OCT1_Q6	0.822871	678	(+)	NNNNATGCAAATNAN	GAACATCCAAATTTC
M00162	V$OCT1_06	0.9375	679	(-)	CWNAWTKWSATRYN	AACATCCAAATTTC
M00248	V$OCT1_07	0.800933	679	(+)	TNTATGNTAATT	AACATCCAAATT
M00162	V$OCT1_06	0.825	684	(+)	CWNAWTKWSATRYN	CCAAATTTCAAGTT
M00162	V$OCT1_06	0.885547	685	(-)	CWNAWTKWSATRYN	CAAATTTCAAGTTT
M00138	V$OCT1_04	0.799247	689	(-)	NNNNNNNWATGCAAATNNNWNNW	TTTCAAGTTTATCATTTATCTTA
M00162	V$OCT1_06	0.821484	693	(+)	CWNAWTKWSATRYN	AAGTTTATCATTTA
M00162	V$OCT1_06	0.839453	705	(-)	CWNAWTKWSATRYN	TATCTTACAATATT
M00138	V$OCT1_04	0.809285	709	(+)	NNNNNNNWATGCAAATNNNWNNW	TTACAATATTTCAAAAA-TATTAA
M00162	V$OCT1_06	0.903516	713	(-)	CWNAWTKWSATRYN	AATATTTCAAAAAT
M00162	V$OCT1_06	0.818359	715	(-)	CWNAWTKWSATRYN	TATTTCAAAAATAT
M00138	V$OCT1_04	0.872438	719	(+)	NNNNNNNWATGCAAATNNNWNNW	TCAAAAATATTAAAA-TAGATACA
M00138	V$OCT1_04	0.801129	720	(-)	NNNNNNNWATGCAAATNNNWNNW	CAAAAATATTAAAA-TAGATACAT
M00135	V$OCT1_01	0.742655	721	(+)	NNNNWTATGCAAATNTNNN	AAAAATATTAAAATAGA-TA
M00136	V$OCT1_02	0.805901	721	(+)	NNGAATATKCANNNN	AAAAATATTAAAATA
M00248	V$OCT1_07	0.836549	726	(-)	TNTATGNTAATT	TATTAAAATAGA
M00162	V$OCT1_06	0.803906	728	(+)	CWNAWTKWSATRYN	TTAAAATAGATACA
M00162	V$OCT1_06	0.911328	737	(+)	CWNAWTKWSATRYN	ATACATGAAATACA
M00162	V$OCT1_06	0.945312	738	(-)	CWNAWTKWSATRYN	TACATGAAATACAG
M00162	V$OCT1_06	0.800781	755	(-)	CWNAWTKWSATRYN	TAAATTAAAGAGAA
M00162	V$OCT1_06	0.829297	768	(+)	CWNAWTKWSATRYN	AAGTATTTTATTTT
M00162	V$OCT1_06	0.894531	769	(-)	CWNAWTKWSATRYN	AGTATTTTATTTTG
M00248	V$OCT1_07	0.817257	769	(+)	TNTATGNTAATT	AGTATTTTATTT
M00138	V$OCT1_04	0.808657	771	(-)	NNNNNNNWATGCAAATNNNWNNW	TATTTTATTTT-GTAAAAAAAAAT
M00138	V$OCT1_04	0.819113	772	(+)	NNNNNNNWATGCAAATNNNWNNW	ATTTTATTTT-GTAAAAAAAAATT

M00137	V$OCT1_03	0.849072	779	(+)	NNNRTAATNANNN	TTTGTAAAAAAAA
M00162	V$OCT1_06	0.814062	789	(-)	CWNAWTKWSATRYN	AAAATTCTAGGTTG
M00162	V$OCT1_06	0.829297	827	(+)	CWNAWTKWSATRYN	AACAATGAAAAATG
M00162	V$OCT1_06	0.868359	834	(+)	CWNAWTKWSATRYN	AAAAATGTGATCTG
M00162	V$OCT1_06	0.800391	842	(-)	CWNAWTKWSATRYN	GATCTGACAGAAAT
M00137	V$OCT1_03	0.908337	876	(+)	NNNRTAATNANNN	TCAGTAATGAAAT
M00162	V$OCT1_06	0.842578	878	(+)	CWNAWTKWSATRYN	AGTAATGAAATGGC
M00138	V$OCT1_04	0.825596	891	(-)	NNNNNNNWATGCAAATNNNWNNW	CTTAAAAATTGGCATA-TAAAATG
M00138	V$OCT1_04	0.780008	892	(+)	NNNNNNNWATGCAAATNNNWNNW	TTAAAAATTGGCATA-TAAAATGC
M00135	V$OCT1_01	0.85826	893	(-)	NNNNWTATGCAAATNTNNN	TAAAAATTGGCATA-TAAAA
M00162	V$OCT1_06	0.9375	895	(+)	CWNAWTKWSATRYN	AAAATTGGCATATA
M00195	V$OCT1_Q6	0.790393	895	(-)	NNNNATGCAAATNAN	AAAATTGGCATATAA
M00136	V$OCT1_02	0.787223	897	(-)	NNGAATATKCANNNN	AATTGGCATATAAAA
M00248	V$OCT1_07	0.902904	897	(-)	TNTATGNTAATT	AATTGGCATATA
M00138	V$OCT1_04	0.854872	903	(+)	NNNNNNNWATGCAAATNNNWNNW	CATA-TAAAATGCTAATTATAAAA
M00135	V$OCT1_01	0.820488	905	(+)	NNNNWTATGCAAATNTNNN	TA-TAAAATGCTAATTATAA
M00136	V$OCT1_02	0.774499	905	(+)	NNGAATATKCANNNN	TATAAAATGCTAATT
M00195	V$OCT1_Q6	0.861627	907	(+)	NNNNATGCAAATNAN	TAAAATGCTAATTAT
M00161	V$OCT1_05	0.959157	908	(-)	MKNATTTGCATAYY	AAAATGCTAATTAT
M00162	V$OCT1_06	0.839844	908	(-)	CWNAWTKWSATRYN	AAAATGCTAATTAT
M00248	V$OCT1_07	0.921772	908	(+)	TNTATGNTAATT	AAAATGCTAATT
M00138	V$OCT1_04	0.837725	909	(+)	NNNNNNNWATGCAAATNNNWNNW	AAATGCTAATTATAAAA-TAAACA
M00137	V$OCT1_03	0.879494	911	(+)	NNNRTAATNANNN	ATGCTAATTATAA
M00137	V$OCT1_03	0.893323	912	(-)	NNNRTAATNANNN	TGCTAATTATAAA
M00136	V$OCT1_02	0.770709	928	(+)	NNGAATATKCANNNN	AACAAAATGTAATAA
M00162	V$OCT1_06	0.886719	930	(+)	CWNAWTKWSATRYN	CAAAATGTAATAAT
M00162	V$OCT1_06	0.870703	931	(-)	CWNAWTKWSATRYN	AAAATGTAATAATA
M00137	V$OCT1_03	0.860925	933	(+)	NNNRTAATNANNN	AATGTAATAATAC
M00162	V$OCT1_06	0.860547	951	(+)	CWNAWTKWSATRYN	CTACATGTAATGAA
M00195	V$OCT1_Q6	0.815502	951	(+)	NNNNATGCAAATNAN	CTACATGTAATGAAC
M00162	V$OCT1_06	0.939844	952	(-)	CWNAWTKWSATRYN	TACATGTAATGAAC
M00137	V$OCT1_03	0.883445	954	(+)	NNNRTAATNANNN	CATGTAATGAACT

M00136	V$OCT1_02	0.778831	964	(-)	NNGAATATKCANNNN	ACTCTGAGTATTATA
M00162	V$OCT1_06	0.805859	981	(+)	CWNAWTKWSATRYN	CTTTTTTGAAGTCT
M00138	V$OCT1_04	0.886031	992	(+)	NNNNNNNWATGCAAATNNNWNNW	TCTT-GACAATGAAAATTTATTTA
M00135	V$OCT1_01	0.759824	994	(+)	NNNNWTATGCAAATNTNNN	TTGACAATGAAAATTTATT
M00137	V$OCT1_03	0.868431	994	(+)	NNNRTAATNANNN	TTGACAATGAAAA
M00162	V$OCT1_06	0.829297	996	(+)	CWNAWTKWSATRYN	GACAATGAAAATTT
M00136	V$OCT1_02	0.788576	997	(-)	NNGAATATKCANNNN	ACAATGAAAATTTAT
M00162	V$OCT1_06	0.804297	997	(-)	CWNAWTKWSATRYN	ACAATGAAAATTTA
M00248	V$OCT1_07	0.824253	997	(+)	TNTATGNTAATT	ACAATGAAAATT
M00136	V$OCT1_02	0.827287	1000	(+)	NNGAATATKCANNNN	ATGAAAATTTATTTA
M00138	V$OCT1_04	0.789628	1002	(+)	NNNNNNNWATGCAAATNNNWNNW	GAAAATTTATTTA-GACTTTTATA
M00195	V$OCT1_Q6	0.803493	1039	(+)	NNNNATGCAAATNAN	AGTAAAACAAATTAC
M00162	V$OCT1_06	0.852734	1041	(-)	CWNAWTKWSATRYN	TAAAACAAATTACG
M00137	V$OCT1_03	0.898459	1044	(-)	NNNRTAATNANNN	AACAAATTACGAA
M00195	V$OCT1_Q6	0.79476	1046	(+)	NNNNATGCAAATNAN	CAAATTACGAATTAG
M00138	V$OCT1_04	0.813049	1049	(-)	NNNNNNNWATGCAAATNNNWNNW	ATTACGAATTAG-CATCCATGAGA
M00135	V$OCT1_01	0.809233	1051	(-)	NNNNWTATGCAAATNTNNN	TACGAATTAG-CATCCATGA
M00195	V$OCT1_Q6	0.855622	1053	(-)	NNNNATGCAAATNAN	CGAATTAGCATCCAT
M00248	V$OCT1_07	0.904812	1055	(-)	TNTATGNTAATT	AATTAGCATCCA
M00138	V$OCT1_04	0.791928	1069	(+)	NNNNNNNWATGCAAATNNNWNNW	AGAAAAA-TATAGAAAAAATTTCT
M00162	V$OCT1_06	0.842187	1083	(-)	CWNAWTKWSATRYN	AAAATTTCTTAATG
M00162	V$OCT1_06	0.821094	1085	(+)	CWNAWTKWSATRYN	AATTTCTTAATGTA
M00137	V$OCT1_03	0.846701	1088	(+)	NNNRTAATNANNN	TTCTTAATGTAGT
M00162	V$OCT1_06	0.839844	1090	(+)	CWNAWTKWSATRYN	CTTAATGTAGTTTG
M00135	V$OCT1_01	0.744182	1094	(-)	NNNNWTATGCAAATNTNNN	ATGTAGTTT-GCAAATCTGG
M00135	V$OCT1_01	0.842999	1095	(+)	NNNNWTATGCAAATNTNNN	TGTAGTTT-GCAAATCTGGG
M00195	V$OCT1_Q6	0.842795	1096	(-)	NNNNATGCAAATNAN	GTAGTTTGCAAATCT
M00195	V$OCT1_Q6	0.810862	1097	(+)	NNNNATGCAAATNAN	TAGTTTGCAAATCTG
M00162	V$OCT1_06	0.860547	1098	(-)	CWNAWTKWSATRYN	AGTTTGCAAATCTG
M00162	V$OCT1_06	0.800781	1105	(+)	CWNAWTKWSATRYN	AAATCTGGGATTGA
M00138	V$OCT1_04	0.821205	1164	(+)	NNNNNNNWATGCAAATNNNWNNW	AA-GAACTTATAAAAATGCAACAA
M00137	V$OCT1_03	0.841169	1169	(+)	NNNRTAATNANNN	CTTATAAAAATGC

M00138	V$OCT1_04	0.828524	1170	(+)	NNNNNNNWATGCAAATNNNWNNW	TTATAAAAATGCAACAAAACAAA
M00136	V$OCT1_02	0.845966	1172	(+)	NNGAATATKCANNNN	ATAAAAATGCAACAA
M00162	V$OCT1_06	0.898437	1191	(+)	CWNAWTKWSATRYN	AACCATTTAATACA
M00162	V$OCT1_06	0.829297	1192	(-)	CWNAWTKWSATRYN	ACCATTTAATACAT
M00162	V$OCT1_06	0.828906	1195	(+)	CWNAWTKWSATRYN	ATTTAATACATTTT
M00138	V$OCT1_04	0.782727	1214	(+)	NNNNNNNWATGCAAATNNNWNNW	AAAA- TAGTATGTATTTTATTTTA
M00136	V$OCT1_02	0.782079	1216	(+)	NNGAATATKCANNNN	AATAGTATGTATTTT
M00162	V$OCT1_06	0.867969	1219	(-)	CWNAWTKWSATRYN	AGTATGTATTTTAT
M00162	V$OCT1_06	0.821484	1222	(+)	CWNAWTKWSATRYN	ATGTATTTTATTTT
M00162	V$OCT1_06	0.812109	1223	(-)	CWNAWTKWSATRYN	TGTATTTTATTTTA
M00248	V$OCT1_07	0.836549	1223	(+)	TNTATGNTAATT	TGTATTTTATTT
M00135	V$OCT1_01	0.731591	1230	(+)	NNNNWTATGCAAATNTNNN	TATTTTATGC- TACAAGGAG
M00162	V$OCT1_06	0.805859	1294	(-)	CWNAWTKWSATRYN	TAAATCACCGAGAG
M00135	V$OCT1_01	0.795498	1330	(-)	NNNNWTATGCAAATNTNNN	AGAAAATAAGCATAGTG- TA
M00136	V$OCT1_02	0.77098	1331	(+)	NNGAATATKCANNNN	GAAAATAAGCATAGT
M00136	V$OCT1_02	0.775582	1334	(-)	NNGAATATKCANNNN	AATAAGCATAGTGTA
M00248	V$OCT1_07	0.882764	1334	(-)	TNTATGNTAATT	AATAAGCATAGT
M00162	V$OCT1_06	0.847266	1350	(-)	CWNAWTKWSATRYN	GATCTCTAAAATTG
M00137	V$OCT1_03	0.873963	1381	(-)	NNNRTAATNANNN	ATAACATTAGGCA
M00135	V$OCT1_01	0.785196	1469	(+)	NNNNWTATGCAAATNTNNN	GAA- GAAATGCTAAAATGAA
M00162	V$OCT1_06	0.834766	1472	(-)	CWNAWTKWSATRYN	GAAATGCTAAAATG
M00162	V$OCT1_06	0.800781	1503	(-)	CWNAWTKWSATRYN	GAAATAAAAGATAT
M00136	V$OCT1_02	0.817001	1524	(+)	NNGAATATKCANNNN	ACAAACATTCATGAA
M00162	V$OCT1_06	0.804297	1525	(+)	CWNAWTKWSATRYN	CAAACATTCATGAA
M00138	V$OCT1_04	0.818904	1526	(+)	NNNNNNNWATGCAAATNNNWNNW	AAACATTCATGAAAATCACTTAG
M00135	V$OCT1_01	0.735788	1528	(+)	NNNNWTATGCAAATNTNNN	ACATTCATGAAAATCACTT
M00162	V$OCT1_06	0.808203	1530	(+)	CWNAWTKWSATRYN	ATTCATGAAAATCA
M00195	V$OCT1_Q6	0.887555	1530	(+)	NNNNATGCAAATNAN	ATTCATGAAAATCAC
M00162	V$OCT1_06	0.860547	1531	(-)	CWNAWTKWSATRYN	TTCATGAAAATCAC
M00162	V$OCT1_06	0.808203	1537	(-)	CWNAWTKWSATRYN	AAAATCACTTAGTT
M00138	V$OCT1_04	0.7867	1554	(+)	NNNNNNNWATGCAAATNNNWNNW	TAGAGAAAAGA- TAAAAATAAAGT
M00162	V$OCT1_06	0.813672	1566	(-)	CWNAWTKWSATRYN	AAAAATAAAGTATT
M00162	V$OCT1_06	0.823828	1573	(+)	CWNAWTKWSATRYN	AAGTATTACCTTCT

M00162	V$OCT1_06	0.834375	1574	(-)	CWNAWTKWSATRYN	AGTATTACCTTCTT
M00138	V$OCT1_04	0.788791	1579	(-)	NNNNNNNWATGCAAATNNNWNNW	TACCTTCTTCTTCAT-ATACATTG
M00138	V$OCT1_04	0.783563	1580	(+)	NNNNNNNWATGCAAATNNNWNNW	ACCTTCTTCTTCAT-ATACATTGT
M00195	V$OCT1_Q6	0.793941	1585	(-)	NNNNATGCAAATNAN	CTTCTTCATATACAT
M00162	V$OCT1_06	0.800781	1640	(+)	CWNAWTKWSATRYN	AGAACTGAAACTTT
M00135	V$OCT1_01	0.736169	1644	(-)	NNNNWTATGCAAATNTNNN	CTGAAACTTTAATATTTTG
M00162	V$OCT1_06	0.916797	1646	(+)	CWNAWTKWSATRYN	GAAACTTTAATATT
M00162	V$OCT1_06	0.816016	1647	(-)	CWNAWTKWSATRYN	AAACTTTAATATTT
M00136	V$OCT1_02	0.777748	1648	(-)	NNGAATATKCANNNN	AACTTTAATATTTTG
M00162	V$OCT1_06	0.877734	1680	(-)	CWNAWTKWSATRYN	AGAATCTAAGACAT
M00162	V$OCT1_06	0.816016	1683	(+)	CWNAWTKWSATRYN	ATCTAAGACATATC
M00138	V$OCT1_04	0.801757	1694	(+)	NNNNNNNWATGCAAATNNNWNNW	ATCTGGCAA-TAAAAATTAATAAA
M00138	V$OCT1_04	0.795483	1695	(-)	NNNNNNNWATGCAAATNNNWNNW	TCTGGCAA-TAAAAATTAATAAAT
M00137	V$OCT1_03	0.853023	1696	(+)	NNNRTAATNANNN	CTGGCAATAAAAA
M00195	V$OCT1_Q6	0.799127	1698	(+)	NNNNATGCAAATNAN	GGCAATAAAAATTAA
M00138	V$OCT1_04	0.827896	1700	(+)	NNNNNNNWATGCAAATNNNWNNW	CAATAAAAATTAATAAA-TAAATA
M00138	V$OCT1_04	0.786909	1704	(+)	NNNNNNNWATGCAAATNNNWNNW	AAAAATTAATAAATAAA-TATTTT
M00162	V$OCT1_06	0.808203	1704	(-)	CWNAWTKWSATRYN	AAAAATTAATAAAT
M00248	V$OCT1_07	0.793513	1707	(-)	TNTATGNTAATT	AATTAATAAATA
M00138	V$OCT1_04	0.800293	1708	(+)	NNNNNNNWATGCAAATNNNWNNW	ATTAATAAATAAA-TATTTTTAAT
M00137	V$OCT1_03	0.847886	1709	(+)	NNNRTAATNANNN	TTAATAAATAAAT
M00138	V$OCT1_04	0.814095	1709	(-)	NNNNNNNWATGCAAATNNNWNNW	TTAATAAATAAA-TATTTTTAATA
M00162	V$OCT1_06	0.855078	1711	(+)	CWNAWTKWSATRYN	AATAAATAAATATT
M00136	V$OCT1_02	0.840282	1713	(-)	NNGAATATKCANNNN	TAAATAAATATTTTT
M00138	V$OCT1_04	0.822668	1714	(+)	NNNNNNNWATGCAAATNNNWNNW	AAATAAATATTTTTAA-TAAGTAA
M00136	V$OCT1_02	0.772604	1716	(+)	NNGAATATKCANNNN	ATAAATATTTTTAAT
M00138	V$OCT1_04	0.836052	1716	(-)	NNNNNNNWATGCAAATNNNWNNW	ATAAATATTTTTAA-TAAGTAAAT
M00162	V$OCT1_06	0.805859	1720	(+)	CWNAWTKWSATRYN	ATATTTTTAATAAG
M00137	V$OCT1_03	0.858949	1723	(+)	NNNRTAATNANNN	TTTTTAATAAGTA
M00135	V$OCT1_01	0.744945	1725	(+)	NNNNWTATGCAAATNTNNN	TTTAA-TAAGTAAATCAATC
M00195	V$OCT1_Q6	0.790939	1727	(+)	NNNNATGCAAATNAN	TAATAAGTAAATCAA
M00162	V$OCT1_06	0.808203	1740	(+)	CWNAWTKWSATRYN	AATCACTTAATTTT
M00162	V$OCT1_06	0.800781	1741	(+)	CWNAWTKWSATRYN	ATCACTTAATTTTT

M00138	V$OCT1_04	0.78189	1742	(-)	NNNNNNNWATGCAAATNNNWNNW	TCACTTAATTTTTCTG-TAAGTAT
M00136	V$OCT1_02	0.788576	1806	(-)	NNGAATATKCANNNN	ACTGTGAATAAGATG
M00162	V$OCT1_06	0.804297	1828	(+)	CWNAWTKWSATRYN	TGAAATAAGATATA
M00248	V$OCT1_07	0.793513	1830	(-)	TNTATGNTAATT	AAATAAGATATA
M00162	V$OCT1_06	0.862891	1884	(+)	CWNAWTKWSATRYN	TAACTTGAAATGTA
M00138	V$OCT1_04	0.791301	1885	(+)	NNNNNNNWATGCAAATNNNWNNW	AACTTGAAATGTAAGAT-TAATGA
M00162	V$OCT1_06	0.837109	1890	(-)	CWNAWTKWSATRYN	GAAATGTAAGATTA
M00137	V$OCT1_03	0.861715	1898	(+)	NNNRTAATNANNN	AGATTAATGAGTA
M00136	V$OCT1_02	0.775853	1901	(-)	NNGAATATKCANNNN	TTAATGAGTAATAGT
M00137	V$OCT1_03	0.8629	1905	(+)	NNNRTAATNANNN	TGAGTAATAGTAA
M00135	V$OCT1_01	0.760397	1929	(+)	NNNNWTATGCAAATNTNNN	GGCCGTATGA-TAAAATGTC
M00162	V$OCT1_06	0.860547	1932	(-)	CWNAWTKWSATRYN	CGTATGATAAAATG
M00138	V$OCT1_04	0.815977	1935	(+)	NNNNNNNWATGCAAATNNNWNNW	ATGATAAAATGTC-TATTAATATT
M00138	V$OCT1_04	0.859264	1941	(+)	NNNNNNNWATGCAAATNNNWNNW	AAATGTCTATTAA-TATTTTTCTA
M00136	V$OCT1_02	0.788035	1946	(-)	NNGAATATKCANNNN	TCTATTAATATTTTT
M00136	V$OCT1_02	0.791012	1949	(+)	NNGAATATKCANNNN	ATTAATATTTTTCTA
M00162	V$OCT1_06	0.898437	2052	(+)	CWNAWTKWSATRYN	GAGTCTTAGATATT
M00138	V$OCT1_04	0.786909	2056	(-)	NNNNNNNWATGCAAATNNNWNNW	CTTAGATATTT-GTATTATACACT
M00135	V$OCT1_01	0.794544	2058	(-)	NNNNWTATGCAAATNTNNN	TAGATATTTGTATTATACA
M00162	V$OCT1_06	0.805469	2061	(+)	CWNAWTKWSATRYN	ATATTTGTATTATA
M00162	V$OCT1_06	0.805859	2077	(+)	CWNAWTKWSATRYN	CTATCTTTGGTTTC
M00138	V$OCT1_04	0.801338	2103	(+)	NNNNNNNWATGCAAATNNNWNNW	GTAATTCTACTTAAA-TAAGAAAA
M00138	V$OCT1_04	0.790046	2104	(-)	NNNNNNNWATGCAAATNNNWNNW	TAATTCTACTTAAATAA-GAAAAT
M00138	V$OCT1_04	0.808867	2111	(+)	NNNNNNNWATGCAAATNNNWNNW	ACTTAAATAAGAAAA-TAGATTGA
M00135	V$OCT1_01	0.754674	2113	(+)	NNNNWTATGCAAATNTNNN	TTAAATAAGAAAA-TAGATT
M00162	V$OCT1_06	0.806641	2126	(+)	CWNAWTKWSATRYN	TAGATTGACAAGTA
M00162	V$OCT1_06	0.823828	2132	(-)	CWNAWTKWSATRYN	GACAAGTAATACAC
M00162	V$OCT1_06	0.829297	2194	(+)	CWNAWTKWSATRYN	CATCCTTGCCTGCC
M00138	V$OCT1_04	0.791928	2230	(+)	NNNNNNNWATGCAAATNNNWNNW	AGAGGTAAATACAGAAAAAATGT
M00138	V$OCT1_04	0.781681	2241	(+)	NNNNNNNWATGCAAATNNNWNNW	CAGAAAAAATGTTGAAA-TAAATA
M00138	V$OCT1_04	0.790255	2243	(+)	NNNNNNNWATGCAAATNNNWNNW	GAAAAAATGTTGAAA-TAAATAAG
M00138	V$OCT1_04	0.790046	2268	(-)	NNNNNNNWATGCAAATNNNWNNW	TAGTACTATCTGCC-TATGTGTAG
M00135	V$OCT1_01	0.736169	2270	(-)	NNNNWTATGCAAATNTNNN	GTACTATCTGCCTATGTGT

M00162	V$OCT1_06	0.847656	2291	(-)	CWNAWTKWSATRYN	AAAATCGCATTACC
M00137	V$OCT1_03	0.875148	2294	(-)	NNNRTAATNANNN	ATCGCATTACCAA
M00138	V$OCT1_04	0.808239	2307	(+)	NNNNNNNWATGCAAATNNNWNNW	CATTGTAAATGTATAAA-TAATGC
M00195	V$OCT1_Q6	0.816594	2313	(+)	NNNNATGCAAATNAN	AAATGTATAAATAAT
M00138	V$OCT1_04	0.798411	2318	(+)	NNNNNNNWATGCAAATNNNWNNW	TATAAA-TAATGCACAATCTCAGA
M00136	V$OCT1_02	0.783162	2323	(-)	NNGAATATKCANNNN	ATAATGCACAATCTC
M00162	V$OCT1_06	0.804297	2329	(+)	CWNAWTKWSATRYN	CACAATCTCAGATT
M00162	V$OCT1_06	0.821484	2330	(-)	CWNAWTKWSATRYN	ACAATCTCAGATTT
M00162	V$OCT1_06	0.847656	2331	(+)	CWNAWTKWSATRYN	CAATCTCAGATTTT
M00138	V$OCT1_04	0.813049	2338	(-)	NNNNNNNWATGCAAATNNNWNNW	AGATTTTTTTT-GAATGCTAAGAA
M00162	V$OCT1_06	0.834375	2342	(+)	CWNAWTKWSATRYN	TTTTTTTGAATGCT
M00138	V$OCT1_04	0.814722	2343	(+)	NNNNNNNWATGCAAATNNNWNNW	TTTTTTGAATGCTAA-GAAAGTCA
M00162	V$OCT1_06	0.842187	2356	(+)	CWNAWTKWSATRYN	AAGAAAGTCATTTA
M00162	V$OCT1_06	0.855078	2357	(-)	CWNAWTKWSATRYN	AGAAAGTCATTTAC
M00162	V$OCT1_06	0.834375	2362	(+)	CWNAWTKWSATRYN	GTCATTTACGTTCA
M00195	V$OCT1_Q6	0.833515	2362	(-)	NNNNATGCAAATNAN	GTCATTTACGTTCAT
M00059	V$YY1_01	0.790301	3	(-)	NNNNNCCATNTWNNNWN	CCTTTAAAATGCTCCCC
M00059	V$YY1_01	0.85616	47	(+)	NNNNNCCATNTWNNNWN	AGAATTCATTTTCTAAT
M00059	V$YY1_01	0.789646	159	(+)	NNNNNCCATNTWNNNWN	TCAGATCATTATCCATT
M00059	V$YY1_01	0.789974	233	(+)	NNNNNCCATNTWNNNWN	TAATTTCATTGTATCTG
M00059	V$YY1_01	0.776868	249	(+)	NNNNNCCATNTWNNNWN	GCTACTCATCTTTATTT
M00059	V$YY1_01	0.79194	517	(-)	NNNNNCCATNTWNNNWN	TTTATACTATGGGAATT
M00059	V$YY1_01	0.797837	579	(+)	NNNNNCCATNTWNNNWN	AGCTAGCATATTTAGTC
M00059	V$YY1_01	0.783421	830	(-)	NNNNNCCATNTWNNNWN	AATGAAAAATGTGATCT
M00059	V$YY1_01	0.780144	879	(-)	NNNNNCCATNTWNNNWN	GTAATGAAATGGCTTAA
M00059	V$YY1_01	0.776868	897	(+)	NNNNNCCATNTWNNNWN	AATTGGCATATAAAATG
M00059	V$YY1_01	0.813565	903	(-)	NNNNNCCATNTWNNNWN	CATATAAAATGCTAATT
M00059	V$YY1_01	0.826343	947	(-)	NNNNNCCATNTWNNNWN	CTCCCTACATGTAATGA
M00059	V$YY1_01	0.787025	948	(+)	NNNNNCCATNTWNNNWN	TCCCTACATGTAATGAA
M00059	V$YY1_01	0.802752	1021	(+)	NNNNNCCATNTWNNNWN	TATAGACATCTTGGATA
M00059	V$YY1_01	0.775885	1086	(-)	NNNNNCCATNTWNNNWN	ATTTCTTAATGTAGTTT
M00059	V$YY1_01	0.815858	1101	(-)	NNNNNCCATNTWNNNWN	TTGCAAATCTGGGATTG
M00059	V$YY1_01	0.821756	1127	(-)	NNNNNCCATNTWNNNWN	GTCAAGAGATGTTGATG

M00059	V$YY1_01	0.818807	1145	(+)	NNNNNCCATNTWNNNWN	CAAGAACATTTTTTTTT
M00059	V$YY1_01	0.824705	1188	(+)	NNNNNCCATNTWNNNWN	ACAAACCATTTAATACA
M00059	V$YY1_01	0.776868	1340	(-)	NNNNNCCATNTWNNNWN	CATAGTGTATGATCTCT
M00059	V$YY1_01	0.80308	1439	(-)	NNNNNCCATNTWNNNWN	AAGAAAACATGTGGATC
M00059	V$YY1_01	0.789318	1449	(-)	NNNNNCCATNTWNNNWN	GTGGATCAATGGAACAA
M00059	V$YY1_01	0.78637	1475	(-)	NNNNNCCATNTWNNNWN	ATGCTAAAATGAAACAG
M00059	V$YY1_01	0.779161	1523	(+)	NNNNNCCATNTWNNNWN	GACAAACATTCATGAAA
M00059	V$YY1_01	0.773263	1585	(+)	NNNNNCCATNTWNNNWN	CTTCTTCATATACATTG
M00059	V$YY1_01	0.797182	1591	(+)	NNNNNCCATNTWNNNWN	CATATACATTGTTTGAT
M00059	V$YY1_01	0.771298	1688	(-)	NNNNNCCATNTWNNNWN	AGACATATCTGGCAATA
M00069	V$YY1_02	0.774366	1855	(-)	NNNCGGCCATCTTGNCTSNW	AAACATCTG-GATGGCTGGCA
M00059	V$YY1_01	0.800131	1935	(-)	NNNNNCCATNTWNNNWN	ATGATAAAATGTCTATT
M00069	V$YY1_02	0.772046	1981	(+)	NNNCGGCCATCTTGNCTSNW	TTTTTGCCATGAGGTTT-GCA
M00059	V$YY1_01	0.833224	2023	(-)	NNNNNCCATNTWNNNWN	GATCAAACCTGGGCTCC
M00059	V$YY1_01	0.776868	2148	(+)	NNNNNCCATNTWNNNWN	TTTCCTCATCTTCCCAT
M00059	V$YY1_01	0.805701	2241	(-)	NNNNNCCATNTWNNNWN	CAGAAAAAATGTTGAAA
M00059	V$YY1_01	0.775229	2301	(+)	NNNNNCCATNTWNNNWN	TACCAACATTGTAAATG
M00059	V$YY1_01	0.83519	2358	(+)	NNNNNCCATNTWNNNWN	GAAAGTCATTTACGTTC
M00059	V$YY1_01	0.771953	2368	(+)	NNNNNCCATNTWNNNWN	TACGTTCATCCACTATC
CSN3 Bos Tarus						
M00252	V$TATA_01	0.78508	7	(+)	STATAAAWRNNNNNN	AGATAAATCTCATGA
M00216	V$TATA_C	0.848957	142	(+)	NCTATAAAAR	ACATTAAAAC
M00252	V$TATA_01	0.774676	142	(+)	STATAAAWRNNNNNN	ACATTAAAACATTCA
M00216	V$TATA_C	0.764721	150	(+)	NCTATAAAAR	ACATTCAAAG
M00216	V$TATA_C	0.774228	209	(+)	NCTATAAAAR	ATTTTAGAAG
M00216	V$TATA_C	0.792976	306	(+)	NCTATAAAAR	TCTGTATAAT
M00216	V$TATA_C	0.77766	319	(+)	NCTATAAAAR	ATGATAAAAT
M00216	V$TATA_C	0.862688	398	(+)	NCTATAAAAR	TTCTTAAAAA
M00216	V$TATA_C	0.841035	399	(+)	NCTATAAAAR	TCTTAAAAAA
M00216	V$TATA_C	0.79324	400	(+)	NCTATAAAAR	CTTAAAAAAA
M00252	V$TATA_01	0.779751	447	(+)	STATAAAWRNNNNNN	AGACAAATGTGGAGG
M00252	V$TATA_01	0.773154	467	(+)	STATAAAWRNNNNNN	CTTTTTAGGAAGATG
M00216	V$TATA_C	0.743597	517	(+)	NCTATAAAAR	ATTTCAAAAT
M00216	V$TATA_C	0.80037	564	(+)	NCTATAAAAR	AGTATACAAC

M0021		0.95590				
6	V$TATA_C	2	572	(+)	NCTATAAAAR	ACTTTAAAAA
M0021		0.76762				
6	V$TATA_C	6	573	(+)	NCTATAAAAR	CTTTAAAAAA
M0025		0.84039				
2	V$TATA_01	6	573	(+)	STATAAAWRNNNNNN	CTTTAAAAAATAACA
M0021						
6	V$TATA_C	0.77634	574	(+)	NCTATAAAAR	TTTAAAAAAT
M0021						
6	V$TATA_C	0.77766	602	(+)	NCTATAAAAR	TTCATGAAAG
M0021		0.79033				
6	V$TATA_C	5	727	(+)	NCTATAAAAR	TGCTAAAAAG
M0021		0.77422				
6	V$TATA_C	8	752	(+)	NCTATAAAAR	ATTTTACAAG
M0021		0.90863				
6	V$TATA_C	5	801	(+)	NCTATAAAAR	ATTATAAAAA
M0025		0.83912				
2	V$TATA_01	7	802	(+)	STATAAAWRNNNNNN	TTATAAAAAATTCCA
M0025		0.78203				
2	V$TATA_01	5	804	(+)	STATAAAWRNNNNNN	ATAAAAAATTCCATG
M0021		0.83390				
6	V$TATA_C	5	823	(+)	NCTATAAAAR	ACCATAGAAA
M0021		0.86691				
6	V$TATA_C	3	849	(+)	NCTATAAAAR	ATCTTAAAAG
M0021		0.77185				
6	V$TATA_C	1	920	(+)	NCTATAAAAR	AGCTTAATAA
M0021		0.77607				
6	V$TATA_C	6	921	(+)	NCTATAAAAR	GCTTAATAAG
M0021		0.83205				
6	V$TATA_C	7	953	(+)	NCTATAAAAR	ACCACAAAAA
M0025		0.77924				
2	V$TATA_01	4	954	(+)	STATAAAWRNNNNNN	CCACAAAAAAGAAAT
M0021		0.77053				
6	V$TATA_C	1	1002	(+)	NCTATAAAAR	TCAATACAAT
M0021						
6	V$TATA_C	0.7568	1013	(+)	NCTATAAAAR	GCAATCAAAT
M0021		0.75336				
6	V$TATA_C	7	1037	(+)	NCTATAAAAR	ACATTAAATA
M0025		0.79548				
2	V$TATA_01	3	1038	(+)	STATAAAWRNNNNNN	CATTAAATAAACATG
M0025		0.79802				
2	V$TATA_01	1	1078	(+)	STATAAAWRNNNNNN	TTATATATTAAATCT
M0025		0.77213				
2	V$TATA_01	9	1082	(+)	STATAAAWRNNNNNN	ATATTAAATCTATTT
M0021		0.76868				
6	V$TATA_C	2	1152	(+)	NCTATAAAAR	TCAATGAAAT
M0021		0.77660				
6	V$TATA_C	4	1323	(+)	NCTATAAAAR	TCTTTATCAA
M0025		0.77112				
2	V$TATA_01	4	1324	(+)	STATAAAWRNNNNNN	CTTTATCAAGCCTGA
M0021		0.76868				
6	V$TATA_C	2	1359	(+)	NCTATAAAAR	AGTCTATAAA
M0025		0.77569				
2	V$TATA_01	1	1360	(+)	STATAAAWRNNNNNN	GTCTATAAAACTCAG
M0021		0.96223				
6	V$TATA_C	9	1361	(+)	NCTATAAAAR	TCTATAAAAC
M0025						
2	V$TATA_01	0.88607	1362	(+)	STATAAAWRNNNNNN	CTATAAAACTCAGTT
M0021		0.87985				
6	V$TATA_C	2	1390	(+)	NCTATAAAAR	TCTATACAAG
M0025						
2	V$TATA_01	0.7932	1391	(+)	STATAAAWRNNNNNN	CTATACAAGCATGAT
M0021		0.85476				
6	V$TATA_C	6	1413	(+)	NCTATAAAAR	GCTATAACAA
M0025		0.81197				
2	V$TATA_01	7	1414	(+)	STATAAAWRNNNNNN	CTATAACAAGATGTA

M00216	V$TATA_C	0.743068	1428	(+)	NCTATAAAAR	AGTATTTAAT
M00216	V$TATA_C	0.776604	1435	(+)	NCTATAAAAR	AATTTATAAA
M00252	V$TATA_01	0.855874	1436	(+)	STATAAAWRNNNNNN	ATTTATAAATGCAAG
M00216	V$TATA_C	0.805123	1437	(+)	NCTATAAAAR	TTTATAAATG
M00252	V$TATA_01	0.874397	1438	(+)	STATAAAWRNNNNNN	TTATAAATGCAAGAT
M00216	V$TATA_C	0.883021	1461	(+)	NCTATAAAAR	ATTTTAAAAA
M00216	V$TATA_C	0.750726	1462	(+)	NCTATAAAAR	TTTTAAAAAT
M00252	V$TATA_01	0.819335	1462	(+)	STATAAAWRNNNNNN	TTTTAAAAATCTAAA
M00216	V$TATA_C	0.776604	1488	(+)	NCTATAAAAR	TCTTTCTAAA
M00216	V$TATA_C	0.801954	1490	(+)	NCTATAAAAR	TTTCTAAAAA
M00216	V$TATA_C	0.752046	1491	(+)	NCTATAAAAR	TTCTAAAAAG
M00252	V$TATA_01	0.775438	1491	(+)	STATAAAWRNNNNNN	TTCTAAAAAGACAAT
M00216	V$TATA_C	0.744653	1492	(+)	NCTATAAAAR	TCTAAAAAGA
M00216	V$TATA_C	0.750198	1510	(+)	NCTATAAAAR	CTTTTCAAAT
M00252	V$TATA_01	0.834813	1521	(+)	STATAAAWRNNNNNN	AAATAAATAGGTATT
M00216	V$TATA_C	0.842884	1553	(+)	NCTATAAAAR	TCTTTAGAAA
M00216	V$TATA_C	0.812781	1595	(+)	NCTATAAAAR	TCCAGAAAAC
M00216	V$TATA_C	0.748878	1629	(+)	NCTATAAAAR	TTCATTAAAT
M00216	V$TATA_C	0.743068	1630	(+)	NCTATAAAAR	TCATTAAATT
M00216	V$TATA_C	0.817798	1650	(+)	NCTATAAAAR	ATTATATAAC
M00252	V$TATA_01	0.798528	1651	(+)	STATAAAWRNNNNNN	TTATATAACTTATGA
M00216	V$TATA_C	0.786639	1681	(+)	NCTATAAAAR	ATTATGAAAA
M00252	V$TATA_01	0.837097	1784	(+)	STATAAAWRNNNNNN	TTATTTATAGGCAAA
M00252	V$TATA_01	0.803349	1786	(+)	STATAAAWRNNNNNN	ATTTATAGGCAAAAA
M00216	V$TATA_C	0.762873	1811	(+)	NCTATAAAAR	GCCAGATAAA
M00252	V$TATA_01	0.773408	1812	(+)	STATAAAWRNNNNNN	CCAGATAAAGGGAAA
M00252	V$TATA_01	0.811215	1814	(+)	STATAAAWRNNNNNN	AGATAAAGGGAAATA
M00216	V$TATA_C	0.772907	1934	(+)	NCTATAAAAR	GCTATCTAAT
M00252	V$TATA_01	0.786602	1955	(+)	STATAAAWRNNNNNN	GCATTTAATGCCTCA
M00216	V$TATA_C	0.74254	2018	(+)	NCTATAAAAR	CCATTCAAAT
M00252	V$TATA_01	0.802081	2020	(+)	STATAAAWRNNNNNN	ATTCAAATGCCCATG
M00252	V$TATA_01	0.815022	2074	(+)	STATAAAWRNNNNNN	CTATTAATCTCTGCA
M00216	V$TATA_C	0.745973	2116	(+)	NCTATAAAAR	CCTACAGAAA

M00216	V$TATA_C	0.757064	2228	(+)	NCTATAAAAR	TTCCTAAAAT
M00216	V$TATA_C	0.806443	2258	(+)	NCTATAAAAR	ACCTTAATAA
M00216	V$TATA_C	0.787431	2259	(+)	NCTATAAAAR	CCTTAATAAG
M00252	V$TATA_01	0.808424	2311	(+)	STATAAAWRNNNNNN	GTACATATGAAACGA
M00252	V$TATA_01	0.78305	2313	(+)	STATAAAWRNNNNNN	ACATATGAAACGAAT
M00216	V$TATA_C	0.767626	2412	(+)	NCTATAAAAR	CTTTTAATAA
M00216	V$TATA_C	0.817798	2415	(+)	NCTATAAAAR	TTAATAAAAT
M00252	V$TATA_01	0.795737	2441	(+)	STATAAAWRNNNNNN	CTATATCTCCCCCTC
M00216	V$TATA_C	0.786639	2472	(+)	NCTATAAAAR	CCCTTAAATC
M00216	V$TATA_C	0.766306	2481	(+)	NCTATAAAAR	CAAATAAAAC
M00216	V$TATA_C	0.814629	2648	(+)	NCTATAAAAR	TGTATCAAAT
M00216	V$TATA_C	0.750726	2657	(+)	NCTATAAAAR	TTTTTTAAAT
M00216	V$TATA_C	0.750726	2658	(+)	NCTATAAAAR	TTTTTAAATT
M00252	V$TATA_01	0.777468	2711	(+)	STATAAAWRNNNNNN	TTTTATAGAACAAAA
M00216	V$TATA_C	0.76921	2712	(+)	NCTATAAAAR	TTTATAGAAC
M00216	V$TATA_C	0.80169	2728	(+)	NCTATAAAAR	GCTACATAAG
M00252	V$TATA_01	0.782543	2729	(+)	STATAAAWRNNNNNN	CTACATAAGTAATTT
M00216	V$TATA_C	0.804595	2762	(+)	NCTATAAAAR	CTTATTAAAG
M00252	V$TATA_01	0.798021	2763	(+)	STATAAAWRNNNNNN	TTATTAAAGTCATAT
M00252	V$TATA_01	0.782035	2805	(+)	STATAAAWRNNNNNN	GAATAAAGAAATCAC
M00216	V$TATA_C	0.752046	2820	(+)	NCTATAAAAR	ACTTTATCAT
M00216	V$TATA_C	0.746501	2829	(+)	NCTATAAAAR	TCTAGAGAAA
M00216	V$TATA_C	0.806443	2839	(+)	NCTATAAAAR	ACCTTTAAAA
M00216	V$TATA_C	0.945075	2840	(+)	NCTATAAAAR	CCTTTAAAAT
M00252	V$TATA_01	0.790916	2841	(+)	STATAAAWRNNNNNN	CTTTAAAATTCTTAT
M00216	V$TATA_C	0.796145	2887	(+)	NCTATAAAAR	TCCTGAAAAT
M00216	V$TATA_C	0.743597	2897	(+)	NCTATAAAAR	ATTTTTAAAT
M00216	V$TATA_C	0.750726	2898	(+)	NCTATAAAAR	TTTTTAAATT
M00216	V$TATA_C	0.76921	2905	(+)	NCTATAAAAR	ATTATAATAT
M00252	V$TATA_01	0.7729	2906	(+)	STATAAAWRNNNNNN	TTATAATATGCACTT
M00216	V$TATA_C	0.802746	2916	(+)	NCTATAAAAR	CACTTAAAAT
M00216	V$TATA_C	0.766306	2928	(+)	NCTATAAAAR	TTCACAAAAA
M00252	V$TATA_01	0.793453	2936	(+)	STATAAAWRNNNNNN	AAATAAATATATAAA

M00252	V$TATA_01	0.785841	2940	(+)	STATAAAWRNNNNNN	AAATATATAAACCAT
M00216	V$TATA_C	0.802218	2941	(+)	NCTATAAAAR	AATATATAAA
M00252	V$TATA_01	0.899518	2942	(+)	STATAAAWRNNNNNN	ATATATAAACCATAG
M00216	V$TATA_C	0.918141	3025	(+)	NCTATAAAAR	TCCTTAAAAT
M00216	V$TATA_C	0.750198	3086	(+)	NCTATAAAAR	CTTTTAATAT
M00252	V$TATA_01	0.824664	3092	(+)	STATAAAWRNNNNNN	ATATTTAAGTGTATT
M00126	V$GATA1_02	0.770625	3	(+)	NNNNNGATANKGNN	AGGAAGATAAATCT
M00127	V$GATA1_03	0.890495	3	(+)	RNSNNGATAANNGN	AGGAAGATAAATCT
M00128	V$GATA1_04	0.908088	4	(+)	NNCWGATARNNNN	GGAAGATAAATCT
M00075	V$GATA1_01	0.817868	5	(+)	SNNGATNNNN	GAAGATAAAT
M00076	V$GATA2_01	0.853857	5	(+)	NNNGATRNNN	GAAGATAAAT
M00077	V$GATA3_01	0.846699	6	(+)	NNGATARNG	AAGATAAAT
M00203	V$GATA_C	0.940665	7	(+)	NGATAAGNMNN	AGATAAATCTC
M00075	V$GATA1_01	0.784798	9	(-)	SNNGATNNNN	ATAAATCTCA
M00076	V$GATA2_01	0.797925	9	(-)	NNNGATRNNN	ATAAATCTCA
M00127	V$GATA1_03	0.794463	27	(-)	RNSNNGATAANNGN	GACACTAACACCCT
M00075	V$GATA1_01	0.835637	29	(-)	SNNGATNNNN	CACTAACACC
M00076	V$GATA2_01	0.79567	29	(-)	NNNGATRNNN	CACTAACACC
M00127	V$GATA1_03	0.850318	52	(-)	RNSNNGATAANNGN	CTGGTTATTTACCT
M00075	V$GATA1_01	0.776407	116	(+)	SNNGATNNNN	GCTGACGGTC
M00127	V$GATA1_03	0.810142	130	(+)	RNSNNGATAANNGN	GGAAAGGTAATCAC
M00075	V$GATA1_01	0.811945	135	(-)	SNNGATNNNN	GGTAATCACA
M00076	V$GATA2_01	0.827244	135	(-)	NNNGATRNNN	GGTAATCACA
M00127	V$GATA1_03	0.811612	219	(-)	RNSNNGATAANNGN	AACATTTTCTTCCT
M00075	V$GATA1_01	0.791708	255	(-)	SNNGATNNNN	GAATATCCTT
M00076	V$GATA2_01	0.872801	255	(-)	NNNGATRNNN	GAATATCCTT
M00203	V$GATA_C	0.830382	259	(-)	NGATAAGNMNN	ATCCTTTGTCA
M00127	V$GATA1_03	0.795688	260	(-)	RNSNNGATAANNGN	TCCTTTGTCATGAT
M00127	V$GATA1_03	0.783684	266	(+)	RNSNNGATAANNGN	GTCATGATTATGAT
M00075	V$GATA1_01	0.870188	268	(+)	SNNGATNNNN	CATGATTATG
M00076	V$GATA2_01	0.825891	268	(+)	NNNGATRNNN	CATGATTATG
M00077	V$GATA3_01	0.876385	269	(+)	NNGATARNG	ATGATTATG
M00126	V$GATA1_02	0.803125	272	(+)	NNNNNGATANKGNN	ATTATGATATTAGT

M00128	V$GATA1_04	0.83701	273	(+)	NNCWGATARNNNN	TTATGATATTAGT
M00075	V$GATA1_01	0.804541	274	(+)	SNNGATNNNN	TATGATATTA
M00076	V$GATA2_01	0.853406	274	(+)	NNNGATRNNN	TATGATATTA
M00077	V$GATA3_01	0.843155	275	(+)	NNGATARNG	ATGATATTA
M00077	V$GATA3_01	0.86132	287	(-)	NNGATARNG	TCTAATCTA
M00076	V$GATA2_01	0.802887	293	(-)	NNNGATRNNN	CTAAATCTTA
M00077	V$GATA3_01	0.848028	293	(-)	NNGATARNG	CTAAATCTT
M00075	V$GATA1_01	0.792201	300	(+)	SNNGATNNNN	TTAGATTCTG
M00077	V$GATA3_01	0.824546	301	(+)	NNGATARNG	TAGATTCTG
M00126	V$GATA1_02	0.819375	316	(+)	NNNNNGATANKGNN	GTTATGATAAAATT
M00128	V$GATA1_04	0.875306	317	(+)	NNCWGATARNNNN	TTATGATAAAATT
M00076	V$GATA2_01	0.798376	318	(+)	NNNGATRNNN	TATGATAAAA
M00077	V$GATA3_01	0.855117	319	(+)	NNGATARNG	ATGATAAAA
M00203	V$GATA_C	0.894998	320	(+)	NGATAAGNMNN	TGATAAAATTT
M00127	V$GATA1_03	0.785154	331	(-)	RNSNNGATAANNGN	ATTTTTAACTTCAC
M00075	V$GATA1_01	0.833169	358	(+)	SNNGATNNNN	CAAGATTTGA
M00076	V$GATA2_01	0.815516	358	(+)	NNNGATRNNN	CAAGATTTGA
M00077	V$GATA3_01	0.853788	359	(+)	NNGATARNG	AAGATTTGA
M00126	V$GATA1_02	0.820625	361	(+)	NNNNNGATANKGNN	GATTTGATACTTGC
M00075	V$GATA1_01	0.825271	363	(+)	SNNGATNNNN	TTTGATACTT
M00076	V$GATA2_01	0.81281	363	(+)	NNNGATRNNN	TTTGATACTT
M00075	V$GATA1_01	0.837117	393	(+)	SNNGATNNNN	GTAGATTCTT
M00076	V$GATA2_01	0.812359	415	(-)	NNNGATRNNN	AAGTATGTTC
M00076	V$GATA2_01	0.793415	424	(+)	NNNGATRNNN	CAAGACAGTG
M00127	V$GATA1_03	0.831945	473	(+)	RNSNNGATAANNGN	AGGAAGATGAAAGT
M00075	V$GATA1_01	0.78233	475	(+)	SNNGATNNNN	GAAGATGAAA
M00076	V$GATA2_01	0.79567	475	(+)	NNNGATRNNN	GAAGATGAAA
M00077	V$GATA3_01	0.824989	476	(+)	NNGATARNG	AAGATGAAA
M00076	V$GATA2_01	0.79161	488	(+)	NNNGATRNNN	CAGCATAATC
M00075	V$GATA1_01	0.808983	491	(-)	SNNGATNNNN	CATAATCCAG
M00077	V$GATA3_01	0.830306	491	(-)	NNGATARNG	CATAATCCA
M00075	V$GATA1_01	0.884501	497	(+)	SNNGATNNNN	CCAGATTGAT
M00076	V$GATA2_01	0.843482	497	(+)	NNNGATRNNN	CCAGATTGAT

M00077	V$GATA3_01	0.835623	498	(+)	NNGATARNG	CAGATTGAT
M00127	V$GATA1_03	0.782705	541	(+)	RNSNNGATAANNGN	AAGGTTATAATTTC
M00076	V$GATA2_01	0.790257	558	(+)	NNNGATRNNN	TGTCATAGTA
M00075	V$GATA1_01	0.773939	580	(-)	SNNGATNNNN	AAATAACAGG
M00203	V$GATA_C	0.837217	587	(-)	NGATAAGNMNN	AGGACTTATAC
M00076	V$GATA2_01	0.807848	594	(-)	NNNGATRNNN	ATACATCCTT
M00075	V$GATA1_01	0.774926	604	(+)	SNNGATNNNN	CATGAAAGCT
M00075	V$GATA1_01	0.847976	639	(+)	SNNGATNNNN	CTGGATTGCC
M00076	V$GATA2_01	0.823636	639	(+)	NNNGATRNNN	CTGGATTGCC
M00126	V$GATA1_02	0.865625	687	(+)	NNNNNGATANKGNN	TAACTGATAAGAAT
M00127	V$GATA1_03	0.788339	687	(+)	RNSNNGATAANNGN	TAACTGATAAGAAT
M00128	V$GATA1_04	0.961091	688	(+)	NNCWGATARNNNN	AACTGATAAGAAT
M00075	V$GATA1_01	0.810958	689	(+)	SNNGATNNNN	ACTGATAAGA
M00076	V$GATA2_01	0.820478	689	(+)	NNNGATRNNN	ACTGATAAGA
M00077	V$GATA3_01	0.89012	690	(+)	NNGATARNG	CTGATAAGA
M00203	V$GATA_C	0.922647	691	(+)	NGATAAGNMNN	TGATAAGAATG
M00203	V$GATA_C	0.84374	700	(-)	NGATAAGNMNN	TGGTCTTATTT
M00127	V$GATA1_03	0.801813	701	(-)	RNSNNGATAANNGN	GGTCTTATTTCTCT
M00127	V$GATA1_03	0.793974	731	(+)	RNSNNGATAANNGN	AAAAAGAGAATAAC
M00127	V$GATA1_03	0.814552	734	(+)	RNSNNGATAANNGN	AAGAGAATAACTAC
M00075	V$GATA1_01	0.880059	758	(+)	SNNGATNNNN	CAAGATGTTT
M00076	V$GATA2_01	0.875056	758	(+)	NNNGATRNNN	CAAGATGTTT
M00075	V$GATA1_01	0.833169	781	(+)	SNNGATNNNN	TGTGATATCA
M00076	V$GATA2_01	0.880469	781	(+)	NNNGATRNNN	TGTGATATCA
M00128	V$GATA1_04	0.832721	781	(-)	NNCWGATARNNNN	TGTGATATCATTT
M00077	V$GATA3_01	0.844484	782	(+)	NNGATARNG	GTGATATCA
M00075	V$GATA1_01	0.79615	783	(-)	SNNGATNNNN	TGATATCATT
M00076	V$GATA2_01	0.839874	783	(-)	NNNGATRNNN	TGATATCATT
M00077	V$GATA3_01	0.827204	783	(-)	NNGATARNG	TGATATCAT
M00075	V$GATA1_01	0.844028	815	(+)	SNNGATNNNN	CATGATTCAC
M00076	V$GATA2_01	0.795219	815	(+)	NNNGATRNNN	CATGATTCAC
M00075	V$GATA1_01	0.807009	845	(-)	SNNGATNNNN	TACAATCTTA
M00076	V$GATA2_01	0.836716	845	(-)	NNNGATRNNN	TACAATCTTA

M00077	V$GATA3_01	0.893221	845	(-)	NNGATARNG	TACAATCTT
M00075	V$GATA1_01	0.818855	909	(-)	SNNGATNNNN	TGTAATCATC
M00076	V$GATA2_01	0.807397	909	(-)	NNNGATRNNN	TGTAATCATC
M00077	V$GATA3_01	0.825432	909	(-)	NNGATARNG	TGTAATCAT
M00127	V$GATA1_03	0.800833	910	(-)	RNSNNGATAANNGN	GTAATCATCAAGCT
M00075	V$GATA1_01	0.846496	912	(-)	SNNGATNNNN	AATCATCAAG
M00127	V$GATA1_03	0.821166	920	(+)	RNSNNGATAANNGN	AGCTTAATAAGAGA
M00126	V$GATA1_02	0.810625	932	(+)	NNNNNGATANKGNN	GACTAGATATTAAT
M00075	V$GATA1_01	0.80849	934	(+)	SNNGATNNNN	CTAGATATTA
M00076	V$GATA2_01	0.792061	934	(+)	NNNGATRNNN	CTAGATATTA
M00077	V$GATA3_01	0.842268	935	(+)	NNGATARNG	TAGATATTA
M00126	V$GATA1_02	0.838125	964	(+)	NNNNNGATANKGNN	GAAATGATAATTAT
M00127	V$GATA1_03	0.855218	964	(+)	RNSNNGATAANNGN	GAAATGATAATTAT
M00128	V$GATA1_04	0.885417	965	(+)	NNCWGATARNNNN	AAATGATAATTAT
M00075	V$GATA1_01	0.813425	966	(+)	SNNGATNNNN	AATGATAATT
M00076	V$GATA2_01	0.835814	966	(+)	NNNGATRNNN	AATGATAATT
M00077	V$GATA3_01	0.843155	967	(+)	NNGATARNG	ATGATAATT
M00203	V$GATA_C	0.851196	968	(+)	NGATAAGNMNN	TGATAATTATG
M00203	V$GATA_C	0.880708	994	(-)	NGATAAGNMNN	GTTAATTATCA
M00126	V$GATA1_02	0.8525	995	(-)	NNNNNGATANKGNN	TTAATTATCAATAC
M00127	V$GATA1_03	0.855218	995	(-)	RNSNNGATAANNGN	TTAATTATCAATAC
M00128	V$GATA1_04	0.825368	995	(-)	NNCWGATARNNNN	TTAATTATCAATA
M00075	V$GATA1_01	0.777394	997	(-)	SNNGATNNNN	AATTATCAAT
M00075	V$GATA1_01	0.791708	1012	(-)	SNNGATNNNN	TGCAATCAAA
M00077	V$GATA3_01	0.82942	1012	(-)	NNGATARNG	TGCAATCAA
M00075	V$GATA1_01	0.807009	1023	(+)	SNNGATNNNN	TATGATGCAC
M00076	V$GATA2_01	0.822282	1023	(+)	NNNGATRNNN	TATGATGCAC
M00075	V$GATA1_01	0.875617	1169	(-)	SNNGATNNNN	AACAATCAGT
M00076	V$GATA2_01	0.830853	1169	(-)	NNNGATRNNN	AACAATCAGT
M00077	V$GATA3_01	0.832078	1169	(-)	NNGATARNG	AACAATCAG
M00203	V$GATA_C	0.832246	1211	(-)	NGATAAGNMNN	AGTTCTTTTCC
M00126	V$GATA1_02	0.807813	1224	(+)	NNNNNGATANKGNN	TCAGTGATATTCCA
M00075	V$GATA1_01	0.845508	1226	(+)	SNNGATNNNN	AGTGATATTC

M00076	V$GATA2_01	0.85521	1226	(+)	NNNGATRNNN	AGTGATATTC
M00077	V$GATA3_01	0.828533	1227	(+)	NNGATARNG	GTGATATTC
M00075	V$GATA1_01	0.832675	1233	(-)	SNNGATNNNN	TTCCATCAGA
M00076	V$GATA2_01	0.829499	1233	(-)	NNNGATRNNN	TTCCATCAGA
M00077	V$GATA3_01	0.824103	1233	(-)	NNGATARNG	TTCCATCAG
M00128	V$GATA1_04	0.818627	1280	(-)	NNCWGATARNNNN	ATTCTTACCTGGA
M00203	V$GATA_C	0.879776	1321	(-)	NGATAAGNMNN	GCTCTTTATCA
M00126	V$GATA1_02	0.803125	1322	(-)	NNNNNGATANKGNN	CTCTTTATCAAGCC
M00127	V$GATA1_03	0.881676	1322	(-)	RNSNNGATAANNGN	CTCTTTATCAAGCC
M00128	V$GATA1_04	0.887255	1322	(-)	NNCWGATARNNNN	CTCTTTATCAAGC
M00075	V$GATA1_01	0.818361	1324	(-)	SNNGATNNNN	CTTTATCAAG
M00077	V$GATA3_01	0.865751	1324	(-)	NNGATARNG	CTTTATCAA
M00127	V$GATA1_03	0.930181	1398	(+)	RNSNNGATAANNGN	AGCATGATAACAAT
M00128	V$GATA1_04	0.897672	1399	(+)	NNCWGATARNNNN	GCATGATAACAAT
M00075	V$GATA1_01	0.846989	1400	(+)	SNNGATNNNN	CATGATAACA
M00076	V$GATA2_01	0.866486	1400	(+)	NNNGATRNNN	CATGATAACA
M00077	V$GATA3_01	0.859105	1401	(+)	NNGATARNG	ATGATAACA
M00203	V$GATA_C	0.849643	1402	(+)	NGATAAGNMNN	TGATAACAATG
M00075	V$GATA1_01	0.851925	1420	(+)	SNNGATNNNN	CAAGATGTAG
M00076	V$GATA2_01	0.847993	1420	(+)	NNNGATRNNN	CAAGATGTAG
M00077	V$GATA3_01	0.82809	1421	(+)	NNGATARNG	AAGATGTAG
M00075	V$GATA1_01	0.816881	1447	(+)	SNNGATNNNN	CAAGATTTAT
M00076	V$GATA2_01	0.798827	1447	(+)	NNNGATRNNN	CAAGATTTAT
M00075	V$GATA1_01	0.788746	1483	(+)	SNNGATNNNN	TATGATCTTT
M00076	V$GATA2_01	0.818674	1483	(+)	NNNGATRNNN	TATGATCTTT
M00128	V$GATA1_04	0.81587	1518	(+)	NNCWGATARNNNN	ATCAAATAAATAG
M00126	V$GATA1_02	0.893437	1558	(+)	NNNNNGATANKGNN	AGAAAGATAAGGTT
M00127	V$GATA1_03	0.898824	1558	(+)	RNSNNGATAANNGN	AGAAAGATAAGGTT
M00128	V$GATA1_04	0.909926	1559	(+)	NNCWGATARNNNN	GAAAGATAAGGTT
M00075	V$GATA1_01	0.816387	1560	(+)	SNNGATNNNN	AAAGATAAGG
M00076	V$GATA2_01	0.866486	1560	(+)	NNNGATRNNN	AAAGATAAGG
M00077	V$GATA3_01	0.954364	1561	(+)	NNGATARNG	AAGATAAGG
M00203	V$GATA_C	0.93352	1562	(+)	NGATAAGNMNN	AGATAAGGTTA

M00127	V$GATA1_03	0.828025	1594	(+)	RNSNNGATAANNGN	ATCCAGAAAACTGT
M00203	V$GATA_C	0.900901	1610	(-)	NGATAAGNMNN	TTGTCATATCT
M00076	V$GATA2_01	0.802436	1611	(+)	NNNGATRNNN	TGTCATATCT
M00126	V$GATA1_02	0.790937	1611	(-)	NNNNNGATANKGNN	TGTCATATCTTACA
M00128	V$GATA1_04	0.876225	1611	(-)	NNCWGATARNNNN	TGTCATATCTTAC
M00075	V$GATA1_01	0.79615	1613	(-)	SNNGATNNNN	TCATATCTTA
M00076	V$GATA2_01	0.870997	1613	(-)	NNNGATRNNN	TCATATCTTA
M00077	V$GATA3_01	0.887461	1613	(-)	NNGATARNG	TCATATCTT
M00127	V$GATA1_03	0.800833	1641	(+)	RNSNNGATAANNGN	ATCAAGCTAATTAT
M00075	V$GATA1_01	0.798124	1661	(+)	SNNGATNNNN	TATGATTACT
M00076	V$GATA2_01	0.822733	1661	(+)	NNNGATRNNN	TATGATTACT
M00127	V$GATA1_03	0.810632	1675	(+)	RNSNNGATAANNGN	AAGCAGATTATGAA
M00075	V$GATA1_01	0.865745	1677	(+)	SNNGATNNNN	GCAGATTATG
M00076	V$GATA2_01	0.850699	1677	(+)	NNNGATRNNN	GCAGATTATG
M00077	V$GATA3_01	0.894107	1678	(+)	NNGATARNG	CAGATTATG
M00075	V$GATA1_01	0.781343	1694	(-)	SNNGATNNNN	CAGTTTCAGG
M00126	V$GATA1_02	0.795312	1698	(+)	NNNNNGATANKGNN	TTCAGGATACTCCC
M00075	V$GATA1_01	0.882034	1700	(+)	SNNGATNNNN	CAGGATACTC
M00076	V$GATA2_01	0.925575	1700	(+)	NNNGATRNNN	CAGGATACTC
M00126	V$GATA1_02	0.80375	1710	(-)	NNNNNGATANKGNN	CCCCCAATCTCTTT
M00127	V$GATA1_03	0.797648	1710	(-)	RNSNNGATAANNGN	CCCCCAATCTCTTT
M00075	V$GATA1_01	0.886476	1712	(-)	SNNGATNNNN	CCCAATCTCT
M00076	V$GATA2_01	0.861525	1712	(-)	NNNGATRNNN	CCCAATCTCT
M00077	V$GATA3_01	0.966327	1712	(-)	NNGATARNG	CCCAATCTC
M00075	V$GATA1_01	0.852419	1739	(-)	SNNGATNNNN	ATCAATCACA
M00076	V$GATA2_01	0.823184	1739	(-)	NNNGATRNNN	ATCAATCACA
M00077	V$GATA3_01	0.835179	1739	(-)	NNGATARNG	ATCAATCAC
M00126	V$GATA1_02	0.8625	1810	(+)	NNNNNGATANKGNN	GGCCAGATAAAGGG
M00127	V$GATA1_03	0.927976	1810	(+)	RNSNNGATAANNGN	GGCCAGATAAAGGG
M00128	V$GATA1_04	0.958333	1811	(+)	NNCWGATARNNNN	GCCAGATAAAGGG
M00075	V$GATA1_01	0.854393	1812	(+)	SNNGATNNNN	CCAGATAAAG
M00076	V$GATA2_01	0.869644	1812	(+)	NNNGATRNNN	CCAGATAAAG
M00077	V$GATA3_01	0.907842	1813	(+)	NNGATARNG	CAGATAAAG

M00203	V$GATA_C	0.868903	1814	(+)	NGATAAGNMNN	AGATAAAGGGA
M00127	V$GATA1_03	0.814797	1837	(+)	RNSNNGATAANNGN	AAGTAGAGAATAGA
M00075	V$GATA1_01	0.898815	1854	(+)	SNNGATNNNN	CAAGATGGAG
M00076	V$GATA2_01	0.884529	1854	(+)	NNNGATRNNN	CAAGATGGAG
M00077	V$GATA3_01	0.888347	1855	(+)	NNGATARNG	AAGATGGAG
M00127	V$GATA1_03	0.815532	1858	(+)	RNSNNGATAANNGN	ATGGAGATCAGTTC
M00075	V$GATA1_01	0.846989	1860	(+)	SNNGATNNNN	GGAGATCAGT
M00075	V$GATA1_01	0.786278	1860	(-)	SNNGATNNNN	GGAGATCAGT
M00076	V$GATA2_01	0.843933	1860	(+)	NNNGATRNNN	GGAGATCAGT
M00076	V$GATA2_01	0.809653	1860	(-)	NNNGATRNNN	GGAGATCAGT
M00128	V$GATA1_04	0.816176	1898	(-)	NNCWGATARNNNN	TTTCTTACCTGAA
M00126	V$GATA1_02	0.770625	1931	(-)	NNNNNGATANKGNN	AAAGCTATCTAATT
M00128	V$GATA1_04	0.829044	1931	(-)	NNCWGATARNNNN	AAAGCTATCTAAT
M00075	V$GATA1_01	0.830207	1933	(-)	SNNGATNNNN	AGCTATCTAA
M00076	V$GATA2_01	0.86829	1933	(-)	NNNGATRNNN	AGCTATCTAA
M00077	V$GATA3_01	0.854674	1933	(-)	NNGATARNG	AGCTATCTA
M00203	V$GATA_C	0.842498	1962	(-)	NGATAAGNMNN	ATGCCTCATCT
M00127	V$GATA1_03	0.785644	1963	(-)	RNSNNGATAANNGN	TGCCTCATCTTTCT
M00075	V$GATA1_01	0.816387	1965	(-)	SNNGATNNNN	CCTCATCTTT
M00076	V$GATA2_01	0.838069	1965	(-)	NNNGATRNNN	CCTCATCTTT
M00077	V$GATA3_01	0.900753	1965	(-)	NNGATARNG	CCTCATCTT
M00203	V$GATA_C	0.838459	1986	(-)	NGATAAGNMNN	ACTTTCTATCC
M00127	V$GATA1_03	0.791769	1987	(-)	RNSNNGATAANNGN	CTTTCTATCCAACT
M00076	V$GATA2_01	0.793866	1989	(-)	NNNGATRNNN	TTCTATCCAA
M00077	V$GATA3_01	0.837395	1989	(-)	NNGATARNG	TTCTATCCA
M00076	V$GATA2_01	0.791159	2032	(+)	NNNGATRNNN	ATGGATATTC
M00077	V$GATA3_01	0.830306	2076	(-)	NNGATARNG	ATTAATCTC
M00128	V$GATA1_04	0.818627	2099	(-)	NNCWGATARNNNN	CCACCTATATGCT
M00076	V$GATA2_01	0.834912	2108	(-)	NNNGATRNNN	TGCTATGTCC
M00075	V$GATA1_01	0.771964	2132	(+)	SNNGATNNNN	ATTGATTTCT
M00203	V$GATA_C	0.830693	2136	(-)	NGATAAGNMNN	ATTTCTTAACT
M00128	V$GATA1_04	0.816176	2137	(-)	NNCWGATARNNNN	TTTCTTAACTGAA
M00203	V$GATA_C	0.837838	2150	(-)	NGATAAGNMNN	CACTTCTATCT

M00126	V$GATA1_02	0.886563	2151	(-)	NNNNNGATANKGNN	ACTTCTATCTGTAG
M00127	V$GATA1_03	0.846154	2151	(-)	RNSNNGATAANNGN	ACTTCTATCTGTAG
M00128	V$GATA1_04	0.920343	2151	(-)	NNCWGATARNNNNN	ACTTCTATCTGTA
M00075	V$GATA1_01	0.803554	2153	(-)	SNNGATNNNN	TTCTATCTGT
M00076	V$GATA2_01	0.836716	2153	(-)	NNNGATRNNN	TTCTATCTGT
M00077	V$GATA3_01	0.901196	2153	(-)	NNGATARNG	TTCTATCTG
M00203	V$GATA_C	0.883194	2190	(-)	NGATAAGNMNN	CTCTTTTATCT
M00126	V$GATA1_02	0.819063	2191	(-)	NNNNNGATANKGNN	TCTTTTATCTAACA
M00128	V$GATA1_04	0.878064	2191	(-)	NNCWGATARNNNNN	TCTTTTATCTAAC
M00077	V$GATA3_01	0.854231	2193	(-)	NNGATARNG	TTTTATCTA
M00075	V$GATA1_01	0.848963	2204	(+)	SNNGATNNNN	AGTGATTTGT
M00076	V$GATA2_01	0.800631	2204	(+)	NNNGATRNNN	AGTGATTTGT
M00075	V$GATA1_01	0.779368	2329	(+)	SNNGATNNNN	CAGGATTAAC
M00076	V$GATA2_01	0.794768	2329	(+)	NNNGATRNNN	CAGGATTAAC
M00076	V$GATA2_01	0.7871	2358	(+)	NNNGATRNNN	TAACATAACC
M00203	V$GATA_C	0.895309	2438	(-)	NGATAAGNMNN	TGGCTATATCT
M00126	V$GATA1_02	0.783438	2439	(-)	NNNNNGATANKGNN	GGCTATATCTCCCC
M00127	V$GATA1_03	0.795933	2439	(-)	RNSNNGATAANNGN	GGCTATATCTCCCC
M00128	V$GATA1_04	0.866728	2439	(-)	NNCWGATARNNNNN	GGCTATATCTCCC
M00075	V$GATA1_01	0.876111	2441	(-)	SNNGATNNNN	CTATATCTCC
M00076	V$GATA2_01	0.889941	2441	(-)	NNNGATRNNN	CTATATCTCC
M00077	V$GATA3_01	0.898981	2441	(-)	NNGATARNG	CTATATCTC
M00126	V$GATA1_02	0.840625	2509	(-)	NNNNNGATANKGNN	TAAATTATCAGCAA
M00127	V$GATA1_03	0.796178	2509	(-)	RNSNNGATAANNGN	TAAATTATCAGCAA
M00128	V$GATA1_04	0.853554	2509	(-)	NNCWGATARNNNNN	TAAATTATCAGCA
M00075	V$GATA1_01	0.898815	2511	(-)	SNNGATNNNN	AATTATCAGC
M00076	V$GATA2_01	0.890843	2511	(-)	NNNGATRNNN	AATTATCAGC
M00077	V$GATA3_01	0.837395	2511	(-)	NNGATARNG	AATTATCAG
M00075	V$GATA1_01	0.812932	2522	(+)	SNNGATNNNN	ACAGATCCTG
M00075	V$GATA1_01	0.836624	2522	(-)	SNNGATNNNN	ACAGATCCTG
M00076	V$GATA2_01	0.82995	2522	(+)	NNNGATRNNN	ACAGATCCTG
M00076	V$GATA2_01	0.85972	2522	(-)	NNNGATRNNN	ACAGATCCTG
M00127	V$GATA1_03	0.782215	2526	(+)	RNSNNGATAANNGN	ATCCTGTTAATCTA

M00075	V$GATA1_01	0.785291	2565	(+)	SNNGATNNNN	AATGATGAAG
M00076	V$GATA2_01	0.780334	2565	(+)	NNNGATRNNN	AATGATGAAG
M00077	V$GATA3_01	0.836509	2566	(+)	NNGATARNG	ATGATGAAG
M00203	V$GATA_C	0.84343	2589	(-)	NGATAAGNMNN	TGTGACTATCC
M00126	V$GATA1_02	0.783125	2590	(-)	NNNNNGATANKGNN	GTGACTATCCTGGC
M00127	V$GATA1_03	0.880206	2590	(-)	RNSNNGATAANNGN	GTGACTATCCTGGC
M00075	V$GATA1_01	0.9077	2592	(-)	SNNGATNNNN	GACTATCCTG
M00076	V$GATA2_01	0.950834	2592	(-)	NNNGATRNNN	GACTATCCTG
M00077	V$GATA3_01	0.849801	2592	(-)	NNGATARNG	GACTATCCT
M00075	V$GATA1_01	0.787266	2637	(-)	SNNGATNNNN	TCTAATCAGT
M00077	V$GATA3_01	0.856447	2637	(-)	NNGATARNG	TCTAATCAG
M00203	V$GATA_C	0.890028	2644	(-)	NGATAAGNMNN	AGTTTGTATCA
M00127	V$GATA1_03	0.784174	2719	(+)	RNSNNGATAANNGN	AACAAAATAGCTAC
M00127	V$GATA1_03	0.801323	2727	(+)	RNSNNGATAANNGN	AGCTACATAAGTAA
M00203	V$GATA_C	0.832246	2731	(+)	NGATAAGNMNN	ACATAAGTAAT
M00127	V$GATA1_03	0.789809	2750	(-)	RNSNNGATAANNGN	ACATTTATTTTTCT
M00075	V$GATA1_01	0.824778	2782	(+)	SNNGATNNNN	CTGGATATTC
M00076	V$GATA2_01	0.832657	2782	(+)	NNNGATRNNN	CTGGATATTC
M00075	V$GATA1_01	0.845015	2811	(-)	SNNGATNNNN	AGAAATCACA
M00076	V$GATA2_01	0.845737	2811	(-)	NNNGATRNNN	AGAAATCACA
M00203	V$GATA_C	0.851817	2818	(-)	NGATAAGNMNN	ACACTTTATCA
M00126	V$GATA1_02	0.839062	2819	(-)	NNNNNGATANKGNN	CACTTTATCATCTA
M00127	V$GATA1_03	0.7805	2819	(-)	RNSNNGATAANNGN	CACTTTATCATCTA
M00128	V$GATA1_04	0.897365	2819	(-)	NNCWGATARNNNN	CACTTTATCATCT
M00075	V$GATA1_01	0.849951	2821	(-)	SNNGATNNNN	CTTTATCATC
M00076	V$GATA2_01	0.855661	2821	(-)	NNNGATRNNN	CTTTATCATC
M00077	V$GATA3_01	0.89012	2821	(-)	NNGATARNG	CTTTATCAT
M00075	V$GATA1_01	0.790227	2824	(-)	SNNGATNNNN	TATCATCTAG
M00077	V$GATA3_01	0.820558	2824	(-)	NNGATARNG	TATCATCTA
M00128	V$GATA1_04	0.839767	2848	(-)	NNCWGATARNNNN	ATTCTTATATGAT
M00126	V$GATA1_02	0.822812	2853	(+)	NNNNNGATANKGNN	TATATGATAGAGAA
M00128	V$GATA1_04	0.910233	2854	(+)	NNCWGATARNNNN	ATATGATAGAGAA
M00075	V$GATA1_01	0.858835	2855	(+)	SNNGATNNNN	TATGATAGAG

M00076	V$GATA2_01	0.892648	2855	(+)	NNNGATRNNN	TATGATAGAG
M00077	V$GATA3_01	0.918476	2856	(+)	NNGATARNG	ATGATAGAG
M00203	V$GATA_C	0.894998	2857	(+)	NGATAAGNMNN	TGATAGAGAAA
M00075	V$GATA1_01	0.851925	2882	(-)	SNNGATNNNN	AAAAATCCTG
M00076	V$GATA2_01	0.853406	2882	(-)	NNNGATRNNN	AAAAATCCTG
M00127	V$GATA1_03	0.843459	2957	(+)	RNSNNGATAANNGN	AGGAAGATCAGAAA
M00075	V$GATA1_01	0.782823	2959	(+)	SNNGATNNNN	GAAGATCAGA
M00075	V$GATA1_01	0.794669	2959	(-)	SNNGATNNNN	GAAGATCAGA
M00076	V$GATA2_01	0.806044	2959	(+)	NNNGATRNNN	GAAGATCAGA
M00076	V$GATA2_01	0.823184	2959	(-)	NNNGATRNNN	GAAGATCAGA
M00203	V$GATA_C	0.830693	2961	(+)	NGATAAGNMNN	AGATCAGAAAT
M00076	V$GATA2_01	0.784844	3020	(-)	NNNGATRNNN	CTAAATCCTT
M00162	V$OCT1_06	0.924609	10	(-)	CWNAWTKWSATRYN	TAAATCTCATGACG
M00162	V$OCT1_06	0.801172	35	(+)	CWNAWTKWSATRYN	CACCCTTTAATTAG
M00248	V$OCT1_07	0.800085	49	(+)	TNTATGNTAATT	TCTCTGGTTATT
M00137	V$OCT1_03	0.909917	133	(+)	NNNRTAATNANNN	AAGGTAATCACAT
M00137	V$OCT1_03	0.847491	139	(-)	NNNRTAATNANNN	ATCACATTAAAAC
M00162	V$OCT1_06	0.842187	141	(-)	CWNAWTKWSATRYN	CACATTAAAACATT
M00138	V$OCT1_04	0.806566	144	(+)	NNNNNNNWATGCAAATNNNWNNW	ATTAAAACATTCAAAGAGAA-TAA
M00136	V$OCT1_02	0.786952	146	(+)	NNGAATATKCANNNN	TAAAACATTCAAAGA
M00136	V$OCT1_02	0.795615	156	(-)	NNGAATATKCANNNN	AAAGAGAATAATTCT
M00138	V$OCT1_04	0.787118	158	(+)	NNNNNNNWATGCAAATNNNWNNW	AGAGAATAATTCTTATTCACAGC
M00136	V$OCT1_02	0.803736	166	(+)	NNGAATATKCANNNN	ATTCTTATTCACAGC
M00162	V$OCT1_06	0.840234	180	(+)	CWNAWTKWSATRYN	CAGACTGTAATTAT
M00162	V$OCT1_06	0.862891	181	(-)	CWNAWTKWSATRYN	AGACTGTAATTATT
M00137	V$OCT1_03	0.877124	183	(+)	NNNRTAATNANNN	ACTGTAATTATTA
M00137	V$OCT1_03	0.885421	184	(-)	NNNRTAATNANNN	CTGTAATTATTAC
M00137	V$OCT1_03	0.906756	187	(-)	NNNRTAATNANNN	TAATTATTACACC
M00136	V$OCT1_02	0.921765	250	(-)	NNGAATATKCANNNN	AGCTTGAATATCCTT
M00136	V$OCT1_02	0.804277	253	(+)	NNGAATATKCANNNN	TTGAATATCCTTTGT
M00138	V$OCT1_04	0.788373	256	(-)	NNNNNNNWATGCAAATNNNWNNW	AATATCCTTTGTCATGATTATGA
M00137	V$OCT1_03	0.946266	267	(-)	NNNRTAATNANNN	TCATGATTATGAT
M00161	V$OCT1_05	0.875809	269	(+)	MKNATTTGCATAYY	ATGATTATGATATT

M00162	V$OCT1_06	0.844922	269	(+)	CWNAWTKWSATRYN	ATGATTATGATATT
M00162	V$OCT1_06	0.834766	288	(-)	CWNAWTKWSATRYN	CTAATCTAAATCTT
M00162	V$OCT1_06	0.821484	294	(-)	CWNAWTKWSATRYN	TAAATCTTAGATTC
M00162	V$OCT1_06	0.898437	295	(+)	CWNAWTKWSATRYN	AAATCTTAGATTCT
M00138	V$OCT1_04	0.822459	298	(-)	NNNNNNNWATGCAAATNNNWNNW	TCTTAGATTCTGTATAATGTTAT
M00137	V$OCT1_03	0.887791	308	(+)	NNNRTAATNANNN	TGTATAATGTTAT
M00138	V$OCT1_04	0.816813	311	(+)	NNNNNNNWATGCAAATNNNWNNW	ATAATGTTATGATAAAATTTATT
M00135	V$OCT1_01	0.742655	313	(+)	NNNNWTATGCAAATNTNNN	AATGTTATGATAAAATTTA
M00162	V$OCT1_06	0.894141	358	(+)	CWNAWTKWSATRYN	CAAGATTTGATACT
M00138	V$OCT1_04	0.825805	383	(+)	NNNNNNNWATGCAAATNNNWNNW	TGAAGACAATGTAGATTCTTAAA
M00195	V$OCT1_Q6	0.7994	387	(+)	NNNNATGCAAATNAN	GACAATGTAGATTCT
M00248	V$OCT1_07	0.797965	388	(+)	TNTATGNTAATT	ACAATGTAGATT
M00138	V$OCT1_04	0.801338	389	(+)	NNNNNNNWATGCAAATNNNWNNW	CAATGTAGAT-TCTTAAAAAAAAA
M00138	V$OCT1_04	0.784609	392	(+)	NNNNNNNWATGCAAATNNNWNNW	TGTAGAT-TCTTAAAAAAAAAAAA
M00137	V$OCT1_03	0.851442	465	(-)	NNNRTAATNANNN	AACTTTTTAGGAA
M00136	V$OCT1_02	0.789117	485	(-)	NNGAATATKCANNNN	GTACAGCATAATCCA
M00162	V$OCT1_06	0.800781	491	(-)	CWNAWTKWSATRYN	CATAATCCAGATTG
M00162	V$OCT1_06	0.860547	509	(+)	CWNAWTKWSATRYN	CTTAATTAATTTCA
M00138	V$OCT1_04	0.780636	510	(+)	NNNNNNNWATGCAAATNNNWNNW	TTAATTAATTTCAAAATTTTATT
M00138	V$OCT1_04	0.795065	510	(-)	NNNNNNNWATGCAAATNNNWNNW	TTAATTAATTTCAAAATTTTATT
M00162	V$OCT1_06	0.821484	514	(-)	CWNAWTKWSATRYN	TTAATTTCAAAATT
M00162	V$OCT1_06	0.852344	516	(-)	CWNAWTKWSATRYN	AATTTCAAAATTTT
M00248	V$OCT1_07	0.791181	516	(-)	TNTATGNTAATT	AATTTCAAAATT
M00162	V$OCT1_06	0.881641	521	(+)	CWNAWTKWSATRYN	CAAAATTTTATTTC
M00162	V$OCT1_06	0.826562	522	(+)	CWNAWTKWSATRYN	AAAATTTTATTTCT
M00162	V$OCT1_06	0.834766	522	(-)	CWNAWTKWSATRYN	AAAATTTTATTTCT
M00137	V$OCT1_03	0.862505	544	(+)	NNNRTAATNANNN	GTTATAATTTCAT
M00162	V$OCT1_06	0.876172	546	(+)	CWNAWTKWSATRYN	TATAATTTCATTTG
M00135	V$OCT1_01	0.750477	561	(+)	NNNNWTATGCAAATNTNNN	CATAGTATACAACTTTAAA
M00138	V$OCT1_04	0.815767	565	(+)	NNNNNNNWATGCAAATNNNWNNW	GTATACAACTTTAAAAAA-TAACA
M00162	V$OCT1_06	0.800781	579	(-)	CWNAWTKWSATRYN	AAAATAACAGGACT
M00138	V$OCT1_04	0.790464	603	(-)	NNNNNNNWATGCAAATNNNWNNW	TCATGAAAGCTGAATAGTTCTGT
M00136	V$OCT1_02	0.816188	609	(-)	NNGAATATKCANNNN	AAGCTGAATAGTTCT

M0016		0.82617				
2	V$OCT1_06	2	623	(-)	CWNAWTKWSATRYN	TGTTTCACAAATTT
M0013		0.83772			NNNNNNNNWATGCAAATNNN	AG-
8	V$OCT1_04	5	650	(+)	WNNW	TTATGAAGGCAAATTCAAGTA
M0013		0.77069				
5	V$OCT1_01	8	652	(+)	NNNNWTATGCAAATNTNNN	TTATGAAGGCAAATTCAAG
M0019		0.82068				
5	V$OCT1_Q6	8	654	(+)	NNNNATGCAAATNAN	ATGAAGGCAAATTCA
M0013		0.77612				
6	V$OCT1_02	3	655	(-)	NNGAATATKCANNNN	TGAAGGCAAATTCAA
M0024		0.82404				
8	V$OCT1_07	1	655	(+)	TNTATGNTAATT	TGAAGGCAAATT
M0013		0.80552			NNNNNNNNWATGCAAATNNN	
8	V$OCT1_04	1	656	(+)	WNNW	GAAGGCAAATTCAAGTACTTACT
M0013		0.85544				
6	V$OCT1_02	1	658	(+)	NNGAATATKCANNNN	AGGCAAATTCAAGTA
M0016		0.85742				
2	V$OCT1_06	2	661	(-)	CWNAWTKWSATRYN	CAAATTCAAGTACT
M0013		0.78147			NNNNNNNNWATGCAAATNNN	
8	V$OCT1_04	2	712	(+)	WNNW	TCTGGTTTCTGTTTATGCTAAAA
M0013		0.88739				
7	V$OCT1_03	6	718	(-)	NNNRTAATNANNN	TTCTGTTTATGCT
M0013		0.82371			NNNNNNNNWATGCAAATNNN	
8	V$OCT1_04	4	718	(+)	WNNW	TTCTGTTTATGCTAAAAAGAGAA
M0013		0.84147				
5	V$OCT1_01	3	720	(+)	NNNNWTATGCAAATNTNNN	CTGTTTATGCTAAAAAGAG
M0013		0.77910				
6	V$OCT1_02	1	720	(+)	NNGAATATKCANNNN	CTGTTTATGCTAAAA
M0019		0.80322				
5	V$OCT1_Q6	1	722	(+)	NNNNATGCAAATNAN	GTTTATGCTAAAAAG
M0024		0.79923				
8	V$OCT1_07	7	723	(+)	TNTATGNTAATT	TTTATGCTAAAA
M0016		0.82656				
2	V$OCT1_06	2	751	(-)	CWNAWTKWSATRYN	CATTTTACAAGATG
M0013		0.81451			NNNNNNNNWATGCAAATNNN	
8	V$OCT1_04	3	759	(-)	WNNW	AAGATGTTTTGTATGAGTGTTT
M0016		0.88281				
2	V$OCT1_06	2	776	(+)	CWNAWTKWSATRYN	GTGTTTGTGATATC
M0013		0.81974			NNNNNNNNWATGCAAATNNN	
8	V$OCT1_04	1	784	(-)	WNNW	GATATCATTTTGCATGAATTATA
M0013						
5	V$OCT1_01	0.78348	786	(-)	NNNNWTATGCAAATNTNNN	TATCATTTTGCATGAATTA
M0024		0.81916				
8	V$OCT1_07	5	790	(-)	TNTATGNTAATT	ATTTTGCATGAA
M0013						
6	V$OCT1_02	0.78614	794	(-)	NNGAATATKCANNNN	TGCATGAATTATAAA
M0013		0.75238				
5	V$OCT1_01	5	798	(+)	NNNNWTATGCAAATNTNNN	TGAATTATAAAAAATTCCA
M0013		0.74074				
5	V$OCT1_01	8	805	(-)	NNNNWTATGCAAATNTNNN	TAAAAAATTCCATGATTCA
M0016		0.81992				
2	V$OCT1_06	2	807	(+)	CWNAWTKWSATRYN	AAAAATTCCATGAT
M0016		0.88554				
2	V$OCT1_06	7	808	(-)	CWNAWTKWSATRYN	AAAATTCCATGATT
M0024		0.79584				
8	V$OCT1_07	5	809	(-)	TNTATGNTAATT	AAATTCCATGAT
M0013					NNNNNNNNWATGCAAATNNN	
8	V$OCT1_04	0.80573	813	(-)	WNNW	TCCATGATTCACCATAGAAATAT
M0024		0.80008				
8	V$OCT1_07	5	819	(-)	TNTATGNTAATT	ATTCACCATAGA
M0016		0.84218				
2	V$OCT1_06	7	847	(+)	CWNAWTKWSATRYN	CAATCTTAAAAGTT
M0013		0.81848			NNNNNNNNWATGCAAATNNN	
8	V$OCT1_04	6	858	(+)	WNNW	GTTAGCCAATGCTTATTGTACAA
M0024		0.80326				
8	V$OCT1_07	5	863	(+)	TNTATGNTAATT	CCAATGCTTATT

M00136	V$OCT1_02	0.815918	887	(+)	NNGAATATKCANNNN	TTCTATATTCACTGT
M00162	V$OCT1_06	0.800391	890	(-)	CWNAWTKWSATRYN	TATATTCACTGTAC
M00248	V$OCT1_07	0.839305	904	(+)	TNTATGNTAATT	TGTATTGTAATC
M00137	V$OCT1_03	0.887396	907	(+)	NNNRTAATNANNN	ATTGTAATCATCA
M00137	V$OCT1_03	0.851047	920	(+)	NNNRTAATNANNN	AGCTTAATAAGAG
M00162	V$OCT1_06	0.809375	922	(+)	CWNAWTKWSATRYN	CTTAATAAGAGACT
M00138	V$OCT1_04	0.807194	931	(-)	NNNNNNNWATGCAAATNNNWNNW	AGACTAGATATTAATTATTCCAA
M00248	V$OCT1_07	0.796269	935	(+)	TNTATGNTAATT	TAGATATTAATT
M00162	V$OCT1_06	0.808203	936	(+)	CWNAWTKWSATRYN	AGATATTAATTATT
M00162	V$OCT1_06	0.808203	937	(-)	CWNAWTKWSATRYN	GATATTAATTATTC
M00137	V$OCT1_03	0.879494	939	(-)	NNNRTAATNANNN	TATTAATTATTCC
M00138	V$OCT1_04	0.780636	939	(+)	NNNNNNNWATGCAAATNNNWNNW	TATTAATTATTCCAACCACAAAA
M00138	V$OCT1_04	0.844835	959	(+)	NNNNNNNWATGCAAATNNNWNNW	AAAAAGAAATGA-TAATTATGTGA
M00195	V$OCT1_Q6	0.837336	963	(+)	NNNNATGCAAATNAN	AGAAATGATAATTAT
M00161	V$OCT1_05	0.921966	964	(-)	MKNATTTGCATAYY	GAAATGATAATTAT
M00162	V$OCT1_06	0.839844	964	(-)	CWNAWTKWSATRYN	GAAATGATAATTAT
M00248	V$OCT1_07	0.829553	964	(+)	TNTATGNTAATT	GAAATGATAATT
M00137	V$OCT1_03	0.928487	967	(+)	NNNRTAATNANNN	ATGATAATTATGT
M00137	V$OCT1_03	0.888186	968	(-)	NNNRTAATNANNN	TGATAATTATGTG
M00162	V$OCT1_06	0.821094	972	(+)	CWNAWTKWSATRYN	AATTATGTGACATG
M00162	V$OCT1_06	0.893359	980	(-)	CWNAWTKWSATRYN	GACATGACAGAGTT
M00138	V$OCT1_04	0.822041	992	(-)	NNNNNNNWATGCAAATNNNWNNW	TTGTTAATTATCAATACAATTGC
M00137	V$OCT1_03	0.871592	993	(-)	NNNRTAATNANNN	TGTTAATTATCAA
M00137	V$OCT1_03	0.849072	1010	(+)	NNNRTAATNANNN	ATTGCAATCAAAT
M00162	V$OCT1_06	0.826562	1012	(-)	CWNAWTKWSATRYN	TGCAATCAAATTAT
M00162	V$OCT1_06	0.873828	1013	(-)	CWNAWTKWSATRYN	GCAATCAAATTATG
M00137	V$OCT1_03	0.889767	1016	(-)	NNNRTAATNANNN	ATCAAATTATGAT
M00162	V$OCT1_06	0.899609	1018	(+)	CWNAWTKWSATRYN	CAAATTATGATGCA
M00162	V$OCT1_06	0.834375	1035	(+)	CWNAWTKWSATRYN	ATACATTAAATAAA
M00162	V$OCT1_06	0.911328	1036	(-)	CWNAWTKWSATRYN	TACATTAAATAAAC
M00138	V$OCT1_04	0.813258	1037	(-)	NNNNNNNWATGCAAATNNNWNNW	ACATTAAATAAACATGTTAGA-CA
M00137	V$OCT1_03	0.862505	1041	(+)	NNNRTAATNANNN	TAAATAAACATGT
M00248	V$OCT1_07	0.817045	1043	(-)	TNTATGNTAATT	AATAAACATGTT

M00162	V$OCT1_06	0.829297	1047	(-)	CWNAWTKWSATRYN	AACATGTTAGACAC
M00162	V$OCT1_06	0.924609	1058	(-)	CWNAWTKWSATRYN	CACATTAAATTAAC
M00138	V$OCT1_04	0.816186	1059	(-)	NNNNNNNWATGCAAATNNNWNNW	ACATTAAATTAACACAATGTTAT
M00135	V$OCT1_01	0.784815	1061	(-)	NNNNWTATGCAAATNTNNN	ATTAAATTAACACAATGTT
M00248	V$OCT1_07	0.871105	1065	(-)	TNTATGNTAATT	AATTAACACAAT
M00162	V$OCT1_06	0.894531	1071	(+)	CWNAWTKWSATRYN	CACAATGTTATATA
M00162	V$OCT1_06	0.812109	1073	(+)	CWNAWTKWSATRYN	CAATGTTATATATT
M00248	V$OCT1_07	0.793513	1079	(+)	TNTATGNTAATT	TATATATTAAAT
M00162	V$OCT1_06	0.842187	1080	(+)	CWNAWTKWSATRYN	ATATATTAAATCTA
M00162	V$OCT1_06	0.847266	1081	(-)	CWNAWTKWSATRYN	TATATTAAATCTAT
M00137	V$OCT1_03	0.853023	1130	(+)	NNNRTAATNANNN	TTCATAAGGAGCT
M00162	V$OCT1_06	0.842578	1151	(+)	CWNAWTKWSATRYN	TTCAATGAAATTTG
M00138	V$OCT1_04	0.826851	1188	(-)	NNNNNNNWATGCAAATNNNWNNW	TGCTCAAATTTGAACTCACATTA
M00162	V$OCT1_06	0.839844	1192	(+)	CWNAWTKWSATRYN	CAAATTTGAACTCA
M00162	V$OCT1_06	0.852734	1199	(-)	CWNAWTKWSATRYN	GAACTCACATTAAG
M00162	V$OCT1_06	0.829297	1204	(-)	CWNAWTKWSATRYN	CACATTAAGTTCTT
M00162	V$OCT1_06	0.839453	1221	(+)	CWNAWTKWSATRYN	CATTCAGTGATATT
M00136	V$OCT1_02	0.82079	1226	(+)	NNGAATATKCANNNN	AGTGATATTCCATCA
M00138	V$OCT1_04	0.780008	1280	(+)	NNNNNNNWATGCAAATNNNWNNW	ATTCTTACCTGGAAATCAAAATT
M00162	V$OCT1_06	0.800781	1290	(-)	CWNAWTKWSATRYN	GGAAATCAAAATTG
M00137	V$OCT1_03	0.843935	1320	(-)	NNNRTAATNANNN	CGCTCTTTATCAA
M00162	V$OCT1_06	0.812109	1338	(-)	CWNAWTKWSATRYN	AACTTCTAACTCTG
M00162	V$OCT1_06	0.860547	1365	(-)	CWNAWTKWSATRYN	TAAAACTCAGTTTG
M00135	V$OCT1_01	0.731973	1370	(+)	NNNNWTATGCAAATNTNNN	CTCAGTTTGCAGGTTTCAA
M00138	V$OCT1_04	0.7844	1388	(-)	NNNNNNNWATGCAAATNNNWNNW	AGTCTATACAAGCATGA-TAACAA
M00138	V$OCT1_04	0.804684	1402	(+)	NNNNNNNWATGCAAATNNNWNNW	TGATAACAATGGCTATAACAA-GA
M00162	V$OCT1_06	0.829297	1427	(+)	CWNAWTKWSATRYN	TAGTATTTAATTTA
M00162	V$OCT1_06	0.9375	1428	(-)	CWNAWTKWSATRYN	AGTATTTAATTTAT
M00248	V$OCT1_07	0.793301	1428	(+)	TNTATGNTAATT	AGTATTTAATTT
M00162	V$OCT1_06	0.828906	1435	(+)	CWNAWTKWSATRYN	AATTTATAAATGCA
M00138	V$OCT1_04	0.838143	1436	(+)	NNNNNNNWATGCAAATNNNWNNW	ATTTATAAATGCAAGATTTATTA
M00135	V$OCT1_01	0.772606	1438	(+)	NNNNWTATGCAAATNTNNN	TTATAAATGCAAGATTTAT
M00162	V$OCT1_06	0.885547	1441	(-)	CWNAWTKWSATRYN	TAAATGCAAGATTT

M00138	V$OCT1_04	0.847344	1447	(+)	NNNNNNNWATGCAAATNNNWNNW	CAAGATTTATTAATATTTTAAAA
M00138	V$OCT1_04	0.806985	1448	(-)	NNNNNNNWATGCAAATNNNWNNW	AAGATTTATTAATATTTTAAAAA
M00162	V$OCT1_06	0.813281	1450	(+)	CWNAWTKWSATRYN	GATTTATTAATATT
M00138	V$OCT1_04	0.806775	1453	(+)	NNNNNNNWATGCAAATNNNWNNW	TTATTAATATTTTAAAAATCTAA
M00138	V$OCT1_04	0.801966	1454	(-)	NNNNNNNWATGCAAATNNNWNNW	TATTAATATTTTAAAAATCTAAA
M00136	V$OCT1_02	0.793449	1455	(+)	NNGAATATKCANNNN	ATTAATATTTTAAAA
M00162	V$OCT1_06	0.826562	1458	(-)	CWNAWTKWSATRYN	AATATTTTAAAAAT
M00162	V$OCT1_06	0.800391	1459	(+)	CWNAWTKWSATRYN	ATATTTTAAAAATC
M00138	V$OCT1_04	0.802175	1462	(+)	NNNNNNNWATGCAAATNNNWNNW	TTTTAAAAATCTAAAATGTTATA
M00162	V$OCT1_06	0.834375	1467	(-)	CWNAWTKWSATRYN	AAAATCTAAAATGT
M00162	V$OCT1_06	0.829297	1468	(+)	CWNAWTKWSATRYN	AAATCTAAAATGTT
M00138	V$OCT1_04	0.781681	1471	(-)	NNNNNNNWATGCAAATNNNWNNW	TCTAAAATGTTATATGATCTTTC
M00162	V$OCT1_06	0.812109	1473	(+)	CWNAWTKWSATRYN	TAAAATGTTATATG
M00138	V$OCT1_04	0.806566	1504	(+)	NNNNNNNWATGCAAATNNNWNNW	ATTTAGCTTTTCAAATCAAATAA
M00162	V$OCT1_06	0.890625	1515	(-)	CWNAWTKWSATRYN	CAAATCAAATAAAT
M00137	V$OCT1_03	0.866061	1520	(+)	NNNRTAATNANNN	CAAATAAATAGGT
M00138	V$OCT1_04	0.790255	1559	(+)	NNNNNNNWATGCAAATNNNWNNW	GAAAGATAAGGTTAATTTATTTC
M00248	V$OCT1_07	0.817045	1564	(+)	TNTATGNTAATT	ATAAGGTTAATT
M00162	V$OCT1_06	0.826562	1569	(+)	CWNAWTKWSATRYN	GTTAATTTATTTCT
M00138	V$OCT1_04	0.839189	1573	(+)	NNNNNNNWATGCAAATNNNWNNW	ATTTATTTCTTCAAATATTTCAT
M00135	V$OCT1_01	0.759252	1575	(+)	NNNNWTATGCAAATNTNNN	TTATTTCTTCAAATATTTC
M00162	V$OCT1_06	0.862891	1585	(+)	CWNAWTKWSATRYN	AAATATTTCATCCA
M00162	V$OCT1_06	0.868359	1586	(-)	CWNAWTKWSATRYN	AATATTTCATCCAG
M00162	V$OCT1_06	0.826953	1600	(+)	CWNAWTKWSATRYN	AAAACTGTAATTGT
M00162	V$OCT1_06	0.85	1601	(-)	CWNAWTKWSATRYN	AAACTGTAATTGTC
M00135	V$OCT1_01	0.751812	1604	(-)	NNNNWTATGCAAATNTNNN	CTGTAATTGTCATATCTTA
M00161	V$OCT1_05	0.873983	1606	(+)	MKNATTTGCATAYY	GTAATTGTCATATC
M00162	V$OCT1_06	0.929688	1606	(+)	CWNAWTKWSATRYN	GTAATTGTCATATC
M00195	V$OCT1_Q6	0.858352	1606	(-)	NNNNATGCAAATNAN	GTAATTGTCATATCT
M00162	V$OCT1_06	0.816016	1607	(-)	CWNAWTKWSATRYN	TAATTGTCATATCT
M00248	V$OCT1_07	0.791393	1608	(-)	TNTATGNTAATT	AATTGTCATATC
M00138	V$OCT1_04	0.806566	1611	(-)	NNNNNNNWATGCAAATNNNWNNW	TGTCATATCTTACATTTTTCAT
M00162	V$OCT1_06	0.898437	1615	(+)	CWNAWTKWSATRYN	ATATCTTACATTTT

M00162	V$OCT1_06	0.847266	1616	(-)	CWNAWTKWSATRYN	TATCTTACATTTTT
M00138	V$OCT1_04	0.812422	1619	(-)	NNNNNNNNWATGCAAATNNNWNNW	CTTACATTTTTTCATTAAATTGA
M00162	V$OCT1_06	0.860547	1623	(+)	CWNAWTKWSATRYN	CATTTTTTCATTAA
M00162	V$OCT1_06	0.847656	1629	(-)	CWNAWTKWSATRYN	TTCATTAAATTGAT
M00138	V$OCT1_04	0.797156	1636	(+)	NNNNNNNNWATGCAAATNNNWNNW	AATTGATCAAGCTAATTATATAA
M00135	V$OCT1_01	0.752385	1638	(+)	NNNNWTATGCAAATNTNNN	TTGATCAAGCTAATTATAT
M00161	V$OCT1_05	0.860203	1641	(-)	MKNATTTGCATAYY	ATCAAGCTAATTAT
M00248	V$OCT1_07	0.823829	1641	(+)	TNTATGNTAATT	ATCAAGCTAATT
M00137	V$OCT1_03	0.865271	1644	(+)	NNNRTAATNANNN	AAGCTAATTATAT
M00137	V$OCT1_03	0.879099	1645	(-)	NNNRTAATNANNN	AGCTAATTATATA
M00137	V$OCT1_03	0.911893	1660	(-)	NNNRTAATNANNN	TTATGATTACTGG
M00137	V$OCT1_03	0.875148	1676	(-)	NNNRTAATNANNN	AGCAGATTATGAA
M00135	V$OCT1_01	0.801221	1678	(+)	NNNNWTATGCAAATNTNNN	CAGATTATGAAAAATGCAG
M00136	V$OCT1_02	0.774228	1678	(+)	NNGAATATKCANNNN	CAGATTATGAAAAAT
M00162	V$OCT1_06	0.816016	1680	(+)	CWNAWTKWSATRYN	GATTATGAAAAATG
M00136	V$OCT1_02	0.795615	1685	(+)	NNGAATATKCANNNN	TGAAAAATGCAGTTT
M00136	V$OCT1_02	0.770168	1688	(-)	NNGAATATKCANNNN	AAAATGCAGTTTCAG
M00137	V$OCT1_03	0.849072	1716	(-)	NNNRTAATNANNN	ATCTCTTTAAGTA
M00136	V$OCT1_02	0.776665	1719	(-)	NNGAATATKCANNNN	TCTTTAAGTATTCCT
M00138	V$OCT1_04	0.784609	1719	(-)	NNNNNNNNWATGCAAATNNNWNNW	TCTTTAAGTATTCCTACTAGATC
M00162	V$OCT1_06	0.813672	1740	(-)	CWNAWTKWSATRYN	TCAATCACAGAATT
M00162	V$OCT1_06	0.804297	1747	(+)	CWNAWTKWSATRYN	CAGAATTAGAGAAT
M00162	V$OCT1_06	0.800781	1748	(-)	CWNAWTKWSATRYN	AGAATTAGAGAATT
M00162	V$OCT1_06	0.855078	1755	(+)	CWNAWTKWSATRYN	GAGAATTTCAGACA
M00162	V$OCT1_06	0.864844	1756	(-)	CWNAWTKWSATRYN	AGAATTTCAGACAT
M00162	V$OCT1_06	0.842578	1765	(-)	CWNAWTKWSATRYN	GACATTTCAGTTCA
M00162	V$OCT1_06	0.873828	1777	(+)	CWNAWTKWSATRYN	CAAACTTTTATTTA
M00138	V$OCT1_04	0.804893	1778	(+)	NNNNNNNNWATGCAAATNNNWNNW	AAACTTTTATTTATAGGCAAAAA
M00138	V$OCT1_04	0.799456	1784	(+)	NNNNNNNNWATGCAAATNNNWNNW	TTATTTATAGGCAAAAAGAATAC
M00135	V$OCT1_01	0.760588	1786	(+)	NNNNWTATGCAAATNTNNN	ATTTATAGGCAAAAAGAAT
M00138	V$OCT1_04	0.785655	1812	(+)	NNNNNNNNWATGCAAATNNNWNNW	CCAGATAAAGGGAAATACTTATT
M00138	V$OCT1_04	0.82936	1818	(+)	NNNNNNNNWATGCAAATNNNWNNW	AAAGGGAAA-TACTTATTCAAAGT
M00138	V$OCT1_04	0.849854	1824	(+)	NNNNNNNNWATGCAAATNNNWNNW	AAATACTTATTCAAAGTAGA-GAA

M00135	V$OCT1_01	0.77852	1826	(+)	NNNNWTATGCAAATNTNNN	ATACTTATTCAAAGTAGAG
M00136	V$OCT1_02	0.80536	1826	(+)	NNGAATATKCANNNN	ATACTTATTCAAAGT
M00195	V$OCT1_Q6	0.811954	1828	(+)	NNNNATGCAAATNAN	ACTTATTCAAAGTAG
M00162	V$OCT1_06	0.819141	1829	(-)	CWNAWTKWSATRYN	CTTATTCAAAGTAG
M00162	V$OCT1_06	0.855469	1934	(-)	CWNAWTKWSATRYN	GCTATCTAATTCTT
M00162	V$OCT1_06	0.816406	1948	(+)	CWNAWTKWSATRYN	TAGCCTGGCATTTA
M00162	V$OCT1_06	0.816406	1953	(+)	CWNAWTKWSATRYN	TGGCATTTAATGCC
M00162	V$OCT1_06	0.880469	1954	(-)	CWNAWTKWSATRYN	GGCATTTAATGCCT
M00162	V$OCT1_06	0.809766	1990	(-)	CWNAWTKWSATRYN	TCTATCCAACTTTG
M00195	V$OCT1_Q6	0.815229	2016	(+)	NNNNATGCAAATNAN	CTCCATTCAAATGCC
M00162	V$OCT1_06	0.814062	2017	(-)	CWNAWTKWSATRYN	TCCATTCAAATGCC
M00138	V$OCT1_04	0.800502	2024	(+)	NNNNNNNWATGCAAATNNNWNNW	AAATGCCCATGGATATTCCTAAG
M00136	V$OCT1_02	0.867894	2029	(-)	NNGAATATKCANNNN	CCCATGGATATTCCT
M00136	V$OCT1_02	0.848944	2032	(+)	NNGAATATKCANNNN	ATGGATATTCCTAAG
M00137	V$OCT1_03	0.84275	2039	(+)	NNNRTAATNANNN	TTCCTAAGGAGTT
M00138	V$OCT1_04	0.796738	2075	(-)	NNNNNNNWATGCAAATNNNWNNW	TATTAATCTCTGCATGGGCAAGT
M00135	V$OCT1_01	0.766501	2101	(+)	NNNNWTATGCAAATNTNNN	ACCTATATGCTATGTCCTA
M00136	V$OCT1_02	0.770439	2101	(+)	NNGAATATKCANNNN	ACCTATATGCTATGT
M00162	V$OCT1_06	0.821484	2104	(-)	CWNAWTKWSATRYN	TATATGCTATGTCC
M00248	V$OCT1_07	0.799237	2104	(+)	TNTATGNTAATT	TATATGCTATGT
M00162	V$OCT1_06	0.800391	2127	(+)	CWNAWTKWSATRYN	AATTTATTGATTTC
M00162	V$OCT1_06	0.832031	2199	(+)	CWNAWTKWSATRYN	CTAACAGTGATTTG
M00195	V$OCT1_Q6	0.85071	2205	(-)	NNNNATGCAAATNAN	GTGATTTGTGTACAT
M00162	V$OCT1_06	0.826953	2227	(+)	CWNAWTKWSATRYN	CTTCCTAAAATTTA
M00162	V$OCT1_06	0.817187	2231	(+)	CWNAWTKWSATRYN	CTAAAATTTATGTT
M00162	V$OCT1_06	0.813672	2233	(-)	CWNAWTKWSATRYN	AAAATTTATGTTAC
M00135	V$OCT1_01	0.730828	2234	(+)	NNNNWTATGCAAATNTNNN	AAATTTATGTTACTTGACC
M00248	V$OCT1_07	0.890396	2237	(+)	TNTATGNTAATT	TTTATGTTACTT
M00195	V$OCT1_Q6	0.805404	2304	(-)	NNNNATGCAAATNAN	GTTATTGGTACATAT
M00162	V$OCT1_06	0.834375	2314	(-)	CWNAWTKWSATRYN	CATATGAAACGAAT
M00162	V$OCT1_06	0.808594	2336	(+)	CWNAWTKWSATRYN	AACCTTGTAATGAC
M00137	V$OCT1_03	0.904386	2339	(+)	NNNRTAATNANNN	CTTGTAATGACTC
M00162	V$OCT1_06	0.800391	2361	(-)	CWNAWTKWSATRYN	CATAACCAAAAATT

M00162	V$OCT1_06	0.932422	2368	(+)	CWNAWTKWSATRYN	AAAAATTAAATTCA
M00138	V$OCT1_04	0.850481	2369	(+)	NNNNNNNWATGCAAATNNNWNNW	AAAATTAAATTCATATATTTCCT
M00162	V$OCT1_06	0.903906	2369	(-)	CWNAWTKWSATRYN	AAAATTAAATTCAT
M00136	V$OCT1_02	0.802112	2371	(+)	NNGAATATKCANNNN	AATTAAATTCATATA
M00162	V$OCT1_06	0.860156	2372	(+)	CWNAWTKWSATRYN	ATTAAATTCATATA
M00162	V$OCT1_06	0.813672	2374	(-)	CWNAWTKWSATRYN	TAAATTCATATATT
M00162	V$OCT1_06	0.821094	2381	(+)	CWNAWTKWSATRYN	ATATATTTCCTACC
M00138	V$OCT1_04	0.814095	2405	(-)	NNNNNNNWATGCAAATNNNWNNW	TCTAAAGCTTTTAATAAAATAGA
M00162	V$OCT1_06	0.813672	2409	(+)	CWNAWTKWSATRYN	AAGCTTTTAATAAA
M00137	V$OCT1_03	0.852232	2412	(+)	NNNRTAATNANNN	CTTTTAATAAAAT
M00162	V$OCT1_06	0.847266	2418	(+)	CWNAWTKWSATRYN	ATAAAATAGATTTT
M00162	V$OCT1_06	0.813281	2425	(+)	CWNAWTKWSATRYN	AGATTTTTCACATT
M00162	V$OCT1_06	0.808594	2455	(+)	CWNAWTKWSATRYN	CATTCTGGCTTTTC
M00162	V$OCT1_06	0.847266	2476	(-)	CWNAWTKWSATRYN	TAAATCAAATAAAA
M00162	V$OCT1_06	0.860547	2531	(+)	CWNAWTKWSATRYN	GTTAATCTAATGTT
M00162	V$OCT1_06	0.842187	2532	(-)	CWNAWTKWSATRYN	TTAATCTAATGTTT
M00138	V$OCT1_04	0.833961	2536	(-)	NNNNNNNWATGCAAATNNNWNNW	TCTAATGTTTTTAATTTAATTTT
M00162	V$OCT1_06	0.869922	2540	(+)	CWNAWTKWSATRYN	ATGTTTTTAATTTA
M00162	V$OCT1_06	0.860547	2545	(+)	CWNAWTKWSATRYN	TTTAATTTAATTTT
M00248	V$OCT1_07	0.831673	2545	(+)	TNTATGNTAATT	TTTAATTTAATT
M00137	V$OCT1_03	0.888186	2560	(+)	NNNRTAATNANNN	GGTGCAATGATGA
M00162	V$OCT1_06	0.816406	2607	(+)	CWNAWTKWSATRYN	AACCCTGCCATTTT
M00162	V$OCT1_06	0.88125	2627	(+)	CWNAWTKWSATRYN	GTTAATTTCATCTA
M00137	V$OCT1_03	0.88226	2635	(+)	NNNRTAATNANNN	CATCTAATCAGTT
M00162	V$OCT1_06	0.950391	2648	(-)	CWNAWTKWSATRYN	TGTATCAAATTTTT
M00138	V$OCT1_04	0.806775	2651	(+)	NNNNNNNWATGCAAATNNNWNNW	ATCAAATTTTTTAAATTTATTGA
M00138	V$OCT1_04	0.810958	2652	(-)	NNNNNNNWATGCAAATNNNWNNW	TCAAATTTTTTAAATTTATTGAA
M00162	V$OCT1_06	0.800391	2655	(+)	CWNAWTKWSATRYN	AATTTTTTAAATTT
M00162	V$OCT1_06	0.890625	2656	(+)	CWNAWTKWSATRYN	ATTTTTTAAATTTA
M00162	V$OCT1_06	0.834375	2662	(-)	CWNAWTKWSATRYN	TAAATTTATTGAAG
M00138	V$OCT1_04	0.83647	2678	(+)	NNNNNNNWATGCAAATNNNWNNW	CTTTAATTGTGTAAATTTCTAAA
M00135	V$OCT1_01	0.835559	2680	(+)	NNNNWTATGCAAATNTNNN	TTAATTGTGTAAATTTCTA
M00248	V$OCT1_07	0.847149	2683	(+)	TNTATGNTAATT	ATTGTGTAAATT

M00138	V$OCT1_04	0.786282	2687	(-)	NNNNNNNNWATGCAAATNNNWNNW	TGTAAATTTCTAAATACAGTTAT
M00162	V$OCT1_06	0.821094	2691	(+)	CWNAWTKWSATRYN	AATTTCTAAATACA
M00138	V$OCT1_04	0.784818	2704	(-)	NNNNNNNNWATGCAAATNNNWNNW	AGTTATATTTTATAGAACAAAAT
M00138	V$OCT1_04	0.789	2720	(-)	NNNNNNNNWATGCAAATNNNWNNW	ACAAAATAGC-TACATAAGTAATT
M00162	V$OCT1_06	0.855078	2732	(-)	CWNAWTKWSATRYN	CATAAGTAATTTAT
M00162	V$OCT1_06	0.813672	2736	(+)	CWNAWTKWSATRYN	AGTAATTTATTTTT
M00195	V$OCT1_Q6	0.803493	2737	(-)	NNNNATGCAAATNAN	GTAATTTATTTTTAC
M00138	V$OCT1_04	0.867838	2739	(-)	NNNNNNNNWATGCAAATNNNWNNW	AATTTATTTTTACATTTATTTTT
M00135	V$OCT1_01	0.758489	2741	(-)	NNNNWTATGCAAATNTNNN	TTTATTTTTACATTTATTT
M00162	V$OCT1_06	0.862891	2743	(+)	CWNAWTKWSATRYN	TATTTTTACATTTA
M00162	V$OCT1_06	0.862891	2749	(-)	CWNAWTKWSATRYN	TACATTTATTTTTC
M00138	V$OCT1_04	0.799038	2750	(-)	NNNNNNNNWATGCAAATNNNWNNW	ACATTTATTTTTCTTATTAAAGT
M00162	V$OCT1_06	0.80625	2762	(-)	CWNAWTKWSATRYN	CTTATTAAAGTCAT
M00162	V$OCT1_06	0.860156	2765	(+)	CWNAWTKWSATRYN	ATTAAAGTCATATT
M00136	V$OCT1_02	0.806714	2767	(-)	NNGAATATKCANNNN	TAAAGTCATATTCTT
M00136	V$OCT1_02	0.797509	2770	(+)	NNGAATATKCANNNN	AGTCATATTCTTCTG
M00138	V$OCT1_04	0.795901	2774	(+)	NNNNNNNNWATGCAAATNNNWNNW	ATATTCTTCTGGATATTCTTCTG
M00136	V$OCT1_02	0.86654	2779	(-)	NNGAATATKCANNNN	CTTCTGGATATTCTT
M00136	V$OCT1_02	0.822144	2782	(+)	NNGAATATKCANNNN	CTGGATATTCTTCTG
M00137	V$OCT1_03	0.86448	2804	(+)	NNNRTAATNANNN	GGAATAAAGAAAT
M00162	V$OCT1_06	0.800391	2837	(+)	CWNAWTKWSATRYN	AAACCTTTAAAATT
M00162	V$OCT1_06	0.828906	2838	(-)	CWNAWTKWSATRYN	AACCTTTAAAATTC
M00136	V$OCT1_02	0.785869	2842	(+)	NNGAATATKCANNNN	TTTAAAATTCTTATA
M00162	V$OCT1_06	0.829297	2863	(+)	CWNAWTKWSATRYN	AGAAAATACATTTG
M00138	V$OCT1_04	0.82225	2881	(+)	NNNNNNNNWATGCAAATNNNWNNW	GAAAAATCCTGAAAATATTTTTA
M00138	V$OCT1_04	0.822459	2891	(+)	NNNNNNNNWATGCAAATNNNWNNW	GAAAATATTTTTAAATTATAATA
M00138	V$OCT1_04	0.849435	2892	(-)	NNNNNNNNWATGCAAATNNNWNNW	AAAATATTTTTAAATTATAATAT
M00162	V$OCT1_06	0.839453	2896	(-)	CWNAWTKWSATRYN	TATTTTTAAATTAT
M00248	V$OCT1_07	0.807717	2896	(+)	TNTATGNTAATT	TATTTTTAAATT
M00138	V$OCT1_04	0.851736	2898	(-)	NNNNNNNNWATGCAAATNNNWNNW	TTTTTAAATTATAATATGCACTT
M00135	V$OCT1_01	0.752766	2900	(-)	NNNNWTATGCAAATNTNNN	TTTAAATTATAATATGCAC
M00162	V$OCT1_06	0.866016	2902	(-)	CWNAWTKWSATRYN	TAAATTATAATATG
M00138	V$OCT1_04	0.826014	2904	(-)	NNNNNNNNWATGCAAATNNNWNNW	AATTATAATATGCACTTAAAATA

M00248	V$OCT1_07	0.813229	2904	(-)	TNTATGNTAATT	AATTATAATATG
M00136	V$OCT1_02	0.914185	2907	(+)	NNGAATATKCANNNN	TATAATATGCACTTA
M00138	V$OCT1_04	0.817231	2909	(+)	NNNNNNNWATGCAAATNNNWNNW	TAATATGCACTTAAAATAATTCA
M00248	V$OCT1_07	0.812381	2917	(-)	TNTATGNTAATT	ACTTAAAATAAT
M00138	V$OCT1_04	0.796529	2919	(+)	NNNNNNNWATGCAAATNNNWNNW	TTAAAATAATTCACAAAAAA-TAA
M00136	V$OCT1_02	0.785328	2921	(+)	NNGAATATKCANNNN	AAAATAATTCACAAA
M00248	V$OCT1_07	0.798177	2926	(-)	TNTATGNTAATT	AATTCACAAAAA
M00138	V$OCT1_04	0.785236	2931	(-)	NNNNNNNWATGCAAATNNNWNNW	ACAAAAAATAAATATATAAAC-CA
M00162	V$OCT1_06	0.855078	2933	(+)	CWNAWTKWSATRYN	AAAAAATAAATATA
M00162	V$OCT1_06	0.800391	2934	(-)	CWNAWTKWSATRYN	AAAAATAAATATAT
M00248	V$OCT1_07	0.793513	2937	(-)	TNTATGNTAATT	AATAAATATATA
M00195	V$OCT1_Q6	0.813046	2940	(+)	NNNNATGCAAATNAN	AAATATATAAACCAT
M00138	V$OCT1_04	0.807403	2970	(+)	NNNNNNNWATGCAAATNNNWNNW	ATGTTTTTAGTTAAATTCCTATT
M00138	V$OCT1_04	0.816604	2971	(-)	NNNNNNNWATGCAAATNNNWNNW	TGTTTTTAGTTAAATTCCTATTT
M00248	V$OCT1_07	0.807717	2975	(+)	TNTATGNTAATT	TTTAGTTAAATT
M00162	V$OCT1_06	0.813672	2981	(-)	CWNAWTKWSATRYN	TAAATTCCTATTTC
M00135	V$OCT1_01	0.762304	2989	(+)	NNNNWTATGCAAATNTNNN	TATTTCATGCAAACTCCAA
M00195	V$OCT1_Q6	0.792576	2991	(+)	NNNNATGCAAATNAN	TTTCATGCAAACTCC
M00248	V$OCT1_07	0.819165	2992	(+)	TNTATGNTAATT	TTCATGCAAACT
M00162	V$OCT1_06	0.826953	2998	(-)	CWNAWTKWSATRYN	CAAACTCCAATTTG
M00162	V$OCT1_06	0.800781	3015	(-)	CWNAWTKWSATRYN	CGAAACTAAATCCT
M00138	V$OCT1_04	0.796738	3019	(+)	NNNNNNNWATGCAAATNNNWNNW	ACTAAATCCTTAAAATATTAGGA
M00162	V$OCT1_06	0.813672	3024	(+)	CWNAWTKWSATRYN	ATCCTTAAAATATT
M00137	V$OCT1_03	0.841169	3030	(-)	NNNRTAATNANNN	AAAATATTAGGAA
M00162	V$OCT1_06	0.813281	3063	(-)	CWNAWTKWSATRYN	AATTTCAAATAACT
M00162	V$OCT1_06	0.883594	3072	(+)	CWNAWTKWSATRYN	TAACTTGACATGTA
M00162	V$OCT1_06	0.841797	3073	(-)	CWNAWTKWSATRYN	AACTTGACATGTAC
M00162	V$OCT1_06	0.814453	3078	(-)	CWNAWTKWSATRYN	GACATGTACTTTTA
M00138	V$OCT1_04	0.82936	3079	(-)	NNNNNNNWATGCAAATNNNWNNW	ACATGTACTTTTAATATTTAAGT
M00161	V$OCT1_05	0.850905	3083	(+)	MKNATTTGCATAYY	GTACTTTTAATATT
M00162	V$OCT1_06	0.895703	3083	(+)	CWNAWTKWSATRYN	GTACTTTTAATATT
M00195	V$OCT1_Q6	0.81714	3083	(-)	NNNNATGCAAATNAN	GTACTTTTAATATTT
M00162	V$OCT1_06	0.836719	3084	(-)	CWNAWTKWSATRYN	TACTTTTAATATTT

M00138	V$OCT1_04	0.790464	3086	(+)	NNNNNNNWATGCAAATNNNWNNW	CTTTTAATATTTAAGTGTATTGT
M00136	V$OCT1_02	0.805901	3088	(+)	NNGAATATKCANNNN	TTTAATATTTAAGTG
M00162	V$OCT1_06	0.855078	3091	(+)	CWNAWTKWSATRYN	AATATTTAAGTGTA
M00162	V$OCT1_06	0.855469	3091	(-)	CWNAWTKWSATRYN	AATATTTAAGTGTA
M00248	V$OCT1_07	0.839305	3101	(+)	TNTATGNTAATT	TGTATTGTAACT
M00138	V$OCT1_04	0.781263	3108	(+)	NNNNNNNWATGCAAATNNNWNNW	TAACTTT
M00223	V$STAT_01	0.808419	173	(+)	TTCCCRKAA	TTCACAGCA
M00223	V$STAT_01	0.888581	212	(-)	TTCCCRKAA	TTAGAAGAA
M00223	V$STAT_01	0.851525	317	(+)	TTCCCRKAA	TTATGATAA
M00223	V$STAT_01	0.888077	317	(-)	TTCCCRKAA	TTATGATAA
M00223	V$STAT_01	0.80968	398	(+)	TTCCCRKAA	TTCTTAAAA
M00223	V$STAT_01	0.853542	469	(-)	TTCCCRKAA	TTTTAGGAA
M00223	V$STAT_01	0.843459	691	(-)	TTCCCRKAA	TGATAAGAA
M00223	V$STAT_01	0.834888	724	(+)	TTCCCRKAA	TTATGCTAA
M00223	V$STAT_01	0.819259	724	(-)	TTCCCRKAA	TTATGCTAA
M00223	V$STAT_01	0.805899	755	(-)	TTCCCRKAA	TTACAAGAT
M00223	V$STAT_01	0.792538	802	(-)	TTCCCRKAA	TTATAAAAA
M00223	V$STAT_01	0.836904	1161	(-)	TTCCCRKAA	TTTGAGGAA
M00223	V$STAT_01	0.795815	1272	(-)	TTCCCRKAA	TTCTAGTCA
M00224	V$STAT1_01	0.738515	1279	(-)	NNNSANTTCCGGGAANTGNSN	CATTCTTACCTGGAAATCAAA
M00225	V$STAT3_01	0.730196	1279	(+)	NGNNATTTCCSGGAARTGNNN	CATTCTTACCTGGAAATCAAA
M00225	V$STAT3_01	0.759594	1279	(-)	NGNNATTTCCSGGAARTGNNN	CATTCTTACCTGGAAATCAAA
M00223	V$STAT_01	0.856819	1285	(+)	TTCCCRKAA	TACCTGGAA
M00223	V$STAT_01	0.799597	1491	(-)	TTCCCRKAA	TTCTAAAAA
M00223	V$STAT_01	0.793043	1579	(+)	TTCCCRKAA	TTCTTCAAA
M00223	V$STAT_01	0.812957	1666	(+)	TTCCCRKAA	TTACTGGAG
M00223	V$STAT_01	0.819259	1682	(-)	TTCCCRKAA	TTATGAAAA
M00223	V$STAT_01	0.832619	1788	(-)	TTCCCRKAA	TTATAGGCA
M00223	V$STAT_01	0.819763	1869	(+)	TTCCCRKAA	TTCTCCAAA
M00224	V$STAT1_01	0.729623	1896	(-)	NNNSANTTCCGGGAANTGNSN	AGTTTCTTACCTGAAGCCACT
M00225	V$STAT3_01	0.713106	1896	(-)	NGNNATTTCCSGGAARTGNNN	AGTTTCTTACCTGAAGCCACT
M00223	V$STAT_01	0.853542	1902	(+)	TTCCCRKAA	TTACCTGAA
M00223	V$STAT_01	0.843711	1902	(-)	TTCCCRKAA	TTACCTGAA

M00223	V$STAT_01	0.846483	1977	(+)	TTCCCRKAA	TTGCCCTAA
M00224	V$STAT1_01	0.761403	2051	(-)	NNNSANTTCCGGGAANTGNSN	TCTCTTTTCCTTGAAGTTCCT
M00225	V$STAT3_01	0.72614	2051	(+)	NGNNATTTCCSGGAARTGNNN	TCTCTTTTCCTTGAAGTTCCT
M00225	V$STAT3_01	0.738306	2051	(-)	NGNNATTTCCSGGAARTGNNN	TCTCTTTTCCTTGAAGTTCCT
M00223	V$STAT_01	0.870683	2057	(+)	TTCCCRKAA	TTCCTTGAA
M00223	V$STAT_01	0.853542	2228	(+)	TTCCCRKAA	TTCCTAAAA
M00223	V$STAT_01	0.791782	2387	(+)	TTCCCRKAA	TTCCTACCA
M00223	V$STAT_01	0.833375	2513	(+)	TTCCCRKAA	TTATCAGCA
M00223	V$STAT_01	0.795059	2596	(+)	TTCCCRKAA	ATCCTGGCA
M00223	V$STAT_01	0.819763	2633	(+)	TTCCCRKAA	TTCATCTAA
M00223	V$STAT_01	0.790522	2760	(+)	TTCCCRKAA	TTCTTATTA
M00223	V$STAT_01	0.839677	2799	(-)	TTCCCRKAA	CTCTAGGAA
M00223	V$STAT_01	0.801865	2875	(-)	TTCCCRKAA	TGGGGGGAA
M00223	V$STAT_01	0.815982	2953	(-)	TTCCCRKAA	ATAGAGGAA
M00224	V$STAT1_01	0.726494	2986	(-)	NNNSANTTCCGGGAANTGNSN	TCCTATTTCATGCAAACTCCA
M00225	V$STAT3_01	0.712962	2986	(-)	NGNNATTTCCSGGAARTGNNN	TCCTATTTCATGCAAACTCCA
M00059	V$YY1_01	0.819135	32	(+)	NNNNNCCATNTWNNNWN	TAACACCCTTTAATTAG
M00059	V$YY1_01	0.803408	215	(+)	NNNNNCCATNTWNNNWN	GAAGAACATTTTCTTCC
M00059	V$YY1_01	0.791612	471	(-)	NNNNNCCATNTWNNNWN	TTAGGAAGATGAAAGTA
M00059	V$YY1_01	0.800459	548	(+)	NNNNNCCATNTWNNNWN	TAATTTCATTTGTCATA
M00059	V$YY1_01	0.80865	691	(-)	NNNNNCCATNTWNNNWN	TGATAAGAATGGTCTTA
M00059	V$YY1_01	0.905636	745	(+)	NNNNNCCATNTWNNNWN	TACTACCATTTTACAAG
M00059	V$YY1_01	0.814875	754	(-)	NNNNNCCATNTWNNNWN	TTTACAAGATGTTTTTG
M00059	V$YY1_01	0.866645	783	(+)	NNNNNCCATNTWNNNWN	TGATATCATTTTGCATG
M00059	V$YY1_01	0.78637	809	(+)	NNNNNCCATNTWNNNWN	AAATTCCATGATTCACC
M00059	V$YY1_01	0.779489	959	(-)	NNNNNCCATNTWNNNWN	AAAAAGAAATGATAATT
M00059	V$YY1_01	0.78211	1138	(+)	NNNNNCCATNTWNNNWN	GAGCTCCATTCTATTCA
M00059	V$YY1_01	0.798165	1215	(+)	NNNNNCCATNTWNNNWN	CTTTTCCATTCAGTGAT
M00059	V$YY1_01	0.770315	1248	(+)	NNNNNCCATNTWNNNWN	TGAAGTCAGTTTAGTAG
M00059	V$YY1_01	0.833879	1280	(-)	NNNNNCCATNTWNNNWN	ATTCTTACCTGGAAATC
M00059	V$YY1_01	0.777851	1393	(-)	NNNNNCCATNTWNNNWN	ATACAAGCATGATAACA
M00059	V$YY1_01	0.780144	1402	(-)	NNNNNCCATNTWNNNWN	TGATAACAATGGCTATA
M00059	V$YY1_01	0.830931	1476	(-)	NNNNNCCATNTWNNNWN	AATGTTATATGATCTTT

M0005 9	V$YY1_01	0.84600 3	1683	(-)	NNNNNCCATNTWNNNWN	TATGAAAAATGCAGTTT
M0005 9	V$YY1_01	0.77195 3	1807	(+)	NNNNNCCATNTWNNNWN	TAGGGCCAGATAAAGGG
M0005 9	V$YY1_01	0.90760 2	1850	(-)	NNNNNCCATNTWNNNWN	AGCCCAAGATGGAGATC
M0005 9	V$YY1_01	0.77195 3	1860	(+)	NNNNNCCATNTWNNNWN	GGAGATCAGTTCTCCAA
M0005 9	V$YY1_01	0.77752 3	1889	(+)	NNNNNCCATNTWNNNWN	TCACCTCAGTTTCTTAC
M0005 9	V$YY1_01	0.77391 9	2013	(+)	NNNNNCCATNTWNNNWN	TCCCTCCATTCAAATGC
M0005 9	V$YY1_01	0.78833 6	2137	(-)	NNNNNCCATNTWNNNWN	TTTCTTAACTGAACACT
M0005 9	V$YY1_01	0.80308	2374	(+)	NNNNNCCATNTWNNNWN	TAAATTCATATATTTCC
M0005 9	V$YY1_01	0.78342 1	2388	(+)	NNNNNCCATNTWNNNWN	TCCTACCATTTGGTTTT
M0005 9	V$YY1_01	0.80275 2	2561	(-)	NNNNNCCATNTWNNNWN	GTGCAATGATGAAGAGT
M0006 9	V$YY1_02	0.83059 6	2608	(+)	NNNCGGCCATCTTGNCTSNW	ACCCTGCCATTTTTGGTGAG
M0005 9	V$YY1_01	0.89056 4	2609	(+)	NNNNNCCATNTWNNNWN	CCCTGCCATTTTTGGTG
M0005 9	V$YY1_01	0.82830 9	2629	(+)	NNNNNCCATNTWNNNWN	TAATTTCATCTAATCAG
M0005 9	V$YY1_01	0.80701 2	2637	(+)	NNNNNCCATNTWNNNWN	TCTAATCAGTTTGTATC
M0005 9	V$YY1_01	0.77785 1	2767	(+)	NNNNNCCATNTWNNNWN	TAAAGTCATATTCTTCT
M0005 9	V$YY1_01	0.77522 9	2821	(+)	NNNNNCCATNTWNNNWN	CTTTATCATCTAGAGAA
M0005 9	V$YY1_01	0.77522 9	2848	(-)	NNNNNCCATNTWNNNWN	ATTCTTATATGATAGAG
M0005 9	V$YY1_01	0.84633	2905	(-)	NNNNNCCATNTWNNNWN	ATTATAATATGCACTTA
M0005 9	V$YY1_01	0.79816 5	2946	(+)	NNNNNCCATNTWNNNWN	ATAAACCATAGAGGAAG
M0005 9	V$YY1_01	0.79456 1	2949	(-)	NNNNNCCATNTWNNNWN	AACCATAGAGGAAGATC
M0005 9	V$YY1_01	0.82241 2	2962	(-)	NNNNNCCATNTWNNNWN	GATCAGAAATGTTTTTA
M0005 9	V$YY1_01	0.78505 9	2981	(+)	NNNNNCCATNTWNNNWN	TAAATTCCTATTTCATG

CSN1S1 *Ovis Aries*

M00252	V$TATA_01	0.77366 2	53	(+)	STATAAAWRNNNNNN	TTATGTATACATATA
M00252	V$TATA_01	0.78787 1	57	(+)	STATAAAWRNNNNNN	GTATACATATAAAAA
M00252	V$TATA_01	0.77848 3	59	(+)	STATAAAWRNNNNNN	ATACATATAAAAAAA
M00216	V$TATA_C	0.78188 5	60	(+)	NCTATAAAAR	TACATATAAA
M00252	V$TATA_01	0.83608 2	61	(+)	STATAAAWRNNNNNN	ACATATAAAAAAACT
M00216	V$TATA_C	0.87325 1	62	(+)	NCTATAAAAR	CATATAAAAA
M00252	V$TATA_01	0.87668 1	63	(+)	STATAAAWRNNNNNN	ATATAAAAAAACTTA
M00216	V$TATA_C	0.75178 2	64	(+)	NCTATAAAAR	TATAAAAAAA
M00252	V$TATA_01	0.77010 9	65	(+)	STATAAAWRNNNNNN	ATAAAAAAACTTATT
M00216	V$TATA_C	0.89384 7	177	(+)	NCTATAAAAR	AGCTTAAAAA

M00216	V$TATA_C	0.811724	178	(+)	NCTATAAAAR	GCTTAAAAAT
M00252	V$TATA_01	0.801319	178	(+)	STATAAAWRNNNNNN	GCTTAAAAATATATT
M00252	V$TATA_01	0.775945	184	(+)	STATAAAWRNNNNNN	AAATATATTTGCAAA
M00252	V$TATA_01	0.818066	186	(+)	STATAAAWRNNNNNN	ATATATTTGCAAATG
M00216	V$TATA_C	0.825191	243	(+)	NCTATAAAAR	CTTTTATAAA
M00252	V$TATA_01	0.821619	244	(+)	STATAAAWRNNNNNN	TTTTATAAAGACCAA
M00216	V$TATA_C	0.793768	245	(+)	NCTATAAAAR	TTTATAAAGA
M00252	V$TATA_01	0.871352	246	(+)	STATAAAWRNNNNNN	TTATAAAGACCAAAT
M00216	V$TATA_C	0.761025	270	(+)	NCTATAAAAR	ATTTTTAAAA
M00216	V$TATA_C	0.872722	271	(+)	NCTATAAAAR	TTTTTAAAAT
M00216	V$TATA_C	0.857407	362	(+)	NCTATAAAAR	TCTGTAAAAT
M00216	V$TATA_C	0.759176	387	(+)	NCTATAAAAR	AATTTATAAT
M00216	V$TATA_C	0.757328	453	(+)	NCTATAAAAR	TCCATGTAAT
M00216	V$TATA_C	0.817798	467	(+)	NCTATAAAAR	TTAATAAAAT
M00216	V$TATA_C	0.81859	479	(+)	NCTATAAAAR	ATTTTATAAA
M00252	V$TATA_01	0.84268	480	(+)	STATAAAWRNNNNNN	TTTTATAAAGGAAAC
M00216	V$TATA_C	0.805123	481	(+)	NCTATAAAAR	TTTATAAAGG
M00252	V$TATA_01	0.825679	482	(+)	STATAAAWRNNNNNN	TTATAAAGGAAACAC
M00252	V$TATA_01	0.793707	512	(+)	STATAAAWRNNNNNN	AGATATAAATGATTA
M00216	V$TATA_C	0.751254	513	(+)	NCTATAAAAR	GATATAAATG
M00252	V$TATA_01	0.861964	514	(+)	STATAAAWRNNNNNN	ATATAAATGATTACT
M00216	V$TATA_C	0.825191	527	(+)	NCTATAAAAR	CTTTTATAAA
M00252	V$TATA_01	0.805126	528	(+)	STATAAAWRNNNNNN	TTTTATAAAGATGAT
M00216	V$TATA_C	0.793768	529	(+)	NCTATAAAAR	TTTATAAAGA
M00252	V$TATA_01	0.821619	530	(+)	STATAAAWRNNNNNN	TTATAAAGATGATTA
M00252	V$TATA_01	0.836336	552	(+)	STATAAAWRNNNNNN	ATATTTATAAGGATA
M00216	V$TATA_C	0.795088	553	(+)	NCTATAAAAR	TATTTATAAG
M00252	V$TATA_01	0.845724	554	(+)	STATAAAWRNNNNNN	ATTTATAAGGATACA
M00252	V$TATA_01	0.777721	556	(+)	STATAAAWRNNNNNN	TTATAAGGATACAAA
M00252	V$TATA_01	0.770109	564	(+)	STATAAAWRNNNNNN	ATACAAATATATGAA
M00252	V$TATA_01	0.794976	568	(+)	STATAAAWRNNNNNN	AAATATATGAAAATA
M00252	V$TATA_01	0.800812	570	(+)	STATAAAWRNNNNNN	ATATATGAAAATAAT
M00216	V$TATA_C	0.751782	571	(+)	NCTATAAAAR	TATATGAAAA

M00216	V$TATA_C	0.824663	597	(+)	NCTATAAAAR	GGCTTATAAA
M00252	V$TATA_01	0.781274	598	(+)	STATAAAWRNNNNNN	GCTTATAAATATGTC
M00216	V$TATA_C	0.79324	599	(+)	NCTATAAAAR	CTTATAAATA
M00252	V$TATA_01	0.855874	600	(+)	STATAAAWRNNNNNN	TTATAAATATGTCTT
M00216	V$TATA_C	0.768154	614	(+)	NCTATAAAAR	TTTTTAACAA
M00252	V$TATA_01	0.777468	642	(+)	STATAAAWRNNNNNN	GTTTGTAAACTGAAG
M00216	V$TATA_C	0.809876	660	(+)	NCTATAAAAR	CCTGTATAAA
M00252	V$TATA_01	0.7729	661	(+)	STATAAAWRNNNNNN	CTGTATAAAATAATC
M00216	V$TATA_C	0.936625	662	(+)	NCTATAAAAR	TGTATAAAAT
M00252	V$TATA_01	0.877442	663	(+)	STATAAAWRNNNNNN	GTATAAAATAATCTG
M00216	V$TATA_C	0.820174	697	(+)	NCTATAAAAR	GGTATAAACA
M00252	V$TATA_01	0.793707	698	(+)	STATAAAWRNNNNNN	GTATAAACAAATTTC
M00252	V$TATA_01	0.773408	732	(+)	STATAAAWRNNNNNN	TTATTTATCTGAAAT
M00216	V$TATA_C	0.781357	759	(+)	NCTATAAAAR	CACATATAAA
M00252	V$TATA_01	0.813753	760	(+)	STATAAAWRNNNNNN	ACATATAAATTTATG
M00252	V$TATA_01	0.82847	762	(+)	STATAAAWRNNNNNN	ATATAAATTTATGTA
M00252	V$TATA_01	0.770363	790	(+)	STATAAAWRNNNNNN	CAATTTAATGGAACT
M00252	V$TATA_01	0.790916	871	(+)	STATAAAWRNNNNNN	GGATATATCAACCAA
M00252	V$TATA_01	0.83253	873	(+)	STATAAAWRNNNNNN	ATATATCAACCAAGC
M00216	V$TATA_C	0.749142	908	(+)	NCTATAAAAR	TCCTTTTAAA
M00216	V$TATA_C	0.840507	909	(+)	NCTATAAAAR	CCTTTTAAAA
M00216	V$TATA_C	0.889622	910	(+)	NCTATAAAAR	CTTTTAAAAA
M00216	V$TATA_C	0.750726	911	(+)	NCTATAAAAR	TTTTAAAAAT
M00252	V$TATA_01	0.784065	911	(+)	STATAAAWRNNNNNN	TTTTAAAAATTTAAT
M00216	V$TATA_C	0.78479	940	(+)	NCTATAAAAR	TCTACATAAT
M00252	V$TATA_01	0.793961	941	(+)	STATAAAWRNNNNNN	CTACATAATGCACAA
M00216	V$TATA_C	0.757064	974	(+)	NCTATAAAAR	ACTCTGAAAG
M00252	V$TATA_01	0.81223	995	(+)	STATAAAWRNNNNNN	GGATAAAGACATCTT
M00216	V$TATA_C	0.841035	1006	(+)	NCTATAAAAR	TCTTTTAAAA
M00216	V$TATA_C	0.889622	1007	(+)	NCTATAAAAR	CTTTTAAAAA
M00252	V$TATA_01	0.772393	1007	(+)	STATAAAWRNNNNNN	CTTTTAAAAATTACA
M00216	V$TATA_C	0.750726	1008	(+)	NCTATAAAAR	TTTTAAAAAT
M00252	V$TATA_01	0.7932	1008	(+)	STATAAAWRNNNNNN	TTTTAAAAATTACAG
M00216	V$TATA_C	0.742276	1020	(+)	NCTATAAAAR	CAGATAAAAT

M00252	V$TATA_01	0.804872	1021	(+)	STATAAAWRNNNNNN	AGATAAAATTAAATG
M00216	V$TATA_C	0.761025	1060	(+)	NCTATAAAAR	ATTTTAACAA
M00216	V$TATA_C	0.865593	1114	(+)	NCTATAAAAR	ATTTTAAAAT
M00252	V$TATA_01	0.797513	1129	(+)	STATAAAWRNNNNNN	ATATTTAAATATTGA
M00252	V$TATA_01	0.817052	1131	(+)	STATAAAWRNNNNNN	ATTTAAATATTGATA
M00216	V$TATA_C	0.740164	1234	(+)	NCTATAAAAR	CTTAGATAAG
M00252	V$TATA_01	0.812992	1271	(+)	STATAAAWRNNNNNN	ATATTTATATTGAAG
M00252	V$TATA_01	0.801319	1273	(+)	STATAAAWRNNNNNN	ATTTATATTGAAGAG
M00216	V$TATA_C	0.873515	1305	(+)	NCTATAAAAR	TGAATAAAAA
M00252	V$TATA_01	0.805379	1306	(+)	STATAAAWRNNNNNN	GAATAAAAATATTAC
M00216	V$TATA_C	0.816477	1319	(+)	NCTATAAAAR	ACTTTCAAAT
M00216	V$TATA_C	0.741748	1341	(+)	NCTATAAAAR	ATCATAATAT
M00216	V$TATA_C	0.756536	1397	(+)	NCTATAAAAR	TTATTATAAG
M00216	V$TATA_C	0.760232	1399	(+)	NCTATAAAAR	ATTATAAGAC
M00216	V$TATA_C	0.805651	1439	(+)	NCTATAAAAR	ACCATGAAAC
M00216	V$TATA_C	0.787167	1499	(+)	NCTATAAAAR	ACAGTAAAAA
M00216	V$TATA_C	0.807499	1501	(+)	NCTATAAAAR	AGTAAAAAAT
M00216	V$TATA_C	0.744125	1618	(+)	NCTATAAAAR	GCTATTAAGG
M00252	V$TATA_01	0.803096	1619	(+)	STATAAAWRNNNNNN	CTATTAAGGTGTTGA
M00216	V$TATA_C	0.751254	1632	(+)	NCTATAAAAR	GATATTAAAG
M00252	V$TATA_01	0.818066	1633	(+)	STATAAAWRNNNNNN	ATATTAAAGCTATAG
M00216	V$TATA_C	0.745709	1696	(+)	NCTATAAAAR	ACTCTAAATA
M00216	V$TATA_C	0.802218	1698	(+)	NCTATAAAAR	TCTAAATAAA
M00216	V$TATA_C	0.775812	1700	(+)	NCTATAAAAR	TAAATAAAAT
M00216	V$TATA_C	0.796145	1714	(+)	NCTATAAAAR	AACTTAAAAT
M00252	V$TATA_01	0.828216	1720	(+)	STATAAAWRNNNNNN	AAATATAAACACCTC
M00216	V$TATA_C	0.744653	1721	(+)	NCTATAAAAR	AATATAAACA
M00252	V$TATA_01	0.812992	1722	(+)	STATAAAWRNNNNNN	ATATAAACACCTCAC
M00216	V$TATA_C	0.900977	1735	(+)	NCTATAAAAR	ACAATAAAAA
M00252	V$TATA_01	0.800558	1736	(+)	STATAAAWRNNNNNN	CAATAAAAAATTAAA
M00216	V$TATA_C	0.760496	1743	(+)	NCTATAAAAR	AAATTAAAAA
M00216	V$TATA_C	0.760496	1750	(+)	NCTATAAAAR	AAATTAAAAA
M00216	V$TATA_C	0.797993	1752	(+)	NCTATAAAAR	ATTAAAAAAG

M00216	V$TATA_C	0.763401	1776	(+)	NCTATAAAAR	ACAATACAAT
M00216	V$TATA_C	0.778981	1781	(+)	NCTATAAAAR	ACAATAAAGA
M00252	V$TATA_01	0.813245	1782	(+)	STATAAAWRNNNNNN	CAATAAAGAGCATCA
M00216	V$TATA_C	0.80882	1790	(+)	NCTATAAAAR	AGCATCAAAG
M00216	V$TATA_C	0.74967	1834	(+)	NCTATAAAAR	GGCTTAACAT
M00252	V$TATA_01	0.799543	1852	(+)	STATAAAWRNNNNNN	ACATATATATGTTCA
M00252	V$TATA_01	0.855874	1854	(+)	STATAAAWRNNNNNN	ATATATATGTTCAAT
M00216	V$TATA_C	0.787167	2017	(+)	NCTATAAAAR	TGCATGAAAT
M00252	V$TATA_01	0.815275	2169	(+)	STATAAAWRNNNNNN	CTATTAAGCCCTATA
M00252	V$TATA_01	0.772393	2313	(+)	STATAAAWRNNNNNN	GTAGAAAGAAAAACT
M00216	V$TATA_C	0.77766	2325	(+)	NCTATAAAAR	ACTAGATAAT
M00216	V$TATA_C	0.740692	2354	(+)	NCTATAAAAR	TTCTCAAAAA
M00216	V$TATA_C	0.752839	2355	(+)	NCTATAAAAR	TCTCAAAAAA
M00252	V$TATA_01	0.820604	2374	(+)	STATAAAWRNNNNNN	TTATTAATGTGAAAC
M00216	V$TATA_C	0.78479	2416	(+)	NCTATAAAAR	TCTAGATAAT
M00252	V$TATA_01	0.785334	2425	(+)	STATAAAWRNNNNNN	TGATAAATAAATAAA
M00252	V$TATA_01	0.828724	2429	(+)	STATAAAWRNNNNNN	AAATAAATAAATAGG
M00252	V$TATA_01	0.848769	2433	(+)	STATAAAWRNNNNNN	AAATAAATAGGAATC
M00252	V$TATA_01	0.777721	2486	(+)	STATAAAWRNNNNNN	GGATATTTGGACACT
M00216	V$TATA_C	0.837338	2504	(+)	NCTATAAAAR	GCTATCAAAT
M00252	V$TATA_01	0.782543	2510	(+)	STATAAAWRNNNNNN	AAATATAGATGCAAA
M00252	V$TATA_01	0.798782	2512	(+)	STATAAAWRNNNNNN	ATATAGATGCAAAAG
M00216	V$TATA_C	0.797465	2644	(+)	NCTATAAAAR	AGCATCAAAA
M00216	V$TATA_C	0.759968	2645	(+)	NCTATAAAAR	GCATCAAAAG
M00252	V$TATA_01	0.778229	2645	(+)	STATAAAWRNNNNNN	GCATCAAAAGAAGGG
M00216	V$TATA_C	0.757328	2678	(+)	NCTATAAAAR	ACGAAAAAAG
M00252	V$TATA_01	0.794976	2752	(+)	STATAAAWRNNNNNN	AGATATAGATCCCCC
M00252	V$TATA_01	0.779751	2754	(+)	STATAAAWRNNNNNN	ATATAGATCCCCCAG
M00252	V$TATA_01	0.77087	2831	(+)	STATAAAWRNNNNNN	ACATAAATCTTGTTC
M00252	V$TATA_01	0.771632	2851	(+)	STATAAAWRNNNNNN	GTTAAAATGCAGATT
M00216	V$TATA_C	0.833905	2876	(+)	NCTATAAAAR	TTTATATAAT
M00252	V$TATA_01	0.807156	2877	(+)	STATAAAWRNNNNNN	TTATATAATTTAATT
M00216	V$TATA_C	0.795088	2893	(+)	NCTATAAAAR	ATGATAAAAA

M00252	V$TATA_01	0.781528	2894	(+)	STATAAAWRNNNNNN	TGATAAAAAATAAAA
M00216	V$TATA_C	0.768682	2900	(+)	NCTATAAAAR	AAAATAAAAT
M00252	V$TATA_01	0.806648	2901	(+)	STATAAAWRNNNNNN	AAATAAAATCCAGAC
M00216	V$TATA_C	0.747557	2963	(+)	NCTATAAAAR	ACTCTAGAAA
M00216	V$TATA_C	0.744653	2965	(+)	NCTATAAAAR	TCTAGAAACA
M00216	V$TATA_C	0.768682	3004	(+)	NCTATAAAAR	TCAATAAATT
M00216	V$TATA_C	0.740164	3052	(+)	NCTATAAAAR	CTCTTAAATA
M00252	V$TATA_01	0.787871	3053	(+)	STATAAAWRNNNNNN	TCTTAAATACAAACT
M00216	V$TATA_C	0.740692	3067	(+)	NCTATAAAAR	TTCTTAAACA
M00216	V$TATA_C	0.842091	3079	(+)	NCTATAAAAR	ACTATTAAAT
M00252	V$TATA_01	0.786856	3080	(+)	STATAAAWRNNNNNN	CTATTAAATGTATAA
M00216	V$TATA_C	0.814629	3088	(+)	NCTATAAAAR	TGTATAATAT
M00252	V$TATA_01	0.818574	3089	(+)	STATAAAWRNNNNNN	GTATAATATTACATG
M00216	V$TATA_C	0.779509	3167	(+)	NCTATAAAAR	TTTTTAACAG
M00216	V$TATA_C	0.842091	3241	(+)	NCTATAAAAR	ACTATAAATT
M00252	V$TATA_01	0.864755	3242	(+)	STATAAAWRNNNNNN	CTATAAATTGTGTAG
M00252	V$TATA_01	0.787871	3266	(+)	STATAAAWRNNNNNN	TGATAAATTGCTAAT
M00252	V$TATA_01	0.778229	3277	(+)	STATAAAWRNNNNNN	TAATATATAGATTGT
M00252	V$TATA_01	0.814768	3279	(+)	STATAAAWRNNNNNN	ATATATAGATTGTAG
M00216	V$TATA_C	0.754687	3308	(+)	NCTATAAAAR	TCTCTAGAAA
M00216	V$TATA_C	0.840243	3310	(+)	NCTATAAAAR	TCTAGAAAAC
M00252	V$TATA_01	0.786095	3311	(+)	STATAAAWRNNNNNN	CTAGAAAACAGCCAG
M00216	V$TATA_C	0.802746	3341	(+)	NCTATAAAAR	GGTATAATAT
M00252	V$TATA_01	0.803603	3421	(+)	STATAAAWRNNNNNN	GTATTTATGATTAAT
M00216	V$TATA_C	0.835226	3431	(+)	NCTATAAAAR	TTAATAAAAA
M00252	V$TATA_01	0.780259	3476	(+)	STATAAAWRNNNNNN	ATATGTATATGTGGT
M00252	V$TATA_01	0.8102	3480	(+)	STATAAAWRNNNNNN	GTATATGTGGTCTAT
M00216	V$TATA_C	0.807499	3573	(+)	NCTATAAAAR	AGTATTAAAT
M00252	V$TATA_01	0.837351	3574	(+)	STATAAAWRNNNNNN	GTATTAAATTAGATA
M00216	V$TATA_C	0.780829	3601	(+)	NCTATAAAAR	CTTTTACAAG
M00252	V$TATA_01	0.807156	3732	(+)	STATAAAWRNNNNNN	TGATAAATACTGAAT
M00252	V$TATA_01	0.794468	3904	(+)	STATAAAWRNNNNNN	TGATAAAGAAGGAAC
M00216	V$TATA_C	0.865593	3940	(+)	NCTATAAAAR	ATTTTAAAAT

M00252	V$TATA_01	0.771885	3940	(+)	STATAAAWRNNNNNN	ATTTTAAAATAAAAT
M00252	V$TATA_01	0.790409	3941	(+)	STATAAAWRNNNNNN	TTTTAAAATAAAATC
M00216	V$TATA_C	0.768682	3945	(+)	NCTATAAAAR	AAAATAAAAT
M00252	V$TATA_01	0.790916	3946	(+)	STATAAAWRNNNNNN	AAATAAAATCATAAA
M00216	V$TATA_C	0.854766	3953	(+)	NCTATAAAAR	ATCATAAAAC
M00252	V$TATA_01	0.808424	3954	(+)	STATAAAWRNNNNNN	TCATAAAACTGAAAC
M00216	V$TATA_C	0.80169	4008	(+)	NCTATAAAAR	CCTAAATAAA
M00252	V$TATA_01	0.819843	4009	(+)	STATAAAWRNNNNNN	CTAAATAAATGGAGA
M00252	V$TATA_01	0.839381	4011	(+)	STATAAAWRNNNNNN	AAATAAATGGAGAAT
M00252	V$TATA_01	0.783811	4030	(+)	STATAAAWRNNNNNN	GTTCAAATGGAAAAA
M00216	V$TATA_C	0.752046	4073	(+)	NCTATAAAAR	CTTTTACAAT
M00216	V$TATA_C	0.78611	4110	(+)	NCTATAAAAR	TCAATGAAAA
M00216	V$TATA_C	0.752839	4183	(+)	NCTATAAAAR	TCTGGAAAAA
M00252	V$TATA_01	0.787617	4205	(+)	STATAAAWRNNNNNN	TTATTTATAAATATT
M00216	V$TATA_C	0.783734	4206	(+)	NCTATAAAAR	TATTTATAAA
M00252	V$TATA_01	0.785587	4207	(+)	STATAAAWRNNNNNN	ATTTATAAATATTTT
M00216	V$TATA_C	0.793768	4208	(+)	NCTATAAAAR	TTTATAAATA
M00252	V$TATA_01	0.808678	4209	(+)	STATAAAWRNNNNNN	TTATAAATATTTTTT
M00252	V$TATA_01	0.878204	4282	(+)	STATAAAWRNNNNNN	TTATATATGCAAATA
M00216	V$TATA_C	0.744653	4293	(+)	NCTATAAAAR	AATATTAAAA
M00216	V$TATA_C	0.802482	4294	(+)	NCTATAAAAR	ATATTAAAAA
M00252	V$TATA_01	0.848008	4294	(+)	STATAAAWRNNNNNN	ATATTAAAAAGCTGC
M00216	V$TATA_C	0.761553	4306	(+)	NCTATAAAAR	TGCTTTAAAT
M00216	V$TATA_C	0.829152	4307	(+)	NCTATAAAAR	GCTTTAAATA
M00252	V$TATA_01	0.829231	4308	(+)	STATAAAWRNNNNNN	CTTTAAATATTAATC
M00252	V$TATA_01	0.811723	4314	(+)	STATAAAWRNNNNNN	ATATTAATCTGTACC
M00216	V$TATA_C	0.776604	4341	(+)	NCTATAAAAR	TCTTTGTAAA
M00216	V$TATA_C	0.775548	4343	(+)	NCTATAAAAR	TTTGTAAAAC
M00216	V$TATA_C	0.802218	4375	(+)	NCTATAAAAR	TCTATGTAAA
M00216	V$TATA_C	0.801162	4384	(+)	NCTATAAAAR	ATTTTATAAT
M00216	V$TATA_C	0.760496	4516	(+)	NCTATAAAAR	AGTATTTAAA
M00252	V$TATA_01	0.830754	4517	(+)	STATAAAWRNNNNNN	GTATTTAAAAGATTT
M00216	V$TATA_C	0.859519	4518	(+)	NCTATAAAAR	TATTTAAAAG

M00252	V$TATA_01	0.797006	4519	(+)	STATAAAWRNNNNNN	ATTTAAAAGATTTGC
M00216	V$TATA_C	0.750726	4560	(+)	NCTATAAAAR	TTTTTAATAT
M00216	V$TATA_C	0.753367	4563	(+)	NCTATAAAAR	TTAATATAAT
M00216	V$TATA_C	0.808292	4601	(+)	NCTATAAAAR	TGTTTAGAAA
M00252	V$TATA_01	0.780005	4602	(+)	STATAAAWRNNNNNN	GTTTAGAAAGGTAAG
M00216	V$TATA_C	0.750726	4824	(+)	NCTATAAAAR	TTTTTTAAAT
M00216	V$TATA_C	0.768154	4825	(+)	NCTATAAAAR	TTTTTAAATA
M00252	V$TATA_01	0.792692	4834	(+)	STATAAAWRNNNNNN	ATTTTTAAGTGCACA
M00252	V$TATA_01	0.79117	4836	(+)	STATAAAWRNNNNNN	TTTTAAGTGCACAGT
M00216	V$TATA_C	0.771851	4863	(+)	NCTATAAAAR	AGCTTAAATA
M00252	V$TATA_01	0.808171	4864	(+)	STATAAAWRNNNNNN	GCTTAAATAAAGAAA
M00252	V$TATA_01	0.772139	4866	(+)	STATAAAWRNNNNNN	TTAAATAAAGAAAAC
M00216	V$TATA_C	0.766306	4930	(+)	NCTATAAAAR	TTCACAAAAA
M00252	V$TATA_01	0.782796	5067	(+)	STATAAAWRNNNNNN	ACATAAATGATATTT
M00252	V$TATA_01	0.77087	5076	(+)	STATAAAWRNNNNNN	ATATTTAAATTATTT
M00216	V$TATA_C	0.743597	5087	(+)	NCTATAAAAR	ATTTTAAAGT
M00216	V$TATA_C	0.789015	5098	(+)	NCTATAAAAR	ACCTCAAAAT
M00252	V$TATA_01	0.794722	5149	(+)	STATAAAWRNNNNNN	ATTTTTAAGGGAAAA
M00216	V$TATA_C	0.824927	5237	(+)	NCTATAAAAR	AGTACAAAAA
M00252	V$TATA_01	0.817052	5238	(+)	STATAAAWRNNNNNN	GTACAAAAAACATTT
M00216	V$TATA_C	0.762873	5249	(+)	NCTATAAAAR	ATTTTAGAAA
M00216	V$TATA_C	0.793768	5251	(+)	NCTATAAAAR	TTTAGAAAAA
M00216	V$TATA_C	0.744389	5254	(+)	NCTATAAAAR	AGAAAAAAAA
M00216	V$TATA_C	0.833905	5292	(+)	NCTATAAAAR	ACTTTCAAAA
M00216	V$TATA_C	0.750198	5293	(+)	NCTATAAAAR	CTTTCAAAAT
M00216	V$TATA_C	0.836282	5380	(+)	NCTATAAAAR	AGTATAAATG
M00252	V$TATA_01	0.885562	5381	(+)	STATAAAWRNNNNNN	GTATAAATGAATGAA
M00216	V$TATA_C	0.851862	5396	(+)	NCTATAAAAR	TGGATAAAAG
M00252	V$TATA_01	0.825425	5397	(+)	STATAAAWRNNNNNN	GGATAAAAGAATGAA
M00216	V$TATA_C	0.795088	5480	(+)	NCTATAAAAR	ACTACATAAA
M00252	V$TATA_01	0.849277	5481	(+)	STATAAAWRNNNNNN	CTACATAAAGCAATA
M00252	V$TATA_01	0.789901	5491	(+)	STATAAAWRNNNNNN	CAATATATCTGCTCC
M00216	V$TATA_C	0.779509	5540	(+)	NCTATAAAAR	TTTTTCAAAG

M00216	V$TATA_C	0.767626	5549	(+)	NCTATAAAAR	GTTTTAATAG
M00252	V$TATA_01	0.809693	5549	(+)	STATAAAWRNNNNNN	GTTTTAATAGCTTTG
M00216	V$TATA_C	0.781885	5563	(+)	NCTATAAAAR	GTTATTAAAA
M00216	V$TATA_C	0.809612	5564	(+)	NCTATAAAAR	TTATTAAAAA
M00252	V$TATA_01	0.825171	5564	(+)	STATAAAWRNNNNNN	TTATTAAAAAAAACA
M00216	V$TATA_C	0.786639	5566	(+)	NCTATAAAAR	ATTAAAAAAA
M00216	V$TATA_C	0.755743	5583	(+)	NCTATAAAAR	GGTATTTAAA
M00252	V$TATA_01	0.828216	5584	(+)	STATAAAWRNNNNNN	GTATTTAAAAAACAT
M00216	V$TATA_C	0.848165	5585	(+)	NCTATAAAAR	TATTTAAAAA
M00216	V$TATA_C	0.761025	5586	(+)	NCTATAAAAR	ATTTAAAAAA
M00252	V$TATA_01	0.822126	5586	(+)	STATAAAWRNNNNNN	ATTTAAAAAACATAC
M00216	V$TATA_C	0.767362	5587	(+)	NCTATAAAAR	TTTAAAAAAC
M00216	V$TATA_C	0.755743	5602	(+)	NCTATAAAAR	GGTATTTAAA
M00252	V$TATA_01	0.810454	5603	(+)	STATAAAWRNNNNNN	GTATTTAAAAATTTA
M00216	V$TATA_C	0.848165	5604	(+)	NCTATAAAAR	TATTTAAAAA
M00216	V$TATA_C	0.743597	5605	(+)	NCTATAAAAR	ATTTAAAAAT
M00252	V$TATA_01	0.81223	5605	(+)	STATAAAWRNNNNNN	ATTTAAAAATTTACA
M00216	V$TATA_C	0.856615	5702	(+)	NCTATAAAAR	ATTTTAAAAC
M00216	V$TATA_C	0.842091	5710	(+)	NCTATAAAAR	ACTATTAAAT
M00252	V$TATA_01	0.797006	5711	(+)	STATAAAWRNNNNNN	CTATTAAATATAATA
M00216	V$TATA_C	0.746237	5720	(+)	NCTATAAAAR	ATAATATAAT
M00252	V$TATA_01	0.786602	5721	(+)	STATAAAWRNNNNNN	TAATATAATGAAAAG
M00216	V$TATA_C	0.807499	5741	(+)	NCTATAAAAR	AGTAAAAAAT
M00216	V$TATA_C	0.748878	5814	(+)	NCTATAAAAR	ACTAAAACAG
M00216	V$TATA_C	0.755743	5839	(+)	NCTATAAAAR	AGAAAAAAAG
M00216	V$TATA_C	0.754423	5906	(+)	NCTATAAAAR	AGCTTAAATT
M00216	V$TATA_C	0.778981	5996	(+)	NCTATAAAAR	TGCTTAAATA
M00252	V$TATA_01	0.792946	6003	(+)	STATAAAWRNNNNNN	ATATTTATACATTAT
M00216	V$TATA_C	0.755743	6099	(+)	NCTATAAAAR	AGAAAAAAAG
M00216	V$TATA_C	0.793768	6151	(+)	NCTATAAAAR	TTTATCAAAA
M00216	V$TATA_C	0.774756	6159	(+)	NCTATAAAAR	AACATATAAA
M00252	V$TATA_01	0.805633	6160	(+)	STATAAAWRNNNNNN	ACATATAAAAATCTA
M00216	V$TATA_C	0.873251	6161	(+)	NCTATAAAAR	CATATAAAAA

M00252	V$TATA_01	0.905608	6162	(+)	STATAAAWRNNNNNN	ATATAAAAATCTACA
M00252	V$TATA_01	0.784572	6203	(+)	STATAAAWRNNNNNN	AGATTAAAGAGGAAA
M00252	V$TATA_01	0.777468	6204	(+)	STATAAAWRNNNNNN	GATTAAAGAGGAAAA
M00216	V$TATA_C	0.768154	6245	(+)	NCTATAAAAR	TTTTTTAAAA
M00216	V$TATA_C	0.872722	6246	(+)	NCTATAAAAR	TTTTTAAAAT
M00252	V$TATA_01	0.813245	6326	(+)	STATAAAWRNNNNNN	TTTTATATTGGAATG
M00216	V$TATA_C	0.754423	6344	(+)	NCTATAAAAR	GTCATGAAAA
M00216	V$TATA_C	0.760496	6345	(+)	NCTATAAAAR	TCATGAAAAA
M00216	V$TATA_C	0.761025	6418	(+)	NCTATAAAAR	ATTTTAATAA
M00252	V$TATA_01	0.805633	6437	(+)	STATAAAWRNNNNNN	TTATATATCATAAAC
M00216	V$TATA_C	0.759176	6443	(+)	NCTATAAAAR	ATCATAAACA
M00216	V$TATA_C	0.829152	6466	(+)	NCTATAAAAR	GCTTTAAGAA
M00252	V$TATA_01	0.77696	6467	(+)	STATAAAWRNNNNNN	CTTTAAGAAAGCTAT
M00216	V$TATA_C	0.762873	6547	(+)	NCTATAAAAR	GCCAGATAAA
M00216	V$TATA_C	0.776604	6604	(+)	NCTATAAAAR	TCTTTATTAA
M00216	V$TATA_C	0.793768	6606	(+)	NCTATAAAAR	TTTATTAAAA
M00216	V$TATA_C	0.792184	6607	(+)	NCTATAAAAR	TTATTAAAAT
M00252	V$TATA_01	0.845217	6607	(+)	STATAAAWRNNNNNN	TTATTAAAATCAAGA
M00216	V$TATA_C	0.752575	6619	(+)	NCTATAAAAR	AGAGTAAAAA
M00216	V$TATA_C	0.928439	6645	(+)	NCTATAAAAR	TGTTTAAAAA
M00252	V$TATA_01	0.87998	6646	(+)	STATAAAWRNNNNNN	GTTTAAAAATGAACA
M00216	V$TATA_C	0.745709	6665	(+)	NCTATAAAAR	ACTCAAAAAA
M00216	V$TATA_C	0.74835	6666	(+)	NCTATAAAAR	CTCAAAAAAT
M00252	V$TATA_01	0.790155	6672	(+)	STATAAAWRNNNNNN	AAATTAAAACCCTGT
M00252	V$TATA_01	0.779244	6700	(+)	STATAAAWRNNNNNN	GGATAAGAGTTCACT
M00216	V$TATA_C	0.753367	7250	(+)	NCTATAAAAR	ACATGAAAAA
M00216	V$TATA_C	0.751518	7316	(+)	NCTATAAAAR	TGCATATTAG
M00252	V$TATA_01	0.78102	7319	(+)	STATAAAWRNNNNNN	ATATTAGAATCAATA
M00252	V$TATA_01	0.778483	7366	(+)	STATAAAWRNNNNNN	CCATATGTAAATATG
M00252	V$TATA_01	0.791423	7370	(+)	STATAAAWRNNNNNN	ATGTAAATATGAACA
M00252	V$TATA_01	0.797767	7372	(+)	STATAAAWRNNNNNN	GTAAATATGAACATC
M00216	V$TATA_C	0.817798	7410	(+)	NCTATAAAAR	TGTTTCAAAG
M00216	V$TATA_C	0.833905	7426	(+)	NCTATAAAAR	ACTTTTAAAA

M00216	V$TATA_C	0.900977	7427	(+)	NCTATAAAAR	CTTTTAAAAG
M00252	V$TATA_01	0.805379	7427	(+)	STATAAAWRNNNNNN	CTTTTAAAAGCTCAT
M00252	V$TATA_01	0.847754	7428	(+)	STATAAAWRNNNNNN	TTTTAAAAGCTCATG
M00216	V$TATA_C	0.801954	7526	(+)	NCTATAAAAR	AGAATATAAA
M00252	V$TATA_01	0.842172	7527	(+)	STATAAAWRNNNNNN	GAATATAAACTCAAC
M00252	V$TATA_01	0.784319	7529	(+)	STATAAAWRNNNNNN	ATATAAACTCAACCC
M00216	V$TATA_C	0.756271	7649	(+)	NCTATAAAAR	GTTTTAATAA
M00252	V$TATA_01	0.823395	7649	(+)	STATAAAWRNNNNNN	GTTTTAATAAAAAGG
M00216	V$TATA_C	0.835226	7652	(+)	NCTATAAAAR	TTAATAAAAA
M00252	V$TATA_01	0.798528	7653	(+)	STATAAAWRNNNNNN	TAATAAAAAGGTTTT
M00216	V$TATA_C	0.761025	7707	(+)	NCTATAAAAR	ATTTTAAATA
M00252	V$TATA_01	0.797513	7708	(+)	STATAAAWRNNNNNN	TTTTAAATAAAATTG
M00216	V$TATA_C	0.775812	7711	(+)	NCTATAAAAR	TAAATAAAAT
M00252	V$TATA_01	0.772647	7712	(+)	STATAAAWRNNNNNN	AAATAAAATTGACAA
M00216	V$TATA_C	0.839187	7727	(+)	NCTATAAAAR	TCCAAAAAAA
M00216	V$TATA_C	0.785582	7728	(+)	NCTATAAAAR	CCAAAAAAAA
M00252	V$TATA_01	0.816037	7760	(+)	STATAAAWRNNNNNN	CTTTTTATACCCAAT
M00216	V$TATA_C	0.794296	7780	(+)	NCTATAAAAR	TCACTAAAAA
M00216	V$TATA_C	0.836282	7804	(+)	NCTATAAAAR	AGTATGAAAG
M00252	V$TATA_01	0.828724	7805	(+)	STATAAAWRNNNNNN	GTATGAAAGTGTGGA
M00216	V$TATA_C	0.806179	7834	(+)	NCTATAAAAR	ATTCTAAAAG
M00216	V$TATA_C	0.76921	7945	(+)	NCTATAAAAR	ATTAGAAAAT
M00216	V$TATA_C	0.742012	7954	(+)	NCTATAAAAR	TGTTTATGAA
M00252	V$TATA_01	0.771885	7955	(+)	STATAAAWRNNNNNN	GTTTATGAAAGTTTG
M00216	V$TATA_C	0.805123	7956	(+)	NCTATAAAAR	TTTATGAAAG
M00216	V$TATA_C	0.814629	7974	(+)	NCTATAAAAR	ACCATAAAGT
M00216	V$TATA_C	0.750198	8021	(+)	NCTATAAAAR	TGTATCTAAT
M00216	V$TATA_C	0.743597	8039	(+)	NCTATAAAAR	ATTTTAACAT
M00252	V$TATA_01	0.801066	8250	(+)	STATAAAWRNNNNNN	AAATATAAACATGAT
M00216	V$TATA_C	0.744653	8251	(+)	NCTATAAAAR	AATATAAACA
M00252	V$TATA_01	0.776706	8252	(+)	STATAAAWRNNNNNN	ATATAAACATGATAT
M00252	V$TATA_01	0.780766	8336	(+)	STATAAAWRNNNNNN	GGAGAAAAAGAGAAG
M00216	V$TATA_C	0.79456	8350	(+)	NCTATAAAAR	GGCATACAAA

M00252	V$TATA_01	0.780766	8351	(+)	STATAAAWRNNNNNN	GCATACAAAGTGATG
M00216	V$TATA_C	0.796145	8369	(+)	NCTATAAAAR	TCCTTAAACT
M00216	V$TATA_C	0.801162	8399	(+)	NCTATAAAAR	AGTTTACAAA
M00216	V$TATA_C	0.77634	8401	(+)	NCTATAAAAR	TTTACAAAAT
M00216	V$TATA_C	0.745445	8431	(+)	NCTATAAAAR	CAGTTAAAAG
M00252	V$TATA_01	0.778483	8432	(+)	STATAAAWRNNNNNN	AGTTAAAAGTGAATT
M00216	V$TATA_C	0.850013	8631	(+)	NCTATAAAAR	GTCATAAAAC
M00252	V$TATA_01	0.776453	8632	(+)	STATAAAWRNNNNNN	TCATAAAACTAACAA
M00252	V$TATA_01	0.771885	8657	(+)	STATAAAWRNNNNNN	TTTTTTAAGGAAATT
M00216	V$TATA_C	0.823607	8707	(+)	NCTATAAAAR	AATTTAAAAT
M00252	V$TATA_01	0.775945	8713	(+)	STATAAAWRNNNNNN	AAATATATTAACATC
M00252	V$TATA_01	0.78305	8715	(+)	STATAAAWRNNNNNN	ATATATTAACATCAT
M00216	V$TATA_C	0.753367	8787	(+)	NCTATAAAAR	ACATTCAAAA
M00216	V$TATA_C	0.793768	8841	(+)	NCTATAAAAR	TTTATTAAAA
M00216	V$TATA_C	0.820966	8842	(+)	NCTATAAAAR	TTATTAAAAG
M00252	V$TATA_01	0.807156	8842	(+)	STATAAAWRNNNNNN	TTATTAAAAGTTAAT
M00216	V$TATA_C	0.742012	8894	(+)	NCTATAAAAR	TGTTGATAAA
M00216	V$TATA_C	0.796145	8977	(+)	NCTATAAAAR	TCCTGAAAAT
M00216	V$TATA_C	0.759704	8982	(+)	NCTATAAAAR	AAAATAAAAC
M00252	V$TATA_01	0.779498	8983	(+)	STATAAAWRNNNNNN	AAATAAAACTGACAA
M00252	V$TATA_01	0.771378	9013	(+)	STATAAAWRNNNNNN	ATATTTAATTCATAC
M00252	V$TATA_01	0.780005	9046	(+)	STATAAAWRNNNNNN	GTATTATAGTCTACT
M00216	V$TATA_C	0.750198	9181	(+)	NCTATAAAAR	AATTTATAAC
M00252	V$TATA_01	0.786602	9182	(+)	STATAAAWRNNNNNN	ATTTATAACTTAAGT
M00252	V$TATA_01	0.836336	9220	(+)	STATAAAWRNNNNNN	ATATTTAAGCATATA
M00216	V$TATA_C	0.85503	9227	(+)	NCTATAAAAR	AGCATATAAA
M00252	V$TATA_01	0.892921	9228	(+)	STATAAAWRNNNNNN	GCATATAAAGCAATT
M00252	V$TATA_01	0.785587	9230	(+)	STATAAAWRNNNNNN	ATATAAAGCAATTAA
M00216	V$TATA_C	0.84658	9241	(+)	NCTATAAAAR	TTAATAAAAG
M00252	V$TATA_01	0.811723	9242	(+)	STATAAAWRNNNNNN	TAATAAAAGCAAGAC
M00252	V$TATA_01	0.78305	9594	(+)	STATAAAWRNNNNNN	TGATATAATGCACAC
M00216	V$TATA_C	0.797465	9861	(+)	NCTATAAAAR	TCAACAAAAG
M00252	V$TATA_01	0.788632	9921	(+)	STATAAAWRNNNNNN	TTATTTATATCTGAC

M00252	V$TATA_01	0.79726	9923	(+)	STATAAAWRNNNNNN	ATTTATATCTGACTG
M00216	V$TATA_C	0.788751	9937	(+)	NCTATAAAAR	GCATTATAAT
M00216	V$TATA_C	0.76921	9939	(+)	NCTATAAAAR	ATTATAATAT
M00216	V$TATA_C	0.799313	9955	(+)	NCTATAAAAR	AGTTAAAAAA
M00216	V$TATA_C	0.781885	9956	(+)	NCTATAAAAR	GTTAAAAAAA
M00216	V$TATA_C	0.768154	9992	(+)	NCTATAAAAR	TTTTTTAAAA
M00216	V$TATA_C	0.890151	9993	(+)	NCTATAAAAR	TTTTTAAAAA
M00216	V$TATA_C	0.768154	9994	(+)	NCTATAAAAR	TTTTAAAAAA
M00252	V$TATA_01	0.827455	9994	(+)	STATAAAWRNNNNNN	TTTTAAAAAAAGAAT
M00216	V$TATA_C	0.793768	9995	(+)	NCTATAAAAR	TTTAAAAAAA
M00252	V$TATA_01	0.792438	10138	(+)	STATAAAWRNNNNNN	CTTTGAATAGGAACA
M00216	V$TATA_C	0.745709	10162	(+)	NCTATAAAAR	ACTGAAAAAA
M00216	V$TATA_C	0.786639	10200	(+)	NCTATAAAAR	ATTATAATAA
M00252	V$TATA_01	0.77087	10201	(+)	STATAAAWRNNNNNN	TTATAATAATATAAA
M00216	V$TATA_C	0.763665	10206	(+)	NCTATAAAAR	ATAATATAAA
M00216	V$TATA_C	0.849221	10208	(+)	NCTATAAAAR	AATATAAAAT
M00252	V$TATA_01	0.852068	10209	(+)	STATAAAWRNNNNNN	ATATAAAATTAAGTC
M00216	V$TATA_C	0.744389	10227	(+)	NCTATAAAAR	AGAATTAAAA
M00252	V$TATA_01	0.783304	10325	(+)	STATAAAWRNNNNNN	GTATTTATTTTCCCA
M00216	V$TATA_C	0.762873	10398	(+)	NCTATAAAAR	GCCAAATAAA
M00216	V$TATA_C	0.774756	10411	(+)	NCTATAAAAR	TCCATTTAAA
M00216	V$TATA_C	0.759968	10412	(+)	NCTATAAAAR	CCATTTAAAA
M00216	V$TATA_C	0.847637	10413	(+)	NCTATAAAAR	CATTTAAAAA
M00216	V$TATA_C	0.761025	10414	(+)	NCTATAAAAR	ATTTAAAAAA
M00252	V$TATA_01	0.866278	10414	(+)	STATAAAWRNNNNNN	ATTTAAAAAACAATG
M00216	V$TATA_C	0.767362	10415	(+)	NCTATAAAAR	TTTAAAAAAC
M00216	V$TATA_C	0.766306	10474	(+)	NCTATAAAAR	TTCATCAAAA
M00216	V$TATA_C	0.743068	10475	(+)	NCTATAAAAR	TCATCAAAAT
M00216	V$TATA_C	0.756007	10487	(+)	NCTATAAAAR	TCTAAGAAAG
M00216	V$TATA_C	0.758648	10538	(+)	NCTATAAAAR	CCTTTATAGT
M00216	V$TATA_C	0.890151	10630	(+)	NCTATAAAAR	CCAATAAAAT
M00252	V$TATA_01	0.774169	10631	(+)	STATAAAWRNNNNNN	CAATAAAATGTTCAA
M00216	V$TATA_C	0.806443	10639	(+)	NCTATAAAAR	TGTTCAAAAA

M00216	V$TATA_C	0.748878	10641	(+)	NCTATAAAAR	TTCAAAAAAT
M00216	V$TATA_C	0.803274	10673	(+)	NCTATAAAAR	ACTCTATAAA
M00216	V$TATA_C	0.971217	10675	(+)	NCTATAAAAR	TCTATAAAAT
M00252	V$TATA_01	0.852829	10676	(+)	STATAAAWRNNNNNN	CTATAAAATTTGCTA
M00216	V$TATA_C	0.850013	10793	(+)	NCTATAAAAR	GTCATAAAAC
M00252	V$TATA_01	0.771885	10823	(+)	STATAAAWRNNNNNN	TTTTTTAAGGAAATT
M00252	V$TATA_01	0.775438	10861	(+)	STATAAAWRNNNNNN	ATATATTTACTAAAT
M00216	V$TATA_C	0.823607	10873	(+)	NCTATAAAAR	AATTTAAAAT
M00252	V$TATA_01	0.822634	10883	(+)	STATAAAWRNNNNNN	ATATTAAATGCACTA
M00216	V$TATA_C	0.842091	10894	(+)	NCTATAAAAR	ACTATAAGAT
M00252	V$TATA_01	0.805379	10895	(+)	STATAAAWRNNNNNN	CTATAAGATGTGCAT
M00216	V$TATA_C	0.770531	10953	(+)	NCTATAAAAR	ATCAAAAAAG
M00216	V$TATA_C	0.809876	11035	(+)	NCTATAAAAR	GCCATAAATT
M00252	V$TATA_01	0.773408	11036	(+)	STATAAAWRNNNNNN	CCATAAATTTTTGTT
M00216	V$TATA_C	0.778981	11059	(+)	NCTATAAAAR	ACCAAATAAG
M00216	V$TATA_C	0.762873	11245	(+)	NCTATAAAAR	GCCAAATAAA
M00252	V$TATA_01	0.784065	11248	(+)	STATAAAWRNNNNNN	AAATAAATTTAGATA
M00252	V$TATA_01	0.78914	11258	(+)	STATAAAWRNNNNNN	AGATATATCAGTACC
M00252	V$TATA_01	0.777975	11260	(+)	STATAAAWRNNNNNN	ATATATCAGTACCTC
M00216	V$TATA_C	0.768682	11273	(+)	NCTATAAAAR	TCAATCAAAT
M00252	V$TATA_01	0.778736	11316	(+)	STATAAAWRNNNNNN	ATATATTTTAGCACC
M00252	V$TATA_01	0.782035	11375	(+)	STATAAAWRNNNNNN	CTATATGGTCCTGCT
M00216	V$TATA_C	0.762873	11482	(+)	NCTATAAAAR	TGAATGAAAG
M00216	V$TATA_C	0.742276	11520	(+)	NCTATAAAAR	TGAGTAAAAT
M00252	V$TATA_01	0.792946	11540	(+)	STATAAAWRNNNNNN	AGATAAAGAGATCTC
M00216	V$TATA_C	0.900977	11578	(+)	NCTATAAAAR	ACCATATAAG
M00252	V$TATA_01	0.85359	11579	(+)	STATAAAWRNNNNNN	CCATATAAGACTATT
M00216	V$TATA_C	0.808292	11634	(+)	NCTATAAAAR	TTTTTATAAT
M00252	V$TATA_01	0.77087	11635	(+)	STATAAAWRNNNNNN	TTTTATAATGTGTGT
M00216	V$TATA_C	0.779509	11660	(+)	NCTATAAAAR	TTTTTAACAG
M00252	V$TATA_01	0.79117	11670	(+)	STATAAAWRNNNNNN	AAATAAATAAAATCC
M00216	V$TATA_C	0.775812	11673	(+)	NCTATAAAAR	TAAATAAAAT
M00252	V$TATA_01	0.847754	11674	(+)	STATAAAWRNNNNNN	AAATAAAATCCAAGG

M00216	V$TATA_C	0.830737	11725	(+)	NCTATAAAAR	TATTTAAAAT
M00216	V$TATA_C	0.776604	11732	(+)	NCTATAAAAR	AATTTATAAA
M00252	V$TATA_01	0.801066	11733	(+)	STATAAAWRNNNNNN	ATTTATAAAAGATAA
M00216	V$TATA_C	0.927119	11734	(+)	NCTATAAAAR	TTTATAAAAG
M00252	V$TATA_01	0.851307	11735	(+)	STATAAAWRNNNNNN	TTATAAAAGATAAAA
M00252	V$TATA_01	0.78711	11742	(+)	STATAAAWRNNNNNN	AGATAAAATGTAAAT
M00216	V$TATA_C	0.764457	11896	(+)	NCTATAAAAR	GTTAGAAAAT
M00216	V$TATA_C	0.761025	11922	(+)	NCTATAAAAR	ATTTTCAAAA
M00216	V$TATA_C	0.750726	11923	(+)	NCTATAAAAR	TTTTCAAAAT
M00216	V$TATA_C	0.777396	11986	(+)	NCTATAAAAR	ATTGTAAAAT
M00252	V$TATA_01	0.814514	12045	(+)	STATAAAWRNNNNNN	CTATTTAGGAACAAC
M00216	V$TATA_C	0.836282	12065	(+)	NCTATAAAAR	AGTATGAAAG
M00252	V$TATA_01	0.831515	12066	(+)	STATAAAWRNNNNNN	GTATGAAAGAGGGAA
M00216	V$TATA_C	0.799313	12139	(+)	NCTATAAAAR	AGTTTAAATA
M00252	V$TATA_01	0.842172	12140	(+)	STATAAAWRNNNNNN	GTTTAAATATGTTTG
M00216	V$TATA_C	0.811724	12154	(+)	NCTATAAAAR	GCTTTTAAAT
M00216	V$TATA_C	0.767626	12155	(+)	NCTATAAAAR	CTTTTAAATA
M00252	V$TATA_01	0.817305	12156	(+)	STATAAAWRNNNNNN	TTTTAAATAGCTTCA
M00216	V$TATA_C	0.823607	12277	(+)	NCTATAAAAR	TCTTTGAAAT
M00252	V$TATA_01	0.791423	12278	(+)	STATAAAWRNNNNNN	CTTTGAAATGGGAGC
M00216	V$TATA_C	0.758648	12327	(+)	NCTATAAAAR	CCTTTGTAAT
M00252	V$TATA_01	0.773154	12458	(+)	STATAAAWRNNNNNN	ATTTATATTTAGGAA
M00252	V$TATA_01	0.7932	12460	(+)	STATAAAWRNNNNNN	TTATATTTAGGAAAT
M00252	V$TATA_01	0.77493	12518	(+)	STATAAAWRNNNNNN	TTATTAATTCTAATT
M00216	V$TATA_C	0.85371	12537	(+)	NCTATAAAAR	CCTTTAGAAG
M00216	V$TATA_C	0.752839	12574	(+)	NCTATAAAAR	TCTGTCAAAA
M00216	V$TATA_C	0.906786	12628	(+)	NCTATAAAAR	TCTATATAAT
M00252	V$TATA_01	0.876935	12629	(+)	STATAAAWRNNNNNN	CTATATAATAGAAAC
M00216	V$TATA_C	0.763137	12630	(+)	NCTATAAAAR	TATATAATAG
M00252	V$TATA_01	0.803096	12631	(+)	STATAAAWRNNNNNN	ATATAATAGAAACTG
M00252	V$TATA_01	0.785334	12704	(+)	STATAAAWRNNNNNN	ATTTTTAAGGTGACA
M00216	V$TATA_C	0.866121	12761	(+)	NCTATAAAAR	CCTATCAAAA
M00252	V$TATA_01	0.826947	12762	(+)	STATAAAWRNNNNNN	CTATCAAAAGCTATG

M00216	V$TATA_C	0.742012	12839	(+)	NCTATAAAAR	GTCTTAGAAG
M00216	V$TATA_C	0.78479	12857	(+)	NCTATAAAAR	TCTAGATAAT
M00252	V$TATA_01	0.785841	12858	(+)	STATAAAWRNNNNNN	CTAGATAATGCAATT
M00216	V$TATA_C	0.761025	12918	(+)	NCTATAAAAR	ATTTCAAAAA
M00216	V$TATA_C	0.935041	12959	(+)	NCTATAAAAR	GCCTTAAAAG
M00252	V$TATA_01	0.804872	12960	(+)	STATAAAWRNNNNNN	CCTTAAAAGATCACC
M00216	V$TATA_C	0.755479	13001	(+)	NCTATAAAAR	CCTATGATAG
M00216	V$TATA_C	0.799313	13045	(+)	NCTATAAAAR	AGTTTTAAAA
M00216	V$TATA_C	0.851862	13046	(+)	NCTATAAAAR	GTTTTAAAAC
M00252	V$TATA_01	0.813499	13046	(+)	STATAAAWRNNNNNN	GTTTTAAAACTGCCC
M00252	V$TATA_01	0.810454	13047	(+)	STATAAAWRNNNNNN	TTTTAAAACTGCCCC
M00216	V$TATA_C	0.866121	13187	(+)	NCTATAAAAR	GCTATGAAAG
M00252	V$TATA_01	0.820858	13188	(+)	STATAAAWRNNNNNN	CTATGAAAGATCAGA
M00216	V$TATA_C	0.743068	13308	(+)	NCTATAAAAR	TCATTCAAAT
M00252	V$TATA_01	0.785334	13326	(+)	STATAAAWRNNNNNN	TTATTAATCTATAGT
M00216	V$TATA_C	0.77502	13465	(+)	NCTATAAAAR	CTTCTAAAAC
M00216	V$TATA_C	0.810668	13514	(+)	NCTATAAAAR	AGTTTTAAAG
M00216	V$TATA_C	0.767626	13515	(+)	NCTATAAAAR	GTTTTAAAGG
M00252	V$TATA_01	0.805633	13515	(+)	STATAAAWRNNNNNN	GTTTTAAAGGTGGTA
M00252	V$TATA_01	0.771124	13516	(+)	STATAAAWRNNNNNN	TTTTAAAGGTGGTAA
M00216	V$TATA_C	0.841035	13588	(+)	NCTATAAAAR	AATTTAAAAA
M00216	V$TATA_C	0.761025	13589	(+)	NCTATAAAAR	ATTTAAAAAA
M00252	V$TATA_01	0.848769	13589	(+)	STATAAAWRNNNNNN	ATTTAAAAAAGAAAC
M00216	V$TATA_C	0.805123	13590	(+)	NCTATAAAAR	TTTAAAAAAG
M00216	V$TATA_C	0.813837	13624	(+)	NCTATAAAAR	GTTTTATAAA
M00252	V$TATA_01	0.780259	13625	(+)	STATAAAWRNNNNNN	TTTTATAAATATGAA
M00216	V$TATA_C	0.793768	13626	(+)	NCTATAAAAR	TTTATAAATA
M00252	V$TATA_01	0.884801	13627	(+)	STATAAAWRNNNNNN	TTATAAATATGAATC
M00252	V$TATA_01	0.775691	13649	(+)	STATAAAWRNNNNNN	GGATTAAACAAGATT
M00216	V$TATA_C	0.783998	13736	(+)	NCTATAAAAR	CTTCTAAAAT
M00216	V$TATA_C	0.757328	13834	(+)	NCTATAAAAR	TCCATGTAAT
M00216	V$TATA_C	0.768154	13843	(+)	NCTATAAAAR	TTTTTTAAAA
M00252	V$TATA_01	0.78914	13843	(+)	STATAAAWRNNNNNN	TTTTTTAAAAAGAGG

M00216	V$TATA_C	0.890151	13844	(+)	NCTATAAAAR	TTTTTAAAAA
M00252	V$TATA_01	0.789394	13844	(+)	STATAAAWRNNNNNN	TTTTTAAAAAGAGGG
M00216	V$TATA_C	0.779509	13845	(+)	NCTATAAAAR	TTTTAAAAAG
M00252	V$TATA_01	0.857904	13845	(+)	STATAAAWRNNNNNN	TTTTAAAAAGAGGGT
M00252	V$TATA_01	0.778229	13913	(+)	STATAAAWRNNNNNN	GCATTTAACTGGGTT
M00252	V$TATA_01	0.826694	14011	(+)	STATAAAWRNNNNNN	ATATTAATACTAATA
M00216	V$TATA_C	0.850541	14114	(+)	NCTATAAAAR	TCCATCAAAG
M00252	V$TATA_01	0.772393	14127	(+)	STATAAAWRNNNNNN	ATATATTTTCCACCT
M00252	V$TATA_01	0.827962	14142	(+)	STATAAAWRNNNNNN	GAATATATAAAGAAT
M00216	V$TATA_C	0.802218	14143	(+)	NCTATAAAAR	AATATATAAA
M00252	V$TATA_01	0.876427	14144	(+)	STATAAAWRNNNNNN	ATATATAAAGAATTA
M00216	V$TATA_C	0.751782	14145	(+)	NCTATAAAAR	TATATAAAGA
M00252	V$TATA_01	0.832784	14146	(+)	STATAAAWRNNNNNN	ATATAAAGAATTACA
M00252	V$TATA_01	0.821111	14158	(+)	STATAAAWRNNNNNN	ACATTAATGCACAGA
M00216	V$TATA_C	0.804067	14175	(+)	NCTATAAAAR	CAAATAAAAG
M00252	V$TATA_01	0.881756	14176	(+)	STATAAAWRNNNNNN	AAATAAAAGGCCATG
M00216	V$TATA_C	0.77766	14221	(+)	NCTATAAAAR	TTCAGAAAAG
M00216	V$TATA_C	0.766306	14377	(+)	NCTATAAAAR	TTCATGAAAA
M00216	V$TATA_C	0.760496	14378	(+)	NCTATAAAAR	TCATGAAAAA
M00216	V$TATA_C	0.834962	14392	(+)	NCTATAAAAR	ACTATAGAAC
M00252	V$TATA_01	0.78711	14393	(+)	STATAAAWRNNNNNN	CTATAGAACAGAAAG
M00216	V$TATA_C	0.856879	14442	(+)	NCTATAAAAR	AGTTTATAAA
M00252	V$TATA_01	0.7932	14443	(+)	STATAAAWRNNNNNN	GTTTATAAATTATAT
M00216	V$TATA_C	0.77634	14444	(+)	NCTATAAAAR	TTTATAAATT
M00252	V$TATA_01	0.777468	14445	(+)	STATAAAWRNNNNNN	TTATAAATTATATAA
M00216	V$TATA_C	0.826776	14451	(+)	NCTATAAAAR	ATTATATAAT
M00252	V$TATA_01	0.786856	14452	(+)	STATAAAWRNNNNNN	TTATATAATAATTAA
M00216	V$TATA_C	0.751782	14453	(+)	NCTATAAAAR	TATATAATAA
M00252	V$TATA_01	0.774676	14454	(+)	STATAAAWRNNNNNN	ATATAATAATTAAAT
M00216	V$TATA_C	0.744125	14589	(+)	NCTATAAAAR	CCTAAGAAAA
M00216	V$TATA_C	0.743068	14947	(+)	NCTATAAAAR	TCATTTAAAT
M00252	V$TATA_01	0.827201	14949	(+)	STATAAAWRNNNNNN	ATTTAAATGACTGCA
M00252	V$TATA_01	0.775438	14974	(+)	STATAAAWRNNNNNN	GTATCAAGGGAAATA

M00216	V$TATA_C	0.763665	14998	(+)	NCTATAAAAR	ATAATATAAA
M00252	V$TATA_01	0.773662	14999	(+)	STATAAAWRNNNNNN	TAATATAAAAATAAA
M00216	V$TATA_C	0.866649	15000	(+)	NCTATAAAAR	AATATAAAAA
M00252	V$TATA_01	0.894189	15001	(+)	STATAAAWRNNNNNN	ATATAAAAATAAATT
M00252	V$TATA_01	0.779244	15007	(+)	STATAAAWRNNNNNN	AAATAAATTTAGAAT
M00252	V$TATA_01	0.781528	15031	(+)	STATAAAWRNNNNNN	ACTTAAATGCTTAGT
M00252	V$TATA_01	0.8102	15092	(+)	STATAAAWRNNNNNN	TGATATATGGCTAAT
M00216	V$TATA_C	0.85239	15240	(+)	NCTATAAAAR	TCTTTAAATG
M00252	V$TATA_01	0.850799	15241	(+)	STATAAAWRNNNNNN	CTTTAAATGCCTTTC
M00216	V$TATA_C	0.746237	15371	(+)	NCTATAAAAR	ATAATATAAT
M00252	V$TATA_01	0.792185	15388	(+)	STATAAAWRNNNNNN	GTTTTTATAACCTTG
M00216	V$TATA_C	0.799313	15389	(+)	NCTATAAAAR	TTTTTATAAC
M00216	V$TATA_C	0.744653	15413	(+)	NCTATAAAAR	AATATAATAA
M00252	V$TATA_01	0.806648	15414	(+)	STATAAAWRNNNNNN	ATATAATAATCTATT
M00252	V$TATA_01	0.841665	15605	(+)	STATAAAWRNNNNNN	ATATGAATACAAAGG
M00216	V$TATA_C	0.753367	15608	(+)	NCTATAAAAR	TGAATACAAA
M00252	V$TATA_01	0.77899	15611	(+)	STATAAAWRNNNNNN	ATACAAAGGTACAAC
M00216	V$TATA_C	0.842091	15632	(+)	NCTATAAAAR	ACTATAAAGT
M00252	V$TATA_01	0.813499	15633	(+)	STATAAAWRNNNNNN	CTATAAAGTTGTTTT
M00252	V$TATA_01	0.839635	15666	(+)	STATAAAWRNNNNNN	CTATATTTAGACACA
M00252	V$TATA_01	0.817813	15668	(+)	STATAAAWRNNNNNN	ATATTTAGACACATT
M00216	V$TATA_C	0.786639	15729	(+)	NCTATAAAAR	ATTATAATAA
M00252	V$TATA_01	0.773915	15745	(+)	STATAAAWRNNNNNN	ATTTAAGTAACAATA
M00216	V$TATA_C	0.874571	15754	(+)	NCTATAAAAR	ACAATAAAAC
M00252	V$TATA_01	0.804111	15755	(+)	STATAAAWRNNNNNN	CAATAAAACTAGCTA
M00216	V$TATA_C	0.753367	15777	(+)	NCTATAAAAR	TTAATATAAT
M00216	V$TATA_C	0.757856	15803	(+)	NCTATAAAAR	TCTACACAAG
M00216	V$TATA_C	0.759176	15865	(+)	NCTATAAAAR	TCTTTATGAT
M00252	V$TATA_01	0.773915	15866	(+)	STATAAAWRNNNNNN	CTTTATGATCACTGG
M00216	V$TATA_C	0.741484	15879	(+)	NCTATAAAAR	GGAATAGAAA
M00216	V$TATA_C	0.820702	15889	(+)	NCTATAAAAR	TACTTAAAAA
M00216	V$TATA_C	0.84526	15890	(+)	NCTATAAAAR	ACTTAAAAAG
M00252	V$TATA_01	0.804111	15890	(+)	STATAAAWRNNNNNN	ACTTAAAAAGTCACA

M00252	V$TATA_01	0.851561	15943	(+)	STATAAAWRNNNNNN	ATTTAAATGTCCATA
M00216	V$TATA_C	0.788487	16044	(+)	NCTATAAAAR	CCCTTACAAC
M00216	V$TATA_C	0.757064	16130	(+)	NCTATAAAAR	ACTGCAAAAG
M00252	V$TATA_01	0.814514	16154	(+)	STATAAAWRNNNNNN	TCATAAATTGCCTGT
M00216	V$TATA_C	0.847901	16164	(+)	NCTATAAAAR	CCTGTAAAAC
M00216	V$TATA_C	0.813573	16171	(+)	NCTATAAAAR	AACTTAAAAA
M00216	V$TATA_C	0.816477	16172	(+)	NCTATAAAAR	ACTTAAAAAT
M00252	V$TATA_01	0.799036	16172	(+)	STATAAAWRNNNNNN	ACTTAAAAATCAGGA
M00216	V$TATA_C	0.754951	16215	(+)	NCTATAAAAR	CACATATAAC
M00252	V$TATA_01	0.773154	16216	(+)	STATAAAWRNNNNNN	ACATATAACTTTGTG
M00216	V$TATA_C	0.870874	16259	(+)	NCTATAAAAR	ACTATTAAAG
M00252	V$TATA_01	0.861964	16260	(+)	STATAAAWRNNNNNN	CTATTAAAGTGTATC
M00216	V$TATA_C	0.744917	16272	(+)	NCTATAAAAR	ATCTTAACAG
M00216	V$TATA_C	0.840507	16290	(+)	NCTATAAAAR	CCTTTCAAAA
M00216	V$TATA_C	0.74122	16291	(+)	NCTATAAAAR	CTTTCAAAAC
M00216	V$TATA_C	0.746501	16397	(+)	NCTATAAAAR	TCTATCCAAA
M00216	V$TATA_C	0.740692	16420	(+)	NCTATAAAAR	GTTTTAGAAT
M00216	V$TATA_C	0.831529	16553	(+)	NCTATAAAAR	GGTATAAAGG
M00252	V$TATA_01	0.919817	16554	(+)	STATAAAWRNNNNNN	GTATAAAGGCCACTG
M00216	V$TATA_C	0.748878	16589	(+)	NCTATAAAAR	TTCATTAAAT
M00216	V$TATA_C	0.743068	16590	(+)	NCTATAAAAR	TCATTAAATT
M00252	V$TATA_01	0.780005	16601	(+)	STATAAAWRNNNNNN	CTATATGGAAAATGT
M00216	V$TATA_C	0.742012	16613	(+)	NCTATAAAAR	TGTTTTTAAA
M00216	V$TATA_C	0.767626	16614	(+)	NCTATAAAAR	GTTTTTAAAG
M00252	V$TATA_01	0.809947	16614	(+)	STATAAAWRNNNNNN	GTTTTTAAAGCCTTT
M00216	V$TATA_C	0.741748	16615	(+)	NCTATAAAAR	TTTTTAAAGC
M00252	V$TATA_01	0.775438	16615	(+)	STATAAAWRNNNNNN	TTTTTAAAGCCTTTG
M00216	V$TATA_C	0.768682	16702	(+)	NCTATAAAAR	TCAATAAATT
M00252	V$TATA_01	0.776453	16703	(+)	STATAAAWRNNNNNN	CAATAAATTACATTT
M00223	V$STAT_01	0.834384	159	(+)	TTCCCRKAA	TTCCTATGA
M00223	V$STAT_01	0.868666	327	(+)	TTCCCRKAA	TTATGGTAA
M00223	V$STAT_01	0.931938	327	(-)	TTCCCRKAA	TTATGGTAA
M00223	V$STAT_01	0.802622	462	(-)	TTCCCRKAA	TTAGGTTAA

M00225	V$STAT3_01	0.759305	730	(+)	NGNNATTTCCSGGAARTGNNN	TCTTATTTATCTGAAATGCAT
M00225	V$STAT3_01	0.744243	730	(-)	NGNNATTTCCSGGAARTGNNN	TCTTATTTATCTGAAATGCAT
M00223	V$STAT_01	0.80968	736	(+)	TTCCCRKAA	TTATCTGAA
M00223	V$STAT_01	0.826569	736	(-)	TTCCCRKAA	TTATCTGAA
M00223	V$STAT_01	0.816738	794	(+)	TTCCCRKAA	TTAATGGAA
M00223	V$STAT_01	0.849761	1080	(-)	TTCCCRKAA	CTACAGGAA
M00223	V$STAT_01	0.816738	1104	(+)	TTCCCRKAA	TTATTGTCA
M00223	V$STAT_01	0.817746	1154	(+)	TTCCCRKAA	TTCCTCTGA
M00223	V$STAT_01	0.790522	1170	(+)	TTCCCRKAA	TTACTATTA
M00223	V$STAT_01	0.888077	1339	(+)	TTCCCRKAA	TTATCATAA
M00223	V$STAT_01	0.851525	1339	(-)	TTCCCRKAA	TTATCATAA
M00223	V$STAT_01	0.816738	1357	(+)	TTCCCRKAA	TTGTTGGAA
M00223	V$STAT_01	0.861356	1397	(+)	TTCCCRKAA	TTATTATAA
M00223	V$STAT_01	0.805899	1497	(-)	TTCCCRKAA	GTACAGTAA
M00223	V$STAT_01	0.832871	2116	(+)	TTCCCRKAA	TTCCAGGCA
M00223	V$STAT_01	0.856819	2116	(-)	TTCCCRKAA	TTCCAGGCA
M00223	V$STAT_01	0.826821	2351	(+)	TTCCCRKAA	TTCTTCTCA
M00223	V$STAT_01	0.8364	2354	(+)	TTCCCRKAA	TTCTCAAAA
M00223	V$STAT_01	0.8606	2451	(-)	TTCCCRKAA	TGACAAGAA
M00223	V$STAT_01	0.8606	2666	(-)	TTCCCRKAA	TGACAGTAA
M00224	V$STAT1_01	0.725836	2670	(+)	NNNSANTTCCGGGAANTGNSN	AGTAAGTTACGAAAAAGGGT
M00223	V$STAT_01	0.799849	2676	(+)	TTCCCRKAA	TTACGAAAA
M00223	V$STAT_01	0.8364	2676	(-)	TTCCCRKAA	TTACGAAAA
M00223	V$STAT_01	0.851525	2891	(+)	TTCCCRKAA	TTATGATAA
M00223	V$STAT_01	0.888077	2891	(-)	TTCCCRKAA	TTATGATAA
M00223	V$STAT_01	0.826317	3170	(+)	TTCCCRKAA	TTAACAGAA
M00223	V$STAT_01	0.809932	3170	(-)	TTCCCRKAA	TTAACAGAA
M00223	V$STAT_01	0.833627	3229	(+)	TTCCCRKAA	TTCTAAGAA
M00223	V$STAT_01	0.912276	3229	(-)	TTCCCRKAA	TTCTAAGAA
M00223	V$STAT_01	0.826317	3263	(-)	TTCCCRKAA	TGATGATAA
M00223	V$STAT_01	0.805899	3604	(-)	TTCCCRKAA	TTACAAGAG
M00223	V$STAT_01	0.819763	3700	(+)	TTCCCRKAA	TTACCCCAA
M00223	V$STAT_01	0.849761	4097	(-)	TTCCCRKAA	CTACAGGAA

M00223	V$STAT_01	0.791782	4137	(-)	TTCCCRKAA	TGGCGGTAA
M00224	V$STAT1_01	0.763544	4175	(+)	NNNSANTTCCGGGAANTGNSN	TGTATTTTTCTGGAAAAATCA
M00223	V$STAT_01	0.8606	4181	(+)	TTCCCRKAA	TTTCTGGAA
M00223	V$STAT_01	0.792034	4181	(-)	TTCCCRKAA	TTTCTGGAA
M00223	V$STAT_01	0.81699	4182	(+)	TTCCCRKAA	TTCTGGAAA
M00223	V$STAT_01	0.870179	4182	(-)	TTCCCRKAA	TTCTGGAAA
M00223	V$STAT_01	0.817242	4399	(+)	TTCCCRKAA	TTCTCATTA
M00223	V$STAT_01	0.792538	4563	(+)	TTCCCRKAA	TTAATATAA
M00224	V$STAT1_01	0.732752	4585	(+)	NNNSANTTCCGGGAANTGNSN	TAGCCTTTTCCAGAAGTGTTT
M00224	V$STAT1_01	0.720896	4585	(-)	NNNSANTTCCGGGAANTGNSN	TAGCCTTTTCCAGAAGTGTTT
M00225	V$STAT3_01	0.751774	4585	(+)	NGNNATTTCCSGGAARTGNNN	TAGCCTTTTCCAGAAGTGTTT
M00223	V$STAT_01	0.870179	4591	(+)	TTCCCRKAA	TTTCCAGAA
M00223	V$STAT_01	0.81699	4591	(-)	TTCCCRKAA	TTTCCAGAA
M00223	V$STAT_01	0.815478	4671	(-)	TTCCCRKAA	TTATGGTAT
M00223	V$STAT_01	0.929418	4810	(+)	TTCCCRKAA	TTACTGGAA
M00223	V$STAT_01	0.887572	4810	(-)	TTCCCRKAA	TTACTGGAA
M00223	V$STAT_01	0.826821	4910	(-)	TTCCCRKAA	TTGCAAGAA
M00223	V$STAT_01	0.800605	5032	(-)	TTCCCRKAA	TAAGGAGAA
M00223	V$STAT_01	0.806907	5153	(+)	TTCCCRKAA	TTAAGGGAA
M00223	V$STAT_01	0.890345	5153	(-)	TTCCCRKAA	TTAAGGGAA
M00223	V$STAT_01	0.966221	5265	(+)	TTCCCRKAA	TTCCTGTAA
M00223	V$STAT_01	0.850769	5265	(-)	TTCCCRKAA	TTCCTGTAA
M00223	V$STAT_01	0.8243	5316	(-)	TTCCCRKAA	TACTGAGAA
M00223	V$STAT_01	0.792538	5378	(+)	TTCCCRKAA	TTAGTATAA
M00223	V$STAT_01	0.812957	5502	(-)	TTCCCRKAA	CTCCAAGAA
M00223	V$STAT_01	0.867658	5656	(+)	TTCCCRKAA	TTCTTGGCA
M00223	V$STAT_01	0.826821	5834	(-)	TTCCCRKAA	TTCAAAGAA
M00223	V$STAT_01	0.801361	5952	(-)	TTCCCRKAA	TGATGGAAA
M00223	V$STAT_01	0.863625	6121	(-)	TTCCCRKAA	TTAAAGGAA
M00223	V$STAT_01	0.792538	6152	(+)	TTCCCRKAA	TTATCAAAA
M00223	V$STAT_01	0.795815	6376	(-)	TTCCCRKAA	TTCTAAGGA
M00223	V$STAT_01	0.800605	6587	(-)	TTCCCRKAA	TCAGGAGAA
M00223	V$STAT_01	0.791782	6686	(+)	TTCCCRKAA	TTTCTGTCA

M00223	V$STAT_01	0.921099	6960	(+)	TTCCCRKAA	TTCCCAGCA
M00223	V$STAT_01	0.803126	6960	(-)	TTCCCRKAA	TTCCCAGCA
M00223	V$STAT_01	0.819763	7042	(+)	TTCCCRKAA	TTCTCCCAA
M00223	V$STAT_01	0.849761	7069	(+)	TTCCCRKAA	TTCCTGTAG
M00223	V$STAT_01	0.802622	7393	(-)	TTCCCRKAA	TTAGGAAAA
M00223	V$STAT_01	0.819259	7924	(-)	TTCCCRKAA	TTATGTTAA
M00224	V$STAT1_01	0.771283	7984	(-)	NNNSANTTCCGGGAANTGNSN	TTACATGTCCCGTAAGGTTTC
M00225	V$STAT3_01	0.731354	7984	(+)	NGNNATTTCCSGGAARTGNNN	TTACATGTCCCGTAAGGTTTC
M00223	V$STAT_01	0.876481	7990	(+)	TTCCCRKAA	GTCCCGTAA
M00223	V$STAT_01	0.803126	7990	(-)	TTCCCRKAA	GTCCCGTAA
M00223	V$STAT_01	0.857827	8114	(+)	TTCCCRKAA	TTCTGGGCA
M00223	V$STAT_01	0.866398	8114	(-)	TTCCCRKAA	TTCTGGGCA
M00223	V$STAT_01	0.866398	8181	(+)	TTCCCRKAA	TTCCCAGAT
M00223	V$STAT_01	0.803126	8181	(-)	TTCCCRKAA	TTCCCAGAT
M00223	V$STAT_01	0.826569	8220	(+)	TTCCCRKAA	TTACAATAA
M00223	V$STAT_01	0.878498	8220	(-)	TTCCCRKAA	TTACAATAA
M00224	V$STAT1_01	0.720896	8265	(-)	NNNSANTTCCGGGAANTGNSN	ATATCCTTTGCGGAACTCAAT
M00223	V$STAT_01	0.827073	8271	(-)	TTCCCRKAA	TTTGCGGAA
M00223	V$STAT_01	0.826317	8776	(+)	TTCCCRKAA	TTAGCAGAA
M00223	V$STAT_01	0.87875	8776	(-)	TTCCCRKAA	TTAGCAGAA
M00223	V$STAT_01	0.796068	8829	(+)	TTCCCRKAA	ATCTGGTAA
M00223	V$STAT_01	0.822536	8829	(-)	TTCCCRKAA	ATCTGGTAA
M00223	V$STAT_01	0.795815	8956	(-)	TTCCCRKAA	TTCTAGTAG
M00223	V$STAT_01	0.894379	9140	(+)	TTCCCRKAA	TTACCGGCA
M00223	V$STAT_01	0.839929	9140	(-)	TTCCCRKAA	TTACCGGCA
M00223	V$STAT_01	0.850517	9581	(+)	TTCCCRKAA	TTCTTAGCA
M00223	V$STAT_01	0.818503	9795	(+)	TTCCCRKAA	TTCCCAACA
M00223	V$STAT_01	0.793043	9828	(+)	TTCCCRKAA	TTCTTCAAA
M00223	V$STAT_01	0.812957	9874	(-)	TTCCCRKAA	TTCCAGTGA
M00224	V$STAT1_01	0.735551	10074	(+)	NNNSANTTCCGGGAANTGNSN	CTTTACTCCTGGGAAAGGGAT
M00224	V$STAT1_01	0.726494	10074	(-)	NNNSANTTCCGGGAANTGNSN	CTTTACTCCTGGGAAAGGGAT
M00225	V$STAT3_01	0.743519	10074	(+)	NGNNATTTCCSGGAARTGNNN	CTTTACTCCTGGGAAAGGGAT
M00225	V$STAT3_01	0.720492	10074	(-)	NGNNATTTCCSGGAARTGNNN	CTTTACTCCTGGGAAAGGGAT

M00223	V$STAT_01	0.803126	10080	(+)	TTCCCRKAA	TCCTGGGAA
M00223	V$STAT_01	0.868162	10080	(-)	TTCCCRKAA	TCCTGGGAA
M00223	V$STAT_01	0.822536	10155	(+)	TTCCCRKAA	TTCTCAGAC
M00223	V$STAT_01	0.861356	10198	(+)	TTCCCRKAA	TTATTATAA
M00223	V$STAT_01	0.861356	10201	(-)	TTCCCRKAA	TTATAATAA
M00223	V$STAT_01	0.833627	10486	(+)	TTCCCRKAA	TTCTAAGAA
M00223	V$STAT_01	0.912276	10486	(-)	TTCCCRKAA	TTCTAAGAA
M00224	V$STAT1_01	0.758439	10651	(-)	NNNSANTTCCGGGAANTGNSN	CTCTCCTTCCCCTAATCCCTA
M00223	V$STAT_01	0.959163	10657	(+)	TTCCCRKAA	TTCCCCTAA
M00223	V$STAT_01	0.806907	10657	(-)	TTCCCRKAA	TTCCCCTAA
M00223	V$STAT_01	0.817242	10694	(-)	TTCCCRKAA	TCATGAGAA
M00223	V$STAT_01	0.880262	10839	(+)	TTCCCRKAA	TTCCCAAAA
M00223	V$STAT_01	0.806907	10839	(-)	TTCCCRKAA	TTCCCAAAA
M00223	V$STAT_01	0.853542	11149	(-)	TTCCCRKAA	TTGTAGGAA
M00223	V$STAT_01	0.795815	11295	(-)	TTCCCRKAA	ATCTAAGAA
M00223	V$STAT_01	0.853542	11305	(-)	TTCCCRKAA	TTACATGAA
M00223	V$STAT_01	0.836904	11559	(-)	TTCCCRKAA	TTTGAGGAA
M00223	V$STAT_01	0.792538	11634	(+)	TTCCCRKAA	TTTTTATAA
M00223	V$STAT_01	0.826317	11663	(+)	TTCCCRKAA	TTAACAGAA
M00223	V$STAT_01	0.809932	11663	(-)	TTCCCRKAA	TTAACAGAA
M00223	V$STAT_01	0.8364	11856	(-)	TTCCCRKAA	TTATAGCAA
M00223	V$STAT_01	0.792538	11951	(-)	TTCCCRKAA	TTATACTAA
M00223	V$STAT_01	0.814721	11977	(+)	TTCCCRKAA	TTCTTGGTA
M00223	V$STAT_01	0.870683	12190	(+)	TTCCCRKAA	TTCCTCTCA
M00223	V$STAT_01	0.80968	12235	(-)	TTCCCRKAA	TTGTAGTAA
M00223	V$STAT_01	0.822536	12247	(-)	TTCCCRKAA	ATCTGAGAA
M00223	V$STAT_01	0.826821	12276	(+)	TTCCCRKAA	TTCTTTGAA
M00223	V$STAT_01	0.819259	12368	(-)	TTCCCRKAA	TTATGTTAA
M00223	V$STAT_01	0.819259	12386	(-)	TTCCCRKAA	TTATGTTAA
M00223	V$STAT_01	0.888581	12540	(-)	TTCCCRKAA	TTAGAAGAA
M00223	V$STAT_01	0.791782	12572	(+)	TTCCCRKAA	TTTCTGTCA
M00223	V$STAT_01	0.88732	12759	(+)	TTCCCRKAA	TTCCTATCA
M00223	V$STAT_01	0.795815	12839	(+)	TTCCCRKAA	GTCTTAGAA

M00223	V$STAT_01	0.888581	12842	(-)	TTCCCRKAA	TTAGAAGAA
M00224	V$STAT1_01	0.725671	12982	(+)	NNNSANTTCCGGGAANTGNSN	TTTTTCTTTCCAGAAACAGCC
M00224	V$STAT1_01	0.736539	12982	(-)	NNNSANTTCCGGGAANTGNSN	TTTTTCTTTCCAGAAACAGCC
M00225	V$STAT3_01	0.724547	12982	(-)	NGNNATTTCCSGGAARTGNNN	TTTTTCTTTCCAGAAACAGCC
M00224	V$STAT1_01	0.732422	12983	(+)	NNNSANTTCCGGGAANTGNSN	TTTTCTTTCCAGAAACAGCCT
M00223	V$STAT_01	0.870179	12988	(+)	TTCCCRKAA	TTTCCAGAA
M00223	V$STAT_01	0.81699	12988	(-)	TTCCCRKAA	TTTCCAGAA
M00223	V$STAT_01	0.792034	12989	(+)	TTCCCRKAA	TTCCAGAAA
M00223	V$STAT_01	0.8606	12989	(-)	TTCCCRKAA	TTCCAGAAA
M00223	V$STAT_01	0.888581	13463	(+)	TTCCCRKAA	TTCTTCTAA
M00223	V$STAT_01	0.851525	13690	(+)	TTCCCRKAA	TTATGATAA
M00223	V$STAT_01	0.888077	13690	(-)	TTCCCRKAA	TTATGATAA
M00223	V$STAT_01	0.888581	13734	(+)	TTCCCRKAA	TTCTTCTAA
M00223	V$STAT_01	0.792538	14009	(-)	TTCCCRKAA	TTATATTAA
M00223	V$STAT_01	0.861356	14049	(+)	TTCCCRKAA	TTATTATAA
M00223	V$STAT_01	0.80968	14156	(-)	TTCCCRKAA	TTACATTAA
M00223	V$STAT_01	0.867658	14308	(+)	TTCCCRKAA	TTCTTGGCA
M00223	V$STAT_01	0.803126	14337	(+)	TTCCCRKAA	TTCTGGGAC
M00223	V$STAT_01	0.866398	14337	(-)	TTCCCRKAA	TTCTGGGAC
M00223	V$STAT_01	0.812957	14525	(+)	TTCCCRKAA	TTCTTGGAG
M00223	V$STAT_01	0.79884	14585	(+)	TTCCCRKAA	GTACCCTAA
M00223	V$STAT_01	0.807663	14617	(+)	TTCCCRKAA	TTACTGTGA
M00223	V$STAT_01	0.815478	14627	(+)	TTCCCRKAA	TTACCATAG
M00223	V$STAT_01	0.801361	14683	(-)	TTCCCRKAA	TGATGCGAA
M00223	V$STAT_01	0.815478	14745	(+)	TTCCCRKAA	TTCTCATAC
M00223	V$STAT_01	0.822536	14902	(-)	TTCCCRKAA	CTCTGAGAA
M00223	V$STAT_01	0.844719	15015	(-)	TTCCCRKAA	TTAGAATAA
M00224	V$STAT1_01	0.727482	15054	(+)	NNNSANTTCCGGGAANTGNSN	CTAGAATTTTCTGAAATGGAA
M00225	V$STAT3_01	0.710355	15054	(+)	NGNNATTTCCSGGAARTGNNN	CTAGAATTTTCTGAAATGGAA
M00224	V$STAT1_01	0.727647	15055	(+)	NNNSANTTCCGGGAANTGNSN	TAGAATTTTCTGAAATGGAAA
M00223	V$STAT_01	0.826317	15078	(-)	TTCCCRKAA	TGATGATAA
M00225	V$STAT3_01	0.723678	15356	(-)	NGNNATTTCCSGGAARTGNNN	TTTCGCTTACTTGAAATAATA
M00223	V$STAT_01	0.826821	15362	(+)	TTCCCRKAA	TTACTTGAA

M00223	V$STAT_01	0.792538	15389	(+)	TTCCCRKAA	TTTTTATAA
M00223	V$STAT_01	0.803126	15437	(+)	TTCCCRKAA	TGCTGGGAA
M00223	V$STAT_01	0.921099	15437	(-)	TTCCCRKAA	TGCTGGGAA
M00223	V$STAT_01	0.815478	15540	(-)	TTCCCRKAA	TTATGAGGA
M00223	V$STAT_01	0.861356	15730	(-)	TTCCCRKAA	TTATAATAA
M00223	V$STAT_01	0.792538	15777	(+)	TTCCCRKAA	TTAATATAA
M00223	V$STAT_01	0.797832	15836	(+)	TTCCCRKAA	TTCTGGTGA
M00223	V$STAT_01	0.822536	15836	(-)	TTCCCRKAA	TTCTGGTGA
M00223	V$STAT_01	0.812957	15874	(+)	TTCCCRKAA	TCACTGGAA
M00223	V$STAT_01	0.805899	15980	(+)	TTCCCRKAA	TTACTGTAT
M00223	V$STAT_01	0.826821	16128	(+)	TTCCCRKAA	TTACTGCAA
M00223	V$STAT_01	0.849761	16162	(+)	TTCCCRKAA	TGCCTGTAA
M00223	V$STAT_01	0.802622	16510	(+)	TTCCCRKAA	TTGTCCTAA
M00223	V$STAT_01	0.812957	16535	(-)	TTCCCRKAA	TTCCAGTAG
M00224	V$STAT1_01	0.739667	16632	(-)	NNNSANTTCCGGGAANTGNSN	TCACCTCTCCTGTAAGTGCCA
M00223	V$STAT_01	0.849761	16638	(+)	TTCCCRKAA	CTCCTGTAA
M00075	V$GATA1_01	0.780355	2	(+)	SNNGATNNNN	CTTGATCATC
M00075	V$GATA1_01	0.798618	2	(-)	SNNGATNNNN	CTTGATCATC
M00076	V$GATA2_01	0.791159	2	(-)	NNNGATRNNN	CTTGATCATC
M00127	V$GATA1_03	0.800833	3	(-)	RNSNNGATAANNGN	TTGATCATCAACCC
M00075	V$GATA1_01	0.827246	5	(-)	SNNGATNNNN	GATCATCAAC
M00127	V$GATA1_03	0.786624	76	(-)	RNSNNGATAANNGN	TATTTTATTTTCCT
M00126	V$GATA1_02	0.780312	91	(-)	NNNNNGATANKGNN	TGTCTCATCTTTAA
M00076	V$GATA2_01	0.795219	93	(-)	NNNGATRNNN	TCTCATCTTT
M00077	V$GATA3_01	0.865751	93	(-)	NNGATARNG	TCTCATCTT
M00075	V$GATA1_01	0.856367	137	(+)	SNNGATNNNN	TGTGATTGCA
M00076	V$GATA2_01	0.852503	137	(+)	NNNGATRNNN	TGTGATTGCA
M00077	V$GATA3_01	0.871068	138	(+)	NNGATARNG	GTGATTGCA
M00076	V$GATA2_01	0.788453	160	(-)	NNNGATRNNN	TCCTATGATA
M00075	V$GATA1_01	0.785785	163	(+)	SNNGATNNNN	TATGATATAC
M00076	V$GATA2_01	0.839423	163	(+)	NNNGATRNNN	TATGATATAC
M00126	V$GATA1_02	0.8225	198	(+)	NNNNNGATANKGNN	ATGTTGATACTATC
M00127	V$GATA1_03	0.856688	198	(+)	RNSNNGATAANNGN	ATGTTGATACTATC

M00075	V$GATA1_01	0.846002	200	(+)	SNNGATNNNN	GTTGATACTA
M00076	V$GATA2_01	0.797474	200	(+)	NNNGATRNNN	GTTGATACTA
M00126	V$GATA1_02	0.844375	203	(-)	NNNNNGATANKGNN	GATACTATCTATCT
M00127	V$GATA1_03	0.868447	203	(-)	RNSNNGATAANNGN	GATACTATCTATCT
M00075	V$GATA1_01	0.786278	205	(-)	SNNGATNNNN	TACTATCTAT
M00076	V$GATA2_01	0.7871	205	(-)	NNNGATRNNN	TACTATCTAT
M00077	V$GATA3_01	0.902525	205	(-)	NNGATARNG	TACTATCTA
M00203	V$GATA_C	0.853992	206	(-)	NGATAAGNMNN	ACTATCTATCT
M00126	V$GATA1_02	0.770937	207	(-)	NNNNNGATANKGNN	CTATCTATCTCAGA
M00128	V$GATA1_04	0.837316	207	(-)	NNCWGATARNNNN	CTATCTATCTCAG
M00075	V$GATA1_01	0.85538	209	(-)	SNNGATNNNN	ATCTATCTCA
M00076	V$GATA2_01	0.898963	209	(-)	NNNGATRNNN	ATCTATCTCA
M00077	V$GATA3_01	0.892335	209	(-)	NNGATARNG	ATCTATCTC
M00075	V$GATA1_01	0.851925	263	(+)	SNNGATNNNN	CCTGATCATT
M00075	V$GATA1_01	0.785785	263	(-)	SNNGATNNNN	CCTGATCATT
M00076	V$GATA2_01	0.820929	263	(+)	NNNGATRNNN	CCTGATCATT
M00076	V$GATA2_01	0.790708	263	(-)	NNNGATRNNN	CCTGATCATT
M00126	V$GATA1_02	0.779375	300	(+)	NNNNNGATANKGNN	ATGTAGATACATAA
M00127	V$GATA1_03	0.841009	300	(+)	RNSNNGATAANNGN	ATGTAGATACATAA
M00075	V$GATA1_01	0.821323	302	(+)	SNNGATNNNN	GTAGATACAT
M00076	V$GATA2_01	0.798376	302	(+)	NNNGATRNNN	GTAGATACAT
M00203	V$GATA_C	0.877912	304	(+)	NGATAAGNMNN	AGATACATAAC
M00127	V$GATA1_03	0.791034	400	(-)	RNSNNGATAANNGN	TTTGTTTTCATGGT
M00127	V$GATA1_03	0.792014	412	(+)	RNSNNGATAANNGN	GTGAAGATCATTTC
M00075	V$GATA1_01	0.806022	414	(+)	SNNGATNNNN	GAAGATCATT
M00076	V$GATA2_01	0.829499	414	(+)	NNNGATRNNN	GAAGATCATT
M00076	V$GATA2_01	0.782589	414	(-)	NNNGATRNNN	GAAGATCATT
M00075	V$GATA1_01	0.774926	488	(-)	SNNGATNNNN	AGGAAACACC
M00126	V$GATA1_02	0.797813	496	(+)	NNNNNGATANKGNN	CCAATGATAGACAA
M00127	V$GATA1_03	0.806712	496	(+)	RNSNNGATAANNGN	CCAATGATAGACAA
M00075	V$GATA1_01	0.824284	498	(+)	SNNGATNNNN	AATGATAGAC
M00076	V$GATA2_01	0.843482	498	(+)	NNNGATRNNN	AATGATAGAC
M00077	V$GATA3_01	0.851573	499	(+)	NNGATARNG	ATGATAGAC

M00203	V$GATA_C	0.884747	500	(+)	NGATAAGNMNN	TGATAGACAAT
M00128	V$GATA1_04	0.841605	509	(+)	NNCWGATARNNNN	ATTAGATATAAAT
M00077	V$GATA3_01	0.822331	511	(+)	NNGATARNG	TAGATATAA
M00203	V$GATA_C	0.835042	512	(+)	NGATAAGNMNN	AGATATAAATG
M00075	V$GATA1_01	0.789733	519	(+)	SNNGATNNNNN	AATGATTACT
M00076	V$GATA2_01	0.790257	519	(+)	NNNGATRNNN	AATGATTACT
M00127	V$GATA1_03	0.784664	532	(+)	RNSNNGATAANNGN	ATAAAGATGATTAA
M00075	V$GATA1_01	0.792695	534	(+)	SNNGATNNNNN	AAAGATGATT
M00076	V$GATA2_01	0.818674	534	(+)	NNNGATRNNN	AAAGATGATT
M00075	V$GATA1_01	0.779368	537	(+)	SNNGATNNNNN	GATGATTAAA
M00077	V$GATA3_01	0.821444	538	(+)	NNGATARNG	ATGATTAAA
M00075	V$GATA1_01	0.778875	548	(+)	SNNGATNNNNN	TTGGATATTT
M00076	V$GATA2_01	0.827244	548	(+)	NNNGATRNNN	TTGGATATTT
M00127	V$GATA1_03	0.823861	558	(+)	RNSNNGATAANNGN	ATAAGGATACAAAT
M00076	V$GATA2_01	0.817772	560	(+)	NNNGATRNNN	AAGGATACAA
M00203	V$GATA_C	0.864554	562	(+)	NGATAAGNMNN	GGATACAAATA
M00127	V$GATA1_03	0.826801	574	(+)	RNSNNGATAANNGN	ATGAAAATAATAAA
M00075	V$GATA1_01	0.84847	630	(+)	SNNGATNNNNN	GTAGATTCTG
M00076	V$GATA2_01	0.7871	630	(+)	NNNGATRNNN	GTAGATTCTG
M00077	V$GATA3_01	0.824546	631	(+)	NNGATARNG	TAGATTCTG
M00075	V$GATA1_01	0.789733	669	(-)	SNNGATNNNNN	AATAATCTGT
M00076	V$GATA2_01	0.790708	669	(-)	NNNGATRNNN	AATAATCTGT
M00077	V$GATA3_01	0.827204	669	(-)	NNGATARNG	AATAATCTG
M00077	V$GATA3_01	0.840496	716	(-)	NNGATARNG	TATAATCTA
M00127	V$GATA1_03	0.794463	728	(-)	RNSNNGATAANNGN	TTTCTTATTTATCT
M00203	V$GATA_C	0.911774	731	(-)	NGATAAGNMNN	CTTATTTATCT
M00126	V$GATA1_02	0.785313	732	(-)	NNNNNGATANKGNN	TTATTTATCTGAAA
M00128	V$GATA1_04	0.929228	732	(-)	NNCWGATARNNNN	TTATTTATCTGAA
M00075	V$GATA1_01	0.78233	734	(-)	SNNGATNNNNN	ATTTATCTGA
M00076	V$GATA2_01	0.847542	734	(-)	NNNGATRNNN	ATTTATCTGA
M00077	V$GATA3_01	0.840939	734	(-)	NNGATARNG	ATTTATCTG
M00126	V$GATA1_02	0.785938	815	(+)	NNNNNGATANKGNN	AACGTGATATGTTT
M00128	V$GATA1_04	0.834865	816	(+)	NNCWGATARNNNN	ACGTGATATGTTT

M00075	V$GATA1_01	0.941757	817	(+)	SNNGATNNNN	CGTGATATGT
M00076	V$GATA2_01	0.906631	817	(+)	NNNGATRNNN	CGTGATATGT
M00077	V$GATA3_01	0.849358	818	(+)	NNGATARNG	GTGATATGT
M00075	V$GATA1_01	0.844521	843	(-)	SNNGATNNNN	TTCAATCATC
M00076	V$GATA2_01	0.799729	843	(-)	NNNGATRNNN	TTCAATCATC
M00077	V$GATA3_01	0.849801	843	(-)	NNGATARNG	TTCAATCAT
M00127	V$GATA1_03	0.837825	844	(-)	RNSNNGATAANNGN	TCAATCATCTTCAT
M00075	V$GATA1_01	0.857354	846	(-)	SNNGATNNNN	AATCATCTTC
M00076	V$GATA2_01	0.865584	846	(-)	NNNGATRNNN	AATCATCTTC
M00203	V$GATA_C	0.878844	870	(-)	NGATAAGNMNN	TGGATATATCA
M00128	V$GATA1_04	0.810355	871	(-)	NNCWGATARNNNN	GGATATATCAACC
M00203	V$GATA_C	0.862069	871	(+)	NGATAAGNMNN	GGATATATCAA
M00075	V$GATA1_01	0.820829	873	(-)	SNNGATNNNN	ATATATCAAC
M00075	V$GATA1_01	0.813919	903	(+)	SNNGATNNNN	CTAGATCCTT
M00076	V$GATA2_01	0.784844	903	(-)	NNNGATRNNN	CTAGATCCTT
M00126	V$GATA1_02	0.782813	939	(+)	NNNNNGATANKGNN	GTCTACATAATGCA
M00127	V$GATA1_03	0.805488	984	(+)	RNSNNGATAANNGN	AGCAGGCTAAAGGA
M00126	V$GATA1_02	0.79375	991	(+)	NNNNNGATANKGNN	TAAAGGATAAAGAC
M00128	V$GATA1_04	0.86489	992	(+)	NNCWGATARNNNN	AAAGGATAAAGAC
M00076	V$GATA2_01	0.834461	993	(+)	NNNGATRNNN	AAGGATAAAG
M00077	V$GATA3_01	0.868409	994	(+)	NNGATARNG	AGGATAAAG
M00203	V$GATA_C	0.948742	995	(+)	NGATAAGNMNN	GGATAAAGACA
M00075	V$GATA1_01	0.810958	1001	(-)	SNNGATNNNN	AGACATCTTT
M00076	V$GATA2_01	0.852503	1001	(-)	NNNGATRNNN	AGACATCTTT
M00126	V$GATA1_02	0.867188	1017	(+)	NNNNNGATANKGNN	TTACAGATAAAATT
M00128	V$GATA1_04	0.89614	1018	(+)	NNCWGATARNNNN	TACAGATAAAATT
M00076	V$GATA2_01	0.785295	1019	(+)	NNNGATRNNN	ACAGATAAAA
M00077	V$GATA3_01	0.87284	1020	(+)	NNGATARNG	CAGATAAAA
M00203	V$GATA_C	0.90028	1021	(+)	NGATAAGNMNN	AGATAAAATTA
M00203	V$GATA_C	0.882572	1049	(-)	NGATAAGNMNN	TTAGTTTATCA
M00126	V$GATA1_02	0.8125	1050	(-)	NNNNNGATANKGNN	TAGTTTATCAATTT
M00127	V$GATA1_03	0.821901	1050	(-)	RNSNNGATAANNGN	TAGTTTATCAATTT
M00128	V$GATA1_04	0.815564	1050	(-)	NNCWGATARNNNN	TAGTTTATCAATT
M00127	V$GATA1_03	0.80499	1072	(-)	RNSNNGATAANNGN	CCAATGATCTACAG

		8				
M00076	V$GATA2_01	0.783491	1108	(+)	NNNGATRNNN	TGTCATATTT
M00126	V$GATA1_02	0.835313	1137	(+)	NNNNNGATANKGNN	ATATTGATACTTCT
M00127	V$GATA1_03	0.798383	1137	(+)	RNSNNGATAANNGN	ATATTGATACTTCT
M00075	V$GATA1_01	0.816881	1139	(+)	SNNGATNNNN	ATTGATACTT
M00076	V$GATA2_01	0.780334	1139	(+)	NNNGATRNNN	ATTGATACTT
M00203	V$GATA_C	0.89593	1160	(+)	NGATAAGNMNN	TGACAAGACCT
M00075	V$GATA1_01	0.785291	1183	(-)	SNNGATNNNN	CAGGATCAAA
M00126	V$GATA1_02	0.810312	1211	(+)	NNNNNGATANKGNN	AGTAAGATATTGGT
M00128	V$GATA1_04	0.860294	1212	(+)	NNCWGATARNNNN	GTAAGATATTGGT
M00075	V$GATA1_01	0.830701	1213	(+)	SNNGATNNNN	TAAGATATTG
M00076	V$GATA2_01	0.907533	1213	(+)	NNNGATRNNN	TAAGATATTG
M00077	V$GATA3_01	0.901639	1214	(+)	NNGATARNG	AAGATATTG
M00203	V$GATA_C	0.974216	1225	(-)	NGATAAGNMNN	ATTTCTTATCT
M00126	V$GATA1_02	0.842187	1226	(-)	NNNNNGATANKGNN	TTTCTTATCTTAGA
M00128	V$GATA1_04	0.871017	1226	(-)	NNCWGATARNNNN	TTTCTTATCTTAG
M00075	V$GATA1_01	0.777887	1228	(-)	SNNGATNNNN	TCTTATCTTA
M00076	V$GATA2_01	0.856112	1228	(-)	NNNGATRNNN	TCTTATCTTA
M00077	V$GATA3_01	0.919362	1228	(-)	NNGATARNG	TCTTATCTT
M00126	V$GATA1_02	0.821562	1233	(+)	NNNNNGATANKGNN	TCTTAGATAAGCAG
M00128	V$GATA1_04	0.839154	1234	(+)	NNCWGATARNNNN	CTTAGATAAGCAG
M00076	V$GATA2_01	0.800631	1235	(+)	NNNGATRNNN	TTAGATAAGC
M00077	V$GATA3_01	0.863093	1236	(+)	NNGATARNG	TAGATAAGC
M00203	V$GATA_C	0.912084	1237	(+)	NGATAAGNMNN	AGATAAGCAGT
M00127	V$GATA1_03	0.801813	1249	(+)	RNSNNGATAANNGN	AGCAAAATAATTTG
M00203	V$GATA_C	0.906803	1334	(-)	NGATAAGNMNN	AACTTTTATCA
M00126	V$GATA1_02	0.821875	1335	(-)	NNNNNGATANKGNN	ACTTTTATCATAAT
M00127	V$GATA1_03	0.825821	1335	(-)	RNSNNGATAANNGN	ACTTTTATCATAAT
M00128	V$GATA1_04	0.915135	1335	(-)	NNCWGATARNNNN	ACTTTTATCATAA
M00076	V$GATA2_01	0.798376	1337	(-)	NNNGATRNNN	TTTTATCATA
M00077	V$GATA3_01	0.855117	1337	(-)	NNGATARNG	TTTTATCAT
M00075	V$GATA1_01	0.841066	1364	(-)	SNNGATNNNN	AAAAATCAAG
M00203	V$GATA_C	0.838459	1400	(+)	NGATAAGNMNN	TTATAAGACCT

M00075	V$GATA1_01	0.78924	1426	(-)	SNNGATNNNN	ATAGATCTTG
M00076	V$GATA2_01	0.798827	1426	(-)	NNNGATRNNN	ATAGATCTTG
M00127	V$GATA1_03	0.797893	1430	(+)	RNSNNGATAANNGN	ATCTTGACAACCAT
M00127	V$GATA1_03	0.799363	1445	(-)	RNSNNGATAANNGN	AAACTTCTCATCCT
M00127	V$GATA1_03	0.790544	1448	(-)	RNSNNGATAANNGN	CTTCTCATCCTTAC
M00076	V$GATA2_01	0.809653	1450	(-)	NNNGATRNNN	TCTCATCCTT
M00077	V$GATA3_01	0.820558	1450	(-)	NNGATARNG	TCTCATCCT
M00127	V$GATA1_03	0.810632	1454	(-)	RNSNNGATAANNGN	ATCCTTACCTGTCT
M00128	V$GATA1_04	0.830882	1454	(-)	NNCWGATARNNNN	ATCCTTACCTGTC
M00076	V$GATA2_01	0.786649	1460	(-)	NNNGATRNNN	ACCTGTCTTG
M00075	V$GATA1_01	0.775913	1472	(+)	SNNGATNNNN	GCTGTTGCTC
M00075	V$GATA1_01	0.837611	1514	(+)	SNNGATNNNN	GAAGATTCTA
M00076	V$GATA2_01	0.825891	1514	(+)	NNNGATRNNN	GAAGATTCTA
M00075	V$GATA1_01	0.84156	1521	(+)	SNNGATNNNN	CTAGATTCTT
M00075	V$GATA1_01	0.829714	1537	(-)	SNNGATNNNN	AGTCATCTCA
M00076	V$GATA2_01	0.878214	1537	(-)	NNNGATRNNN	AGTCATCTCA
M00075	V$GATA1_01	0.823297	1565	(-)	SNNGATNNNN	TCTCATCAAG
M00126	V$GATA1_02	0.83	1575	(+)	NNNNNGATANKGNN	TGCAAGATATTGTA
M00075	V$GATA1_01	0.891412	1577	(+)	SNNGATNNNN	CAAGATATTG
M00076	V$GATA2_01	0.916554	1577	(+)	NNNGATRNNN	CAAGATATTG
M00077	V$GATA3_01	0.901639	1578	(+)	NNGATARNG	AAGATATTG
M00075	V$GATA1_01	0.83613	1600	(+)	SNNGATNNNN	TTTGATGGTC
M00076	V$GATA2_01	0.806044	1600	(+)	NNNGATRNNN	TTTGATGGTC
M00126	V$GATA1_02	0.800625	1627	(+)	NNNNNGATANKGNN	GTGTTGATATTAAA
M00127	V$GATA1_03	0.793974	1627	(+)	RNSNNGATAANNGN	GTGTTGATATTAAA
M00075	V$GATA1_01	0.824778	1629	(+)	SNNGATNNNN	GTTGATATTA
M00076	V$GATA2_01	0.786198	1629	(+)	NNNGATRNNN	GTTGATATTA
M00076	V$GATA2_01	0.815968	1646	(-)	NNNGATRNNN	AGTAATCCTT
M00203	V$GATA_C	0.834421	1688	(-)	NGATAAGNMNN	AAGACTTAACT
M00126	V$GATA1_02	0.775938	1758	(+)	NNNNNGATANKGNN	AAAGAAATAAGGTA
M00075	V$GATA1_01	0.810464	1789	(-)	SNNGATNNNN	GAGCATCAAA
M00076	V$GATA2_01	0.780785	1789	(-)	NNNGATRNNN	GAGCATCAAA
M00127	V$GATA1_03	0.817491	1797	(+)	RNSNNGATAANNGN	AAGAAGGTAATTAG

M00076	V$GATA2_01	0.786649	1838	(+)	NNNGATRNNN	TAACATATTT
M00076	V$GATA2_01	0.785295	1930	(+)	NNNGATRNNN	CACCATAGAC
M00075	V$GATA1_01	0.846496	1957	(-)	SNNGATNNNN	TGCCATCCCT
M00076	V$GATA2_01	0.881822	1957	(-)	NNNGATRNNN	TGCCATCCCT
M00077	V$GATA3_01	0.82942	1957	(-)	NNGATARNG	TGCCATCCC
M00075	V$GATA1_01	0.826259	1966	(+)	SNNGATNNNN	TGGGATTCTC
M00076	V$GATA2_01	0.860171	1966	(+)	NNNGATRNNN	TGGGATTCTC
M00077	V$GATA3_01	0.824989	2059	(-)	NNGATARNG	CCCACTCTT
M00127	V$GATA1_03	0.791524	2085	(-)	RNSNNGATAANNGN	CAGCCTACCAGGCT
M00075	V$GATA1_01	0.78924	2100	(-)	SNNGATNNNN	CTCCATCCAT
M00075	V$GATA1_01	0.805035	2109	(+)	SNNGATNNNN	TGGGATTTTC
M00076	V$GATA2_01	0.848895	2109	(+)	NNNGATRNNN	TGGGATTTTC
M00127	V$GATA1_03	0.822391	2187	(-)	RNSNNGATAANNGN	CAGTGTATCCACTT
M00075	V$GATA1_01	0.802073	2189	(-)	SNNGATNNNN	GTGTATCCAC
M00076	V$GATA2_01	0.809202	2189	(-)	NNNGATRNNN	GTGTATCCAC
M00075	V$GATA1_01	0.894867	2226	(+)	SNNGATNNNN	TGAGATGGCT
M00076	V$GATA2_01	0.929635	2226	(+)	NNNGATRNNN	TGAGATGGCT
M00077	V$GATA3_01	0.842712	2227	(+)	NNGATARNG	GAGATGGCT
M00127	V$GATA1_03	0.78099	2259	(+)	RNSNNGATAANNGN	GTACAGAAAGTTGG
M00075	V$GATA1_01	0.774926	2282	(+)	SNNGATNNNN	ACTGATTCAA
M00126	V$GATA1_02	0.8875	2324	(+)	NNNNNGATANKGNN	AACTAGATAATGTA
M00127	V$GATA1_03	0.865997	2324	(+)	RNSNNGATAANNGN	AACTAGATAATGTA
M00128	V$GATA1_04	0.827819	2325	(+)	NNCWGATARNNNN	ACTAGATAATGTA
M00075	V$GATA1_01	0.837117	2326	(+)	SNNGATNNNN	CTAGATAATG
M00076	V$GATA2_01	0.820027	2326	(+)	NNNGATRNNN	CTAGATAATG
M00077	V$GATA3_01	0.909171	2327	(+)	NNGATARNG	TAGATAATG
M00203	V$GATA_C	0.863312	2328	(+)	NGATAAGNMNN	AGATAATGTAA
M00126	V$GATA1_02	0.881563	2415	(+)	NNNNNGATANKGNN	CTCTAGATAATGAT
M00127	V$GATA1_03	0.874326	2415	(+)	RNSNNGATAANNGN	CTCTAGATAATGAT
M00128	V$GATA1_04	0.865502	2416	(+)	NNCWGATARNNNN	TCTAGATAATGAT
M00075	V$GATA1_01	0.837117	2417	(+)	SNNGATNNNN	CTAGATAATG
M00076	V$GATA2_01	0.820027	2417	(+)	NNNGATRNNN	CTAGATAATG
M00077	V$GATA3_01	0.909171	2418	(+)	NNGATARNG	TAGATAATG

M00203	V$GATA_C	0.899348	2419	(+)	NGATAAGNMNN	AGATAATGATA
M00126	V$GATA1_02	0.794687	2421	(+)	NNNNNGATANKGNN	ATAATGATAAATAA
M00127	V$GATA1_03	0.863057	2421	(+)	RNSNNGATAANNGN	ATAATGATAAATAA
M00128	V$GATA1_04	0.884498	2422	(+)	NNCWGATARNNNN	TAATGATAAATAA
M00075	V$GATA1_01	0.773939	2423	(+)	SNNGATNNNN	AATGATAAAT
M00076	V$GATA2_01	0.79567	2423	(+)	NNNGATRNNN	AATGATAAAT
M00077	V$GATA3_01	0.823217	2424	(+)	NNGATARNG	ATGATAAAT
M00203	V$GATA_C	0.932277	2425	(+)	NGATAAGNMNN	TGATAAATAAA
M00075	V$GATA1_01	0.857848	2441	(-)	SNNGATNNNN	AGGAATCAAC
M00076	V$GATA2_01	0.781687	2441	(-)	NNNGATRNNN	AGGAATCAAC
M00203	V$GATA_C	0.839391	2451	(+)	NGATAAGNMNN	TGACAAGAAAG
M00126	V$GATA1_02	0.857813	2469	(+)	NNNNNGATANKGNN	AATAAGATAATAGT
M00127	V$GATA1_03	0.844439	2469	(+)	RNSNNGATAANNGN	AATAAGATAATAGT
M00128	V$GATA1_04	0.912684	2470	(+)	NNCWGATARNNNN	ATAAGATAATAGT
M00076	V$GATA2_01	0.849797	2471	(+)	NNNGATRNNN	TAAGATAATA
M00077	V$GATA3_01	0.898538	2472	(+)	NNGATARNG	AAGATAATA
M00203	V$GATA_C	0.871389	2473	(+)	NGATAAGNMNN	AGATAATAGTT
M00075	V$GATA1_01	0.778875	2484	(+)	SNNGATNNNN	TTGGATATTT
M00076	V$GATA2_01	0.827244	2484	(+)	NNNGATRNNN	TTGGATATTT
M00128	V$GATA1_04	0.825368	2501	(-)	NNCWGATARNNNN	CAAGCTATCAAAT
M00075	V$GATA1_01	0.850938	2503	(-)	SNNGATNNNN	AGCTATCAAA
M00076	V$GATA2_01	0.857014	2503	(-)	NNNGATRNNN	AGCTATCAAA
M00077	V$GATA3_01	0.831192	2503	(-)	NNGATARNG	AGCTATCAA
M00126	V$GATA1_02	0.789062	2537	(+)	NNNNNGATANKGNN	GCTGAGATATTCTA
M00075	V$GATA1_01	0.833169	2539	(+)	SNNGATNNNN	TGAGATATTC
M00076	V$GATA2_01	0.898963	2539	(+)	NNNGATRNNN	TGAGATATTC
M00077	V$GATA3_01	0.852016	2540	(+)	NNGATARNG	GAGATATTC
M00126	V$GATA1_02	0.785	2558	(-)	NNNNNGATANKGNN	ACTCCTATTTTCTA
M00075	V$GATA1_01	0.794669	2586	(-)	SNNGATNNNN	TAGGATCACT
M00076	V$GATA2_01	0.848444	2586	(+)	NNNGATRNNN	TAGGATCACT
M00127	V$GATA1_03	0.800833	2594	(-)	RNSNNGATAANNGN	CTAATAATCCAGCT
M00075	V$GATA1_01	0.797631	2596	(-)	SNNGATNNNN	AATAATCCAG
M00075	V$GATA1_01	0.825271	2643	(-)	SNNGATNNNN	AAGCATCAAA

M00076	V$GATA2_01	0.784393	2643	(-)	NNNGATRNNN	AAGCATCAAA
M00203	V$GATA_C	0.838459	2656	(-)	NGATAAGNMNN	AGGGAATATCT
M00128	V$GATA1_04	0.85723	2657	(-)	NNCWGATARNNNN	GGGAATATCTGAC
M00075	V$GATA1_01	0.825271	2659	(-)	SNNGATNNNN	GAATATCTGA
M00076	V$GATA2_01	0.898963	2659	(-)	NNNGATRNNN	GAATATCTGA
M00077	V$GATA3_01	0.828977	2659	(-)	NNGATARNG	GAATATCTG
M00203	V$GATA_C	0.922647	2721	(-)	NGATAAGNMNN	AGTCCATATCT
M00126	V$GATA1_02	0.861875	2722	(-)	NNNNNGATANKGNN	GTCCATATCTTTTC
M00127	V$GATA1_03	0.793974	2722	(-)	RNSNNGATAANNGN	GTCCATATCTTTTC
M00128	V$GATA1_04	0.904105	2722	(-)	NNCWGATARNNNN	GTCCATATCTTTT
M00075	V$GATA1_01	0.83465	2724	(-)	SNNGATNNNN	CCATATCTTT
M00076	V$GATA2_01	0.881371	2724	(-)	NNNGATRNNN	CCATATCTTT
M00077	V$GATA3_01	0.922463	2724	(-)	NNGATARNG	CCATATCTT
M00203	V$GATA_C	0.850885	2734	(-)	NGATAAGNMNN	TCCTTTTATCA
M00126	V$GATA1_02	0.844063	2735	(-)	NNNNNGATANKGNN	CCTTTTATCAGTGA
M00127	V$GATA1_03	0.792994	2735	(-)	RNSNNGATAANNGN	CCTTTTATCAGTGA
M00128	V$GATA1_04	0.870098	2735	(-)	NNCWGATARNNNN	CCTTTTATCAGTG
M00077	V$GATA3_01	0.849358	2737	(-)	NNGATARNG	TTTTATCAG
M00126	V$GATA1_02	0.79125	2748	(+)	NNNNNGATANKGNN	AAACAGATATAGAT
M00128	V$GATA1_04	0.90288	2749	(+)	NNCWGATARNNNN	AACAGATATAGAT
M00075	V$GATA1_01	0.803554	2750	(+)	SNNGATNNNN	ACAGATATAG
M00076	V$GATA2_01	0.843031	2750	(+)	NNNGATRNNN	ACAGATATAG
M00077	V$GATA3_01	0.875942	2751	(+)	NNGATARNG	CAGATATAG
M00203	V$GATA_C	0.868593	2752	(+)	NGATAAGNMNN	AGATATAGATC
M00075	V$GATA1_01	0.808983	2756	(-)	SNNGATNNNN	ATAGATCCCC
M00076	V$GATA2_01	0.826793	2756	(-)	NNNGATRNNN	ATAGATCCCC
M00075	V$GATA1_01	0.82922	2772	(+)	SNNGATNNNN	ACAGATTCTT
M00076	V$GATA2_01	0.81687	2772	(+)	NNNGATRNNN	ACAGATTCTT
M00075	V$GATA1_01	0.831688	2799	(-)	SNNGATNNNN	AAACATCATT
M00076	V$GATA2_01	0.822282	2799	(-)	NNNGATRNNN	AAACATCATT
M00075	V$GATA1_01	0.816881	2833	(-)	SNNGATNNNN	ATAAATCTTG
M00076	V$GATA2_01	0.798827	2833	(-)	NNNGATRNNN	ATAAATCTTG
M00075	V$GATA1_01	0.891412	2859	(+)	SNNGATNNNN	GCAGATTGAG
M00076	V$GATA2_01	0.86197	2859	(+)	NNNGATRNNN	GCAGATTGAG

		6				
M00077	V$GATA3_01	0.902525	2860	(+)	NNGATARNG	CAGATTGAG
M00126	V$GATA1_02	0.794375	2890	(+)	NNNNNGATANKGNN	TTTATGATAAAAAA
M00128	V$GATA1_04	0.901654	2891	(+)	NNCWGATARNNNN	TTATGATAAAAAA
M00076	V$GATA2_01	0.798376	2892	(+)	NNNGATRNNN	TATGATAAAA
M00077	V$GATA3_01	0.855117	2893	(+)	NNGATARNG	ATGATAAAA
M00203	V$GATA_C	0.936626	2894	(+)	NGATAAGNMNN	TGATAAAAAAT
M00075	V$GATA1_01	0.780355	2904	(-)	SNNGATNNNN	TAAAATCCAG
M00127	V$GATA1_03	0.792014	2908	(+)	RNSNNGATAANNGN	ATCCAGACAAACAA
M00203	V$GATA_C	0.852439	2912	(+)	NGATAAGNMNN	AGACAAACAAT
M00075	V$GATA1_01	0.798124	2926	(+)	SNNGATNNNN	TCAGATTATT
M00076	V$GATA2_01	0.823184	2926	(+)	NNNGATRNNN	TCAGATTATT
M00077	V$GATA3_01	0.827204	2927	(+)	NNGATARNG	CAGATTATT
M00203	V$GATA_C	0.861758	2933	(-)	NGATAAGNMNN	ATTTTTTGTCT
M00127	V$GATA1_03	0.785154	2947	(-)	RNSNNGATAANNGN	TATATTTTCTCCAC
M00126	V$GATA1_02	0.83	2973	(+)	NNNNNGATANKGNN	CAGAAGATAATTTA
M00127	V$GATA1_03	0.856688	2973	(+)	RNSNNGATAANNGN	CAGAAGATAATTTA
M00128	V$GATA1_04	0.849265	2974	(+)	NNCWGATARNNNN	AGAAGATAATTTA
M00075	V$GATA1_01	0.857354	2975	(+)	SNNGATNNNN	GAAGATAATT
M00076	V$GATA2_01	0.894001	2975	(+)	NNNGATRNNN	GAAGATAATT
M00077	V$GATA3_01	0.866637	2976	(+)	NNGATARNG	AAGATAATT
M00203	V$GATA_C	0.85306	2977	(+)	NGATAAGNMNN	AGATAATTTAC
M00203	V$GATA_C	0.841876	2996	(-)	NGATAAGNMNN	TATTTTTGTCA
M00127	V$GATA1_03	0.785154	3014	(-)	RNSNNGATAANNGN	TTTTTTTTCCTCCC
M00126	V$GATA1_02	0.775313	3107	(+)	NNNNNGATANKGNN	CTTGTGATATTATT
M00075	V$GATA1_01	0.833169	3109	(+)	SNNGATNNNN	TGTGATATTA
M00076	V$GATA2_01	0.861525	3109	(+)	NNNGATRNNN	TGTGATATTA
M00077	V$GATA3_01	0.860434	3110	(+)	NNGATARNG	GTGATATTA
M00075	V$GATA1_01	0.814906	3177	(-)	SNNGATNNNN	AAACATCCTA
M00076	V$GATA2_01	0.880469	3177	(-)	NNNGATRNNN	AAACATCCTA
M00126	V$GATA1_02	0.85125	3180	(-)	NNNNNGATANKGNN	CATCCTATCAAGCA
M00127	V$GATA1_03	0.78197	3180	(-)	RNSNNGATAANNGN	CATCCTATCAAGCA
M00128	V$GATA1_04	0.844669	3180	(-)	NNCWGATARNNNN	CATCCTATCAAGC

M00075	V$GATA1_01	0.88845	3182	(-)	SNNGATNNNN	TCCTATCAAG
M00076	V$GATA2_01	0.823636	3182	(-)	NNNGATRNNN	TCCTATCAAG
M00077	V$GATA3_01	0.899867	3182	(-)	NNGATARNG	TCCTATCAA
M00075	V$GATA1_01	0.806515	3261	(+)	SNNGATNNNN	AGTGATGATA
M00076	V$GATA2_01	0.785747	3261	(+)	NNNGATRNNN	AGTGATGATA
M00077	V$GATA3_01	0.838724	3262	(+)	NNGATARNG	GTGATGATA
M00126	V$GATA1_02	0.807187	3262	(+)	NNNNNGATANKGNN	GTGATGATAAATTG
M00127	V$GATA1_03	0.870407	3262	(+)	RNSNNGATAANNGN	GTGATGATAAATTG
M00128	V$GATA1_04	0.867953	3263	(+)	NNCWGATARNNNN	TGATGATAAATTG
M00075	V$GATA1_01	0.838598	3264	(+)	SNNGATNNNN	GATGATAAAT
M00076	V$GATA2_01	0.84258	3264	(+)	NNNGATRNNN	GATGATAAAT
M00077	V$GATA3_01	0.823217	3265	(+)	NNGATARNG	ATGATAAAT
M00203	V$GATA_C	0.835663	3266	(+)	NGATAAGNMNN	TGATAAATTGC
M00126	V$GATA1_02	0.78125	3281	(+)	NNNNNGATANKGNN	ATATAGATTGTAGT
M00077	V$GATA3_01	0.868852	3284	(+)	NNGATARNG	TAGATTGTA
M00126	V$GATA1_02	0.77625	3303	(-)	NNNNNGATANKGNN	TTCCTTCTCTAGAA
M00127	V$GATA1_03	0.804508	3309	(+)	RNSNNGATAANNGN	CTCTAGAAAACAGC
M00075	V$GATA1_01	0.868707	3328	(-)	SNNGATNNNN	TCACATCCGC
M00076	V$GATA2_01	0.879567	3328	(-)	NNNGATRNNN	TCACATCCGC
M00126	V$GATA1_02	0.834063	3343	(-)	NNNNNGATANKGNN	TATAATATCTTCAA
M00075	V$GATA1_01	0.840079	3345	(-)	SNNGATNNNN	TAATATCTTC
M00076	V$GATA2_01	0.879116	3345	(-)	NNNGATRNNN	TAATATCTTC
M00077	V$GATA3_01	0.866637	3345	(-)	NNGATARNG	TAATATCTT
M00075	V$GATA1_01	0.787266	3371	(-)	SNNGATNNNN	ATTGATCTGC
M00076	V$GATA2_01	0.797474	3371	(-)	NNNGATRNNN	ATTGATCTGC
M00128	V$GATA1_04	0.824755	3471	(+)	NNCWGATARNNNN	ATAGGATATGTAT
M00075	V$GATA1_01	0.827246	3472	(+)	SNNGATNNNN	TAGGATATGT
M00076	V$GATA2_01	0.915201	3472	(+)	NNNGATRNNN	TAGGATATGT
M00203	V$GATA_C	0.880708	3474	(+)	NGATAAGNMNN	GGATATGTATA
M00075	V$GATA1_01	0.777887	3551	(+)	SNNGATNNNN	CTTGATCTCA
M00076	V$GATA2_01	0.796121	3551	(-)	NNNGATRNNN	CTTGATCTCA
M00126	V$GATA1_02	0.809375	3580	(+)	NNNNNGATANKGNN	AATTAGATACTCTA
M00075	V$GATA1_01	0.789733	3582	(+)	SNNGATNNNN	TTAGATACTC

M00076	V$GATA2_01	0.820478	3582	(+)	NNNGATRNNN	TTAGATACTC
M00203	V$GATA_C	0.862069	3586	(-)	NGATAAGNMNN	ATACTCTATCA
M00126	V$GATA1_02	0.81625	3587	(-)	NNNNNGATANKGNN	TACTCTATCACACA
M00128	V$GATA1_04	0.85723	3587	(-)	NNCWGATARNNNN	TACTCTATCACAC
M00075	V$GATA1_01	0.887463	3589	(-)	SNNGATNNNN	CTCTATCACA
M00076	V$GATA2_01	0.900767	3589	(-)	NNNGATRNNN	CTCTATCACA
M00077	V$GATA3_01	0.935755	3589	(-)	NNGATARNG	CTCTATCAC
M00075	V$GATA1_01	0.794176	3615	(-)	SNNGATNNNN	ATTAATCTTC
M00076	V$GATA2_01	0.789355	3615	(-)	NNNGATRNNN	ATTAATCTTC
M00126	V$GATA1_02	0.81125	3625	(-)	NNNNNGATANKGNN	CACCGTATCCACTG
M00127	V$GATA1_03	0.814552	3625	(-)	RNSNNGATAANNGN	CACCGTATCCACTG
M00075	V$GATA1_01	0.880059	3627	(-)	SNNGATNNNN	CCGTATCCAC
M00076	V$GATA2_01	0.87235	3627	(-)	NNNGATRNNN	CCGTATCCAC
M00077	V$GATA3_01	0.83385	3627	(-)	NNGATARNG	CCGTATCCA
M00077	V$GATA3_01	0.839167	3651	(-)	NNGATARNG	TTTAATCTG
M00127	V$GATA1_03	0.823616	3669	(+)	RNSNNGATAANNGN	AGCCAGACAGCAGA
M00075	V$GATA1_01	0.782823	3678	(+)	SNNGATNNNN	GCAGAGAGGT
M00127	V$GATA1_03	0.785644	3680	(+)	RNSNNGATAANNGN	AGAGAGGTAAGATT
M00075	V$GATA1_01	0.816881	3687	(+)	SNNGATNNNN	TAAGATTCTT
M00076	V$GATA2_01	0.841227	3687	(+)	NNNGATRNNN	TAAGATTCTT
M00076	V$GATA2_01	0.798376	3721	(+)	NNNGATRNNN	TCAGTTAGCT
M00076	V$GATA2_01	0.830853	3727	(-)	NNNGATRNNN	AGCTATGATA
M00128	V$GATA1_04	0.848346	3729	(+)	NNCWGATARNNNN	CTATGATAAATAC
M00075	V$GATA1_01	0.78233	3730	(+)	SNNGATNNNN	TATGATAAAT
M00076	V$GATA2_01	0.828146	3730	(+)	NNNGATRNNN	TATGATAAAT
M00077	V$GATA3_01	0.823217	3731	(+)	NNGATARNG	ATGATAAAT
M00203	V$GATA_C	0.952159	3732	(+)	NGATAAGNMNN	TGATAAATACT
M00127	V$GATA1_03	0.782705	3787	(-)	RNSNNGATAANNGN	AATTGTATCCAGGT
M00075	V$GATA1_01	0.785785	3789	(-)	SNNGATNNNN	TTGTATCCAG
M00127	V$GATA1_03	0.784664	3793	(+)	RNSNNGATAANNGN	ATCCAGGTACTCAG
M00126	V$GATA1_02	0.836563	3801	(+)	NNNNNGATANKGNN	ACTCAGATAGTTGA
M00127	V$GATA1_03	0.811612	3801	(+)	RNSNNGATAANNGN	ACTCAGATAGTTGA
M00128	V$GATA1_04	0.830576	3802	(+)	NNCWGATARNNNN	CTCAGATAGTTGA
M00075	V$GATA1_01	0.88696	3803	(+)	SNNGATNNNN	TCAGATAGTT

		9				
M00076	V$GATA2_01	0.939107	3803	(+)	NNNGATRNNN	TCAGATAGTT
M00077	V$GATA3_01	0.889233	3804	(+)	NNGATARNG	CAGATAGTT
M00203	V$GATA_C	0.833178	3805	(+)	NGATAAGNMNN	AGATAGTTGAT
M00075	V$GATA1_01	0.803554	3810	(+)	SNNGATNNNN	GTTGATTACC
M00127	V$GATA1_03	0.791034	3811	(-)	RNSNNGATAANNGN	TTGATTACCCTCTC
M00126	V$GATA1_02	0.795625	3816	(-)	NNNNNGATANKGNN	TACCCTCTCACTTC
M00077	V$GATA3_01	0.852459	3818	(-)	NNGATARNG	CCCTCTCAC
M00127	V$GATA1_03	0.795688	3823	(-)	RNSNNGATAANNGN	TCACTTCTCTGTTT
M00128	V$GATA1_04	0.812806	3823	(-)	NNCWGATARNNNN	TCACTTCTCTGTT
M00127	V$GATA1_03	0.794708	3830	(-)	RNSNNGATAANNGN	TCTGTTTTCATTAC
M00126	V$GATA1_02	0.840938	3868	(+)	NNNNNGATANKGNN	CATTTGATAAGGAA
M00128	V$GATA1_04	0.912684	3869	(+)	NNCWGATARNNNN	ATTTGATAAGGAA
M00075	V$GATA1_01	0.809477	3870	(+)	SNNGATNNNN	TTTGATAAGG
M00076	V$GATA2_01	0.806044	3870	(+)	NNNGATRNNN	TTTGATAAGG
M00077	V$GATA3_01	0.906513	3871	(+)	NNGATARNG	TTGATAAGG
M00203	V$GATA_C	0.932277	3872	(+)	NGATAAGNMNN	TGATAAGGAAG
M00126	V$GATA1_02	0.80875	3900	(+)	NNNNNGATANKGNN	GGCCTGATAAAGAA
M00127	V$GATA1_03	0.881676	3900	(+)	RNSNNGATAANNGN	GGCCTGATAAAGAA
M00128	V$GATA1_04	0.931985	3901	(+)	NNCWGATARNNNN	GCCTGATAAAGAA
M00075	V$GATA1_01	0.875123	3902	(+)	SNNGATNNNN	CCTGATAAAG
M00076	V$GATA2_01	0.858367	3902	(+)	NNNGATRNNN	CCTGATAAAG
M00077	V$GATA3_01	0.88436	3903	(+)	NNGATARNG	CTGATAAAG
M00203	V$GATA_C	0.905871	3904	(+)	NGATAAGNMNN	TGATAAAGAAG
M00075	V$GATA1_01	0.788746	3917	(-)	SNNGATNNNN	ACCAAACATG
M00075	V$GATA1_01	0.780849	3936	(+)	SNNGATNNNN	TCAGATTTTA
M00076	V$GATA2_01	0.8083	3936	(+)	NNNGATRNNN	TCAGATTTTA
M00077	V$GATA3_01	0.827204	3937	(+)	NNGATARNG	CAGATTTTA
M00075	V$GATA1_01	0.780849	3949	(-)	SNNGATNNNN	TAAAATCATA
M00076	V$GATA2_01	0.788904	3949	(-)	NNNGATRNNN	TAAAATCATA
M00203	V$GATA_C	0.849953	3967	(-)	NGATAAGNMNN	ACTAATTATCT
M00126	V$GATA1_02	0.839062	3968	(-)	NNNNNGATANKGNN	CTAATTATCTCTTA
M00127	V$GATA1_03	0.798138	3968	(-)	RNSNNGATAANNGN	CTAATTATCTCTTA

M00128	V$GATA1_04	0.892463	3968	(-)	NNCWGATARNNNN	CTAATTATCTCTT
M00075	V$GATA1_01	0.821323	3970	(-)	SNNGATNNNN	AATTATCTCT
M00076	V$GATA2_01	0.85521	3970	(-)	NNNGATRNNN	AATTATCTCT
M00077	V$GATA3_01	0.883917	3970	(-)	NNGATARNG	AATTATCTC
M00127	V$GATA1_03	0.78001	4007	(+)	RNSNNGATAANNGN	ACCTAAATAAATGG
M00203	V$GATA_C	0.903386	4144	(-)	NGATAAGNMNN	AAGTATTATCT
M00126	V$GATA1_02	0.850313	4145	(-)	NNNNNGATANKGNN	AGTATTATCTACTT
M00127	V$GATA1_03	0.882656	4145	(-)	RNSNNGATAANNGN	AGTATTATCTACTT
M00128	V$GATA1_04	0.863358	4145	(-)	NNCWGATARNNNN	AGTATTATCTACT
M00075	V$GATA1_01	0.785785	4147	(-)	SNNGATNNNN	TATTATCTAC
M00076	V$GATA2_01	0.782589	4147	(-)	NNNGATRNNN	TATTATCTAC
M00077	V$GATA3_01	0.874169	4147	(-)	NNGATARNG	TATTATCTA
M00075	V$GATA1_01	0.836624	4188	(-)	SNNGATNNNN	AAAAATCAAC
M00127	V$GATA1_03	0.785399	4351	(-)	RNSNNGATAANNGN	ACACTTATGCTGTC
M00076	V$GATA2_01	0.801534	4353	(-)	NNNGATRNNN	ACTTATGCTG
M00077	V$GATA3_01	0.875498	4370	(-)	NNGATARNG	CATAATCTA
M00126	V$GATA1_02	0.781875	4395	(-)	NNNNNGATANKGNN	TCCATTCTCATTAT
M00127	V$GATA1_03	0.804508	4395	(-)	RNSNNGATAANNGN	TCCATTCTCATTAT
M00076	V$GATA2_01	0.782589	4403	(-)	NNNGATRNNN	CATTATGATG
M00075	V$GATA1_01	0.811945	4406	(+)	SNNGATNNNN	TATGATGGAA
M00076	V$GATA2_01	0.82138	4406	(+)	NNNGATRNNN	TATGATGGAA
M00077	V$GATA3_01	0.829863	4407	(+)	NNGATARNG	ATGATGGAA
M00203	V$GATA_C	0.941286	4418	(-)	NGATAAGNMNN	TTCTCTTATCT
M00126	V$GATA1_02	0.957187	4419	(-)	NNNNNGATANKGNN	TCTCTTATCTCTAC
M00127	V$GATA1_03	0.917687	4419	(-)	RNSNNGATAANNGN	TCTCTTATCTCTAC
M00128	V$GATA1_04	0.917279	4419	(-)	NNCWGATARNNNN	TCTCTTATCTCTA
M00075	V$GATA1_01	0.798124	4421	(-)	SNNGATNNNN	TCTTATCTCT
M00076	V$GATA2_01	0.831755	4421	(-)	NNNGATRNNN	TCTTATCTCT
M00077	V$GATA3_01	0.936642	4421	(-)	NNGATARNG	TCTTATCTC
M00075	V$GATA1_01	0.851925	4439	(+)	SNNGATNNNN	CTGGATGTGT
M00076	V$GATA2_01	0.814163	4439	(+)	NNNGATRNNN	CTGGATGTGT
M00075	V$GATA1_01	0.784798	4524	(+)	SNNGATNNNN	AAAGATTTGC
M00076	V$GATA2_01	0.80018	4524	(+)	NNNGATRNNN	AAAGATTTGC

M00077	V$GATA3_01	0.821887	4525	(+)	NNGATARNG	AAGATTTGC
M00075	V$GATA1_01	0.807996	4555	(+)	SNNGATNNNN	AATGATTTTT
M00076	V$GATA2_01	0.786198	4555	(+)	NNNGATRNNN	AATGATTTTT
M00077	V$GATA3_01	0.864865	4567	(-)	NNGATARNG	TATAATCTT
M00127	V$GATA1_03	0.810632	4606	(+)	RNSNNGATAANNGN	AGAAAGGTAAGAAA
M00203	V$GATA_C	0.830693	4610	(+)	NGATAAGNMNN	AGGTAAGAAAT
M00075	V$GATA1_01	0.836624	4709	(-)	SNNGATNNNN	AAAAATCACT
M00076	V$GATA2_01	0.794317	4709	(-)	NNNGATRNNN	AAAAATCACT
M00075	V$GATA1_01	0.813425	4750	(-)	SNNGATNNNN	AGAGATCAAG
M00126	V$GATA1_02	0.799687	4754	(+)	NNNNNGATANKGNN	ATCAAGATAACTCA
M00127	V$GATA1_03	0.895884	4754	(+)	RNSNNGATAANNGN	ATCAAGATAACTCA
M00128	V$GATA1_04	0.882047	4755	(+)	NNCWGATARNNNN	TCAAGATAACTCA
M00075	V$GATA1_01	0.861797	4756	(+)	SNNGATNNNN	CAAGATAACT
M00076	V$GATA2_01	0.907533	4756	(+)	NNNGATRNNN	CAAGATAACT
M00077	V$GATA3_01	0.850687	4757	(+)	NNGATARNG	AAGATAACT
M00203	V$GATA_C	0.870146	4758	(+)	NGATAAGNMNN	AGATAACTCAG
M00127	V$GATA1_03	0.789319	4781	(+)	RNSNNGATAANNGN	AACAAGATGAAAGA
M00075	V$GATA1_01	0.786772	4783	(+)	SNNGATNNNN	CAAGATGAAA
M00076	V$GATA2_01	0.790257	4783	(+)	NNNGATRNNN	CAAGATGAAA
M00077	V$GATA3_01	0.824989	4784	(+)	NNGATARNG	AAGATGAAA
M00126	V$GATA1_02	0.844688	4788	(+)	NNNNNGATANKGNN	TGAAAGATAATAAT
M00127	V$GATA1_03	0.817246	4788	(+)	RNSNNGATAANNGN	TGAAAGATAATAAT
M00128	V$GATA1_04	0.889706	4789	(+)	NNCWGATARNNNN	GAAAGATAATAAT
M00076	V$GATA2_01	0.817321	4790	(+)	NNNGATRNNN	AAAGATAATA
M00077	V$GATA3_01	0.898538	4791	(+)	NNGATARNG	AAGATAATA
M00127	V$GATA1_03	0.821166	4791	(+)	RNSNNGATAANNGN	AAGATAATAATAGT
M00203	V$GATA_C	0.892513	4792	(+)	NGATAAGNMNN	AGATAATAATA
M00126	V$GATA1_02	0.829688	4886	(+)	NNNNNGATANKGNN	AAGGAGATAACGCA
M00127	V$GATA1_03	0.905683	4886	(+)	RNSNNGATAANNGN	AAGGAGATAACGCA
M00128	V$GATA1_04	0.908088	4887	(+)	NNCWGATARNNNN	AGGAGATAACGCA
M00075	V$GATA1_01	0.897335	4888	(+)	SNNGATNNNN	GGAGATAACG
M00076	V$GATA2_01	0.934145	4888	(+)	NNNGATRNNN	GGAGATAACG
M00077	V$GATA3_01	0.934869	4889	(+)	NNGATARNG	GAGATAACG

M00203	V$GATA_C	0.907114	4890	(+)	NGATAAGNMNN	AGATAACGCAA
M00126	V$GATA1_02	0.858125	4901	(+)	NNNNNGATANKGNN	GAAATGATATTGCA
M00128	V$GATA1_04	0.831495	4902	(+)	NNCWGATARNNNNN	AAATGATATTGCA
M00075	V$GATA1_01	0.84304	4903	(+)	SNNGATNNNN	AATGATATTG
M00076	V$GATA2_01	0.86378	4903	(+)	NNNGATRNNN	AATGATATTG
M00077	V$GATA3_01	0.878157	4904	(+)	NNGATARNG	ATGATATTG
M00075	V$GATA1_01	0.801086	4974	(+)	SNNGATNNNN	TAAGATACAT
M00076	V$GATA2_01	0.865584	4974	(+)	NNNGATRNNN	TAAGATACAT
M00203	V$GATA_C	0.836285	4976	(+)	NGATAAGNMNN	AGATACATTTC
M00128	V$GATA1_04	0.857843	4988	(+)	NNCWGATARNNNNN	TACAGATATCCAT
M00075	V$GATA1_01	0.816881	4989	(+)	SNNGATNNNN	ACAGATATCC
M00076	V$GATA2_01	0.885431	4989	(+)	NNNGATRNNN	ACAGATATCC
M00127	V$GATA1_03	0.788829	4989	(-)	RNSNNGATAANNGN	ACAGATATCCATCC
M00075	V$GATA1_01	0.770484	4991	(-)	SNNGATNNNN	AGATATCCAT
M00076	V$GATA2_01	0.813712	4991	(-)	NNNGATRNNN	AGATATCCAT
M00075	V$GATA1_01	0.777887	4995	(-)	SNNGATNNNN	ATCCATCCAT
M00127	V$GATA1_03	0.810142	5017	(-)	RNSNNGATAANNGN	CTTCTTTTCTGTCC
M00128	V$GATA1_04	0.823223	5017	(-)	NNCWGATARNNNNN	CTTCTTTTCTGTC
M00075	V$GATA1_01	0.780849	5038	(-)	SNNGATNNNN	GAACATCAAT
M00127	V$GATA1_03	0.822636	5063	(+)	RNSNNGATAANNGN	AGGAACATAAATGA
M00126	V$GATA1_02	0.782188	5070	(+)	NNNNNGATANKGNN	TAAATGATATTTAA
M00128	V$GATA1_04	0.818321	5071	(+)	NNCWGATARNNNNN	AAATGATATTTAA
M00075	V$GATA1_01	0.831688	5072	(+)	SNNGATNNNN	AATGATATTT
M00076	V$GATA2_01	0.850699	5072	(+)	NNNGATRNNN	AATGATATTT
M00075	V$GATA1_01	0.827739	5102	(-)	SNNGATNNNN	CAAAATCATA
M00076	V$GATA2_01	0.831755	5102	(-)	NNNGATRNNN	CAAAATCATA
M00077	V$GATA3_01	0.844484	5102	(-)	NNGATARNG	CAAAATCAT
M00075	V$GATA1_01	0.787759	5168	(+)	SNNGATNNNN	AGTGATGAAC
M00126	V$GATA1_02	0.881563	5177	(+)	NNNNNGATANKGNN	CTCAAGATAATGAA
M00127	V$GATA1_03	0.890495	5177	(+)	RNSNNGATAANNGN	CTCAAGATAATGAA
M00128	V$GATA1_04	0.890319	5178	(+)	NNCWGATARNNNNN	TCAAGATAATGAA
M00075	V$GATA1_01	0.873149	5179	(+)	SNNGATNNNN	CAAGATAATG
M00076	V$GATA2_01	0.901669	5179	(+)	NNNGATRNNN	CAAGATAATG

M00077	V$GATA3_01	0.93354	5180	(+)	NNGATARNG	AAGATAATG
M00203	V$GATA_C	0.902144	5181	(+)	NGATAAGNMNN	AGATAATGAAA
M00127	V$GATA1_03	0.785154	5186	(+)	RNSNNGATAANNGN	ATGAAAATACGAAA
M00076	V$GATA2_01	0.80424	5212	(-)	NNNGATRNNN	TGGTATGTCG
M00075	V$GATA1_01	0.810958	5304	(+)	SNNGATNNNN	ACTGATGAGA
M00076	V$GATA2_01	0.792061	5304	(+)	NNNGATRNNN	ACTGATGAGA
M00077	V$GATA3_01	0.836509	5305	(+)	NNGATARNG	CTGATGAGA
M00076	V$GATA2_01	0.794317	5309	(+)	NNNGATRNNN	TGAGATCTAC
M00126	V$GATA1_02	0.80125	5364	(+)	NNNNNGATANKGNN	GAAGAGACAAGGCA
M00203	V$GATA_C	0.899969	5368	(+)	NGATAAGNMNN	AGACAAGGCAT
M00126	V$GATA1_02	0.828125	5393	(+)	NNNNNGATANKGNN	GAATGGATAAAAGA
M00127	V$GATA1_03	0.843704	5393	(+)	RNSNNGATAANNGN	GAATGGATAAAAGA
M00203	V$GATA_C	0.900901	5397	(+)	NGATAAGNMNN	GGATAAAAGAA
M00127	V$GATA1_03	0.784664	5432	(-)	RNSNNGATAANNGN	CAGACTATTTTCAC
M00075	V$GATA1_01	0.833169	5444	(+)	SNNGATNNNN	ACTGATCGCC
M00075	V$GATA1_01	0.861797	5444	(-)	SNNGATNNNN	ACTGATCGCC
M00076	V$GATA2_01	0.846189	5444	(+)	NNNGATRNNN	ACTGATCGCC
M00076	V$GATA2_01	0.840776	5444	(-)	NNNGATRNNN	ACTGATCGCC
M00128	V$GATA1_04	0.85723	5491	(-)	NNCWGATARNNNN	CAATATATCTGCT
M00075	V$GATA1_01	0.856861	5493	(-)	SNNGATNNNN	ATATATCTGC
M00076	V$GATA2_01	0.876861	5493	(-)	NNNGATRNNN	ATATATCTGC
M00126	V$GATA1_02	0.831562	5645	(-)	NNNNNGATANKGNN	CTAAGTATCTTTTC
M00127	V$GATA1_03	0.83268	5645	(-)	RNSNNGATAANNGN	CTAAGTATCTTTTC
M00075	V$GATA1_01	0.832182	5647	(-)	SNNGATNNNN	AAGTATCTTT
M00076	V$GATA2_01	0.873252	5647	(-)	NNNGATRNNN	AAGTATCTTT
M00126	V$GATA1_02	0.786563	5661	(+)	NNNNNGATANKGNN	GGCAGGATATTGGG
M00127	V$GATA1_03	0.839784	5661	(+)	RNSNNGATAANNGN	GGCAGGATATTGGG
M00075	V$GATA1_01	0.886969	5663	(+)	SNNGATNNNN	CAGGATATTG
M00076	V$GATA2_01	0.930988	5663	(+)	NNNGATRNNN	CAGGATATTG
M00077	V$GATA3_01	0.856447	5664	(+)	NNGATARNG	AGGATATTG
M00075	V$GATA1_01	0.816881	5691	(+)	SNNGATNNNN	TAAGATTCTT
M00076	V$GATA2_01	0.841227	5691	(+)	NNNGATRNNN	TAAGATTCTT
M00127	V$GATA1_03	0.791034	5725	(+)	RNSNNGATAANNGN	ATAATGAAAAGAAA

M00075	V$GATA1_01	0.814906	5747	(-)	SNNGATNNNN	AAATATCCTA
M00076	V$GATA2_01	0.908886	5747	(-)	NNNGATRNNN	AAATATCCTA
M00075	V$GATA1_01	0.785785	5753	(-)	SNNGATNNNN	CCTAATCAAA
M00077	V$GATA3_01	0.87284	5753	(-)	NNGATARNG	CCTAATCAA
M00126	V$GATA1_02	0.784375	5758	(-)	NNNNNGATANKGNN	TCAAATATCCTTTA
M00075	V$GATA1_01	0.806515	5760	(-)	SNNGATNNNN	AAATATCCTT
M00076	V$GATA2_01	0.87641	5760	(-)	NNNGATRNNN	AAATATCCTT
M00075	V$GATA1_01	0.83613	5771	(-)	SNNGATNNNN	AAGTATCCTA
M00076	V$GATA2_01	0.920162	5771	(-)	NNNGATRNNN	AAGTATCCTA
M00127	V$GATA1_03	0.791034	5775	(-)	RNSNNGATAANNGN	ATCCTAATCAAGAT
M00075	V$GATA1_01	0.846496	5777	(-)	SNNGATNNNN	CCTAATCAAG
M00077	V$GATA3_01	0.87284	5777	(-)	NNGATARNG	CCTAATCAA
M00127	V$GATA1_03	0.796178	5781	(+)	RNSNNGATAANNGN	ATCAAGATGAATGA
M00075	V$GATA1_01	0.82231	5783	(+)	SNNGATNNNN	CAAGATGAAT
M00076	V$GATA2_01	0.820027	5783	(+)	NNNGATRNNN	CAAGATGAAT
M00128	V$GATA1_04	0.871936	5819	(+)	NNCWGATARNNNNN	AACAGATATCTCT
M00075	V$GATA1_01	0.831688	5820	(+)	SNNGATNNNN	ACAGATATCT
M00076	V$GATA2_01	0.889039	5820	(+)	NNNGATRNNN	ACAGATATCT
M00126	V$GATA1_02	0.81125	5820	(-)	NNNNNGATANKGNN	ACAGATATCTCTAA
M00128	V$GATA1_04	0.835784	5820	(-)	NNCWGATARNNNNN	ACAGATATCTCTA
M00075	V$GATA1_01	0.839585	5822	(-)	SNNGATNNNN	AGATATCTCT
M00076	V$GATA2_01	0.889039	5822	(-)	NNNGATRNNN	AGATATCTCT
M00077	V$GATA3_01	0.836066	5822	(-)	NNGATARNG	AGATATCTC
M00127	V$GATA1_03	0.888045	5875	(+)	RNSNNGATAANNGN	GGCTGGATAACTAA
M00075	V$GATA1_01	0.821323	5877	(+)	SNNGATNNNN	CTGGATAACT
M00076	V$GATA2_01	0.840325	5877	(+)	NNNGATRNNN	CTGGATAACT
M00203	V$GATA_C	0.882883	5879	(+)	NGATAAGNMNN	GGATAACTAAA
M00076	V$GATA2_01	0.802436	5891	(+)	NNNGATRNNN	CAACATAGTA
M00075	V$GATA1_01	0.826752	5918	(-)	SNNGATNNNN	ATGGATCATC
M00076	V$GATA2_01	0.80424	5918	(-)	NNNGATRNNN	ATGGATCATC
M00075	V$GATA1_01	0.814906	5921	(-)	SNNGATNNNN	GATCATCTCA
M00076	V$GATA2_01	0.855661	5921	(-)	NNNGATRNNN	GATCATCTCA
M00077	V$GATA3_01	0.830306	5921	(-)	NNGATARNG	GATCATCTC
M00127	V$GATA1_03	0.78099	5930	(-)	RNSNNGATAANNGN	ACTGCTCTCTGTAC

M00076	V$GATA2_01	0.818223	5944	(-)	NNNGATRNNN	TGCTATGGTG
M00127	V$GATA1_03	0.784664	5948	(+)	RNSNNGATAANNGN	ATGGTGATGGAAAC
M00075	V$GATA1_01	0.896841	5950	(+)	SNNGATNNNN	GGTGATGGAA
M00076	V$GATA2_01	0.843933	5950	(+)	NNNGATRNNN	GGTGATGGAA
M00077	V$GATA3_01	0.847142	5951	(+)	NNGATARNG	GTGATGGAA
M00127	V$GATA1_03	0.792504	5979	(+)	RNSNNGATAANNGN	ATGGAGAAAATTCA
M00203	V$GATA_C	0.846226	6039	(-)	NGATAAGNMNN	TTTATTTGTCA
M00126	V$GATA1_02	0.780312	6048	(-)	NNNNNGATANKGNN	CACATAATCTTTAA
M00075	V$GATA1_01	0.780355	6050	(-)	SNNGATNNNN	CATAATCTTT
M00076	V$GATA2_01	0.79567	6050	(-)	NNNGATRNNN	CATAATCTTT
M00077	V$GATA3_01	0.899867	6050	(-)	NNGATARNG	CATAATCTT
M00075	V$GATA1_01	0.787266	6108	(+)	SNNGATNNNN	GAAGTTAGGT
M00076	V$GATA2_01	0.792061	6108	(+)	NNNGATRNNN	GAAGTTAGGT
M00076	V$GATA2_01	0.785747	6113	(+)	NNNGATRNNN	TAGGTTAGTT
M00203	V$GATA_C	0.94315	6147	(-)	NGATAAGNMNN	ATGATTTATCA
M00126	V$GATA1_02	0.799063	6148	(-)	NNNNNGATANKGNN	TGATTTATCAAAAC
M00128	V$GATA1_04	0.855699	6148	(-)	NNCWGATARNNNNN	TGATTTATCAAAA
M00075	V$GATA1_01	0.815893	6166	(-)	SNNGATNNNN	AAAAATCTAC
M00127	V$GATA1_03	0.822146	6199	(+)	RNSNNGATAANNGN	ATGCAGATTAAAGA
M00075	V$GATA1_01	0.779368	6201	(+)	SNNGATNNNN	GCAGATTAAA
M00077	V$GATA3_01	0.839167	6202	(+)	NNGATARNG	CAGATTAAA
M00076	V$GATA2_01	0.794768	6223	(+)	NNNGATRNNN	TCAGTTAGCC
M00127	V$GATA1_03	0.802058	6223	(-)	RNSNNGATAANNGN	TCAGTTAGCCTGGT
M00127	V$GATA1_03	0.800343	6229	(+)	RNSNNGATAANNGN	AGCCTGGTAGGTAG
M00128	V$GATA1_04	0.820159	6257	(-)	NNCWGATARNNNNN	TTCCTTACCAGTT
M00203	V$GATA_C	0.894688	6315	(-)	NGATAAGNMNN	GGTAATTATCA
M00126	V$GATA1_02	0.800625	6316	(-)	NNNNNGATANKGNN	GTAATTATCATTTT
M00127	V$GATA1_03	0.888045	6316	(-)	RNSNNGATAANNGN	GTAATTATCATTTT
M00128	V$GATA1_04	0.887868	6316	(-)	NNCWGATARNNNNN	GTAATTATCATTT
M00075	V$GATA1_01	0.813425	6318	(-)	SNNGATNNNN	AATTATCATT
M00076	V$GATA2_01	0.835814	6318	(-)	NNNGATRNNN	AATTATCATT
M00077	V$GATA3_01	0.843155	6318	(-)	NNGATARNG	AATTATCAT
M00203	V$GATA_C	0.838459	6337	(-)	NGATAAGNMNN	AATGTTTGTCA

M00126	V$GATA1_02	0.798437	6365	(-)	NNNNNGATANKGNN	TCTCTTTTCTTTTC
M00127	V$GATA1_03	0.788339	6365	(-)	RNSNNGATAANNGN	TCTCTTTTCTTTTC
M00075	V$GATA1_01	0.83465	6380	(-)	SNNGATNNNN	AAGGATCAAG
M00075	V$GATA1_01	0.861303	6395	(+)	SNNGATNNNN	GAAGATGCTA
M00076	V$GATA2_01	0.861976	6395	(+)	NNNGATRNNN	GAAGATGCTA
M00127	V$GATA1_03	0.782705	6396	(+)	RNSNNGATAANNGN	AAGATGCTAAGGTA
M00076	V$GATA2_01	0.82995	6408	(+)	NNNGATRNNN	TAAGATCTTT
M00128	V$GATA1_04	0.840993	6437	(-)	NNCWGATARNNNN	TTATATATCATAA
M00075	V$GATA1_01	0.800592	6439	(-)	SNNGATNNNN	ATATATCATA
M00076	V$GATA2_01	0.843031	6439	(-)	NNNGATRNNN	ATATATCATA
M00126	V$GATA1_02	0.803438	6449	(+)	NNNNNGATANKGNN	AACAAGATATGCTC
M00127	V$GATA1_03	0.808672	6449	(+)	RNSNNGATAANNGN	AACAAGATATGCTC
M00128	V$GATA1_04	0.844975	6450	(+)	NNCWGATARNNNN	ACAAGATATGCTC
M00075	V$GATA1_01	0.877591	6451	(+)	SNNGATNNNN	CAAGATATGC
M00076	V$GATA2_01	0.90618	6451	(+)	NNNGATRNNN	CAAGATATGC
M00077	V$GATA3_01	0.85556	6452	(+)	NNGATARNG	AAGATATGC
M00076	V$GATA2_01	0.834461	6453	(-)	NNNGATRNNN	AGATATGCTC
M00203	V$GATA_C	0.867661	6453	(+)	NGATAAGNMNN	AGATATGCTCT
M00127	V$GATA1_03	0.83023	6474	(-)	RNSNNGATAANNGN	AAAGCTATCCGCTG
M00128	V$GATA1_04	0.811887	6474	(-)	NNCWGATARNNNN	AAAGCTATCCGCT
M00075	V$GATA1_01	0.938796	6476	(-)	SNNGATNNNN	AGCTATCCGC
M00076	V$GATA2_01	0.986919	6476	(-)	NNNGATRNNN	AGCTATCCGC
M00077	V$GATA3_01	0.82809	6476	(-)	NNGATARNG	AGCTATCCG
M00075	V$GATA1_01	0.810958	6494	(+)	SNNGATNNNN	TGCGATTGAA
M00076	V$GATA2_01	0.801534	6494	(+)	NNNGATRNNN	TGCGATTGAA
M00077	V$GATA3_01	0.860877	6495	(+)	NNGATARNG	GCGATTGAA
M00126	V$GATA1_02	0.771562	6514	(-)	NNNNNGATANKGNN	AGTGCTATCATACC
M00127	V$GATA1_03	0.78197	6514	(-)	RNSNNGATAANNGN	AGTGCTATCATACC
M00128	V$GATA1_04	0.886949	6514	(-)	NNCWGATARNNNN	AGTGCTATCATAC
M00075	V$GATA1_01	0.851431	6516	(-)	SNNGATNNNN	TGCTATCATA
M00076	V$GATA2_01	0.908886	6516	(-)	NNNGATRNNN	TGCTATCATA
M00077	V$GATA3_01	0.887461	6516	(-)	NNGATARNG	TGCTATCAT
M00076	V$GATA2_01	0.801985	6519	(+)	NNNGATRNNN	TATCATACCC
M00126	V$GATA1_02	0.842188	6546	(+)	NNNNNGATANKGNN	TGCCAGATAAAGTA

M00127	V$GATA1_03	0.783439	6546	(+)	RNSNNGATAANNGN	TGCCAGATAAAGTA
M00128	V$GATA1_04	0.902267	6547	(+)	NNCWGATARNNNNN	GCCAGATAAAGTA
M00075	V$GATA1_01	0.854393	6548	(+)	SNNGATNNNN	CCAGATAAAG
M00076	V$GATA2_01	0.869644	6548	(+)	NNNGATRNNN	CCAGATAAAG
M00077	V$GATA3_01	0.907842	6549	(+)	NNGATARNG	CAGATAAAG
M00203	V$GATA_C	0.90991	6550	(+)	NGATAAGNMNN	AGATAAAGTAA
M00128	V$GATA1_04	0.814645	6581	(+)	NNCWGATARNNNNN	ACATGATCAGGAG
M00075	V$GATA1_01	0.854886	6582	(+)	SNNGATNNNN	CATGATCAGG
M00075	V$GATA1_01	0.863277	6582	(-)	SNNGATNNNN	CATGATCAGG
M00076	V$GATA2_01	0.832206	6582	(+)	NNNGATRNNN	CATGATCAGG
M00076	V$GATA2_01	0.83401	6582	(-)	NNNGATRNNN	CATGATCAGG
M00075	V$GATA1_01	0.805528	6611	(-)	SNNGATNNNN	TAAAATCAAG
M00075	V$GATA1_01	0.825271	6656	(-)	SNNGATNNNN	GAACATCTGA
M00076	V$GATA2_01	0.870546	6656	(-)	NNNGATRNNN	GAACATCTGA
M00075	V$GATA1_01	0.782823	6682	(+)	SNNGATNNNN	CCTGTTTCTG
M00075	V$GATA1_01	0.789733	6690	(-)	SNNGATNNNN	TGTCATCCCA
M00076	V$GATA2_01	0.862878	6690	(-)	NNNGATRNNN	TGTCATCCCA
M00126	V$GATA1_02	0.832812	6696	(+)	NNNNNGATANKGNN	CCCAGGATAAGAGT
M00127	V$GATA1_03	0.89172	6696	(+)	RNSNNGATAANNGN	CCCAGGATAAGAGT
M00075	V$GATA1_01	0.834156	6698	(+)	SNNGATNNNN	CAGGATAAGA
M00076	V$GATA2_01	0.879567	6698	(+)	NNNGATRNNN	CAGGATAAGA
M00077	V$GATA3_01	0.874169	6699	(+)	NNGATARNG	AGGATAAGA
M00203	V$GATA_C	0.930413	6700	(+)	NGATAAGNMNN	GGATAAGAGTT
M00077	V$GATA3_01	0.837838	6711	(-)	NNGATARNG	CACTCTCTT
M00076	V$GATA2_01	0.789355	6770	(+)	NNNGATRNNN	TAGGATCAAT
M00076	V$GATA2_01	0.861976	6781	(-)	NNNGATRNNN	GGCTATGCTG
M00076	V$GATA2_01	0.799278	6805	(-)	NNNGATRNNN	AGTCATGCCC
M00076	V$GATA2_01	0.787551	6837	(+)	NNNGATRNNN	GAGCATGCCA
M00076	V$GATA2_01	0.788453	6837	(-)	NNNGATRNNN	GAGCATGCCA
M00075	V$GATA1_01	0.859329	6862	(-)	SNNGATNNNN	TACCATCTCA
M00076	V$GATA2_01	0.88092	6862	(-)	NNNGATRNNN	TACCATCTCA
M00077	V$GATA3_01	0.890563	6862	(-)	NNGATARNG	TACCATCTC
M00075	V$GATA1_01	0.846002	6904	(+)	SNNGATNNNN	AGTGATGCTA

M00076	V$GATA2_01	0.811908	6904	(+)	NNNGATRNNN	AGTGATGCTA
M00127	V$GATA1_03	0.78197	6907	(-)	RNSNNGATAANNGN	GATGCTATCCAACC
M00075	V$GATA1_01	0.790227	6909	(-)	SNNGATNNNN	TGCTATCCAA
M00076	V$GATA2_01	0.852954	6909	(-)	NNNGATRNNN	TGCTATCCAA
M00077	V$GATA3_01	0.841382	6909	(-)	NNGATARNG	TGCTATCCA
M00075	V$GATA1_01	0.894867	6917	(-)	SNNGATNNNN	AACCATCTCA
M00076	V$GATA2_01	0.91069	6917	(-)	NNNGATRNNN	AACCATCTCA
M00077	V$GATA3_01	0.858662	6917	(-)	NNGATARNG	AACCATCTC
M00075	V$GATA1_01	0.775913	6919	(-)	SNNGATNNNN	CCATCTCACC
M00075	V$GATA1_01	0.799605	6932	(-)	SNNGATNNNN	TGACATCCCT
M00076	V$GATA2_01	0.845286	6932	(-)	NNNGATRNNN	TGACATCCCT
M00075	V$GATA1_01	0.802073	6938	(-)	SNNGATNNNN	CCCTGTCTCC
M00076	V$GATA2_01	0.813261	6938	(-)	NNNGATRNNN	CCCTGTCTCC
M00075	V$GATA1_01	0.806022	6952	(-)	SNNGATNNNN	CTCAATCTTT
M00076	V$GATA2_01	0.806946	6952	(-)	NNNGATRNNN	CTCAATCTTT
M00077	V$GATA3_01	0.908285	6952	(-)	NNGATARNG	CTCAATCTT
M00075	V$GATA1_01	0.775913	6961	(-)	SNNGATNNNN	TCCCAGCACC
M00203	V$GATA_C	0.851196	6972	(-)	NGATAAGNMNN	AGGTCTTTTCT
M00075	V$GATA1_01	0.86772	6997	(-)	SNNGATNNNN	TTGCATCAGG
M00076	V$GATA2_01	0.813261	6997	(-)	NNNGATRNNN	TTGCATCAGG
M00075	V$GATA1_01	0.838105	7033	(-)	SNNGATNNNN	TAGCATCAGT
M00076	V$GATA2_01	0.811908	7033	(-)	NNNGATRNNN	TAGCATCAGT
M00075	V$GATA1_01	0.844521	7059	(+)	SNNGATNNNN	CAGGATTGAC
M00076	V$GATA2_01	0.846189	7059	(+)	NNNGATRNNN	CAGGATTGAC
M00075	V$GATA1_01	0.857848	7076	(+)	SNNGATNNNN	AGTGATTCCT
M00076	V$GATA2_01	0.824538	7076	(+)	NNNGATRNNN	AGTGATTCCT
M00076	V$GATA2_01	0.785747	7100	(+)	NNNGATRNNN	TAGCATGTTG
M00075	V$GATA1_01	0.797631	7103	(+)	SNNGATNNNN	CATGTTGGGC
M00203	V$GATA_C	0.89593	7142	(-)	NGATAAGNMNN	GTGTCATATCT
M00076	V$GATA2_01	0.802436	7143	(+)	NNNGATRNNN	TGTCATATCT
M00126	V$GATA1_02	0.859062	7143	(-)	NNNNNGATANKGNN	TGTCATATCTTTTA
M00128	V$GATA1_04	0.873468	7143	(-)	NNCWGATARNNNNN	TGTCATATCTTTT
M00075	V$GATA1_01	0.787759	7145	(-)	SNNGATNNNN	TCATATCTTT

M00076	V$GATA2_01	0.838521	7145	(-)	NNNGATRNNN	TCATATCTTT
M00077	V$GATA3_01	0.887461	7145	(-)	NNGATARNG	TCATATCTT
M00076	V$GATA2_01	0.852954	7228	(-)	NNNGATRNNN	GGCTATGCTA
M00075	V$GATA1_01	0.829714	7238	(-)	SNNGATNNNN	CCTAATCTCA
M00076	V$GATA2_01	0.84258	7238	(-)	NNNGATRNNN	CCTAATCTCA
M00077	V$GATA3_01	0.937971	7238	(-)	NNGATARNG	CCTAATCTC
M00075	V$GATA1_01	0.821323	7299	(+)	SNNGATNNNN	AGAGATGACT
M00076	V$GATA2_01	0.845737	7299	(+)	NNNGATRNNN	AGAGATGACT
M00127	V$GATA1_03	0.803283	7303	(-)	RNSNNGATAANNGN	ATGACTATTTTGCT
M00203	V$GATA_C	0.832867	7326	(-)	NGATAAGNMNN	AATCAATATCT
M00126	V$GATA1_02	0.7825	7327	(-)	NNNNNGATANKGNN	ATCAATATCTTAGT
M00075	V$GATA1_01	0.830701	7329	(-)	SNNGATNNNN	CAATATCTTA
M00076	V$GATA2_01	0.907533	7329	(-)	NNNGATRNNN	CAATATCTTA
M00077	V$GATA3_01	0.901639	7329	(-)	NNGATARNG	CAATATCTT
M00075	V$GATA1_01	0.837117	7355	(-)	SNNGATNNNN	AGAAATCAGA
M00076	V$GATA2_01	0.845737	7355	(-)	NNNGATRNNN	AGAAATCAGA
M00075	V$GATA1_01	0.770484	7360	(+)	SNNGATNNNN	TCAGATCCAT
M00076	V$GATA2_01	0.809202	7360	(+)	NNNGATRNNN	TCAGATCCAT
M00077	V$GATA3_01	0.885689	7417	(+)	NNGATARNG	AAGATTAGA
M00203	V$GATA_C	0.844672	7418	(+)	NGATAAGNMNN	AGATTAGAACT
M00075	V$GATA1_01	0.818855	7464	(+)	SNNGATNNNN	ATTGATTGTT
M00126	V$GATA1_02	0.810938	7548	(+)	NNNNNGATANKGNN	TTTCAGATAGCCCA
M00128	V$GATA1_04	0.893689	7549	(+)	NNCWGATARNNNN	TTCAGATAGCCCA
M00075	V$GATA1_01	0.872162	7550	(+)	SNNGATNNNN	TCAGATAGCC
M00076	V$GATA2_01	0.954443	7550	(+)	NNNGATRNNN	TCAGATAGCC
M00077	V$GATA3_01	0.873283	7551	(+)	NNGATARNG	CAGATAGCC
M00075	V$GATA1_01	0.812438	7568	(+)	SNNGATNNNN	TAGGATTCTT
M00076	V$GATA2_01	0.855661	7568	(+)	NNNGATRNNN	TAGGATTCTT
M00203	V$GATA_C	0.896241	7590	(-)	NGATAAGNMNN	CATTTTTATCA
M00126	V$GATA1_02	0.82125	7591	(-)	NNNNNGATANKGNN	ATTTTTATCACGAA
M00127	V$GATA1_03	0.788829	7591	(-)	RNSNNGATAANNGN	ATTTTTATCACGAA
M00128	V$GATA1_04	0.898284	7591	(-)	NNCWGATARNNNN	ATTTTTATCACGA
M00075	V$GATA1_01	0.83613	7593	(-)	SNNGATNNNN	TTTTATCACG

M00076	V$GATA2_01	0.815516	7593	(-)	NNNGATRNNN	TTTTATCACG
M00077	V$GATA3_01	0.872397	7593	(-)	NNGATARNG	TTTTATCAC
M00126	V$GATA1_02	0.834375	7633	(-)	NNNNNGATANKGNN	GAAAGTATCTGTAT
M00127	V$GATA1_03	0.840519	7633	(-)	RNSNNGATAANNGN	GAAAGTATCTGTAT
M00075	V$GATA1_01	0.852912	7635	(-)	SNNGATNNNN	AAGTATCTGT
M00076	V$GATA2_01	0.881371	7635	(-)	NNNGATRNNN	AAGTATCTGT
M00127	V$GATA1_03	0.826311	7682	(-)	RNSNNGATAANNGN	GTTATTATACTCCC
M00076	V$GATA2_01	0.79567	7701	(+)	NNNGATRNNN	CAACATATTT
M00075	V$GATA1_01	0.787266	7722	(-)	SNNGATNNNN	GACAATCCAA
M00076	V$GATA2_01	0.79567	7722	(-)	NNNGATRNNN	GACAATCCAA
M00075	V$GATA1_01	0.780355	7754	(-)	SNNGATNNNN	CTGAATCTTT
M00076	V$GATA2_01	0.781687	7754	(-)	NNNGATRNNN	CTGAATCTTT
M00077	V$GATA3_01	0.828977	7754	(-)	NNGATARNG	CTGAATCTT
M00075	V$GATA1_01	0.862784	7796	(-)	SNNGATNNNN	AGCAATCCAG
M00076	V$GATA2_01	0.827244	7796	(-)	NNNGATRNNN	AGCAATCCAG
M00075	V$GATA1_01	0.814413	7860	(+)	SNNGATNNNN	AATGATTCTC
M00076	V$GATA2_01	0.793866	7860	(+)	NNNGATRNNN	AATGATTCTC
M00127	V$GATA1_03	0.810632	7861	(-)	RNSNNGATAANNGN	ATGATTCTCTTTCT
M00076	V$GATA2_01	0.839423	7911	(-)	NNNGATRNNN	AGGTATGCCA
M00075	V$GATA1_01	0.864758	7935	(-)	SNNGATNNNN	AAGTATCCCA
M00076	V$GATA2_01	0.928281	7935	(-)	NNNGATRNNN	AAGTATCCCA
M00203	V$GATA_C	0.835974	8017	(-)	NGATAAGNMNN	ACTATGTATCT
M00076	V$GATA2_01	0.783942	8020	(-)	NNNGATRNNN	ATGTATCTAA
M00077	V$GATA3_01	0.86132	8025	(-)	NNGATARNG	TCTAATCTA
M00075	V$GATA1_01	0.82231	8029	(-)	SNNGATNNNN	ATCTATCCTA
M00076	V$GATA2_01	0.905277	8029	(-)	NNNGATRNNN	ATCTATCCTA
M00077	V$GATA3_01	0.829863	8029	(-)	NNGATARNG	ATCTATCCT
M00126	V$GATA1_02	0.783438	8051	(-)	NNNNNGATANKGNN	AAGGCTATCAACCC
M00127	V$GATA1_03	0.872366	8051	(-)	RNSNNGATAANNGN	AAGGCTATCAACCC
M00075	V$GATA1_01	0.892399	8053	(-)	SNNGATNNNN	GGCTATCAAC
M00076	V$GATA2_01	0.867839	8053	(-)	NNNGATRNNN	GGCTATCAAC
M00077	V$GATA3_01	0.831192	8053	(-)	NNGATARNG	GGCTATCAA
M00203	V$GATA_C	0.852128	8083	(-)	NGATAAGNMNN	ATTATTTGTCA

M00075	V$GATA1_01	0.787266	8104	(+)	SNNGATNNNN	AAAGATTTCT
M00076	V$GATA2_01	0.816419	8104	(+)	NNNGATRNNN	AAAGATTTCT
M00127	V$GATA1_03	0.801813	8118	(+)	RNSNNGATAANNGN	GGGCAAATAATCCT
M00075	V$GATA1_01	0.772952	8123	(-)	SNNGATNNNN	AATAATCCTA
M00076	V$GATA2_01	0.829499	8123	(-)	NNNGATRNNN	AATAATCCTA
M00203	V$GATA_C	0.884126	8125	(-)	NGATAAGNMNN	TAATCCTATCT
M00126	V$GATA1_02	0.895312	8126	(-)	NNNNNGATANKGNN	AATCCTATCTGGAT
M00127	V$GATA1_03	0.881676	8126	(-)	RNSNNGATAANNGN	AATCCTATCTGGAT
M00128	V$GATA1_04	0.906556	8126	(-)	NNCWGATARNNNN	AATCCTATCTGGA
M00075	V$GATA1_01	0.924482	8128	(-)	SNNGATNNNN	TCCTATCTGG
M00076	V$GATA2_01	0.924673	8128	(-)	NNNGATRNNN	TCCTATCTGG
M00077	V$GATA3_01	0.941958	8128	(-)	NNGATARNG	TCCTATCTG
M00127	V$GATA1_03	0.811857	8132	(+)	RNSNNGATAANNGN	ATCTGGATATCTGT
M00075	V$GATA1_01	0.839585	8134	(+)	SNNGATNNNN	CTGGATATCT
M00076	V$GATA2_01	0.85521	8134	(+)	NNNGATRNNN	CTGGATATCT
M00127	V$GATA1_03	0.784664	8134	(-)	RNSNNGATAANNGN	CTGGATATCTGTGG
M00075	V$GATA1_01	0.816881	8136	(-)	SNNGATNNNN	GGATATCTGT
M00076	V$GATA2_01	0.885431	8136	(-)	NNNGATRNNN	GGATATCTGT
M00076	V$GATA2_01	0.827695	8145	(-)	NNNGATRNNN	TGGTATCCAA
M00075	V$GATA1_01	0.877098	8156	(+)	SNNGATNNNN	CATGATTTTT
M00076	V$GATA2_01	0.827695	8156	(+)	NNNGATRNNN	CATGATTTTT
M00075	V$GATA1_01	0.877098	8184	(+)	SNNGATNNNN	CCAGATTTTT
M00076	V$GATA2_01	0.847091	8184	(+)	NNNGATRNNN	CCAGATTTTT
M00075	V$GATA1_01	0.781343	8200	(-)	SNNGATNNNN	TTAAATCTTG
M00203	V$GATA_C	0.835353	8202	(-)	NGATAAGNMNN	AAATCTTGTCA
M00127	V$GATA1_03	0.784174	8228	(-)	RNSNNGATAANNGN	ATGGCTATGCTTCT
M00076	V$GATA2_01	0.820478	8230	(-)	NNNGATRNNN	GGCTATGCTT
M00128	V$GATA1_04	0.853554	8258	(+)	NNCWGATARNNNN	ACATGATATATCC
M00075	V$GATA1_01	0.861303	8259	(+)	SNNGATNNNN	CATGATATAT
M00076	V$GATA2_01	0.852052	8259	(+)	NNNGATRNNN	CATGATATAT
M00203	V$GATA_C	0.867971	8260	(-)	NGATAAGNMNN	ATGATATATCC
M00203	V$GATA_C	0.884747	8261	(+)	NGATAAGNMNN	TGATATATCCT
M00076	V$GATA2_01	0.836265	8263	(-)	NNNGATRNNN	ATATATCCTT

M00075	V$GATA1_01	0.790721	8280	(-)	SNNGATNNNN	CTCAATCAAT
M00077	V$GATA3_01	0.860434	8280	(-)	NNGATARNG	CTCAATCAA
M00076	V$GATA2_01	0.812359	8308	(-)	NNNGATRNNN	TGGAATCCTT
M00127	V$GATA1_03	0.785154	8334	(+)	RNSNNGATAANNGN	GAGGAGAAAAAGAG
M00126	V$GATA1_02	0.775938	8340	(+)	NNNNNGATANKGNN	AAAAAGAGAAGGCA
M00077	V$GATA3_01	0.830306	8343	(+)	NNGATARNG	AAGAGAAGG
M00203	V$GATA_C	0.844051	8344	(+)	NGATAAGNMNN	AGAGAAGGCAT
M00076	V$GATA2_01	0.801083	8364	(-)	NNNGATRNNN	TGAAATCCTT
M00075	V$GATA1_01	0.793189	8391	(-)	SNNGATNNNN	CTTCATCCAG
M00075	V$GATA1_01	0.813425	8450	(-)	SNNGATNNNN	CACAATCCAA
M00076	V$GATA2_01	0.812359	8450	(-)	NNNGATRNNN	CACAATCCAA
M00077	V$GATA3_01	0.858662	8450	(-)	NNGATARNG	CACAATCCA
M00127	V$GATA1_03	0.787849	8477	(+)	RNSNNGATAANNGN	CTGGTGAAAGCAGT
M00126	V$GATA1_02	0.774062	8486	(+)	NNNNNGATANKGNN	GCAGTGAGAAGAGA
M00127	V$GATA1_03	0.789319	8491	(+)	RNSNNGATAANNGN	GAGAAGAGAAAAGA
M00126	V$GATA1_02	0.798438	8493	(+)	NNNNNGATANKGNN	GAAGAGAAAAGAGA
M00127	V$GATA1_03	0.788339	8493	(+)	RNSNNGATAANNGN	GAAGAGAAAAGAGA
M00075	V$GATA1_01	0.827246	8515	(+)	SNNGATNNNN	TTTGATTGTT
M00076	V$GATA2_01	0.807397	8522	(-)	NNNGATRNNN	GTTCATCTTA
M00203	V$GATA_C	0.87667	8524	(-)	NGATAAGNMNN	TCATCTTATCT
M00126	V$GATA1_02	0.850625	8525	(-)	NNNNNGATANKGNN	CATCTTATCTAAAT
M00127	V$GATA1_03	0.822146	8525	(-)	RNSNNGATAANNGN	CATCTTATCTAAAT
M00128	V$GATA1_04	0.875	8525	(-)	NNCWGATARNNNN	CATCTTATCTAAA
M00077	V$GATA3_01	0.894993	8527	(-)	NNGATARNG	TCTTATCTA
M00075	V$GATA1_01	0.793189	8558	(-)	SNNGATNNNN	TAAAATCAGT
M00126	V$GATA1_02	0.868125	8571	(+)	NNNNNGATANKGNN	CCACAGATAAGCTA
M00127	V$GATA1_03	0.846889	8571	(+)	RNSNNGATAANNGN	CCACAGATAAGCTA
M00128	V$GATA1_04	0.833946	8572	(+)	NNCWGATARNNNN	CACAGATAAGCTA
M00075	V$GATA1_01	0.810958	8573	(+)	SNNGATNNNN	ACAGATAAGC
M00076	V$GATA2_01	0.857916	8573	(+)	NNNGATRNNN	ACAGATAAGC
M00077	V$GATA3_01	0.881701	8574	(+)	NNGATARNG	CAGATAAGC
M00203	V$GATA_C	0.926064	8575	(+)	NGATAAGNMNN	AGATAAGCTAT
M00076	V$GATA2_01	0.839874	8580	(-)	NNNGATRNNN	AGCTATGATG

M00075	V$GATA1_01	0.852419	8583	(+)	SNNGATNNNN	TATGATGTGT
M00076	V$GATA2_01	0.861074	8583	(+)	NNNGATRNNN	TATGATGTGT
M00203	V$GATA_C	0.907425	8605	(-)	NGATAAGNMNN	CATTTTTATCT
M00126	V$GATA1_02	0.839062	8606	(-)	NNNNNGATANKGNN	ATTTTTATCTTGAA
M00127	V$GATA1_03	0.798628	8606	(-)	RNSNNGATAANNGN	ATTTTTATCTTGAA
M00128	V$GATA1_04	0.912377	8606	(-)	NNCWGATARNNNN	ATTTTTATCTTGA
M00075	V$GATA1_01	0.786772	8608	(-)	SNNGATNNNN	TTTTATCTTG
M00076	V$GATA2_01	0.818674	8608	(-)	NNNGATRNNN	TTTTATCTTG
M00077	V$GATA3_01	0.8786	8608	(-)	NNGATARNG	TTTTATCTT
M00128	V$GATA1_04	0.822304	8690	(+)	NNCWGATARNNNN	GTGAGATATATTC
M00075	V$GATA1_01	0.80849	8691	(+)	SNNGATNNNN	TGAGATATAT
M00076	V$GATA2_01	0.862427	8691	(+)	NNNGATRNNN	TGAGATATAT
M00077	V$GATA3_01	0.832078	8692	(+)	NNGATARNG	GAGATATAT
M00203	V$GATA_C	0.846226	8693	(+)	NGATAAGNMNN	AGATATATTCA
M00127	V$GATA1_03	0.792014	8716	(-)	RNSNNGATAANNGN	TATATTAACATCAT
M00075	V$GATA1_01	0.860809	8721	(-)	SNNGATNNNN	TAACATCATC
M00076	V$GATA2_01	0.839423	8721	(-)	NNNGATRNNN	TAACATCATC
M00075	V$GATA1_01	0.832675	8724	(-)	SNNGATNNNN	CATCATCCAG
M00076	V$GATA2_01	0.806044	8724	(-)	NNNGATRNNN	CATCATCCAG
M00075	V$GATA1_01	0.875617	8731	(+)	SNNGATNNNN	CAGGATATCT
M00076	V$GATA2_01	0.936852	8731	(+)	NNNGATRNNN	CAGGATATCT
M00128	V$GATA1_04	0.811887	8731	(-)	NNCWGATARNNNN	CAGGATATCTTAA
M00075	V$GATA1_01	0.804541	8733	(-)	SNNGATNNNN	GGATATCTTA
M00076	V$GATA2_01	0.909788	8733	(-)	NNNGATRNNN	GGATATCTTA
M00075	V$GATA1_01	0.873149	8798	(+)	SNNGATNNNN	GAAGATGTGC
M00076	V$GATA2_01	0.883175	8798	(+)	NNNGATRNNN	GAAGATGTGC
M00203	V$GATA_C	0.859273	8822	(-)	NGATAAGNMNN	CTGGGTTATCT
M00126	V$GATA1_02	0.81125	8823	(-)	NNNNNGATANKGNN	TGGGTTATCTGGTA
M00127	V$GATA1_03	0.796178	8823	(-)	RNSNNGATAANNGN	TGGGTTATCTGGTA
M00128	V$GATA1_04	0.912071	8823	(-)	NNCWGATARNNNN	TGGGTTATCTGGT
M00075	V$GATA1_01	0.86772	8825	(-)	SNNGATNNNN	GGTTATCTGG
M00076	V$GATA2_01	0.912043	8825	(-)	NNNGATRNNN	GGTTATCTGG
M00077	V$GATA3_01	0.844927	8825	(-)	NNGATARNG	GGTTATCTG

M00075	V$GATA1_01	0.903258	8866	(-)	SNNGATNNNN	ATGCATCAGG
M00076	V$GATA2_01	0.843031	8866	(-)	NNNGATRNNN	ATGCATCAGG
M00126	V$GATA1_02	0.819375	8893	(+)	NNNNNGATANKGNN	GTGTTGATAAATTA
M00127	V$GATA1_03	0.83807	8893	(+)	RNSNNGATAANNGN	GTGTTGATAAATTA
M00128	V$GATA1_04	0.815257	8894	(+)	NNCWGATARNNNN	TGTTGATAAATTA
M00075	V$GATA1_01	0.802567	8895	(+)	SNNGATNNNN	GTTGATAAAT
M00203	V$GATA_C	0.899348	8897	(+)	NGATAAGNMNN	TGATAAATTAT
M00203	V$GATA_C	0.863933	8899	(-)	NGATAAGNMNN	ATAAATTATCT
M00126	V$GATA1_02	0.849375	8900	(-)	NNNNNGATANKGNN	TAAATTATCTCTCC
M00127	V$GATA1_03	0.865997	8900	(-)	RNSNNGATAANNGN	TAAATTATCTCTCC
M00128	V$GATA1_04	0.829044	8900	(-)	NNCWGATARNNNN	TAAATTATCTCTC
M00075	V$GATA1_01	0.821323	8902	(-)	SNNGATNNNN	AATTATCTCT
M00076	V$GATA2_01	0.85521	8902	(-)	NNNGATRNNN	AATTATCTCT
M00077	V$GATA3_01	0.883917	8902	(-)	NNGATARNG	AATTATCTC
M00127	V$GATA1_03	0.791034	8910	(-)	RNSNNGATAANNGN	CTCCTTTTCAATAT
M00126	V$GATA1_02	0.823438	8916	(-)	NNNNNGATANKGNN	TTCAATATCTGCTA
M00128	V$GATA1_04	0.873775	8916	(-)	NNCWGATARNNNN	TTCAATATCTGCT
M00075	V$GATA1_01	0.9077	8918	(-)	SNNGATNNNN	CAATATCTGC
M00076	V$GATA2_01	0.930086	8918	(-)	NNNGATRNNN	CAATATCTGC
M00077	V$GATA3_01	0.895879	8918	(-)	NNGATARNG	CAATATCTG
M00075	V$GATA1_01	0.793682	8951	(+)	SNNGATNNNN	ACAGATTCTA
M00076	V$GATA2_01	0.7871	8951	(+)	NNNGATRNNN	ACAGATTCTA
M00075	V$GATA1_01	0.82231	8968	(+)	SNNGATNNNN	ACTGATCCCT
M00075	V$GATA1_01	0.777887	8968	(-)	SNNGATNNNN	ACTGATCCCT
M00076	V$GATA2_01	0.824538	8968	(+)	NNNGATRNNN	ACTGATCCCT
M00076	V$GATA2_01	0.811457	8968	(-)	NNNGATRNNN	ACTGATCCCT
M00203	V$GATA_C	0.836906	8992	(+)	NGATAAGNMNN	TGACAAGTTTT
M00077	V$GATA3_01	0.862206	9002	(-)	NNGATARNG	TTTAATCTC
M00075	V$GATA1_01	0.840079	9009	(+)	SNNGATNNNN	TCAGATATTT
M00076	V$GATA2_01	0.902571	9009	(+)	NNNGATRNNN	TCAGATATTT
M00077	V$GATA3_01	0.828977	9010	(+)	NNGATARNG	CAGATATTT
M00203	V$GATA_C	0.830382	9011	(+)	NGATAAGNMNN	AGATATTTAAT
M00076	V$GATA2_01	0.814614	9062	(+)	NNNGATRNNN	TCAGATCTGA

M00076	V$GATA2_01	0.814614	9062	(-)	NNNGATRNNN	TCAGATCTGA
M00128	V$GATA1_04	0.822304	9066	(+)	NNCWGATARNNNN	ATCTGATTAGCAC
M00075	V$GATA1_01	0.816387	9067	(+)	SNNGATNNNN	TCTGATTAGC
M00076	V$GATA2_01	0.814614	9067	(+)	NNNGATRNNN	TCTGATTAGC
M00077	V$GATA3_01	0.824546	9068	(+)	NNGATARNG	CTGATTAGC
M00077	V$GATA3_01	0.82942	9099	(+)	NNGATARNG	TAGATTTGA
M00076	V$GATA2_01	0.809653	9144	(-)	NNNGATRNNN	CGGCATGTGA
M00126	V$GATA1_02	0.78875	9147	(+)	NNNNNGATANKGNN	CATGTGATATTAGA
M00128	V$GATA1_04	0.831495	9148	(+)	NNCWGATARNNNN	ATGTGATATTAGA
M00075	V$GATA1_01	0.833169	9149	(+)	SNNGATNNNN	TGTGATATTA
M00076	V$GATA2_01	0.861525	9149	(+)	NNNGATRNNN	TGTGATATTA
M00077	V$GATA3_01	0.860434	9150	(+)	NNGATARNG	GTGATATTA
M00126	V$GATA1_02	0.830313	9154	(+)	NNNNNGATANKGNN	TATTAGATAGTTTC
M00128	V$GATA1_04	0.818015	9155	(+)	NNCWGATARNNNN	ATTAGATAGTTTC
M00075	V$GATA1_01	0.830207	9156	(+)	SNNGATNNNN	TTAGATAGTT
M00076	V$GATA2_01	0.849346	9156	(+)	NNNGATRNNN	TTAGATAGTT
M00077	V$GATA3_01	0.870625	9157	(+)	NNGATARNG	TAGATAGTT
M00203	V$GATA_C	0.975148	9164	(-)	NGATAAGNMNN	TTTCCTTATCT
M00126	V$GATA1_02	0.8725	9165	(-)	NNNNNGATANKGNN	TTCCTTATCTGCTG
M00127	V$GATA1_03	0.897844	9165	(-)	RNSNNGATAANNGN	TTCCTTATCTGCTG
M00128	V$GATA1_04	0.975184	9165	(-)	NNCWGATARNNNN	TTCCTTATCTGCT
M00075	V$GATA1_01	0.901777	9167	(-)	SNNGATNNNN	CCTTATCTGC
M00076	V$GATA2_01	0.921516	9167	(-)	NNNGATRNNN	CCTTATCTGC
M00077	V$GATA3_01	0.948604	9167	(-)	NNGATARNG	CCTTATCTG
M00076	V$GATA2_01	0.811908	9253	(+)	NNNGATRNNN	AGACATAGTG
M00126	V$GATA1_02	0.844063	9287	(-)	NNNNNGATANKGNN	GCTAGTATCAGAAA
M00128	V$GATA1_04	0.829963	9287	(-)	NNCWGATARNNNN	GCTAGTATCAGAA
M00075	V$GATA1_01	0.846496	9289	(-)	SNNGATNNNN	TAGTATCAGA
M00076	V$GATA2_01	0.872801	9289	(-)	NNNGATRNNN	TAGTATCAGA
M00075	V$GATA1_01	0.831688	9298	(-)	SNNGATNNNN	AAACATCATT
M00076	V$GATA2_01	0.822282	9298	(-)	NNNGATRNNN	AAACATCATT
M00075	V$GATA1_01	0.784304	9313	(-)	SNNGATNNNN	CCTCATCCAA
M00076	V$GATA2_01	0.803338	9313	(-)	NNNGATRNNN	CCTCATCCAA

M00077	V$GATA3_01	0.831192	9313	(-)	NNGATARNG	CCTCATCCA
M00075	V$GATA1_01	0.837117	9341	(-)	SNNGATNNNN	AAAAATCAGA
M00076	V$GATA2_01	0.826793	9341	(-)	NNNGATRNNN	AAAAATCAGA
M00126	V$GATA1_02	0.773125	9357	(+)	NNNNNGATANKGNN	ATGGTGCTAGTGGT
M00127	V$GATA1_03	0.813817	9357	(+)	RNSNNGATAANNGN	ATGGTGCTAGTGGT
M00075	V$GATA1_01	0.810464	9359	(+)	SNNGATNNNN	GGTGCTAGTG
M00127	V$GATA1_03	0.811367	9402	(+)	RNSNNGATAANNGN	GGCTTGATCACTGG
M00075	V$GATA1_01	0.795163	9404	(+)	SNNGATNNNN	CTTGATCACT
M00075	V$GATA1_01	0.830701	9421	(+)	SNNGATNNNN	GAAGATCCCC
M00075	V$GATA1_01	0.833662	9421	(-)	SNNGATNNNN	GAAGATCCCC
M00076	V$GATA2_01	0.870997	9421	(+)	NNNGATRNNN	GAAGATCCCC
M00076	V$GATA2_01	0.863329	9421	(-)	NNNGATRNNN	GAAGATCCCC
M00076	V$GATA2_01	0.820929	9438	(+)	NNNGATRNNN	GGGCATGGCA
M00076	V$GATA2_01	0.789806	9438	(-)	NNNGATRNNN	GGGCATGGCA
M00127	V$GATA1_03	0.792014	9498	(-)	RNSNNGATAANNGN	AGGGCTATATCCAT
M00203	V$GATA_C	0.876359	9499	(-)	NGATAAGNMNN	GGGCTATATCC
M00075	V$GATA1_01	0.80306	9511	(-)	SNNGATNNNN	TAGGATCACA
M00076	V$GATA2_01	0.818674	9511	(+)	NNNGATRNNN	TAGGATCACA
M00076	V$GATA2_01	0.8083	9511	(-)	NNNGATRNNN	TAGGATCACA
M00075	V$GATA1_01	0.805528	9563	(-)	SNNGATNNNN	GCAAATCTGT
M00076	V$GATA2_01	0.8083	9563	(-)	NNNGATRNNN	GCAAATCTGT
M00128	V$GATA1_04	0.848958	9591	(+)	NNCWGATARNNNNN	TTCTGATATAATG
M00075	V$GATA1_01	0.785785	9592	(+)	SNNGATNNNN	TCTGATATAA
M00076	V$GATA2_01	0.82138	9592	(+)	NNNGATRNNN	TCTGATATAA
M00076	V$GATA2_01	0.784844	9655	(-)	NNNGATRNNN	AGTCATGTCC
M00075	V$GATA1_01	0.809477	9699	(-)	SNNGATNNNN	GACCAACACC
M00076	V$GATA2_01	0.784844	9723	(+)	NNNGATRNNN	GCTCATGTCC
M00075	V$GATA1_01	0.846002	9737	(+)	SNNGATNNNN	AGTGATGCCA
M00076	V$GATA2_01	0.830853	9737	(+)	NNNGATRNNN	AGTGATGCCA
M00075	V$GATA1_01	0.790227	9742	(-)	SNNGATNNNN	TGCCATCCAA
M00076	V$GATA2_01	0.824538	9742	(-)	NNNGATRNNN	TGCCATCCAA
M00075	V$GATA1_01	0.829714	9755	(-)	SNNGATNNNN	TCTCATCCTC
M00076	V$GATA2_01	0.856563	9755	(-)	NNNGATRNNN	TCTCATCCTC

M00077	V$GATA3_01	0.820558	9755	(-)	NNGATARNG	TCTCATCCT
M00127	V$GATA1_03	0.827536	9768	(-)	RNSNNGATAANNGN	CCTCTTCTCCTCTC
M00127	V$GATA1_03	0.797158	9773	(-)	RNSNNGATAANNGN	TCTCCTCTCCTGCC
M00077	V$GATA3_01	0.873283	9787	(-)	NNGATARNG	TTCAATCTT
M00075	V$GATA1_01	0.772458	9796	(-)	SNNGATNNNN	TCCCAACATC
M00075	V$GATA1_01	0.932873	9799	(-)	SNNGATNNNN	CAACATCAGG
M00076	V$GATA2_01	0.88498	9799	(-)	NNNGATRNNN	CAACATCAGG
M00075	V$GATA1_01	0.834156	9819	(-)	SNNGATNNNN	ATGCATCAGT
M00076	V$GATA2_01	0.801534	9819	(-)	NNNGATRNNN	ATGCATCAGT
M00075	V$GATA1_01	0.87463	9832	(-)	SNNGATNNNN	TCAAATCAGG
M00076	V$GATA2_01	0.812359	9832	(-)	NNNGATRNNN	TCAAATCAGG
M00077	V$GATA3_01	0.824546	9832	(-)	NNGATARNG	TCAAATCAG
M00075	V$GATA1_01	0.828727	9892	(+)	SNNGATNNNN	ACTGATTTCT
M00076	V$GATA2_01	0.813261	9892	(+)	NNNGATRNNN	ACTGATTTCT
M00075	V$GATA1_01	0.78381	9904	(+)	SNNGATNNNN	TAGGATTGAC
M00076	V$GATA2_01	0.837167	9904	(+)	NNNGATRNNN	TAGGATTGAC
M00203	V$GATA_C	0.866729	9922	(-)	NGATAAGNMNN	TATTTATATCT
M00128	V$GATA1_04	0.893382	9923	(-)	NNCWGATARNNNN	ATTTATATCTGAC
M00076	V$GATA2_01	0.832657	9925	(-)	NNNGATRNNN	TTATATCTGA
M00077	V$GATA3_01	0.840939	9925	(-)	NNGATARNG	TTATATCTG
M00075	V$GATA1_01	0.878578	10006	(+)	SNNGATNNNN	AATGATTGGG
M00076	V$GATA2_01	0.842129	10006	(+)	NNNGATRNNN	AATGATTGGG
M00077	V$GATA3_01	0.925565	10007	(+)	NNGATARNG	ATGATTGGG
M00075	V$GATA1_01	0.777394	10053	(+)	SNNGATNNNN	TAAGATTATT
M00076	V$GATA2_01	0.815065	10053	(+)	NNNGATRNNN	TAAGATTATT
M00077	V$GATA3_01	0.832964	10054	(+)	NNGATARNG	AAGATTATT
M00127	V$GATA1_03	0.83072	10087	(+)	RNSNNGATAANNGN	AAAGGGATACCATG
M00075	V$GATA1_01	0.820829	10089	(+)	SNNGATNNNN	AGGGATACCA
M00076	V$GATA2_01	0.88498	10089	(+)	NNNGATRNNN	AGGGATACCA
M00126	V$GATA1_02	0.8075	10095	(+)	NNNNNGATANKGNN	ACCATGATAGATGG
M00127	V$GATA1_03	0.876041	10095	(+)	RNSNNGATAANNGN	ACCATGATAGATGG
M00128	V$GATA1_04	0.827512	10096	(+)	NNCWGATARNNNN	CCATGATAGATGG
M00075	V$GATA1_01	0.908193	10097	(+)	SNNGATNNNN	CATGATAGAT
M00076	V$GATA2_01	0.88858	10097	(+)	NNNGATRNNN	CATGATAGAT

		8				
M00077	V$GATA3_01	0.851573	10098	(+)	NNGATARNG	ATGATAGAT
M00075	V$GATA1_01	0.786278	10101	(+)	SNNGATNNNN	ATAGATGGTA
M00077	V$GATA3_01	0.848914	10102	(+)	NNGATARNG	TAGATGGTA
M00075	V$GATA1_01	0.820829	10106	(-)	SNNGATNNNN	TGGTATCCAC
M00076	V$GATA2_01	0.842129	10106	(-)	NNNGATRNNN	TGGTATCCAC
M00075	V$GATA1_01	0.798618	10181	(-)	SNNGATNNNN	CCCCAGCTGG
M00075	V$GATA1_01	0.775913	10267	(-)	SNNGATNNNN	CTACATCAAA
M00075	V$GATA1_01	0.853899	10272	(-)	SNNGATNNNN	TCAAATCATG
M00076	V$GATA2_01	0.80424	10272	(-)	NNNGATRNNN	TCAAATCATG
M00077	V$GATA3_01	0.830306	10272	(-)	NNGATARNG	TCAAATCAT
M00075	V$GATA1_01	0.771964	10278	(+)	SNNGATNNNN	CATGTTAGAG
M00075	V$GATA1_01	0.789733	10295	(+)	SNNGATNNNN	AATGATTCAT
M00127	V$GATA1_03	0.801813	10325	(-)	RNSNNGATAANNGN	GTATTTATTTTCCC
M00075	V$GATA1_01	0.839585	10337	(-)	SNNGATNNNN	CCAAATCCAG
M00076	V$GATA2_01	0.791159	10337	(-)	NNNGATRNNN	CCAAATCCAG
M00075	V$GATA1_01	0.886969	10355	(+)	SNNGATNNNN	GGTGATTCCA
M00076	V$GATA2_01	0.841678	10355	(+)	NNNGATRNNN	GGTGATTCCA
M00076	V$GATA2_01	0.819576	10366	(-)	NNNGATRNNN	ACCTATGCCT
M00127	V$GATA1_03	0.788339	10386	(+)	RNSNNGATAANNGN	GCACAGATCATTGC
M00075	V$GATA1_01	0.792695	10388	(-)	SNNGATNNNN	ACAGATCATT
M00076	V$GATA2_01	0.790708	10388	(+)	NNNGATRNNN	ACAGATCATT
M00076	V$GATA2_01	0.792512	10388	(-)	NNNGATRNNN	ACAGATCATT
M00075	V$GATA1_01	0.919052	10442	(-)	SNNGATNNNN	AGGAATCAGG
M00076	V$GATA2_01	0.866035	10442	(-)	NNNGATRNNN	AGGAATCAGG
M00075	V$GATA1_01	0.813425	10449	(-)	SNNGATNNNN	AGGGATCCCA
M00076	V$GATA2_01	0.820478	10449	(+)	NNNGATRNNN	AGGGATCCCA
M00076	V$GATA2_01	0.882724	10449	(-)	NNNGATRNNN	AGGGATCCCA
M00075	V$GATA1_01	0.844028	10506	(+)	SNNGATNNNN	GTTGATTGAT
M00076	V$GATA2_01	0.78304	10510	(-)	NNNGATRNNN	ATTGATCTCA
M00203	V$GATA_C	0.894066	10542	(-)	NGATAAGNMNN	TATAGTTATCT
M00126	V$GATA1_02	0.798125	10543	(-)	NNNNNGATANKGNN	ATAGTTATCTTCAC
M00127	V$GATA1_03	0.920872	10543	(-)	RNSNNGATAANNGN	ATAGTTATCTTCAC

M00128	V$GATA1_04	0.900429	10543	(-)	NNCWGATARNNNN	ATAGTTATCTTCA
M00075	V$GATA1_01	0.857354	10545	(-)	SNNGATNNNN	AGTTATCTTC
M00076	V$GATA2_01	0.912945	10545	(-)	NNNGATRNNN	AGTTATCTTC
M00077	V$GATA3_01	0.850687	10545	(-)	NNGATARNG	AGTTATCTT
M00075	V$GATA1_01	0.815893	10645	(-)	SNNGATNNNN	AAAAATCTCT
M00076	V$GATA2_01	0.805593	10645	(-)	NNNGATRNNN	AAAAATCTCT
M00127	V$GATA1_03	0.794708	10659	(-)	RNSNNGATAANNGN	CCCCTAATCCCTAG
M00075	V$GATA1_01	0.816881	10661	(-)	SNNGATNNNN	CCTAATCCCT
M00076	V$GATA2_01	0.824538	10661	(-)	NNNGATRNNN	CCTAATCCCT
M00077	V$GATA3_01	0.892778	10661	(-)	NNGATARNG	CCTAATCCC
M00127	V$GATA1_03	0.810142	10684	(-)	RNSNNGATAANNGN	TTTGCTATGTTCAT
M00076	V$GATA2_01	0.826793	10686	(-)	NNNGATRNNN	TGCTATGTTC
M00077	V$GATA3_01	0.838724	10728	(-)	NNGATARNG	TTTAATCAC
M00126	V$GATA1_02	0.876563	10733	(+)	NNNNNGATANKGNN	TCACAGATAAGCTG
M00127	V$GATA1_03	0.796178	10733	(+)	RNSNNGATAANNGN	TCACAGATAAGCTG
M00128	V$GATA1_04	0.850184	10734	(+)	NNCWGATARNNNN	CACAGATAAGCTG
M00075	V$GATA1_01	0.810958	10735	(+)	SNNGATNNNN	ACAGATAAGC
M00076	V$GATA2_01	0.857916	10735	(+)	NNNGATRNNN	ACAGATAAGC
M00077	V$GATA3_01	0.881701	10736	(+)	NNGATARNG	CAGATAAGC
M00203	V$GATA_C	0.873253	10737	(+)	NGATAAGNMNN	AGATAAGCTGT
M00075	V$GATA1_01	0.881046	10745	(+)	SNNGATNNNN	TGTGATGTGT
M00076	V$GATA2_01	0.869193	10745	(+)	NNNGATRNNN	TGTGATGTGT
M00203	V$GATA_C	0.907425	10767	(-)	NGATAAGNMNN	CATTTTTATCT
M00126	V$GATA1_02	0.839062	10768	(-)	NNNNNGATANKGNN	ATTTTTATCTTGAA
M00127	V$GATA1_03	0.798628	10768	(-)	RNSNNGATAANNGN	ATTTTTATCTTGAA
M00128	V$GATA1_04	0.912377	10768	(-)	NNCWGATARNNNN	ATTTTTATCTTGA
M00075	V$GATA1_01	0.786772	10770	(-)	SNNGATNNNN	TTTTATCTTG
M00076	V$GATA2_01	0.818674	10770	(-)	NNNGATRNNN	TTTTATCTTG
M00077	V$GATA3_01	0.8786	10770	(-)	NNGATARNG	TTTTATCTT
M00128	V$GATA1_04	0.819853	10856	(+)	NNCWGATARNNNN	GTGAGATATATTT
M00075	V$GATA1_01	0.80849	10857	(+)	SNNGATNNNN	TGAGATATAT
M00076	V$GATA2_01	0.862427	10857	(+)	NNNGATRNNN	TGAGATATAT
M00077	V$GATA3_01	0.832078	10858	(+)	NNGATARNG	GAGATATAT

M00075	V$GATA1_01	0.816881	10898	(+)	SNNGATNNNN	TAAGATGTGC
M00076	V$GATA2_01	0.868742	10898	(+)	NNNGATRNNN	TAAGATGTGC
M00126	V$GATA1_02	0.835625	10926	(-)	NNNNNGATANKGNN	TAAACTATCTGCTG
M00127	V$GATA1_03	0.83317	10926	(-)	RNSNNGATAANNGN	TAAACTATCTGCTG
M00128	V$GATA1_04	0.85815	10926	(-)	NNCWGATARNNNN	TAAACTATCTGCT
M00075	V$GATA1_01	0.943238	10928	(-)	SNNGATNNNN	AACTATCTGC
M00076	V$GATA2_01	0.953541	10928	(-)	NNNGATRNNN	AACTATCTGC
M00077	V$GATA3_01	0.889233	10928	(-)	NNGATARNG	AACTATCTG
M00126	V$GATA1_02	0.840313	10934	(+)	NNNNNGATANKGNN	CTGCTGATAGTATG
M00127	V$GATA1_03	0.865017	10934	(+)	RNSNNGATAANNGN	CTGCTGATAGTATG
M00128	V$GATA1_04	0.859375	10935	(+)	NNCWGATARNNNN	TGCTGATAGTATG
M00075	V$GATA1_01	0.92843	10936	(+)	SNNGATNNNN	GCTGATAGTA
M00076	V$GATA2_01	0.912494	10936	(+)	NNNGATRNNN	GCTGATAGTA
M00077	V$GATA3_01	0.897652	10937	(+)	NNGATARNG	CTGATAGTA
M00126	V$GATA1_02	0.806562	10943	(+)	NNNNNGATANKGNN	GTATGGATAGATCA
M00127	V$GATA1_03	0.781725	10943	(+)	RNSNNGATAANNGN	GTATGGATAGATCA
M00075	V$GATA1_01	0.777887	10945	(+)	SNNGATNNNN	ATGGATAGAT
M00076	V$GATA2_01	0.791159	10945	(+)	NNNGATRNNN	ATGGATAGAT
M00203	V$GATA_C	0.891892	10947	(+)	NGATAAGNMNN	GGATAGATCAA
M00126	V$GATA1_02	0.859375	10958	(-)	NNNNNGATANKGNN	AAAAGTATCTATTA
M00075	V$GATA1_01	0.79615	10960	(-)	SNNGATNNNN	AAGTATCTAT
M00076	V$GATA2_01	0.79161	10960	(-)	NNNGATRNNN	AAGTATCTAT
M00075	V$GATA1_01	0.874136	10973	(-)	SNNGATNNNN	CACAATCCAG
M00076	V$GATA2_01	0.82138	10973	(-)	NNNGATRNNN	CACAATCCAG
M00077	V$GATA3_01	0.858662	10973	(-)	NNGATARNG	CACAATCCA
M00075	V$GATA1_01	0.781343	10993	(-)	SNNGATNNNN	TAGCATCAAT
M00075	V$GATA1_01	0.790227	10997	(-)	SNNGATNNNN	ATCAATCCTT
M00076	V$GATA2_01	0.8083	10997	(-)	NNNGATRNNN	ATCAATCCTT
M00075	V$GATA1_01	0.846496	11009	(-)	SNNGATNNNN	GAGTATCATA
M00076	V$GATA2_01	0.890843	11009	(-)	NNNGATRNNN	GAGTATCATA
M00076	V$GATA2_01	0.811908	11012	(+)	NNNGATRNNN	TATCATAGTT
M00075	V$GATA1_01	0.835143	11069	(+)	SNNGATNNNN	CTTGATTAGT
M00203	V$GATA_C	0.83908	11090	(+)	NGATAAGNMNN	GGAGAAGGACT
M00203	V$GATA_C	0.908667	11095	(-)	NGATAAGNMNN	AGGACTTGTCT

M00203	V$GATA_C	0.862069	11257	(-)	NGATAAGNMNN	TAGATATATCA
M00126	V$GATA1_02	0.7825	11258	(-)	NNNNNGATANKGNN	AGATATATCAGTAC
M00128	V$GATA1_04	0.860907	11258	(-)	NNCWGATARNNNN	AGATATATCAGTA
M00203	V$GATA_C	0.844362	11258	(+)	NGATAAGNMNN	AGATATATCAG
M00075	V$GATA1_01	0.812932	11260	(-)	SNNGATNNNN	ATATATCAGT
M00076	V$GATA2_01	0.818674	11260	(-)	NNNGATRNNN	ATATATCAGT
M00075	V$GATA1_01	0.799112	11272	(-)	SNNGATNNNN	CTCAATCAAA
M00077	V$GATA3_01	0.860434	11272	(-)	NNGATARNG	CTCAATCAA
M00203	V$GATA_C	0.858962	11278	(-)	NGATAAGNMNN	CAAATTTATCA
M00126	V$GATA1_02	0.840313	11279	(-)	NNNNNGATANKGNN	AAATTTATCATTAC
M00127	V$GATA1_03	0.821411	11279	(-)	RNSNNGATAANNGN	AAATTTATCATTAC
M00128	V$GATA1_04	0.85815	11279	(-)	NNCWGATARNNNN	AAATTTATCATTA
M00075	V$GATA1_01	0.773939	11281	(-)	SNNGATNNNN	ATTTATCATT
M00076	V$GATA2_01	0.79567	11281	(-)	NNNGATRNNN	ATTTATCATT
M00077	V$GATA3_01	0.823217	11281	(-)	NNGATARNG	ATTTATCAT
M00203	V$GATA_C	0.832246	11288	(-)	NGATAAGNMNN	ATTACTAATCT
M00077	V$GATA3_01	0.82942	11291	(-)	NNGATARNG	ACTAATCTA
M00126	V$GATA1_02	0.819063	11334	(+)	NNNNNGATANKGNN	GTTATGATATGATT
M00128	V$GATA1_04	0.843137	11335	(+)	NNCWGATARNNNN	TTATGATATGATT
M00075	V$GATA1_01	0.816881	11336	(+)	SNNGATNNNN	TATGATATGA
M00076	V$GATA2_01	0.85972	11336	(+)	NNNGATRNNN	TATGATATGA
M00077	V$GATA3_01	0.863979	11337	(+)	NNGATARNG	ATGATATGA
M00203	V$GATA_C	0.843119	11338	(+)	NGATAAGNMNN	TGATATGATTA
M00128	V$GATA1_04	0.817402	11340	(+)	NNCWGATARNNNN	ATATGATTAGGCC
M00075	V$GATA1_01	0.821816	11341	(+)	SNNGATNNNN	TATGATTAGG
M00076	V$GATA2_01	0.823184	11341	(+)	NNNGATRNNN	TATGATTAGG
M00077	V$GATA3_01	0.897209	11342	(+)	NNGATARNG	ATGATTAGG
M00076	V$GATA2_01	0.794317	11351	(-)	NNNGATRNNN	CCGTAACCTC
M00076	V$GATA2_01	0.847542	11387	(+)	NNNGATRNNN	GCTCATAGTG
M00076	V$GATA2_01	0.804691	11419	(+)	NNNGATRNNN	GCTCATAGTA
M00075	V$GATA1_01	0.807009	11447	(+)	SNNGATNNNN	ATAGATGGTC
M00076	V$GATA2_01	0.784844	11447	(+)	NNNGATRNNN	ATAGATGGTC
M00076	V$GATA2_01	0.793866	11466	(+)	NNNGATRNNN	TAGCATAGAC

M00127	V$GATA1_03	0.811367	11505	(-)	RNSNNGATAANNGN	TCTGTTATTCACAC
M00126	V$GATA1_02	0.789062	11536	(+)	NNNNNGATANKGNN	ACTAAGATAAAGAG
M00127	V$GATA1_03	0.815287	11536	(+)	RNSNNGATAANNGN	ACTAAGATAAAGAG
M00128	V$GATA1_04	0.876225	11537	(+)	NNCWGATARNNNNN	CTAAGATAAAGAG
M00075	V$GATA1_01	0.772952	11538	(+)	SNNGATNNNN	TAAGATAAAG
M00076	V$GATA2_01	0.852503	11538	(+)	NNNGATRNNN	TAAGATAAAG
M00077	V$GATA3_01	0.913602	11539	(+)	NNGATARNG	AAGATAAAG
M00203	V$GATA_C	0.89593	11540	(+)	NGATAAGNMNN	AGATAAAGAGA
M00075	V$GATA1_01	0.788253	11546	(+)	SNNGATNNNN	AGAGATCTCT
M00075	V$GATA1_01	0.788253	11546	(-)	SNNGATNNNN	AGAGATCTCT
M00076	V$GATA2_01	0.824538	11546	(+)	NNNGATRNNN	AGAGATCTCT
M00076	V$GATA2_01	0.824538	11546	(-)	NNNGATRNNN	AGAGATCTCT
M00128	V$GATA1_04	0.826287	11578	(+)	NNCWGATARNNNNN	ACCATATAAGACT
M00075	V$GATA1_01	0.792201	11596	(-)	SNNGATNNNN	CTAAATCTAG
M00077	V$GATA3_01	0.82366	11596	(-)	NNGATARNG	CTAAATCTA
M00075	V$GATA1_01	0.804541	11605	(-)	SNNGATNNNN	GAATATCTTA
M00076	V$GATA2_01	0.890843	11605	(-)	NNNGATRNNN	GAATATCTTA
M00077	V$GATA3_01	0.834736	11605	(-)	NNGATARNG	GAATATCTT
M00126	V$GATA1_02	0.863438	11710	(+)	NNNNNGATANKGNN	TATTTGATAATGTT
M00128	V$GATA1_04	0.84038	11711	(+)	NNCWGATARNNNNN	ATTTGATAATGTT
M00075	V$GATA1_01	0.797137	11712	(+)	SNNGATNNNN	TTTGATAATG
M00076	V$GATA2_01	0.799729	11712	(+)	NNNGATRNNN	TTTGATAATG
M00077	V$GATA3_01	0.885689	11713	(+)	NNGATARNG	TTGATAATG
M00203	V$GATA_C	0.855235	11714	(+)	NGATAAGNMNN	TGATAATGTTT
M00126	V$GATA1_02	0.847187	11738	(+)	NNNNNGATANKGNN	TAAAAGATAAAATG
M00128	V$GATA1_04	0.888174	11739	(+)	NNCWGATARNNNNN	AAAAGATAAAATG
M00077	V$GATA3_01	0.8786	11741	(+)	NNGATARNG	AAGATAAAA
M00203	V$GATA_C	0.856167	11742	(+)	NGATAAGNMNN	AGATAAAATGT
M00075	V$GATA1_01	0.778875	11758	(+)	SNNGATNNNN	AGTGATTAAT
M00075	V$GATA1_01	0.793189	11804	(+)	SNNGATNNNN	GTGGATTATT
M00127	V$GATA1_03	0.801813	11805	(-)	RNSNNGATAANNGN	TGGATTATTTTCCC
M00126	V$GATA1_02	0.790625	11817	(+)	NNNNNGATANKGNN	CCCCAGATATGAAA
M00128	V$GATA1_04	0.830882	11818	(+)	NNCWGATARNNNNN	CCCAGATATGAAA

M00075	V$GATA1_01	0.877591	11819	(+)	SNNGATNNNN	CCAGATATGA
M00076	V$GATA2_01	0.888137	11819	(+)	NNNGATRNNN	CCAGATATGA
M00077	V$GATA3_01	0.881701	11820	(+)	NNGATARNG	CAGATATGA
M00203	V$GATA_C	0.89096	11821	(+)	NGATAAGNMNN	AGATATGAAAC
M00075	V$GATA1_01	0.772458	11834	(+)	SNNGATNNNN	ATAGATTCTT
M00203	V$GATA_C	0.940665	11838	(-)	NGATAAGNMNN	ATTCTTTATCC
M00126	V$GATA1_02	0.78375	11839	(-)	NNNNNGATANKGNN	TTCTTTATCCTTCC
M00127	V$GATA1_03	0.865507	11839	(-)	RNSNNGATAANNGN	TTCTTTATCCTTCC
M00128	V$GATA1_04	0.841605	11839	(-)	NNCWGATARNNNN	TTCTTTATCCTTC
M00076	V$GATA2_01	0.834461	11841	(-)	NNNGATRNNN	CTTTATCCTT
M00077	V$GATA3_01	0.868409	11841	(-)	NNGATARNG	CTTTATCCT
M00127	V$GATA1_03	0.824351	11953	(-)	RNSNNGATAANNGN	ATACTAATCTTCAT
M00075	V$GATA1_01	0.846002	11955	(-)	SNNGATNNNN	ACTAATCTTC
M00076	V$GATA2_01	0.835814	11955	(-)	NNNGATRNNN	ACTAATCTTC
M00077	V$GATA3_01	0.853788	11955	(-)	NNGATARNG	ACTAATCTT
M00077	V$GATA3_01	0.820558	11966	(+)	NNGATARNG	TAGATGATA
M00128	V$GATA1_04	0.822304	11967	(+)	NNCWGATARNNNN	AGATGATATATTC
M00075	V$GATA1_01	0.856861	11968	(+)	SNNGATNNNN	GATGATATAT
M00076	V$GATA2_01	0.857465	11968	(+)	NNNGATRNNN	GATGATATAT
M00203	V$GATA_C	0.840944	11970	(+)	NGATAAGNMNN	TGATATATTCT
M00075	V$GATA1_01	0.785785	12003	(-)	SNNGATNNNN	TTACATCAGA
M00076	V$GATA2_01	0.792963	12003	(-)	NNNGATRNNN	TTACATCAGA
M00127	V$GATA1_03	0.801323	12009	(+)	RNSNNGATAANNGN	CAGAAGATATCTAG
M00128	V$GATA1_04	0.86152	12010	(+)	NNCWGATARNNNN	AGAAGATATCTAG
M00075	V$GATA1_01	0.875617	12011	(+)	SNNGATNNNN	GAAGATATCT
M00076	V$GATA2_01	0.92783	12011	(+)	NNNGATRNNN	GAAGATATCT
M00075	V$GATA1_01	0.844028	12013	(-)	SNNGATNNNN	AGATATCTAG
M00076	V$GATA2_01	0.840776	12013	(-)	NNNGATRNNN	AGATATCTAG
M00127	V$GATA1_03	0.782705	12017	(+)	RNSNNGATAANNGN	ATCTAGGTAACCTA
M00076	V$GATA2_01	0.801083	12031	(+)	NNNGATRNNN	GAACATATTT
M00126	V$GATA1_02	0.855625	12119	(+)	NNNNNGATANKGNN	ATAATGATAATATG
M00127	V$GATA1_03	0.888535	12119	(+)	RNSNNGATAANNGN	ATAATGATAATATG
M00128	V$GATA1_04	0.845282	12120	(+)	NNCWGATARNNNN	TAATGATAATATG

M00075	V$GATA1_01	0.777887	12121	(+)	SNNGATNNNN	AATGATAATA
M00076	V$GATA2_01	0.806044	12121	(+)	NNNGATRNNN	AATGATAATA
M00077	V$GATA3_01	0.875055	12122	(+)	NNGATARNG	ATGATAATA
M00075	V$GATA1_01	0.772458	12191	(-)	SNNGATNNNN	TCCTCTCAGG
M00127	V$GATA1_03	0.791524	12237	(-)	RNSNNGATAANNGN	GTAGTAATCAATCT
M00075	V$GATA1_01	0.823791	12243	(-)	SNNGATNNNN	ATCAATCTGA
M00076	V$GATA2_01	0.834461	12243	(-)	NNNGATRNNN	ATCAATCTGA
M00077	V$GATA3_01	0.835623	12243	(-)	NNGATARNG	ATCAATCTG
M00128	V$GATA1_04	0.810662	12247	(+)	NNCWGATARNNNN	ATCTGAGAAAGGA
M00075	V$GATA1_01	0.784798	12306	(-)	SNNGATNNNN	CAGTCTCACC
M00075	V$GATA1_01	0.784798	12319	(-)	SNNGATNNNN	CAGCAGCACC
M00076	V$GATA2_01	0.7871	12366	(-)	NNNGATRNNN	GGTTATGTTA
M00076	V$GATA2_01	0.7871	12384	(-)	NNNGATRNNN	GGTTATGTTA
M00128	V$GATA1_04	0.8125	12397	(-)	NNCWGATARNNNN	TCTCTTTTCAGGT
M00203	V$GATA_C	0.875427	12418	(-)	NGATAAGNMNN	ATTTTATATCT
M00126	V$GATA1_02	0.781563	12419	(-)	NNNNNGATANKGNN	TTTTATATCTTTAT
M00128	V$GATA1_04	0.842525	12419	(-)	NNCWGATARNNNN	TTTTATATCTTTA
M00076	V$GATA2_01	0.792061	12421	(-)	NNNGATRNNN	TTATATCTTT
M00077	V$GATA3_01	0.846699	12421	(-)	NNGATARNG	TTATATCTT
M00127	V$GATA1_03	0.801813	12425	(-)	RNSNNGATAANNGN	ATCTTTATGTCCAT
M00075	V$GATA1_01	0.853899	12436	(+)	SNNGATNNNN	CATGATTTGA
M00076	V$GATA2_01	0.80424	12436	(+)	NNNGATRNNN	CATGATTTGA
M00077	V$GATA3_01	0.830306	12437	(+)	NNGATARNG	ATGATTTGA
M00075	V$GATA1_01	0.801579	12441	(+)	SNNGATNNNN	TTTGATTCTT
M00127	V$GATA1_03	0.791524	12483	(-)	RNSNNGATAANNGN	ATAATTATGCTCTT
M00076	V$GATA2_01	0.800631	12485	(-)	NNNGATRNNN	AATTATGCTC
M00075	V$GATA1_01	0.841066	12512	(+)	SNNGATNNNN	GGAGATTTAT
M00076	V$GATA2_01	0.812359	12512	(+)	NNNGATRNNN	GGAGATTTAT
M00203	V$GATA_C	0.835353	12545	(-)	NGATAAGNMNN	AGAAACTATCT
M00126	V$GATA1_02	0.844375	12546	(-)	NNNNNGATANKGNN	GAAACTATCTGGAG
M00127	V$GATA1_03	0.848849	12546	(-)	RNSNNGATAANNGN	GAAACTATCTGGAG
M00128	V$GATA1_04	0.857843	12546	(-)	NNCWGATARNNNN	GAAACTATCTGGA
M00075	V$GATA1_01	0.94768	12548	(-)	SNNGATNNNN	AACTATCTGG

M00076	V$GATA2_01	0.948128	12548	(-)	NNNGATRNNN	AACTATCTGG
M00077	V$GATA3_01	0.889233	12548	(-)	NNGATARNG	AACTATCTG
M00126	V$GATA1_02	0.861563	12556	(+)	NNNNNGATANKGNN	GGAGAGATAGTAAA
M00127	V$GATA1_03	0.876776	12556	(+)	RNSNNGATAANNGN	GGAGAGATAGTAAA
M00128	V$GATA1_04	0.856618	12557	(+)	NNCWGATARNNNN	GAGAGATAGTAAA
M00075	V$GATA1_01	0.850938	12558	(+)	SNNGATNNNN	AGAGATAGTA
M00076	V$GATA2_01	0.876861	12558	(+)	NNNGATRNNN	AGAGATAGTA
M00077	V$GATA3_01	0.944174	12559	(+)	NNGATARNG	GAGATAGTA
M00203	V$GATA_C	0.858652	12560	(+)	NGATAAGNMNN	AGATAGTAAAT
M00127	V$GATA1_03	0.784664	12588	(-)	RNSNNGATAANNGN	TTTCTGATCTGTTT
M00128	V$GATA1_04	0.816176	12588	(-)	NNCWGATARNNNN	TTTCTGATCTGTT
M00075	V$GATA1_01	0.821816	12590	(+)	SNNGATNNNN	TCTGATCTGT
M00076	V$GATA2_01	0.833108	12590	(+)	NNNGATRNNN	TCTGATCTGT
M00127	V$GATA1_03	0.78197	12604	(-)	RNSNNGATAANNGN	CCTTCCATCTGTAT
M00075	V$GATA1_01	0.803554	12606	(-)	SNNGATNNNN	TTCCATCTGT
M00076	V$GATA2_01	0.8083	12606	(-)	NNNGATRNNN	TTCCATCTGT
M00077	V$GATA3_01	0.847585	12606	(-)	NNGATARNG	TTCCATCTG
M00203	V$GATA_C	0.955887	12620	(-)	NGATAAGNMNN	TGTTTTTATCT
M00126	V$GATA1_02	0.820625	12621	(-)	NNNNNGATANKGNN	GTTTTTATCTATAT
M00127	V$GATA1_03	0.882166	12621	(-)	RNSNNGATAANNGN	GTTTTTATCTATAT
M00128	V$GATA1_04	0.875613	12621	(-)	NNCWGATARNNNN	GTTTTTATCTATA
M00077	V$GATA3_01	0.854231	12623	(-)	NNGATARNG	TTTTATCTA
M00127	V$GATA1_03	0.814307	12627	(+)	RNSNNGATAANNGN	ATCTATATAATAGA
M00075	V$GATA1_01	0.849951	12642	(+)	SNNGATNNNN	ACTGATTCTT
M00076	V$GATA2_01	0.805593	12642	(+)	NNNGATRNNN	ACTGATTCTT
M00075	V$GATA1_01	0.801579	12695	(+)	SNNGATNNNN	TCTGATTTCA
M00076	V$GATA2_01	0.815968	12695	(+)	NNNGATRNNN	TCTGATTTCA
M00127	V$GATA1_03	0.83072	12710	(+)	RNSNNGATAANNGN	AAGGTGACAACCAG
M00126	V$GATA1_02	0.856563	12718	(+)	NNNNNGATANKGNN	AACCAGATACTGTG
M00127	V$GATA1_03	0.849829	12718	(+)	RNSNNGATAANNGN	AACCAGATACTGTG
M00128	V$GATA1_04	0.815564	12719	(+)	NNCWGATARNNNN	ACCAGATACTGTG
M00075	V$GATA1_01	0.933366	12720	(+)	SNNGATNNNN	CCAGATACTG
M00076	V$GATA2_01	0.935949	12720	(+)	NNNGATRNNN	CCAGATACTG

M00077	V$GATA3_01	0.876828	12721	(+)	NNGATARNG	CAGATACTG
M00076	V$GATA2_01	0.809202	12732	(-)	NNNGATRNNN	AAATATGTGC
M00075	V$GATA1_01	0.858342	12754	(+)	SNNGATNNNN	TCTGATTCCT
M00076	V$GATA2_01	0.857014	12754	(+)	NNNGATRNNN	TCTGATTCCT
M00203	V$GATA_C	0.906803	12757	(-)	NGATAAGNMNN	GATTCCTATCA
M00126	V$GATA1_02	0.836875	12758	(-)	NNNNNGATANKGNN	ATTCCTATCAAAAG
M00128	V$GATA1_04	0.884498	12758	(-)	NNCWGATARNNNN	ATTCCTATCAAAA
M00075	V$GATA1_01	0.827739	12760	(-)	SNNGATNNNN	TCCTATCAAA
M00076	V$GATA2_01	0.814614	12760	(-)	NNNGATRNNN	TCCTATCAAA
M00077	V$GATA3_01	0.899867	12760	(-)	NNGATARNG	TCCTATCAA
M00076	V$GATA2_01	0.864682	12770	(-)	NNNGATRNNN	AGCTATGCCA
M00075	V$GATA1_01	0.80849	12782	(-)	SNNGATNNNN	TTCAATCACT
M00077	V$GATA3_01	0.86708	12782	(-)	NNGATARNG	TTCAATCAC
M00203	V$GATA_C	0.859584	12849	(-)	NGATAAGNMNN	AAATATTATCT
M00126	V$GATA1_02	0.87125	12850	(-)	NNNNNGATANKGNN	AATATTATCTAGAT
M00127	V$GATA1_03	0.882166	12850	(-)	RNSNNGATAANNGN	AATATTATCTAGAT
M00128	V$GATA1_04	0.815257	12850	(-)	NNCWGATARNNNN	AATATTATCTAGA
M00075	V$GATA1_01	0.790227	12852	(-)	SNNGATNNNN	TATTATCTAG
M00077	V$GATA3_01	0.874169	12852	(-)	NNGATARNG	TATTATCTA
M00126	V$GATA1_02	0.901875	12856	(+)	NNNNNGATANKGNN	ATCTAGATAATGCA
M00127	V$GATA1_03	0.882166	12856	(+)	RNSNNGATAANNGN	ATCTAGATAATGCA
M00128	V$GATA1_04	0.849571	12857	(+)	NNCWGATARNNNN	TCTAGATAATGCA
M00075	V$GATA1_01	0.837117	12858	(+)	SNNGATNNNN	CTAGATAATG
M00076	V$GATA2_01	0.820027	12858	(+)	NNNGATRNNN	CTAGATAATG
M00077	V$GATA3_01	0.909171	12859	(+)	NNGATARNG	TAGATAATG
M00203	V$GATA_C	0.907114	12860	(+)	NGATAAGNMNN	AGATAATGCAA
M00075	V$GATA1_01	0.849457	12896	(+)	SNNGATNNNN	CCAGATCTCT
M00075	V$GATA1_01	0.811945	12896	(-)	SNNGATNNNN	CCAGATCTCT
M00076	V$GATA2_01	0.866035	12896	(+)	NNNGATRNNN	CCAGATCTCT
M00076	V$GATA2_01	0.824989	12896	(-)	NNNGATRNNN	CCAGATCTCT
M00077	V$GATA3_01	0.824103	12896	(-)	NNGATARNG	CCAGATCTC
M00075	V$GATA1_01	0.771964	12902	(-)	SNNGATNNNN	CTCTAACATG
M00075	V$GATA1_01	0.873643	12965	(-)	SNNGATNNNN	AAAGATCACC

M00076	V$GATA2_01	0.797925	12965	(+)	NNNGATRNNN	AAAGATCACC
M00076	V$GATA2_01	0.841227	12965	(-)	NNNGATRNNN	AAAGATCACC
M00075	V$GATA1_01	0.778381	12992	(-)	SNNGATNNNN	CAGAAACAGC
M00076	V$GATA2_01	0.814614	13000	(-)	NNNGATRNNN	GCCTATGATA
M00126	V$GATA1_02	0.794375	13001	(+)	NNNNNGATANKGNN	CCTATGATAGCAGT
M00127	V$GATA1_03	0.785154	13001	(+)	RNSNNGATAANNGN	CCTATGATAGCAGT
M00128	V$GATA1_04	0.828125	13002	(+)	NNCWGATARNNNN	CTATGATAGCAGT
M00075	V$GATA1_01	0.851431	13003	(+)	SNNGATNNNN	TATGATAGCA
M00076	V$GATA2_01	0.908886	13003	(+)	NNNGATRNNN	TATGATAGCA
M00077	V$GATA3_01	0.887461	13004	(+)	NNGATARNG	ATGATAGCA
M00075	V$GATA1_01	0.864758	13013	(-)	SNNGATNNNN	GTGAATCAGG
M00076	V$GATA2_01	0.803338	13013	(-)	NNNGATRNNN	GTGAATCAGG
M00128	V$GATA1_04	0.836397	13017	(+)	NNCWGATARNNNN	ATCAGGTAAGTGT
M00126	V$GATA1_02	0.850938	13061	(-)	NNNNNGATANKGNN	CAAACTATCTATTG
M00127	V$GATA1_03	0.800343	13061	(-)	RNSNNGATAANNGN	CAAACTATCTATTG
M00075	V$GATA1_01	0.821816	13063	(-)	SNNGATNNNN	AACTATCTAT
M00076	V$GATA2_01	0.81687	13063	(-)	NNNGATRNNN	AACTATCTAT
M00077	V$GATA3_01	0.870625	13063	(-)	NNGATARNG	AACTATCTA
M00075	V$GATA1_01	0.846496	13107	(-)	SNNGATNNNN	TTACATCAGG
M00076	V$GATA2_01	0.801985	13107	(-)	NNNGATRNNN	TTACATCAGG
M00076	V$GATA2_01	0.786649	13180	(+)	NNNGATRNNN	CAACATTGCT
M00075	V$GATA1_01	0.809477	13193	(-)	SNNGATNNNN	AAAGATCAGA
M00076	V$GATA2_01	0.826793	13193	(-)	NNNGATRNNN	AAAGATCAGA
M00075	V$GATA1_01	0.867226	13239	(-)	SNNGATNNNN	GGCCATCCCT
M00076	V$GATA2_01	0.907984	13239	(-)	NNNGATRNNN	GGCCATCCCT
M00127	V$GATA1_03	0.792014	13251	(+)	RNSNNGATAANNGN	ATGAAGAGAGCTTC
M00203	V$GATA_C	0.881951	13300	(-)	NGATAAGNMNN	TATTCATATCA
M00126	V$GATA1_02	0.794063	13301	(-)	NNNNNGATANKGNN	ATTCATATCATTCA
M00128	V$GATA1_04	0.871936	13301	(-)	NNCWGATARNNNN	ATTCATATCATTC
M00075	V$GATA1_01	0.80849	13303	(-)	SNNGATNNNN	TCATATCATT
M00076	V$GATA2_01	0.827244	13303	(-)	NNNGATRNNN	TCATATCATT
M00077	V$GATA3_01	0.863979	13303	(-)	NNGATARNG	TCATATCAT
M00076	V$GATA2_01	0.79161	13374	(-)	NNNGATRNNN	TCTAATCTTA

M00077	V$GATA3_01	0.885689	13374	(-)	NNGATARNG	TCTAATCTT
M00127	V$GATA1_03	0.782705	13377	(-)	RNSNNGATAANNGN	AATCTTATGACCTT
M00203	V$GATA_C	0.840323	13401	(-)	NGATAAGNMNN	TGTTCTTCTCT
M00203	V$GATA_C	0.83877	13474	(-)	NGATAAGNMNN	CTCACATATCT
M00076	V$GATA2_01	0.813712	13475	(+)	NNNGATRNNN	TCACATATCT
M00126	V$GATA1_02	0.845938	13475	(-)	NNNNNGATANKGNN	TCACATATCTTGAG
M00127	V$GATA1_03	0.804998	13475	(-)	RNSNNGATAANNGN	TCACATATCTTGAG
M00128	V$GATA1_04	0.865196	13475	(-)	NNCWGATARNNNN	TCACATATCTTGA
M00075	V$GATA1_01	0.892399	13477	(-)	SNNGATNNNN	ACATATCTTG
M00076	V$GATA2_01	0.909788	13477	(-)	NNNGATRNNN	ACATATCTTG
M00077	V$GATA3_01	0.85556	13477	(-)	NNGATARNG	ACATATCTT
M00127	V$GATA1_03	0.834885	13521	(+)	RNSNNGATAANNGN	AAGGTGGTAACTGC
M00075	V$GATA1_01	0.842547	13558	(+)	SNNGATNNNN	ATTGATGGTT
M00076	V$GATA2_01	0.782589	13579	(-)	NNNGATRNNN	TAGTATGCTA
M00075	V$GATA1_01	0.798124	13635	(-)	SNNGATNNNN	ATGAATCTGA
M00076	V$GATA2_01	0.809202	13635	(-)	NNNGATRNNN	ATGAATCTGA
M00126	V$GATA1_02	0.8825	13638	(+)	NNNNNGATANKGNN	AATCTGATAAGGGA
M00127	V$GATA1_03	0.841499	13638	(+)	RNSNNGATAANNGN	AATCTGATAAGGGA
M00128	V$GATA1_04	0.97549	13639	(+)	NNCWGATARNNNN	ATCTGATAAGGGA
M00075	V$GATA1_01	0.866239	13640	(+)	SNNGATNNNN	TCTGATAAGG
M00076	V$GATA2_01	0.895805	13640	(+)	NNNGATRNNN	TCTGATAAGG
M00077	V$GATA3_01	0.925122	13641	(+)	NNGATARNG	CTGATAAGG
M00203	V$GATA_C	0.942839	13642	(+)	NGATAAGNMNN	TGATAAGGGAT
M00075	V$GATA1_01	0.802567	13657	(+)	SNNGATNNNN	CAAGATTCAA
M00076	V$GATA2_01	0.780334	13657	(+)	NNNGATRNNN	CAAGATTCAA
M00075	V$GATA1_01	0.777394	13675	(-)	SNNGATNNNN	AGTCATCAAT
M00075	V$GATA1_01	0.779368	13679	(-)	SNNGATNNNN	ATCAATCAAT
M00126	V$GATA1_02	0.818438	13689	(+)	NNNNNGATANKGNN	TTTATGATAATCAT
M00128	V$GATA1_04	0.878983	13690	(+)	NNCWGATARNNNN	TTATGATAATCAT
M00075	V$GATA1_01	0.807009	13691	(+)	SNNGATNNNN	TATGATAATC
M00076	V$GATA2_01	0.864682	13691	(+)	NNNGATRNNN	TATGATAATC
M00077	V$GATA3_01	0.843155	13692	(+)	NNGATARNG	ATGATAATC
M00127	V$GATA1_03	0.784174	13692	(-)	RNSNNGATAANNGN	ATGATAATCATTCC

M00203	V$GATA_C	0.877912	13693	(+)	NGATAAGNMNN	TGATAATCATT
M00075	V$GATA1_01	0.774926	13694	(-)	SNNGATNNNN	GATAATCATT
M00075	V$GATA1_01	0.783317	13711	(-)	SNNGATNNNN	GTGAATCATA
M00076	V$GATA2_01	0.786198	13711	(-)	NNNGATRNNN	GTGAATCATA
M00126	V$GATA1_02	0.770625	13775	(+)	NNNNNGATANKGNN	GAAAAGAAAAGACA
M00203	V$GATA_C	0.831314	13779	(+)	NGATAAGNMNN	AGAAAAGACAA
M00126	V$GATA1_02	0.869062	13817	(-)	NNNNNGATANKGNN	TACACTATCCTCAC
M00127	V$GATA1_03	0.865017	13817	(-)	RNSNNGATAANNGN	TACACTATCCTCAC
M00075	V$GATA1_01	0.929418	13819	(-)	SNNGATNNNN	CACTATCCTC
M00076	V$GATA2_01	0.972936	13819	(-)	NNNGATRNNN	CACTATCCTC
M00077	V$GATA3_01	0.916704	13819	(-)	NNGATARNG	CACTATCCT
M00127	V$GATA1_03	0.807692	13852	(+)	RNSNNGATAANNGN	AAGAGGGTAATCAT
M00127	V$GATA1_03	0.782705	13855	(-)	RNSNNGATAANNGN	AGGGTAATCATTCT
M00075	V$GATA1_01	0.774926	13857	(-)	SNNGATNNNN	GGTAATCATT
M00076	V$GATA2_01	0.786649	13857	(-)	NNNGATRNNN	GGTAATCATT
M00076	V$GATA2_01	0.79161	13866	(-)	NNNGATRNNN	TCTAATCTTA
M00077	V$GATA3_01	0.885689	13866	(-)	NNGATARNG	TCTAATCTT
M00127	V$GATA1_03	0.796178	13913	(-)	RNSNNGATAANNGN	GCATTTAACTGGGT
M00075	V$GATA1_01	0.791214	13927	(-)	SNNGATNNNN	TTGAATCACA
M00077	V$GATA3_01	0.840939	13949	(-)	NNGATARNG	TCCTCTCTC
M00127	V$GATA1_03	0.78246	13949	(-)	RNSNNGATAANNGN	TCCTCTCTCTCCTT
M00077	V$GATA3_01	0.835179	13951	(-)	NNGATARNG	CTCTCTCTC
M00075	V$GATA1_01	0.780355	14000	(+)	SNNGATNNNN	TTTGATTTTT
M00127	V$GATA1_03	0.811367	14017	(+)	RNSNNGATAANNGN	ATACTAATAAGAGA
M00075	V$GATA1_01	0.809477	14029	(-)	SNNGATNNNN	GAAAATCTCA
M00076	V$GATA2_01	0.834461	14029	(-)	NNNGATRNNN	GAAAATCTCA
M00075	V$GATA1_01	0.804541	14036	(+)	SNNGATNNNN	TCAGATATCA
M00076	V$GATA2_01	0.891746	14036	(+)	NNNGATRNNN	TCAGATATCA
M00128	V$GATA1_04	0.845588	14036	(-)	NNCWGATARNNNN	TCAGATATCATAT
M00077	V$GATA3_01	0.844927	14037	(+)	NNGATARNG	CAGATATCA
M00075	V$GATA1_01	0.840079	14038	(-)	SNNGATNNNN	AGATATCATA
M00076	V$GATA2_01	0.90212	14038	(-)	NNNGATRNNN	AGATATCATA
M00127	V$GATA1_03	0.800833	14048	(-)	RNSNNGATAANNGN	TTTATTATAATCGT

M00077	V$GATA3_01	0.835179	14053	(-)	NNGATARNG	TATAATCGT
M00126	V$GATA1_02	0.807813	14060	(+)	NNNNNGATANKGNN	GTTAGGATAGGCAA
M00127	V$GATA1_03	0.804018	14060	(+)	RNSNNGATAANNGN	GTTAGGATAGGCAA
M00128	V$GATA1_04	0.834559	14061	(+)	NNCWGATARNNNN	TTAGGATAGGCAA
M00075	V$GATA1_01	0.859329	14062	(+)	SNNGATNNNN	TAGGATAGGC
M00076	V$GATA2_01	0.948128	14062	(+)	NNNGATRNNN	TAGGATAGGC
M00077	V$GATA3_01	0.870625	14063	(+)	NNGATARNG	AGGATAGGC
M00203	V$GATA_C	0.897484	14064	(+)	NGATAAGNMNN	GGATAGGCAAC
M00075	V$GATA1_01	0.775913	14113	(-)	SNNGATNNNN	TTCCATCAAA
M00127	V$GATA1_03	0.785644	14128	(-)	RNSNNGATAANNGN	TATATTTTCCACCT
M00127	V$GATA1_03	0.789319	14172	(+)	RNSNNGATAANNGN	AAACAAATAAAAGG
M00128	V$GATA1_04	0.810968	14173	(+)	NNCWGATARNNNN	AACAAATAAAAGG
M00076	V$GATA2_01	0.798827	14184	(-)	NNNGATRNNN	GGCCATGATA
M00126	V$GATA1_02	0.817187	14185	(+)	NNNNNGATANKGNN	GCCATGATAGATTA
M00127	V$GATA1_03	0.797893	14185	(+)	RNSNNGATAANNGN	GCCATGATAGATTA
M00075	V$GATA1_01	0.908193	14187	(+)	SNNGATNNNN	CATGATAGAT
M00076	V$GATA2_01	0.888588	14187	(+)	NNNGATRNNN	CATGATAGAT
M00077	V$GATA3_01	0.851573	14188	(+)	NNGATARNG	ATGATAGAT
M00203	V$GATA_C	0.850885	14189	(+)	NGATAAGNMNN	TGATAGATTAA
M00128	V$GATA1_04	0.816176	14221	(+)	NNCWGATARNNNN	TTCAGAAAAGAAA
M00203	V$GATA_C	0.832246	14224	(+)	NGATAAGNMNN	AGAAAAGAAAT
M00126	V$GATA1_02	0.785313	14227	(+)	NNNNNGATANKGNN	AAAGAAATAATGGT
M00127	V$GATA1_03	0.798138	14227	(+)	RNSNNGATAANNGN	AAAGAAATAATGGT
M00126	V$GATA1_02	0.79125	14251	(-)	NNNNNGATANKGNN	AACCTTATAGTTAC
M00127	V$GATA1_03	0.784664	14293	(-)	RNSNNGATAANNGN	ATTCTTTTCTCTTT
M00127	V$GATA1_03	0.789319	14295	(-)	RNSNNGATAANNGN	TCTTTTCTCTTTAT
M00075	V$GATA1_01	0.775913	14321	(-)	SNNGATNNNN	TGAAATCCCT
M00076	V$GATA2_01	0.809202	14321	(-)	NNNGATRNNN	TGAAATCCCT
M00126	V$GATA1_02	0.781875	14353	(-)	NNNNNGATANKGNN	TTCCCTTTCTTTGA
M00077	V$GATA3_01	0.823217	14355	(-)	NNGATARNG	CCCTTTCTT
M00076	V$GATA2_01	0.785295	14367	(+)	NNNGATRNNN	GCTCATTGCC
M00127	V$GATA1_03	0.808672	14491	(+)	RNSNNGATAANNGN	ACTTTGATAGCTGG
M00075	V$GATA1_01	0.850938	14493	(+)	SNNGATNNNN	TTTGATAGCT

M00076	V$GATA2_01	0.857014	14493	(+)	NNNGATRNNN	TTTGATAGCT
M00077	V$GATA3_01	0.831192	14494	(+)	NNGATARNG	TTGATAGCT
M00075	V$GATA1_01	0.797137	14561	(+)	SNNGATNNNN	AGTGATTTAT
M00075	V$GATA1_01	0.807996	14576	(-)	SNNGATNNNN	ACCCCTCAGG
M00075	V$GATA1_01	0.782823	14595	(-)	SNNGATNNNN	AAAAATCCTT
M00076	V$GATA2_01	0.811908	14595	(-)	NNNGATRNNN	AAAAATCCTT
M00075	V$GATA1_01	0.790721	14621	(+)	SNNGATNNNN	TGTGATTTAC
M00076	V$GATA2_01	0.78304	14621	(+)	NNNGATRNNN	TGTGATTTAC
M00076	V$GATA2_01	0.839423	14628	(+)	NNNGATRNNN	TACCATAGGG
M00127	V$GATA1_03	0.827536	14652	(-)	RNSNNGATAANNGN	TCTGTTTTCCTCAC
M00075	V$GATA1_01	0.839585	14671	(-)	SNNGATNNNN	AAACATCTCT
M00076	V$GATA2_01	0.841678	14671	(-)	NNNGATRNNN	AAACATCTCT
M00075	V$GATA1_01	0.866732	14681	(+)	SNNGATNNNN	TGTGATGCGA
M00076	V$GATA2_01	0.850699	14681	(+)	NNNGATRNNN	TGTGATGCGA
M00127	V$GATA1_03	0.807202	14685	(+)	RNSNNGATAANNGN	ATGCGAATAGCCAT
M00076	V$GATA2_01	0.789355	14693	(-)	NNNGATRNNN	AGCCATGTCT
M00075	V$GATA1_01	0.789733	14714	(+)	SNNGATNNNN	AATGATTCAT
M00075	V$GATA1_01	0.828727	14739	(+)	SNNGATNNNN	ACTGATTTCT
M00076	V$GATA2_01	0.813261	14739	(+)	NNNGATRNNN	ACTGATTTCT
M00203	V$GATA_C	0.867661	14810	(-)	NGATAAGNMNN	GACGCCTATCC
M00126	V$GATA1_02	0.830625	14811	(-)	NNNNNGATANKGNN	ACGCCTATCCATCT
M00127	V$GATA1_03	0.901519	14811	(-)	RNSNNGATAANNGN	ACGCCTATCCATCT
M00075	V$GATA1_01	0.814906	14813	(-)	SNNGATNNNN	GCCTATCCAT
M00076	V$GATA2_01	0.83401	14813	(-)	NNNGATRNNN	GCCTATCCAT
M00077	V$GATA3_01	0.846256	14813	(-)	NNGATARNG	GCCTATCCA
M00075	V$GATA1_01	0.908193	14817	(-)	SNNGATNNNN	ATCCATCTGG
M00076	V$GATA2_01	0.879567	14817	(-)	NNNGATRNNN	ATCCATCTGG
M00127	V$GATA1_03	0.795688	14829	(-)	RNSNNGATAANNGN	CCTGGTATTACCTT
M00075	V$GATA1_01	0.858835	14861	(+)	SNNGATNNNN	ACTGATGCCC
M00076	V$GATA2_01	0.857014	14861	(+)	NNNGATRNNN	ACTGATGCCC
M00075	V$GATA1_01	0.799605	14881	(-)	SNNGATNNNN	TGACATCCCT
M00076	V$GATA2_01	0.845286	14881	(-)	NNNGATRNNN	TGACATCCCT
M00075	V$GATA1_01	0.825271	14888	(-)	SNNGATNNNN	CCTAATCCCA

M00076	V$GATA2_01	0.857014	14888	(-)	NNNGATRNNN	CCTAATCCCA
M00077	V$GATA3_01	0.892778	14888	(-)	NNGATARNG	CCTAATCCC
M00076	V$GATA2_01	0.815968	14923	(-)	NNNGATRNNN	TACTATGCCA
M00127	V$GATA1_03	0.789074	14935	(+)	RNSNNGATAANNGN	GTGGTGGTAAGTTC
M00203	V$GATA_C	0.87667	14969	(-)	NGATAAGNMNN	CTGCCGTATCA
M00126	V$GATA1_02	0.869375	14970	(-)	NNNNNGATANKGNN	TGCCGTATCAAGGG
M00127	V$GATA1_03	0.781725	14970	(-)	RNSNNGATAANNGN	TGCCGTATCAAGGG
M00075	V$GATA1_01	0.909674	14972	(-)	SNNGATNNNN	CCGTATCAAG
M00076	V$GATA2_01	0.841227	14972	(-)	NNNGATRNNN	CCGTATCAAG
M00077	V$GATA3_01	0.85556	14972	(-)	NNGATARNG	CCGTATCAA
M00127	V$GATA1_03	0.808427	14980	(+)	RNSNNGATAANNGN	AGGGAAATAGAAGA
M00076	V$GATA2_01	0.814163	15047	(-)	NNNGATRNNN	TCCTATGCTA
M00126	V$GATA1_02	0.795938	15077	(+)	NNNNNGATANKGNN	TTGATGATAACTTT
M00127	V$GATA1_03	0.803038	15077	(+)	RNSNNGATAANNGN	TTGATGATAACTTT
M00128	V$GATA1_04	0.854167	15078	(+)	NNCWGATARNNNNN	TGATGATAACTTT
M00075	V$GATA1_01	0.878085	15079	(+)	SNNGATNNNN	GATGATAACT
M00076	V$GATA2_01	0.901669	15079	(+)	NNNGATRNNN	GATGATAACT
M00077	V$GATA3_01	0.827204	15080	(+)	NNGATARNG	ATGATAACT
M00203	V$GATA_C	0.83908	15081	(+)	NGATAAGNMNN	TGATAACTTTC
M00128	V$GATA1_04	0.883578	15089	(+)	NNCWGATARNNNNN	TTCTGATATATGG
M00075	V$GATA1_01	0.821323	15090	(+)	SNNGATNNNN	TCTGATATAT
M00076	V$GATA2_01	0.85115	15090	(+)	NNNGATRNNN	TCTGATATAT
M00076	V$GATA2_01	0.792061	15124	(+)	NNNGATRNNN	GAACATGTGG
M00126	V$GATA1_02	0.832188	15137	(-)	NNNNNGATANKGNN	AGTGCTATCTATTT
M00127	V$GATA1_03	0.83317	15137	(-)	RNSNNGATAANNGN	AGTGCTATCTATTT
M00128	V$GATA1_04	0.856005	15137	(-)	NNCWGATARNNNNN	AGTGCTATCTATT
M00075	V$GATA1_01	0.786278	15139	(-)	SNNGATNNNN	TGCTATCTAT
M00076	V$GATA2_01	0.806044	15139	(-)	NNNGATRNNN	TGCTATCTAT
M00077	V$GATA3_01	0.886575	15139	(-)	NNGATARNG	TGCTATCTA
M00126	V$GATA1_02	0.85875	15146	(+)	NNNNNGATANKGNN	TATTTGATAAGTGA
M00128	V$GATA1_04	0.897059	15147	(+)	NNCWGATARNNNNN	ATTTGATAAGTGA
M00075	V$GATA1_01	0.798124	15148	(+)	SNNGATNNNN	TTTGATAAGT
M00076	V$GATA2_01	0.792963	15148	(+)	NNNGATRNNN	TTTGATAAGT
M00077	V$GATA3_01	0.83961	15149	(+)	NNGATARNG	TTGATAAGT

M00203	V$GATA_C	0.937558	15150	(+)	NGATAAGNMNN	TGATAAGTGAT
M00126	V$GATA1_02	0.845313	15153	(+)	NNNNNGATANKGNN	TAAGTGATAATCAT
M00127	V$GATA1_03	0.788339	15153	(+)	RNSNNGATAANNGN	TAAGTGATAATCAT
M00128	V$GATA1_04	0.873162	15154	(+)	NNCWGATARNNNN	AAGTGATAATCAT
M00075	V$GATA1_01	0.827246	15155	(+)	SNNGATNNNN	AGTGATAATC
M00076	V$GATA2_01	0.840325	15155	(+)	NNNGATRNNN	AGTGATAATC
M00077	V$GATA3_01	0.860434	15156	(+)	NNGATARNG	GTGATAATC
M00127	V$GATA1_03	0.817001	15156	(-)	RNSNNGATAANNGN	GTGATAATCATTCT
M00203	V$GATA_C	0.877912	15157	(+)	NGATAAGNMNN	TGATAATCATT
M00075	V$GATA1_01	0.774926	15158	(-)	SNNGATNNNN	GATAATCATT
M00127	V$GATA1_03	0.820676	15168	(+)	RNSNNGATAANNGN	CTGATGAAAATAGG
M00127	V$GATA1_03	0.846644	15171	(+)	RNSNNGATAANNGN	ATGAAAATAGGAGG
M00127	V$GATA1_03	0.792504	15179	(+)	RNSNNGATAANNGN	AGGAGGAAAATTTT
M00203	V$GATA_C	0.861448	15211	(-)	NGATAAGNMNN	CAACTTTATCC
M00127	V$GATA1_03	0.890005	15212	(-)	RNSNNGATAANNGN	AACTTTATCCTCCT
M00128	V$GATA1_04	0.820466	15212	(-)	NNCWGATARNNNN	AACTTTATCCTCC
M00075	V$GATA1_01	0.824778	15214	(-)	SNNGATNNNN	CTTTATCCTC
M00076	V$GATA2_01	0.881371	15214	(-)	NNNGATRNNN	CTTTATCCTC
M00077	V$GATA3_01	0.868409	15214	(-)	NNGATARNG	CTTTATCCT
M00076	V$GATA2_01	0.785295	15235	(-)	NNNGATRNNN	GCTAATCTTT
M00077	V$GATA3_01	0.853788	15235	(-)	NNGATARNG	GCTAATCTT
M00127	V$GATA1_03	0.798873	15258	(-)	RNSNNGATAANNGN	TGGATTATACCCAT
M00076	V$GATA2_01	0.789806	15266	(-)	NNNGATRNNN	ACCCATGATA
M00075	V$GATA1_01	0.846496	15269	(+)	SNNGATNNNN	CATGATATAC
M00076	V$GATA2_01	0.848444	15269	(+)	NNNGATRNNN	CATGATATAC
M00203	V$GATA_C	0.852128	15271	(+)	NGATAAGNMNN	TGATATACATT
M00127	V$GATA1_03	0.813327	15293	(+)	RNSNNGATAANNGN	TGGGGGATAAACTG
M00075	V$GATA1_01	0.827246	15295	(+)	SNNGATNNNN	GGGGATAAAC
M00076	V$GATA2_01	0.872801	15295	(+)	NNNGATRNNN	GGGGATAAAC
M00075	V$GATA1_01	0.884008	15306	(+)	SNNGATNNNN	GCAGATTTTG
M00076	V$GATA2_01	0.865584	15306	(+)	NNNGATRNNN	GCAGATTTTG
M00077	V$GATA3_01	0.862206	15307	(+)	NNGATARNG	CAGATTTTG
M00126	V$GATA1_02	0.778125	15326	(-)	NNNNNGATANKGNN	AGTCCTAACTTGAA

M00075	V$GATA1_01	0.816387	15336	(-)	SNNGATNNNN	TGAAATCCTG
M00076	V$GATA2_01	0.84258	15336	(-)	NNNGATRNNN	TGAAATCCTG
M00203	V$GATA_C	0.834731	15339	(-)	NGATAAGNMNN	AATCCTGATCT
M00127	V$GATA1_03	0.784664	15340	(-)	RNSNNGATAANNGN	ATCCTGATCTTTTT
M00128	V$GATA1_04	0.815564	15340	(-)	NNCWGATARNNNN	ATCCTGATCTTTT
M00075	V$GATA1_01	0.870188	15342	(+)	SNNGATNNNN	CCTGATCTTT
M00076	V$GATA2_01	0.835814	15342	(+)	NNNGATRNNN	CCTGATCTTT
M00076	V$GATA2_01	0.801985	15342	(-)	NNNGATRNNN	CCTGATCTTT
M00077	V$GATA3_01	0.838724	15342	(-)	NNGATARNG	CCTGATCTT
M00127	V$GATA1_03	0.785154	15348	(-)	RNSNNGATAANNGN	CTTTTTATTTTCGC
M00075	V$GATA1_01	0.831688	15378	(+)	SNNGATNNNN	AATGATGTTT
M00076	V$GATA2_01	0.822282	15378	(+)	NNNGATRNNN	AATGATGTTT
M00075	V$GATA1_01	0.771964	15381	(+)	SNNGATNNNN	GATGTTTGTT
M00127	V$GATA1_03	0.792014	15388	(-)	RNSNNGATAANNGN	GTTTTTATAACCTT
M00075	V$GATA1_01	0.807996	15405	(+)	SNNGATNNNN	GGTGATTAAA
M00077	V$GATA3_01	0.838724	15406	(+)	NNGATARNG	GTGATTAAA
M00076	V$GATA2_01	0.792963	15429	(+)	NNNGATRNNN	AAGCATACTG
M00076	V$GATA2_01	0.78304	15556	(-)	NNNGATRNNN	AATTATGGTC
M00203	V$GATA_C	0.878844	15581	(-)	NGATAAGNMNN	ATTGCTTGTCT
M00127	V$GATA1_03	0.823861	15582	(-)	RNSNNGATAANNGN	TTGCTTGTCTTGAT
M00127	V$GATA1_03	0.792014	15588	(-)	RNSNNGATAANNGN	GTCTTGATCCTCTT
M00075	V$GATA1_01	0.819842	15590	(+)	SNNGATNNNN	CTTGATCCTC
M00075	V$GATA1_01	0.773445	15590	(-)	SNNGATNNNN	CTTGATCCTC
M00076	V$GATA2_01	0.81687	15590	(-)	NNNGATRNNN	CTTGATCCTC
M00128	V$GATA1_04	0.813419	15596	(-)	NNCWGATARNNNN	CCTCTTTTCATAT
M00126	V$GATA1_02	0.81125	15685	(+)	NNNNNGATANKGNN	ACTTAGATAATATC
M00127	V$GATA1_03	0.799118	15685	(+)	RNSNNGATAANNGN	ACTTAGATAATATC
M00077	V$GATA3_01	0.874169	15688	(+)	NNGATARNG	TAGATAATA
M00203	V$GATA_C	0.870457	15689	(+)	NGATAAGNMNN	AGATAATATCA
M00126	V$GATA1_02	0.825	15690	(-)	NNNNNGATANKGNN	GATAATATCAATAA
M00075	V$GATA1_01	0.770484	15869	(+)	SNNGATNNNN	TATGATCACT
M00076	V$GATA2_01	0.822733	15869	(+)	NNNGATRNNN	TATGATCACT
M00076	V$GATA2_01	0.831304	15900	(+)	NNNGATRNNN	TCACATAGTT

M00203	V$GATA_C	0.877291	15916	(-)	NGATAAGNMNN	AGTATTTGTCT
M00128	V$GATA1_04	0.8269	15923	(+)	NNCWGATARNNNN	GTCTGTTAAGAAG
M00127	V$GATA1_03	0.837825	15930	(+)	RNSNNGATAANNGN	AAGAAGAAAACAGA
M00075	V$GATA1_01	0.787266	15955	(+)	SNNGATNNNN	ATAGATGTGT
M00076	V$GATA2_01	0.812359	16080	(+)	NNNGATRNNN	TGACATACGT
M00076	V$GATA2_01	0.79161	16114	(+)	NNNGATRNNN	TGCCATATTT
M00075	V$GATA1_01	0.897828	16176	(-)	SNNGATNNNN	AAAAATCAGG
M00076	V$GATA2_01	0.835814	16176	(-)	NNNGATRNNN	AAAAATCAGG
M00076	V$GATA2_01	0.788904	16192	(-)	NNNGATRNNN	GGGTATGTGT
M00075	V$GATA1_01	0.772952	16233	(-)	SNNGATNNNN	CTTCATCTTA
M00076	V$GATA2_01	0.824087	16233	(-)	NNNGATRNNN	CTTCATCTTA
M00077	V$GATA3_01	0.859991	16233	(-)	NNGATARNG	CTTCATCTT
M00075	V$GATA1_01	0.772952	16241	(+)	SNNGATNNNN	TAAGATGAAG
M00076	V$GATA2_01	0.824087	16241	(+)	NNNGATRNNN	TAAGATGAAG
M00077	V$GATA3_01	0.859991	16242	(+)	NNGATARNG	AAGATGAAG
M00127	V$GATA1_03	0.792994	16245	(+)	RNSNNGATAANNGN	ATGAAGAGAAAATG
M00203	V$GATA_C	0.836285	16265	(-)	NGATAAGNMNN	AAAGTGTATCT
M00126	V$GATA1_02	0.816562	16266	(-)	NNNNNGATANKGNN	AAGTGTATCTTAAC
M00075	V$GATA1_01	0.786278	16268	(-)	SNNGATNNNN	GTGTATCTTA
M00076	V$GATA2_01	0.861976	16268	(-)	NNNGATRNNN	GTGTATCTTA
M00076	V$GATA2_01	0.814163	16304	(+)	NNNGATRNNN	CAGCATAACT
M00126	V$GATA1_02	0.785937	16336	(+)	NNNNNGATANKGNN	TTACAGATGGGATT
M00075	V$GATA1_01	0.85538	16338	(+)	SNNGATNNNN	ACAGATGGGA
M00076	V$GATA2_01	0.854759	16338	(+)	NNNGATRNNN	ACAGATGGGA
M00077	V$GATA3_01	0.888347	16339	(+)	NNGATARNG	CAGATGGGA
M00075	V$GATA1_01	0.791708	16343	(+)	SNNGATNNNN	TGGGATTGAA
M00076	V$GATA2_01	0.819125	16343	(+)	NNNGATRNNN	TGGGATTGAA
M00077	V$GATA3_01	0.84537	16344	(+)	NNGATARNG	GGGATTGAA
M00203	V$GATA_C	0.86269	16375	(-)	NGATAAGNMNN	TTCATCTATCA
M00126	V$GATA1_02	0.851562	16376	(-)	NNNNNGATANKGNN	TCATCTATCATGTC
M00127	V$GATA1_03	0.843214	16376	(-)	RNSNNGATAANNGN	TCATCTATCATGTC
M00128	V$GATA1_04	0.885417	16376	(-)	NNCWGATARNNNN	TCATCTATCATGT
M00075	V$GATA1_01	0.908193	16378	(-)	SNNGATNNNN	ATCTATCATG

M00076	V$GATA2_01	0.888588	16378	(-)	NNNGATRNNN	ATCTATCATG
M00077	V$GATA3_01	0.851573	16378	(-)	NNGATARNG	ATCTATCAT
M00076	V$GATA2_01	0.793866	16396	(-)	NNNGATRNNN	TTCTATCCAA
M00077	V$GATA3_01	0.837395	16396	(-)	NNGATARNG	TTCTATCCA
M00075	V$GATA1_01	0.856367	16432	(-)	SNNGATNNNN	GACAATCTCA
M00076	V$GATA2_01	0.870997	16432	(-)	NNNGATRNNN	GACAATCTCA
M00077	V$GATA3_01	0.8786	16432	(-)	NNGATARNG	GACAATCTC
M00075	V$GATA1_01	0.817868	16482	(+)	SNNGATNNNN	TTAGATTGTG
M00076	V$GATA2_01	0.797925	16482	(+)	NNNGATRNNN	TTAGATTGTG
M00077	V$GATA3_01	0.903855	16483	(+)	NNGATARNG	TAGATTGTG
M00075	V$GATA1_01	0.770977	16492	(-)	SNNGATNNNN	TAACATCCTT
M00076	V$GATA2_01	0.818223	16492	(-)	NNNGATRNNN	TAACATCCTT
M00126	V$GATA1_02	0.8	16539	(-)	NNNNNGATANKGNN	AGTAGTATCATGCT
M00127	V$GATA1_03	0.849829	16539	(-)	RNSNNGATAANNGN	AGTAGTATCATGCT
M00075	V$GATA1_01	0.886476	16541	(-)	SNNGATNNNN	TAGTATCATG
M00076	V$GATA2_01	0.873703	16541	(-)	NNNGATRNNN	TAGTATCATG
M00077	V$GATA3_01	0.824103	16541	(-)	NNGATARNG	TAGTATCAT
M00075	V$GATA1_01	0.847483	16627	(-)	SNNGATNNNN	TTGAATCACC
M00076	V$GATA2_01	0.782589	16627	(-)	NNNGATRNNN	TTGAATCACC
M00075	V$GATA1_01	0.84304	16648	(-)	SNNGATNNNN	TGCCATCATT
M00076	V$GATA2_01	0.847993	16648	(-)	NNNGATRNNN	TGCCATCATT
M00077	V$GATA3_01	0.83385	16648	(-)	NNGATARNG	TGCCATCAT
M00075	V$GATA1_01	0.835637	16680	(+)	SNNGATNNNN	TGAGATTTTG
M00076	V$GATA2_01	0.85115	16680	(+)	NNNGATRNNN	TGAGATTTTG
M00077	V$GATA3_01	0.885246	16681	(+)	NNGATARNG	GAGATTTTG
M00203	V$GATA_C	0.836906	16682	(-)	NGATAAGNMNN	AGATTTTGTCT
M00162	V$OCT1_06	0.800781	34	(+)	CWNAWTKWSATRYN	CAGTCTTGGGTTCA
M00138	V$OCT1_04	0.783982	46	(-)	NNNNNNNWATGCAAATNNNWNNW	CAAGGTATTATGTATACATATAA
M00138	V$OCT1_04	0.820577	47	(+)	NNNNNNNWATGCAAATNNNWNNW	AAGGTATTATGTATACATATAAA
M00136	V$OCT1_02	0.779101	49	(+)	NNGAATATKCANNNN	GGTATTATGTATACA
M00138	V$OCT1_04	0.866583	51	(+)	NNNNNNNWATGCAAATNNNWNNW	TATTATGTATACATATAAAAAAA
M00138	V$OCT1_04	0.801757	53	(+)	NNNNNNNWATGCAAATNNNWNNW	TTATGTATACATATAAAAAAACT
M00138	V$OCT1_04	0.791928	55	(+)	NNNNNNNWATGCAAATNNNWNNW	ATGTATACATATAAAAAAACTTA

M00138	V$OCT1_04	0.807821	56	(-)	NNNNNNNWATGCAAATNNNWNNW	TGTATACATATAAAAAAACTTAT
M00138	V$OCT1_04	0.813885	57	(+)	NNNNNNNWATGCAAATNNNWNNW	GTATACATATAAAAAAACTTATT
M00162	V$OCT1_06	0.813672	62	(-)	CWNAWTKWSATRYN	CATATAAAAAAACT
M00162	V$OCT1_06	0.832031	74	(+)	CWNAWTKWSATRYN	CTTATTTTATTTTC
M00138	V$OCT1_04	0.789628	75	(-)	NNNNNNNWATGCAAATNNNWNNW	TTATTTTATTTTCCTCTGTCTCA
M00162	V$OCT1_06	0.829297	96	(-)	CWNAWTKWSATRYN	CATCTTTAATTCTT
M00138	V$OCT1_04	0.810121	98	(-)	NNNNNNNWATGCAAATNNNWNNW	TCTTTAATTCTTCACTAATACGC
M00136	V$OCT1_02	0.799675	111	(+)	NNGAATATKCANNNN	ACTAATACGCAGTTG
M00137	V$OCT1_03	0.876729	136	(-)	NNNRTAATNANNN	ATGTGATTGCAAG
M00162	V$OCT1_06	0.821484	139	(-)	CWNAWTKWSATRYN	TGATTGCAAGTATT
M00162	V$OCT1_06	0.813672	153	(+)	CWNAWTKWSATRYN	GGTACTTTCCTATG
M00138	V$OCT1_04	0.804057	173	(-)	NNNNNNNWATGCAAATNNNWNNW	TGTTAGCTTAAAAATATATTTGC
M00138	V$OCT1_04	0.788164	183	(-)	NNNNNNNWATGCAAATNNNWNNW	AAAATATATTTGCAAATGTTGAT
M00138	V$OCT1_04	0.837725	184	(+)	NNNNNNNWATGCAAATNNNWNNW	AAATATATTTGCAAATGTTGATA
M00135	V$OCT1_01	0.842617	185	(-)	NNNNWTATGCAAATNTNNN	AATATATTTGCAAATGTTG
M00135	V$OCT1_01	0.858642	186	(+)	NNNNWTATGCAAATNTNNN	ATATATTTGCAAATGTTGA
M00195	V$OCT1_Q6	0.814683	188	(+)	NNNNATGCAAATNAN	ATATTTGCAAATGTT
M00162	V$OCT1_06	0.834375	189	(-)	CWNAWTKWSATRYN	TATTTGCAAATGTT
M00138	V$OCT1_04	0.801757	190	(+)	NNNNNNNWATGCAAATNNNWNNW	ATTTGCAAATGTTGATACTATCT
M00162	V$OCT1_06	0.878516	195	(+)	CWNAWTKWSATRYN	CAAATGTTGATACT
M00162	V$OCT1_06	0.800781	210	(-)	CWNAWTKWSATRYN	TCTATCTCAGAGCT
M00162	V$OCT1_06	0.945312	231	(+)	CWNAWTKWSATRYN	AAAAATTAAATACT
M00162	V$OCT1_06	0.813672	231	(-)	CWNAWTKWSATRYN	AAAAATTAAATACT
M00162	V$OCT1_06	0.885547	232	(-)	CWNAWTKWSATRYN	AAAATTAAATACTT
M00248	V$OCT1_07	0.793301	233	(-)	TNTATGNTAATT	AAATTAAATACT
M00137	V$OCT1_03	0.840774	245	(+)	NNNRTAATNANNN	TTTATAAAGACCA
M00138	V$OCT1_04	0.832079	275	(+)	NNNNNNNWATGCAAATNNNWNNW	TAAAATAAATTCTTATATACTGA
M00162	V$OCT1_06	0.821484	275	(-)	CWNAWTKWSATRYN	TAAAATAAATTCTT
M00162	V$OCT1_06	0.842187	282	(+)	CWNAWTKWSATRYN	AATTCTTATATACT
M00138	V$OCT1_04	0.780217	292	(+)	NNNNNNNWATGCAAATNNNWNNW	TACTGAAAATGTAGATACATAAC
M00136	V$OCT1_02	0.788847	294	(+)	NNGAATATKCANNNN	CTGAAAATGTAGATA
M00162	V$OCT1_06	0.860156	297	(+)	CWNAWTKWSATRYN	AAAATGTAGATACA
M00138	V$OCT1_04	0.789419	298	(+)	NNNNNNNWATGCAAATNNNWNNW	AAATGTAGATACATAACTTCAGT

M00138	V$OCT1_04	0.833752	321	(+)	NNNNNNNWATGCAAATNNNNWNNW	ATAGATTTATGGTAAAATAATTT
M00135	V$OCT1_01	0.794353	323	(+)	NNNNWTATGCAAATNTNNN	AGATTTATGGTAAAATAAT
M00248	V$OCT1_07	0.795209	326	(+)	TNTATGNTAATT	TTTATGGTAAAA
M00138	V$OCT1_04	0.856545	333	(-)	NNNNNNNWATGCAAATNNNNWNNW	TAAAATAATTTGAATCATTTTTG
M00135	V$OCT1_01	0.736169	335	(-)	NNNNWTATGCAAATNTNNN	AAATAATTTGAATCATTTT
M00162	V$OCT1_06	0.847266	336	(+)	CWNAWTKWSATRYN	AATAATTTGAATCA
M00195	V$OCT1_Q6	0.822871	337	(-)	NNNNATGCAAATNAN	ATAATTTGAATCATT
M00248	V$OCT1_07	0.797965	339	(-)	TNTATGNTAATT	AATTTGAATCAT
M00162	V$OCT1_06	0.826172	349	(+)	CWNAWTKWSATRYN	ATTTTTGTCAAATT
M00162	V$OCT1_06	0.867969	369	(+)	CWNAWTKWSATRYN	AATGTTGTCATACA
M00138	V$OCT1_04	0.793601	370	(+)	NNNNNNNWATGCAAATNNNNWNNW	ATGTTGTCATACAGAATAATTTA
M00138	V$OCT1_04	0.824969	380	(+)	NNNNNNNWATGCAAATNNNNWNNW	ACAGAATAATTTATAATTTTTTT
M00138	V$OCT1_04	0.816395	382	(-)	NNNNNNNWATGCAAATNNNNWNNW	AGAATAATTTATAATTTTTTTGT
M00195	V$OCT1_Q6	0.809225	385	(-)	NNNNATGCAAATNAN	ATAATTTATAATTTT
M00137	V$OCT1_03	0.858949	389	(+)	NNNRTAATNANNN	TTTATAATTTTTT
M00138	V$OCT1_04	0.780427	396	(-)	NNNNNNNWATGCAAATNNNNWNNW	TTTTTTTGTTTTCATGGTGAAGA
M00136	V$OCT1_02	0.798322	425	(-)	NNGAATATKCANNNN	CTGTAGAATATTTCG
M00162	V$OCT1_06	0.806641	447	(+)	CWNAWTKWSATRYN	TTTTATTCCATGTA
M00162	V$OCT1_06	0.846094	453	(-)	CWNAWTKWSATRYN	TCCATGTAATTAGG
M00137	V$OCT1_03	0.934413	455	(+)	NNNRTAATNANNN	CATGTAATTAGGT
M00137	V$OCT1_03	0.871197	456	(-)	NNNRTAATNANNN	ATGTAATTAGGTT
M00138	V$OCT1_04	0.851108	456	(+)	NNNNNNNWATGCAAATNNNNWNNW	ATGTAATTAGGTTAATAAAATTA
M00135	V$OCT1_01	0.736551	458	(+)	NNNNWTATGCAAATNTNNN	GTAATTAGGTTAATAAAAT
M00162	V$OCT1_06	0.826562	470	(+)	CWNAWTKWSATRYN	ATAAAATTAATTTT
M00162	V$OCT1_06	0.826562	472	(-)	CWNAWTKWSATRYN	AAAATTAATTTTAT
M00137	V$OCT1_03	0.860925	503	(+)	NNNRTAATNANNN	TAGACAATTAGAT
M00162	V$OCT1_06	0.945312	505	(+)	CWNAWTKWSATRYN	GACAATTAGATATA
M00138	V$OCT1_04	0.796947	506	(+)	NNNNNNNWATGCAAATNNNNWNNW	ACAATTAGATATAAATGATTACT
M00138	V$OCT1_04	0.81744	507	(-)	NNNNNNNWATGCAAATNNNNWNNW	CAATTAGATATAAATGATTACTT
M00137	V$OCT1_03	0.894508	518	(-)	NNNRTAATNANNN	AAATGATTACTTT
M00138	V$OCT1_04	0.793392	524	(+)	NNNNNNNWATGCAAATNNNNWNNW	TTACTTTTATAAAGATGATTAAA
M00138	V$OCT1_04	0.781681	525	(-)	NNNNNNNWATGCAAATNNNNWNNW	TACTTTTATAAAGATGATTAAAT
M00137	V$OCT1_03	0.893718	529	(+)	NNNRTAATNANNN	TTTATAAAGATGA

M00138	V$OCT1_04	0.819113	530	(+)	NNNNNNNWATGCAAATNNNNWNNW	TTATAAAGATGATTAAATTTGGA
M00162	V$OCT1_06	0.837109	537	(+)	CWNAWTKWSATRYN	GATGATTAAATTTG
M00138	V$OCT1_04	0.909243	539	(-)	NNNNNNNWATGCAAATNNNNWNNW	TGATTAAATTTGGATATTTATAA
M00138	V$OCT1_04	0.799038	540	(+)	NNNNNNNWATGCAAATNNNNWNNW	GATTAAATTTGGATATTTATAAG
M00135	V$OCT1_01	0.887066	541	(-)	NNNNWTATGCAAATNTNNN	ATTAAATTTGGATATTTAT
M00135	V$OCT1_01	0.730065	542	(+)	NNNNWTATGCAAATNTNNN	TTAAATTTGGATATTTATA
M00162	V$OCT1_06	0.873437	543	(+)	CWNAWTKWSATRYN	TAAATTTGGATATT
M00195	V$OCT1_Q6	0.791212	543	(-)	NNNNATGCAAATNAN	TAAATTTGGATATTT
M00136	V$OCT1_02	0.859773	545	(-)	NNGAATATKCANNNN	AATTTGGATATTTAT
M00138	V$OCT1_04	0.793392	545	(-)	NNNNNNNWATGCAAATNNNNWNNW	AATTTGGATATTTATAAGGATAC
M00248	V$OCT1_07	0.854993	545	(-)	TNTATGNTAATT	AATTTGGATATT
M00138	V$OCT1_04	0.820368	546	(+)	NNNNNNNWATGCAAATNNNNWNNW	ATTTGGATATTTATAAGGATACA
M00136	V$OCT1_02	0.785869	548	(+)	NNGAATATKCANNNN	TTGGATATTTATAAG
M00138	V$OCT1_04	0.851736	556	(+)	NNNNNNNWATGCAAATNNNNWNNW	TTATAAGGATACAAATATATGAA
M00135	V$OCT1_01	0.77356	558	(+)	NNNNWTATGCAAATNTNNN	ATAAGGATACAAATATATG
M00138	V$OCT1_04	0.850899	566	(+)	NNNNNNNWATGCAAATNNNNWNNW	ACAAATATATGAAAATAATAAAC
M00135	V$OCT1_01	0.813239	568	(+)	NNNNWTATGCAAATNTNNN	AAATATATGAAAATAATAA
M00195	V$OCT1_Q6	0.901201	570	(+)	NNNNATGCAAATNAN	ATATATGAAAATAAT
M00162	V$OCT1_06	0.942578	571	(-)	CWNAWTKWSATRYN	TATATGAAAATAAT
M00248	V$OCT1_07	0.794785	571	(+)	TNTATGNTAATT	TATATGAAAATA
M00162	V$OCT1_06	0.812109	597	(-)	CWNAWTKWSATRYN	GGCTTATAAATATG
M00137	V$OCT1_03	0.88147	599	(+)	NNNRTAATNANNN	CTTATAAATATGT
M00138	V$OCT1_04	0.787118	599	(-)	NNNNNNNWATGCAAATNNNNWNNW	CTTATAAATATGTCTTTTTAAC
M00138	V$OCT1_04	0.791928	600	(+)	NNNNNNNWATGCAAATNNNNWNNW	TTATAAATATGTCTTTTTAACA
M00136	V$OCT1_02	0.783433	602	(+)	NNGAATATKCANNNN	ATAAATATGTCTTTT
M00162	V$OCT1_06	0.828906	604	(+)	CWNAWTKWSATRYN	AAATATGTCTTTTT
M00162	V$OCT1_06	0.875781	605	(-)	CWNAWTKWSATRYN	AATATGTCTTTTTT
M00162	V$OCT1_06	0.855078	662	(-)	CWNAWTKWSATRYN	TGTATAAAATAATC
M00137	V$OCT1_03	0.852627	675	(-)	NNNRTAATNANNN	CTGTCATTAGTCT
M00138	V$OCT1_04	0.78189	688	(+)	NNNNNNNWATGCAAATNNNNWNNW	GCTGACTAAGGTATAAACAAATT
M00138	V$OCT1_04	0.781054	694	(+)	NNNNNNNWATGCAAATNNNNWNNW	TAAGGTATAAACAAATTTCATGT
M00135	V$OCT1_01	0.791683	696	(+)	NNNNWTATGCAAATNTNNN	AGGTATAAACAAATTTCAT
M00137	V$OCT1_03	0.856183	697	(+)	NNNRTAATNANNN	GGTATAAACAAAT

M00162	V$OCT1_06	0.889062	704	(+)	CWNAWTKWSATRYN	ACAAATTTCATGTA
M00138	V$OCT1_04	0.807821	705	(+)	NNNNNNNWATGCAAATNNNNWNNW	CAAATTTCATGTATAATCTAATT
M00162	V$OCT1_06	0.885547	705	(-)	CWNAWTKWSATRYN	CAAATTTCATGTAT
M00136	V$OCT1_02	0.788847	710	(-)	NNGAATATKCANNNN	TTCATGTATAATCTA
M00138	V$OCT1_04	0.782936	711	(+)	NNNNNNNWATGCAAATNNNNWNNW	TCATGTATAATCTAATTTTTCTT
M00135	V$OCT1_01	0.753338	713	(+)	NNNNWTATGCAAATNTNNN	ATGTATAATCTAATTTTTC
M00137	V$OCT1_03	0.855788	714	(+)	NNNRTAATNANNN	TGTATAATCTAAT
M00248	V$OCT1_07	0.838457	716	(+)	TNTATGNTAATT	TATAATCTAATT
M00162	V$OCT1_06	0.868359	717	(-)	CWNAWTKWSATRYN	ATAATCTAATTTTT
M00135	V$OCT1_01	0.753148	721	(-)	NNNNWTATGCAAATNTNNN	TCTAATTTTTCTTATTTAT
M00137	V$OCT1_03	0.84196	730	(-)	NNNRTAATNANNN	TCTTATTTATCTG
M00162	V$OCT1_06	0.842187	736	(+)	CWNAWTKWSATRYN	TTATCTGAAATGCA
M00162	V$OCT1_06	0.821094	737	(-)	CWNAWTKWSATRYN	TATCTGAAATGCAT
M00162	V$OCT1_06	0.847656	740	(+)	CWNAWTKWSATRYN	CTGAAATGCATTTT
M00195	V$OCT1_Q6	0.801856	740	(-)	NNNNATGCAAATNAN	CTGAAATGCATTTTT
M00136	V$OCT1_02	0.824851	742	(-)	NNGAATATKCANNNN	GAAATGCATTTTTTC
M00162	V$OCT1_06	0.829297	742	(-)	CWNAWTKWSATRYN	GAAATGCATTTTTT
M00138	V$OCT1_04	0.781681	754	(+)	NNNNNNNWATGCAAATNNNNWNNW	TTCAGCACATATAAATTTATGTA
M00138	V$OCT1_04	0.81033	755	(-)	NNNNNNNWATGCAAATNNNNWNNW	TCAGCACATATAAATTTATGTAT
M00135	V$OCT1_01	0.7314	756	(+)	NNNNWTATGCAAATNTNNN	CAGCACATATAAATTTATG
M00162	V$OCT1_06	0.847656	759	(+)	CWNAWTKWSATRYN	CACATATAAATTTA
M00162	V$OCT1_06	0.819922	759	(-)	CWNAWTKWSATRYN	CACATATAAATTTA
M00162	V$OCT1_06	0.804297	761	(-)	CWNAWTKWSATRYN	CATATAAATTTATG
M00162	V$OCT1_06	0.821484	765	(-)	CWNAWTKWSATRYN	TAAATTTATGTATT
M00162	V$OCT1_06	0.800391	768	(+)	CWNAWTKWSATRYN	ATTTATGTATTTTA
M00162	V$OCT1_06	0.817187	769	(-)	CWNAWTKWSATRYN	TTTATGTATTTTAG
M00162	V$OCT1_06	0.808594	773	(-)	CWNAWTKWSATRYN	TGTATTTTAGGGTC
M00162	V$OCT1_06	0.800781	783	(+)	CWNAWTKWSATRYN	GGTCTTGCAATTTA
M00162	V$OCT1_06	0.821094	823	(+)	CWNAWTKWSATRYN	ATGTTTGACTTATA
M00162	V$OCT1_06	0.807031	829	(-)	CWNAWTKWSATRYN	GACTTATAATTCTG
M00136	V$OCT1_02	0.770709	846	(+)	NNGAATATKCANNNN	AATCATCTTCATACA
M00162	V$OCT1_06	0.839453	847	(+)	CWNAWTKWSATRYN	ATCATCTTCATACA
M00162	V$OCT1_06	0.868359	855	(+)	CWNAWTKWSATRYN	CATACAGTCATGTC

M00162	V$OCT1_06	0.842187	860	(+)	CWNAWTKWSATRYN	AGTCATGTCATGGA
M00162	V$OCT1_06	0.837109	861	(-)	CWNAWTKWSATRYN	GTCATGTCATGGAT
M00162	V$OCT1_06	0.804297	887	(+)	CWNAWTKWSATRYN	CAAAATTAAGTAAT
M00162	V$OCT1_06	0.800781	887	(-)	CWNAWTKWSATRYN	CAAAATTAAGTAAT
M00162	V$OCT1_06	0.868359	915	(+)	CWNAWTKWSATRYN	AAAAATTTAATGAA
M00138	V$OCT1_04	0.802593	916	(+)	NNNNNNNWATGCAAATNNNWNNW	AAAATTTAATGAAGGTTAATAGT
M00162	V$OCT1_06	0.924609	916	(-)	CWNAWTKWSATRYN	AAAATTTAATGAAG
M00162	V$OCT1_06	0.834375	959	(+)	CWNAWTKWSATRYN	ATGCTTTTCATGAA
M00195	V$OCT1_Q6	0.826692	959	(-)	NNNNATGCAAATNAN	ATGCTTTTCATGAAG
M00162	V$OCT1_06	0.855078	960	(-)	CWNAWTKWSATRYN	TGCTTTTCATGAAG
M00137	V$OCT1_03	0.873568	994	(+)	NNNRTAATNANNN	AGGATAAAGACAT
M00162	V$OCT1_06	0.806641	996	(+)	CWNAWTKWSATRYN	GATAAAGACATCTT
M00162	V$OCT1_06	0.800391	1005	(+)	CWNAWTKWSATRYN	ATCTTTTAAAAATT
M00162	V$OCT1_06	0.875781	1012	(+)	CWNAWTKWSATRYN	AAAAATTACAGATA
M00162	V$OCT1_06	0.813672	1013	(-)	CWNAWTKWSATRYN	AAAATTACAGATAA
M00162	V$OCT1_06	0.896875	1024	(+)	CWNAWTKWSATRYN	TAAAATTAAATGTA
M00162	V$OCT1_06	0.834375	1025	(-)	CWNAWTKWSATRYN	AAAATTAAATGTAA
M00162	V$OCT1_06	0.860547	1029	(+)	CWNAWTKWSATRYN	TTAAATGTAATTTA
M00195	V$OCT1_Q6	0.831332	1029	(+)	NNNNATGCAAATNAN	TTAAATGTAATTTAC
M00162	V$OCT1_06	0.945312	1030	(-)	CWNAWTKWSATRYN	TAAATGTAATTTAC
M00248	V$OCT1_07	0.812381	1030	(+)	TNTATGNTAATT	TAAATGTAATTT
M00195	V$OCT1_Q6	0.790939	1035	(-)	NNNNATGCAAATNAN	GTAATTTACCAGTTT
M00138	V$OCT1_04	0.804475	1047	(-)	NNNNNNNWATGCAAATNNNWNNW	TTTTAGTTTATCAATTTTAACAA
M00138	V$OCT1_04	0.780636	1052	(+)	NNNNNNNWATGCAAATNNNWNNW	GTTTATCAATTTTAACAAATCCA
M00138	V$OCT1_04	0.800711	1053	(-)	NNNNNNNWATGCAAATNNNWNNW	TTTATCAATTTTAACAAATCCAA
M00162	V$OCT1_06	0.885547	1067	(-)	CWNAWTKWSATRYN	CAAATCCAATGATC
M00162	V$OCT1_06	0.834375	1080	(+)	CWNAWTKWSATRYN	CTACAGGAAATTTT
M00162	V$OCT1_06	0.875781	1081	(-)	CWNAWTKWSATRYN	TACAGGAAATTTTT
M00138	V$OCT1_04	0.82455	1087	(-)	NNNNNNNWATGCAAATNNNWNNW	AAATTTTTTTTAATTTTTATTG
M00162	V$OCT1_06	0.826562	1091	(+)	CWNAWTKWSATRYN	TTTTTTTTAATTTT
M00248	V$OCT1_07	0.831673	1091	(+)	TNTATGNTAATT	TTTTTTTTAATT
M00138	V$OCT1_04	0.868465	1093	(-)	NNNNNNNWATGCAAATNNNWNNW	TTTTTTAATTTTTATTGTCATAT
M00195	V$OCT1_Q6	0.804858	1097	(-)	NNNNATGCAAATNAN	TTAATTTTTATTGTC

M00138	V$OCT1_04	0.840652	1099	(-)	NNNNNNNWATGCAAATNNNNWNNW	AATTTTTATTGTCATATTTTAAA
M00135	V$OCT1_01	0.745326	1101	(-)	NNNNWTATGCAAATNTNNN	TTTTTATTGTCATATTTTA
M00162	V$OCT1_06	0.886328	1103	(+)	CWNAWTKWSATRYN	TTTATTGTCATATT
M00195	V$OCT1_Q6	0.813046	1103	(-)	NNNNATGCAAATNAN	TTTATTGTCATATTT
M00138	V$OCT1_04	0.794437	1107	(-)	NNNNNNNWATGCAAATNNNNWNNW	TTGTCATATTTTAAAATTTGTAA
M00162	V$OCT1_06	0.865625	1111	(+)	CWNAWTKWSATRYN	CATATTTTAAAATT
M00162	V$OCT1_06	0.821484	1111	(-)	CWNAWTKWSATRYN	CATATTTTAAAATT
M00162	V$OCT1_06	0.800391	1112	(+)	CWNAWTKWSATRYN	ATATTTTAAAATTT
M00162	V$OCT1_06	0.865625	1113	(-)	CWNAWTKWSATRYN	TATTTTAAAATTTG
M00138	V$OCT1_04	0.807612	1115	(-)	NNNNNNNWATGCAAATNNNNWNNW	TTTTAAAATTTGTAATATTTAAA
M00138	V$OCT1_04	0.816813	1116	(-)	NNNNNNNWATGCAAATNNNNWNNW	TTTAAAATTTGTAATATTTAAAT
M00162	V$OCT1_06	0.903516	1120	(+)	CWNAWTKWSATRYN	AAATTTGTAATATT
M00138	V$OCT1_04	0.7821	1121	(+)	NNNNNNNWATGCAAATNNNNWNNW	AATTTGTAATATTTAAATATTGA
M00162	V$OCT1_06	0.849609	1121	(-)	CWNAWTKWSATRYN	AATTTGTAATATTT
M00137	V$OCT1_03	0.855788	1123	(+)	NNNRTAATNANNN	TTTGTAATATTTA
M00138	V$OCT1_04	0.841489	1123	(+)	NNNNNNNWATGCAAATNNNNWNNW	TTTGTAATATTTAAATATTGATA
M00138	V$OCT1_04	0.868256	1124	(-)	NNNNNNNWATGCAAATNNNNWNNW	TTGTAATATTTAAATATTGATAC
M00135	V$OCT1_01	0.804273	1125	(+)	NNNNWTATGCAAATNTNNN	TGTAATATTTAAATATTGA
M00136	V$OCT1_02	0.817001	1125	(+)	NNGAATATKCANNNN	TGTAATATTTAAATA
M00135	V$OCT1_01	0.820679	1126	(-)	NNNNWTATGCAAATNTNNN	GTAATATTTAAATATTGAT
M00162	V$OCT1_06	0.9375	1128	(+)	CWNAWTKWSATRYN	AATATTTAAATATT
M00162	V$OCT1_06	0.9375	1128	(-)	CWNAWTKWSATRYN	AATATTTAAATATT
M00136	V$OCT1_02	0.857607	1130	(-)	NNGAATATKCANNNN	TATTTAAATATTGAT
M00138	V$OCT1_04	0.810749	1130	(-)	NNNNNNNWATGCAAATNNNNWNNW	TATTTAAATATTGATACTTCTCT
M00138	V$OCT1_04	0.783982	1131	(+)	NNNNNNNWATGCAAATNNNNWNNW	ATTTAAATATTGATACTTCTCTA
M00136	V$OCT1_02	0.772063	1133	(+)	NNGAATATKCANNNN	TTAAATATTGATACT
M00162	V$OCT1_06	0.807031	1183	(+)	CWNAWTKWSATRYN	CAGGATCAAATGCT
M00162	V$OCT1_06	0.842187	1184	(-)	CWNAWTKWSATRYN	AGGATCAAATGCTT
M00138	V$OCT1_04	0.79151	1193	(-)	NNNNNNNWATGCAAATNNNNWNNW	TGCTTTACTTTGAAGTGTAGTAA
M00162	V$OCT1_06	0.834766	1216	(+)	CWNAWTKWSATRYN	GATATTGGTATTTC
M00135	V$OCT1_01	0.772797	1242	(+)	NNNNWTATGCAAATNTNNN	AGCAGTAAGCAAAATAATT
M00138	V$OCT1_04	0.83187	1251	(-)	NNNNNNNWATGCAAATNNNNWNNW	CAAAATAATTTGAATGGTAAATA
M00162	V$OCT1_06	0.839844	1255	(+)	CWNAWTKWSATRYN	ATAATTTGAATGGT

M00195	V$OCT1_Q6	0.81905	1255	(-)	NNNNATGCAAATNAN	ATAATTTGAATGGTA
M00138	V$OCT1_04	0.806566	1256	(+)	NNNNNNNWATGCAAATNNNWNNW	TAATTTGAATGGTAAATATTTAT
M00248	V$OCT1_07	0.843969	1257	(-)	TNTATGNTAATT	AATTTGAATGGT
M00248	V$OCT1_07	0.843969	1261	(+)	TNTATGNTAATT	TGAATGGTAAAT
M00136	V$OCT1_02	0.815918	1264	(-)	NNGAATATKCANNNN	ATGGTAAATATTTAT
M00137	V$OCT1_03	0.860925	1264	(+)	NNNRTAATNANNN	ATGGTAAATATTT
M00138	V$OCT1_04	0.80343	1265	(+)	NNNNNNNWATGCAAATNNNWNNW	TGGTAAATATTTATATTGAAGAG
M00136	V$OCT1_02	0.826475	1267	(+)	NNGAATATKCANNNN	GTAAATATTTATATT
M00162	V$OCT1_06	0.801562	1283	(-)	CWNAWTKWSATRYN	AAGAGCAAAATTAG
M00162	V$OCT1_06	0.894141	1288	(+)	CWNAWTKWSATRYN	CAAAATTAGAAACT
M00138	V$OCT1_04	0.804057	1296	(+)	NNNNNNNWATGCAAATNNNWNNW	GAAACTAAATGAATAAAAATATT
M00138	V$OCT1_04	0.792765	1300	(+)	NNNNNNNWATGCAAATNNNWNNW	CTAAATGAATAAAAATATTACTT
M00136	V$OCT1_02	0.828912	1301	(-)	NNGAATATKCANNNN	TAAATGAATAAAAAT
M00138	V$OCT1_04	0.830615	1301	(-)	NNNNNNNWATGCAAATNNNWNNW	TAAATGAATAAAAATATTACTTT
M00137	V$OCT1_03	0.857369	1305	(+)	NNNRTAATNANNN	TGAATAAAAATAT
M00162	V$OCT1_06	0.860547	1305	(-)	CWNAWTKWSATRYN	TGAATAAAAATATT
M00136	V$OCT1_02	0.783974	1307	(-)	NNGAATATKCANNNN	AATAAAAATATTACT
M00248	V$OCT1_07	0.812381	1307	(-)	TNTATGNTAATT	AATAAAAATATT
M00162	V$OCT1_06	0.836719	1312	(+)	CWNAWTKWSATRYN	AAATATTACTTTCA
M00162	V$OCT1_06	0.826562	1316	(+)	CWNAWTKWSATRYN	ATTACTTTCAAATG
M00162	V$OCT1_06	0.808594	1324	(-)	CWNAWTKWSATRYN	CAAATGAAACAACT
M00135	V$OCT1_01	0.736742	1333	(-)	NNNNWTATGCAAATNTNNN	CAACTTTTATCATAATATA
M00162	V$OCT1_06	0.813281	1365	(-)	CWNAWTKWSATRYN	AAAATCAAGAATTT
M00136	V$OCT1_02	0.781267	1368	(-)	NNGAATATKCANNNN	ATCAAGAATTTTTTT
M00138	V$OCT1_04	0.857382	1372	(-)	NNNNNNNWATGCAAATNNNWNNW	AGAATTTTTTTCATGAATCTAA
M00162	V$OCT1_06	0.826953	1385	(+)	CWNAWTKWSATRYN	ATGAATCTAATTTT
M00162	V$OCT1_06	0.889062	1386	(-)	CWNAWTKWSATRYN	TGAATCTAATTTTA
M00162	V$OCT1_06	0.857812	1391	(+)	CWNAWTKWSATRYN	CTAATTTTATTATA
M00138	V$OCT1_04	0.789419	1392	(-)	NNNNNNNWATGCAAATNNNWNNW	TAATTTTATTATAAGACCTATTT
M00137	V$OCT1_03	0.908337	1394	(-)	NNNRTAATNANNN	ATTTTATTATAAG
M00248	V$OCT1_07	0.791817	1408	(+)	TNTATGNTAATT	CCTATTTTATTT
M00138	V$OCT1_04	0.797574	1413	(-)	NNNNNNNWATGCAAATNNNWNNW	TTTATTTTCTTACATAGATCTTG
M00137	V$OCT1_03	0.856183	1452	(-)	NNNRTAATNANNN	TCATCCTTACCTG

M00162	V$OCT1_06	0.85	1453	(+)	CWNAWTKWSATRYN	CATCCTTACCTGTC
M00138	V$OCT1_04	0.784818	1493	(+)	NNNNNNNWATGCAAATNNNWNNW	GTGAGTACAGTAAAAAATTTAGA
M00162	V$OCT1_06	0.821094	1498	(-)	CWNAWTKWSATRYN	TACAGTAAAAAATT
M00162	V$OCT1_06	0.809375	1501	(-)	CWNAWTKWSATRYN	AGTAAAAAATTTAG
M00138	V$OCT1_04	0.787118	1503	(-)	NNNNNNNWATGCAAATNNNWNNW	TAAAAAATTTAGAAGATTCTAGA
M00162	V$OCT1_06	0.800391	1516	(+)	CWNAWTKWSATRYN	AGATTCTAGATTCT
M00135	V$OCT1_01	0.750858	1542	(+)	NNNNWTATGCAAATNTNNN	TCTCAAATGCAATTTGATG
M00162	V$OCT1_06	0.868359	1549	(+)	CWNAWTKWSATRYN	TGCAATTTGATGCA
M00138	V$OCT1_04	0.807612	1550	(+)	NNNNNNNWATGCAAATNNNWNNW	GCAATTTGATGCAAGTCTCATCA
M00135	V$OCT1_01	0.755246	1552	(+)	NNNNWTATGCAAATNTNNN	AATTTGATGCAAGTCTCAT
M00137	V$OCT1_03	0.892137	1589	(+)	NNNRTAATNANNN	GTCGTAAAGAATT
M00162	V$OCT1_06	0.868359	1595	(+)	CWNAWTKWSATRYN	AAGAATTTGATGGT
M00162	V$OCT1_06	0.816406	1596	(-)	CWNAWTKWSATRYN	AGAATTTGATGGTC
M00162	V$OCT1_06	0.813672	1632	(-)	CWNAWTKWSATRYN	GATATTAAAGCTAT
M00162	V$OCT1_06	0.821484	1650	(+)	CWNAWTKWSATRYN	ATCCTTCACATTTT
M00195	V$OCT1_Q6	0.812773	1650	(-)	NNNNATGCAAATNAN	ATCCTTCACATTTTG
M00162	V$OCT1_06	0.817187	1651	(-)	CWNAWTKWSATRYN	TCCTTCACATTTTG
M00138	V$OCT1_04	0.791301	1652	(-)	NNNNNNNWATGCAAATNNNWNNW	CCTTCACATTTTGATCATTATTA
M00162	V$OCT1_06	0.809766	1656	(+)	CWNAWTKWSATRYN	CACATTTTGATCAT
M00138	V$OCT1_04	0.789419	1660	(+)	NNNNNNNWATGCAAATNNNWNNW	TTTTGATCATTATTATTTAGTTT
M00137	V$OCT1_03	0.888186	1663	(-)	NNNRTAATNANNN	TGATCATTATTAT
M00137	V$OCT1_03	0.898459	1666	(-)	NNNRTAATNANNN	TCATTATTATTTA
M00136	V$OCT1_02	0.781538	1668	(+)	NNGAATATKCANNNN	ATTATTATTTAGTTT
M00138	V$OCT1_04	0.792974	1675	(+)	NNNNNNNWATGCAAATNNNWNNW	TTTAGTTTATTCAAAGACTTAAC
M00135	V$OCT1_01	0.748951	1677	(+)	NNNNWTATGCAAATNTNNN	TAGTTTATTCAAAGACTTA
M00136	V$OCT1_02	0.806714	1677	(+)	NNGAATATKCANNNN	TAGTTTATTCAAAGA
M00195	V$OCT1_Q6	0.790393	1679	(+)	NNNNATGCAAATNAN	GTTTATTCAAAGACT
M00162	V$OCT1_06	0.860937	1699	(+)	CWNAWTKWSATRYN	CTAAATAAAATTTC
M00162	V$OCT1_06	0.847656	1700	(-)	CWNAWTKWSATRYN	TAAATAAAATTTCT
M00138	V$OCT1_04	0.795274	1708	(+)	NNNNNNNWATGCAAATNNNWNNW	ATTTCTAACTTAAAATATAAACA
M00138	V$OCT1_04	0.794647	1709	(-)	NNNNNNNWATGCAAATNNNWNNW	TTTCTAACTTAAAATATAAACAC
M00248	V$OCT1_07	0.831673	1715	(-)	TNTATGNTAATT	ACTTAAAATATA
M00137	V$OCT1_03	0.860134	1732	(+)	NNNRTAATNANNN	CTCACAATAAAAA

M00162	V$OCT1_06	0.808203	1734	(-)	CWNAWTKWSATRYN	CACAATAAAAAATT
M00138	V$OCT1_04	0.847135	1737	(+)	NNNNNNNWATGCAAATNNNWNNW	AATAAAAAATTAAAAATTAAAAA
M00138	V$OCT1_04	0.797156	1738	(+)	NNNNNNNWATGCAAATNNNWNNW	ATAAAAAATTAAAAATTAAAAAA
M00162	V$OCT1_06	0.855078	1741	(+)	CWNAWTKWSATRYN	AAAAATTAAAAATT
M00162	V$OCT1_06	0.808203	1741	(-)	CWNAWTKWSATRYN	AAAAATTAAAAATT
M00162	V$OCT1_06	0.834375	1742	(-)	CWNAWTKWSATRYN	AAAATTAAAAATTA
M00138	V$OCT1_04	0.830824	1744	(+)	NNNNNNNWATGCAAATNNNWNNW	AATTAAAAATTAAAAAAGAAATA
M00162	V$OCT1_06	0.826562	1748	(-)	CWNAWTKWSATRYN	AAAAATTAAAAAAG
M00248	V$OCT1_07	0.836549	1751	(-)	TNTATGNTAATT	AATTAAAAAAGA
M00135	V$OCT1_01	0.755628	1760	(+)	NNNNWTATGCAAATNTNNN	AGAAATAAGGTAATTAACA
M00248	V$OCT1_07	0.873861	1763	(+)	TNTATGNTAATT	AATAAGGTAATT
M00137	V$OCT1_03	0.844725	1766	(+)	NNNRTAATNANNN	AAGGTAATTAACA
M00248	V$OCT1_07	0.803265	1771	(-)	TNTATGNTAATT	AATTAACAATAC
M00162	V$OCT1_06	0.860547	1790	(-)	CWNAWTKWSATRYN	AGCATCAAAGAAGG
M00137	V$OCT1_03	0.870012	1800	(+)	NNNRTAATNANNN	AAGGTAATTAGTC
M00138	V$OCT1_04	0.818486	1829	(-)	NNNNNNNWATGCAAATNNNWNNW	TTATAGGCTTAACATATTTGTAA
M00135	V$OCT1_01	0.776994	1831	(-)	NNNNWTATGCAAATNTNNN	ATAGGCTTAACATATTTGT
M00161	V$OCT1_05	0.873651	1833	(+)	MKNATTTGCATAYY	AGGCTTAACATATT
M00162	V$OCT1_06	0.821484	1833	(+)	CWNAWTKWSATRYN	AGGCTTAACATATT
M00195	V$OCT1_Q6	0.826146	1833	(-)	NNNNATGCAAATNAN	AGGCTTAACATATTT
M00138	V$OCT1_04	0.795065	1837	(-)	NNNNNNNWATGCAAATNNNWNNW	TTAACATATTTGTAAACATATAT
M00138	V$OCT1_04	0.780845	1838	(+)	NNNNNNNWATGCAAATNNNWNNW	TAACATATTTGTAAACATATATA
M00135	V$OCT1_01	0.759443	1839	(-)	NNNNWTATGCAAATNTNNN	AACATATTTGTAAACATAT
M00138	V$OCT1_04	0.792137	1841	(-)	NNNNNNNWATGCAAATNNNWNNW	CATATTTGTAAACATATATATGT
M00137	V$OCT1_03	0.888977	1845	(+)	NNNRTAATNANNN	TTTGTAAACATAT
M00138	V$OCT1_04	0.821414	1847	(-)	NNNNNNNWATGCAAATNNNWNNW	TGTAAACATATATATGTTCAATT
M00135	V$OCT1_01	0.741129	1854	(+)	NNNNWTATGCAAATNTNNN	ATATATATGTTCAATTTTG
M00248	V$OCT1_07	0.792453	1857	(+)	TNTATGNTAATT	TATATGTTCAAT
M00162	V$OCT1_06	0.860547	1859	(-)	CWNAWTKWSATRYN	TATGTTCAATTTTG
M00138	V$OCT1_04	0.85299	1861	(-)	NNNNNNNWATGCAAATNNNWNNW	TGTTCAATTTTGTATTACCAAAT
M00135	V$OCT1_01	0.750286	1863	(-)	NNNNWTATGCAAATNTNNN	TTCAATTTTGTATTACCAA
M00162	V$OCT1_06	0.800391	1866	(+)	CWNAWTKWSATRYN	AATTTTGTATTACC
M00135	V$OCT1_01	0.73884	1869	(-)	NNNNWTATGCAAATNTNNN	TTTGTATTACCAAATATAC

M00248	V$OCT1_07	0.795209	1873	(-)	TNTATGNTAATT	TATTACCAAATA
M00162	V$OCT1_06	0.857031	1961	(+)	CWNAWTKWSATRYN	ATCCCTGGGATTCT
M00162	V$OCT1_06	0.819531	2017	(-)	CWNAWTKWSATRYN	TGCATGAAATCAAA
M00162	V$OCT1_06	0.903906	2022	(-)	CWNAWTKWSATRYN	GAAATCAAAAAGTG
M00162	V$OCT1_06	0.808984	2035	(-)	CWNAWTKWSATRYN	GAAATTGAAGTCTC
M00162	V$OCT1_06	0.821484	2073	(+)	CWNAWTKWSATRYN	CTCCATGGACTGCA
M00162	V$OCT1_06	0.877734	2104	(+)	CWNAWTKWSATRYN	ATCCATGGGATTTT
M00195	V$OCT1_Q6	0.795033	2162	(+)	NNNNATGCAAATNAN	AAATATACTATTAAG
M00162	V$OCT1_06	0.809766	2163	(-)	CWNAWTKWSATRYN	AATATACTATTAAG
M00162	V$OCT1_06	0.873437	2190	(-)	CWNAWTKWSATRYN	TGTATCCACTTTTG
M00162	V$OCT1_06	0.847656	2209	(-)	CWNAWTKWSATRYN	TAAATCAAACTCTC
M00162	V$OCT1_06	0.860937	2214	(+)	CWNAWTKWSATRYN	CAAACTCTCATTTG
M00162	V$OCT1_06	0.862891	2291	(-)	CWNAWTKWSATRYN	AAGATCTAATAATT
M00137	V$OCT1_03	0.854998	2293	(+)	NNNRTAATNANNN	GATCTAATAATTT
M00137	V$OCT1_03	0.886606	2296	(+)	NNNRTAATNANNN	CTAATAATTTGGT
M00138	V$OCT1_04	0.831242	2325	(+)	NNNNNNNWATGCAAATNNNWNNW	ACTAGATAATGTAAAGTAACTTA
M00137	V$OCT1_03	0.854208	2327	(+)	NNNRTAATNANNN	TAGATAATGTAAA
M00162	V$OCT1_06	0.855078	2329	(+)	CWNAWTKWSATRYN	GATAATGTAAAGTA
M00195	V$OCT1_Q6	0.836517	2329	(+)	NNNNATGCAAATNAN	GATAATGTAAAGTAA
M00248	V$OCT1_07	0.793089	2330	(+)	TNTATGNTAATT	ATAATGTAAAGT
M00162	V$OCT1_06	0.800781	2340	(+)	CWNAWTKWSATRYN	GTAACTTAAGTTTC
M00162	V$OCT1_06	0.852734	2360	(-)	CWNAWTKWSATRYN	AAAAACAAATTCAG
M00136	V$OCT1_02	0.786681	2362	(+)	NNGAATATKCANNNN	AAACAAATTCAGTTA
M00162	V$OCT1_06	0.809375	2365	(+)	CWNAWTKWSATRYN	CAAATTCAGTTATT
M00162	V$OCT1_06	0.808594	2365	(-)	CWNAWTKWSATRYN	CAAATTCAGTTATT
M00162	V$OCT1_06	0.821484	2371	(+)	CWNAWTKWSATRYN	CAGTTATTAATGTG
M00162	V$OCT1_06	0.852344	2376	(+)	CWNAWTKWSATRYN	ATTAATGTGAAACA
M00162	V$OCT1_06	0.800391	2383	(-)	CWNAWTKWSATRYN	TGAAACAAAAAGTT
M00162	V$OCT1_06	0.804297	2388	(+)	CWNAWTKWSATRYN	CAAAAAGTTATTCT
M00138	V$OCT1_04	0.842953	2416	(+)	NNNNNNNWATGCAAATNNNWNNW	TCTAGATAATGATAAATAAATAA
M00137	V$OCT1_03	0.930462	2418	(+)	NNNRTAATNANNN	TAGATAATGATAA
M00138	V$OCT1_04	0.794647	2418	(-)	NNNNNNNWATGCAAATNNNWNNW	TAGATAATGATAAATAAATAAAT
M00138	V$OCT1_04	0.79151	2419	(+)	NNNNNNNWATGCAAATNNNWNNW	AGATAATGATAAATAAATAAATA

M00137	V$OCT1_03	0.846306	2424	(+)	NNNRTAATNANNN	ATGATAAATAAAT
M00137	V$OCT1_03	0.855788	2432	(+)	NNNRTAATNANNN	TAAATAAATAGGA
M00195	V$OCT1_Q6	0.792849	2455	(+)	NNNNATGCAAATNAN	AAGAAAGCAATTCAA
M00138	V$OCT1_04	0.822668	2456	(+)	NNNNNNNWATGCAAATNNNWNNW	AGAAAGCAATTCAAATAAGATAA
M00195	V$OCT1_Q6	0.871452	2460	(+)	NNNNATGCAAATNAN	AGCAATTCAAATAAG
M00162	V$OCT1_06	0.832422	2461	(-)	CWNAWTKWSATRYN	GCAATTCAAATAAG
M00248	V$OCT1_07	0.792665	2463	(-)	TNTATGNTAATT	AATTCAAATAAG
M00138	V$OCT1_04	0.799247	2476	(+)	NNNNNNNWATGCAAATNNNWNNW	TAATAGTTTTGGATATTTGGACA
M00135	V$OCT1_01	0.795879	2477	(-)	NNNNWTATGCAAATNTNNN	AATAGTTTTGGATATTTGG
M00162	V$OCT1_06	0.834375	2479	(+)	CWNAWTKWSATRYN	TAGTTTTGGATATT
M00136	V$OCT1_02	0.791825	2481	(-)	NNGAATATKCANNNN	GTTTTGGATATTTGG
M00135	V$OCT1_01	0.786341	2485	(-)	NNNNWTATGCAAATNTNNN	TGGATATTTGGACACTCAA
M00162	V$OCT1_06	0.839453	2487	(+)	CWNAWTKWSATRYN	GATATTTGGACACT
M00162	V$OCT1_06	0.808594	2503	(+)	CWNAWTKWSATRYN	AGCTATCAAATATA
M00162	V$OCT1_06	0.852734	2504	(-)	CWNAWTKWSATRYN	GCTATCAAATATAG
M00162	V$OCT1_06	0.894141	2509	(+)	CWNAWTKWSATRYN	CAAATATAGATGCA
M00138	V$OCT1_04	0.840862	2510	(+)	NNNNNNNWATGCAAATNNNWNNW	AAATATAGATGCAAAAGTTTCTG
M00135	V$OCT1_01	0.796452	2512	(+)	NNNNWTATGCAAATNTNNN	ATATAGATGCAAAAGTTTC
M00162	V$OCT1_06	0.829297	2515	(-)	CWNAWTKWSATRYN	TAGATGCAAAAGTT
M00162	V$OCT1_06	0.898437	2526	(+)	CWNAWTKWSATRYN	GTTTCTGAAATGCT
M00162	V$OCT1_06	0.855078	2534	(+)	CWNAWTKWSATRYN	AATGCTGAGATATT
M00135	V$OCT1_01	0.738268	2539	(+)	NNNNWTATGCAAATNTNNN	TGAGATATTCTATTGTTAA
M00162	V$OCT1_06	0.821875	2542	(-)	CWNAWTKWSATRYN	GATATTCTATTGTT
M00162	V$OCT1_06	0.834375	2571	(-)	CWNAWTKWSATRYN	AAAGTGTAAGTAAT
M00162	V$OCT1_06	0.808203	2574	(+)	CWNAWTKWSATRYN	GTGTAAGTAATGTA
M00162	V$OCT1_06	0.867969	2575	(-)	CWNAWTKWSATRYN	TGTAAGTAATGTAG
M00195	V$OCT1_Q6	0.79012	2643	(+)	NNNNATGCAAATNAN	AAGCATCAAAAGAAG
M00162	V$OCT1_06	0.942578	2644	(-)	CWNAWTKWSATRYN	AGCATCAAAAGAAG
M00162	V$OCT1_06	0.808594	2652	(+)	CWNAWTKWSATRYN	AAGAAGGGAATATC
M00162	V$OCT1_06	0.865625	2662	(-)	CWNAWTKWSATRYN	TATCTGACAGTAAG
M00162	V$OCT1_06	0.821484	2775	(+)	CWNAWTKWSATRYN	GATTCTTTAATCCT
M00138	V$OCT1_04	0.80092	2801	(-)	NNNNNNNWATGCAAATNNNWNNW	ACATCATTTTTTAATGCTAACAT
M00162	V$OCT1_06	0.9375	2805	(+)	CWNAWTKWSATRYN	CATTTTTTAATGCT

M00135	V$OCT1_01	0.746852	2808	(+)	NNNNWTATGCAAATNTNNN	TTTTTAATGCTAACATTTA
M00162	V$OCT1_06	0.842187	2819	(-)	CWNAWTKWSATRYN	AACATTTAACAAAC
M00138	V$OCT1_04	0.802802	2820	(-)	NNNNNNNWATGCAAATNNNWNNW	ACATTTAACAAACATAAATCTTG
M00248	V$OCT1_07	0.797329	2822	(-)	TNTATGNTAATT	ATTTAACAAACA
M00248	V$OCT1_07	0.792453	2826	(-)	TNTATGNTAATT	AACAAACATAAA
M00138	V$OCT1_04	0.815349	2849	(+)	NNNNNNNWATGCAAATNNNWNNW	CAGTTAAAATGCAGATTGAGTTA
M00135	V$OCT1_01	0.758298	2851	(+)	NNNNWTATGCAAATNTNNN	GTTAAAATGCAGATTGAGT
M00136	V$OCT1_02	0.842989	2851	(+)	NNGAATATKCANNNN	GTTAAAATGCAGATT
M00136	V$OCT1_02	0.802653	2854	(-)	NNGAATATKCANNNN	AAAATGCAGATTGAG
M00248	V$OCT1_07	0.799873	2854	(+)	TNTATGNTAATT	AAAATGCAGATT
M00138	V$OCT1_04	0.790882	2859	(+)	NNNNNNNWATGCAAATNNNWNNW	GCAGATTGAGTTAAAATTTTATA
M00162	V$OCT1_06	0.826562	2865	(-)	CWNAWTKWSATRYN	TGAGTTAAAATTTT
M00138	V$OCT1_04	0.797365	2868	(-)	NNNNNNNWATGCAAATNNNWNNW	GTTAAAATTTTATATAATTTAAT
M00135	V$OCT1_01	0.754865	2870	(-)	NNNNWTATGCAAATNTNNN	TAAAATTTTATATAATTTA
M00162	V$OCT1_06	0.825	2870	(+)	CWNAWTKWSATRYN	TAAAATTTTATATA
M00162	V$OCT1_06	0.868359	2880	(+)	CWNAWTKWSATRYN	TATAATTTAATTTA
M00248	V$OCT1_07	0.831673	2880	(+)	TNTATGNTAATT	TATAATTTAATT
M00138	V$OCT1_04	0.787118	2881	(+)	NNNNNNNWATGCAAATNNNWNNW	ATAATTTAATTTATGATAAAAAA
M00162	V$OCT1_06	0.847656	2881	(-)	CWNAWTKWSATRYN	ATAATTTAATTTAT
M00138	V$OCT1_04	0.781054	2883	(-)	NNNNNNNWATGCAAATNNNWNNW	AATTTAATTTATGATAAAAAATA
M00138	V$OCT1_04	0.847762	2885	(+)	NNNNNNNWATGCAAATNNNWNNW	TTTAATTTATGATAAAAAATAAA
M00136	V$OCT1_02	0.776665	2902	(+)	NNGAATATKCANNNN	AATAAAATCCAGACA
M00136	V$OCT1_02	0.775853	2917	(+)	NNGAATATKCANNNN	AACAATATTTCAGAT
M00162	V$OCT1_06	0.867969	2919	(+)	CWNAWTKWSATRYN	CAATATTTCAGATT
M00162	V$OCT1_06	0.842187	2920	(-)	CWNAWTKWSATRYN	AATATTTCAGATTA
M00137	V$OCT1_03	0.845516	2925	(-)	NNNRTAATNANNN	TTCAGATTATTTT
M00138	V$OCT1_04	0.800293	2935	(-)	NNNNNNNWATGCAAATNNNWNNW	TTTTTGTCTTTTTATATTTTCTC
M00162	V$OCT1_06	0.834375	2978	(+)	CWNAWTKWSATRYN	GATAATTTACTTTT
M00162	V$OCT1_06	0.834375	2979	(+)	CWNAWTKWSATRYN	ATAATTTACTTTTC
M00195	V$OCT1_Q6	0.840338	2979	(-)	NNNNATGCAAATNAN	ATAATTTACTTTTCT
M00138	V$OCT1_04	0.7844	2993	(+)	NNNNNNNWATGCAAATNNNWNNW	TTTTATTTTTGTCAATAAATTTT
M00162	V$OCT1_06	0.865625	3038	(-)	CWNAWTKWSATRYN	GGTGTCAAATTTAG
M00162	V$OCT1_06	0.817187	3043	(+)	CWNAWTKWSATRYN	CAAATTTAGCTCTT

M00162	V$OCT1_06	0.864844	3050	(+)	CWNAWTKWSATRYN	AGCTCTTAAATACA
M00162	V$OCT1_06	0.803906	3054	(+)	CWNAWTKWSATRYN	CTTAAATACAAACT
M00162	V$OCT1_06	0.940234	3078	(+)	CWNAWTKWSATRYN	CACTATTAAATGTA
M00138	V$OCT1_04	0.849435	3079	(+)	NNNNNNNWATGCAAATNNNWNNW	ACTATTAAATGTATAATATTACA
M00162	V$OCT1_06	0.826562	3079	(-)	CWNAWTKWSATRYN	ACTATTAAATGTAT
M00136	V$OCT1_02	0.788847	3084	(-)	NNGAATATKCANNNN	TAAATGTATAATATT
M00162	V$OCT1_06	0.821094	3084	(-)	CWNAWTKWSATRYN	TAAATGTATAATAT
M00137	V$OCT1_03	0.857369	3091	(-)	NNNRTAATNANNN	ATAATATTACATG
M00162	V$OCT1_06	0.878516	3092	(+)	CWNAWTKWSATRYN	TAATATTACATGTG
M00162	V$OCT1_06	0.834375	3093	(-)	CWNAWTKWSATRYN	AATATTACATGTGC
M00162	V$OCT1_06	0.826562	3104	(+)	CWNAWTKWSATRYN	TGCCTTGTGATATT
M00138	V$OCT1_04	0.804893	3108	(-)	NNNNNNNWATGCAAATNNNWNNW	TTGTGATATTATTATTATTGTAT
M00138	V$OCT1_04	0.791719	3111	(-)	NNNNNNNWATGCAAATNNNWNNW	TGATATTATTATTATTGTATTTT
M00137	V$OCT1_03	0.906361	3113	(-)	NNNRTAATNANNN	ATATTATTATTAT
M00138	V$OCT1_04	0.791092	3113	(+)	NNNNNNNWATGCAAATNNNWNNW	ATATTATTATTATTGTATTTTGA
M00137	V$OCT1_03	0.873568	3116	(-)	NNNRTAATNANNN	TTATTATTATTGT
M00162	V$OCT1_06	0.805859	3121	(+)	CWNAWTKWSATRYN	ATTATTGTATTTTG
M00248	V$OCT1_07	0.815137	3121	(+)	TNTATGNTAATT	ATTATTGTATTT
M00138	V$OCT1_04	0.805521	3123	(-)	NNNNNNNWATGCAAATNNNWNNW	TATTGTATTTTGAGTGCTTTTGT
M00162	V$OCT1_06	0.800391	3127	(+)	CWNAWTKWSATRYN	GTATTTTGAGTGCT
M00138	V$OCT1_04	0.80092	3148	(-)	NNNNNNNWATGCAAATNNNWNNW	TTACAGTTCTTGCATTTTTTTTT
M00135	V$OCT1_01	0.746089	3150	(-)	NNNNWTATGCAAATNTNNN	ACAGTTCTTGCATTTTTTT
M00162	V$OCT1_06	0.864844	3152	(+)	CWNAWTKWSATRYN	AGTTCTTGCATTTT
M00136	V$OCT1_02	0.850568	3154	(-)	NNGAATATKCANNNN	TTCTTGCATTTTTTT
M00138	V$OCT1_04	0.814304	3162	(-)	NNNNNNNWATGCAAATNNNWNNW	TTTTTTTTTTAACAGAAACATCC
M00162	V$OCT1_06	0.804297	3178	(-)	CWNAWTKWSATRYN	AACATCCTATCAAG
M00138	V$OCT1_04	0.824969	3265	(+)	NNNNNNNWATGCAAATNNNWNNW	ATGATAAATTGCTAATATATAGA
M00135	V$OCT1_01	0.738459	3267	(+)	NNNNWTATGCAAATNTNNN	GATAAATTGCTAATATATA
M00137	V$OCT1_03	0.840379	3341	(+)	NNNRTAATNANNN	GGTATAATATCTT
M00136	V$OCT1_02	0.775853	3362	(-)	NNGAATATKCANNNN	GAGCTGACTATTGAT
M00195	V$OCT1_Q6	0.80131	3406	(-)	NNNNATGCAAATNAN	GGCATTTGGACTTAC
M00138	V$OCT1_04	0.790255	3408	(-)	NNNNNNNWATGCAAATNNNWNNW	CATTTGGACTTACGTATTTATGA
M00137	V$OCT1_03	0.877914	3419	(-)	NNNRTAATNANNN	ACGTATTTATGAT

M00138	V$OCT1_04	0.842325	3419	(+)	NNNNNNNWATGCAAATNNNWNNW	ACGTATTTATGATTAATAAAAAT
M00138	V$OCT1_04	0.785236	3422	(+)	NNNNNNNWATGCAAATNNNWNNW	TATTTATGATTAATAAAAATTTT
M00137	V$OCT1_03	0.870802	3425	(-)	NNNRTAATNANNN	TTATGATTAATAA
M00138	V$OCT1_04	0.82936	3426	(+)	NNNNNNNWATGCAAATNNNWNNW	TATGATTAATAAAAATTTTATTA
M00138	V$OCT1_04	0.819741	3427	(-)	NNNNNNNWATGCAAATNNNWNNW	ATGATTAATAAAAATTTTATTAT
M00137	V$OCT1_03	0.851047	3431	(+)	NNNRTAATNANNN	TTAATAAAAATTT
M00162	V$OCT1_06	0.826562	3437	(+)	CWNAWTKWSATRYN	AAAATTTTATTATG
M00162	V$OCT1_06	0.881641	3437	(-)	CWNAWTKWSATRYN	AAAATTTTATTATG
M00138	V$OCT1_04	0.800293	3439	(-)	NNNNNNNWATGCAAATNNNWNNW	AATTTTATTATGCAAGAGCAATA
M00137	V$OCT1_03	0.92098	3440	(-)	NNNRTAATNANNN	ATTTTATTATGCA
M00138	V$OCT1_04	0.806775	3440	(+)	NNNNNNNWATGCAAATNNNWNNW	ATTTTATTATGCAAGAGCAATAG
M00135	V$OCT1_01	0.792827	3442	(+)	NNNNWTATGCAAATNTNNN	TTTATTATGCAAGAGCAAT
M00136	V$OCT1_02	0.838116	3442	(+)	NNGAATATKCANNNN	TTTATTATGCAAGAG
M00162	V$OCT1_06	0.834375	3457	(+)	CWNAWTKWSATRYN	CAATAGTTAATTCA
M00162	V$OCT1_06	0.806641	3469	(+)	CWNAWTKWSATRYN	CAATAGGATATGTA
M00138	V$OCT1_04	0.806775	3470	(+)	NNNNNNNWATGCAAATNNNWNNW	AATAGGATATGTATATGTGGTCT
M00135	V$OCT1_01	0.734071	3471	(-)	NNNNWTATGCAAATNTNNN	ATAGGATATGTATATGTGG
M00135	V$OCT1_01	0.78367	3472	(+)	NNNNWTATGCAAATNTNNN	TAGGATATGTATATGTGGT
M00136	V$OCT1_02	0.789659	3472	(+)	NNGAATATKCANNNN	TAGGATATGTATATG
M00162	V$OCT1_06	0.868359	3475	(-)	CWNAWTKWSATRYN	GATATGTATATGTG
M00135	V$OCT1_01	0.742083	3488	(-)	NNNNWTATGCAAATNTNNN	GGTCTATTTTCATGTATTA
M00138	V$OCT1_04	0.792974	3490	(-)	NNNNNNNWATGCAAATNNNWNNW	TCTATTTTCATGTATTATTCATT
M00162	V$OCT1_06	0.825	3490	(+)	CWNAWTKWSATRYN	TCTATTTTCATGTA
M00195	V$OCT1_Q6	0.813592	3490	(-)	NNNNATGCAAATNAN	TCTATTTTCATGTAT
M00138	V$OCT1_04	0.839398	3497	(-)	NNNNNNNWATGCAAATNNNWNNW	TCATGTATTATTCATTCTTTCTC
M00136	V$OCT1_02	0.844071	3500	(+)	NNGAATATKCANNNN	TGTATTATTCATTCT
M00162	V$OCT1_06	0.813281	3501	(+)	CWNAWTKWSATRYN	GTATTATTCATTCT
M00162	V$OCT1_06	0.834375	3534	(+)	CWNAWTKWSATRYN	AAACATATGATTTA
M00162	V$OCT1_06	0.812109	3535	(-)	CWNAWTKWSATRYN	AACATATGATTTAC
M00195	V$OCT1_Q6	0.831332	3540	(-)	NNNNATGCAAATNAN	ATGATTTACAACTTG
M00135	V$OCT1_01	0.785006	3556	(-)	NNNNWTATGCAAATNTNNN	TCTCAATTTACACACTGAG
M00248	V$OCT1_07	0.852025	3560	(-)	TNTATGNTAATT	AATTTACACACT
M00136	V$OCT1_02	0.785869	3567	(-)	NNGAATATKCANNNN	ACACTGAGTATTAAA

M00136	V$OCT1_02	0.774228	3570	(+)	NNGAATATKCANNNN	CTGAGTATTAAATTA
M00162	V$OCT1_06	0.803516	3572	(+)	CWNAWTKWSATRYN	GAGTATTAAATTAG
M00162	V$OCT1_06	0.804297	3573	(-)	CWNAWTKWSATRYN	AGTATTAAATTAGA
M00162	V$OCT1_06	0.894141	3577	(+)	CWNAWTKWSATRYN	TTAAATTAGATACT
M00162	V$OCT1_06	0.808594	3578	(-)	CWNAWTKWSATRYN	TAAATTAGATACTC
M00162	V$OCT1_06	0.847656	3646	(+)	CWNAWTKWSATRYN	CATTTTTTAATCTG
M00137	V$OCT1_03	0.854208	3694	(-)	NNNRTAATNANNN	CTTTCTTTACCCC
M00138	V$OCT1_04	0.780636	3723	(+)	NNNNNNNWATGCAAATNNNNWNNW	AGTTAGCTATGATAAATACTGAA
M00248	V$OCT1_07	0.790545	3728	(+)	TNTATGNTAATT	GCTATGATAAAT
M00162	V$OCT1_06	0.803125	3736	(+)	CWNAWTKWSATRYN	AAATACTGAATGTC
M00162	V$OCT1_06	0.804297	3741	(+)	CWNAWTKWSATRYN	CTGAATGTCTTCTT
M00138	V$OCT1_04	0.84609	3749	(-)	NNNNNNNWATGCAAATNNNNWNNW	CTTCTTCATTTCCATTTTCATTT
M00135	V$OCT1_01	0.787867	3751	(-)	NNNNWTATGCAAATNTNNN	TCTTCATTTCCATTTTCAT
M00161	V$OCT1_05	0.878632	3753	(+)	MKNATTTGCATAYY	TTCATTTCCATTTT
M00195	V$OCT1_Q6	0.845251	3753	(-)	NNNNATGCAAATNAN	TTCATTTCCATTTTC
M00136	V$OCT1_02	0.772875	3755	(-)	NNGAATATKCANNNN	CATTTCCATTTTCAT
M00138	V$OCT1_04	0.841489	3755	(-)	NNNNNNNWATGCAAATNNNNWNNW	CATTTCCATTTTCATTTGTATAC
M00248	V$OCT1_07	0.795845	3755	(-)	TNTATGNTAATT	CATTTCCATTTT
M00135	V$OCT1_01	0.763068	3757	(-)	NNNNWTATGCAAATNTNNN	TTTCCATTTTCATTTGTAT
M00138	V$OCT1_04	0.828315	3761	(-)	NNNNNNNWATGCAAATNNNNWNNW	CATTTTCATTTGTATACATTCGT
M00135	V$OCT1_01	0.769935	3763	(-)	NNNNWTATGCAAATNTNNN	TTTTCATTTGTATACATTC
M00195	V$OCT1_Q6	0.8619	3765	(-)	NNNNATGCAAATNAN	TTCATTTGTATACAT
M00162	V$OCT1_06	0.805859	3790	(-)	CWNAWTKWSATRYN	TGTATCCAGGTACT
M00137	V$OCT1_03	0.876333	3809	(-)	NNNRTAATNANNN	AGTTGATTACCCT
M00137	V$OCT1_03	0.897669	3834	(-)	NNNRTAATNANNN	TTTTCATTACATT
M00162	V$OCT1_06	0.85	3835	(+)	CWNAWTKWSATRYN	TTTCATTACATTCC
M00162	V$OCT1_06	0.834766	3836	(-)	CWNAWTKWSATRYN	TTCATTACATTCCT
M00137	V$OCT1_03	0.846701	3871	(+)	NNNRTAATNANNN	TTGATAAGGAAGA
M00137	V$OCT1_03	0.862505	3903	(+)	NNNRTAATNANNN	CTGATAAAGAAGG
M00162	V$OCT1_06	0.839844	3931	(+)	CWNAWTKWSATRYN	CTCCTTCAGATTTT
M00162	V$OCT1_06	0.852734	3937	(+)	CWNAWTKWSATRYN	CAGATTTTAAAATA
M00162	V$OCT1_06	0.805859	3944	(-)	CWNAWTKWSATRYN	TAAAATAAAATCAT
M00162	V$OCT1_06	0.805859	3950	(-)	CWNAWTKWSATRYN	AAAATCATAAAACT

M00162	V$OCT1_06	0.855078	3963	(-)	CWNAWTKWSATRYN	TGAAACTAATTATC
M00137	V$OCT1_03	0.912288	3966	(-)	NNNRTAATNANNN	AACTAATTATCTC
M00138	V$OCT1_04	0.845462	3975	(-)	NNNNNNNWATGCAAATNNNWNNW	TCTCTTATTTTACCTAAGAAATA
M00162	V$OCT1_06	0.839453	3994	(+)	CWNAWTKWSATRYN	AATATTTTGGTTTA
M00138	V$OCT1_04	0.811167	3996	(-)	NNNNNNNWATGCAAATNNNWNNW	TATTTTGGTTTACCTAAATAAAT
M00138	V$OCT1_04	0.802802	3999	(+)	NNNNNNNWATGCAAATNNNWNNW	TTTGGTTTACCTAAATAAATGGA
M00138	V$OCT1_04	0.780427	4009	(+)	NNNNNNNWATGCAAATNNNWNNW	CTAAATAAATGGAGAATTTGTGT
M00138	V$OCT1_04	0.813676	4022	(+)	NNNNNNNWATGCAAATNNNWNNW	GAATTTGTGTTCAAATGGAAAAA
M00138	V$OCT1_04	0.828315	4028	(+)	NNNNNNNWATGCAAATNNNWNNW	GTGTTCAAATGGAAAAACATTCT
M00138	V$OCT1_04	0.785027	4048	(-)	NNNNNNNWATGCAAATNNNWNNW	TCTCCTTTTTTGACTGTGTTTTT
M00162	V$OCT1_06	0.813672	4052	(+)	CWNAWTKWSATRYN	CTTTTTTGACTGTG
M00162	V$OCT1_06	0.800391	4063	(+)	CWNAWTKWSATRYN	GTGTTTTTCACTTT
M00195	V$OCT1_Q6	0.801856	4063	(-)	NNNNATGCAAATNAN	GTGTTTTTCACTTTT
M00162	V$OCT1_06	0.829297	4071	(+)	CWNAWTKWSATRYN	CACTTTTACAATTC
M00162	V$OCT1_06	0.925	4084	(+)	CWNAWTKWSATRYN	CACAATTTAATTCC
M00162	V$OCT1_06	0.821875	4085	(-)	CWNAWTKWSATRYN	ACAATTTAATTCCT
M00138	V$OCT1_04	0.792974	4105	(+)	NNNNNNNWATGCAAATNNNWNNW	AGTCCTCAATGAAAATTTACTCA
M00135	V$OCT1_01	0.736742	4107	(+)	NNNNWTATGCAAATNTNNN	TCCTCAATGAAAATTTACT
M00162	V$OCT1_06	0.847656	4109	(+)	CWNAWTKWSATRYN	CTCAATGAAAATTT
M00136	V$OCT1_02	0.779643	4110	(-)	NNGAATATKCANNNN	TCAATGAAAATTTAC
M00162	V$OCT1_06	0.804297	4110	(-)	CWNAWTKWSATRYN	TCAATGAAAATTTA
M00248	V$OCT1_07	0.843545	4110	(+)	TNTATGNTAATT	TCAATGAAAATT
M00136	V$OCT1_02	0.815918	4113	(+)	NNGAATATKCANNNN	ATGAAAATTTACTCA
M00162	V$OCT1_06	0.85	4171	(-)	CWNAWTKWSATRYN	CAAATGTATTTTTC
M00138	V$OCT1_04	0.813049	4176	(+)	NNNNNNNWATGCAAATNNNWNNW	GTATTTTTCTGGAAAAATCAACT
M00135	V$OCT1_01	0.735025	4178	(+)	NNNNWTATGCAAATNTNNN	ATTTTTCTGGAAAAATCAA
M00162	V$OCT1_06	0.839453	4189	(-)	CWNAWTKWSATRYN	AAAATCAACTTAAT
M00162	V$OCT1_06	0.813672	4192	(+)	CWNAWTKWSATRYN	ATCAACTTAATTTT
M00138	V$OCT1_04	0.806566	4193	(+)	NNNNNNNWATGCAAATNNNWNNW	TCAACTTAATTTTTATTTATAAA
M00138	V$OCT1_04	0.802175	4194	(-)	NNNNNNNWATGCAAATNNNWNNW	CAACTTAATTTTTATTTATAAAT
M00138	V$OCT1_04	0.831451	4198	(-)	NNNNNNNWATGCAAATNNNWNNW	TTAATTTTTATTTATAAATATTT
M00195	V$OCT1_Q6	0.820415	4198	(-)	NNNNATGCAAATNAN	TTAATTTTTATTTAT
M00138	V$OCT1_04	0.836052	4199	(+)	NNNNNNNWATGCAAATNNNWNNW	TAATTTTTATTTATAAATATTTT

M00138	V$OCT1_04	0.786909	4200	(-)	NNNNNNNWATGCAAATNNNWNNW	AATTTTTATTTATAAATATTTTT
M00138	V$OCT1_04	0.857591	4202	(-)	NNNNNNNWATGCAAATNNNWNNW	TTTTTATTTATAAATATTTTTTC
M00137	V$OCT1_03	0.845516	4203	(-)	NNNRTAATNANNN	TTTTATTTATAAA
M00138	V$OCT1_04	0.851945	4203	(+)	NNNNNNNWATGCAAATNNNWNNW	TTTTATTTATAAATATTTTTTCA
M00138	V$OCT1_04	0.801547	4204	(-)	NNNNNNNWATGCAAATNNNWNNW	TTTATTTATAAATATTTTTTCAC
M00136	V$OCT1_02	0.833243	4208	(-)	NNGAATATKCANNNN	TTTATAAATATTTTT
M00137	V$OCT1_03	0.859344	4208	(+)	NNNRTAATNANNN	TTTATAAATATTT
M00136	V$OCT1_02	0.780996	4211	(+)	NNGAATATKCANNNN	ATAAATATTTTTTCA
M00138	V$OCT1_04	0.791092	4233	(-)	NNNNNNNWATGCAAATNNNWNNW	AAATTTTTTCTTCATTTGCTCAC
M00136	V$OCT1_02	0.83189	4266	(-)	NNGAATATKCANNNN	ACAAAGAATATTGAA
M00138	V$OCT1_04	0.836888	4267	(+)	NNNNNNNWATGCAAATNNNWNNW	CAAAGAATATTGAAATTATATAT
M00135	V$OCT1_01	0.767264	4269	(+)	NNNNWTATGCAAATNTNNN	AAGAATATTGAAATTATAT
M00136	V$OCT1_02	0.856524	4269	(+)	NNGAATATKCANNNN	AAGAATATTGAAATT
M00195	V$OCT1_Q6	0.814956	4271	(+)	NNNNATGCAAATNAN	GAATATTGAAATTAT
M00161	V$OCT1_05	0.897393	4272	(-)	MKNATTTGCATAYY	AATATTGAAATTAT
M00162	V$OCT1_06	0.847656	4272	(+)	CWNAWTKWSATRYN	AATATTGAAATTAT
M00162	V$OCT1_06	0.852734	4272	(-)	CWNAWTKWSATRYN	AATATTGAAATTAT
M00248	V$OCT1_07	0.889124	4272	(+)	TNTATGNTAATT	AATATTGAAATT
M00138	V$OCT1_04	0.817022	4279	(-)	NNNNNNNWATGCAAATNNNWNNW	AAATTATATATGCAAATATTAAA
M00138	V$OCT1_04	0.928691	4280	(+)	NNNNNNNWATGCAAATNNNWNNW	AATTATATATGCAAATATTAAAA
M00135	V$OCT1_01	0.7562	4281	(-)	NNNNWTATGCAAATNTNNN	ATTATATATGCAAATATTA
M00135	V$OCT1_01	0.953453	4282	(+)	NNNNWTATGCAAATNTNNN	TTATATATGCAAATATTAA
M00136	V$OCT1_02	0.837845	4282	(+)	NNGAATATKCANNNN	TTATATATGCAAATA
M00195	V$OCT1_Q6	0.889465	4284	(+)	NNNNATGCAAATNAN	ATATATGCAAATATT
M00162	V$OCT1_06	0.9375	4285	(-)	CWNAWTKWSATRYN	TATATGCAAATATT
M00248	V$OCT1_07	0.873225	4285	(+)	TNTATGNTAATT	TATATGCAAATA
M00136	V$OCT1_02	0.775853	4287	(-)	NNGAATATKCANNNN	TATGCAAATATTAAA
M00138	V$OCT1_04	0.786282	4288	(+)	NNNNNNNWATGCAAATNNNWNNW	ATGCAAATATTAAAAAGCTGCTT
M00162	V$OCT1_06	0.869922	4293	(-)	CWNAWTKWSATRYN	AATATTAAAAAGCT
M00162	V$OCT1_06	0.916797	4305	(+)	CWNAWTKWSATRYN	CTGCTTTAAATATT
M00195	V$OCT1_Q6	0.807314	4305	(-)	NNNNATGCAAATNAN	CTGCTTTAAATATTA
M00136	V$OCT1_02	0.822144	4307	(-)	NNGAATATKCANNNN	GCTTTAAATATTAAT
M00138	V$OCT1_04	0.781054	4307	(-)	NNNNNNNWATGCAAATNNNWNNW	GCTTTAAATATTAATCTGTACCT

M00162	V$OCT1_06	0.804687	4311	(-)	CWNAWTKWSATRYN	TAAATATTAATCTG
M00138	V$OCT1_04	0.816395	4327	(-)	NNNNNNNWATGCAAATNNNWNNW	CCTATACATTTGACTCTTTGTAA
M00162	V$OCT1_06	0.813281	4330	(+)	CWNAWTKWSATRYN	ATACATTTGACTCT
M00162	V$OCT1_06	0.81875	4340	(+)	CWNAWTKWSATRYN	CTCTTTGTAAAACA
M00138	V$OCT1_04	0.803639	4359	(+)	NNNNNNNWATGCAAATNNNWNNW	GCTGTCTTGTGCATAATCTATGT
M00135	V$OCT1_01	0.777375	4360	(-)	NNNNWTATGCAAATNTNNN	CTGTCTTGTGCATAATCTA
M00136	V$OCT1_02	0.865999	4364	(-)	NNGAATATKCANNNN	CTTGTGCATAATCTA
M00138	V$OCT1_04	0.859473	4370	(+)	NNNNNNNWATGCAAATNNNWNNW	CATAATCTATGTAAATTTTATAA
M00135	V$OCT1_01	0.831934	4372	(+)	NNNNWTATGCAAATNTNNN	TAATCTATGTAAATTTTAT
M00195	V$OCT1_Q6	0.839793	4374	(+)	NNNNATGCAAATNAN	ATCTATGTAAATTTT
M00162	V$OCT1_06	0.88125	4375	(-)	CWNAWTKWSATRYN	TCTATGTAAATTTT
M00248	V$OCT1_07	0.96926	4375	(+)	TNTATGNTAATT	TCTATGTAAATT
M00138	V$OCT1_04	0.818904	4379	(-)	NNNNNNNWATGCAAATNNNWNNW	TGTAAATTTTATAATTTCCATTC
M00162	V$OCT1_06	0.829297	4381	(-)	CWNAWTKWSATRYN	TAAATTTTATAATT
M00162	V$OCT1_06	0.800781	4383	(+)	CWNAWTKWSATRYN	AATTTTATAATTTC
M00138	V$OCT1_04	0.901715	4385	(-)	NNNNNNNWATGCAAATNNNWNNW	TTTTATAATTTCCATTCTCATTA
M00135	V$OCT1_01	0.789012	4387	(-)	NNNNWTATGCAAATNTNNN	TTATAATTTCCATTCTCAT
M00161	V$OCT1_05	0.936909	4389	(+)	MKNATTTGCATAYY	ATAATTTCCATTCT
M00162	V$OCT1_06	0.860547	4389	(+)	CWNAWTKWSATRYN	ATAATTTCCATTCT
M00195	V$OCT1_Q6	0.883188	4389	(-)	NNNNATGCAAATNAN	ATAATTTCCATTCTC
M00162	V$OCT1_06	0.808594	4390	(-)	CWNAWTKWSATRYN	TAATTTCCATTCTC
M00248	V$OCT1_07	0.898664	4391	(-)	TNTATGNTAATT	AATTTCCATTCT
M00137	V$OCT1_03	0.962465	4399	(-)	NNNRTAATNANNN	TTCTCATTATGAT
M00162	V$OCT1_06	0.801953	4401	(+)	CWNAWTKWSATRYN	CTCATTATGATGGA
M00136	V$OCT1_02	0.814835	4408	(-)	NNGAATATKCANNNN	TGATGGAATGTTCTC
M00136	V$OCT1_02	0.817813	4430	(+)	NNGAATATKCANNNN	TACAATATTCTGGAT
M00162	V$OCT1_06	0.800391	4434	(+)	CWNAWTKWSATRYN	ATATTCTGGATGTG
M00137	V$OCT1_03	0.843935	4456	(+)	NNNRTAATNANNN	TCTGTAATTTCAT
M00162	V$OCT1_06	0.808594	4467	(+)	CWNAWTKWSATRYN	ATGACTGAATTGTC
M00136	V$OCT1_02	0.789388	4468	(-)	NNGAATATKCANNNN	TGACTGAATTGTCAA
M00162	V$OCT1_06	0.821484	4473	(-)	CWNAWTKWSATRYN	GAATTGTCAAACAG
M00138	V$OCT1_04	0.806985	4486	(+)	NNNNNNNWATGCAAATNNNWNNW	GAACACAAATAGAAATTTTATTT
M00135	V$OCT1_01	0.7314	4488	(+)	NNNNWTATGCAAATNTNNN	ACACAAATAGAAATTTTAT

M00162	V$OCT1_06	0.860547	4491	(+)	CWNAWTKWSATRYN	CAAATAGAAATTTT
M00162	V$OCT1_06	0.834766	4496	(+)	CWNAWTKWSATRYN	AGAAATTTTATTTC
M00162	V$OCT1_06	0.826562	4497	(+)	CWNAWTKWSATRYN	GAAATTTTATTTCA
M00162	V$OCT1_06	0.813672	4502	(-)	CWNAWTKWSATRYN	TTTATTTCAAGACC
M00162	V$OCT1_06	0.890625	4516	(-)	CWNAWTKWSATRYN	AGTATTTAAAAGAT
M00138	V$OCT1_04	0.836888	4553	(-)	NNNNNNNWATGCAAATNNNWNNW	TGAATGATTTTTAATATAATCTT
M00162	V$OCT1_06	0.826562	4557	(+)	CWNAWTKWSATRYN	TGATTTTTAATATA
M00162	V$OCT1_06	0.868359	4611	(-)	CWNAWTKWSATRYN	GGTAAGAAATTCTG
M00162	V$OCT1_06	0.853125	4622	(+)	CWNAWTKWSATRYN	CTGAATAGAATATA
M00136	V$OCT1_02	0.812128	4624	(-)	NNGAATATKCANNNN	GAATAGAATATACTG
M00138	V$OCT1_04	0.797574	4628	(+)	NNNNNNNWATGCAAATNNNWNNW	AGAATATACTGCAGAATTAACAA
M00248	V$OCT1_07	0.862201	4642	(-)	TNTATGNTAATT	AATTAACAAAGC
M00136	V$OCT1_02	0.789117	4647	(-)	NNGAATATKCANNNN	ACAAAGCATTTTATT
M00162	V$OCT1_06	0.829297	4650	(+)	CWNAWTKWSATRYN	AAGCATTTTATTCT
M00162	V$OCT1_06	0.807031	4651	(-)	CWNAWTKWSATRYN	AGCATTTTATTCTA
M00162	V$OCT1_06	0.804297	4661	(-)	CWNAWTKWSATRYN	TCTATGTTATTTAT
M00248	V$OCT1_07	0.895272	4661	(+)	TNTATGNTAATT	TCTATGTTATTT
M00135	V$OCT1_01	0.757535	4667	(+)	NNNNWTATGCAAATNTNNN	TTATTTATGGTATTATACC
M00248	V$OCT1_07	0.795209	4670	(+)	TNTATGNTAATT	TTTATGGTATTA
M00138	V$OCT1_04	0.799038	4673	(+)	NNNNNNNWATGCAAATNNNWNNW	ATGGTATTATACCAATTCTTTAT
M00135	V$OCT1_01	0.737314	4675	(+)	NNNNWTATGCAAATNTNNN	GGTATTATACCAATTCTTT
M00162	V$OCT1_06	0.834375	4690	(+)	CWNAWTKWSATRYN	CTTTATTGCCTTTT
M00162	V$OCT1_06	0.800391	4710	(-)	CWNAWTKWSATRYN	AAAATCACTTAGAT
M00162	V$OCT1_06	0.826953	4713	(+)	CWNAWTKWSATRYN	ATCACTTAGATTAT
M00137	V$OCT1_03	0.885421	4720	(-)	NNNRTAATNANNN	AGATTATTATTAG
M00135	V$OCT1_01	0.736169	4723	(-)	NNNNWTATGCAAATNTNNN	TTATTATTAGATTATTGAA
M00137	V$OCT1_03	0.847096	4723	(-)	NNNRTAATNANNN	TTATTATTAGATT
M00138	V$OCT1_04	0.791092	4787	(-)	NNNNNNNWATGCAAATNNNWNNW	ATGAAAGATAATAATAGTCAAAT
M00137	V$OCT1_03	0.867641	4791	(+)	NNNRTAATNANNN	AAGATAATAATAG
M00138	V$OCT1_04	0.828524	4794	(+)	NNNNNNNWATGCAAATNNNWNNW	ATAATAATAGTCAAATTTACTGG
M00135	V$OCT1_01	0.763258	4796	(+)	NNNNWTATGCAAATNTNNN	AATAATAGTCAAATTTACT
M00136	V$OCT1_02	0.817001	4796	(+)	NNGAATATKCANNNN	AATAATAGTCAAATT
M00248	V$OCT1_07	0.795209	4799	(+)	TNTATGNTAATT	AATAGTCAAATT

M00138	V$OCT1_04	0.811585	4818	(+)	NNNNNNNWATGCAAATNNNWNNW	ACAAAATTTTTTAAATATTTTTA
M00138	V$OCT1_04	0.830615	4819	(-)	NNNNNNNWATGCAAATNNNWNNW	CAAAATTTTTTAAATATTTTTAA
M00162	V$OCT1_06	0.903516	4823	(+)	CWNAWTKWSATRYN	ATTTTTTAAATATT
M00136	V$OCT1_02	0.831077	4825	(-)	NNGAATATKCANNNN	TTTTTAAATATTTTT
M00137	V$OCT1_03	0.84275	4867	(+)	NNNRTAATNANNN	TAAATAAAGAAAA
M00138	V$OCT1_04	0.783563	4902	(+)	NNNNNNNWATGCAAATNNNWNNW	AAATGATATTGCAAGAAATATTA
M00135	V$OCT1_01	0.753338	4931	(-)	NNNNWTATGCAAATNTNNN	TCACAAAAAGCATATTTAT
M00136	V$OCT1_02	0.875744	4935	(-)	NNGAATATKCANNNN	AAAAAGCATATTTAT
M00136	V$OCT1_02	0.841635	4938	(+)	NNGAATATKCANNNN	AAGCATATTTATTTT
M00162	V$OCT1_06	0.804297	4941	(+)	CWNAWTKWSATRYN	CATATTTATTTTTT
M00162	V$OCT1_06	0.842187	4941	(-)	CWNAWTKWSATRYN	CATATTTATTTTTT
M00138	V$OCT1_04	0.78189	4943	(-)	NNNNNNNWATGCAAATNNNWNNW	TATTTATTTTTTAATTTGCAGCA
M00162	V$OCT1_06	0.821484	4947	(+)	CWNAWTKWSATRYN	TATTTTTTAATTTG
M00248	V$OCT1_07	0.831673	4947	(+)	TNTATGNTAATT	TATTTTTTAATT
M00138	V$OCT1_04	0.863446	4949	(-)	NNNNNNNWATGCAAATNNNWNNW	TTTTTTAATTTGCAGCAGCAAAA
M00135	V$OCT1_01	0.744182	4951	(-)	NNNNWTATGCAAATNTNNN	TTTTAATTTGCAGCAGCAA
M00162	V$OCT1_06	0.873437	4967	(+)	CWNAWTKWSATRYN	CAAAATGTAAGATA
M00162	V$OCT1_06	0.898437	4968	(-)	CWNAWTKWSATRYN	AAAATGTAAGATAC
M00162	V$OCT1_06	0.833984	4978	(+)	CWNAWTKWSATRYN	ATACATTTCTTACA
M00162	V$OCT1_06	0.842187	4979	(-)	CWNAWTKWSATRYN	TACATTTCTTACAG
M00162	V$OCT1_06	0.821094	4982	(+)	CWNAWTKWSATRYN	ATTTCTTACAGATA
M00136	V$OCT1_02	0.772334	4989	(+)	NNGAATATKCANNNN	ACAGATATCCATCCA
M00162	V$OCT1_06	0.855078	4998	(+)	CWNAWTKWSATRYN	CATCCATTCATGTT
M00162	V$OCT1_06	0.800391	5039	(-)	CWNAWTKWSATRYN	AACATCAATGAACT
M00248	V$OCT1_07	0.792453	5062	(-)	TNTATGNTAATT	AAGGAACATAAA
M00162	V$OCT1_06	0.868359	5069	(+)	CWNAWTKWSATRYN	ATAAATGATATTTA
M00138	V$OCT1_04	0.860728	5070	(+)	NNNNNNNWATGCAAATNNNWNNW	TAAATGATATTTAAATTATTTTA
M00162	V$OCT1_06	0.804297	5070	(-)	CWNAWTKWSATRYN	TAAATGATATTTAA
M00138	V$OCT1_04	0.846717	5071	(-)	NNNNNNNWATGCAAATNNNWNNW	AAATGATATTTAAATTATTTTAA
M00135	V$OCT1_01	0.734071	5072	(+)	NNNNWTATGCAAATNTNNN	AATGATATTTAAATTATTT
M00136	V$OCT1_02	0.794532	5072	(+)	NNGAATATKCANNNN	AATGATATTTAAATT
M00135	V$OCT1_01	0.7438	5073	(-)	NNNNWTATGCAAATNTNNN	ATGATATTTAAATTATTTT
M00195	V$OCT1_Q6	0.804312	5074	(+)	NNNNATGCAAATNAN	TGATATTTAAATTAT

M00162	V$OCT1_06	0.847656	5075	(+)	CWNAWTKWSATRYN	GATATTTAAATTAT
M00162	V$OCT1_06	0.916797	5075	(-)	CWNAWTKWSATRYN	GATATTTAAATTAT
M00248	V$OCT1_07	0.872589	5075	(+)	TNTATGNTAATT	GATATTTAAATT
M00138	V$OCT1_04	0.812003	5079	(+)	NNNNNNNWATGCAAATNNNWNNW	TTTAAATTATTTTAAAGTTACCT
M00138	V$OCT1_04	0.781681	5081	(-)	NNNNNNNWATGCAAATNNNWNNW	TAAATTATTTTAAAGTTACCTCA
M00162	V$OCT1_06	0.839453	5084	(+)	CWNAWTKWSATRYN	ATTATTTTAAAGTT
M00162	V$OCT1_06	0.81875	5086	(-)	CWNAWTKWSATRYN	TATTTTAAAGTTAC
M00162	V$OCT1_06	0.839453	5097	(-)	CWNAWTKWSATRYN	TACCTCAAAATCAT
M00162	V$OCT1_06	0.878906	5103	(-)	CWNAWTKWSATRYN	AAAATCATAATTTG
M00137	V$OCT1_03	0.845121	5106	(+)	NNNRTAATNANNN	ATCATAATTTGCA
M00136	V$OCT1_02	0.781808	5108	(+)	NNGAATATKCANNNN	CATAATTTGCAACCT
M00195	V$OCT1_Q6	0.824509	5109	(-)	NNNNATGCAAATNAN	ATAATTTGCAACCTA
M00137	V$OCT1_03	0.841169	5137	(+)	NNNRTAATNANNN	TTGACAAAGAGAA
M00138	V$OCT1_04	0.791719	5148	(+)	NNNNNNNWATGCAAATNNNWNNW	AATTTTTAAGGGAAAAACAGAGT
M00138	V$OCT1_04	0.87683	5178	(+)	NNNNNNNWATGCAAATNNNWNNW	TCAAGATAATGAAAATACGAAAA
M00135	V$OCT1_01	0.779473	5180	(+)	NNNNWTATGCAAATNTNNN	AAGATAATGAAAATACGAA
M00137	V$OCT1_03	0.897669	5180	(+)	NNNRTAATNANNN	AAGATAATGAAAA
M00195	V$OCT1_Q6	0.852074	5182	(+)	NNNNATGCAAATNAN	GATAATGAAAATACG
M00161	V$OCT1_05	0.858376	5183	(-)	MKNATTTGCATAYY	ATAATGAAAATACG
M00162	V$OCT1_06	0.866016	5183	(-)	CWNAWTKWSATRYN	ATAATGAAAATACG
M00248	V$OCT1_07	0.791817	5185	(-)	TNTATGNTAATT	AATGAAAATACG
M00138	V$OCT1_04	0.805939	5186	(+)	NNNNNNNWATGCAAATNNNWNNW	ATGAAAATACGAAAATTTTCTTT
M00135	V$OCT1_01	0.768791	5188	(+)	NNNNWTATGCAAATNTNNN	GAAAATACGAAAATTTTCT
M00162	V$OCT1_06	0.860156	5191	(-)	CWNAWTKWSATRYN	AATACGAAAATTTT
M00162	V$OCT1_06	0.875781	5259	(+)	CWNAWTKWSATRYN	AAAAATTTCCTGTA
M00162	V$OCT1_06	0.821484	5290	(-)	CWNAWTKWSATRYN	GAACTTTCAAAATG
M00162	V$OCT1_06	0.821094	5322	(+)	CWNAWTKWSATRYN	GAATTCTAAATATT
M00162	V$OCT1_06	0.834375	5322	(-)	CWNAWTKWSATRYN	GAATTCTAAATATT
M00136	V$OCT1_02	0.826746	5324	(-)	NNGAATATKCANNNN	ATTCTAAATATTGAT
M00136	V$OCT1_02	0.772875	5327	(+)	NNGAATATKCANNNN	CTAAATATTGATTTC
M00135	V$OCT1_01	0.740557	5348	(+)	NNNNWTATGCAAATNTNNN	TTAACTTTGCAACTGTGAA
M00138	V$OCT1_04	0.806775	5370	(-)	NNNNNNNWATGCAAATNNNWNNW	ACAAGGCATTAGTATAAATGAAT
M00135	V$OCT1_01	0.802556	5372	(-)	NNNNWTATGCAAATNTNNN	AAGGCATTAGTATAAATGA

M00248	V$OCT1_07	0.800297	5376	(-)	TNTATGNTAATT	CATTAGTATAAA
M00162	V$OCT1_06	0.834375	5384	(-)	CWNAWTKWSATRYN	TAAATGAATGAATG
M00162	V$OCT1_06	0.842187	5404	(-)	CWNAWTKWSATRYN	AGAATGAATTAGTG
M00162	V$OCT1_06	0.839453	5477	(-)	CWNAWTKWSATRYN	AGTACTACATAAAG
M00162	V$OCT1_06	0.804297	5484	(+)	CWNAWTKWSATRYN	CATAAAGCAATATA
M00138	V$OCT1_04	0.830197	5506	(-)	NNNNNNNWATGCAAATNNNWNNW	AAGAATGTTTTACATGTCAATTT
M00162	V$OCT1_06	0.90625	5510	(+)	CWNAWTKWSATRYN	ATGTTTTACATGTC
M00195	V$OCT1_Q6	0.80786	5510	(-)	NNNNATGCAAATNAN	ATGTTTTACATGTCA
M00138	V$OCT1_04	0.802175	5511	(+)	NNNNNNNWATGCAAATNNNWNNW	TGTTTTACATGTCAATTTGATCA
M00135	V$OCT1_01	0.741702	5513	(+)	NNNNWTATGCAAATNTNNN	TTTTACATGTCAATTTGAT
M00162	V$OCT1_06	0.992188	5516	(-)	CWNAWTKWSATRYN	TACATGTCAATTTG
M00248	V$OCT1_07	0.842061	5516	(+)	TNTATGNTAATT	TACATGTCAATT
M00162	V$OCT1_06	0.808203	5538	(-)	CWNAWTKWSATRYN	TGTTTTTCAAAGTT
M00138	V$OCT1_04	0.818068	5558	(+)	NNNNNNNWATGCAAATNNNWNNW	GCTTTGTTATTAAAAAAAACATA
M00137	V$OCT1_03	0.852627	5561	(-)	NNNRTAATNANNN	TTGTTATTAAAAA
M00248	V$OCT1_07	0.797329	5571	(-)	TNTATGNTAATT	AAAAAACATACA
M00162	V$OCT1_06	0.890625	5583	(-)	CWNAWTKWSATRYN	GGTATTTAAAAAAC
M00248	V$OCT1_07	0.797329	5590	(-)	TNTATGNTAATT	AAAAAACATACA
M00138	V$OCT1_04	0.852363	5597	(+)	NNNNNNNWATGCAAATNNNWNNW	ATACAGGTATTTAAAAATTTACA
M00162	V$OCT1_06	0.826562	5602	(+)	CWNAWTKWSATRYN	GGTATTTAAAAATT
M00162	V$OCT1_06	0.898437	5602	(-)	CWNAWTKWSATRYN	GGTATTTAAAAATT
M00138	V$OCT1_04	0.837097	5606	(-)	NNNNNNNWATGCAAATNNNWNNW	TTTAAAAATTTACAGCTTCTTAA
M00136	V$OCT1_02	0.813752	5621	(-)	NNGAATATKCANNNN	CTTCTTAATATTCTT
M00136	V$OCT1_02	0.848132	5624	(+)	NNGAATATKCANNNN	CTTAATATTCTTCAT
M00162	V$OCT1_06	0.813281	5628	(+)	CWNAWTKWSATRYN	ATATTCTTCATACC
M00138	V$OCT1_04	0.786282	5694	(+)	NNNNNNNWATGCAAATNNNWNNW	GATTCTTTATTTTAAAACTATTA
M00138	V$OCT1_04	0.802384	5697	(-)	NNNNNNNWATGCAAATNNNWNNW	TCTTTATTTTAAAACTATTAAAT
M00162	V$OCT1_06	0.90625	5709	(+)	CWNAWTKWSATRYN	AACTATTAAATATA
M00162	V$OCT1_06	0.804297	5717	(-)	CWNAWTKWSATRYN	AATATAATATAATG
M00138	V$OCT1_04	0.803639	5720	(+)	NNNNNNNWATGCAAATNNNWNNW	ATAATATAATGAAAAGAAAGAAG
M00137	V$OCT1_03	0.890162	5722	(+)	NNNRTAATNANNN	AATATAATGAAAA
M00138	V$OCT1_04	0.81765	5743	(+)	NNNNNNNWATGCAAATNNNWNNW	TAAAAAATATCCTAATCAAATAT
M00135	V$OCT1_01	0.789203	5745	(+)	NNNNWTATGCAAATNTNNN	AAAAATATCCTAATCAAAT

M00136	V$OCT1_02	0.776936	5745	(+)	NNGAATATKCANNNN	AAAAATATCCTAATC
M00195	V$OCT1_Q6	0.812227	5747	(+)	NNNNATGCAAATNAN	AAATATCCTAATCAA
M00137	V$OCT1_03	0.891347	5751	(+)	NNNRTAATNANNN	ATCCTAATCAAAT
M00162	V$OCT1_06	0.804687	5753	(+)	CWNAWTKWSATRYN	CCTAATCAAATATC
M00162	V$OCT1_06	0.800781	5754	(-)	CWNAWTKWSATRYN	CTAATCAAATATCC
M00136	V$OCT1_02	0.790742	5755	(-)	NNGAATATKCANNNN	TAATCAAATATCCTT
M00138	V$OCT1_04	0.78921	5765	(-)	NNNNNNNWATGCAAATNNNWNNW	TCCTTTAAGTATCCTAATCAAGA
M00135	V$OCT1_01	0.791873	5769	(+)	NNNNWTATGCAAATNTNNN	TTAAGTATCCTAATCAAGA
M00195	V$OCT1_Q6	0.804585	5771	(+)	NNNNATGCAAATNAN	AAGTATCCTAATCAA
M00137	V$OCT1_03	0.890952	5775	(+)	NNNRTAATNANNN	ATCCTAATCAAGA
M00162	V$OCT1_06	0.821094	5788	(-)	CWNAWTKWSATRYN	TGAATGAACAATTC
M00162	V$OCT1_06	0.813672	5810	(-)	CWNAWTKWSATRYN	CAAAACTAAAACAG
M00162	V$OCT1_06	0.821094	5823	(-)	CWNAWTKWSATRYN	GATATCTCTAATTC
M00138	V$OCT1_04	0.825178	5825	(+)	NNNNNNNWATGCAAATNNNWNNW	TATCTCTAATTCAAAGAAAAAAA
M00138	V$OCT1_04	0.792765	5841	(+)	NNNNNNNWATGCAAATNNNWNNW	AAAAAAAGATGCAACATGTATGT
M00136	V$OCT1_02	0.78641	5843	(+)	NNGAATATKCANNNN	AAAAAGATGCAACAT
M00162	V$OCT1_06	0.804297	5868	(+)	CWNAWTKWSATRYN	CAAAATTGGCTGGA
M00162	V$OCT1_06	0.813672	5934	(+)	CWNAWTKWSATRYN	CTCTCTGTACTGCT
M00162	V$OCT1_06	0.800781	5971	(-)	CWNAWTKWSATRYN	CAAACTACATGGAG
M00136	V$OCT1_02	0.778018	5979	(-)	NNGAATATKCANNNN	ATGGAGAAAATTCAT
M00136	V$OCT1_02	0.891716	5982	(+)	NNGAATATKCANNNN	GAGAAAATTCATTTT
M00162	V$OCT1_06	0.834375	5983	(+)	CWNAWTKWSATRYN	AGAAAATTCATTTT
M00162	V$OCT1_06	0.855469	5984	(-)	CWNAWTKWSATRYN	GAAAATTCATTTTG
M00138	V$OCT1_04	0.809912	5986	(-)	NNNNNNNWATGCAAATNNNWNNW	AAATTCATTTTGCTTAAATATTT
M00135	V$OCT1_01	0.779855	5988	(-)	NNNNWTATGCAAATNTNNN	ATTCATTTTGCTTAAATAT
M00138	V$OCT1_04	0.805312	5990	(-)	NNNNNNNWATGCAAATNNNWNNW	TCATTTTGCTTAAATATTTATAC
M00136	V$OCT1_02	0.840552	5996	(-)	NNGAATATKCANNNN	TGCTTAAATATTTAT
M00138	V$OCT1_04	0.824341	5996	(-)	NNNNNNNWATGCAAATNNNWNNW	TGCTTAAATATTTATACATTATT
M00136	V$OCT1_02	0.810233	5999	(+)	NNGAATATKCANNNN	TTAAATATTTATACA
M00138	V$OCT1_04	0.854454	6000	(-)	NNNNNNNWATGCAAATNNNWNNW	TAAATATTTATACATTATTGAAC
M00137	V$OCT1_03	0.853418	6001	(-)	NNNRTAATNANNN	AAATATTTATACA
M00136	V$OCT1_02	0.773687	6044	(-)	NNGAATATKCANNNN	TTGTCACATAATCTT
M00137	V$OCT1_03	0.866851	6048	(+)	NNNRTAATNANNN	CACATAATCTTTA

M00162	V$OCT1_06	0.808594	6058	(-)	CWNAWTKWSATRYN	TTAATCCAATGCTC
M00138	V$OCT1_04	0.790673	6117	(+)	NNNNNNNWATGCAAATNNNWNNW	TTAGTTAAAGGAAAATGTAGAAT
M00138	V$OCT1_04	0.790673	6123	(+)	NNNNNNNWATGCAAATNNNWNNW	AAAGGAAAATGTAGAATATTTAC
M00136	V$OCT1_02	0.785328	6125	(+)	NNGAATATKCANNNN	AGGAAAATGTAGAAT
M00138	V$OCT1_04	0.789419	6125	(-)	NNNNNNNWATGCAAATNNNWNNW	AGGAAAATGTAGAATATTTACAA
M00162	V$OCT1_06	0.821094	6128	(-)	CWNAWTKWSATRYN	AAAATGTAGAATAT
M00136	V$OCT1_02	0.836221	6131	(-)	NNGAATATKCANNNN	ATGTAGAATATTTAC
M00136	V$OCT1_02	0.844613	6134	(+)	NNGAATATKCANNNN	TAGAATATTTACAAT
M00138	V$OCT1_04	0.790464	6134	(-)	NNNNNNNWATGCAAATNNNWNNW	TAGAATATTTACAATGATTTATC
M00137	V$OCT1_03	0.874753	6141	(+)	NNNRTAATNANNN	TTTACAATGATTT
M00135	V$OCT1_01	0.750095	6158	(+)	NNNNWTATGCAAATNTNNN	AAACATATAAAAATCTACA
M00162	V$OCT1_06	0.862891	6159	(-)	CWNAWTKWSATRYN	AACATATAAAAATC
M00138	V$OCT1_04	0.784191	6163	(-)	NNNNNNNWATGCAAATNNNWNNW	TATAAAAATCTACAGCACTTTTT
M00162	V$OCT1_06	0.800391	6167	(-)	CWNAWTKWSATRYN	AAAATCTACAGCAC
M00162	V$OCT1_06	0.872656	6190	(+)	CWNAWTKWSATRYN	AGCTCTGAAATGCA
M00138	V$OCT1_04	0.817231	6191	(+)	NNNNNNNWATGCAAATNNNWNNW	GCTCTGAAATGCAGATTAAAGAG
M00135	V$OCT1_01	0.738459	6193	(+)	NNNNWTATGCAAATNTNNN	TCTGAAATGCAGATTAAAG
M00195	V$OCT1_Q6	0.806496	6195	(+)	NNNNATGCAAATNAN	TGAAATGCAGATTAA
M00136	V$OCT1_02	0.782891	6196	(-)	NNGAATATKCANNNN	GAAATGCAGATTAAA
M00138	V$OCT1_04	0.795483	6204	(+)	NNNNNNNWATGCAAATNNNWNNW	GATTAAAGAGGAAAATTCTTCAG
M00136	V$OCT1_02	0.784786	6209	(-)	NNGAATATKCANNNN	AAGAGGAAAATTCTT
M00136	V$OCT1_02	0.802653	6212	(+)	NNGAATATKCANNNN	AGGAAAATTCTTCAG
M00138	V$OCT1_04	0.790464	6239	(+)	NNNNNNNWATGCAAATNNNWNNW	GTAGGCTTTTTTAAAATTTTCCT
M00138	V$OCT1_04	0.803848	6241	(-)	NNNNNNNWATGCAAATNNNWNNW	AGGCTTTTTTAAAATTTTCCTTA
M00162	V$OCT1_06	0.826562	6244	(+)	CWNAWTKWSATRYN	CTTTTTTAAAATTT
M00138	V$OCT1_04	0.81284	6247	(-)	NNNNNNNWATGCAAATNNNWNNW	TTTTAAAATTTTCCTTACCAGTT
M00137	V$OCT1_03	0.851442	6255	(-)	NNNRTAATNANNN	TTTTCCTTACCAG
M00138	V$OCT1_04	0.78921	6289	(-)	NNNNNNNWATGCAAATNNNWNNW	TATTTCCTTTGGCATCCATTTTA
M00162	V$OCT1_06	0.834375	6299	(-)	CWNAWTKWSATRYN	GGCATCCATTTTAT
M00162	V$OCT1_06	0.808594	6302	(+)	CWNAWTKWSATRYN	ATCCATTTTATTTG
M00248	V$OCT1_07	0.893152	6310	(+)	TNTATGNTAATT	TATTTGGTAATT
M00138	V$OCT1_04	0.803848	6312	(-)	NNNNNNNWATGCAAATNNNWNNW	TTTGGTAATTATCATTTTATATT
M00137	V$OCT1_03	0.898459	6313	(+)	NNNRTAATNANNN	TTGGTAATTATCA

M00135	V$OCT1_01	0.743991	6314	(-)	NNNNWTATGCAAATNTNNN	TGGTAATTATCATTTTATA
M00137	V$OCT1_03	0.868827	6314	(-)	NNNRTAATNANNN	TGGTAATTATCAT
M00161	V$OCT1_05	0.915657	6316	(+)	MKNATTTGCATAYY	GTAATTATCATTTT
M00162	V$OCT1_06	0.852734	6316	(+)	CWNAWTKWSATRYN	GTAATTATCATTTT
M00195	V$OCT1_Q6	0.815502	6316	(-)	NNNNATGCAAATNAN	GTAATTATCATTTTA
M00248	V$OCT1_07	0.843333	6318	(-)	TNTATGNTAATT	AATTATCATTTT
M00138	V$OCT1_04	0.786491	6319	(-)	NNNNNNNWATGCAAATNNNWNNW	ATTATCATTTTATATTGGAATGT
M00138	V$OCT1_04	0.807612	6325	(-)	NNNNNNNWATGCAAATNNNWNNW	ATTTTATATTGGAATGTTTGTCA
M00162	V$OCT1_06	0.868359	6329	(+)	CWNAWTKWSATRYN	TATATTGGAATGTT
M00138	V$OCT1_04	0.796529	6334	(-)	NNNNNNNWATGCAAATNNNWNNW	TGGAATGTTTGTCATGAAAAATA
M00162	V$OCT1_06	0.834375	6338	(+)	CWNAWTKWSATRYN	ATGTTTGTCATGAA
M00138	V$OCT1_04	0.808448	6339	(+)	NNNNNNNWATGCAAATNNNWNNW	TGTTTGTCATGAAAAATAAAGTT
M00162	V$OCT1_06	0.826562	6354	(+)	CWNAWTKWSATRYN	ATAAAGTTAATTCT
M00248	V$OCT1_07	0.817045	6354	(+)	TNTATGNTAATT	ATAAAGTTAATT
M00162	V$OCT1_06	0.829297	6355	(-)	CWNAWTKWSATRYN	TAAAGTTAATTCTC
M00135	V$OCT1_01	0.780809	6393	(+)	NNNNWTATGCAAATNTNNN	TGGAAGATGCTAAGGTAAG
M00138	V$OCT1_04	0.795692	6405	(-)	NNNNNNNWATGCAAATNNNWNNW	AGGTAAGATCTTTATTTTAATAA
M00138	V$OCT1_04	0.785864	6410	(+)	NNNNNNNWATGCAAATNNNWNNW	AGATCTTTATTTTAATAAACTCT
M00138	V$OCT1_04	0.801757	6411	(-)	NNNNNNNWATGCAAATNNNWNNW	GATCTTTATTTTAATAAACTCTA
M00135	V$OCT1_01	0.732354	6412	(+)	NNNNWTATGCAAATNTNNN	ATCTTTATTTTAATAAACT
M00248	V$OCT1_07	0.831673	6415	(+)	TNTATGNTAATT	TTTATTTTAATA
M00162	V$OCT1_06	0.855078	6438	(-)	CWNAWTKWSATRYN	TATATATCATAAAC
M00137	V$OCT1_03	0.870407	6443	(+)	NNNRTAATNANNN	ATCATAAACAAGA
M00162	V$OCT1_06	0.813672	6522	(-)	CWNAWTKWSATRYN	CATACCCAAGAATG
M00162	V$OCT1_06	0.839453	6566	(+)	CWNAWTKWSATRYN	GGAAATTTGACATT
M00162	V$OCT1_06	0.919141	6568	(+)	CWNAWTKWSATRYN	AAATTTGACATTTA
M00162	V$OCT1_06	0.860156	6569	(-)	CWNAWTKWSATRYN	AATTTGACATTTAC
M00138	V$OCT1_04	0.823505	6570	(-)	NNNNNNNWATGCAAATNNNWNNW	ATTTGACATTTACATGATCAGGA
M00135	V$OCT1_01	0.839374	6572	(-)	NNNNWTATGCAAATNTNNN	TTGACATTTACATGATCAG
M00162	V$OCT1_06	0.876172	6574	(+)	CWNAWTKWSATRYN	GACATTTACATGAT
M00162	V$OCT1_06	0.821484	6574	(-)	CWNAWTKWSATRYN	GACATTTACATGAT
M00195	V$OCT1_Q6	0.810862	6574	(-)	NNNNATGCAAATNAN	GACATTTACATGATC
M00248	V$OCT1_07	0.793089	6576	(-)	TNTATGNTAATT	CATTTACATGAT

M00135	V$OCT1_01	0.731591	6592	(-)	NNNNWTATGCAAATNTNNN	AGAAACTTTGCTTCTTTAT
M00162	V$OCT1_06	0.826562	6593	(+)	CWNAWTKWSATRYN	GAAACTTTGCTTCT
M00137	V$OCT1_03	0.840379	6601	(-)	NNNRTAATNANNN	GCTTCTTTATTAA
M00138	V$OCT1_04	0.827687	6601	(+)	NNNNNNNWATGCAAATNNNNWNNW	GCTTCTTTATTAAAATCAAGAGT
M00162	V$OCT1_06	0.834375	6605	(+)	CWNAWTKWSATRYN	CTTTATTAAAATCA
M00162	V$OCT1_06	0.852734	6625	(-)	CWNAWTKWSATRYN	AAAAGCAAAATGTG
M00138	V$OCT1_04	0.783145	6627	(-)	NNNNNNNWATGCAAATNNNNWNNW	AAGCAAAATGTGCATGAGTGTTT
M00135	V$OCT1_01	0.790538	6629	(-)	NNNNWTATGCAAATNTNNN	GCAAAATGTGCATGAGTGT
M00138	V$OCT1_04	0.810958	6638	(+)	NNNNNNNWATGCAAATNNNNWNNW	GCATGAGTGTTTAAAAATGAACA
M00162	V$OCT1_06	0.847266	6643	(-)	CWNAWTKWSATRYN	AGTGTTTAAAAATG
M00162	V$OCT1_06	0.860547	6651	(-)	CWNAWTKWSATRYN	AAAATGAACATCTG
M00162	V$OCT1_06	0.813281	6663	(-)	CWNAWTKWSATRYN	TGACTCAAAAAATT
M00162	V$OCT1_06	0.800781	6671	(-)	CWNAWTKWSATRYN	AAAATTAAAACCCT
M00162	V$OCT1_06	0.821484	6685	(+)	CWNAWTKWSATRYN	GTTTCTGTCATCCC
M00162	V$OCT1_06	0.842187	6747	(+)	CWNAWTKWSATRYN	AAGAATTTATTGCA
M00162	V$OCT1_06	0.821484	6748	(-)	CWNAWTKWSATRYN	AGAATTTATTGCAG
M00137	V$OCT1_03	0.847886	6758	(+)	NNNRTAATNANNN	GCAGCAATTATGT
M00162	V$OCT1_06	0.813672	6774	(+)	CWNAWTKWSATRYN	ATCAATGGGCTATG
M00162	V$OCT1_06	0.829297	6800	(+)	CWNAWTKWSATRYN	CACTCAGTCATGCC
M00162	V$OCT1_06	0.893359	6838	(-)	CWNAWTKWSATRYN	AGCATGCCAGGCTT
M00162	V$OCT1_06	0.834375	6899	(+)	CWNAWTKWSATRYN	CAGTCAGTGATGCT
M00162	V$OCT1_06	0.808594	6933	(-)	CWNAWTKWSATRYN	GACATCCCTGTCTC
M00135	V$OCT1_01	0.756009	6990	(-)	NNNNWTATGCAAATNTNNN	CGGTTCTTTGCATCAGGTG
M00195	V$OCT1_Q6	0.835972	6992	(-)	NNNNATGCAAATNAN	GTTCTTTGCATCAGG
M00162	V$OCT1_06	0.800781	7014	(+)	CWNAWTKWSATRYN	AGTATTGAAACTTC
M00248	V$OCT1_07	0.796057	7014	(+)	TNTATGNTAATT	AGTATTGAAACT
M00135	V$OCT1_01	0.732926	7026	(-)	NNNNWTATGCAAATNTNNN	TCAGCTTTAGCATCAGTTC
M00136	V$OCT1_02	0.930157	7047	(-)	NNGAATATKCANNNN	CCAATGAATATTCAG
M00136	V$OCT1_02	0.963184	7050	(+)	NNGAATATKCANNNN	ATGAATATTCAGGAT
M00162	V$OCT1_06	0.813672	7053	(-)	CWNAWTKWSATRYN	AATATTCAGGATTG
M00135	V$OCT1_01	0.742846	7093	(-)	NNNNWTATGCAAATNTNNN	TCTCCAGTAGCATGTTGGG
M00162	V$OCT1_06	0.808594	7133	(-)	CWNAWTKWSATRYN	CATCTTTCAGTGTC
M00135	V$OCT1_01	0.757345	7154	(-)	NNNNWTATGCAAATNTNNN	TTAACGTTTTCATACTGTT

M00162	V$OCT1_06	0.855078	7156	(+)	CWNAWTKWSATRYN	AACGTTTTCATACT
M00162	V$OCT1_06	0.829687	7209	(-)	CWNAWTKWSATRYN	CCCATTCCATTCTC
M00195	V$OCT1_Q6	0.815775	7248	(+)	NNNNATGCAAATNAN	AAACATGAAAAAAAT
M00162	V$OCT1_06	0.924219	7249	(-)	CWNAWTKWSATRYN	AACATGAAAAAAAT
M00136	V$OCT1_02	0.825122	7255	(+)	NNGAATATKCANNNN	AAAAAAATTCATTAG
M00137	V$OCT1_03	0.884631	7260	(-)	NNNRTAATNANNN	AATTCATTAGCCC
M00137	V$OCT1_03	0.855788	7277	(-)	NNNRTAATNANNN	TACTTTTTACTCA
M00138	V$OCT1_04	0.793183	7306	(-)	NNNNNNNWATGCAAATNNNWNNW	ACTATTTTGCTGCATATTAGAAT
M00136	V$OCT1_02	0.864645	7312	(-)	NNGAATATKCANNNN	TTGCTGCATATTAGA
M00135	V$OCT1_01	0.752766	7316	(-)	NNNNWTATGCAAATNTNNN	TGCATATTAGAATCAATAT
M00138	V$OCT1_04	0.78189	7319	(+)	NNNNNNNWATGCAAATNNNWNNW	ATATTAGAATCAATATCTTAGTA
M00162	V$OCT1_06	0.826562	7324	(-)	CWNAWTKWSATRYN	AGAATCAATATCTT
M00135	V$OCT1_01	0.73884	7327	(+)	NNNNWTATGCAAATNTNNN	ATCAATATCTTAGTATGTA
M00162	V$OCT1_06	0.886719	7330	(-)	CWNAWTKWSATRYN	AATATCTTAGTATG
M00162	V$OCT1_06	0.812109	7338	(-)	CWNAWTKWSATRYN	AGTATGTACTTGGG
M00138	V$OCT1_04	0.787327	7356	(+)	NNNNNNNWATGCAAATNNNWNNW	GAAATCAGATCCATATGTAAATA
M00138	V$OCT1_04	0.854872	7362	(+)	NNNNNNNWATGCAAATNNNWNNW	AGATCCATATGTAAATATGAACA
M00135	V$OCT1_01	0.909958	7364	(+)	NNNNWTATGCAAATNTNNN	ATCCATATGTAAATATGAA
M00136	V$OCT1_02	0.812128	7364	(+)	NNGAATATKCANNNN	ATCCATATGTAAATA
M00195	V$OCT1_Q6	0.822052	7366	(+)	NNNNATGCAAATNAN	CCATATGTAAATATG
M00162	V$OCT1_06	0.860547	7367	(+)	CWNAWTKWSATRYN	CATATGTAAATATG
M00162	V$OCT1_06	0.971484	7367	(-)	CWNAWTKWSATRYN	CATATGTAAATATG
M00248	V$OCT1_07	0.821709	7367	(+)	TNTATGNTAATT	CATATGTAAATA
M00136	V$OCT1_02	0.793449	7369	(-)	NNGAATATKCANNNN	TATGTAAATATGAAC
M00137	V$OCT1_03	0.857764	7369	(+)	NNNRTAATNANNN	TATGTAAATATGA
M00138	V$OCT1_04	0.821414	7369	(-)	NNNNNNNWATGCAAATNNNWNNW	TATGTAAATATGAACATCTATGA
M00135	V$OCT1_01	0.764021	7372	(+)	NNNNWTATGCAAATNTNNN	GTAAATATGAACATCTATG
M00136	V$OCT1_02	0.777206	7372	(+)	NNGAATATKCANNNN	GTAAATATGAACATC
M00162	V$OCT1_06	0.816016	7381	(-)	CWNAWTKWSATRYN	AACATCTATGACTT
M00138	V$OCT1_04	0.797365	7387	(+)	NNNNNNNWATGCAAATNNNWNNW	TATGACTTAGGAAAAAAACTAAA
M00162	V$OCT1_06	0.862891	7400	(+)	CWNAWTKWSATRYN	AAAAACTAAATGTT
M00162	V$OCT1_06	0.834375	7400	(-)	CWNAWTKWSATRYN	AAAAACTAAATGTT
M00162	V$OCT1_06	0.808203	7401	(-)	CWNAWTKWSATRYN	AAAACTAAATGTTT

M00162	V$OCT1_06	0.800391	7408	(-)	CWNAWTKWSATRYN	AATGTTTCAAAGAT
M00138	V$OCT1_04	0.780008	7413	(-)	NNNNNNNWATGCAAATNNNWNNW	TTCAAAGATTAGAACTTTTAAAA
M00162	V$OCT1_06	0.816016	7425	(+)	CWNAWTKWSATRYN	AACTTTTAAAAGCT
M00162	V$OCT1_06	0.821484	7436	(+)	CWNAWTKWSATRYN	GCTCATGTAATTTT
M00162	V$OCT1_06	0.827734	7437	(-)	CWNAWTKWSATRYN	CTCATGTAATTTTA
M00162	V$OCT1_06	0.805859	7442	(+)	CWNAWTKWSATRYN	GTAATTTTAGTTCC
M00162	V$OCT1_06	0.834766	7455	(+)	CWNAWTKWSATRYN	CATTCTGAAATTGA
M00138	V$OCT1_04	0.878294	7469	(-)	NNNNNNNWATGCAAATNNNWNNW	TTGTTCTATTTTCATATTTCCAA
M00135	V$OCT1_01	0.826593	7471	(-)	NNNNWTATGCAAATNTNNN	GTTCTATTTTCATATTTCC
M00162	V$OCT1_06	0.817187	7473	(+)	CWNAWTKWSATRYN	TCTATTTTCATATT
M00195	V$OCT1_Q6	0.828057	7473	(-)	NNNNATGCAAATNAN	TCTATTTTCATATTT
M00136	V$OCT1_02	0.789117	7475	(-)	NNGAATATKCANNNN	TATTTTCATATTTCC
M00138	V$OCT1_04	0.788582	7477	(-)	NNNNNNNWATGCAAATNNNWNNW	TTTTCATATTTCCAATTTATTTA
M00162	V$OCT1_06	0.804297	7481	(+)	CWNAWTKWSATRYN	CATATTTCCAATTT
M00162	V$OCT1_06	0.808203	7481	(-)	CWNAWTKWSATRYN	CATATTTCCAATTT
M00162	V$OCT1_06	0.839453	7483	(-)	CWNAWTKWSATRYN	TATTTCCAATTTAT
M00138	V$OCT1_04	0.853618	7488	(-)	NNNNNNNWATGCAAATNNNWNNW	CCAATTTATTTACATCAAAATTG
M00135	V$OCT1_01	0.735025	7490	(-)	NNNNWTATGCAAATNTNNN	AATTTATTTACATCAAAAT
M00162	V$OCT1_06	0.873437	7498	(-)	CWNAWTKWSATRYN	TACATCAAAATTGC
M00136	V$OCT1_02	0.79778	7522	(-)	NNGAATATKCANNNN	TGTGAGAATATAAAC
M00162	V$OCT1_06	0.853125	7563	(+)	CWNAWTKWSATRYN	CTCAATAGGATTCT
M00136	V$OCT1_02	0.776936	7569	(+)	NNGAATATKCANNNN	AGGATTCTTCATTCC
M00162	V$OCT1_06	0.808594	7576	(-)	CWNAWTKWSATRYN	TTCATTCCAGGTTT
M00137	V$OCT1_03	0.874753	7589	(-)	NNNRTAATNANNN	TCATTTTTATCAC
M00138	V$OCT1_04	0.816186	7592	(+)	NNNNNNNWATGCAAATNNNWNNW	TTTTTATCACGAAAATTCATATG
M00136	V$OCT1_02	0.808879	7597	(-)	NNGAATATKCANNNN	ATCACGAAAATTCAT
M00138	V$OCT1_04	0.828733	7598	(+)	NNNNNNNWATGCAAATNNNWNNW	TCACGAAAATTCATATGCATTTG
M00136	V$OCT1_02	0.871142	7600	(+)	NNGAATATKCANNNN	ACGAAAATTCATATG
M00162	V$OCT1_06	0.860547	7601	(+)	CWNAWTKWSATRYN	CGAAAATTCATATG
M00136	V$OCT1_02	0.820249	7603	(-)	NNGAATATKCANNNN	AAAATTCATATGCAT
M00138	V$OCT1_04	0.877039	7603	(-)	NNNNNNNWATGCAAATNNNWNNW	AAAATTCATATGCATTTGCATTC
M00162	V$OCT1_06	0.812109	7603	(+)	CWNAWTKWSATRYN	AAAATTCATATGCA
M00138	V$OCT1_04	0.787955	7604	(+)	NNNNNNNWATGCAAATNNNWNNW	AAATTCATATGCATTTGCATTCT

M00135	V$OCT1_01	0.745517	7605	(-)	NNNNWTATGCAAATNTNNN	AATTCATATGCATTTGCAT
M00136	V$OCT1_02	0.887385	7606	(+)	NNGAATATKCANNNN	ATTCATATGCATTTG
M00136	V$OCT1_02	0.850298	7609	(-)	NNGAATATKCANNNN	CATATGCATTTGCAT
M00138	V$OCT1_04	0.840443	7609	(-)	NNNNNNNWATGCAAATNNNWNNW	CATATGCATTTGCATTCTACTCA
M00135	V$OCT1_01	0.829264	7611	(-)	NNNNWTATGCAAATNTNNN	TATGCATTTGCATTCTACT
M00136	V$OCT1_02	0.815106	7612	(+)	NNGAATATKCANNNN	ATGCATTTGCATTCT
M00161	V$OCT1_05	0.888594	7613	(+)	MKNATTTGCATAYY	TGCATTTGCATTCT
M00162	V$OCT1_06	0.834766	7613	(+)	CWNAWTKWSATRYN	TGCATTTGCATTCT
M00195	V$OCT1_Q6	0.845251	7613	(-)	NNNNATGCAAATNAN	TGCATTTGCATTCTA
M00136	V$OCT1_02	0.772063	7615	(-)	NNGAATATKCANNNN	CATTTGCATTCTACT
M00248	V$OCT1_07	0.804749	7615	(-)	TNTATGNTAATT	CATTTGCATTCT
M00138	V$OCT1_04	0.796529	7632	(-)	NNNNNNNWATGCAAATNNNWNNW	AGAAAGTATCTGTATTTGTTTTA
M00162	V$OCT1_06	0.809375	7636	(-)	CWNAWTKWSATRYN	AGTATCTGTATTTG
M00138	V$OCT1_04	0.783145	7641	(+)	NNNNNNNWATGCAAATNNNWNNW	CTGTATTTGTTTTAATAAAAAGG
M00162	V$OCT1_06	0.826562	7641	(+)	CWNAWTKWSATRYN	CTGTATTTGTTTTA
M00138	V$OCT1_04	0.788791	7642	(-)	NNNNNNNWATGCAAATNNNWNNW	TGTATTTGTTTTAATAAAAAGGT
M00137	V$OCT1_03	0.84196	7649	(+)	NNNRTAATNANNN	GTTTTAATAAAAA
M00137	V$OCT1_03	0.879494	7652	(+)	NNNRTAATNANNN	TTAATAAAAAGGT
M00137	V$OCT1_03	0.88384	7680	(-)	NNNRTAATNANNN	AAGTTATTATACT
M00138	V$OCT1_04	0.805939	7701	(-)	NNNNNNNWATGCAAATNNNWNNW	CAACATATTTTAAATAAAATTGA
M00162	V$OCT1_06	0.826562	7705	(+)	CWNAWTKWSATRYN	ATATTTTAAATAAA
M00248	V$OCT1_07	0.807717	7707	(-)	TNTATGNTAATT	ATTTTAAATAAA
M00162	V$OCT1_06	0.847656	7711	(-)	CWNAWTKWSATRYN	TAAATAAAATTGAC
M00138	V$OCT1_04	0.781681	7712	(+)	NNNNNNNWATGCAAATNNNWNNW	AAATAAAATTGACAATCCAAAAA
M00162	V$OCT1_06	0.842187	7716	(+)	CWNAWTKWSATRYN	AAAATTGACAATCC
M00138	V$OCT1_04	0.815558	7718	(+)	NNNNNNNWATGCAAATNNNWNNW	AATTGACAATCCAAAAAAAAGTA
M00135	V$OCT1_01	0.747806	7730	(+)	NNNNWTATGCAAATNTNNN	AAAAAAAAGTAAATTTTAT
M00162	V$OCT1_06	0.842187	7733	(+)	CWNAWTKWSATRYN	AAAAAGTAAATTTT
M00162	V$OCT1_06	0.855078	7733	(-)	CWNAWTKWSATRYN	AAAAAGTAAATTTT
M00248	V$OCT1_07	0.793089	7733	(+)	TNTATGNTAATT	AAAAAGTAAATT
M00162	V$OCT1_06	0.834375	7757	(+)	CWNAWTKWSATRYN	AATCTTTTTATACC
M00137	V$OCT1_03	0.871592	7758	(-)	NNNRTAATNANNN	ATCTTTTTATACC
M00162	V$OCT1_06	0.834375	7765	(-)	CWNAWTKWSATRYN	TATACCCAATTGTT

M00162	V$OCT1_06	0.847656	7796	(-)	CWNAWTKWSATRYN	AGCAATCCAGTATG
M00135	V$OCT1_01	0.790538	7801	(+)	NNNNWTATGCAAATNTNNN	TCCAGTATGAAAGTGTGGA
M00162	V$OCT1_06	0.950781	7804	(-)	CWNAWTKWSATRYN	AGTATGAAAGTGTG
M00138	V$OCT1_04	0.79632	7820	(+)	NNNNNNNWATGCAAATNNNWNNW	ATAGTTTCCTTCTAATTCTAAAA
M00162	V$OCT1_06	0.808203	7832	(-)	CWNAWTKWSATRYN	TAATTCTAAAAGTC
M00138	V$OCT1_04	0.795065	7868	(-)	NNNNNNNWATGCAAATNNNWNNW	TCTTTCTTTTAGCAAATGAAAGC
M00135	V$OCT1_01	0.746852	7870	(-)	NNNNWTATGCAAATNTNNN	TTTCTTTTAGCAAATGAAA
M00135	V$OCT1_01	0.762495	7909	(+)	NNNNWTATGCAAATNTNNN	TGAGGTATGCCATTTTTAT
M00162	V$OCT1_06	0.816406	7911	(+)	CWNAWTKWSATRYN	AGGTATGCCATTTT
M00162	V$OCT1_06	0.932422	7912	(-)	CWNAWTKWSATRYN	GGTATGCCATTTTT
M00138	V$OCT1_04	0.84128	7918	(+)	NNNNNNNWATGCAAATNNNWNNW	CCATTTTTATGTTAATTAAGTAT
M00135	V$OCT1_01	0.816101	7920	(+)	NNNNWTATGCAAATNTNNN	ATTTTTATGTTAATTAAGT
M00248	V$OCT1_07	0.98834	7923	(+)	TNTATGNTAATT	TTTATGTTAATT
M00137	V$OCT1_03	0.853023	7926	(+)	NNNRTAATNANNN	ATGTTAATTAAGT
M00162	V$OCT1_06	0.834375	7928	(+)	CWNAWTKWSATRYN	GTTAATTAAGTATC
M00138	V$OCT1_04	0.809912	7931	(+)	NNNNNNNWATGCAAATNNNWNNW	AATTAAGTATCCCAATTAGAAAA
M00135	V$OCT1_01	0.762304	7933	(+)	NNNNWTATGCAAATNTNNN	TTAAGTATCCCAATTAGAA
M00195	V$OCT1_Q6	0.837336	7935	(+)	NNNNATGCAAATNAN	AAGTATCCCAATTAG
M00162	V$OCT1_06	0.948047	7936	(-)	CWNAWTKWSATRYN	AGTATCCCAATTAG
M00248	V$OCT1_07	0.791605	7936	(+)	TNTATGNTAATT	AGTATCCCAATT
M00137	V$OCT1_03	0.840379	7939	(+)	NNNRTAATNANNN	ATCCCAATTAGAA
M00135	V$OCT1_01	0.733308	7940	(-)	NNNNWTATGCAAATNTNNN	TCCCAATTAGAAAATGTTT
M00137	V$OCT1_03	0.849862	7940	(-)	NNNRTAATNANNN	TCCCAATTAGAAA
M00248	V$OCT1_07	0.793725	7944	(-)	TNTATGNTAATT	AATTAGAAAATG
M00138	V$OCT1_04	0.810749	7945	(+)	NNNNNNNWATGCAAATNNNWNNW	ATTAGAAAATGTTTATGAAAGTT
M00137	V$OCT1_03	0.891347	7951	(-)	NNNRTAATNANNN	AAATGTTTATGAA
M00138	V$OCT1_04	0.863446	7951	(+)	NNNNNNNWATGCAAATNNNWNNW	AAATGTTTATGAAAGTTTGTTGA
M00135	V$OCT1_01	0.798741	7953	(+)	NNNNWTATGCAAATNTNNN	ATGTTTATGAAAGTTTGTT
M00162	V$OCT1_06	0.808203	7955	(+)	CWNAWTKWSATRYN	GTTTATGAAAGTTT
M00162	V$OCT1_06	0.886719	7956	(-)	CWNAWTKWSATRYN	TTTATGAAAGTTTG
M00248	V$OCT1_07	0.794785	7956	(+)	TNTATGNTAATT	TTTATGAAAGTT
M00135	V$OCT1_01	0.734834	7977	(-)	NNNNWTATGCAAATNTNNN	ATAAAGTTTACATGTCCCG
M00162	V$OCT1_06	0.870703	7979	(+)	CWNAWTKWSATRYN	AAAGTTTACATGTC

M00162	V$OCT1_06	0.867969	8021	(-)	CWNAWTKWSATRYN	TGTATCTAATCTAT
M00138	V$OCT1_04	0.824132	8025	(+)	NNNNNNNWATGCAAATNNNWNNW	TCTAATCTATCCTAATTTTAACA
M00135	V$OCT1_01	0.783098	8027	(+)	NNNNWTATGCAAATNTNNN	TAATCTATCCTAATTTTAA
M00248	V$OCT1_07	0.903116	8030	(+)	TNTATGNTAATT	TCTATCCTAATT
M00135	V$OCT1_01	0.776994	8036	(-)	NNNNWTATGCAAATNTNNN	CTAATTTTAACATACAAGG
M00162	V$OCT1_06	0.857812	8036	(+)	CWNAWTKWSATRYN	CTAATTTTAACATA
M00162	V$OCT1_06	0.855078	8038	(+)	CWNAWTKWSATRYN	AATTTTAACATACA
M00248	V$OCT1_07	0.797329	8040	(-)	TNTATGNTAATT	TTTTAACATACA
M00162	V$OCT1_06	0.842187	8067	(-)	CWNAWTKWSATRYN	AAAATGTAAGAAAA
M00137	V$OCT1_03	0.842355	8078	(-)	NNNRTAATNANNN	AAACAATTATTTG
M00138	V$OCT1_04	0.781054	8080	(-)	NNNNNNNWATGCAAATNNNWNNW	ACAATTATTTGTCAGAGACAAAA
M00162	V$OCT1_06	0.808203	8085	(-)	CWNAWTKWSATRYN	TATTTGTCAGAGAC
M00137	V$OCT1_03	0.843145	8099	(+)	NNNRTAATNANNN	AAAATAAAGATTT
M00135	V$OCT1_01	0.73636	8127	(-)	NNNNWTATGCAAATNTNNN	ATCCTATCTGGATATCTGT
M00135	V$OCT1_01	0.739412	8143	(+)	NNNNWTATGCAAATNTNNN	TGTGGTATCCAAACATGAT
M00162	V$OCT1_06	0.860547	8146	(-)	CWNAWTKWSATRYN	GGTATCCAAACATG
M00138	V$OCT1_04	0.812631	8155	(+)	NNNNNNNWATGCAAATNNNWNNW	ACATGATTTTTCTAATTTTTTT
M00135	V$OCT1_01	0.764784	8157	(+)	NNNNWTATGCAAATNTNNN	ATGATTTTTCTAATTTTTT
M00162	V$OCT1_06	0.805859	8160	(+)	CWNAWTKWSATRYN	ATTTTTCTAATTTT
M00248	V$OCT1_07	0.819165	8160	(+)	TNTATGNTAATT	ATTTTTCTAATT
M00138	V$OCT1_04	0.78189	8183	(-)	NNNNNNNWATGCAAATNNNWNNW	CCCAGATTTTTGTAGAATTAAAT
M00135	V$OCT1_01	0.746852	8185	(-)	NNNNWTATGCAAATNTNNN	CAGATTTTTGTAGAATTAA
M00162	V$OCT1_06	0.807031	8195	(+)	CWNAWTKWSATRYN	TAGAATTAAATCTT
M00162	V$OCT1_06	0.868359	8196	(-)	CWNAWTKWSATRYN	AGAATTAAATCTTG
M00138	V$OCT1_04	0.81765	8205	(+)	NNNNNNNWATGCAAATNNNWNNW	TCTTGTCACTGAAAATTACAATA
M00162	V$OCT1_06	0.800781	8209	(+)	CWNAWTKWSATRYN	GTCACTGAAAATTA
M00195	V$OCT1_Q6	0.821234	8209	(+)	NNNNATGCAAATNAN	GTCACTGAAAATTAC
M00138	V$OCT1_04	0.782518	8212	(-)	NNNNNNNWATGCAAATNNNWNNW	ACTGAAAATTACAATAATGGCTA
M00135	V$OCT1_01	0.768409	8214	(-)	NNNNWTATGCAAATNTNNN	TGAAAATTACAATAATGGC
M00162	V$OCT1_06	0.908984	8216	(-)	CWNAWTKWSATRYN	AAAATTACAATAAT
M00248	V$OCT1_07	0.91308	8218	(-)	TNTATGNTAATT	AATTACAATAAT
M00138	V$OCT1_04	0.801547	8220	(+)	NNNNNNNWATGCAAATNNNWNNW	TTACAATAATGGCTATGCTTCTA
M00138	V$OCT1_04	0.807403	8226	(+)	NNNNNNNWATGCAAATNNNWNNW	TAATGGCTATGCTTCTAAATTGT

M00136	V$OCT1_02	0.798863	8228	(+)	NNGAATATKCANNNN	ATGGCTATGCTTCTA
M00162	V$OCT1_06	0.842187	8243	(+)	CWNAWTKWSATRYN	AATTGTGAAATATA
M00137	V$OCT1_03	0.846701	8251	(+)	NNNRTAATNANNN	AATATAAACATGA
M00162	V$OCT1_06	0.85	8256	(+)	CWNAWTKWSATRYN	AAACATGATATATC
M00162	V$OCT1_06	0.842187	8257	(-)	CWNAWTKWSATRYN	AACATGATATATCC
M00248	V$OCT1_07	0.797329	8290	(+)	TNTATGNTAATT	TCTATGTTAAAG
M00162	V$OCT1_06	0.801172	8303	(+)	CWNAWTKWSATRYN	GGCACTGGAATCCT
M00162	V$OCT1_06	0.813281	8311	(+)	CWNAWTKWSATRYN	AATCCTTTGCTATT
M00138	V$OCT1_04	0.786491	8341	(+)	NNNNNNNWATGCAAATNNNWNNW	AAAAGAGAAGGCATACAAAGTGA
M00162	V$OCT1_06	0.812109	8386	(+)	CWNAWTKWSATRYN	CAGAACTTCATCCA
M00137	V$OCT1_03	0.841565	8396	(-)	NNNRTAATNANNN	TCCAGTTTACAAA
M00138	V$OCT1_04	0.788791	8455	(-)	NNNNNNNWATGCAAATNNNWNNW	TCCAACATTCTGAATAACTTCCC
M00136	V$OCT1_02	0.773146	8461	(-)	NNGAATATKCANNNN	ATTCTGAATAACTTC
M00136	V$OCT1_02	0.782079	8503	(+)	NNGAATATKCANNNN	GAGAACATGTACTTT
M00162	V$OCT1_06	0.800391	8511	(+)	CWNAWTKWSATRYN	GTACTTTGATTGTT
M00138	V$OCT1_04	0.7821	8523	(+)	NNNNNNNWATGCAAATNNNWNNW	TTCATCTTATCTAAATTTACCCA
M00138	V$OCT1_04	0.794228	8524	(-)	NNNNNNNWATGCAAATNNNWNNW	TCATCTTATCTAAATTTACCCAG
M00135	V$OCT1_01	0.769172	8525	(+)	NNNNWTATGCAAATNTNNN	CATCTTATCTAAATTTACC
M00162	V$OCT1_06	0.852734	8528	(+)	CWNAWTKWSATRYN	CTTATCTAAATTTA
M00162	V$OCT1_06	0.804297	8528	(-)	CWNAWTKWSATRYN	CTTATCTAAATTTA
M00248	V$OCT1_07	0.822769	8528	(+)	TNTATGNTAATT	CTTATCTAAATT
M00138	V$OCT1_04	0.780845	8530	(-)	NNNNNNNWATGCAAATNNNWNNW	TATCTAAATTTACCCAGGAATTT
M00137	V$OCT1_03	0.849467	8562	(-)	NNNRTAATNANNN	ATCAGTTTACCAC
M00138	V$OCT1_04	0.84609	8593	(-)	NNNNNNNWATGCAAATNNNWNNW	CTGGTTAATTAGCATTTTTATCT
M00135	V$OCT1_01	0.842617	8595	(-)	NNNNWTATGCAAATNTNNN	GGTTAATTAGCATTTTTAT
M00161	V$OCT1_05	0.896231	8597	(+)	MKNATTTGCATAYY	TTAATTAGCATTTT
M00195	V$OCT1_Q6	0.877456	8597	(-)	NNNNATGCAAATNAN	TTAATTAGCATTTTT
M00136	V$OCT1_02	0.796968	8599	(-)	NNGAATATKCANNNN	AATTAGCATTTTTAT
M00248	V$OCT1_07	0.921772	8599	(-)	TNTATGNTAATT	AATTAGCATTTT
M00138	V$OCT1_04	0.806148	8606	(-)	NNNNNNNWATGCAAATNNNWNNW	ATTTTTATCTTGAATGTAAATTA
M00162	V$OCT1_06	0.821484	8610	(+)	CWNAWTKWSATRYN	TTATCTTGAATGTA
M00138	V$OCT1_04	0.814513	8611	(+)	NNNNNNNWATGCAAATNNNWNNW	TATCTTGAATGTAAATTAATGTC
M00136	V$OCT1_02	0.779643	8612	(-)	NNGAATATKCANNNN	ATCTTGAATGTAAAT

M00135	V$OCT1_01	0.750668	8613	(+)	NNNNWTATGCAAATNTNNN	TCTTGAATGTAAATTAATG
M00195	V$OCT1_Q6	0.84798	8615	(+)	NNNNATGCAAATNAN	TTGAATGTAAATTAA
M00162	V$OCT1_06	0.88125	8616	(-)	CWNAWTKWSATRYN	TGAATGTAAATTAA
M00248	V$OCT1_07	0.9152	8616	(+)	TNTATGNTAATT	TGAATGTAAATT
M00138	V$OCT1_04	0.787537	8617	(+)	NNNNNNNWATGCAAATNNNWNNW	GAATGTAAATTAATGTCATAAAA
M00162	V$OCT1_06	0.873437	8625	(+)	CWNAWTKWSATRYN	ATTAATGTCATAAA
M00162	V$OCT1_06	0.855078	8642	(-)	CWNAWTKWSATRYN	AACAATACATTTTT
M00135	V$OCT1_01	0.742083	8657	(+)	NNNNWTATGCAAATNTNNN	TTTTTAAGGAAATTGTTC
M00248	V$OCT1_07	0.869197	8660	(+)	TNTATGNTAATT	TTTAAGGAAATT
M00136	V$OCT1_02	0.842177	8693	(+)	NNGAATATKCANNNN	AGATATATTCACTAA
M00162	V$OCT1_06	0.800391	8696	(-)	CWNAWTKWSATRYN	TATATTCACTAAAT
M00162	V$OCT1_06	0.808203	8699	(+)	CWNAWTKWSATRYN	ATTCACTAAATTTA
M00138	V$OCT1_04	0.781054	8701	(+)	NNNNNNNWATGCAAATNNNWNNW	TCACTAAATTTAAAATATATTAA
M00138	V$OCT1_04	0.81033	8701	(-)	NNNNNNNWATGCAAATNNNWNNW	TCACTAAATTTAAAATATATTAA
M00138	V$OCT1_04	0.783145	8702	(-)	NNNNNNNWATGCAAATNNNWNNW	CACTAAATTTAAAATATATTAAC
M00135	V$OCT1_01	0.730446	8704	(-)	NNNNWTATGCAAATNTNNN	CTAAATTTAAAATATATTA
M00162	V$OCT1_06	0.865625	8704	(+)	CWNAWTKWSATRYN	CTAAATTTAAAATA
M00162	V$OCT1_06	0.890625	8705	(-)	CWNAWTKWSATRYN	TAAATTTAAAATAT
M00162	V$OCT1_06	0.834375	8706	(+)	CWNAWTKWSATRYN	AAATTTAAAATATA
M00248	V$OCT1_07	0.831673	8708	(-)	TNTATGNTAATT	ATTTAAAATATA
M00138	V$OCT1_04	0.802593	8712	(-)	NNNNNNNWATGCAAATNNNWNNW	AAAATATATTAACATCATCCAGG
M00135	V$OCT1_01	0.779092	8714	(-)	NNNNWTATGCAAATNTNNN	AATATATTAACATCATCCA
M00162	V$OCT1_06	0.81875	8716	(-)	CWNAWTKWSATRYN	TATATTAACATCAT
M00162	V$OCT1_06	0.834375	8734	(-)	CWNAWTKWSATRYN	GATATCTTAAATTT
M00162	V$OCT1_06	0.877734	8735	(+)	CWNAWTKWSATRYN	ATATCTTAAATTTT
M00138	V$OCT1_04	0.816395	8736	(+)	NNNNNNNWATGCAAATNNNWNNW	TATCTTAAATTTTAATTAAACTT
M00136	V$OCT1_02	0.772604	8737	(-)	NNGAATATKCANNNN	ATCTTAAATTTTAAT
M00138	V$OCT1_04	0.841489	8737	(-)	NNNNNNNWATGCAAATNNNWNNW	ATCTTAAATTTTAATTAAACTTT
M00138	V$OCT1_04	0.801966	8738	(-)	NNNNNNNWATGCAAATNNNWNNW	TCTTAAATTTTAATTAAACTTTT
M00248	V$OCT1_07	0.875556	8741	(+)	TNTATGNTAATT	TAAATTTTAATT
M00138	V$OCT1_04	0.789	8742	(+)	NNNNNNNWATGCAAATNNNWNNW	AAATTTTAATTAAACTTTTTATT
M00138	V$OCT1_04	0.820159	8743	(-)	NNNNNNNWATGCAAATNNNWNNW	AATTTTAATTAAACTTTTTATTT
M00138	V$OCT1_04	0.797365	8748	(+)	NNNNNNNWATGCAAATNNNWNNW	TAATTAAACTTTTTATTTTTGA

M00162	V$OCT1_06	0.834375	8753	(+)	CWNAWTKWSATRYN	AAACTTTTTATTTT
M00138	V$OCT1_04	0.859264	8758	(-)	NNNNNNNWATGCAAATNNNWNNW	TTTTATTTTTTGAATTTTTTAGC
M00162	V$OCT1_06	0.869922	8762	(+)	CWNAWTKWSATRYN	ATTTTTTGAATTTT
M00195	V$OCT1_Q6	0.803221	8762	(-)	NNNNATGCAAATNAN	ATTTTTTGAATTTTT
M00136	V$OCT1_02	0.814293	8764	(-)	NNGAATATKCANNNN	TTTTTGAATTTTTTA
M00138	V$OCT1_04	0.835425	8768	(-)	NNNNNNNWATGCAAATNNNWNNW	TGAATTTTTTAGCAGAAGTACAT
M00162	V$OCT1_06	0.876172	8780	(+)	CWNAWTKWSATRYN	CAGAAGTACATTCA
M00136	V$OCT1_02	0.794261	8783	(+)	NNGAATATKCANNNN	AAGTACATTCAAAAG
M00138	V$OCT1_04	0.791301	8832	(-)	NNNNNNNWATGCAAATNNNWNNW	TGGTAAAATTTTATTAAAAGTTA
M00138	V$OCT1_04	0.842953	8836	(+)	NNNNNNNWATGCAAATNNNWNNW	AAAATTTTATTAAAAGTTAATCA
M00137	V$OCT1_03	0.852232	8839	(-)	NNNRTAATNANNN	ATTTTATTAAAAG
M00138	V$OCT1_04	0.790255	8907	(-)	NNNNNNNWATGCAAATNNNWNNW	TCTCTCCTTTTCAATATCTGCTA
M00135	V$OCT1_01	0.732163	8918	(+)	NNNNWTATGCAAATNTNNN	CAATATCTGCTAAACGTGA
M00162	V$OCT1_06	0.862891	8987	(+)	CWNAWTKWSATRYN	AAAACTGACAAGTT
M00162	V$OCT1_06	0.821094	8988	(-)	CWNAWTKWSATRYN	AAACTGACAAGTTT
M00162	V$OCT1_06	0.800781	8997	(+)	CWNAWTKWSATRYN	AGTTTTTTAATCTC
M00162	V$OCT1_06	0.813672	9003	(-)	CWNAWTKWSATRYN	TTAATCTCAGATAT
M00162	V$OCT1_06	0.877734	9011	(+)	CWNAWTKWSATRYN	AGATATTTAATTCA
M00195	V$OCT1_Q6	0.790666	9011	(+)	NNNNATGCAAATNAN	AGATATTTAATTCAT
M00162	V$OCT1_06	0.911719	9012	(-)	CWNAWTKWSATRYN	GATATTTAATTCAT
M00162	V$OCT1_06	0.833984	9015	(+)	CWNAWTKWSATRYN	ATTTAATTCATACT
M00136	V$OCT1_02	0.773416	9017	(-)	NNGAATATKCANNNN	TTAATTCATACTCTC
M00162	V$OCT1_06	0.860937	9023	(+)	CWNAWTKWSATRYN	CATACTCTCATTTG
M00138	V$OCT1_04	0.858637	9025	(-)	NNNNNNNWATGCAAATNNNWNNW	TACTCTCATTTGAATACTTGTGT
M00135	V$OCT1_01	0.819916	9027	(-)	NNNNWTATGCAAATNTNNN	CTCTCATTTGAATACTTGT
M00161	V$OCT1_05	0.905363	9029	(+)	MKNATTTGCATAYY	CTCATTTGAATACT
M00162	V$OCT1_06	0.922266	9029	(+)	CWNAWTKWSATRYN	CTCATTTGAATACT
M00195	V$OCT1_Q6	0.882642	9029	(-)	NNNNATGCAAATNAN	CTCATTTGAATACTT
M00136	V$OCT1_02	0.815918	9031	(-)	NNGAATATKCANNNN	CATTTGAATACTTGT
M00248	V$OCT1_07	0.800085	9031	(-)	TNTATGNTAATT	CATTTGAATACT
M00138	V$OCT1_04	0.787746	9064	(-)	NNNNNNNWATGCAAATNNNWNNW	AGATCTGATTAGCACAGTATTGA
M00137	V$OCT1_03	0.919399	9066	(-)	NNNRTAATNANNN	ATCTGATTAGCAC
M00195	V$OCT1_Q6	0.820415	9068	(-)	NNNNATGCAAATNAN	CTGATTAGCACAGTA

M00135	V$OCT1_01	0.751431	9076	(+)	NNNNWTATGCAAATNTNNN	CACAGTATTGAAATGGCAC
M00162	V$OCT1_06	0.834766	9079	(+)	CWNAWTKWSATRYN	AGTATTGAAATGGC
M00248	V$OCT1_07	0.796057	9079	(+)	TNTATGNTAATT	AGTATTGAAATG
M00162	V$OCT1_06	0.801172	9109	(+)	CWNAWTKWSATRYN	CAGACTTGAGTTTG
M00162	V$OCT1_06	0.916797	9144	(+)	CWNAWTKWSATRYN	CGGCATGTGATATT
M00162	V$OCT1_06	0.801562	9145	(-)	CWNAWTKWSATRYN	GGCATGTGATATTA
M00162	V$OCT1_06	0.808594	9152	(-)	CWNAWTKWSATRYN	GATATTAGATAGTT
M00162	V$OCT1_06	0.813672	9173	(+)	CWNAWTKWSATRYN	CTGCTGTAAATTTA
M00162	V$OCT1_06	0.800781	9186	(+)	CWNAWTKWSATRYN	ATAACTTAAGTTCC
M00162	V$OCT1_06	0.800781	9192	(-)	CWNAWTKWSATRYN	TAAGTTCCAAAGTG
M00138	V$OCT1_04	0.794856	9204	(-)	NNNNNNNWATGCAAATNNNNWNNW	TGACAAAATAGTAATAATATTTA
M00137	V$OCT1_03	0.913868	9211	(+)	NNNRTAATNANNN	ATAGTAATAATAT
M00137	V$OCT1_03	0.843935	9214	(+)	NNNRTAATNANNN	GTAATAATATTTA
M00136	V$OCT1_02	0.850568	9216	(+)	NNGAATATKCANNNN	AATAATATTTAAGCA
M00138	V$OCT1_04	0.824341	9218	(+)	NNNNNNNWATGCAAATNNNNWNNW	TAATATTTAAGCATATAAAGCAA
M00135	V$OCT1_01	0.761923	9219	(-)	NNNNWTATGCAAATNTNNN	AATATTTAAGCATATAAAG
M00138	V$OCT1_04	0.783772	9222	(+)	NNNNNNNWATGCAAATNNNNWNNW	ATTTAAGCATATAAAGCAATTAA
M00136	V$OCT1_02	0.798592	9223	(-)	NNGAATATKCANNNN	TTTAAGCATATAAAG
M00195	V$OCT1_Q6	0.813319	9226	(+)	NNNNATGCAAATNAN	AAGCATATAAAGCAA
M00162	V$OCT1_06	0.862891	9249	(-)	CWNAWTKWSATRYN	AGCAAGACATAGTG
M00195	V$OCT1_Q6	0.805131	9256	(+)	NNNNATGCAAATNAN	CATAGTGCAAAGTAA
M00162	V$OCT1_06	0.81875	9290	(-)	CWNAWTKWSATRYN	AGTATCAGAAACAT
M00162	V$OCT1_06	0.829297	9314	(-)	CWNAWTKWSATRYN	CTCATCCAATATTC
M00136	V$OCT1_02	0.77098	9315	(-)	NNGAATATKCANNNN	TCATCCAATATTCTG
M00135	V$OCT1_01	0.767837	9318	(+)	NNNNWTATGCAAATNTNNN	TCCAATATTCTGATTTAAT
M00136	V$OCT1_02	0.807255	9318	(+)	NNGAATATKCANNNN	TCCAATATTCTGATT
M00162	V$OCT1_06	0.852734	9321	(+)	CWNAWTKWSATRYN	AATATTCTGATTTA
M00248	V$OCT1_07	0.819165	9321	(+)	TNTATGNTAATT	AATATTCTGATT
M00138	V$OCT1_04	0.873693	9328	(-)	NNNNNNNWATGCAAATNNNNWNNW	TGATTTAATTTGCAAAAATCAGA
M00138	V$OCT1_04	0.80322	9329	(+)	NNNNNNNWATGCAAATNNNNWNNW	GATTTAATTTGCAAAAATCAGAA
M00135	V$OCT1_01	0.819916	9330	(-)	NNNNWTATGCAAATNTNNN	ATTTAATTTGCAAAAATCA
M00135	V$OCT1_01	0.830408	9331	(+)	NNNNWTATGCAAATNTNNN	TTTAATTTGCAAAAATCAG
M00136	V$OCT1_02	0.79372	9331	(+)	NNGAATATKCANNNN	TTTAATTTGCAAAAA

M00195	V$OCT1_Q6	0.842795	9332	(-)	NNNNATGCAAATNAN	TTAATTTGCAAAAAT
M00162	V$OCT1_06	0.821094	9334	(-)	CWNAWTKWSATRYN	AATTTGCAAAAATC
M00248	V$OCT1_07	0.873225	9334	(-)	TNTATGNTAATT	AATTTGCAAAAA
M00162	V$OCT1_06	0.821484	9349	(-)	CWNAWTKWSATRYN	GAAAACAAATGGTG
M00137	V$OCT1_03	0.844725	9366	(+)	NNNRTAATNANNN	GTGGTAAAGAGCC
M00135	V$OCT1_01	0.743609	9469	(-)	NNNNWTATGCAAATNTNNN	TTGAGAATTCCATAGACAA
M00162	V$OCT1_06	0.864844	9472	(-)	CWNAWTKWSATRYN	AGAATTCCATAGAC
M00162	V$OCT1_06	0.839844	9512	(-)	CWNAWTKWSATRYN	AGGATCACAGAGAG
M00162	V$OCT1_06	0.826562	9550	(-)	CWNAWTKWSATRYN	CACAGCACAGAAAG
M00135	V$OCT1_01	0.7686	9555	(+)	NNNNWTATGCAAATNTNNN	CACAGAAAGCAAATCTGTT
M00195	V$OCT1_Q6	0.81441	9557	(+)	NNNNATGCAAATNAN	CAGAAAGCAAATCTG
M00162	V$OCT1_06	0.847656	9558	(-)	CWNAWTKWSATRYN	AGAAAGCAAATCTG
M00162	V$OCT1_06	0.826953	9569	(+)	CWNAWTKWSATRYN	CTGTTTTCAATTTT
M00162	V$OCT1_06	0.834375	9570	(-)	CWNAWTKWSATRYN	TGTTTTCAATTTTC
M00136	V$OCT1_02	0.787764	9595	(+)	NNGAATATKCANNNN	GATATAATGCACACT
M00135	V$OCT1_01	0.765548	9606	(+)	NNNNWTATGCAAATNTNNN	CACTGTAAGCAAAACTGTC
M00162	V$OCT1_06	0.834375	9609	(-)	CWNAWTKWSATRYN	TGTAAGCAAAACTG
M00162	V$OCT1_06	0.829297	9650	(+)	CWNAWTKWSATRYN	CACTCAGTCATGTC
M00137	V$OCT1_03	0.850257	9665	(-)	NNNRTAATNANNN	AACTCTTTGTGAC
M00162	V$OCT1_06	0.831641	9667	(+)	CWNAWTKWSATRYN	CTCTTTGTGACACA
M00162	V$OCT1_06	0.834375	9732	(+)	CWNAWTKWSATRYN	CATTCAGTGATGCC
M00195	V$OCT1_Q6	0.797216	9826	(+)	NNNNATGCAAATNAN	AGTTCTTCAAATCAG
M00136	V$OCT1_02	0.923389	9876	(-)	NNGAATATKCANNNN	CCAGTGAATATTCAG
M00136	V$OCT1_02	0.934218	9879	(+)	NNGAATATKCANNNN	GTGAATATTCAGGAC
M00162	V$OCT1_06	0.800781	9882	(-)	CWNAWTKWSATRYN	AATATTCAGGACTG
M00136	V$OCT1_02	0.858148	9910	(-)	NNGAATATKCANNNN	TGACTGAATAATTAT
M00137	V$OCT1_03	0.905571	9914	(+)	NNNRTAATNANNN	TGAATAATTATTT
M00138	V$OCT1_04	0.784818	9914	(-)	NNNNNNNWATGCAAATNNNWNNW	TGAATAATTATTTATATCTGACT
M00137	V$OCT1_03	0.854208	9915	(-)	NNNRTAATNANNN	GAATAATTATTTA
M00138	V$OCT1_04	0.838352	9915	(+)	NNNNNNNWATGCAAATNNNWNNW	GAATAATTATTTATATCTGACTG
M00138	V$OCT1_04	0.789	9916	(-)	NNNNNNNWATGCAAATNNNWNNW	AATAATTATTTATATCTGACTGC
M00136	V$OCT1_02	0.770439	9917	(+)	NNGAATATKCANNNN	ATAATTATTTATATC
M00136	V$OCT1_02	0.802382	9932	(-)	NNGAATATKCANNNN	TGACTGCATTATAAT

M00138	V$OCT1_04	0.810958	9932	(-)	NNNNNNNWATGCAAATNNNWNNW	TGACTGCATTATAATATATTTTA
M00162	V$OCT1_06	0.804297	9936	(-)	CWNAWTKWSATRYN	TGCATTATAATATA
M00162	V$OCT1_06	0.812109	9938	(+)	CWNAWTKWSATRYN	CATTATAATATATT
M00138	V$OCT1_04	0.81033	9941	(+)	NNNNNNNWATGCAAATNNNWNNW	TATAATATATTTTAAGTTAAAAA
M00138	V$OCT1_04	0.782727	9942	(-)	NNNNNNNWATGCAAATNNNWNNW	ATAATATATTTTAAGTTAAAAAA
M00138	V$OCT1_04	0.802593	9943	(-)	NNNNNNNWATGCAAATNNNWNNW	TAATATATTTTAAGTTAAAAAAA
M00248	V$OCT1_07	0.831673	9946	(+)	TNTATGNTAATT	TATATTTTAAGT
M00138	V$OCT1_04	0.788582	9947	(+)	NNNNNNNWATGCAAATNNNWNNW	ATATTTTAAGTTAAAAAAAATGC
M00138	V$OCT1_04	0.787746	9958	(+)	NNNNNNNWATGCAAATNNNWNNW	TAAAAAAAATGCAACACAGTTGG
M00136	V$OCT1_02	0.818896	9960	(+)	NNGAATATKCANNNN	AAAAAAATGCAACAC
M00195	V$OCT1_Q6	0.795033	9962	(+)	NNNNATGCAAATNAN	AAAAATGCAACACAG
M00162	V$OCT1_06	0.834766	9963	(-)	CWNAWTKWSATRYN	AAAATGCAACACAG
M00162	V$OCT1_06	0.813672	9979	(+)	CWNAWTKWSATRYN	GGTTTTCTAATGTT
M00137	V$OCT1_03	0.845516	9982	(+)	NNNRTAATNANNN	TTTCTAATGTTTT
M00138	V$OCT1_04	0.784191	9986	(+)	NNNNNNNWATGCAAATNNNWNNW	TAATGTTTTTTTAAAAAAAGAAT
M00138	V$OCT1_04	0.80092	10005	(+)	NNNNNNNWATGCAAATNNNWNNW	GAATGATTGGGCTAATCCAAATT
M00138	V$OCT1_04	0.807194	10011	(+)	NNNNNNNWATGCAAATNNNWNNW	TTGGGCTAATCCAAATTCTCTGT
M00135	V$OCT1_01	0.758108	10013	(+)	NNNNWTATGCAAATNTNNN	GGGCTAATCCAAATTCTCT
M00195	V$OCT1_Q6	0.793668	10015	(+)	NNNNATGCAAATNAN	GCTAATCCAAATTCT
M00162	V$OCT1_06	0.80625	10016	(-)	CWNAWTKWSATRYN	CTAATCCAAATTCT
M00162	V$OCT1_06	0.852734	10043	(-)	CWNAWTKWSATRYN	AACACTAAAGTAAG
M00137	V$OCT1_03	0.843935	10052	(-)	NNNRTAATNANNN	GTAAGATTATTAG
M00162	V$OCT1_06	0.816016	10056	(+)	CWNAWTKWSATRYN	GATTATTAGTTTCC
M00162	V$OCT1_06	0.834375	10063	(-)	CWNAWTKWSATRYN	AGTTTCCCATTCTT
M00162	V$OCT1_06	0.800781	10084	(+)	CWNAWTKWSATRYN	GGGAAAGGGATACC
M00138	V$OCT1_04	0.792346	10131	(-)	NNNNNNNWATGCAAATNNNWNNW	TTTTTCTCTTTGAATAGGAACAG
M00136	V$OCT1_02	0.797239	10137	(-)	NNGAATATKCANNNN	TCTTTGAATAGGAAC
M00162	V$OCT1_06	0.842187	10152	(-)	CWNAWTKWSATRYN	AGCTTCTCAGACTG
M00162	V$OCT1_06	0.834375	10184	(+)	CWNAWTKWSATRYN	CAGCTGGTAATGTT
M00137	V$OCT1_03	0.90004	10187	(+)	NNNRTAATNANNN	CTGGTAATGTTTT
M00138	V$OCT1_04	0.814095	10192	(+)	NNNNNNNWATGCAAATNNNWNNW	AATGTTTTATTATAATAATATAA

M00138	V$OCT1_04	0.825178	10193	(-)	NNNNNNNWATGCAAATNNNNWNNW	ATGTTTTATTATAATAATATAAA
M00137	V$OCT1_03	0.854998	10195	(-)	NNNRTAATNANNN	GTTTTATTATAAT
M00138	V$OCT1_04	0.820159	10195	(+)	NNNNNNNWATGCAAATNNNNWNNW	GTTTTATTATAATAATATAAAAT
M00138	V$OCT1_04	0.814722	10196	(-)	NNNNNNNWATGCAAATNNNNWNNW	TTTTATTATAATAATATAAAATT
M00135	V$OCT1_01	0.746089	10197	(+)	NNNNWTATGCAAATNTNNN	TTTATTATAATAATATAAA
M00137	V$OCT1_03	0.910707	10200	(+)	NNNRTAATNANNN	ATTATAATAATAT
M00138	V$OCT1_04	0.81054	10201	(+)	NNNNNNNWATGCAAATNNNNWNNW	TTATAATAATATAAAATTAAGTC
M00162	V$OCT1_06	0.899609	10208	(-)	CWNAWTKWSATRYN	AATATAAAATTAAG
M00138	V$OCT1_04	0.808239	10223	(-)	NNNNNNNWATGCAAATNNNNWNNW	CTACAGAATTAAAATAATTAAGT
M00135	V$OCT1_01	0.782335	10225	(-)	NNNNWTATGCAAATNTNNN	ACAGAATTAAAATAATTAA
M00195	V$OCT1_Q6	0.794214	10226	(+)	NNNNATGCAAATNAN	CAGAATTAAAATAAT
M00162	V$OCT1_06	0.908984	10227	(-)	CWNAWTKWSATRYN	AGAATTAAAATAAT
M00248	V$OCT1_07	0.910324	10229	(-)	TNTATGNTAATT	AATTAAAATAAT
M00137	V$OCT1_03	0.876333	10233	(+)	NNNRTAATNANNN	AAAATAATTAAGT
M00137	V$OCT1_03	0.86448	10234	(-)	NNNRTAATNANNN	AAATAATTAAGTG
M00162	V$OCT1_06	0.847266	10241	(-)	CWNAWTKWSATRYN	TAAGTGAAATTTAC
M00138	V$OCT1_04	0.800711	10247	(-)	NNNNNNNWATGCAAATNNNNWNNW	AAATTTACTTTGACTAAATTCTA
M00248	V$OCT1_07	0.817257	10248	(-)	TNTATGNTAATT	AATTTACTTTGA
M00162	V$OCT1_06	0.901953	10268	(-)	CWNAWTKWSATRYN	TACATCAAATCATG
M00137	V$OCT1_03	0.848281	10298	(-)	NNNRTAATNANNN	GATTCATTATATT
M00162	V$OCT1_06	0.84218	10299	(+)	CWNAWTKWSATRYN	ATTCATTATATTCT

		7				
M00162	V$OCT1_06	0.804687	10300	(-)	CWNAWTKWSATRYN	TTCATTATATTCTG
M00162	V$OCT1_06	0.808594	10307	(+)	CWNAWTKWSATRYN	TATTCTGGAATTTA
M00162	V$OCT1_06	0.800781	10323	(+)	CWNAWTKWSATRYN	CTGTATTTATTTTC
M00138	V$OCT1_04	0.784818	10324	(-)	NNNNNNNWATGCAAATNNNWNNW	TGTATTTATTTTCCCAAATCCAG
M00162	V$OCT1_06	0.834375	10324	(-)	CWNAWTKWSATRYN	TGTATTTATTTTCC
M00162	V$OCT1_06	0.834766	10338	(-)	CWNAWTKWSATRYN	CAAATCCAGTTCTG
M00138	V$OCT1_04	0.781681	10361	(-)	NNNNNNNWATGCAAATNNNWNNW	TCCATACCTATGCCTCCTTGTGA
M00137	V$OCT1_03	0.864085	10390	(-)	NNNRTAATNANNN	AGATCATTGCCAA
M00138	V$OCT1_04	0.789	10396	(-)	NNNNNNNWATGCAAATNNNWNNW	TTGCCAAATAAACATTCCATTTA
M00248	V$OCT1_07	0.808141	10402	(-)	TNTATGNTAATT	AATAAACATTCC
M00136	V$OCT1_02	0.778831	10403	(+)	NNGAATATKCANNNN	ATAAACATTCCATTT
M00162	V$OCT1_06	0.85	10405	(+)	CWNAWTKWSATRYN	AAACATTCCATTTA
M00162	V$OCT1_06	0.868359	10406	(-)	CWNAWTKWSATRYN	AACATTCCATTTAA
M00138	V$OCT1_04	0.786073	10407	(-)	NNNNNNNWATGCAAATNNNWNNW	ACATTCCATTTAAAAAACAATGT
M00162	V$OCT1_06	0.834375	10411	(-)	CWNAWTKWSATRYN	TCCATTTAAAAAAC
M00162	V$OCT1_06	0.808594	10450	(-)	CWNAWTKWSATRYN	GGGATCCCATACAT
M00135	V$OCT1_01	0.770126	10456	(+)	NNNNWTATGCAAATNTNNN	CCATACATTCAAATTGCTT
M00195	V$OCT1_Q6	0.845524	10458	(+)	NNNNATGCAAATNAN	ATACATTCAAATTGC
M00162	V$OCT1_06	0.847656	10459	(-)	CWNAWTKWSATRYN	TACATTCAAATTGC
M00248	V$OCT1_07	0.858385	10459	(+)	TNTATGNTAATT	TACATTCAAATT

M00162	V$OCT1_06	0.873437	10474	(-)	CWNAWTKWSATRYN	TTCATCAAAATTTT
M00162	V$OCT1_06	0.834375	10495	(+)	CWNAWTKWSATRYN	AGAATTGGCAGGTT
M00137	V$OCT1_03	0.883445	10514	(-)	NNNRTAATNANNN	ATCTCATTGTCTC
M00138	V$OCT1_04	0.807821	10563	(+)	NNNNNNNWATGCAAATNNNWNNW	ACGTTGTTTTGAATATTGAATGA
M00135	V$OCT1_01	0.780046	10564	(-)	NNNNWTATGCAAATNTNNN	CGTTGTTTTGAATATTGAA
M00162	V$OCT1_06	0.813672	10566	(+)	CWNAWTKWSATRYN	TTGTTTTGAATATT
M00136	V$OCT1_02	0.890363	10568	(-)	NNGAATATKCANNNN	GTTTTGAATATTGAA
M00136	V$OCT1_02	0.817001	10571	(+)	NNGAATATKCANNNN	TTGAATATTGAATGA
M00162	V$OCT1_06	0.816406	10573	(+)	CWNAWTKWSATRYN	GAATATTGAATGAG
M00138	V$OCT1_04	0.783145	10619	(+)	NNNNNNNWATGCAAATNNNWNNW	TTGTGGCAAGTCCAATAAAATGT
M00162	V$OCT1_06	0.821875	10674	(+)	CWNAWTKWSATRYN	CTCTATAAAATTTG
M00137	V$OCT1_03	0.895693	10708	(-)	NNNRTAATNANNN	ACAATATTATGAA
M00138	V$OCT1_04	0.782727	10708	(+)	NNNNNNNWATGCAAATNNNWNNW	ACAATATTATGAAGGTTTTTTTT
M00137	V$OCT1_03	0.84354	10726	(+)	NNNRTAATNANNN	TTTTTAATCACAG
M00138	V$OCT1_04	0.84609	10755	(-)	NNNNNNNWATGCAAATNNNWNNW	CTGGTTAATTAGCATTTTTATCT
M00135	V$OCT1_01	0.842617	10757	(-)	NNNNWTATGCAAATNTNNN	GGTTAATTAGCATTTTTAT
M00161	V$OCT1_05	0.896231	10759	(+)	MKNATTTGCATAYY	TTAATTAGCATTTT
M00195	V$OCT1_Q6	0.877456	10759	(-)	NNNNATGCAAATNAN	TTAATTAGCATTTTT
M00136	V$OCT1_02	0.796968	10761	(-)	NNGAATATKCANNNN	AATTAGCATTTTTAT
M00248	V$OCT1_07	0.921772	10761	(-)	TNTATGNTAATT	AATTAGCATTTT
M00138	V$OCT1_04	0.80614	10768	(-)	NNNNNNNWATGCAAATNNNW	ATTTTTATCTTGAATGTAAATTA

		8			NNW	
M00162	V$OCT1_06	0.821484	10772	(+)	CWNAWTKWSATRYN	TTATCTTGAATGTA
M00138	V$OCT1_04	0.814513	10773	(+)	NNNNNNNWATGCAAATNNNWNNW	TATCTTGAATGTAAATTAATGTC
M00136	V$OCT1_02	0.779643	10774	(-)	NNGAATATKCANNNN	ATCTTGAATGTAAAT
M00135	V$OCT1_01	0.750668	10775	(+)	NNNNWTATGCAAATNTNNN	TCTTGAATGTAAATTAATG
M00195	V$OCT1_Q6	0.84798	10777	(+)	NNNNATGCAAATNAN	TTGAATGTAAATTAA
M00162	V$OCT1_06	0.88125	10778	(-)	CWNAWTKWSATRYN	TGAATGTAAATTAA
M00248	V$OCT1_07	0.9152	10778	(+)	TNTATGNTAATT	TGAATGTAAATT
M00138	V$OCT1_04	0.787537	10779	(+)	NNNNNNNWATGCAAATNNNWNNW	GAATGTAAATTAATGTCATAAAA
M00162	V$OCT1_06	0.873437	10787	(+)	CWNAWTKWSATRYN	ATTAATGTCATAAA
M00162	V$OCT1_06	0.801172	10803	(+)	CWNAWTKWSATRYN	TAATAATACATGTT
M00162	V$OCT1_06	0.821094	10804	(-)	CWNAWTKWSATRYN	AATAATACATGTTT
M00135	V$OCT1_01	0.742083	10823	(+)	NNNNWTATGCAAATNTNNN	TTTTTTAAGGAAATTGTTC
M00248	V$OCT1_07	0.869197	10826	(+)	TNTATGNTAATT	TTTAAGGAAATT
M00137	V$OCT1_03	0.874753	10861	(-)	NNNRTAATNANNN	ATATATTTACTAA
M00162	V$OCT1_06	0.821094	10862	(-)	CWNAWTKWSATRYN	TATATTTACTAAAT
M00162	V$OCT1_06	0.808203	10865	(+)	CWNAWTKWSATRYN	ATTTACTAAATTTA
M00138	V$OCT1_04	0.802802	10866	(+)	NNNNNNNWATGCAAATNNNWNNW	TTTACTAAATTTAAAATATATTA
M00138	V$OCT1_04	0.784191	10867	(+)	NNNNNNNWATGCAAATNNNWNNW	TTACTAAATTTAAAATATATTAA
M00138	V$OCT1_04	0.790046	10867	(-)	NNNNNNNWATGCAAATNNNWNNW	TTACTAAATTTAAAATATATTAA
M00138	V$OCT1_04	0.810958	10868	(-)	NNNNNNNWATGCAAATNNNWNNW	TACTAAATTTAAAATATATTAAA
M00135	V$OCT1_01	0.73044	10870	(-)	NNNNWTATGCAAATNTNNN	CTAAATTTAAAATATATTA

		6				
M00162	V$OCT1_06	0.86562 5	10870	(+)	CWNAWTKWSATRYN	CTAAATTTAAAATA
M00162	V$OCT1_06	0.89062 5	10871	(-)	CWNAWTKWSATRYN	TAAATTTAAAATAT
M00162	V$OCT1_06	0.83437 5	10872	(+)	CWNAWTKWSATRYN	AAATTTAAAATATA
M00248	V$OCT1_07	0.83167 3	10874	(-)	TNTATGNTAATT	ATTTAAAATATA
M00162	V$OCT1_06	0.91914 1	10881	(+)	CWNAWTKWSATRYN	ATATATTAAATGCA
M00162	V$OCT1_06	0.89062 5	10882	(-)	CWNAWTKWSATRYN	TATATTAAATGCAC
M00162	V$OCT1_06	0.86328 1	10893	(+)	CWNAWTKWSATRYN	CACTATAAGATGTG
M00135	V$OCT1_01	0.75486 5	10897	(-)	NNNNWTATGCAAATNTNNN	ATAAGATGTGCATTTGAAC
M00162	V$OCT1_06	0.80859 4	10899	(+)	CWNAWTKWSATRYN	AAGATGTGCATTTG
M00136	V$OCT1_02	0.79290 7	10901	(-)	NNGAATATKCANNNN	GATGTGCATTTGAAC
M00138	V$OCT1_04	0.81765	10961	(-)	NNNNNNNWATGCAAATNNNW NNW	AGTATCTATTAGCACAATCCAGC
M00135	V$OCT1_01	0.80942 4	10963	(-)	NNNNWTATGCAAATNTNNN	TATCTATTAGCACAATCCA
M00138	V$OCT1_04	0.81074 9	10984	(-)	NNNNNNNWATGCAAATNNNW NNW	TTCACTATTTAGCATCAATCCTT
M00135	V$OCT1_01	0.74360 9	10986	(-)	NNNNWTATGCAAATNTNNN	CACTATTTAGCATCAATCC
M00248	V$OCT1_07	0.80199 3	10990	(-)	TNTATGNTAATT	ATTTAGCATCAA
M00138	V$OCT1_04	0.78356 3	10998	(-)	NNNNNNNWATGCAAATNNNW NNW	TCAATCCTTTTGAGTATCATAGT
M00138	V$OCT1_04	0.78314 5	11003	(-)	NNNNNNNWATGCAAATNNNW NNW	CCTTTTGAGTATCATAGTTTTTC
M00162	V$OCT1_06	0.85273 4	11010	(-)	CWNAWTKWSATRYN	AGTATCATAGTTTT
M00162	V$OCT1_06	0.81328 1	11040	(+)	CWNAWTKWSATRYN	AAATTTTTGTTTTA
M00138	V$OCT1_04	0.79109	11049	(+)	NNNNNNNWATGCAAATNNNW	TTTTAGTTATACCAAATAAGCTT

		2			NNW	
M00135	V$OCT1_01	0.796833	11104	(-)	NNNNWTATGCAAATNTNNN	CTTAATTTAGCAAAATTAC
M00135	V$OCT1_01	0.739985	11105	(+)	NNNNWTATGCAAATNTNNN	TTAATTTAGCAAAATTACA
M00135	V$OCT1_01	0.752575	11112	(-)	NNNNWTATGCAAATNTNNN	AGCAAAATTACATTTTCAC
M00162	V$OCT1_06	0.979297	11114	(+)	CWNAWTKWSATRYN	CAAAATTACATTTT
M00195	V$OCT1_Q6	0.803766	11114	(-)	NNNNATGCAAATNAN	CAAAATTACATTTTC
M00162	V$OCT1_06	0.924609	11115	(-)	CWNAWTKWSATRYN	AAAATTACATTTTC
M00136	V$OCT1_02	0.808067	11116	(-)	NNGAATATKCANNNN	AAATTACATTTTCAC
M00138	V$OCT1_04	0.823296	11116	(-)	NNNNNNNWATGCAAATNNNWNNW	AAATTACATTTTCACATTTTTAT
M00248	V$OCT1_07	0.793089	11116	(-)	TNTATGNTAATT	AAATTACATTTT
M00135	V$OCT1_01	0.766501	11118	(-)	NNNNWTATGCAAATNTNNN	ATTACATTTTCACATTTTT
M00162	V$OCT1_06	0.868359	11122	(+)	CWNAWTKWSATRYN	CATTTTCACATTTT
M00162	V$OCT1_06	0.873828	11128	(+)	CWNAWTKWSATRYN	CACATTTTTATTCT
M00137	V$OCT1_03	0.850257	11129	(-)	NNNRTAATNANNN	ACATTTTTATTCT
M00162	V$OCT1_06	0.808594	11161	(-)	CWNAWTKWSATRYN	GGAAGTCAATAATG
M00137	V$OCT1_03	0.888582	11166	(+)	NNNRTAATNANNN	TCAATAATGAATT
M00162	V$OCT1_06	0.821484	11167	(+)	CWNAWTKWSATRYN	CAATAATGAATTTA
M00136	V$OCT1_02	0.789117	11169	(-)	NNGAATATKCANNNN	ATAATGAATTTAATT
M00162	V$OCT1_06	0.891016	11172	(+)	CWNAWTKWSATRYN	ATGAATTTAATTTG
M00162	V$OCT1_06	0.881641	11173	(-)	CWNAWTKWSATRYN	TGAATTTAATTTGG
M00162	V$OCT1_06	0.87343	11209	(-)	CWNAWTKWSATRYN	AGTTTCCAATTTTG

		7				
M00138	V$OCT1_04	0.781263	11246	(+)	NNNNNNNWATGCAAATNNNWNNW	CCAAATAAATTTAGATATATCAG
M00138	V$OCT1_04	0.807612	11247	(-)	NNNNNNNWATGCAAATNNNWNNW	CAAATAAATTTAGATATATCAGT
M00135	V$OCT1_01	0.802366	11249	(-)	NNNNWTATGCAAATNTNNN	AATAAATTTAGATATATCA
M00162	V$OCT1_06	0.894141	11251	(+)	CWNAWTKWSATRYN	TAAATTTAGATATA
M00248	V$OCT1_07	0.867501	11253	(-)	TNTATGNTAATT	AATTTAGATATA
M00162	V$OCT1_06	0.834766	11259	(-)	CWNAWTKWSATRYN	GATATATCAGTACC
M00162	V$OCT1_06	0.860937	11272	(+)	CWNAWTKWSATRYN	CTCAATCAAATTTA
M00162	V$OCT1_06	0.852734	11273	(-)	CWNAWTKWSATRYN	TCAATCAAATTTAT
M00138	V$OCT1_04	0.802384	11275	(-)	NNNNNNNWATGCAAATNNNWNNW	AATCAAATTTATCATTACTAATC
M00138	V$OCT1_04	0.785236	11280	(+)	NNNNNNNWATGCAAATNNNWNNW	AATTTATCATTACTAATCTAAGA
M00137	V$OCT1_03	0.947056	11283	(-)	NNNRTAATNANNN	TTATCATTACTAA
M00162	V$OCT1_06	0.800781	11292	(-)	CWNAWTKWSATRYN	CTAATCTAAGAAAT
M00138	V$OCT1_04	0.816395	11296	(-)	NNNNNNNWATGCAAATNNNWNNW	TCTAAGAAATTACATGAATAATA
M00137	V$OCT1_03	0.840379	11299	(-)	NNNRTAATNANNN	AAGAAATTACATG
M00138	V$OCT1_04	0.797365	11300	(-)	NNNNNNNWATGCAAATNNNWNNW	AGAAATTACATGAATAATATATT
M00162	V$OCT1_06	0.876172	11300	(+)	CWNAWTKWSATRYN	AGAAATTACATGAA
M00138	V$OCT1_04	0.813049	11301	(+)	NNNNNNNWATGCAAATNNNWNNW	GAAATTACATGAATAATATATTT
M00162	V$OCT1_06	0.877734	11301	(-)	CWNAWTKWSATRYN	GAAATTACATGAAT
M00248	V$OCT1_07	0.812381	11302	(-)	TNTATGNTAATT	AAATTACATGAA
M00136	V$OCT1_02	0.86031	11306	(-)	NNGAATATKCANNNN	TACATGAATAATATA

		4				
M00162	V$OCT1_06	0.83398 4	11306	(-)	CWNAWTKWSATRYN	TACATGAATAATAT
M00137	V$OCT1_03	0.91821 4	11340	(-)	NNNRTAATNANNN	ATATGATTAGGCC
M00138	V$OCT1_04	0.79192 8	11419	(-)	NNNNNNNWATGCAAATNNNW NNW	GCTCATAGTATTCATTATTATTT
M00136	V$OCT1_02	0.84136 4	11422	(+)	NNGAATATKCANNNN	CATAGTATTCATTAT
M00137	V$OCT1_03	0.89450 8	11427	(-)	NNNRTAATNANNN	TATTCATTATTAT
M00137	V$OCT1_03	0.89845 9	11430	(-)	NNNRTAATNANNN	TCATTATTATTTA
M00136	V$OCT1_02	0.78749 3	11432	(+)	NNGAATATKCANNNN	ATTATTATTTATTTC
M00162	V$OCT1_06	0.84726 6	11438	(+)	CWNAWTKWSATRYN	ATTTATTTCATAGA
M00162	V$OCT1_06	0.82148 4	11439	(-)	CWNAWTKWSATRYN	TTTATTTCATAGAT
M00138	V$OCT1_04	0.78941 9	11457	(-)	NNNNNNNWATGCAAATNNNW NNW	CCTAGCATTTAGCATAGACCCCC
M00135	V$OCT1_01	0.79320 9	11459	(-)	NNNNWTATGCAAATNTNNN	TAGCATTTAGCATAGACCC
M00248	V$OCT1_07	0.90205 6	11463	(-)	TNTATGNTAATT	ATTTAGCATAGA
M00162	V$OCT1_06	0.89101 6	11482	(-)	CWNAWTKWSATRYN	TGAATGAAAGTGCC
M00162	V$OCT1_06	0.82148 4	11499	(-)	CWNAWTKWSATRYN	CAAATGTCTGTTAT
M00162	V$OCT1_06	0.80078 1	11502	(+)	CWNAWTKWSATRYN	ATGTCTGTTATTCA
M00136	V$OCT1_02	0.77666 5	11505	(+)	NNGAATATKCANNNN	TCTGTTATTCACACA
M00162	V$OCT1_06	0.80585 9	11525	(-)	CWNAWTKWSATRYN	AAAATCAATGTACT
M00137	V$OCT1_03	0.87712 4	11539	(+)	NNNRTAATNANNN	AAGATAAAGAGAT
M00248	V$OCT1_07	0.79520 9	11574	(-)	TNTATGNTAATT	CATGACCATATA
M00138	V$OCT1_04	0.78084	11576	(-)	NNNNNNNWATGCAAATNNNW	TGACCATATAAGACTATTTACTA

		5			NNW	
M00136	V$OCT1_02	0.809962	11585	(+)	NNGAATATKCANNNN	AAGACTATTTACTAA
M00162	V$OCT1_06	0.813281	11597	(-)	CWNAWTKWSATRYN	TAAATCTAGAATAT
M00162	V$OCT1_06	0.860547	11606	(-)	CWNAWTKWSATRYN	AATATCTTAGTATT
M00138	V$OCT1_04	0.784191	11629	(-)	NNNNNNNWATGCAAATNNNWNNW	TGTTGTTTTTATAATGTGTGTTC
M00162	V$OCT1_06	0.813672	11633	(+)	CWNAWTKWSATRYN	GTTTTTATAATGTG
M00137	V$OCT1_03	0.861715	11636	(+)	NNNRTAATNANNN	TTTATAATGTGTG
M00138	V$OCT1_04	0.791719	11653	(-)	NNNNNNNWATGCAAATNNNWNNW	TCTTTCTTTTTTAACAGAAATAA
M00138	V$OCT1_04	0.801338	11655	(-)	NNNNNNNWATGCAAATNNNWNNW	TTTCTTTTTTAACAGAAATAAAT
M00138	V$OCT1_04	0.785027	11659	(-)	NNNNNNNWATGCAAATNNNWNNW	TTTTTTAACAGAAATAAATAAAA
M00162	V$OCT1_06	0.883203	11678	(-)	CWNAWTKWSATRYN	AAAATCCAAGGGAG
M00162	V$OCT1_06	0.855469	11708	(-)	CWNAWTKWSATRYN	CATATTTGATAATG
M00137	V$OCT1_03	0.888977	11713	(+)	NNNRTAATNANNN	TTGATAATGTTTT
M00138	V$OCT1_04	0.84128	11718	(+)	NNNNNNNWATGCAAATNNNWNNW	AATGTTTTATTTAAAATTTATAA
M00138	V$OCT1_04	0.803011	11719	(+)	NNNNNNNWATGCAAATNNNWNNW	ATGTTTTATTTAAAATTTATAAA
M00135	V$OCT1_01	0.732736	11720	(+)	NNNNWTATGCAAATNTNNN	TGTTTTATTTAAAATTTAT
M00138	V$OCT1_04	0.851527	11720	(-)	NNNNNNNWATGCAAATNNNWNNW	TGTTTTATTTAAAATTTATAAAA
M00162	V$OCT1_06	0.834375	11723	(-)	CWNAWTKWSATRYN	TTTATTTAAAATTT
M00248	V$OCT1_07	0.807717	11723	(+)	TNTATGNTAATT	TTTATTTAAAAT
M00138	V$OCT1_04	0.804893	11725	(+)	NNNNNNNWATGCAAATNNNWNNW	TATTTAAAATTTATAAAAGATAA
M00162	V$OCT1_06	0.82656	11730	(-)	CWNAWTKWSATRYN	AAAATTTATAAAAG

		2				
M00138	V$OCT1_04	0.817859	11741	(+)	NNNNNNNWATGCAAATNNNWNNW	AAGATAAAATGTAAATGAGTGAT
M00135	V$OCT1_01	0.757917	11743	(+)	NNNNWTATGCAAATNTNNN	GATAAAATGTAAATGAGTG
M00195	V$OCT1_Q6	0.883734	11745	(+)	NNNNATGCAAATNAN	TAAAATGTAAATGAG
M00161	V$OCT1_05	0.896231	11746	(-)	MKNATTTGCATAYY	AAAATGTAAATGAG
M00162	V$OCT1_06	0.950781	11746	(-)	CWNAWTKWSATRYN	AAAATGTAAATGAG
M00248	V$OCT1_07	0.793089	11746	(+)	TNTATGNTAATT	AAAATGTAAATG
M00136	V$OCT1_02	0.825122	11762	(+)	NNGAATATKCANNNN	ATTAATAGTCATGAA
M00135	V$OCT1_01	0.736551	11819	(+)	NNNNWTATGCAAATNTNNN	CCAGATATGAAACTTATAG
M00161	V$OCT1_05	0.886103	11822	(-)	MKNATTTGCATAYY	GATATGAAACTTAT
M00162	V$OCT1_06	0.860547	11822	(-)	CWNAWTKWSATRYN	GATATGAAACTTAT
M00162	V$OCT1_06	0.808203	11829	(+)	CWNAWTKWSATRYN	AACTTATAGATTCT
M00195	V$OCT1_Q6	0.795306	11845	(+)	NNNNATGCAAATNAN	ATCCTTCCAAATTAT
M00137	V$OCT1_03	0.854603	11850	(-)	NNNRTAATNANNN	TCCAAATTATAGC
M00138	V$OCT1_04	0.804684	11869	(-)	NNNNNNNWATGCAAATNNNWNNW	AACATTGAGTTGAATATTTTTTC
M00136	V$OCT1_02	0.898755	11875	(-)	NNGAATATKCANNNN	GAGTTGAATATTTTT
M00136	V$OCT1_02	0.799134	11878	(+)	NNGAATATKCANNNN	TTGAATATTTTTTCT
M00162	V$OCT1_06	0.834375	11901	(-)	CWNAWTKWSATRYN	AAAATGAAAGCTAT
M00138	V$OCT1_04	0.7821	11908	(+)	NNNNNNNWATGCAAATNNNWNNW	AAGCTATATTGCTAATTTTCAAA
M00135	V$OCT1_01	0.749523	11910	(+)	NNNNWTATGCAAATNTNNN	GCTATATTGCTAATTTTCA
M00248	V$OCT1_07	0.82382	11913	(+)	TNTATGNTAATT	ATATTGCTAATT

		9				
M00135	V$OCT1_01	0.749523	11917	(-)	NNNNWTATGCAAATNTNNN	TGCTAATTTTCAAAATTTT
M00136	V$OCT1_02	0.776936	11918	(+)	NNGAATATKCANNNN	GCTAATTTTCAAAAT
M00161	V$OCT1_05	0.875145	11919	(+)	MKNATTTGCATAYY	CTAATTTTCAAAAT
M00162	V$OCT1_06	0.801562	11919	(+)	CWNAWTKWSATRYN	CTAATTTTCAAAAT
M00195	V$OCT1_Q6	0.826965	11919	(-)	NNNNATGCAAATNAN	CTAATTTTCAAAATT
M00162	V$OCT1_06	0.808203	11920	(-)	CWNAWTKWSATRYN	TAATTTTCAAAATT
M00162	V$OCT1_06	0.800391	11921	(-)	CWNAWTKWSATRYN	AATTTTCAAAATTT
M00135	V$OCT1_01	0.800649	11947	(+)	NNNNWTATGCAAATNTNNN	CAGTTTATACTAATCTTCA
M00248	V$OCT1_07	0.800297	11950	(+)	TNTATGNTAATT	TTTATACTAATC
M00138	V$OCT1_04	0.823923	11952	(-)	NNNNNNNWATGCAAATNNNWNNW	TATACTAATCTTCATAGATGATA
M00136	V$OCT1_02	0.786952	11955	(+)	NNGAATATKCANNNN	ACTAATCTTCATAGA
M00162	V$OCT1_06	0.801562	11956	(+)	CWNAWTKWSATRYN	CTAATCTTCATAGA
M00195	V$OCT1_Q6	0.821234	11956	(-)	NNNNATGCAAATNAN	CTAATCTTCATAGAT
M00248	V$OCT1_07	0.799661	11958	(-)	TNTATGNTAATT	AATCTTCATAGA
M00248	V$OCT1_07	0.839093	11977	(+)	TNTATGNTAATT	TTCTTGGTAATT
M00137	V$OCT1_03	0.884631	11980	(+)	NNNRTAATNANNN	TTGGTAATTGTAA
M00162	V$OCT1_06	0.81875	11983	(+)	CWNAWTKWSATRYN	GTAATTGTAAAATG
M00162	V$OCT1_06	0.821484	11989	(+)	CWNAWTKWSATRYN	GTAAAATGAATGTG
M00162	V$OCT1_06	0.821484	11991	(-)	CWNAWTKWSATRYN	AAAATGAATGTGTT
M00162	V$OCT1_06	0.839453	11994	(+)	CWNAWTKWSATRYN	ATGAATGTGTTACA

M00162	V$OCT1_06	0.826562	12014	(-)	CWNAWTKWSATRYN	GATATCTAGGTAAC
M00137	V$OCT1_03	0.860925	12043	(-)	NNNRTAATNANNN	TTCTATTTAGGAA
M00162	V$OCT1_06	0.847656	12065	(-)	CWNAWTKWSATRYN	AGTATGAAAGAGGG
M00162	V$OCT1_06	0.834375	12089	(+)	CWNAWTKWSATRYN	CACCAGGTAATATT
M00137	V$OCT1_03	0.852627	12092	(+)	NNNRTAATNANNN	CAGGTAATATTTT
M00138	V$OCT1_04	0.799875	12095	(+)	NNNNNNNWATGCAAATNNNWNNW	GTAATATTTTGCTAAATGAATTA
M00135	V$OCT1_01	0.742274	12097	(+)	NNNNWTATGCAAATNTNNN	AATATTTTGCTAAATGAAT
M00162	V$OCT1_06	0.873437	12106	(+)	CWNAWTKWSATRYN	CTAAATGAATTACA
M00162	V$OCT1_06	0.808203	12107	(-)	CWNAWTKWSATRYN	TAAATGAATTACAT
M00135	V$OCT1_01	0.760206	12108	(-)	NNNNWTATGCAAATNTNNN	AAATGAATTACATAATGAT
M00162	V$OCT1_06	0.868359	12110	(+)	CWNAWTKWSATRYN	ATGAATTACATAAT
M00195	V$OCT1_Q6	0.8256	12110	(-)	NNNNATGCAAATNAN	ATGAATTACATAATG
M00162	V$OCT1_06	0.924609	12111	(-)	CWNAWTKWSATRYN	TGAATTACATAATG
M00136	V$OCT1_02	0.784515	12112	(-)	NNGAATATKCANNNN	GAATTACATAATGAT
M00135	V$OCT1_01	0.771652	12116	(+)	NNNNWTATGCAAATNTNNN	TACATAATGATAATATGTT
M00137	V$OCT1_03	0.945081	12116	(+)	NNNRTAATNANNN	TACATAATGATAA
M00162	V$OCT1_06	0.809766	12119	(-)	CWNAWTKWSATRYN	ATAATGATAATATG
M00137	V$OCT1_03	0.858949	12122	(+)	NNNRTAATNANNN	ATGATAATATGTT
M00138	V$OCT1_04	0.792555	12133	(-)	NNNNNNNWATGCAAATNNNWNNW	TTGCAAAGTTTAAATATGTTTGC
M00162	V$OCT1_06	0.842187	12137	(+)	CWNAWTKWSATRYN	AAAGTTTAAATATG
M00162	V$OCT1_06	0.86054	12137	(-)	CWNAWTKWSATRYN	AAAGTTTAAATATG

		7				
M00136	V$OCT1_02	0.778289	12139	(-)	NNGAATATKCANNNN	AGTTTAAATATGTTT
M00138	V$OCT1_04	0.827478	12149	(-)	NNNNNNNWATGCAAATNNNWNNW	TGTTTGCTTTTAAATAGCTTCAC
M00162	V$OCT1_06	0.860156	12164	(-)	CWNAWTKWSATRYN	AGCTTCACAATTTT
M00138	V$OCT1_04	0.796738	12226	(-)	NNNNNNNWATGCAAATNNNWNNW	TGAAACAATTTGTAGTAATCAAT
M00137	V$OCT1_03	0.873173	12237	(+)	NNNRTAATNANNN	GTAGTAATCAATC
M00138	V$OCT1_04	0.794856	12259	(-)	NNNNNNNWATGCAAATNNNWNNW	AGAACTATTTGTCATTCTTCTTT
M00162	V$OCT1_06	0.929688	12263	(+)	CWNAWTKWSATRYN	CTATTTGTCATTCT
M00162	V$OCT1_06	0.862891	12264	(-)	CWNAWTKWSATRYN	TATTTGTCATTCTT
M00162	V$OCT1_06	0.800781	12326	(+)	CWNAWTKWSATRYN	ACCTTTGTAATTCT
M00162	V$OCT1_06	0.85	12343	(+)	CWNAWTKWSATRYN	CACTATGAAGTGTT
M00248	V$OCT1_07	0.803265	12349	(+)	TNTATGNTAATT	GAAGTGTTAATT
M00138	V$OCT1_04	0.863028	12351	(-)	NNNNNNNWATGCAAATNNNWNNW	AGTGTTAATTTCCATGGTTATGT
M00135	V$OCT1_01	0.748378	12353	(-)	NNNNWTATGCAAATNTNNN	TGTTAATTTCCATGGTTAT
M00195	V$OCT1_Q6	0.838428	12355	(-)	NNNNATGCAAATNAN	TTAATTTCCATGGTT
M00138	V$OCT1_04	0.782518	12356	(+)	NNNNNNNWATGCAAATNNNWNNW	TAATTTCCATGGTTATGTTAATT
M00248	V$OCT1_07	0.898664	12357	(-)	TNTATGNTAATT	AATTTCCATGGT
M00138	V$OCT1_04	0.806985	12362	(+)	NNNNNNNWATGCAAATNNNWNNW	CCATGGTTATGTTAATTTCCATG
M00135	V$OCT1_01	0.879817	12364	(+)	NNNNWTATGCAAATNTNNN	ATGGTTATGTTAATTTCCA
M00162	V$OCT1_06	0.805859	12367	(+)	CWNAWTKWSATRYN	GTTATGTTAATTTC
M00248	V$OCT1_07	0.955268	12367	(+)	TNTATGNTAATT	GTTATGTTAATT

M00138	V$OCT1_04	0.86595 6	12369	(-)	NNNNNNNWATGCAAATNNNW NNW	TATGTTAATTTCCATGGTTATGT
M00135	V$OCT1_01	0.74837 8	12371	(-)	NNNNWTATGCAAATNTNNN	TGTTAATTTCCATGGTTAT
M00195	V$OCT1_Q6	0.83842 8	12373	(-)	NNNNATGCAAATNAN	TTAATTTCCATGGTT
M00138	V$OCT1_04	0.78251 8	12374	(+)	NNNNNNNWATGCAAATNNNW NNW	TAATTTCCATGGTTATGTTAATT
M00248	V$OCT1_07	0.89866 4	12375	(-)	TNTATGNTAATT	AATTTCCATGGT
M00138	V$OCT1_04	0.84420 7	12380	(+)	NNNNNNNWATGCAAATNNNW NNW	CCATGGTTATGTTAATTTCTCTT
M00135	V$OCT1_01	0.87275 8	12382	(+)	NNNNWTATGCAAATNTNNN	ATGGTTATGTTAATTTCTC
M00162	V$OCT1_06	0.80585 9	12385	(+)	CWNAWTKWSATRYN	GTTATGTTAATTTC
M00248	V$OCT1_07	0.95526 8	12385	(+)	TNTATGNTAATT	GTTATGTTAATT
M00138	V$OCT1_04	0.83898	12412	(-)	NNNNNNNWATGCAAATNNNW NNW	TCCTAAATTTTATATCTTTATGT
M00162	V$OCT1_06	0.88671 9	12414	(+)	CWNAWTKWSATRYN	CTAAATTTTATATC
M00195	V$OCT1_Q6	0.79830 8	12414	(-)	NNNNATGCAAATNAN	CTAAATTTTATATCT
M00162	V$OCT1_06	0.80859 4	12415	(-)	CWNAWTKWSATRYN	TAAATTTTATATCT
M00138	V$OCT1_04	0.78711 8	12418	(-)	NNNNNNNWATGCAAATNNNW NNW	ATTTTATATCTTTATGTCCATGA
M00137	V$OCT1_03	0.89529 8	12423	(-)	NNNRTAATNANNN	ATATCTTTATGTC
M00162	V$OCT1_06	0.88125	12436	(+)	CWNAWTKWSATRYN	CATGATTTGATTCT
M00162	V$OCT1_06	0.88554 7	12444	(+)	CWNAWTKWSATRYN	GATTCTTAAATTCA
M00138	V$OCT1_04	0.80531 2	12450	(+)	NNNNNNNWATGCAAATNNNW NNW	TAAATTCAATTTATATTTAGGAA
M00162	V$OCT1_06	0.90390 6	12450	(-)	CWNAWTKWSATRYN	TAAATTCAATTTAT
M00248	V$OCT1_07	0.81344 1	12457	(-)	TNTATGNTAATT	AATTTATATTTA
M00137	V$OCT1_03	0.85104	12460	(-)	NNNRTAATNANNN	TTATATTTAGGAA

		7				
M00162	V$OCT1_06	0.800391	12461	(-)	CWNAWTKWSATRYN	TATATTTAGGAAAT
M00162	V$OCT1_06	0.808203	12464	(+)	CWNAWTKWSATRYN	ATTTAGGAAATTCA
M00138	V$OCT1_04	0.800293	12465	(+)	NNNNNNNWATGCAAATNNNNWNNW	TTTAGGAAATTCAAACTCATAAT
M00136	V$OCT1_02	0.812669	12467	(+)	NNGAATATKCANNNN	TAGGAAATTCAAACT
M00138	V$OCT1_04	0.795483	12471	(+)	NNNNNNNWATGCAAATNNNNWNNW	AAATTCAAACTCATAATTATGCT
M00137	V$OCT1_03	0.923746	12480	(+)	NNNRTAATNANNN	CTCATAATTATGC
M00137	V$OCT1_03	0.930462	12481	(-)	NNNRTAATNANNN	TCATAATTATGCT
M00135	V$OCT1_01	0.755437	12483	(+)	NNNNWTATGCAAATNTNNN	ATAATTATGCTCTTTCAAG
M00136	V$OCT1_02	0.812128	12483	(+)	NNGAATATKCANNNN	ATAATTATGCTCTTT
M00162	V$OCT1_06	0.812109	12500	(-)	CWNAWTKWSATRYN	AGCAAGCCATTTGG
M00135	V$OCT1_01	0.761541	12503	(-)	NNNNWTATGCAAATNTNNN	AAGCCATTTGGAGATTTAT
M00162	V$OCT1_06	0.847266	12507	(+)	CWNAWTKWSATRYN	CATTTGGAGATTTA
M00138	V$OCT1_04	0.791092	12511	(-)	NNNNNNNWATGCAAATNNNNWNNW	TGGAGATTTATTAATTCTAATTG
M00162	V$OCT1_06	0.800391	12515	(+)	CWNAWTKWSATRYN	GATTTATTAATTCT
M00138	V$OCT1_04	0.827687	12516	(+)	NNNNNNNWATGCAAATNNNNWNNW	ATTTATTAATTCTAATTGAAACC
M00135	V$OCT1_01	0.744754	12518	(+)	NNNNWTATGCAAATNTNNN	TTATTAATTCTAATTGAAA
M00248	V$OCT1_07	0.88234	12521	(+)	TNTATGNTAATT	TTAATTCTAATT
M00138	V$OCT1_04	0.795483	12522	(+)	NNNNNNNWATGCAAATNNNNWNNW	TAATTCTAATTGAAACCTTTAGA
M00162	V$OCT1_06	0.816016	12560	(+)	CWNAWTKWSATRYN	AGATAGTAAATGTT
M00162	V$OCT1_06	0.808203	12561	(-)	CWNAWTKWSATRYN	GATAGTAAATGTTT

M00138	V$OCT1_04	0.809285	12619	(+)	NNNNNNNNWATGCAAATNNNWNNW	GTGTTTTTATCTATATAATAGAA
M00138	V$OCT1_04	0.83187	12620	(-)	NNNNNNNNWATGCAAATNNNWNNW	TGTTTTTATCTATATAATAGAAA
M00162	V$OCT1_06	0.878125	12655	(+)	CWNAWTKWSATRYN	CTGCCTTGCATTCC
M00195	V$OCT1_Q6	0.8256	12655	(-)	NNNNATGCAAATNAN	CTGCCTTGCATTCCA
M00162	V$OCT1_06	0.911719	12661	(-)	CWNAWTKWSATRYN	TGCATTCCATTGAT
M00162	V$OCT1_06	0.839453	12723	(+)	CWNAWTKWSATRYN	GATACTGTGAAATA
M00162	V$OCT1_06	0.841797	12732	(+)	CWNAWTKWSATRYN	AAATATGTGCTGCT
M00162	V$OCT1_06	0.819141	12743	(-)	CWNAWTKWSATRYN	GCTATTACAATTCT
M00138	V$OCT1_04	0.78189	12766	(+)	NNNNNNNNWATGCAAATNNNWNNW	CAAAAGCTATGCCAATTTCAATC
M00135	V$OCT1_01	0.819535	12768	(+)	NNNNWTATGCAAATNTNNN	AAAGCTATGCCAATTTCAA
M00195	V$OCT1_Q6	0.82833	12770	(+)	NNNNATGCAAATNAN	AGCTATGCCAATTTC
M00162	V$OCT1_06	0.847656	12771	(-)	CWNAWTKWSATRYN	GCTATGCCAATTTC
M00248	V$OCT1_07	0.874709	12771	(+)	TNTATGNTAATT	GCTATGCCAATT
M00162	V$OCT1_06	0.814062	12777	(-)	CWNAWTKWSATRYN	CCAATTTCAATCAC
M00162	V$OCT1_06	0.847656	12789	(+)	CWNAWTKWSATRYN	ACTACTGTCATTTA
M00136	V$OCT1_02	0.770168	12819	(-)	NNGAATATKCANNNN	TTGTTGAGTATGTTT
M00138	V$OCT1_04	0.787955	12822	(-)	NNNNNNNNWATGCAAATNNNWNNW	TTGAGTATGTTTCATATGTCTTA
M00162	V$OCT1_06	0.834375	12826	(+)	CWNAWTKWSATRYN	GTATGTTTCATATG
M00136	V$OCT1_02	0.770168	12844	(-)	NNGAATATKCANNNN	AGAAGAAATATTATC
M00135	V$OCT1_01	0.765929	12859	(+)	NNNNWTATGCAAATNTNNN	TAGATAATGCAATTGGCAA
M00136	V$OCT1_02	0.801029	12859	(+)	NNGAATATKCANNNN	TAGATAATGCAATTG

M00195	V$OCT1_Q6	0.814138	12861	(+)	NNNNATGCAAATNAN	GATAATGCAATTGGC
M00138	V$OCT1_04	0.802802	12863	(-)	NNNNNNNWATGCAAATNNNWNNW	TAATGCAATTGGCAAAATTATTA
M00135	V$OCT1_01	0.781381	12865	(-)	NNNNWTATGCAAATNTNNN	ATGCAATTGGCAAAATTAT
M00135	V$OCT1_01	0.739794	12866	(+)	NNNNWTATGCAAATNTNNN	TGCAATTGGCAAAATTATT
M00137	V$OCT1_03	0.840379	12874	(-)	NNNRTAATNANNN	GCAAAATTATTAG
M00136	V$OCT1_02	0.825393	12885	(-)	NNGAATATKCANNNN	AGTGTGAATTTCCAG
M00138	V$OCT1_04	0.813676	12885	(-)	NNNNNNNWATGCAAATNNNWNNW	AGTGTGAATTTCCAGATCTCTAA
M00135	V$OCT1_01	0.77375	12887	(-)	NNNNWTATGCAAATNTNNN	TGTGAATTTCCAGATCTCT
M00162	V$OCT1_06	0.808203	12889	(-)	CWNAWTKWSATRYN	TGAATTTCCAGATC
M00248	V$OCT1_07	0.836125	12891	(-)	TNTATGNTAATT	AATTTCCAGATC
M00162	V$OCT1_06	0.829297	12900	(+)	CWNAWTKWSATRYN	ATCTCTAACATGCA
M00138	V$OCT1_04	0.832079	12901	(+)	NNNNNNNWATGCAAATNNNWNNW	TCTCTAACATGCAAAGCATTTCA
M00135	V$OCT1_01	0.750095	12903	(+)	NNNNWTATGCAAATNTNNN	TCTAACATGCAAAGCATTT
M00136	V$OCT1_02	0.770168	12903	(+)	NNGAATATKCANNNN	TCTAACATGCAAAGC
M00195	V$OCT1_Q6	0.899836	12905	(+)	NNNNATGCAAATNAN	TAACATGCAAAGCAT
M00161	V$OCT1_05	0.938569	12906	(-)	MKNATTTGCATAYY	AACATGCAAAGCAT
M00162	V$OCT1_06	0.898437	12906	(-)	CWNAWTKWSATRYN	AACATGCAAAGCAT
M00162	V$OCT1_06	0.9375	12915	(-)	CWNAWTKWSATRYN	AGCATTTCAAAAAG
M00162	V$OCT1_06	0.800391	12917	(-)	CWNAWTKWSATRYN	CATTTCAAAAAGTT
M00162	V$OCT1_06	0.829297	12932	(+)	CWNAWTKWSATRYN	GCTTCTTACATTTT
M00136	V$OCT1_02	0.792637	12934	(-)	NNGAATATKCANNNN	TTCTTACATTTTCTG

M00162	V$OCT1_06	0.821094	12958	(-)	CWNAWTKWSATRYN	AGCCTTAAAAGATC
M00162	V$OCT1_06	0.813672	12979	(+)	CWNAWTKWSATRYN	CTCTTTTTCTTTCC
M00162	V$OCT1_06	0.826562	13038	(+)	CWNAWTKWSATRYN	CTGTTTGAGTTTTA
M00162	V$OCT1_06	0.816016	13132	(-)	CWNAWTKWSATRYN	AGTATTTCTTAGTT
M00162	V$OCT1_06	0.821094	13135	(+)	CWNAWTKWSATRYN	ATTTCTTAGTTGTT
M00162	V$OCT1_06	0.808594	13218	(-)	CWNAWTKWSATRYN	GAAATGAAAGAACA
M00162	V$OCT1_06	0.826562	13269	(+)	CWNAWTKWSATRYN	CAACTTTTAAGTTA
M00162	V$OCT1_06	0.860547	13270	(-)	CWNAWTKWSATRYN	AACTTTTAAGTTAG
M00138	V$OCT1_04	0.81514	13293	(+)	NNNNNNNWATGCAAATNNNWNNW	AACCAACTATTCATATCATTCAA
M00136	V$OCT1_02	0.799675	13295	(+)	NNGAATATKCANNNN	CCAACTATTCATATC
M00162	V$OCT1_06	0.847266	13296	(+)	CWNAWTKWSATRYN	CAACTATTCATATC
M00162	V$OCT1_06	0.834375	13301	(+)	CWNAWTKWSATRYN	ATTCATATCATTCA
M00138	V$OCT1_04	0.813258	13302	(+)	NNNNNNNWATGCAAATNNNWNNW	TTCATATCATTCAAATTTGACTT
M00135	V$OCT1_01	0.785006	13304	(+)	NNNNWTATGCAAATNTNNN	CATATCATTCAAATTTGAC
M00136	V$OCT1_02	0.771521	13304	(+)	NNGAATATKCANNNN	CATATCATTCAAATT
M00162	V$OCT1_06	0.814062	13307	(+)	CWNAWTKWSATRYN	ATCATTCAAATTTG
M00162	V$OCT1_06	0.873828	13307	(-)	CWNAWTKWSATRYN	ATCATTCAAATTTG
M00248	V$OCT1_07	0.839093	13307	(+)	TNTATGNTAATT	ATCATTCAAATT
M00138	V$OCT1_04	0.832079	13309	(-)	NNNNNNNWATGCAAATNNNWNNW	CATTCAAATTTGACTTTTTATTA
M00135	V$OCT1_01	0.744754	13311	(-)	NNNNWTATGCAAATNTNNN	TTCAAATTTGACTTTTTAT

M00162	V$OCT1_06	0.839844	13313	(+)	CWNAWTKWSATRYN	CAAATTTGACTTTT
M00162	V$OCT1_06	0.828906	13314	(+)	CWNAWTKWSATRYN	AAATTTGACTTTTT
M00138	V$OCT1_04	0.799665	13315	(-)	NNNNNNNWATGCAAATNNNWNNW	AATTTGACTTTTTATTAATCTAT
M00162	V$OCT1_06	0.826562	13351	(+)	CWNAWTKWSATRYN	CTTCTTGAGTTTTC
M00136	V$OCT1_02	0.795885	13352	(-)	NNGAATATKCANNNN	TTCTTGAGTTTTCAG
M00162	V$OCT1_06	0.855469	13364	(+)	CWNAWTKWSATRYN	CAGTCTTAGATCTA
M00138	V$OCT1_04	0.802384	13430	(+)	NNNNNNNWATGCAAATNNNWNNW	TTTGTGAAATTAATATGAATCTA
M00138	V$OCT1_04	0.807821	13431	(-)	NNNNNNNWATGCAAATNNNWNNW	TTGTGAAATTAATATGAATCTAT
M00162	V$OCT1_06	0.813672	13433	(+)	CWNAWTKWSATRYN	GTGAAATTAATATG
M00138	V$OCT1_04	0.782727	13436	(+)	NNNNNNNWATGCAAATNNNWNNW	AAATTAATATGAATCTATTGATT
M00248	V$OCT1_07	0.837397	13437	(-)	TNTATGNTAATT	AATTAATATGAA
M00136	V$OCT1_02	0.82431	13438	(+)	NNGAATATKCANNNN	ATTAATATGAATCTA
M00162	V$OCT1_06	0.828906	13445	(-)	CWNAWTKWSATRYN	TGAATCTATTGATT
M00162	V$OCT1_06	0.813281	13447	(+)	CWNAWTKWSATRYN	AATCTATTGATTTA
M00135	V$OCT1_01	0.734452	13468	(-)	NNNNWTATGCAAATNTNNN	CTAAAACTCACATATCTTG
M00162	V$OCT1_06	0.855469	13470	(+)	CWNAWTKWSATRYN	AAAACTCACATATC
M00162	V$OCT1_06	0.800391	13471	(-)	CWNAWTKWSATRYN	AAACTCACATATCT
M00138	V$OCT1_04	0.830824	13482	(-)	NNNNNNNWATGCAAATNNNWNNW	TCTTGAGTTTTGCAGTGTTTTTT
M00162	V$OCT1_06	0.829297	13513	(+)	CWNAWTKWSATRYN	CAGTTTTAAAGGTG
M00162	V$OCT1_06	0.808594	13526	(+)	CWNAWTKWSATRYN	GGTAACTGCATTCG
M00138	V$OCT1_04	0.79862	13563	(-)	NNNNNNNWATGCAAATNNNW	TGGTTGAACTTACATATAGTATG

					NNW	
M00135	V$OCT1_01	0.79587 9	13565	(-)	NNNNWTATGCAAATNTNNN	GTTGAACTTACATATAGTA
M00162	V$OCT1_06	0.86835 9	13567	(+)	CWNAWTKWSATRYN	TGAACTTACATATA
M00162	V$OCT1_06	0.81367 2	13568	(-)	CWNAWTKWSATRYN	GAACTTACATATAG
M00248	V$OCT1_07	0.86644 1	13569	(-)	TNTATGNTAATT	AACTTACATATA
M00138	V$OCT1_04	0.86804 7	13575	(+)	NNNNNNNWATGCAAATNNNW NNW	CATATAGTATGCTAATTTAAAAA
M00135	V$OCT1_01	0.90614 3	13577	(+)	NNNNWTATGCAAATNTNNN	TATAGTATGCTAATTTAAA
M00136	V$OCT1_02	0.77801 8	13577	(+)	NNGAATATKCANNNN	TATAGTATGCTAATT
M00162	V$OCT1_06	0.80429 7	13580	(-)	CWNAWTKWSATRYN	AGTATGCTAATTTA
M00248	V$OCT1_07	0.98070 8	13580	(+)	TNTATGNTAATT	AGTATGCTAATT
M00138	V$OCT1_04	0.80071 1	13581	(+)	NNNNNNNWATGCAAATNNNW NNW	GTATGCTAATTTAAAAAAGAAAC
M00162	V$OCT1_06	0.82695 3	13586	(-)	CWNAWTKWSATRYN	CTAATTTAAAAAAG
M00195	V$OCT1_Q6	0.86790 4	13601	(+)	NNNNATGCAAATNAN	AACCGTGCAAAGAAG
M00138	V$OCT1_04	0.78105 4	13616	(-)	NNNNNNNWATGCAAATNNNW NNW	ACCATGGAGTTTTATAAATATGA
M00138	V$OCT1_04	0.80907 6	13620	(-)	NNNNNNNWATGCAAATNNNW NNW	TGGAGTTTTATAAATATGAATCT
M00162	V$OCT1_06	0.80039 1	13624	(+)	CWNAWTKWSATRYN	GTTTTATAAATATG
M00136	V$OCT1_02	0.80806 7	13626	(-)	NNGAATATKCANNNN	TTTATAAATATGAAT
M00137	V$OCT1_03	0.87119 7	13626	(+)	NNNRTAATNANNN	TTTATAAATATGA
M00138	V$OCT1_04	0.81221 2	13626	(-)	NNNNNNNWATGCAAATNNNW NNW	TTTATAAATATGAATCTGATAAG
M00138	V$OCT1_04	0.81618 6	13627	(+)	NNNNNNNWATGCAAATNNNW NNW	TTATAAATATGAATCTGATAAGG
M00136	V$OCT1_02	0.78343	13629	(+)	NNGAATATKCANNNN	ATAAATATGAATCTG

		3				
M00162	V$OCT1_06	0.804687	13630	(-)	CWNAWTKWSATRYN	TAAATATGAATCTG
M00138	V$OCT1_04	0.792137	13653	(+)	NNNNNNNWATGCAAATNNNWNNW	TAAACAAGATTCAAAACATAGTA
M00136	V$OCT1_02	0.792637	13655	(+)	NNGAATATKCANNNN	AACAAGATTCAAAAC
M00162	V$OCT1_06	0.800781	13658	(-)	CWNAWTKWSATRYN	AAGATTCAAAACAT
M00138	V$OCT1_04	0.84588	13684	(+)	NNNNNNNWATGCAAATNNNWNNW	TCAATTTTATGATAATCATTCCT
M00135	V$OCT1_01	0.743228	13686	(+)	NNNNWTATGCAAATNTNNN	AATTTTATGATAATCATTC
M00138	V$OCT1_04	0.800711	13688	(-)	NNNNNNNWATGCAAATNNNWNNW	TTTTATGATAATCATTCCTTAAT
M00161	V$OCT1_05	0.862693	13689	(-)	MKNATTTGCATAYY	TTTATGATAATCAT
M00248	V$OCT1_07	0.818741	13689	(+)	TNTATGNTAATT	TTTATGATAATC
M00137	V$OCT1_03	0.883445	13692	(+)	NNNRTAATNANNN	ATGATAATCATTC
M00162	V$OCT1_06	0.821484	13700	(+)	CWNAWTKWSATRYN	CATTCCTTAATGTG
M00162	V$OCT1_06	0.865625	13705	(+)	CWNAWTKWSATRYN	CTTAATGTGAATCA
M00195	V$OCT1_Q6	0.831878	13705	(+)	NNNNATGCAAATNAN	CTTAATGTGAATCAT
M00162	V$OCT1_06	0.886719	13712	(-)	CWNAWTKWSATRYN	TGAATCATATTTTG
M00162	V$OCT1_06	0.804297	13728	(+)	CWNAWTKWSATRYN	CTCAATTTCTTCTA
M00162	V$OCT1_06	0.826562	13780	(-)	CWNAWTKWSATRYN	GAAAAGACAATTAC
M00137	V$OCT1_03	0.847096	13784	(-)	NNNRTAATNANNN	AGACAATTACGTA
M00162	V$OCT1_06	0.862891	13785	(+)	CWNAWTKWSATRYN	GACAATTACGTACT
M00162	V$OCT1_06	0.847656	13793	(-)	CWNAWTKWSATRYN	CGTACTTCATTGAG
M00137	V$OCT1_03	0.84314	13806	(+)	NNNRTAATNANNN	GGTCTAAAGAGTA

		5				
M00138	V$OCT1_04	0.788164	13824	(-)	NNNNNNNWATGCAAATNNNWNNW	TCCTCACCTTTCCATGTAATTTT
M00162	V$OCT1_06	0.876172	13828	(+)	CWNAWTKWSATRYN	CACCTTTCCATGTA
M00162	V$OCT1_06	0.821484	13833	(+)	CWNAWTKWSATRYN	TTCCATGTAATTTT
M00162	V$OCT1_06	0.896875	13834	(-)	CWNAWTKWSATRYN	TCCATGTAATTTTT
M00248	V$OCT1_07	0.817257	13834	(+)	TNTATGNTAATT	TCCATGTAATTT
M00137	V$OCT1_03	0.849072	13836	(+)	NNNRTAATNANNN	CATGTAATTTTTT
M00137	V$OCT1_03	0.885026	13855	(+)	NNNRTAATNANNN	AGGGTAATCATTC
M00138	V$OCT1_04	0.801129	13856	(+)	NNNNNNNWATGCAAATNNNWNNW	GGGTAATCATTCTAATCTTAATG
M00135	V$OCT1_01	0.757535	13858	(+)	NNNNWTATGCAAATNTNNN	GTAATCATTCTAATCTTAA
M00137	V$OCT1_03	0.883445	13870	(+)	NNNRTAATNANNN	ATCTTAATGAGAG
M00162	V$OCT1_06	0.882812	13928	(-)	CWNAWTKWSATRYN	TGAATCACAAAACT
M00162	V$OCT1_06	0.813672	13966	(+)	CWNAWTKWSATRYN	GGAACTGGCCTACT
M00138	V$OCT1_04	0.830197	13992	(-)	NNNNNNNWATGCAAATNNNWNNW	TGAATTTATTTGATTTTTTATAT
M00162	V$OCT1_06	0.821484	13992	(-)	CWNAWTKWSATRYN	TGAATTTATTTGAT
M00162	V$OCT1_06	0.903516	13995	(+)	CWNAWTKWSATRYN	ATTTATTTGATTTT
M00138	V$OCT1_04	0.8555	13998	(-)	NNNNNNNWATGCAAATNNNWNNW	TATTTGATTTTTTATATTAATAC
M00138	V$OCT1_04	0.825387	13999	(+)	NNNNNNNWATGCAAATNNNWNNW	ATTTGATTTTTTATATTAATACT
M00138	V$OCT1_04	0.803848	14000	(-)	NNNNNNNWATGCAAATNNNWNNW	TTTGATTTTTTATATTAATACTA
M00138	V$OCT1_04	0.819113	14003	(+)	NNNNNNNWATGCAAATNNNWNNW	GATTTTTTATATTAATACTAATA
M00138	V$OCT1_04	0.85654	14004	(-)	NNNNNNNWATGCAAATNNNW	ATTTTTTATATTAATACTAATAA

		5			NNW	
M00135	V$OCT1_01	0.739985	14005	(+)	NNNNWTATGCAAATNTNNN	TTTTTTATATTAATACTAA
M00248	V$OCT1_07	0.793513	14008	(+)	TNTATGNTAATT	TTTATATTAATA
M00138	V$OCT1_04	0.83187	14009	(+)	NNNNNNNWATGCAAATNNNWNNW	TTATATTAATACTAATAAGAGAA
M00135	V$OCT1_01	0.740557	14011	(+)	NNNNWTATGCAAATNTNNN	ATATTAATACTAATAAGAG
M00195	V$OCT1_Q6	0.818777	14013	(+)	NNNNATGCAAATNAN	ATTAATACTAATAAG
M00137	V$OCT1_03	0.850652	14017	(+)	NNNRTAATNANNN	ATACTAATAAGAG
M00162	V$OCT1_06	0.890625	14030	(-)	CWNAWTKWSATRYN	AAAATCTCAGATAT
M00162	V$OCT1_06	0.821484	14031	(+)	CWNAWTKWSATRYN	AAATCTCAGATATC
M00162	V$OCT1_06	0.852734	14039	(-)	CWNAWTKWSATRYN	GATATCATATTTAT
M00138	V$OCT1_04	0.797574	14041	(-)	NNNNNNNWATGCAAATNNNWNNW	TATCATATTTATTATAATCGTTA
M00137	V$OCT1_03	0.853418	14043	(-)	NNNRTAATNANNN	TCATATTTATTAT
M00138	V$OCT1_04	0.819532	14043	(+)	NNNNNNNWATGCAAATNNNWNNW	TCATATTTATTATAATCGTTAGG
M00137	V$OCT1_03	0.872382	14046	(-)	NNNRTAATNANNN	TATTTATTATAAT
M00137	V$OCT1_03	0.853813	14051	(+)	NNNRTAATNANNN	ATTATAATCGTTA
M00162	V$OCT1_06	0.919141	14082	(+)	CWNAWTKWSATRYN	AAACTTGAAATGCT
M00162	V$OCT1_06	0.836719	14083	(-)	CWNAWTKWSATRYN	AACTTGAAATGCTT
M00162	V$OCT1_06	0.81875	14094	(+)	CWNAWTKWSATRYN	CTTTCTTTGGTTTT
M00135	V$OCT1_01	0.741511	14100	(+)	NNNNWTATGCAAATNTNNN	TTGGTTTTTCTAATTCCAT
M00248	V$OCT1_07	0.805385	14103	(+)	TNTATGNTAATT	GTTTTTCTAATT
M00248	V$OCT1_07	0.792453	14120	(-)	TNTATGNTAATT	AAAGAACATATA

M00138	V$OCT1_04	0.782727	14125	(-)	NNNNNNNWATGCAAATNNNWNNW	ACATATATTTTCCACCTGAATAT
M00138	V$OCT1_04	0.794647	14132	(+)	NNNNNNNWATGCAAATNNNWNNW	TTTTCCACCTGAATATATAAAGA
M00136	V$OCT1_02	0.792366	14137	(-)	NNGAATATKCANNNN	CACCTGAATATATAA
M00138	V$OCT1_04	0.797783	14138	(+)	NNNNNNNWATGCAAATNNNWNNW	ACCTGAATATATAAAGAATTACA
M00138	V$OCT1_04	0.79151	14139	(-)	NNNNNNNWATGCAAATNNNWNNW	CCTGAATATATAAAGAATTACAT
M00195	V$OCT1_Q6	0.841976	14142	(+)	NNNNATGCAAATNAN	GAATATATAAAGAAT
M00162	V$OCT1_06	0.847266	14143	(-)	CWNAWTKWSATRYN	AATATATAAAGAAT
M00137	V$OCT1_03	0.841565	14145	(+)	NNNRTAATNANNN	TATATAAAGAATT
M00138	V$OCT1_04	0.781263	14147	(-)	NNNNNNNWATGCAAATNNNWNNW	TATAAAGAATTACATTAATGCAC
M00162	V$OCT1_06	0.863281	14151	(+)	CWNAWTKWSATRYN	AAGAATTACATTAA
M00162	V$OCT1_06	0.916797	14152	(-)	CWNAWTKWSATRYN	AGAATTACATTAAT
M00248	V$OCT1_07	0.815137	14154	(-)	TNTATGNTAATT	AATTACATTAAT
M00162	V$OCT1_06	0.826562	14155	(+)	CWNAWTKWSATRYN	ATTACATTAATGCA
M00162	V$OCT1_06	0.800781	14208	(-)	CWNAWTKWSATRYN	CCTATTCCAAAATT
M00162	V$OCT1_06	0.817187	14215	(+)	CWNAWTKWSATRYN	CAAAATTTCAGAAA
M00162	V$OCT1_06	0.821484	14216	(-)	CWNAWTKWSATRYN	AAAATTTCAGAAAA
M00162	V$OCT1_06	0.842187	14225	(-)	CWNAWTKWSATRYN	GAAAAGAAATAATG
M00137	V$OCT1_03	0.86132	14230	(+)	NNNRTAATNANNN	GAAATAATGGTTT
M00137	V$OCT1_03	0.877124	14255	(-)	NNNRTAATNANNN	TTATAGTTACGAA
M00137	V$OCT1_03	0.873568	14299	(-)	NNNRTAATNANNN	TTCTCTTTATTCT
M00162	V$OCT1_06	0.87773	14307	(+)	CWNAWTKWSATRYN	ATTCTTGGCATTTC

		4				
M00195	V$OCT1_Q6	0.862445	14307	(-)	NNNNATGCAAATNAN	ATTCTTGGCATTTCT
M00162	V$OCT1_06	0.808594	14316	(+)	CWNAWTKWSATRYN	ATTTCTGAAATCCC
M00162	V$OCT1_06	0.800781	14322	(-)	CWNAWTKWSATRYN	GAAATCCCTGTATT
M00138	V$OCT1_04	0.809076	14372	(+)	NNNNNNNWATGCAAATNNNWNNW	TTGCCTTCATGAAAAACTAAACT
M00162	V$OCT1_06	0.847266	14376	(+)	CWNAWTKWSATRYN	CTTCATGAAAAACT
M00138	V$OCT1_04	0.818904	14398	(+)	NNNNNNNWATGCAAATNNNWNNW	GAACAGAAAGGCAAAAATGACCT
M00135	V$OCT1_01	0.73388	14400	(+)	NNNNWTATGCAAATNTNNN	ACAGAAAGGCAAAAATGAC
M00162	V$OCT1_06	0.821484	14403	(-)	CWNAWTKWSATRYN	GAAAGGCAAAAATG
M00162	V$OCT1_06	0.862891	14410	(+)	CWNAWTKWSATRYN	AAAAATGACCTTCT
M00162	V$OCT1_06	0.842187	14411	(-)	CWNAWTKWSATRYN	AAAATGACCTTCTC
M00135	V$OCT1_01	0.770126	14421	(+)	NNNNWTATGCAAATNTNNN	TCTCTAATCCAAATGTCCA
M00162	V$OCT1_06	0.814062	14424	(-)	CWNAWTKWSATRYN	CTAATCCAAATGTC
M00135	V$OCT1_01	0.731209	14428	(-)	NNNNWTATGCAAATNTNNN	TCCAAATGTCCATGAGTTT
M00162	V$OCT1_06	0.847656	14430	(-)	CWNAWTKWSATRYN	CAAATGTCCATGAG
M00138	V$OCT1_04	0.785236	14437	(+)	NNNNNNNWATGCAAATNNNWNNW	CCATGAGTTTATAAATTATATAA
M00195	V$OCT1_Q6	0.806769	14441	(+)	NNNNATGCAAATNAN	GAGTTTATAAATTAT
M00162	V$OCT1_06	0.804297	14447	(+)	CWNAWTKWSATRYN	ATAAATTATATAAT
M00137	V$OCT1_03	0.884235	14453	(+)	NNNRTAATNANNN	TATATAATAATTA
M00248	V$OCT1_07	0.819801	14453	(+)	TNTATGNTAATT	TATATAATAATT
M00137	V$OCT1_03	0.890952	14456	(+)	NNNRTAATNANNN	ATAATAATTAAAT

M00162	V$OCT1_06	0.932422	14458	(+)	CWNAWTKWSATRYN	AATAATTAAATTCA
M00162	V$OCT1_06	0.826953	14459	(-)	CWNAWTKWSATRYN	ATAATTAAATTCAC
M00136	V$OCT1_02	0.784786	14461	(+)	NNGAATATKCANNNN	AATTAAATTCACACT
M00162	V$OCT1_06	0.800391	14509	(+)	CWNAWTKWSATRYN	GTGTTAGTAATGCA
M00138	V$OCT1_04	0.795065	14510	(+)	NNNNNNNWATGCAAATNNNWNNW	TGTTAGTAATGCAGGTTCTTGGA
M00138	V$OCT1_04	0.797783	14560	(-)	NNNNNNNWATGCAAATNNNWNNW	AAGTGATTTATTCATTACCCCTC
M00137	V$OCT1_03	0.921375	14568	(-)	NNNRTAATNANNN	TATTCATTACCCC
M00135	V$OCT1_01	0.795498	14606	(-)	NNNNWTATGCAAATNTNNN	CCAAAATTTCCTTACTGTG
M00162	V$OCT1_06	0.821484	14608	(-)	CWNAWTKWSATRYN	AAAATTTCCTTACT
M00248	V$OCT1_07	0.854781	14610	(-)	TNTATGNTAATT	AATTTCCTTACT
M00162	V$OCT1_06	0.935156	14616	(+)	CWNAWTKWSATRYN	CTTACTGTGATTTA
M00135	V$OCT1_01	0.749332	14621	(-)	NNNNWTATGCAAATNTNNN	TGTGATTTACCATAGGGAA
M00248	V$OCT1_07	0.853297	14625	(-)	TNTATGNTAATT	ATTTACCATAGG
M00135	V$OCT1_01	0.7314	14679	(+)	NNNNWTATGCAAATNTNNN	CTTGTGATGCGAATAGCCA
M00136	V$OCT1_02	0.792366	14684	(-)	NNGAATATKCANNNN	GATGCGAATAGCCAT
M00162	V$OCT1_06	0.808594	14697	(+)	CWNAWTKWSATRYN	ATGTCTGAAATGAA
M00162	V$OCT1_06	0.833984	14698	(-)	CWNAWTKWSATRYN	TGTCTGAAATGAAT
M00162	V$OCT1_06	0.847656	14701	(+)	CWNAWTKWSATRYN	CTGAAATGAATGCA
M00162	V$OCT1_06	0.90625	14707	(-)	CWNAWTKWSATRYN	TGAATGCAATGATT
M00138	V$OCT1_04	0.800084	14715	(-)	NNNNNNNWATGCAAATNNNWNNW	ATGATTCATTTTCAGAGATTCAA
M00162	V$OCT1_06	0.821875	14729	(-)	CWNAWTKWSATRYN	GAGATTCAAAACTG

M00162	V$OCT1_06	0.808203	14742	(-)	CWNAWTKWSATRYN	GATTTCTCATACAC
M00138	V$OCT1_04	0.785236	14756	(-)	NNNNNNNWATGCAAATNNNWNNW	TGTTGCTTTTTCAATGGTCTTTC
M00162	V$OCT1_06	0.804687	14924	(-)	CWNAWTKWSATRYN	ACTATGCCACTGTG
M00162	V$OCT1_06	0.834375	14940	(+)	CWNAWTKWSATRYN	GGTAAGTTCATTTA
M00138	V$OCT1_04	0.832915	14941	(+)	NNNNNNNWATGCAAATNNNWNNW	GTAAGTTCATTTAAATGACTGCA
M00138	V$OCT1_04	0.804057	14942	(-)	NNNNNNNWATGCAAATNNNWNNW	TAAGTTCATTTAAATGACTGCAT
M00195	V$OCT1_Q6	0.800764	14945	(+)	NNNNATGCAAATNAN	GTTCATTTAAATGAC
M00162	V$OCT1_06	0.847656	14946	(-)	CWNAWTKWSATRYN	TTCATTTAAATGAC
M00136	V$OCT1_02	0.918517	14956	(-)	NNGAATATKCANNNN	TGACTGCATATTGCT
M00138	V$OCT1_04	0.797156	14993	(+)	NNNNNNNWATGCAAATNNNWNNW	AAAACATAATATAAAAATAAATT
M00138	V$OCT1_04	0.796738	14995	(+)	NNNNNNNWATGCAAATNNNWNNW	AACATAATATAAAAATAAATTTA
M00138	V$OCT1_04	0.818068	14996	(-)	NNNNNNNWATGCAAATNNNWNNW	ACATAATATAAAAATAAATTTAG
M00162	V$OCT1_06	0.809375	15000	(-)	CWNAWTKWSATRYN	AATATAAAAATAAA
M00138	V$OCT1_04	0.7867	15005	(+)	NNNNNNNWATGCAAATNNNWNNW	AAAAATAAATTTAGAATAAGCAT
M00162	V$OCT1_06	0.852734	15005	(-)	CWNAWTKWSATRYN	AAAAATAAATTTAG
M00138	V$OCT1_04	0.84839	15007	(-)	NNNNNNNWATGCAAATNNNWNNW	AAATAAATTTAGAATAAGCATGA
M00135	V$OCT1_01	0.793972	15009	(-)	NNNNWTATGCAAATNTNNN	ATAAATTTAGAATAAGCAT
M00136	V$OCT1_02	0.813211	15013	(-)	NNGAATATKCANNNN	ATTTAGAATAAGCAT
M00138	V$OCT1_04	0.83187	15013	(-)	NNNNNNNWATGCAAATNNNWNNW	ATTTAGAATAAGCATGACACTTA
M00248	V$OCT1_07	0.793725	15013	(-)	TNTATGNTAATT	ATTTAGAATAAG

M00135	V$OCT1_01	0.7314	15015	(-)	NNNNWTATGCAAATNTNNN	TTAGAATAAGCATGACACT
M00136	V$OCT1_02	0.808879	15016	(+)	NNGAATATKCANNNN	TAGAATAAGCATGAC
M00248	V$OCT1_07	0.810049	15019	(-)	TNTATGNTAATT	AATAAGCATGAC
M00162	V$OCT1_06	0.823828	15022	(+)	CWNAWTKWSATRYN	AAGCATGACACTTA
M00162	V$OCT1_06	0.825	15023	(-)	CWNAWTKWSATRYN	AGCATGACACTTAA
M00162	V$OCT1_06	0.919531	15028	(+)	CWNAWTKWSATRYN	GACACTTAAATGCT
M00162	V$OCT1_06	0.808594	15028	(-)	CWNAWTKWSATRYN	GACACTTAAATGCT
M00135	V$OCT1_01	0.732545	15045	(+)	NNNNWTATGCAAATNTNNN	TGTCCTATGCTAGAATTTT
M00136	V$OCT1_02	0.821873	15052	(-)	NNGAATATKCANNNN	TGCTAGAATTTTCTG
M00138	V$OCT1_04	0.832079	15061	(+)	NNNNNNNWATGCAAATNNNWNNW	TTTCTGAAATGGAAAATTGATGA
M00135	V$OCT1_01	0.749142	15063	(+)	NNNNWTATGCAAATNTNNN	TCTGAAATGGAAAATTGAT
M00162	V$OCT1_06	0.855469	15066	(-)	CWNAWTKWSATRYN	GAAATGGAAAATTG
M00162	V$OCT1_06	0.826562	15085	(+)	CWNAWTKWSATRYN	AACTTTCTGATATA
M00162	V$OCT1_06	0.847656	15087	(+)	CWNAWTKWSATRYN	CTTTCTGATATATG
M00138	V$OCT1_04	0.781263	15097	(+)	NNNNNNNWATGCAAATNNNWNNW	TATGGCTAATGTTAATCCATTAC
M00135	V$OCT1_01	0.754101	15099	(+)	NNNNWTATGCAAATNTNNN	TGGCTAATGTTAATCCATT
M00137	V$OCT1_03	0.852627	15099	(+)	NNNRTAATNANNN	TGGCTAATGTTAA
M00248	V$OCT1_07	0.791605	15102	(+)	TNTATGNTAATT	CTAATGTTAATC
M00137	V$OCT1_03	0.86448	15110	(-)	NNNRTAATNANNN	AATCCATTACTCA
M00162	V$OCT1_06	0.813672	15143	(+)	CWNAWTKWSATRYN	ATCTATTTGATAAG
M00138	V$OCT1_04	0.780845	15147	(+)	NNNNNNNWATGCAAATNNNWNNW	ATTTGATAAGTGATAATCATTCT

M00195	V$OCT1_Q6	0.796397	15152	(+)	NNNNATGCAAATNAN	ATAAGTGATAATCAT
M00137	V$OCT1_03	0.896879	15156	(+)	NNNRTAATNANNN	GTGATAATCATTC
M00138	V$OCT1_04	0.811167	15157	(+)	NNNNNNNWATGCAAATNNNWNNW	TGATAATCATTCTGATGAAAATA
M00138	V$OCT1_04	0.832915	15163	(+)	NNNNNNNWATGCAAATNNNWNNW	TCATTCTGATGAAAATAGGAGGA
M00135	V$OCT1_01	0.739222	15165	(+)	NNNNWTATGCAAATNTNNN	ATTCTGATGAAAATAGGAG
M00138	V$OCT1_04	0.834797	15200	(+)	NNNNNNNWATGCAAATNNNWNNW	AAGTAAAAATTCAACTTTATCCT
M00136	V$OCT1_02	0.82837	15202	(+)	NNGAATATKCANNNN	GTAAAAATTCAACTT
M00138	V$OCT1_04	0.800711	15225	(+)	NNNNNNNWATGCAAATNNNWNNW	TTGCACTTTTGCTAATCTTTAAA
M00135	V$OCT1_01	0.797978	15227	(+)	NNNNWTATGCAAATNTNNN	GCACTTTTGCTAATCTTTA
M00162	V$OCT1_06	0.90625	15238	(+)	CWNAWTKWSATRYN	AATCTTTAAATGCC
M00162	V$OCT1_06	0.813672	15238	(-)	CWNAWTKWSATRYN	AATCTTTAAATGCC
M00162	V$OCT1_06	0.829297	15244	(-)	CWNAWTKWSATRYN	TAAATGCCTTTCTT
M00138	V$OCT1_04	0.837725	15266	(-)	NNNNNNNWATGCAAATNNNWNNW	ACCCATGATATACATTAGAATGC
M00136	V$OCT1_02	0.78262	15279	(+)	NNGAATATKCANNNN	ATTAGAATGCAATGT
M00162	V$OCT1_06	0.855469	15282	(-)	CWNAWTKWSATRYN	AGAATGCAATGTGG
M00162	V$OCT1_06	0.865625	15307	(+)	CWNAWTKWSATRYN	CAGATTTTGACATT
M00162	V$OCT1_06	0.90625	15309	(+)	CWNAWTKWSATRYN	GATTTTGACATTCC
M00162	V$OCT1_06	0.80625	15330	(+)	CWNAWTKWSATRYN	CTAACTTGAAATCC
M00162	V$OCT1_06	0.806641	15332	(-)	CWNAWTKWSATRYN	AACTTGAAATCCTG
M00162	V$OCT1_06	0.834375	15345	(+)	CWNAWTKWSATRYN	GATCTTTTTATTTT
M00138	V$OCT1_04	0.810958	15347	(-)	NNNNNNNWATGCAAATNNNWNNW	TCTTTTTATTTTCGCTTACTTGA
M00138	V$OCT1_04	0.792346	15358	(-)	NNNNNNNWATGCAAATNNNWNNW	TCGCTTACTTGAAATAATATAAT

M00137	V$OCT1_03	0.92256	15373	(+)	NNNRTAATNANNN	AATATAATGATGT
M00138	V$OCT1_04	0.802175	15374	(+)	NNNNNNNWATGCAAATNNNWNNW	ATATAATGATGTTTGTTTTTATA
M00137	V$OCT1_03	0.852232	15386	(-)	NNNRTAATNANNN	TTGTTTTTATAAC
M00162	V$OCT1_06	0.842187	15405	(+)	CWNAWTKWSATRYN	GGTGATTAAATATA
M00138	V$OCT1_04	0.815558	15408	(+)	NNNNNNNWATGCAAATNNNWNNW	GATTAAATATAATAATCTATTAA
M00135	V$OCT1_01	0.750286	15410	(+)	NNNNWTATGCAAATNTNNN	TTAAATATAATAATCTATT
M00162	V$OCT1_06	0.85	15411	(-)	CWNAWTKWSATRYN	TAAATATAATAATC
M00137	V$OCT1_03	0.851837	15413	(+)	NNNRTAATNANNN	AATATAATAATCT
M00138	V$OCT1_04	0.789628	15420	(-)	NNNNNNNWATGCAAATNNNWNNW	TAATCTATTAAGCATACTGCTGG
M00135	V$OCT1_01	0.765357	15422	(-)	NNNNWTATGCAAATNTNNN	ATCTATTAAGCATACTGCT
M00136	V$OCT1_02	0.795344	15426	(-)	NNGAATATKCANNNN	ATTAAGCATACTGCT
M00162	V$OCT1_06	0.929688	15456	(+)	CWNAWTKWSATRYN	CATTTTTTGATTTA
M00138	V$OCT1_04	0.834797	15459	(-)	NNNNNNNWATGCAAATNNNWNNW	TTTTTGATTTAGCAAAATTTTAT
M00135	V$OCT1_01	0.77356	15461	(-)	NNNNWTATGCAAATNTNNN	TTTGATTTAGCAAAATTTT
M00138	V$OCT1_04	0.782518	15461	(+)	NNNNNNNWATGCAAATNNNWNNW	TTTGATTTAGCAAAATTTTATTA
M00135	V$OCT1_01	0.739031	15462	(+)	NNNNWTATGCAAATNTNNN	TTGATTTAGCAAAATTTTA
M00162	V$OCT1_06	0.804687	15471	(+)	CWNAWTKWSATRYN	CAAAATTTTATTAC
M00162	V$OCT1_06	0.839453	15472	(+)	CWNAWTKWSATRYN	AAAATTTTATTACT
M00162	V$OCT1_06	0.834766	15472	(-)	CWNAWTKWSATRYN	AAAATTTTATTACT
M00137	V$OCT1_03	0.881865	15475	(-)	NNNRTAATNANNN	ATTTTATTACTGA
M00248	V$OCT1_07	0.800085	15564	(+)	TNTATGNTAATT	TCTTTGGTAAGT

M00162	V$OCT1_06	0.829297	15577	(+)	CWNAWTKWSATRYN	GGAAATTGCTTGTC
M00195	V$OCT1_Q6	0.794487	15577	(-)	NNNNATGCAAATNAN	GGAAATTGCTTGTCT
M00138	V$OCT1_04	0.818695	15592	(-)	NNNNNNNWATGCAAATNNNWNNW	TGATCCTCTTTTCATATGAATAC
M00135	V$OCT1_01	0.76116	15594	(-)	NNNNWTATGCAAATNTNNN	ATCCTCTTTTCATATGAAT
M00162	V$OCT1_06	0.860547	15596	(+)	CWNAWTKWSATRYN	CCTCTTTTCATATG
M00138	V$OCT1_04	0.851317	15598	(-)	NNNNNNNWATGCAAATNNNWNNW	TCTTTTCATATGAATACAAAGGT
M00162	V$OCT1_06	0.803906	15667	(+)	CWNAWTKWSATRYN	TATATTTAGACACA
M00137	V$OCT1_03	0.843145	15675	(-)	NNNRTAATNANNN	GACACATTACACT
M00162	V$OCT1_06	0.873828	15677	(-)	CWNAWTKWSATRYN	CACATTACACTTAG
M00138	V$OCT1_04	0.836679	15687	(-)	NNNNNNNWATGCAAATNNNWNNW	TTAGATAATATCAATAATTAAAT
M00248	V$OCT1_07	0.791181	15693	(-)	TNTATGNTAATT	AATATCAATAAT
M00137	V$OCT1_03	0.878309	15697	(+)	NNNRTAATNANNN	TCAATAATTAAAT
M00162	V$OCT1_06	0.842578	15699	(+)	CWNAWTKWSATRYN	AATAATTAAATTGG
M00138	V$OCT1_04	0.783354	15701	(-)	NNNNNNNWATGCAAATNNNWNNW	TAATTAAATTGGCTTCAACATTT
M00138	V$OCT1_04	0.806566	15716	(-)	NNNNNNNWATGCAAATNNNWNNW	CAACATTTTTTGTATTATAATAA
M00138	V$OCT1_04	0.829778	15721	(+)	NNNNNNNWATGCAAATNNNWNNW	TTTTTTGTATTATAATAATATCA
M00138	V$OCT1_04	0.833961	15722	(-)	NNNNNNNWATGCAAATNNNWNNW	TTTTTGTATTATAATAATATCAA
M00138	V$OCT1_04	0.781472	15724	(+)	NNNNNNNWATGCAAATNNNWNNW	TTTGTATTATAATAATATCAAAT
M00138	V$OCT1_04	0.80343	15725	(-)	NNNNNNNWATGCAAATNNNWNNW	TTGTATTATAATAATATCAAATT
M00135	V$OCT1_01	0.757535	15726	(+)	NNNNWTATGCAAATNTNNN	TGTATTATAATAATATCAA
M00162	V$OCT1_06	0.84492	15726	(-)	CWNAWTKWSATRYN	TGTATTATAATAAT

		2				
M00137	V$OCT1_03	0.910707	15729	(+)	NNNRTAATNANNN	ATTATAATAATAT
M00138	V$OCT1_04	0.822041	15730	(+)	NNNNNNNWATGCAAATNNNWNNW	TTATAATAATATCAAATTTAAGT
M00162	V$OCT1_06	0.886328	15737	(-)	CWNAWTKWSATRYN	AATATCAAATTTAA
M00138	V$OCT1_04	0.813676	15738	(-)	NNNNNNNWATGCAAATNNNWNNW	ATATCAAATTTAAGTAACAATAA
M00162	V$OCT1_06	0.891016	15742	(-)	CWNAWTKWSATRYN	CAAATTTAAGTAAC
M00138	V$OCT1_04	0.837097	15762	(+)	NNNNNNNWATGCAAATNNNWNNW	ACTAGCTAATTTTAATTAATATA
M00138	V$OCT1_04	0.814513	15763	(-)	NNNNNNNWATGCAAATNNNWNNW	CTAGCTAATTTTAATTAATATAA
M00162	V$OCT1_06	0.858203	15767	(+)	CWNAWTKWSATRYN	CTAATTTTAATTAA
M00195	V$OCT1_Q6	0.802402	15767	(-)	NNNNATGCAAATNAN	CTAATTTTAATTAAT
M00248	V$OCT1_07	0.830825	15767	(+)	TNTATGNTAATT	CTAATTTTAATT
M00138	V$OCT1_04	0.826432	15768	(+)	NNNNNNNWATGCAAATNNNWNNW	TAATTTTAATTAATATAATTTAT
M00162	V$OCT1_06	0.834375	15768	(-)	CWNAWTKWSATRYN	TAATTTTAATTAAT
M00138	V$OCT1_04	0.83417	15769	(-)	NNNNNNNWATGCAAATNNNWNNW	AATTTTAATTAATATAATTTATT
M00135	V$OCT1_01	0.749142	15771	(-)	NNNNWTATGCAAATNTNNN	TTTTAATTAATATAATTTA
M00138	V$OCT1_04	0.797783	15773	(-)	NNNNNNNWATGCAAATNNNWNNW	TTAATTAATATAATTTATTATAT
M00248	V$OCT1_07	0.872165	15775	(-)	TNTATGNTAATT	AATTAATATAAT
M00162	V$OCT1_06	0.839844	15776	(+)	CWNAWTKWSATRYN	ATTAATATAATTTA
M00137	V$OCT1_03	0.858159	15784	(-)	NNNRTAATNANNN	AATTTATTATATT
M00138	V$OCT1_04	0.844835	15786	(+)	NNNNNNNWATGCAAATNNNWNNW	TTTATTATATTGAAATTTCTACA
M00138	V$OCT1_04	0.81451	15787	(-)	NNNNNNNWATGCAAATNNNW	TTATTATATTGAAATTTCTACAC

		3			NNW	
M00135	V$OCT1_01	0.784052	15788	(+)	NNNNWTATGCAAATNTNNN	TATTATATTGAAATTTCTA
M00162	V$OCT1_06	0.855469	15791	(+)	CWNAWTKWSATRYN	TATATTGAAATTTC
M00162	V$OCT1_06	0.860547	15791	(-)	CWNAWTKWSATRYN	TATATTGAAATTTC
M00248	V$OCT1_07	0.908416	15791	(+)	TNTATGNTAATT	TATATTGAAATT
M00137	V$OCT1_03	0.86448	15862	(-)	NNNRTAATNANNN	TATTCTTTATGAT
M00162	V$OCT1_06	0.80625	15873	(+)	CWNAWTKWSATRYN	ATCACTGGAATAGA
M00138	V$OCT1_04	0.790882	15874	(+)	NNNNNNNWATGCAAATNNNWNNW	TCACTGGAATAGAAATACTTAAA
M00162	V$OCT1_06	0.826562	15879	(+)	CWNAWTKWSATRYN	GGAATAGAAATACT
M00138	V$OCT1_04	0.79381	15880	(+)	NNNNNNNWATGCAAATNNNWNNW	GAATAGAAATACTTAAAAAGTCA
M00138	V$OCT1_04	0.782936	15882	(+)	NNNNNNNWATGCAAATNNNWNNW	ATAGAAATACTTAAAAAGTCACA
M00162	V$OCT1_06	0.808203	15896	(-)	CWNAWTKWSATRYN	AAAGTCACATAGTT
M00162	V$OCT1_06	0.821094	15909	(-)	CWNAWTKWSATRYN	TAAATCAAGTATTT
M00138	V$OCT1_04	0.78189	15935	(+)	NNNNNNNWATGCAAATNNNWNNW	GAAAACAGATTTAAATGTCCATA
M00138	V$OCT1_04	0.79611	15936	(-)	NNNNNNNWATGCAAATNNNWNNW	AAAACAGATTTAAATGTCCATAG
M00162	V$OCT1_06	0.945703	15940	(+)	CWNAWTKWSATRYN	CAGATTTAAATGTC
M00162	V$OCT1_06	0.842578	15940	(-)	CWNAWTKWSATRYN	CAGATTTAAATGTC
M00135	V$OCT1_01	0.740939	15944	(-)	NNNNWTATGCAAATNTNNN	TTTAAATGTCCATAGATGT
M00248	V$OCT1_07	0.874073	15948	(-)	TNTATGNTAATT	AATGTCCATAGA
M00138	V$OCT1_04	0.785236	15957	(-)	NNNNNNNWATGCAAATNNNWNNW	AGATGTGTTTTGAGTACTTCAGT
M00162	V$OCT1_06	0.806641	15992	(-)	CWNAWTKWSATRYN	AGCTTGTAGTTGTG

M00162	V$OCT1_06	0.852734	16053	(-)	CWNAWTKWSATRYN	CAGATCAAAAGAAG
M00162	V$OCT1_06	0.803125	16066	(-)	CWNAWTKWSATRYN	GGAAAGTAAAAGTC
M00162	V$OCT1_06	0.893359	16075	(+)	CWNAWTKWSATRYN	AAGTCTGACATACG
M00138	V$OCT1_04	0.827687	16094	(-)	NNNNNNNWATGCAAATNNNWNNW	GTTTTTACTTTGAATTATTTTGC
M00195	V$OCT1_Q6	0.794487	16098	(-)	NNNNATGCAAATNAN	TTACTTTGAATTATT
M00136	V$OCT1_02	0.77098	16100	(-)	NNGAATATKCANNNN	ACTTTGAATTATTTT
M00138	V$OCT1_04	0.788791	16104	(-)	NNNNNNNWATGCAAATNNNWNNW	TGAATTATTTTGCCATATTTTCT
M00135	V$OCT1_01	0.732926	16107	(-)	NNNNWTATGCAAATNTNNN	ATTATTTTGCCATATTTTC
M00162	V$OCT1_06	0.855078	16156	(+)	CWNAWTKWSATRYN	ATAAATTGCCTGTA
M00138	V$OCT1_04	0.781681	16157	(+)	NNNNNNNWATGCAAATNNNWNNW	TAAATTGCCTGTAAAACTTAAAA
M00162	V$OCT1_06	0.836719	16162	(-)	CWNAWTKWSATRYN	TGCCTGTAAAACTT
M00162	V$OCT1_06	0.821484	16169	(+)	CWNAWTKWSATRYN	AAAACTTAAAAATC
M00162	V$OCT1_06	0.808203	16169	(-)	CWNAWTKWSATRYN	AAAACTTAAAAATC
M00195	V$OCT1_Q6	0.806223	16170	(+)	NNNNATGCAAATNAN	AAACTTAAAAATCAG
M00162	V$OCT1_06	0.833203	16215	(-)	CWNAWTKWSATRYN	CACATATAACTTTG
M00162	V$OCT1_06	0.804297	16234	(-)	CWNAWTKWSATRYN	TTCATCTTAAGATG
M00162	V$OCT1_06	0.804297	16236	(+)	CWNAWTKWSATRYN	CATCTTAAGATGAA
M00138	V$OCT1_04	0.787118	16248	(+)	NNNNNNNWATGCAAATNNNWNNW	AAGAGAAAATGACTATTAAAGTG
M00136	V$OCT1_02	0.79372	16253	(-)	NNGAATATKCANNNN	AAAATGACTATTAAA
M00138	V$OCT1_04	0.795901	16254	(+)	NNNNNNNWATGCAAATNNNWNNW	AAATGACTATTAAAGTGTATCTT
M00136	V$OCT1_02	0.772604	16256	(+)	NNGAATATKCANNNN	ATGACTATTAAAGTG

M00136	V$OCT1_02	0.77856	16295	(+)	NNGAATATKCANNNN	CAAAACATGCAGCAT
M00136	V$OCT1_02	0.818354	16313	(-)	NNGAATATKCANNNN	TAACTACATATTTCT
M00162	V$OCT1_06	0.842187	16319	(-)	CWNAWTKWSATRYN	CATATTTCTTTTTT
M00138	V$OCT1_04	0.799665	16327	(-)	NNNNNNNWATGCAAATNNNWNNW	TTTTTTGATTTACAGATGGGATT
M00135	V$OCT1_01	0.75124	16329	(-)	NNNNWTATGCAAATNTNNN	TTTTGATTTACAGATGGGA
M00195	V$OCT1_Q6	0.790666	16331	(-)	NNNNATGCAAATNAN	TTGATTTACAGATGG
M00195	V$OCT1_Q6	0.817959	16344	(+)	NNNNATGCAAATNAN	GGGATTGAAAATTCC
M00136	V$OCT1_02	0.799134	16345	(-)	NNGAATATKCANNNN	GGATTGAAAATTCCA
M00162	V$OCT1_06	0.813672	16345	(-)	CWNAWTKWSATRYN	GGATTGAAAATTCC
M00135	V$OCT1_01	0.737123	16348	(-)	NNNNWTATGCAAATNTNNN	TTGAAAATTCCATGCTCTA
M00136	V$OCT1_02	0.783162	16348	(+)	NNGAATATKCANNNN	TTGAAAATTCCATGC
M00162	V$OCT1_06	0.896875	16350	(+)	CWNAWTKWSATRYN	GAAAATTCCATGCT
M00162	V$OCT1_06	0.872656	16351	(-)	CWNAWTKWSATRYN	AAAATTCCATGCTC
M00248	V$OCT1_07	0.800721	16352	(-)	TNTATGNTAATT	AAATTCCATGCT
M00162	V$OCT1_06	0.816016	16359	(+)	CWNAWTKWSATRYN	ATGCTCTACATGTC
M00195	V$OCT1_Q6	0.813319	16359	(-)	NNNNATGCAAATNAN	ATGCTCTACATGTCT
M00162	V$OCT1_06	0.834375	16364	(+)	CWNAWTKWSATRYN	CTACATGTCTTTTC
M00162	V$OCT1_06	0.806641	16365	(-)	CWNAWTKWSATRYN	TACATGTCTTTTCA
M00138	V$OCT1_04	0.781472	16415	(-)	NNNNNNNWATGCAAATNNNWNNW	TTGCTGTTTTAGAATAGGACAAT
M00162	V$OCT1_06	0.847266	16427	(-)	CWNAWTKWSATRYN	AATAGGACAATCTC
M00162	V$OCT1_06	0.860547	16433	(-)	CWNAWTKWSATRYN	ACAATCTCAAATTG

M00162	V$OCT1_06	0.83476<br>6	16470	(+)	CWNAWTKWSATRYN	CTCTATTGTATTTT
M00248	V$OCT1_07	0.83930<br>5	16471	(+)	TNTATGNTAATT	TCTATTGTATTT
M00162	V$OCT1_06	0.84765<br>6	16476	(-)	CWNAWTKWSATRYN	TGTATTTTAGATTG
M00162	V$OCT1_06	0.82656<br>2	16477	(+)	CWNAWTKWSATRYN	GTATTTTAGATTGT
M00162	V$OCT1_06	0.84218<br>7	16501	(-)	CWNAWTKWSATRYN	TAAGTGAAATTGTC
M00162	V$OCT1_06	0.80078<br>1	16525	(+)	CWNAWTKWSATRYN	GTTACCTAAATTCC
M00162	V$OCT1_06	0.81406<br>2	16531	(-)	CWNAWTKWSATRYN	TAAATTCCAGTAGT
M00162	V$OCT1_06	0.82929<br>7	16576	(+)	CWNAWTKWSATRYN	GGGAATTACAGTCT
M00162	V$OCT1_06	0.87812<br>5	16577	(-)	CWNAWTKWSATRYN	GGAATTACAGTCTT
M00137	V$OCT1_03	0.84037<br>9	16587	(-)	NNNRTAATNANNN	TCTTCATTAAATT
M00162	V$OCT1_06	0.91171<br>9	16588	(+)	CWNAWTKWSATRYN	CTTCATTAAATTTC
M00162	V$OCT1_06	0.84765<br>6	16589	(-)	CWNAWTKWSATRYN	TTCATTAAATTTCT
M00138	V$OCT1_04	0.83375<br>2	16590	(-)	NNNNNNNWATGCAAATNNNW<br>NNW	TCATTAAATTTCTATATGGAAAA
M00135	V$OCT1_01	0.80885<br>2	16592	(-)	NNNNWTATGCAAATNTNNN	ATTAAATTTCTATATGGAA
M00162	V$OCT1_06	0.86054<br>7	16594	(-)	CWNAWTKWSATRYN	TAAATTTCTATATG
M00248	V$OCT1_07	0.82552<br>5	16596	(-)	TNTATGNTAATT	AATTTCTATATG
M00138	V$OCT1_04	0.80008<br>4	16597	(+)	NNNNNNNWATGCAAATNNNW<br>NNW	ATTTCTATATGGAAAATGTTTTT
M00135	V$OCT1_01	0.80713<br>5	16599	(+)	NNNNWTATGCAAATNTNNN	TTCTATATGGAAAATGTTT
M00162	V$OCT1_06	0.81367<br>2	16601	(+)	CWNAWTKWSATRYN	CTATATGGAAAATG
M00248	V$OCT1_07	0.86919<br>7	16602	(+)	TNTATGNTAATT	TATATGGAAAAT

M00162	V$OCT1_06	0.867969	16651	(+)	CWNAWTKWSATRYN	CATCATTTCAAATA
M00195	V$OCT1_Q6	0.810044	16652	(+)	NNNNATGCAAATNAN	ATCATTTCAAATAAC
M00162	V$OCT1_06	0.800391	16654	(-)	CWNAWTKWSATRYN	CATTTCAAATAACT
M00162	V$OCT1_06	0.916797	16675	(+)	CWNAWTKWSATRYN	GTAACTGAGATTTT
M00162	V$OCT1_06	0.804297	16676	(-)	CWNAWTKWSATRYN	TAACTGAGATTTTG
M00162	V$OCT1_06	0.821094	16683	(+)	CWNAWTKWSATRYN	GATTTTGTCTTTCT
M00138	V$OCT1_04	0.7867	16692	(-)	NNNNNNNWATGCAAATNNNWNNW	TTTCTTCTTTTCAATAAATTACA
M00162	V$OCT1_06	0.945312	16705	(+)	CWNAWTKWSATRYN	ATAAATTACATTTT
M00195	V$OCT1_Q6	0.818231	16705	(-)	NNNNATGCAAATNAN	ATAAATTACATTTTA
M00162	V$OCT1_06	0.868359	16706	(-)	CWNAWTKWSATRYN	TAAATTACATTTTA
M00248	V$OCT1_07	0.793089	16707	(-)	TNTATGNTAATT	AAATTACATTTT
M00059	V$YY1_01	0.797837	56	(+)	NNNNNCCATNTWNNNWN	TGTATACATATAAAAAA
M00059	V$YY1_01	0.809633	190	(-)	NNNNNCCATNTWNNNWN	ATTTGCAAATGTTGATA
M00059	V$YY1_01	0.884666	263	(+)	NNNNNCCATNTWNNNWN	CCTGATCATTTTTAAAA
M00059	V$YY1_01	0.848952	292	(-)	NNNNNCCATNTWNNNWN	TACTGAAAATGTAGATA
M00059	V$YY1_01	0.801114	362	(-)	NNNNNCCATNTWNNNWN	TCTGTAAAATGTTGTCA
M00059	V$YY1_01	0.855505	414	(+)	NNNNNCCATNTWNNNWN	GAAGATCATTTCTGTAG
M00069	V$YY1_02	0.855052	437	(+)	NNNCGGCCATCTTGNCTSNW	TCGAGGCCATTTTTATTCCA
M00059	V$YY1_01	0.886959	438	(+)	NNNNNCCATNTWNNNWN	CGAGGCCATTTTTATTC
M00059	V$YY1_01	0.794561	449	(+)	NNNNNCCATNTWNNNWN	TTATTCCATGTAATTAG

M00059	V$YY1_01	0.774574	512	(-)	NNNNNCCATNTWNNNWN	AGATATAAATGATTACT
M00059	V$YY1_01	0.790301	566	(-)	NNNNNCCATNTWNNNWN	ACAAATATATGAAAATA
M00059	V$YY1_01	0.780799	600	(-)	NNNNNCCATNTWNNNWN	TTATAAATATGTCTTTT
M00059	V$YY1_01	0.79194	616	(-)	NNNNNCCATNTWNNNWN	TTTAACAAATGCAGGTA
M00059	V$YY1_01	0.833879	706	(+)	NNNNNCCATNTWNNNWN	AAATTTCATGTATAATC
M00059	V$YY1_01	0.863041	742	(+)	NNNNNCCATNTWNNNWN	GAAATGCATTTTTTCAG
M00059	V$YY1_01	0.813565	849	(+)	NNNNNCCATNTWNNNWN	CATCTTCATACAGTCAT
M00059	V$YY1_01	0.780472	861	(-)	NNNNNCCATNTWNNNWN	GTCATGTCATGGATATA
M00059	V$YY1_01	0.797837	951	(-)	NNNNNCCATNTWNNNWN	CACAAACAATGCTTTTC
M00059	V$YY1_01	0.814875	998	(+)	NNNNNCCATNTWNNNWN	TAAAGACATCTTTTAAA
M00059	V$YY1_01	0.782765	1038	(+)	NNNNNCCATNTWNNNWN	ATTTACCAGTTTTAGTT
M00059	V$YY1_01	0.863041	1105	(+)	NNNNNCCATNTWNNNWN	TATTGTCATATTTTAAA
M00059	V$YY1_01	0.777195	1296	(-)	NNNNNCCATNTWNNNWN	GAAACTAAATGAATAAA
M00059	V$YY1_01	0.790301	1821	(+)	NNNNNCCATNTWNNNWN	TGGGTCCATTATAGGCT
M00059	V$YY1_01	0.817169	1835	(+)	NNNNNCCATNTWNNNWN	GCTTAACATATTTGTAA
M00059	V$YY1_01	0.831586	1927	(+)	NNNNNCCATNTWNNNWN	CAACACCATAGACAGCC
M00059	V$YY1_01	0.780144	1980	(-)	NNNNNCCATNTWNNNWN	CAAGAACACTGGAGTGG
M00059	V$YY1_01	0.783093	1996	(+)	NNNNNCCATNTWNNNWN	GGTTGCCATTTCCTTCT
M00059	V$YY1_01	0.801114	2069	(-)	NNNNNCCATNTWNNNWN	GCGACTCCATGGACTGC
M00059	V$YY1_01	0.783421	2070	(+)	NNNNNCCATNTWNNNWN	CGACTCCATGGACTGCA
M00059	V$YY1_01	0.81323	2100	(-)	NNNNNCCATNTWNNNWN	CTCCATCCATGGGATTT

		7				
M00059	V$YY1_01	0.78768	2190	(+)	NNNNNCCATNTWNNNWN	TGTATCCACTTTTGGAA
M00059	V$YY1_01	0.809961	2365	(+)	NNNNNCCATNTWNNNWN	CAAATTCAGTTATTAAT
M00059	V$YY1_01	0.813893	2527	(-)	NNNNNCCATNTWNNNWN	TTTCTGAAATGCTGAGA
M00059	V$YY1_01	0.806356	2719	(+)	NNNNNCCATNTWNNNWN	GTAGTCCATATCTTTTC
M00059	V$YY1_01	0.906291	2799	(+)	NNNNNCCATNTWNNNWN	AAACATCATTTTTTAAT
M00059	V$YY1_01	0.787025	2815	(+)	NNNNNCCATNTWNNNWN	TGCTAACATTTAACAAA
M00059	V$YY1_01	0.827326	2849	(-)	NNNNNCCATNTWNNNWN	CAGTTAAAATGCAGATT
M00059	V$YY1_01	0.795216	3202	(+)	NNNNNCCATNTWNNNWN	TCTCTCCAGTGAGTGTT
M00059	V$YY1_01	0.800786	3317	(+)	NNNNNCCATNTWNNNWN	AACAGCCAGTTTCACAT
M00069	V$YY1_02	0.775437	3394	(-)	NNNCGGCCATCTTGNCTSNW	TTTAGAGAAGAGGGCATTTG
M00059	V$YY1_01	0.836828	3492	(+)	NNNNNCCATNTWNNNWN	TATTTTCATGTATTATT
M00059	V$YY1_01	0.797182	3554	(+)	NNNNNCCATNTWNNNWN	GATCTCAATTTACACAC
M00059	V$YY1_01	0.852228	3640	(+)	NNNNNCCATNTWNNNWN	TTAGTTCATTTTTTAAT
M00059	V$YY1_01	0.869266	3755	(+)	NNNNNCCATNTWNNNWN	CATTTCCATTTTCATTT
M00059	V$YY1_01	0.800131	3761	(+)	NNNNNCCATNTWNNNWN	CATTTTCATTTGTATAC
M00059	V$YY1_01	0.807339	3790	(+)	NNNNNCCATNTWNNNWN	TGTATCCAGGTACTCAG
M00059	V$YY1_01	0.90924	3916	(-)	NNNNNCCATNTWNNNWN	AACCAAACATGGTGTCT
M00069	V$YY1_02	0.753124	3916	(+)	NNNCGGCCATCTTGNCTSNW	AACCAAACATGGTGTCTCCT
M00059	V$YY1_01	0.853539	4009	(-)	NNNNNCCATNTWNNNWN	CTAAATAAATGGAGAAT
M00059	V$YY1_01	0.794233	4028	(-)	NNNNNCCATNTWNNNWN	GTGTTCAAATGGAAAAA

M00059	V$YY1_01	0.779817	4045	(+)	NNNNNCCATNTWNNNWN	CATTCTCCTTTTTTGAC
M00059	V$YY1_01	0.796855	4093	(-)	NNNNNCCATNTWNNNWN	ATTCCTACAGGAAGTCC
M00059	V$YY1_01	0.783093	4239	(+)	NNNNNCCATNTWNNNWN	TTTCTTCATTTGCTCAC
M00059	V$YY1_01	0.820118	4280	(-)	NNNNNCCATNTWNNNWN	AATTATATATGCAAATA
M00059	V$YY1_01	0.792595	4391	(+)	NNNNNCCATNTWNNNWN	AATTTCCATTCTCATTA
M00059	V$YY1_01	0.79751	4509	(+)	NNNNNCCATNTWNNNWN	CAAGACCAGTATTTAAA
M00059	V$YY1_01	0.783093	4665	(-)	NNNNNCCATNTWNNNWN	TGTTATTTATGGTATTA
M00059	V$YY1_01	0.789646	4856	(+)	NNNNNCCATNTWNNNWN	ACTGACCAGCTTAAATA
M00059	V$YY1_01	0.801114	4896	(-)	NNNNNCCATNTWNNNWN	CGCAAGAAATGATATTG
M00059	V$YY1_01	0.783421	4935	(+)	NNNNNCCATNTWNNNWN	AAAAAGCATATTTATTT
M00059	V$YY1_01	0.836501	4992	(+)	NNNNNCCATNTWNNNWN	GATATCCATCCATTCAT
M00059	V$YY1_01	0.797182	4996	(+)	NNNNNCCATNTWNNNWN	TCCATCCATTCATGTTT
M00059	V$YY1_01	0.780799	5035	(+)	NNNNNCCATNTWNNNWN	GGAGAACATCAATGAAC
M00059	V$YY1_01	0.800786	5038	(-)	NNNNNCCATNTWNNNWN	GAACATCAATGAACTGA
M00059	V$YY1_01	0.839122	5065	(-)	NNNNNCCATNTWNNNWN	GAACATAAATGATATTT
M00059	V$YY1_01	0.849279	5218	(-)	NNNNNCCATNTWNNNWN	GTCGAAAAATGCTTTGT
M00059	V$YY1_01	0.82962	5242	(+)	NNNNNCCATNTWNNNWN	AAAAAACATTTTAGAAA
M00069	V$YY1_02	0.752588	5291	(-)	NNNCGGCCATCTTGNCTSNW	AACTTTCAAAATGACTGATG
M00059	V$YY1_01	0.787353	5293	(-)	NNNNNCCATNTWNNNWN	CTTTCAAAATGACTGAT
M00059	V$YY1_01	0.776868	5325	(-)	NNNNNCCATNTWNNNWN	TTCTAAATATTGATTTC
M00059	V$YY1_01	0.798165	5387	(-)	NNNNNCCATNTWNNNWN	ATGAATGAATGGATAAA

M00059	V$YY1_01	0.869921	5415	(-)	NNNNNCCATNTWNNNWN	GTGCAATTATGGACTGA
M00059	V$YY1_01	0.770642	5502	(-)	NNNNNCCATNTWNNNWN	CTCCAAGAATGTTTTAC
M00059	V$YY1_01	0.787025	5848	(-)	NNNNNCCATNTWNNNWN	GATGCAACATGTATGTT
M00059	V$YY1_01	0.814548	5940	(-)	NNNNNCCATNTWNNNWN	GTACTGCTATGGTGATG
M00059	V$YY1_01	0.815858	5953	(-)	NNNNNCCATNTWNNNWN	GATGGAAACTGAACACA
M00059	V$YY1_01	0.817824	5971	(-)	NNNNNCCATNTWNNNWN	CAAACTACATGGAGAAA
M00059	V$YY1_01	0.811927	5985	(+)	NNNNNCCATNTWNNNWN	AAAATTCATTTTGCTTA
M00059	V$YY1_01	0.806684	6155	(+)	NNNNNCCATNTWNNNWN	TCAAAACATATAAAAAT
M00059	V$YY1_01	0.783421	6191	(-)	NNNNNCCATNTWNNNWN	GCTCTGAAATGCAGATT
M00059	V$YY1_01	0.777523	6259	(+)	NNNNNCCATNTWNNNWN	CCTTACCAGTTCTCTAT
M00059	V$YY1_01	0.781127	6277	(+)	NNNNNCCATNTWNNNWN	GCCACCCATTTCTATTT
M00059	V$YY1_01	0.847641	6299	(+)	NNNNNCCATNTWNNNWN	GGCATCCATTTTATTTG
M00059	V$YY1_01	0.839122	6318	(+)	NNNNNCCATNTWNNNWN	AATTATCATTTTATATT
M00059	V$YY1_01	0.79882	6385	(+)	NNNNNCCATNTWNNNWN	TCAAGCCATGGAAGATG
M00059	V$YY1_01	0.793906	6391	(-)	NNNNNCCATNTWNNNWN	CATGGAAGATGCTAAGG
M00059	V$YY1_01	0.778834	6626	(-)	NNNNNCCATNTWNNNWN	AAAGCAAAATGTGCATG
M00059	V$YY1_01	0.929882	6646	(-)	NNNNNCCATNTWNNNWN	GTTTAAAAATGAACATC
M00059	V$YY1_01	0.866972	6889	(+)	NNNNNCCATNTWNNNWN	CATGTCCATTCAGTCAG
M00059	V$YY1_01	0.778178	7065	(+)	NNNNNCCATNTWNNNWN	TGACTTCCTGTAGTGAT
M00059	V$YY1_01	0.832569	7127	(+)	NNNNNCCATNTWNNNWN	GGAGTTCATCTTTCAGT
M00059	V$YY1_01	0.78112	7166	(-)	NNNNNCCATNTWNNNWN	TACTGTTCATGGGGTTC

		7				
M00059	V$YY1_01	0.852556	7210	(+)	NNNNNCCATNTWNNNWN	CCATTCCATTCTCCAAT
M00059	V$YY1_01	0.816841	7244	(-)	NNNNNCCATNTWNNNWN	CTCAAAACATGAAAAAA
M00059	V$YY1_01	0.789318	7312	(+)	NNNNNCCATNTWNNNWN	TTGCTGCATATTAGAAT
M00059	V$YY1_01	0.780472	7333	(-)	NNNNNCCATNTWNNNWN	ATCTTAGTATGTACTTG
M00059	V$YY1_01	0.835518	7361	(+)	NNNNNCCATNTWNNNWN	CAGATCCATATGTAAAT
M00059	V$YY1_01	0.893512	7370	(-)	NNNNNCCATNTWNNNWN	ATGTAAATATGAACATC
M00059	V$YY1_01	0.804718	7377	(+)	NNNNNCCATNTWNNNWN	TATGAACATCTATGACT
M00059	V$YY1_01	0.855505	7449	(+)	NNNNNCCATNTWNNNWN	TAGTTCCATTCTGAAAT
M00059	V$YY1_01	0.863041	7475	(+)	NNNNNCCATNTWNNNWN	TATTTTCATATTTCCAA
M00059	V$YY1_01	0.820118	7577	(+)	NNNNNCCATNTWNNNWN	TCATTCCAGGTTTCATT
M00059	V$YY1_01	0.782438	7698	(+)	NNNNNCCATNTWNNNWN	CTCCAACATATTTTAAA
M00059	V$YY1_01	0.793578	7797	(+)	NNNNNCCATNTWNNNWN	GCAATCCAGTATGAAAG
M00059	V$YY1_01	0.785059	7799	(-)	NNNNNCCATNTWNNNWN	AATCCAGTATGAAAGTG
M00059	V$YY1_01	0.795872	7853	(-)	NNNNNCCATNTWNNNWN	CAGTAACAATGATTCTC
M00059	V$YY1_01	0.798493	7882	(-)	NNNNNCCATNTWNNNWN	AATGAAAGCTGGAAGCA
M00059	V$YY1_01	0.794889	7913	(+)	NNNNNCCATNTWNNNWN	GTATGCCATTTTTATGT
M00059	V$YY1_01	0.843381	7945	(-)	NNNNNCCATNTWNNNWN	ATTAGAAAATGTTTATG
M00059	V$YY1_01	0.84633	8126	(-)	NNNNNCCATNTWNNNWN	AATCCTATCTGGATATC
M00059	V$YY1_01	0.86599	8149	(-)	NNNNNCCATNTWNNNWN	ATCCAAACATGATTTTT
M00059	V$YY1_01	0.813237	8220	(-)	NNNNNCCATNTWNNNWN	TTACAATAATGGCTATG

M00059	V$YY1_01	0.770315	8410	(+)	NNNNNCCATNTWNNNWN	TAAACCCAGTTCCAATG
M00059	V$YY1_01	0.79194	8501	(-)	NNNNNCCATNTWNNNWN	AAGAGAACATGTACTTT
M00059	V$YY1_01	0.808322	8558	(+)	NNNNNCCATNTWNNNWN	TAAAATCAGTTTACCAC
M00059	V$YY1_01	0.77228	8576	(-)	NNNNNCCATNTWNNNWN	GATAAGCTATGATGTGT
M00059	V$YY1_01	0.783421	8643	(+)	NNNNNCCATNTWNNNWN	ACAATACATTTTTTTTT
M00059	V$YY1_01	0.802425	8721	(+)	NNNNNCCATNTWNNNWN	TAACATCATCCAGGATA
M00059	V$YY1_01	0.809633	8788	(-)	NNNNNCCATNTWNNNWN	CATTCAAAAGGAAGATG
M00059	V$YY1_01	0.797837	8880	(-)	NNNNNCCATNTWNNNWN	GAGCAAGAATGTTGTGT
M00059	V$YY1_01	0.812582	9080	(-)	NNNNNCCATNTWNNNWN	GTATTGAAATGGCACGC
M00059	V$YY1_01	0.784731	9203	(-)	NNNNNCCATNTWNNNWN	GTGACAAAATAGTAATA
M00059	V$YY1_01	0.814875	9274	(-)	NNNNNCCATNTWNNNWN	CTCGGTAAATGTGGCTA
M00059	V$YY1_01	0.859109	9298	(+)	NNNNNCCATNTWNNNWN	AAACATCATTTTTTCCC
M00069	V$YY1_02	0.767226	9347	(-)	NNNCGGCCATCTTGNCTSNW	CAGAAAACAAATGGTGCTAG
M00059	V$YY1_01	0.83945	9349	(-)	NNNNNCCATNTWNNNWN	GAAAACAAATGGTGCTA
M00059	V$YY1_01	0.859109	9473	(+)	NNNNNCCATNTWNNNWN	GAATTCCATAGACAAAG
M00059	V$YY1_01	0.783421	9503	(+)	NNNNNCCATNTWNNNWN	TATATCCATAGGATCAC
M00059	V$YY1_01	0.87287	9726	(+)	NNNNNCCATNTWNNNWN	CATGTCCATTCAGTGAT
M00059	V$YY1_01	0.810616	10091	(+)	NNNNNCCATNTWNNNWN	GGATACCATGATAGATG
M00069	V$YY1_02	0.810782	10095	(-)	NNNCGGCCATCTTGNCTSNW	ACCATGATAGATGGTATCCA
M00059	V$YY1_01	0.885321	10097	(-)	NNNNNCCATNTWNNNWN	CATGATAGATGGTATCC
M00059	V$YY1_01	0.802097	10138	(-)	NNNNNCCATNTWNNNWN	CTTTGAATAGGAACAGC
M00059	V$YY1_01	0.81487	10272	(+)	NNNNNCCATNTWNNNWN	TCAAATCATGTTAGAGC

		5				
M00059	V$YY1_01	0.78800 8	10338	(+)	NNNNNCCATNTWNNNWN	CAAATCCAGTTCTGAGT
M00059	V$YY1_01	0.87188 7	10407	(+)	NNNNNCCATNTWNNNWN	ACATTCCATTTAAAAAA
M00059	V$YY1_01	0.79849 3	10451	(+)	NNNNNCCATNTWNNNWN	GGATCCCATACATTCAA
M00059	V$YY1_01	0.78866 3	10804	(-)	NNNNNCCATNTWNNNWN	AATAATACATGTTTTTT
M00059	V$YY1_01	0.83256 9	10909	(-)	NNNNNCCATNTWNNNWN	TTTGAACTATGAAGTCA
M00059	V$YY1_01	0.83387 9	10937	(-)	NNNNNCCATNTWNNNWN	CTGATAGTATGGATAGA
M00059	V$YY1_01	0.78407 6	11031	(+)	NNNNNCCATNTWNNNWN	GGAGGCCATAAATTTTT
M00059	V$YY1_01	0.82175 6	11116	(+)	NNNNNCCATNTWNNNWN	AAATTACATTTTCACAT
M00059	V$YY1_01	0.78211	11164	(-)	NNNNNCCATNTWNNNWN	AGTCAATAATGAATTTA
M00059	V$YY1_01	0.81094 4	11334	(-)	NNNNNCCATNTWNNNWN	GTTATGATATGATTAGG
M00059	V$YY1_01	0.81815 2	11371	(-)	NNNNNCCATNTWNNNWN	AGAACTATATGGTCCTG
M00059	V$YY1_01	0.78473 1	11396	(+)	NNNNNCCATNTWNNNWN	GTTCATCATTCATAGTG
M00069	V$YY1_02	0.78668 3	11441	(-)	NNNCGGCCATCTTGNCTSNW	TATTTCATAGATGGTCCCTA
M00059	V$YY1_01	0.87385 3	11443	(-)	NNNNNCCATNTWNNNWN	TTTCATAGATGGTCCCT
M00059	V$YY1_01	0.77686 8	11456	(+)	NNNNNCCATNTWNNNWN	CCCTAGCATTTAGCATA
M00059	V$YY1_01	0.87090 4	11574	(+)	NNNNNCCATNTWNNNWN	CATGACCATATAAGACT
M00059	V$YY1_01	0.77162 5	11584	(+)	NNNNNCCATNTWNNNWN	TAAGACTATTTACTAAA
M00059	V$YY1_01	0.85550 5	11702	(+)	NNNNNCCATNTWNNNWN	ACTACCCATATTTGATA
M00059	V$YY1_01	0.80275 2	11741	(-)	NNNNNCCATNTWNNNWN	AAGATAAAATGTAAATG
M00059	V$YY1_01	0.77260 8	11817	(-)	NNNNNCCATNTWNNNWN	CCCCAGATATGAAACTT

M00059	V$YY1_01	0.847313	11896	(-)	NNNNNCCATNTWNNNWN	GTTAGAAAATGAAAGCT
M00059	V$YY1_01	0.808322	11958	(+)	NNNNNCCATNTWNNNWN	AATCTTCATAGATGATA
M00059	V$YY1_01	0.800786	11961	(-)	NNNNNCCATNTWNNNWN	CTTCATAGATGATATAT
M00059	V$YY1_01	0.845347	11986	(-)	NNNNNCCATNTWNNNWN	ATTGTAAAATGAATGTG
M00059	V$YY1_01	0.770315	12028	(+)	NNNNNCCATNTWNNNWN	CTAGAACATATTTCCTT
M00059	V$YY1_01	0.814875	12056	(+)	NNNNNCCATNTWNNNWN	CAACTTCATAGTATGAA
M00059	V$YY1_01	0.795216	12067	(-)	NNNNNCCATNTWNNNWN	TATGAAAGAGGGAAACC
M00059	V$YY1_01	0.796199	12086	(+)	NNNNNCCATNTWNNNWN	GCCCACCAGGTAATATT
M00059	V$YY1_01	0.814548	12102	(-)	NNNNNCCATNTWNNNWN	TTTGCTAAATGAATTAC
M00059	V$YY1_01	0.810616	12114	(-)	NNNNNCCATNTWNNNWN	ATTACATAATGATAATA
M00059	V$YY1_01	0.816841	12140	(-)	NNNNNCCATNTWNNNWN	GTTTAAATATGTTTGCT
M00059	V$YY1_01	0.852228	12430	(+)	NNNNNCCATNTWNNNWN	TATGTCCATGATTTGAT
M00059	V$YY1_01	0.851245	12501	(+)	NNNNNCCATNTWNNNWN	GCAAGCCATTTGGAGAT
M00059	V$YY1_01	0.822084	12546	(-)	NNNNNCCATNTWNNNWN	GAAACTATCTGGAGAGA
M00059	V$YY1_01	0.802425	12561	(-)	NNNNNCCATNTWNNNWN	GATAGTAAATGTTTCTG
M00059	V$YY1_01	0.781782	12603	(+)	NNNNNCCATNTWNNNWN	CCCTTCCATCTGTATAG
M00059	V$YY1_01	0.837811	12662	(+)	NNNNNCCATNTWNNNWN	GCATTCCATTGATTTAG
M00059	V$YY1_01	0.844364	12697	(+)	NNNNNCCATNTWNNNWN	TGATTTCATTTTTAAGG
M00059	V$YY1_01	0.811927	12715	(+)	NNNNNCCATNTWNNNWN	GACAACCAGATACTGTG
M00059	V$YY1_01	0.772936	12728	(-)	NNNNNCCATNTWNNNWN	TGTGAAATATGTGCTGC

M00059	V$YY1_01	0.833224	12791	(+)	NNNNNCCATNTWNNNWN	TACTGTCATTTAACGAG
M00059	V$YY1_01	0.780472	12911	(+)	NNNNNCCATNTWNNNWN	GCAAAGCATTTCAAAAA
M00059	V$YY1_01	0.815203	13143	(-)	NNNNNCCATNTWNNNWN	GTTGTTGACTGGTGTTG
M00059	V$YY1_01	0.825033	13236	(+)	NNNNNCCATNTWNNNWN	TATGGCCATCCCTGAAT
M00059	V$YY1_01	0.846003	13243	(-)	NNNNNCCATNTWNNNWN	ATCCCTGAATGAAGAGA
M00059	V$YY1_01	0.788336	13436	(-)	NNNNNCCATNTWNNNWN	AAATTAATATGAATCTA
M00059	V$YY1_01	0.852556	13507	(+)	NNNNNCCATNTWNNNWN	TGTCTTCAGTTTTAAAG
M00059	V$YY1_01	0.79882	13551	(+)	NNNNNCCATNTWNNNWN	TTGGTCCATTGATGGTT
M00059	V$YY1_01	0.783748	13569	(+)	NNNNNCCATNTWNNNWN	AACTTACATATAGTATG
M00059	V$YY1_01	0.773263	13611	(-)	NNNNNCCATNTWNNNWN	AGAAGACCATGGAGTTT
M00069	V$YY1_02	0.757051	13611	(+)	NNNCGGCCATCTTGNCTSNW	AGAAGACCATGGAGTTTTAT
M00059	V$YY1_01	0.806029	13612	(+)	NNNNNCCATNTWNNNWN	GAAGACCATGGAGTTTT
M00059	V$YY1_01	0.813237	13627	(-)	NNNNNCCATNTWNNNWN	TTATAAATATGAATCTG
M00059	V$YY1_01	0.795216	13694	(+)	NNNNNCCATNTWNNNWN	GATAATCATTCCTTAAT
M00059	V$YY1_01	0.809961	13830	(+)	NNNNNCCATNTWNNNWN	CCTTTCCATGTAATTTT
M00059	V$YY1_01	0.790957	13847	(-)	NNNNNCCATNTWNNNWN	TTAAAAAGAGGGTAATC
M00059	V$YY1_01	0.796199	14038	(+)	NNNNNCCATNTWNNNWN	AGATATCATATTTATTA
M00059	V$YY1_01	0.827326	14110	(+)	NNNNNCCATNTWNNNWN	TAATTCCATCAAAGAAC
M00059	V$YY1_01	0.79882	14120	(+)	NNNNNCCATNTWNNNWN	AAAGAACATATATTTTC
M00059	V$YY1_01	0.789646	14153	(+)	NNNNNCCATNTWNNNWN	GAATTACATTAATGCAC
M00059	V$YY1_01	0.795872	14181	(+)	NNNNNCCATNTWNNNWN	AAAGGCCATGATAGATT

M00059	V$YY1_01	0.824377	14228	(-)	NNNNNCCATNTWNNNWN	AAGAAATAATGGTTTTG
M00059	V$YY1_01	0.796199	14309	(+)	NNNNNCCATNTWNNNWN	TCTTGGCATTTCTGAAA
M00059	V$YY1_01	0.801769	14322	(+)	NNNNNCCATNTWNNNWN	GAAATCCCTGTATTATT
M00059	V$YY1_01	0.857143	14406	(-)	NNNNNCCATNTWNNNWN	AGGCAAAAATGACCTTC
M00059	V$YY1_01	0.815203	14493	(-)	NNNNNCCATNTWNNNWN	TTTGATAGCTGGACCTG
M00059	V$YY1_01	0.794561	14533	(-)	NNNNNCCATNTWNNNWN	GTTCAATACTGGCTATT
M00059	V$YY1_01	0.843709	14690	(+)	NNNNNCCATNTWNNNWN	AATAGCCATGTCTGAAA
M00059	V$YY1_01	0.79325	14715	(+)	NNNNNCCATNTWNNNWN	ATGATTCATTTTCAGAG
M00059	V$YY1_01	0.816186	14732	(-)	NNNNNCCATNTWNNNWN	ATTCAAAACTGATTTCT
M00059	V$YY1_01	0.855177	14761	(-)	NNNNNCCATNTWNNNWN	CTTTTTCAATGGTCTTT
M00069	V$YY1_02	0.767047	14797	(+)	NNNCGGCCATCTTGNCTSNW	ATTCTACCAGCTGGACGCCT
M00059	V$YY1_01	0.790301	14814	(+)	NNNNNCCATNTWNNNWN	CCTATCCATCTGGTGCC
M00059	V$YY1_01	0.789646	14868	(+)	NNNNNCCATNTWNNNWN	CCCCCTCATTCTCTGAC
M00059	V$YY1_01	0.78637	14942	(+)	NNNNNCCATNTWNNNWN	TAAGTTCATTTAAATGA
M00059	V$YY1_01	0.793578	14956	(+)	NNNNNCCATNTWNNNWN	TGACTGCATATTGCTGC
M00059	V$YY1_01	0.802425	15061	(-)	NNNNNCCATNTWNNNWN	TTTCTGAAATGGAAAAT
M00059	V$YY1_01	0.774574	15090	(-)	NNNNNCCATNTWNNNWN	TCTGATATATGGCTAAT
M00059	V$YY1_01	0.821429	15239	(-)	NNNNNCCATNTWNNNWN	ATCTTTAAATGCCTTTC
M00059	V$YY1_01	0.810288	15301	(+)	NNNNNCCATNTWNNNWN	AAACTGCAGATTTTGAC
M00059	V$YY1_01	0.771625	15371	(-)	NNNNNCCATNTWNNNWN	ATAATATAATGATGTTT
M00059	V$YY1_01	0.85059	15450	(+)	NNNNNCCATNTWNNNWN	AGTGCTCATTTTTTGAT

M00059	V$YY1_01	0.79062 9	15552	(-)	NNNNNCCATNTWNNNWN	CCACAATTATGGTCTTT
M00059	V$YY1_01	0.79194	15599	(-)	NNNNNCCATNTWNNNWN	CTTTTCATATGAATACA
M00059	V$YY1_01	0.79587 2	15713	(+)	NNNNNCCATNTWNNNWN	CTTCAACATTTTTTGTA
M00059	V$YY1_01	0.78047 2	15830	(-)	NNNNNCCATNTWNNNWN	TACTAATTCTGGTGACC
M00059	V$YY1_01	0.79062 9	15948	(+)	NNNNNCCATNTWNNNWN	AATGTCCATAGATGTGT
M00059	V$YY1_01	0.79914 8	15951	(-)	NNNNNCCATNTWNNNWN	GTCCATAGATGTGTTTT
M00059	V$YY1_01	0.83453 5	16111	(+)	NNNNNCCATNTWNNNWN	TTTTGCCATATTTTCTG
M00059	V$YY1_01	0.87975 1	16230	(+)	NNNNNCCATNTWNNNWN	GAACTTCATCTTAAGAT
M00059	V$YY1_01	0.86304 1	16237	(-)	NNNNNCCATNTWNNNWN	ATCTTAAGATGAAGAGA
M00059	V$YY1_01	0.82241 2	16248	(-)	NNNNNCCATNTWNNNWN	AAGAGAAAATGACTATT
M00059	V$YY1_01	0.82568 8	16313	(+)	NNNNNCCATNTWNNNWN	TAACTACATATTTCTTT
M00059	V$YY1_01	0.80733 9	16334	(-)	NNNNNCCATNTWNNNWN	ATTTACAGATGGGATTG
M00059	V$YY1_01	0.77195 3	16352	(+)	NNNNNCCATNTWNNNWN	AAATTCCATGCTCTACA
M00059	V$YY1_01	0.77064 2	16361	(+)	NNNNNCCATNTWNNNWN	GCTCTACATGTCTTTTC
M00059	V$YY1_01	0.83289 6	16371	(+)	NNNNNCCATNTWNNNWN	TCTTTTCATCTATCATG
M00059	V$YY1_01	0.78178 2	16388	(+)	NNNNNCCATNTWNNNWN	TCAAACCATTCTATCCA
M00059	V$YY1_01	0.83748 4	16597	(-)	NNNNNCCATNTWNNNWN	ATTTCTATATGGAAAAT
M00059	V$YY1_01	0.82241 2	16604	(-)	NNNNNCCATNTWNNNWN	TATGGAAAATGTTTTTA
M00069	V$YY1_02	0.78561 2	16644	(+)	NNNCGGCCATCTTGNCTSNW	TAAGTGCCATCATTTCAAAT
M00059	V$YY1_01	0.79292 3	16648	(+)	NNNNNCCATNTWNNNWN	TGCCATCATTTCAAATA
M00059	V$YY1_01	0.79128	16707	(+)	NNNNNCCATNTWNNNWN	AAATTACATTTTAAGGC

		4				
CSN1S2 *Ovis Aries*						
M00216	V$TATA_C	0.779509	120	(+)	NCTATAAAAR	TTTTTTAAAG
M00252	V$TATA_01	0.778229	120	(+)	STATAAAWRNNNNNN	TTTTTTAAAGAAACT
M00216	V$TATA_C	0.768154	121	(+)	NCTATAAAAR	TTTTTAAAGA
M00216	V$TATA_C	0.789015	146	(+)	NCTATAAAAR	ACCTTAAGAT
M00216	V$TATA_C	0.744125	177	(+)	NCTATAAAAR	GCTAACAAAG
M00216	V$TATA_C	0.862688	213	(+)	NCTATAAAAR	TTCTTAAAAA
M00216	V$TATA_C	0.841035	214	(+)	NCTATAAAAR	TCTTAAAAAA
M00216	V$TATA_C	0.79324	215	(+)	NCTATAAAAR	CTTAAAAAAA
M00252	V$TATA_01	0.775184	282	(+)	STATAAAWRNNNNNN	GTTTTAAGGATGAGC
M00216	V$TATA_C	0.768154	311	(+)	NCTATAAAAR	TTTTTGAAAA
M00216	V$TATA_C	0.750726	312	(+)	NCTATAAAAR	TTTTGAAAAT
M00252	V$TATA_01	0.777721	312	(+)	STATAAAWRNNNNNN	TTTTGAAAATCCAGG
M00216	V$TATA_C	0.753103	505	(+)	NCTATAAAAR	AAGATAAAAA
M00252	V$TATA_01	0.8102	506	(+)	STATAAAWRNNNNNN	AGATAAAAACATTTA
M00252	V$TATA_01	0.77696	518	(+)	STATAAAWRNNNNNN	TTATTTAACTCTCAG
M00216	V$TATA_C	0.768682	571	(+)	NCTATAAAAR	AAAATAAAAT
M00252	V$TATA_01	0.777468	607	(+)	STATAAAWRNNNNNN	CTATACATAATGAAC
M00216	V$TATA_C	0.749142	637	(+)	NCTATAAAAR	AACTTATAAA
M00216	V$TATA_C	0.897808	639	(+)	NCTATAAAAR	CTTATAAAAT
M00252	V$TATA_01	0.820096	640	(+)	STATAAAWRNNNNNN	TTATAAAATTTAATA
M00252	V$TATA_01	0.862979	652	(+)	STATAAAWRNNNNNN	ATATATATATCATCT
M00252	V$TATA_01	0.803857	654	(+)	STATAAAWRNNNNNN	ATATATATCATCTTA
M00216	V$TATA_C	0.856615	668	(+)	NCTATAAAAR	ATTTTAAAAC
M00252	V$TATA_01	0.776453	668	(+)	STATAAAWRNNNNNN	ATTTTAAAACATTGT
M00216	V$TATA_C	0.76921	684	(+)	NCTATAAAAR	ATTATGAAAT
M00216	V$TATA_C	0.836546	693	(+)	NCTATAAAAR	TGCTTATAAA
M00252	V$TATA_01	0.806648	694	(+)	STATAAAWRNNNNNN	GCTTATAAAGTGAAT
M00216	V$TATA_C	0.775812	695	(+)	NCTATAAAAR	CTTATAAAGT
M00252	V$TATA_01	0.813499	696	(+)	STATAAAWRNNNNNN	TTATAAAGTGAATGT
M00216	V$TATA_C	0.999472	723	(+)	NCTATAAAAR	CCTATAAAAG
M00252	V$TATA_01	0.944177	724	(+)	STATAAAWRNNNNNN	CTATAAAAGGAAACA

M00216	V$TATA_C	0.744917	798	(+)	NCTATAAAAR	ATCTTAAGAG
M00216	V$TATA_C	0.775284	883	(+)	NCTATAAAAR	CAAATAAAAT
M00252	V$TATA_01	0.774169	884	(+)	STATAAAWRNNNNNN	AAATAAAATCTTGCC
M00216	V$TATA_C	0.762873	896	(+)	NCTATAAAAR	GCCATTTAAA
M00216	V$TATA_C	0.759968	897	(+)	NCTATAAAAR	CCATTTAAAA
M00252	V$TATA_01	0.775184	897	(+)	STATAAAWRNNNNNN	CCATTTAAAAATAAT
M00216	V$TATA_C	0.847637	898	(+)	NCTATAAAAR	CATTTAAAAA
M00216	V$TATA_C	0.743597	899	(+)	NCTATAAAAR	ATTTAAAAAT
M00252	V$TATA_01	0.822888	899	(+)	STATAAAWRNNNNNN	ATTTAAAAATAATTG
M00252	V$TATA_01	0.770363	1075	(+)	STATAAAWRNNNNNN	TTATATGTCAAAAAG
M00216	V$TATA_C	0.815949	1223	(+)	NCTATAAAAR	TGCATGAAAG
M00252	V$TATA_01	0.778483	1224	(+)	STATAAAWRNNNNNN	GCATGAAAGTGAAAA
M00216	V$TATA_C	0.762873	1233	(+)	NCTATAAAAR	TGAAAAAAAG
M00216	V$TATA_C	0.752839	1358	(+)	NCTATAAAAR	TCTGCAAAAA
M00216	V$TATA_C	0.815949	1360	(+)	NCTATAAAAR	TGCAAAAAAG
M00216	V$TATA_C	0.797465	1368	(+)	NCTATAAAAR	AGCATTAAAA
M00216	V$TATA_C	0.853182	1369	(+)	NCTATAAAAR	GCATTAAAAT
M00252	V$TATA_01	0.793453	1369	(+)	STATAAAWRNNNNNN	GCATTAAAATAACTT
M00216	V$TATA_C	0.757064	1413	(+)	NCTATAAAAR	ACTGGAAAAG
M00252	V$TATA_01	0.786349	1429	(+)	STATAAAWRNNNNNN	CGATATGTAGTCAGC
M00252	V$TATA_01	0.801319	1457	(+)	STATAAAWRNNNNNN	TTATATGTGGTCATA
M00216	V$TATA_C	0.781885	1504	(+)	NCTATAAAAR	AGTTAAAAAT
M00252	V$TATA_01	0.770617	1504	(+)	STATAAAWRNNNNNN	AGTTAAAAATCTCGT
M00216	V$TATA_C	0.881965	1628	(+)	NCTATAAAAR	CCCTTATAAG
M00252	V$TATA_01	0.832784	1629	(+)	STATAAAWRNNNNNN	CCTTATAAGGGAGGC
M00252	V$TATA_01	0.792185	1631	(+)	STATAAAWRNNNNNN	TTATAAGGGAGGCAG
M00216	V$TATA_C	0.883021	1668	(+)	NCTATAAAAR	ATTTTAAAAA
M00216	V$TATA_C	0.779509	1669	(+)	NCTATAAAAR	TTTTAAAAAG
M00252	V$TATA_01	0.811977	1669	(+)	STATAAAWRNNNNNN	TTTTAAAAAGATTTC
M00252	V$TATA_01	0.780259	1719	(+)	STATAAAWRNNNNNN	ATTTAAGAAGAATTG
M00216	V$TATA_C	0.794824	1730	(+)	NCTATAAAAR	ATTGTAAAAA
M00216	V$TATA_C	0.832057	1732	(+)	NCTATAAAAR	TGTAAAAAAA
M00252	V$TATA_01	0.810708	1733	(+)	STATAAAWRNNNNNN	GTAAAAAAAAATATG

M00216	V$TATA_C	0.824927	1756	(+)	NCTATAAAAR	AGTATAAACA
M00252	V$TATA_01	0.823649	1757	(+)	STATAAAWRNNNNNN	GTATAAACATCACTA
M00252	V$TATA_01	0.788379	1829	(+)	STATAAAWRNNNNNN	GAATAAGTGGGTAAA
M00252	V$TATA_01	0.775184	1849	(+)	STATAAAWRNNNNNN	AAATAAATTAGCCAT
M00252	V$TATA_01	0.822634	1860	(+)	STATAAAWRNNNNNN	CCATTTATACATAGG
M00252	V$TATA_01	0.776199	1864	(+)	STATAAAWRNNNNNN	TTATACATAGGGTCA
M00252	V$TATA_01	0.782035	1866	(+)	STATAAAWRNNNNNN	ATACATAGGGTCAGT
M00252	V$TATA_01	0.779751	1917	(+)	STATAAAWRNNNNNN	CTATTTAGAAACTAA
M00252	V$TATA_01	0.783304	1999	(+)	STATAAAWRNNNNNN	GTATTCAAATCCATG
M00216	V$TATA_C	0.741484	2014	(+)	NCTATAAAAR	CGTTTATCAA
M00216	V$TATA_C	0.794824	2107	(+)	NCTATAAAAR	TGATTATAAG
M00252	V$TATA_01	0.82238	2108	(+)	STATAAAWRNNNNNN	GATTATAAGGAGAGT
M00252	V$TATA_01	0.774676	2110	(+)	STATAAAWRNNNNNN	TTATAAGGAGAGTAA
M00252	V$TATA_01	0.7729	2130	(+)	STATAAAWRNNNNNN	TTATTAATTAGCTTC
M00216	V$TATA_C	0.744389	2218	(+)	NCTATAAAAR	AGCATGTAAG
M00216	V$TATA_C	0.824927	2226	(+)	NCTATAAAAR	AGTATAATAA
M00252	V$TATA_01	0.849784	2227	(+)	STATAAAWRNNNNNN	GTATAATAAGATACA
M00252	V$TATA_01	0.770363	2254	(+)	STATAAAWRNNNNNN	TTATGTAAATATAAG
M00252	V$TATA_01	0.792438	2258	(+)	STATAAAWRNNNNNN	GTAAATATAAGAATA
M00216	V$TATA_C	0.740164	2259	(+)	NCTATAAAAR	TAAATATAAG
M00252	V$TATA_01	0.795991	2260	(+)	STATAAAWRNNNNNN	AAATATAAGAATATT
M00216	V$TATA_C	0.744653	2261	(+)	NCTATAAAAR	AATATAAGAA
M00252	V$TATA_01	0.784065	2262	(+)	STATAAAWRNNNNNN	ATATAAGAATATTAA
M00252	V$TATA_01	0.773154	2270	(+)	STATAAAWRNNNNNN	ATATTAAATTGTTTA
M00216	V$TATA_C	0.753367	2279	(+)	NCTATAAAAR	TGTTTATGAG
M00252	V$TATA_01	0.792946	2280	(+)	STATAAAWRNNNNNN	GTTTATGAGGATAAT
M00216	V$TATA_C	0.768682	2326	(+)	NCTATAAAAR	AGTGTATAAA
M00216	V$TATA_C	0.814629	2328	(+)	NCTATAAAAR	TGTATAAATT
M00252	V$TATA_01	0.860442	2329	(+)	STATAAAWRNNNNNN	GTATAAATTTTGAAA
M00216	V$TATA_C	0.744125	2344	(+)	NCTATAAAAR	CCTATAATCA
M00216	V$TATA_C	0.740164	2373	(+)	NCTATAAAAR	CTTATGTAAG
M00252	V$TATA_01	0.773662	2383	(+)	STATAAAWRNNNNNN	TTATGAAGATGGACA
M00216	V$TATA_C	0.810668	2453	(+)	NCTATAAAAR	AGTTAAAAAG

M0025		0.7886				
2	V$TATA_01	32	2453	(+)	STATAAAWRNNNNNN	AGTTAAAAAGCGTAC
M0025		0.7779				
2	V$TATA_01	75	2454	(+)	STATAAAWRNNNNNN	GTTAAAAAGCGTACA
M0021		0.8391				
6	V$TATA_C	87	2475	(+)	NCTATAAAAR	TCCATAAACA
M0021		0.8740				
6	V$TATA_C	43	2525	(+)	NCTATAAAAR	TTCTTAAAAG
M0021		0.8320				
6	V$TATA_C	57	2548	(+)	NCTATAAAAR	ACCATAACAA
M0025		0.7772				
2	V$TATA_01	14	2549	(+)	STATAAAWRNNNNNN	CCATAACAAGGACTT
M0021		0.8523				
6	V$TATA_C	9	2563	(+)	NCTATAAAAR	TCTTTTAAAG
M0021		0.7501				
6	V$TATA_C	98	2564	(+)	NCTATAAAAR	CTTTTAAAGT
M0025		0.8142				
2	V$TATA_01	6	2564	(+)	STATAAAWRNNNNNN	CTTTTAAAGTTCAGG
M0021		0.8135				
6	V$TATA_C	73	2850	(+)	NCTATAAAAR	TCCTTAAACA
M0021		0.8339				
6	V$TATA_C	05	2930	(+)	NCTATAAAAR	ACTTTGAAAA
M0021		0.7501				
6	V$TATA_C	98	2931	(+)	NCTATAAAAR	CTTTGAAAAT
M0025		0.8008				
2	V$TATA_01	12	3034	(+)	STATAAAWRNNNNNN	TGATAAAGAAGGACT
M0020		0.8369				
3	V$GATA_C	06	14	(-)	NGATAAGNMNN	ACTACTTGTCT
M0012		0.8503				
7	V$GATA1_03	18	15	(-)	RNSNNGATAANNGN	CTACTTGTCTTCCT
M0020		0.9164				
3	V$GATA_C	34	50	(-)	NGATAAGNMNN	TTCATTTATCT
M0012		0.8462				
6	V$GATA1_02	5	51	(-)	NNNNNGATANKGNN	TCATTTATCTTTTT
M0012		0.8770				
7	V$GATA1_03	21	51	(-)	RNSNNGATAANNGN	TCATTTATCTTTTT
M0012		0.9065				
8	V$GATA1_04	56	51	(-)	NNCWGATARNNNN	TCATTTATCTTTT
M0007		0.8069				
6	V$GATA2_01	46	53	(-)	NNNGATRNNN	ATTTATCTTT
M0007		0.8466				
7	V$GATA3_01	99	53	(-)	NNGATARNG	ATTTATCTT
M0012		0.8168				
6	V$GATA1_02	75	132	(-)	NNNNNGATANKGNN	ACTAATATCAGTGT
M0012		0.7981				
7	V$GATA1_03	38	132	(-)	RNSNNGATAANNGN	ACTAATATCAGTGT
M0007		0.8168				
5	V$GATA1_01	81	134	(-)	SNNGATNNNN	TAATATCAGT
M0007		0.8290				
6	V$GATA2_01	48	134	(-)	NNNGATRNNN	TAATATCAGT
M0007		0.8373				
7	V$GATA3_01	95	134	(-)	NNGATARNG	TAATATCAG
M0007		0.8168				
5	V$GATA1_01	81	150	(+)	SNNGATNNNN	TAAGATTCTT
M0007		0.8412				
6	V$GATA2_01	27	150	(+)	NNNGATRNNN	TAAGATTCTT
M0012		0.8379				
8	V$GATA1_04	29	169	(+)	NNCWGATARNNNN	GTTTGATAGCTAA
M0007		0.8509				
5	V$GATA1_01	38	170	(+)	SNNGATNNNN	TTTGATAGCT
M0007		0.8570				
6	V$GATA2_01	14	170	(+)	NNNGATRNNN	TTTGATAGCT
M0007		0.8311				
7	V$GATA3_01	92	171	(+)	NNGATARNG	TTGATAGCT
M0020		0.8381				
3	V$GATA_C	48	172	(+)	NGATAAGNMNN	TGATAGCTAAC

M00075	V$GATA1_01	0.78924	189	(+)	SNNGATNNNN	ACTGATTTAT
M00203	V$GATA_C	0.916744	190	(-)	NGATAAGNMNN	CTGATTTATCT
M00126	V$GATA1_02	0.7975	191	(-)	NNNNNGATANKGNN	TGATTTATCTCCTT
M00127	V$GATA1_03	0.864527	191	(-)	RNSNNGATAANNGN	TGATTTATCTCCTT
M00128	V$GATA1_04	0.899816	191	(-)	NNCWGATARNNNN	TGATTTATCTCCT
M00075	V$GATA1_01	0.846496	193	(-)	SNNGATNNNN	ATTTATCTCC
M00076	V$GATA2_01	0.861976	193	(-)	NNNGATRNNN	ATTTATCTCC
M00077	V$GATA3_01	0.863979	193	(-)	NNGATARNG	ATTTATCTC
M00127	V$GATA1_03	0.817001	245	(-)	RNSNNGATAANNGN	ATTGTTATACTGCT
M00203	V$GATA_C	0.879466	254	(-)	NGATAAGNMNN	CTGCTCTATCT
M00126	V$GATA1_02	0.840313	255	(-)	NNNNNGATANKGNN	TGCTCTATCTTATG
M00128	V$GATA1_04	0.914216	255	(-)	NNCWGATARNNNN	TGCTCTATCTTAT
M00075	V$GATA1_01	0.838105	257	(-)	SNNGATNNNN	CTCTATCTTA
M00076	V$GATA2_01	0.903924	257	(-)	NNNGATRNNN	CTCTATCTTA
M00077	V$GATA3_01	0.941958	257	(-)	NNGATARNG	CTCTATCTT
M00075	V$GATA1_01	0.785785	287	(+)	SNNGATNNNN	AAGGATGAGC
M00076	V$GATA2_01	0.835814	287	(+)	NNNGATRNNN	AAGGATGAGC
M00075	V$GATA1_01	0.795656	295	(-)	SNNGATNNNN	GCTAATCATA
M00076	V$GATA2_01	0.806495	295	(-)	NNNGATRNNN	GCTAATCATA
M00077	V$GATA3_01	0.830306	295	(-)	NNGATARNG	GCTAATCAT
M00075	V$GATA1_01	0.801086	316	(-)	SNNGATNNNN	GAAAATCCAG
M00075	V$GATA1_01	0.802073	353	(-)	SNNGATNNNN	CTGGATCACT
M00127	V$GATA1_03	0.803283	357	(-)	RNSNNGATAANNGN	ATCACTTTCTTCAT
M00075	V$GATA1_01	0.785291	365	(-)	SNNGATNNNN	CTTCATCTGT
M00076	V$GATA2_01	0.799729	365	(-)	NNNGATRNNN	CTTCATCTGT
M00077	V$GATA3_01	0.854231	365	(-)	NNGATARNG	CTTCATCTG
M00076	V$GATA2_01	0.825891	428	(+)	NNNGATRNNN	CAACATACCT
M00075	V$GATA1_01	0.913129	457	(+)	SNNGATNNNN	GCGGATGCTT
M00076	V$GATA2_01	0.914299	457	(+)	NNNGATRNNN	GCGGATGCTT
M00127	V$GATA1_03	0.78148	474	(-)	RNSNNGATAANNGN	ACAACTATTTCTAT
M00203	V$GATA_C	0.873563	492	(-)	NGATAAGNMNN	CTCATTTATCA
M00126	V$GATA1_02	0.8425	493	(-)	NNNNNGATANKGNN	TCATTTATCAACAA
M00127	V$GATA1_03	0.800343	493	(-)	RNSNNGATAANNGN	TCATTTATCAACAA

M00128	V$GATA1_04	0.855086	493	(-)	NNCWGATARNNNN	TCATTTATCAACA
M00075	V$GATA1_01	0.802567	495	(-)	SNNGATNNNN	ATTTATCAAC
M00126	V$GATA1_02	0.791875	502	(+)	NNNNNGATANKGNN	AACAAGATAAAAAC
M00127	V$GATA1_03	0.898334	502	(+)	RNSNNGATAANNGN	AACAAGATAAAAAC
M00128	V$GATA1_04	0.926777	503	(+)	NNCWGATARNNNN	ACAAGATAAAAAC
M00075	V$GATA1_01	0.786772	504	(+)	SNNGATNNNN	CAAGATAAAA
M00076	V$GATA2_01	0.818674	504	(+)	NNNGATRNNN	CAAGATAAAA
M00077	V$GATA3_01	0.8786	505	(+)	NNGATARNG	AAGATAAAA
M00203	V$GATA_C	0.955887	506	(+)	NGATAAGNMNN	AGATAAAAACA
M00203	V$GATA_C	0.846536	535	(-)	NGATAAGNMNN	AATATTTGTCC
M00126	V$GATA1_02	0.772813	575	(-)	NNNNNGATANKGNN	TAAAATATCTTAAG
M00075	V$GATA1_01	0.819348	577	(-)	SNNGATNNNN	AAATATCTTA
M00076	V$GATA2_01	0.894452	577	(-)	NNNGATRNNN	AAATATCTTA
M00077	V$GATA3_01	0.834736	577	(-)	NNGATARNG	AAATATCTT
M00203	V$GATA_C	0.857098	653	(-)	NGATAAGNMNN	TATATATATCA
M00128	V$GATA1_04	0.857843	654	(-)	NNCWGATARNNNN	ATATATATCATCT
M00075	V$GATA1_01	0.856861	656	(-)	SNNGATNNNN	ATATATCATC
M00076	V$GATA2_01	0.857465	656	(-)	NNNGATRNNN	ATATATCATC
M00076	V$GATA2_01	0.82138	659	(-)	NNNGATRNNN	TATCATCTTA
M00077	V$GATA3_01	0.844927	659	(-)	NNGATARNG	TATCATCTT
M00127	V$GATA1_03	0.783195	692	(+)	RNSNNGATAANNGN	ATGCTTATAAAGTG
M00203	V$GATA_C	0.888164	714	(-)	NGATAAGNMNN	GTGCATTATCC
M00126	V$GATA1_02	0.8175	715	(-)	NNNNNGATANKGNN	TGCATTATCCTATA
M00128	V$GATA1_04	0.835784	715	(-)	NNCWGATARNNNN	TGCATTATCCTAT
M00075	V$GATA1_01	0.807996	717	(-)	SNNGATNNNN	CATTATCCTA
M00076	V$GATA2_01	0.907082	717	(-)	NNNGATRNNN	CATTATCCTA
M00077	V$GATA3_01	0.888347	717	(-)	NNGATARNG	CATTATCCT
M00126	V$GATA1_02	0.804688	738	(-)	NNNNNGATANKGNN	AAGAATATCTGGGA
M00075	V$GATA1_01	0.885982	740	(-)	SNNGATNNNN	GAATATCTGG
M00076	V$GATA2_01	0.907984	740	(-)	NNNGATRNNN	GAATATCTGG
M00077	V$GATA3_01	0.828977	740	(-)	NNGATARNG	GAATATCTG
M00075	V$GATA1_01	0.841066	747	(+)	SNNGATNNNN	TGGGATTCTT
M00076	V$GATA2_01	0.86378	747	(+)	NNNGATRNNN	TGGGATTCTT

M00126	V$GATA1_02	0.825937	762	(+)	NNNNNGATANKGNN	GAAATGATAAATTA
M00127	V$GATA1_03	0.805243	762	(+)	RNSNNGATAANNGN	GAAATGATAAATTA
M00128	V$GATA1_04	0.858456	763	(+)	NNCWGATARNNNN	AAATGATAAATTA
M00075	V$GATA1_01	0.773939	764	(+)	SNNGATNNNN	AATGATAAAT
M00076	V$GATA2_01	0.79567	764	(+)	NNNGATRNNN	AATGATAAAT
M00077	V$GATA3_01	0.823217	765	(+)	NNGATARNG	ATGATAAAT
M00203	V$GATA_C	0.893445	766	(+)	NGATAAGNMNN	TGATAAATTAA
M00076	V$GATA2_01	0.788453	788	(-)	NNNGATRNNN	GGCAATGCTA
M00075	V$GATA1_01	0.774926	794	(-)	SNNGATNNNN	GCTAATCTTA
M00076	V$GATA2_01	0.817772	794	(-)	NNNGATRNNN	GCTAATCTTA
M00077	V$GATA3_01	0.853788	794	(-)	NNGATARNG	GCTAATCTT
M00127	V$GATA1_03	0.791034	849	(+)	RNSNNGATAANNGN	GCAAAGATCACCAG
M00075	V$GATA1_01	0.873643	851	(-)	SNNGATNNNN	AAAGATCACC
M00076	V$GATA2_01	0.797925	851	(+)	NNNGATRNNN	AAAGATCACC
M00076	V$GATA2_01	0.841227	851	(-)	NNNGATRNNN	AAAGATCACC
M00075	V$GATA1_01	0.820829	887	(-)	SNNGATNNNN	TAAAATCTTG
M00076	V$GATA2_01	0.809202	887	(-)	NNNGATRNNN	TAAAATCTTG
M00077	V$GATA3_01	0.832964	887	(-)	NNGATARNG	TAAAATCTT
M00127	V$GATA1_03	0.791524	1002	(-)	RNSNNGATAANNGN	CAGCCTACCAGGCT
M00075	V$GATA1_01	0.78924	1017	(-)	SNNGATNNNN	CTCCATCCAT
M00075	V$GATA1_01	0.777394	1026	(+)	SNNGATNNNN	TGGGATCTTC
M00075	V$GATA1_01	0.80997	1026	(-)	SNNGATNNNN	TGGGATCTTC
M00076	V$GATA2_01	0.848895	1026	(+)	NNNGATRNNN	TGGGATCTTC
M00076	V$GATA2_01	0.844835	1026	(-)	NNNGATRNNN	TGGGATCTTC
M00075	V$GATA1_01	0.7769	1095	(+)	SNNGATNNNN	GCTGCTGCTG
M00075	V$GATA1_01	0.826259	1172	(+)	SNNGATNNNN	TGGGATTCTC
M00076	V$GATA2_01	0.860171	1172	(+)	NNNGATRNNN	TGGGATTCTC
M00127	V$GATA1_03	0.784174	1179	(+)	RNSNNGATAANNGN	CTCCAGACAAGAAT
M00128	V$GATA1_04	0.818627	1180	(+)	NNCWGATARNNNN	TCCAGACAAGAAT
M00203	V$GATA_C	0.879466	1183	(+)	NGATAAGNMNN	AGACAAGAATA
M00075	V$GATA1_01	0.786278	1225	(+)	SNNGATNNNN	CATGAAAGTG
M00127	V$GATA1_03	0.791524	1288	(-)	RNSNNGATAANNGN	CAGCCTACCAGGCT
M00075	V$GATA1_01	0.78924	1303	(-)	SNNGATNNNN	CTCCATCCAT

M0007<br>5	V$GATA1_01	0.8050<br>35	1312	(+)	SNNGATNNNN	TGGGATTTTC
M0007<br>6	V$GATA2_01	0.8488<br>95	1312	(+)	NNNGATRNNN	TGGGATTTTC
M0020<br>3	V$GATA_C	0.8381<br>48	1350	(-)	NGATAAGNMNN	TTGCCTTCTCT
M0012<br>6	V$GATA1_02	0.7834<br>38	1351	(-)	NNNNNGATANKGNN	TGCCTTCTCTGCAA
M0012<br>8	V$GATA1_04	0.8174<br>02	1351	(-)	NNCWGATARNNNN	TGCCTTCTCTGCA
M0007<br>7	V$GATA3_01	0.8245<br>46	1353	(-)	NNGATARNG	CCTTCTCTG
M0012<br>7	V$GATA1_03	0.7956<br>88	1395	(-)	RNSNNGATAANNGN	TCAGTTATTTGTTT
M0012<br>6	V$GATA1_02	0.78	1425	(+)	NNNNNGATANKGNN	GCATCGATATGTAG
M0007<br>5	V$GATA1_01	0.8020<br>73	1427	(+)	SNNGATNNNN	ATCGATATGT
M0007<br>6	V$GATA2_01	0.7834<br>91	1427	(+)	NNNGATRNNN	ATCGATATGT
M0020<br>3	V$GATA_C	0.8403<br>23	1452	(-)	NGATAAGNMNN	GGGACTTATAT
M0007<br>6	V$GATA2_01	0.8046<br>91	1465	(+)	NNNGATRNNN	GGTCATAGTA
M0012<br>6	V$GATA1_02	0.7721<br>88	1475	(+)	NNNNNGATANKGNN	TTAGAGATTGAGCT
M0007<br>5	V$GATA1_01	0.8346<br>5	1477	(+)	SNNGATNNNN	AGAGATTGAG
M0007<br>6	V$GATA2_01	0.8150<br>65	1477	(+)	NNNGATRNNN	AGAGATTGAG
M0007<br>7	V$GATA3_01	0.9255<br>65	1478	(+)	NNGATARNG	GAGATTGAG
M0007<br>5	V$GATA1_01	0.8336<br>62	1492	(+)	SNNGATNNNN	GGGGATCTTC
M0007<br>5	V$GATA1_01	0.8307<br>01	1492	(-)	SNNGATNNNN	GGGGATCTTC
M0007<br>6	V$GATA2_01	0.8633<br>29	1492	(+)	NNNGATRNNN	GGGGATCTTC
M0007<br>6	V$GATA2_01	0.8709<br>97	1492	(-)	NNNGATRNNN	GGGGATCTTC
M0007<br>5	V$GATA1_01	0.8849<br>95	1508	(-)	SNNGATNNNN	AAAAATCTCG
M0007<br>6	V$GATA2_01	0.8470<br>91	1508	(-)	NNNGATRNNN	AAAAATCTCG
M0012<br>7	V$GATA1_03	0.7974<br>03	1522	(-)	RNSNNGATAANNGN	GTGGTTATTCTGTC
M0007<br>5	V$GATA1_01	0.8455<br>08	1542	(+)	SNNGATNNNN	AGTGATGTTC
M0007<br>6	V$GATA2_01	0.8267<br>93	1542	(+)	NNNGATRNNN	AGTGATGTTC
M0007<br>5	V$GATA1_01	0.8667<br>32	1552	(+)	SNNGATNNNN	AGTGATGCTC
M0007<br>6	V$GATA2_01	0.8380<br>69	1552	(+)	NNNGATRNNN	AGTGATGCTC
M0007<br>5	V$GATA1_01	0.7714<br>71	1572	(+)	SNNGATNNNN	TGGGATCAGC
M0007<br>5	V$GATA1_01	0.8514<br>31	1572	(-)	SNNGATNNNN	TGGGATCAGC
M0007<br>6	V$GATA2_01	0.8403<br>25	1572	(+)	NNNGATRNNN	TGGGATCAGC
M0007<br>6	V$GATA2_01	0.8416<br>78	1572	(-)	NNNGATRNNN	TGGGATCAGC
M0012<br>7	V$GATA1_03	0.8072<br>02	1585	(-)	RNSNNGATAANNGN	CTTCTTGTCCCCAG
M0012<br>7	V$GATA1_03	0.7920<br>14	1602	(-)	RNSNNGATAANNGN	AGTGTTATTTTCAC

M00127	V$GATA1_03	0.784174	1605	(-)	RNSNNGATAANNGN	GTTATTTTCACCTG
M00127	V$GATA1_03	0.828025	1619	(-)	RNSNNGATAANNGN	CCAATTCTCCCCTT
M00075	V$GATA1_01	0.805528	1664	(+)	SNNGATNNNN	CTTGATTTTA
M00075	V$GATA1_01	0.787266	1675	(+)	SNNGATNNNN	AAAGATTTCT
M00076	V$GATA2_01	0.816419	1675	(+)	NNNGATRNNN	AAAGATTTCT
M00075	V$GATA1_01	0.842547	1700	(+)	SNNGATNNNN	TCTGATGATT
M00076	V$GATA2_01	0.847993	1700	(+)	NNNGATRNNN	TCTGATGATT
M00075	V$GATA1_01	0.857848	1703	(+)	SNNGATNNNN	GATGATTTTC
M00076	V$GATA2_01	0.829499	1703	(+)	NNNGATRNNN	GATGATTTTC
M00075	V$GATA1_01	0.781343	1715	(+)	SNNGATNNNN	CAAGATTTAA
M00076	V$GATA2_01	0.79161	1741	(-)	NNNGATRNNN	AAATATGGCA
M00075	V$GATA1_01	0.860316	1761	(-)	SNNGATNNNN	AAACATCACT
M00076	V$GATA2_01	0.830401	1761	(-)	NNNGATRNNN	AAACATCACT
M00127	V$GATA1_03	0.782215	1769	(-)	RNSNNGATAANNGN	CTATTTTTCATCAC
M00126	V$GATA1_02	0.845	1780	(-)	NNNNNGATANKGNN	CACTGTATCTTGAT
M00127	V$GATA1_03	0.841009	1780	(-)	RNSNNGATAANNGN	CACTGTATCTTGAT
M00075	V$GATA1_01	0.873149	1782	(-)	SNNGATNNNN	CTGTATCTTG
M00076	V$GATA2_01	0.887686	1782	(-)	NNNGATRNNN	CTGTATCTTG
M00077	V$GATA3_01	0.86265	1782	(-)	NNGATARNG	CTGTATCTT
M00075	V$GATA1_01	0.864758	1788	(+)	SNNGATNNNN	CTTGATGTCT
M00076	V$GATA2_01	0.801083	1788	(+)	NNNGATRNNN	CTTGATGTCT
M00076	V$GATA2_01	0.820478	1809	(+)	NNNGATRNNN	TGACATAGCA
M00126	V$GATA1_02	0.810937	1864	(+)	NNNNNGATANKGNN	TTATACATAGGGTC
M00076	V$GATA2_01	0.813261	1878	(-)	NNNGATRNNN	AGTTATGCCA
M00076	V$GATA2_01	0.824087	1894	(-)	NNNGATRNNN	TGGTATGCCC
M00126	V$GATA1_02	0.818125	1913	(-)	NNNNNGATANKGNN	TCCACTATTTAGAA
M00076	V$GATA2_01	0.819125	1929	(+)	NNNGATRNNN	TAAGATTTCA
M00127	V$GATA1_03	0.791034	1942	(-)	RNSNNGATAANNGN	TTCCTTCTCCAGAT
M00075	V$GATA1_01	0.84156	1950	(+)	SNNGATNNNN	CCAGATTTTA
M00076	V$GATA2_01	0.817321	1950	(+)	NNNGATRNNN	CCAGATTTTA
M00077	V$GATA3_01	0.827204	1951	(+)	NNGATARNG	CAGATTTTA
M00076	V$GATA2_01	0.793866	1960	(-)	NNNGATRNNN	CATTATGTTG
M00203	V$GATA_C	0.908357	2012	(-)	NGATAAGNMNN	TGCGTTTATCA

M00126	V$GATA1_02	0.842187	2013	(-)	NNNNNGATANKGNN	GCGTTTATCAATGA
M00127	V$GATA1_03	0.783684	2013	(-)	RNSNNGATAANNGN	GCGTTTATCAATGA
M00075	V$GATA1_01	0.797631	2023	(-)	SNNGATNNNN	ATGAATCTAC
M00203	V$GATA_C	0.900901	2030	(-)	NGATAAGNMNN	TACTTTTATCA
M00126	V$GATA1_02	0.815313	2031	(-)	NNNNNGATANKGNN	ACTTTTATCAGTCT
M00127	V$GATA1_03	0.902499	2031	(-)	RNSNNGATAANNGN	ACTTTTATCAGTCT
M00128	V$GATA1_04	0.930453	2031	(-)	NNCWGATARNNNN	ACTTTTATCAGTC
M00077	V$GATA3_01	0.849358	2033	(-)	NNGATARNG	TTTTATCAG
M00203	V$GATA_C	0.832867	2057	(-)	NGATAAGNMNN	TCTAATTATCA
M00126	V$GATA1_02	0.779687	2058	(-)	NNNNNGATANKGNN	CTAATTATCATCTG
M00127	V$GATA1_03	0.888045	2058	(-)	RNSNNGATAANNGN	CTAATTATCATCTG
M00128	V$GATA1_04	0.895221	2058	(-)	NNCWGATARNNNN	CTAATTATCATCT
M00075	V$GATA1_01	0.878085	2060	(-)	SNNGATNNNN	AATTATCATC
M00076	V$GATA2_01	0.882724	2060	(-)	NNNGATRNNN	AATTATCATC
M00077	V$GATA3_01	0.843155	2060	(-)	NNGATARNG	AATTATCAT
M00075	V$GATA1_01	0.786278	2063	(-)	SNNGATNNNN	TATCATCTGA
M00076	V$GATA2_01	0.829499	2063	(-)	NNNGATRNNN	TATCATCTGA
M00077	V$GATA3_01	0.839167	2063	(-)	NNGATARNG	TATCATCTG
M00126	V$GATA1_02	0.844375	2069	(+)	NNNNNGATANKGNN	CTGAAGATAATTTA
M00127	V$GATA1_03	0.872856	2069	(+)	RNSNNGATAANNGN	CTGAAGATAATTTA
M00128	V$GATA1_04	0.837316	2070	(+)	NNCWGATARNNNN	TGAAGATAATTTA
M00075	V$GATA1_01	0.857354	2071	(+)	SNNGATNNNN	GAAGATAATT
M00076	V$GATA2_01	0.894001	2071	(+)	NNNGATRNNN	GAAGATAATT
M00077	V$GATA3_01	0.866637	2072	(+)	NNGATARNG	AAGATAATT
M00203	V$GATA_C	0.85803	2073	(+)	NGATAAGNMNN	AGATAATTTAA
M00076	V$GATA2_01	0.794768	2100	(-)	NNNGATRNNN	AAATATGTGA
M00075	V$GATA1_01	0.791214	2105	(+)	SNNGATNNNN	TGTGATTATA
M00076	V$GATA2_01	0.782138	2105	(+)	NNNGATRNNN	TGTGATTATA
M00077	V$GATA3_01	0.858662	2106	(+)	NNGATARNG	GTGATTATA
M00127	V$GATA1_03	0.823371	2106	(+)	RNSNNGATAANNGN	GTGATTATAAGGAG
M00127	V$GATA1_03	0.790544	2106	(-)	RNSNNGATAANNGN	GTGATTATAAGGAG
M00127	V$GATA1_03	0.801323	2133	(-)	RNSNNGATAANNGN	TTAATTAGCTTCTT
M00203	V$GATA_C	0.910531	2140	(-)	NGATAAGNMNN	GCTTCTTATCT

M00126	V$GATA1_02	0.857187	2141	(-)	NNNNNGATANKGNN	CTTCTTATCTATTT
M00127	V$GATA1_03	0.897844	2141	(-)	RNSNNGATAANNGN	CTTCTTATCTATTT
M00128	V$GATA1_04	0.91299	2141	(-)	NNCWGATARNNNN	CTTCTTATCTATT
M00077	V$GATA3_01	0.894993	2143	(-)	NNGATARNG	TCTTATCTA
M00075	V$GATA1_01	0.777887	2157	(+)	SNNGATNNNN	CAGGATCAAG
M00075	V$GATA1_01	0.846002	2157	(-)	SNNGATNNNN	CAGGATCAAG
M00076	V$GATA2_01	0.811457	2157	(+)	NNNGATRNNN	CAGGATCAAG
M00127	V$GATA1_03	0.811612	2184	(-)	RNSNNGATAANNGN	ATTTTTACCTGCCT
M00127	V$GATA1_03	0.783684	2223	(+)	RNSNNGATAANNGN	GTAAGTATAATAAG
M00126	V$GATA1_02	0.795625	2231	(+)	NNNNNGATANKGNN	AATAAGATACAGAT
M00128	V$GATA1_04	0.856311	2232	(+)	NNCWGATARNNNN	ATAAGATACAGAT
M00075	V$GATA1_01	0.812438	2233	(+)	SNNGATNNNN	TAAGATACAG
M00076	V$GATA2_01	0.878665	2233	(+)	NNNGATRNNN	TAAGATACAG
M00077	V$GATA3_01	0.86265	2234	(+)	NNGATARNG	AAGATACAG
M00203	V$GATA_C	0.853681	2235	(+)	NGATAAGNMNN	AGATACAGATG
M00127	V$GATA1_03	0.793974	2237	(+)	RNSNNGATAANNGN	ATACAGATGATAAT
M00075	V$GATA1_01	0.777887	2239	(+)	SNNGATNNNN	ACAGATGATA
M00076	V$GATA2_01	0.797023	2239	(+)	NNNGATRNNN	ACAGATGATA
M00077	V$GATA3_01	0.839167	2240	(+)	NNGATARNG	CAGATGATA
M00126	V$GATA1_02	0.8325	2240	(+)	NNNNNGATANKGNN	CAGATGATAATATA
M00127	V$GATA1_03	0.856198	2240	(+)	RNSNNGATAANNGN	CAGATGATAATATA
M00128	V$GATA1_04	0.846201	2241	(+)	NNCWGATARNNNN	AGATGATAATATA
M00075	V$GATA1_01	0.842547	2242	(+)	SNNGATNNNN	GATGATAATA
M00076	V$GATA2_01	0.852954	2242	(+)	NNNGATRNNN	GATGATAATA
M00077	V$GATA3_01	0.875055	2243	(+)	NNGATARNG	ATGATAATA
M00203	V$GATA_C	0.851196	2244	(+)	NGATAAGNMNN	TGATAATATAT
M00127	V$GATA1_03	0.784174	2258	(+)	RNSNNGATAANNGN	GTAAATATAAGAAT
M00127	V$GATA1_03	0.782215	2278	(-)	RNSNNGATAANNGN	TTGTTTATGAGGAT
M00126	V$GATA1_02	0.819375	2284	(+)	NNNNNGATANKGNN	ATGAGGATAATGTC
M00127	V$GATA1_03	0.921362	2284	(+)	RNSNNGATAANNGN	ATGAGGATAATGTC
M00075	V$GATA1_01	0.864265	2286	(+)	SNNGATNNNN	GAGGATAATG
M00076	V$GATA2_01	0.921516	2286	(+)	NNNGATRNNN	GAGGATAATG
M00077	V$GATA3_01	0.888347	2287	(+)	NNGATARNG	AGGATAATG

M00203	V$GATA_C	0.863312	2288	(+)	NGATAAGNMNN	GGATAATGTCA
M00127	V$GATA1_03	0.823616	2300	(+)	RNSNNGATAANNGN	GGGTAGACAGCAGG
M00075	V$GATA1_01	0.84304	2353	(+)	SNNGATNNNN	AAAGATGGCC
M00076	V$GATA2_01	0.885431	2353	(+)	NNNGATRNNN	AAAGATGGCC
M00077	V$GATA3_01	0.825432	2354	(+)	NNGATARNG	AAGATGGCC
M00127	V$GATA1_03	0.785154	2385	(+)	RNSNNGATAANNGN	ATGAAGATGGACAA
M00075	V$GATA1_01	0.868213	2387	(+)	SNNGATNNNN	GAAGATGGAC
M00076	V$GATA2_01	0.873252	2387	(+)	NNNGATRNNN	GAAGATGGAC
M00077	V$GATA3_01	0.821444	2388	(+)	NNGATARNG	AAGATGGAC
M00203	V$GATA_C	0.83908	2393	(+)	NGATAAGNMNN	GGACAAAAATA
M00075	V$GATA1_01	0.814906	2427	(+)	SNNGATNNNN	TAGGATTGGA
M00076	V$GATA2_01	0.857465	2427	(+)	NNNGATRNNN	TAGGATTGGA
M00077	V$GATA3_01	0.868852	2428	(+)	NNGATARNG	AGGATTGGA
M00075	V$GATA1_01	0.787266	2436	(+)	SNNGATNNNN	AAAGATTTTT
M00076	V$GATA2_01	0.797474	2436	(+)	NNNGATRNNN	AAAGATTTTT
M00075	V$GATA1_01	0.84304	2516	(-)	SNNGATNNNN	GAGAATCACT
M00076	V$GATA2_01	0.801985	2516	(-)	NNNGATRNNN	GAGAATCACT
M00075	V$GATA1_01	0.82231	2596	(+)	SNNGATNNNN	GTAGATTCCC
M00076	V$GATA2_01	0.789355	2596	(+)	NNNGATRNNN	GTAGATTCCC
M00075	V$GATA1_01	0.886476	2607	(-)	SNNGATNNNN	AACCATCTCT
M00076	V$GATA2_01	0.878214	2607	(-)	NNNGATRNNN	AACCATCTCT
M00077	V$GATA3_01	0.858662	2607	(-)	NNGATARNG	AACCATCTC
M00076	V$GATA2_01	0.797474	2620	(-)	NNNGATRNNN	GCATATGTCA
M00126	V$GATA1_02	0.815937	2672	(+)	NNNNNGATANKGNN	ACTTTGATAGGAAA
M00127	V$GATA1_03	0.78148	2672	(+)	RNSNNGATAANNGN	ACTTTGATAGGAAA
M00128	V$GATA1_04	0.810662	2673	(+)	NNCWGATARNNNN	CTTTGATAGGAAA
M00075	V$GATA1_01	0.827739	2674	(+)	SNNGATNNNN	TTTGATAGGA
M00076	V$GATA2_01	0.814614	2674	(+)	NNNGATRNNN	TTTGATAGGA
M00077	V$GATA3_01	0.899867	2675	(+)	NNGATARNG	TTGATAGGA
M00203	V$GATA_C	0.909599	2676	(+)	NGATAAGNMNN	TGATAGGAAAC
M00075	V$GATA1_01	0.771471	2680	(-)	SNNGATNNNN	AGGAAACAGG
M00075	V$GATA1_01	0.847483	2687	(+)	SNNGATNNNN	AGGGATGTGT
M00076	V$GATA2_01	0.862427	2687	(+)	NNNGATRNNN	AGGGATGTGT

M00075	V$GATA1_01	0.801086	2715	(+)	SNNGATNNNN	GGAGTTGGGC
M00075	V$GATA1_01	0.832675	2746	(-)	SNNGATNNNN	AAGAATCCAC
M00076	V$GATA2_01	0.788453	2746	(-)	NNNGATRNNN	AAGAATCCAC
M00075	V$GATA1_01	0.807009	2777	(-)	SNNGATNNNN	TTCCATCCCT
M00076	V$GATA2_01	0.822733	2777	(-)	NNNGATRNNN	TTCCATCCCT
M00077	V$GATA3_01	0.825432	2777	(-)	NNGATARNG	TTCCATCCC
M00127	V$GATA1_03	0.786134	2792	(+)	RNSNNGATAANNGN	AGGAAGATCCCATG
M00075	V$GATA1_01	0.80997	2794	(+)	SNNGATNNNN	GAAGATCCCA
M00075	V$GATA1_01	0.777394	2794	(-)	SNNGATNNNN	GAAGATCCCA
M00076	V$GATA2_01	0.844835	2794	(+)	NNNGATRNNN	GAAGATCCCA
M00076	V$GATA2_01	0.848895	2794	(-)	NNNGATRNNN	GAAGATCCCA
M00126	V$GATA1_02	0.784375	2945	(+)	NNNNNGATANKGNN	TGCCAGATAACCTA
M00127	V$GATA1_03	0.822146	2945	(+)	RNSNNGATAANNGN	TGCCAGATAACCTA
M00128	V$GATA1_04	0.869179	2946	(+)	NNCWGATARNNNN	GCCAGATAACCTA
M00075	V$GATA1_01	0.86772	2947	(+)	SNNGATNNNN	CCAGATAACC
M00076	V$GATA2_01	0.912043	2947	(+)	NNNGATRNNN	CCAGATAACC
M00077	V$GATA3_01	0.844927	2948	(+)	NNGATARNG	CAGATAACC
M00203	V$GATA_C	0.847158	2949	(+)	NGATAAGNMNN	AGATAACCTAA
M00075	V$GATA1_01	0.886969	2988	(-)	SNNGATNNNN	CAACATCTTC
M00076	V$GATA2_01	0.89355	2988	(-)	NNNGATRNNN	CAACATCTTC
M00077	V$GATA3_01	0.848028	2988	(-)	NNGATARNG	CAACATCTT
M00203	V$GATA_C	0.881951	3018	(-)	NGATAAGNMNN	TATTCATATCA
M00128	V$GATA1_04	0.883885	3019	(-)	NNCWGATARNNNN	ATTCATATCATAC
M00075	V$GATA1_01	0.816881	3021	(-)	SNNGATNNNN	TCATATCATA
M00076	V$GATA2_01	0.85972	3021	(-)	NNNGATRNNN	TCATATCATA
M00077	V$GATA3_01	0.863979	3021	(-)	NNGATARNG	TCATATCAT
M00126	V$GATA1_02	0.824688	3030	(+)	NNNNNGATANKGNN	ACAGTGATAAAGAA
M00127	V$GATA1_03	0.856198	3030	(+)	RNSNNGATAANNGN	ACAGTGATAAAGAA
M00128	V$GATA1_04	0.833946	3031	(+)	NNCWGATARNNNN	CAGTGATAAAGAA
M00075	V$GATA1_01	0.813919	3032	(+)	SNNGATNNNN	AGTGATAAAG
M00076	V$GATA2_01	0.81687	3032	(+)	NNNGATRNNN	AGTGATAAAG
M00077	V$GATA3_01	0.907399	3033	(+)	NNGATARNG	GTGATAAAG
M00203	V$GATA_C	0.905871	3034	(+)	NGATAAGNMNN	TGATAAAGAAG

M00075	V$GATA1_01	0.865252	3047	(+)	SNNGATNNNN	CTAGATGCTT
M00076	V$GATA2_01	0.804691	3047	(+)	NNNGATRNNN	CTAGATGCTT
M00126	V$GATA1_02	0.886875	3070	(+)	NNNNNGATANKGNN	TTGAAGATACAGGC
M00127	V$GATA1_03	0.803283	3070	(+)	RNSNNGATAANNGN	TTGAAGATACAGGC
M00128	V$GATA1_04	0.841912	3071	(+)	NNCWGATARNNNN	TGAAGATACAGGC
M00075	V$GATA1_01	0.868707	3072	(+)	SNNGATNNNN	GAAGATACAG
M00076	V$GATA2_01	0.893099	3072	(+)	NNNGATRNNN	GAAGATACAG
M00077	V$GATA3_01	0.86265	3073	(+)	NNGATARNG	AAGATACAG
M00203	V$GATA_C	0.881019	3074	(+)	NGATAAGNMNN	AGATACAGGCT
M00075	V$GATA1_01	0.819348	3085	(-)	SNNGATNNNN	TGCAATCTGT
M00076	V$GATA2_01	0.831304	3085	(-)	NNNGATRNNN	TGCAATCTGT
M00077	V$GATA3_01	0.871511	3085	(-)	NNGATARNG	TGCAATCTG
M00076	V$GATA2_01	0.7871	3090	(+)	NNNGATRNNN	TCTGTTAGCT
M00127	V$GATA1_03	0.863792	3090	(-)	RNSNNGATAANNGN	TCTGTTAGCTCCCT
M00203	V$GATA_C	0.934141	3122	(-)	NGATAAGNMNN	TTCCCTTATCC
M00126	V$GATA1_02	0.86375	3123	(-)	NNNNNGATANKGNN	TCCCTTATCCTGCA
M00127	V$GATA1_03	0.850808	3123	(-)	RNSNNGATAANNGN	TCCCTTATCCTGCA
M00128	V$GATA1_04	0.869485	3123	(-)	NNCWGATARNNNN	TCCCTTATCCTGC
M00075	V$GATA1_01	0.881046	3125	(-)	SNNGATNNNN	CCTTATCCTG
M00076	V$GATA2_01	0.922418	3125	(-)	NNNGATRNNN	CCTTATCCTG
M00077	V$GATA3_01	0.909171	3125	(-)	NNGATARNG	CCTTATCCT
M00075	V$GATA1_01	0.788746	3145	(-)	SNNGATNNNN	TGCCAACACC
M00126	V$GATA1_02	0.78	3179	(+)	NNNNNGATANKGNN	GTTCAGATTGGGCT
M00128	V$GATA1_04	0.820159	3180	(+)	NNCWGATARNNNN	TTCAGATTGGGCT
M00075	V$GATA1_01	0.886969	3181	(+)	SNNGATNNNN	TCAGATTGGG
M00076	V$GATA2_01	0.894001	3181	(+)	NNNGATRNNN	TCAGATTGGG
M00077	V$GATA3_01	0.943288	3182	(+)	NNGATARNG	CAGATTGGG
M00203	V$GATA_C	0.835974	3187	(-)	NGATAAGNMNN	TGGGCTTCTCT
M00203	V$GATA_C	0.840323	3209	(+)	NGATAAGNMNN	TGCTAAGGACT
M00076	V$GATA2_01	0.787551	3225	(+)	NNNGATRNNN	TGCCATGCTG
M00076	V$GATA2_01	0.807397	3225	(-)	NNNGATRNNN	TGCCATGCTG
M00127	V$GATA1_03	0.802303	3243	(+)	RNSNNGATAANNGN	GGTTTGATACCAGG
M00075	V$GATA1_01	0.789733	3245	(+)	SNNGATNNNN	TTTGATACCA

M00076	V$GATA2_01	0.801985	3245	(+)	NNNGATRNNN	TTTGATACCA
M00075	V$GATA1_01	0.830701	3262	(+)	SNNGATNNNN	GAAGATCCCC
M00075	V$GATA1_01	0.833662	3262	(-)	SNNGATNNNN	GAAGATCCCC
M00076	V$GATA2_01	0.870997	3262	(+)	NNNGATRNNN	GAAGATCCCC
M00076	V$GATA2_01	0.863329	3262	(-)	NNNGATRNNN	GAAGATCCCC
M00075	V$GATA1_01	0.774926	3335	(+)	SNNGATNNNN	CCAGAGGGTT
M00127	V$GATA1_03	0.783439	3351	(+)	RNSNNGATAANNGN	AGTCAGATACTTTC
M00075	V$GATA1_01	0.861303	3353	(+)	SNNGATNNNN	TCAGATACTT
M00076	V$GATA2_01	0.913848	3353	(+)	NNNGATRNNN	TCAGATACTT
M00128	V$GATA1_04	0.816483	3373	(-)	NNCWGATARNNNN	TCACTTTTCAGAT
M00224	V$STAT1_01	0.720402	23	(+)	NNNSANTTCCGGGAANTGNSN	CTTCCTTTTAGGAAAA-GAGGT
M00223	V$STAT_01	0.853542	28	(-)	TTCCCRKAA	TTTTAGGAA
M00223	V$STAT_01	0.802622	30	(-)	TTCCCRKAA	TTAGGAAAA
M00223	V$STAT_01	0.816738	108	(-)	TTCCCRKAA	TTCCAAAAA
M00223	V$STAT_01	0.80968	213	(+)	TTCCCRKAA	TTCTTAAAA
M00223	V$STAT_01	0.795815	236	(+)	TTCCCRKAA	ATACTAGAA
M00223	V$STAT_01	0.832619	721	(+)	TTCCCRKAA	ATCCTATAA
M00223	V$STAT_01	0.799597	756	(+)	TTCCCRKAA	TTAGTAGAA
M00223	V$STAT_01	0.809932	756	(-)	TTCCCRKAA	TTAGTAGAA
M00223	V$STAT_01	0.832871	1033	(+)	TTCCCRKAA	TTCCAGGCA
M00223	V$STAT_01	0.856819	1033	(-)	TTCCCRKAA	TTCCAGGCA
M00224	V$STAT1_01	0.778363	1162	(-)	NNNSANTTCCGGGAANTGNSN	CCTCTGTTCCTGGGATTCTCC
M00223	V$STAT_01	0.858583	1168	(+)	TTCCCRKAA	TTCCTGGGA
M00223	V$STAT_01	0.832871	1319	(+)	TTCCCRKAA	TTCCAGGCA
M00223	V$STAT_01	0.856819	1319	(-)	TTCCCRKAA	TTCCAGGCA
M00223	V$STAT_01	0.791782	1355	(+)	TTCCCRKAA	TTCTCTGCA
M00223	V$STAT_01	0.804638	1592	(+)	TTCCCRKAA	TCCCCAGCA
M00223	V$STAT_01	0.792538	1754	(+)	TTCCCRKAA	TTAGTATAA
M00223	V$STAT_01	0.922359	1980	(+)	TTCCCRKAA	TTCTTGTAA
M00223	V$STAT_01	0.833627	1980	(-)	TTCCCRKAA	TTCTTGTAA
M00223	V$STAT_01	0.815478	2282	(-)	TTCCCRKAA	TTATGAGGA
M00223	V$STAT_01	0.80968	2285	(-)	TTCCCRKAA	TGAGGATAA
M00223	V$STAT_01	0.839677	2424	(-)	TTCCCRKAA	TTCTAGGAT

M00223	V$STAT_01	0.829846	2512	(+)	TTCCCRKAA	CTCCGAGAA
M00223	V$STAT_01	0.839677	2512	(-)	TTCCCRKAA	CTCCGAGAA
M00223	V$STAT_01	0.80968	2525	(+)	TTCCCRKAA	TTCTTAAAA
M00223	V$STAT_01	0.819763	2562	(+)	TTCCCRKAA	TTCTTTTAA
M00223	V$STAT_01	0.88732	2676	(-)	TTCCCRKAA	TGATAGGAA
M00223	V$STAT_01	0.795815	2995	(-)	TTCCCRKAA	TTCTAGTGA
M00223	V$STAT_01	0.853542	3009	(-)	TTCCCRKAA	TTACAGAAA
M00137	V$OCT1_03	0.849072	39	(+)	NNNRTAATNANNN	GAGGTAATATTTT
M00138	V$OCT1_04	0.878712	40	(-)	NNNNNNNWATGCAAATNNNWNNW	AGGTAA-TATTTTCATTTATCTTT
M00135	V$OCT1_01	0.788821	42	(-)	NNNNWTATGCAAATNTNNN	GTAATATTTTCATTTATCT
M00162	V$OCT1_06	0.9375	44	(+)	CWNAWTKWSATRYN	AATATTTTCATTTA
M00195	V$OCT1_Q6	0.822325	44	(-)	NNNNATGCAAATNAN	AATATTTTCATTTAT
M00248	V$OCT1_07	0.812381	44	(+)	TNTATGNTAATT	AATATTTTCATT
M00138	V$OCT1_04	0.791719	46	(-)	NNNNNNNWATGCAAATNNNWNNW	TATTTTCATTTATCTTTTGC-TA
M00135	V$OCT1_01	0.732163	57	(+)	NNNNWTATGCAAATNTNNN	ATCTTTTTGCTATTCTATG
M00162	V$OCT1_06	0.805469	57	(+)	CWNAWTKWSATRYN	ATCTTTTTGCTATT
M00162	V$OCT1_06	0.834375	76	(+)	CWNAWTKWSATRYN	TACCTTGTAATATT
M00162	V$OCT1_06	0.806641	77	(-)	CWNAWTKWSATRYN	ACCTTGTAATATTG
M00137	V$OCT1_03	0.884631	79	(+)	NNNRTAATNANNN	CTTGTAATATTGT
M00138	V$OCT1_04	0.786909	115	(-)	NNNNNNNWATGCAAATNNNWNNW	AAGTATTTTTTAAA-GAAACTAAT
M00138	V$OCT1_04	0.885822	217	(-)	NNNNNNNWATGCAAATNNNWNNW	TAAAAAAATTAACATA-CACATAC
M00135	V$OCT1_01	0.85578	219	(-)	NNNNWTATGCAAATNTNNN	AAAAAATTAACATACACAT
M00162	V$OCT1_06	0.88125	221	(+)	CWNAWTKWSATRYN	AAAATTAACATACA
M00248	V$OCT1_07	0.993216	223	(-)	TNTATGNTAATT	AATTAACATACA
M00162	V$OCT1_06	0.873437	242	(+)	CWNAWTKWSATRYN	GAAATTGTTATACT
M00162	V$OCT1_06	0.804297	258	(-)	CWNAWTKWSATRYN	TCTATCTTATGATG
M00162	V$OCT1_06	0.801562	265	(+)	CWNAWTKWSATRYN	TATGATGAAATGCC
M00162	V$OCT1_06	0.803516	271	(-)	CWNAWTKWSATRYN	GAAATGCCACAGTT
M00137	V$OCT1_03	0.881865	293	(+)	NNNRTAATNANNN	GAGCTAATCATAA
M00137	V$OCT1_03	0.975504	299	(+)	NNNRTAATNANNN	ATCATAATGAGGT
M00162	V$OCT1_06	0.88125	301	(+)	CWNAWTKWSATRYN	CATAATGAGGTTTT
M00135	V$OCT1_01	0.734071	308	(+)	NNNNWTATGCAAATNTNNN	AGGTTTTTGAAAATCCAGG
M00136	V$OCT1_02	0.785057	311	(-)	NNGAATATKCANNNN	TTTTTGAAAATCCAG

M00162	V$OCT1_06	0.862891	332	(+)	CWNAWTKWSATRYN	GCACATTAGATGTA
M00162	V$OCT1_06	0.821484	333	(-)	CWNAWTKWSATRYN	CACATTAGATGTAT
M00137	V$OCT1_03	0.866456	371	(+)	NNNRTAATNANNN	CTGTTAATCAGAC
M00137	V$OCT1_03	0.860925	465	(+)	NNNRTAATNANNN	TTCATAACTACAA
M00162	V$OCT1_06	0.813672	486	(+)	CWNAWTKWSATRYN	ATGTTTCTCATTTA
M00162	V$OCT1_06	0.860156	487	(-)	CWNAWTKWSATRYN	TGTTTCTCATTTAT
M00162	V$OCT1_06	0.855078	513	(-)	CWNAWTKWSATRYN	AACATTTATTTAAC
M00162	V$OCT1_06	0.800391	516	(+)	CWNAWTKWSATRYN	ATTTATTTAACTCT
M00136	V$OCT1_02	0.783974	532	(+)	NNGAATATKCANNNN	GTTAATATTTGTCCA
M00162	V$OCT1_06	0.800781	545	(-)	CWNAWTKWSATRYN	CATGTTCCAGTTTC
M00162	V$OCT1_06	0.834375	554	(+)	CWNAWTKWSATRYN	GTTTCTTTCATCCA
M00138	V$OCT1_04	0.840234	559	(+)	NNNNNNNNWATGCAAATNNNWNNW	TTTCATCCATGGAAAA-TAAAATA
M00248	V$OCT1_07	0.820013	564	(+)	TNTATGNTAATT	TCCATGGAAAAT
M00162	V$OCT1_06	0.855469	570	(+)	CWNAWTKWSATRYN	GAAAATAAAATATC
M00162	V$OCT1_06	0.813672	570	(-)	CWNAWTKWSATRYN	GAAAATAAAATATC
M00162	V$OCT1_06	0.821484	571	(-)	CWNAWTKWSATRYN	AAAATAAAATATCT
M00248	V$OCT1_07	0.798601	572	(-)	TNTATGNTAATT	AAATAAAATATC
M00138	V$OCT1_04	0.800502	601	(+)	NNNNNNNNWATGCAAATNNNWNNW	AAGTGGCTATA-CATAATGAACTT
M00137	V$OCT1_03	0.894508	610	(+)	NNNRTAATNANNN	TACATAATGAACT
M00138	V$OCT1_04	0.793601	616	(+)	NNNNNNNNWATGCAAATNNNWNNW	ATGAACTTATTTCAAATTTTTAA
M00162	V$OCT1_06	0.821484	621	(-)	CWNAWTKWSATRYN	CTTATTTCAAATTT
M00162	V$OCT1_06	0.860156	623	(-)	CWNAWTKWSATRYN	TATTTCAAATTTTT
M00138	V$OCT1_04	0.786073	626	(-)	NNNNNNNNWATGCAAATNNNWNNW	TTCAAATTTTTAACTTATAAAAT
M00162	V$OCT1_06	0.800391	629	(+)	CWNAWTKWSATRYN	AAATTTTTAACTTA
M00138	V$OCT1_04	0.782309	639	(-)	NNNNNNNNWATGCAAATNNNWNNW	CTTATAAAATTTAATATATATAT
M00138	V$OCT1_04	0.807612	641	(-)	NNNNNNNNWATGCAAATNNNWNNW	TA-TAAAATTTAATATATATATCA
M00135	V$OCT1_01	0.738459	643	(-)	NNNNWTATGCAAATNTNNN	TAAAATTTAATATATATAT
M00162	V$OCT1_06	0.88125	643	(+)	CWNAWTKWSATRYN	TAAAATTTAATATA
M00138	V$OCT1_04	0.799038	644	(+)	NNNNNNNNWATGCAAATNNNWNNW	AAAATTTAATATATATATCATCT
M00162	V$OCT1_06	0.898437	644	(-)	CWNAWTKWSATRYN	AAAATTTAATATAT
M00138	V$OCT1_04	0.807194	645	(-)	NNNNNNNNWATGCAAATNNNWNNW	AAATTTAATATATATATCATCTT
M00138	V$OCT1_04	0.794437	646	(+)	NNNNNNNNWATGCAAATNNNWNNW	AATTTAATATATATATCATCTTA

M00138	V$OCT1_04	0.808867	647	(-)	NNNNNNNWATGCAAATNNNWNNW	ATTTAATATATATATCATCTTAT
M00248	V$OCT1_07	0.793513	647	(-)	TNTATGNTAATT	ATTTAATATATA
M00162	V$OCT1_06	0.857812	665	(+)	CWNAWTKWSATRYN	CTTATTTTAAAACA
M00138	V$OCT1_04	0.792765	671	(-)	NNNNNNNWATGCAAATNNNWNNW	TTAAAACATT-GTCATTATGAAAT
M00162	V$OCT1_06	0.847656	675	(+)	CWNAWTKWSATRYN	AACATTGTCATTAT
M00162	V$OCT1_06	0.812109	676	(-)	CWNAWTKWSATRYN	ACATTGTCATTATG
M00137	V$OCT1_03	0.957329	679	(-)	NNNRTAATNANNN	TTGTCATTATGAA
M00162	V$OCT1_06	0.953125	683	(+)	CWNAWTKWSATRYN	CATTATGAAATGCT
M00138	V$OCT1_04	0.835425	684	(+)	NNNNNNNWATGCAAATNNNWNNW	AT-TATGAAATGCTTATAAAGTGA
M00162	V$OCT1_06	0.842187	684	(-)	CWNAWTKWSATRYN	ATTATGAAATGCTT
M00137	V$OCT1_03	0.843935	695	(+)	NNNRTAATNANNN	CTTATAAAGTGAA
M00162	V$OCT1_06	0.821484	698	(+)	CWNAWTKWSATRYN	ATAAAGTGAATGTC
M00162	V$OCT1_06	0.932422	703	(+)	CWNAWTKWSATRYN	GTGAATGTCATGTG
M00162	V$OCT1_06	0.85	704	(-)	CWNAWTKWSATRYN	TGAATGTCATGTGC
M00135	V$OCT1_01	0.76612	707	(-)	NNNNWTATGCAAATNTNNN	ATGTCATGTGCATTATCCT
M00161	V$OCT1_05	0.850905	709	(+)	MKNATTTGCATAYY	GTCATGTGCATTAT
M00195	V$OCT1_Q6	0.804858	709	(-)	NNNNATGCAAATNAN	GTCATGTGCATTATC
M00136	V$OCT1_02	0.823227	711	(-)	NNGAATATKCANNNN	CATGTGCATTATCCT
M00136	V$OCT1_02	0.770439	735	(-)	NNGAATATKCANNNN	AACAAGAATATCTGG
M00162	V$OCT1_06	0.869922	742	(+)	CWNAWTKWSATRYN	ATATCTGGGATTCT
M00138	V$OCT1_04	0.835425	757	(+)	NNNNNNNWATGCAAATNNNWNNW	TAGTAGAAATGA-TAAATTAATAA
M00162	V$OCT1_06	0.831641	775	(-)	CWNAWTKWSATRYN	AATAACAAAAGCAG
M00138	V$OCT1_04	0.802593	784	(+)	NNNNNNNWATGCAAATNNNWNNW	AG-CAGGCAATGCTAATCTTAA-GA
M00135	V$OCT1_01	0.772987	786	(+)	NNNNWTATGCAAATNTNNN	CAGGCAATGCTAATCTTAA
M00195	V$OCT1_Q6	0.817959	788	(+)	NNNNATGCAAATNAN	GGCAATGCTAATCTT
M00248	V$OCT1_07	0.814925	789	(+)	TNTATGNTAATT	GCAATGCTAATC
M00136	V$OCT1_02	0.776123	804	(-)	NNGAATATKCANNNN	AGAGAGAATAATTCC
M00138	V$OCT1_04	0.785864	811	(-)	NNNNNNNWATGCAAATNNNWNNW	ATAATTCCCATGCATA-CACATTC
M00162	V$OCT1_06	0.804297	811	(+)	CWNAWTKWSATRYN	ATAATTCCCATGCA
M00195	V$OCT1_Q6	0.820415	811	(-)	NNNNATGCAAATNAN	ATAATTCCCATGCAT
M00138	V$OCT1_04	0.794228	812	(+)	NNNNNNNWATGCAAATNNNWNNW	TAATTCCCATGCATA-CACATTCT
M00248	V$OCT1_07	0.849693	813	(-)	TNTATGNTAATT	AATTCCCATGCA

M00136	V$OCT1_02	0.812128	817	(-)	NNGAATATKCANNNN	CCCATGCATACACAT
M00162	V$OCT1_06	0.809375	830	(-)	CWNAWTKWSATRYN	ATTCTCTAAATTTG
M00138	V$OCT1_04	0.844835	832	(-)	NNNNNNNWATGCAAATNNNWNNW	TCTCTAAATTT-GCACAGGCAAAG
M00135	V$OCT1_01	0.823922	834	(-)	NNNNWTATGCAAATNTNNN	TCTAAATTTGCACAGGCAA
M00248	V$OCT1_07	0.833369	838	(-)	TNTATGNTAATT	AATTTGCACAGG
M00138	V$OCT1_04	0.80343	839	(+)	NNNNNNNWATGCAAATNNNWNNW	ATTTGCACAGGCAAA-GATCACCA
M00135	V$OCT1_01	0.742655	841	(+)	NNNNWTATGCAAATNTNNN	TTGCACAGGCAAAGATCAC
M00162	V$OCT1_06	0.816016	844	(-)	CWNAWTKWSATRYN	CACAGGCAAAGATC
M00162	V$OCT1_06	0.816406	863	(-)	CWNAWTKWSATRYN	CAAATTTAACAATT
M00138	V$OCT1_04	0.809076	872	(+)	NNNNNNNWATGCAAATNNNWNNW	CAATTTTGAGTCAAA-TAAAATCT
M00162	V$OCT1_06	0.842187	890	(+)	CWNAWTKWSATRYN	AATCTTGCCATTTA
M00162	V$OCT1_06	0.808203	905	(+)	CWNAWTKWSATRYN	AAATAATTGATTTC
M00162	V$OCT1_06	0.821484	906	(+)	CWNAWTKWSATRYN	AATAATTGATTTCA
M00138	V$OCT1_04	0.814931	907	(+)	NNNNNNNWATGCAAATNNNWNNW	ATAATTGATTTCAAATTT-GTAGA
M00162	V$OCT1_06	0.839844	912	(-)	CWNAWTKWSATRYN	TGATTTCAAATTTG
M00138	V$OCT1_04	0.783563	914	(-)	NNNNNNNWATGCAAATNNNWNNW	ATTTCAAATTT-GTAGATCTACAG
M00138	V$OCT1_04	0.784818	915	(+)	NNNNNNNWATGCAAATNNNWNNW	TTTCAAATTT-GTAGATCTACAGA
M00135	V$OCT1_01	0.775849	916	(-)	NNNNWTATGCAAATNTNNN	TTCAAATTTGTAGATCTAC
M00162	V$OCT1_06	0.821484	1021	(+)	CWNAWTKWSATRYN	ATCCATGGGATCTT
M00135	V$OCT1_01	0.740176	1058	(-)	NNNNWTATGCAAATNTNNN	TTGCCATTTCCACACTATT
M00137	V$OCT1_03	0.86132	1069	(-)	NNNRTAATNANNN	ACACTATTATATG
M00162	V$OCT1_06	0.871094	1070	(+)	CWNAWTKWSATRYN	CACTATTATATGTC
M00162	V$OCT1_06	0.950391	1076	(-)	CWNAWTKWSATRYN	TATATGTCAAAAAG
M00162	V$OCT1_06	0.869922	1167	(+)	CWNAWTKWSATRYN	GTTCCTGGGATTCT
M00162	V$OCT1_06	0.868359	1223	(-)	CWNAWTKWSATRYN	TGCATGAAAGTGAA
M00138	V$OCT1_04	0.801757	1224	(+)	NNNNNNNWATGCAAATNNNWNNW	GCATGAAAGTGAAAAAAAGTTAA
M00162	V$OCT1_06	0.839453	1229	(-)	CWNAWTKWSATRYN	AAAGTGAAAAAAAG
M00162	V$OCT1_06	0.877734	1307	(+)	CWNAWTKWSATRYN	ATCCATGGGATTTT
M00138	V$OCT1_04	0.821205	1363	(+)	NNNNNNNWATGCAAATNNNWNNW	AAAAAAGCATTAAAA-TAACTTTA
M00138	V$OCT1_04	0.806357	1364	(-)	NNNNNNNWATGCAAATNNNWNNW	AAAAAGCATTAAAA-TAACTTTAT
M00195	V$OCT1_Q6	0.819323	1367	(+)	NNNNATGCAAATNAN	AAGCATTAAAATAAC
M00162	V$OCT1_06	0.929688	1368	(-)	CWNAWTKWSATRYN	AGCATTAAAATAAC
M00248	V$OCT1_07	0.798601	1370	(-)	TNTATGNTAATT	CATTAAAATAAC

M00162	V$OCT1_06	0.805859	1377	(+)	CWNAWTKWSATRYN	ATAACTTTATTTCA
M00162	V$OCT1_06	0.847266	1381	(+)	CWNAWTKWSATRYN	CTTTATTTCACTTT
M00138	V$OCT1_04	0.785027	1384	(-)	NNNNNNNNWATGCAAATNNNWNNW	TATTTCACTTTTCAG-TTATTTGT
M00162	V$OCT1_06	0.821484	1389	(-)	CWNAWTKWSATRYN	CACTTTTCAGTTAT
M00135	V$OCT1_01	0.740366	1396	(-)	NNNNWTATGCAAATNTNNN	CAGTTATTTGTTTATGCAC
M00195	V$OCT1_Q6	0.811681	1398	(-)	NNNNATGCAAATNAN	GTTATTTGTTTATGC
M00137	V$OCT1_03	0.878309	1401	(-)	NNNRTAATNANNN	ATTTGTTTATGCA
M00136	V$OCT1_02	0.789117	1403	(+)	NNGAATATKCANNNN	TTGTTTATGCACTGG
M00136	V$OCT1_02	0.784245	1427	(+)	NNGAATATKCANNNN	ATCGATATGTAGTCA
M00162	V$OCT1_06	0.829687	1452	(+)	CWNAWTKWSATRYN	GGGACTTATATGTG
M00162	V$OCT1_06	0.839844	1471	(-)	CWNAWTKWSATRYN	AGTATTAGAGATTG
M00162	V$OCT1_06	0.804297	1509	(-)	CWNAWTKWSATRYN	AAAATCTCGTTTGG
M00136	V$OCT1_02	0.783433	1522	(+)	NNGAATATKCANNNN	GTGGTTATTCTGTCA
M00162	V$OCT1_06	0.800391	1537	(+)	CWNAWTKWSATRYN	GGTTCAGTGATGTT
M00162	V$OCT1_06	0.804687	1599	(+)	CWNAWTKWSATRYN	CAGAGTGTTATTTT
M00195	V$OCT1_Q6	0.800491	1605	(-)	NNNNATGCAAATNAN	GTTATTTTCACCTGC
M00162	V$OCT1_06	0.821484	1613	(-)	CWNAWTKWSATRYN	CACCTGCCAATTCT
M00162	V$OCT1_06	0.80625	1660	(+)	CWNAWTKWSATRYN	CTCACTTGATTTTA
M00138	V$OCT1_04	0.846508	1684	(-)	NNNNNNNNWATGCAAATNNNWNNW	TGTTGTTTTTT-GCATTTCTGATG
M00135	V$OCT1_01	0.795498	1686	(-)	NNNNWTATGCAAATNTNNN	TTGTTTTTTGCATTTCTGA
M00162	V$OCT1_06	0.877734	1688	(+)	CWNAWTKWSATRYN	GTTTTTTGCATTTC
M00195	V$OCT1_Q6	0.894378	1688	(-)	NNNNATGCAAATNAN	GTTTTTTGCATTTCT
M00138	V$OCT1_04	0.792555	1722	(+)	NNNNNNNNWATGCAAATNNNWNNW	TAAGAAGAATT-GTAAAAAAAAAT
M00138	V$OCT1_04	0.790673	1723	(+)	NNNNNNNNWATGCAAATNNNWNNW	AAGAAGAATT-GTAAAAAAAAATA
M00162	V$OCT1_06	0.829297	1741	(+)	CWNAWTKWSATRYN	AAATATGGCATTAT
M00162	V$OCT1_06	0.889062	1742	(-)	CWNAWTKWSATRYN	AATATGGCATTATT
M00137	V$OCT1_03	0.840774	1745	(-)	NNNRTAATNANNN	ATGGCATTATTAG
M00138	V$OCT1_04	0.835634	1746	(-)	NNNNNNNNWATGCAAATNNNWNNW	TGGCATTATTAG-TATAAACATCA
M00135	V$OCT1_01	0.792255	1748	(-)	NNNNWTATGCAAATNTNNN	GCATTATTAGTATAAACAT
M00137	V$OCT1_03	0.840379	1748	(-)	NNNRTAATNANNN	GCATTATTAGTAT
M00195	V$OCT1_Q6	0.808133	1750	(-)	NNNNATGCAAATNAN	ATTATTAGTATAAAC
M00138	V$OCT1_04	0.831451	1752	(-)	NNNNNNNNWATGCAAATNNNWNNW	TATTAGTATAAACATCAC-TATTT
M00248	V$OCT1_07	0.800297	1752	(-)	TNTATGNTAATT	TATTAGTATAAA

M00162	V$OCT1_06	0.860156	1762	(-)	CWNAWTKWSATRYN	AACATCACTATTTT
M00138	V$OCT1_04	0.805521	1765	(-)	NNNNNNNWATGCAAATNNNWNNW	ATCACTATTTTTCATCACTG-TAT
M00162	V$OCT1_06	0.821484	1804	(+)	CWNAWTKWSATRYN	GTACCTGACATAGC
M00138	V$OCT1_04	0.834588	1829	(+)	NNNNNNNWATGCAAATNNNWNNW	GAATAAGTGGGTAAATTAA-TAAA
M00138	V$OCT1_04	0.802593	1835	(+)	NNNNNNNWATGCAAATNNNWNNW	GTGGGTAAATTAATAAA-TAAATT
M00248	V$OCT1_07	0.793513	1842	(-)	TNTATGNTAATT	AATTAATAAATA
M00137	V$OCT1_03	0.847886	1844	(+)	NNNRTAATNANNN	TTAATAAATAAAT
M00162	V$OCT1_06	0.809375	1846	(-)	CWNAWTKWSATRYN	AATAAATAAATTAG
M00138	V$OCT1_04	0.780217	1849	(-)	NNNNNNNWATGCAAATNNNWNNW	AAATAAATT-AGCCATTTATACAT
M00162	V$OCT1_06	0.834375	1895	(-)	CWNAWTKWSATRYN	GGTATGCCCAAATG
M00162	V$OCT1_06	0.808203	1923	(-)	CWNAWTKWSATRYN	AGAAACTAAGATTT
M00162	V$OCT1_06	0.834766	1924	(+)	CWNAWTKWSATRYN	GAAACTAAGATTTC
M00162	V$OCT1_06	0.808594	1931	(+)	CWNAWTKWSATRYN	AGATTTCACATTTC
M00162	V$OCT1_06	0.826562	1932	(-)	CWNAWTKWSATRYN	GATTTCACATTTCC
M00162	V$OCT1_06	0.837109	1937	(-)	CWNAWTKWSATRYN	CACATTTCCTTCTC
M00138	V$OCT1_04	0.786491	1948	(-)	NNNNNNNWATGCAAATNNNWNNW	CTCCAGAT-TTTACATTATGTTGT
M00135	V$OCT1_01	0.765738	1950	(-)	NNNNWTATGCAAATNTNNN	CCAGATTTTACATTATGTT
M00162	V$OCT1_06	0.821484	1952	(+)	CWNAWTKWSATRYN	AGATTTTACATTAT
M00162	V$OCT1_06	0.860547	1953	(-)	CWNAWTKWSATRYN	GATTTTACATTATG
M00248	V$OCT1_07	0.793089	1954	(-)	TNTATGNTAATT	ATTTTACATTAT
M00137	V$OCT1_03	0.855393	1956	(-)	NNNRTAATNANNN	TTTACATTATGTT
M00138	V$OCT1_04	0.797992	1961	(+)	NNNNNNNWATGCAAATNNNWNNW	ATTATGTT-GTGTTAATTTATTCT
M00135	V$OCT1_01	0.739412	1963	(+)	NNNNWTATGCAAATNTNNN	TATGTTGTGTTAATTTATT
M00248	V$OCT1_07	0.857325	1966	(+)	TNTATGNTAATT	GTTGTGTTAATT
M00195	V$OCT1_Q6	0.798581	1979	(+)	NNNNATGCAAATNAN	ATTCTTGTAAAGAAC
M00137	V$OCT1_03	0.847886	1982	(+)	NNNRTAATNANNN	CTTGTAAAGAACT
M00137	V$OCT1_03	0.850257	1991	(-)	NNNRTAATNANNN	AACTCATTGTATT
M00135	V$OCT1_01	0.776421	1995	(+)	NNNNWTATGCAAATNTNNN	CATTGTATTCAAATCCATG
M00195	V$OCT1_Q6	0.795306	1997	(+)	NNNNATGCAAATNAN	TTGTATTCAAATCCA
M00162	V$OCT1_06	0.826953	1998	(-)	CWNAWTKWSATRYN	TGTATTCAAATCCA
M00248	V$OCT1_07	0.819377	1998	(+)	TNTATGNTAATT	TGTATTCAAATC
M00136	V$OCT1_02	0.775041	2020	(-)	NNGAATATKCANNNN	TCAATGAATCTACTT
M00137	V$OCT1_03	0.8629	2042	(-)	NNNRTAATNANNN	TCTTCATTGCCCT

M00248	V$OCT1_07	0.798601	2052	(+)	TNTATGNTAATT	CCTTTTCTAATT
M00138	V$OCT1_04	0.781054	2054	(-)	NNNNNNNWATGCAAATNNNWNNW	TTTTCTAATTATCATCTGAA-GAT
M00137	V$OCT1_03	0.847491	2055	(+)	NNNRTAATNANNN	TTTCTAATTATCA
M00137	V$OCT1_03	0.925326	2056	(-)	NNNRTAATNANNN	TTCTAATTATCAT
M00161	V$OCT1_05	0.865682	2058	(+)	MKNATTTGCATAYY	CTAATTATCATCTG
M00162	V$OCT1_06	0.809766	2058	(+)	CWNAWTKWSATRYN	CTAATTATCATCTG
M00162	V$OCT1_06	0.9375	2074	(+)	CWNAWTKWSATRYN	GATAATTTAATACA
M00138	V$OCT1_04	0.836679	2075	(+)	NNNNNNNWATGCAAATNNNWNNW	ATAATTTAATACATAATTAATAA
M00162	V$OCT1_06	0.808594	2075	(-)	CWNAWTKWSATRYN	ATAATTTAATACAT
M00137	V$OCT1_03	0.890952	2084	(+)	NNNRTAATNANNN	TACATAATTAATA
M00137	V$OCT1_03	0.873963	2085	(-)	NNNRTAATNANNN	ACATAATTAATAA
M00137	V$OCT1_03	0.852232	2088	(+)	NNNRTAATNANNN	TAATTAATAAGGA
M00135	V$OCT1_01	0.803892	2090	(+)	NNNNWTATGCAAATNTNNN	ATTAATAAGGAAATATGTG
M00162	V$OCT1_06	0.842187	2093	(+)	CWNAWTKWSATRYN	AATAAGGAAATATG
M00162	V$OCT1_06	0.817187	2093	(-)	CWNAWTKWSATRYN	AATAAGGAAATATG
M00136	V$OCT1_02	0.772063	2098	(+)	NNGAATATKCANNNN	GGAAATATGTGATTA
M00162	V$OCT1_06	0.834375	2100	(+)	CWNAWTKWSATRYN	AAATATGTGATTAT
M00162	V$OCT1_06	0.832812	2101	(-)	CWNAWTKWSATRYN	AATATGTGATTATA
M00137	V$OCT1_03	0.932833	2104	(-)	NNNRTAATNANNN	ATGTGATTATAAG
M00162	V$OCT1_06	0.847266	2132	(+)	CWNAWTKWSATRYN	ATTAATTAGCTTCT
M00162	V$OCT1_06	0.813281	2150	(-)	CWNAWTKWSATRYN	TATTTCACAGGATC
M00137	V$OCT1_03	0.866851	2163	(+)	NNNRTAATNANNN	CAAGTAAACATGA
M00162	V$OCT1_06	0.816016	2168	(+)	CWNAWTKWSATRYN	AAACATGAAGTTCT
M00162	V$OCT1_06	0.862891	2169	(-)	CWNAWTKWSATRYN	AACATGAAGTTCTT
M00162	V$OCT1_06	0.808203	2175	(+)	CWNAWTKWSATRYN	AAGTTCTTCATTTT
M00138	V$OCT1_04	0.783772	2179	(-)	NNNNNNNWATGCAAATNNNWNNW	TCTTCATTTTTAC-CTGCCTTTTG
M00137	V$OCT1_03	0.864876	2182	(-)	NNNRTAATNANNN	TCATTTTTACCTG
M00162	V$OCT1_06	0.862891	2183	(+)	CWNAWTKWSATRYN	CATTTTTACCTGCC
M00195	V$OCT1_Q6	0.829421	2208	(+)	NNNNATGCAAATNAN	GCCCTTGCAAAGCAT
M00135	V$OCT1_01	0.736169	2215	(+)	NNNNWTATGCAAATNTNNN	CAAAGCATGTAAGTATAAT
M00162	V$OCT1_06	0.896875	2218	(-)	CWNAWTKWSATRYN	AGCATGTAAGTATA
M00137	V$OCT1_03	0.914658	2226	(+)	NNNRTAATNANNN	AGTATAATAAGAT
M00162	V$OCT1_06	0.825	2228	(+)	CWNAWTKWSATRYN	TATAATAAGATACA

M00138	V$OCT1_04	0.792765	2229	(+)	NNNNNNNWATGCAAATNNNWNNW	ATAATAAGATACAGATGA-TAATA
M00138	V$OCT1_04	0.801129	2235	(+)	NNNNNNNWATGCAAATNNNWNNW	AGATACAGATGA-TAATATATTAT
M00135	V$OCT1_01	0.751431	2237	(+)	NNNNWTATGCAAATNTNNN	ATACAGATGATAATATATT
M00138	V$OCT1_04	0.888122	2248	(+)	NNNNNNNWATGCAAATNNNWNNW	AATATATTATGTAAA-TATAAGAA
M00135	V$OCT1_01	0.872758	2250	(+)	NNNNWTATGCAAATNTNNN	TATATTATGTAAATATAAG
M00162	V$OCT1_06	0.839453	2253	(+)	CWNAWTKWSATRYN	ATTATGTAAATATA
M00162	V$OCT1_06	0.825	2253	(-)	CWNAWTKWSATRYN	ATTATGTAAATATA
M00248	V$OCT1_07	0.847149	2253	(+)	TNTATGNTAATT	ATTATGTAAATA
M00138	V$OCT1_04	0.780427	2254	(+)	NNNNNNNWATGCAAATNNNWNNW	TTATGTAAATATAAGAA-TATTAA
M00137	V$OCT1_03	0.851442	2255	(+)	NNNRTAATNANNN	TATGTAAATATAA
M00138	V$OCT1_04	0.857382	2257	(-)	NNNNNNNWATGCAAATNNNWNNW	TGTAAATATAAGAA-TATTAAATT
M00135	V$OCT1_01	0.750286	2259	(-)	NNNNWTATGCAAATNTNNN	TAAATATAAGAATATTAAA
M00138	V$OCT1_04	0.795483	2262	(+)	NNNNNNNWATGCAAATNNNWNNW	ATATAAGAATATTAAATT-GTTTA
M00136	V$OCT1_02	0.86248	2263	(-)	NNGAATATKCANNNN	TATAAGAATATTAAA
M00136	V$OCT1_02	0.838657	2266	(+)	NNGAATATKCANNNN	AAGAATATTAAATTG
M00162	V$OCT1_06	0.829297	2268	(+)	CWNAWTKWSATRYN	GAATATTAAATTGT
M00162	V$OCT1_06	0.924609	2269	(-)	CWNAWTKWSATRYN	AATATTAAATTGTT
M00137	V$OCT1_03	0.875543	2276	(-)	NNNRTAATNANNN	AATTGTTTATGAG
M00137	V$OCT1_03	0.84354	2287	(+)	NNNRTAATNANNN	AGGATAATGTCAG
M00138	V$OCT1_04	0.781681	2319	(+)	NNNNNNNWATGCAAATNNNWNNW	GAGAGGAAGTG-TATAAATTTTGA
M00138	V$OCT1_04	0.790673	2328	(-)	NNNNNNNWATGCAAATNNNWNNW	TGTATAAATTTTGAAAC-CTATAA
M00137	V$OCT1_03	0.866456	2344	(+)	NNNRTAATNANNN	CCTATAATCAAAG
M00162	V$OCT1_06	0.826562	2346	(-)	CWNAWTKWSATRYN	TATAATCAAAGATG
M00135	V$OCT1_01	0.795307	2370	(+)	NNNNWTATGCAAATNTNNN	TGCCTTATGTAAGTTATGA
M00162	V$OCT1_06	0.847656	2373	(-)	CWNAWTKWSATRYN	CTTATGTAAGTTAT
M00248	V$OCT1_07	0.821709	2373	(+)	TNTATGNTAATT	CTTATGTAAGTT
M00138	V$OCT1_04	0.797365	2465	(-)	NNNNNNNWATGCAAATNNNWNNW	TACACTTTCTTCCA-TAAACATTT
M00138	V$OCT1_04	0.801547	2471	(-)	NNNNNNNWATGCAAATNNNWNNW	TTCTTCCATAAACATTT-GAATTT
M00137	V$OCT1_03	0.865271	2475	(+)	NNNRTAATNANNN	TCCATAAACATTT
M00135	V$OCT1_01	0.765929	2479	(-)	NNNNWTATGCAAATNTNNN	TAAACATTTGAATTTACTG
M00162	V$OCT1_06	0.821094	2480	(+)	CWNAWTKWSATRYN	AAACATTTGAATTT
M00162	V$OCT1_06	0.891016	2481	(+)	CWNAWTKWSATRYN	AACATTTGAATTTA
M00162	V$OCT1_06	0.812109	2481	(-)	CWNAWTKWSATRYN	AACATTTGAATTTA

M00195	V$OCT1_Q6	0.792849	2481	(-)	NNNNATGCAAATNAN	AACATTTGAATTTAC
M00136	V$OCT1_02	0.793449	2483	(-)	NNGAATATKCANNNN	CATTTGAATTTACTG
M00162	V$OCT1_06	0.834375	2502	(+)	CWNAWTKWSATRYN	AATATTTTCACTCC
M00162	V$OCT1_06	0.808203	2529	(-)	CWNAWTKWSATRYN	TAAAAGTAAGAAAC
M00137	V$OCT1_03	0.842355	2548	(+)	NNNRTAATNANNN	ACCATAACAAGGA
M00162	V$OCT1_06	0.839453	2561	(+)	CWNAWTKWSATRYN	CTTCTTTTAAAGTT
M00138	V$OCT1_04	0.785864	2586	(+)	NNNNNNNWATGCAAATNNNWNNW	CAGAGAAAATGTAGAT-TCCCAAA
M00136	V$OCT1_02	0.783162	2588	(+)	NNGAATATKCANNNN	GAGAAAATGTAGATT
M00195	V$OCT1_Q6	0.833515	2590	(+)	NNNNATGCAAATNAN	GAAAATGTAGATTCC
M00162	V$OCT1_06	0.834375	2591	(+)	CWNAWTKWSATRYN	AAAATGTAGATTCC
M00162	V$OCT1_06	0.834375	2591	(-)	CWNAWTKWSATRYN	AAAATGTAGATTCC
M00248	V$OCT1_07	0.793089	2591	(+)	TNTATGNTAATT	AAAATGTAGATT
M00162	V$OCT1_06	0.800391	2610	(-)	CWNAWTKWSATRYN	CATCTCTCAAGCAT
M00136	V$OCT1_02	0.780455	2618	(+)	NNGAATATKCANNNN	AAGCATATGTCAGAA
M00162	V$OCT1_06	0.90625	2621	(-)	CWNAWTKWSATRYN	CATATGTCAGAATT
M00162	V$OCT1_06	0.825391	2628	(+)	CWNAWTKWSATRYN	CAGAATTTCATCAT
M00162	V$OCT1_06	0.85	2629	(-)	CWNAWTKWSATRYN	AGAATTTCATCATT
M00162	V$OCT1_06	0.866016	2669	(+)	CWNAWTKWSATRYN	CATACTTTGATAGG
M00162	V$OCT1_06	0.821484	2682	(+)	CWNAWTKWSATRYN	GAAACAGGGATGTG
M00137	V$OCT1_03	0.841169	2740	(+)	NNNRTAATNANNN	GTGGTAAAGAATC
M00162	V$OCT1_06	0.847656	2795	(-)	CWNAWTKWSATRYN	AAGATCCCATGGAG
M00162	V$OCT1_06	0.816016	2880	(+)	CWNAWTKWSATRYN	GGCCATGAGTTGCA
M00162	V$OCT1_06	0.813672	2928	(+)	CWNAWTKWSATRYN	CAACTTTGAAAATG
M00195	V$OCT1_Q6	0.826692	2929	(+)	NNNNATGCAAATNAN	AACTTTGAAAATGAC
M00162	V$OCT1_06	0.870703	2935	(+)	CWNAWTKWSATRYN	GAAAATGACTTGCC
M00138	V$OCT1_04	0.808657	2951	(+)	NNNNNNNWATGCAAATNNNWNNW	ATAAC-CTAATGCAAAGTCAACCC
M00135	V$OCT1_01	0.784624	2953	(+)	NNNNWTATGCAAATNTNNN	AACCTAATGCAAAGTCAAC
M00195	V$OCT1_Q6	0.802129	2955	(+)	NNNNATGCAAATNAN	CCTAATGCAAAGTCA
M00162	V$OCT1_06	0.821484	2968	(+)	CWNAWTKWSATRYN	CAACCCTTCATTTC
M00162	V$OCT1_06	0.826562	3008	(+)	CWNAWTKWSATRYN	GTTACAGAAATATT
M00136	V$OCT1_02	0.842177	3010	(-)	NNGAATATKCANNNN	TACAGAAATATTCAT
M00138	V$OCT1_04	0.801547	3010	(-)	NNNNNNNWATGCAAATNNNWNNW	TACAGAAATATTCAT-ATCATACA
M00138	V$OCT1_04	0.852363	3011	(+)	NNNNNNNWATGCAAATNNNWNNW	ACAGAAATATTCATATCAT-ACAG

M00135	V$OCT1_01	0.762495	3012	(-)	NNNNWTATGCAAATNTNNN	CAGAAATATTCATATCATA
M00135	V$OCT1_01	0.734643	3013	(+)	NNNNWTATGCAAATNTNNN	AGAAATATTCATATCATAC
M00136	V$OCT1_02	0.906876	3013	(+)	NNGAATATKCANNNN	AGAAATATTCATATC
M00162	V$OCT1_06	0.847266	3014	(+)	CWNAWTKWSATRYN	GAAATATTCATATC
M00162	V$OCT1_06	0.805859	3016	(-)	CWNAWTKWSATRYN	AATATTCATATCAT
M00162	V$OCT1_06	0.847266	3019	(+)	CWNAWTKWSATRYN	ATTCATATCATACA
M00137	V$OCT1_03	0.858949	3033	(+)	NNNRTAATNANNN	GTGATAAAGAAGG
M00195	V$OCT1_Q6	0.796124	3051	(-)	NNNNATGCAAATNAN	ATGCTTCACATGAAG
M00162	V$OCT1_06	0.860156	3052	(-)	CWNAWTKWSATRYN	TGCTTCACATGAAG
M00162	V$OCT1_06	0.821484	3135	(+)	CWNAWTKWSATRYN	CAGCCTGTGCTGCC
M00162	V$OCT1_06	0.839844	3182	(+)	CWNAWTKWSATRYN	CAGATTGGGCTTCT
M00162	V$OCT1_06	0.855469	3220	(+)	CWNAWTKWSATRYN	CTGCCTGCCATGCT
M00162	V$OCT1_06	0.85	3221	(-)	CWNAWTKWSATRYN	TGCCTGCCATGCTG
M00162	V$OCT1_06	0.807031	3312	(+)	CWNAWTKWSATRYN	GAGAATTCCATGGA
M00162	V$OCT1_06	0.864844	3313	(-)	CWNAWTKWSATRYN	AGAATTCCATGGAC
M00162	V$OCT1_06	0.924609	3356	(+)	CWNAWTKWSATRYN	GATACTTTCATTCA
M00059	V$YY1_01	0.806029	20	(+)	NNNNNCCATNTWNNNWN	TGTCTTCCTTTTAGGAA
M00059	V$YY1_01	0.829292	46	(+)	NNNNNCCATNTWNNNWN	TATTTTCATTTATCTTT
M00059	V$YY1_01	0.782438	97	(+)	NNNNNCCATNTWNNNWN	TAATAGCATTGTTCCAA
M00059	V$YY1_01	0.807995	488	(+)	NNNNNCCATNTWNNNWN	GTTTCTCATTTATCAAC
M00059	V$YY1_01	0.807012	509	(+)	NNNNNCCATNTWNNNWN	TAAAAACATTTATTTAA
M00059	V$YY1_01	0.812254	539	(+)	NNNNNCCATNTWNNNWN	TTTGTCCATGTTCCAGT
M00059	V$YY1_01	0.772608	559	(-)	NNNNNCCATNTWNNNWN	TTTCATCCATGGAAAAT
M00059	V$YY1_01	0.798493	608	(-)	NNNNNCCATNTWNNNWN	TATACATAATGAACTTA
M00059	V$YY1_01	0.790957	677	(+)	NNNNNCCATNTWNNNWN	CATTGTCATTATGAAAT
M00059	V$YY1_01	0.773263	679	(-)	NNNNNCCATNTWNNNWN	TTGTCATTATGAAATGC
M00059	V$YY1_01	0.794233	684	(-)	NNNNNCCATNTWNNNWN	ATTATGAAATGCTTATA
M00059	V$YY1_01	0.792595	738	(-)	NNNNNCCATNTWNNNWN	AAGAATATCTGGGATTC
M00059	V$YY1_01	0.793578	813	(+)	NNNNNCCATNTWNNNWN	AATTCCCATGCATACAC
M00059	V$YY1_01	0.883683	892	(+)	NNNNNCCATNTWNNNWN	TCTTGCCATTTAAAAAT
M00059	V$YY1_01	0.802425	1017	(-)	NNNNNCCATNTWNNNWN	CTCCATCCATGGGATCT
M00059	V$YY1_01	0.864351	1056	(+)	NNNNNCCATNTWNNNWN	GGTTGCCATTTCCACAC
M00059	V$YY1_01	0.771953	1133	(+)	NNNNNCCATNTWNNNWN	TGACCCCATACACAGCA

M00069	V$YY1_02	0.763656	1145	(+)	NNNCGGCCATCTTGNCTSNW	CAGCAGCCCACTAGGCTCCT
M00059	V$YY1_01	0.783093	1202	(+)	NNNNNCCATNTWNNNWN	GGTTGCCATTTCCTTCT
M00059	V$YY1_01	0.813237	1303	(-)	NNNNNCCATNTWNNNWN	CTCCATCCATGGGATTT
M00059	V$YY1_01	0.773263	1545	(+)	NNNNNCCATNTWNNNWN	GATGTTCAGTGATGCTC
M00059	V$YY1_01	0.859436	1644	(+)	NNNNNCCATNTWNNNWN	AGGCTCCATGTTTTGGC
M00059	V$YY1_01	0.865334	1737	(-)	NNNNNCCATNTWNNNWN	AAAAAAATATGGCATTA
M00059	V$YY1_01	0.804063	1764	(+)	NNNNNCCATNTWNNNWN	CATCACTATTTTTCATC
M00059	V$YY1_01	0.828637	1855	(+)	NNNNNCCATNTWNNNWN	ATTAGCCATTTATACAT
M00059	V$YY1_01	0.788336	1898	(-)	NNNNNCCATNTWNNNWN	ATGCCCAAATGAGCCTC
M00059	V$YY1_01	0.770315	1945	(+)	NNNNNCCATNTWNNNWN	CTTCTCCAGATTTTACA
M00059	V$YY1_01	0.814548	2015	(-)	NNNNNCCATNTWNNNWN	GTTTATCAATGAATCTA
M00069	V$YY1_02	0.758122	2037	(-)	NNNCGGCCATCTTGNCTSNW	ATCAGTCTTCATTGCCCTTT
M00059	V$YY1_01	0.822084	2046	(+)	NNNNNCCATNTWNNNWN	CATTGCCCTTTTCTAAT
M00059	V$YY1_01	0.78637	2096	(-)	NNNNNCCATNTWNNNWN	AAGGAAATATGTGATTA
M00059	V$YY1_01	0.778506	2119	(-)	NNNNNCCATNTWNNNWN	GAGTAAAACTGTTATTA
M00059	V$YY1_01	0.869594	2164	(-)	NNNNNCCATNTWNNNWN	AAGTAAACATGAAGTTC
M00059	V$YY1_01	0.808978	2177	(+)	NNNNNCCATNTWNNNWN	GTTCTTCATTTTTACCT
M00059	V$YY1_01	0.775557	2214	(+)	NNNNNCCATNTWNNNWN	GCAAAGCATGTAAGTAT
M00069	V$YY1_02	0.863977	2347	(-)	NNNCGGCCATCTTGNCTSNW	ATAATCAAAGATGGCCTTGG
M00059	V$YY1_01	0.905308	2349	(-)	NNNNNCCATNTWNNNWN	AATCAAAGATGGCCTTG
M00059	V$YY1_01	0.816514	2383	(-)	NNNNNCCATNTWNNNWN	TTATGAAGATGGACAAA
M00059	V$YY1_01	0.774574	2535	(-)	NNNNNCCATNTWNNNWN	TAAGAAACATGAAACCA
M00059	V$YY1_01	0.836173	2586	(-)	NNNNNCCATNTWNNNWN	CAGAGAAAATGTAGATT
M00059	V$YY1_01	0.857471	2604	(+)	NNNNNCCATNTWNNNWN	CCAAACCATCTCTCAAG
M00059	V$YY1_01	0.834862	2663	(+)	NNNNNCCATNTWNNNWN	AACAACCATACTTTGAT
M00059	V$YY1_01	0.783093	2807	(-)	NNNNNCCATNTWNNNWN	AGAAGGAAATGGCAACC
M00059	V$YY1_01	0.871887	2931	(-)	NNNNNCCATNTWNNNWN	CTTTGAAAATGACTTGC
M00059	V$YY1_01	0.817824	2970	(+)	NNNNNCCATNTWNNNWN	ACCCTTCATTTCTCAAA
M00059	V$YY1_01	0.795872	3021	(+)	NNNNNCCATNTWNNNWN	TCATATCATACAGTGAT
M00059	V$YY1_01	0.783748	3176	(+)	NNNNNCCATNTWNNNWN	TCAGTTCAGATTGGGCT
M00069	V$YY1_02	0.791503	3221	(+)	NNNCGGCCATCTTGNCTSNW	TGCCTGCCATGCTGGAGACG
M00059	V$YY1_01	0.780799	3224	(-)	NNNNNCCATNTWNNNWN	CTGCCATGCTGGAGACG
M00059	V$YY1_01	0.780472	3314	(+)	NNNNNCCATNTWNNNWN	GAATTCCATGGACTACA

M00069	V$YY1_02	0.779186	3329	(-)	NNNCGGCCATCTTGNCTSNW	CACAGTCCAGAGGGTT-GCAA
CSN2 *Ovis Aries*						
M00252	V$TATA_01	0.77899	60	(+)	STATAAAWRNNNNNN	AGATATAATTTCAGT
M00252	V$TATA_01	0.777214	62	(+)	STATAAAWRNNNNNN	ATATAATTTCAGTGT
M00216	V$TATA_C	0.790335	104	(+)	NCTATAAAAR	AGGTTAAAAT
M00216	V$TATA_C	0.784526	120	(+)	NCTATAAAAR	AGAATATAAT
M00252	V$TATA_01	0.787364	121	(+)	STATAAAWRNNNNNN	GAATATAATTGCTTA
M00252	V$TATA_01	0.780005	123	(+)	STATAAAWRNNNNNN	ATATAATTGCTTAAA
M00216	V$TATA_C	0.883549	130	(+)	NCTATAAAAR	TGCTTAAAAT
M00216	V$TATA_C	0.855558	142	(+)	NCTATAAAAR	ATCTTAAAAA
M00216	V$TATA_C	0.823607	143	(+)	NCTATAAAAR	TCTTAAAAAT
M00252	V$TATA_01	0.784319	143	(+)	STATAAAWRNNNNNN	TCTTAAAAATAAGGG
M00252	V$TATA_01	0.778736	145	(+)	STATAAAWRNNNNNN	TTAAAAATAAGGGTG
M00216	V$TATA_C	0.789015	158	(+)	NCTATAAAAR	TGTTTAAGAT
M00216	V$TATA_C	0.769475	193	(+)	NCTATAAAAR	ATGTTAAAAA
M00216	V$TATA_C	0.806443	194	(+)	NCTATAAAAR	TGTTAAAAAA
M00216	V$TATA_C	0.764457	195	(+)	NCTATAAAAR	GTTAAAAAAT
M00216	V$TATA_C	0.859519	212	(+)	NCTATAAAAR	ACTATAAAGA
M00252	V$TATA_01	0.827201	213	(+)	STATAAAWRNNNNNN	CTATAAAGATTTGAT
M00216	V$TATA_C	0.802218	223	(+)	NCTATAAAAR	TTGATAAAAA
M00216	V$TATA_C	0.779773	256	(+)	NCTATAAAAR	GGAATATAAT
M00252	V$TATA_01	0.822634	257	(+)	STATAAAWRNNNNNN	GAATATAATAAGATG
M00216	V$TATA_C	0.744653	258	(+)	NCTATAAAAR	AATATAATAA
M00252	V$TATA_01	0.805126	259	(+)	STATAAAWRNNNNNN	ATATAATAAGATGTA
M00216	V$TATA_C	0.814629	293	(+)	NCTATAAAAR	TCTTTAACAC
M00216	V$TATA_C	0.832057	320	(+)	NCTATAAAAR	TGTATTAAAA
M00216	V$TATA_C	0.809084	321	(+)	NCTATAAAAR	GTATTAAAAG
M00252	V$TATA_01	0.86171	321	(+)	STATAAAWRNNNNNN	GTATTAAAAGATCTT
M00216	V$TATA_C	0.838659	377	(+)	NCTATAAAAR	GCCATAAAGG
M00252	V$TATA_01	0.840396	378	(+)	STATAAAWRNNNNNN	CCATAAAGGCAAGCA
M00216	V$TATA_C	0.785582	443	(+)	NCTATAAAAR	CCAACAAAAA
M00252	V$TATA_01	0.85968	453	(+)	STATAAAWRNNNNNN	GTTTATAGAGCTAGC
M00252	V$TATA_01	0.776706	466	(+)	STATAAAWRNNNNNN	GCATATTTAGTCAAG

M00252	V$TATA_01	0.821365	481	(+)	STATAAAWRNNNNNN	AGATAAAGAGGGTTG
M00252	V$TATA_01	0.81629	499	(+)	STATAAAWRNNNNNN	GGATATATATGCTAT
M00252	V$TATA_01	0.844456	501	(+)	STATAAAWRNNNNNN	ATATATATGCTATTT
M00252	V$TATA_01	0.821365	521	(+)	STATAAAWRNNNNNN	GTATTTATAAAAGAA
M00216	V$TATA_C	0.783734	522	(+)	NCTATAAAAR	TATTTATAAA
M00252	V$TATA_01	0.848769	523	(+)	STATAAAWRNNNNNN	ATTTATAAAAGAAGA
M00216	V$TATA_C	0.927119	524	(+)	NCTATAAAAR	TTTATAAAAG
M00252	V$TATA_01	0.904339	525	(+)	STATAAAWRNNNNNN	TTATAAAAGAAGAGT
M00252	V$TATA_01	0.813245	538	(+)	STATAAAWRNNNNNN	GTATATTTATCAAAA
M00252	V$TATA_01	0.784572	540	(+)	STATAAAWRNNNNNN	ATATTTATCAAAATT
M00216	V$TATA_C	0.793768	543	(+)	NCTATAAAAR	TTTATCAAAA
M00216	V$TATA_C	0.773699	588	(+)	NCTATAAAAR	ATCTTATAAT
M00216	V$TATA_C	0.775812	590	(+)	NCTATAAAAR	CTTATAATAT
M00216	V$TATA_C	0.761025	598	(+)	NCTATAAAAR	ATTTCAAAAA
M00216	V$TATA_C	0.744653	606	(+)	NCTATAAAAR	AATATTAAAA
M00216	V$TATA_C	0.785054	607	(+)	NCTATAAAAR	ATATTAAAAT
M00252	V$TATA_01	0.846739	607	(+)	STATAAAWRNNNNNN	ATATTAAAATAGATA
M00252	V$TATA_01	0.780259	638	(+)	STATAAAWRNNNNNN	AAATTAAAGAGAAAG
M00216	V$TATA_C	0.820174	663	(+)	NCTATAAAAR	GGTAAAAAAA
M00252	V$TATA_01	0.780259	664	(+)	STATAAAWRNNNNNN	GTAAAAAAAAAATTC
M00216	V$TATA_C	0.778981	710	(+)	NCTATAAAAR	ACAATGAAAA
M00216	V$TATA_C	0.889094	769	(+)	NCTATAAAAR	GGCTTAAAAA
M00216	V$TATA_C	0.811724	770	(+)	NCTATAAAAR	GCTTAAAAAT
M00252	V$TATA_01	0.787871	770	(+)	STATAAAWRNNNNNN	GCTTAAAAATTGGTA
M00216	V$TATA_C	0.87774	781	(+)	NCTATAAAAR	GGTATATAAA
M00252	V$TATA_01	0.882771	782	(+)	STATAAAWRNNNNNN	GTATATAAAATGCTA
M00216	V$TATA_C	0.856351	783	(+)	NCTATAAAAR	TATATAAAAT
M00252	V$TATA_01	0.902309	784	(+)	STATAAAWRNNNNNN	ATATAAAATGCTAGT
M00216	V$TATA_C	0.886454	797	(+)	NCTATAAAAR	GTTATAAAAT
M00252	V$TATA_01	0.822634	798	(+)	STATAAAWRNNNNNN	TTATAAAATAAACAA
M00216	V$TATA_C	0.7568	816	(+)	NCTATAAAAR	GCAATAATAT
M00216	V$TATA_C	0.900977	877	(+)	NCTATAAAAR	ACAATAAAAA
M00252	V$TATA_01	0.776199	878	(+)	STATAAAWRNNNNNN	CAATAAAAATTTTTT

M00216	V$TATA_C	0.743597	889	(+)	NCTATAAAAR	TTTTTAGAAC
M00216	V$TATA_C	0.769475	897	(+)	NCTATAAAAR	ACTTTATAGA
M00252	V$TATA_01	0.803603	898	(+)	STATAAAWRNNNNNN	CTTTATAGACATCTT
M00252	V$TATA_01	0.800304	913	(+)	STATAAAWRNNNNNN	GAATAAAGTGAAACA
M00216	V$TATA_C	0.746501	953	(+)	NCTATAAAAR	AATATAGAAA
M00216	V$TATA_C	0.751782	955	(+)	NCTATAAAAR	TATAGAAAAA
M00216	V$TATA_C	0.799313	977	(+)	NCTATAAAAR	AGTTTGAAAA
M00252	V$TATA_01	0.788632	978	(+)	STATAAAWRNNNNNN	GTTTGAAAATCTGGG
M00216	V$TATA_C	0.749142	1045	(+)	NCTATAAAAR	AACTTATAAA
M00216	V$TATA_C	0.915236	1047	(+)	NCTATAAAAR	CTTATAAAAA
M00252	V$TATA_01	0.900025	1048	(+)	STATAAAWRNNNNNN	TTATAAAAATGCAAC
M00252	V$TATA_01	0.771124	1050	(+)	STATAAAWRNNNNNN	ATAAAAATGCAACAA
M00216	V$TATA_C	0.747821	1058	(+)	NCTATAAAAR	GCAACAAAAC
M00216	V$TATA_C	0.750198	1070	(+)	NCTATAAAAR	ACCATTTAAT
M00252	V$TATA_01	0.795991	1185	(+)	STATAAAWRNNNNNN	AGATAAGTACACAGT
M00252	V$TATA_01	0.785587	1201	(+)	STATAAAWRNNNNNN	CTATGTAGAGAAAAT
M00216	V$TATA_C	0.857407	1231	(+)	NCTATAAAAR	TCTCTAAAAT
M00216	V$TATA_C	0.759176	1316	(+)	NCTATAAAAR	CCAAGAAAAC
M00216	V$TATA_C	0.792712	1382	(+)	NCTATAAAAR	GAAATAAAAG
M00252	V$TATA_01	0.780513	1383	(+)	STATAAAWRNNNNNN	AAATAAAAGATATGA
M00252	V$TATA_01	0.78102	1392	(+)	STATAAAWRNNNNNN	ATATGAATAAGACAA
M00216	V$TATA_C	0.766306	1410	(+)	NCTATAAAAR	TTCATGAAAA
M00216	V$TATA_C	0.743068	1411	(+)	NCTATAAAAR	TCATGAAAAT
M00216	V$TATA_C	0.753103	1440	(+)	NCTATAAAAR	AAGATAAAAA
M00252	V$TATA_01	0.838366	1441	(+)	STATAAAWRNNNNNN	AGATAAAAATAAAGT
M00216	V$TATA_C	0.759704	1494	(+)	NCTATAAAAR	TCAAGAAAAC
M00216	V$TATA_C	0.896224	1575	(+)	NCTATAAAAR	GCAATAAAAA
M00252	V$TATA_01	0.801319	1576	(+)	STATAAAWRNNNNNN	CAATAAAAATAATAA
M00252	V$TATA_01	0.782035	1585	(+)	STATAAAWRNNNNNN	TAATAAATATATATT
M00252	V$TATA_01	0.771632	1591	(+)	STATAAAWRNNNNNN	ATATATATTTTTTAA
M00216	V$TATA_C	0.768154	1599	(+)	NCTATAAAAR	TTTTTAATAA
M00216	V$TATA_C	0.752839	1630	(+)	NCTATAAAAR	TCTGTAAATA
M00252	V$TATA_01	0.78508	1631	(+)	STATAAAWRNNNNNN	CTGTAAATATCTGTA

M00216	V$TATA_C	0.771059	1725	(+)	NCTATAAAAR	AGCAGAAAAC
M00216	V$TATA_C	0.745445	1806	(+)	NCTATAAAAR	GCCATATGAT
M00216	V$TATA_C	0.77766	1811	(+)	NCTATAAAAR	ATGATAAAAT
M00216	V$TATA_C	0.742012	1820	(+)	NCTATAAAAR	TGTTTATTAA
M00216	V$TATA_C	0.784526	1834	(+)	NCTATAAAAR	TTTCTAAAAT
M00216	V$TATA_C	0.749142	1964	(+)	NCTATAAAAR	TCCTTGTAAA
M00252	V$TATA_01	0.781528	1987	(+)	STATAAAWRNNNNNN	ACTTAAATAAGAAAA
M00216	V$TATA_C	0.740164	1988	(+)	NCTATAAAAR	CTTAAATAAG
M00252	V$TATA_01	0.804364	2132	(+)	STATAAAWRNNNNNN	AAATAAATAAGACTA
M00252	V$TATA_01	0.783811	2134	(+)	STATAAAWRNNNNNN	ATAAATAAGACTAGT
M00216	V$TATA_C	0.814629	2163	(+)	NCTATAAAAR	TGTAGAAAAT
M00252	V$TATA_01	0.813499	2164	(+)	STATAAAWRNNNNNN	GTAGAAAATCGCATT
M00216	V$TATA_C	0.770531	2185	(+)	NCTATAAAAR	ATCATAAATG
M00252	V$TATA_01	0.802081	2186	(+)	STATAAAWRNNNNNN	TCATAAATGTATAAA
M00216	V$TATA_C	0.832057	2193	(+)	NCTATAAAAR	TGTATAAATA
M00252	V$TATA_01	0.887084	2194	(+)	STATAAAWRNNNNNN	GTATAAATAATGCAC
M00216	V$TATA_C	0.744125	2231	(+)	NCTATAAAAR	GCTAAGAAAG
M00216	V$TATA_C	0.745973	2337	(+)	NCTATAAAAR	CCTAAAGAAA
M00216	V$TATA_C	0.744389	2396	(+)	NCTATAAAAR	AGAACAAAAA
M00252	V$TATA_01	0.779751	2418	(+)	STATAAAWRNNNNNN	GCACAAATACAATTG
M00216	V$TATA_C	0.802218	2446	(+)	NCTATAAAAR	TCTAGATAAA
M00252	V$TATA_01	0.779498	2447	(+)	STATAAAWRNNNNNN	CTAGATAAATATATC
M00252	V$TATA_01	0.791677	2449	(+)	STATAAAWRNNNNNN	AGATAAATATATCTT
M00216	V$TATA_C	0.752575	2564	(+)	NCTATAAAAR	ACAATAATAC
M00252	V$TATA_01	0.772139	2570	(+)	STATAAAWRNNNNNN	ATACATATGTTCATA
M00216	V$TATA_C	0.786639	2585	(+)	NCTATAAAAR	ATTATAATAA
M00252	V$TATA_01	0.788886	2586	(+)	STATAAAWRNNNNNN	TTATAATAAAAAGTG
M00216	V$TATA_C	0.828096	2588	(+)	NCTATAAAAR	ATAATAAAAA
M00252	V$TATA_01	0.791423	2589	(+)	STATAAAWRNNNNNN	TAATAAAAAGTGTTC
M00216	V$TATA_C	0.794296	2602	(+)	NCTATAAAAR	TCAGTAAAAA
M00216	V$TATA_C	0.743068	2609	(+)	NCTATAAAAR	AAATTAAAAT
M00216	V$TATA_C	0.9493	2635	(+)	NCTATAAAAR	GCCATAAAAA
M00252	V$TATA_01	0.849531	2636	(+)	STATAAAWRNNNNNN	CCATAAAAACTCTTC

M0025		0.80385				
2	V$TATA_01	7	2683	(+)	STATAAAWRNNNNNN	CTATTAATTTAAAAT
M0021		0.82360				
6	V$TATA_C	7	2688	(+)	NCTATAAAAR	AATTTAAAAT
M0025						
2	V$TATA_01	0.79726	2689	(+)	STATAAAWRNNNNNN	ATTTAAAATACTCTC
M0021		0.74570				
6	V$TATA_C	9	2698	(+)	NCTATAAAAR	ACTCTCAAAA
M0021		0.74016				
6	V$TATA_C	4	2699	(+)	NCTATAAAAR	CTCTCAAAAA
M0021		0.75283				
6	V$TATA_C	9	2700	(+)	NCTATAAAAR	TCTCAAAAAA
M0021		0.81410				
6	V$TATA_C	1	2710	(+)	NCTATAAAAR	CGTATAACAT
M0025		0.77670				
2	V$TATA_01	6	2785	(+)	STATAAAWRNNNNNN	ATATTTAATAATGAG
M0025						
2	V$TATA_01	0.79117	2875	(+)	STATAAAWRNNNNNN	ATTTTTATACAAAGA
M0021		0.79561				
6	V$TATA_C	7	2878	(+)	NCTATAAAAR	TTTATACAAA
M0025		0.77924				
2	V$TATA_01	4	2879	(+)	STATAAAWRNNNNNN	TTATACAAAGAAAAA
M0021		0.85027				
6	V$TATA_C	7	2904	(+)	NCTATAAAAR	ACTGTAAAAT
M0025		0.77036				
2	V$TATA_01	3	2905	(+)	STATAAAWRNNNNNN	CTGTAAAATAGTAAC
M0021		0.77607				
6	V$TATA_C	6	2971	(+)	NCTATAAAAR	CCTTTATAGA
M0025		0.81781				
2	V$TATA_01	3	2972	(+)	STATAAAWRNNNNNN	CTTTATAGACTGTGG
M0021		0.74782				
6	V$TATA_C	1	3048	(+)	NCTATAAAAR	GCAACAAAAC
M0025		0.82364				
2	V$TATA_01	9	3079	(+)	STATAAAWRNNNNNN	ACATTAATGGCAACC
M0021		0.76419				
6	V$TATA_C	3	3365	(+)	NCTATAAAAR	TCTCTAATAG
M0021		0.75019				
6	V$TATA_C	8	3520	(+)	NCTATAAAAR	TGTATATATT
M0025		0.85079				
2	V$TATA_01	9	3521	(+)	STATAAAWRNNNNNN	GTATATATTTTAAAG
M0021		0.77237				
6	V$TATA_C	9	3527	(+)	NCTATAAAAR	ATTTTAAAGG
M0021						
6	V$TATA_C	0.75099	3535	(+)	NCTATAAAAR	GGAATAAATG
M0025		0.84978				
2	V$TATA_01	4	3536	(+)	STATAAAWRNNNNNN	GAATAAATGTGTAGA
M0021		0.77713				
6	V$TATA_C	2	3552	(+)	NCTATAAAAR	GGTTTAACAT
M0025		0.77264				
2	V$TATA_01	7	3689	(+)	STATAAAWRNNNNNN	GCATTTATTGAGCAC
M0021		0.76947				
6	V$TATA_C	5	3758	(+)	NCTATAAAAR	ACTTTGTAAA
M0021		0.77185				
6	V$TATA_C	1	3818	(+)	NCTATAAAAR	AGTAAATAAG
M0025		0.79446				
2	V$TATA_01	8	3819	(+)	STATAAAWRNNNNNN	GTAAATAAGAGGCAT
M0025		0.77467				
2	V$TATA_01	6	3821	(+)	STATAAAWRNNNNNN	AAATAAGAGGCATTG
M0025		0.77822				
2	V$TATA_01	9	3921	(+)	STATAAAWRNNNNNN	GGTTAAATAGTGCAT
M0025		0.78330				
2	V$TATA_01	4	3962	(+)	STATAAAWRNNNNNN	ACATAAGATCCTATG
M0021		0.81594				
6	V$TATA_C	9	4113	(+)	NCTATAAAAR	TGCATGAAAG
M0025		0.77848				
2	V$TATA_01	3	4114	(+)	STATAAAWRNNNNNN	GCATGAAAGTGAAAA

M00216	V$TATA_C	0.760496	4259	(+)	NCTATAAAAR	TCCTTATAGG
M00216	V$TATA_C	0.780829	4280	(+)	NCTATAAAAR	TTCTTATAAT
M00216	V$TATA_C	0.773699	4290	(+)	NCTATAAAAR	TCATTAGAAG
M00216	V$TATA_C	0.809876	4337	(+)	NCTATAAAAR	GCCATGAAAT
M00216	V$TATA_C	0.771851	4343	(+)	NCTATAAAAR	AAATTAAAAG
M00252	V$TATA_01	0.787364	4343	(+)	STATAAAWRNNNNNN	AAATTAAAAGACGCT
M00252	V$TATA_01	0.77087	4344	(+)	STATAAAWRNNNNNN	AATTAAAAGACGCTT
M00216	V$TATA_C	0.75099	4366	(+)	NCTATAAAAR	GGAAGAAAAG
M00252	V$TATA_01	0.797006	4398	(+)	STATAAAWRNNNNNN	ATTCAAAAGCAGAGA
M00252	V$TATA_01	0.803603	4935	(+)	STATAAAWRNNNNNN	TTATATATTTTCAAT
M00216	V$TATA_C	0.743068	4960	(+)	NCTATAAAAR	CTTTTAGAAC
M00216	V$TATA_C	0.764193	5251	(+)	NCTATAAAAR	TCTGTTAAAG
M00216	V$TATA_C	0.76921	5315	(+)	NCTATAAAAR	TTTATACAAC
M00216	V$TATA_C	0.931872	5359	(+)	NCTATAAAAR	GCCATAAAAT
M00252	V$TATA_01	0.800304	5360	(+)	STATAAAWRNNNNNN	CCATAAAATAATGAT
M00216	V$TATA_C	0.741748	5375	(+)	NCTATAAAAR	ATCATCAAAT
M00216	V$TATA_C	0.774756	5391	(+)	NCTATAAAAR	TCCATATTAA
M00252	V$TATA_01	0.783304	5392	(+)	STATAAAWRNNNNNN	CCATATTAACCTATT
M00216	V$TATA_C	0.848693	5401	(+)	NCTATAAAAR	CCTATTAAAT
M00252	V$TATA_01	0.808424	5402	(+)	STATAAAWRNNNNNN	CTATTAAATGTCTTC
M00216	V$TATA_C	0.752046	5449	(+)	NCTATAAAAR	TTCTTTAAAG
M00216	V$TATA_C	0.814629	5450	(+)	NCTATAAAAR	TCTTTAAAGC
M00252	V$TATA_01	0.800558	5451	(+)	STATAAAWRNNNNNN	CTTTAAAGCTAAACC
M00252	V$TATA_01	0.854098	5526	(+)	STATAAAWRNNNNNN	ATATTTAAACGTACA
M00216	V$TATA_C	0.761025	5540	(+)	NCTATAAAAR	ATTTTTAAAA
M00216	V$TATA_C	0.890151	5541	(+)	NCTATAAAAR	TTTTTAAAAA
M00216	V$TATA_C	0.741748	5542	(+)	NCTATAAAAR	TTTTAAAAAC
M00252	V$TATA_01	0.811215	5542	(+)	STATAAAWRNNNNNN	TTTTAAAAACTTCGT
M00252	V$TATA_01	0.78508	5562	(+)	STATAAAWRNNNNNN	ATTTTTATAAACAGC
M00216	V$TATA_C	0.82572	5563	(+)	NCTATAAAAR	TTTTTATAAA
M00252	V$TATA_01	0.821873	5564	(+)	STATAAAWRNNNNNN	TTTTATAAACAGCAT
M00216	V$TATA_C	0.793768	5565	(+)	NCTATAAAAR	TTTATAAACA
M00252	V$TATA_01	0.791931	5566	(+)	STATAAAWRNNNNNN	TTATAAACAGCATTT

M00216	V$TATA_C	0.814629	5604	(+)	NCTATAAAAR	ACCAGAAAAT
M00216	V$TATA_C	0.810668	5619	(+)	NCTATAAAAR	AGTTTCAAAG
M00252	V$TATA_01	0.816798	5669	(+)	STATAAAWRNNNNNN	GTATATTTTGAGAAG
M00216	V$TATA_C	0.760232	5685	(+)	NCTATAAAAR	ATTATGAAAC
M00216	V$TATA_C	0.845788	5699	(+)	NCTATAAAAR	CACATAAAAA
M00216	V$TATA_C	0.764721	5700	(+)	NCTATAAAAR	ACATAAAAAG
M00252	V$TATA_01	0.828724	5700	(+)	STATAAAWRNNNNNN	ACATAAAAAGATTTA
M00216	V$TATA_C	0.754423	5709	(+)	NCTATAAAAR	GATTTATAAT
M00216	V$TATA_C	0.793768	5711	(+)	NCTATAAAAR	TTTATAATAA
M00216	V$TATA_C	0.758912	5741	(+)	NCTATAAAAR	GTAATATAAA
M00252	V$TATA_01	0.78508	5742	(+)	STATAAAWRNNNNNN	TAATATAAATGTCAG
M00216	V$TATA_C	0.756007	5743	(+)	NCTATAAAAR	AATATAAATG
M00252	V$TATA_01	0.902055	5744	(+)	STATAAAWRNNNNNN	ATATAAATGTCAGTG
M00216	V$TATA_C	0.772379	5790	(+)	NCTATAAAAR	ATTTTAAAGG
M00252	V$TATA_01	0.788632	5791	(+)	STATAAAWRNNNNNN	TTTTAAAGGTAGAAA
M00252	V$TATA_01	0.797767	5809	(+)	STATAAAWRNNNNNN	ATTTTTAAACACATA
M00216	V$TATA_C	0.768154	5810	(+)	NCTATAAAAR	TTTTTAAACA
M00216	V$TATA_C	0.809348	5836	(+)	NCTATAAAAR	AGTATAGAAT
M00216	V$TATA_C	0.744389	5841	(+)	NCTATAAAAR	AGAATAAATA
M00252	V$TATA_01	0.891652	5842	(+)	STATAAAWRNNNNNN	GAATAAATACCCAAG
M00216	V$TATA_C	0.756271	5897	(+)	NCTATAAAAR	GTTTTAATAA
M00252	V$TATA_01	0.779751	5897	(+)	STATAAAWRNNNNNN	GTTTTAATAAATGCA
M00252	V$TATA_01	0.805633	5901	(+)	STATAAAWRNNNNNN	TAATAAATGCATATT
M00216	V$TATA_C	0.751518	5908	(+)	NCTATAAAAR	TGCATATTAG
M00252	V$TATA_01	0.800558	5911	(+)	STATAAAWRNNNNNN	ATATTAGTAGGAACA
M00216	V$TATA_C	0.772115	6029	(+)	NCTATAAAAR	GCTGTATAAC
M00252	V$TATA_01	0.788886	6076	(+)	STATAAAWRNNNNNN	CTGTAAATTGAGAAT
M00252	V$TATA_01	0.784572	6093	(+)	STATAAAWRNNNNNN	TCATAATTACCCAGA
M00252	V$TATA_01	0.776706	6177	(+)	STATAAAWRNNNNNN	TTATGAATGTGTGGA
M00216	V$TATA_C	0.790071	6192	(+)	NCTATAAAAR	GTTCTAAAAA
M00216	V$TATA_C	0.752046	6193	(+)	NCTATAAAAR	TTCTAAAAAG
M00216	V$TATA_C	0.826776	6247	(+)	NCTATAAAAR	AGTATACAAA
M00252	V$TATA_01	0.808678	6248	(+)	STATAAAWRNNNNNN	GTATACAAATGTGTG

M00252	V$TATA_01	0.787364	6250	(+)	STATAAAWRNNNNNN	ATACAAATGTGTGAC
M00252	V$TATA_01	0.809947	6277	(+)	STATAAAWRNNNNNN	TTATTAATTAGCAGA
M00216	V$TATA_C	0.900977	6295	(+)	NCTATAAAAR	TGCTTAAAAA
M00216	V$TATA_C	0.811724	6296	(+)	NCTATAAAAR	GCTTAAAAAT
M00252	V$TATA_01	0.811215	6296	(+)	STATAAAWRNNNNNN	GCTTAAAAATAAGGA
M00252	V$TATA_01	0.775438	6298	(+)	STATAAAWRNNNNNN	TTAAAAATAAGGATT
M00216	V$TATA_C	0.902033	6320	(+)	NCTATAAAAR	AGCATAAAAT
M00252	V$TATA_01	0.814007	6321	(+)	STATAAAWRNNNNNN	GCATAAAATTCTTTC
M00252	V$TATA_01	0.770363	6358	(+)	STATAAAWRNNNNNN	ATATTTATCTAATTG
M00252	V$TATA_01	0.797767	6375	(+)	STATAAAWRNNNNNN	ATATTTAAGCTTTCC
M00216	V$TATA_C	0.740692	6391	(+)	NCTATAAAAR	GTTTTAGAAT
M00216	V$TATA_C	0.813045	6466	(+)	NCTATAAAAR	GCCTTTAAAG
M00216	V$TATA_C	0.840507	6467	(+)	NCTATAAAAR	CCTTTAAAGA
M00252	V$TATA_01	0.839888	6468	(+)	STATAAAWRNNNNNN	CTTTAAAGACTCAAG
M00216	V$TATA_C	0.751518	6542	(+)	NCTATAAAAR	TGAATTAAAA
M00216	V$TATA_C	0.755743	6543	(+)	NCTATAAAAR	GAATTAAAAA
M00252	V$TATA_01	0.77696	6544	(+)	STATAAAWRNNNNNN	AATTAAAAAAACACA
M00216	V$TATA_C	0.786639	6545	(+)	NCTATAAAAR	ATTAAAAAAA
M00252	V$TATA_01	0.794468	6546	(+)	STATAAAWRNNNNNN	TTAAAAAAACACATA
M00252	V$TATA_01	0.803349	6558	(+)	STATAAAWRNNNNNN	ATATTAAGACTTAGT
M00216	V$TATA_C	0.839187	6574	(+)	NCTATAAAAR	TCCATTAAAA
M00216	V$TATA_C	0.855558	6575	(+)	NCTATAAAAR	CCATTAAAAC
M00252	V$TATA_01	0.831007	6575	(+)	STATAAAWRNNNNNN	CCATTAAAACAAACA
M00216	V$TATA_C	0.780829	6608	(+)	NCTATAAAAR	ACTTTTTAAG
M00216	V$TATA_C	0.776604	6618	(+)	NCTATAAAAR	TCTTTTTAAA
M00216	V$TATA_C	0.767626	6619	(+)	NCTATAAAAR	CTTTTTAAAA
M00216	V$TATA_C	0.872722	6620	(+)	NCTATAAAAR	TTTTTAAAAT
M00252	V$TATA_01	0.777721	6620	(+)	STATAAAWRNNNNNN	TTTTTAAAATAGATC
M00252	V$TATA_01	0.774169	6621	(+)	STATAAAWRNNNNNN	TTTTAAAATAGATCT
M00252	V$TATA_01	0.800558	6643	(+)	STATAAAWRNNNNNN	TTATATGAAATCAGT
M00216	V$TATA_C	0.806443	6892	(+)	NCTATAAAAR	TGTTTTAAAA
M00216	V$TATA_C	0.878268	6893	(+)	NCTATAAAAR	GTTTTAAAAA
M00252	V$TATA_01	0.81629	6893	(+)	STATAAAWRNNNNNN	GTTTTAAAAAGAGGA

M00216	V$TATA_C	0.779509	6894	(+)	NCTATAAAAR	TTTTAAAAAG
M00252	V$TATA_01	0.854605	6894	(+)	STATAAAWRNNNNNN	TTTTAAAAAGAGGAG
M00216	V$TATA_C	0.759176	6917	(+)	NCTATAAAAR	ATCATAAATA
M00252	V$TATA_01	0.830246	6918	(+)	STATAAAWRNNNNNN	TCATAAATAACAATT
M00216	V$TATA_C	0.815949	6987	(+)	NCTATAAAAR	AGGATAAAAT
M00252	V$TATA_01	0.849277	6988	(+)	STATAAAWRNNNNNN	GGATAAAATCCACCC
M00216	V$TATA_C	0.812252	7060	(+)	NCTATAAAAR	CCCACAAAAC
M00216	V$TATA_C	0.817798	7237	(+)	NCTATAAAAR	TGTTGAAAAG
M00216	V$TATA_C	0.866121	7538	(+)	NCTATAAAAR	CCTACAAAAA
M00252	V$TATA_01	0.796245	7539	(+)	STATAAAWRNNNNNN	CTACAAAAAAATCAA
M00216	V$TATA_C	0.761553	7541	(+)	NCTATAAAAR	ACAAAAAAAT
M00216	V$TATA_C	0.781885	7606	(+)	NCTATAAAAR	GTTATAAATA
M00252	V$TATA_01	0.858412	7607	(+)	STATAAAWRNNNNNN	TTATAAATAGAATAA
M00252	V$TATA_01	0.79726	7704	(+)	STATAAAWRNNNNNN	CTATGTAGAAGAAGC
M00216	V$TATA_C	0.752575	7793	(+)	NCTATAAAAR	ACAATAAAGC
M00252	V$TATA_01	0.799543	7794	(+)	STATAAAWRNNNNNN	CAATAAAGCCAGAAT
M00216	V$TATA_C	0.752046	7812	(+)	NCTATAAAAR	ACTTGATAAT
M00216	V$TATA_C	0.744389	7834	(+)	NCTATAAAAR	AGAATAACAA
M00252	V$TATA_01	0.808424	7932	(+)	STATAAAWRNNNNNN	TGATATATGAGCATA
M00252	V$TATA_01	0.844202	7934	(+)	STATAAAWRNNNNNN	ATATATGAGCATATG
M00216	V$TATA_C	0.744389	7941	(+)	NCTATAAAAR	AGCATATGAG
M00252	V$TATA_01	0.829231	7942	(+)	STATAAAWRNNNNNN	GCATATGAGGGCAAA
M00252	V$TATA_01	0.791677	8170	(+)	STATAAAWRNNNNNN	GGATAAGACCCAATA
M00216	V$TATA_C	0.797465	8208	(+)	NCTATAAAAR	AGCATAAAGA
M00252	V$TATA_01	0.795737	8209	(+)	STATAAAWRNNNNNN	GCATAAAGATTAGTT
M00216	V$TATA_C	0.781885	8220	(+)	NCTATAAAAR	AGTTTTAAAT
M00252	V$TATA_01	0.772139	8301	(+)	STATAAAWRNNNNNN	CTGTATATATATGGT
M00216	V$TATA_C	0.767626	8302	(+)	NCTATAAAAR	TGTATATATA
M00252	V$TATA_01	0.905861	8303	(+)	STATAAAWRNNNNNN	GTATATATATGGTGT
M00252	V$TATA_01	0.873382	8305	(+)	STATAAAWRNNNNNN	ATATATATGGTGTTG
M00252	V$TATA_01	0.786095	8375	(+)	STATAAAWRNNNNNN	ATTTATAGAAAAAAC
M00216	V$TATA_C	0.795617	8376	(+)	NCTATAAAAR	TTTATAGAAA
M00216	V$TATA_C	0.751782	8378	(+)	NCTATAAAAR	TATAGAAAAA

M00252	V$TATA_01	0.784826	8411	(+)	STATAAAWRNNNNNN	TTATGTAAACCTGTT
M00216	V$TATA_C	0.761025	8433	(+)	NCTATAAAAR	ATTTTAAAGA
M00252	V$TATA_01	0.801827	8434	(+)	STATAAAWRNNNNNN	TTTTAAAGATCAACT
M00216	V$TATA_C	0.802746	8497	(+)	NCTATAAAAR	CACTTAAAAT
M00252	V$TATA_01	0.781781	8505	(+)	STATAAAWRNNNNNN	ATTTATATATTGTCT
M00252	V$TATA_01	0.804364	8507	(+)	STATAAAWRNNNNNN	TTATATATTGTCTTT
M00216	V$TATA_C	0.820174	8563	(+)	NCTATAAAAR	GACTTAAAAG
M00252	V$TATA_01	0.827455	8564	(+)	STATAAAWRNNNNNN	ACTTAAAAGGGGAAT
M00216	V$TATA_C	0.740692	8596	(+)	NCTATAAAAR	TTCTTAAAGA
M00216	V$TATA_C	0.761025	8639	(+)	NCTATAAAAR	ATTTTAATAA
M00216	V$TATA_C	0.80882	8642	(+)	NCTATAAAAR	TTAATAAAAC
M00216	V$TATA_C	0.799313	8687	(+)	NCTATAAAAR	TTTTTATAAC
M00216	V$TATA_C	0.823079	8696	(+)	NCTATAAAAR	CCTTTAAGAT
M00216	V$TATA_C	0.793768	8741	(+)	NCTATAAAAR	TTTATGAAAA
M00252	V$TATA_01	0.770617	8742	(+)	STATAAAWRNNNNNN	TTATGAAAAAGTCAC
M00216	V$TATA_C	0.764457	8787	(+)	NCTATAAAAR	GTTATAACAT
M00216	V$TATA_C	0.74835	8792	(+)	NCTATAAAAR	AACATATAAC
M00252	V$TATA_01	0.827201	8793	(+)	STATAAAWRNNNNNN	ACATATAACTGAATG
M00216	V$TATA_C	0.743597	8890	(+)	NCTATAAAAR	ATTTTTAAAT
M00216	V$TATA_C	0.779509	8891	(+)	NCTATAAAAR	TTTTTAAATG
M00252	V$TATA_01	0.813753	8892	(+)	STATAAAWRNNNNNN	TTTTAAATGAGTATA
M00252	V$TATA_01	0.797767	8902	(+)	STATAAAWRNNNNNN	GTATACATGGAACAA
M00216	V$TATA_C	0.750198	8982	(+)	NCTATAAAAR	ACCATATACT
M00216	V$TATA_C	0.833905	8989	(+)	NCTATAAAAR	ACTTTCAAAA
M00216	V$TATA_C	0.750198	8990	(+)	NCTATAAAAR	CTTTCAAAAT
M00216	V$TATA_C	0.930816	9014	(+)	NCTATAAAAR	AGCATAAAAG
M00252	V$TATA_01	0.870845	9015	(+)	STATAAAWRNNNNNN	GCATAAAAGTTCAGT
M00216	V$TATA_C	0.751254	9052	(+)	NCTATAAAAR	CATATCAAAA
M00252	V$TATA_01	0.776453	9061	(+)	STATAAAWRNNNNNN	ATATGTATAAAAATA
M00216	V$TATA_C	0.954053	9064	(+)	NCTATAAAAR	TGTATAAAAA
M00252	V$TATA_01	0.891144	9065	(+)	STATAAAWRNNNNNN	GTATAAAAATAATTT
M00216	V$TATA_C	0.841035	9100	(+)	NCTATAAAAR	TCTTTAAGAA
M00252	V$TATA_01	0.792946	9101	(+)	STATAAAWRNNNNNN	CTTTAAGAATCTATT

M0021		0.76868				
6	V$TATA_C	2	9131	(+)	NCTATAAAAR	TCAATAAATT
M0025		0.79421				
2	V$TATA_01	5	9132	(+)	STATAAAWRNNNNNN	CAATAAATTAACCCT
M0012		0.78466				
7	V$GATA1_03	4	43	(+)	RNSNNGATAANNGN	AAAAAGGTAAGAAT
M0007		0.84550				
5	V$GATA1_01	8	51	(-)	SNNGATNNNN	AAGAATCTCA
M0007		0.84934				
6	V$GATA2_01	6	51	(-)	NNNGATRNNN	AAGAATCTCA
M0007		0.83265				
6	V$GATA2_01	7	58	(+)	NNNGATRNNN	TCAGATATAA
M0007		0.84093				
7	V$GATA3_01	9	59	(+)	NNGATARNG	CAGATATAA
M0020		0.83379				
3	V$GATA_C	9	60	(+)	NGATAAGNMNN	AGATATAATTT
M0012		0.79906				
6	V$GATA1_02	3	71	(-)	NNNNNGATANKGNN	CAGTGTATCTGCTA
M0012		0.81678				
8	V$GATA1_04	9	71	(-)	NNCWGATARNNNN	CAGTGTATCTGCT
M0007		0.86327				
5	V$GATA1_01	7	73	(-)	SNNGATNNNN	GTGTATCTGC
M0007		0.88452				
6	V$GATA2_01	9	73	(-)	NNNGATRNNN	GTGTATCTGC
M0012		0.80769				
7	V$GATA1_03	2	82	(-)	RNSNNGATAANNGN	CTACTCATCTTTAT
M0007		0.80503				
5	V$GATA1_01	5	84	(-)	SNNGATNNNN	ACTCATCTTT
M0007		0.82498				
6	V$GATA2_01	9	84	(-)	NNNGATRNNN	ACTCATCTTT
M0007						
7	V$GATA3_01	0.83385	84	(-)	NNGATARNG	ACTCATCTT
M0012		0.83782				
7	V$GATA1_03	5	119	(+)	RNSNNGATAANNGN	AAGAATATAATTGC
M0007		0.78980				
6	V$GATA2_01	6	138	(-)	NNNGATRNNN	ATAGATCTTA
M0012						
6	V$GATA1_02	0.83875	145	(+)	NNNNNGATANKGNN	TTAAAAATAAGGGT
M0012		0.91406				
6	V$GATA1_02	2	160	(+)	NNNNNGATANKGNN	TTTAAGATAAGGTT
M0012		0.91911				
8	V$GATA1_04	8	161	(+)	NNCWGATARNNNN	TTAAGATAAGGTT
M0007		0.82477				
5	V$GATA1_01	8	162	(+)	SNNGATNNNN	TAAGATAAGG
M0007		0.89896				
6	V$GATA2_01	3	162	(+)	NNNGATRNNN	TAAGATAAGG
M0007		0.95436				
7	V$GATA3_01	4	163	(+)	NNGATARNG	AAGATAAGG
M0020		0.93942				
3	V$GATA_C	2	164	(+)	NGATAAGNMNN	AGATAAGGTTT
M0007		0.78664				
6	V$GATA2_01	9	189	(-)	NNNGATRNNN	AAATATGTTA
M0012		0.78368				
7	V$GATA1_03	4	209	(-)	RNSNNGATAANNGN	GTGACTATAAAGAT
M0007		0.85378				
7	V$GATA3_01	8	218	(+)	NNGATARNG	AAGATTTGA
M0012		0.78468				
6	V$GATA1_02	7	220	(+)	NNNNNGATANKGNN	GATTTGATAAAAAT
M0012		0.88878				
8	V$GATA1_04	7	221	(+)	NNCWGATARNNNN	ATTTGATAAAAAT
M0007		0.83074				
7	V$GATA3_01	9	223	(+)	NNGATARNG	TTGATAAAA
M0020						
3	V$GATA_C	0.93383	224	(+)	NGATAAGNMNN	TGATAAAAATT
M0012		0.85276				
7	V$GATA1_03	8	255	(+)	RNSNNGATAANNGN	AGGAATATAATAAG

M00075	V$GATA1_01	0.791214	265	(+)	SNNGATNNNN	TAAGATGTAG
M00076	V$GATA2_01	0.838972	265	(+)	NNNGATRNNN	TAAGATGTAG
M00077	V$GATA3_01	0.82809	266	(+)	NNGATARNG	AAGATGTAG
M00127	V$GATA1_03	0.810142	283	(-)	RNSNNGATAANNGN	GTTGCTATTTTCTT
M00126	V$GATA1_02	0.7775	286	(-)	NNNNNGATANKGNN	GCTATTTTCTTTAA
M00127	V$GATA1_03	0.784174	305	(+)	RNSNNGATAANNGN	GACTAGTTAACAAG
M00127	V$GATA1_03	0.807692	307	(-)	RNSNNGATAANNGN	CTAGTTAACAAGCT
M00076	V$GATA2_01	0.797474	327	(+)	NNNGATRNNN	AAAGATCTTT
M00076	V$GATA2_01	0.797474	327	(-)	NNNGATRNNN	AAAGATCTTT
M00076	V$GATA2_01	0.807397	404	(-)	NNNGATRNNN	TACTATGGGG
M00075	V$GATA1_01	0.852912	410	(+)	SNNGATNNNN	GGGGATTTGA
M00076	V$GATA2_01	0.843482	410	(+)	NNNGATRNNN	GGGGATTTGA
M00077	V$GATA3_01	0.825875	411	(+)	NNGATARNG	GGGATTTGA
M00075	V$GATA1_01	0.824284	432	(+)	SNNGATNNNN	GAAGATCTCT
M00075	V$GATA1_01	0.773445	432	(-)	SNNGATNNNN	GAAGATCTCT
M00076	V$GATA2_01	0.863329	432	(+)	NNNGATRNNN	GAAGATCTCT
M00076	V$GATA2_01	0.801985	432	(-)	NNNGATRNNN	GAAGATCTCT
M00076	V$GATA2_01	0.801083	464	(+)	NNNGATRNNN	TAGCATATTT
M00126	V$GATA1_02	0.836875	477	(+)	NNNNNGATANKGNN	CAAGAGATAAAGAG
M00127	V$GATA1_03	0.849339	477	(+)	RNSNNGATAANNGN	CAAGAGATAAAGAG
M00128	V$GATA1_04	0.932598	478	(+)	NNCWGATARNNNN	AAGAGATAAAGAG
M00075	V$GATA1_01	0.793189	479	(+)	SNNGATNNNN	AGAGATAAAG
M00076	V$GATA2_01	0.828146	479	(+)	NNNGATRNNN	AGAGATAAAG
M00077	V$GATA3_01	0.930882	480	(+)	NNGATARNG	GAGATAAAG
M00203	V$GATA_C	0.864244	481	(+)	NGATAAGNMNN	AGATAAAGAGG
M00075	V$GATA1_01	0.77542	497	(+)	SNNGATNNNN	TAGGATATAT
M00076	V$GATA2_01	0.868742	497	(+)	NNNGATRNNN	TAGGATATAT
M00203	V$GATA_C	0.929481	539	(-)	NGATAAGNMNN	TATATTTATCA
M00127	V$GATA1_03	0.78001	540	(-)	RNSNNGATAANNGN	ATATTTATCAAAAT
M00128	V$GATA1_04	0.87163	540	(-)	NNCWGATARNNNN	ATATTTATCAAAA
M00076	V$GATA2_01	0.795219	560	(-)	NNNGATRNNN	GAACATCCAA
M00203	V$GATA_C	0.848089	574	(-)	NGATAAGNMNN	CAAGTTTATCA
M00126	V$GATA1_02	0.833125	575	(-)	NNNNNGATANKGNN	AAGTTTATCATTTA

M00128	V$GATA1_04	0.863664	575	(-)	NNCWGATARNNNN	AAGTTTATCATTT
M00076	V$GATA2_01	0.792061	577	(-)	NNNGATRNNN	GTTTATCATT
M00077	V$GATA3_01	0.823217	577	(-)	NNGATARNG	GTTTATCAT
M00203	V$GATA_C	0.922336	581	(-)	NGATAAGNMNN	ATCATTTATCT
M00126	V$GATA1_02	0.825625	582	(-)	NNNNNGATANKGNN	TCATTTATCTTATA
M00128	V$GATA1_04	0.918505	582	(-)	NNCWGATARNNNN	TCATTTATCTTAT
M00076	V$GATA2_01	0.839423	584	(-)	NNNGATRNNN	ATTTATCTTA
M00077	V$GATA3_01	0.846699	584	(-)	NNGATARNG	ATTTATCTT
M00126	V$GATA1_02	0.834063	613	(+)	NNNNNGATANKGNN	AAATAGATACATGA
M00127	V$GATA1_03	0.812347	613	(+)	RNSNNGATAANNGN	AAATAGATACATGA
M00203	V$GATA_C	0.855856	617	(+)	NGATAAGNMNN	AGATACATGAA
M00127	V$GATA1_03	0.794953	818	(-)	RNSNNGATAANNGN	AATAATATCCTCCC
M00075	V$GATA1_01	0.835637	820	(-)	SNNGATNNNN	TAATATCCTC
M00076	V$GATA2_01	0.89355	820	(-)	NNNGATRNNN	TAATATCCTC
M00077	V$GATA3_01	0.821444	820	(-)	NNGATARNG	TAATATCCT
M00127	V$GATA1_03	0.792014	850	(-)	RNSNNGATAANNGN	GGTATTATGCTCTT
M00075	V$GATA1_01	0.880059	904	(-)	SNNGATNNNN	AGACATCTTG
M00076	V$GATA2_01	0.894001	904	(-)	NNNGATRNNN	AGACATCTTG
M00075	V$GATA1_01	0.86229	982	(-)	SNNGATNNNN	GAAAATCTGG
M00076	V$GATA2_01	0.843482	982	(-)	NNNGATRNNN	GAAAATCTGG
M00075	V$GATA1_01	0.791708	989	(+)	SNNGATNNNN	TGGGATTGAA
M00076	V$GATA2_01	0.819125	989	(+)	NNNGATRNNN	TGGGATTGAA
M00077	V$GATA3_01	0.84537	990	(+)	NNGATARNG	GGGATTGAA
M00075	V$GATA1_01	0.887957	996	(+)	SNNGATNNNN	GAAGATGTGT
M00076	V$GATA2_01	0.886784	996	(+)	NNNGATRNNN	GAAGATGTGT
M00075	V$GATA1_01	0.850938	1010	(+)	SNNGATNNNN	AGAGATGTTG
M00076	V$GATA2_01	0.854759	1010	(+)	NNNGATRNNN	AGAGATGTTG
M00077	V$GATA3_01	0.865308	1011	(+)	NNGATARNG	GAGATGTTG
M00075	V$GATA1_01	0.807009	1013	(+)	SNNGATNNNN	GATGTTGGTG
M00075	V$GATA1_01	0.783317	1090	(-)	SNNGATNNNN	CAAAATCAAT
M00077	V$GATA3_01	0.820115	1090	(-)	NNGATARNG	CAAAATCAA
M00075	V$GATA1_01	0.773939	1142	(+)	SNNGATNNNN	GGGGACTGGG
M00126	V$GATA1_02	0.836875	1181	(+)	NNNNNGATANKGNN	CAAGAGATAAGTAC

M00127	V$GATA1_03	0.871877	1181	(+)	RNSNNGATAANNGN	CAAGAGATAAGTAC
M00128	V$GATA1_04	0.936275	1182	(+)	NNCWGATARNNNN	AAGAGATAAGTAC
M00075	V$GATA1_01	0.833662	1183	(+)	SNNGATNNNN	AGAGATAAGT
M00076	V$GATA2_01	0.861525	1183	(+)	NNNGATRNNN	AGAGATAAGT
M00077	V$GATA3_01	0.904741	1184	(+)	NNGATARNG	GAGATAAGT
M00203	V$GATA_C	0.983846	1185	(+)	NGATAAGNMNN	AGATAAGTACA
M00127	V$GATA1_03	0.793484	1205	(+)	RNSNNGATAANNGN	GTAGAGAAAATAAG
M00127	V$GATA1_03	0.807692	1208	(+)	RNSNNGATAANNGN	GAGAAAATAAGCAT
M00076	V$GATA2_01	0.818223	1216	(+)	NNNGATRNNN	AAGCATAGTG
M00075	V$GATA1_01	0.788746	1226	(+)	SNNGATNNNN	TATGATCTCT
M00076	V$GATA2_01	0.837618	1226	(+)	NNNGATRNNN	TATGATCTCT
M00075	V$GATA1_01	0.785785	1256	(+)	SNNGATNNNN	AGAGATGACA
M00076	V$GATA2_01	0.815968	1256	(+)	NNNGATRNNN	AGAGATGACA
M00077	V$GATA3_01	0.846256	1257	(+)	NNGATARNG	GAGATGACA
M00076	V$GATA2_01	0.805593	1268	(-)	NNNGATRNNN	AGGCATGTGG
M00127	V$GATA1_03	0.791524	1274	(+)	RNSNNGATAANNGN	GTGGGGATGAAGAC
M00075	V$GATA1_01	0.853406	1276	(+)	SNNGATNNNN	GGGGATGAAG
M00076	V$GATA2_01	0.861074	1276	(+)	NNNGATRNNN	GGGGATGAAG
M00077	V$GATA3_01	0.832078	1277	(+)	NNGATARNG	GGGATGAAG
M00127	V$GATA1_03	0.784664	1289	(+)	RNSNNGATAANNGN	GAGTAGAGAAGAAA
M00075	V$GATA1_01	0.806515	1301	(-)	SNNGATNNNN	AACAATCTAA
M00076	V$GATA2_01	0.784844	1301	(-)	NNNGATRNNN	AACAATCTAA
M00077	V$GATA3_01	0.836952	1301	(-)	NNGATARNG	AACAATCTA
M00075	V$GATA1_01	0.787266	1306	(-)	SNNGATNNNN	TCTAATCAGT
M00077	V$GATA3_01	0.856447	1306	(-)	NNGATARNG	TCTAATCAG
M00075	V$GATA1_01	0.908687	1322	(-)	SNNGATNNNN	AAACATCTCG
M00076	V$GATA2_01	0.883175	1322	(-)	NNNGATRNNN	AAACATCTCG
M00075	V$GATA1_01	0.801579	1328	(+)	SNNGATNNNN	CTCGATCAGT
M00075	V$GATA1_01	0.819842	1328	(-)	SNNGATNNNN	CTCGATCAGT
M00076	V$GATA2_01	0.803789	1328	(-)	NNNGATRNNN	CTCGATCAGT
M00127	V$GATA1_03	0.789319	1379	(+)	RNSNNGATAANNGN	CTGGAAATAAAAGA
M00126	V$GATA1_02	0.83875	1386	(+)	NNNNNGATANKGNN	TAAAAGATATGAAT
M00128	V$GATA1_04	0.8894	1387	(+)	NNCWGATARNNNN	AAAAGATATGAAT

M00075	V$GATA1_01	0.787759	1388	(+)	SNNGATNNNN	AAAGATATGA
M00076	V$GATA2_01	0.838521	1388	(+)	NNNGATRNNN	AAAGATATGA
M00077	V$GATA3_01	0.887461	1389	(+)	NNGATARNG	AAGATATGA
M00203	V$GATA_C	0.893135	1390	(+)	NGATAAGNMNN	AGATATGAATA
M00203	V$GATA_C	0.858962	1401	(+)	NGATAAGNMNN	AGACAAAAATT
M00075	V$GATA1_01	0.821816	1415	(-)	SNNGATNNNN	GAAAATCACT
M00076	V$GATA2_01	0.790708	1415	(-)	NNNGATRNNN	GAAAATCACT
M00126	V$GATA1_02	0.785	1432	(+)	NNNNNGATANKGNN	GTAGAGAAAAGATA
M00126	V$GATA1_02	0.84375	1437	(+)	NNNNNGATANKGNN	GAAAAGATAAAAAT
M00127	V$GATA1_03	0.849339	1437	(+)	RNSNNGATAANNGN	GAAAAGATAAAAAT
M00128	V$GATA1_04	0.921569	1438	(+)	NNCWGATARNNNNN	AAAAGATAAAAAT
M00077	V$GATA3_01	0.8786	1440	(+)	NNGATARNG	AAGATAAAA
M00203	V$GATA_C	0.939112	1441	(+)	NGATAAGNMNN	AGATAAAAATA
M00076	V$GATA2_01	0.809202	1452	(-)	NNNGATRNNN	AAGTATGACC
M00075	V$GATA1_01	0.807009	1483	(+)	SNNGATNNNN	TCAGATGCAC
M00076	V$GATA2_01	0.841678	1483	(+)	NNNGATRNNN	TCAGATGCAC
M00127	V$GATA1_03	0.795198	1493	(+)	RNSNNGATAANNGN	CTCAAGAAAACTGA
M00075	V$GATA1_01	0.778381	1547	(-)	SNNGATNNNN	TCTAATCCCA
M00076	V$GATA2_01	0.814163	1547	(-)	NNNGATRNNN	TCTAATCCCA
M00077	V$GATA3_01	0.857776	1547	(-)	NNGATARNG	TCTAATCCC
M00075	V$GATA1_01	0.792201	1555	(-)	SNNGATNNNN	CAGAATCTAA
M00077	V$GATA3_01	0.824546	1555	(-)	NNGATARNG	CAGAATCTA
M00203	V$GATA_C	0.837838	1563	(-)	NGATAAGNMNN	AAGCGATATCT
M00075	V$GATA1_01	0.854393	1564	(+)	SNNGATNNNN	AGCGATATCT
M00076	V$GATA2_01	0.885882	1564	(+)	NNNGATRNNN	AGCGATATCT
M00126	V$GATA1_02	0.772188	1564	(-)	NNNNNGATANKGNN	AGCGATATCTGGCA
M00128	V$GATA1_04	0.882966	1564	(-)	NNCWGATARNNNNN	AGCGATATCTGGC
M00075	V$GATA1_01	0.912142	1566	(-)	SNNGATNNNN	CGATATCTGG
M00076	V$GATA2_01	0.943618	1566	(-)	NNNGATRNNN	CGATATCTGG
M00077	V$GATA3_01	0.879929	1566	(-)	NNGATARNG	CGATATCTG
M00127	V$GATA1_03	0.793974	1578	(+)	RNSNNGATAANNGN	ATAAAAAATAATAAA
M00075	V$GATA1_01	0.803554	1609	(-)	SNNGATNNNN	GTGAATCAAC
M00203	V$GATA_C	0.838459	1632	(-)	NGATAAGNMNN	TGTAAATATCT

M00126	V$GATA1_02	0.795625	1633	(-)	NNNNNGATANKGNN	GTAAATATCTGTAA
M00128	V$GATA1_04	0.853248	1633	(-)	NNCWGATARNNNNN	GTAAATATCTGTA
M00075	V$GATA1_01	0.831688	1635	(-)	SNNGATNNNN	AAATATCTGT
M00076	V$GATA2_01	0.870095	1635	(-)	NNNGATRNNN	AAATATCTGT
M00077	V$GATA3_01	0.828977	1635	(-)	NNGATARNG	AAATATCTG
M00076	V$GATA2_01	0.789355	1689	(+)	NNNGATRNNN	TGAGATGAAA
M00077	V$GATA3_01	0.842268	1690	(+)	NNGATARNG	GAGATGAAA
M00075	V$GATA1_01	0.786772	1709	(+)	SNNGATNNNN	TAGGATATAG
M00076	V$GATA2_01	0.881822	1709	(+)	NNNGATRNNN	TAGGATATAG
M00077	V$GATA3_01	0.836509	1710	(+)	NNGATARNG	AGGATATAG
M00203	V$GATA_C	0.841255	1711	(+)	NGATAAGNMNN	GGATATAGGAT
M00075	V$GATA1_01	0.779368	1716	(+)	SNNGATNNNN	TAGGATGTTA
M00076	V$GATA2_01	0.850699	1716	(+)	NNNGATRNNN	TAGGATGTTA
M00127	V$GATA1_03	0.830475	1717	(+)	RNSNNGATAANNGN	AGGATGTTAGCAGA
M00075	V$GATA1_01	0.840079	1731	(-)	SNNGATNNNN	AAACATCTGA
M00076	V$GATA2_01	0.874154	1731	(-)	NNNGATRNNN	AAACATCTGA
M00126	V$GATA1_02	0.778125	1755	(-)	NNNNNGATANKGNN	AACATTAACTTGAA
M00076	V$GATA2_01	0.782589	1805	(+)	NNNGATRNNN	GGCCATATGA
M00076	V$GATA2_01	0.794768	1807	(-)	NNNGATRNNN	CCATATGATA
M00128	V$GATA1_04	0.89185	1809	(+)	NNCWGATARNNNNN	ATATGATAAAATG
M00076	V$GATA2_01	0.798376	1810	(+)	NNNGATRNNN	TATGATAAAA
M00077	V$GATA3_01	0.855117	1811	(+)	NNGATARNG	ATGATAAAA
M00203	V$GATA_C	0.844983	1812	(+)	NGATAAGNMNN	TGATAAAATGT
M00076	V$GATA2_01	0.789806	1861	(-)	NNNGATRNNN	TGCCATGAGG
M00075	V$GATA1_01	0.835637	1875	(+)	SNNGATNNNN	CAGGATCTTG
M00075	V$GATA1_01	0.861303	1875	(-)	SNNGATNNNN	CAGGATCTTG
M00076	V$GATA2_01	0.866486	1875	(+)	NNNGATRNNN	CAGGATCTTG
M00076	V$GATA2_01	0.863329	1875	(-)	NNNGATRNNN	CAGGATCTTG
M00075	V$GATA1_01	0.773939	1896	(-)	SNNGATNNNN	AGGGATCAAA
M00126	V$GATA1_02	0.9075	1949	(-)	NNNNNGATANKGNN	TACACTATCTTTGG
M00127	V$GATA1_03	0.809652	1949	(-)	RNSNNGATAANNGN	TACACTATCTTTGG
M00075	V$GATA1_01	0.8692	1951	(-)	SNNGATNNNN	CACTATCTTT
M00076	V$GATA2_01	0.911592	1951	(-)	NNNGATRNNN	CACTATCTTT

M00077	V$GATA3_01	0.961896	1951	(-)	NNGATARNG	CACTATCTT
M00077	V$GATA3_01	0.883917	2002	(+)	NNGATARNG	TAGATTGAG
M00203	V$GATA_C	0.831625	2024	(-)	NGATAAGNMNN	TTTCCTCATCT
M00127	V$GATA1_03	0.826801	2025	(-)	RNSNNGATAANNGN	TTCCTCATCTTCCC
M00075	V$GATA1_01	0.881046	2027	(-)	SNNGATNNNN	CCTCATCTTC
M00076	V$GATA2_01	0.88498	2027	(-)	NNNGATRNNN	CCTCATCTTC
M00077	V$GATA3_01	0.900753	2027	(-)	NNGATARNG	CCTCATCTT
M00075	V$GATA1_01	0.856367	2045	(-)	SNNGATNNNN	AGGAATCGAG
M00076	V$GATA2_01	0.784393	2045	(-)	NNNGATRNNN	AGGAATCGAG
M00075	V$GATA1_01	0.811945	2067	(-)	SNNGATNNNN	CCTCATCCTT
M00076	V$GATA2_01	0.852503	2067	(-)	NNNGATRNNN	CCTCATCCTT
M00077	V$GATA3_01	0.85556	2067	(-)	NNGATARNG	CCTCATCCT
M00075	V$GATA1_01	0.772458	2077	(-)	SNNGATNNNN	GCCTGTCTGG
M00076	V$GATA2_01	0.791159	2077	(-)	NNNGATRNNN	GCCTGTCTGG
M00127	V$GATA1_03	0.785644	2104	(+)	RNSNNGATAANNGN	AGAGAGGTAAATAC
M00127	V$GATA1_03	0.788094	2146	(-)	RNSNNGATAANNGN	AGTACTGTCTGCCT
M00076	V$GATA2_01	0.801534	2156	(-)	NNNGATRNNN	GCCTATGTGT
M00127	V$GATA1_03	0.837335	2162	(+)	RNSNNGATAANNGN	GTGTAGAAAATCGC
M00075	V$GATA1_01	0.824284	2167	(-)	SNNGATNNNN	GAAAATCGCA
M00076	V$GATA2_01	0.831304	2167	(-)	NNNGATRNNN	GAAAATCGCA
M00127	V$GATA1_03	0.792014	2173	(-)	RNSNNGATAANNGN	CGCATTACCAACAT
M00075	V$GATA1_01	0.851431	2181	(-)	SNNGATNNNN	CAACATCATA
M00076	V$GATA2_01	0.867839	2181	(-)	NNNGATRNNN	CAACATCATA
M00077	V$GATA3_01	0.824546	2181	(-)	NNGATARNG	CAACATCAT
M00126	V$GATA1_02	0.797188	2194	(+)	NNNNNGATANKGNN	GTATAAATAATGCA
M00075	V$GATA1_01	0.882527	2206	(-)	SNNGATNNNN	CACAATCTCA
M00076	V$GATA2_01	0.887686	2206	(-)	NNNGATRNNN	CACAATCTCA
M00077	V$GATA3_01	0.945503	2206	(-)	NNGATARNG	CACAATCTC
M00075	V$GATA1_01	0.7769	2213	(+)	SNNGATNNNN	TCAGATTTAT
M00076	V$GATA2_01	0.797925	2213	(+)	NNNGATRNNN	TCAGATTTAT
M00127	V$GATA1_03	0.812837	2241	(-)	RNSNNGATAANNGN	TCATTTATGTTCAT
M00203	V$GATA_C	0.838459	2253	(-)	NGATAAGNMNN	ATCCACTATCT
M00126	V$GATA1_02	0.940313	2254	(-)	NNNNNGATANKGNN	TCCACTATCTCAAC

M00127	V$GATA1_03	0.820431	2254	(-)	RNSNNGATAANNGN	TCCACTATCTCAAC
M00128	V$GATA1_04	0.879902	2254	(-)	NNCWGATARNNNN	TCCACTATCTCAA
M00075	V$GATA1_01	0.906219	2256	(-)	SNNGATNNNN	CACTATCTCA
M00076	V$GATA2_01	0.952188	2256	(-)	NNNGATRNNN	CACTATCTCA
M00077	V$GATA3_01	0.979176	2256	(-)	NNGATARNG	CACTATCTC
M00126	V$GATA1_02	0.805312	2265	(-)	NNNNNGATANKGNN	AACAGTATCCTATA
M00075	V$GATA1_01	0.847483	2267	(-)	SNNGATNNNN	CAGTATCCTA
M00076	V$GATA2_01	0.933243	2267	(-)	NNNGATRNNN	CAGTATCCTA
M00077	V$GATA3_01	0.837395	2267	(-)	NNGATARNG	CAGTATCCT
M00126	V$GATA1_02	0.772188	2315	(-)	NNNNNGATANKGNN	TGTACCATCTGTAC
M00075	V$GATA1_01	0.84304	2317	(-)	SNNGATNNNN	TACCATCTGT
M00076	V$GATA2_01	0.848444	2317	(-)	NNNGATRNNN	TACCATCTGT
M00077	V$GATA3_01	0.867523	2317	(-)	NNGATARNG	TACCATCTG
M00127	V$GATA1_03	0.800833	2387	(+)	RNSNNGATAANNGN	ACACAGACAAGAAC
M00203	V$GATA_C	0.896241	2391	(+)	NGATAAGNMNN	AGACAAGAACA
M00075	V$GATA1_01	0.831688	2441	(-)	SNNGATNNNN	TACAATCTAG
M00077	V$GATA3_01	0.868852	2441	(-)	NNGATARNG	TACAATCTA
M00126	V$GATA1_02	0.798125	2445	(+)	NNNNNGATANKGNN	ATCTAGATAAATAT
M00127	V$GATA1_03	0.872856	2445	(+)	RNSNNGATAANNGN	ATCTAGATAAATAT
M00128	V$GATA1_04	0.870711	2446	(+)	NNCWGATARNNNN	TCTAGATAAATAT
M00075	V$GATA1_01	0.786278	2447	(+)	SNNGATNNNN	CTAGATAAAT
M00077	V$GATA3_01	0.822331	2448	(+)	NNGATARNG	TAGATAAAT
M00203	V$GATA_C	0.940665	2449	(+)	NGATAAGNMNN	AGATAAATATA
M00128	V$GATA1_04	0.834865	2453	(-)	NNCWGATARNNNN	AAATATATCTTAT
M00075	V$GATA1_01	0.779862	2455	(-)	SNNGATNNNN	ATATATCTTA
M00076	V$GATA2_01	0.854308	2455	(-)	NNNGATRNNN	ATATATCTTA
M00203	V$GATA_C	0.921404	2457	(-)	NGATAAGNMNN	ATATCTTATCC
M00126	V$GATA1_02	0.778125	2458	(-)	NNNNNGATANKGNN	TATCTTATCCCAGT
M00127	V$GATA1_03	0.796178	2458	(-)	RNSNNGATAANNGN	TATCTTATCCCAGT
M00075	V$GATA1_01	0.802073	2460	(-)	SNNGATNNNN	TCTTATCCCA
M00076	V$GATA2_01	0.878665	2460	(-)	NNNGATRNNN	TCTTATCCCA
M00077	V$GATA3_01	0.891449	2460	(-)	NNGATARNG	TCTTATCCC
M00203	V$GATA_C	0.878534	2479	(-)	NGATAAGNMNN	TATTTGTATCT

M00126	V$GATA1_02	0.826875	2480	(-)	NNNNNGATANKGNN	ATTTGTATCTCTTA
M00128	V$GATA1_04	0.826287	2480	(-)	NNCWGATARNNNNN	ATTTGTATCTCTT
M00075	V$GATA1_01	0.785785	2482	(-)	SNNGATNNNN	TTGTATCTCT
M00076	V$GATA2_01	0.811457	2482	(-)	NNNGATRNNN	TTGTATCTCT
M00077	V$GATA3_01	0.844927	2482	(-)	NNGATARNG	TTGTATCTC
M00075	V$GATA1_01	0.811451	2500	(+)	SNNGATNNNN	GGTGCTAGGT
M00075	V$GATA1_01	0.821816	2530	(+)	SNNGATNNNN	CAGGATGATA
M00076	V$GATA2_01	0.844835	2530	(+)	NNNGATRNNN	CAGGATGATA
M00126	V$GATA1_02	0.785	2531	(+)	NNNNNGATANKGNN	AGGATGATACTCCA
M00127	V$GATA1_03	0.873346	2531	(+)	RNSNNGATAANNGN	AGGATGATACTCCA
M00075	V$GATA1_01	0.902764	2533	(+)	SNNGATNNNN	GATGATACTC
M00076	V$GATA2_01	0.905277	2533	(+)	NNNGATRNNN	GATGATACTC
M00075	V$GATA1_01	0.817374	2545	(+)	SNNGATNNNN	GAGGATGACA
M00076	V$GATA2_01	0.869193	2545	(+)	NNNGATRNNN	GAGGATGACA
M00203	V$GATA_C	0.897173	2549	(-)	NGATAAGNMNN	ATGACATATCA
M00076	V$GATA2_01	0.783942	2550	(+)	NNNGATRNNN	TGACATATCA
M00128	V$GATA1_04	0.887255	2550	(-)	NNCWGATARNNNNN	TGACATATCAGAC
M00075	V$GATA1_01	0.873149	2552	(-)	SNNGATNNNN	ACATATCAGA
M00076	V$GATA2_01	0.897609	2552	(-)	NNNGATRNNN	ACATATCAGA
M00077	V$GATA3_01	0.826318	2552	(-)	NNGATARNG	ACATATCAG
M00076	V$GATA2_01	0.807397	2572	(-)	NNNGATRNNN	ACATATGTTC
M00127	V$GATA1_03	0.791524	2576	(+)	RNSNNGATAANNGN	ATGTTCATAATTAT
M00127	V$GATA1_03	0.784174	2582	(+)	RNSNNGATAANNGN	ATAATTATAATAAA
M00128	V$GATA1_04	0.844669	2621	(-)	NNCWGATARNNNNN	CTCCTTTTCTGTT
M00076	V$GATA2_01	0.783491	2628	(+)	NNNGATRNNN	TCTGTTAGCC
M00075	V$GATA1_01	0.828727	2729	(+)	SNNGATNNNN	TCAGATTTGT
M00076	V$GATA2_01	0.844384	2729	(+)	NNNGATRNNN	TCAGATTTGT
M00126	V$GATA1_02	0.811875	2740	(+)	NNNNNGATANKGNN	AAATAGATAGCTCT
M00127	V$GATA1_03	0.823861	2740	(+)	RNSNNGATAANNGN	AAATAGATAGCTCT
M00128	V$GATA1_04	0.850797	2741	(+)	NNCWGATARNNNNN	AATAGATAGCTCT
M00075	V$GATA1_01	0.821816	2742	(+)	SNNGATNNNN	ATAGATAGCT
M00076	V$GATA2_01	0.835814	2742	(+)	NNNGATRNNN	ATAGATAGCT
M00077	V$GATA3_01	0.854674	2743	(+)	NNGATARNG	TAGATAGCT

M00075	V$GATA1_01	0.819348	2869	(+)	SNNGATNNNN	TAAGATATTT
M00076	V$GATA2_01	0.894452	2869	(+)	NNNGATRNNN	TAAGATATTT
M00077	V$GATA3_01	0.834736	2870	(+)	NNGATARNG	AAGATATTT
M00075	V$GATA1_01	0.842547	2924	(+)	SNNGATNNNN	TCTGATGATT
M00076	V$GATA2_01	0.847993	2924	(+)	NNNGATRNNN	TCTGATGATT
M00075	V$GATA1_01	0.844521	2927	(+)	SNNGATNNNN	GATGATTTAG
M00076	V$GATA2_01	0.806044	2927	(+)	NNNGATRNNN	GATGATTTAG
M00077	V$GATA3_01	0.824546	2928	(+)	NNGATARNG	ATGATTTAG
M00126	V$GATA1_02	0.859063	3006	(+)	NNNNNGATANKGNN	GGTAAGATAATGTT
M00127	V$GATA1_03	0.808427	3006	(+)	RNSNNGATAANNGN	GGTAAGATAATGTT
M00128	V$GATA1_04	0.875919	3007	(+)	NNCWGATARNNNNN	GTAAGATAATGTT
M00075	V$GATA1_01	0.812438	3008	(+)	SNNGATNNNN	TAAGATAATG
M00076	V$GATA2_01	0.892648	3008	(+)	NNNGATRNNN	TAAGATAATG
M00077	V$GATA3_01	0.93354	3009	(+)	NNGATARNG	AAGATAATG
M00203	V$GATA_C	0.855545	3010	(+)	NGATAAGNMNN	AGATAATGTTC
M00075	V$GATA1_01	0.848963	3037	(+)	SNNGATNNNN	CCAGATTTAG
M00076	V$GATA2_01	0.820027	3037	(+)	NNNGATRNNN	CCAGATTTAG
M00077	V$GATA3_01	0.842268	3038	(+)	NNGATARNG	CAGATTTAG
M00203	V$GATA_C	0.868593	3059	(-)	NGATAAGNMNN	CTGTATTATCT
M00126	V$GATA1_02	0.87125	3060	(-)	NNNNNGATANKGNN	TGTATTATCTTTTT
M00127	V$GATA1_03	0.865997	3060	(-)	RNSNNGATAANNGN	TGTATTATCTTTTT
M00128	V$GATA1_04	0.882966	3060	(-)	NNCWGATARNNNNN	TGTATTATCTTTT
M00076	V$GATA2_01	0.817321	3062	(-)	NNNGATRNNN	TATTATCTTT
M00077	V$GATA3_01	0.898538	3062	(-)	NNGATARNG	TATTATCTT
M00127	V$GATA1_03	0.821166	3157	(+)	RNSNNGATAANNGN	GGGTTGCTAAGAGT
M00076	V$GATA2_01	0.797925	3172	(+)	NNNGATRNNN	GGACATGACG
M00127	V$GATA1_03	0.785889	3196	(-)	RNSNNGATAANNGN	TCACTTTTCACTTT
M00075	V$GATA1_01	0.786278	3204	(-)	SNNGATNNNN	CACTTTCATG
M00075	V$GATA1_01	0.826259	3257	(-)	SNNGATNNNN	GAGAATCCCA
M00076	V$GATA2_01	0.860171	3257	(-)	NNNGATRNNN	GAGAATCCCA
M00076	V$GATA2_01	0.793415	3293	(-)	NNNGATRNNN	GTCTATGGGG
M00075	V$GATA1_01	0.7769	3313	(+)	SNNGATNNNN	TTGGATACGA
M00076	V$GATA2_01	0.815065	3313	(+)	NNNGATRNNN	TTGGATACGA

M0020		0.86424				
3	V$GATA_C	4	3315	(+)	NGATAAGNMNN	GGATACGACTG
M0007						
5	V$GATA1_01	0.7769	3337	(-)	SNNGATNNNN	CAGCAGCAGC
M0012		0.80181				
7	V$GATA1_03	3	3351	(+)	RNSNNGATAANNGN	GGGCAGTTAAGGCT
M0012		0.83180				
8	V$GATA1_04	1	3352	(+)	NNCWGATARNNNN	GGCAGTTAAGGCT
M0020		0.83970				
3	V$GATA_C	2	3355	(+)	NGATAAGNMNN	AGTTAAGGCTT
M0007		0.79565				
5	V$GATA1_01	6	3417	(+)	SNNGATNNNN	TTCGATTCCT
M0007		0.82181				
5	V$GATA1_01	6	3427	(+)	SNNGATNNNN	GAAGATCTGC
M0007		0.83020				
5	V$GATA1_01	7	3427	(-)	SNNGATNNNN	GAAGATCTGC
M0007		0.84709				
6	V$GATA2_01	1	3427	(+)	NNNGATRNNN	GAAGATCTGC
M0007		0.84889				
6	V$GATA2_01	5	3427	(-)	NNNGATRNNN	GAAGATCTGC
M0012						
6	V$GATA1_02	0.855	3441	(+)	NNNNNGATANKGNN	GAAGGGATAGGCTA
M0012						
7	V$GATA1_03	0.82974	3441	(+)	RNSNNGATAANNGN	GAAGGGATAGGCTA
M0007		0.87956				
5	V$GATA1_01	6	3443	(+)	SNNGATNNNN	AGGGATAGGC
M0007		0.92377				
6	V$GATA2_01	1	3443	(+)	NNNGATRNNN	AGGGATAGGC
M0007		0.88790				
7	V$GATA3_01	4	3444	(+)	NNGATARNG	GGGATAGGC
M0020		0.85865				
3	V$GATA_C	2	3445	(+)	NGATAAGNMNN	GGATAGGCTAC
M0020		0.83442				
3	V$GATA_C	1	3593	(-)	NGATAAGNMNN	AAATTGTATCA
M0012		0.80218				
6	V$GATA1_02	8	3594	(-)	NNNNNGATANKGNN	AATTGTATCACTGT
M0012		0.78270				
7	V$GATA1_03	5	3594	(-)	RNSNNGATAANNGN	AATTGTATCACTGT
M0007		0.80651				
5	V$GATA1_01	5	3596	(-)	SNNGATNNNN	TTGTATCACT
M0007						
6	V$GATA2_01	0.80018	3596	(-)	NNNGATRNNN	TTGTATCACT
M0007		0.82144				
7	V$GATA3_01	4	3596	(-)	NNGATARNG	TTGTATCAC
M0012		0.84713				
7	V$GATA1_03	4	3641	(-)	RNSNNGATAANNGN	CCTCTTTTCTGTCT
M0012		0.82506				
8	V$GATA1_04	1	3641	(-)	NNCWGATARNNNN	CCTCTTTTCTGTC
M0012		0.80867				
7	V$GATA1_03	2	3665	(-)	RNSNNGATAANNGN	CTGTTTACCTTCTT
M0007		0.80207				
5	V$GATA1_01	3	3750	(+)	SNNGATNNNN	AGGGATGCAC
M0007		0.82363				
6	V$GATA2_01	6	3750	(+)	NNNGATRNNN	AGGGATGCAC
M0007		0.80920				
6	V$GATA2_01	2	3788	(+)	NNNGATRNNN	GGACATATTT
M0007		0.86722				
5	V$GATA1_01	6	3810	(-)	SNNGATNNNN	AGCAATCTAG
M0007						
6	V$GATA2_01	0.81281	3810	(-)	NNNGATRNNN	AGCAATCTAG
M0007		0.82100				
7	V$GATA3_01	1	3810	(-)	NNGATARNG	AGCAATCTA
M0007		0.84452				
5	V$GATA1_01	1	3851	(-)	SNNGATNNNN	CTAAATCTGC
M0007						
6	V$GATA2_01	0.82544	3851	(-)	NNNGATRNNN	CTAAATCTGC

M00077	V$GATA3_01	0.842268	3851	(-)	NNGATARNG	CTAAATCTG
M00127	V$GATA1_03	0.846399	3872	(+)	RNSNNGATAANNGN	AGGTAGATATTAAC
M00075	V$GATA1_01	0.804047	3874	(+)	SNNGATNNNN	GTAGATATTA
M00076	V$GATA2_01	0.797474	3874	(+)	NNNGATRNNN	GTAGATATTA
M00077	V$GATA3_01	0.842268	3875	(+)	NNGATARNG	TAGATATTA
M00203	V$GATA_C	0.831935	3876	(+)	NGATAAGNMNN	AGATATTAACC
M00076	V$GATA2_01	0.865133	3885	(-)	NNNGATRNNN	CCCTATGCCA
M00127	V$GATA1_03	0.795198	3905	(-)	RNSNNGATAANNGN	TCTGTCATCAGCTG
M00075	V$GATA1_01	0.863277	3907	(-)	SNNGATNNNN	TGTCATCAGC
M00076	V$GATA2_01	0.851601	3907	(-)	NNNGATRNNN	TGTCATCAGC
M00075	V$GATA1_01	0.816387	3915	(+)	SNNGATNNNN	GCTGTTGGTT
M00127	V$GATA1_03	0.808672	3958	(+)	RNSNNGATAANNGN	AAGGACATAAGATC
M00076	V$GATA2_01	0.811457	3965	(+)	NNNGATRNNN	TAAGATCCTA
M00076	V$GATA2_01	0.814614	3965	(-)	NNNGATRNNN	TAAGATCCTA
M00127	V$GATA1_03	0.784664	3968	(-)	RNSNNGATAANNGN	GATCCTATGCTGCT
M00076	V$GATA2_01	0.823184	3970	(-)	NNNGATRNNN	TCCTATGCTG
M00075	V$GATA1_01	0.7769	3976	(+)	SNNGATNNNN	GCTGCTGCTG
M00075	V$GATA1_01	0.7769	3979	(+)	SNNGATNNNN	GCTGCTGCTG
M00075	V$GATA1_01	0.7769	3982	(+)	SNNGATNNNN	GCTGCTGCTG
M00076	V$GATA2_01	0.818674	4016	(-)	NNNGATRNNN	CTCTATGCGA
M00076	V$GATA2_01	0.793415	4026	(+)	NNNGATRNNN	CCCCATAGAC
M00075	V$GATA1_01	0.826259	4062	(+)	SNNGATNNNN	TGGGATTCTC
M00076	V$GATA2_01	0.860171	4062	(+)	NNNGATRNNN	TGGGATTCTC
M00075	V$GATA1_01	0.786278	4115	(+)	SNNGATNNNN	CATGAAAGTG
M00126	V$GATA1_02	0.785938	4119	(+)	NNNNNGATANKGNN	AAAGTGAAAAGGGA
M00127	V$GATA1_03	0.795198	4119	(+)	RNSNNGATAANNGN	AAAGTGAAAAGGGA
M00076	V$GATA2_01	0.784844	4147	(-)	NNNGATRNNN	AGTCATGTCC
M00127	V$GATA1_03	0.807447	4158	(-)	RNSNNGATAANNGN	ACTCCTAGCTACCC
M00127	V$GATA1_03	0.791524	4181	(-)	RNSNNGATAANNGN	CAGCCTACCAGGCT
M00075	V$GATA1_01	0.805035	4205	(+)	SNNGATNNNN	TGGGATTTTC
M00076	V$GATA2_01	0.848895	4205	(+)	NNNGATRNNN	TGGGATTTTC
M00126	V$GATA1_02	0.7775	4239	(-)	NNNNNGATANKGNN	GCCATTACCTTCTC
M00127	V$GATA1_03	0.830475	4239	(-)	RNSNNGATAANNGN	GCCATTACCTTCTC

M00077	V$GATA3_01	0.824546	4246	(-)	NNGATARNG	CCTTCTCTG
M00075	V$GATA1_01	0.845508	4254	(+)	SNNGATNNNN	GAAGATCCTT
M00076	V$GATA2_01	0.855661	4254	(+)	NNNGATRNNN	GAAGATCCTT
M00076	V$GATA2_01	0.8083	4254	(-)	NNNGATRNNN	GAAGATCCTT
M00127	V$GATA1_03	0.804018	4258	(+)	RNSNNGATAANNGN	ATCCTTATAGGAGA
M00127	V$GATA1_03	0.791034	4301	(+)	RNSNNGATAANNGN	CTAAACATAACCAG
M00127	V$GATA1_03	0.821166	4322	(+)	RNSNNGATAANNGN	AGAATGATTATTGC
M00075	V$GATA1_01	0.789733	4324	(+)	SNNGATNNNN	AATGATTATT
M00076	V$GATA2_01	0.801985	4374	(-)	NNNGATRNNN	AGTTATGACC
M00076	V$GATA2_01	0.806495	4392	(+)	NNNGATRNNN	CAGCATATTC
M00075	V$GATA1_01	0.836624	4432	(-)	SNNGATNNNN	GTCCATCTAG
M00076	V$GATA2_01	0.786198	4432	(-)	NNNGATRNNN	GTCCATCTAG
M00076	V$GATA2_01	0.802887	4446	(-)	NNNGATRNNN	GGCTATGGTT
M00127	V$GATA1_03	0.804508	4492	(+)	RNSNNGATAANNGN	GTGAAGAAAGCTGA
M00075	V$GATA1_01	0.79615	4516	(+)	SNNGATNNNN	ATAGATGCTT
M00075	V$GATA1_01	0.79615	4535	(+)	SNNGATNNNN	GGTGTTGGAG
M00075	V$GATA1_01	0.799112	4574	(+)	SNNGATNNNN	GGAGATCCAA
M00076	V$GATA2_01	0.793866	4574	(+)	NNNGATRNNN	GGAGATCCAA
M00127	V$GATA1_03	0.815532	4597	(+)	RNSNNGATAANNGN	AAGGAGATCAGCCC
M00075	V$GATA1_01	0.832182	4599	(+)	SNNGATNNNN	GGAGATCAGC
M00075	V$GATA1_01	0.850938	4599	(-)	SNNGATNNNN	GGAGATCAGC
M00076	V$GATA2_01	0.840325	4599	(+)	NNNGATRNNN	GGAGATCAGC
M00076	V$GATA2_01	0.856563	4599	(-)	NNNGATRNNN	GGAGATCAGC
M00203	V$GATA_C	0.840323	4601	(+)	NGATAAGNMNN	AGATCAGCCCT
M00075	V$GATA1_01	0.819842	4611	(+)	SNNGATNNNN	TGGGATTTCT
M00076	V$GATA2_01	0.871448	4611	(+)	NNNGATRNNN	TGGGATTTCT
M00075	V$GATA1_01	0.817374	4630	(+)	SNNGATNNNN	AATGATGCTA
M00076	V$GATA2_01	0.803789	4630	(+)	NNNGATRNNN	AATGATGCTA
M00075	V$GATA1_01	0.836624	4699	(+)	SNNGATNNNN	TTTGATGCTG
M00076	V$GATA2_01	0.797474	4699	(+)	NNNGATRNNN	TTTGATGCTG
M00075	V$GATA1_01	0.830207	4711	(+)	SNNGATNNNN	AGGGATTGAG
M00076	V$GATA2_01	0.829499	4711	(+)	NNNGATRNNN	AGGGATTGAG
M00077	V$GATA3_01	0.880372	4712	(+)	NNGATARNG	GGGATTGAG

M00127	V$GATA1_03	0.870162	4724	(+)	RNSNNGATAANNGN	AGGAGGAGAAGGGG
M00075	V$GATA1_01	0.802073	4728	(+)	SNNGATNNNN	GGAGAAGGGG
M00075	V$GATA1_01	0.829714	4744	(+)	SNNGATNNNN	GAGGATGAGA
M00076	V$GATA2_01	0.856563	4744	(+)	NNNGATRNNN	GAGGATGAGA
M00077	V$GATA3_01	0.820558	4745	(+)	NNGATARNG	AGGATGAGA
M00075	V$GATA1_01	0.894867	4749	(+)	SNNGATNNNN	TGAGATGGCT
M00076	V$GATA2_01	0.929635	4749	(+)	NNNGATRNNN	TGAGATGGCT
M00077	V$GATA3_01	0.842712	4750	(+)	NNGATARNG	GAGATGGCT
M00075	V$GATA1_01	0.850938	4757	(+)	SNNGATNNNN	CTGGATGGCA
M00076	V$GATA2_01	0.833559	4757	(+)	NNNGATRNNN	CTGGATGGCA
M00075	V$GATA1_01	0.846002	4762	(-)	SNNGATNNNN	TGGCATCACT
M00076	V$GATA2_01	0.830853	4762	(-)	NNNGATRNNN	TGGCATCACT
M00075	V$GATA1_01	0.851431	4774	(+)	SNNGATNNNN	CTCGATGGAC
M00076	V$GATA2_01	0.78304	4774	(+)	NNNGATRNNN	CTCGATGGAC
M00075	V$GATA1_01	0.814906	4804	(+)	SNNGATNNNN	GGAGTTGGTG
M00075	V$GATA1_01	0.917572	4810	(+)	SNNGATNNNN	GGTGATGGAC
M00076	V$GATA2_01	0.870095	4810	(+)	NNNGATRNNN	GGTGATGGAC
M00077	V$GATA3_01	0.856447	4906	(+)	NNGATARNG	ATGATTAAG
M00127	V$GATA1_03	0.814062	4925	(-)	RNSNNGATAANNGN	GTGGTTATTATTAT
M00127	V$GATA1_03	0.788339	4951	(-)	RNSNNGATAANNGN	ACTATTATTCTTTT
M00075	V$GATA1_01	0.807502	4969	(+)	SNNGATNNNN	CCAGATGAAA
M00076	V$GATA2_01	0.798376	4969	(+)	NNNGATRNNN	CCAGATGAAA
M00075	V$GATA1_01	0.780355	4984	(-)	SNNGATNNNN	AGAGATCATT
M00076	V$GATA2_01	0.790708	4984	(+)	NNNGATRNNN	AGAGATCATT
M00076	V$GATA2_01	0.805142	4984	(-)	NNNGATRNNN	AGAGATCATT
M00127	V$GATA1_03	0.853993	5003	(+)	RNSNNGATAANNGN	AAGGAGAAAACAGG
M00127	V$GATA1_03	0.814797	5014	(+)	RNSNNGATAANNGN	AGGGTGATTGAGGG
M00075	V$GATA1_01	0.920039	5016	(+)	SNNGATNNNN	GGTGATTGAG
M00076	V$GATA2_01	0.850699	5016	(+)	NNNGATRNNN	GGTGATTGAG
M00077	V$GATA3_01	0.902082	5017	(+)	NNGATARNG	GTGATTGAG
M00076	V$GATA2_01	0.782138	5067	(-)	NNNGATRNNN	CTGTATGACC
M00076	V$GATA2_01	0.784393	5106	(-)	NNNGATRNNN	GTCTATGGGA
M00075	V$GATA1_01	0.805035	5111	(+)	SNNGATNNNN	TGGGATTTTC

M00076	V$GATA2_01	0.848895	5111	(+)	NNNGATRNNNN	TGGGATTTTC
M00075	V$GATA1_01	0.785785	5178	(-)	SNNGATNNNN	TTGCATCTCT
M00076	V$GATA2_01	0.78304	5178	(-)	NNNGATRNNNN	TTGCATCTCT
M00126	V$GATA1_02	0.771562	5183	(-)	NNNNNGATANKGNN	TCTCTTATGTCTCC
M00127	V$GATA1_03	0.814307	5183	(-)	RNSNNGATAANNGN	TCTCTTATGTCTCC
M00126	V$GATA1_02	0.798437	5218	(-)	NNNNNGATANKGNN	ACCACTAGCACCAC
M00127	V$GATA1_03	0.797158	5218	(-)	RNSNNGATAANNGN	ACCACTAGCACCAC
M00075	V$GATA1_01	0.810464	5220	(-)	SNNGATNNNN	CACTAGCACC
M00127	V$GATA1_03	0.795198	5241	(-)	RNSNNGATAANNGN	CCAATTAGCATCTG
M00075	V$GATA1_01	0.817374	5246	(-)	SNNGATNNNN	TAGCATCTGT
M00076	V$GATA2_01	0.823184	5246	(-)	NNNGATRNNNN	TAGCATCTGT
M00128	V$GATA1_04	0.818627	5250	(+)	NNCWGATARNNNN	ATCTGTTAAAGCC
M00127	V$GATA1_03	0.828025	5274	(-)	RNSNNGATAANNGN	TCTCTAATCCTCTT
M00075	V$GATA1_01	0.806022	5276	(-)	SNNGATNNNN	TCTAATCCTC
M00076	V$GATA2_01	0.820478	5276	(-)	NNNGATRNNNN	TCTAATCCTC
M00077	V$GATA3_01	0.840496	5276	(-)	NNGATARNG	TCTAATCCT
M00127	V$GATA1_03	0.784664	5283	(-)	RNSNNGATAANNGN	CTCTTTTTCTTGAC
M00075	V$GATA1_01	0.889437	5296	(-)	SNNGATNNNN	CTGCATCATC
M00076	V$GATA2_01	0.853406	5296	(-)	NNNGATRNNNN	CTGCATCATC
M00075	V$GATA1_01	0.804047	5299	(-)	SNNGATNNNN	CATCATCTTT
M00076	V$GATA2_01	0.831755	5299	(-)	NNNGATRNNNN	CATCATCTTT
M00077	V$GATA3_01	0.879929	5299	(-)	NNGATARNG	CATCATCTT
M00075	V$GATA1_01	0.791214	5351	(-)	SNNGATNNNN	AACTTTCAGC
M00127	V$GATA1_03	0.792014	5367	(+)	RNSNNGATAANNGN	ATAATGATATCATC
M00075	V$GATA1_01	0.79615	5369	(+)	SNNGATNNNN	AATGATATCA
M00076	V$GATA2_01	0.839874	5369	(+)	NNNGATRNNNN	AATGATATCA
M00077	V$GATA3_01	0.827204	5370	(+)	NNGATARNG	ATGATATCA
M00075	V$GATA1_01	0.860809	5371	(-)	SNNGATNNNN	TGATATCATC
M00076	V$GATA2_01	0.886784	5371	(-)	NNNGATRNNNN	TGATATCATC
M00077	V$GATA3_01	0.827204	5371	(-)	NNGATARNG	TGATATCAT
M00075	V$GATA1_01	0.797631	5421	(+)	SNNGATNNNN	TCTGATTTAT
M00076	V$GATA2_01	0.786649	5421	(+)	NNNGATRNNNN	TCTGATTTAT
M00075	V$GATA1_01	0.86229	5464	(+)	SNNGATNNNN	CCTGATTTTA

M00076	V$GATA2_01	0.806044	5464	(+)	NNNGATRNNN	CCTGATTTTA
M00075	V$GATA1_01	0.772458	5489	(-)	SNNGATNNNN	AGGAATCTAT
M00075	V$GATA1_01	0.807996	5502	(-)	SNNGATNNNN	ACACATCAAT
M00075	V$GATA1_01	0.780355	5558	(+)	SNNGATNNNN	TTTGATTTTT
M00075	V$GATA1_01	0.845015	5588	(+)	SNNGATNNNN	TGTGATTTTT
M00076	V$GATA2_01	0.826793	5588	(+)	NNNGATRNNN	TGTGATTTTT
M00127	V$GATA1_03	0.804998	5603	(+)	RNSNNGATAANNGN	AACCAGAAAATTGA
M00076	V$GATA2_01	0.800631	5661	(-)	NNNGATRNNN	CACTATGAGT
M00127	V$GATA1_03	0.784664	5717	(-)	RNSNNGATAANNGN	ATAATTATGTTCAG
M00076	V$GATA2_01	0.786198	5719	(-)	NNNGATRNNN	AATTATGTTC
M00127	V$GATA1_03	0.784664	5815	(+)	RNSNNGATAANNGN	AAACACATAATCAA
M00075	V$GATA1_01	0.773445	5820	(-)	SNNGATNNNN	CATAATCAAA
M00077	V$GATA3_01	0.852016	5820	(-)	NNGATARNG	CATAATCAA
M00127	V$GATA1_03	0.805488	5864	(-)	RNSNNGATAANNGN	ACTGGTATATTCAT
M00127	V$GATA1_03	0.828025	5940	(-)	RNSNNGATAANNGN	ACTGTGATCCCCTT
M00075	V$GATA1_01	0.823791	5942	(+)	SNNGATNNNN	TGTGATCCCC
M00075	V$GATA1_01	0.794669	5942	(-)	SNNGATNNNN	TGTGATCCCC
M00076	V$GATA2_01	0.853406	5942	(+)	NNNGATRNNN	TGTGATCCCC
M00076	V$GATA2_01	0.841227	5942	(-)	NNNGATRNNN	TGTGATCCCC
M00075	V$GATA1_01	0.839092	5959	(+)	SNNGATNNNN	AATGATGGAT
M00076	V$GATA2_01	0.818674	5959	(+)	NNNGATRNNN	AATGATGGAT
M00127	V$GATA1_03	0.794463	5987	(+)	RNSNNGATAANNGN	AGGGTGACAGTGTG
M00075	V$GATA1_01	0.810464	5989	(+)	SNNGATNNNN	GGTGACAGTG
M00076	V$GATA2_01	0.79567	5989	(+)	NNNGATRNNN	GGTGACAGTG
M00127	V$GATA1_03	0.851053	6001	(-)	RNSNNGATAANNGN	GCACTAATCCTCAT
M00075	V$GATA1_01	0.84156	6003	(-)	SNNGATNNNN	ACTAATCCTC
M00076	V$GATA2_01	0.850248	6003	(-)	NNNGATRNNN	ACTAATCCTC
M00203	V$GATA_C	0.911153	6018	(-)	NGATAAGNMNN	ATCATTTATCA
M00126	V$GATA1_02	0.800938	6019	(-)	NNNNNGATANKGNN	TCATTTATCAGCTG
M00127	V$GATA1_03	0.867222	6019	(-)	RNSNNGATAANNGN	TCATTTATCAGCTG
M00128	V$GATA1_04	0.928002	6019	(-)	NNCWGATARNNNNN	TCATTTATCAGCT
M00075	V$GATA1_01	0.859329	6021	(-)	SNNGATNNNN	ATTTATCAGC
M00076	V$GATA2_01	0.850699	6021	(-)	NNNGATRNNN	ATTTATCAGC

M0012		0.78270				
7	V$GATA1_03	5	6028	(+)	RNSNNGATAANNGN	AGCTGTATAACCTT
M0007		0.77739				
5	V$GATA1_01	4	6058	(-)	SNNGATNNNN	GTACATCATT
M0012		0.81014				
7	V$GATA1_03	2	6062	(-)	RNSNNGATAANNGN	ATCATTTTCTTCAC
M0007		0.78775				
5	V$GATA1_01	9	6108	(+)	SNNGATNNNN	GTTGATGAAC
M0012		0.77343				
6	V$GATA1_02	8	6152	(+)	NNNNNGATANKGNN	TATTTGATAGCTTT
M0012		0.82077				
8	V$GATA1_04	2	6153	(+)	NNCWGATARNNNN	ATTTGATAGCTTT
M0007		0.85093				
5	V$GATA1_01	8	6154	(+)	SNNGATNNNN	TTTGATAGCT
M0007		0.85701				
6	V$GATA2_01	4	6154	(+)	NNNGATRNNN	TTTGATAGCT
M0007		0.83119				
7	V$GATA3_01	2	6155	(+)	NNGATARNG	TTGATAGCT
M0012		0.81185				
7	V$GATA1_03	7	6161	(-)	RNSNNGATAANNGN	GCTTTTATACTCAC
M0012		0.80450				
7	V$GATA1_03	8	6207	(-)	RNSNNGATAANNGN	TCCATTACCCAGAT
M0012		0.82116				
7	V$GATA1_03	6	6213	(+)	RNSNNGATAANNGN	ACCCAGATGAGAGA
M0007		0.85932				
5	V$GATA1_01	9	6215	(+)	SNNGATNNNN	CCAGATGAGA
M0007		0.84483				
6	V$GATA2_01	5	6215	(+)	NNNGATRNNN	CCAGATGAGA
M0007		0.85999				
7	V$GATA3_01	1	6216	(+)	NNGATARNG	CAGATGAGA
M0007		0.79837				
6	V$GATA2_01	6	6262	(+)	NNNGATRNNN	GACCATACCA
M0007		0.84032				
6	V$GATA2_01	5	6269	(+)	NNNGATRNNN	CCACATAGTT
M0012		0.79062				
6	V$GATA1_02	5	6298	(+)	NNNNNGATANKGNN	TTAAAAATAAGGAT
M0020		0.83473				
3	V$GATA_C	1	6302	(+)	NGATAAGNMNN	AAATAAGGATT
M0007		0.80651				
5	V$GATA1_01	5	6306	(+)	SNNGATNNNN	AAGGATTTGG
M0007		0.83130				
6	V$GATA2_01	4	6306	(+)	NNNGATRNNN	AAGGATTTGG
M0007		0.84359				
7	V$GATA3_01	8	6307	(+)	NNGATARNG	AGGATTTGG
M0007		0.79747				
6	V$GATA2_01	4	6314	(+)	NNNGATRNNN	GGGGACAGCA
M0007		0.78664				
6	V$GATA2_01	9	6354	(+)	NNNGATRNNN	TAACATATTT
M0020		0.90897				
3	V$GATA_C	8	6357	(-)	NGATAAGNMNN	CATATTTATCT
M0012						
8	V$GATA1_04	0.89185	6358	(-)	NNCWGATARNNNN	ATATTTATCTAAT
M0007		0.82233				
7	V$GATA3_01	1	6360	(-)	NNGATARNG	ATTTATCTA
M0012						
8	V$GATA1_04	0.8125	6370	(+)	NNCWGATARNNNN	TTGTGATATTTAA
M0007		0.86870				
5	V$GATA1_01	7	6371	(+)	SNNGATNNNN	TGTGATATTT
M0007		0.89129				
6	V$GATA2_01	5	6371	(+)	NNNGATRNNN	TGTGATATTT
M0007		0.82853				
7	V$GATA3_01	3	6372	(+)	NNGATARNG	GTGATATTT
M0007		0.85932				
5	V$GATA1_01	9	6402	(+)	SNNGATNNNN	GAAGATTGAT
M0007		0.84077				
6	V$GATA2_01	6	6402	(+)	NNNGATRNNN	GAAGATTGAT

M00077	V$GATA3_01	0.841382	6403	(+)	NNGATARNG	AAGATTGAT
M00075	V$GATA1_01	0.818855	6406	(+)	SNNGATNNNN	ATTGATTGTT
M00075	V$GATA1_01	0.885982	6460	(+)	SNNGATNNNN	CTTGATGCCT
M00076	V$GATA2_01	0.812359	6460	(+)	NNNGATRNNN	CTTGATGCCT
M00127	V$GATA1_03	0.807202	6477	(+)	RNSNNGATAANNGN	CTCAAGATTACCAC
M00075	V$GATA1_01	0.823297	6479	(+)	SNNGATNNNN	CAAGATTACC
M00076	V$GATA2_01	0.839423	6479	(+)	NNNGATRNNN	CAAGATTACC
M00127	V$GATA1_03	0.782705	6480	(-)	RNSNNGATAANNGN	AAGATTACCACCTT
M00127	V$GATA1_03	0.807692	6496	(+)	RNSNNGATAANNGN	ACCAAGAGAAGTAG
M00075	V$GATA1_01	0.793189	6648	(-)	SNNGATNNNN	TGAAATCAGT
M00076	V$GATA2_01	0.783491	6648	(-)	NNNGATRNNN	TGAAATCAGT
M00203	V$GATA_C	0.846536	6661	(-)	NGATAAGNMNN	GACTATTATCC
M00126	V$GATA1_02	0.808438	6662	(-)	NNNNNGATANKGNN	ACTATTATCCAAAG
M00127	V$GATA1_03	0.801813	6662	(-)	RNSNNGATAANNGN	ACTATTATCCAAAG
M00076	V$GATA2_01	0.782589	6664	(-)	NNNGATRNNN	TATTATCCAA
M00077	V$GATA3_01	0.828977	6664	(-)	NNGATARNG	TATTATCCA
M00127	V$GATA1_03	0.784174	6679	(-)	RNSNNGATAANNGN	GTACCTACCACTCT
M00075	V$GATA1_01	0.845015	6725	(-)	SNNGATNNNN	TGAAATCTCC
M00076	V$GATA2_01	0.841678	6725	(-)	NNNGATRNNN	TGAAATCTCC
M00077	V$GATA3_01	0.834293	6725	(-)	NNGATARNG	TGAAATCTC
M00075	V$GATA1_01	0.86229	6739	(+)	SNNGATNNNN	CCTGATTTCA
M00076	V$GATA2_01	0.824989	6739	(+)	NNNGATRNNN	CCTGATTTCA
M00075	V$GATA1_01	0.801579	6757	(+)	SNNGATNNNN	TCTGATTTCA
M00076	V$GATA2_01	0.815968	6757	(+)	NNNGATRNNN	TCTGATTTCA
M00075	V$GATA1_01	0.787266	6776	(+)	SNNGATNNNN	GAAGTTGGGT
M00075	V$GATA1_01	0.782823	6791	(-)	SNNGATNNNN	AGAAATCCTT
M00076	V$GATA2_01	0.830853	6791	(-)	NNNGATRNNN	AGAAATCCTT
M00203	V$GATA_C	0.830693	6843	(-)	NGATAAGNMNN	ATTTCTTAACT
M00075	V$GATA1_01	0.837117	6864	(+)	SNNGATNNNN	GAAGATTTTC
M00076	V$GATA2_01	0.840776	6864	(+)	NNNGATRNNN	GAAGATTTTC
M00127	V$GATA1_03	0.785644	6865	(-)	RNSNNGATAANNGN	AAGATTTTCTTTCT
M00127	V$GATA1_03	0.785154	6871	(-)	RNSNNGATAANNGN	TTCTTTCTCTTCAC
M00127	V$GATA1_03	0.905683	6904	(+)	RNSNNGATAANNGN	AGGAGGATAATTCA

M00075	V$GATA1_01	0.852912	6906	(+)	SNNGATNNNN	GAGGATAATT
M00076	V$GATA2_01	0.908435	6906	(+)	NNNGATRNNN	GAGGATAATT
M00077	V$GATA3_01	0.821444	6907	(+)	NNGATARNG	AGGATAATT
M00203	V$GATA_C	0.893756	6908	(+)	NGATAAGNMNN	GGATAATTCAT
M00075	V$GATA1_01	0.78233	6913	(-)	SNNGATNNNN	ATTCATCATA
M00076	V$GATA2_01	0.799729	6913	(-)	NNNGATRNNN	ATTCATCATA
M00127	V$GATA1_03	0.802058	6927	(+)	RNSNNGATAANNGN	ACAATTATAACTGG
M00127	V$GATA1_03	0.788339	6935	(+)	RNSNNGATAANNGN	AACTGGATTATGGA
M00075	V$GATA1_01	0.808983	6937	(+)	SNNGATNNNN	CTGGATTATG
M00077	V$GATA3_01	0.830306	6938	(+)	NNGATARNG	TGGATTATG
M00075	V$GATA1_01	0.80306	6974	(+)	SNNGATNNNN	CAGGATGAAC
M00076	V$GATA2_01	0.830853	6974	(+)	NNNGATRNNN	CAGGATGAAC
M00075	V$GATA1_01	0.78233	6986	(+)	SNNGATNNNN	CAGGATAAAA
M00076	V$GATA2_01	0.833108	6986	(+)	NNNGATRNNN	CAGGATAAAA
M00077	V$GATA3_01	0.833407	6987	(+)	NNGATARNG	AGGATAAAA
M00203	V$GATA_C	0.898105	6988	(+)	NGATAAGNMNN	GGATAAAATCC
M00075	V$GATA1_01	0.775913	6991	(-)	SNNGATNNNN	TAAAATCCAC
M00126	V$GATA1_02	0.804062	7023	(-)	NNNNNGATANKGNN	TAGTCTATCCCTTC
M00127	V$GATA1_03	0.797893	7023	(-)	RNSNNGATAANNGN	TAGTCTATCCCTTC
M00075	V$GATA1_01	0.827739	7025	(-)	SNNGATNNNN	GTCTATCCCT
M00076	V$GATA2_01	0.877312	7025	(-)	NNNGATRNNN	GTCTATCCCT
M00077	V$GATA3_01	0.847142	7025	(-)	NNGATARNG	GTCTATCCC
M00075	V$GATA1_01	0.879566	7042	(-)	SNNGATNNNN	GCCCATCCCT
M00076	V$GATA2_01	0.895354	7042	(-)	NNNGATRNNN	GCCCATCCCT
M00077	V$GATA3_01	0.834293	7042	(-)	NNGATARNG	GCCCATCCC
M00127	V$GATA1_03	0.810142	7046	(-)	RNSNNGATAANNGN	ATCCCTAACAGCCT
M00128	V$GATA1_04	0.812194	7046	(-)	NNCWGATARNNNN	ATCCCTAACAGCC
M00075	V$GATA1_01	0.840079	7048	(-)	SNNGATNNNN	CCCTAACAGC
M00076	V$GATA2_01	0.801985	7048	(-)	NNNGATRNNN	CCCTAACAGC
M00075	V$GATA1_01	0.875617	7066	(-)	SNNGATNNNN	AAACATCCTG
M00076	V$GATA2_01	0.88949	7066	(-)	NNNGATRNNN	AAACATCCTG
M00076	V$GATA2_01	0.783942	7150	(-)	NNNGATRNNN	GACTATGGTT
M00127	V$GATA1_03	0.784664	7185	(-)	RNSNNGATAANNGN	CTAAATATCCAGTT

M00075	V$GATA1_01	0.839585	7187	(-)	SNNGATNNNN	AAATATCCAG
M00076	V$GATA2_01	0.836265	7187	(-)	NNNGATRNNN	AAATATCCAG
M00075	V$GATA1_01	0.863771	7232	(+)	SNNGATNNNN	ACTGATGTTG
M00076	V$GATA2_01	0.843482	7232	(+)	NNNGATRNNN	ACTGATGTTG
M00127	V$GATA1_03	0.805732	7236	(+)	RNSNNGATAANNGN	ATGTTGAAAAGCTG
M00075	V$GATA1_01	0.774926	7309	(-)	SNNGATNNNN	AACCGTCATG
M00077	V$GATA3_01	0.840496	7341	(-)	NNGATARNG	CCCTTTCTC
M00127	V$GATA1_03	0.821656	7341	(-)	RNSNNGATAANNGN	CCCTTTCTCAGCCC
M00128	V$GATA1_04	0.810968	7341	(-)	NNCWGATARNNNN	CCCTTTCTCAGCC
M00126	V$GATA1_02	0.790937	7389	(+)	NNNNNGATANKGNN	AGAGAGATATGCCC
M00127	V$GATA1_03	0.818471	7389	(+)	RNSNNGATAANNGN	AGAGAGATATGCCC
M00128	V$GATA1_04	0.860907	7390	(+)	NNCWGATARNNNN	GAGAGATATGCCC
M00075	V$GATA1_01	0.837117	7391	(+)	SNNGATNNNN	AGAGATATGC
M00076	V$GATA2_01	0.872801	7391	(+)	NNNGATRNNN	AGAGATATGC
M00077	V$GATA3_01	0.87284	7392	(+)	NNGATARNG	GAGATATGC
M00076	V$GATA2_01	0.84258	7393	(-)	NNNGATRNNN	AGATATGCCC
M00203	V$GATA_C	0.905561	7393	(+)	NGATAAGNMNN	AGATATGCCCA
M00075	V$GATA1_01	0.884008	7399	(-)	SNNGATNNNN	GCCCATCCAG
M00076	V$GATA2_01	0.847091	7399	(-)	NNNGATRNNN	GCCCATCCAG
M00203	V$GATA_C	0.844672	7438	(-)	NGATAAGNMNN	TGGTCCTGTCC
M00075	V$GATA1_01	0.860809	7503	(+)	SNNGATNNNN	TCTGATGTTT
M00076	V$GATA2_01	0.862878	7503	(+)	NNNGATRNNN	TCTGATGTTT
M00128	V$GATA1_04	0.822917	7513	(+)	NNCWGATARNNNN	GTATGATATTTGA
M00075	V$GATA1_01	0.840079	7514	(+)	SNNGATNNNN	TATGATATTT
M00076	V$GATA2_01	0.883175	7514	(+)	NNNGATRNNN	TATGATATTT
M00075	V$GATA1_01	0.771964	7545	(-)	SNNGATNNNN	AAAAATCAAT
M00076	V$GATA2_01	0.824087	7573	(+)	NNNGATRNNN	AAGCATAGCT
M00076	V$GATA2_01	0.78304	7582	(+)	NNNGATRNNN	TGAGATTAAT
M00077	V$GATA3_01	0.830306	7583	(+)	NNGATARNG	GAGATTAAT
M00075	V$GATA1_01	0.814413	7589	(+)	SNNGATNNNN	AATGATTCTC
M00076	V$GATA2_01	0.793866	7589	(+)	NNNGATRNNN	AATGATTCTC
M00127	V$GATA1_03	0.823861	7590	(-)	RNSNNGATAANNGN	ATGATTCTCAGCAT
M00127	V$GATA1_03	0.782705	7621	(+)	RNSNNGATAANNGN	AGCTGGAAAACCTT

M0007 5	V$GATA1_01	0.79911 2	7629	(-)	SNNGATNNNN	AACCTTCACC
M0007 5	V$GATA1_01	0.81391 9	7651	(+)	SNNGATNNNN	CCAGATCTCA
M0007 5	V$GATA1_01	0.82033 6	7651	(-)	SNNGATNNNN	CCAGATCTCA
M0007 6	V$GATA2_01	0.83626 5	7651	(+)	NNNGATRNNN	CCAGATCTCA
M0007 6	V$GATA2_01	0.85746 5	7651	(-)	NNNGATRNNN	CCAGATCTCA
M0007 7	V$GATA3_01	0.82410 3	7651	(-)	NNGATARNG	CCAGATCTC
M0007 5	V$GATA1_01	0.91312 9	7679	(+)	SNNGATNNNN	GGAGATTCTG
M0007 6	V$GATA2_01	0.87686 1	7679	(+)	NNNGATRNNN	GGAGATTCTG
M0007 7	V$GATA3_01	0.86619 4	7680	(+)	NNGATARNG	GAGATTCTG
M0012 6	V$GATA1_02	0.80187 5	7731	(-)	NNNNNGATANKGNN	AATAATATCATGGA
M0007 5	V$GATA1_01	0.86525 2	7733	(-)	SNNGATNNNN	TAATATCATG
M0007 6	V$GATA2_01	0.86242 7	7733	(-)	NNNGATRNNN	TAATATCATG
M0007 7	V$GATA3_01	0.84315 5	7733	(-)	NNGATARNG	TAATATCAT
M0007 7	V$GATA3_01	0.83562 3	7773	(+)	NNGATARNG	TAGATGAAG
M0007 5	V$GATA1_01	0.79417 6	7775	(+)	SNNGATNNNN	GATGAAGGGG
M0007 5	V$GATA1_01	0.82971 4	7781	(+)	SNNGATNNNN	GGGGATTAAG
M0007 6	V$GATA2_01	0.82498 9	7781	(+)	NNNGATRNNN	GGGGATTAAG
M0007 7	V$GATA3_01	0.85201 6	7782	(+)	NNGATARNG	GGGATTAAG
M0012 6	V$GATA1_02	0.81843 8	7811	(+)	NNNNNGATANKGNN	AACTTGATAATCTC
M0012 7	V$GATA1_03	0.87922 6	7811	(+)	RNSNNGATAANNGN	AACTTGATAATCTC
M0007 5	V$GATA1_01	0.83168 8	7813	(+)	SNNGATNNNN	CTTGATAATC
M0007 6	V$GATA2_01	0.79206 1	7813	(+)	NNNGATRNNN	CTTGATAATC
M0020 3	V$GATA_C	0.84995 3	7815	(+)	NGATAAGNMNN	TGATAATCTCA
M0007 5	V$GATA1_01	0.79121 4	7816	(-)	SNNGATNNNN	GATAATCTCA
M0007 6	V$GATA2_01	0.81957 6	7816	(-)	NNNGATRNNN	GATAATCTCA
M0007 7	V$GATA3_01	0.85024 4	7816	(-)	NNGATARNG	GATAATCTC
M0007 5	V$GATA1_01	0.85686 1	7918	(-)	SNNGATNNNN	TCACATCTTG
M0007 6	V$GATA2_01	0.85160 1	7918	(-)	NNNGATRNNN	TCACATCTTG
M0007 7	V$GATA3_01	0.83385	7918	(-)	NNGATARNG	TCACATCTT
M0012 6	V$GATA1_02	0.77093 8	7928	(+)	NNNNNGATANKGNN	TTTTTGATATATGA
M0007 6	V$GATA2_01	0.79206 1	7940	(+)	NNNGATRNNN	GAGCATATGA
M0007 6	V$GATA2_01	0.79521 9	7942	(-)	NNNGATRNNN	GCATATGAGG
M0007 5	V$GATA1_01	0.8692	7961	(+)	SNNGATNNNN	TGAGATGCTT

M00076	V$GATA2_01	0.885431	7961	(+)	NNNGATRNNN	TGAGATGCTT
M00075	V$GATA1_01	0.811945	8065	(+)	SNNGATNNNN	GAGGATTTCA
M00076	V$GATA2_01	0.847993	8065	(+)	NNNGATRNNN	GAGGATTTCA
M00075	V$GATA1_01	0.839092	8111	(+)	SNNGATNNNN	GGAGATTAGA
M00076	V$GATA2_01	0.814163	8111	(+)	NNNGATRNNN	GGAGATTAGA
M00077	V$GATA3_01	0.902969	8112	(+)	NNGATARNG	GAGATTAGA
M00075	V$GATA1_01	0.782823	8126	(+)	SNNGATNNNN	ACTGATCATT
M00075	V$GATA1_01	0.774432	8126	(-)	SNNGATNNNN	ACTGATCATT
M00203	V$GATA_C	0.842808	8141	(-)	NGATAAGNMNN	AGTTAATATCT
M00126	V$GATA1_02	0.808125	8142	(-)	NNNNNGATANKGNN	GTTAATATCTTTTA
M00128	V$GATA1_04	0.845895	8142	(-)	NNCWGATARNNNN	GTTAATATCTTTT
M00075	V$GATA1_01	0.77542	8144	(-)	SNNGATNNNN	TAATATCTTT
M00076	V$GATA2_01	0.832206	8144	(-)	NNNGATRNNN	TAATATCTTT
M00077	V$GATA3_01	0.866637	8144	(-)	NNGATARNG	TAATATCTT
M00126	V$GATA1_02	0.813438	8166	(+)	NNNNNGATANKGNN	TCCTGGATAAGACC
M00075	V$GATA1_01	0.798124	8168	(+)	SNNGATNNNN	CTGGATAAGA
M00076	V$GATA2_01	0.797925	8168	(+)	NNNGATRNNN	CTGGATAAGA
M00077	V$GATA3_01	0.849801	8169	(+)	NNGATARNG	TGGATAAGA
M00203	V$GATA_C	0.968313	8170	(+)	NGATAAGNMNN	GGATAAGACCC
M00203	V$GATA_C	0.891271	8190	(-)	NGATAAGNMNN	AATTTCTATCA
M00126	V$GATA1_02	0.820625	8191	(-)	NNNNNGATANKGNN	ATTTCTATCAGTAT
M00127	V$GATA1_03	0.848849	8191	(-)	RNSNNGATAANNGN	ATTTCTATCAGTAT
M00128	V$GATA1_04	0.910233	8191	(-)	NNCWGATARNNNN	ATTTCTATCAGTA
M00075	V$GATA1_01	0.824284	8193	(-)	SNNGATNNNN	TTCTATCAGT
M00076	V$GATA2_01	0.82544	8193	(-)	NNNGATRNNN	TTCTATCAGT
M00077	V$GATA3_01	0.877714	8193	(-)	NNGATARNG	TTCTATCAG
M00127	V$GATA1_03	0.795198	8197	(+)	RNSNNGATAANNGN	ATCAGTATACCAGC
M00075	V$GATA1_01	0.781343	8213	(+)	SNNGATNNNN	AAAGATTAGT
M00076	V$GATA2_01	0.788904	8213	(+)	NNNGATRNNN	AAAGATTAGT
M00077	V$GATA3_01	0.853788	8214	(+)	NNGATARNG	AAGATTAGT
M00075	V$GATA1_01	0.82922	8238	(+)	SNNGATNNNN	AGTGATTGAC
M00076	V$GATA2_01	0.7871	8238	(+)	NNNGATRNNN	AGTGATTGAC
M00077	V$GATA3_01	0.835179	8239	(+)	NNGATARNG	GTGATTGAC

M00127	V$GATA1_03	0.788829	8246	(-)	RNSNNGATAANNGN	ACTGTTATTTACTG
M00203	V$GATA_C	0.888785	8267	(-)	NGATAAGNMNN	ATTATGTATCT
M00126	V$GATA1_02	0.8	8268	(-)	NNNNNGATANKGNN	TTATGTATCTGTTA
M00128	V$GATA1_04	0.839767	8268	(-)	NNCWGATARNNNNN	TTATGTATCTGTT
M00075	V$GATA1_01	0.813425	8270	(-)	SNNGATNNNN	ATGTATCTGT
M00076	V$GATA2_01	0.841227	8270	(-)	NNNGATRNNN	ATGTATCTGT
M00075	V$GATA1_01	0.784798	8320	(+)	SNNGATNNNN	ACAGATTTGA
M00076	V$GATA2_01	0.782138	8320	(+)	NNNGATRNNN	ACAGATTTGA
M00077	V$GATA3_01	0.848028	8321	(+)	NNGATARNG	CAGATTTGA
M00075	V$GATA1_01	0.839585	8325	(+)	SNNGATNNNN	TTTGATTGGT
M00077	V$GATA3_01	0.834293	8326	(+)	NNGATARNG	TTGATTGGT
M00127	V$GATA1_03	0.800833	8345	(-)	RNSNNGATAANNGN	TTGCCTATATCCTT
M00203	V$GATA_C	0.849332	8346	(-)	NGATAAGNMNN	TGCCTATATCC
M00126	V$GATA1_02	0.784375	8347	(-)	NNNNNGATANKGNN	GCCTATATCCTTAT
M00127	V$GATA1_03	0.805488	8347	(-)	RNSNNGATAANNGN	GCCTATATCCTTAT
M00075	V$GATA1_01	0.778381	8349	(-)	SNNGATNNNN	CTATATCCTT
M00076	V$GATA2_01	0.849346	8349	(-)	NNNGATRNNN	CTATATCCTT
M00077	V$GATA3_01	0.836509	8349	(-)	NNGATARNG	CTATATCCT
M00075	V$GATA1_01	0.783317	8362	(+)	SNNGATNNNN	ATTGATTGTA
M00077	V$GATA3_01	0.84537	8363	(+)	NNGATARNG	TTGATTGTA
M00077	V$GATA3_01	0.825432	8368	(-)	NNGATARNG	TGTAATCAT
M00075	V$GATA1_01	0.792695	8420	(+)	SNNGATNNNN	CCTGTTAGAG
M00075	V$GATA1_01	0.808983	8438	(-)	SNNGATNNNN	AAAGATCAAC
M00203	V$GATA_C	0.830693	8457	(-)	NGATAAGNMNN	ATTTCTAATCT
M00075	V$GATA1_01	0.778875	8460	(-)	SNNGATNNNN	TCTAATCTAG
M00077	V$GATA3_01	0.86132	8460	(-)	NNGATARNG	TCTAATCTA
M00127	V$GATA1_03	0.817001	8469	(-)	RNSNNGATAANNGN	GTCATTATGAGCTT
M00076	V$GATA2_01	0.796121	8471	(-)	NNNGATRNNN	CATTATGAGC
M00203	V$GATA_C	0.946878	8486	(-)	NGATAAGNMNN	TTGTTTTATCT
M00126	V$GATA1_02	0.770625	8487	(-)	NNNNNGATANKGNN	TGTTTTATCTCACT
M00127	V$GATA1_03	0.800098	8487	(-)	RNSNNGATAANNGN	TGTTTTATCTCACT
M00128	V$GATA1_04	0.902574	8487	(-)	NNCWGATARNNNNN	TGTTTTATCTCAC
M00076	V$GATA2_01	0.817772	8489	(-)	NNNGATRNNN	TTTTATCTCA

M00077	V$GATA3_01	0.895879	8489	(-)	NNGATARNG	TTTTATCTC
M00126	V$GATA1_02	0.915625	8551	(+)	NNNNNGATANKGNN	GTCCAGATATGGGA
M00127	V$GATA1_03	0.830475	8551	(+)	RNSNNGATAANNGN	GTCCAGATATGGGA
M00128	V$GATA1_04	0.907169	8552	(+)	NNCWGATARNNNN	TCCAGATATGGGA
M00075	V$GATA1_01	0.924482	8553	(+)	SNNGATNNNNN	CCAGATATGG
M00076	V$GATA2_01	0.930988	8553	(+)	NNNGATRNNN	CCAGATATGG
M00077	V$GATA3_01	0.916704	8554	(+)	NNGATARNG	CAGATATGG
M00076	V$GATA2_01	0.810555	8555	(-)	NNNGATRNNN	AGATATGGGA
M00203	V$GATA_C	0.870767	8555	(+)	NGATAAGNMNN	AGATATGGGAC
M00127	V$GATA1_03	0.785154	8571	(+)	RNSNNGATAANNGN	AGGGGAATAGCATA
M00126	V$GATA1_02	0.792188	8587	(+)	NNNNNGATANKGNN	GTTTTGATATTCTT
M00075	V$GATA1_01	0.78924	8589	(+)	SNNGATNNNNN	TTTGATATTC
M00076	V$GATA2_01	0.797925	8589	(+)	NNNGATRNNN	TTTGATATTC
M00126	V$GATA1_02	0.79375	8599	(+)	NNNNNGATANKGNN	TTAAAGATATACAT
M00128	V$GATA1_04	0.842525	8600	(+)	NNCWGATARNNNN	TAAAGATATACAT
M00076	V$GATA2_01	0.818223	8601	(+)	NNNGATRNNN	AAAGATATAC
M00203	V$GATA_C	0.852439	8603	(+)	NGATAAGNMNN	AGATATACATC
M00075	V$GATA1_01	0.771471	8607	(-)	SNNGATNNNNN	ATACATCTTT
M00076	V$GATA2_01	0.793415	8607	(-)	NNNGATRNNN	ATACATCTTT
M00127	V$GATA1_03	0.807202	8616	(-)	RNSNNGATAANNGN	TTTGTGATCATGAT
M00075	V$GATA1_01	0.810464	8618	(+)	SNNGATNNNNN	TGTGATCATG
M00075	V$GATA1_01	0.795656	8618	(-)	SNNGATNNNNN	TGTGATCATG
M00076	V$GATA2_01	0.824989	8618	(+)	NNNGATRNNN	TGTGATCATG
M00076	V$GATA2_01	0.801985	8618	(-)	NNNGATRNNN	TGTGATCATG
M00075	V$GATA1_01	0.870188	8624	(+)	SNNGATNNNNN	CATGATTCAG
M00076	V$GATA2_01	0.811908	8624	(+)	NNNGATRNNN	CATGATTCAG
M00126	V$GATA1_02	0.827813	8662	(+)	NNNNNGATANKGNN	GAGCCGATACTTGG
M00127	V$GATA1_03	0.792994	8662	(+)	RNSNNGATAANNGN	GAGCCGATACTTGG
M00075	V$GATA1_01	0.932379	8664	(+)	SNNGATNNNNN	GCCGATACTT
M00076	V$GATA2_01	0.925124	8664	(+)	NNNGATRNNN	GCCGATACTT
M00126	V$GATA1_02	0.914062	8698	(+)	NNNNNGATANKGNN	TTTAAGATAAGGCA
M00128	V$GATA1_04	0.941176	8699	(+)	NNCWGATARNNNN	TTAAGATAAGGCA
M00075	V$GATA1_01	0.824778	8700	(+)	SNNGATNNNNN	TAAGATAAGG

M00076	V$GATA2_01	0.898963	8700	(+)	NNNGATRNNN	TAAGATAAGG
M00077	V$GATA3_01	0.954364	8701	(+)	NNGATARNG	AAGATAAGG
M00203	V$GATA_C	0.975148	8702	(+)	NGATAAGNMNN	AGATAAGGCAC
M00076	V$GATA2_01	0.831304	8712	(+)	NNNGATRNNN	CAGCATGGTG
M00076	V$GATA2_01	0.788453	8712	(-)	NNNGATRNNN	CAGCATGGTG
M00075	V$GATA1_01	0.795656	8731	(+)	SNNGATNNNN	TAAGATTTCT
M00076	V$GATA2_01	0.848895	8731	(+)	NNNGATRNNN	TAAGATTTCT
M00075	V$GATA1_01	0.801579	8776	(+)	SNNGATNNNN	TGAGATGAAG
M00076	V$GATA2_01	0.832206	8776	(+)	NNNGATRNNN	TGAGATGAAG
M00077	V$GATA3_01	0.877271	8777	(+)	NNGATARNG	GAGATGAAG
M00076	V$GATA2_01	0.79161	8847	(-)	NNNGATRNNN	AAATATGGCA
M00077	V$GATA3_01	0.82809	8861	(-)	NNGATARNG	TTCAATCCT
M00075	V$GATA1_01	0.846989	8869	(-)	SNNGATNNNN	TTGCATCATG
M00076	V$GATA2_01	0.805142	8869	(-)	NNNGATRNNN	TTGCATCATG
M00126	V$GATA1_02	0.803125	8881	(+)	NNNNNGATANKGNN	ACTAAGATAATTTT
M00127	V$GATA1_03	0.789809	8881	(+)	RNSNNGATAANNGN	ACTAAGATAATTTT
M00075	V$GATA1_01	0.801086	8883	(+)	SNNGATNNNN	TAAGATAATT
M00076	V$GATA2_01	0.879567	8883	(+)	NNNGATRNNN	TAAGATAATT
M00077	V$GATA3_01	0.866637	8884	(+)	NNGATARNG	AAGATAATT
M00203	V$GATA_C	0.861137	8885	(+)	NGATAAGNMNN	AGATAATTTTT
M00076	V$GATA2_01	0.781236	8953	(-)	NNNGATRNNN	TTTCATCTTA
M00077	V$GATA3_01	0.824989	8953	(-)	NNGATARNG	TTTCATCTT
M00203	V$GATA_C	0.832246	9037	(-)	NGATAAGNMNN	TGGAAATATCA
M00126	V$GATA1_02	0.790312	9038	(-)	NNNNNGATANKGNN	GGAAATATCATGAA
M00128	V$GATA1_04	0.818934	9038	(-)	NNCWGATARNNNN	GGAAATATCATGA
M00075	V$GATA1_01	0.90079	9040	(-)	SNNGATNNNN	AAATATCATG
M00076	V$GATA2_01	0.892197	9040	(-)	NNNGATRNNN	AAATATCATG
M00203	V$GATA_C	0.861448	9048	(-)	NGATAAGNMNN	TGAACATATCA
M00076	V$GATA2_01	0.790257	9049	(+)	NNNGATRNNN	GAACATATCA
M00075	V$GATA1_01	0.816387	9051	(-)	SNNGATNNNN	ACATATCAAA
M00076	V$GATA2_01	0.807848	9051	(-)	NNNGATRNNN	ACATATCAAA
M00075	V$GATA1_01	0.826752	9087	(+)	SNNGATNNNN	TGTGATTATT
M00076	V$GATA2_01	0.811908	9087	(+)	NNNGATRNNN	TGTGATTATT

M00077	V$GATA3_01	0.826761	9088	(+)	NNGATARNG	GTGATTATT
M00075	V$GATA1_01	0.772458	9105	(-)	SNNGATNNNN	AAGAATCTAT
M00223	V$STAT_01	0.802622	161	(-)	TTCCCRKAA	TTAAGATAA
M00223	V$STAT_01	0.853542	251	(-)	TTCCCRKAA	TTTTAGGAA
M00223	V$STAT_01	0.792538	544	(+)	TTCCCRKAA	TTATCAAAA
M00223	V$STAT_01	0.938997	554	(+)	TTCCCRKAA	TTCTCAGAA
M00223	V$STAT_01	0.902445	554	(-)	TTCCCRKAA	TTCTCAGAA
M00224	V$STAT1_01	0.729294	689	(+)	NNNSANTTCCGGGAANTGNSN	AGAGAATGCCAGGAAACAAAA
M00224	V$STAT1_01	0.725671	689	(-)	NNNSANTTCCGGGAANTGNSN	AGAGAATGCCAGGAAACAAAA
M00225	V$STAT3_01	0.746416	689	(+)	NGNNATTTCCSGGAARTGNNN	AGAGAATGCCAGGAAACAAAA
M00225	V$STAT3_01	0.758726	689	(-)	NGNNATTTCCSGGAARTGNNN	AGAGAATGCCAGGAAACAAAA
M00223	V$STAT_01	0.91152	695	(-)	TTCCCRKAA	TGCCAGGAA
M00223	V$STAT_01	0.839677	845	(-)	TTCCCRKAA	TTCTAGGTA
M00223	V$STAT_01	0.816738	875	(-)	TTCCCRKAA	TGACAATAA
M00223	V$STAT_01	0.799597	889	(+)	TTCCCRKAA	TTTTTAGAA
M00223	V$STAT_01	0.802622	965	(+)	TTCCCRKAA	TTTTCCTAA
M00223	V$STAT_01	0.826821	1038	(-)	TTCCCRKAA	TTTCAAGAA
M00223	V$STAT_01	0.792538	1048	(-)	TTCCCRKAA	TTATAAAAA
M00223	V$STAT_01	0.812957	1314	(-)	TTCCCRKAA	GTCCAAGAA
M00224	V$STAT1_01	0.77507	1370	(+)	NNNSANTTCCGGGAANTGNSN	GAAGTCTTACTGGAAATAAAA
M00225	V$STAT3_01	0.77147	1370	(-)	NGNNATTTCCSGGAARTGNNN	GAAGTCTTACTGGAAATAAAA
M00223	V$STAT_01	0.929418	1376	(+)	TTCCCRKAA	TTACTGGAA
M00223	V$STAT_01	0.887572	1376	(-)	TTCCCRKAA	TTACTGGAA
M00224	V$STAT1_01	0.733575	1545	(-)	NNNSANTTCCGGGAANTGNSN	GCTCTAATCCCAGAATCTAAG
M00223	V$STAT_01	0.866398	1551	(+)	TTCCCRKAA	ATCCCAGAA
M00223	V$STAT_01	0.803126	1551	(-)	TTCCCRKAA	ATCCCAGAA
M00223	V$STAT_01	0.853542	1628	(+)	TTCCCRKAA	TTTCTGTAA
M00223	V$STAT_01	0.816738	1661	(-)	TTCCCRKAA	TTCCAAAAA
M00223	V$STAT_01	0.826317	1723	(+)	TTCCCRKAA	TTAGCAGAA
M00223	V$STAT_01	0.87875	1723	(-)	TTCCCRKAA	TTAGCAGAA
M00225	V$STAT3_01	0.714699	1754	(-)	NGNNATTTCCSGGAARTGNNN	AAACATTAACTTGAAATGTAA
M00223	V$STAT_01	0.790522	1785	(-)	TTCCCRKAA	TAATAGTAA
M00223	V$STAT_01	0.805899	1964	(+)	TTCCCRKAA	TCCTTGTAA

M00224	V$STAT1_01	0.73127	2035	(+)	NNNSANTTCCGGGAANTGNSN	TCCCATTCACAGGAATCGAGA
M00224	V$STAT1_01	0.740326	2035	(-)	NNNSANTTCCGGGAANTGNSN	TCCCATTCACAGGAATCGAGA
M00225	V$STAT3_01	0.743085	2035	(-)	NGNNATTTCCSGGAARTGNNN	TCCCATTCACAGGAATCGAGA
M00223	V$STAT_01	0.851525	2041	(-)	TTCCCRKAA	TCACAGGAA
M00223	V$STAT_01	0.850517	2230	(-)	TTCCCRKAA	TGCTAAGAA
M00223	V$STAT_01	0.805899	2491	(+)	TTCCCRKAA	TTACTGTAG
M00223	V$STAT_01	0.861356	2586	(-)	TTCCCRKAA	TTATAATAA
M00223	V$STAT_01	0.799849	2652	(+)	TTCCCRKAA	TTAAGGTAA
M00223	V$STAT_01	0.846483	2652	(-)	TTCCCRKAA	TTAAGGTAA
M00223	V$STAT_01	0.805899	2902	(+)	TTCCCRKAA	TAACTGTAA
M00223	V$STAT_01	0.826317	2933	(+)	TTCCCRKAA	TTAGCAGAA
M00223	V$STAT_01	0.87875	2933	(-)	TTCCCRKAA	TTAGCAGAA
M00224	V$STAT1_01	0.774741	3028	(+)	NNNSANTTCCGGGAANTGNSN	GGCAATTTCCCAGATTTAGAG
M00225	V$STAT3_01	0.738885	3028	(+)	NGNNATTTCCSGGAARTGNNN	GGCAATTTCCCAGATTTAGAG
M00225	V$STAT3_01	0.737437	3028	(-)	NGNNATTTCCSGGAARTGNNN	GGCAATTTCCCAGATTTAGAG
M00223	V$STAT_01	0.866398	3034	(+)	TTCCCRKAA	TTCCCAGAT
M00223	V$STAT_01	0.803126	3034	(-)	TTCCCRKAA	TTCCCAGAT
M00223	V$STAT_01	0.819763	3043	(-)	TTCCCRKAA	TTAGAGCAA
M00223	V$STAT_01	0.80968	3077	(-)	TTCCCRKAA	TTACATTAA
M00225	V$STAT3_01	0.712238	3102	(-)	NGNNATTTCCSGGAARTGNNN	TACTCTTGCCTGGAAAACCCC
M00223	V$STAT_01	0.856819	3108	(+)	TTCCCRKAA	TGCCTGGAA
M00223	V$STAT_01	0.832871	3108	(-)	TTCCCRKAA	TGCCTGGAA
M00223	V$STAT_01	0.863625	3530	(-)	TTCCCRKAA	TTAAAGGAA
M00223	V$STAT_01	0.843711	3561	(+)	TTCCCRKAA	TTCTAGTAA
M00223	V$STAT_01	0.912276	3561	(-)	TTCCCRKAA	TTCTAGTAA
M00223	V$STAT_01	0.885556	3586	(+)	TTCCCRKAA	TTATTGGAA
M00223	V$STAT_01	0.870431	3586	(-)	TTCCCRKAA	TTATTGGAA
M00223	V$STAT_01	0.795815	3814	(-)	TTCCCRKAA	ATCTAGTAA
M00224	V$STAT1_01	0.804709	4052	(-)	NNNSANTTCCGGGAANTGNSN	CCCCCATTCCTGGGATTCTCC
M00225	V$STAT3_01	0.716003	4052	(-)	NGNNATTTCCSGGAARTGNNN	CCCCCATTCCTGGGATTCTCC
M00223	V$STAT_01	0.858583	4058	(+)	TTCCCRKAA	TTCCTGGGA
M00223	V$STAT_01	0.832871	4212	(+)	TTCCCRKAA	TTCCAGGCA
M00223	V$STAT_01	0.856819	4212	(-)	TTCCCRKAA	TTCCAGGCA

M00223	V$STAT_01	0.853542	4248	(+)	TTCCCRKAA	TTCTCTGAA
M00223	V$STAT_01	0.833627	4248	(-)	TTCCCRKAA	TTCTCTGAA
M00223	V$STAT_01	0.832619	4262	(-)	TTCCCRKAA	TTATAGGAG
M00223	V$STAT_01	0.905218	4280	(+)	TTCCCRKAA	TTCTTATAA
M00223	V$STAT_01	0.812957	4361	(+)	TTCCCRKAA	TCCTTGGAA
M00223	V$STAT_01	0.793043	4364	(-)	TTCCCRKAA	TTGGAAGAA
M00225	V$STAT3_01	0.73092	4450	(+)	NGNNATTTCCSGGAARTGNNN	ATGGTTTTCCCAGTAGTCATG
M00223	V$STAT_01	0.868162	4456	(+)	TTCCCRKAA	TTCCCAGTA
M00223	V$STAT_01	0.803126	4456	(-)	TTCCCRKAA	TTCCCAGTA
M00223	V$STAT_01	0.804638	4704	(-)	TTCCCRKAA	TGCTGGGAG
M00224	V$STAT1_01	0.766178	4793	(+)	NNNSANTTCCGGGAANTGNSN	AGTGAACTCCGGGAGTTGGTG
M00224	V$STAT1_01	0.748065	4793	(-)	NNNSANTTCCGGGAANTGNSN	AGTGAACTCCGGGAGTTGGTG
M00225	V$STAT3_01	0.768139	4793	(+)	NGNNATTTCCSGGAARTGNNN	AGTGAACTCCGGGAGTTGGTG
M00225	V$STAT3_01	0.717451	4793	(-)	NGNNATTTCCSGGAARTGNNN	AGTGAACTCCGGGAGTTGGTG
M00223	V$STAT_01	0.802622	4996	(+)	TTCCCRKAA	TTTTCCTAA
M00223	V$STAT_01	0.800605	5002	(-)	TTCCCRKAA	TAAGGAGAA
M00223	V$STAT_01	0.832871	5118	(+)	TTCCCRKAA	TTCCAGGCA
M00223	V$STAT_01	0.856819	5118	(-)	TTCCCRKAA	TTCCAGGCA
M00223	V$STAT_01	0.792538	5563	(+)	TTCCCRKAA	TTTTTATAA
M00223	V$STAT_01	0.822536	5602	(+)	TTCCCRKAA	TAACCAGAA
M00223	V$STAT_01	0.797832	5602	(-)	TTCCCRKAA	TAACCAGAA
M00223	V$STAT_01	0.8364	5674	(-)	TTCCCRKAA	TTTTGAGAA
M00223	V$STAT_01	0.861356	5712	(-)	TTCCCRKAA	TTATAATAA
M00223	V$STAT_01	0.795815	5730	(-)	TTCCCRKAA	GTCTAAGAA
M00223	V$STAT_01	0.844719	5952	(+)	TTCCCRKAA	TTATTCTAA
M00223	V$STAT_01	0.799597	6193	(-)	TTCCCRKAA	TTCTAAAAA
M00223	V$STAT_01	0.826317	6284	(+)	TTCCCRKAA	TTAGCAGAA
M00223	V$STAT_01	0.87875	6284	(-)	TTCCCRKAA	TTAGCAGAA
M00223	V$STAT_01	0.799849	6348	(+)	TTCCCRKAA	TTGTGGTAA
M00223	V$STAT_01	0.8364	6348	(-)	TTCCCRKAA	TTGTGGTAA
M00223	V$STAT_01	0.816738	6573	(-)	TTCCCRKAA	TTCCATTAA
M00223	V$STAT_01	0.8364	6643	(-)	TTCCCRKAA	TTATATGAA
M00223	V$STAT_01	0.817242	6786	(-)	TTCCCRKAA	TAATGAGAA

M00224	V$STAT1_01	0.741808	6874	(+)	NNNSANTTCCGGGAANTGNSN	TTTCTCTTCACTGAATTATGT
M00225	V$STAT3_01	0.713396	6874	(-)	NGNNATTTCCSGGAARTGNNN	TTTCTCTTCACTGAATTATGT
M00224	V$STAT1_01	0.722542	6965	(-)	NNNSANTTCCGGGAANTGNSN	CCTTCTTTCCAGGATGAACTC
M00223	V$STAT_01	0.856819	6971	(-)	TTCCCRKAA	TTCCAGGAT
M00223	V$STAT_01	0.793043	7080	(+)	TTCCCRKAA	TTACTCAAA
M00224	V$STAT1_01	0.732752	7175	(-)	NNNSANTTCCGGGAANTGNSN	ATGCCCTTCCCTAAATATCCA
M00223	V$STAT_01	0.794807	7181	(+)	TTCCCRKAA	TTCCCTAAA
M00223	V$STAT_01	0.826821	7206	(+)	TTCCCRKAA	TTACTGAAA
M00223	V$STAT_01	0.836904	7302	(+)	TTCCCRKAA	TTCCTCCAA
M00225	V$STAT3_01	0.736568	7363	(+)	NGNNATTTCCSGGAARTGNNN	GCCTGTTCCCCAGAAAGCAGT
M00223	V$STAT_01	0.866398	7369	(+)	TTCCCRKAA	TCCCCAGAA
M00223	V$STAT_01	0.80489	7369	(-)	TTCCCRKAA	TCCCCAGAA
M00224	V$STAT1_01	0.741973	7458	(-)	NNNSANTTCCGGGAANTGNSN	TCCCTATTCTTGTAAGTCTAA
M00223	V$STAT_01	0.922359	7464	(+)	TTCCCRKAA	TTCTTGTAA
M00223	V$STAT_01	0.833627	7464	(-)	TTCCCRKAA	TTCTTGTAA
M00223	V$STAT_01	0.793043	7521	(-)	TTCCCRKAA	TTTGAGTAA
M00223	V$STAT_01	0.877237	7594	(+)	TTCCCRKAA	TTCTCAGCA
M00223	V$STAT_01	0.819763	7772	(-)	TTCCCRKAA	TTAGATGAA
M00223	V$STAT_01	0.826569	7791	(+)	TTCCCRKAA	TTACAATAA
M00223	V$STAT_01	0.878498	7791	(-)	TTCCCRKAA	TTACAATAA
M00224	V$STAT1_01	0.736539	8090	(-)	NNNSANTTCCGGGAANTGNSN	CCTCACTTTTGGTAAGCTTTA
M00223	V$STAT_01	0.799849	8096	(+)	TTCCCRKAA	TTTTGGTAA
M00223	V$STAT_01	0.8364	8096	(-)	TTCCCRKAA	TTTTGGTAA
M00223	V$STAT_01	0.79884	8108	(-)	TTCCCRKAA	TTAGGAGAT
M00223	V$STAT_01	0.815982	8116	(-)	TTCCCRKAA	TTAGAGGCA
M00224	V$STAT1_01	0.732752	8159	(+)	NNNSANTTCCGGGAANTGNSN	TTAATTTTCCTGGATAAGACC
M00223	V$STAT_01	0.856819	8165	(+)	TTCCCRKAA	TTCCTGGAT
M00223	V$STAT_01	0.8364	8377	(-)	TTCCCRKAA	TTATAGAAA
M00223	V$STAT_01	0.815982	8678	(-)	TTCCCRKAA	CTAGAGGAA
M00223	V$STAT_01	0.792538	8687	(+)	TTCCCRKAA	TTTTTATAA
M00223	V$STAT_01	0.802622	8699	(-)	TTCCCRKAA	TTAAGATAA
M00223	V$STAT_01	0.80968	8722	(+)	TTCCCRKAA	TTTTTGTAA
M00223	V$STAT_01	0.819259	8742	(-)	TTCCCRKAA	TTATGAAAA

M00225	V$STAT3_01	0.711224	8818	(+)	NGNNATTTCCSGGAARTGNNN	CTCTATTCCACAGAATTGACT
M00225	V$STAT3_01	0.712527	8818	(-)	NGNNATTTCCSGGAARTGNNN	CTCTATTCCACAGAATTGACT
M00223	V$STAT_01	0.819259	9012	(+)	TTCCCRKAA	TTAGCATAA
M00223	V$STAT_01	0.834888	9012	(-)	TTCCCRKAA	TTAGCATAA
M00224	V$STAT1_01	0.776881	9071	(+)	NNNSANTTCCGGGAANTGNSN	AAATAATTTCTGGAATTGTGA
M00224	V$STAT1_01	0.733575	9071	(-)	NNNSANTTCCGGGAANTGNSN	AAATAATTTCTGGAATTGTGA
M00225	V$STAT3_01	0.738306	9071	(-)	NGNNATTTCCSGGAARTGNNN	AAATAATTTCTGGAATTGTGA
M00223	V$STAT_01	0.8606	9077	(+)	TTCCCRKAA	TTTCTGGAA
M00223	V$STAT_01	0.792034	9077	(-)	TTCCCRKAA	TTTCTGGAA
M00162	V$OCT1_06	0.816016	44	(-)	CWNAWTKWSATRYN	AAAAGGTAAGAATC
M00162	V$OCT1_06	0.890625	52	(-)	CWNAWTKWSATRYN	AGAATCTCAGATAT
M00162	V$OCT1_06	0.834375	53	(+)	CWNAWTKWSATRYN	GAATCTCAGATATA
M00138	V$OCT1_04	0.793601	60	(-)	NNNNNNNWATGCAAATNNNWNNW	AGATATAATTTCAGTG-TATCTGC
M00137	V$OCT1_03	0.854998	86	(-)	NNNRTAATNANNN	TCATCTTTATTTT
M00138	V$OCT1_04	0.794437	96	(+)	NNNNNNNWATGCAAATNNNWNNW	TTT-GGACTAGGTTAAAATGTAGA
M00136	V$OCT1_02	0.806443	116	(-)	NNGAATATKCANNNN	AGAAAGAATATAATT
M00162	V$OCT1_06	0.842578	120	(-)	CWNAWTKWSATRYN	AGAATATAATTGCT
M00137	V$OCT1_03	0.846701	123	(-)	NNNRTAATNANNN	ATATAATTGCTTA
M00138	V$OCT1_04	0.791301	136	(+)	NNNNNNNWATGCAAATNNNWNNW	AAATAGATCTTAAAAA-TAAGGGT
M00135	V$OCT1_01	0.736551	179	(+)	NNNNWTATGCAAATNTNNN	AATTTTCAGCAAATATGTT
M00138	V$OCT1_04	0.853827	185	(+)	NNNNNNNWATGCAAATNNNWNNW	CAGCAAATATGTTAAAAAA-TAGA
M00135	V$OCT1_01	0.769363	187	(+)	NNNNWTATGCAAATNTNNN	GCAAATATGTTAAAAAATA
M00138	V$OCT1_04	0.791301	207	(+)	NNNNNNNWATGCAAATNNNWNNW	AAGTGACTATAAAGATTTGA-TAA
M00137	V$OCT1_03	0.842355	212	(+)	NNNRTAATNANNN	ACTATAAAGATTT
M00138	V$OCT1_04	0.812003	219	(-)	NNNNNNNWATGCAAATNNNWNNW	AGATTTGA-TAAAAATTATAGTGA
M00137	V$OCT1_03	0.856183	223	(+)	NNNRTAATNANNN	TTGATAAAAATTA
M00162	V$OCT1_06	0.80625	229	(-)	CWNAWTKWSATRYN	AAAATTATAGTGAC
M00138	V$OCT1_04	0.788373	234	(+)	NNNNNNNWATGCAAATNNNWNNW	TATAGTGACTG-CAAATGTTTTAG
M00135	V$OCT1_01	0.747043	236	(+)	NNNNWTATGCAAATNTNNN	TAGTGACTGCAAATGTTTT
M00162	V$OCT1_06	0.821484	239	(-)	CWNAWTKWSATRYN	TGACTGCAAATGTT
M00138	V$OCT1_04	0.837307	247	(+)	NNNNNNNWATGCAAATNNNWNNW	AATGTTTTAGGAATATAATAA-GA
M00136	V$OCT1_02	0.777477	252	(-)	NNGAATATKCANNNN	TTTAGGAATATAATA
M00137	V$OCT1_03	0.899644	258	(+)	NNNRTAATNANNN	AATATAATAAGAT

M00162	V$OCT1_06	0.832812	260	(+)	CWNAWTKWSATRYN	TATAATAAGATGTA
M00162	V$OCT1_06	0.903516	320	(-)	CWNAWTKWSATRYN	TGTATTAAAAGATC
M00138	V$OCT1_04	0.80092	337	(-)	NNNNNNNWATGCAAATNNNWNNW	TCTTGAATTAAATATTTTCAATT
M00162	V$OCT1_06	0.868359	339	(+)	CWNAWTKWSATRYN	TTGAATTAAATATT
M00162	V$OCT1_06	0.898437	340	(-)	CWNAWTKWSATRYN	TGAATTAAATATTT
M00136	V$OCT1_02	0.803194	341	(-)	NNGAATATKCANNNN	GAATTAAATATTTTC
M00138	V$OCT1_04	0.783982	343	(-)	NNNNNNNWATGCAAATNNNWNNW	ATTAAATATTTTCAATTTGATTA
M00138	V$OCT1_04	0.810958	344	(-)	NNNNNNNWATGCAAATNNNWNNW	TTAAATATTTTCAATTTGATTAA
M00162	V$OCT1_06	0.847266	347	(+)	CWNAWTKWSATRYN	AATATTTTCAATTT
M00162	V$OCT1_06	0.800781	348	(+)	CWNAWTKWSATRYN	ATATTTTCAATTTG
M00138	V$OCT1_04	0.800502	350	(-)	NNNNNNNWATGCAAATNNNWNNW	ATTTTCAATTTGATTAAACCTAC
M00135	V$OCT1_01	0.740939	352	(-)	NNNNWTATGCAAATNTNNN	TTTCAATTTGATTAAACCT
M00248	V$OCT1_07	0.814501	356	(-)	TNTATGNTAATT	AATTTGATTAAA
M00137	V$OCT1_03	0.856578	357	(-)	NNNRTAATNANNN	ATTTGATTAAACC
M00162	V$OCT1_06	0.821484	390	(+)	CWNAWTKWSATRYN	GCACATTTAATTTA
M00162	V$OCT1_06	0.932422	391	(-)	CWNAWTKWSATRYN	CACATTTAATTTAT
M00138	V$OCT1_04	0.870765	407	(-)	NNNNNNNWATGCAAATNNNWNNW	TATGGGGATTTGAATAATTATTA
M00135	V$OCT1_01	0.85311	409	(-)	NNNNWTATGCAAATNTNNN	TGGGGATTTGAATAATTAT
M00161	V$OCT1_05	0.872157	411	(+)	MKNATTTGCATAYY	GGGATTTGAATAAT
M00162	V$OCT1_06	0.80625	411	(+)	CWNAWTKWSATRYN	GGGATTTGAATAAT
M00195	V$OCT1_Q6	0.856441	411	(-)	NNNNATGCAAATNAN	GGGATTTGAATAATT
M00136	V$OCT1_02	0.860585	413	(-)	NNGAATATKCANNNN	GATTTGAATAATTAT
M00248	V$OCT1_07	0.795209	413	(-)	TNTATGNTAATT	GATTTGAATAAT
M00137	V$OCT1_03	0.898854	417	(+)	NNNRTAATNANNN	TGAATAATTATTA
M00137	V$OCT1_03	0.875148	418	(-)	NNNRTAATNANNN	GAATAATTATTAC
M00137	V$OCT1_03	0.88226	421	(-)	NNNRTAATNANNN	TAATTATTACTGA
M00135	V$OCT1_01	0.778138	457	(-)	NNNNWTATGCAAATNTNNN	ATAGAGCTAGCATATTTAG
M00136	V$OCT1_02	0.846779	461	(-)	NNGAATATKCANNNN	AGCTAGCATATTTAG
M00136	V$OCT1_02	0.790471	464	(+)	NNGAATATKCANNNN	TAGCATATTTAGTCA
M00137	V$OCT1_03	0.867246	480	(+)	NNNRTAATNANNN	GAGATAAAGAGGG
M00138	V$OCT1_04	0.780008	494	(-)	NNNNNNNWATGCAAATNNNWNNW	TGTTAGGATATATATGCTATTTG
M00138	V$OCT1_04	0.786073	499	(+)	NNNNNNNWATGCAAATNNNWNNW	GGATATATATGCTATTTGAAAGG
M00135	V$OCT1_01	0.820679	501	(+)	NNNNWTATGCAAATNTNNN	ATATATATGCTATTTGAAA

M00136	V$OCT1_02	0.802653	501	(+)	NNGAATATKCANNNN	ATATATATGCTATTT
M00248	V$OCT1_07	0.89718	504	(+)	TNTATGNTAATT	TATATGCTATTT
M00162	V$OCT1_06	0.847266	510	(+)	CWNAWTKWSATRYN	CTATTTGAAAGGTA
M00162	V$OCT1_06	0.813281	511	(-)	CWNAWTKWSATRYN	TATTTGAAAGGTAT
M00138	V$OCT1_04	0.811167	515	(+)	NNNNNNNWATGCAAATNNNWNNW	TGAAAGGTATTTATAAAAGAA-GA
M00162	V$OCT1_06	0.826562	520	(-)	CWNAWTKWSATRYN	GGTATTTATAAAAG
M00136	V$OCT1_02	0.792366	536	(+)	NNGAATATKCANNNN	GAGTATATTTATCAA
M00137	V$OCT1_03	0.84275	538	(-)	NNNRTAATNANNN	GTATATTTATCAA
M00138	V$OCT1_04	0.818904	538	(+)	NNNNNNNWATGCAAATNNNWNNW	GTATATTTATCAAAATTTCTCAG
M00138	V$OCT1_04	0.781054	539	(-)	NNNNNNNWATGCAAATNNNWNNW	TATATTTATCAAAATTTCTCAGA
M00135	V$OCT1_01	0.783861	540	(+)	NNNNWTATGCAAATNTNNN	ATATTTATCAAAATTTCTC
M00162	V$OCT1_06	0.865625	543	(-)	CWNAWTKWSATRYN	TTTATCAAAATTTC
M00248	V$OCT1_07	0.795845	543	(+)	TNTATGNTAATT	TTTATCAAAATT
M00138	V$OCT1_04	0.780217	556	(+)	NNNNNNNWATGCAAATNNNWNNW	CTCAGAACATCCAAATTTCAAGT
M00135	V$OCT1_01	0.819725	558	(+)	NNNNWTATGCAAATNTNNN	CAGAACATCCAAATTTCAA
M00136	V$OCT1_02	0.796156	558	(+)	NNGAATATKCANNNN	CAGAACATCCAAATT
M00195	V$OCT1_Q6	0.822871	560	(+)	NNNNATGCAAATNAN	GAACATCCAAATTTC
M00162	V$OCT1_06	0.9375	561	(-)	CWNAWTKWSATRYN	AACATCCAAATTTC
M00248	V$OCT1_07	0.800933	561	(+)	TNTATGNTAATT	AACATCCAAATT
M00162	V$OCT1_06	0.825	566	(+)	CWNAWTKWSATRYN	CCAAATTTCAAGTT
M00162	V$OCT1_06	0.885547	567	(-)	CWNAWTKWSATRYN	CAAATTTCAAGTTT
M00138	V$OCT1_04	0.799247	571	(-)	NNNNNNNWATGCAAATNNNWNNW	TTTCAAGTTTATCATTTATCTTA
M00162	V$OCT1_06	0.821484	575	(+)	CWNAWTKWSATRYN	AAGTTTATCATTTA
M00138	V$OCT1_04	0.786909	582	(+)	NNNNNNNWATGCAAATNNNWNNW	TCATTTATCTTATAATATTTCAA
M00138	V$OCT1_04	0.82476	591	(+)	NNNNNNNWATGCAAATNNNWNNW	TTATAATATTTCAAAAA-TATTAA
M00138	V$OCT1_04	0.789419	591	(-)	NNNNNNNWATGCAAATNNNWNNW	TTATAATATTTCAAAAA-TATTAA
M00162	V$OCT1_06	0.903516	595	(-)	CWNAWTKWSATRYN	AATATTTCAAAAAT
M00162	V$OCT1_06	0.818359	597	(-)	CWNAWTKWSATRYN	TATTTCAAAAATAT
M00138	V$OCT1_04	0.872438	601	(+)	NNNNNNNWATGCAAATNNNWNNW	TCAAAAATATTAAAATAGATA-CA
M00138	V$OCT1_04	0.801129	602	(-)	NNNNNNNWATGCAAATNNNWNNW	CAAAAATATTAAAATAGATA-CAT
M00135	V$OCT1_01	0.742655	603	(+)	NNNNWTATGCAAATNTNNN	AAAAATATTAAAATAGATA
M00136	V$OCT1_02	0.805901	603	(+)	NNGAATATKCANNNN	AAAAATATTAAAATA
M00248	V$OCT1_07	0.836549	608	(-)	TNTATGNTAATT	TATTAAAATAGA

M00162	V$OCT1_06	0.803906	610	(+)	CWNAWTKWSATRYN	TTAAAATAGATACA
M00162	V$OCT1_06	0.911328	619	(+)	CWNAWTKWSATRYN	ATACATGAAATACA
M00162	V$OCT1_06	0.945312	620	(-)	CWNAWTKWSATRYN	TACATGAAATACAG
M00162	V$OCT1_06	0.800781	637	(-)	CWNAWTKWSATRYN	TAAATTAAAGAGAA
M00248	V$OCT1_07	0.817257	651	(+)	TNTATGNTAATT	AGTATTTTACTT
M00137	V$OCT1_03	0.856578	661	(+)	NNNRTAATNANNN	TTGGTAAAAAAAA
M00162	V$OCT1_06	0.814062	672	(-)	CWNAWTKWSATRYN	AAAATTCTAGGTTG
M00162	V$OCT1_06	0.821484	691	(-)	CWNAWTKWSATRYN	AGAATGCCAGGAAA
M00162	V$OCT1_06	0.829297	709	(+)	CWNAWTKWSATRYN	AACAATGAAAAATG
M00137	V$OCT1_03	0.908337	756	(+)	NNNRTAATNANNN	TCAGTAATGAAAT
M00162	V$OCT1_06	0.842578	758	(+)	CWNAWTKWSATRYN	AGTAATGAAATGGC
M00135	V$OCT1_01	0.770126	773	(-)	NNNNWTATGCAAATNTNNN	TAAAAATTGGTATATAAAA
M00162	V$OCT1_06	0.860547	775	(+)	CWNAWTKWSATRYN	AAAATTGGTATATA
M00138	V$OCT1_04	0.802593	776	(+)	NNNNNNNWATGCAAATNNNWNNW	AAATTGGTATA-TAAAATGCTAGT
M00248	V$OCT1_07	0.806021	777	(-)	TNTATGNTAATT	AATTGGTATATA
M00162	V$OCT1_06	0.842187	782	(+)	CWNAWTKWSATRYN	GTATATAAAATGCT
M00138	V$OCT1_04	0.816186	783	(+)	NNNNNNNWATGCAAATNNNWNNW	TATATAAAATGCTAG-TTATAAAA
M00135	V$OCT1_01	0.752385	785	(+)	NNNNWTATGCAAATNTNNN	TATAAAATGCTAGTTATAA
M00136	V$OCT1_02	0.774499	785	(+)	NNGAATATKCANNNN	TATAAAATGCTAGTT
M00161	V$OCT1_05	0.882451	788	(-)	MKNATTTGCATAYY	AAAATGCTAGTTAT
M00162	V$OCT1_06	0.826953	788	(-)	CWNAWTKWSATRYN	AAAATGCTAGTTAT
M00248	V$OCT1_07	0.823829	788	(+)	TNTATGNTAATT	AAAATGCTAGTT
M00135	V$OCT1_01	0.737696	808	(+)	NNNNWTATGCAAATNTNNN	AACAAAATGCAATAATATC
M00136	V$OCT1_02	0.863021	808	(+)	NNGAATATKCANNNN	AACAAAATGCAATAA
M00162	V$OCT1_06	0.817578	810	(+)	CWNAWTKWSATRYN	CAAAATGCAATAAT
M00162	V$OCT1_06	0.85	811	(-)	CWNAWTKWSATRYN	AAAATGCAATAATA
M00136	V$OCT1_02	0.787223	818	(+)	NNGAATATKCANNNN	AATAATATCCTCCCT
M00162	V$OCT1_06	0.860547	831	(+)	CWNAWTKWSATRYN	CTACATGTAATGAA
M00195	V$OCT1_Q6	0.799672	831	(+)	NNNNATGCAAATNAN	CTACATGTAATGAAT
M00162	V$OCT1_06	0.939844	832	(-)	CWNAWTKWSATRYN	TACATGTAATGAAT
M00137	V$OCT1_03	0.909127	834	(+)	NNNRTAATNANNN	CATGTAATGAATT
M00135	V$OCT1_01	0.73636	850	(+)	NNNNWTATGCAAATNTNNN	GGTATTATGCTCTTTTTTG
M00136	V$OCT1_02	0.80157	850	(+)	NNGAATATKCANNNN	GGTATTATGCTCTTT

M00162	V$OCT1_06	0.805859	861	(+)	CWNAWTKWSATRYN	CTTTTTTGAAGTCT
M00138	V$OCT1_04	0.828315	872	(+)	NNNNNNNWATGCAAATNNNWNNW	TCTTGACAATAAAAATTTTTTTA
M00137	V$OCT1_03	0.84196	874	(+)	NNNRTAATNANNN	TTGACAATAAAAA
M00162	V$OCT1_06	0.808203	876	(-)	CWNAWTKWSATRYN	GACAATAAAAATTT
M00136	V$OCT1_02	0.779913	908	(-)	NNGAATATKCANNNN	ATCTTGAATAAAGTG
M00162	V$OCT1_06	0.816016	924	(-)	CWNAWTKWSATRYN	AACAATTAAGAATT
M00138	V$OCT1_04	0.826642	928	(-)	NNNNNNNWATGCAAATNNNWNNW	ATTAAGAATTAGCATCCATGA-GA
M00135	V$OCT1_01	0.801221	930	(-)	NNNNWTATGCAAATNTNNN	TAAGAATTAGCATCCATGA
M00195	V$OCT1_Q6	0.839793	932	(-)	NNNNATGCAAATNAN	AGAATTAGCATCCAT
M00248	V$OCT1_07	0.904812	934	(-)	TNTATGNTAATT	AATTAGCATCCA
M00135	V$OCT1_01	0.739985	950	(+)	NNNNWTATGCAAATNTNNN	AAAAATATAGAAAAATTTT
M00138	V$OCT1_04	0.782309	950	(+)	NNNNNNNWATGCAAATNNNWNNW	AAAAATATAGAAAAATTTTCC-TA
M00138	V$OCT1_04	0.854245	957	(-)	NNNNNNNWATGCAAATNNNWNNW	TAGAAAAATTTTCCTAATTTAGT
M00135	V$OCT1_01	0.772034	959	(-)	NNNNWTATGCAAATNTNNN	GAAAAATTTTCCTAATTTA
M00135	V$OCT1_01	0.754674	961	(+)	NNNNWTATGCAAATNTNNN	AAAATTTTCCTAATTTAGT
M00137	V$OCT1_03	0.84433	967	(+)	NNNRTAATNANNN	TTCCTAATTTAGT
M00135	V$OCT1_01	0.760206	974	(+)	NNNNWTATGCAAATNTNNN	TTTAGTTTGAAAATCTGGG
M00162	V$OCT1_06	0.873437	977	(-)	CWNAWTKWSATRYN	AGTTTGAAAATCTG
M00162	V$OCT1_06	0.800781	984	(+)	CWNAWTKWSATRYN	AAATCTGGGATTGA
M00138	V$OCT1_04	0.800293	1029	(-)	NNNNNNNWATGCAAATNNNWNNW	ACATTTTTTTTTCAAGAACTTAT
M00138	V$OCT1_04	0.821205	1042	(+)	NNNNNNNWATGCAAATNNNWNNW	AA-GAACTTATAAAAATGCAACAA
M00137	V$OCT1_03	0.841169	1047	(+)	NNNRTAATNANNN	CTTATAAAAATGC
M00138	V$OCT1_04	0.828524	1048	(+)	NNNNNNNWATGCAAATNNNWNNW	TTATAAAAATGCAACAAAACAAA
M00136	V$OCT1_02	0.845966	1050	(+)	NNGAATATKCANNNN	ATAAAAATGCAACAA
M00162	V$OCT1_06	0.898437	1069	(+)	CWNAWTKWSATRYN	AACCATTTAATACA
M00162	V$OCT1_06	0.829297	1070	(-)	CWNAWTKWSATRYN	ACCATTTAATACAT
M00162	V$OCT1_06	0.828906	1073	(+)	CWNAWTKWSATRYN	ATTTAATACATTTT
M00162	V$OCT1_06	0.808594	1090	(-)	CWNAWTKWSATRYN	CAAAATCAATAATG
M00138	V$OCT1_04	0.780008	1093	(+)	NNNNNNNWATGCAAATNNNWNNW	AATCAATAATGTATTTTATTTTA
M00162	V$OCT1_06	0.834375	1097	(+)	CWNAWTKWSATRYN	AATAATGTATTTTA
M00162	V$OCT1_06	0.821484	1101	(+)	CWNAWTKWSATRYN	ATGTATTTTATTTT
M00162	V$OCT1_06	0.812109	1102	(-)	CWNAWTKWSATRYN	TGTATTTTATTTTA
M00248	V$OCT1_07	0.836549	1102	(+)	TNTATGNTAATT	TGTATTTTATTT

M00162	V$OCT1_06	0.805859	1173	(-)	CWNAWTKWSATRYN	TAAATTACCAAGAG
M00248	V$OCT1_07	0.794361	1175	(-)	TNTATGNTAATT	AATTACCAAGAG
M00138	V$OCT1_04	0.800711	1195	(+)	NNNNNNNWATGCAAATNNNWNNW	ACAGTTCTATGTAGAGAAAA-TAA
M00135	V$OCT1_01	0.795498	1209	(-)	NNNNWTATGCAAATNTNNN	AGAAAATAAGCATAGTGTA
M00136	V$OCT1_02	0.77098	1210	(+)	NNGAATATKCANNNN	GAAAATAAGCATAGT
M00136	V$OCT1_02	0.775582	1213	(-)	NNGAATATKCANNNN	AATAAGCATAGTGTA
M00248	V$OCT1_07	0.882764	1213	(-)	TNTATGNTAATT	AATAAGCATAGT
M00137	V$OCT1_03	0.846306	1234	(-)	NNNRTAATNANNN	CTAAAATTATGTG
M00162	V$OCT1_06	0.833984	1238	(+)	CWNAWTKWSATRYN	AATTATGTGAGACA
M00162	V$OCT1_06	0.825391	1257	(-)	CWNAWTKWSATRYN	GAGATGACATTAGG
M00137	V$OCT1_03	0.865271	1260	(-)	NNNRTAATNANNN	ATGACATTAGGCA
M00162	V$OCT1_06	0.873437	1323	(-)	CWNAWTKWSATRYN	AACATCTCGATCAG
M00135	V$OCT1_01	0.785196	1348	(+)	NNNNWTATGCAAATNTNNN	GAAGAAATGCTAAAATGAA
M00162	V$OCT1_06	0.834766	1351	(-)	CWNAWTKWSATRYN	GAAATGCTAAAATG
M00162	V$OCT1_06	0.800781	1382	(-)	CWNAWTKWSATRYN	GAAATAAAAGATAT
M00138	V$OCT1_04	0.823714	1385	(-)	NNNNNNNWATGCAAATNNNWNNW	ATAAAAGATATGAATAAGA-CAAA
M00138	V$OCT1_04	0.825387	1386	(+)	NNNNNNNWATGCAAATNNNWNNW	TAAAAGATATGAATAAGA-CAAAA
M00135	V$OCT1_01	0.732163	1387	(-)	NNNNWTATGCAAATNTNNN	AAAAGATATGAATAAGACA
M00195	V$OCT1_Q6	0.799945	1388	(+)	NNNNATGCAAATNAN	AAAGATATGAATAAG
M00136	V$OCT1_02	0.785328	1391	(-)	NNGAATATKCANNNN	GATATGAATAAGACA
M00162	V$OCT1_06	0.800391	1391	(-)	CWNAWTKWSATRYN	GATATGAATAAGAC
M00162	V$OCT1_06	0.826172	1397	(-)	CWNAWTKWSATRYN	AATAAGACAAAAAT
M00136	V$OCT1_02	0.831348	1403	(+)	NNGAATATKCANNNN	ACAAAAATTCATGAA
M00162	V$OCT1_06	0.825	1404	(+)	CWNAWTKWSATRYN	CAAAAATTCATGAA
M00138	V$OCT1_04	0.818904	1405	(+)	NNNNNNNWATGCAAATNNNWNNW	AAAAATTCATGAAAATCAC-TTAG
M00135	V$OCT1_01	0.730637	1407	(+)	NNNNWTATGCAAATNTNNN	AAATTCATGAAAATCACTT
M00162	V$OCT1_06	0.808203	1409	(+)	CWNAWTKWSATRYN	ATTCATGAAAATCA
M00195	V$OCT1_Q6	0.887555	1409	(+)	NNNNATGCAAATNAN	ATTCATGAAAATCAC
M00162	V$OCT1_06	0.860547	1410	(-)	CWNAWTKWSATRYN	TTCATGAAAATCAC
M00162	V$OCT1_06	0.808203	1416	(-)	CWNAWTKWSATRYN	AAAATCACTTAGTT
M00138	V$OCT1_04	0.7867	1433	(+)	NNNNNNNWATGCAAATNNNWNNW	TAGAGAAAAGATAAAAA-TAAAGT
M00162	V$OCT1_06	0.839844	1445	(-)	CWNAWTKWSATRYN	AAAAATAAAGTATG
M00162	V$OCT1_06	0.823828	1452	(+)	CWNAWTKWSATRYN	AAGTATGACCTTCT

M00162	V$OCT1_06	0.855078	1453	(-)	CWNAWTKWSATRYN	AGTATGACCTTCTT
M00138	V$OCT1_04	0.780845	1456	(+)	NNNNNNNWATGCAAATNNNWNNW	ATGACCTTCTTCATATACATTGT
M00195	V$OCT1_Q6	0.793941	1461	(-)	NNNNATGCAAATNAN	CTTCTTCATATACAT
M00162	V$OCT1_06	0.826562	1522	(+)	CWNAWTKWSATRYN	GAAACATTAATATT
M00136	V$OCT1_02	0.779913	1524	(-)	NNGAATATKCANNNN	AACATTAATATTTTG
M00162	V$OCT1_06	0.847266	1524	(-)	CWNAWTKWSATRYN	AACATTAATATTTT
M00162	V$OCT1_06	0.821484	1556	(-)	CWNAWTKWSATRYN	AGAATCTAAGCGAT
M00138	V$OCT1_04	0.801338	1571	(-)	NNNNNNNWATGCAAATNNNWNNW	TCTGGCAATAAAAATAATAAA-TA
M00137	V$OCT1_03	0.853023	1572	(+)	NNNRTAATNANNN	CTGGCAATAAAAA
M00195	V$OCT1_Q6	0.81905	1574	(+)	NNNNATGCAAATNAN	GGCAATAAAAATAAT
M00248	V$OCT1_07	0.812381	1577	(-)	TNTATGNTAATT	AATAAAAATAAT
M00138	V$OCT1_04	0.81514	1579	(+)	NNNNNNNWATGCAAATNNNWNNW	TAAAAATAA-TAAATATATATTTT
M00138	V$OCT1_04	0.797783	1580	(-)	NNNNNNNWATGCAAATNNNWNNW	AAAAATAA-TAAATATATATTTTT
M00137	V$OCT1_03	0.847096	1581	(+)	NNNRTAATNANNN	AAAATAATAAATA
M00162	V$OCT1_06	0.828906	1582	(+)	CWNAWTKWSATRYN	AAATAATAAATATA
M00138	V$OCT1_04	0.813049	1583	(+)	NNNNNNNWATGCAAATNNNWNNW	AATAATAAATATATATTTTTTAA
M00162	V$OCT1_06	0.813281	1583	(-)	CWNAWTKWSATRYN	AATAATAAATATAT
M00137	V$OCT1_03	0.853813	1584	(+)	NNNRTAATNANNN	ATAATAAATATAT
M00138	V$OCT1_04	0.818277	1584	(-)	NNNNNNNWATGCAAATNNNWNNW	ATAATAAATATATATTTTTTAAT
M00248	V$OCT1_07	0.793513	1586	(-)	TNTATGNTAATT	AATAAATATATA
M00138	V$OCT1_04	0.840652	1592	(-)	NNNNNNNWATGCAAATNNNWNNW	TATATATTTTTTAATAAGTGAAT
M00138	V$OCT1_04	0.780217	1620	(-)	NNNNNNNWATGCAAATNNNWNNW	ACTTAATTTTTCTGTAAATATCT
M00138	V$OCT1_04	0.832915	1623	(+)	NNNNNNNWATGCAAATNNNWNNW	TAATTTTTCTGTAAATATCTGTA
M00135	V$OCT1_01	0.793781	1625	(+)	NNNNWTATGCAAATNTNNN	ATTTTTCTGTAAATATCTG
M00162	V$OCT1_06	0.868359	1684	(+)	CWNAWTKWSATRYN	GTGAATGAGATGAA
M00162	V$OCT1_06	0.847656	1690	(-)	CWNAWTKWSATRYN	GAGATGAAAAAGAG
M00248	V$OCT1_07	0.800297	1706	(-)	TNTATGNTAATT	AAGTAGGATATA
M00162	V$OCT1_06	0.821484	1711	(+)	CWNAWTKWSATRYN	GGATATAGGATGTT
M00135	V$OCT1_01	0.734071	1717	(-)	NNNNWTATGCAAATNTNNN	AGGATGTTAGCAGAAAACA
M00162	V$OCT1_06	0.862891	1760	(+)	CWNAWTKWSATRYN	TAACTTGAAATGTA
M00138	V$OCT1_04	0.791301	1761	(+)	NNNNNNNWATGCAAATNNNWNNW	AACTTGAAATGTAAGAT-TAATGA
M00162	V$OCT1_06	0.837109	1766	(-)	CWNAWTKWSATRYN	GAAATGTAAGATTA
M00137	V$OCT1_03	0.861715	1774	(+)	NNNRTAATNANNN	AGATTAATGAGTA

M00136	V$OCT1_02	0.775853	1777	(-)	NNGAATATKCANNNN	TTAATGAGTAATAGT
M00137	V$OCT1_03	0.8629	1781	(+)	NNNRTAATNANNN	TGAGTAATAGTAA
M00135	V$OCT1_01	0.779855	1805	(+)	NNNNWTATGCAAATNTNNN	GGCCATATGATAAAATGTT
M00162	V$OCT1_06	0.860547	1808	(-)	CWNAWTKWSATRYN	CATATGATAAAATG
M00138	V$OCT1_04	0.834797	1811	(+)	NNNNNNNWATGCAAATNNNWNNW	ATGATAAAATGTTTATTAATATT
M00138	V$OCT1_04	0.782727	1812	(-)	NNNNNNNWATGCAAATNNNWNNW	TGATAAAATGTTTATTAATATTT
M00138	V$OCT1_04	0.794647	1816	(-)	NNNNNNNWATGCAAATNNNWNNW	AAAATGTTTATTAATATTTTTCT
M00248	V$OCT1_07	0.817045	1816	(+)	TNTATGNTAATT	AAAATGTTTATT
M00137	V$OCT1_03	0.864876	1817	(-)	NNNRTAATNANNN	AAATGTTTATTAA
M00138	V$OCT1_04	0.878921	1817	(+)	NNNNNNNWATGCAAATNNNWNNW	AAATGTTTATTAATATTTTTCTA
M00138	V$OCT1_04	0.793183	1818	(-)	NNNNNNNWATGCAAATNNNWNNW	AATGTTTATTAATATTTTTCTAA
M00136	V$OCT1_02	0.801029	1822	(-)	NNGAATATKCANNNN	TTTATTAATATTTTT
M00136	V$OCT1_02	0.791012	1825	(+)	NNGAATATKCANNNN	ATTAATATTTTTCTA
M00135	V$OCT1_01	0.788249	1934	(-)	NNNNWTATGCAAATNTNNN	TGGACATTTGTATTATACA
M00162	V$OCT1_06	0.805859	1953	(+)	CWNAWTKWSATRYN	CTATCTTTGGTTCC
M00137	V$OCT1_03	0.856578	1966	(+)	NNNRTAATNANNN	CTTGTAAAGGGAA
M00135	V$OCT1_01	0.749142	1978	(-)	NNNNWTATGCAAATNTNNN	AGTAATTTTACTTAAATAA
M00138	V$OCT1_04	0.820995	1979	(+)	NNNNNNNWATGCAAATNNNWNNW	GTAATTTTACTTAAATAA-GAAAA
M00138	V$OCT1_04	0.813467	1980	(-)	NNNNNNNWATGCAAATNNNWNNW	TAATTTTACTTAAATAA-GAAAAT
M00138	V$OCT1_04	0.808867	1987	(+)	NNNNNNNWATGCAAATNNNWNNW	ACTTAAATAAGAAAA-TAGATTGA
M00135	V$OCT1_01	0.754674	1989	(+)	NNNNWTATGCAAATNTNNN	TTAAATAAGAAAATAGATT
M00162	V$OCT1_06	0.829297	2070	(+)	CWNAWTKWSATRYN	CATCCTTGCCTGTC
M00138	V$OCT1_04	0.79611	2118	(+)	NNNNNNNWATGCAAATNNNWNNW	AGAAAAAAATGTTGAAA-TAAATA
M00138	V$OCT1_04	0.790255	2120	(+)	NNNNNNNWATGCAAATNNNWNNW	AAAAAAATGTTGAAATAAA-TAAG
M00162	V$OCT1_06	0.847656	2168	(-)	CWNAWTKWSATRYN	AAAATCGCATTACC
M00137	V$OCT1_03	0.875148	2171	(-)	NNNRTAATNANNN	ATCGCATTACCAA
M00138	V$OCT1_04	0.792765	2184	(+)	NNNNNNNWATGCAAATNNNWNNW	CATCATAAATGTATAAA-TAATGC
M00195	V$OCT1_Q6	0.816594	2190	(+)	NNNNATGCAAATNAN	AAATGTATAAATAAT
M00138	V$OCT1_04	0.798411	2195	(+)	NNNNNNNWATGCAAATNNNWNNW	TATAAA-TAATGCACAATCTCAGA
M00136	V$OCT1_02	0.783162	2200	(-)	NNGAATATKCANNNN	ATAATGCACAATCTC
M00162	V$OCT1_06	0.804297	2206	(+)	CWNAWTKWSATRYN	CACAATCTCAGATT
M00162	V$OCT1_06	0.821484	2207	(-)	CWNAWTKWSATRYN	ACAATCTCAGATTT
M00162	V$OCT1_06	0.847656	2208	(+)	CWNAWTKWSATRYN	CAATCTCAGATTTA

M00162	V$OCT1_06	0.855078	2220	(+)	CWNAWTKWSATRYN	TATTTTTTAATGCT
M00138	V$OCT1_04	0.828733	2221	(+)	NNNNNNNWATGCAAATNNNWNNW	ATTTTTTAATGCTAAGAAAGTCA
M00162	V$OCT1_06	0.842187	2234	(+)	CWNAWTKWSATRYN	AAGAAAGTCATTTA
M00162	V$OCT1_06	0.855078	2235	(-)	CWNAWTKWSATRYN	AGAAAGTCATTTAT
M00248	V$OCT1_07	0.792453	2244	(+)	TNTATGNTAATT	TTTATGTTCATC
M00162	V$OCT1_06	0.808594	2326	(-)	CWNAWTKWSATRYN	TACCTTCAATTCCT
M00137	V$OCT1_03	0.854208	2335	(+)	NNNRTAATNANNN	TTCCTAAAGAAAA
M00136	V$OCT1_02	0.776936	2403	(-)	NNGAATATKCANNNN	AAAATGAGTAATAGT
M00138	V$OCT1_04	0.814304	2426	(+)	NNNNNNNWATGCAAATNNNWNNW	ACAATTGCACGCATATACAATCT
M00136	V$OCT1_02	0.842718	2431	(-)	NNGAATATKCANNNN	TGCACGCATATACAA
M00136	V$OCT1_02	0.805631	2434	(+)	NNGAATATKCANNNN	ACGCATATACAATCT
M00138	V$OCT1_04	0.791928	2438	(-)	NNNNNNNWATGCAAATNNNWNNW	ATATACAATCTAGATAAATATAT
M00137	V$OCT1_03	0.858159	2448	(+)	NNNRTAATNANNN	TAGATAAATATAT
M00162	V$OCT1_06	0.80625	2461	(-)	CWNAWTKWSATRYN	CTTATCCCAGTGAT
M00162	V$OCT1_06	0.842187	2469	(+)	CWNAWTKWSATRYN	AGTGATGAAATATT
M00138	V$OCT1_04	0.780217	2473	(-)	NNNNNNNWATGCAAATNNNWNNW	ATGAAATATTTGTATCTCTTACT
M00135	V$OCT1_01	0.747806	2475	(-)	NNNNWTATGCAAATNTNNN	GAAATATTTGTATCTCTTA
M00195	V$OCT1_Q6	0.797216	2477	(-)	NNNNATGCAAATNAN	AATATTTGTATCTCT
M00162	V$OCT1_06	0.855078	2483	(-)	CWNAWTKWSATRYN	TGTATCTCTTACTG
M00162	V$OCT1_06	0.857812	2545	(+)	CWNAWTKWSATRYN	GAGGATGACATATC
M00138	V$OCT1_04	0.798202	2562	(+)	NNNNNNNWATGCAAATNNNWNNW	CAACAATAATACATATGTTCATA
M00162	V$OCT1_06	0.862891	2565	(+)	CWNAWTKWSATRYN	CAATAATACATATG
M00138	V$OCT1_04	0.794228	2568	(+)	NNNNNNNWATGCAAATNNNWNNW	TAATACATATGTTCATAATTATA
M00138	V$OCT1_04	0.809912	2569	(-)	NNNNNNNWATGCAAATNNNWNNW	AATACATATGTTCATAATTATAA
M00135	V$OCT1_01	0.747615	2570	(+)	NNNNWTATGCAAATNTNNN	ATACATATGTTCATAATTA
M00138	V$OCT1_04	0.80803	2570	(+)	NNNNNNNWATGCAAATNNNWNNW	ATACATATGTTCATAATTATAAT
M00135	V$OCT1_01	0.790919	2571	(-)	NNNNWTATGCAAATNTNNN	TACATATGTTCATAATTAT
M00162	V$OCT1_06	0.809375	2573	(+)	CWNAWTKWSATRYN	CATATGTTCATAAT
M00138	V$OCT1_04	0.803639	2574	(+)	NNNNNNNWATGCAAATNNNWNNW	ATATGTTCATAATTATAATAAAA
M00138	V$OCT1_04	0.801129	2577	(+)	NNNNNNNWATGCAAATNNNWNNW	TGTTCATAATTATAATAAAAAGT
M00137	V$OCT1_03	0.943501	2579	(+)	NNNRTAATNANNN	TTCATAATTATAA
M00137	V$OCT1_03	0.914263	2580	(-)	NNNRTAATNANNN	TCATAATTATAAT
M00248	V$OCT1_07	0.857961	2584	(-)	TNTATGNTAATT	AATTATAATAAA

M00137	V$OCT1_03	0.884631	2585	(+)	NNNRTAATNANNN	ATTATAATAAAAA
M00162	V$OCT1_06	0.826562	2587	(-)	CWNAWTKWSATRYN	TATAATAAAAAGTG
M00138	V$OCT1_04	0.791719	2596	(+)	NNNNNNNWATGCAAATNNNWNNW	AAGTGTTCAG-TAAAAATTAAAAT
M00138	V$OCT1_04	0.822459	2603	(+)	NNNNNNNWATGCAAATNNNWNNW	CAGTAAAAATTAAAA-TAACTCCT
M00138	V$OCT1_04	0.836261	2604	(-)	NNNNNNNWATGCAAATNNNWNNW	AGTAAAAATTAAAA-TAACTCCTT
M00135	V$OCT1_01	0.758489	2606	(-)	NNNNWTATGCAAATNTNNN	TAAAAATTAAAATAACTCC
M00195	V$OCT1_Q6	0.833515	2607	(+)	NNNNATGCAAATNAN	AAAAATTAAAATAAC
M00162	V$OCT1_06	0.908984	2608	(-)	CWNAWTKWSATRYN	AAAATTAAAATAAC
M00248	V$OCT1_07	0.896544	2610	(-)	TNTATGNTAATT	AATTAAAATAAC
M00137	V$OCT1_03	0.857369	2646	(-)	NNNRTAATNANNN	TCTTCATTAAGGT
M00138	V$OCT1_04	0.787955	2647	(+)	NNNNNNNWATGCAAATNNNWNNW	CTTCATTAAGGTAAAACAAAAAT
M00162	V$OCT1_06	0.805469	2658	(-)	CWNAWTKWSATRYN	TAAAACAAAAATAT
M00138	V$OCT1_04	0.799038	2683	(-)	NNNNNNNWATGCAAATNNNWNNW	CTATTAATTTAAAA-TACTCTCAA
M00162	V$OCT1_06	0.839453	2685	(+)	CWNAWTKWSATRYN	ATTAATTTAAAATA
M00162	V$OCT1_06	0.813672	2686	(-)	CWNAWTKWSATRYN	TTAATTTAAAATAC
M00162	V$OCT1_06	0.805859	2687	(-)	CWNAWTKWSATRYN	TAATTTAAAATACT
M00248	V$OCT1_07	0.817257	2689	(-)	TNTATGNTAATT	ATTTAAAATACT
M00162	V$OCT1_06	0.868359	2710	(-)	CWNAWTKWSATRYN	CGTATAACATTTTT
M00162	V$OCT1_06	0.847656	2715	(+)	CWNAWTKWSATRYN	AACATTTTTATTTT
M00138	V$OCT1_04	0.837516	2718	(-)	NNNNNNNWATGCAAATNNNWNNW	ATTTTTATTTTTCAGATTTGTGA
M00162	V$OCT1_06	0.808203	2723	(-)	CWNAWTKWSATRYN	TATTTTTCAGATTT
M00162	V$OCT1_06	0.813672	2724	(+)	CWNAWTKWSATRYN	ATTTTTCAGATTTG
M00162	V$OCT1_06	0.805469	2731	(+)	CWNAWTKWSATRYN	AGATTTGTGAAATA
M00162	V$OCT1_06	0.836719	2760	(-)	CWNAWTKWSATRYN	AGCAAGTAAAAGTT
M00138	V$OCT1_04	0.792765	2770	(+)	NNNNNNNWATGCAAATNNNWNNW	AGTTAGGTAGGAAAAA-TATTTAA
M00162	V$OCT1_06	0.813281	2775	(-)	CWNAWTKWSATRYN	GGTAGGAAAAATAT
M00136	V$OCT1_02	0.831077	2781	(+)	NNGAATATKCANNNN	AAAAATATTTAATAA
M00162	V$OCT1_06	0.834375	2783	(+)	CWNAWTKWSATRYN	AAATATTTAATAAT
M00162	V$OCT1_06	0.945312	2784	(-)	CWNAWTKWSATRYN	AATATTTAATAATG
M00137	V$OCT1_03	0.846701	2786	(+)	NNNRTAATNANNN	TATTTAATAATGA
M00137	V$OCT1_03	0.943896	2789	(+)	NNNRTAATNANNN	TTAATAATGAGTT
M00162	V$OCT1_06	0.821484	2809	(-)	CWNAWTKWSATRYN	GGAACGAAAGTTTT
M00137	V$OCT1_03	0.849467	2831	(-)	NNNRTAATNANNN	CTCTCTTTAGCAG

M00162	V$OCT1_06	0.813672	2846	(-)	CWNAWTKWSATRYN	AGAACTCAATGTAG
M00138	V$OCT1_04	0.812003	2867	(+)	NNNNNNNWATGCAAATNNNWNNW	GGTAAGATATTTTTATACAAA-GA
M00135	V$OCT1_01	0.746852	2870	(-)	NNNNWTATGCAAATNTNNN	AAGATATTTTTATACAAAG
M00162	V$OCT1_06	0.873437	2872	(+)	CWNAWTKWSATRYN	GATATTTTTATACA
M00137	V$OCT1_03	0.861715	2873	(-)	NNNRTAATNANNN	ATATTTTTATACA
M00138	V$OCT1_04	0.859264	2873	(+)	NNNNNNNWATGCAAATNNNWNNW	ATATTTTTATACAAA-GAAAAAAA
M00135	V$OCT1_01	0.733499	2875	(+)	NNNNWTATGCAAATNTNNN	ATTTTTATACAAAGAAAAA
M00162	V$OCT1_06	0.834375	2890	(+)	CWNAWTKWSATRYN	AAAAAATTAATTTA
M00138	V$OCT1_04	0.785236	2891	(+)	NNNNNNNWATGCAAATNNNWNNW	AAAAATTAATTTAACTG-TAAAAT
M00138	V$OCT1_04	0.796947	2892	(-)	NNNNNNNWATGCAAATNNNWNNW	AAAATTAATTTAACTGTAAAA-TA
M00162	V$OCT1_06	0.826562	2892	(-)	CWNAWTKWSATRYN	AAAATTAATTTAAC
M00162	V$OCT1_06	0.847266	2902	(-)	CWNAWTKWSATRYN	TAACTGTAAAATAG
M00135	V$OCT1_01	0.745135	2927	(-)	NNNNWTATGCAAATNTNNN	GATGATTTAGCAGAAGACT
M00137	V$OCT1_03	0.843935	2947	(-)	NNNRTAATNANNN	AGCTAATTGTCAA
M00162	V$OCT1_06	0.865625	2949	(+)	CWNAWTKWSATRYN	CTAATTGTCAATTT
M00195	V$OCT1_Q6	0.794487	2949	(-)	NNNNATGCAAATNAN	CTAATTGTCAATTTT
M00162	V$OCT1_06	0.855078	2950	(-)	CWNAWTKWSATRYN	TAATTGTCAATTTT
M00138	V$OCT1_04	0.808239	2951	(+)	NNNNNNNWATGCAAATNNNWNNW	AATTGTCAATTTTTATTTTTCCT
M00138	V$OCT1_04	0.782936	2952	(-)	NNNNNNNWATGCAAATNNNWNNW	ATTGTCAATTTTTATTTTTCCTT
M00162	V$OCT1_06	0.826562	2957	(+)	CWNAWTKWSATRYN	CAATTTTTATTTTT
M00138	V$OCT1_04	0.794647	2959	(-)	NNNNNNNWATGCAAATNNNWNNW	ATTTTTATTTTTCCTTTATAGAC
M00162	V$OCT1_06	0.816016	2988	(+)	CWNAWTKWSATRYN	AAGCCTTTCAAGCA
M00162	V$OCT1_06	0.834375	2989	(-)	CWNAWTKWSATRYN	AGCCTTTCAAGCAG
M00136	V$OCT1_02	0.805089	3011	(+)	NNGAATATKCANNNN	GATAATGTTCATTCA
M00162	V$OCT1_06	0.839453	3012	(+)	CWNAWTKWSATRYN	ATAATGTTCATTCA
M00248	V$OCT1_07	0.817045	3012	(+)	TNTATGNTAATT	ATAATGTTCATT
M00138	V$OCT1_04	0.7821	3013	(+)	NNNNNNNWATGCAAATNNNWNNW	TAATGTTCATTCAGAGGCAATTT
M00162	V$OCT1_06	0.800391	3031	(-)	CWNAWTKWSATRYN	AATTTCCCAGATTT
M00162	V$OCT1_06	0.800781	3055	(+)	CWNAWTKWSATRYN	AACACTGTATTATC
M00162	V$OCT1_06	0.844531	3068	(+)	CWNAWTKWSATRYN	CTTTTTGTGTTACA
M00162	V$OCT1_06	0.839453	3073	(-)	CWNAWTKWSATRYN	TGTGTTACATTAAT
M00138	V$OCT1_04	0.806148	3192	(-)	NNNNNNNWATGCAAATNNNWNNW	ACTTTCACTTTTCACTTTCATGC
M00162	V$OCT1_06	0.868359	3202	(+)	CWNAWTKWSATRYN	TTCACTTTCATGCA

M00162	V$OCT1_06	0.857031	3258	(-)	CWNAWTKWSATRYN	AGAATCCCAGGGAC
M00162	V$OCT1_06	0.800781	3301	(-)	CWNAWTKWSATRYN	GGTCTTACAGTGTT
M00162	V$OCT1_06	0.834766	3308	(+)	CWNAWTKWSATRYN	CAGTGTTGGATACG
M00137	V$OCT1_03	0.870802	3380	(+)	NNNRTAATNANNN	TTGGTAAAGAATA
M00136	V$OCT1_02	0.845425	3383	(-)	NNGAATATKCANNNN	GTAAAGAATATGCCT
M00136	V$OCT1_02	0.906605	3386	(+)	NNGAATATKCANNNN	AAGAATATGCCTGCC
M00162	V$OCT1_06	0.829297	3392	(+)	CWNAWTKWSATRYN	ATGCCTGCCATGCA
M00162	V$OCT1_06	0.842187	3393	(-)	CWNAWTKWSATRYN	TGCCTGCCATGCAG
M00135	V$OCT1_01	0.832697	3467	(+)	NNNNWTATGCAAATNTNNN	TTCTTTAAGCTAATGTGGC
M00248	V$OCT1_07	0.799237	3470	(+)	TNTATGNTAATT	TTTAAGCTAATG
M00138	V$OCT1_04	0.781054	3511	(+)	NNNNNNNWATGCAAATNNNWNNW	AATTTCCACTGTATATATTTTAA
M00248	V$OCT1_07	0.800721	3511	(-)	TNTATGNTAATT	AATTTCCACTGT
M00138	V$OCT1_04	0.811794	3547	(-)	NNNNNNNWATGCAAATNNNWNNW	TAGAAGGTTTAACATTCTAG-TAA
M00162	V$OCT1_06	0.808594	3551	(+)	CWNAWTKWSATRYN	AGGTTTAACATTCT
M00162	V$OCT1_06	0.826953	3557	(-)	CWNAWTKWSATRYN	AACATTCTAGTAAT
M00137	V$OCT1_03	0.850257	3563	(+)	NNNRTAATNANNN	CTAGTAATTTCTA
M00138	V$OCT1_04	0.80803	3581	(+)	NNNNNNNWATGCAAATNNNWNNW	GTTTGTTATTGGAAATTGTATCA
M00248	V$OCT1_07	0.815137	3586	(+)	TNTATGNTAATT	TTATTGGAAATT
M00162	V$OCT1_06	0.805859	3597	(-)	CWNAWTKWSATRYN	TGTATCACTGTGCC
M00162	V$OCT1_06	0.88125	3623	(+)	CWNAWTKWSATRYN	CTTAATGAACTGTA
M00162	V$OCT1_06	0.800781	3649	(+)	CWNAWTKWSATRYN	CTGTCTTAAACTCT
M00137	V$OCT1_03	0.842355	3663	(-)	NNNRTAATNANNN	CTCTGTTTACCTT
M00162	V$OCT1_06	0.808594	3682	(+)	CWNAWTKWSATRYN	TGCTTTGGCATTTA
M00162	V$OCT1_06	0.821484	3757	(+)	CWNAWTKWSATRYN	CACTTTGTAAAGTG
M00138	V$OCT1_04	0.805312	3772	(+)	NNNNNNNWATGCAAATNNNWNNW	GGGCAGA-CATGCAAATGGACATA
M00135	V$OCT1_01	0.812476	3774	(+)	NNNNWTATGCAAATNTNNN	GCAGACATGCAAATGGACA
M00195	V$OCT1_Q6	0.862172	3776	(+)	NNNNATGCAAATNAN	AGACATGCAAATGGA
M00135	V$OCT1_01	0.740748	3781	(-)	NNNNWTATGCAAATNTNNN	TGCAAATGGACATATTTTA
M00162	V$OCT1_06	0.894141	3783	(+)	CWNAWTKWSATRYN	CAAATGGACATATT
M00162	V$OCT1_06	0.865625	3791	(+)	CWNAWTKWSATRYN	CATATTTTATTATT
M00162	V$OCT1_06	0.855469	3791	(-)	CWNAWTKWSATRYN	CATATTTTATTATT
M00137	V$OCT1_03	0.887001	3794	(-)	NNNRTAATNANNN	ATTTTATTATTAT
M00137	V$OCT1_03	0.911497	3797	(-)	NNNRTAATNANNN	TTATTATTATACC

M00137	V$OCT1_03	0.858554	3816	(+)	NNNRTAATNANNN	CTAGTAAATAAGA
M00136	V$OCT1_02	0.789117	3931	(-)	NNGAATATKCANNNN	TGCATCAATATACTT
M00162	V$OCT1_06	0.839453	3931	(-)	CWNAWTKWSATRYN	TGCATCAATATACT
M00162	V$OCT1_06	0.869922	4057	(+)	CWNAWTKWSATRYN	ATTCCTGGGATTCT
M00162	V$OCT1_06	0.868359	4113	(-)	CWNAWTKWSATRYN	TGCATGAAAGTGAA
M00138	V$OCT1_04	0.783982	4114	(+)	NNNNNNNWATGCAAATNNNWNNW	GCATGAAAGTGAAAAGGGAAAGT
M00162	V$OCT1_06	0.808594	4142	(+)	CWNAWTKWSATRYN	CGCTCAGTCATGTC
M00162	V$OCT1_06	0.877734	4200	(+)	CWNAWTKWSATRYN	GTCCATGGGATTTT
M00136	V$OCT1_02	0.792637	4268	(+)	NNGAATATKCANNNN	GAGAGTATGTATTTC
M00162	V$OCT1_06	0.862891	4271	(-)	CWNAWTKWSATRYN	AGTATGTATTTCTT
M00162	V$OCT1_06	0.821094	4274	(+)	CWNAWTKWSATRYN	ATGTATTTCTTATA
M00136	V$OCT1_02	0.770168	4282	(+)	NNGAATATKCANNNN	CTTATAATTCATTAG
M00137	V$OCT1_03	0.886606	4287	(-)	NNNRTAATNANNN	AATTCATTAGAAG
M00162	V$OCT1_06	0.828906	4288	(+)	CWNAWTKWSATRYN	ATTCATTAGAAGCC
M00137	V$OCT1_03	0.841565	4298	(+)	NNNRTAATNANNN	AGCCTAAACATAA
M00136	V$OCT1_02	0.771792	4322	(-)	NNGAATATKCANNNN	AGAATGATTATTGCA
M00137	V$OCT1_03	0.858949	4323	(-)	NNNRTAATNANNN	GAATGATTATTGC
M00162	V$OCT1_06	0.812109	4337	(-)	CWNAWTKWSATRYN	GCCATGAAATTAAA
M00162	V$OCT1_06	0.883203	4342	(-)	CWNAWTKWSATRYN	GAAATTAAAAGACG
M00248	V$OCT1_07	0.791817	4344	(-)	TNTATGNTAATT	AATTAAAAGACG
M00136	V$OCT1_02	0.875203	4389	(-)	NNGAATATKCANNNN	AGACAGCATATTCAA
M00138	V$OCT1_04	0.81033	4390	(+)	NNNNNNNWATGCAAATNNNWNNW	GACAGCATATTCAAAA-GCAGAGA
M00135	V$OCT1_01	0.771461	4392	(+)	NNNNWTATGCAAATNTNNN	CAGCATATTCAAAAGCAGA
M00136	V$OCT1_02	0.884678	4392	(+)	NNGAATATKCANNNN	CAGCATATTCAAAAG
M00162	V$OCT1_06	0.804297	4395	(+)	CWNAWTKWSATRYN	CATATTCAAAAGCA
M00162	V$OCT1_06	0.808594	4411	(-)	CWNAWTKWSATRYN	GACATTACTTTGCC
M00162	V$OCT1_06	0.83125	4606	(+)	CWNAWTKWSATRYN	AGCCCTGGGATTTC
M00138	V$OCT1_04	0.797156	4626	(+)	NNNNNNNWATGCAAATNNNWNNW	AAGGAATGATGCTAAA-GCTGAAA
M00135	V$OCT1_01	0.7562	4628	(+)	NNNNWTATGCAAATNTNNN	GGAATGATGCTAAAGCTGA
M00162	V$OCT1_06	0.924609	4694	(+)	CWNAWTKWSATRYN	AAGACTTTGATGCT
M00137	V$OCT1_03	0.855393	4904	(-)	NNNRTAATNANNN	GAATGATTAAGAA
M00138	V$OCT1_04	0.804893	4923	(+)	NNNNNNNWATGCAAATNNNWNNW	TAGTGGTTATTATTATATATTTT
M00137	V$OCT1_03	0.853023	4926	(-)	NNNRTAATNANNN	TGGTTATTATTAT

M00195	V$OCT1_Q6	0.798035	4928	(-)	NNNNATGCAAATNAN	GTTATTATTATATAT
M00137	V$OCT1_03	0.896484	4929	(-)	NNNRTAATNANNN	TTATTATTATATA
M00138	V$OCT1_04	0.792974	4929	(+)	NNNNNNNWATGCAAATNNNWNNW	TTATTATTATATATTTTCAATGA
M00162	V$OCT1_06	0.806641	4930	(+)	CWNAWTKWSATRYN	TATTATTATATATT
M00138	V$OCT1_04	0.832706	4935	(-)	NNNNNNNWATGCAAATNNNWNNW	TTATATATTTTCAATGACTATTA
M00138	V$OCT1_04	0.786073	4940	(+)	NNNNNNNWATGCAAATNNNWNNW	TATTTTCAATGACTATTATTCTT
M00136	V$OCT1_02	0.805089	4945	(-)	NNGAATATKCANNNN	TCAATGACTATTATT
M00136	V$OCT1_02	0.813481	4951	(+)	NNGAATATKCANNNN	ACTATTATTCTTTTA
M00135	V$OCT1_01	0.772797	4967	(+)	NNNNWTATGCAAATNTNNN	AACCAGATGAAAATATTAG
M00162	V$OCT1_06	0.868359	4970	(-)	CWNAWTKWSATRYN	CAGATGAAAATATT
M00162	V$OCT1_06	0.828906	4977	(+)	CWNAWTKWSATRYN	AAATATTAGAGATC
M00162	V$OCT1_06	0.808203	4987	(+)	CWNAWTKWSATRYN	GATCATTTGTTTTC
M00195	V$OCT1_Q6	0.798035	4988	(-)	NNNNATGCAAATNAN	ATCATTTGTTTTCCT
M00137	V$OCT1_03	0.848281	4998	(+)	NNNRTAATNANNN	TTCCTAAGGAGAA
M00135	V$OCT1_01	0.747425	5027	(-)	NNNNWTATGCAAATNTNNN	GACAGGTTTGCATGCAAAG
M00162	V$OCT1_06	0.876172	5029	(+)	CWNAWTKWSATRYN	CAGGTTTGCATGCA
M00135	V$OCT1_01	0.778901	5032	(+)	NNNNWTATGCAAATNTNNN	GTTTGCATGCAAAGTTGTT
M00195	V$OCT1_Q6	0.820961	5034	(+)	NNNNATGCAAATNAN	TTGCATGCAAAGTTG
M00162	V$OCT1_06	0.945312	5035	(-)	CWNAWTKWSATRYN	TGCATGCAAAGTTG
M00248	V$OCT1_07	0.824041	5035	(+)	TNTATGNTAATT	TGCATGCAAAGT
M00162	V$OCT1_06	0.817187	5068	(-)	CWNAWTKWSATRYN	TGTATGACCCTATG
M00162	V$OCT1_06	0.816016	5088	(-)	CWNAWTKWSATRYN	AACCTGCCAGGTTC
M00162	V$OCT1_06	0.877734	5106	(+)	CWNAWTKWSATRYN	GTCTATGGGATTTT
M00248	V$OCT1_07	0.805809	5107	(+)	TNTATGNTAATT	TCTATGGGATTT
M00135	V$OCT1_01	0.762495	5171	(-)	NNNNWTATGCAAATNTNNN	ATTGAACTTGCATCTCTTA
M00195	V$OCT1_Q6	0.796943	5173	(-)	NNNNATGCAAATNAN	TGAACTTGCATCTCT
M00162	V$OCT1_06	0.832031	5235	(-)	CWNAWTKWSATRYN	GGAAGCCCAATTAG
M00135	V$OCT1_01	0.816864	5239	(-)	NNNNWTATGCAAATNTNNN	GCCCAATTAGCATCTGTTA
M00195	V$OCT1_Q6	0.813046	5241	(-)	NNNNATGCAAATNAN	CCAATTAGCATCTGT
M00248	V$OCT1_07	0.855205	5243	(-)	TNTATGNTAATT	AATTAGCATCTG
M00136	V$OCT1_02	0.788305	5293	(-)	NNGAATATKCANNNN	TGACTGCATCATCTT
M00138	V$OCT1_04	0.799456	5323	(+)	NNNNNNNWATGCAAATNNNWNNW	ACAGCCTTATTCAGAATAGTG-GA
M00136	V$OCT1_02	0.800217	5325	(+)	NNGAATATKCANNNN	AGCCTTATTCAGAAT

M00137	V$OCT1_03	0.911893	5364	(+)	NNNRTAATNANNN	AAAATAATGATAT
M00136	V$OCT1_02	0.774228	5387	(-)	NNGAATATKCANNNN	GCTGTCCATATTAAC
M00138	V$OCT1_04	0.780427	5389	(-)	NNNNNNNWATGCAAATNNNWNNW	TGTCCATATTAAC-CTATTAAATG
M00135	V$OCT1_01	0.794353	5391	(-)	NNNNWTATGCAAATNTNNN	TCCATATTAACCTATTAAA
M00162	V$OCT1_06	0.817187	5393	(+)	CWNAWTKWSATRYN	CATATTAACCTATT
M00162	V$OCT1_06	0.824219	5400	(+)	CWNAWTKWSATRYN	ACCTATTAAATGTC
M00162	V$OCT1_06	0.800781	5401	(-)	CWNAWTKWSATRYN	CCTATTAAATGTCT
M00162	V$OCT1_06	0.852734	5416	(+)	CWNAWTKWSATRYN	CATTTTCTGATTTA
M00195	V$OCT1_Q6	0.795852	5422	(-)	NNNNATGCAAATNAN	CTGATTTATGTTGAC
M00138	V$OCT1_04	0.86721	5432	(-)	NNNNNNNWATGCAAATNNNWNNW	TTGACAAATATGAATTTTTCTTT
M00135	V$OCT1_01	0.730256	5434	(-)	NNNNWTATGCAAATNTNNN	GACAAATATGAATTTTTCT
M00136	V$OCT1_02	0.788305	5435	(+)	NNGAATATKCANNNN	ACAAATATGAATTTT
M00162	V$OCT1_06	0.839844	5436	(+)	CWNAWTKWSATRYN	CAAATATGAATTTT
M00162	V$OCT1_06	0.816016	5437	(+)	CWNAWTKWSATRYN	AAATATGAATTTTT
M00136	V$OCT1_02	0.844342	5438	(-)	NNGAATATKCANNNN	AATATGAATTTTTCT
M00162	V$OCT1_06	0.867969	5438	(-)	CWNAWTKWSATRYN	AATATGAATTTTTC
M00138	V$OCT1_04	0.81033	5465	(+)	NNNNNNNWATGCAAATNNNWNNW	CTGATTTTATTTTTATTTTTCCA
M00162	V$OCT1_06	0.832031	5465	(+)	CWNAWTKWSATRYN	CTGATTTTATTTTT
M00138	V$OCT1_04	0.845462	5466	(-)	NNNNNNNWATGCAAATNNNWNNW	TGATTTTATTTTTATTTTTCCAA
M00195	V$OCT1_Q6	0.815502	5470	(-)	NNNNATGCAAATNAN	TTTATTTTTATTTTT
M00248	V$OCT1_07	0.831673	5470	(+)	TNTATGNTAATT	TTTATTTTTATT
M00137	V$OCT1_03	0.842355	5492	(-)	NNNRTAATNANNN	AATCTATTACACA
M00162	V$OCT1_06	0.828906	5493	(+)	CWNAWTKWSATRYN	ATCTATTACACACA
M00162	V$OCT1_06	0.804297	5501	(-)	CWNAWTKWSATRYN	CACACATCAATAAG
M00138	V$OCT1_04	0.812422	5513	(-)	NNNNNNNWATGCAAATNNNWNNW	AGGTAAAAC-CTTCATATTTAAAC
M00138	V$OCT1_04	0.781054	5514	(+)	NNNNNNNWATGCAAATNNNWNNW	GGTAAAAC-CTTCATATTTAAACG
M00135	V$OCT1_01	0.731973	5515	(-)	NNNNWTATGCAAATNTNNN	GTAAAACCTTCATATTTAA
M00162	V$OCT1_06	0.847266	5517	(+)	CWNAWTKWSATRYN	AAAACCTTCATATT
M00138	V$OCT1_04	0.791719	5521	(-)	NNNNNNNWATGCAAATNNNWNNW	CCTTCATATTTAAACGTACATTT
M00162	V$OCT1_06	0.88125	5525	(+)	CWNAWTKWSATRYN	CATATTTAAACGTA
M00162	V$OCT1_06	0.847266	5539	(+)	CWNAWTKWSATRYN	CATTTTTAAAAACT
M00195	V$OCT1_Q6	0.800218	5559	(-)	NNNNATGCAAATNAN	TTGATTTTTATAAAC
M00137	V$OCT1_03	0.846701	5565	(+)	NNNRTAATNANNN	TTTATAAACAGCA

M00162	V$OCT1_06	0.889062	5574	(-)	CWNAWTKWSATRYN	AGCATTTCTTTATG
M00137	V$OCT1_03	0.879494	5577	(-)	NNNRTAATNANNN	ATTTCTTTATGTG
M00162	V$OCT1_06	0.867969	5609	(+)	CWNAWTKWSATRYN	AAAATTGAGAAGTT
M00162	V$OCT1_06	0.808594	5617	(-)	CWNAWTKWSATRYN	GAAGTTTCAAAGTG
M00162	V$OCT1_06	0.844922	5619	(-)	CWNAWTKWSATRYN	AGTTTCAAAGTGAG
M00137	V$OCT1_03	0.8629	5648	(+)	NNNRTAATNANNN	GAGGTAATTTGTT
M00138	V$OCT1_04	0.809285	5680	(+)	NNNNNNNWATGCAAATNNNWNNW	GAAGTATTATGAAACATAACACA
M00137	V$OCT1_03	0.88226	5699	(+)	NNNRTAATNANNN	CACATAAAAAGAT
M00162	V$OCT1_06	0.842187	5699	(-)	CWNAWTKWSATRYN	CACATAAAAAGATT
M00162	V$OCT1_06	0.804297	5701	(+)	CWNAWTKWSATRYN	CATAAAAAGATTTA
M00138	V$OCT1_04	0.812212	5702	(+)	NNNNNNNWATGCAAATNNNWNNW	ATAAAAAGATTTATAA-TAATTAT
M00138	V$OCT1_04	0.82455	5704	(-)	NNNNNNNWATGCAAATNNNWNNW	AAAAAGATTTATAA-TAATTATGT
M00137	V$OCT1_03	0.905176	5711	(+)	NNNRTAATNANNN	TTTATAATAATTA
M00248	V$OCT1_07	0.819801	5711	(+)	TNTATGNTAATT	TTTATAATAATT
M00137	V$OCT1_03	0.916634	5714	(+)	NNNRTAATNANNN	ATAATAATTATGT
M00137	V$OCT1_03	0.896879	5715	(-)	NNNRTAATNANNN	TAATAATTATGTT
M00138	V$OCT1_04	0.796738	5715	(+)	NNNNNNNWATGCAAATNNNWNNW	TAATAATTATGTTCAGTCTAA-GA
M00138	V$OCT1_04	0.796529	5730	(+)	NNNNNNNWATGCAAATNNNWNNW	GTCTAAGAATGGTAA-TATAAATG
M00135	V$OCT1_01	0.791492	5732	(+)	NNNNWTATGCAAATNTNNN	CTAAGAATGGTAATATAAA
M00162	V$OCT1_06	0.81875	5735	(+)	CWNAWTKWSATRYN	AGAATGGTAATATA
M00248	V$OCT1_07	0.824677	5735	(+)	TNTATGNTAATT	AGAATGGTAATA
M00138	V$OCT1_04	0.788582	5736	(+)	NNNNNNNWATGCAAATNNNWNNW	GAATGGTAA-TATAAATGTCAGTG
M00135	V$OCT1_01	0.734071	5738	(+)	NNNNWTATGCAAATNTNNN	ATGGTAATATAAATGTCAG
M00137	V$OCT1_03	0.84275	5738	(+)	NNNRTAATNANNN	ATGGTAATATAAA
M00162	V$OCT1_06	0.834375	5741	(+)	CWNAWTKWSATRYN	GTAATATAAATGTC
M00138	V$OCT1_04	0.781054	5742	(+)	NNNNNNNWATGCAAATNNNWNNW	TAATATAAATGTCAGTGTAA-GAA
M00162	V$OCT1_06	0.863281	5747	(-)	CWNAWTKWSATRYN	TAAATGTCAGTGTA
M00162	V$OCT1_06	0.842187	5762	(-)	CWNAWTKWSATRYN	GAAATGAAAACTTT
M00138	V$OCT1_04	0.832915	5774	(+)	NNNNNNNWATGCAAATNNNWNNW	TTGACAAAATGAAAA-TATTTTAA
M00135	V$OCT1_01	0.779092	5776	(+)	NNNNWTATGCAAATNTNNN	GACAAAATGAAAATATTTT
M00195	V$OCT1_Q6	0.809225	5778	(+)	NNNNATGCAAATNAN	CAAAATGAAAATATT
M00162	V$OCT1_06	0.9375	5779	(-)	CWNAWTKWSATRYN	AAAATGAAAATATT
M00248	V$OCT1_07	0.812381	5781	(-)	TNTATGNTAATT	AATGAAAATATT

M00162	V$OCT1_06	0.821094	5788	(+)	CWNAWTKWSATRYN	ATATTTTAAAGGTA
M00162	V$OCT1_06	0.81875	5789	(-)	CWNAWTKWSATRYN	TATTTTAAAGGTAG
M00162	V$OCT1_06	0.847266	5801	(-)	CWNAWTKWSATRYN	AGAAACACATTTTT
M00162	V$OCT1_06	0.847266	5808	(+)	CWNAWTKWSATRYN	CATTTTTAAACACA
M00135	V$OCT1_01	0.798359	5817	(+)	NNNNWTATGCAAATNTNNN	ACACATAATCAAATTTCAG
M00137	V$OCT1_03	0.92335	5818	(+)	NNNRTAATNANNN	CACATAATCAAAT
M00162	V$OCT1_06	0.86875	5820	(+)	CWNAWTKWSATRYN	CATAATCAAATTTC
M00162	V$OCT1_06	0.813672	5820	(-)	CWNAWTKWSATRYN	CATAATCAAATTTC
M00162	V$OCT1_06	0.803516	5826	(-)	CWNAWTKWSATRYN	CAAATTTCAGAGTA
M00136	V$OCT1_02	0.82837	5863	(-)	NNGAATATKCANNNN	TACTGGTATATTCAT
M00138	V$OCT1_04	0.829987	5863	(-)	NNNNNNNWATGCAAATNNNWNNW	TACTGGTATATTCATTTTACTAA
M00136	V$OCT1_02	0.87412	5866	(+)	NNGAATATKCANNNN	TGGTATATTCATTTT
M00162	V$OCT1_06	0.826562	5867	(+)	CWNAWTKWSATRYN	GGTATATTCATTTT
M00138	V$OCT1_04	0.801129	5869	(-)	NNNNNNNWATGCAAATNNNWNNW	TATATTCATTTTACTAATGGTAT
M00162	V$OCT1_06	0.826562	5869	(-)	CWNAWTKWSATRYN	TATATTCATTTTAC
M00137	V$OCT1_03	0.858554	5879	(+)	NNNRTAATNANNN	TTACTAATGGTAT
M00138	V$OCT1_04	0.792137	5898	(-)	NNNNNNNWATGCAAATNNNWNNW	TTTTAATAAATGCATATTAG-TAG
M00138	V$OCT1_04	0.873066	5899	(+)	NNNNNNNWATGCAAATNNNWNNW	TTTAATAAATGCATATTAG-TAGG
M00135	V$OCT1_01	0.767455	5900	(-)	NNNNWTATGCAAATNTNNN	TTAATAAATGCATATTAGT
M00135	V$OCT1_01	0.757726	5901	(+)	NNNNWTATGCAAATNTNNN	TAATAAATGCATATTAGTA
M00162	V$OCT1_06	0.855078	5902	(+)	CWNAWTKWSATRYN	AATAAATGCATATT
M00195	V$OCT1_Q6	0.856168	5903	(+)	NNNNATGCAAATNAN	ATAAATGCATATTAG
M00136	V$OCT1_02	0.935842	5904	(-)	NNGAATATKCANNNN	TAAATGCATATTAGT
M00162	V$OCT1_06	0.852734	5904	(-)	CWNAWTKWSATRYN	TAAATGCATATTAG
M00248	V$OCT1_07	0.819165	5904	(+)	TNTATGNTAATT	TAAATGCATATT
M00162	V$OCT1_06	0.814062	5937	(+)	CWNAWTKWSATRYN	GGGACTGTGATCCC
M00135	V$OCT1_01	0.787867	5948	(+)	NNNNWTATGCAAATNTNNN	CCCCTTATTCTAATGATGG
M00161	V$OCT1_05	0.857214	5951	(-)	MKNATTTGCATAYY	CTTATTCTAATGAT
M00162	V$OCT1_06	0.801953	5951	(+)	CWNAWTKWSATRYN	CTTATTCTAATGAT
M00248	V$OCT1_07	0.793725	5951	(+)	TNTATGNTAATT	CTTATTCTAATG
M00137	V$OCT1_03	0.877914	5954	(+)	NNNRTAATNANNN	ATTCTAATGATGG
M00136	V$OCT1_02	0.799675	5960	(-)	NNGAATATKCANNNN	ATGATGGATATACTG
M00136	V$OCT1_02	0.914727	6082	(-)	NNGAATATKCANNNN	ATTGAGAATATTCAT

M00138	V$OCT1_04	0.833333	6082	(-)	NNNNNNNWATGCAAATNNNWNNW	ATTGAGAA-TATTCATAATTACCC
M00138	V$OCT1_04	0.856336	6083	(+)	NNNNNNNWATGCAAATNNNWNNW	TTGAGAA-TATTCATAATTACCCA
M00135	V$OCT1_01	0.760015	6084	(-)	NNNNWTATGCAAATNTNNN	TGAGAATATTCATAATTAC
M00136	V$OCT1_02	0.961289	6085	(+)	NNGAATATKCANNNN	GAGAATATTCATAAT
M00161	V$OCT1_05	0.870496	6086	(+)	MKNATTTGCATAYY	AGAATATTCATAAT
M00137	V$OCT1_03	0.867246	6092	(+)	NNNRTAATNANNN	TTCATAATTACCC
M00137	V$OCT1_03	0.936784	6093	(-)	NNNRTAATNANNN	TCATAATTACCCA
M00136	V$OCT1_02	0.806443	6124	(-)	NNGAATATKCANNNN	ACAGTGACTATTCAA
M00136	V$OCT1_02	0.862209	6127	(+)	NNGAATATKCANNNN	GTGACTATTCAATGG
M00162	V$OCT1_06	0.808594	6130	(-)	CWNAWTKWSATRYN	ACTATTCAATGGTT
M00138	V$OCT1_04	0.807403	6135	(-)	NNNNNNNWATGCAAATNNNWNNW	TCAATGGTTTTATATTATATTTG
M00162	V$OCT1_06	0.808594	6150	(-)	CWNAWTKWSATRYN	TATATTTGATAGCT
M00137	V$OCT1_03	0.841565	6171	(-)	NNNRTAATNANNN	TCACATTTATGAA
M00138	V$OCT1_04	0.782727	6171	(+)	NNNNNNNWATGCAAATNNNWNNW	TCACATTTATGAATGTGTG-GAGT
M00162	V$OCT1_06	0.829297	6172	(-)	CWNAWTKWSATRYN	CACATTTATGAATG
M00162	V$OCT1_06	0.821484	6174	(+)	CWNAWTKWSATRYN	CATTTATGAATGTG
M00138	V$OCT1_04	0.827478	6197	(-)	NNNNNNNWATGCAAATNNNWNNW	AAAAAGTATTTCCATTACCCAGA
M00135	V$OCT1_01	0.772034	6199	(-)	NNNNWTATGCAAATNTNNN	AAAGTATTTCCATTACCCA
M00162	V$OCT1_06	0.839453	6201	(-)	CWNAWTKWSATRYN	AGTATTTCCATTAC
M00195	V$OCT1_Q6	0.814956	6201	(-)	NNNNATGCAAATNAN	AGTATTTCCATTACC
M00137	V$OCT1_03	0.881865	6205	(-)	NNNRTAATNANNN	TTTCCATTACCCA
M00162	V$OCT1_06	0.821484	6239	(+)	CWNAWTKWSATRYN	AACAATTGAGTATA
M00136	V$OCT1_02	0.774499	6241	(-)	NNGAATATKCANNNN	CAATTGAGTATACAA
M00138	V$OCT1_04	0.785655	6242	(+)	NNNNNNNWATGCAAATNNNWNNW	AATTGAGTATA-CAAATGTGTGAC
M00135	V$OCT1_01	0.861694	6244	(+)	NNNNWTATGCAAATNTNNN	TTGAGTATACAAATGTGTG
M00195	V$OCT1_Q6	0.834334	6246	(+)	NNNNATGCAAATNAN	GAGTATACAAATGTG
M00162	V$OCT1_06	0.873828	6247	(-)	CWNAWTKWSATRYN	AGTATACAAATGTG
M00162	V$OCT1_06	0.808203	6265	(-)	CWNAWTKWSATRYN	CATACCACATAGTT
M00137	V$OCT1_03	0.856183	6271	(-)	NNNRTAATNANNN	ACATAGTTATTAA
M00135	V$OCT1_01	0.77852	6278	(-)	NNNNWTATGCAAATNTNNN	TATTAATTAGCAGAACTTG
M00137	V$OCT1_03	0.864876	6278	(-)	NNNRTAATNANNN	TATTAATTAGCAG
M00248	V$OCT1_07	0.864109	6282	(-)	TNTATGNTAATT	AATTAGCAGAAC
M00135	V$OCT1_01	0.758298	6287	(-)	NNNNWTATGCAAATNTNNN	GCAGAACTTGCTTAAAAAT

M00162	V$OCT1_06	0.809375	6295	(-)	CWNAWTKWSATRYN	TGCTTAAAAATAAG
M00162	V$OCT1_06	0.842578	6319	(+)	CWNAWTKWSATRYN	CAGCATAAAATTCT
M00162	V$OCT1_06	0.876172	6320	(-)	CWNAWTKWSATRYN	AGCATAAAATTCTT
M00136	V$OCT1_02	0.784515	6322	(+)	NNGAATATKCANNNN	CATAAAATTCTTTCA
M00138	V$OCT1_04	0.780845	6323	(-)	NNNNNNNWATGCAAATNNNWNNW	ATAAAATTCTTTCATTATATTAC
M00162	V$OCT1_06	0.821484	6327	(+)	CWNAWTKWSATRYN	AATTCTTTCATTAT
M00137	V$OCT1_03	0.867246	6331	(-)	NNNRTAATNANNN	CTTTCATTATATT
M00136	V$OCT1_02	0.77098	6351	(-)	NNGAATATKCANNNN	TGGTAACATATTTAT
M00138	V$OCT1_04	0.783145	6351	(-)	NNNNNNNWATGCAAATNNNWNNW	TGGTAACATATTTATCTAATTGT
M00137	V$OCT1_03	0.86448	6356	(-)	NNNRTAATNANNN	ACATATTTATCTA
M00162	V$OCT1_06	0.894531	6361	(-)	CWNAWTKWSATRYN	TTTATCTAATTGTG
M00137	V$OCT1_03	0.88305	6364	(-)	NNNRTAATNANNN	ATCTAATTGTGAT
M00162	V$OCT1_06	0.960937	6366	(+)	CWNAWTKWSATRYN	CTAATTGTGATATT
M00162	V$OCT1_06	0.829297	6374	(-)	CWNAWTKWSATRYN	GATATTTAAGCTTT
M00162	V$OCT1_06	0.813672	6389	(+)	CWNAWTKWSATRYN	CTGTTTTAGAATTG
M00162	V$OCT1_06	0.834766	6396	(-)	CWNAWTKWSATRYN	AGAATTGAAGATTG
M00136	V$OCT1_02	0.804819	6397	(-)	NNGAATATKCANNNN	GAATTGAAGATTGAT
M00138	V$OCT1_04	0.785864	6433	(+)	NNNNNNNWATGCAAATNNNWNNW	ATTTTCTAAGTCAAAATGAATTT
M00136	V$OCT1_02	0.810233	6445	(-)	NNGAATATKCANNNN	AAAATGAATTTACAA
M00162	V$OCT1_06	0.808203	6465	(-)	CWNAWTKWSATRYN	TGCCTTTAAAGACT
M00137	V$OCT1_03	0.895693	6478	(-)	NNNRTAATNANNN	TCAAGATTACCAC
M00162	V$OCT1_06	0.882812	6542	(-)	CWNAWTKWSATRYN	TGAATTAAAAAAAC
M00248	V$OCT1_07	0.798601	6544	(-)	TNTATGNTAATT	AATTAAAAAAAC
M00138	V$OCT1_04	0.807821	6546	(+)	NNNNNNNWATGCAAATNNNWNNW	TTAAAAAAACACATATTAA-GACT
M00136	V$OCT1_02	0.790742	6551	(-)	NNGAATATKCANNNN	AAAACACATATTAAG
M00162	V$OCT1_06	0.808594	6563	(+)	CWNAWTKWSATRYN	AAGACTTAGTTTCC
M00138	V$OCT1_04	0.799247	6564	(-)	NNNNNNNWATGCAAATNNNWNNW	AGACTTAG-TTTCCATTAAAACAA
M00248	V$OCT1_07	0.815137	6570	(-)	TNTATGNTAATT	AGTTTCCATTAA
M00138	V$OCT1_04	0.786073	6615	(-)	NNNNNNNWATGCAAATNNNWNNW	AAGTCTTTTTAAAATAGATCTTT
M00162	V$OCT1_06	0.831641	6634	(+)	CWNAWTKWSATRYN	CTTTCTTTGTTATA
M00162	V$OCT1_06	0.813672	6654	(+)	CWNAWTKWSATRYN	CAGTTTGGACTATT
M00135	V$OCT1_01	0.750477	6662	(+)	NNNNWTATGCAAATNTNNN	ACTATTATCCAAAGTATGT
M00136	V$OCT1_02	0.775311	6662	(+)	NNGAATATKCANNNN	ACTATTATCCAAAGT

M00161	V$OCT1_05	0.872157	6665	(-)	MKNATTTGCATAYY	ATTATCCAAAGTAT
M00162	V$OCT1_06	0.81875	6665	(-)	CWNAWTKWSATRYN	ATTATCCAAAGTAT
M00162	V$OCT1_06	0.834375	6696	(-)	CWNAWTKWSATRYN	GGCAACTCAGGAAG
M00137	V$OCT1_03	0.899249	6782	(+)	NNNRTAATNANNN	GGGTTAATGAGAA
M00162	V$OCT1_06	0.847266	6784	(+)	CWNAWTKWSATRYN	GTTAATGAGAAATC
M00136	V$OCT1_02	0.792095	6802	(-)	NNGAATATKCANNNN	AGCGAGCATTTTACT
M00137	V$OCT1_03	0.881865	6813	(-)	NNNRTAATNANNN	TACTCATTAGTCT
M00138	V$OCT1_04	0.790046	6816	(+)	NNNNNNNWATGCAAATNNNWNNW	TCATTAGTCTTCATATGACCCCA
M00162	V$OCT1_06	0.816406	6858	(-)	CWNAWTKWSATRYN	CAAATGGAAGATTT
M00162	V$OCT1_06	0.834375	6865	(+)	CWNAWTKWSATRYN	AAGATTTTCTTTCT
M00136	V$OCT1_02	0.771521	6881	(-)	NNGAATATKCANNNN	TCACTGAATTATGTT
M00162	V$OCT1_06	0.81875	6892	(-)	CWNAWTKWSATRYN	TGTTTTAAAAAGAG
M00138	V$OCT1_04	0.781054	6905	(+)	NNNNNNNWATGCAAATNNNWNNW	GGAGGATAATTCATCATAAA-TAA
M00136	V$OCT1_02	0.825393	6907	(+)	NNGAATATKCANNNN	AGGATAATTCATCAT
M00138	V$OCT1_04	0.801547	6907	(-)	NNNNNNNWATGCAAATNNNWNNW	AGGATAATTCATCATAAA-TAACA
M00162	V$OCT1_06	0.852734	6921	(-)	CWNAWTKWSATRYN	TAAATAACAATTAT
M00137	V$OCT1_03	0.857369	6925	(-)	NNNRTAATNANNN	TAACAATTATAAC
M00162	V$OCT1_06	0.813672	6946	(-)	CWNAWTKWSATRYN	GGACTCAAAGTTTT
M00162	V$OCT1_06	0.834766	7115	(+)	CWNAWTKWSATRYN	CAGCCTGAAATAAT
M00162	V$OCT1_06	0.875781	7116	(-)	CWNAWTKWSATRYN	AGCCTGAAATAATG
M00137	V$OCT1_03	0.853813	7121	(+)	NNNRTAATNANNN	GAAATAATGGGAG
M00162	V$OCT1_06	0.821484	7123	(+)	CWNAWTKWSATRYN	AATAATGGGAGTCC
M00162	V$OCT1_06	0.863281	7166	(+)	CWNAWTKWSATRYN	CACAAGGAAATGCC
M00136	V$OCT1_02	0.773146	7169	(+)	NNGAATATKCANNNN	AAGGAAATGCCCTTC
M00162	V$OCT1_06	0.813672	7180	(+)	CWNAWTKWSATRYN	CTTCCCTAAATATC
M00136	V$OCT1_02	0.786952	7182	(-)	NNGAATATKCANNNN	TCCCTAAATATCCAG
M00136	V$OCT1_02	0.801841	7185	(+)	NNGAATATKCANNNN	CTAAATATCCAGTTG
M00162	V$OCT1_06	0.852734	7188	(-)	CWNAWTKWSATRYN	AATATCCAGTTGAG
M00162	V$OCT1_06	0.911719	7271	(+)	CWNAWTKWSATRYN	CAGTCTTGGATGCA
M00162	V$OCT1_06	0.804297	7307	(+)	CWNAWTKWSATRYN	CCAACCGTCATGTT
M00135	V$OCT1_01	0.742655	7391	(+)	NNNNWTATGCAAATNTNNN	AGAGATATGCCCATCCAGG
M00136	V$OCT1_02	0.787493	7391	(+)	NNGAATATKCANNNN	AGAGATATGCCCATC
M00162	V$OCT1_06	0.865625	7484	(+)	CWNAWTKWSATRYN	CTAACTGTGCTGTT

M00248	V$OCT1_07	0.823617	7512	(+)	TNTATGNTAATT	TGTATGATATTT
M00138	V$OCT1_04	0.798628	7513	(-)	NNNNNNNWATGCAAATNNNWNNW	GTATGATATTTGAGTAATTAA-GA
M00135	V$OCT1_01	0.797024	7515	(-)	NNNNWTATGCAAATNTNNN	ATGATATTTGAGTAATTAA
M00162	V$OCT1_06	0.826953	7517	(-)	CWNAWTKWSATRYN	GATATTTGAGTAAT
M00137	V$OCT1_03	0.905571	7523	(+)	NNNRTAATNANNN	TGAGTAATTAAGA
M00137	V$OCT1_03	0.840379	7524	(-)	NNNRTAATNANNN	GAGTAATTAAGAG
M00162	V$OCT1_06	0.821484	7545	(-)	CWNAWTKWSATRYN	AAAAATCAATAATG
M00137	V$OCT1_03	0.858949	7550	(+)	NNNRTAATNANNN	TCAATAATGAATG
M00138	V$OCT1_04	0.813258	7564	(-)	NNNNNNNWATGCAAATNNNWNNW	TTCCAAAATAAGCATAGCTGA-GA
M00135	V$OCT1_01	0.787676	7566	(-)	NNNNWTATGCAAATNTNNN	CCAAAATAAGCATAGCTGA
M00248	V$OCT1_07	0.868985	7570	(-)	TNTATGNTAATT	AATAAGCATAGC
M00162	V$OCT1_06	0.855078	7598	(+)	CWNAWTKWSATRYN	CAGCATTAGTTATA
M00137	V$OCT1_03	0.874753	7606	(+)	NNNRTAATNANNN	GTTATAAATAGAA
M00136	V$OCT1_02	0.791012	7611	(-)	NNGAATATKCANNNN	AAATAGAATAAGCTG
M00248	V$OCT1_07	0.793725	7611	(-)	TNTATGNTAATT	AAATAGAATAAG
M00162	V$OCT1_06	0.855469	7652	(-)	CWNAWTKWSATRYN	CAGATCTCAATGTC
M00162	V$OCT1_06	0.839844	7680	(+)	CWNAWTKWSATRYN	GAGATTCTGATTTA
M00162	V$OCT1_06	0.860547	7686	(+)	CWNAWTKWSATRYN	CTGATTTAACTGTT
M00162	V$OCT1_06	0.812109	7731	(+)	CWNAWTKWSATRYN	AATAATATCATGGA
M00162	V$OCT1_06	0.847656	7767	(+)	CWNAWTKWSATRYN	AGAACTTAGATGAA
M00248	V$OCT1_07	0.834429	7789	(-)	TNTATGNTAATT	AGTTACAATAAA
M00162	V$OCT1_06	0.821875	7803	(+)	CWNAWTKWSATRYN	CAGAATTGAACTTG
M00137	V$OCT1_03	0.879889	7814	(+)	NNNRTAATNANNN	TTGATAATCTCAT
M00162	V$OCT1_06	0.855469	7816	(+)	CWNAWTKWSATRYN	GATAATCTCATTTG
M00162	V$OCT1_06	0.817578	7817	(-)	CWNAWTKWSATRYN	ATAATCTCATTTGG
M00138	V$OCT1_04	0.788373	7818	(-)	NNNNNNNWATGCAAATNNNWNNW	TAATCTCATTTGGCTAAGAA-TAA
M00162	V$OCT1_06	0.800781	7834	(-)	CWNAWTKWSATRYN	AGAATAACAAACCT
M00138	V$OCT1_04	0.819113	7865	(-)	NNNNNNNWATGCAAATNNNWNNW	TCTACAATTTTGAAGTTTTCCTT
M00162	V$OCT1_06	0.813672	7869	(+)	CWNAWTKWSATRYN	CAATTTTGAAGTTT
M00162	V$OCT1_06	0.808203	7870	(+)	CWNAWTKWSATRYN	AATTTTGAAGTTTT
M00138	V$OCT1_04	0.785027	7879	(-)	NNNNNNNWATGCAAATNNNWNNW	GTTTTCCTTATGCACAATTATTT
M00137	V$OCT1_03	0.849072	7880	(-)	NNNRTAATNANNN	TTTTCCTTATGCA
M00135	V$OCT1_01	0.812285	7882	(+)	NNNNWTATGCAAATNTNNN	TTCCTTATGCACAATTATT

M00136	V$OCT1_02	0.797509	7882	(+)	NNGAATATKCANNNN	TTCCTTATGCACAAT
M00137	V$OCT1_03	0.868431	7889	(+)	NNNRTAATNANNN	TGCACAATTATTT
M00162	V$OCT1_06	0.839453	7925	(+)	CWNAWTKWSATRYN	TTGTTTTTGATATA
M00162	V$OCT1_06	0.834375	7927	(+)	CWNAWTKWSATRYN	GTTTTTGATATATG
M00135	V$OCT1_01	0.806372	7933	(-)	NNNNWTATGCAAATNTNNN	GATATATGAGCATATGAGG
M00162	V$OCT1_06	0.809375	7935	(-)	CWNAWTKWSATRYN	TATATGAGCATATG
M00136	V$OCT1_02	0.795885	7937	(-)	NNGAATATKCANNNN	TATGAGCATATGAGG
M00162	V$OCT1_06	0.945312	7956	(+)	CWNAWTKWSATRYN	AATACTGAGATGCT
M00138	V$OCT1_04	0.82476	7957	(+)	NNNNNNNWATGCAAATNNNWNNW	ATACTGAGATGCTTATTTCAA-TA
M00135	V$OCT1_01	0.747234	7959	(+)	NNNNWTATGCAAATNTNNN	ACTGAGATGCTTATTTCAA
M00135	V$OCT1_01	0.761541	7966	(-)	NNNNWTATGCAAATNTNNN	TGCTTATTTCAATACTCAG
M00162	V$OCT1_06	0.878906	7968	(+)	CWNAWTKWSATRYN	CTTATTTCAATACT
M00162	V$OCT1_06	0.826953	7968	(-)	CWNAWTKWSATRYN	CTTATTTCAATACT
M00195	V$OCT1_Q6	0.815502	7968	(-)	NNNNATGCAAATNAN	CTTATTTCAATACTC
M00136	V$OCT1_02	0.774228	7970	(-)	NNGAATATKCANNNN	TATTTCAATACTCAG
M00248	V$OCT1_07	0.796057	7970	(-)	TNTATGNTAATT	TATTTCAATACT
M00136	V$OCT1_02	0.787223	7973	(+)	NNGAATATKCANNNN	TTCAATACTCAGGGA
M00162	V$OCT1_06	0.855078	7987	(+)	CWNAWTKWSATRYN	AAAATTTTCTTGCC
M00135	V$OCT1_01	0.76612	8006	(-)	NNNNWTATGCAAATNTNNN	GCAAGAATTGTATAATTCA
M00136	V$OCT1_02	0.773416	8014	(+)	NNGAATATKCANNNN	TGTATAATTCATTCA
M00162	V$OCT1_06	0.821094	8015	(+)	CWNAWTKWSATRYN	GTATAATTCATTCA
M00162	V$OCT1_06	0.834375	8016	(-)	CWNAWTKWSATRYN	TATAATTCATTCAC
M00162	V$OCT1_06	0.844922	8029	(+)	CWNAWTKWSATRYN	CTTATTTTATTTTA
M00248	V$OCT1_07	0.831673	8034	(+)	TNTATGNTAATT	TTTATTTTATTT
M00138	V$OCT1_04	0.816813	8037	(-)	NNNNNNNWATGCAAATNNNWNNW	ATTTTATTTTTTAATTTTTAAGG
M00162	V$OCT1_06	0.834375	8041	(+)	CWNAWTKWSATRYN	TATTTTTTAATTTT
M00248	V$OCT1_07	0.831673	8041	(+)	TNTATGNTAATT	TATTTTTTAATT
M00162	V$OCT1_06	0.800391	8042	(+)	CWNAWTKWSATRYN	ATTTTTTAATTTTT
M00138	V$OCT1_04	0.784609	8044	(-)	NNNNNNNWATGCAAATNNNWNNW	TTTTTAATTTTTAAGGTCTAAGA
M00138	V$OCT1_04	0.7821	8062	(-)	NNNNNNNWATGCAAATNNNWNNW	TAAGAGGAT-TTCAATGTGAATGC
M00162	V$OCT1_06	0.821875	8066	(+)	CWNAWTKWSATRYN	AGGATTTCAATGTG
M00162	V$OCT1_06	0.881641	8066	(-)	CWNAWTKWSATRYN	AGGATTTCAATGTG
M00138	V$OCT1_04	0.809912	8126	(-)	NNNNNNNWATGCAAATNNNWNNW	ACTGATCATTTTTATAGTTAATA

M00195	V$OCT1_Q6	0.819596	8130	(-)	NNNNATGCAAATNAN	ATCATTTTTATAGTT
M00162	V$OCT1_06	0.808203	8138	(-)	CWNAWTKWSATRYN	TATAGTTAATATCT
M00138	V$OCT1_04	0.842744	8144	(-)	NNNNNNNWATGCAAATNNNWNNW	TAATATCTTTTACATTTAATTTT
M00162	V$OCT1_06	0.898437	8148	(+)	CWNAWTKWSATRYN	ATCTTTTACATTTA
M00162	V$OCT1_06	0.834375	8153	(+)	CWNAWTKWSATRYN	TTACATTTAATTTT
M00162	V$OCT1_06	0.953125	8154	(-)	CWNAWTKWSATRYN	TACATTTAATTTTC
M00138	V$OCT1_04	0.834588	8155	(-)	NNNNNNNWATGCAAATNNNWNNW	ACATTTAATTTTCCTGGATAA-GA
M00138	V$OCT1_04	0.790673	8184	(-)	NNNNNNNWATGCAAATNNNWNNW	AGTAGCAATTTCTATCAG-TATAC
M00135	V$OCT1_01	0.760015	8200	(-)	NNNNWTATGCAAATNTNNN	AGTATACCAGCATAAAGAT
M00137	V$OCT1_03	0.877914	8208	(+)	NNNRTAATNANNN	AGCATAAAGATTA
M00162	V$OCT1_06	0.809375	8208	(-)	CWNAWTKWSATRYN	AGCATAAAGATTAG
M00138	V$OCT1_04	0.834797	8215	(-)	NNNNNNNWATGCAAATNNNWNNW	AGATTAGTTTTAAATTTATTTTC
M00162	V$OCT1_06	0.829297	8219	(+)	CWNAWTKWSATRYN	TAGTTTTAAATTTA
M00162	V$OCT1_06	0.839453	8220	(-)	CWNAWTKWSATRYN	AGTTTTAAATTTAT
M00162	V$OCT1_06	0.889062	8259	(-)	CWNAWTKWSATRYN	GACCTGAAATTATG
M00162	V$OCT1_06	0.826562	8272	(+)	CWNAWTKWSATRYN	GTATCTGTTATATT
M00138	V$OCT1_04	0.797156	8275	(+)	NNNNNNNWATGCAAATNNNWNNW	TCTGTTATATTTCAAATAATGCA
M00162	V$OCT1_06	0.847266	8280	(-)	CWNAWTKWSATRYN	TATATTTCAAATAA
M00162	V$OCT1_06	0.860156	8282	(-)	CWNAWTKWSATRYN	TATTTCAAATAATG
M00138	V$OCT1_04	0.833961	8285	(+)	NNNNNNNWATGCAAATNNNWNNW	TTCAAATAATGCAAAACTG-TATA
M00135	V$OCT1_01	0.816864	8287	(+)	NNNNWTATGCAAATNTNNN	CAAATAATGCAAAACTGTA
M00195	V$OCT1_Q6	0.808679	8289	(+)	NNNNATGCAAATNAN	AATAATGCAAAACTG
M00162	V$OCT1_06	0.834766	8290	(-)	CWNAWTKWSATRYN	ATAATGCAAAACTG
M00138	V$OCT1_04	0.816813	8293	(+)	NNNNNNNWATGCAAATNNNWNNW	ATGCAAAACTG-TATATATATGGT
M00162	V$OCT1_06	0.804297	8296	(+)	CWNAWTKWSATRYN	CAAAACTGTATATA
M00138	V$OCT1_04	0.787327	8331	(-)	NNNNNNNWATGCAAATNNNWNNW	TGGTTTTCTTTCAATTGCCTATA
M00248	V$OCT1_07	0.800297	8350	(+)	TNTATGNTAATT	TATATCCTTATT
M00137	V$OCT1_03	0.841169	8351	(-)	NNNRTAATNANNN	ATATCCTTATTAT
M00137	V$OCT1_03	0.9032	8354	(-)	NNNRTAATNANNN	TCCTTATTATTGA
M00137	V$OCT1_03	0.919795	8366	(+)	NNNRTAATNANNN	ATTGTAATCATTT
M00138	V$OCT1_04	0.780008	8371	(+)	NNNNNNNWATGCAAATNNNWNNW	AATCATTTATA-GAAAAAACTGAA
M00138	V$OCT1_04	0.804266	8381	(+)	NNNNNNNWATGCAAATNNNWNNW	AGAAAAAACTGAAAA-TAATTTCT
M00195	V$OCT1_Q6	0.818777	8385	(+)	NNNNATGCAAATNAN	AAAACTGAAAATAAT

M00162	V$OCT1_06	0.839453	8386	(-)	CWNAWTKWSATRYN	AAACTGAAAATAAT
M00162	V$OCT1_06	0.867969	8394	(+)	CWNAWTKWSATRYN	AATAATTTCTTATA
M00162	V$OCT1_06	0.812109	8404	(+)	CWNAWTKWSATRYN	TATACTTTTATGTA
M00138	V$OCT1_04	0.780427	8405	(+)	NNNNNNNWATGCAAATNNNWNNW	ATACTTTTATGTAAACCTGTTAG
M00135	V$OCT1_01	0.760397	8407	(+)	NNNNWTATGCAAATNTNNN	ACTTTTATGTAAACCTGTT
M00162	V$OCT1_06	0.812109	8410	(-)	CWNAWTKWSATRYN	TTTATGTAAACCTG
M00162	V$OCT1_06	0.821484	8442	(+)	CWNAWTKWSATRYN	ATCAACTGCATTCA
M00162	V$OCT1_06	0.834375	8448	(-)	CWNAWTKWSATRYN	TGCATTCACATTTC
M00195	V$OCT1_Q6	0.798035	8448	(-)	NNNNATGCAAATNAN	TGCATTCACATTTCT
M00137	V$OCT1_03	0.948637	8467	(-)	NNNRTAATNANNN	TAGTCATTATGAG
M00162	V$OCT1_06	0.855078	8471	(+)	CWNAWTKWSATRYN	CATTATGAGCTTCA
M00138	V$OCT1_04	0.782936	8486	(-)	NNNNNNNWATGCAAATNNNWNNW	TTGTTTTATCTCACTTAAAATTT
M00138	V$OCT1_04	0.791301	8491	(+)	NNNNNNNWATGCAAATNNNWNNW	TTATCTCACTTAAAATTTATATA
M00138	V$OCT1_04	0.784609	8492	(-)	NNNNNNNWATGCAAATNNNWNNW	TATCTCACTTAAAATTTATATAT
M00162	V$OCT1_06	0.826953	8495	(+)	CWNAWTKWSATRYN	CTCACTTAAAATTT
M00138	V$OCT1_04	0.832915	8498	(-)	NNNNNNNWATGCAAATNNNWNNW	ACTTAAAATTTATATATTGTCTT
M00135	V$OCT1_01	0.805799	8500	(-)	NNNNWTATGCAAATNTNNN	TTAAAATTTATATATTGTC
M00138	V$OCT1_04	0.788164	8500	(-)	NNNNNNNWATGCAAATNNNWNNW	TTAAAATTTATATATTGTCTTTT
M00162	V$OCT1_06	0.88125	8502	(+)	CWNAWTKWSATRYN	AAAATTTATATATT
M00162	V$OCT1_06	0.834375	8502	(-)	CWNAWTKWSATRYN	AAAATTTATATATT
M00248	V$OCT1_07	0.848209	8504	(-)	TNTATGNTAATT	AATTTATATATT
M00162	V$OCT1_06	0.813672	8515	(+)	CWNAWTKWSATRYN	TGTCTTTTAATTCA
M00248	V$OCT1_07	0.836549	8515	(+)	TNTATGNTAATT	TGTCTTTTAATT
M00162	V$OCT1_06	0.813672	8534	(-)	CWNAWTKWSATRYN	CAAAATACAATTTC
M00135	V$OCT1_01	0.804273	8571	(-)	NNNNWTATGCAAATNTNNN	AGGGGAATAGCATATAGTT
M00162	V$OCT1_06	0.834766	8573	(+)	CWNAWTKWSATRYN	GGGAATAGCATATA
M00195	V$OCT1_Q6	0.823963	8573	(-)	NNNNATGCAAATNAN	GGGAATAGCATATAG
M00136	V$OCT1_02	0.79399	8575	(-)	NNGAATATKCANNNN	GAATAGCATATAGTT
M00248	V$OCT1_07	0.799237	8575	(-)	TNTATGNTAATT	GAATAGCATATA
M00162	V$OCT1_06	0.832812	8579	(-)	CWNAWTKWSATRYN	AGCATATAGTTTTG
M00162	V$OCT1_06	0.852344	8584	(+)	CWNAWTKWSATRYN	ATAGTTTTGATATT
M00136	V$OCT1_02	0.780184	8586	(-)	NNGAATATKCANNNN	AGTTTTGATATTCTT
M00162	V$OCT1_06	0.821484	8586	(+)	CWNAWTKWSATRYN	AGTTTTGATATTCT

M00138	V$OCT1_04	0.800084	8587	(+)	NNNNNNNWATGCAAATNNNWNNW	GTTTTGATATTCTTAAAGATATA
M00136	V$OCT1_02	0.800758	8589	(+)	NNGAATATKCANNNN	TTTGATATTCTTAAA
M00137	V$OCT1_03	0.860134	8596	(+)	NNNRTAATNANNN	TTCTTAAAGATAT
M00138	V$OCT1_04	0.821832	8598	(-)	NNNNNNNWATGCAAATNNNWNNW	CTTAAAGATATACATCTTTTTGT
M00162	V$OCT1_06	0.817187	8598	(+)	CWNAWTKWSATRYN	CTTAAAGATATACA
M00162	V$OCT1_06	0.812109	8608	(-)	CWNAWTKWSATRYN	TACATCTTTTTGTG
M00138	V$OCT1_04	0.789837	8631	(+)	NNNNNNNWATGCAAATNNNWNNW	CAGCAGACATTTTAA-TAAAACAA
M00138	V$OCT1_04	0.806148	8632	(-)	NNNNNNNWATGCAAATNNNWNNW	AGCAGACATTTTAA-TAAAACAAT
M00135	V$OCT1_01	0.752385	8634	(-)	NNNNWTATGCAAATNTNNN	CAGACATTTTAATAAAACA
M00162	V$OCT1_06	0.839844	8636	(+)	CWNAWTKWSATRYN	GACATTTTAATAAA
M00162	V$OCT1_06	0.80625	8651	(-)	CWNAWTKWSATRYN	CAATTCCAAGTGAG
M00136	V$OCT1_02	0.789659	8679	(-)	NNGAATATKCANNNN	TAGAGGAATTTTTAT
M00138	V$OCT1_04	0.831033	8679	(-)	NNNNNNNWATGCAAATNNNWNNW	TAGAGGAATTTTTATAACCTTTA
M00135	V$OCT1_01	0.742655	8681	(-)	NNNNWTATGCAAATNTNNN	GAGGAATTTTTATAACCTT
M00195	V$OCT1_Q6	0.803493	8683	(-)	NNNNATGCAAATNAN	GGAATTTTTATAACC
M00138	V$OCT1_04	0.7821	8717	(-)	NNNNNNNWATGCAAATNNNWNNW	TGGTGTTTTTGTAATAAGATTTC
M00162	V$OCT1_06	0.805859	8721	(+)	CWNAWTKWSATRYN	GTTTTTGTAATAAG
M00137	V$OCT1_03	0.941525	8724	(+)	NNNRTAATNANNN	TTTGTAATAAGAT
M00138	V$OCT1_04	0.783354	8736	(+)	NNNNNNNWATGCAAATNNNWNNW	TTTCTTTTATGAAAAAGTCACAC
M00135	V$OCT1_01	0.779855	8738	(+)	NNNNWTATGCAAATNTNNN	TCTTTTATGAAAAAGTCAC
M00162	V$OCT1_06	0.834375	8741	(-)	CWNAWTKWSATRYN	TTTATGAAAAAGTC
M00162	V$OCT1_06	0.860547	8759	(+)	CWNAWTKWSATRYN	CAAAATTGGAAATG
M00162	V$OCT1_06	0.842187	8786	(+)	CWNAWTKWSATRYN	AGTTATAACATATA
M00138	V$OCT1_04	0.782309	8788	(-)	NNNNNNNWATGCAAATNNNWNNW	TTATAACATA-TAACTGAATGTAC
M00136	V$OCT1_02	0.814293	8798	(-)	NNGAATATKCANNNN	TAACTGAATGTACAT
M00162	V$OCT1_06	0.88125	8802	(-)	CWNAWTKWSATRYN	TGAATGTACATTTG
M00248	V$OCT1_07	0.817257	8802	(+)	TNTATGNTAATT	TGAATGTACATT
M00162	V$OCT1_06	0.842578	8828	(+)	CWNAWTKWSATRYN	CAGAATTGACTGCG
M00162	V$OCT1_06	0.813672	8840	(+)	CWNAWTKWSATRYN	CGACTGGAAATATG
M00162	V$OCT1_06	0.834375	8855	(+)	CWNAWTKWSATRYN	CAACTTTTCAATCC
M00162	V$OCT1_06	0.821484	8856	(-)	CWNAWTKWSATRYN	AACTTTTCAATCCT
M00135	V$OCT1_01	0.73884	8862	(-)	NNNNWTATGCAAATNTNNN	TCAATCCTTGCATCATGCT
M00135	V$OCT1_01	0.749332	8870	(+)	NNNNWTATGCAAATNTNNN	TGCATCATGCTACTAAGAT

M00248	V$OCT1_07	0.804325	8881	(+)	TNTATGNTAATT	ACTAAGATAATT
M00138	V$OCT1_04	0.83898	8885	(-)	NNNNNNNWATGCAAATNNNWNNW	AGATAATTTTAAATGAG-TATAC
M00162	V$OCT1_06	0.829297	8889	(+)	CWNAWTKWSATRYN	AATTTTTAAATGAG
M00162	V$OCT1_06	0.852734	8889	(-)	CWNAWTKWSATRYN	AATTTTTAAATGAG
M00135	V$OCT1_01	0.732736	8893	(-)	NNNNWTATGCAAATNTNNN	TTTAAATGAGTATACATGG
M00136	V$OCT1_02	0.832972	8895	(-)	NNGAATATKCANNNN	TAAATGAGTATACAT
M00248	V$OCT1_07	0.805173	8897	(-)	TNTATGNTAATT	AATGAGTATACA
M00136	V$OCT1_02	0.799134	8898	(+)	NNGAATATKCANNNN	ATGAGTATACATGGA
M00162	V$OCT1_06	0.842187	8916	(+)	CWNAWTKWSATRYN	AAAAATGAAACTTT
M00162	V$OCT1_06	0.812109	8917	(-)	CWNAWTKWSATRYN	AAAATGAAACTTTA
M00138	V$OCT1_04	0.780427	8921	(-)	NNNNNNNWATGCAAATNNNWNNW	TGAAACTTTATTCCTTTATTTAT
M00162	V$OCT1_06	0.826562	8934	(+)	CWNAWTKWSATRYN	CTTTATTTATTTTA
M00162	V$OCT1_06	0.855078	8938	(+)	CWNAWTKWSATRYN	ATTTATTTTATGCT
M00136	V$OCT1_02	0.791825	8944	(-)	NNGAATATKCANNNN	TTTATGCTTTTTCAT
M00138	V$OCT1_04	0.840234	8944	(-)	NNNNNNNWATGCAAATNNNWNNW	TTTATGCTTTTTCATCTTAATTT
M00248	V$OCT1_07	0.799237	8944	(+)	TNTATGNTAATT	TTTATGCTTTTT
M00138	V$OCT1_04	0.783772	8950	(-)	NNNNNNNWATGCAAATNNNWNNW	CTTTTTCATCTTAATTTGAATTT
M00162	V$OCT1_06	0.830469	8954	(-)	CWNAWTKWSATRYN	TTCATCTTAATTTG
M00248	V$OCT1_07	0.837397	8954	(+)	TNTATGNTAATT	TTCATCTTAATT
M00138	V$OCT1_04	0.815767	8956	(-)	NNNNNNNWATGCAAATNNNWNNW	CATCTTAATTTGAATTTGAGTCA
M00135	V$OCT1_01	0.746852	8958	(-)	NNNNWTATGCAAATNTNNN	TCTTAATTTGAATTTGAGT
M00162	V$OCT1_06	0.865625	8959	(+)	CWNAWTKWSATRYN	CTTAATTTGAATTT
M00162	V$OCT1_06	0.826953	8960	(+)	CWNAWTKWSATRYN	TTAATTTGAATTTG
M00136	V$OCT1_02	0.798051	8962	(-)	NNGAATATKCANNNN	AATTTGAATTTGAGT
M00138	V$OCT1_04	0.801129	8962	(-)	NNNNNNNWATGCAAATNNNWNNW	AATTTGAATTTGAG-TCATAAACC
M00248	V$OCT1_07	0.813653	8962	(-)	TNTATGNTAATT	AATTTGAATTTG
M00162	V$OCT1_06	0.814062	8966	(-)	CWNAWTKWSATRYN	TGAATTTGAGTCAT
M00138	V$OCT1_04	0.81514	8990	(+)	NNNNNNNWATGCAAATNNNWNNW	CTTTCAAAATGTTAATTCAACAT
M00135	V$OCT1_01	0.806753	8992	(+)	NNNNWTATGCAAATNTNNN	TTCAAAATGTTAATTCAAC
M00195	V$OCT1_Q6	0.796124	8994	(+)	NNNNATGCAAATNAN	CAAAATGTTAATTCA
M00162	V$OCT1_06	0.826562	8995	(+)	CWNAWTKWSATRYN	AAAATGTTAATTCA
M00248	V$OCT1_07	0.914988	8995	(+)	TNTATGNTAATT	AAAATGTTAATT
M00138	V$OCT1_04	0.795483	8996	(+)	NNNNNNNWATGCAAATNNNWNNW	AAATGTTAATTCAACATTAG-CAT

M00138	V$OCT1_04	0.820368	8998	(-)	NNNNNNNWATGCAAATNNNWNNW	ATGTTAATTCAACATTAG-CATAA
M00138	V$OCT1_04	0.842325	9004	(-)	NNNNNNNWATGCAAATNNNWNNW	ATTCAACATTAG-CATAAAAGTTC
M00135	V$OCT1_01	0.889164	9006	(-)	NNNNWTATGCAAATNTNNN	TCAACATTAGCATAAAAGT
M00137	V$OCT1_03	0.851442	9006	(-)	NNNRTAATNANNN	TCAACATTAGCAT
M00248	V$OCT1_07	0.89718	9010	(-)	TNTATGNTAATT	CATTAGCATAAA
M00162	V$OCT1_06	0.821484	9019	(-)	CWNAWTKWSATRYN	AAAAGTTCAGTTTT
M00138	V$OCT1_04	0.794437	9028	(+)	NNNNNNNWATGCAAATNNNWNNW	GTTTTAACTTGGAAA-TATCATGA
M00135	V$OCT1_01	0.740557	9030	(+)	NNNNWTATGCAAATNTNNN	TTTAACTTGGAAATATCAT
M00162	V$OCT1_06	0.829297	9039	(-)	CWNAWTKWSATRYN	GAAATATCATGAAC
M00138	V$OCT1_04	0.794437	9047	(+)	NNNNNNNWATGCAAATNNNWNNW	ATGAACATATCAAAA-TATGTATA
M00138	V$OCT1_04	0.805312	9048	(-)	NNNNNNNWATGCAAATNNNWNNW	TGAACATATCAAAA-TATGTATAA
M00135	V$OCT1_01	0.756391	9049	(+)	NNNNWTATGCAAATNTNNN	GAACATATCAAAATATGTA
M00162	V$OCT1_06	0.806641	9050	(-)	CWNAWTKWSATRYN	AACATATCAAAATA
M00162	V$OCT1_06	0.955859	9052	(-)	CWNAWTKWSATRYN	CATATCAAAATATG
M00138	V$OCT1_04	0.862401	9054	(-)	NNNNNNNWATGCAAATNNNWNNW	TATCAAAATATGTATAAAAA-TAA
M00138	V$OCT1_04	0.824132	9055	(+)	NNNNNNNWATGCAAATNNNWNNW	ATCAAAATATGTATAAAAA-TAAT
M00135	V$OCT1_01	0.763449	9056	(-)	NNNNWTATGCAAATNTNNN	TCAAAATATGTATAAAAAT
M00136	V$OCT1_02	0.814835	9057	(+)	NNGAATATKCANNNN	CAAAATATGTATAAA
M00138	V$OCT1_04	0.843789	9059	(+)	NNNNNNNWATGCAAATNNNWNNW	AAATATGTATAAAAA-TAATTTCT
M00138	V$OCT1_04	0.795483	9060	(-)	NNNNNNNWATGCAAATNNNWNNW	AATATGTATAAAAA-TAATTTCTG
M00195	V$OCT1_Q6	0.837882	9063	(+)	NNNNATGCAAATNAN	ATGTATAAAAATAAT
M00137	V$OCT1_03	0.854998	9064	(+)	NNNRTAATNANNN	TGTATAAAAATAA
M00162	V$OCT1_06	0.865625	9064	(-)	CWNAWTKWSATRYN	TGTATAAAAATAAT
M00162	V$OCT1_06	0.832031	9082	(+)	CWNAWTKWSATRYN	GGAATTGTGATTAT
M00136	V$OCT1_02	0.80536	9085	(-)	NNGAATATKCANNNN	ATTGTGATTATTATT
M00137	V$OCT1_03	0.904781	9086	(-)	NNNRTAATNANNN	TTGTGATTATTAT
M00195	V$OCT1_Q6	0.808133	9088	(-)	NNNNATGCAAATNAN	GTGATTATTATTTCT
M00137	V$OCT1_03	0.854208	9089	(-)	NNNRTAATNANNN	TGATTATTATTTC
M00137	V$OCT1_03	0.840774	9097	(-)	NNNRTAATNANNN	ATTTCTTTAAGAA
M00162	V$OCT1_06	0.821484	9106	(-)	CWNAWTKWSATRYN	AGAATCTATTTCCT
M00138	V$OCT1_04	0.820159	9121	(-)	NNNNNNNWATGCAAATNNNWNNW	ACCAGTCATTTCAA-TAAATTAAC
M00135	V$OCT1_01	0.740557	9123	(-)	NNNNWTATGCAAATNTNNN	CAGTCATTTCAATAAATTA
M00248	V$OCT1_07	0.810473	9127	(-)	TNTATGNTAATT	CATTTCAATAAA

M00059	V$YY1_01	0.776868	81	(+)	NNNNNCCATNTWNNNWN	GCTACTCATCTTTATTT
M00059	V$YY1_01	0.781127	185	(-)	NNNNNCCATNTWNNNWN	CAGCAAATATGTTAAAA
M00059	V$YY1_01	0.774902	195	(-)	NNNNNCCATNTWNNNWN	GTTAAAAAATAGAAGTG
M00059	V$YY1_01	0.81848	400	(-)	NNNNNCCATNTWNNNWN	TTTATACTATGGGGATT
M00059	V$YY1_01	0.797837	461	(+)	NNNNNCCATNTWNNNWN	AGCTAGCATATTTAGTC
M00059	V$YY1_01	0.780144	759	(-)	NNNNNCCATNTWNNNWN	GTAATGAAATGGCTTAA
M00059	V$YY1_01	0.776212	783	(-)	NNNNNCCATNTWNNNWN	TATATAAAATGCTAGTT
M00059	V$YY1_01	0.826343	827	(-)	NNNNNCCATNTWNNNWN	CTCCCTACATGTAATGA
M00059	V$YY1_01	0.787025	828	(+)	NNNNNCCATNTWNNNWN	TCCCTACATGTAATGAA
M00059	V$YY1_01	0.789974	901	(+)	NNNNNCCATNTWNNNWN	TATAGACATCTTGAATA
M00059	V$YY1_01	0.80308	980	(-)	NNNNNCCATNTWNNNWN	TTGAAAATCTGGGATTG
M00059	V$YY1_01	0.800131	1006	(-)	NNNNNCCATNTWNNNWN	GTCAAGAGATGTTGGTG
M00069	V$YY1_02	0.7608	1007	(-)	NNNCGGCCATCTTGNCTSNW	TCAAGAGATGTTGGTGGCAA
M00059	V$YY1_01	0.818807	1024	(+)	NNNNNCCATNTWNNNWN	CAAGAACATTTTTTTTT
M00059	V$YY1_01	0.824705	1066	(+)	NNNNNCCATNTWNNNWN	ACAAACCATTTAATACA
M00059	V$YY1_01	0.776868	1219	(-)	NNNNNCCATNTWNNNWN	CATAGTGTATGATCTCT
M00059	V$YY1_01	0.777195	1252	(-)	NNNNNCCATNTWNNNWN	AAGGAGAGATGACATTA
M00059	V$YY1_01	0.78637	1354	(-)	NNNNNCCATNTWNNNWN	ATGCTAAAATGAAACAG
M00059	V$YY1_01	0.815531	1448	(-)	NNNNNCCATNTWNNNWN	AATAAAGTATGACCTTC
M00059	V$YY1_01	0.773263	1461	(+)	NNNNNCCATNTWNNNWN	CTTCTTCATATACATTG
M00059	V$YY1_01	0.797182	1467	(+)	NNNNNCCATNTWNNNWN	CATATACATTGTTTGAT
M00059	V$YY1_01	0.792595	1564	(-)	NNNNNCCATNTWNNNWN	AGCGATATCTGGCAATA
M00059	V$YY1_01	0.777523	1802	(+)	NNNNNCCATNTWNNNWN	TTTGGCCATATGATAAA
M00059	V$YY1_01	0.81291	1811	(-)	NNNNNCCATNTWNNNWN	ATGATAAAATGTTTATT
M00069	V$YY1_02	0.772046	1857	(+)	NNNCGGCCATCTTGNCTSNW	TTTTTGCCATGAGGTTTGCA
M00059	V$YY1_01	0.85059	1917	(-)	NNNNNCCATNTWNNNWN	CTGGAAGCATGGAGTCT
M00059	V$YY1_01	0.776868	2024	(+)	NNNNNCCATNTWNNNWN	TTTCCTCATCTTCCCAT
M00059	V$YY1_01	0.790957	2181	(+)	NNNNNCCATNTWNNNWN	CAACATCATAAATGTAT
M00059	V$YY1_01	0.791612	2184	(-)	NNNNNCCATNTWNNNWN	CATCATAAATGTATAAA
M00059	V$YY1_01	0.847969	2236	(+)	NNNNNCCATNTWNNNWN	GAAAGTCATTTATGTTC
M00059	V$YY1_01	0.793578	2246	(+)	NNNNNCCATNTWNNNWN	TATGTTCATCCACTATC
M00059	V$YY1_01	0.777523	2490	(-)	NNNNNCCATNTWNNNWN	CTTACTGTAGGGTGCTA
M00059	V$YY1_01	0.778506	2575	(+)	NNNNNCCATNTWNNNWN	TATGTTCATAATTATAA

M00059	V$YY1_01	0.771625	2787	(-)	NNNNNCCATNTWNNNWN	ATTTAATAATGAGTTGA
M00059	V$YY1_01	0.783093	3014	(+)	NNNNNCCATNTWNNNWN	AATGTTCATTCAGAGGC
M00059	V$YY1_01	0.802097	3077	(-)	NNNNNCCATNTWNNNWN	TTACATTAATGGCAACC
M00059	V$YY1_01	0.783093	3220	(-)	NNNNNCCATNTWNNNWN	AGAAGGAAATGGCAACC
M00059	V$YY1_01	0.780144	3236	(+)	NNNNNCCATNTWNNNWN	CCACTCCAGTGTTCTTG
M00069	V$YY1_02	0.799179	3273	(+)	NNNCGGCCATCTTGNCTSNW	GGGGAGCCTTGTAGGCTGCT
M00059	V$YY1_01	0.790629	3406	(+)	NNNNNCCATNTWNNNWN	GAAGACCCTGGTTCGAT
M00059	V$YY1_01	0.773263	3456	(+)	NNNNNCCATNTWNNNWN	CCACTCCAGTGTTCTTT
M00059	V$YY1_01	0.882045	3569	(-)	NNNNNCCATNTWNNNWN	ATTTCTAAATGGGTTTG
M00059	V$YY1_01	0.790629	3619	(-)	NNNNNCCATNTWNNNWN	TTTCCTTAATGAACTGT
M00059	V$YY1_01	0.778178	3684	(+)	NNNNNCCATNTWNNNWN	CTTTGGCATTTATTGAG
M00059	V$YY1_01	0.789646	3742	(-)	NNNNNCCATNTWNNNWN	CTGGAAAGAGGGATGCA
M00059	V$YY1_01	0.796527	3778	(-)	NNNNNCCATNTWNNNWN	ACATGCAAATGGACATA
M00059	V$YY1_01	0.820446	3785	(+)	NNNNNCCATNTWNNNWN	AATGGACATATTTTATT
M00059	V$YY1_01	0.771625	3831	(-)	NNNNNCCATNTWNNNWN	CATTGAGAATGAGCGTA
M00059	V$YY1_01	0.777851	4023	(+)	NNNNNCCATNTWNNNWN	CGACCCCATAGACGGCA
M00059	V$YY1_01	0.780144	4076	(-)	NNNNNCCATNTWNNNWN	CAAGAACACTGGAGTGG
M00059	V$YY1_01	0.783093	4092	(+)	NNNNNCCATNTWNNNWN	GGTTGCCATTTCCTTCT
M00059	V$YY1_01	0.783421	4120	(-)	NNNNNCCATNTWNNNWN	AAGTGAAAAGGGAAAGT
M00059	V$YY1_01	0.78768	4196	(-)	NNNNNCCATNTWNNNWN	CTCTGTCCATGGGATTT
M00059	V$YY1_01	0.801769	4317	(-)	NNNNNCCATNTWNNNWN	AACTAAGAATGATTATT
M00059	V$YY1_01	0.830603	4389	(+)	NNNNNCCATNTWNNNWN	AGACAGCATATTCAAAA
M00069	V$YY1_02	0.763834	4428	(+)	NNNCGGCCATCTTGNCTSNW	TAAGGTCCATCTAGTCAAGG
M00059	V$YY1_01	0.853211	4429	(+)	NNNNNCCATNTWNNNWN	AAGGTCCATCTAGTCAA
M00059	V$YY1_01	0.829292	4461	(+)	NNNNNCCATNTWNNNWN	AGTAGTCATGTATGGAT
M00059	V$YY1_01	0.796199	4464	(-)	NNNNNCCATNTWNNNWN	AGTCATGTATGGATGTG
M00059	V$YY1_01	0.784731	4512	(-)	NNNNNCCATNTWNNNWN	AAGAATAGATGCTTTTG
M00069	V$YY1_02	0.788647	4751	(-)	NNNCGGCCATCTTGNCTSNW	AGATGGCTGGATGGCATCAC
M00059	V$YY1_01	0.801114	4753	(-)	NNNNNCCATNTWNNNWN	ATGGCTGGATGGCATCA
M00069	V$YY1_02	0.778829	4768	(-)	NNNCGGCCATCTTGNCTSNW	CACTGACTCGATGGACGTGA
M00059	V$YY1_01	0.818807	4770	(-)	NNNNNCCATNTWNNNWN	CTGACTCGATGGACGTG
M00059	V$YY1_01	0.777195	4984	(+)	NNNNNCCATNTWNNNWN	AGAGATCATTTGTTTTC
M00059	V$YY1_01	0.81979	5006	(-)	NNNNNCCATNTWNNNWN	GAGAAAACAGGGTGATT

M00059	V$YY1_01	0.775557	5071	(-)	NNNNNCCATNTWNNNWN	ATGACCCTATGGACTGT
M00059	V$YY1_01	0.803408	5102	(-)	NNNNNCCATNTWNNNWN	CTCTGTCTATGGGATTT
M00059	V$YY1_01	0.771625	5362	(-)	NNNNNCCATNTWNNNWN	ATAAAATAATGATATCA
M00059	V$YY1_01	0.860419	5387	(+)	NNNNNCCATNTWNNNWN	GCTGTCCATATTAACCT
M00059	V$YY1_01	0.898427	5410	(+)	NNNNNCCATNTWNNNWN	TGTCTTCATTTTCTGAT
M00059	V$YY1_01	0.791612	5433	(-)	NNNNNCCATNTWNNNWN	TGACAAATATGAATTTT
M00059	V$YY1_01	0.886632	5519	(+)	NNNNNCCATNTWNNNWN	AACCTTCATATTTAAAC
M00059	V$YY1_01	0.832569	5533	(+)	NNNNNCCATNTWNNNWN	AACGTACATTTTTAAAA
M00059	V$YY1_01	0.792595	5570	(+)	NNNNNCCATNTWNNNWN	AAACAGCATTTCTTTAT
M00059	V$YY1_01	0.895806	5730	(-)	NNNNNCCATNTWNNNWN	GTCTAAGAATGGTAATA
M00059	V$YY1_01	0.867955	5774	(-)	NNNNNCCATNTWNNNWN	TTGACAAAATGAAAATA
M00059	V$YY1_01	0.847641	5802	(+)	NNNNNCCATNTWNNNWN	GAAACACATTTTTAAAC
M00059	V$YY1_01	0.830931	5869	(+)	NNNNNCCATNTWNNNWN	TATATTCATTTTACTAA
M00059	V$YY1_01	0.82536	5899	(-)	NNNNNCCATNTWNNNWN	TTTAATAAATGCATATT
M00059	V$YY1_01	0.816186	5904	(+)	NNNNNCCATNTWNNNWN	TAAATGCATATTAGTAG
M00059	V$YY1_01	0.827654	5955	(-)	NNNNNCCATNTWNNNWN	TTCTAATGATGGATATA
M00059	V$YY1_01	0.770642	6006	(+)	NNNNNCCATNTWNNNWN	AATCCTCATTTGATCAT
M00059	V$YY1_01	0.804718	6014	(+)	NNNNNCCATNTWNNNWN	TTTGATCATTTATCAGC
M00059	V$YY1_01	0.801114	6130	(-)	NNNNNCCATNTWNNNWN	ACTATTCAATGGTTTTA
M00059	V$YY1_01	0.807012	6168	(+)	NNNNNCCATNTWNNNWN	TACTCACATTTATGAAT
M00059	V$YY1_01	0.780472	6259	(+)	NNNNNCCATNTWNNNWN	TGTGACCATACCACATA
M00059	V$YY1_01	0.857798	6440	(-)	NNNNNCCATNTWNNNWN	AAGTCAAAATGAATTTA
M00059	V$YY1_01	0.792595	6518	(+)	NNNNNCCATNTWNNNWN	GTTGGCCATTGTTAAGG
M00059	V$YY1_01	0.793578	6551	(+)	NNNNNCCATNTWNNNWN	AAAACACATATTAAGAC
M00059	V$YY1_01	0.780144	6570	(+)	NNNNNCCATNTWNNNWN	AGTTTCCATTAAAACAA
M00059	V$YY1_01	0.830275	6639	(-)	NNNNNCCATNTWNNNWN	TTTGTTATATGAAATCA
M00059	V$YY1_01	0.778834	6648	(+)	NNNNNCCATNTWNNNWN	TGAAATCAGTTTGGACT
M00059	V$YY1_01	0.779161	6822	(-)	NNNNNCCATNTWNNNWN	GTCTTCATATGACCCCA
M00069	V$YY1_02	0.756337	6851	(-)	NNNCGGCCATCTTGNCTSNW	ACTAAACCAAATGGAAGATT
M00059	V$YY1_01	0.797182	6936	(-)	NNNNNCCATNTWNNNWN	ACTGGATTATGGACTCA
M00059	V$YY1_01	0.818807	6970	(-)	NNNNNCCATNTWNNNWN	TTTCCAGGATGAACTCC
M00059	V$YY1_01	0.781782	6980	(+)	NNNNNCCATNTWNNNWN	GAACTCCAGGATAAAAT
M00059	V$YY1_01	0.807339	7119	(-)	NNNNNCCATNTWNNNWN	CTGAAATAATGGGAGTC

M0006		0.77026				
9	V$YY1_02	1	7305	(+)	NNNCGGCCATCTTGNCTSNW	CTCCAACCGTCATGTTTCCT
M0006		0.79810				
9	V$YY1_02	8	7308	(+)	NNNCGGCCATCTTGNCTSNW	CAACCGTCATGTTTCCTCCT
M0005		0.82634				
9	V$YY1_01	3	7309	(+)	NNNNNCCATNTWNNNWN	AACCGTCATGTTTCCTC
M0005		0.78374				
9	V$YY1_01	8	7336	(+)	NNNNNCCATNTWNNNWN	GCTGTCCCTTTCTCAGC
M0005		0.77195				
9	V$YY1_01	3	7396	(+)	NNNNNCCATNTWNNNWN	TATGCCCATCCAGGCCT
M0005						
9	V$YY1_01	0.81848	7552	(-)	NNNNNCCATNTWNNNWN	AATAATGAATGGTTCCA
M0005		0.81651				
9	V$YY1_01	4	7646	(+)	NNNNNCCATNTWNNNWN	CACCACCAGATCTCAAT
M0005		0.77195				
9	V$YY1_01	3	7733	(+)	NNNNNCCATNTWNNNWN	TAATATCATGGACTATG
M0005		0.79390				
9	V$YY1_01	6	7768	(-)	NNNNNCCATNTWNNNWN	GAACTTAGATGAAGGGG
M0005		0.79947				
9	V$YY1_01	6	7797	(+)	NNNNNCCATNTWNNNWN	TAAAGCCAGAATTGAAC
M0005		0.84436				
9	V$YY1_01	4	7930	(-)	NNNNNCCATNTWNNNWN	TTTGATATATGAGCATA
M0005		0.85747				
9	V$YY1_01	1	8126	(+)	NNNNNCCATNTWNNNWN	ACTGATCATTTTTATAG
M0005		0.77064				
9	V$YY1_01	2	8231	(+)	NNNNNCCATNTWNNNWN	TATTTTCAGTGATTGAC
M0005						
9	V$YY1_01	0.79751	8293	(-)	NNNNNCCATNTWNNNWN	ATGCAAAACTGTATATA
M0005		0.91644				
9	V$YY1_01	8	8303	(-)	NNNNNCCATNTWNNNWN	GTATATATATGGTGTTG
M0005		0.86271				
9	V$YY1_01	3	8368	(+)	NNNNNCCATNTWNNNWN	TGTAATCATTTATAGAA
M0005		0.79849				
9	V$YY1_01	3	8548	(+)	NNNNNCCATNTWNNNWN	ACAGTCCAGATATGGGA
M0005		0.83813				
9	V$YY1_01	9	8551	(-)	NNNNNCCATNTWNNNWN	GTCCAGATATGGGACTT
M0005		0.79259				
9	V$YY1_01	5	8563	(-)	NNNNNCCATNTWNNNWN	GACTTAAAAGGGGAATA
M0005		0.78604				
9	V$YY1_01	2	8575	(+)	NNNNNCCATNTWNNNWN	GAATAGCATATAGTTTT
M0005		0.82699				
9	V$YY1_01	9	8708	(-)	NNNNNCCATNTWNNNWN	GGCACAGCATGGTGTTT
M0005		0.78833				
9	V$YY1_01	6	8756	(-)	NNNNNCCATNTWNNNWN	CACCAAAATTGGAAATG
M0005		0.84469				
9	V$YY1_01	2	8762	(-)	NNNNNCCATNTWNNNWN	AATTGGAAATGGGGTGA
M0005		0.77686				
9	V$YY1_01	8	8804	(+)	NNNNNCCATNTWNNNWN	AATGTACATTTGTTCTC
M0005		0.88597				
9	V$YY1_01	6	8843	(-)	NNNNNCCATNTWNNNWN	CTGGAAATATGGCAACT
M0005		0.82667				
9	V$YY1_01	1	8890	(-)	NNNNNCCATNTWNNNWN	ATTTTTAAATGAGTATA
M0006		0.75544				
9	V$YY1_02	4	8898	(-)	NNNCGGCCATCTTGNCTSNW	ATGAGTATACATGGAACAAA
M0005		0.80275				
9	V$YY1_01	2	8900	(-)	NNNNNCCATNTWNNNWN	GAGTATACATGGAACAA
M0005		0.83912				
9	V$YY1_01	2	8912	(-)	NNNNNCCATNTWNNNWN	AACAAAAAATGAAACTT
M0005		0.79030				
9	V$YY1_01	1	8978	(+)	NNNNNCCATNTWNNNWN	ATAAACCATATACTTTC
M0005		0.84829				
9	V$YY1_01	6	8990	(-)	NNNNNCCATNTWNNNWN	CTTTCAAAATGTTAATT
M0005		0.78276				
9	V$YY1_01	5	9020	(+)	NNNNNCCATNTWNNNWN	AAAGTTCAGTTTTAACT
M0005		0.79226				
9	V$YY1_01	7	9046	(+)	NNNNNCCATNTWNNNWN	CATGAACATATCAAAAT

M00059	V$YY1_01	0.79325	9055	(-)	NNNNNCCATNTWNNNWN	ATCAAAATATGTATAAA
CSN3 *Ovis Aries*						
M00216	V$TATA_C	0.848957	71	(+)	NCTATAAAAR	ACATTAAAAC
M00252	V$TATA_01	0.77493	71	(+)	STATAAAWRNNNNNN	ACATTAAAACATTCT
M00216	V$TATA_C	0.774228	138	(+)	NCTATAAAAR	ATTTTAGAAG
M00216	V$TATA_C	0.802218	216	(+)	NCTATAAAAR	TCTATCTAAA
M00216	V$TATA_C	0.792976	234	(+)	NCTATAAAAR	TCTGTATAAT
M00216	V$TATA_C	0.740692	326	(+)	NCTATAAAAR	TTCTTTAAAA
M00216	V$TATA_C	0.963031	327	(+)	NCTATAAAAR	TCTTTAAAAA
M00216	V$TATA_C	0.767626	328	(+)	NCTATAAAAR	CTTTAAAAAA
M00252	V$TATA_01	0.850546	328	(+)	STATAAAWRNNNNNN	CTTTAAAAAAAAAAA
M00216	V$TATA_C	0.793768	329	(+)	NCTATAAAAR	TTTAAAAAAA
M00252	V$TATA_01	0.779751	375	(+)	STATAAAWRNNNNNN	AGACAAATGTGGAGG
M00252	V$TATA_01	0.773154	395	(+)	STATAAAWRNNNNNN	CTTTTTAGGAAGATG
M00127	V$GATA1_03	0.783684	43	(+)	RNSNNGATAANNGN	GAGCTGACAGTCAC
M00075	V$GATA1_01	0.776407	45	(+)	SNNGATNNNN	GCTGACAGTC
M00127	V$GATA1_03	0.810142	59	(+)	RNSNNGATAANNGN	GGAAAGGTAATCAC
M00075	V$GATA1_01	0.811945	64	(-)	SNNGATNNNN	GGTAATCACA
M00076	V$GATA2_01	0.827244	64	(-)	NNNGATRNNN	GGTAATCACA
M00075	V$GATA1_01	0.783317	108	(+)	SNNGATNNNN	GCAGTTTGTG
M00127	V$GATA1_03	0.811612	148	(-)	RNSNNGATAANNGN	AACATTTTCTTCCT
M00075	V$GATA1_01	0.791708	184	(-)	SNNGATNNNN	GAATATCCTT
M00076	V$GATA2_01	0.872801	184	(-)	NNNGATRNNN	GAATATCCTT
M00203	V$GATA_C	0.830382	188	(-)	NGATAAGNMNN	ATCCTTTGTCA
M00127	V$GATA1_03	0.812347	189	(-)	RNSNNGATAANNGN	TCCTTTGTCATCAT
M00127	V$GATA1_03	0.791034	192	(-)	RNSNNGATAANNGN	TTTGTCATCATTAT
M00075	V$GATA1_01	0.777887	194	(-)	SNNGATNNNN	TGTCATCATT
M00076	V$GATA2_01	0.796572	194	(-)	NNNGATRNNN	TGTCATCATT
M00126	V$GATA1_02	0.803125	201	(+)	NNNNNGATANKGNN	ATTATGATATTAGT
M00128	V$GATA1_04	0.83701	202	(+)	NNCWGATARNNNN	TTATGATATTAGT
M00075	V$GATA1_01	0.804541	203	(+)	SNNGATNNNN	TATGATATTA
M00076	V$GATA2_01	0.853406	203	(+)	NNNGATRNNN	TATGATATTA
M00077	V$GATA3_01	0.843155	204	(+)	NNGATARNG	ATGATATTA

M00203	V$GATA_C	0.91923	212	(-)	NGATAAGNMNN	AGTTTCTATCT
M00126	V$GATA1_02	0.816875	213	(-)	NNNNNGATANKGNN	GTTTCTATCTAAAC
M00128	V$GATA1_04	0.871936	213	(-)	NNCWGATARNNNN	GTTTCTATCTAAA
M00077	V$GATA3_01	0.882588	215	(-)	NNGATARNG	TTCTATCTA
M00075	V$GATA1_01	0.792201	228	(+)	SNNGATNNNN	TTAGATTCTG
M00077	V$GATA3_01	0.824546	229	(+)	NNGATARNG	TAGATTCTG
M00127	V$GATA1_03	0.785154	259	(-)	RNSNNGATAANNGN	ATTTTTAACTTCAC
M00075	V$GATA1_01	0.833169	286	(+)	SNNGATNNNN	CAAGATTTGA
M00076	V$GATA2_01	0.815516	286	(+)	NNNGATRNNN	CAAGATTTGA
M00077	V$GATA3_01	0.853788	287	(+)	NNGATARNG	AAGATTTGA
M00126	V$GATA1_02	0.820625	289	(+)	NNNNNGATANKGNN	GATTTGATACTTGC
M00075	V$GATA1_01	0.825271	291	(+)	SNNGATNNNN	TTTGATACTT
M00076	V$GATA2_01	0.81281	291	(+)	NNNGATRNNN	TTTGATACTT
M00126	V$GATA1_02	0.771563	310	(+)	NNNNNGATANKGNN	TTGAAGAAAATGTA
M00075	V$GATA1_01	0.837117	321	(+)	SNNGATNNNN	GTAGATTCTT
M00076	V$GATA2_01	0.812359	343	(-)	NNNGATRNNN	AAGTATGTTC
M00076	V$GATA2_01	0.793415	352	(+)	NNNGATRNNN	CAAGACAGTG
M00127	V$GATA1_03	0.831945	401	(+)	RNSNNGATAANNGN	AGGAAGATGAAAGT
M00075	V$GATA1_01	0.78233	403	(+)	SNNGATNNNN	GAAGATGAAA
M00076	V$GATA2_01	0.79567	403	(+)	NNNGATRNNN	GAAGATGAAA
M00077	V$GATA3_01	0.824989	404	(+)	NNGATARNG	AAGATGAAA
M00126	V$GATA1_02	0.805313	414	(+)	NNNNNGATANKGNN	TACAGGATAATCCA
M00075	V$GATA1_01	0.842547	416	(+)	SNNGATNNNN	CAGGATAATC
M00076	V$GATA2_01	0.899414	416	(+)	NNNGATRNNN	CAGGATAATC
M00077	V$GATA3_01	0.821444	417	(+)	NNGATARNG	AGGATAATC
M00203	V$GATA_C	0.845294	418	(+)	NGATAAGNMNN	GGATAATCCAG
M00075	V$GATA1_01	0.782823	419	(-)	SNNGATNNNN	GATAATCCAG
M00075	V$GATA1_01	0.884501	425	(+)	SNNGATNNNN	CCAGATTGAT
M00076	V$GATA2_01	0.843482	425	(+)	NNNGATRNNN	CCAGATTGAT
M00077	V$GATA3_01	0.835623	426	(+)	NNGATARNG	CAGATTGAT
M00223	V$STAT_01	0.795815	82	(-)	TTCCCRKAA	TTCTAAGAG
M00223	V$STAT_01	0.825561	102	(+)	TTCCCRKAA	TTCACGGCA
M00223	V$STAT_01	0.888581	141	(-)	TTCCCRKAA	TTAGAAGAA

M00223	V$STAT_01	0.853542	397	(-)	TTCCCRKAA	TTTTAGGAA
M00137	V$OCT1_03	0.909917	62	(+)	NNNRTAATNANNN	AAGGTAATCACAT
M00137	V$OCT1_03	0.847491	68	(-)	NNNRTAATNANNN	ATCACATTAAAAC
M00162	V$OCT1_06	0.842187	70	(-)	CWNAWTKWSATRYN	CACATTAAAACATT
M00136	V$OCT1_02	0.80157	85	(-)	NNGAATATKCANNNN	TAAGAGAATAATTCT
M00136	V$OCT1_02	0.776936	112	(-)	NNGAATATKCANNNN	TTTGTGCTTATTACA
M00248	V$OCT1_07	0.799237	112	(+)	TNTATGNTAATT	TTTGTGCTTATT
M00137	V$OCT1_03	0.890952	116	(-)	NNNRTAATNANNN	TGCTTATTACACC
M00136	V$OCT1_02	0.921765	179	(-)	NNGAATATKCANNNN	AGCTTGAATATCCTT
M00136	V$OCT1_02	0.804277	182	(+)	NNGAATATKCANNNN	TTGAATATCCTTTGT
M00137	V$OCT1_03	0.95891	196	(-)	NNNRTAATNANNN	TCATCATTATGAT
M00161	V$OCT1_05	0.875809	198	(+)	MKNATTTGCATAYY	ATCATTATGATATT
M00162	V$OCT1_06	0.844922	198	(+)	CWNAWTKWSATRYN	ATCATTATGATATT
M00162	V$OCT1_06	0.919141	223	(+)	CWNAWTKWSATRYN	AAACATTAGATTCT
M00162	V$OCT1_06	0.881641	224	(-)	CWNAWTKWSATRYN	AACATTAGATTCTG
M00137	V$OCT1_03	0.887791	236	(+)	NNNRTAATNANNN	TGTATAATGTTAT
M00162	V$OCT1_06	0.804297	252	(+)	CWNAWTKWSATRYN	CAAATTTATTTTTA
M00162	V$OCT1_06	0.894141	286	(+)	CWNAWTKWSATRYN	CAAGATTTGATACT
M00138	V$OCT1_04	0.831451	311	(+)	NNNNNNNWATGCAAATNNNWNNW	TGAAGAAAATGTAGAT-TCTTTAA
M00136	V$OCT1_02	0.798051	313	(+)	NNGAATATKCANNNN	AAGAAAATGTAGATT
M00195	V$OCT1_Q6	0.817686	315	(+)	NNNNATGCAAATNAN	GAAAATGTAGATTCT
M00162	V$OCT1_06	0.847266	316	(+)	CWNAWTKWSATRYN	AAAATGTAGATTCT
M00162	V$OCT1_06	0.834375	316	(-)	CWNAWTKWSATRYN	AAAATGTAGATTCT
M00248	V$OCT1_07	0.793089	316	(+)	TNTATGNTAATT	AAAATGTAGATT
M00138	V$OCT1_04	0.794647	320	(+)	NNNNNNNWATGCAAATNNNWNNW	TGTAGAT-TCTTTAAAAAAAAAAA
M00137	V$OCT1_03	0.851442	393	(-)	NNNRTAATNANNN	AACTTTTTAGGAA
M00162	V$OCT1_06	0.800781	419	(-)	CWNAWTKWSATRYN	GATAATCCAGATTG
M00059	V$YY1_01	0.803408	144	(+)	NNNNNCCATNTWNNNWN	GAAGAACATTTTCTTCC
M00059	V$YY1_01	0.792267	194	(+)	NNNNNCCATNTWNNNWN	TGTCATCATTATGATAT
M00059	V$YY1_01	0.77097	311	(-)	NNNNNCCATNTWNNNWN	TGAAGAAAATGTAGATT
M00059	V$YY1_01	0.791612	399	(-)	NNNNNCCATNTWNNNWN	TTAGGAAGATGAAAGTA
